# LGK974 suppresses lipopolysaccharide-induced endotoxemia in mice by modulating the crosstalk between the Wnt/β-catenin and NF-κB pathways

**DOI:** 10.1038/s12276-021-00577-z

**Published:** 2021-03-10

**Authors:** Jaewoong Jang, Jaewon Song, Hyunji Lee, Inae Sim, Young V. Kwon, Eek-hoon Jho, Yoosik Yoon

**Affiliations:** 1 Department of Microbiology, Chung-Ang University College of Medicine, Seoul, 06974 Republic of Korea; 2 Department of Biochemistry, University of Washington, Seattle, WA 98195 USA; 3 Department of Life Science, University of Seoul, Seoul, 02504 Republic of Korea

**Keywords:** Cytokines, Sepsis

## Abstract

Endotoxemia, a type of sepsis caused by gram-negative bacterial endotoxin [i.e., lipopolysaccharide (LPS)], is associated with manifestations such as cytokine storm; failure of multiple organs, including the liver; and a high mortality rate. We investigated the effect and mechanism of action of LGK974, a Wnt signaling inhibitor, in mice with LPS-induced endotoxemia, an animal model of sepsis. LGK974 significantly and dose-dependently increased the survival rate and reduced plasma cytokine levels in mice with LPS-induced endotoxemia. Transcriptome analysis of liver tissues revealed significant changes in the expression of genes associated with the Wnt pathway as well as cytokine and NF-κB signaling during endotoxemia. LGK974 treatment suppressed the activation of NF-κB signaling and cytokine expression as well as the Wnt/β-catenin pathway in the livers of endotoxemic mice. Coimmunoprecipitation of phospho-IκB and β-transducin repeat-containing protein (β-TrCP) was increased in the livers of endotoxemic mice but was reduced by LGK974 treatment. Moreover, LGK974 treatment decreased the coimmunoprecipitation and colocalization of β-catenin and NF-κB, which were elevated in the livers of endotoxemic mice. Our results reveal crosstalk between the Wnt/β-catenin and NF-κB pathways via interactions between β-TrCP and phospho-IκB and between β-catenin and NF-κB during endotoxemia. The results of this study strongly suggest that the crosstalk between the Wnt/β-catenin and NF-κB pathways contributes to the mutual activation of these two pathways during endotoxemia, which results in amplified cytokine production, liver damage and death, and that LGK974 suppresses this vicious amplification cycle by reducing the crosstalk between these two pathways.

## Inflammation: Stopping the out-of-control spiral

A cancer drug called LGK974 may help treat sepsis, an exaggerated inflammatory response to infection leading to multiple organ failure and a leading cause of death in patients in intensive care. Sepsis can result when the body’s response to infection spirals out of control. Although the mechanism is poorly understood, the Wnt signaling pathway was recently reported to be involved. Yoosik Yoon at Chung-Ang University College of Medicine and Eek-hoon Jho at the University of Seoul, both in South Korea, investigated how LGK974, developed to fight cancers dependent on Wnt signaling, would affect sepsis, using a mouse model. They discovered that LGK974 interrupted feedback between Wnt and another signaling pathway, preventing uncontrolled amplification of inflammation. The mice showed decreased organ damage and increased survival. These results present a promising research direction for treating sepsis.

## Introduction

Endotoxemia is mediated by the overproduction of proinflammatory cytokines caused by the endotoxin [i.e., lipopolysaccharide (LPS)] of gram-negative bacteria^1^. Under normal physiological conditions, cytokine release is limited to regulate the immune response; however, the endotoxemic state induces unlimited cytokine production, a phenomenon termed “cytokine storm”. This phenomenon is mediated by the activation of a cascade that induces autoamplification of cytokine production, which eventually results in multiple organ failure and death^2,3^. Sepsis is defined as life-threatening organ dysfunction caused by a dysregulated host response to infection^4^, whereas endotoxemia is a type of sepsis-induced by infection with gram-negative bacteria^5^. The prevalence of endotoxemia is as high as to 82% in patients with sepsis^6^. Therefore, mice with LPS-induced endotoxemia have been used as an animal model of sepsis^7–10^.

Liver dysfunction caused by bacteria and endotoxin is an essential event in sepsis accompanied by cytokine storm^11^. Elevated cytokine expression in the liver is a marker of sepsis^12^, and sepsis-induced liver dysfunction increases mortality^13^; therefore, attenuation of liver injury and restoration of liver function significantly lower the mortality risk of patients with sepsis^14^.

Recently, Wnt signaling has been reported to be involved in inflammation and sepsis. Microbial stimulation of human mononuclear cells induces WNT5A, which upregulates the expression of proinflammatory cytokines, including IL-6 and IL-1β^15^. An inhibitor of β-catenin-mediated transcription, iCRT3, was reported to reduce LPS-induced WNT signaling and proinflammatory cytokine production^16^. It was reported that WNT5A levels were significantly increased in the sera of patients with sepsis^17^ and that multiple WNT ligands were expressed in the peripheral blood of patients with septic shock^18^. However, the crosstalk between the Wnt/β-catenin and NF-κB pathways has not yet been elucidated in the context of sepsis. In this study, we analyzed the effects of LGK974, a Wnt signaling inhibitor, in LPS-induced endotoxemic mice through analyses of the crosstalk between the Wnt/β-catenin and NF-κB pathways in the liver as well as the effects of this crosstalk on the survival rate and induction of cytokine storm.

LGK974 has been developed as a candidate anticancer drug against Wnt-driven cancers^19^; it has shown suppressive effects against ovarian cancer^20^, lung cancer^21^, squamous cell carcinoma^22,23^, glioblastoma^24,25^, and colon cancer^26^. It is currently in phase 1 clinical trial in patients with colorectal cancer^27^. Other effects of this Wnt signaling inhibitor have also been reported; LGK974 has been shown to ameliorate cystogenesis in a mouse model of polycystic kidney disease^28^. We previously reported that LGK974 exerts anti-inflammatory effects in LPS-stimulated human bronchial epithelial cells and human umbilical vein endothelial cells^29^. However, no studies have investigated its effects on sepsis. Sepsis is the most common cause of death among critically ill patients in noncoronary intensive care units, and there is an urgent need for an effective treatment for sepsis^30^. Thus, in the present study, we examined the effect and mechanism of action of LGK974 in mice with LPS-induced endotoxemia, an animal model of sepsis.

## Materials and methods

### Reagents

Anti-IκB (cat# 9242), anti-phospho-IκB (Ser 32/36) (cat# 9246), anti-NF-κB p65 (cat# 8242), anti-GSK3β (cat# 9315), anti-phospho-GSK3β (Ser 9) (cat# 9336), anti-AXIN (cat# 2087), anti-LRP6 (cat# 3395), anti-phospho-LRP6 (Ser 1490) (cat# 2568), and anti-β-TrCP (cat# 4394S) primary antibodies, as well as anti-mouse (cat# 7076S) and anti-rabbit (cat# 7074S) secondary antibodies, were purchased from Cell Signaling Technology (Danvers, MA, USA). An anti-β-catenin antibody (cat# 610153) was purchased from BD Transduction Laboratories Inc. (Lexington, KY, USA). Anti-β-actin (cat# 47778) and anti-TBP (cat# 204) antibodies were purchased from Santa Cruz Biotechnology Inc. (Santa Cruz, CA, USA). LPS from *Klebsiella pneumoniae* was purchased from Sigma-Aldrich (St. Louis, MO, USA). LGK974 was obtained from MedChemExpress (Monmouth Junction, NJ, USA).

### Animal experiments

Animal experimental protocols were approved by the institutional animal care and use committee of Chung-Ang University (approval numbers 2017-00099 and 2018-00042), and all experiments were performed in accordance with the animal research: reporting of in vivo experiments guidelines. Male C57BL/6 mice (4 weeks old) were purchased from Central Lab Animal Inc. (Seoul, Republic of Korea). LGK974 was injected *i.p*. 2 h before *i.p*. injection of LPS in most experiments. For survival experiments, various doses of LGK974 were injected 2 h before, simultaneously with, or 1 h after LPS injection. For another survival experiment, various doses of LGK974 were injected simultaneously with *Escherichia coli (E. coli)*. *E. coli* strain K12 was cultured overnight in LB broth, and cell numbers were determined based on the optical density at 600 nm. Then, 10^11^ colony-forming units (CFUs) of *E. coli* were resuspended in 200 μl of saline immediately before *i.p*. injection. Both LGK974 and LPS were dissolved in saline by vigorous vortexing immediately before injection. Saline-injected mice served as a control group. Mice were anesthetized via *i.p*. injection of 10 mg/kg alfaxalone (Jurox, Rutherford, Australia) 6 h after LPS injection. Liver tissue was collected and stored at −70 °C for further analyses. Blood samples were collected in EDTA tubes and were then centrifuged at 2000 × *g* for 10 min to collect plasma. A magnetic Luminex assay with antibody-based 9-plex immunoassays (Luminex, Austin, TX, USA) was used to measure the plasma concentrations of selected cytokines, including TNF-α, IL-6, IL-1β, IL-1α, IL-12, IFN-γ, MCP-1, RANTES, and IL-10. Measurements were performed using a Magpix Luminex instrument (Luminex) and MasterPlex QT 2010 software (MiraiBio, San Francisco, CA, USA).

### RNA-seq and gene set enrichment analysis (GSEA)

RNA samples extracted from the livers of mice were analyzed by RNA-seq, and the original raw data have been deposited in the NCBI Gene Expression Omnibus database under accession number GSE127459 (https://www.ncbi.nlm.nih.gov/geo/query/acc.cgi?acc=GSE127459).

GSEA was conducted to compare the RNA-seq data sets. The primary result of GSEA is an enrichment score (ES), which indicates the degree to which a gene set is overrepresented among upregulated genes or downregulated genes in a ranked list of genes in the expression data set. A positive ES indicates enrichment of the gene set among the upregulated genes in the ranked list; a negative ES indicates enrichment of the gene set among the downregulated genes in the ranked list. The normalized ES (NES) is the ES for the gene set after normalization across all analyzed gene sets. The significance (*P* value) of the NES is primarily affected by the false discovery rate, which is the probability that a gene set with a given NES represents a false-positive finding (http://software.broadinstitute.org/gsea/index.jsp)^31^.

### Quantitative reverse-transcription (RT-q)PCR

Total RNA was isolated from liver tissue using an RNeasy Kit (Qiagen; Cat. No. 74106) in a homogenizer. One microgram of RNA was reverse transcribed using a cDNA Reverse Transcription Kit (Applied Biosystems Inc.; Cat. No. 43-688-13). RT-qPCR was conducted as described previously^29^. Assay-on-Demand gene expression products (Applied Biosystems, Inc., Foster City, CA, USA) were used for RT-qPCR to evaluate the mRNA levels of *Tnf* (Mm00443258_m1), *Il6* (Mm00446190_m1), *Il1b* (Mm00434228_m1), *Il1a* (Mm00439620_m1), *Wnt3a* (Mm00437337_m1), *Wnt5a* (Mm00437347_m1), *Wnt10a* (Mm00437325_m1), and *Wnt10b* (Mm00442104_m1). For each experimental group, the mRNA levels were normalized to the 18S ribosomal RNA level, and the ratios of the normalized mRNA levels in each group were compared to those in the control group using the comparative Ct method^32^.

### Protein extraction and western blotting

Liver tissues were lysed in a homogenizer using RIPA buffer containing 25 mM Tris–HCl (pH 7.6), 150 mM NaCl, 1% Nonidet P-40, 1% sodium deoxycholate, 0.1% sodium dodecyl sulfate, and protease inhibitor cocktail (Sigma-Aldrich; Cat. No. P8340) for 30 min at 4 °C. Total tissue lysates were obtained after removing the insoluble material by centrifugation at 20,000 × *g* for 20 min at 4 °C. Nuclear lysates were collected from liver tissue using a nuclear extraction kit (Active Motif, Carlsbad, CA, USA). Protein concentrations were determined using a BCA protein assay kit (Pierce, Waltham, MA, USA), and 10–100 μg of protein was analyzed by western blotting as described previously^29^. β-Actin and TATA box-binding protein (TBP) were used as loading controls for total tissue lysates and nuclear lysates, respectively. Band intensities—corresponding to protein expression levels—were quantified by ImageJ (NIH, Bethesda, MD, USA) and normalized to those of the loading controls.

### Target DNA-binding activity of NF-κB

Nuclear fractions were collected from frozen tissues using a nuclear extraction kit (Active Motif, Carlsbad, CA, USA), and protein concentrations were determined using a BCA protein assay kit (Pierce). The binding affinity of NF-κB to its target DNA sequence (5′-GGGACTTTCC-3′) was measured using a TransAM NF-κB ELISA Kit (Active Motif) according to the manufacturer’s instructions. Briefly, 10 μg of protein (nuclear fraction) was added to a 96-well plate coated with oligonucleotides containing the target DNA sequence. After incubation and washing, an anti-NF-κB antibody was added to the wells, followed by the horseradish peroxidase-conjugated secondary antibody provided in the kit. The absorbance at 450 nm was measured after sequential addition of the developing solution and the stop solution provided in the kit.

### Coimmunoprecipitation assay for protein–protein binding interactions

Coimmunoprecipitation experiments were performed using an immunoprecipitation kit from BioVision (Mountain View, CA, USA) according to the manufacturer’s instructions. Frozen tissues were lysed in a homogenizer using a nondenaturing lysis buffer provided in the kit at 4 °C for 30 min and were then centrifuged at 10,000 × *g* for 10 min at 4 °C. The supernatant was supplemented with 2 μg/ml of an antibody specific for the bait protein and incubated overnight at 4 °C. After adding 25 µl of protein A/G bead slurry and incubating for 1 h at 4 °C, the beads were collected by centrifugation at 2000 × *g* for 2 min. The collected beads were washed three times with 1 ml of wash buffer provided with the kit and mixed with 40 μl of 2× sodium dodecyl sulfate-polyacrylamide gel electrophoresis loading buffer. The amount of target protein bound to the beads was measured by western blotting with an antibody specific for the target protein. Band intensities were quantified using ImageJ (NIH, Bethesda, MD, USA).

### Confocal microscopy for evaluation of protein colocalization

Tissue preparation and confocal microscopy were conducted as described previously with some modifications^33^. Liver tissues were snap-frozen and embedded in OCT compound (Sakura, Tokyo, Japan). Embedded tissues were cryosectioned at a thickness of 10 μm. For immunofluorescence, sections were fixed with cold acetone for 10 min and incubated first in blocking buffer (CAS-Block; Thermo Fisher Scientific) for 1 h and then with a mouse anti-β-catenin antibody (Transduction Laboratory, #610153; 1:200) and a rabbit anti-NF-κB p65 antibody (Cell Signaling Technology #8242, 1:200) in phosphate-buffered saline (PBS) overnight. Sections were washed three times in PBS containing 0.03% Triton X-100 and incubated with a Cy3-labeled donkey anti-mouse IgG antibody (cat# 715-165-150, Jackson ImmunoResearch Laboratories, Inc., West Grove, PA, USA, 1:400) and FITC-labeled donkey anti-rabbit IgG (cat# 715-095-152, Jackson ImmunoResearch Laboratories, Inc., 1:400) in PBS for 1 h. After counterstaining with 1 μg/ml DAPI (cat# D9542, Sigma-Aldrich), the tissues were mounted and visualized under a confocal microscope (Nikon A1Si, Nikon, Tokyo, Japan). Images from different experimental groups were acquired under the same exposure and detection settings. Colocalization of fluorescence signals was analyzed using ImageJ.

### Statistical analysis

All data are expressed as the mean ± standard deviation values. Differences among experimental groups were analyzed using one-way ANOVA with Duncan’s multiple range test. *P* < 0.05 was considered significant. All analyses were performed using SPSS ver. 14 (SPSS, Chicago, IL, USA).

## Results and discussion

### LPS-induced endotoxemic mice show dose-dependent lethality and cytokine storm

**Fig. 1:**
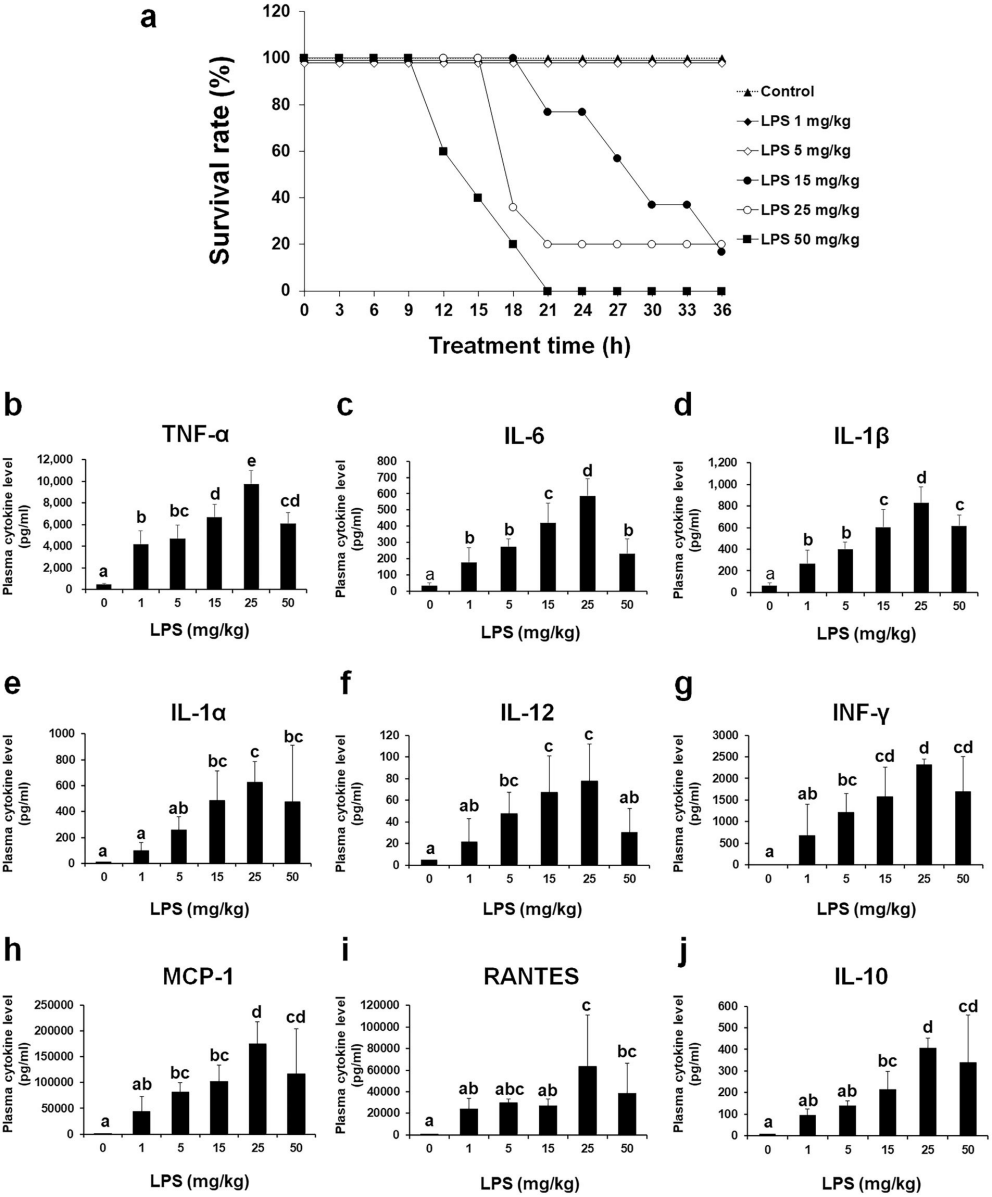
Survival rates and plasma cytokine levels in mice with LPS-induced endotoxemia. C57BL/6 mice were injected *i.p*. with 1–50 mg/kg LPS dissolved in saline. Mice in the control group were injected with the same volume of saline. **a** Survival in each group was monitored at 3-h intervals (*n* = 5). **b**–**j** Cytokine concentrations in plasma collected from mice were measured using a magnetic Luminex assay. The data are presented as the mean ± standard deviation values (*n* = 7). Differences among experimental groups were analyzed using one-way ANOVA with Duncan’s multiple range test. The different letters indicate significant differences (^*^P < 0.05).

LPS decreased the survival rate of C57BL/6 mice in a dose-dependent manner (Fig. 1a). To evaluate whether LPS-induced endotoxemia induces cytokine storm in mice, the concentrations of plasma cytokines were measured after the injection of LPS at various doses (Fig. 1b–j). The plasma concentrations of TNF-α, IL-6, IL-1β, IL-1α, IL-12, INF-γ, MCP-1, RANTES, and IL-10 were markedly and dose-dependently increased in mice injected with 1–25 mg/kg LPS. As the highest plasma cytokine levels were observed after injection of 25 mg/kg LPS, this dose was used to induce endotoxemia in subsequent experiments.

### LGK974 improves the survival rate and suppresses cytokine storm in mice with LPS-induced endotoxemia

**Fig. 2:**
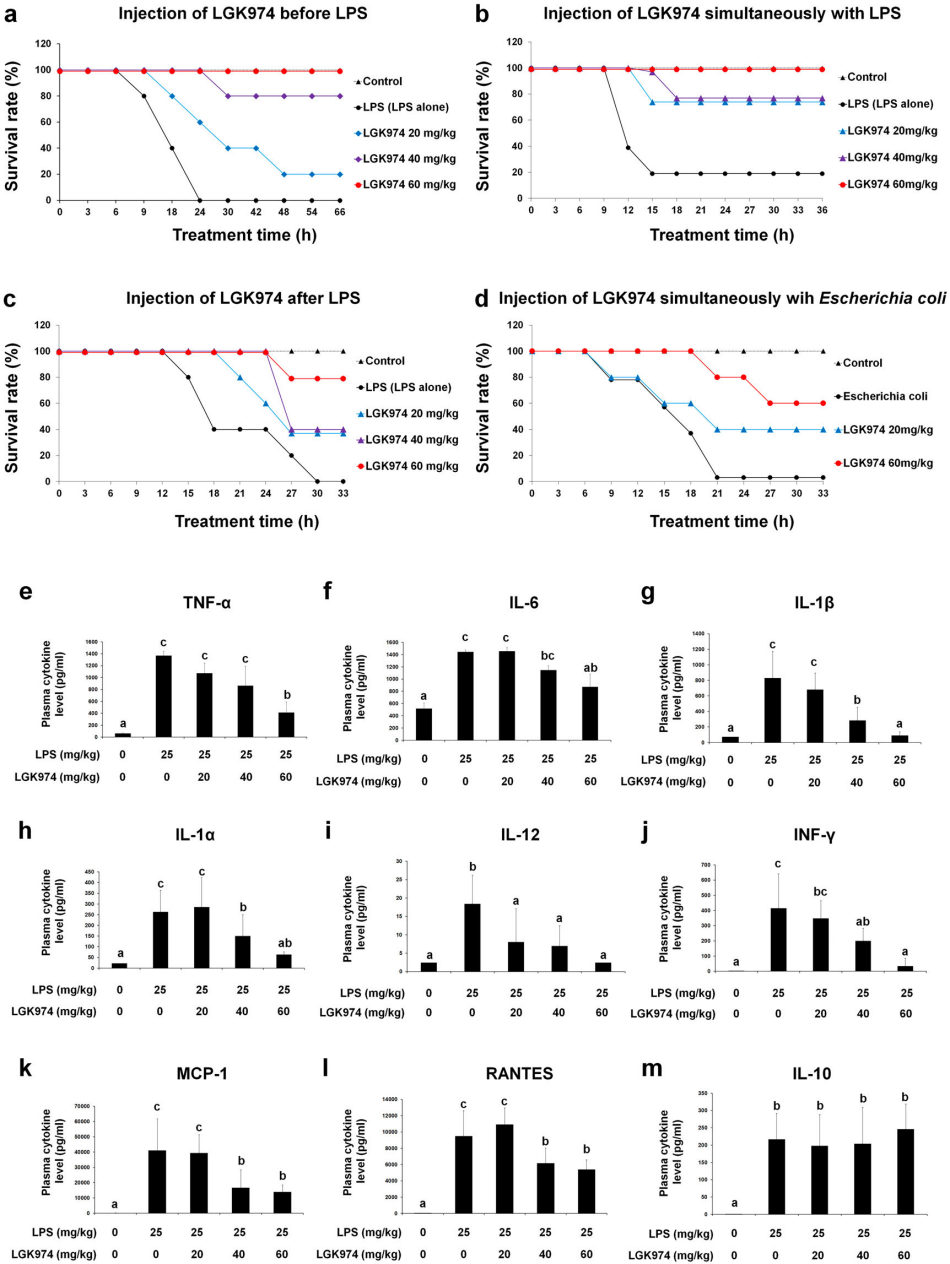
Effect of LGK974 on the survival rate and cytokine storm in endotoxemic mice. **a**–**d** Effect of LGK974 on the survival rate in various endotoxemic mouse models (*n* = 5). LGK974 (0–60 mg/kg) was injected *i.p*. **a** 2 h before, **b** simultaneously with, and **c** 1 h after *i.p*. injection of 25 mg/kg of LPS. **d** LGK974 (0–60 mg/kg) was injected *i.p*. simultaneously with 10^11^ CFU of viable *Escherichia coli* cells. Mice in the control group were injected with the same volume of saline. **e**–**m** LGK974 suppresses cytokine storm in endotoxemic mice. C57BL/6 mice were injected *i.p*. with 0–60 mg/kg LGK974 2 h before being injected with 25 mg/kg LPS. Cytokine concentrations in the plasma were determined using a magnetic Luminex assay. The data are presented as the mean ± standard deviation values (*n* = 7). Differences among experimental groups were analyzed using one-way ANOVA with Duncan’s multiple range test. The different letters indicate significant differences (^*^P < 0.05).

We analyzed the effect of LGK974 on the survival rate of mice with LPS-induced endotoxemia (4-week‒old male mice). The survival rate of mice that received 25 mg/kg LPS alone was 0%. Mice treated with 20 and 40 mg/kg LGK974 before LPS injection exhibited survival rates of 20 and 80%, respectively, whereas mice treated with 60 mg/kg LGK974 had a 100% survival rate (5 mice per group) (Fig. 2a). It should be noted that LGK974 increased the survival rate of mice with LPS-induced endotoxemia regardless of whether it was administered simultaneously with LPS injection (Fig. 2b) or after LPS injection (Fig. 2c). Moreover, LGK974 increased the survival rate of mice injected with viable *E. coli* cells (Fig. 2d). Overall, these results showed that LGK974 improved the survival rate of mice in which sepsis was induced by injection of endotoxin or bacteria in a dose-dependent manner. We also conducted survival experiments in 4-week-old female mice and 13-week-old male mice (Supplementary Data 1). LGK974 increased the survival rate in both experiments, suggesting that the protective effect of LGK974 may be independent of the sex and age of the mice. The effects of LGK974 on mice older than 13 weeks remain to be investigated in further studies.

When mice were injected with 0–60 mg/kg LGK974 and 25 mg/kg LPS, the levels of proinflammatory cytokines, including TNF-α, IL-6, IL-1β, IL-1α, IL-12, INF-γ, MCP-1, and RANTES, were significantly and dose-dependently downregulated by LGK974 (Fig. 2e–l). However, the level of IL-10 was not altered upon LGK974 treatment (Fig. 2m). IL-10 is a potent anti-inflammatory cytokine that plays a central role in limiting the host immune response, thereby preventing damage to host tissues^34^. The effects of LGK974 on other anti-inflammatory cytokines remain to be elucidated. Our data suggest that LGK974 has a suppressive effect on proinflammatory cytokine production in mice with LPS-induced endotoxemia.

### RNA-seq and GSEA of liver tissue reveal significant changes in the expression of genes in the Wnt pathway as well as cytokine and NF-κB signaling during LPS-induced endotoxemia

Among the multiple organs damaged in endotoxemia, we focused on the liver, as liver dysfunction caused by bacteria or endotoxin has been shown to be an essential event in sepsis, along with cytokine storm^11^. RNA-seq experiments were conducted to analyze gene expression patterns in liver tissues from saline-injected mice (control group) and mice injected with 25 mg/kg LPS (endotoxemic group) or with 60 mg/kg LGK974 and 25 mg/kg LPS (LGK974 group). The complete set of RNA-seq data, including fold changes, *P* values, and expression levels for more than 23,000 genes, are presented in Supplementary Data 2 (RNA-seq data).

**Fig. 3:**
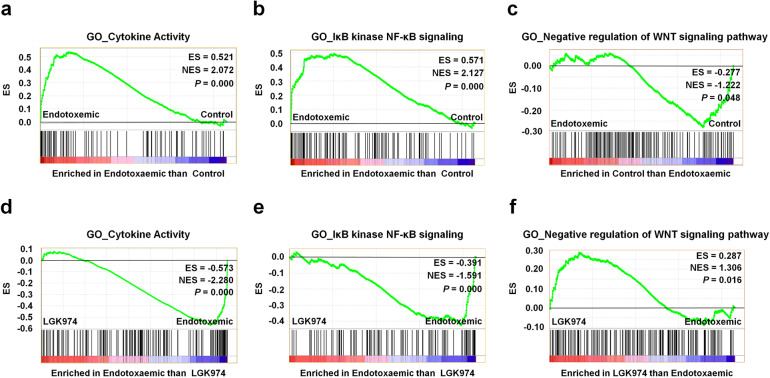
Gene set enrichment analysis (GSEA) of RNA-seq data from liver tissues of endotoxemic mice. RNA was isolated from the liver tissues of mice injected with saline (control group), 25 mg/kg LPS (endotoxemic group), or 60 mg/kg LGK974 and 25 mg/kg LPS (LGK974 group). **a**–**c** GSEA showing an overrepresentation of the GO term “cytokine activity” (**a**), the overrepresentation of the GO term “IκB kinase NF-κB signaling” (**b**), and underrepresentation of the GO term “negative regulation of Wnt signaling pathway” (**c**) in the endotoxemic group compared with the control group. **d**–**f** GSEA showing underrepresentation of the GO term “cytokine activity” (**d**), underrepresentation of the GO term “IκB kinase NF-κB signaling” (**e**), and overrepresentation of the GO term “negative regulation of Wnt signaling pathway” (**f**) in the LGK974 group compared with the endotoxemic group. The plot shows the running ES for the gene set as the analysis proceeded. The score at the peak of the plot (furthest from 0.0) is the ES for the gene set. ES enrichment score, NES normalized enrichment score.

GSEA showed that many gene ontology (GO) terms related to cytokines, NF-κB signaling, and the Wnt pathway were significantly altered in the liver upon LPS and LGK974 treatment; among these alterations, the changes in three GO terms are illustrated in Fig. 3, and the complete set of GSEA data for all GO terms is shown in Supplementary Data 3 (GSEA data). Most of the GO terms related to cytokine and NF-κB signaling were overrepresented in the endotoxemic group compared with the control group. The GO terms “cytokine activity” and “IκB kinase NF-κB signaling” are illustrated in Fig. 3a, b. Among the GO terms related to the Wnt pathway, the GO term “negative regulation of Wnt signaling pathway” was significantly underrepresented (Fig. 3c), while the GO term “positive regulation of Wnt signaling pathway” was not significantly changed (Supplementary Data 3 (GSEA data)). The GO terms “cytokine activity” and “IκB kinase NF-κB signaling” were significantly underrepresented, whereas the GO term “negative regulation of Wnt signaling pathway” was significantly overrepresented in the LGK974 group compared with the endotoxemic group (Fig. 3d–f). The list of genes in these selected GO terms is shown in Supplementary Data 4 (List of genes in selected GO terms). Collectively, these results suggested that the levels of transcripts associated with the Wnt pathway as well as with NF-κB signaling and cytokines were significantly changed in the livers of LPS-induced endotoxemic mice and that these changes were suppressed by LGK974 treatment.

### NF-κB signaling and cytokine expression are accompanied by Wnt signaling activation in the livers of mice with LPS-induced endotoxemia

**Fig. 4:**
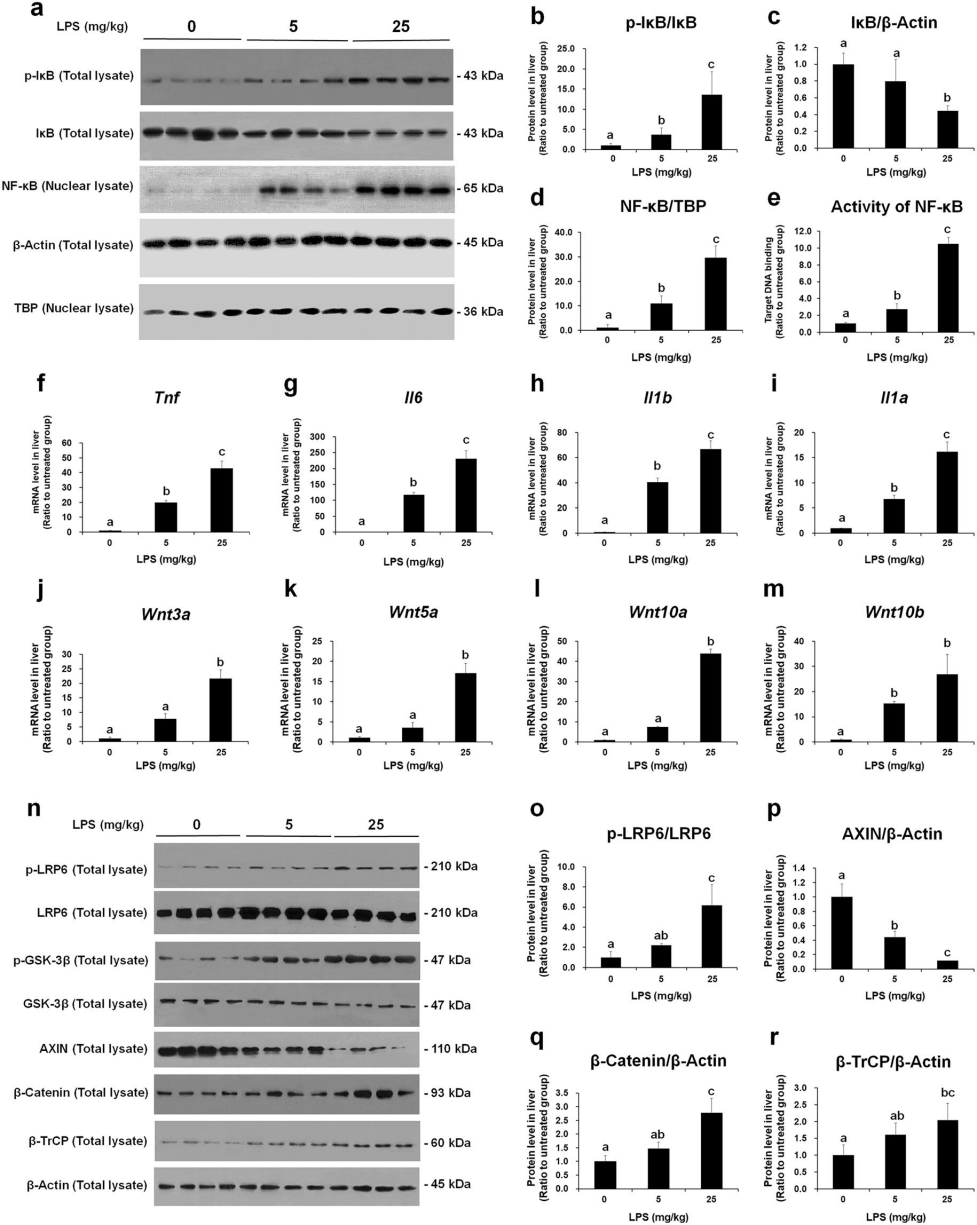
The NF-κB pathway and Wnt signaling are upregulated in the livers of mice with LPS-induced endotoxemia. Protein and RNA were extracted from the liver tissues of mice injected with 0, 5, or 25 mg/kg LPS. **a** Western blotting was performed to measure the levels of phospho-IκB and IκB in total tissue lysates and the NF-κB protein in nuclear lysates. β-Actin and TATA box-binding protein (TBP) was used as loading controls for total tissue lysates and nuclear lysates, respectively. Western blot raw image data are provided in Supplementary Data 5. **b**–**d** Band intensities were measured using ImageJ. **e** The binding activity of NF-κB to its target DNA sequence was measured using ELISA. **f**–**i** Messenger RNA levels of major proinflammatory cytokines was measured by RT-qPCR. **j**–**m** Messenger RNA levels of Wnt ligands were quantified by RT-qPCR. **n** Levels of the Wnt pathway components were measured by western blotting. β-Actin was used as the loading control. Western blot raw image data are provided in Supplementary Data 5. **o**–**r** Band intensities were quantified using ImageJ. The data are presented as the mean ± standard deviation values of quadruplicate experiments. Differences among experimental groups were analyzed using one-way ANOVA with Duncan’s multiple range test. The different letters indicate significant differences (^*^P < 0.05).

To confirm the findings of RNA-seq and GSEA, western blot and RT-qPCR experiments were conducted. Proteins were extracted from the livers of mice injected with 0, 5, or 25 mg/kg LPS and analyzed by western blotting (Fig. 4a). All original western blot images are provided in Supplementary Data 5. In the livers of mice with LPS-induced endotoxemia, phosphorylation of IκB increased, whereas the amount of IκB decreased (Fig. 4a–c). Moreover, the nuclear levels of NF-κB (Fig. 4a, d) and the target DNA-binding activity of NF-κB (Fig. 4e) were increased. NF-κB is a major proinflammatory transcription factor that binds to the regulatory region of genes encoding proinflammatory cytokines, thus inducing proinflammatory cytokine expression^35,36^. The relative mRNA expression levels of *Tnf*, *Il6*, *Ilb*, and *Ila* (the genes encoding TNF-α, IL-6, IL-1β, and IL-1α, respectively) were significantly increased by LPS in a dose-dependent manner (Fig. 4f–i).

As the GSEA results suggested that Wnt signaling in the liver was significantly changed during endotoxemia, we analyzed this pathway in detail. The mRNA levels of Wnt genes such as *Wnt3a*, *Wnt5a*, *Wnt10a*, *and Wnt10b* were significantly increased by LPS in a dose-dependent manner (Fig. 4j–m). The protein levels of phospho-lipoprotein receptor-related protein 6 (LRP6) (Fig. 4n, o), and phospho-glycogen synthase kinase-3β (GSK-3β) (Fig. 4n), two positive regulators of Wnt signaling, were increased by LPS. Conversely, AXIN, a negative regulator of Wnt signaling, was downregulated (Fig. 4n, p). β-Catenin, the executor protein of Wnt signaling, was significantly upregulated (Fig. 4n, q). Similarly, the expression of β-transducin repeat-containing protein (β-TrCP), which is one of the target genes of β-catenin^37^, was upregulated (Fig. 4n, r). Taken together, the above results confirmed that NF-κB signaling, cytokine expression, Wnt ligand expression, and the Wnt/β-catenin pathway were upregulated in the livers of mice with LPS-induced endotoxemia. It has been reported that transcripts of some Wnt ligands, including *WNT5A* and *WNT10B*, were induced by NF-κB^38–40^, and the promoters of *WNT5A* and *WNT10B* were found to contain NF-κB-binding sites^41,42^. We suggest that the LPS-induced Wnt ligand expression and Wnt pathway activation during LPS-induced endotoxemia (Fig. 4j–r) may be attributed to increased NF-κB signaling and its target DNA binding activity (Fig. 4a–e).

### LGK974 suppresses Wnt/β-catenin signaling, NF-κB signaling, and cytokine expression in the livers of mice with LPS-induced endotoxemia

**Fig. 5:**
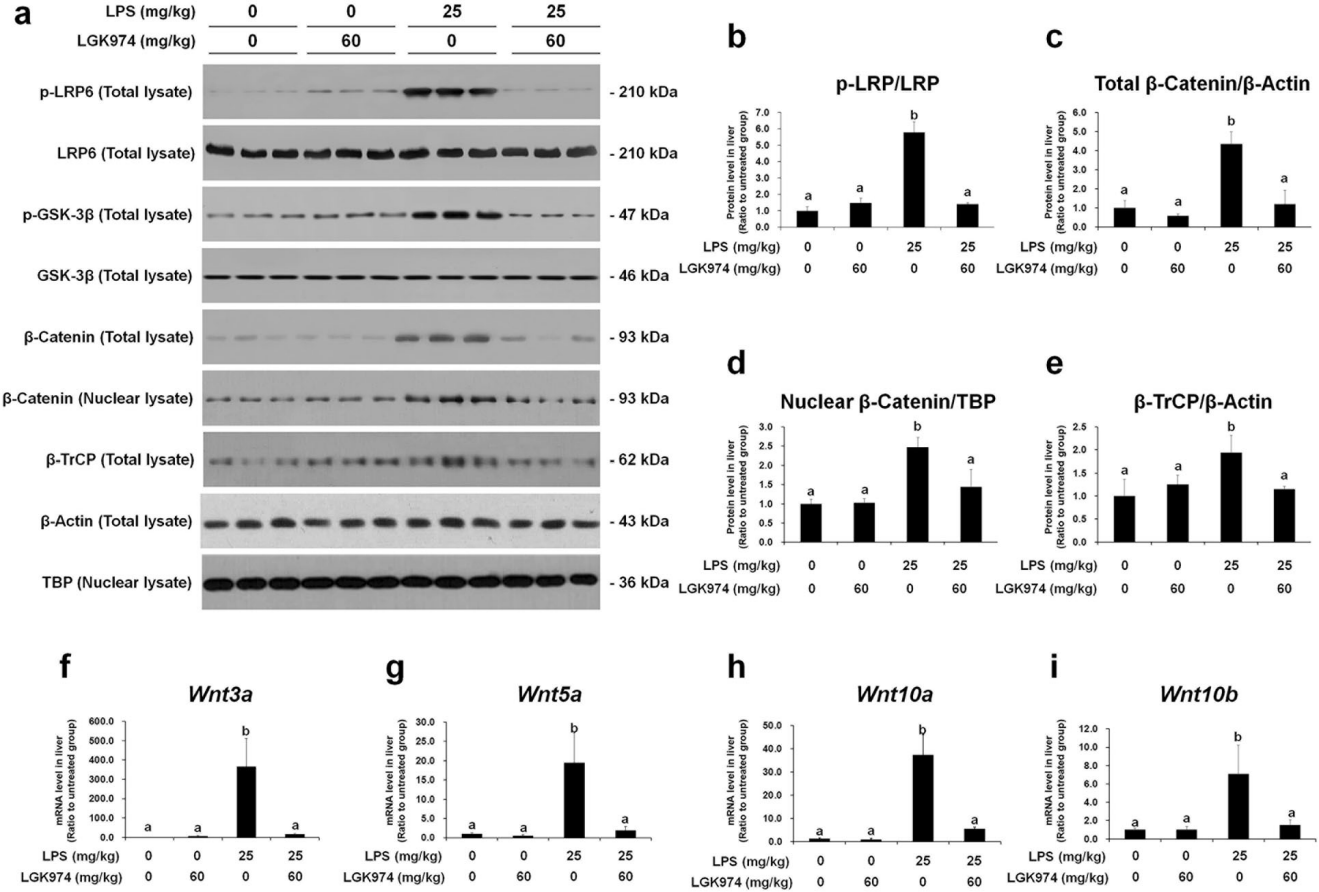
LGK974 inhibits Wnt signaling in the livers of endotoxemic mice. Protein and mRNA were extracted from the liver tissues of mice injected with 0 or 60 mg/kg LGK974 and 0 or 25 mg/kg LPS. **a** Levels of the Wnt pathway components were measured by western blotting. β-Actin and TBP were used as loading controls for total tissue lysates and nuclear lysates, respectively. Western blot raw image data are provided in Supplementary Data 5**. b**–**e** Band intensities were quantified using ImageJ. **f**–**i** Messenger RNA expression levels of Wnt ligands was measured by RT-qPCR. The data are presented as the mean ± standard deviation values of triplicate experiments. Differences among experimental groups were analyzed using one-way ANOVA with Duncan’s multiple range test. The different letters indicate significant differences (^*^P < 0.05).

We evaluated whether the Wnt signaling inhibitor LGK974 can modulate Wnt/β-catenin signaling, NF-κB signaling, and cytokine expression in the livers of endotoxemic mice. Mice were injected *i.p*. with 0 or 60 mg/kg LGK974 and 0 or 25 mg/kg LPS. Protein and RNA were then extracted from the livers of the mice, and the expression of components of the Wnt/β-catenin pathway was analyzed by western blotting and RT-qPCR. The results confirmed the GSEA data (Fig. 3f), showing that upregulated Wnt signaling in the livers of endotoxemic mice was inhibited by LGK974. The levels of phospho-LRP6 and phospho-GSK-3β, which were increased by LPS, were significantly reduced after LGK974 treatment (Fig. 5a, b). The total tissue and nuclear levels of β-catenin, the executor protein of the Wnt signaling pathway, were reduced by LGK974 (Fig. 5a, c, d). In addition, the level of β-TrCP, a target gene of β-catenin^37^, was reduced by LPK974 (Fig. 5a, e). The mRNA levels of *Wnt3a*, *Wnt5a*, *Wnt10a*, and *Wnt10b* in the livers of endotoxemic mice were reduced by LGK974 treatment (Fig. 5f–i). LGK974 alone had no effect on Wnt signaling. The dose-dependent effects of LGK974 on Wnt signaling in the livers of endotoxemic mice are shown in Supplementary Data 6.

**Fig. 6:**
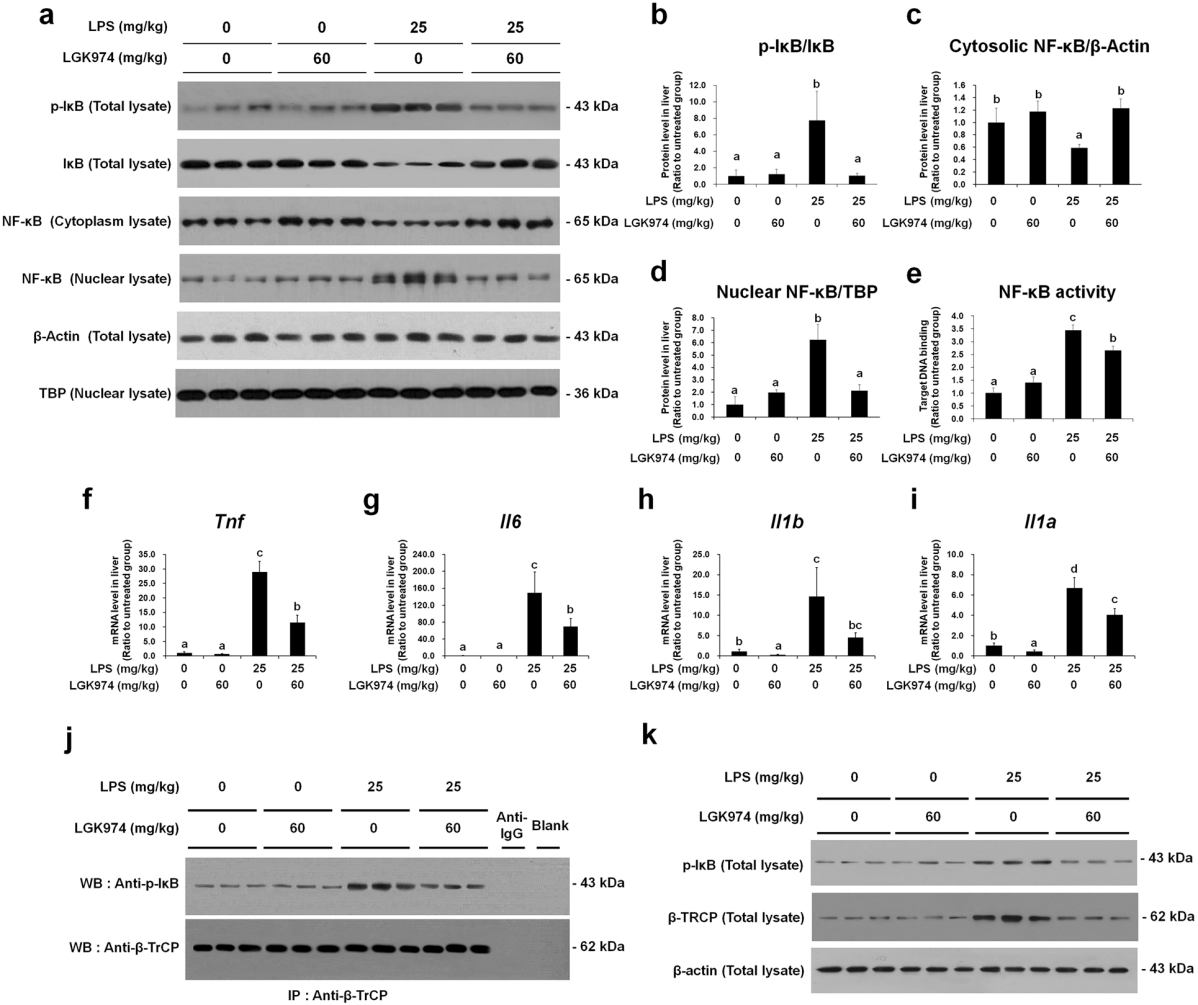
LGK974 inhibits the NF-κB pathway and reduces coimmunoprecipitation of phospho-IκB and β-TrCP in the livers of endotoxemic mice. Protein and RNA were extracted from the liver tissues of mice injected with 0 or 60 mg/kg LGK974 and 0 or 25 mg/kg LPS. **a** The levels of the NF-κB pathway components were measured by western blotting. β-Actin and TBP were used as loading controls for total tissue lysates and nuclear lysates, respectively. Complete western blot images are provided in Supplementary Data 5. **b**–**d** Band intensities were quantified using ImageJ. **e** The target DNA binding activity of NF-κB was measured via ELISA. **f**–**i** Messenger RNA levels of proinflammatory cytokines were measured by RT-qPCR. Differences among experimental groups were analyzed using one-way ANOVA with Duncan’s multiple range test. The different letters indicate significant differences (^*^P < 0.05). **j** The protein-protein interaction between phospho-IκB and β-TrCP was analyzed by coimmunoprecipitation experiments. The lane marked “Blank” shows coimmunoprecipitation using lysis buffer instead of tissue extract, and the lane marked “Anti-IgG” shows coimmunoprecipitation using an anti-mouse IgG secondary antibody instead of an anti-β-TrCP antibody. **k** Input samples from the coimmunoprecipitation assay were analyzed by western blotting. IP immunoprecipitation, WB western blot, β-TrCP β-transducin repeat-containing protein.

Next, using the same liver protein and RNA samples, we analyzed the expression of NF-κB pathway components and proinflammatory cytokines. The phospho-IκB level was decreased by LGK974 (Fig. 6a, b). The level of cytoplasmic NF-κB was increased but that of nuclear NF-κB was decreased by LGK974 (Fig. 6a, c, d). The binding affinity of NF-κB for its target DNA was significantly decreased by LGK974 (Fig. 6e), as were the transcript levels of genes encoding proinflammatory cytokines, such as *Tnf*, *Il6*, *Ilb*, and *Ila* (Fig. 6f–i). When mice were treated with LGK974 alone, no significant changes were observed in this group compared with the saline-treated control group. The dose-dependent effects of LGK974 on NF-κB signaling and proinflammatory cytokine expression are shown in Supplementary Data 7.

### LGK974 reduces the interaction between phospho-IκB and β-TrCP in the livers of mice with LPS-induced endotoxemia

We found that the protein-protein interaction between phospho-IκB and β-TrCP identified by coimmunoprecipitation was increased in mice with LPS-induced endotoxemia and decreased by LGK974 treatment (Fig. 6j). Western blot analysis of the input samples from the coimmunoprecipitation experiment showed that the protein levels of both β-TrCP and phospho-IκB were increased by LPS and decreased by LGK974 (Fig. 6k), the same pattern as that shown in Fig. 5e and Fig. 6b. Our data showed that the level of the protein–protein interaction between β-TrCP and phospho-IκB was proportional to the levels of these proteins in the livers of endotoxemic mice, which were reduced by LGK974.

To date, the interaction between β-TrCP and phospho-IκB has not been reported in the context of endotoxemia or sepsis. β-TrCP is a component of the IκB-ubiquitin ligase that induces the ubiquitination of phospho-IκB^43–45^; β-TrCP is a rate-limiting mediator that regulates the degradation of phospho-IκB, with increased binding resulting in increased degradation^46^. We suggest that the increased levels of β-TrCP and phospho-IκB and the interaction between these two proteins may induce IκB degradation followed by NF-κB nuclear translocation and proinflammatory cytokine expression in the livers of endotoxemic mice.

### LGK974 reduces the interaction between β-catenin and NF-κB in the livers of mice with LPS-induced endotoxemia

**Fig. 7:**
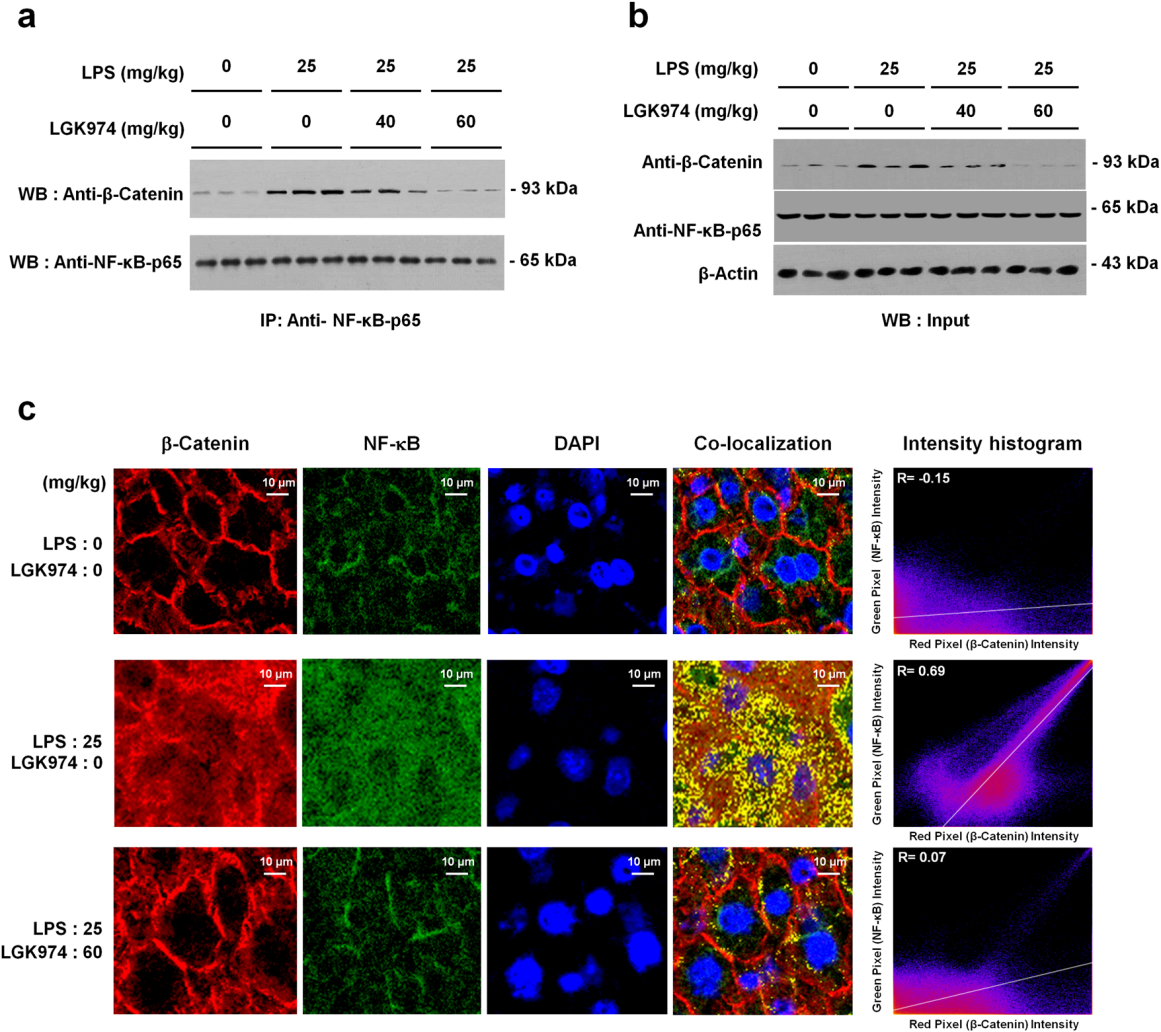
LGK974 reduces the coimmunoprecipitation and colocalization of β-catenin and NF-κB in the liver tissues of endotoxemic mice. **a**, **b** Protein was extracted from the liver tissues of mice injected with 0, 40, or 60 mg/kg LGK974 and 0 or 25 mg/kg LPS. The protein–protein interaction between β-catenin and NF-κB p65 was analyzed by coimmunoprecipitation (**a**). Input samples from the coimmunoprecipitation assay were analyzed by western blotting (**b**). IP immunoprecipitation, WB western blot. **c** Images showing immunofluorescence staining for β-catenin and NF-κB and DAPI staining for nuclei. All images were acquired at a magnification of 600× using a Nikon Asia confocal system (Nikon, Tokyo, Japan). Images are representative of three experiments. Colocalization was quantified using ImageJ to calculate Pearson correlation coefficient (*R*) values.

β-Catenin is the major effector protein of the Wnt/β-catenin pathway. When this pathway is inactive, β-catenin is phosphorylated by GSK-3β in a complex containing AXIN and adenomatous polyposis coli (APC) for subsequent proteolysis. Alternatively, when this pathway is active, phosphorylated LRP5/6 recruits AXIN and disrupts the GSK-3β–AXIN–APC complex, subsequently dephosphorylating and stabilizing β-catenin^47^. Because the levels of the Wnt/β-catenin and NF-κB signaling components were modulated by LPS and LGK974, the interaction between β-catenin and NF-κB was analyzed using coimmunoprecipitation and colocalization experiments. Coimmunoprecipitation of β-catenin and NF-κB was elevated by LPS; however, it was significantly reduced by LGK974 treatment (Fig. 7a). Data for input samples used in the coimmunoprecipitation assays are shown in Fig. 7b. We also assessed the colocalization of β-catenin and NF-κB by confocal fluorescence microscopy of liver tissues (Fig. 7c). In saline-treated control mice, β-catenin and NF-κB did not colocalize (*R* = −0.15); however, in endotoxemic mice, they did (*R* = 0.69), and this colocalization was suppressed by LGK974 treatment (*R* = 0.07).

The interaction between β-catenin and NF-κB during endotoxemia or sepsis has not been defined to date, even though recent studies have reported the existence of this interaction in other conditions. It has been reported that overexpression of β-catenin induces nuclear localization of NF-κB in cardiomyocytes during acute myocardial infarction^48^. In contrast, depletion of β-catenin by siRNA reduces LPS-induced NF-κB activation in human bronchial epithelial cells^49^. However, it should be noted that the interaction between β-catenin and NF-κB is complex; β-catenin exerts not only a proinflammatory function by enhancing NF-κB activity but also an anti-inflammatory function by repressing it. It has been reported that β-catenin reduces NF-κB activity in human articular chondrocytes^50^. The effect is often cell- and tissue-dependent or stimulus-specific and needs to be investigated in a context-dependent manner^51^.

### LGK974 reduces liver damage in mice with LPS-induced endotoxemia

**Fig. 8:**
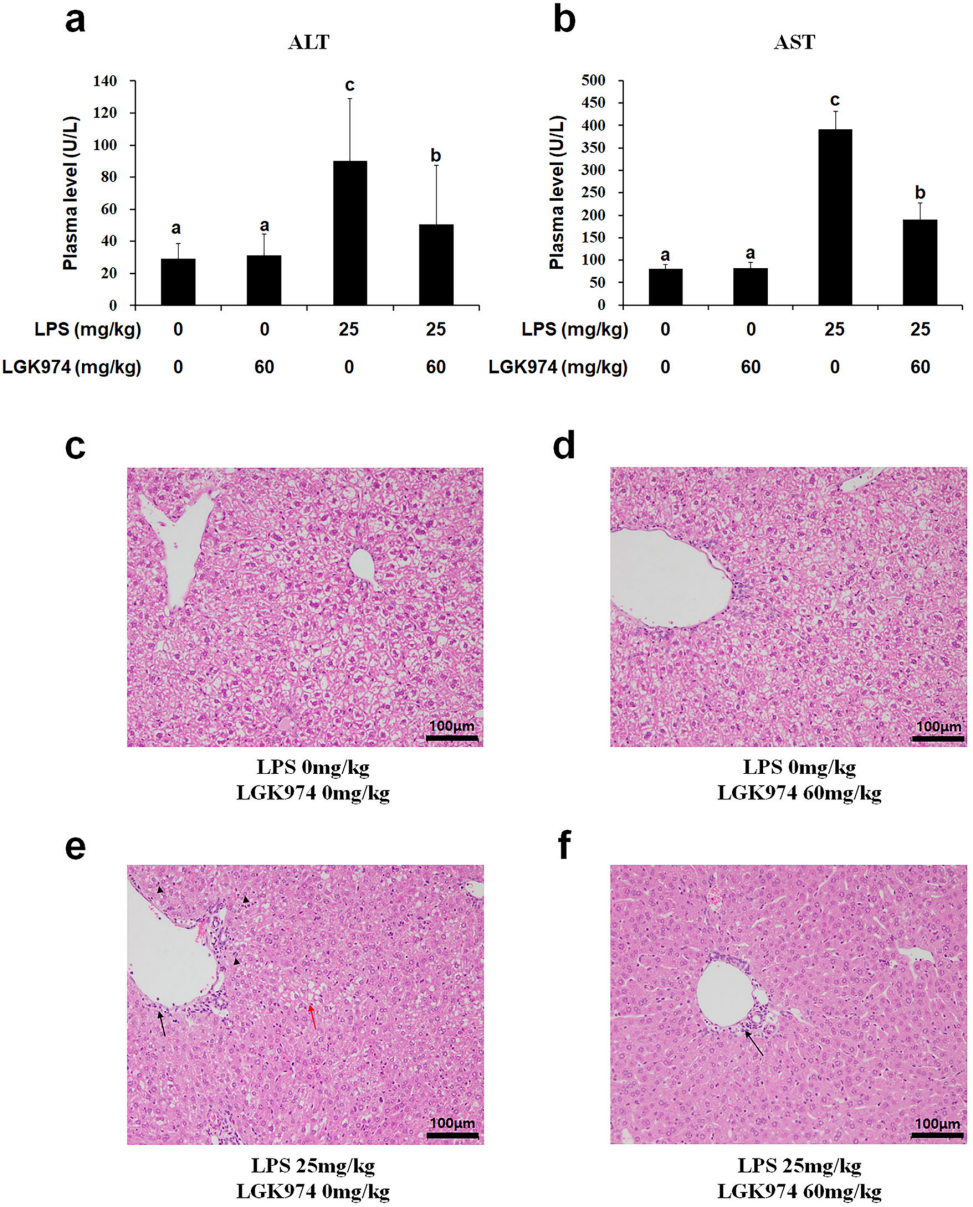
LGK974 suppressed the increases in ALT and AST levels and the pathological changes in the liver tissue of endotoxemic mice. Plasma and liver tissues were obtained from mice injected with 0 or 60 mg/kg LGK974 and 0 or 25 mg/kg LPS. **a**, **b** The plasma levels of ALT and AST, well-known markers of liver damage, were measured using a veterinary clinical chemistry analyzer. Differences among experimental groups were analyzed using one-way ANOVA with Duncan’s multiple range test. The different letters indicate statistically significant differences (^*^P < 0.05). **c**–**f** Pathological changes in liver tissues were observed using hematoxylin and eosin staining after formalin fixation. The black arrows indicate inflammatory cell infiltration. The black arrowheads show necrotic cells. The red arrow shows hepatocyte vacuolation. ALT alanine aminotransferase, AST aspartate aminotransferase.

We also found that the plasma levels of alanine aminotransferase (ALT) and aspartate aminotransferase (AST), well-known markers of liver damage, were elevated by LPS and significantly reduced by LGK974 (Fig. 8a, b). In addition, LPS-induced pathological changes in liver tissue, such as necrosis and vacuolation of hepatocytes as well as infiltration of inflammatory cells, were suppressed by LGK974 treatment (Fig. 8c–f).

Recent studies have suggested the involvement of Wnt signaling in sepsis^15^. It was reported that the levels of multiple WNT ligands were increased in the peripheral blood of patients with septic shock as well as in splenic tissue of endotoxemic mice^18^. WNT5A levels were significantly increased in the sera of patients with sepsis^17^ and in lung biopsies from patients with septic acute respiratory distress syndrome^52^. iCRT3, a Wnt signaling inhibitor, reportedly decreased the plasma levels of proinflammatory cytokines and ameliorated lung injury in a mouse model of sepsis induced by cecal ligation and puncture^16^. However, the crosstalk between the Wnt/β-catenin and NF-κB pathways has not been elucidated in sepsis.

In this study, we identified crosstalk between two pathways in an animal model of sepsis. Our results strongly suggest that the crosstalk between the Wnt/β-catenin and NF-κB pathways contributes to the mutual activation of these two pathways, which results in amplified proinflammatory cytokine production; damage to organs, including the liver; and death. LPS-induced NF-κB signaling may activate the Wnt pathway. Our results showed that the mRNA levels of Wnt ligands, including *Wnt3a*, *Wnt5a*, *Wnt10a*, and *Wnt10b*, correlated with the target DNA binding activity of NF-κB (Fig. 4e, j–m), as the promoters of some Wnt ligands have binding sites for NF-κB^38–42^. Wnt ligands, expressed by NF-κB, activated Wnt signaling and increased the levels of both β-catenin and β-TrCP (Fig. 4q, r), subsequently enhancing their protein-protein interactions with NF-κB (Fig. 7a, c) and phospho-IκB (Fig. 6j), which in turn activated NF-κB signaling.

**Fig. 9:**
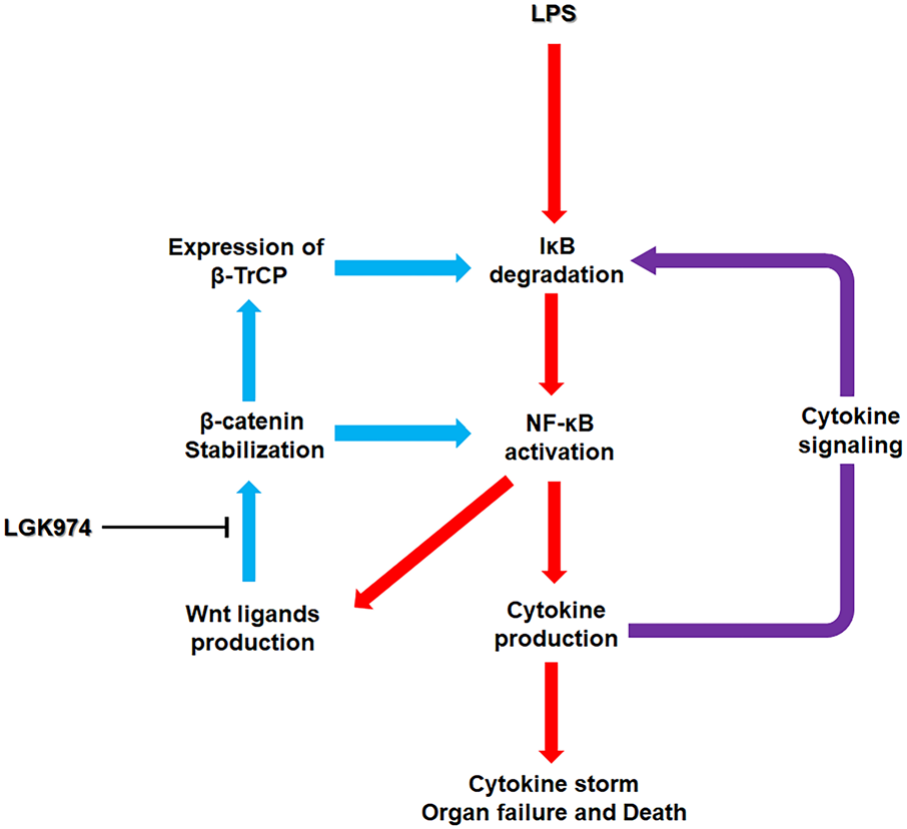
Possible mechanism of the crosstalk between the Wnt/β-catenin and NF-κB pathways during endotoxemia. The red arrows represent LPS-induced NF-κB signaling and Wnt ligand expression, and the blue arrows represent Wnt/β-catenin signaling along with its crosstalk with NF-κB signaling, both of which were observed in this study. The violet arrow represents cytokine-induced NF-κB pathway activation, which has been reported in previous studies. LGK974 exerts a suppressive effect on endotoxemia by suppressing the upregulation of β-catenin and β-TrCP as well as their interactions with NF-κB and phospho-IκB, respectively.

The findings of this study are summarized in Fig. 9. Our experimental data showed LPS-induced NF-κB signaling and Wnt ligand expression (red lines) as well as increased Wnt/β-catenin signaling and interactions between β-catenin and NF-κB and between β-TrCP and IκB (blue lines). LGK974 exerts a suppressive effect on cytokine production by suppressing the upregulation of β-catenin and β-TrCP as well as their interactions with NF-κB and phospho-IκB, respectively. Cytokine-induced NF-κB pathway activation during sepsis has been documented in previous studies (violet line)^53^.

We suggest that these interactions may occur in diverse cell types, including endothelial cells and macrophage-type cells in the liver. The liver is considered the main source of cytokine production during sepsis; Kupffer cells, which are macrophage-type cells in the liver, as well as natural killer cells, CD8+ T cells, endothelial cells, hepatic stellate cells, and hepatocytes, have been reported to participate in the production of proinflammatory cytokines during sepsis^14^. In our previous study, we showed that LGK974 suppressed proinflammatory cytokine production via modulation of the Wnt pathway in LPS-stimulated human endothelial cells^29^. Recently, we found that the protein–protein interaction between β-catenin and NF-κB was enhanced by LPS and reduced by LGK974 in RAW264.7 murine macrophage cells (unpublished observation). In this study, we did not analyze the types of cells in liver tissue, but various cell types, including endothelial cells and macrophage-type cells, may be involved in this process. Further study is necessary to confirm the results.

This study has some limitations. We primarily focused on liver dysfunction; thus, further studies are needed to analyze the effects on other organs, such as the lungs, heart, and kidneys. Furthermore, this study focused on the early phase of endotoxemia; most experiments were conducted using liver tissue and blood samples collected 6 h after LPS injection. Further studies are needed to analyze the time course and late phase of endotoxemia. The endotoxin and *E. coli* injection mouse models used in this study have been widely used for sepsis studies to date. As animal models of sepsis only partially recapitulate human sepsis, further studies employing more diverse animal models, including cecal ligation and puncture models, colon ascendent stent peritonitis models, pneumonia models, and implantation models, are needed^10^.

In conclusion, we demonstrated that the Wnt signaling inhibitor LGK974 downregulates proinflammatory cytokine production by modulating the crosstalk between the Wnt/β-catenin and NF-κB pathways, thereby suppressing the induction of cytokine storm, liver damage, and lethality in mice with LPS-induced endotoxemia. Our data suggest that LGK974 may be a therapeutic candidate for endotoxemia and sepsis.

## Supplementary information

Supplementary Data
