# Supplementary material for: LGK974 suppresses lipopolysaccharide-induced endotoxemia in mice by modulating the crosstalk between the Wnt/β-catenin and NF-κB pathways
Source: Exp Mol Med. 2021 Mar 10;53(3):407–21. doi: 10.1038/s12276-021-00577-z (PMC8080716; doi:10.1038/s12276-021-00577-z)
Supplement: Supplementary file 1 — Supplementary Data [file 12276_2021_577_MOESM1_ESM.pdf]

Supplementary Data

LGK974 suppresses lipopolysaccharide-induced endotoxemia in mice by modulating the crosstalk between the Wnt/β-catenin and NF-κB pathways

Jaewoong Jang[1], Jaewon Song[1], Hyunji Lee[1], Inae Sim[1], Young V. Kwon[2], Eek-hoon Jho[3*] & Yoosik Yoon[1,*]

[1] Department of Microbiology, Chung-Ang University College of Medicine, Seoul 06974, Republic of Korea

[2] Department of Biochemistry, University of Washington, Seattle, WA 98195, USA

[3] Department of Life Science, University of Seoul, Seoul 02504, Republic of Korea

*Corresponding authors: ej70@uos.ac.kr (E-h. Jho) and thanks@cau.ac.kr (Y. Yoon)

Running title: Anti-endotoxemic activity and mechanism of LGK974

Supplementary Data 1. Effect of LGK974 on the survival rate of female mice and 13-week-old mice 3

Supplementary Data 5. Original uncut images of western blots 4

Supplementary Data 6. Dose-dependent effects of LGK974 on Wnt signaling in the liver of endotoxemic mice 8

Supplementary Data 7. Dose-dependent effects of LGK974 on NF-κB pathway and proinflammatory cytokine expression in the liver of endotoxemic mice 9

* Supplementary Data 2. RNAseq data **10**

* Supplementary Data 3. GSEA data **267**

1. Enriched in Endotoxemia than Control 268
2. Enriched in Control than Endotoxemia 292
3. Enriched in LGK974 than Endotoxemia 311
4. Enriched in Endotoxemia than LGK974 329

* Supplementary Data 4. Gene list of selected GO terms **354**

1. Cytokine activity 355
2. IkB kinase NF-kB siganling 357
3. Negative regulation of Wnt signaling 360

* Supplementary Data 2, 3 and 4 are available as excel files upon request to thanks@cau.ac.kr

Supplementary Data 1. Effect of LGK974 on the survival rate of female mice and 13-week-old mice

We conducted the survival experiments in 4-week–old female mice (a) and 13-week-old male mice (b). LGK974 was found to increase the survival rate of both female and 13-week-old male endotoxemic mice, suggesting that the protective effect of LGK974 may not be related to the sex and age of mice. The effects of LGK974 on mice older than 13 weeks remain to be investigated in a further study.

a

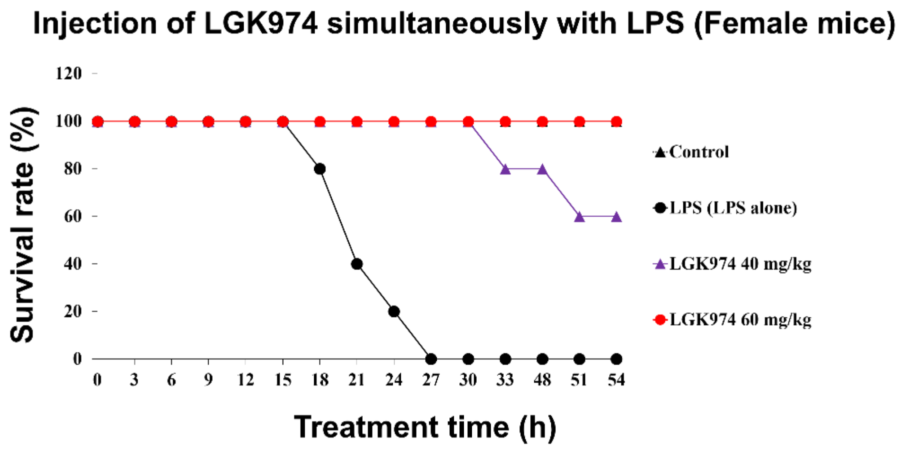

b

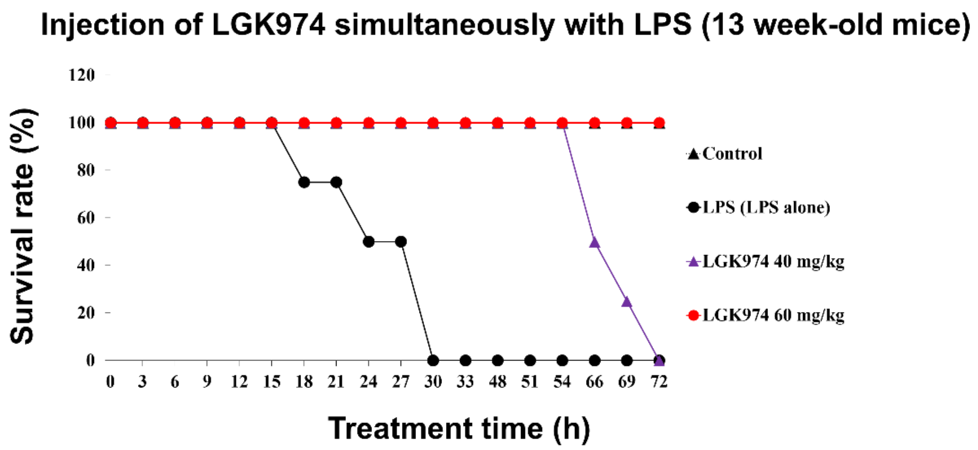

Supplementary Data 5. Original uncut images of western blots

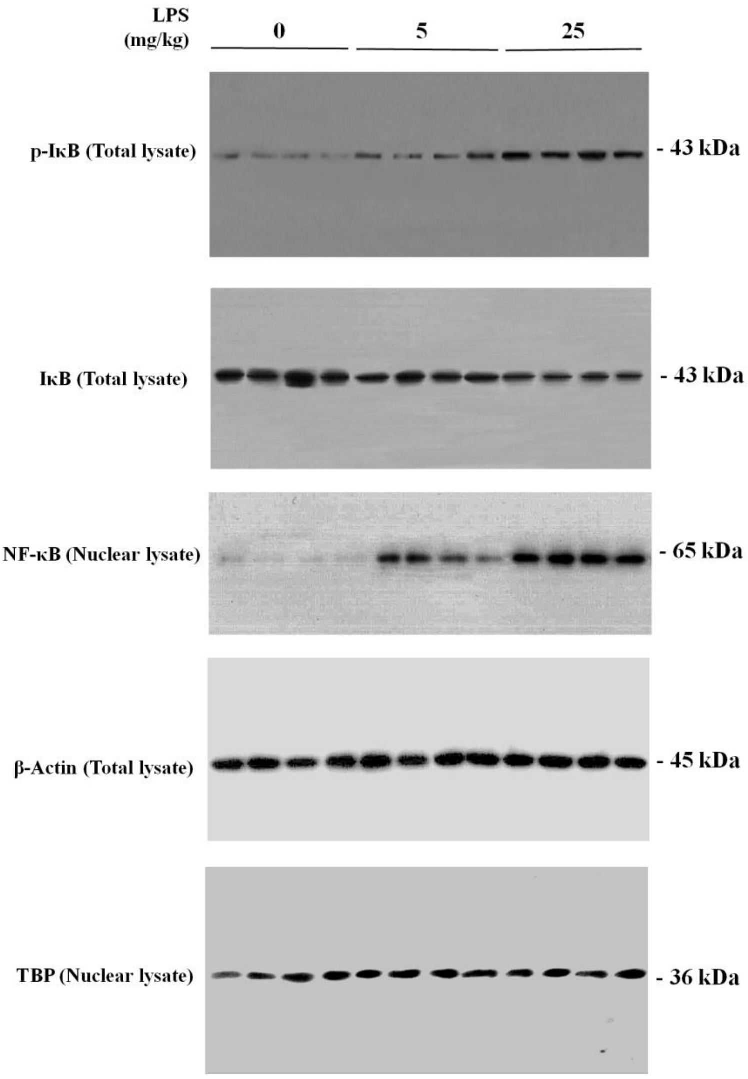

Fig 4a. The NF-κB pathway was upregulated in the liver tissue of LPS-induced endotoxemic mice. Protein was extracted from the liver tissues of mice injected with 0–25 mg/kg LPS. Western blotting was performed to measure phospho-IκB and IκB protein levels in the total tissue lysate and the expression of NF-κB protein was investigated in the nuclear lysate. β-Actin and TBP were used as loading controls for the total tissue lysate and nuclear lysate, respectively.

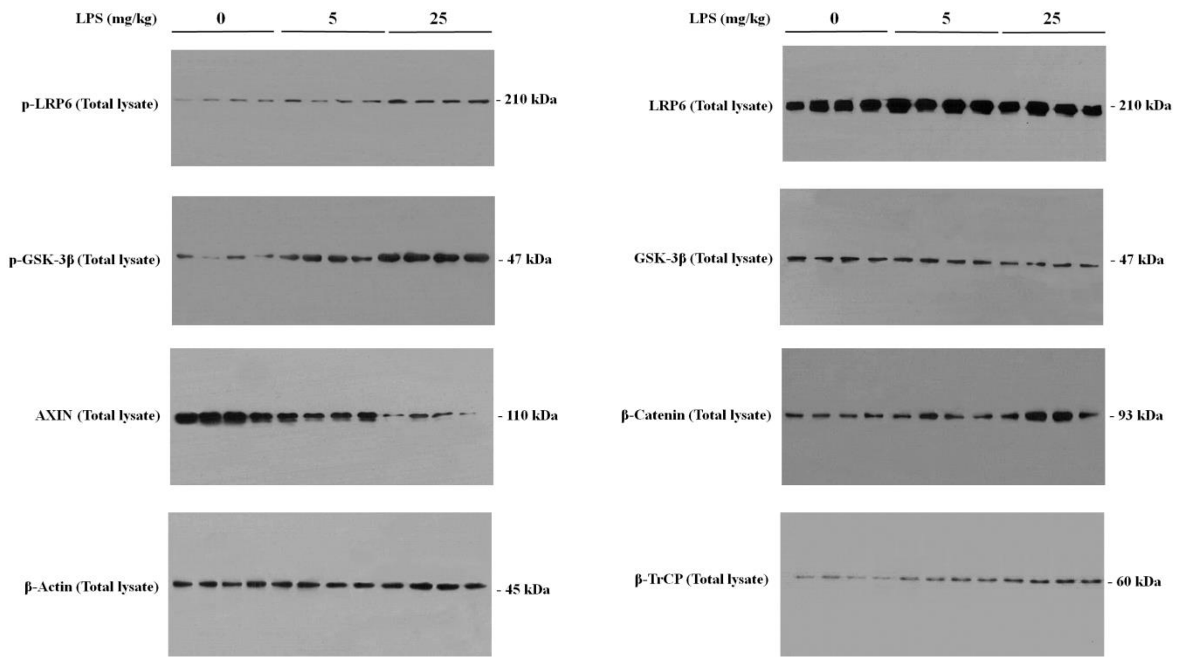

Fig 4n. The Wnt signaling pathway was upregulated in the liver tissue of LPS-induced endotoxemic mice. C57BL/6 mice were i.p. injected with 0–25 mg/kg LPS, and liver tissue was collected for protein extraction. The expression of the members of Wnt pathway was measured by western blotting. β-Actin was used as a loading control for the total tissue lysate.

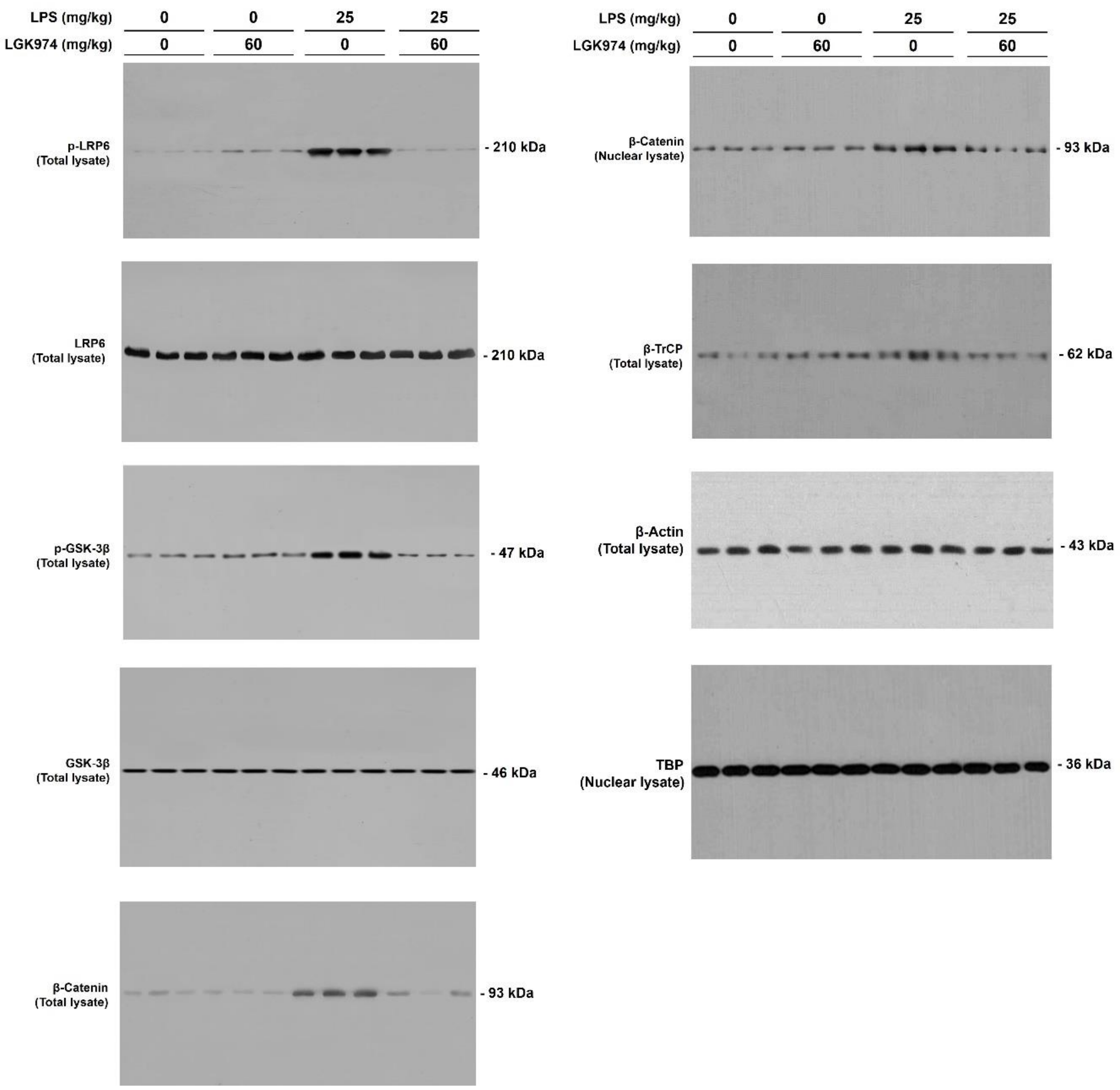

Fig 5a. LGK974 inhibited Wnt signaling in the liver tissues of endotoxemic mice. C57BL/6 mice were i.p. injected with 0 or 60 mg/kg LGK974 and 0 or 25 mg/kg LPS, and liver tissue was collected for protein extraction. The expression of the members of Wnt pathway was measured by western blotting. β-Actin and TBP were used as loading controls for the total tissue lysate and nuclear lysate, respectively.

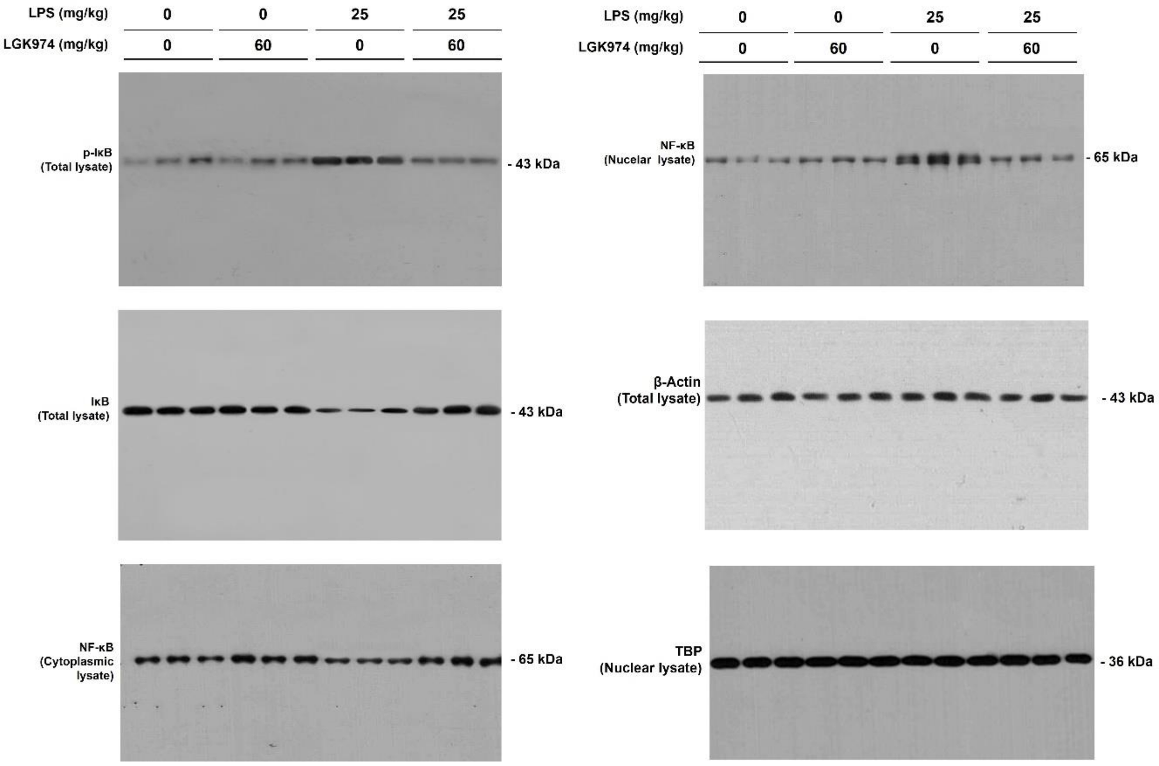

Fig 6a. LGK974 inhibited the NF-κB pathway in the liver tissues of endotoxemic mice. C57BL/6 mice were i.p. injected with 0 or 60 mg/kg LGK974 and 0 or 25 mg/kg LPS, and liver tissue was collected for protein extraction. The expression of the members of NF-κB pathway was measured by western blotting. β-Actin and TBP were used as loading controls for the total tissue lysate and nuclear lysate, respectively.

Supplementary Data 6. Dose-dependent effects of LGK974 on Wnt signaling in the liver of endotoxemic mice

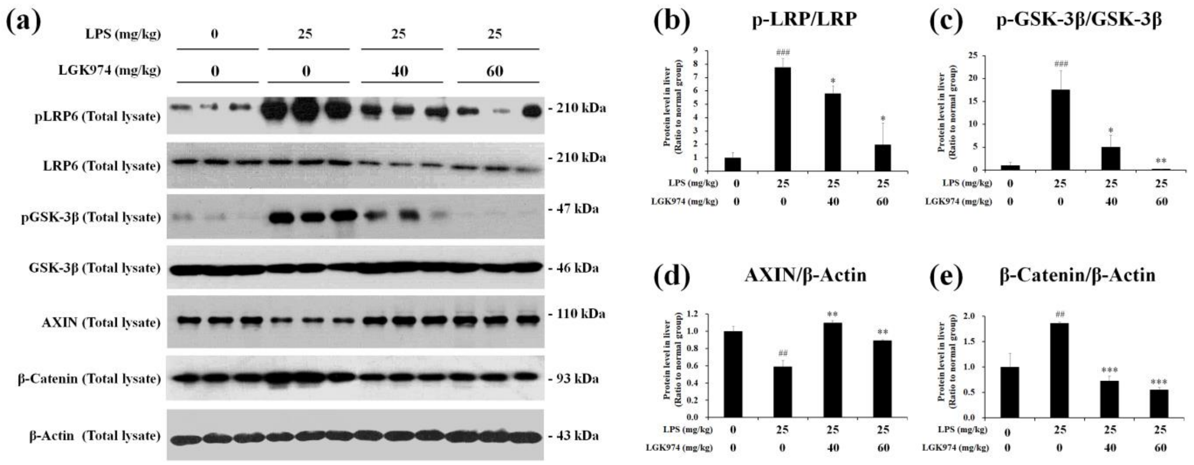

Proteins were extracted from the liver tissues of mice injected with 0, 40, or 60 mg/kg LGK974 and 25 mg/kg LPS. **(a)** The expression of the members of Wnt pathway was measured by western blotting. β-Actin was used as a loading control. **(b-e)** Band intensities were quantified using ImageJ. The data are presented as mean ± standard deviation of triplicate experiments. ***P* < 0.01, ****P* < 0.001 compared with the group injected with 25 mg/kg LPS. ##*P* < 0.01, ###*P* < 0.001 compared with the control group.

Supplementary Data 7. Dose-dependent effects of LGK974 on NF-κB pathway and proinflammatory cytokine expression in the liver of endotoxemic mice

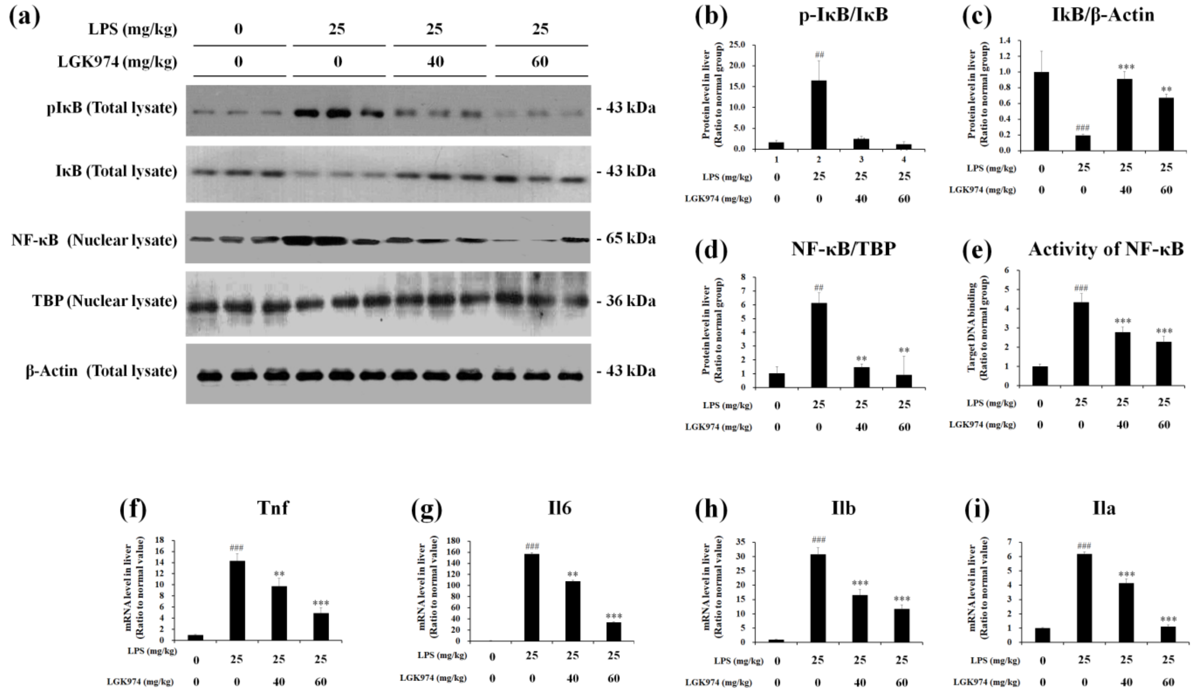

Proteins and RNA were extracted from the liver tissues of mice injected with 0, 40, or 60 mg/kg LGK974 and 25 mg/kg LPS. **(a)** The expression of the members of NF-κB pathway was measured by western blotting. β-Actin and TBP were used as loading controls for the total cell lysate and nuclear lysate, respectively. **(b-d)** Band intensities were quantified using ImageJ. (**e**) The binding affinity of NF-κB to its target DNA sequence was measured using ELISA. (**f-i)** Expression of genes encoding proinflammatory cytokines was measured by RT-qPCR. The data are presented as mean ± standard deviation of triplicate experiments. $^{**}P < 0.01$, $^{***}P < 0.001$ compared with the group injected with 25 mg/kg LPS. ##$P < 0.01$, ###$P < 0.001$ compared with the control group.

Supplementary Data 2. RNAseq data

Filter: 23282		Fold change			p-value			Average of normalized RC (log2)			Annotation									Gene information
ID	Gene symbol	Endotoxemic/ Normal	LGK974/ Endotoxemic	LGK974/ Normal	Endotoxemic/ Normal	LGK974/ Endotoxemic	LGK974/ Normal	Normal	Endotoxemic	LGK974	Entrez ID	Gene ID	chromosome	strand	transcript_id	p_id	bioType	Aliases	Description	NCBI search
1	Xkr4	0.716	0.995	0.712	0.182	1.000	0.423	0.490	0.008	0.000	497097	Xkr4	chr1	-	NM_0010	P15391	protein_cc	AY534250	X-linked K	Xkr4
2	Rp1	0.552	0.990	0.547	0.182	1.000	0.423	0.871	0.015	0.000	19888	Rp1	chr1	-	NM_0011	P17361	protein_cc	Dcdc3| m	retinitis pi	Rp1
3	Sox17	0.124	6.068	0.754	0.003	0.006	0.491	4.849	1.841	4.442	20671	Sox17	chr1	-	NM_0012	P1695	protein_cc	.	SRY (sex d	Sox17
4	Mrpl15	0.472	0.703	0.332	0.021	0.190	0.114	7.182	6.100	5.591	27395	Mrpl15	chr1	-	NR_03353	.	protein_cc	HSPC145|	mitochond	Mrpl15
5	Lypla1	0.389	1.634	0.635	0.003	0.004	0.149	10.090	8.728	9.436	18777	Lypla1	chr1	+	NM_0088	P6116	protein_cc	Pla1a	lysophosp	Lypla1
6	Tcea1	1.168	0.844	0.986	0.048	0.031	0.923	8.161	8.385	8.141	21399	Tcea1	chr1	+	NM_0011	P17796	protein_cc	S-II	transcripti	Tcea1
7	Rgs20	0.735	1.798	1.321	0.191	0.000	0.366	0.466	0.021	0.867	58175	Rgs20	chr1	-	NM_0012	P10335	protein_cc	2900073E	regulator	Rgs20
8	Atp6v1h	1.212	1.081	1.311	0.179	0.640	0.323	8.642	8.920	9.033	108664	Atp6v1h	chr1	+	NM_1338	P23793	protein_cc	0710001F	ATPase, H	Atp6v1h
9	Oprk1	1.000	1.000	1.000	1.000	1.000	1.000	0.000	0.000	0.000	18387	Oprk1	chr1	+	NM_0110	P20526	protein_cc	K-OR-1| K	opioid rec	Oprk1
10	Npbwr1	1.005	1.439	1.446	0.000	0.182	0.423	0.000	0.008	0.532	226304	Npbwr1	chr1	-	NM_0103	P8280	protein_cc	Gpr7| Nbp	neuropept	Npbwr1
11	Rb1cc1	0.876	0.766	0.671	0.047	0.005	0.023	9.681	9.490	9.106	12421	Rb1cc1	chr1	+	NM_0098	P16634	protein_cc	2900055E	RB1-induc	Rb1cc1
12	Fam150a	1.000	1.000	1.000	1.000	1.000	1.000	0.000	0.000	0.000	620393	Fam150a	chr1	+	NM_0011	P19323	protein_cc	EG620393	family wit	Fam150a
13	St18	1.000	1.000	1.000	1.000	1.000	1.000	0.000	0.000	0.000	240690	St18	chr1	+	NM_0012	P26336	protein_cc	AV348974	suppressio	St18
14	Pcmtd1	0.436	0.774	0.338	0.000	0.106	0.014	11.431	10.234	9.864	319263	Pcmtd1	chr1	+	NM_1830	P18329	protein_cc	8430411F	protein-L-	Pcmtd1
15	Sntg1	1.027	0.797	0.818	0.938	0.391	0.804	1.110	1.149	0.821	71096	Sntg1	chr1	-	NM_0012	P6945	protein_cc	4933426D	syntrophin	Sntg1
16	Rrs1	2.113	0.969	2.047	0.004	0.765	0.040	7.984	9.064	9.018	59014	Rrs1	chr1	+	NM_0215	P15667	protein_cc	5730466A	ribosome	Rrs1
17	Adhfe1	1.192	0.758	0.904	0.124	0.120	0.722	8.793	9.047	8.648	76187	Adhfe1	chr1	+	NR_02766	.	protein_cc	6330565B	alcohol de	Adhfe1
18	2610203C	1.329	0.361	0.480	0.002	0.000	0.022	4.054	4.464	2.994	72481	2610203C	chr1	-	NR_01547	.	lncRNA	2310007M	RIKEN cDN	2610203C
19	3110035E	11.191	1.048	11.723	0.000	0.745	0.043	0.002	3.486	3.553	76982	3110035E	chr1	+	NM_1783	P4914	protein_cc	AI415019|	RIKEN cDN	3110035E1
20	Mybl1	19.259	0.847	16.304	0.022	0.719	0.246	0.988	5.255	5.015	17864	Mybl1	chr1	-	NM_0086	P12430	protein_cc	A-myb| G	myeloblas	Mybl1
21	Vcpip1	1.393	0.741	1.033	0.006	0.093	0.908	8.242	8.721	8.289	70675	Vcpip1	chr1	-	NM_1734	P3023	protein_cc	4932442A	valosin co	Vcpip1
22	1700034P	1.276	0.889	1.135	0.677	0.740	0.891	2.457	2.809	2.639	73331	1700034P	chr1	+	NR_04046	.	lncRNA	.	RIKEN cDN	1700034P1
23	Sgk3	0.893	0.890	0.794	0.386	0.434	0.048	6.886	6.723	6.554	170755	Sgk3	chr1	+	NM_1332	P19798	protein_cc	2510015P	serum/glu	Sgk3
24	Mcmdc2	1.000	1.000	1.000	1.000	1.000	1.000	0.000	0.000	0.000	240697	Mcmdc2	chr1	+	NM_1777	P17023	protein_cc	6030422M	minichrom	Mcmdc2
25	Snhg6	1.279	3.305	4.227	0.737	0.243	0.430	7.184	7.539	9.264	73824	Snhg6	chr1	-	NR_02406	.	lncRNA	1110008H	small nucl	Snhg6
26	Snord87	5.817	0.346	2.013	0.005	0.013	0.264	3.007	5.548	4.017	266793	Snord87	chr1	-	NR_00441	.	snoRNA	MBII-276|	small nucl	Snord87
27	Tcf24	0.251	1.376	0.346	0.005	0.467	0.149	6.258	4.266	4.726	10003959	Tcf24	chr1	-	NM_0012	P13148	protein_cc	10003959	transcripti	Tcf24
28	Ppp1r42	0.828	0.725	0.600	0.536	0.005	0.439	5.027	4.754	4.290	69312	Ppp1r42	chr1	-	NM_1456	P18655	protein_cc	1700011J1	protein ph	Ppp1r42
29	Cops5	1.048	1.389	1.456	0.535	0.007	0.033	9.665	9.734	10.208	26754	Cops5	chr1	-	NR_10228	.	protein_cc	AI303502|	COP9 sign	Cops5
30	Cspp1	0.779	0.765	0.596	0.284	0.442	0.224	7.312	6.952	6.566	211660	Cspp1	chr1	+	NM_0264	P17010	protein_cc	2310020J1	centrosom	Cspp1
31	Arfgef1	0.833	0.948	0.790	0.017	0.117	0.072	8.984	8.721	8.644	211673	Arfgef1	chr1	-	NM_0011	P12121	protein_cc	ARFGEP1|	ADP-ribos	Arfgef1
32	Cpa6	1.000	1.000	1.000	1.000	1.000	1.000	0.000	0.000	0.000	329093	Cpa6	chr1	-	NM_0012	P15515	protein_cc	9030616D	carboxype	Cpa6
33	Prex2	0.838	1.295	1.086	0.449	0.226	0.726	8.444	8.189	8.562	109294	Prex2	chr1	+	NM_0295	P4722	protein_cc	6230420N	phosphati	Prex2
34	A830018L	1.000	1.000	1.000	1.000	1.000	1.000	0.000	0.000	0.000	320492	A830018L	chr1	+	NM_0011	P1699	protein_cc	.	RIKEN cDN	A830018L1
35	Gm17644	0.716	0.995	0.712	0.182	1.000	0.423	0.490	0.008	0.000	329096	Gm17644	chr1	+	NR_04529	.	lncRNA	4932411L	predicted	Gm17644
36	Sulf1	1.190	0.253	0.302	0.838	1.000	0.422	1.729	1.980	0.000	240725	Sulf1	chr1	+	NM_0011	P26541	protein_cc	AI467640|	sulfatase 1	Sulf1
37	Slco5a1	0.448	1.836	0.822	0.002	0.187	0.702	1.193	0.033	0.910	240726	Slco5a1	chr1	-	NM_1728	P3874	protein_cc	4732454B	solute car	Slco5a1
38	Prdm14	2.134	0.469	1.000	0.305	1.000	0.423	0.000	1.094	0.000	383491	Prdm14	chr1	-	NM_0010	P25940	protein_cc	.	PR domain	Prdm14
39	Ncoa2	1.670	1.229	2.053	0.000	0.410	0.264	7.123	7.863	8.161	17978	Ncoa2	chr1	-	NM_0013	P16813	protein_cc	bHLHe75|	nuclear re	Ncoa2
40	Tram1	1.042	1.161	1.210	0.713	0.163	0.121	10.206	10.265	10.481	72265	Tram1	chr1	-	NM_0281	P3457	protein_cc	1810049E	translocat	Tram1
41	Lactb2	0.357	2.039	0.728	0.003	0.007	0.286	8.644	7.158	8.186	212442	Lactb2	chr1	-	NM_1453	P26788	protein_cc	Cgi-83| E4	lactamase	Lactb2
42	Xkr9	0.330	1.741	0.575	0.001	0.388	0.414	5.622	4.023	4.823	381246	Xkr9	chr1	+	NM_0010	P14456	protein_cc	Gm1620|	X-linked K	Xkr9
43	Gm5523	0.746	0.957	0.714	0.456	0.922	0.063	3.579	3.156	3.093	433273	Gm5523	chr1	-	NR_00444	.	misc_RNA	EG433273	predicted	Gm5523
44	Eya1	1.005	1.405	1.412	0.000	0.182	0.423	0.000	0.008	0.498	14048	Eya1	chr1	-	NM_0012	P2118	protein_cc	bor	EYA transc	Eya1
45	Msc	0.607	1.441	0.875	0.176	0.323	0.657	3.284	2.564	3.090	17681	Msc	chr1	-	NM_0108	P2949	protein_cc	bHLHa22|	musculin	Msc
46	Trpa1	1.000	1.000	1.000	1.000	1.000	1.000	0.000	0.000	0.000	277328	Trpa1	chr1	-	NM_1777	P3256	protein_cc	Anktm1| T	transient r	Trpa1
47	Kcnb2	1.000	1.000	1.000	1.000	1.000	1.000	0.000	0.000	0.000	98741	Kcnb2	chr1	+	NM_0010	P6376	protein_cc	9630047L	potassium	Kcnb2
48	Terf1	1.065	0.788	0.839	0.731	0.264	0.406	6.370	6.461	6.118	21749	Terf1	chr1	+	NM_0093	P3266	protein_cc	Pin2| Trbf	telomeric	Terf1
49	Sbspon	0.552	0.990	0.547	0.182	1.000	0.423	0.871	0.015	0.000	226866	Sbspon	chr1	-	NM_0010	P7413	protein_cc	Gm106| R	somatome	Sbspon
50	4930444P	1.005	1.405	1.412	0.000	0.182	0.423	0.000	0.008	0.498	75799	4930444P	chr1	-	NM_0012	P5023	protein_cc	.	RIKEN cDN	4930444P1
51	Rpl7	1.337	0.863	1.154	0.000	0.092	0.374	9.828	10.247	10.035	19989	Rpl7	chr1	-	NM_0112	P20503	protein_cc	Rpl7a| Sur	ribosomal	Rpl7
52	Rdh10	0.945	0.595	0.562	0.502	0.016	0.121	8.072	7.991	7.241	98711	Rdh10	chr1	+	NM_1338	P9642	protein_cc	3110069K	retinol del	Rdh10
53	Stau2	0.322	1.905	0.613	0.003	0.089	0.293	4.984	3.349	4.279	29819	Stau2	chr1	-	NM_0011	P4878	protein_cc	.	staufen (R	Stau2
54	Ube2w	1.156	0.697	0.806	0.018	0.006	0.169	7.055	7.264	6.744	66799	Ube2w	chr1	-	NM_0012	P3981	protein_cc	6130401J	ubiquitin-c	Ube2w
55	Tceb1	2.206	0.879	1.939	0.013	0.451	0.074	9.105	10.246	10.060	67923	Tceb1	chr1	-	NM_0264	P12574	protein_cc	2610043E	transcripti	Tceb1
56	D030040B	1.005	1.405	1.412	0.000	0.182	0.423	0.000	0.008	0.498	329101	D030040B	chr1	+	NR_03799	.	lncRNA	D030040B	RIKEN cDN	D030040B
57	Tmem70	1.359	0.900	1.223	0.001	0.053	0.110	9.269	9.712	9.559	70397	Tmem70	chr1	+	NM_0274	P12011	protein_cc	1110020A	transmem	Tmem70
58	Ly96	2.559	0.898	2.298	0.000	0.457	0.127	5.436	6.792	6.637	17087	Ly96	chr1	+	NM_0011	P23854	protein_cc	ESOP-1| M	lymphocyt	Ly96
59	Jph1	0.528	2.732	1.444	0.175	0.021	0.261	4.257	3.337	4.787	57339	Jph1	chr1	-	NM_0206	P8024	protein_cc	ENSMUSG	junctophil	Jph1
60	Gdap1	1.005	1.439	1.446	0.000	0.182	0.423	0.000	0.008	0.532	14545	Gdap1	chr1	+	NM_0102	P21457	protein_cc	.	gangliosid	Gdap1
61	Pi15	25.471	1.641	41.809	0.000	0.005	0.013	0.988	5.658	6.373	94227	Pi15	chr1	+	NM_0531	P26779	protein_cc	P24TI| P2	peptidase	Pi15
62	Gm16070	1.000	1.000	1.000	1.000	1.000	1.000	0.000	0.000	0.000	10263491	Gm16070	chr1	-	NR_13031	.	lncRNA	OTTMUSG	predicted	Gm16070
63	Crispld1	0.928	1.880	1.745	0.863	0.351	0.611	0.804	0.697	1.608	83691	Crispld1	chr1	+	NM_0314	P1116	protein_cc	Cocoacrisp	cysteine-r	Crispld1
64	Crisp4	1.000	1.000	1.000	1.000	1.000	1.000	0.000	0.000	0.000	78081	Crisp4	chr1	-	NM_0300	P21530	protein_cc	9230112K	cysteine-r	Crisp4
65	Defb44-ps	1.000	1.000	1.000	1.000	1.000	1.000	0.000	0.000	0.000	654454	Defb44-ps	chr1	-	NR_00287	.	misc_RNA	Defb44| D	defensin b	Defb44-ps
66	Defb18	1.000	1.000	1.000	1.000	1.000	1.000	0.000	0.000	0.000	654460	Defb18	chr1	-	NM_0010	P10352	protein_cc	BD-18| EG	defensin b	Defb18
67	Defb41	1.000	1.000	1.000	1.000	1.000	1.000	0.000	0.000	0.000	77673	Defb41	chr1	-	NM_1831	P22650	protein_cc	9230102D	defensin b	Defb41
68	Gm15386	1.000	1.000	1.000	1.000	1.000	1.000	0.000	0.000	0.000	.	Gm15386	chr1	-	NM_0010	P14244	.	.	.	Gm15386
69	Tfap2d	1.000	1.000	1.000	1.000	1.000	1.000	0.000	0.000	0.000	226896	Tfap2d	chr1	+	NM_1531	P24869	protein_cc	Tcfap2d	transcripti	Tfap2d
70	Tfap2b	0.325	0.978	0.318	0.039	1.000	0.201	1.655	0.033	0.000	21419	Tfap2b	chr1	+	NM_0012	P16725	protein_cc	AI606113|	transcripti	Tfap2b
71	Pkhd1	0.710	0.734	0.521	0.168	0.008	0.227	3.643	3.149	2.703	241035	Pkhd1	chr1	-	NM_1531	P25486	protein_cc	AI118496|	polycystic	Pkhd1
72	4930486I0	1.000	1.000	1.000	1.000	1.000	1.000	0.000	0.000	0.000	75028	4930486I0	chr1	+	NR_04573	.	lncRNA	.	RIKEN cDN	4930486I0
73	Linc-md1	1.000	1.000	1.000	1.000	1.000	1.000	0.000	0.000	0.000	10566173	Linc-md1	chr1	+	NR_13124	.	lncRNA	.	long interg	Linc-md1
74	Il17a	1.000	1.000	1.000	1.000	1.000	1.000	0.000	0.000	0.000	16171	Il17a	chr1	+	NM_0105	P7458	protein_cc	Ctla-8| Ctl	interleukin	Il17a
75	Il17f	1.000	1.000	1.000	1.000	1.000	1.000	0.000	0.000	0.000	257630	Il17f	chr1	-	NM_1458	P17855	protein_cc	C87042| Il	interleukin	Il17f
76	Mcm3	1.507	1.213	1.828	0.010	0.086	0.044	6.094	6.685	6.964	17215	Mcm3	chr1	-	NM_0085	P22539	protein_cc	AL033361	minichrom	Mcm3
77	6720483E	1.000	1.000	1.000	1.000	1.000	1.000	0.000	0.000	0.000	77741	6720483E	chr1	-	NR_04049	.	lncRNA	.	RIKEN cDN	6720483E
78	Paqr8	1.616	0.855	1.383	0.460	0.794	0.636	0.432	1.125	0.899	74229	Paqr8	chr1	+	NM_0288	P7910	protein_cc	1700019B	progestin	Paqr8

79	Efhc1	2.312	0.231	0.534	0.203	0.087	0.523	1.303	2.512	0.397	71877	Efhc1	chr1	+	NM_0279	P12286	protein_cc	1700029F:	EF-hand d	Efhc1
80	Tram2	0.694	0.143	0.099	0.615	1.000	0.149	3.334	2.808	0.000	170829	Tram2	chr1	-	NM_1774	P20074	protein_cc	C330003D	translocat	Tram2
81	Tmem14a	0.281	0.373	0.105	0.014	0.172	0.063	7.719	5.891	4.468	75712	Tmem14a	chr1	+	NM_0293	P19939	protein_cc	5730496E:	transmem	Tmem14a
82	Gsta3	0.474	0.681	0.323	0.004	0.199	0.070	13.937	12.860	12.305	14859	Gsta3	chr1	+	NM_0010	P12529	protein_cc	Gst2-3	glutathion	Gsta3
83	Gm4956	0.480	2.080	0.998	0.027	0.052	0.993	3.890	2.830	3.887	241041	Gm4956	chr1	-	NR_00285	.	misc_RNA	EG241041	predicted	Gm4956
84	Khdc1a	1.000	1.000	1.000	1.000	1.000	1.000	0.000	0.000	0.000	368204	Khdc1a	chr1	+	NM_1833	P6204	protein_cc	AU014713	KH domai	Khdc1a
85	Khdc1c	1.000	1.000	1.000	1.000	1.000	1.000	0.000	0.000	0.000	433278	Khdc1c	chr1	+	NM_0010	P22928	protein_cc	Khdc1b	KH domai	Khdc1c
86	Khdc1b	1.000	1.000	1.000	1.000	1.000	1.000	0.000	0.000	0.000	98582	Khdc1b	chr1	+	NM_0011	P19992	protein_cc	AU014713	KH domai	Khdc1b
87	Kcnq5	0.560	1.371	0.768	0.184	0.191	0.687	0.857	0.021	0.477	226922	Kcnq5	chr1	-	NM_0011	P22432	protein_cc	7730402H	potassium	Kcnq5
88	Rims1	1.000	1.000	1.000	1.000	1.000	1.000	0.000	0.000	0.000	116837	Rims1	chr1	-	NM_1830	P20713	protein_cc	C030033M	regulating	Rims1
89	4933415F	1.005	1.405	1.412	0.000	0.182	0.423	0.000	0.008	0.498	66755	4933415F:	chr1	-	NM_0257	P19747	protein_cc	.	RIKEN cDN	4933415F2
90	Ogfrl1	0.863	1.379	1.190	0.141	0.040	0.281	8.652	8.440	8.903	70155	Ogfrl1	chr1	-	NM_0010	P8910	protein_cc	2210417C	opioid gro	Ogfrl1
91	B3gat2	1.300	0.983	1.278	0.083	0.881	0.126	7.794	8.173	8.148	280645	B3gat2	chr1	+	NM_1721	P4089	protein_cc	GlcAT-S| (	beta-1,3-g	B3gat2
92	Smap1	1.164	0.925	1.077	0.079	0.191	0.414	9.192	9.411	9.299	98366	Smap1	chr1	-	NR_11097	.	protein_cc	1700056O	small ArfG	Smap1
93	Sdhaf4	0.410	2.161	0.887	0.000	0.004	0.497	9.059	7.774	8.886	68002	Sdhaf4	chr1	-	NM_0265	P25383	protein_cc	1110058L:	succinate	Sdhaf4
94	Fam135a	1.553	0.952	1.479	0.101	0.794	0.057	7.613	8.248	8.178	68187	Fam135a	chr1	-	NM_0266	P3306	protein_cc	4921533L:	family wit	Fam135a
95	Col9a1	1.000	1.000	1.000	1.000	1.000	1.000	0.000	0.000	0.000	12839	Col9a1	chr1	+	NM_0077	P14088	protein_cc	Col9a-1	collagen, t	Col9a1
96	Col19a1	0.716	0.995	0.712	0.182	1.000	0.423	0.490	0.008	0.000	12823	Col19a1	chr1	-	NM_0077	P8637	protein_cc	.	collagen, t	Col19a1
97	Lmbrd1	0.649	1.184	0.768	0.004	0.129	0.023	9.557	8.933	9.176	68421	Lmbrd1	chr1	+	NM_0267	P2330	protein_cc	0910001K	LMBR1 do	Lmbrd1
98	Adgrb3	1.000	1.000	1.000	1.000	1.000	1.000	0.000	0.000	0.000	210933	Adgrb3	chr1	-	NM_1756	P3687	protein_cc	A830096D	adhesion (	Adgrb3
99	Gm20172	1.000	1.000	1.000	1.000	1.000	1.000	0.000	0.000	0.000	10050432	Gm20172	chr1	-	NM_0012	P461	protein_cc	.	predicted	Gm20172
100	4931408C	1.000	1.000	1.000	1.000	1.000	1.000	0.000	0.000	0.000	210940	4931408C	chr1	-	NM_0010	P18189	protein_cc	.	RIKEN cDN	4931408C2
101	Gm597	0.716	0.995	0.712	0.182	1.000	0.423	0.490	0.008	0.000	210962	Gm597	chr1	-	NM_0010	P4916	protein_cc	.	predicted	Gm597
102	Phf3	0.933	0.825	0.770	0.175	0.014	0.117	9.913	9.813	9.536	213109	Phf3	chr1	-	NM_0010	P2300	protein_cc	2310061N	PHD finge	Phf3
103	Ptp4a1	1.867	0.801	1.495	0.000	0.015	0.088	10.123	11.023	10.703	19243	Ptp4a1	chr1	-	NM_0112	P13001	protein_cc	AA415290	protein ty	Ptp4a1
104	Gm13363	2.188	0.709	1.552	0.000	0.013	0.133	9.490	10.620	10.124	433406	Gm13363	chr1	-	NR_00268	.	misc_RNA	OTTMUSG	predicted	Gm13363
105	4931428L	1.000	1.000	1.000	1.000	1.000	1.000	0.000	0.000	0.000	70988	4931428L:	chr1	-	NR_03344	.	lncRNA	4930563O	RIKEN cDN	4931428L1
106	Lgsn	1.000	1.000	1.000	1.000	1.000	1.000	0.000	0.000	0.000	266744	Lgsn	chr1	+	NM_1536	P4809	protein_cc	Gluld1| le	lengsin, le	Lgsn
107	Pih1d3	1.000	1.000	1.000	1.000	1.000	1.000	0.000	0.000	0.000	74708	Pih1d3	chr1	+	NM_0290	P18607	protein_cc	4930521A	PIH1 dom	Pih1d3
108	Gm4850	1.000	1.000	1.000	1.000	1.000	1.000	0.000	0.000	0.000	226957	Gm4850	chr1	+	NR_01534	.	misc_RNA	EG226957	predicted	Gm4850
109	Khdrbs2	1.000	1.000	1.000	1.000	1.000	1.000	0.000	0.000	0.000	170771	Khdrbs2	chr1	+	NM_1332	P24307	protein_cc	6330586C	KH domai	Khdrbs2
110	Gm9839	1.000	1.000	1.000	1.000	1.000	1.000	0.000	0.000	0.000	408192	Gm9839	chr1	-	NM_0011	P22181	protein_cc	ENSMUSG	predicted	Gm9839
111	Gm5415	1.000	1.000	1.000	1.000	1.000	1.000	0.000	0.000	0.000	408191	Gm5415	chr1	-	NM_0011	P1922	protein_cc	EG408191	predicted	Gm5415
112	Prim2	0.904	1.676	1.516	0.767	0.047	0.274	4.316	4.171	4.916	19076	Prim2	chr1	-	NM_0089	P16482	protein_cc	AI323589	DNA prima	Prim2
113	1700001G	1.005	1.439	1.446	0.000	0.182	0.423	0.000	0.008	0.532	67503	1700001G	chr1	-	NR_03319	.	lncRNA		RIKEN cDN	1700001G1
114	Rab23	0.534	1.244	0.664	0.029	0.603	0.408	5.752	4.846	5.160	19335	Rab23	chr1	+	NM_0089	P5526	protein_cc	AW54538	RAB23, me	Rab23
115	Bag2	0.372	1.340	0.498	0.048	0.446	0.260	4.766	3.338	3.760	213539	Bag2	chr1	-	NM_1453	P2887	protein_cc	2610042A	BCL2-asso	Bag2
116	Zfp451	0.820	0.914	0.749	0.441	0.693	0.371	6.883	6.596	6.467	98403	Zfp451	chr1	-	NM_1338	P14575	protein_cc	4930515K	zinc finger	Zfp451
117	Gm15455	1.135	0.407	0.462	0.331	0.002	0.161	4.023	4.206	2.910	433287	Gm15455	chr1	-	NM_0011	P15901	protein_cc	AA692849	predicted	Gm15455
118	Bend6	0.944	0.784	0.740	0.935	0.714	0.740	1.552	1.469	1.119	320705	Bend6	chr1	-	NM_1772	P26893	protein_cc	B230209C	BEN doma	Bend6
119	Dst	2.003	0.681	1.365	0.000	0.001	0.038	8.126	9.128	8.574	13518	Dst	chr1	+	NM_0012	P26247	protein_cc	2310001O	dystonin	Dst
120	Ccdc115	1.621	0.992	1.609	0.061	0.964	0.112	8.672	9.369	9.358	69668	Ccdc115	chr1	-	NM_0271	P10147	protein_cc	2310061I	coiled-coil	Ccdc115
121	Imp4	1.198	0.965	1.156	0.244	0.808	0.331	8.336	8.597	8.545	27993	Imp4	chr1	+	NM_1786	P17765	protein_cc	AA409888	IMP4, U3 :	Imp4
122	Ptpn18	0.789	0.763	0.602	0.033	0.047	0.001	5.412	5.069	4.679	19253	Ptpn18	chr1	+	NM_0112	P22812	protein_cc	FLP1| HSC	protein ty	Ptpn18
123	Prss39	1.000	1.000	1.000	1.000	1.000	1.000	0.000	0.000	0.000	21755	Prss39	chr1	-	NM_0093	P24466	protein_cc	Tesp1	protease,	Prss39
124	Cfc1	1.000	1.000	1.000	1.000	1.000	1.000	0.000	0.000	0.000	12627	Cfc1	chr1	+	NM_0076	P18053	protein_cc	AV265756	cripto, FRI	Cfc1
125	Prss40	1.000	1.000	1.000	1.000	1.000	1.000	0.000	0.000	0.000	21756	Prss40	chr1	-	NM_0093	P15637	protein_cc	Tesp2	protease,	Prss40
126	Amer3	1.000	1.000	1.000	1.000	1.000	1.000	0.000	0.000	0.000	211383	Amer3	chr1	+	NM_2137	P12480	protein_cc	9430069J	APC mem	Amer3
127	AA619741	1.000	1.000	1.000	1.000	1.000	1.000	0.000	0.000	0.000	241051	AA619741	chr1	-	NR_03362	.	misc_RNA	.	expressed	AA619741
128	Arhgef4	1.000	1.000	1.000	1.000	1.000	1.000	0.000	0.000	0.000	226970	Arhgef4	chr1	+	NM_1830	P7141	protein_cc	9330140K	Rho guani	Arhgef4
129	Fam168b	1.340	0.792	1.061	0.003	0.002	0.482	8.227	8.650	8.313	214469	Fam168b	chr1	-	NM_0011	P1284	protein_cc	mKIAA404	family wit	Fam168b
130	Plekhb2	0.749	1.146	0.859	0.185	0.701	0.732	7.484	7.068	7.265	226971	Plekhb2	chr1	+	NM_1754	P360	protein_cc	2310009M	pleckstrin	Plekhb2
131	Hs6st1	0.308	1.161	0.357	0.001	0.237	0.028	10.193	8.493	8.709	50785	Hs6st1	chr1	+	NM_0158	P18861	protein_cc	6Ost1	heparan s	Hs6st1
132	Uggt1	1.280	0.814	1.042	0.299	0.360	0.820	9.934	10.290	9.993	320011	Uggt1	chr1	-	NM_1988	P21031	protein_cc	0910001L:	UDP-glucc	Uggt1
133	Neurl3	5.540	0.240	1.331	0.010	0.014	0.446	4.929	7.399	5.341	214854	Neurl3	chr1	-	NM_1534	P8117	protein_cc	2010300P	neuralized	Neurl3
134	Arid5a	50.719	0.393	19.953	0.000	0.012	0.234	2.848	8.512	7.167	214855	Arid5a	chr1	+	NM_0011	P312	protein_cc	D430024K	AT rich int	Arid5a
135	Kansl3	1.038	1.042	1.082	0.901	0.893	0.698	8.101	8.155	8.215	226976	Kansl3	chr1	-	NM_1726	P22446	protein_cc	4632411B	KAT8 regu	Kansl3
136	Fer1l5	0.352	0.822	0.289	0.259	0.624	0.462	2.215	0.709	0.425	10053427	Fer1l5	chr1	+	NM_0012	P20668	protein_cc	4930533C	fer-1-like 5	Fer1l5
137	Lman2l	0.656	0.525	0.345	0.009	0.070	0.061	6.175	5.568	4.639	214895	Lman2l	chr1	-	NM_0010	P14819	protein_cc	A630028F	lectin, ma	Lman2l
138	Cnnm4	0.815	0.494	0.403	0.253	0.079	0.077	4.554	4.258	3.242	94220	Cnnm4	chr1	+	NM_0335	P10769	protein_cc	5430430O	cyclin M4	Cnnm4
139	Cnnm3	0.375	0.847	0.318	0.001	0.391	0.013	7.858	6.442	6.203	94218	Cnnm3	chr1	+	NM_0531	P8424	protein_cc	Acdp3| Cl	cyclin M3	Cnnm3
140	Ankrd23	1.362	0.221	0.301	0.511	0.101	0.078	2.577	3.023	0.845	78321	Ankrd23	chr1	-	NM_1535	P5522	protein_cc	1110058D	ankyrin re	Ankrd23
141	Ankrd39	0.767	1.425	1.092	0.131	0.102	0.612	5.174	4.790	5.301	109346	Ankrd39	chr1	-	NM_0262	P26862	protein_cc	9130416N	ankyrin re	Ankrd39
142	Sema4c	10.816	0.672	7.264	0.006	0.137	0.017	4.962	8.397	7.823	20353	Sema4c	chr1	-	NM_0013	P9427	protein_cc	AI426163|	sema dom	Sema4c
143	Fam178b	2.755	0.800	2.204	0.304	0.735	0.139	0.001	1.463	1.141	381337	Fam178b	chr1	-	NM_0011	P25422	protein_cc	1700024G	family wit	Fam178b
144	Cox5b	0.888	1.035	0.920	0.092	0.480	0.421	11.244	11.073	11.123	12859	Cox5b	chr1	+	NM_0099	P16322	protein_cc	.	cytochrom	Cox5b
145	Actr1b	1.089	1.082	1.178	0.431	0.070	0.371	8.373	8.495	8.609	226977	Actr1b	chr1	-	NM_1461	P4888	protein_cc	2310066K	ARP1 actir	Actr1b
146	4933424G	1.000	1.000	1.000	1.000	1.000	1.000	0.000	0.000	0.000	71166	4933424G	chr1	-	NR_04029	.	lncRNA	1700104I1	RIKEN cDN	4933424G(
147	Zap70	0.175	0.707	0.124	0.001	0.147	0.023	7.036	4.519	4.020	22637	Zap70	chr1	+	NM_0095	P2304	protein_cc	mrtle| mu	zeta-chain	Zap70
148	Tmem131	1.140	0.584	0.666	0.260	0.000	0.187	6.229	6.418	5.642	56030	Tmem131	chr1	-	NM_0188	P22857	protein_cc	2610524E	transmem	Tmem131
149	Cnga3	1.000	1.000	1.000	1.000	1.000	1.000	0.000	0.000	0.000	12790	Cnga3	chr1	+	NM_0012	P13811	protein_cc	CNG3	cyclic nucl	Cnga3
150	Inpp4a	1.115	0.434	0.484	0.746	0.129	0.001	5.452	5.609	4.405	269180	Inpp4a	chr1	+	NM_0302	P12739	protein_cc	107kDa| 9	inositol pc	Inpp4a
151	Coa5	0.599	1.330	0.797	0.000	0.001	0.045	9.628	8.888	9.300	76178	Coa5	chr1	-	NM_1980	P16319	protein_cc	1700001A	cytochrom	Coa5
152	Unc50	1.170	0.748	0.875	0.361	0.121	0.491	8.717	8.944	8.524	67387	Unc50	chr1	+	NM_0261	P21191	protein_cc	1110002A	unc-50 ho	Unc50
153	Mgat4a	2.304	0.399	0.919	0.004	0.019	0.882	3.372	4.576	3.250	269181	Mgat4a	chr1	-	NM_1738	P14155	protein_cc	9530018I	mannosid	Mgat4a
154	4930594C	1.136	0.725	0.823	0.293	0.088	0.437	5.055	5.239	4.774	77633	4930594C	chr1	-	NR_02401	.	misc_RNA	.	RIKEN cDN	4930594C1
155	2010300C	0.462	1.837	0.849	0.184	0.187	0.844	1.146	0.033	0.910	72097	2010300C	chr1	-	NM_0280	P7493	protein_cc	.	RIKEN cDN	2010300C(
156	Tsga10	0.620	0.589	0.365	0.140	0.145	0.245	3.384	2.693	1.929	211484	Tsga10	chr1	-	NM_2072	P12251	protein_cc	4933432N	testis spe	Tsga10
157	Lipt1	0.573	1.102	0.632	0.014	0.573	0.029	6.856	6.053	6.193	623661	Lipt1	chr1	+	NM_0010	P1582	protein_cc	EG623661	lipoyltrans	Lipt1
158	Mitd1	4.136	0.426	1.761	0.000	0.001	0.038	7.331	9.379	8.148	69028	Mitd1	chr1	-	NM_0269	P20633	protein_cc	1500032H	MIT, micro	Mitd1
159	Mrpl30	2.431	0.691	1.679	0.000	0.000	0.060	8.878	10.160	9.626	107734	Mrpl30	chr1	+	NM_0270	P10995	protein_cc	2310001L	mitochond	Mrpl30
160	Lyg2	1.000	1.000	1.000	1.000	1.000	1.000	0.000	0.000	0.000	332427	Lyg2	chr1	-	NM_0010	P14831	protein_cc	Gm776	lysozyme	Lyg2
161	Lyg1	1.000	1.000	1.000	1.000	1.000	1.000	0.000	0.000	0.000	69541	Lyg1	chr1	-	NM_0271	P26212	protein_cc	2300002O	lysozyme	Lyg1
162	Txndc9	1.852	0.817	1.513	0.000	0.082	0.156	9.409	10.298	10.006	98258	Txndc9	chr1	-	NM_1720	P8556	protein_cc	AI098020|	thioredoxi	Txndc9
163	Eif5b	0.869	0.811	0.705	0.006	0.020	0.061	11.495	11.292	10.991	226982	Eif5b	chr1	+	NM_1983	P17006	protein_cc	A030003E	eukaryotic	Eif5b
164	Rev1	0.965	0.962	0.929	0.891	0.901	0.854	5.929	5.878	5.822	56210	Rev1	chr1	-	NM_0195	P17878	protein_cc	1110027I2	REV1, DNA	Rev1
165	Aff3	0.718	0.846	0.607	0.280	0.681	0.302	2.255	1.777	1.536	16764	Aff3	chr1	-	NM_0012	P173	protein_cc	3222402O	AF4/FMR2	Aff3
166	Lonrf2	1.000	1.000	1.000	1.000	1.000	1.000	0.000	0.000	0.000	381338	Lonrf2	chr1	-	NM_0010	P4793	protein_cc	2900060P	LON pepti	Lonrf2
167	Chst10	0.405	0.985	0.399	0.181	1.000	0.423	1.324	0.022	0.000	98388	Chst10	chr1	-	NM_1451	P5692	protein_cc	AI507003|	carbohydr	Chst10
168	Nms	1.000	1.000	1.000	1.000	1.000	1.000	0.000	0.000	0.000	433292	Nms	chr1	+	NM_0010	P15731	protein_cc	AB164466	neuromed	Nms
169	Pdcl3	1.633	0.953	1.556	0.008	0.348	0.126	6.276	6.984	6.914	68833	Pdcl3	chr1	+	NM_0268	P21659	protein_cc	1110061A	phosducin	Pdcl3

170	Npas2	18.844	0.064	1.205	0.005	0.005	0.825	2.400	6.636	2.669	18143	Npas2	chr1	+	NM_0087	P1855	protein_cc	bHLHe9| N	neuronal P	Npas2
171	Rpl31	1.524	1.200	1.828	0.000	0.000	0.000	9.598	10.206	10.468	114641	Rpl31	chr1	+	NM_0532	P3289	protein_cc	Dts	ribosomal	Rpl31
172	Tbc1d8	2.030	0.381	0.773	0.011	0.004	0.575	6.373	7.394	6.001	54610	Tbc1d8	chr1	-	NM_0187	P26341	protein_cc	AD3| HBLI	TBC1 dom	Tbc1d8
173	Cnot11	2.037	0.988	2.012	0.037	0.955	0.132	6.263	7.289	7.272	52846	Cnot11	chr1	+	NM_0280	P11708	protein_cc	2410015L:	CCR4-NOT	Cnot11
174	Snord89	1.856	0.883	1.639	0.009	0.427	0.167	7.597	8.489	8.310	10021746	Snord89	chr1	-	NR_02855	.	snoRNA	MBII-289	small nucl	Snord89
175	Rnf149	1.226	1.270	1.557	0.025	0.238	0.278	11.667	11.961	12.306	67702	Rnf149	chr1	-	NM_0010	P11102	protein_cc	1600023E:	ring finger	Rnf149
176	Creg2	1.210	2.158	2.611	0.633	0.021	0.027	0.422	0.697	1.807	263764	Creg2	chr1	-	NM_1705	P1958	protein_cc	A830098L:	cellular re	Creg2
177	Rfx8	2.191	0.817	1.790	0.000	0.453	0.423	0.001	1.132	0.840	619289	Rfx8	chr1	-	NM_0011	P8979	protein_cc	4933400N	regulatory	Rfx8
178	Gm3646	1.000	1.000	1.000	1.000	1.000	1.000	0.000	0.000	0.000	10004206:	Gm3646	chr1	-	NM_0011	P10239	protein_cc	10004206:	predicted	Gm3646
179	1700066B	1.000	1.000	1.000	1.000	1.000	1.000	0.000	0.000	0.000	73426	1700066B	chr1	-	NR_04046	.	lncRNA	.	RIKEN cDN	1700066B:
180	Map4k4	3.278	0.935	3.066	0.002	0.763	0.167	8.287	10.000	9.904	26921	Map4k4	chr1	+	NM_0086	P25008	protein_cc	9430080K	mitogen-a	Map4k4
181	Gm16894	2.203	1.578	3.478	0.299	0.276	0.002	0.001	1.141	1.799	10004713:	Gm16894	chr1	-	NR_03798	.	lncRNA	.	predicted	Gm16894
182	Il1r2	138.606	0.546	75.735	0.006	0.071	0.003	0.816	7.931	7.059	16178	Il1r2	chr1	+	NM_0105	P20720	protein_cc	CD121b| I	interleukir	Il1r2
183	Il1r1	9.456	1.071	10.123	0.001	0.677	0.034	6.761	10.002	10.100	16177	Il1r1	chr1	+	NM_0083	P4011	protein_cc	CD121a| (	interleukir	Il1r1
184	Il1rl2	0.467	2.271	1.061	0.185	0.185	0.947	1.136	0.038	1.221	107527	Il1rl2	chr1	+	NM_1331	P11455	protein_cc	AI481289|	interleukir	Il1rl2
185	Il1rl1	2.523	0.551	1.389	0.107	0.237	0.626	0.410	1.745	0.884	17082	Il1rl1	chr1	+	NM_0012	P5899	protein_cc	DER4| Fit-	interleukir	Il1rl1
186	Il18r1	0.947	0.967	0.916	0.890	0.902	0.916	2.294	2.215	2.167	16182	Il18r1	chr1	+	NM_0011	P17579	protein_cc	Il18ralpha	interleukir	Il18r1
187	Il18rap	15.836	0.326	5.165	0.155	0.292	0.398	1.233	5.218	3.601	16174	Il18rap	chr1	+	NM_0105	P3293	protein_cc	AcPL| IL-1	interleukir	Il18rap
188	Slc9a4	0.726	1.387	1.007	0.187	0.187	0.988	0.477	0.015	0.487	110895	Slc9a4	chr1	+	NM_1770	P23323	protein_cc	AW99055:	solute car	Slc9a4
189	Slc9a2	1.000	1.000	1.000	1.000	1.000	1.000	0.000	0.000	0.000	226999	Slc9a2	chr1	+	NM_0010	P19005	protein_cc	2210416H	solute car	Slc9a2
190	Mfsd9	0.479	1.629	0.780	0.084	0.268	0.190	5.031	3.969	4.673	211798	Mfsd9	chr1	-	NM_1724	P25282	protein_cc	4931419K	major faci	Mfsd9
191	Tmem182	1.000	1.000	1.000	1.000	1.000	1.000	0.000	0.000	0.000	381339	Tmem182	chr1	+	NM_0010	P6295	protein_cc	2310079P	transmem	Tmem182
192	4930448I(	1.000	1.000	1.000	1.000	1.000	1.000	0.000	0.000	0.000	74897	4930448I(	chr1	-	NR_04047	.	lncRNA	.	RIKEN cDN	4930448I0
193	Pantr1	1.005	1.405	1.412	0.000	0.182	0.423	0.000	0.008	0.498	66297	Pantr1	chr1	-	NR_02782	.	lncRNA	2610017I(	POU doma	Pantr1
194	Pou3f3	1.572	0.636	1.000	0.304	1.000	0.423	0.000	0.653	0.000	18993	Pou3f3	chr1	+	NM_0089	P4239	protein_cc	Brn-1| Brr	POU doma	Pou3f3
195	2900092D	1.014	2.240	2.272	0.000	0.182	0.423	0.000	0.021	1.185	73100	2900092D	chr1	+	NR_02789	.	lncRNA	.	RIKEN cDN	2900092D:
196	Pantr2	1.000	1.000	1.000	1.000	1.000	1.000	0.000	0.000	0.000	66620	Pantr2	chr1	-	NR_11049	.	lncRNA	2610207O	POU doma	Pantr2
197	Dalir	1.000	1.000	1.000	1.000	1.000	1.000	0.000	0.000	0.000	10494069	Dalir	chr1	-	NR_13090	.	lncRNA	Dali| Gm2	DNMT1 as	Dalir
198	Mrps9	0.761	1.465	1.114	0.023	0.021	0.568	8.091	7.696	8.247	69527	Mrps9	chr1	+	NM_0235	P1236	protein_cc	2310002A	mitochon	Mrps9
199	Gpr45	1.000	1.000	1.000	1.000	1.000	1.000	0.000	0.000	0.000	93690	Gpr45	chr1	+	NM_0531	P23511	protein_cc	9230112G	G protein-	Gpr45
200	Tgfbrap1	0.721	1.027	0.741	0.015	0.492	0.139	7.834	7.362	7.402	73122	Tgfbrap1	chr1	-	NM_0010	P26804	protein_cc	3110018K	transform	Tgfbrap1
201	AI597479	1.611	0.589	0.949	0.002	0.004	0.840	5.856	6.544	5.781	98404	AI597479	chr1	+	NM_1338	P9081	protein_cc	.	expressed	AI597479
202	Fhl2	1.310	2.185	2.863	0.177	0.000	0.018	2.011	2.401	3.529	14200	Fhl2	chr1	-	NM_0102	P19173	protein_cc	FHL-2| SLI	four and a	Fhl2
203	Nck2	0.505	0.946	0.478	0.340	0.943	0.403	4.544	3.560	3.480	17974	Nck2	chr1	+	NM_0108	P22843	protein_cc	4833426I1	non-catal	Nck2
204	1500015O	0.552	0.990	0.547	0.182	1.000	0.423	0.871	0.015	0.000	78896	1500015O	chr1	+	NM_0242	P16805	protein_cc	Ecrg4	esophagea	1500015O
205	Uxs1	0.430	4.140	1.779	0.010	0.028	0.338	3.035	1.817	3.866	67883	Uxs1	chr1	-	NM_0264	P20751	protein_cc	1600025I1	UDP-glucu	Uxs1
206	Tpp2	1.833	0.562	1.030	0.009	0.013	0.876	8.060	8.934	8.103	22019	Tpp2	chr1	+	NM_0094	P9900	protein_cc	TPP-2| Tp	tripeptidy	Tpp2
207	Mettl21c	1.000	1.000	1.000	1.000	1.000	1.000	0.000	0.000	0.000	433294	Mettl21c	chr1	-	NM_0010	P12657	protein_cc	A530098C	methyltra	Mettl21c
208	Tex30	2.442	1.036	2.531	0.000	0.708	0.051	6.344	7.632	7.684	75623	Tex30	chr1	-	NM_0293	P15841	protein_cc	1700029F(	testis expr	Tex30
209	Kdelc1	1.473	1.529	2.251	0.002	0.001	0.009	4.898	5.457	6.069	72050	Kdelc1	chr1	-	NM_0236	P18831	protein_cc	1810049A	KDEL (Lys-	Kdelc1
210	Bivm	0.419	1.618	0.678	0.002	0.060	0.096	6.505	5.249	5.943	246229	Bivm	chr1	+	NM_1445	P11274	protein_cc	AI854267	basic, imm	Bivm
211	Ercc5	1.195	1.033	1.234	0.099	0.774	0.300	7.518	7.775	7.822	22592	Ercc5	chr1	+	NM_0117	P18768	protein_cc	Xpg	excision re	Ercc5
212	Mettl21e	1.580	0.633	1.000	0.305	1.000	0.423	0.000	0.660	0.000	403183	Mettl21e	chr1	-	NM_2072	P5313	protein_cc	4832428D	methyltra	Mettl21e
213	Gulp1	0.355	0.981	0.348	0.056	1.000	0.240	1.524	0.028	0.000	70676	Gulp1	chr1	+	NM_0284	P25337	protein_cc	3110030A	GULP, eng	Gulp1
214	4930521E	1.000	1.000	1.000	1.000	1.000	1.000	0.000	0.000	0.000	75066	4930521E(	chr1	-	NR_04060	.	lncRNA	.	RIKEN cDN	4930521E(
215	Col3a1	0.833	1.024	0.853	0.096	0.722	0.297	9.105	8.842	8.876	12825	Col3a1	chr1	+	NM_0099	P17866	protein_cc	AW55062!	collagen, t	Col3a1
216	Col5a2	0.296	1.782	0.528	0.023	0.290	0.193	5.465	3.711	4.545	12832	Col5a2	chr1	-	NM_0077	P5846	protein_cc	1110014L:	collagen, t	Col5a2
217	Wdr75	2.156	0.739	1.593	0.000	0.026	0.171	6.528	7.636	7.200	73674	Wdr75	chr1	+	NM_0285	P24619	protein_cc	1300003A	WD repea	Wdr75
218	Slc40a1	0.027	3.605	0.098	0.000	0.148	0.010	10.141	4.943	6.793	53945	Slc40a1	chr1	-	NM_0169	P10296	protein_cc	Dusg| Fpn	solute car	Slc40a1
219	Stk-ps2	1.000	1.000	1.000	1.000	1.000	1.000	0.000	0.000	0.000	212225	Stk-ps2	chr1	-	NR_03769	.	misc_RNA	4930509O	serine/thr	Stk-ps2
220	Dnah7b	0.535	0.874	0.468	0.352	0.735	0.517	1.594	0.692	0.498	227058	Dnah7b	chr1	+	NM_0011	P23386	protein_cc	Dnahc7b|	dynein, ax	Dnah7b
221	Slc39a10	15.145	0.623	9.438	0.000	0.002	0.000	4.488	8.409	7.726	227059	Slc39a10	chr1	-	NM_1726	P26436	protein_cc	2900042E:	solute car	Slc39a10
222	Tmeff2	1.000	1.000	1.000	1.000	1.000	1.000	0.000	0.000	0.000	56363	Tmeff2	chr1	+	NM_0197	P18130	protein_cc	4832418D	transmem	Tmeff2
223	9330175M	1.000	1.000	1.000	1.000	1.000	1.000	0.000	0.000	0.000	442821	9330175M	chr1	-	NR_04515	.	lncRNA	.	RIKEN cDN	9330175M
224	Sdpr	0.045	7.384	0.353	0.000	0.072	0.088	9.391	5.003	7.887	20324	Sdpr	chr1	+	NM_1387	P18810	protein_cc	.	serum dep	Sdpr
225	Nabp1	3.690	0.828	3.057	0.000	0.003	0.005	6.279	8.162	7.890	109019	Nabp1	chr1	-	NM_0286	P2124	protein_cc	4930434H	nucleic ac	Nabp1
226	Myo1b	0.264	1.300	0.344	0.000	0.001	0.001	10.339	8.419	8.798	17912	Myo1b	chr1	-	NM_0011	P1260	protein_cc	AA406997	myosin IB	Myo1b
227	Stat4	1.214	0.818	0.994	0.755	0.704	0.993	1.928	2.208	1.919	20849	Stat4	chr1	+	NM_0113	P5657	protein_cc	.	signal trar	Stat4
228	Stat1	4.628	0.328	1.516	0.000	0.001	0.138	7.624	9.835	8.225	20846	Stat1	chr1	+	NM_0012	P16907	protein_cc	2010005J(	signal trar	Stat1
229	Gls	5.870	0.812	4.768	0.009	0.334	0.012	5.177	7.730	7.430	14660	Gls	chr1	-	NM_0010	P10397	protein_cc	6330442B	glutamina	Gls
230	Nab1	1.742	0.910	1.585	0.001	0.376	0.170	9.580	10.381	10.244	17936	Nab1	chr1	-	NM_0086	P5933	protein_cc	.	Ngfi-A bin	Nab1
231	Tmem194	2.512	0.716	1.799	0.030	0.188	0.197	1.200	2.528	2.047	227094	Tmem194	chr1	+	NM_0011	P18887	protein_cc	5330401P(	transmem	Tmem194I
232	Mfsd6	1.888	0.996	1.880	0.000	0.885	0.000	6.656	7.573	7.567	98682	Mfsd6	chr1	-	NM_1338	P20163	protein_cc	2210010L(	major faci	Mfsd6
233	Inpp1	1.391	0.486	0.676	0.032	0.003	0.242	6.299	6.775	5.735	16329	Inpp1	chr1	-	NM_0083	P7969	protein_cc	2300002C	inositol po	Inpp1
234	Hibch	0.282	1.230	0.347	0.000	0.070	0.010	9.081	7.254	7.554	227095	Hibch	chr1	+	NM_1461	P16310	protein_cc	2610509I1	3-hydroxy	Hibch
235	1700019D	1.000	1.000	1.000	1.000	1.000	1.000	0.000	0.000	0.000	67080	1700019D	chr1	-	NM_1449	P21146	protein_cc	2410136C	RIKEN cDN	1700019D(
236	Mstn	1.000	1.000	1.000	1.000	1.000	1.000	0.000	0.000	0.000	17700	Mstn	chr1	-	NM_0108	P3455	protein_cc	Cmpt| Gd	myostatin	Mstn
237	1700019A	1.572	0.636	1.000	0.304	1.000	0.423	0.000	0.653	0.000	69397	1700019A	chr1	+	NM_0270	P547	protein_cc	.	RIKEN cDN	1700019A(
238	Pms1	0.706	0.971	0.685	0.195	0.892	0.432	5.546	5.044	5.000	227099	Pms1	chr1	-	NM_1535	P22132	protein_cc	.	postmeiot	Pms1
239	Ormdl1	0.579	1.324	0.766	0.006	0.083	0.331	7.840	7.051	7.456	227102	Ormdl1	chr1	+	NM_1455	P10410	protein_cc	C730042F	ORM1-like	Ormdl1
240	Osgepl1	0.885	1.311	1.160	0.181	0.059	0.522	7.305	7.128	7.519	72085	Osgepl1	chr1	+	NM_0012	P21905	protein_cc	2610001M	O-sialoglyc	Osgepl1
241	Asnsd1	0.588	1.525	0.897	0.000	0.005	0.387	8.874	8.108	8.716	70396	Asnsd1	chr1	-	NM_1337	P22138	protein_cc	2210409M	asparagine	Asnsd1
242	Dnah7a	0.716	0.995	0.712	0.182	1.000	0.423	0.490	0.008	0.000	627872	Dnah7a	chr1	-	NM_0012	P8223	protein_cc	Dnahc7| D	dynein, ax	Dnah7a
243	Stk17b	1.572	1.267	1.992	0.259	0.455	0.154	5.643	6.296	6.638	98267	Stk17b	chr1	-	NM_1338	P22428	protein_cc	3110009A	serine/thr	Stk17b
244	Hecw2	0.289	3.113	0.900	0.000	0.000	0.304	4.216	2.425	4.063	329152	Hecw2	chr1	-	NM_0010	P21852	protein_cc	A730039N	HECT, C2 a	Hecw2
245	Ccdc150	0.318	0.977	0.311	0.000	1.000	0.009	1.685	0.033	0.000	78016	Ccdc150	chr1	+	NM_0300	P19714	protein_cc	4930511H	coiled-coil	Ccdc150
246	Gtf3c3	1.322	0.766	1.013	0.014	0.003	0.939	6.160	6.563	6.178	98488	Gtf3c3	chr1	-	NM_0010	P18831	protein_cc	TF3C-gam	general tra	Gtf3c3
247	Pgap1	0.036	4.573	0.166	0.000	0.039	0.009	7.850	3.068	5.261	241062	Pgap1	chr1	-	NM_0011	P25336	protein_cc	5033403E:	post-GPI a	Pgap1
248	Ankrd44	0.922	0.815	0.751	0.554	0.282	0.160	5.333	5.216	4.920	329154	Ankrd44	chr1	-	NM_0010	P14985	protein_cc	4930444A	ankyrin re	Ankrd44
249	Sf3b1	1.463	0.835	1.222	0.015	0.108	0.117	9.887	10.436	10.176	81898	Sf3b1	chr1	-	NM_0311	P7710	protein_cc	155kDa| 2	splicing fa	Sf3b1
250	Coq10b	0.842	0.804	0.677	0.097	0.052	0.057	7.996	7.747	7.433	67876	Coq10b	chr1	+	NM_0264	P16547	protein_cc	1500041J(	coenzyme	Coq10b
251	Hspd1	0.630	1.021	0.643	0.000	0.766	0.034	11.636	10.968	10.999	15510	Hspd1	chr1	-	NM_0104	P4247	protein_cc	60kDa| Hs	heat shocl	Hspd1
252	Hspe1	0.843	0.858	0.723	0.244	0.259	0.180	10.794	10.547	10.325	15528	Hspe1	chr1	+	NM_0083	P25234	protein_cc	10kDa| Hs	heat shocl	Hspe1
253	Mob4	1.201	0.879	1.056	0.348	0.459	0.796	9.722	9.986	9.800	19070	Mob4	chr1	+	NM_0252	P25033	protein_cc	2610109B	MOB fami	Mob4
254	Rftn2	0.644	1.153	0.743	0.066	0.533	0.487	4.350	3.716	3.922	74013	Rftn2	chr1	-	NM_0287	P20853	protein_cc	2700010E(	raftlin fam	Rftn2
255	Mars2	2.392	1.416	3.386	0.037	0.115	0.112	3.555	4.814	5.315	212679	Mars2	chr1	+	NM_1754	P21288	protein_cc	C730026E	methionin	Mars2
256	Boll	0.457	1.401	0.640	0.183	0.194	0.600	1.158	0.027	0.514	75388	Boll	chr1	-	NM_0011	P23975	protein_cc	4930554P	bol, boule	Boll
257	Plcl1	6.784	3.144	21.331	0.003	0.008	0.051	1.931	4.693	6.346	227120	Plcl1	chr1	+	NM_0011	P26055	protein_cc	C230017K	phospholi	Plcl1
258	9130227L	1.000	1.000	1.000	1.000	1.000	1.000	0.000	0.000	0.000	329159	9130227L(	chr1	+	NR_04583	.	lncRNA	.	RIKEN cDN	9130227L(
259	1700003I2	1.000	1.000	1.000	1.000	1.000	1.000	0.000	0.000	0.000	69356	1700003I2	chr1	+	NR_12607	.	lncRNA	.	RIKEN cDN	1700003I2
260	Hsfy2	1.000	1.000	1.000	1.000	1.000	1.000	0.000	0.000	0.000	71066	Hsfy2	chr1	-	NM_0276	P1792	protein_cc	4933413G	heat shocl	Hsfy2

261	Satb2	3.718	0.594	2.207	0.030	0.141	0.234	1.026	2.920	2.168	212712	Satb2	chr1	-	NM_1391	P15153	protein_cc	mKIAA103	special AT	Satb2
262	9130024F	1.210	1.985	2.403	0.638	0.334	0.491	0.427	0.702	1.691	329160	9130024F	chr1	+	NR_02432	.	lncRNA	B530002L(	RIKEN cDN	9130024F1
263	BC055402	0.451	0.986	0.444	0.182	1.000	0.423	1.170	0.021	0.000	407809	BC055402	chr1	-	NR_03799	.	lncRNA		cDNA seq	BC055402
264	4930558J1	1.000	1.000	1.000	1.000	1.000	1.000	0.000	0.000	0.000	75323	4930558J1	chr1	-	NR_03799	.	lncRNA	.	RIKEN cDN	4930558J1
265	1700066M	0.308	1.906	0.587	0.001	0.049	0.170	6.876	5.178	6.109	73467	1700066M	chr1	+	NM_0285	P7053	protein_cc	AI428479	RIKEN cDN	1700066M
266	Tyw5	0.616	1.699	1.046	0.032	0.002	0.808	7.225	6.525	7.290	68736	Tyw5	chr1	-	NM_0013	P8211	protein_cc	1110034B	tRNA-yW	Tyw5
267	9430016H	1.195	1.364	1.629	0.346	0.297	0.372	7.624	7.880	8.328	68115	9430016H	chr1	+	NM_0010	P17356	protein_cc	AA409999	RIKEN cDN	9430016H
268	Spats2l	10.006	0.496	4.960	0.001	0.019	0.161	4.127	7.450	6.437	67198	Spats2l	chr1	+	NM_1448	P14803	protein_cc	2810022L(	spermatog	Spats2l
269	Kctd18	2.114	1.188	2.511	0.012	0.398	0.195	4.611	5.691	5.939	51960	Kctd18	chr1	-	NM_02763	.	protein_cc	4932411A	potassium	Kctd18
270	Sgol2a	5.242	0.551	2.887	0.017	0.120	0.224	1.011	3.402	2.541	68549	Sgol2a	chr1	+	NM_0011	P5409	protein_cc	1110007N	shugoshin	Sgol2a
271	Aox1	0.328	1.145	0.376	0.000	0.616	0.055	7.607	6.000	6.195	11761	Aox1	chr1	+	NM_0096	P1837	protein_cc	AI196512 |	aldehyde	Aox1
272	Aox3	0.073	3.296	0.239	0.000	0.233	0.061	10.135	6.350	8.070	71724	Aox3	chr1	+	NM_0236	P18400	protein_cc	1200011D	aldehyde	Aox3
273	Aox4	1.000	1.000	1.000	1.000	1.000	1.000	0.000	0.000	0.000	71872	Aox4	chr1	+	NM_0236	P8249	protein_cc	2310003G	aldehyde	Aox4
274	Aox2	1.000	1.000	1.000	1.000	1.000	1.000	0.000	0.000	0.000	213043	Aox2	chr1	+	NM_0010	P16570	protein_cc	Aoh3 | Ao	aldehyde	Aox2
275	Bzw1	2.079	0.902	1.875	0.000	0.262	0.078	8.687	9.742	9.593	66882	Bzw1	chr1	+	NM_0258	P21623	protein_cc	1200015E	basic leuc	Bzw1
276	Clk1	1.073	0.748	0.802	0.684	0.133	0.306	7.356	7.457	7.038	12747	Clk1	chr1	-	NR_02785	.	protein_cc	STY	CDC-like k	Clk1
277	Ppil3	2.001	0.862	1.725	0.001	0.020	0.070	5.353	6.354	6.140	70225	Ppil3	chr1	-	NM_0012	P22171	protein_cc	2310076N	peptidylp	Ppil3
278	Nif3l1	1.815	0.976	1.771	0.000	0.443	0.011	6.476	7.336	7.301	65102	Nif3l1	chr1	+	NM_0229	P16333	protein_cc	1110030G	Ngg1 inte	Nif3l1
279	Orc2	1.457	1.344	1.958	0.204	0.383	0.295	6.198	6.741	7.167	18393	Orc2	chr1	-	NM_0010	P5513	protein_cc	AU041563	origin rec	Orc2
280	Fam126b	0.548	1.381	0.757	0.000	0.000	0.013	9.614	8.747	9.213	213056	Fam126b	chr1	-	NM_1725	P22278	protein_cc	C130065N	family wit	Fam126b
281	Ndufb3	0.528	1.255	0.663	0.012	0.183	0.100	11.606	10.684	11.012	66495	Ndufb3	chr1	+	NM_0255	P15631	protein_cc	2700033I1	NADH deh	Ndufb3
282	Gm20257	3.026	1.124	3.402	0.021	0.552	0.039	4.063	5.660	5.829	10050450	Gm20257	chr1	+	NR_04500	.	misc_RNA	.	predicted	Gm20257
283	Als2cr12	0.210	1.097	0.231	0.035	0.834	0.201	2.983	0.733	0.867	108812	Als2cr12	chr1	-	NM_1753	P10369	protein_cc	4933405P	amyotrop	Als2cr12
284	Cflar	1.562	0.516	0.807	0.010	0.010	0.405	9.163	9.807	8.853	12633	Cflar	chr1	+	NM_0012	P5010	protein_cc	2310024N	CASP8 an	Cflar
285	Casp8	2.178	1.135	2.472	0.013	0.373	0.011	7.342	8.466	8.648	12370	Casp8	chr1	+	NM_0098	P2957	protein_cc	CASP-8 | F	caspase 8	Casp8
286	Trak2	2.986	1.001	2.990	0.001	0.992	0.083	6.568	8.146	8.148	70827	Trak2	chr1	-	NM_1724	P5582	protein_cc	2900022D	trafficking	Trak2
287	Stradb	0.153	1.502	0.230	0.000	0.159	0.026	8.340	5.632	6.219	227154	Stradb	chr1	+	NM_1726	P16143	protein_cc	AA792893	STE20-rela	Stradb
288	Als2cr11	1.000	1.000	1.000	1.000	1.000	1.000	0.000	0.000	0.000	73463	Als2cr11	chr1	-	NM_1752	P17221	protein_cc	1700052H	amyotrop	Als2cr11
289	Tmem237	0.719	1.670	1.200	0.458	0.320	0.789	7.163	6.686	7.425	381259	Tmem237	chr1	-	NM_0010	P13651	protein_cc	AI853305 |	transmem	Tmem237
290	Mpp4	0.476	0.912	0.434	0.001	0.698	0.093	8.354	7.283	7.150	227157	Mpp4	chr1	-	NM_0011	P19336	protein_cc	Dlg6	membran	Mpp4
291	Als2	0.504	1.112	0.561	0.001	0.631	0.159	8.521	7.533	7.686	74018	Als2	chr1	-	NM_0011	P24628	protein_cc	3222402C	amyotrop	Als2
292	Cdk15	0.716	0.995	0.712	0.182	1.000	0.423	0.490	0.008	0.000	271697	Cdk15	chr1	+	NM_0010	P14229	protein_cc	Als2cr7 | P	cyclin-dep	Cdk15
293	Fzd7	0.908	0.701	0.636	0.632	0.066	0.320	5.075	4.936	4.423	14369	Fzd7	chr1	+	NM_0080	P8783	protein_cc	Fz7	frizzled cl	Fzd7
294	Gm973	1.152	0.629	0.725	0.722	1.000	0.422	0.465	0.669	0.000	381260	Gm973	chr1	+	NM_0010	P6650	protein_cc	AW55451	predicted	Gm973
295	Sumo1	1.886	0.746	1.407	0.001	0.012	0.042	7.838	8.754	8.331	22218	Sumo1	chr1	-	NM_0094	P18031	protein_cc	GMP1 | Pl	small ubiq	Sumo1
296	Nop58	6.405	0.650	4.166	0.000	0.032	0.074	8.587	11.266	10.645	55989	Nop58	chr1	+	NM_0188	P22938	protein_cc	MSSP | No	NOP58 rib	Nop58
297	Snord70	1.023	3.048	3.117	0.000	0.001	0.028	0.000	0.033	1.641	10021745	Snord70	chr1	+	NR_02855	.	snoRNA	MBII-234 |	small nucl	Snord70
298	Snord11	5.454	1.089	5.940	0.007	0.886	0.428	2.169	4.617	4.740	10021742	Snord11	chr1	+	NR_02852	.	snoRNA	MBII-95	small nucl	Snord11
299	Bmpr2	2.515	0.862	2.167	0.000	0.087	0.023	8.376	9.706	9.491	12168	Bmpr2	chr1	+	NM_0075	P10396	protein_cc	2610024H	bone mor	Bmpr2
300	Fam117b	1.627	1.065	1.732	0.119	0.846	0.467	6.037	6.739	6.829	72750	Fam117b	chr1	+	NM_0010	P7019	protein_cc	2810425F	family wit	Fam117b
301	Ica1l	1.000	1.000	1.000	1.000	1.000	1.000	0.000	0.000	0.000	70375	Ica1l	chr1	-	NM_0274	P21156	protein_cc	1700030B	islet cell a	Ica1l
302	Wdr12	1.600	0.845	1.353	0.008	0.108	0.069	6.719	7.398	7.155	57750	Wdr12	chr1	-	NM_0213	P10184	protein_cc	4933402C	WD repea	Wdr12
303	Carf	1.610	0.633	1.020	0.389	0.404	0.980	3.742	4.429	3.770	241066	Carf	chr1	+	NM_0012	P22972	protein_cc	Als2cr8 | E	calcium re	Carf
304	Nbeal1	0.747	1.338	1.000	0.003	0.155	0.999	8.303	7.883	8.302	269198	Nbeal1	chr1	+	NM_1734	P4495	protein_cc	2310076G	neurobea	Nbeal1
305	Cyp20a1	0.662	3.260	2.157	0.524	0.036	0.399	2.132	1.536	3.240	77951	Cyp20a1	chr1	+	NM_0300	P13528	protein_cc	A930011N	cytochron	Cyp20a1
306	Abi2	1.538	0.797	1.226	0.002	0.000	0.220	6.277	6.898	6.572	329165	Abi2	chr1	+	NM_0011	P5203	protein_cc	8430425M	abl-intera	Abi2
307	Raph1	3.573	0.737	2.635	0.000	0.005	0.000	9.684	11.521	11.082	77300	Raph1	chr1	-	NM_0010	P19241	protein_cc	9430025M	Ras associ	Raph1
308	Cd28	8.077	6.387	51.590	0.000	0.002	0.039	1.841	4.855	7.530	12487	Cd28	chr1	+	NM_0076	P19513	protein_cc	.	CD28 anti	Cd28
309	Ctla4	4.469	0.224	0.999	0.144	1.000	0.423	0.001	2.161	0.000	12477	Ctla4	chr1	+	NM_0012	P3570	protein_cc	Cd152 | Ct	cytotoxic	Ctla4
310	Icos	1.159	0.216	0.250	0.762	0.072	0.295	3.887	4.100	1.889	54167	Icos	chr1	+	NM_0174	P19090	protein_cc	AILIM | CC	inducible	Icos
311	9530026F	1.000	1.000	1.000	1.000	1.000	1.000	0.000	0.000	0.000	77341	9530026F	chr1	+	NR_04048	.	lncRNA	.	RIKEN cDN	9530026F
312	Pard3b	0.393	1.607	0.632	0.004	0.013	0.190	6.073	4.726	5.411	72823	Pard3b	chr1	+	NM_0010	P6824	protein_cc	1810008K	par-3 fami	Pard3b
313	Nrp2	2.846	0.547	1.557	0.001	0.006	0.056	6.740	8.249	7.378	18187	Nrp2	chr1	+	NM_0010	P7296	protein_cc	1110048P	neuropilin	Nrp2
314	4930487H	6.346	0.657	4.167	0.203	0.570	0.004	0.002	2.667	2.061	74992	4930487H	chr1	-	NR_04060	.	lncRNA		RIKEN cDN	4930487H
315	Ino80d	1.240	0.856	1.061	0.064	0.164	0.815	7.281	7.592	7.367	227195	Ino80d	chr1	-	NM_0011	P464	protein_cc	7330405I1	INO80 cor	Ino80d
316	Ino80dos	0.650	0.258	0.168	0.396	0.245	0.039	3.868	3.245	1.292	10038094	Ino80dos	chr1	+	NR_04591	.	lncRNA	Gm11602	INO80 cor	Ino80dos
317	Ndufs1	0.574	1.230	0.706	0.000	0.002	0.003	9.865	9.065	9.363	227197	Ndufs1	chr1	-	NM_0011	P18029	protein_cc	5830412M	NADH deh	Ndufs1
318	Eef1b2	1.292	1.299	1.678	0.001	0.056	0.101	11.698	12.068	12.445	55949	Eef1b2	chr1	+	NM_0187	P24614	protein_cc	2810017J(	eukaryotic	Eef1b2
319	Snora41	1.575	0.845	1.331	0.376	0.785	0.796	1.876	2.531	2.288	10021746	Snora41	chr1	+	NR_02855	.	snoRNA	MBI-83	small nucl	Snora41
320	Gpr1	1.000	1.000	1.000	1.000	1.000	1.000	0.000	0.000	0.000	241070	Gpr1	chr1	-	NM_1462	P1759	protein_cc	.	G protein-	Gpr1
321	Zdbf2	1.275	1.659	2.116	0.685	0.489	0.556	0.786	1.136	1.867	73884	Zdbf2	chr1	+	NM_0012	P13312	protein_cc	4930431J(	zinc finger	Zdbf2
322	Adam23	0.121	3.103	0.375	0.000	0.043	0.038	7.011	3.963	5.596	23792	Adam23	chr1	+	NM_0117	P16377	protein_cc	AW04639(	a disinteg	Adam23
323	Dytn	1.000	1.000	1.000	1.000	1.000	1.000	0.000	0.000	0.000	241073	Dytn	chr1	-	NM_0010	P19489	protein_cc	Gm215	dystroteli	Dytn
324	Mdh1b	1.000	1.000	1.000	1.000	1.000	1.000	0.000	0.000	0.000	76668	Mdh1b	chr1	-	NM_0296	P19885	protein_cc	1700124B	malate de	Mdh1b
325	Fastkd2	1.071	1.279	1.370	0.068	0.000	0.002	7.222	7.320	7.676	75619	Fastkd2	chr1	+	NM_1724	P9498	protein_cc	2810421I2	FAST kina	Fastkd2
326	4933402D	1.000	1.000	1.000	1.000	1.000	1.000	0.000	0.000	0.000	74426	4933402D	chr1	-	NM_0012	P7244	protein_cc	.	RIKEN cDN	4933402D
327	Gm13749	1.000	1.000	1.000	1.000	1.000	1.000	0.000	0.000	0.000	433315	Gm13749	chr1	-	NR_02782	.	lncRNA	OTTMUSG	predicted	Gm13749
328	Klf7	1.520	0.745	1.132	0.024	0.043	0.343	6.357	6.961	6.535	93691	Klf7	chr1	-	NM_0335	P23789	protein_cc	9830124P	Kruppel-li	Klf7
329	Creb1	1.202	0.655	0.787	0.044	0.008	0.140	8.090	8.356	7.745	12912	Creb1	chr1	+	NM_1338	P22325	protein_cc	2310001E	cAMP resp	Creb1
330	Mettl21a	1.385	1.319	1.826	0.053	0.129	0.111	6.424	6.893	7.293	67099	Mettl21a	chr1	-	NM_0259	P15623	protein_cc	2310038H	methyltra	Mettl21a
331	2810408I1	0.426	3.929	1.672	0.316	0.011	0.522	3.802	2.569	4.544	69941	2810408I1	chr1	-	NR_03800	.	lncRNA	.	RIKEN cDN	2810408I1
332	Ccnyl1	3.087	0.818	2.525	0.001	0.312	0.187	4.686	6.312	6.022	227210	Ccnyl1	chr1	+	NM_0010	P18116	protein_cc	9630037P	cyclin Y-lik	Ccnyl1
333	Fzd5	0.548	0.559	0.306	0.048	0.164	0.184	6.302	5.435	4.596	14367	Fzd5	chr1	-	NM_0227	P18149	protein_cc	5330434N	frizzled cl	Fzd5
334	Plekhm3	2.262	0.854	1.933	0.016	0.392	0.131	4.429	5.606	5.379	241075	Plekhm3	chr1	-	NM_0010	P15832	protein_cc	9430067K	pleckstrin	Plekhm3
335	Akr1cl	1.000	1.000	1.000	1.000	1.000	1.000	0.000	0.000	0.000	70861	Akr1cl	chr1	-	NM_0275	P14013	protein_cc	4921521F	aldo-keto	Akr1cl
336	Cryge	1.000	1.000	1.000	1.000	1.000	1.000	0.000	0.000	0.000	12968	Cryge	chr1	-	NM_0077	P15770	protein_cc	Cryg-2 | Cr	crystallin,	Cryge
337	Crygd	1.000	1.000	1.000	1.000	1.000	1.000	0.000	0.000	0.000	12967	Crygd	chr1	-	NM_0077	P5842	protein_cc	Aey4 | Cry	crystallin,	Crygd
338	Crygc	1.000	1.000	1.000	1.000	1.000	1.000	0.000	0.000	0.000	12966	Crygc	chr1	-	NM_0077	P18106	protein_cc	Cryg-5	crystallin,	Crygc
339	Crygb	1.000	1.000	1.000	1.000	1.000	1.000	0.000	0.000	0.000	12965	Crygb	chr1	-	NM_1447	P18635	protein_cc	Cryg-3 | D(	crystallin,	Crygb
340	Cryga	1.000	1.000	1.000	1.000	1.000	1.000	0.000	0.000	0.000	12964	Cryga	chr1	-	NM_0077	P4843	protein_cc	Cryg-4 | D(	crystallin,	Cryga
341	D630023F	1.000	1.000	1.000	1.000	1.000	1.000	0.000	0.000	0.000	98303	D630023F	chr1	-	NM_0012	P6746	protein_cc	AI314969	RIKEN cDN	D630023F
342	Idh1	0.158	2.338	0.370	0.000	0.000	0.020	12.492	9.833	11.058	15926	Idh1	chr1	-	NM_0011	P24259	protein_cc	AI314845 |	isocitrate	Idh1
343	Pikfyve	0.694	1.282	0.889	0.043	0.142	0.098	6.870	6.343	6.701	18711	Pikfyve	chr1	+	NM_0110	P2772	protein_cc	5230400C	phosphoir	Pikfyve
344	Pth2r	1.000	1.000	1.000	1.000	1.000	1.000	0.000	0.000	0.000	213527	Pth2r	chr1	-	NM_1392	P2094	protein_cc	Pthr2	parathyro	Pth2r
345	Crygf	1.000	1.000	1.000	1.000	1.000	1.000	0.000	0.000	0.000	12969	Crygf	chr1	+	NM_0270	P24592	protein_cc	3110001K	crystallin,	Crygf
346	Map2	0.362	0.783	0.283	0.002	0.362	0.010	3.696	2.229	1.876	17756	Map2	chr1	+	NM_0086	P7105	protein_cc	G1-397-34	microtubu	Map2
347	Unc80	0.726	1.387	1.007	0.187	0.187	0.988	0.477	0.015	0.487	329178	Unc80	chr1	+	NM_1755	P24532	protein_cc	C030018G	unc-80, N	Unc80
348	Rpe	1.051	1.178	1.238	0.711	0.245	0.125	7.685	7.756	7.993	66646	Rpe	chr1	+	NM_0256	P4464	protein_cc	2810429B	ribulose-5	Rpe
349	Kansl1l	0.984	1.007	0.991	0.899	0.947	0.974	7.067	7.044	7.054	68691	Kansl1l	chr1	-	NM_0011	P12896	protein_cc	1110028C	KAT8 regu	Kansl1l
350	Acadl	0.381	1.410	0.538	0.000	0.008	0.011	11.567	10.176	10.672	11363	Acadl	chr1	-	NM_0073	P12168	protein_cc	AA960361	acyl-Coen	Acadl
351	Myl1	1.000	1.000	1.000	1.000	1.000	1.000	0.000	0.000	0.000	17901	Myl1	chr1	-	NM_0011	P18983	protein_cc	AI325107 |	myosin, li	Myl1

352	Lancl1	0.498	1.734	0.864	0.039	0.001	0.657	7.216	6.211	7.005	14768	Lancl1	chr1	-	NM_0212	P12959	protein_cc	AW12473	LanC (bact	Lancl1
353	Cps1	0.256	0.810	0.207	0.000	0.521	0.037	13.619	11.654	11.350	227231	Cps1	chr1	+	NM_0010	P8559	protein_cc	4732433M	carbamoy	Cps1
354	Erbb4	0.716	0.995	0.712	0.182	1.000	0.423	0.490	0.008	0.000	13869	Erbb4	chr1	-	NM_0101	P26637	protein_cc	c-erbB-4|	erb-b2 rec	Erbb4
355	Ikzf2	2.709	0.607	1.644	0.161	0.333	0.518	1.486	2.924	2.203	22779	Ikzf2	chr1	-	NM_0117	P3423	protein_cc	A730095J	IKAROS fa	Ikzf2
356	Spag16	1.000	1.000	1.000	1.000	1.000	1.000	0.000	0.000	0.000	66722	Spag16	chr1	+	NM_0291	P19688	protein_cc	4921511D	sperm ass	Spag16
357	Vwc2l	0.716	0.995	0.712	0.182	1.000	0.423	0.490	0.008	0.000	320460	Vwc2l	chr1	+	NM_1771	P7545	protein_cc	A830006F	von Willeb	Vwc2l
358	Bard1	1.723	0.195	0.335	0.045	1.000	0.179	1.576	2.361	0.000	12021	Bard1	chr1	-	NM_0075	P16217	protein_cc	ENSMUSG	BRCA1 ass	Bard1
359	Abca12	1.000	1.000	1.000	1.000	1.000	1.000	0.000	0.000	0.000	74591	Abca12	chr1	-	NM_1752	P19120	protein_cc	4832428G	ATP-bindi	Abca12
360	Atic	1.079	1.336	1.443	0.731	0.171	0.297	7.014	7.124	7.543	108147	Atic	chr1	+	NM_0261	P16783	protein_cc	2610509C	5-aminoim	Atic
361	Fn1	0.985	1.106	1.089	0.774	0.113	0.213	12.751	12.729	12.874	14268	Fn1	chr1	-	NM_0102	P1597	protein_cc	E330027I	fibronecti	Fn1
362	Apol7d	0.858	1.048	0.900	0.806	0.938	0.911	1.352	1.132	1.200	10004281	Apol7d	chr1	+	NR_04030	.	lncRNA	ENSMUSG	apolipopro	Apol7d
363	Gm8883	5.219	1.608	8.390	0.056	0.159	0.039	3.809	6.193	6.878	667931	Gm8883	chr1	+	NR_02765	.	lncRNA	EG667931	predicted	Gm8883
364	4933417E	6.414	1.729	11.088	0.021	0.370	0.295	0.002	2.683	3.473	71134	4933417E	chr1	-	NR_04045	.	lncRNA		RIKEN cDN	4933417E1
365	Mreg	0.148	0.892	0.132	0.000	0.464	0.001	9.864	7.110	6.945	381269	Mreg	chr1	-	NM_0010	P14657	protein_cc	dsu| Gm9	melanoreg	Mreg
366	Pecr	0.220	0.848	0.187	0.000	0.144	0.002	12.100	9.917	9.678	111175	Pecr	chr1	-	NM_0235	P12543	protein_cc	2400003B	peroxisom	Pecr
367	Tmem169	9.358	0.703	6.576	0.016	0.253	0.003	0.002	3.228	2.719	271711	Tmem169	chr1	+	NM_1755	P25373	protein_cc	A830020B	transmem	Tmem169
368	Xrcc5	0.866	1.178	1.021	0.157	0.173	0.925	7.245	7.038	7.275	22596	Xrcc5	chr1	+	NM_0095	P23388	protein_cc	AI314015|	X-ray repa	Xrcc5
369	March4	1.005	1.405	1.412	0.000	0.182	0.423	0.000	0.008	0.498	381270	March4	chr1	-	NM_0010	P5909	protein_cc	BC056494	membran	March4
370	Smarcal1	0.412	1.353	0.557	0.000	0.179	0.073	5.866	4.585	5.022	54380	Smarcal1	chr1	+	NM_0188	P667	protein_cc	6030401P	SWI/SNF r	Smarcal1
371	Ankar	1.000	1.000	1.000	1.000	1.000	1.000	0.000	0.000	0.000	319695	Ankar	chr1	-	NM_1769	P3985	protein_cc	4932422E	ankyrin ar	Ankar
372	Rpl37a	1.169	1.074	1.255	0.207	0.426	0.331	9.731	9.956	10.059	19981	Rpl37a	chr1	+	NM_0090	P8633	protein_cc	.	ribosomal	Rpl37a
373	Igfbp2	0.201	0.571	0.115	0.005	0.220	0.056	11.641	9.328	8.519	16008	Igfbp2	chr1	+	NM_0083	P13190	protein_cc	AI255832|	insulin-like	Igfbp2
374	Igfbp5	0.138	10.591	1.462	0.005	0.061	0.648	6.379	3.523	6.927	16011	Igfbp5	chr1	-	NM_0105	P19349	protein_cc	AI256729|	insulin-like	Igfbp5
375	Tnp1	1.000	1.000	1.000	1.000	1.000	1.000	0.000	0.000	0.000	21958	Tnp1	chr1	-	NM_0094	P24173	protein_cc	Stp-1| Tp-	transition	Tnp1
376	1700027A	1.000	1.000	1.000	1.000	1.000	1.000	0.000	0.000	0.000	69449	1700027A	chr1	+	NR_03800	.	lncRNA	.	RIKEN cDN	1700027A
377	Pinc	1.000	1.000	1.000	1.000	1.000	1.000	0.000	0.000	0.000	723792	Pinc	chr1	-	NR_00320	.	lncRNA	.	pregnancy	Pinc
378	D530049I	1.000	1.000	1.000	1.000	1.000	1.000	0.000	0.000	0.000	78718	D530049I	chr1	+	NR_04060	.	lncRNA	.	RIKEN cDN	D530049I
379	6030407O	0.167	1.334	0.223	0.000	0.225	0.010	2.653	0.069	0.485	109198	6030407O	chr1	-	NR_04531	.	lncRNA	.	RIKEN cDN	6030407O
380	Tns1	1.049	0.881	0.924	0.518	0.169	0.606	9.380	9.449	9.266	21961	Tns1	chr1	-	NM_0012	P12939	protein_cc	1110018I2	tensin 1	Tns1
381	Rufy4	0.552	0.990	0.547	0.182	1.000	0.423	0.871	0.015	0.000	435626	Rufy4	chr1	+	NM_0011	P18591	protein_cc	F930048N	RUN and F	Rufy4
382	Cxcr2	22.314	0.677	15.104	0.000	0.001	0.002	1.579	6.059	5.496	12765	Cxcr2	chr1	+	NM_0099	P23571	protein_cc	CD128| Cl	chemokin	Cxcr2
383	Cxcr1	1.000	1.000	1.000	1.000	1.000	1.000	0.000	0.000	0.000	227288	Cxcr1	chr1	-	NM_1782	P13264	protein_cc	Il8ra	chemokin	Cxcr1
384	Arpc2	1.163	1.105	1.286	0.027	0.040	0.090	11.041	11.260	11.404	76709	Arpc2	chr1	+	NM_0297	P13796	protein_cc	2210023N	actin relat	Arpc2
385	Gpbar1	0.552	0.990	0.547	0.182	1.000	0.423	0.871	0.015	0.000	227289	Gpbar1	chr1	+	NM_1749	P180	protein_cc	BG37| GP	G protein-	Gpbar1
386	Aamp	0.540	1.190	0.643	0.040	0.442	0.227	11.656	10.768	11.019	227290	Aamp	chr1	-	NM_0011	P493	protein_cc	Aamp-rs|	angio-asso	Aamp
387	Pnkd	0.074	2.526	0.187	0.000	0.163	0.019	9.856	6.101	7.438	56695	Pnkd	chr1	+	NM_0255	P1787	protein_cc	2210013N	paroxysm	Pnkd
388	Tmbim1	0.735	1.334	0.981	0.047	0.003	0.917	8.111	7.667	8.083	69660	Tmbim1	chr1	-	NM_0271	P23443	protein_cc	2310061B	transmem	Tmbim1
389	Catip	0.602	4.822	2.904	0.199	0.182	0.505	0.793	0.061	2.331	241112	Catip	chr1	+	NM_0010	P480	protein_cc	Gm216	ciliogenes	Catip
390	Slc11a1	3.820	1.174	4.485	0.000	0.206	0.041	4.874	6.808	7.040	18173	Slc11a1	chr1	+	NM_0136	P8104	protein_cc	Bcg| ity| I	solute car	Slc11a1
391	Ctdsp1	0.368	1.098	0.404	0.001	0.473	0.025	9.020	7.577	7.712	227292	Ctdsp1	chr1	+	NM_1530	P21461	protein_cc	GIP| Nif3|	CTD (carbo	Ctdsp1
392	Vil1	1.207	0.682	0.823	0.587	0.386	0.780	2.764	3.035	2.483	22349	Vil1	chr1	+	NM_0095	P12342	protein_cc	Vil	villin 1	Vil1
393	Usp37	3.053	0.754	2.301	0.001	0.085	0.140	4.754	6.364	5.956	319651	Usp37	chr1	-	NM_1769	P10019	protein_cc	4932415L	ubiquitin s	Usp37
394	Rqcd1	1.134	1.071	1.214	0.475	0.676	0.526	6.589	6.771	6.869	.	Rqcd1	chr1	+	NM_0213	P21814	.	.	.	Rqcd1
395	Plcd4	1.885	0.436	0.822	0.319	0.225	0.804	1.074	1.989	0.791	18802	Plcd4	chr1	+	NM_0010	P26867	protein_cc	4921507K	phospholi	Plcd4
396	Zfp142	1.988	0.638	1.269	0.007	0.025	0.157	4.459	5.451	4.803	77264	Zfp142	chr1	-	NM_0298	P26163	protein_cc	9330177B	zinc finger	Zfp142
397	Bcs1l	0.467	1.770	0.826	0.005	0.121	0.584	7.268	6.168	6.992	66821	Bcs1l	chr1	+	NM_0013	P18729	protein_cc	9130022O	BCS1-like	Bcs1l
398	Rnf25	1.245	1.086	1.352	0.745	0.878	0.698	2.623	2.939	3.058	57751	Rnf25	chr1	-	NM_0013	P14459	protein_cc	0610009H	ring finger	Rnf25
399	Stk36	0.714	1.130	0.807	0.524	0.833	0.751	2.870	2.385	2.561	269209	Stk36	chr1	+	NM_1750	P24097	protein_cc	1700112N	serine/thr	Stk36
400	Ttll4	6.937	0.453	3.145	0.016	0.074	0.122	3.293	6.088	4.947	67534	Ttll4	chr1	+	NM_0010	P16336	protein_cc	4632407P	tubulin ty	Ttll4
401	Cyp27a1	0.451	1.523	0.687	0.001	0.158	0.262	11.967	10.817	11.424	104086	Cyp27a1	chr1	+	NM_0242	P8046	protein_cc	1300013A	cytochron	Cyp27a1
402	Prkag3	0.552	0.990	0.547	0.182	1.000	0.423	0.871	0.015	0.000	241113	Prkag3	chr1	-	NM_1537	P13918	protein_cc	Amkg3| A	protein kir	Prkag3
403	Wnt6	0.743	2.213	1.644	0.196	0.183	0.569	0.456	0.027	1.173	22420	Wnt6	chr1	+	NM_0095	P5247	protein_cc	AA409270	wingless-t	Wnt6
404	Wnt10a	1.000	1.000	1.000	1.000	1.000	1.000	0.000	0.000	0.000	22409	Wnt10a	chr1	+	NM_0095	P8560	protein_cc	.	wingless-t	Wnt10a
405	Cdk5r2	0.750	2.619	1.964	0.200	0.183	0.524	0.448	0.033	1.422	12570	Cdk5r2	chr1	+	NM_0098	P16995	protein_cc	B230310J	cyclin-dep	Cdk5r2
406	Fev	1.005	1.405	1.412	0.000	0.182	0.423	0.000	0.008	0.498	260298	Fev	chr1	-	NM_1531	P8039	protein_cc	mPet-1| P	FEV (ETS o	Fev
407	Cryba2	1.000	1.000	1.000	1.000	1.000	1.000	0.000	0.000	0.000	12958	Cryba2	chr1	-	NM_0215	P1598	protein_cc	E130107M	crystallin,	Cryba2
408	Ccdc108	1.000	1.000	1.000	1.000	1.000	1.000	0.000	0.000	0.000	241116	Ccdc108	chr1	-	NM_0010	P21117	protein_cc	A230051F	coiled-coil	Ccdc108
409	Ihh	0.207	0.604	0.125	0.065	1.000	0.223	3.003	0.728	0.000	16147	Ihh	chr1	-	NM_0105	P24795	protein_cc	HHG-2	Indian hed	Ihh
410	Nhej1	1.076	0.740	0.796	0.759	0.099	0.674	4.579	4.685	4.250	75570	Nhej1	chr1	-	NM_0293	P7755	protein_cc	1700029B	nonhomol	Nhej1
411	Slc23a3	3.269	0.306	1.000	0.305	1.000	0.423	0.001	1.710	0.000	22626	Slc23a3	chr1	-	NM_1943	P20810	protein_cc	SVCT3| Ys	solute car	Slc23a3
412	Cnppd1	0.586	1.063	0.623	0.000	0.709	0.124	10.156	9.385	9.473	69171	Cnppd1	chr1	-	NM_0269	P22924	protein_cc	1810031K	cyclin Pas	Cnppd1
413	Fam134a	0.268	1.647	0.441	0.000	0.012	0.030	10.225	8.325	9.044	227298	Fam134a	chr1	+	NM_0013	P11751	protein_cc	.	family wit	Fam134a
414	Zfand2b	0.561	1.956	1.097	0.001	0.000	0.380	7.712	6.878	7.846	68818	Zfand2b	chr1	+	NM_0011	P18479	protein_cc	1110060O	zinc finger	Zfand2b
415	Abcb6	0.185	1.263	0.233	0.000	0.131	0.017	8.661	6.225	6.561	74104	Abcb6	chr1	-	NM_0237	P23751	protein_cc	1200005B	ATP-bindi	Abcb6
416	Atg9a	0.682	1.090	0.743	0.016	0.514	0.054	7.964	7.412	7.536	245860	Atg9a	chr1	-	NM_0010	P11465	protein_cc	Apg9l1| A	autophagy	Atg9a
417	Ankzf1	0.987	0.779	0.769	0.827	0.004	0.131	6.903	6.883	6.523	52231	Ankzf1	chr1	+	NM_0012	P14448	protein_cc	1300008P	ankyrin re	Ankzf1
418	Glb1l	0.935	0.873	0.817	0.633	0.406	0.055	6.601	6.504	6.309	74577	Glb1l	chr1	-	NM_0290	P6036	protein_cc	4833408P	galactosid	Glb1l
419	Stk16	0.328	1.563	0.512	0.000	0.073	0.069	11.305	9.695	10.340	20872	Stk16	chr1	+	NM_0012	P26793	protein_cc	EDPK| Krc	serine/thr	Stk16
420	Tuba4a	0.316	1.384	0.437	0.000	0.141	0.038	12.214	10.553	11.021	22145	Tuba4a	chr1	-	NM_0094	P14988	protein_cc	M[a]4| Tu	tubulin, al	Tuba4a
421	A630095N	1.000	1.000	1.000	1.000	1.000	1.000	0.000	0.000	0.000	381272	A630095N	chr1	-	NM_0012	P24732	protein_cc	Gm1624	RIKEN cDN	A630095N
422	Dnajb2	1.380	0.636	0.879	0.001	0.009	0.550	8.504	8.969	8.317	56812	Dnajb2	chr1	+	NM_0011	P6754	protein_cc	2700059H	DnaJ heat	Dnajb2
423	Ptprn	0.181	1.756	0.318	0.001	0.199	0.075	2.533	0.068	0.881	19275	Ptprn	chr1	-	NM_0089	P25986	protein_cc	IA-2| mIA-	protein ty	Ptprn
424	Resp18	1.000	1.000	1.000	1.000	1.000	1.000	0.000	0.000	0.000	19711	Resp18	chr1	-	NM_0090	P24277	protein_cc	AI851012	regulated	Resp18
425	Dnpep	1.614	0.921	1.487	0.018	0.469	0.019	8.270	8.961	8.842	13437	Dnpep	chr1	-	NM_0168	P25394	protein_cc	AA407814	aspartyl a	Dnpep
426	Des	0.208	3.484	0.723	0.008	0.099	0.575	4.684	2.415	4.216	13346	Des	chr1	+	NM_0100	P18636	protein_cc	.	desmin	Des
427	Gm15179	1.030	3.842	3.956	0.000	0.181	0.423	0.001	0.043	1.985	10050324	Gm15179	chr1	-	NR_03797	.	lncRNA	OTTMUSG	predicted	Gm15179
428	Speg	0.462	1.585	0.732	0.096	0.406	0.668	2.921	1.807	2.471	11790	Speg	chr1	+	NM_0074	P26572	protein_cc	Apeg1| A\	SPEG com	Speg
429	Gmppa	2.042	0.724	1.478	0.005	0.055	0.119	7.207	8.237	7.771	69080	Gmppa	chr1	+	NM_1337	P11823	protein_cc	1810012N	GDP-man	Gmppa
430	Asic4	1.000	1.000	1.000	1.000	1.000	1.000	0.000	0.000	0.000	241118	Asic4	chr1	+	NM_1830	P14624	protein_cc	Accn4| BN	acid-sensi	Asic4
431	Chpf	2.226	0.851	1.893	0.005	0.531	0.400	4.300	5.454	5.220	74241	Chpf	chr1	-	NM_0010	P22162	protein_cc	1700028N	chondroiti	Chpf
432	Tmem198	1.238	0.456	0.565	0.421	1.000	0.422	0.824	1.132	0.000	319998	Tmem198	chr1	+	NM_1770	P5320	protein_cc	A230078I	transmem	Tmem198
433	Obsl1	0.526	0.868	0.456	0.156	0.727	0.285	1.630	0.702	0.497	98733	Obsl1	chr1	-	NM_1788	P477	protein_cc	AW822221	obscurin-l	Obsl1
434	Inha	1.000	1.000	1.000	1.000	1.000	1.000	0.000	0.000	0.000	16322	Inha	chr1	+	NM_0105	P9912	protein_cc	AW55507	inhibin alp	Inha
435	Stk11ip	1.217	1.008	1.226	0.000	0.865	0.117	4.623	4.906	4.917	71728	Stk11ip	chr1	+	NM_0278	P2311	protein_cc	1200014D	serine/thr	Stk11ip
436	Slc4a3	0.668	0.355	0.237	0.311	1.000	0.214	2.079	1.496	0.000	20536	Slc4a3	chr1	+	NM_0092	P14086	protein_cc	A930038D	solute car	Slc4a3
437	Gm17751	1.000	1.000	1.000	1.000	1.000	1.000	0.000	0.000	0.000	620079	Gm17751	chr1	-	NR_03801	.	lncRNA		predicted	Gm17751
438	Epha4	2.298	1.364	3.134	0.039	0.244	0.102	2.211	3.411	3.859	13838	Epha4	chr1	-	NM_0079	P18452	protein_cc	2900005C	Eph recep	Epha4
439	Pax3	1.005	1.405	1.412	0.000	0.182	0.423	0.000	0.008	0.498	18505	Pax3	chr1	-	NM_0011	P9871	protein_cc	Pax-3| Sp	paired box	Pax3
440	Sgpp2	1.548	1.875	2.902	0.442	0.053	0.196	2.702	3.332	4.239	433323	Sgpp2	chr1	+	NM_0010	P16400	protein_cc	Spp2| SPP	sphingosir	Sgpp2
441	Farsb	1.226	0.934	1.145	0.110	0.580	0.381	9.946	10.239	10.141	23874	Farsb	chr1	-	NM_0012	P24033	protein_cc	C76708| F	phenylalar	Farsb
442	Mogat1	0.528	1.765	0.932	0.005	0.408	0.933	2.954	2.033	2.852	68393	Mogat1	chr1	+	NM_0267	P3609	protein_cc	0610030A	monoacyl	Mogat1

443	Acsl3	0.443	1.494	0.662	0.000	0.290	0.369	9.089	7.915	8.495	74205	Acsl3	chr1	+	NM_0288	P18269	protein_cc	2610510B	acyl-CoA s	Acsl3
444	Utp14b	1.957	0.605	1.183	0.001	0.092	0.790	5.117	6.086	5.360	195434	Utp14b	chr1	+	NM_0010	P18229	protein_cc	4932411L	UTP14B sr	Utp14b
445	Kcne4	61.417	0.659	40.474	0.000	0.128	0.158	2.199	8.140	7.538	57814	Kcne4	chr1	+	NM_0213	P725	protein_cc	AW48793	potassium	Kcne4
446	Scg2	0.673	1.387	0.933	0.185	0.187	0.889	0.587	0.015	0.486	20254	Scg2	chr1	-	NM_0091	P3963	protein_cc	Chgc| SgII	secretogra	Scg2
447	Ap1s3	1.250	0.715	0.894	0.471	0.343	0.620	4.253	4.575	4.092	252903	Ap1s3	chr1	-	NM_1830	P24053	protein_cc	1190009B	adaptor-re	Ap1s3
448	Wdfy1	0.790	0.908	0.717	0.080	0.399	0.100	8.483	8.142	8.003	69368	Wdfy1	chr1	-	NM_0011	P14059	protein_cc	1700013B	WD repea	Wdfy1
449	Mrpl44	1.077	0.849	0.915	0.562	0.293	0.516	7.780	7.887	7.652	69163	Mrpl44	chr1	+	NM_0010	P8341	protein_cc	1810030E	mitochond	Mrpl44
450	Serpine2	0.643	1.473	0.947	0.344	0.399	0.747	4.745	4.108	4.667	20720	Serpine2	chr1	-	NM_0092	P23085	protein_cc	B230326N	serine (or	Serpine2
451	Fam124b	1.000	1.000	1.000	1.000	1.000	1.000	0.000	0.000	0.000	241128	Fam124b	chr1	-	NM_1734	P8092	protein_cc	A830043J	family wit	Fam124b
452	Cul3	0.871	0.997	0.868	0.030	0.984	0.607	8.868	8.669	8.664	26554	Cul3	chr1	-	NM_0167	P26666	protein_cc	KIAA0617	cullin 3	Cul3
453	1700016L	1.000	1.000	1.000	1.000	1.000	1.000	0.000	0.000	0.000	72208	1700016L	chr1	+	NR_04046	.	lncRNA	Jr3	RIKEN cDN	1700016L2
454	Dock10	3.552	0.442	1.570	0.003	0.007	0.004	3.862	5.690	4.512	210293	Dock10	chr1	-	NM_1752	P3634	protein_cc	9330153B	dedicator	Dock10
455	Nyap2	1.000	1.000	1.000	1.000	1.000	1.000	0.000	0.000	0.000	241134	Nyap2	chr1	+	NM_1728	P5866	protein_cc	9430031J1	neuronal t	Nyap2
456	Irs1	0.336	1.312	0.441	0.000	0.616	0.211	7.902	6.328	6.720	16367	Irs1	chr1	-	NM_0105	P16792	protein_cc	G972R| IR	insulin rec	Irs1
457	Rhbdd1	2.043	0.867	1.772	0.000	0.289	0.166	7.072	8.103	7.898	76867	Rhbdd1	chr1	+	NM_0297	P7867	protein_cc	4930418P	rhomboid	Rhbdd1
458	Col4a4	0.838	0.461	0.386	0.776	1.000	0.422	1.373	1.118	0.000	12829	Col4a4	chr1	-	NM_0077	P25821	protein_cc	E130010N	collagen, t	Col4a4
459	Col4a3	0.397	0.671	0.267	0.106	0.063	0.260	4.582	3.250	2.675	12828	Col4a3	chr1	+	NM_0077	P25205	protein_cc	alpha3(IV)	collagen, t	Col4a3
460	Mff	1.475	1.122	1.655	0.015	0.293	0.053	8.062	8.623	8.789	75734	Mff	chr1	+	NM_0294	P399	protein_cc	5230400G	mitochond	Mff
461	Tm4sf20	53.897	2.843	153.237	0.001	0.208	0.293	1.328	7.080	8.587	66261	Tm4sf20	chr1	-	NM_0254	P14728	protein_cc	1810018L	transmem	Tm4sf20
462	Agfg1	3.900	0.679	2.646	0.000	0.008	0.060	6.779	8.742	8.183	15463	Agfg1	chr1	+	NM_0104	P19102	protein_cc	AU045498	ArfGAP wi	Agfg1
463	Slc19a3	1.581	0.879	1.390	0.301	0.744	0.423	0.000	0.661	0.476	80721	Slc19a3	chr1	-	NM_0305	P2102	protein_cc	A230084E	solute car	Slc19a3
464	Ccl20	1.660	5.038	8.360	0.269	0.141	0.340	0.001	0.732	3.065	20297	Ccl20	chr1	+	NM_0011	P16639	protein_cc	CKb4| exc	chemokin	Ccl20
465	Daw1	1.000	1.000	1.000	1.000	1.000	1.000	0.000	0.000	0.000	71227	Daw1	chr1	+	NM_0277	P17029	protein_cc	4930563E	dynein ass	Daw1
466	Sphkap	1.000	1.000	1.000	1.000	1.000	1.000	0.000	0.000	0.000	77629	Sphkap	chr1	-	NM_1724	P13498	protein_cc	4930544G	SPHK1 inte	Sphkap
467	Pid1	0.977	1.400	1.367	0.490	0.001	0.024	10.682	10.648	11.133	98496	Pid1	chr1	-	NM_0010	P7410	protein_cc	5033414K	phosphoty	Pid1
468	Dner	1.000	1.000	1.000	1.000	1.000	1.000	0.000	0.000	0.000	227325	Dner	chr1	-	NM_1529	P24608	protein_cc	A930026D	delta/notc	Dner
469	Trip12	1.502	0.815	1.224	0.009	0.089	0.158	9.603	10.190	9.895	14897	Trip12	chr1	-	NM_1339	P10944	protein_cc	1110036I	thyroid ho	Trip12
470	Fbxo36	0.274	2.166	0.594	0.002	0.006	0.139	5.130	3.264	4.378	66153	Fbxo36	chr1	+	NM_0253	P2290	protein_cc	0610008D	F-box prot	Fbxo36
471	Slc16a14	1.000	1.000	1.000	1.000	1.000	1.000	0.000	0.000	0.000	71781	Slc16a14	chr1	-	NM_0279	P22513	protein_cc	1110004H	solute car	Slc16a14
472	A530032D	12.300	0.260	3.198	0.070	0.125	0.272	0.002	3.622	1.679	381287	A530032D	chr1	-	NM_2136	P26575	protein_cc	Gm1888	RIKEN cDN	A530032D
473	Gm7609	1.581	0.906	1.432	0.301	0.802	0.423	0.000	0.661	0.519	665378	Gm7609	chr1	+	NM_0010	P11096	protein_cc	EG665378	predicted	Gm7609
474	C130026I	2.934	0.447	1.312	0.356	0.430	0.333	0.402	1.954	0.793	620078	C130026I	chr1	-	NM_1752	P1104	protein_cc	4930565N	RIKEN cDN	C130026I2
475	Sp110	3.409	0.558	1.902	0.003	0.015	0.041	4.221	5.990	5.149	109032	Sp110	chr1	-	NM_0301	P18320	protein_cc	5031415C	Sp110 nuc	Sp110
476	Sp140	6.829	0.336	2.296	0.004	0.009	0.109	4.659	7.431	5.858	434484	Sp140	chr1	+	NM_0010	P20870	protein_cc	.	Sp140 nuc	Sp140
477	Sp100	1.070	0.425	0.454	0.834	0.113	0.091	4.056	4.154	2.918	20684	Sp100	chr1	+	NM_0136	P10232	protein_cc	A430075G	nuclear an	Sp100
478	A630001G	1.352	0.632	0.855	0.336	0.353	0.822	2.598	3.033	2.372	319997	A630001G	chr1	-	NM_1770	P17092	protein_cc	.	RIKEN cDN	A630001G
479	9930111H	1.000	1.000	1.000	1.000	1.000	1.000	0.000	0.000	0.000	77762	9930111H	chr1	+	NR_10808	.	lncRNA	.	RIKEN cDN	9930111H
480	Cab39	1.316	0.918	1.208	0.045	0.373	0.413	8.583	8.979	8.856	12283	Cab39	chr1	+	NM_1337	P2825	protein_cc	AA408805	calcium bi	Cab39
481	Itm2c	1.952	1.081	2.110	0.081	0.724	0.055	8.877	9.842	9.954	64294	Itm2c	chr1	+	NM_0224	P6887	protein_cc	3110038L	integral m	Itm2c
482	4933407L	1.000	1.000	1.000	1.000	1.000	1.000	0.000	0.000	0.000	71141	4933407L	chr1	+	NR_03769	.	lncRNA	.	RIKEN cDN	4933407L2
483	Gpr55	1.000	1.000	1.000	1.000	1.000	1.000	0.000	0.000	0.000	227326	Gpr55	chr1	-	NM_0010	P20217	protein_cc	CTFL| Gm	G protein-	Gpr55
484	Spata3	1.000	1.000	1.000	1.000	1.000	1.000	0.000	0.000	0.000	70060	Spata3	chr1	+	NM_0011	P11507	protein_cc	1700011N	spermatog	Spata3
485	2810459M	0.458	1.961	0.899	0.008	0.000	0.628	8.581	7.455	8.427	72792	2810459M	chr1	+	NM_0011	P2993	protein_cc	.	RIKEN cDN	2810459M
486	Psmd1	0.875	1.030	0.902	0.050	0.678	0.472	11.058	10.866	10.908	70247	Psmd1	chr1	+	NM_0273	P22994	protein_cc	2410026J1	proteason	Psmd1
487	Htr2b	1.572	0.636	1.000	0.304	1.000	0.423	0.000	0.653	0.000	15559	Htr2b	chr1	-	NM_0083	P531	protein_cc	5-HT2B| A	5-hydroxy	Htr2b
488	Armc9	0.912	0.325	0.297	0.863	0.091	0.398	2.516	2.382	0.762	78795	Armc9	chr1	+	NM_0301	P585	protein_cc	3830422A	armadillo	Armc9
489	B3gnt7	1.000	1.000	1.000	1.000	1.000	1.000	0.000	0.000	0.000	227327	B3gnt7	chr1	+	NM_1452	P19812	protein_cc	beta-3GnT	UDP-GlcN	B3gnt7
490	Ncl	1.454	0.945	1.373	0.021	0.515	0.043	11.864	12.403	12.321	17975	Ncl	chr1	-	NM_0108	P13462	protein_cc	B530004O	nucleolin	Ncl
491	Snora75	0.722	4.140	2.990	0.667	0.056	0.303	1.988	1.519	3.568	10030374	Snora75	chr1	-	NR_02847	.	snoRNA	MBI-55	small nucl	Snora75
492	Gm24148	1.000	1.000	1.000	1.000	1.000	1.000	0.000	0.000	0.000	10246589	Gm24148	chr1	-	NR_10618	.	miRNA	mir-3535|	predicted	Gm24148
493	Snord82	1.580	0.633	1.000	0.305	1.000	0.423	0.000	0.660	0.000	80828	Snord82	chr1	-	NR_00285	.	snoRNA	Rnu82| U8	small nucl	Snord82
494	C130036L	2.945	0.665	1.958	0.172	0.484	0.382	1.116	2.674	2.085	319336	C130036L	chr1	+	NR_01550	.	lncRNA	ENSMUSG	RIKEN cDN	C130036L2
495	Nmur1	1.000	1.000	1.000	1.000	1.000	1.000	0.000	0.000	0.000	14767	Nmur1	chr1	-	NM_0103	P1276	protein_cc	FM-3| Gpr	neuromed	Nmur1
496	1700019O	1.000	1.000	1.000	1.000	1.000	1.000	0.000	0.000	0.000	71863	1700019O	chr1	+	NM_0279	P16168	protein_cc	.	RIKEN cDN	1700019O
497	Ptma	1.239	1.020	1.264	0.003	0.709	0.090	11.471	11.780	11.808	19231	Ptma	chr1	+	NM_0089	P17601	protein_cc	Thym	prothymo	Ptma
498	Pde6d	1.575	1.060	1.669	0.038	0.801	0.371	6.420	7.075	7.159	18582	Pde6d	chr1	-	NM_0088	P8957	protein_cc	AI841218|	phosphod	Pde6d
499	Cops7b	1.182	1.097	1.297	0.378	0.547	0.181	6.870	7.111	7.245	26895	Cops7b	chr1	+	NM_1729	P19070	protein_cc	D1Wsu66e	COP9 sign	Cops7b
500	Nppc	1.000	1.000	1.000	1.000	1.000	1.000	0.000	0.000	0.000	18159	Nppc	chr1	-	NM_0109	P2788	protein_cc	CNP| Ibab	natriureti	Nppc
501	Dis3l2	0.576	2.243	1.293	0.001	0.000	0.086	5.382	4.587	5.753	208718	Dis3l2	chr1	+	NM_0011	P14175	protein_cc	4930429A	DIS3 like 3	Dis3l2
502	Alppl2	1.000	1.000	1.000	1.000	1.000	1.000	0.000	0.000	0.000	11650	Alppl2	chr1	-	NM_0074	P13009	protein_cc	Akp5| C77	alkaline ph	Alppl2
503	Alpi	1.000	1.000	1.000	1.000	1.000	1.000	0.000	0.000	0.000	76768	Alpi	chr1	-	NM_0010	P4851	protein_cc	2010001C	alkaline ph	Alpi
504	Akp3	1.000	1.000	1.000	1.000	1.000	1.000	0.000	0.000	0.000	11648	Akp3	chr1	+	NM_0074	P16473	protein_cc	Akp-3| dIA	alkaline ph	Akp3
505	Ecel1	1.000	1.000	1.000	1.000	1.000	1.000	0.000	0.000	0.000	13599	Ecel1	chr1	-	NM_0012	P10328	protein_cc	DINE| xce	endothelin	Ecel1
506	Prss56	1.572	0.636	1.000	0.304	1.000	0.423	0.000	0.653	0.000	69453	Prss56	chr1	+	NM_0270	P16185	protein_cc	1700027L	protease,	Prss56
507	Chrnd	1.000	1.000	1.000	1.000	1.000	1.000	0.000	0.000	0.000	11447	Chrnd	chr1	+	NM_0216	P13240	protein_cc	Achr-4| Ac	cholinergi	Chrnd
508	Chrng	1.000	1.000	1.000	1.000	1.000	1.000	0.000	0.000	0.000	11449	Chrng	chr1	+	NM_0096	P6905	protein_cc	Achr-3| Ac	cholinergi	Chrng
509	Eif4e2	1.244	1.040	1.294	0.021	0.790	0.412	9.112	9.427	9.484	26987	Eif4e2	chr1	+	NM_0233	P4958	protein_cc	2700069E	eukaryoti	Eif4e2
510	Efhd1	0.467	2.169	1.012	0.185	0.185	0.989	1.137	0.038	1.155	98363	Efhd1	chr1	+	NM_0288	P16142	protein_cc	4931430I	EF hand d	Efhd1
511	Gigyf2	1.094	0.545	0.596	0.296	0.005	0.150	8.208	8.337	7.461	227331	Gigyf2	chr1	+	NM_0011	P10317	protein_cc	2610016F	GRB10 int	Gigyf2
512	Kcnj13	1.572	0.636	1.000	0.304	1.000	0.423	0.000	0.653	0.000	10004059	Kcnj13	chr1	-	NM_0011	P9798	protein_cc	10004059	potassium	Kcnj13
513	3110079O	0.716	0.995	0.712	0.182	1.000	0.423	0.490	0.008	0.000	73234	3110079O	chr1	+	NM_0284	P14792	protein_cc	Snorc	RIKEN cDN	3110079O
514	Ngef	0.027	3.839	0.104	0.000	0.041	0.003	7.643	2.432	4.373	53972	Ngef	chr1	-	NM_0198	P11108	protein_cc	ephexin|	neuronal g	Ngef
515	Neu2	0.167	1.818	0.303	0.042	0.290	0.273	3.796	1.211	2.074	23956	Neu2	chr1	+	NM_0011	P5640	protein_cc	MBS| MSS	neuramini	Neu2
516	Inpp5d	1.452	1.252	1.818	0.162	0.177	0.276	3.969	4.507	4.831	16331	Inpp5d	chr1	+	NM_0105	P3310	protein_cc	p150Ship|	inositol po	Inpp5d
517	Atg16l1	1.107	1.070	1.184	0.596	0.701	0.066	7.244	7.391	7.488	77040	Atg16l1	chr1	+	NM_0298	P3615	protein_cc	1500009K	autophagy	Atg16l1
518	Scarna6	2.878	0.654	1.884	0.035	0.142	0.268	1.004	2.530	1.918	10021741	Scarna6	chr1	+	NR_02851	.	guide_RNA	MBI-46	small Caja	Scarna6
519	Sag	1.580	0.633	1.000	0.305	1.000	0.423	0.000	0.660	0.000	20215	Sag	chr1	+	NM_0091	P23573	protein_cc	A930001K	S-antigen,	Sag
520	Dgkd	1.189	0.778	0.925	0.561	0.407	0.653	6.515	6.765	6.403	227333	Dgkd	chr1	+	NM_1776	P4957	protein_cc	AI841987|	diacylglyc	Dgkd
521	Usp40	0.493	1.193	0.588	0.004	0.519	0.145	8.122	7.100	7.355	227334	Usp40	chr1	-	NM_0011	P10140	protein_cc	B230215L	ubiquitin s	Usp40
522	Ugt1a10	0.111	1.041	0.116	0.000	0.873	0.022	5.005	1.835	1.893	394430	Ugt1a10	chr1	+	NM_2016	P20143	protein_cc	A13| Ugt1	UDP glyco	Ugt1a10
523	Ugt1a9	1.000	1.000	1.000	1.000	1.000	1.000	0.000	0.000	0.000	394434	Ugt1a9	chr1	+	NM_2016	P16982	protein_cc	mUGTBr/F	UDP glucu	Ugt1a9
524	Ugt1a7c	1.000	1.000	1.000	1.000	1.000	1.000	0.000	0.000	0.000	394432	Ugt1a7c	chr1	+	NM_2016	P18762	protein_cc	A10'| A13	UDP glucu	Ugt1a7c
525	Ugt1a6b	0.321	0.881	0.283	0.000	0.043	0.001	14.092	12.452	12.270	394435	Ugt1a6b	chr1	+	NM_2014	P24121	protein_cc	A9'| Ugt1a	UDP glucu	Ugt1a6b
526	Ugt1a6a	0.552	0.990	0.547	0.182	1.000	0.423	0.871	0.015	0.000	94284	Ugt1a6a	chr1	+	NM_1450	P14042	protein_cc	UDPGT| U	UDP glucu	Ugt1a6a
527	Ugt1a5	0.498	0.410	0.204	0.000	0.024	0.026	5.826	4.819	3.534	394433	Ugt1a5	chr1	+	NM_2016	P15849	protein_cc	.	UDP glucu	Ugt1a5
528	Ugt1a2	1.000	1.000	1.000	1.000	1.000	1.000	0.000	0.000	0.000	22236	Ugt1a2	chr1	+	NM_0137	P18775	protein_cc	UDPGT 1-	UDP glucu	Ugt1a2
529	Dnajb3	0.732	1.245	0.911	0.637	0.730	0.929	1.589	1.138	1.454	15504	Dnajb3	chr1	-	NM_0082	P23016	protein_cc	Hsj3| MSJ	DnaJ heat	Dnajb3
530	Ugt1a1	0.224	0.431	0.097	0.024	0.013	0.126	5.223	3.067	1.851	394436	Ugt1a1	chr1	+	NM_2016	P5483	protein_cc	Gnt1| UDI	UDP glucu	Ugt1a1
531	Mroh2a	0.576	1.122	0.646	0.415	0.868	0.576	4.518	3.723	3.888	10004076	Mroh2a	chr1	+	NM_0012	P1455	protein_cc	ENSMUSG	maestro h	Mroh2a
532	Hjurp	0.310	1.536	0.476	0.000	0.065	0.061	8.911	7.221	7.840	381280	Hjurp	chr1	-	NM_1986	P3506	protein_cc	6430706D	Holliday ju	Hjurp
533	6430706D	0.312	1.777	0.554	0.001	0.039	0.140	8.453	6.771	7.600	.	6430706D	chr1	-	NR_04029	.	.	.	.	6430706D2

534	A730008H	0.457	1.541	0.704	0.160	0.458	0.588	4.280	3.149	3.773	.	A730008H	chr1	-	NM_1725(	P26240	.	.	.	A730008H
535	Trpm8	1.000	1.000	1.000	1.000	1.000	1.000	0.000	0.000	0.000	171382	Trpm8	chr1	+	NM_1342	P3392	protein_cc	CMR1| LT	transient r	Trpm8
536	Spp2	0.397	0.714	0.283	0.000	0.012	0.005	10.597	9.264	8.777	75396	Spp2	chr1	+	NM_0292(	P22641	protein_cc	06100380	secreted p	Spp2
537	Glrp1	5.090	0.196	0.999	0.001	1.000	0.423	0.001	2.349	0.000	14659	Glrp1	chr1	-	NM_0081	P1086	protein_cc	GRP-1	glutamine	Glrp1
538	Gm19589	8.271	2.851	23.580	0.021	0.026	0.074	0.002	3.050	4.562	10050319	Gm19589	chr1	+	NR_03797	.	lncRNA	.	predicted	Gm19589
539	Arl4c	0.832	0.591	0.491	0.513	0.229	0.047	5.013	4.747	3.988	320982	Arl4c	chr1	-	NM_1773(	P13206	protein_cc	A630084N	ADP-ribos	Arl4c
540	Platr5	1.000	1.000	1.000	1.000	1.000	1.000	0.000	0.000	0.000	78650	Platr5	chr1	+	NR_04049	.	lncRNA	2410088K	pluripoter	Platr5
541	1700067G	1.000	1.000	1.000	1.000	1.000	1.000	0.000	0.000	0.000	73475	1700067G	chr1	+	NR_04047	.	lncRNA	.	RIKEN cDN	1700067G
542	Sh3bp4	1.690	0.663	1.120	0.032	0.123	0.807	2.652	3.409	2.815	98402	Sh3bp4	chr1	+	NM_1338	P3147	protein_cc	AI594717|	SH3-doma	Sh3bp4
543	C030007H	1.000	1.000	1.000	1.000	1.000	1.000	0.000	0.000	0.000	77331	C030007H	chr1	-	NR_04048	.	lncRNA	.	RIKEN cDN	C030007H
544	Agap1	1.291	1.161	1.498	0.026	0.275	0.165	7.209	7.577	7.792	347722	Agap1	chr1	+	NM_1781	P15975	protein_cc	Centg2| G	ArfGAP wi	Agap1
545	4933400F	2.536	0.771	1.954	0.351	0.711	0.142	2.051	3.393	3.017	74403	4933400F	chr1	-	NR_01554	.	lncRNA	1700091C	RIKEN cDN	4933400F2
546	Gbx2	1.580	0.633	1.000	0.305	1.000	0.423	0.000	0.660	0.000	14472	Gbx2	chr1	-	NM_0102(	P3534	protein_cc	D130058E	gastrulatic	Gbx2
547	Asb18	1.000	1.000	1.000	1.000	1.000	1.000	0.000	0.000	0.000	208372	Asb18	chr1	-	NM_1391	P16428	protein_cc	Asb-18	ankyrin re	Asb18
548	Iqca	1.000	1.000	1.000	1.000	1.000	1.000	0.000	0.000	0.000	74918	Iqca	chr1	-	NM_0291	P9774	protein_cc	4930465P	IQ motif c	Iqca
549	Ackr3	2.953	3.991	11.787	0.139	0.035	0.128	2.919	4.482	6.478	12778	Ackr3	chr1	+	NM_0077	P6786	protein_cc	AW54127(	atypical ch	Ackr3
550	Cops8	1.299	1.247	1.620	0.007	0.021	0.055	8.000	8.377	8.696	108679	Cops8	chr1	+	NM_1338(	P940	protein_cc	1700009J(	COP9 sign	Cops8
551	Col6a3	0.236	0.956	0.226	0.015	0.904	0.109	6.939	4.859	4.794	12835	Col6a3	chr1	-	NM_0012	P17484	protein_cc	AI507288|	collagen, t	Col6a3
552	Mlph	0.122	3.901	0.477	0.066	0.051	0.475	3.818	0.785	2.748	171531	Mlph	chr1	+	NM_0530	P24634	protein_cc	2210418F	melanoph	Mlph
553	Prlh	1.000	1.000	1.000	1.000	1.000	1.000	0.000	0.000	0.000	623503	Prlh	chr1	+	NM_0011(	P12260	protein_cc	EG623503	prolactin r	Prlh
554	Rab17	0.545	1.030	0.562	0.013	0.864	0.076	8.469	7.594	7.637	19329	Rab17	chr1	-	NM_0089	P623	protein_cc	AW41347	RAB17, m	Rab17
555	Lrrfip1	1.976	0.666	1.317	0.000	0.000	0.105	9.149	10.132	9.547	16978	Lrrfip1	chr1	+	NM_0011	P26417	protein_cc	AU02455C	leucine ric	Lrrfip1
556	Rbm44	0.716	0.995	0.712	0.182	1.000	0.423	0.490	0.008	0.000	329207	Rbm44	chr1	+	NM_0010	P13493	protein_cc	Gm817	RNA bindi	Rbm44
557	Ramp1	0.378	1.181	0.447	0.066	0.790	0.125	4.915	3.513	3.753	51801	Ramp1	chr1	+	NM_0168	P8923	protein_cc	9130218E	receptor (	Ramp1
558	Ube2f	2.022	0.797	1.611	0.000	0.148	0.250	7.935	8.951	8.623	67921	Ube2f	chr1	+	NM_0264	P13022	protein_cc	2510010F	ubiquitin-	Ube2f
559	Scly	0.622	0.763	0.475	0.079	0.339	0.233	9.750	9.065	8.675	50880	Scly	chr1	+	NM_0167	P12522	protein_cc	9830169H	selenocyst	Scly
560	Espnl	1.000	1.000	1.000	1.000	1.000	1.000	0.000	0.000	0.000	227357	Espnl	chr1	+	NM_0010	P11155	protein_cc	Gm556	espin-like	Espnl
561	Klhl30	1.580	0.633	1.000	0.305	1.000	0.423	0.000	0.660	0.000	70788	Klhl30	chr1	+	NM_0275	P22860	protein_cc	4631423F(	kelch-like	Klhl30
562	Fam132b	1.256	1.483	1.863	0.215	0.024	0.029	4.766	5.095	5.664	227358	Fam132b	chr1	+	NM_1733	P2134	protein_cc	4832406C	family wit	Fam132b
563	Ilkap	0.705	1.196	0.842	0.053	0.311	0.324	8.448	7.942	8.200	67444	Ilkap	chr1	-	NM_0233	P10462	protein_cc	0710007A	integrin-li	Ilkap
564	1700020N	1.000	1.000	1.000	1.000	1.000	1.000	0.000	0.000	0.000	67086	1700020N	chr1	-	NR_02692	.	lncRNA	.	RIKEN cDN	1700020N
565	Hes6	0.093	5.097	0.473	0.000	0.007	0.085	9.620	6.190	8.540	55927	Hes6	chr1	-	NM_0194	P19202	protein_cc	AI326893|	hairy and e	Hes6
566	Per2	0.148	16.341	2.413	0.002	0.034	0.357	6.675	3.915	7.945	18627	Per2	chr1	-	NM_0110	P6418	protein_cc	mKIAA034	period circ	Per2
567	Traf3ip1	1.335	0.923	1.232	0.421	0.796	0.020	4.981	5.398	5.282	74019	Traf3ip1	chr1	+	NM_0287	P13080	protein_cc	3930402D	TRAF3 inte	Traf3ip1
568	Asb1	0.758	1.333	1.010	0.183	0.081	0.973	6.129	5.729	6.143	65247	Asb1	chr1	+	NM_0010	P14477	protein_cc	17000290	ankyrin re	Asb1
569	Twist2	0.773	4.257	3.292	0.218	0.006	0.105	0.423	0.052	2.142	13345	Twist2	chr1	+	NM_0078	P22511	protein_cc	bHLHa39|	twist basic	Twist2
570	Hdac4	2.209	0.575	1.271	0.007	0.028	0.490	4.707	5.851	5.053	208727	Hdac4	chr1	-	NM_2072	P9629	protein_cc	4932408F	histone de	Hdac4
571	Ndufa10	0.475	1.196	0.568	0.013	0.247	0.125	11.916	10.843	11.101	67273	Ndufa10	chr1	-	NM_0241	P24149	protein_cc	2900053E	NADH deh	Ndufa10
572	Olfr1416	1.000	1.000	1.000	1.000	1.000	1.000	0.000	0.000	0.000	259040	Olfr1416	chr1	-	NM_1470	P15957	protein_cc	MOR103-3	olfactory r	Olfr1416
573	Olfr1415	1.000	1.000	1.000	1.000	1.000	1.000	0.000	0.000	0.000	258228	Olfr1415	chr1	-	NM_0010	P3739	protein_cc	MOR103-1	olfactory r	Olfr1415
574	Olfr1414	1.000	1.000	1.000	1.000	1.000	1.000	0.000	0.000	0.000	259041	Olfr1414	chr1	-	NM_1470	P7915	protein_cc	MOR103-2	olfactory r	Olfr1414
575	Olfr1413	1.000	1.000	1.000	1.000	1.000	1.000	0.000	0.000	0.000	259039	Olfr1413	chr1	+	NM_1470	P2230	protein_cc	MOR208-1	olfactory r	Olfr1413
576	Olfr1412	1.000	1.000	1.000	1.000	1.000	1.000	0.000	0.000	0.000	258274	Olfr1412	chr1	+	NM_1462	P21284	protein_cc	MOR208-4	olfactory r	Olfr1412
577	Olfr1411	1.000	1.000	1.000	1.000	1.000	1.000	0.000	0.000	0.000	258483	Olfr1411	chr1	+	NM_1464	P21115	protein_cc	MOR208-3	olfactory r	Olfr1411
578	Olfr1410	1.000	1.000	1.000	1.000	1.000	1.000	0.000	0.000	0.000	258484	Olfr1410	chr1	+	NM_1464	P20076	protein_cc	MOR208-2	olfactory r	Olfr1410
579	Olfr12	1.000	1.000	1.000	1.000	1.000	1.000	0.000	0.000	0.000	257890	Olfr12	chr1	+	NM_2068	P5948	protein_cc	MOR208-5	olfactory r	Olfr12
580	Myeov2	1.215	1.154	1.402	0.056	0.152	0.071	10.592	10.873	11.079	66915	Myeov2	chr1	-	NM_0011	P23264	protein_cc	1110002N	myeloma	Myeov2
581	Otos	1.000	1.000	1.000	1.000	1.000	1.000	0.000	0.000	0.000	260301	Otos	chr1	-	NM_1531	P17661	protein_cc	OTOSP	otospiralir	Otos
582	Gpc1	1.211	1.665	2.017	0.535	0.191	0.285	3.441	3.717	4.453	14733	Gpc1	chr1	+	NM_0166	P23419	protein_cc	AI462976	glypican 1	Gpc1
583	Ankmy1	1.000	1.000	1.000	1.000	1.000	1.000	0.000	0.000	0.000	241158	Ankmy1	chr1	-	NM_1728	P10056	protein_cc	4930483I1	ankyrin re	Ankmy1
584	Dusp28	0.722	1.010	0.729	0.015	0.941	0.103	6.897	6.427	6.441	67446	Dusp28	chr1	+	NM_1751	P4055	protein_cc	0710001B	dual speci	Dusp28
585	Rnpepl1	0.315	1.234	0.388	0.000	0.073	0.012	8.278	6.610	6.913	108657	Rnpepl1	chr1	+	NM_1814	P10406	protein_cc	1110014H	arginyl am	Rnpepl1
586	9430060I(	1.304	0.803	1.048	0.638	0.570	0.957	2.002	2.386	2.070	10003726	9430060I(	chr1	-	NR_01552	.	lncRNA	.	RIKEN cDN	9430060I0
587	Capn10	0.457	1.975	0.903	0.015	0.047	0.711	7.392	6.263	7.245	23830	Capn10	chr1	+	NM_0117	P14640	protein_cc	AW04967	calpain 10	Capn10
588	Gpr35	7.138	1.080	7.711	0.005	0.720	0.067	2.229	5.065	5.176	64095	Gpr35	chr1	+	NM_0011	P14598	protein_cc	KPG_007	G protein-	Gpr35
589	Aqp12	1.000	1.000	1.000	1.000	1.000	1.000	0.000	0.000	0.000	208760	Aqp12	chr1	+	NM_0011	P12512	protein_cc	AB084104	aquaporin	Aqp12
590	Kif1a	0.726	1.425	1.034	0.187	0.186	0.943	0.476	0.015	0.525	16560	Kif1a	chr1	-	NM_0012	P15205	protein_cc	ATSV| C6:	kinesin fa	Kif1a
591	Agxt	0.182	0.854	0.155	0.000	0.563	0.012	11.371	8.911	8.684	11611	Agxt	chr1	+	NM_0012	P4259	protein_cc	AGT| Agt1	alanine-gl	Agxt
592	2310007B	0.726	1.387	1.007	0.187	0.187	0.988	0.477	0.015	0.487	71874	2310007B	chr1	-	NM_0011	P7114	protein_cc	.	RIKEN cDN	2310007B
593	Sned1	0.901	0.886	0.798	0.813	0.763	0.725	0.834	0.684	0.509	208777	Sned1	chr1	+	NM_1724	P16590	protein_cc	67204551	sushi, nido	Sned1
594	Mterf4	0.919	0.635	0.583	0.471	0.009	0.077	7.630	7.507	6.851	69821	Mterf4	chr1	-	NM_1780	P11616	protein_cc	1810059A	mitochonc	Mterf4
595	Pask	0.605	4.917	2.974	0.200	0.052	0.313	0.789	0.064	2.361	269224	Pask	chr1	-	NM_0808	P14711	protein_cc	mKIAA013	PAS doma	Pask
596	Ppp1r7	1.131	1.058	1.197	0.343	0.611	0.098	8.622	8.800	8.881	66385	Ppp1r7	chr1	+	NM_0232	P7192	protein_cc	2310014J(	protein ph	Ppp1r7
597	Ano7	2.134	0.469	1.000	0.305	1.000	0.423	0.000	1.094	0.000	404545	Ano7	chr1	+	NM_0012	P22026	protein_cc	Ngep| Ng	anoctamin	Ano7
598	Hdlbp	1.196	0.976	1.167	0.024	0.141	0.207	11.224	11.481	11.446	110611	Hdlbp	chr1	-	NM_1338	P25884	protein_cc	1110005P	high densi	Hdlbp
599	Sept2	1.454	0.808	1.175	0.000	0.001	0.097	9.276	9.816	9.508	18000	Sept2	chr1	+	NM_0011	P20766	protein_cc	AW20899	septin 2	Sept2
600	Farp2	0.514	0.982	0.505	0.029	0.927	0.109	6.683	5.724	5.697	227377	Farp2	chr1	+	NM_1455	P4039	protein_cc	AI465173|	FERM, Rho	Farp2
601	Stk25	0.823	1.300	1.071	0.162	0.103	0.789	7.432	7.152	7.531	59041	Stk25	chr1	-	NM_0215	P22803	protein_cc	1500019J1	serine/thr	Stk25
602	Bok	0.308	2.340	0.720	0.000	0.002	0.113	5.823	4.122	5.349	51800	Bok	chr1	+	NM_0167	P4687	protein_cc	matador|	BCL2-relat	Bok
603	Thap4	0.937	1.089	1.021	0.292	0.282	0.889	7.105	7.011	7.135	67026	Thap4	chr1	-	NM_0259	P16076	protein_cc	2010320B	THAP dom	Thap4
604	Atg4b	0.869	0.911	0.791	0.029	0.154	0.163	8.132	7.929	7.794	66615	Atg4b	chr1	+	NM_1748	P3438	protein_cc	2510009N	autophagy	Atg4b
605	Dtymk	1.721	1.194	2.054	0.007	0.056	0.046	6.301	7.084	7.339	21915	Dtymk	chr1	-	NM_0011	P17701	protein_cc	AU044245	deoxythyr	Dtymk
606	Ing5	1.076	0.929	1.000	0.644	0.552	1.000	6.758	6.863	6.757	66262	Ing5	chr1	+	NM_0254	P7627	protein_cc	1700001C	inhibitor o	Ing5
607	D2hgdh	0.306	1.010	0.309	0.000	0.800	0.006	6.018	4.310	4.325	98314	D2hgdh	chr1	+	NM_1788	P4145	protein_cc	AA408776	D-2-hydro	D2hgdh
608	Gal3st2	0.326	2.147	0.699	0.000	0.018	0.213	1.667	0.048	1.151	381334	Gal3st2	chr1	+	NM_1993	P4494	protein_cc	Gm988| G	galactose-	Gal3st2
609	Gm9994	1.000	1.000	1.000	1.000	1.000	1.000	0.000	0.000	0.000	10004159	Gm9994	chr1	+	NM_0012	P2712	protein_cc	ENSMUSG	predicted	Gm9994
610	Gm6086	1.000	1.000	1.000	1.000	1.000	1.000	0.000	0.000	0.000	619597	Gm6086	chr1	+	NM_0010	P4746	protein_cc	EG619597	predicted	Gm6086
611	Neu4	1.005	1.405	1.412	0.000	0.182	0.423	0.000	0.008	0.498	241159	Neu4	chr1	+	NM_1737	P1957	protein_cc	93301661(	sialidase 4	Neu4
612	Pdcd1	0.743	2.306	1.713	0.196	0.183	0.557	0.456	0.027	1.232	18566	Pdcd1	chr1	-	NM_0087	P16929	protein_cc	Ly101| PD	programm	Pdcd1
613	4930440C	1.000	1.000	1.000	1.000	1.000	1.000	0.000	0.000	0.000	74689	4930440C	chr1	+	NR_04047	.	lncRNA	.	RIKEN cDN	4930440C
614	Fam174a	0.933	1.107	1.033	0.614	0.205	0.898	7.221	7.121	7.267	67698	Fam174a	chr1	+	NM_0263	P10940	protein_cc	2310044D	family wit	Fam174a
615	St8sia4	0.431	1.622	0.700	0.076	0.341	0.170	3.906	2.692	3.390	20452	St8sia4	chr1	-	NM_0091	P21248	protein_cc	PST| PST-	ST8 alpha-	St8sia4
616	4930598F	1.000	1.000	1.000	1.000	1.000	1.000	0.000	0.000	0.000	75381	4930598F	chr1	-	NR_04047	.	lncRNA	.	RIKEN cDN	4930598F1
617	1700063A	1.000	1.000	1.000	1.000	1.000	1.000	0.000	0.000	0.000	73454	1700063A	chr1	-	NR_04046	.	lncRNA	.	RIKEN cDN	1700063A
618	4930533P	1.000	1.000	1.000	1.000	1.000	1.000	0.000	0.000	0.000	75159	4930533P	chr1	-	NR_04047	.	lncRNA	.	RIKEN cDN	4930533P
619	Slco4c1	1.000	1.000	1.000	1.000	1.000	1.000	0.000	0.000	0.000	227394	Slco4c1	chr1	-	NM_1726	P26604	protein_cc	9530051F(	solute car	Slco4c1
620	Pact2	1.589	1.138	1.809	0.298	0.679	0.003	0.000	0.669	0.855	329217	Pact2	chr1	+	NR_13196	.	lncRNA	9530060I(	pluripoter	Pact2
621	Slco6b1	1.000	1.000	1.000	1.000	1.000	1.000	0.000	0.000	0.000	67854	Slco6b1	chr1	-	NR_12050	.	lncRNA	1700022N	solute car	Slco6b1
622	Slco6c1	1.000	1.000	1.000	1.000	1.000	1.000	0.000	0.000	0.000	74441	Slco6c1	chr1	-	NM_0289	P13774	protein_cc	4933404A	solute car	Slco6c1
623	D1Ertd622	1.398	0.822	1.149	0.088	0.051	0.615	6.354	6.837	6.554	52392	D1Ertd622	chr1	-	NM_1338	P23708	protein_cc	AI987691|	DNA segm	D1Ertd622
624	Ppip5k2	0.595	1.907	1.134	0.000	0.017	0.603	8.865	8.116	9.047	227399	Ppip5k2	chr1	-	NM_1737	P26286	protein_cc	AW55581	diphospho	Ppip5k2

625	Gin1	0.944	1.082	1.022	0.572	0.445	0.603	5.859	5.776	5.890	252876	Gin1	chr1	+	NM_0262	P26660	protein_cc	4930429M	gypsy retr	Gin1
626	Pam	1.205	0.617	0.744	0.682	0.363	0.555	7.726	7.995	7.299	18484	Pam	chr1	-	NM_0136	P17683	protein_cc	PHM	peptidylgl	Pam
627	B230216N	2.046	0.514	1.051	0.402	0.425	0.910	0.425	1.457	0.496	78603	B230216N	chr1	-	NR_03799	.	lncRNA	.	RIKEN cDN	B230216N
628	1810006J(	0.716	0.995	0.712	0.182	1.000	0.423	0.490	0.008	0.000	69030	1810006J(	chr1	+	NR_04043	.	lncRNA	.	RIKEN cDN	1810006J0
629	Slco6d1	1.000	1.000	1.000	1.000	1.000	1.000	0.000	0.000	0.000	70866	Slco6d1	chr1	+	NM_0011	P12437	protein_cc	4921511C	solute car	Slco6d1
630	Cntnap5b	1.000	1.000	1.000	1.000	1.000	1.000	0.000	0.000	0.000	241175	Cntnap5b	chr1	+	NM_1728	P24210	protein_cc	C230078M	contactin	Cntnap5b
631	Gm20268	1.000	1.000	1.000	1.000	1.000	1.000	0.000	0.000	0.000	10050452	Gm20268	chr1	+	NR_03798	.	lncRNA	.	predicted	Gm20268
632	Cdh20	0.552	0.990	0.547	0.182	1.000	0.423	0.871	0.015	0.000	23836	Cdh20	chr1	+	NM_0118	P7984	protein_cc	Cdh7	cadherin 2	Cdh20
633	Rnf152	0.097	1.601	0.156	0.000	0.015	0.007	10.141	6.780	7.460	320311	Rnf152	chr1	-	NM_0011	P23466	protein_cc	A930029B	ring finger	Rnf152
634	Pign	2.340	0.778	1.822	0.000	0.018	0.081	5.221	6.447	6.086	27392	Pign	chr1	-	NM_0137	P12509	protein_cc	Gm20308	phosphati	Pign
635	2310035C	0.892	1.147	1.023	0.045	0.003	0.712	6.214	6.049	6.247	227446	2310035C	chr1	+	NM_1731	P25258	protein_cc	6430401N	RIKEN cDN	2310035C
636	Tnfrsf11a	6.074	0.653	3.968	0.033	0.252	0.070	1.453	4.056	3.441	21934	Tnfrsf11a	chr1	+	NM_0093	P8127	protein_cc	Ly109| mF	tumor nec	Tnfrsf11a
637	Zcchc2	0.648	0.434	0.281	0.019	0.000	0.043	7.879	7.253	6.049	227449	Zcchc2	chr1	+	NM_0011	P10826	protein_cc	9930114B	zinc finger	Zcchc2
638	Gm20753	1.005	1.439	1.446	0.000	0.182	0.423	0.000	0.008	0.532	626049	Gm20753	chr1	-	NR_04063	.	lncRNA	.	predicted	Gm20753
639	Phlpp1	0.241	1.234	0.297	0.002	0.558	0.012	7.223	5.169	5.473	98432	Phlpp1	chr1	+	NM_1338	P14290	protein_cc	AI836256|	PH domai	Phlpp1
640	Bcl2	1.369	0.745	1.019	0.121	0.205	0.972	4.854	5.307	4.882	12043	Bcl2	chr1	-	NM_0097	P13194	protein_cc	AW98625(	B cell leuk	Bcl2
641	Kdsr	0.406	1.494	0.607	0.001	0.033	0.012	8.568	7.268	7.847	70750	Kdsr	chr1	-	NM_0275	P14210	protein_cc	6330410P	3-ketodihy	Kdsr
642	Vps4b	1.049	0.936	0.982	0.101	0.598	0.933	9.103	9.172	9.077	20479	Vps4b	chr1	-	NM_0091	P18388	protein_cc	8030489C	vacuolar p	Vps4b
643	Serpinb5	1.010	1.885	1.904	0.000	0.182	0.423	0.000	0.015	0.929	20724	Serpinb5	chr1	+	NM_0092	P20602	protein_cc	1110036M	serine (or	Serpinb5
644	Serpinb12	1.000	1.000	1.000	1.000	1.000	1.000	0.000	0.000	0.000	71869	Serpinb12	chr1	+	NM_0279	P11514	protein_cc	2300003F(	serine (or	Serpinb12
645	Serpinb13	1.000	1.000	1.000	1.000	1.000	1.000	0.000	0.000	0.000	241196	Serpinb13	chr1	+	NM_1728	P8981	protein_cc	5430417G	serine (or	Serpinb13
646	Serpinb3a	1.000	1.000	1.000	1.000	1.000	1.000	0.000	0.000	0.000	20248	Serpinb3a	chr1	-	NM_0091	P17770	protein_cc	1110001H	serine (or	Serpinb3a
647	Serpinb3d	1.000	1.000	1.000	1.000	1.000	1.000	0.000	0.000	0.000	394252	Serpinb3d	chr1	-	NM_2013	P9706	protein_cc	Serpinb3l1	serine (or	Serpinb3d
648	Serpinb3b	1.000	1.000	1.000	1.000	1.000	1.000	0.000	0.000	0.000	383548	Serpinb3b	chr1	-	NM_1986	P14712	protein_cc	Scca2-rs|	serine (or	Serpinb3b
649	Serpinb3c	1.000	1.000	1.000	1.000	1.000	1.000	0.000	0.000	0.000	381286	Serpinb3c	chr1	-	NM_2013	P11359	protein_cc	1110001H	serine (or	Serpinb3c
650	Serpinb11	1.000	1.000	1.000	1.000	1.000	1.000	0.000	0.000	0.000	66957	Serpinb11	chr1	+	NM_0258	P14788	protein_cc	2310046M	serine (or	Serpinb11
651	Serpinb7	1.005	1.405	1.412	0.000	0.182	0.423	0.000	0.008	0.498	116872	Serpinb7	chr1	+	NM_0275	P16992	protein_cc	4631416M	serine (or	Serpinb7
652	Serpinb2	1.030	3.842	3.956	0.000	0.181	0.423	0.001	0.043	1.985	18788	Serpinb2	chr1	+	NM_0011	P23352	protein_cc	ovalbumir	serine (or	Serpinb2
653	Serpinb10	0.728	0.915	0.666	0.632	0.900	0.682	1.935	1.477	1.349	241197	Serpinb10	chr1	+	NM_1980	P18569	protein_cc	9830131G	serine (or	Serpinb10
654	Serpinb8	0.232	1.552	0.360	0.030	0.177	0.249	4.153	2.048	2.681	20725	Serpinb8	chr1	+	NM_0114	P10312	protein_cc	CAP-2| CA	serine (or	Serpinb8
655	D830032E	1.005	1.405	1.412	0.000	0.182	0.423	0.000	0.008	0.498	100503	D830032E	chr1	+	NR_10230	.	lncRNA	.	RIKEN cDN	D830032E
656	Cdh7	1.000	1.000	1.000	1.000	1.000	1.000	0.000	0.000	0.000	241201	Cdh7	chr1	+	NM_1728	P14333	protein_cc	9330156F(	cadherin 7	Cdh7
657	Cdh19	1.000	1.000	1.000	1.000	1.000	1.000	0.000	0.000	0.000	227485	Cdh19	chr1	-	NM_0010	P22218	protein_cc	.	cadherin 1	Cdh19
658	Dsel	1.571	1.885	2.961	0.147	0.014	0.061	3.606	4.257	5.172	319901	Dsel	chr1	-	NM_0010	P21817	protein_cc	9330132E(	dermatan	Dsel
659	Cntnap5a	1.000	1.000	1.000	1.000	1.000	1.000	0.000	0.000	0.000	636808	Cntnap5a	chr1	+	NM_0010	P26401	protein_cc	Caspr5-1|	contactin	Cntnap5a
660	Tsn	1.045	1.107	1.157	0.587	0.194	0.182	10.441	10.504	10.652	22099	Tsn	chr1	-	NM_0116	P10675	protein_cc	2610034C	translin	Tsn
661	Nifk	2.235	0.919	2.053	0.002	0.568	0.118	8.170	9.330	9.208	67949	Nifk	chr1	+	NM_0264	P6203	protein_cc	AI852665|	nucleolar	Nifk
662	Clasp1	1.328	0.937	1.244	0.490	0.857	0.589	5.580	5.989	5.895	76707	Clasp1	chr1	+	NM_0297	P26491	protein_cc	1700030C	CLIP assoc	Clasp1
663	2900060B	1.023	1.213	1.240	0.960	0.616	0.668	9.764	9.796	10.074	68204	2900060B	chr1	-	NR_02790	.	lncRNA	.	RIKEN cDN	2900060B
664	Tfcp2l1	0.777	0.877	0.681	0.002	0.033	0.001	5.537	5.172	4.983	81879	Tfcp2l1	chr1	+	NM_0237	P1125	protein_cc	1810030F(	transcripti	Tfcp2l1
665	Gli2	1.010	1.825	1.844	0.000	0.182	0.423	0.000	0.015	0.883	14633	Gli2	chr1	-	NM_0010	P612	protein_cc	.	GLI-Krupp	Gli2
666	Inhbb	18.871	0.920	17.367	0.000	0.553	0.050	5.066	9.304	9.184	16324	Inhbb	chr1	-	NM_0083	P21414	protein_cc	.	inhibin be	Inhbb
667	Ralb	2.095	1.091	2.287	0.001	0.159	0.001	6.704	7.771	7.897	64143	Ralb	chr1	-	NM_0223	P15110	protein_cc	57304720	v-ral simia	Ralb
668	Tmem185	1.613	0.628	1.014	0.064	0.079	0.929	6.632	7.322	6.651	226351	Tmem185	chr1	+	NM_1461	P965	protein_cc	2500001K	transmem	Tmem185
669	3830432H	1.000	1.000	1.000	1.000	1.000	1.000	0.000	0.000	0.000	320069	3830432H	chr1	-	NR_12648	.	lncRNA	.	RIKEN cDN	3830432H
670	Epb4.1l5	0.328	1.845	0.605	0.006	0.011	0.188	7.649	6.041	6.925	.	Epb4.1l5	chr1	-	NM_1455	P12536	.	.	.	Epb4.1l5
671	Ptpn4	1.967	0.928	1.826	0.350	0.881	0.500	4.073	5.049	4.942	19258	Ptpn4	chr1	-	NM_0199	P19629	protein_cc	hPTP-MEG	protein ty	Ptpn4
672	Tmem177	0.819	1.865	1.527	0.342	0.067	0.424	5.879	5.591	6.490	66343	Tmem177	chr1	-	NM_1751	P18598	protein_cc	2300008B	transmem	Tmem177
673	Cfap221	0.716	0.995	0.712	0.182	1.000	0.423	0.490	0.008	0.000	226356	Cfap221	chr1	-	NM_0011	P11725	protein_cc	Gm101| P	cilia and fl	Cfap221
674	Sctr	1.000	1.000	1.000	1.000	1.000	1.000	0.000	0.000	0.000	319229	Sctr	chr1	+	NM_0010	P3876	protein_cc	65304020	secretin re	Sctr
675	Tmem37	0.075	6.304	0.474	0.000	0.019	0.094	9.527	5.794	8.450	170706	Tmem37	chr1	-	NM_0194	P6492	protein_cc	AI173373|	transmem	Tmem37
676	Dbi	0.978	1.188	1.161	0.405	0.210	0.503	14.461	14.429	14.677	13167	Dbi	chr1	-	NM_0078	P11107	protein_cc	ACBD1| A	diazepam	Dbi
677	3110009E	0.696	0.928	0.646	0.374	0.917	0.612	3.679	3.156	3.049	73103	3110009E	chr1	+	NM_0284	P22310	protein_cc	.	RIKEN cDN	3110009E
678	Steap3	0.126	1.969	0.248	0.000	0.182	0.028	6.845	3.853	4.831	68428	Steap3	chr1	-	NM_1331	P18105	protein_cc	1010001D	STEAP fam	Steap3
679	C1ql2	0.716	0.995	0.712	0.182	1.000	0.423	0.490	0.008	0.000	226359	C1ql2	chr1	+	NM_2072	P15801	protein_cc	BC040774	compleme	C1ql2
680	Marco	13.073	0.923	12.062	0.001	0.674	0.057	6.196	9.904	9.788	17167	Marco	chr1	-	NM_0107	P2607	protein_cc	AI323439|	macropha	Marco
681	En1	1.000	1.000	1.000	1.000	1.000	1.000	0.000	0.000	0.000	13798	En1	chr1	+	NM_0101	P24148	protein_cc	En-1| Mo-	engrailed	En1
682	Celrr	1.000	1.000	1.000	1.000	1.000	1.000	0.000	0.000	0.000	98452	Celrr	chr1	-	NR_03800	.	lncRNA	AI848258|	cerebellun	Celrr
683	Insig2	0.224	1.302	0.291	0.000	0.001	0.004	11.304	9.144	9.525	72999	Insig2	chr1	-	NM_0012	P25127	protein_cc	29000531I	insulin ind	Insig2
684	Ccdc93	1.402	1.066	1.494	0.001	0.081	0.002	5.981	6.468	6.560	70829	Ccdc93	chr1	+	NM_0299	P19245	protein_cc	4633402D	coiled-coil	Ccdc93
685	Htr5b	1.000	1.000	1.000	1.000	1.000	1.000	0.000	0.000	0.000	15564	Htr5b	chr1	-	NM_0104	P18705	protein_cc	5-Ht5b	5-hydroxy	Htr5b
686	Ddx18	2.174	0.638	1.387	0.000	0.001	0.131	8.277	9.397	8.749	66942	Ddx18	chr1	-	NM_0258	P18490	protein_cc	2310005B	DEAD (Asp	Ddx18
687	Dpp10	1.000	1.000	1.000	1.000	1.000	1.000	0.000	0.000	0.000	269109	Dpp10	chr1	-	NM_1990	P4176	protein_cc	6430601K	dipeptidyl	Dpp10
688	Actr3	2.052	0.773	1.587	0.000	0.012	0.068	8.770	9.807	9.436	74117	Actr3	chr1	-	NM_0120	P12432	protein_cc	1200003A	ARP3 actir	Actr3
689	Slc35f5	2.252	0.581	1.308	0.003	0.003	0.529	8.431	9.602	8.818	74150	Slc35f5	chr1	+	NM_0287	P18475	protein_cc	1300003P	solute car	Slc35f5
690	Gpr39	2.773	1.196	3.317	0.041	0.417	0.020	4.428	5.899	6.157	71111	Gpr39	chr1	+	NM_0276	P24870	protein_cc	4933415E	G protein-	Gpr39
691	Lypd1	4.991	1.093	5.455	0.115	0.831	0.028	0.361	2.681	2.809	72585	Lypd1	chr1	-	NM_1451	P4704	protein_cc	2700050C	Ly6/Plaur	Lypd1
692	Nckap5	1.394	0.660	0.920	0.611	0.420	0.935	2.551	3.031	2.431	210356	Nckap5	chr1	-	NM_1769	P12535	protein_cc	8430408F	NCK-assoc	Nckap5
693	Mgat5	4.279	1.638	7.009	0.006	0.010	0.005	0.717	2.814	3.527	107895	Mgat5	chr1	+	NM_1451	P10640	protein_cc	4930471A	mannosid	Mgat5
694	Tmem163	0.233	2.085	0.485	0.000	0.191	0.210	2.164	0.061	1.120	72160	Tmem163	chr1	-	NM_0281	P10797	protein_cc	2610024A	transmem	Tmem163
695	Acmsd	0.283	0.686	0.194	0.000	0.022	0.001	5.893	4.072	3.528	266645	Acmsd	chr1	+	NM_0010	P4506	protein_cc	.	amino car	Acmsd
696	2900009J(	0.352	1.230	0.433	0.051	0.745	0.019	3.718	2.211	2.510	10050274	2900009J(	chr1	-	NR_04529	.	lncRNA	.	RIKEN cDN	2900009J0
697	Ccnt2	0.580	1.246	0.723	0.024	0.227	0.190	7.305	6.520	6.838	72949	Ccnt2	chr1	+	NM_0283	P3301	protein_cc	29000411I	cyclin T2	Ccnt2
698	Map3k19	1.000	1.000	1.000	1.000	1.000	1.000	0.000	0.000	0.000	22625	Map3k19	chr1	-	NM_0117	P8880	protein_cc	Ysk4	mitogen-a	Map3k19
699	Rab3gap1	1.116	1.017	1.134	0.371	0.891	0.397	8.948	9.106	9.130	226407	Rab3gap1	chr1	+	NM_1786	P9196	protein_cc	1700003B	RAB3 GTP	Rab3gap1
700	Zranb3	0.260	0.411	0.107	0.071	0.171	0.199	4.514	2.572	1.290	226409	Zranb3	chr1	-	NM_0012	P1201	protein_cc	4933425L	zinc finger	Zranb3
701	R3hdm1	1.668	0.698	1.165	0.023	0.030	0.489	6.842	7.581	7.063	226412	R3hdm1	chr1	+	NM_1817	P10804	protein_cc	R3hdm	R3H doma	R3hdm1
702	Ubxn4	1.125	0.725	0.815	0.059	0.003	0.237	11.183	11.352	10.888	67812	Ubxn4	chr1	+	NM_0263	P8865	protein_cc	1300013G	UBX doma	Ubxn4
703	Lct	1.000	1.000	1.000	1.000	1.000	1.000	0.000	0.000	0.000	226413	Lct	chr1	-	NM_0010	P20346	protein_cc	Gm100| L	lactase	Lct
704	Mcm6	1.152	1.366	1.574	0.032	0.200	0.324	5.438	5.642	6.093	17219	Mcm6	chr1	-	NM_0085	P833	protein_cc	ASP-I1| D	minichron	Mcm6
705	Dars	0.952	0.852	0.811	0.674	0.099	0.341	8.763	8.692	8.460	226414	Dars	chr1	-	NM_1774	P24733	protein_cc	5730439G	aspartyl-t	Dars
706	Cxcr4	2.693	1.351	3.637	0.063	0.152	0.065	2.635	4.064	4.497	12767	Cxcr4	chr1	-	NM_0099	P12599	protein_cc	b2b220Clc	chemokin	Cxcr4
707	Thsd7b	1.000	1.000	1.000	1.000	1.000	1.000	0.000	0.000	0.000	210417	Thsd7b	chr1	+	NM_1724	P7665	protein_cc	1700074E	thrombos	Thsd7b
708	Cd55b	1.097	0.632	0.693	0.205	0.122	0.440	2.796	2.930	2.267	13137	Cd55b	chr1	-	NM_0078	P15518	protein_cc	AI323748|	CD55 mol	Cd55b
709	Cd55	1.356	1.440	1.953	0.000	0.010	0.048	6.078	6.517	7.043	13136	Cd55	chr1	-	NM_0100	P2737	protein_cc	Daf| Daf-(	CD55 mol	Cd55
710	Zp3r	1.146	0.632	0.725	0.729	1.000	0.422	0.465	0.661	0.000	22789	Zp3r	chr1	-	NM_0095	P8067	protein_cc	AV264399	zona pellu	Zp3r
711	C4bp	1.196	1.298	1.552	0.060	0.005	0.017	11.075	11.333	11.709	12269	C4bp	chr1	-	NM_0075	P7262	protein_cc	AI195242|	compleme	C4bp
712	C4bp-ps1	6.559	1.649	10.814	0.038	0.139	0.062	2.187	4.901	5.622	12270	C4bp-ps1	chr1	-	NR_02830	.	misc_RNA	C4bpb	compleme	C4bp-ps1
713	Pfkfb2	1.857	1.058	1.964	0.003	0.787	0.274	6.443	7.336	7.416	18640	Pfkfb2	chr1	-	NR_02785	.	protein_cc	4930568D	6-phosphc	Pfkfb2
714	Yod1	1.701	0.704	1.197	0.003	0.001	0.389	6.942	7.708	7.201	226418	Yod1	chr1	+	NM_1786	P22022	protein_cc	6330564D	YOD1 deu	Yod1
715	AA986860	1.361	1.688	2.297	0.133	0.038	0.112	3.457	3.902	4.657	212439	AA986860	chr1	+	NM_1776	P24924	protein_cc	E030025M	expressed	AA986860

716	Fcamr	1.000	1.000	1.000	1.000	1.000	1.000	0.000	0.000	0.000	64435	Fcamr	chr1	+	NM_1449	P24152	protein_cc	.	Fc recept	Fcamr
717	Pigr	0.255	0.725	0.185	0.000	0.034	0.003	12.831	10.861	10.397	18703	Pigr	chr1	+	NM_0110	P1577	protein_cc	.	polymeric	Pigr
718	Fcmr	0.367	2.638	0.967	0.266	0.195	0.976	2.178	0.731	2.130	69169	Fcmr	chr1	+	NM_0269	P19735	protein_cc	1810037B	Fc fragme	Fcmr
719	Il24	1.000	1.000	1.000	1.000	1.000	1.000	0.000	0.000	0.000	93672	Il24	chr1	-	NM_0530	P18139	protein_cc	FISP| Mda	interleukin	Il24
720	Il20	1.000	1.000	1.000	1.000	1.000	1.000	0.000	0.000	0.000	58181	Il20	chr1	-	NM_0213	P608	protein_cc	Zcyto10	interleukin	Il20
721	Il19	13.627	0.964	13.131	0.091	0.956	0.351	0.002	3.771	3.717	329244	Il19	chr1	-	NM_0010	P21422	protein_cc	.	interleukin	Il19
722	Il10	19.364	1.162	22.503	0.000	0.547	0.104	0.987	5.263	5.479	16153	Il10	chr1	+	NM_0105	P6921	protein_cc	CSIF| Il-10	interleukin	Il10
723	Mapkapk2	1.729	0.863	1.493	0.000	0.300	0.267	9.072	9.862	9.651	17164	Mapkapk2	chr1	-	NM_0085	P17171	protein_cc	AA960234	MAP kinas	Mapkapk2
724	Dyrk3	3.159	0.568	1.795	0.040	0.129	0.266	3.939	5.598	4.783	226419	Dyrk3	chr1	-	NM_1455	P25921	protein_cc	BC006704	dual-speci	Dyrk3
725	Eif2d	1.137	1.176	1.338	0.198	0.515	0.540	7.192	7.378	7.612	16865	Eif2d	chr1	+	NM_0107	P3369	protein_cc	D1Ertd5e|	eukaryotic	Eif2d
726	Rassf5	0.979	0.863	0.844	0.917	0.466	0.655	7.217	7.186	6.973	54354	Rassf5	chr1	-	NM_0187	P14762	protein_cc	1300019G	Ras associ	Rassf5
727	Ikbke	4.404	0.498	2.192	0.000	0.000	0.033	6.560	8.699	7.693	56489	Ikbke	chr1	-	NM_0197	P25173	protein_cc	AW55820	inhibitor o	Ikbke
728	Srgap2	0.603	0.676	0.408	0.009	0.297	0.122	6.321	5.591	5.027	14270	Srgap2	chr1	-	NM_0010	P25530	protein_cc	9930124L	SLIT-ROBO	Srgap2
729	Fam72a	1.386	0.507	0.702	0.529	0.225	0.602	3.526	3.997	3.016	108900	Fam72a	chr1	+	NM_1753	P15116	protein_cc	2700049P	family wit	Fam72a
730	Avpr1b	1.000	1.000	1.000	1.000	1.000	1.000	0.000	0.000	0.000	26361	Avpr1b	chr1	+	NM_0119	P5538	protein_cc	AVPR3| V3	arginine va	Avpr1b
731	Ctse	0.849	1.572	1.335	0.775	0.316	0.778	2.259	2.023	2.675	13034	Ctse	chr1	+	NM_0077	P15906	protein_cc	A430072C	cathepsin	Ctse
732	Rab7b	0.181	2.164	0.392	0.004	0.004	0.111	4.894	2.428	3.542	226421	Rab7b	chr1	+	NM_1455	P19862	protein_cc	5430435G	RAB7B, m	Rab7b
733	Slc26a9	3.908	0.256	0.999	0.003	1.000	0.423	0.001	1.967	0.000	320718	Slc26a9	chr1	+	NM_1772	P13886	protein_cc	E030002L	solute car	Slc26a9
734	Pm20d1	0.364	0.827	0.301	0.000	0.253	0.003	9.283	7.824	7.549	212933	Pm20d1	chr1	+	NM_1780	P16669	protein_cc	4732466D	peptidase	Pm20d1
735	Slc41a1	6.743	0.799	5.385	0.002	0.554	0.303	4.454	7.207	6.883	98396	Slc41a1	chr1	+	NM_1738	P1874	protein_cc	AI573938|	solute car	Slc41a1
736	Rab29	0.878	1.259	1.105	0.135	0.001	0.451	7.602	7.415	7.747	226422	Rab29	chr1	+	NM_1448	P14152	protein_cc	Rab7l1	RAB29, m	Rab29
737	Nucks1	1.183	1.414	1.674	0.193	0.013	0.012	11.486	11.729	12.229	98415	Nucks1	chr1	+	NM_0011	P21937	protein_cc	2700010L	nuclear ca	Nucks1
738	Slc45a3	1.223	0.447	0.547	0.070	0.002	0.170	7.511	7.802	6.640	212980	Slc45a3	chr1	+	NM_0011	P9322	protein_cc	2210413P	solute car	Slc45a3
739	Elk4	1.761	0.621	1.094	0.464	0.518	0.448	3.424	4.241	3.553	13714	Elk4	chr1	+	NM_0079	P16663	protein_cc	2310011G	ELK4, mem	Elk4
740	Mfsd4	0.838	2.618	2.194	0.700	0.030	0.043	1.431	1.175	2.564	.	Mfsd4	chr1	-	NM_1725	P25899	.	.	.	Mfsd4
741	Cdk18	0.330	1.625	0.536	0.000	0.030	0.046	7.190	5.591	6.291	18557	Cdk18	chr1	-	NM_0087	P16300	protein_cc	AA682070	cyclin-dep	Cdk18
742	Lemd1	1.000	1.000	1.000	1.000	1.000	1.000	0.000	0.000	0.000	213409	Lemd1	chr1	+	NM_0010	P14767	protein_cc	4930540I2	LEM doma	Lemd1
743	Klhdc8a	0.569	0.947	0.539	0.325	0.941	0.088	4.156	3.343	3.264	213417	Klhdc8a	chr1	+	NM_1448	P14431	protein_cc	A630065K	kelch dom	Klhdc8a
744	Nuak2	0.905	0.727	0.658	0.214	0.303	0.438	6.112	5.968	5.508	74137	Nuak2	chr1	+	NM_0287	P15199	protein_cc	1200013B	NUAK fam	Nuak2
745	Tmcc2	0.604	1.276	0.770	0.108	0.066	0.527	4.988	4.260	4.611	68875	Tmcc2	chr1	-	NM_1788	P5235	protein_cc	1110063G	transmem	Tmcc2
746	Dstyk	1.397	0.619	0.866	0.013	0.005	0.030	6.529	7.011	6.320	213452	Dstyk	chr1	+	NM_1725	P25969	protein_cc	A930019K	dual serin	Dstyk
747	Rbbp5	1.412	0.644	0.910	0.004	0.002	0.044	7.574	8.072	7.438	213464	Rbbp5	chr1	+	NM_1725	P8133	protein_cc	4933411J2	retinoblas	Rbbp5
748	Tmem81	1.179	1.227	1.447	0.857	0.773	0.683	2.127	2.364	2.660	74626	Tmem81	chr1	+	NM_0290	P16107	protein_cc	4930429O	transmem	Tmem81
749	Cntn2	0.318	2.636	0.837	0.017	0.210	0.773	3.194	1.539	2.938	21367	Cntn2	chr1	-	NM_1771	P23251	protein_cc	D130012K	contactin	Cntn2
750	Nfasc	0.085	2.206	0.188	0.003	0.190	0.069	5.398	1.842	2.983	269116	Nfasc	chr1	-	NM_0011	P21244	protein_cc	AA387016	neurofasc	Nfasc
751	Gm10538	1.000	1.000	1.000	1.000	1.000	1.000	0.000	0.000	0.000	10003865	Gm10538	chr1	-	NR_04589	.	lncRNA	ENSMUSG	predicted	Gm10538
752	Lrrn2	1.005	1.405	1.412	0.000	0.182	0.423	0.000	0.008	0.498	16980	Lrrn2	chr1	+	NM_0107	P12476	protein_cc	5730406J0	leucine ric	Lrrn2
753	Mdm4	1.427	0.692	0.987	0.001	0.161	0.979	7.137	7.649	7.118	17248	Mdm4	chr1	-	NM_0013	P22741	protein_cc	4933417N	transform	Mdm4
754	Pik3c2b	0.330	1.453	0.480	0.000	0.002	0.000	5.446	3.847	4.386	240752	Pik3c2b	chr1	+	NM_0010	P11573	protein_cc	C330011J1	phosphoin	Pik3c2b
755	Ppp1r15b	0.957	0.989	0.946	0.765	0.953	0.771	9.633	9.570	9.554	108954	Ppp1r15b	chr1	+	NM_1338	P2125	protein_cc	1810033K	protein ph	Ppp1r15b
756	Plekha6	0.330	0.830	0.274	0.002	0.547	0.006	8.251	6.650	6.380	240753	Plekha6	chr1	+	NM_1829	P5014	protein_cc	9530028L	pleckstrin	Plekha6
757	Gm19461	0.246	0.970	0.239	0.003	1.000	0.061	2.067	0.044	0.000	10050293	Gm19461	chr1	-	NR_03798	.	lncRNA	.	predicted	Gm19461
758	Golt1a	0.594	1.831	1.088	0.018	0.002	0.634	8.008	7.258	8.130	68338	Golt1a	chr1	+	NM_0266	P9892	protein_cc	0610012C	golgi trans	Golt1a
759	Kiss1	0.524	1.473	0.772	0.203	0.488	0.335	2.704	1.773	2.332	280287	Kiss1	chr1	+	NM_1782	P7259	protein_cc	kisspeptin	KiSS-1 me	Kiss1
760	Ren2	1.000	1.000	1.000	1.000	1.000	1.000	0.000	0.000	0.000	19702	Ren2	chr1	+	NM_0311	P13393	protein_cc	Ren| Ren-	renin 2 ta	Ren2
761	Ren1	2.144	0.641	1.374	0.303	0.482	0.423	0.000	1.101	0.458	19701	Ren1	chr1	+	NM_0311	P166	protein_cc	D19352| F	renin 1 str	Ren1
762	Etnk2	0.266	0.970	0.258	0.000	0.805	0.006	11.244	9.336	9.292	214253	Etnk2	chr1	+	NM_1754	P4910	protein_cc	4933417N	ethanolam	Etnk2
763	Sox13	0.808	1.173	0.949	0.326	0.605	0.909	6.201	5.894	6.125	20668	Sox13	chr1	-	NM_0114	P2665	protein_cc	AA407916	SRY (sex d	Sox13
764	Snrpe	1.307	1.021	1.335	0.013	0.681	0.083	9.303	9.689	9.720	20643	Snrpe	chr1	-	NM_0092	P3220	protein_cc	AL022645	small nucl	Snrpe
765	Zc3h11a	1.495	0.621	0.928	0.006	0.004	0.485	9.777	10.358	9.670	70579	Zc3h11a	chr1	-	NM_1445	P19509	protein_cc	1110003F	zinc finger	Zc3h11a
766	Zbed6	1.402	0.696	0.976	0.064	0.037	0.891	8.215	8.703	8.181	667118	Zbed6	chr1	-	NM_0011	P12350	protein_cc	667118| G	zinc finger	Zbed6
767	Lax1	0.810	0.408	0.331	0.511	0.001	0.267	2.074	1.770	0.477	240754	Lax1	chr1	-	NM_0011	P4995	protein_cc	A530029E	lymphocyt	Lax1
768	Atp2b4	0.482	1.528	0.737	0.011	0.126	0.445	6.489	5.437	6.049	381290	Atp2b4	chr1	-	NM_2136	P11553	protein_cc	Pmca4	ATPase, C	Atp2b4
769	Optc	0.299	1.720	0.515	0.182	0.193	0.582	1.787	0.047	0.830	269120	Optc	chr1	-	NM_0011	P9169	protein_cc	.	opticin	Optc
770	Prelp	0.466	0.610	0.284	0.004	0.070	0.059	8.042	6.942	6.228	116847	Prelp	chr1	-	NM_0540	P5375	protein_cc	7330409J1	proline ar	Prelp
771	Fmod	0.563	0.167	0.094	0.018	0.015	0.002	4.248	3.418	0.834	14264	Fmod	chr1	+	NM_0213	P3688	protein_cc	AI131919|	fibromodu	Fmod
772	Btg2	5.955	0.347	2.069	0.000	0.000	0.192	8.114	10.688	9.163	12227	Btg2	chr1	-	NM_0075	P18282	protein_cc	AA959598	B cell tran	Btg2
773	Chit1	0.716	0.995	0.712	0.182	1.000	0.423	0.490	0.008	0.000	71884	Chit1	chr1	+	NM_0279	P24043	protein_cc	2300002L	chitinase	Chit1
774	Chil1	85.399	0.339	28.959	0.002	0.011	0.064	1.234	7.650	6.089	12654	Chil1	chr1	+	NM_0076	P18108	protein_cc	AW20876	chitinase-	Chil1
775	Mybph	1.146	0.632	0.725	0.729	1.000	0.422	0.465	0.661	0.000	53311	Mybph	chr1	+	NM_0167	P15401	protein_cc	AI385643|	myosin bin	Mybph
776	Adora1	0.142	2.371	0.336	0.000	0.284	0.123	8.987	6.169	7.414	11539	Adora1	chr1	-	NM_0012	P12362	protein_cc	A1-AR| A1	adenosine	Adora1
777	Myog	1.010	1.821	1.840	0.000	0.000	0.002	0.000	0.015	0.880	17928	Myog	chr1	+	NM_0311	P13765	protein_cc	bHLHc3| M	myogenin	Myog
778	Ppfia4	0.178	2.132	0.380	0.001	0.127	0.051	3.683	1.194	2.286	68507	Ppfia4	chr1	-	NM_0011	P980	protein_cc	10004238	protein ty	Ppfia4
779	Tmem183	0.532	1.396	0.742	0.000	0.006	0.062	10.353	9.441	9.922	57439	Tmem183	chr1	-	NM_0010	P23417	protein_cc	1300007B	transmem	Tmem183a
780	4933406M	1.000	1.000	1.000	1.000	1.000	1.000	0.000	0.000	0.000	240755	4933406M	chr1	+	NM_1737	P26106	protein_cc	.	RIKEN cDN	4933406M
781	Cyb5r1	0.733	1.605	1.177	0.182	0.042	0.576	6.645	6.197	6.880	72017	Cyb5r1	chr1	+	NM_0280	P26650	protein_cc	1500005G	cytochrom	Cyb5r1
782	Adipor1	0.584	1.303	0.761	0.017	0.092	0.169	9.014	8.237	8.619	72674	Adipor1	chr1	+	NM_0283	P6466	protein_cc	2810031L	adiponect	Adipor1
783	Klhl12	1.093	0.969	1.059	0.611	0.681	0.840	6.850	6.978	6.932	240756	Klhl12	chr1	+	NM_1531	P24280	protein_cc	C3ip1	kelch-like	Klhl12
784	Rabif	2.337	0.554	1.294	0.000	0.005	0.413	6.407	7.632	6.779	98710	Rabif	chr1	+	NM_1455	P22995	protein_cc	AI842864|	RAB intera	Rabif
785	Mgat4e	1.000	1.000	1.000	1.000	1.000	1.000	0.000	0.000	0.000	71001	Mgat4e	chr1	-	NM_0012	P20765	protein_cc	4931440L	MGAT4 fa	Mgat4e
786	Kdm5b	1.709	0.868	1.483	0.005	0.146	0.171	7.317	8.090	7.885	75605	Kdm5b	chr1	+	NM_1528	P10890	protein_cc	2010009J1	lysine (K)-	Kdm5b
787	Syt2	1.059	0.629	0.666	0.888	1.000	0.423	0.587	0.669	0.000	20980	Syt2	chr1	+	NM_0093	P21020	protein_cc	IP4BP| mk	synaptota	Syt2
788	Ppp1r12b	1.071	0.407	0.436	0.856	0.055	0.449	2.711	2.810	1.514	329251	Ppp1r12b	chr1	-	NM_0010	P12314	protein_cc	1810037O	protein ph	Ppp1r12b
789	Ube2t	1.009	1.157	1.167	0.929	0.806	0.880	2.379	2.391	2.602	67196	Ube2t	chr1	+	NM_0012	P26318	protein_cc	2700084L	ubiquitin-	Ube2t
790	Lgr6	1.000	1.000	1.000	1.000	1.000	1.000	0.000	0.000	0.000	329252	Lgr6	chr1	-	NM_0010	P10625	protein_cc	A530037C	leucine-ric	Lgr6
791	Ptprv	1.000	1.000	1.000	1.000	1.000	1.000	0.000	0.000	0.000	13924	Ptprv	chr1	-	NM_0079	P5168	protein_cc	Esp| mOS	protein ty	Ptprv
792	Ptpn7	0.981	1.118	1.097	0.939	0.823	0.903	2.714	2.687	2.848	320139	Ptpn7	chr1	+	NM_1770	P15079	protein_cc	BPTP-4| C	protein ty	Ptpn7
793	Arl8a	2.408	0.433	1.042	0.000	0.000	0.825	6.205	7.473	6.264	68724	Arl8a	chr1	+	NM_0268	P19483	protein_cc	1110033P	ADP-ribos	Arl8a
794	Gpr37l1	1.581	0.906	1.432	0.301	0.802	0.423	0.000	0.661	0.519	171469	Gpr37l1	chr1	-	NM_1344	P5457	protein_cc	AW04723	G protein-	Gpr37l1
795	Elf3	12.370	0.676	8.359	0.018	0.255	0.031	2.473	6.101	5.536	13710	Elf3	chr1	-	NM_0011	P17117	protein_cc	ESE-1| ES	E74-like fa	Elf3
796	Rnpep	1.288	0.705	0.908	0.022	0.091	0.782	8.480	8.845	8.341	215615	Rnpep	chr1	-	NM_0011	P13283	protein_cc	.	arginyl am	Rnpep
797	Timm17a	1.074	1.246	1.338	0.000	0.000	0.001	8.486	8.588	8.906	21854	Timm17a	chr1	-	NM_0115	P9744	protein_cc	17kDa| m	translocas	Timm17a
798	Lmod1	0.070	3.817	0.267	0.010	0.004	0.151	5.062	1.224	3.156	93689	Lmod1	chr1	+	NM_0531	P15855	protein_cc	9530015K	leiomodin	Lmod1
799	Shisa4	2.562	1.004	2.573	0.366	0.995	0.119	0.396	1.753	1.760	77552	Shisa4	chr1	-	NM_1752	P20219	protein_cc	9330132O	shisa fami	Shisa4
800	Ipo9	0.878	0.869	0.763	0.126	0.131	0.189	7.207	7.018	6.817	226432	Ipo9	chr1	-	NM_1537	P10039	protein_cc	0710008K	importin 9	Ipo9
801	Nav1	0.479	0.974	0.466	0.006	0.896	0.110	6.458	5.395	5.357	215690	Nav1	chr1	-	NM_1734	P9002	protein_cc	9530089B	neuron na	Nav1
802	Csrp1	1.639	0.740	1.213	0.071	0.267	0.608	6.442	7.155	6.720	13007	Csrp1	chr1	+	NM_0077	P13	protein_cc	AA408841	cysteine a	Csrp1
803	Phlda3	26.748	0.550	14.708	0.000	0.019	0.061	2.348	7.089	6.227	27280	Phlda3	chr1	+	NM_0137	P15049	protein_cc	Tih1	pleckstrin	Phlda3
804	Tnni1	1.000	1.000	1.000	1.000	1.000	1.000	0.000	0.000	0.000	21952	Tnni1	chr1	+	NM_0214	P5913	protein_cc	2700018B	troponin I	Tnni1
805	Lad1	0.119	3.835	0.457	0.009	0.001	0.214	3.841	0.773	2.712	16763	Lad1	chr1	+	NM_1336	P7774	protein_cc	.	ladinin	Lad1
806	Tnnt2	0.167	3.248	0.543	0.005	0.016	0.146	4.118	1.538	3.237	21956	Tnnt2	chr1	+	NM_0116	P21890	protein_cc	cTnT| Tnt	troponin T	Tnnt2

807	Pkp1	1.000	1.000	1.000	1.000	1.000	1.000	0.000	0.000	0.000	18772	Pkp1	chr1	-	NM_0196	P12703	protein_cc	.	plakophilii	Pkp1
808	Igfn1	0.716	0.995	0.712	0.182	1.000	0.423	0.490	0.008	0.000	226438	Igfn1	chr1	-	NM_1776	P8912	protein_cc	9830123N	immunogl	Igfn1
809	Tmem9	0.233	3.014	0.703	0.001	0.001	0.229	7.745	5.644	7.236	66241	Tmem9	chr1	+	NM_0011	P17921	protein_cc	1500015G	transmem	Tmem9
810	Ascl5	1.000	1.000	1.000	1.000	1.000	1.000	0.000	0.000	0.000	226439	Ascl5	chr1	+	NM_0012	P1522	protein_cc	Ascl5-ps|	achaete-s	Ascl5
811	Cacna1s	1.428	0.466	0.666	0.562	1.000	0.423	0.587	1.101	0.000	12292	Cacna1s	chr1	+	NM_0010	P1208	protein_cc	AW49310	calcium ch	Cacna1s
812	Gm15850	1.000	1.000	1.000	1.000	1.000	1.000	0.000	0.000	0.000	329253	Gm15850	chr1	-	NR_04616	.	lncRNA	6530439I2	predicted	Gm15850
813	Kif21b	1.455	1.122	1.633	0.584	0.854	0.568	2.958	3.499	3.665	16565	Kif21b	chr1	+	NM_0010	P5759	protein_cc	2610511N	kinesin fa	Kif21b
814	5730559C	1.015	2.326	2.360	0.000	0.182	0.423	0.000	0.021	1.239	67313	5730559C	chr1	-	NM_0288	P10120	protein_cc	1700034N	RIKEN cDN	5730559C
815	Gpr25	1.580	0.633	1.000	0.305	1.000	0.423	0.000	0.660	0.000	383563	Gpr25	chr1	-	NM_0011	P22896	protein_cc	Gm1300	G protein-	Gpr25
816	Camsap2	1.445	0.754	1.090	0.001	0.047	0.694	7.349	7.881	7.473	67886	Camsap2	chr1	-	NM_0010	P22517	protein_cc	1600013L	calmoduli	Camsap2
817	9230116N	2.170	1.230	2.669	0.299	0.685	0.223	0.001	1.119	1.417	320054	9230116N	chr1	-	NR_02432	.	lncRNA	.	RIKEN cDN	9230116N
818	Ddx59	1.021	0.853	0.871	0.928	0.577	0.641	5.070	5.100	4.870	67997	Ddx59	chr1	+	NM_0265	P9157	protein_cc	1210002B	DEAD (Asp	Ddx59
819	Kif14	0.787	1.903	1.497	0.653	0.417	0.723	1.496	1.150	2.078	381293	Kif14	chr1	+	NM_0012	P22283	protein_cc	D1Ertd367	kinesin fa	Kif14
820	Zfp281	3.447	0.369	1.271	0.001	0.001	0.468	9.259	11.045	9.606	226442	Zfp281	chr1	+	NM_1776	P3222	protein_cc	Znf281	zinc finger	Zfp281
821	Gm19705	102.830	0.533	54.853	0.000	0.000	0.001	2.450	9.134	8.228	10050346	Gm19705	chr1	+	NR_04532	.	lncRNA	.	predicted	Gm19705
822	Platr22	9.167	0.229	2.099	0.003	0.005	0.069	1.092	4.288	2.161	10050347	Platr22	chr1	+	NR_03798	.	lncRNA	Gm16880	pluripoter	Platr22
823	Nr5a2	0.559	0.676	0.378	0.000	0.011	0.008	9.626	8.788	8.223	26424	Nr5a2	chr1	-	NM_0306	P6974	protein_cc	AU020803	nuclear re	Nr5a2
824	Ptprc	3.189	0.544	1.736	0.012	0.054	0.258	6.015	7.689	6.811	19264	Ptprc	chr1	-	NM_0112	P20570	protein_cc	B220| Cd4	protein ty	Ptprc
825	Atp6v1g3	1.000	1.000	1.000	1.000	1.000	1.000	0.000	0.000	0.000	338375	Atp6v1g3	chr1	+	NM_1773	P24842	protein_cc	.	ATPase, H	Atp6v1g3
826	Nek7	2.270	0.724	1.643	0.006	0.062	0.008	7.757	8.940	8.473	59125	Nek7	chr1	-	NM_0216	P20047	protein_cc	2810460C	NIMA (nev	Nek7
827	Gm5833	1.000	1.000	1.000	1.000	1.000	1.000	0.000	0.000	0.000	545363	Gm5833	chr1	-	NR_04030	.	lncRNA	545363	predicted	Gm5833
828	Lhx9	1.000	1.000	1.000	1.000	1.000	1.000	0.000	0.000	0.000	16876	Lhx9	chr1	-	NM_0107	P10541	protein_cc	31100090	LIM home	Lhx9
829	2310009B	1.082	1.041	1.126	0.767	0.873	0.229	6.766	6.880	6.938	69549	2310009B	chr1	-	NM_0010	P21274	protein_cc	.	RIKEN cDN	2310009B
830	Dennd1b	0.889	0.843	0.750	0.443	0.352	0.132	7.263	7.094	6.847	329260	Dennd1b	chr1	+	NM_0011	P23516	protein_cc	4632404N	DENN/MA	Dennd1b
831	4930596I2	3.536	1.562	5.524	0.020	0.038	0.011	0.726	2.548	3.191	78229	4930596I2	chr1	+	NR_10810	.	lncRNA	.	RIKEN cDN	4930596I2
832	Crb1	1.000	1.000	1.000	1.000	1.000	1.000	0.000	0.000	0.000	170788	Crb1	chr1	-	NM_1332	P233	protein_cc	7530426H	crumbs fa	Crb1
833	4933436E	1.000	1.000	1.000	1.000	1.000	1.000	0.000	0.000	0.000	71209	4933436E	chr1	+	NR_04045	.	lncRNA	.	RIKEN cDN	4933436E
834	Zbtb41	1.734	0.685	1.187	0.003	0.009	0.443	6.816	7.610	7.064	226470	Zbtb41	chr1	+	NM_1726	P4079	protein_cc	8430415N	zinc finger	Zbtb41
835	Aspm	2.123	0.349	0.742	0.369	0.273	0.570	3.283	4.369	2.852	12316	Aspm	chr1	+	NM_0097	P11244	protein_cc	Calmbp1|	asp (abno	Aspm
836	F13b	0.272	1.010	0.275	0.002	0.970	0.037	11.327	9.451	9.466	14060	F13b	chr1	+	NM_0311	P2011	protein_cc	Cf-13b| Cf	coagulatic	F13b
837	Cfhr1	0.363	0.858	0.312	0.000	0.075	0.016	9.437	7.976	7.755	50702	Cfhr1	chr1	-	NM_0157	P23244	protein_cc	AI194696|	compleme	Cfhr1
838	Gm4788	0.504	0.749	0.378	0.003	0.001	0.036	9.770	8.782	8.366	214403	Gm4788	chr1	-	NM_0011	P14383	protein_cc	Cfhrc| EG	predicted	Gm4788
839	Cfhr2	0.435	1.329	0.579	0.005	0.066	0.142	10.972	9.772	10.183	545366	Cfhr2	chr1	-	NM_0010	P23913	protein_cc	Cfhrb_4/2	compleme	Cfhr2
840	Cfh	1.111	1.126	1.251	0.277	0.082	0.168	11.305	11.456	11.627	12628	Cfh	chr1	-	NM_0098	P18392	protein_cc	Mud-1| N	compleme	Cfh
841	Kcnt2	4.059	2.608	10.584	0.331	0.107	0.060	0.354	2.375	3.757	240776	Kcnt2	chr1	+	NM_0010	P6918	protein_cc	E330038N	potassium	Kcnt2
842	4930590L	1.000	1.000	1.000	1.000	1.000	1.000	0.000	0.000	0.000	75910	4930590L	chr1	-	NR_04060	.	lncRNA	.	RIKEN cDN	4930590L
843	Cdc73	2.631	0.713	1.876	0.002	0.107	0.204	5.963	7.358	6.870	214498	Cdc73	chr1	-	NM_1459	P25009	protein_cc	8430414L	cell divisio	Cdc73
844	B3galt2	1.582	0.511	0.808	0.550	0.422	0.731	0.801	1.463	0.493	26878	B3galt2	chr1	+	NM_0200	P2894	protein_cc	.	UDP-Gal:b	B3galt2
845	Glrx2	0.629	1.348	0.848	0.009	0.061	0.357	8.673	8.004	8.435	69367	Glrx2	chr1	+	NM_0010	P16387	protein_cc	1700010P	glutaredo	Glrx2
846	Trove2	1.265	1.316	1.665	0.438	0.158	0.185	6.209	6.548	6.944	20822	Trove2	chr1	-	NM_0138	P7127	protein_cc	1810007I1	TROVE do	Trove2
847	Uchl5	0.652	0.923	0.601	0.038	0.683	0.003	7.999	7.381	7.264	56207	Uchl5	chr1	+	NM_0195	P23547	protein_cc	5830413B	ubiquitin	Uchl5
848	Rgs2	6.112	1.170	7.153	0.000	0.575	0.150	7.341	9.953	10.180	19735	Rgs2	chr1	-	NM_0090	P8991	protein_cc	GOS8	regulator	Rgs2
849	Rgs13	0.552	0.990	0.547	0.182	1.000	0.423	0.871	0.015	0.000	246709	Rgs13	chr1	-	NM_1531	P10395	protein_cc	.	regulator	Rgs13
850	Rgs1	3.702	0.331	1.226	0.004	0.006	0.751	5.318	7.206	5.612	50778	Rgs1	chr1	-	NM_0158	P4727	protein_cc	BL34	regulator	Rgs1
851	Rgs21	1.000	1.000	1.000	1.000	1.000	1.000	0.000	0.000	0.000	624910	Rgs21	chr1	-	NR_11089	.	protein_cc	EG624910	regulator	Rgs21
852	Rgs18	1.987	0.331	0.657	0.169	0.086	0.205	2.143	3.133	1.537	64214	Rgs18	chr1	-	NM_0228	P13362	protein_cc	.	regulator	Rgs18
853	Brinp3	0.560	1.371	0.768	0.184	0.191	0.687	0.857	0.021	0.477	215378	Brinp3	chr1	+	NM_1535	P24497	protein_cc	B830045N	bone mor	Brinp3
854	Pla2g4a	2.963	0.391	1.159	0.004	0.000	0.808	3.210	4.777	3.423	18783	Pla2g4a	chr1	-	NM_0013	P21901	protein_cc	cPLA2| cP	phospholi	Pla2g4a
855	Ptgs2os	1.000	1.000	1.000	1.000	1.000	1.000	0.000	0.000	0.000	320019	Ptgs2os	chr1	-	NR_01546	.	lncRNA	7530420F	prostaglar	Ptgs2os
856	Ptgs2	46.004	0.035	1.630	0.016	0.017	0.016	0.003	5.526	0.707	19225	Ptgs2	chr1	+	NM_0111	P3909	protein_cc	Cox-2| CO	prostaglar	Ptgs2
857	Ptgs2os2	7.458	0.185	1.379	0.000	0.000	0.424	0.001	2.900	0.466	10263956	Ptgs2os2	chr1	-	NR_11042	.	lncRNA	Gm26687	prostaglar	Ptgs2os2
858	Pdc	0.716	0.995	0.712	0.182	1.000	0.423	0.490	0.008	0.000	20028	Pdc	chr1	+	NM_0244	P19372	protein_cc	Rpr-1| Rp	phosducin	Pdc
859	2310030A	1.000	1.000	1.000	1.000	1.000	1.000	0.000	0.000	0.000	75584	2310030A	chr1	-	NR_04060	.	lncRNA	.	RIKEN cDN	2310030A
860	BC003331	0.783	1.021	0.800	0.266	0.923	0.207	9.220	8.867	8.897	226499	BC003331	chr1	-	NM_0010	P19273	protein_cc	1810053E	cDNA seq	BC003331
861	Tpr	1.388	0.845	1.172	0.018	0.072	0.373	10.240	10.713	10.469	108989	Tpr	chr1	+	NM_1337	P24693	protein_cc	2610029N	translocat	Tpr
862	Prg4	3.042	0.942	2.867	0.000	0.209	0.018	8.550	10.156	10.070	96875	Prg4	chr1	-	NM_0011	P25230	protein_cc	CACP| DO	proteogly	Prg4
863	Hmcn1	0.463	1.652	0.765	0.127	0.560	0.747	2.907	1.797	2.521	545370	Hmcn1	chr1	-	NM_0010	P23167	protein_cc	EG545370	hemicenti	Hmcn1
864	C730036E	0.267	1.224	0.327	0.000	0.348	0.003	5.696	3.791	4.082	402734	C730036E	chr1	+	NR_03801	.	lncRNA	lncLSTR	RIKEN cDN	C730036E
865	Ivns1abp	0.574	1.039	0.597	0.000	0.716	0.032	9.525	8.725	8.781	117198	Ivns1abp	chr1	+	NM_0541	P11342	protein_cc	1190004N	influenza	Ivns1abp
866	Swt1	1.833	0.564	1.034	0.017	0.013	0.860	8.335	9.209	8.384	66875	Swt1	chr1	-	NM_0258	P16880	protein_cc	1200016B	SWT1 RNA	Swt1
867	Trmt1l	0.999	0.601	0.600	0.996	0.168	0.203	7.056	7.054	6.320	98685	Trmt1l	chr1	+	NM_0268	P19255	protein_cc	1190005F	tRNA met	Trmt1l
868	Rnf2	1.487	0.875	1.301	0.000	0.413	0.457	7.701	8.273	8.080	19821	Rnf2	chr1	-	NM_0112	P17199	protein_cc	AI326319|	ring finger	Rnf2
869	Fam129a	0.452	0.881	0.398	0.118	0.839	0.097	3.831	2.686	2.503	63913	Fam129a	chr1	+	NM_0220	P6644	protein_cc	AI256368|	family wit	Fam129a
870	Edem3	1.447	0.935	1.354	0.029	0.547	0.135	7.871	8.404	8.308	66967	Edem3	chr1	+	NM_0010	P5550	protein_cc	2310050N	ER degrad	Edem3
871	1700025G	0.552	1.568	0.865	0.002	0.005	0.064	5.645	4.787	5.436	69399	1700025G	chr1	-	NM_1979	P6862	protein_cc	2610510E	RIKEN cDN	1700025G
872	Tsen15	1.882	0.818	1.539	0.022	0.339	0.287	5.936	6.848	6.558	66637	Tsen15	chr1	-	NM_0256	P3566	protein_cc	5730449L	tRNA splic	Tsen15
873	Colgalt2	0.136	2.545	0.345	0.004	0.191	0.176	2.971	0.089	1.437	269132	Colgalt2	chr1	+	NM_1777	P19236	protein_cc	AI427652|	collagen b	Colgalt2
874	Rgl1	0.691	0.708	0.490	0.077	0.119	0.125	6.305	5.773	5.275	19731	Rgl1	chr1	-	NM_0168	P880	protein_cc	mKIAA095	ral guanin	Rgl1
875	Apobec4	1.000	1.000	1.000	1.000	1.000	1.000	0.000	0.000	0.000	71281	Apobec4	chr1	+	NM_0010	P20508	protein_cc	4933431N	apolipopro	Apobec4
876	Arpc5	1.509	0.887	1.338	0.007	0.300	0.322	9.611	10.204	10.031	67771	Arpc5	chr1	+	NM_0263	P25706	protein_cc	5830443F	actin relat	Arpc5
877	Ncf2	1.895	0.953	1.806	0.052	0.876	0.363	4.554	5.476	5.407	17970	Ncf2	chr1	+	NM_0108	P12329	protein_cc	Ncf-2| NO	neutrophi	Ncf2
878	Smg7	1.768	1.109	1.961	0.013	0.520	0.121	9.024	9.847	9.996	226517	Smg7	chr1	-	NM_0011	P15488	protein_cc	9430023P	Smg-7 hor	Smg7
879	Nmnat2	0.908	3.591	3.259	0.765	0.050	0.227	0.863	0.723	2.567	226518	Nmnat2	chr1	+	NM_1754	P24649	protein_cc	AI843915|	nicotinam	Nmnat2
880	Lamc2	1.832	0.829	1.520	0.124	0.632	0.695	1.504	2.378	2.108	16782	Lamc2	chr1	-	NM_0084	P4422	protein_cc	AA589349	laminin, g	Lamc2
881	Lamc1	1.200	0.771	0.925	0.306	0.182	0.018	6.803	7.066	6.691	226519	Lamc1	chr1	-	NM_0106	P7883	protein_cc	Lamb2	laminin, g	Lamc1
882	E330020D	0.552	0.990	0.547	0.182	1.000	0.423	0.871	0.015	0.000	626058	E330020D	chr1	-	NR_03373	.	lncRNA	EG626058	Riken cDN	E330020D
883	Shcbp1l	2.870	3.444	9.885	0.053	0.005	0.038	0.717	2.238	4.022	71836	Shcbp1l	chr1	+	NM_0010	P20929	protein_cc	1700012A	Shc SH2-d	Shcbp1l
884	Dhx9	1.827	0.824	1.505	0.077	0.447	0.208	8.116	8.985	8.706	13211	Dhx9	chr1	-	NM_0078	P20490	protein_cc	AI326842|	DEAH (Asp	Dhx9
885	Npl	0.373	0.981	0.366	0.018	1.000	0.140	1.450	0.027	0.000	74091	Npl	chr1	-	NM_0287	P5020	protein_cc	0610033B	N-acetylne	Npl
886	Rgs8	1.010	1.885	1.904	0.000	0.182	0.423	0.000	0.015	0.929	67792	Rgs8	chr1	+	NM_0263	P24450	protein_cc	6530413N	regulator	Rgs8
887	C230024C	0.716	0.995	0.712	0.182	1.000	0.423	0.490	0.008	0.000	329271	C230024C	chr1	-	NR_04617	.	lncRNA	C230024C	RIKEN cDN	C230024C
888	Rgs16	0.060	1.642	0.098	0.000	0.522	0.026	10.233	6.167	6.882	19734	Rgs16	chr1	+	NM_0112	P23048	protein_cc	Rgs14| Rg	regulator	Rgs16
889	Rnasel	3.336	0.349	1.164	0.014	0.018	0.374	4.066	5.804	4.286	24014	Rnasel	chr1	+	NM_0118	P14736	protein_cc	E230029I0	ribonuclea	Rnasel
890	Rgsl1	1.014	2.240	2.272	0.000	0.182	0.423	0.000	0.021	1.185	240816	Rgsl1	chr1	-	NM_0012	P3667	protein_cc	4930415K	regulator	Rgsl1
891	Teddm2	1.589	1.141	1.813	0.298	0.765	0.423	0.000	0.669	0.859	240817	Teddm2	chr1	-	NM_1782	P855	protein_cc	5830403L	transmem	Teddm2
892	Teddm1b	1.099	3.121	3.431	0.819	0.006	0.050	0.588	0.724	2.366	433365	Teddm1b	chr1	-	NM_0010	P14193	protein_cc	EG433365	transmem	Teddm1b
893	Teddm1a	1.000	1.000	1.000	1.000	1.000	1.000	0.000	0.000	0.000	240819	Teddm1a	chr1	+	NM_1782	P13242	protein_cc	e9-1| Epd	transmem	Teddm1a
894	Glul	0.505	1.115	0.563	0.002	0.769	0.273	13.750	12.764	12.921	14645	Glul	chr1	-	NM_0081	P6555	protein_cc	Glns| GS	glutamate	Glul
895	Zfp648	1.000	1.000	1.000	1.000	1.000	1.000	0.000	0.000	0.000	10050335	Zfp648	chr1	+	NM_0012	P18249	protein_cc	ENSMUSG	zinc finger	Zfp648
896	4930452N	1.000	1.000	1.000	1.000	1.000	1.000	0.000	0.000	0.000	78112	4930452N	chr1	-	NR_04062	.	lncRNA	.	RIKEN cDN	4930452N
897	4930532M	1.000	1.000	1.000	1.000	1.000	1.000	0.000	0.000	0.000	75233	4930532M	chr1	+	NR_10805	.	lncRNA	.	RIKEN cDN	4930532M

898	Cacna1e	1.000	1.000	1.000	1.000	1.000	1.000	0.000	0.000	0.000	12290	Cacna1e	chr1	-	NM_0097	P12374	protein_cc	A430040I1	calcium ch	Cacna1e
899	Ier5	6.778	0.779	5.278	0.004	0.227	0.017	6.237	8.998	8.637	15939	Ier5	chr1	-	NM_0105	P7507	protein_cc	.	immediate	Ier5
900	Mr1	0.407	2.552	1.039	0.058	0.004	0.931	4.556	3.260	4.612	15064	Mr1	chr1	-	NM_0082	P2707	protein_cc	H2ls	major hist	Mr1
901	Stx6	2.188	0.824	1.803	0.005	0.342	0.296	5.737	6.867	6.588	58244	Stx6	chr1	+	NM_0214	P26801	protein_cc	2310039E	syntaxin 6	Stx6
902	Xpr1	0.553	1.321	0.730	0.002	0.044	0.001	8.372	7.517	7.918	19775	Xpr1	chr1	-	NM_0112	P9955	protein_cc	Rmc-1 | Rr	xenotropi	Xpr1
903	Gm5532	1.615	0.205	0.331	0.043	0.001	0.181	2.439	3.130	0.845	433367	Gm5532	chr1	-	NR_13212	.	lncRNA	EG433367	predicted	Gm5532
904	Acbd6	0.869	1.259	1.094	0.121	0.086	0.665	7.949	7.746	8.078	72482	Acbd6	chr1	+	NM_0266	P14150	protein_cc	0610010G	acyl-Coen	Acbd6
905	Lhx4	1.000	1.000	1.000	1.000	1.000	1.000	0.000	0.000	0.000	16872	Lhx4	chr1	-	NM_0107	P2827	protein_cc	A330062J	LIM home	Lhx4
906	Qsox1	2.158	0.926	1.997	0.000	0.007	0.009	12.546	13.655	13.544	104009	Qsox1	chr1	-	NM_0232	P7279	protein_cc	1300003H	quiescin Q	Qsox1
907	Cep350	1.441	0.693	0.999	0.002	0.016	0.997	7.861	8.388	7.860	74081	Cep350	chr1	-	NM_0010	P1968	protein_cc	4933409L	centrosom	Cep350
908	Tor1aip1	1.822	0.793	1.445	0.091	0.443	0.424	9.333	10.199	9.864	208263	Tor1aip1	chr1	-	NM_1447	P20340	protein_cc	LAP1 | Lap	torsin A in	Tor1aip1
909	Tor1aip2	1.030	0.765	0.789	0.657	0.000	0.168	8.768	8.812	8.426	240832	Tor1aip2	chr1	+	NM_1728	P6262	protein_cc	1110020D	torsin A in	Tor1aip2
910	Fam163a	1.586	0.463	0.734	0.473	1.000	0.422	0.446	1.111	0.000	329274	Fam163a	chr1	-	NM_1778	P20230	protein_cc	A230106N	family wit	Fam163a
911	Tdrd5	1.000	1.000	1.000	1.000	1.000	1.000	0.000	0.000	0.000	214575	Tdrd5	chr1	-	NM_0012	P12120	protein_cc	Gm103	tudor dom	Tdrd5
912	Nphs2	1.000	1.000	1.000	1.000	1.000	1.000	0.000	0.000	0.000	170484	Nphs2	chr1	+	NM_1304	P22332	protein_cc	PDCN | SR	nephrosis	Nphs2
913	Soat1	1.464	0.810	1.186	0.081	0.354	0.665	5.071	5.620	5.316	20652	Soat1	chr1	-	NM_0092	P16321	protein_cc	8430426K	sterol O-a	Soat1
914	Abl2	2.504	0.824	2.064	0.005	0.250	0.074	6.260	7.584	7.305	11352	Abl2	chr1	+	NM_0095	P25872	protein_cc	AA536808	v-abl Abel	Abl2
915	Tor3a	4.362	0.470	2.050	0.000	0.001	0.110	5.653	7.778	6.688	30935	Tor3a	chr1	-	NM_0231	P16294	protein_cc	Adir	torsin fam	Tor3a
916	Fam20b	1.022	1.203	1.229	0.913	0.309	0.267	7.219	7.250	7.516	215015	Fam20b	chr1	-	NM_1454	P16870	protein_cc	C530043G	family wit	Fam20b
917	Ralgps2	0.438	1.077	0.472	0.001	0.754	0.089	7.536	6.345	6.452	78255	Ralgps2	chr1	-	NM_0011	P5884	protein_cc	1810020P	Ral GEF w	Ralgps2
918	Angptl1	2.134	0.469	1.000	0.305	1.000	0.423	0.000	1.094	0.000	72713	Angptl1	chr1	+	NM_0283	P16467	protein_cc	2810039D	angiopoie	Angptl1
919	Tex35	0.552	0.990	0.547	0.182	1.000	0.423	0.871	0.015	0.000	73435	Tex35	chr1	-	NM_0013	P23359	protein_cc	1700057K	testis expr	Tex35
920	Rasal2	0.536	1.377	0.739	0.009	0.133	0.384	7.026	6.127	6.589	226525	Rasal2	chr1	-	NM_1776	P12938	protein_cc	6530401P	RAS prote	Rasal2
921	2810025M	0.244	1.644	0.401	0.000	0.097	0.035	6.514	4.479	5.196	69953	2810025M	chr1	+	NR_02798	.	lncRNA	AI429618	RIKEN cDN	2810025M
922	BC026585	0.490	2.075	1.016	0.326	0.060	0.984	1.746	0.716	1.769	226527	BC026585	chr1	+	NM_0010	P12971	protein_cc	.	cDNA seq	BC026585
923	Sec16b	0.535	0.907	0.485	0.001	0.393	0.008	8.284	7.381	7.239	89867	Sec16b	chr1	+	NM_0333	P1429	protein_cc	Lztr2 | Rgp	SEC16 hor	Sec16b
924	Brinp2	1.000	1.000	1.000	1.000	1.000	1.000	0.000	0.000	0.000	240843	Brinp2	chr1	-	NM_2075	P22929	protein_cc	6430517E	bone mor	Brinp2
925	Astn1	0.534	0.990	0.528	0.000	1.000	0.015	0.921	0.015	0.000	11899	Astn1	chr1	+	NM_0074	P14959	protein_cc	Astn | GC1	astrotacti	Astn1
926	Pappa2	1.019	2.648	2.698	0.000	0.182	0.423	0.000	0.027	1.432	23850	Pappa2	chr1	-	NM_0010	P7466	protein_cc	PAPP-A2 |	pappalysi	Pappa2
927	Rfwd2	0.939	0.968	0.909	0.416	0.719	0.385	7.507	7.416	7.369	26374	Rfwd2	chr1	+	NM_0119	P2208	protein_cc	AI316802 |	ring finger	Rfwd2
928	Scarna3a	1.146	0.632	0.725	0.729	1.000	0.422	0.465	0.661	0.000	10021741	Scarna3a	chr1	+	NR_02851	.	guide_RN	MBI-114	small Caja	Scarna3a
929	Tnr	0.552	0.990	0.547	0.182	1.000	0.423	0.871	0.015	0.000	21960	Tnr	chr1	+	NM_0223	P8033	protein_cc	J1-tenasci	tenascin R	Tnr
930	4930523C	0.422	0.564	0.238	0.038	0.002	0.135	7.098	5.854	5.028	67647	4930523C	chr1	+	NR_02811	.	protein_cc	.	RIKEN cDN	4930523C
931	4930562F	0.303	0.760	0.230	0.043	0.576	0.150	5.885	4.163	3.766	75255	4930562F	chr1	-	NR_03803	.	lncRNA	.	RIKEN cDN	4930562F
932	Tnn	1.603	1.661	2.662	0.292	0.102	0.002	0.001	0.682	1.413	329278	Tnn	chr1	-	NM_1778	P22523	protein_cc	tenascin-V	tenascin N	Tnn
933	Mrps14	0.860	1.053	0.906	0.000	0.016	0.010	8.511	8.293	8.368	64659	Mrps14	chr1	+	NM_0254	P13963	protein_cc	1810032L	mitochon	Mrps14
934	Cacybp	1.367	0.742	1.014	0.008	0.054	0.960	8.273	8.724	8.292	12301	Cacybp	chr1	-	NM_0097	P6992	protein_cc	SIP	calcyclin b	Cacybp
935	Rabgap1l	0.154	2.899	0.448	0.000	0.000	0.027	8.036	5.340	6.876	29809	Rabgap1l	chr1	-	NM_0010	P1279	protein_cc	5830411O	RAB GTPa	Rabgap1l
936	Gpr52	1.000	1.000	1.000	1.000	1.000	1.000	0.000	0.000	0.000	620246	Gpr52	chr1	-	NM_0011	P18086	protein_cc	EG620246	G protein-	Gpr52
937	Rc3h1	1.838	0.703	1.292	0.024	0.072	0.158	7.195	8.073	7.564	381305	Rc3h1	chr1	+	NM_0010	P395	protein_cc	5730557L	RING CCC	Rc3h1
938	Serpinc1	0.454	0.873	0.396	0.001	0.113	0.020	14.048	12.907	12.712	11905	Serpinc1	chr1	+	NM_0808	P24948	protein_cc	AI114908 |	serine (or	Serpinc1
939	Zbtb37	2.351	1.025	2.410	0.119	0.940	0.283	2.970	4.203	4.239	240869	Zbtb37	chr1	-	NM_1734	P20022	protein_cc	D430004I	zinc finger	Zbtb37
940	Gas5	1.191	1.294	1.541	0.289	0.203	0.289	9.625	9.878	10.249	14455	Gas5	chr1	+	NR_00284	.	lncRNA	Gas-5 | Mi	growth ar	Gas5
941	Snord47	4.870	0.446	2.174	0.227	0.392	0.492	1.016	3.300	2.136	10021744	Snord47	chr1	+	NR_02854	.	snoRNA	MBII-96	small nucl	Snord47
942	Dars2	0.658	1.499	0.987	0.013	0.069	0.958	6.719	6.116	6.701	226539	Dars2	chr1	-	NM_1726	P16220	protein_cc	5830468K	aspartyl-t	Dars2
943	Cenpl	0.630	1.232	0.776	0.001	0.057	0.189	7.035	6.368	6.668	70454	Cenpl	chr1	+	NM_0011	P15271	protein_cc	2610300B	centrome	Cenpl
944	Klhl20	1.126	0.561	0.632	0.539	0.045	0.457	5.979	6.150	5.316	226541	Klhl20	chr1	-	NM_0010	P23026	protein_cc	AI504637 |	kelch-like	Klhl20
945	Ankrd45	2.729	0.800	2.185	0.303	0.755	0.423	0.001	1.449	1.128	73844	Ankrd45	chr1	+	NM_0286	P25174	protein_cc	4933409K	ankyrin re	Ankrd45
946	4930469G	1.581	0.879	1.390	0.301	0.744	0.423	0.000	0.661	0.476	10050259	4930469G	chr1	-	NM_0011	P12105	protein_cc	.	RIKEN cDN	4930469G
947	Prdx6	0.210	1.533	0.322	0.000	0.010	0.006	12.892	10.643	11.259	11758	Prdx6	chr1	-	NR_13015	.	protein_cc	1-Cys Prx |	peroxired	Prdx6
948	Tnfsf4	1.000	1.000	1.000	1.000	1.000	1.000	0.000	0.000	0.000	22164	Tnfsf4	chr1	+	NM_0094	P6589	protein_cc	Ath-1 | Ath	tumor nec	Tnfsf4
949	Tnfsf18	1.000	1.000	1.000	1.000	1.000	1.000	0.000	0.000	0.000	240873	Tnfsf18	chr1	+	NM_1833	P21485	protein_cc	Gitrl | Tnlg	tumor nec	Tnfsf18
950	Fasl	1.572	0.636	1.000	0.304	1.000	0.423	0.000	0.653	0.000	14103	Fasl	chr1	-	NM_0012	P11883	protein_cc	APT1LG1 |	Fas ligand	Fasl
951	Suco	4.141	0.775	3.209	0.000	0.112	0.081	7.556	9.606	9.238	226551	Suco	chr1	-	NM_1726	P16396	protein_cc	4732491B	SUN doma	Suco
952	4930558K	1.000	1.000	1.000	1.000	1.000	1.000	0.000	0.000	0.000	75368	4930558K	chr1	-	NM_0012	P24221	protein_cc	.	RIKEN cDN	4930558K
953	Pigc	0.815	0.870	0.710	0.014	0.105	0.020	8.383	8.089	7.888	67292	Pigc	chr1	+	NM_0260	P4979	protein_cc	3110030E	phosphati	Pigc
954	Dnm3	0.483	3.750	1.810	0.190	0.183	0.632	1.107	0.056	1.963	103967	Dnm3	chr1	-	NM_0010	P16145	protein_cc	9630020E	dynamin 3	Dnm3
955	2810442N	0.451	0.986	0.444	0.182	1.000	0.423	1.170	0.021	0.000	72753	2810442N	chr1	+	NR_04056	.	lncRNA	.	RIKEN cDN	2810442N
956	Dnm3os	0.288	0.989	0.285	0.020	0.970	0.156	3.326	1.532	1.515	474332	Dnm3os	chr1	+	NR_00287	.	lncRNA	6030416H	dynamin 3	Dnm3os
957	Mettl13	1.809	0.970	1.755	0.031	0.668	0.185	4.917	5.772	5.728	71449	Mettl13	chr1	-	NM_1448	P23302	protein_cc	5630401D	methyltra	Mettl13
958	Vamp4	1.271	1.338	1.701	0.220	0.113	0.143	4.234	4.581	5.000	53330	Vamp4	chr1	+	NM_0167	P3569	protein_cc	D1Ertd147	vesicle-as	Vamp4
959	Myoc	1.000	1.000	1.000	1.000	1.000	1.000	0.000	0.000	0.000	17926	Myoc	chr1	+	NM_0108	P6065	protein_cc	AI957332 |	myocilin	Myoc
960	7420461P	1.000	1.000	1.000	1.000	1.000	1.000	0.000	0.000	0.000	10003865	7420461P	chr1	-	NM_0011	P6261	protein_cc	ENSMUSG	RIKEN cDN	7420461P
961	Prrc2c	1.423	0.567	0.807	0.002	0.005	0.362	9.597	10.107	9.288	226562	Prrc2c	chr1	-	NM_0010	P24684	protein_cc	1810043M	proline-ri	Prrc2c
962	Fmo4	0.795	1.983	1.576	0.650	0.148	0.121	3.495	3.163	4.151	226564	Fmo4	chr1	-	NM_1448	P12749	protein_cc	D1Ertd532	flavin cont	Fmo4
963	Fmo1	0.168	0.536	0.090	0.000	0.002	0.006	10.310	7.738	6.838	14261	Fmo1	chr1	-	NM_0102	P13114	protein_cc	.	flavin cont	Fmo1
964	Fmo2	0.129	1.117	0.144	0.001	0.690	0.030	6.870	3.912	4.072	55990	Fmo2	chr1	-	NM_0188	P7075	protein_cc	2310008D	flavin cont	Fmo2
965	Fmo6	1.000	1.000	1.000	1.000	1.000	1.000	0.000	0.000	0.000	226565	Fmo6	chr1	-	NM_0011	P16856	protein_cc	.	flavin cont	Fmo6
966	Fmo3	0.040	0.098	0.004	0.001	0.000	0.030	9.962	5.316	1.968	14262	Fmo3	chr1	-	NM_0080	P22791	protein_cc	AW11179	flavin cont	Fmo3
967	Mroh9	1.000	1.000	1.000	1.000	1.000	1.000	0.000	0.000	0.000	78258	Mroh9	chr1	-	NM_0300	P2159	protein_cc	4921528O	maestro h	Mroh9
968	Prrx1	0.451	1.850	0.833	0.001	0.425	0.837	2.958	1.808	2.695	18933	Prrx1	chr1	-	NM_1756	P19043	protein_cc	A230024N	paired rela	Prrx1
969	Gorab	1.633	1.036	1.692	0.150	0.878	0.091	5.337	6.044	6.095	98376	Gorab	chr1	-	NM_1788	P18243	protein_cc	NTKL-BP1	golgin, RA	Gorab
970	Mettl11b	1.000	1.000	1.000	1.000	1.000	1.000	0.000	0.000	0.000	240879	Mettl11b	chr1	-	NM_0011	P12307	protein_cc	Gm207 | N	methyltra	Mettl11b
971	Kifap3	0.561	0.741	0.416	0.001	0.052	0.029	6.759	5.926	5.494	16579	Kifap3	chr1	+	NM_0013	P19596	protein_cc	KAP-3 | KA	kinesin-as	Kifap3
972	Scyl3	1.349	0.986	1.330	0.049	0.742	0.217	6.532	6.963	6.943	240880	Scyl3	chr1	+	NM_0012	P8752	protein_cc	1200016D	SCY1-like	Scyl3
973	BC055324	2.675	1.003	2.682	0.000	0.983	0.087	3.931	5.351	5.354	381306	BC055324	chr1	-	NM_2013	P12093	protein_cc	.	cDNA seq	BC055324
974	Mettl18	3.711	0.644	2.390	0.000	0.051	0.169	4.689	6.581	5.946	69962	Mettl18	chr1	+	NM_0272	P4695	protein_cc	2810422O	methyltra	Mettl18
975	Sele	18.894	0.367	6.940	0.000	0.000	0.064	0.002	4.242	2.797	20339	Sele	chr1	+	NM_0113	P3572	protein_cc	CD62E | E-	selectin, e	Sele
976	Sell	23.240	0.255	5.937	0.002	0.005	0.019	2.704	7.242	5.273	20343	Sell	chr1	+	NM_0110	P7945	protein_cc	AI528707 |	selectin, ly	Sell
977	Selp	40.605	0.801	32.511	0.005	0.402	0.062	3.190	8.534	8.213	20344	Selp	chr1	+	NM_0113	P6521	protein_cc	CD62P | GI	selectin, p	Selp
978	Gm16548	0.338	1.408	0.475	0.000	0.005	0.001	7.159	5.593	6.087	10050381	Gm16548	chr1	-	NR_03798	.	lncRNA	.	predicted	Gm16548
979	F5	0.181	1.296	0.235	0.000	0.010	0.000	13.486	11.021	11.395	14067	F5	chr1	+	NM_0079	P8575	protein_cc	AI173222 |	coagulatio	F5
980	Slc19a2	0.288	1.349	0.389	0.003	0.547	0.068	9.557	7.762	8.194	116914	Slc19a2	chr1	+	NM_0012	P5163	protein_cc	AV276020	solute car	Slc19a2
981	Ccdc181	1.016	1.149	1.168	0.905	0.372	0.437	6.080	6.103	6.304	74895	Ccdc181	chr1	+	NM_0291	P12016	protein_cc	4930455F	coiled-coil	Ccdc181
982	Blzf1	2.272	0.839	1.907	0.001	0.048	0.013	6.513	7.697	7.444	66352	Blzf1	chr1	-	NM_0255	P4852	protein_cc	1700030G	basic leuc	Blzf1
983	Nme7	0.294	1.859	0.547	0.001	0.033	0.127	9.139	7.374	8.268	171567	Nme7	chr1	+	NM_0012	P13583	protein_cc	D530024H	NME/NM2	Nme7
984	Atp1b1	0.218	1.827	0.399	0.000	0.002	0.021	10.389	8.193	9.062	11931	Atp1b1	chr1	-	NM_0097	P26224	protein_cc	Atp4b | At	ATPase, N	Atp1b1
985	Dpt	0.245	4.357	1.068	0.000	0.000	0.556	6.103	4.074	6.197	56429	Dpt	chr1	+	NM_0197	P2816	protein_cc	1810032B	dermatop	Dpt
986	Gm20743	1.000	1.000	1.000	1.000	1.000	1.000	0.000	0.000	0.000	433374	Gm20743	chr1	+	NR_04030	.	lncRNA	.	predicted	Gm20743
987	Xcl1	5.640	0.059	0.331	0.015	0.009	0.015	1.953	4.449	0.358	16963	Xcl1	chr1	-	NM_0085	P8004	protein_cc	AI661682 |	chemokin	Xcl1
988	4930568G	1.000	1.000	1.000	1.000	1.000	1.000	0.000	0.000	0.000	75857	4930568G	chr1	-	NR_04048	.	lncRNA	.	RIKEN cDN	4930568G

989	Tbx19	1.000	1.000	1.000	1.000	1.000	1.000	0.000	0.000	0.000	83993	Tbx19	chr1	-	NM_0320	P16969	protein_cc	AU01925C	T-box 19	Tbx19
990	Sft2d2	0.261	1.879	0.490	0.000	0.002	0.039	9.417	7.477	8.387	108735	Sft2d2	chr1	-	NM_1455	P10539	protein_cc	20100050	SFT2 doma	Sft2d2
991	Tiprl	0.992	1.239	1.229	0.748	0.109	0.341	8.228	8.216	8.525	226591	Tiprl	chr1	-	NM_1455	P11559	protein_cc	1810011K	TIP41, TOI	Tiprl
992	Gpr161	2.134	0.469	1.000	0.305	1.000	0.423	0.000	1.094	0.000	240888	Gpr161	chr1	+	NM_0010	P22246	protein_cc	Gm208| G	G protein-	Gpr161
993	Dcaf6	0.173	1.878	0.324	0.000	0.011	0.008	8.884	6.349	7.258	74106	Dcaf6	chr1	-	NM_0287	P17772	protein_cc	1200006M	DDB1 and	Dcaf6
994	Mpc2	0.312	0.994	0.310	0.001	0.945	0.023	12.252	10.571	10.562	70456	Mpc2	chr1	+	NM_0274	P4866	protein_cc	0610006C	mitochond	Mpc2
995	Adcy10	0.716	0.995	0.712	0.182	1.000	0.423	0.490	0.008	0.000	271639	Adcy10	chr1	+	NM_1730	P14099	protein_cc	4930431D	adenylate	Adcy10
996	Mpzl1	0.652	0.895	0.584	0.003	0.067	0.043	6.556	5.940	5.780	68481	Mpzl1	chr1	-	NM_0010	P2434	protein_cc	1110007A	myelin pro	Mpzl1
997	Rcsd1	1.390	0.926	1.288	0.012	0.570	0.449	5.093	5.569	5.458	226594	Rcsd1	chr1	-	NM_1785	P16902	protein_cc	A430105K	RCSD dom	Rcsd1
998	Creg1	0.302	1.307	0.394	0.000	0.011	0.002	13.186	11.457	11.844	433375	Creg1	chr1	+	NM_0118	P6253	protein_cc	AA755314	cellular re	Creg1
999	Cd247	1.197	0.217	0.260	0.624	0.037	0.169	3.799	4.058	1.854	12503	Cd247	chr1	+	NR_10371	.	protein_cc	4930549J(	CD247 ant	Cd247
1000	Pou2f1	0.895	1.028	0.920	0.302	0.902	0.825	7.807	7.647	7.686	18986	Pou2f1	chr1	-	NM_0111	P20142	protein_cc	2810482H	POU doma	Pou2f1
1001	Dusp27	1.000	1.000	1.000	1.000	1.000	1.000	0.000	0.000	0.000	240892	Dusp27	chr1	-	NM_0010	P19558	protein_cc	C130085G	dual speci	Dusp27
1002	Gpa33	1.000	1.000	1.000	1.000	1.000	1.000	0.000	0.000	0.000	59290	Gpa33	chr1	+	NM_0216	P1294	protein_cc	2010310L	glycoprote	Gpa33
1003	Mael	1.000	1.000	1.000	1.000	1.000	1.000	0.000	0.000	0.000	98558	Mael	chr1	-	NM_1752	P23265	protein_cc	4933405K	maelstrom	Mael
1004	Ildr2	0.159	2.211	0.352	0.000	0.109	0.060	9.643	6.993	8.138	10003979	Ildr2	chr1	+	NM_0011	P5686	protein_cc	2810478N	immunogl	Ildr2
1005	Tada1	1.438	1.202	1.729	0.138	0.465	0.244	7.641	8.165	8.431	27878	Tada1	chr1	+	NM_0302	P9310	protein_cc	2900026B	transcripti	Tada1
1006	Pogk	2.332	0.406	0.947	0.008	0.014	0.891	6.501	7.723	6.423	71592	Pogk	chr1	-	NM_0011	P4622	protein_cc	9130401E	pogo trans	Pogk
1007	Gm4846	1.000	1.000	1.000	1.000	1.000	1.000	0.000	0.000	0.000	226601	Gm4846	chr1	-	NM_0011	P19282	protein_cc	EG226601	predicted	Gm4846
1008	Gm4847	1.000	1.000	1.000	1.000	1.000	1.000	0.000	0.000	0.000	226604	Gm4847	chr1	-	NM_0011	P12023	protein_cc	EG226604	predicted	Gm4847
1009	Fmo9	1.000	1.000	1.000	1.000	1.000	1.000	0.000	0.000	0.000	240894	Fmo9	chr1	-	NM_1728	P5872	protein_cc	4831428F	flavin cont	Fmo9
1010	Gm16701	1.000	1.000	1.000	1.000	1.000	1.000	0.000	0.000	0.000	10050396	Gm16701	chr1	-	NR_03798	.	lncRNA	.	predicted	Gm16701
1011	Fam78b	3.772	0.405	1.529	0.036	0.072	0.523	0.743	2.659	1.356	226610	Fam78b	chr1	+	NM_0011	P13011	protein_cc	C030014K	family wit	Fam78b
1012	Uck2	12.948	0.584	7.560	0.000	0.017	0.099	5.483	9.178	8.401	80914	Uck2	chr1	-	NM_0307	P18852	protein_cc	AA407809	uridine-cy	Uck2
1013	Tmco1	0.533	1.059	0.564	0.001	0.740	0.089	10.646	9.738	9.820	68944	Tmco1	chr1	+	NM_0010	P15885	protein_cc	1190006A	transmem	Tmco1
1014	Aldh9a1	0.787	0.936	0.737	0.040	0.543	0.039	10.733	10.388	10.293	56752	Aldh9a1	chr1	+	NM_0199	P6621	protein_cc	AA139417	aldehyde	Aldh9a1
1015	Mgst3	0.506	0.498	0.252	0.044	0.208	0.020	6.664	5.682	4.676	66447	Mgst3	chr1	-	NM_0255	P20205	protein_cc	2010012L	microsom	Mgst3
1016	Lrrc52	1.000	1.000	1.000	1.000	1.000	1.000	0.000	0.000	0.000	240899	Lrrc52	chr1	-	NM_0010	P485	protein_cc	4930413P	leucine ric	Lrrc52
1017	Rxrg	0.156	1.114	0.174	0.000	0.812	0.005	6.712	4.030	4.185	20183	Rxrg	chr1	+	NM_0091	P2003	protein_cc	Nr2b3	retinoid X	Rxrg
1018	Lmx1a	1.010	1.885	1.904	0.000	0.182	0.423	0.000	0.015	0.929	110648	Lmx1a	chr1	+	NM_0336	P7080	protein_cc	dr| dreher	LIM home	Lmx1a
1019	Pbx1	0.128	2.392	0.307	0.000	0.000	0.018	9.333	6.370	7.629	18514	Pbx1	chr1	-	NM_0087	P6172	protein_cc	2310056B	pre B cell	Pbx1
1020	Nuf2	0.248	2.627	0.650	0.183	0.187	0.726	2.078	0.064	1.457	66977	Nuf2	chr1	-	NM_0232	P7658	protein_cc	2410003C	NUF2, ND	Nuf2
1021	Rgs5	1.741	1.159	2.018	0.035	0.390	0.065	6.253	7.053	7.265	19737	Rgs5	chr1	+	NM_0090	P5708	protein_cc	1110070A	regulator	Rgs5
1022	Rgs4	1.086	1.260	1.368	0.707	0.623	0.691	5.775	5.893	6.227	19736	Rgs4	chr1	-	NM_0090	P1565	protein_cc	AA004315	regulator	Rgs4
1023	1700084C	1.000	1.000	1.000	1.000	1.000	1.000	0.000	0.000	0.000	.	1700084C	chr1	+	NM_0010	P14574	.	.	.	1700084C
1024	Hsd17b7	1.106	2.277	2.518	0.315	0.002	0.048	9.512	9.658	10.845	15490	Hsd17b7	chr1	-	NM_0104	P17581	protein_cc	AI266814|	hydroxyst	Hsd17b7
1025	3110045C	0.916	1.416	1.297	0.765	0.122	0.566	4.672	4.545	5.047	67303	3110045C	chr1	+	NR_04043	.	lncRNA	AI838577	RIKEN cDN	3110045C
1026	Ddr2	0.783	0.986	0.772	0.160	0.936	0.041	6.489	6.136	6.115	18214	Ddr2	chr1	-	NM_0225	P36	protein_cc	AW49525	discoidin	Ddr2
1027	Uap1	1.586	0.709	1.125	0.025	0.049	0.405	8.546	9.212	8.715	107652	Uap1	chr1	-	NM_1338	P25799	protein_cc	AA420407	UDP-N-ac	Uap1
1028	Uhmk1	3.615	0.838	3.031	0.094	0.649	0.010	1.906	3.760	3.506	16589	Uhmk1	chr1	-	NM_0106	P15057	protein_cc	4732477C	U2AF hom	Uhmk1
1029	Sh2d1b2	1.000	1.000	1.000	1.000	1.000	1.000	0.000	0.000	0.000	545378	Sh2d1b2	chr1	+	NM_0010	P14358	protein_cc	EAT-2B| E	SH2 doma	Sh2d1b2
1030	Sh2d1b1	1.000	1.000	1.000	1.000	1.000	1.000	0.000	0.000	0.000	26904	Sh2d1b1	chr1	+	NM_0120	P26595	protein_cc	EAT-2| EA	SH2 doma	Sh2d1b1
1031	Gm7694	5.638	0.622	3.510	0.000	0.011	0.031	5.155	7.650	6.966	665574	Gm7694	chr1	-	NM_0011	P9584	protein_cc	EG665574	predicted	Gm7694
1032	1700015E	1.000	1.000	1.000	1.000	1.000	1.000	0.000	0.000	0.000	76925	1700015E	chr1	+	NM_0010	P22422	protein_cc	.	RIKEN cDN	1700015E1
1033	Nos1ap	6.033	0.500	3.014	0.001	0.011	0.053	4.745	7.338	6.337	70729	Nos1ap	chr1	-	NM_0011	P3923	protein_cc	6330408P	nitric oxid	Nos1ap
1034	Olfml2b	1.398	0.553	0.774	0.442	0.260	0.288	3.629	4.113	3.259	320078	Olfml2b	chr1	+	NM_1770	P70	protein_cc	1110018N	olfactome	Olfml2b
1035	Atf6	0.644	1.387	0.894	0.011	0.188	0.727	8.739	8.106	8.577	226641	Atf6	chr1	-	NM_0010	P13097	protein_cc	9130025P	activating	Atf6
1036	Dusp12	0.577	1.123	0.647	0.008	0.677	0.314	7.517	6.723	6.890	80915	Dusp12	chr1	-	NM_0231	P11968	protein_cc	1190004O	dual speci	Dusp12
1037	Fcrlb	1.010	1.825	1.844	0.000	0.182	0.423	0.000	0.015	0.883	435653	Fcrlb	chr1	-	NM_0010	P19534	protein_cc	FcRL2| Fcr	Fc recepto	Fcrlb
1038	Fcrla	0.649	0.838	0.544	0.090	0.646	0.189	3.320	2.697	2.442	98752	Fcrla	chr1	-	NM_1451	P19527	protein_cc	BB219290	Fc recepto	Fcrla
1039	Fcgr2b	0.433	0.818	0.354	0.013	0.028	0.098	7.487	6.280	5.990	14130	Fcgr2b	chr1	-	NM_0101	P19429	protein_cc	AI528646|	Fc recepto	Fcgr2b
1040	Fcgr4	14.523	0.479	6.958	0.003	0.033	0.099	5.855	9.715	8.654	246256	Fcgr4	chr1	+	NM_1445	P17754	protein_cc	4833442P	Fc recepto	Fcgr4
1041	Fcgr3	2.935	0.757	2.223	0.000	0.034	0.069	6.843	8.397	7.996	14131	Fcgr3	chr1	-	NM_0101	P25670	protein_cc	CD16	Fc recepto	Fcgr3
1042	Cfap126	0.305	2.339	0.713	0.009	0.039	0.412	4.777	3.062	4.288	75472	Cfap126	chr1	+	NM_0010	P20253	protein_cc	1700009P	cilia and fl	Cfap126
1043	Sdhc	0.565	0.951	0.538	0.000	0.313	0.006	11.391	10.567	10.495	66052	Sdhc	chr1	-	NM_0253	P25482	protein_cc	0610010E	succinate	Sdhc
1044	Mpz	0.857	1.335	1.144	0.832	0.700	0.907	1.721	1.499	1.916	17528	Mpz	chr1	+	NM_0086	P2274	protein_cc	Mpp| P-ze	myelin pro	Mpz
1045	Pcp4l1	0.015	1.989	0.029	0.000	0.346	0.013	6.906	0.828	1.820	66425	Pcp4l1	chr1	-	NM_0255	P15959	protein_cc	1500036F	Purkinje c	Pcp4l1
1046	Nr1i3	0.024	1.791	0.043	0.000	0.150	0.010	9.129	3.732	4.573	12355	Nr1i3	chr1	+	NM_0098	P6838	protein_cc	AA209988	nuclear re	Nr1i3
1047	Tomm40l	0.057	2.083	0.119	0.000	0.132	0.006	9.898	5.766	6.824	641376	Tomm40l	chr1	-	NM_0010	P20730	protein_cc	.	translocas	Tomm40l
1048	Apoa2	0.528	0.906	0.478	0.004	0.362	0.050	16.473	15.551	15.409	11807	Apoa2	chr1	+	NM_0013	P11438	protein_cc	Alp-2| Apo	apolipopro	Apoa2
1049	Fcer1g	5.040	0.501	2.525	0.003	0.016	0.034	7.702	10.035	9.038	14127	Fcer1g	chr1	-	NM_0101	P10881	protein_cc	AI573376|	Fc recepto	Fcer1g
1050	Ndufs2	0.654	1.106	0.723	0.000	0.163	0.054	10.602	9.989	10.134	226646	Ndufs2	chr1	-	NM_1530	P13430	protein_cc	CI-49kD	NADH deh	Ndufs2
1051	Adamts4	79.811	0.417	33.304	0.002	0.039	0.208	0.589	6.908	5.647	240913	Adamts4	chr1	+	NM_1728	P25718	protein_cc	3830423K	a disintegr	Adamts4
1052	B4galt3	1.256	0.985	1.237	0.291	0.908	0.600	6.281	6.610	6.588	57370	B4galt3	chr1	+	NM_0205	P13499	protein_cc	9530061M	UDP-Gal:b	B4galt3
1053	Ppox	0.721	1.155	0.833	0.359	0.638	0.596	4.531	4.060	4.267	19044	Ppox	chr1	-	NM_0089	P18999	protein_cc	Ppo	protoporp	Ppox
1054	Usp21	1.046	0.740	0.774	0.673	0.272	0.612	6.404	6.468	6.034	30941	Usp21	chr1	-	NM_0139	P22839	protein_cc	ESTM28|	ubiquitin s	Usp21
1055	Ufc1	1.303	0.985	1.283	0.000	0.626	0.039	9.675	10.057	10.035	66155	Ufc1	chr1	-	NM_0253	P10409	protein_cc	1110021H	ubiquitin-	Ufc1
1056	Dedd	0.585	1.121	0.655	0.001	0.195	0.028	9.418	8.643	8.808	21945	Dedd	chr1	+	NM_0011	P13756	protein_cc	CASP8IP1|	death effe	Dedd
1057	Nit1	0.387	1.269	0.491	0.001	0.068	0.064	8.791	7.420	7.764	27045	Nit1	chr1	-	NM_0120	P2959	protein_cc	AI255805|	nitrilase 1	Nit1
1058	Pfdn2	1.022	1.042	1.064	0.896	0.797	0.725	8.891	8.921	8.980	18637	Pfdn2	chr1	+	NM_0110	P25498	protein_cc	ESTM27|	prefoldin	Pfdn2
1059	Klhdc9	0.629	0.771	0.485	0.279	0.489	0.374	3.366	2.697	2.322	68874	Klhdc9	chr1	-	NM_0010	P11202	protein_cc	1190002J	kelch dom	Klhdc9
1060	Pvrl4	0.674	1.938	1.307	0.199	0.152	0.673	2.089	1.521	2.475	.	Pvrl4	chr1	+	NM_0278	P19276	.	.	.	Pvrl4
1061	Arhgap30	2.551	0.747	1.907	0.000	0.101	0.214	5.487	6.838	6.418	226652	Arhgap30	chr1	+	NM_0010	P8125	protein_cc	6030405P	Rho GTPa	Arhgap30
1062	Usf1	0.650	1.403	0.911	0.033	0.132	0.756	4.201	3.579	4.067	22278	Usf1	chr1	+	NM_0013	P6310	protein_cc	bHLHb11	upstream	Usf1
1063	Tstd1	0.650	0.912	0.592	0.009	0.718	0.207	7.885	7.263	7.130	226654	Tstd1	chr1	+	NM_0011	P5788	protein_cc	AV065366	thiosulfate	Tstd1
1064	F11r	0.806	1.595	1.286	0.082	0.003	0.062	10.661	10.350	11.024	16456	F11r	chr1	+	NM_1726	P6994	protein_cc	9130004G	F11 recept	F11r
1065	Alyref2	2.601	0.607	1.579	0.000	0.000	0.071	5.459	6.838	6.117	56009	Alyref2	chr1	+	NM_0194	P18274	protein_cc	C130042O	Aly/REF ex	Alyref2
1066	Itln1	1.000	1.000	1.000	1.000	1.000	1.000	0.000	0.000	0.000	16429	Itln1	chr1	-	NM_0105	P5936	protein_cc	IntL| Itln|	intelectin	Itln1
1067	Cd244	4.168	0.536	2.235	0.007	0.028	0.100	0.735	2.794	1.895	18106	Cd244	chr1	+	NM_0187	P23804	protein_cc	2B4| C9.1	CD244 nat	Cd244
1068	Ly9	2.324	0.818	1.901	0.046	0.541	0.347	2.555	3.771	3.481	17085	Ly9	chr1	-	NM_0085	P12765	protein_cc	AI893573|	lymphocyt	Ly9
1069	Slamf7	53.560	0.255	13.653	0.000	0.003	0.241	2.662	8.405	6.434	75345	Slamf7	chr1	-	NM_1445	P7667	protein_cc	19A| 19A	SLAM fam	Slamf7
1070	Cd48	1.856	0.590	1.095	0.081	0.092	0.761	4.602	5.495	4.733	12506	Cd48	chr1	+	NM_0076	P23967	protein_cc	AI449234|	CD48 anti	Cd48
1071	Slamf1	112.005	0.274	30.699	0.004	0.017	0.218	0.697	7.505	5.637	27218	Slamf1	chr1	+	NM_0137	P3913	protein_cc	4933415F	signaling l	Slamf1
1072	Cd84	1.343	0.825	1.108	0.337	0.417	0.886	4.947	5.373	5.095	12523	Cd84	chr1	+	NM_0134	P25015	protein_cc	A130013D	CD84 anti	Cd84
1073	Slamf6	1.962	0.639	1.254	0.192	0.365	0.778	0.779	1.752	1.106	30925	Slamf6	chr1	+	NM_0307	P3753	protein_cc	KAL1| KAL	SLAM fam	Slamf6
1074	Vangl2	0.912	0.866	0.789	0.906	0.841	0.815	1.884	1.751	1.543	93840	Vangl2	chr1	-	NM_0335	P1559	protein_cc	C530001F	vang-like	Vangl2
1075	Nhlh1	1.000	1.000	1.000	1.000	1.000	1.000	0.000	0.000	0.000	18071	Nhlh1	chr1	-	NM_0109	P4893	protein_cc	bHLHa35|	nescient h	Nhlh1
1076	Ncstn	1.282	1.045	1.340	0.508	0.909	0.571	6.375	6.734	6.797	59287	Ncstn	chr1	-	NM_0216	P8428	protein_cc	9430068N	nicastrin	Ncstn
1077	Copa	1.208	0.755	0.912	0.107	0.042	0.367	10.544	10.816	10.410	12847	Copa	chr1	+	NM_0099	P17380	protein_cc	AU040324	coatomer	Copa
1078	Pex19	0.612	1.637	1.002	0.001	0.018	0.992	9.332	8.624	9.335	19298	Pex19	chr1	+	NM_0011	P2679	protein_cc	Pxf	peroxisom	Pex19
1079	Dcaf8	0.577	1.334	0.769	0.008	0.043	0.138	9.971	9.177	9.593	98193	Dcaf8	chr1	+	NM_1535	P16366	protein_cc	AA408877	DDB1 and	Dcaf8

1080	Pea15a	0.851	1.204	1.024	0.256	0.362	0.944	7.954	7.720	7.988	18611	Pea15a	chr1	-	NM_0110	P15888	protein_cc	Mat1| PEA	phosphop	Pea15a
1081	Casq1	1.000	1.000	1.000	1.000	1.000	1.000	0.000	0.000	0.000	12372	Casq1	chr1	-	NM_0098	P17487	protein_cc	CSQ| CSQ	calseques	Casq1
1082	Atp1a4	0.735	1.802	1.324	0.191	0.184	0.672	0.466	0.021	0.871	27222	Atp1a4	chr1	-	NM_0137	P6396	protein_cc	.	ATPase, N	Atp1a4
1083	Atp1a2	0.046	16.957	0.772	0.030	0.000	0.677	4.628	0.171	4.255	98660	Atp1a2	chr1	-	NM_1784	P6757	protein_cc	Atpa-3| A	ATPase, N	Atp1a2
1084	Igsf8	1.403	0.701	0.983	0.151	0.098	0.960	6.944	7.432	6.920	140559	Igsf8	chr1	+	NM_0804	P9018	protein_cc	ESTM34| I	immunogl	Igsf8
1085	Kcnj9	0.716	0.995	0.712	0.182	1.000	0.423	0.490	0.008	0.000	16524	Kcnj9	chr1	-	NM_0084	P2004	protein_cc	1700085N	potassium	Kcnj9
1086	Kcnj10	0.143	0.438	0.063	0.003	1.000	0.035	3.993	1.192	0.000	16513	Kcnj10	chr1	+	NM_0010	P11378	protein_cc	BIR10| BIF	potassium	Kcnj10
1087	Pigm	2.601	0.915	2.380	0.000	0.675	0.215	6.458	7.837	7.709	67556	Pigm	chr1	+	NM_0262	P19048	protein_cc	4933437L(	phosphati	Pigm
1088	Slamf9	7.565	1.823	13.794	0.000	0.003	0.014	1.625	4.544	5.411	98365	Slamf9	chr1	+	NM_0296	P9260	protein_cc	2310026IC	SLAM fam	Slamf9
1089	Igsf9	0.257	3.823	0.983	0.001	0.082	0.976	2.710	0.750	2.685	93842	Igsf9	chr1	+	NM_0011	P9058	protein_cc	644ETD8|	immunogl	Igsf9
1090	Tagln2	3.086	0.837	2.583	0.002	0.435	0.195	8.429	10.055	9.799	21346	Tagln2	chr1	+	NM_1785	P20178	protein_cc	2700094C	transgelin	Tagln2
1091	Cfap45	1.572	0.636	1.000	0.304	1.000	0.423	0.000	0.653	0.000	71870	Cfap45	chr1	+	NM_0279	P7961	protein_cc	1700028D	cilia and fl	Cfap45
1092	4933439K	1.005	1.405	1.412	0.000	0.182	0.423	0.000	0.008	0.498	71319	4933439K	chr1	-	NR_04055	.	lncRNA	.	RIKEN cDN	4933439K1
1093	Vsig8	1.572	0.636	1.000	0.304	1.000	0.423	0.000	0.653	0.000	240916	Vsig8	chr1	+	NM_1777	P22043	protein_cc	A030011N	V-set and	Vsig8
1094	Slamf8	21.953	0.219	4.805	0.000	0.001	0.351	4.534	8.990	6.798	74748	Slamf8	chr1	-	NM_0290	P20103	protein_cc	5830408F(	SLAM fam	Slamf8
1095	Fcrl6	1.000	1.000	1.000	1.000	1.000	1.000	0.000	0.000	0.000	677296	Fcrl6	chr1	-	NM_0011	P20717	protein_cc	ENSMUSG	Fc recepto	Fcrl6
1096	Dusp23	1.148	1.045	1.199	0.209	0.821	0.623	8.456	8.656	8.719	68440	Dusp23	chr1	-	NM_0267	P5603	protein_cc	1300005N	dual speci	Dusp23
1097	Crp	3.021	0.620	1.873	0.002	0.047	0.206	12.450	14.045	13.356	12944	Crp	chr1	+	NM_0077	P18633	protein_cc	AI255847	C-reactive	Crp
1098	Apcs	23.057	0.803	18.506	0.000	0.001	0.002	10.085	14.612	14.295	20219	Apcs	chr1	-	NM_0113	P17163	protein_cc	Sap	serum am	Apcs
1099	Olfr16	1.010	1.885	1.904	0.000	0.182	0.423	0.000	0.015	0.929	18313	Olfr16	chr1	+	NM_0087	P7830	protein_cc	MOR23| N	olfactory r	Olfr16
1100	Olfr1408	1.000	1.000	1.000	1.000	1.000	1.000	0.000	0.000	0.000	258759	Olfr1408	chr1	-	NM_1467	P2542	protein_cc	MOR267-4	olfactory r	Olfr1408
1101	Olfr1406	1.000	1.000	1.000	1.000	1.000	1.000	0.000	0.000	0.000	258758	Olfr1406	chr1	-	NM_1467	P15414	protein_cc	MOR267-5	olfactory r	Olfr1406
1102	Olfr218	1.000	1.000	1.000	1.000	1.000	1.000	0.000	0.000	0.000	258880	Olfr218	chr1	+	NM_0010	P1220	protein_cc	MOR267-3	olfactory r	Olfr218
1103	Olfr1404	1.000	1.000	1.000	1.000	1.000	1.000	0.000	0.000	0.000	258881	Olfr1404	chr1	+	NM_1468	P6611	protein_cc	MOR267-2	olfactory r	Olfr1404
1104	Fcer1a	1.000	1.000	1.000	1.000	1.000	1.000	0.000	0.000	0.000	14125	Fcer1a	chr1	-	NM_0101	P23055	protein_cc	Fce1a| fce	Fc recepto	Fcer1a
1105	Olfr418	1.000	1.000	1.000	1.000	1.000	1.000	0.000	0.000	0.000	258645	Olfr418	chr1	+	NM_1466	P24613	protein_cc	MOR267-1	olfactory r	Olfr418
1106	Mptx2	1.000	1.000	1.000	1.000	1.000	1.000	0.000	0.000	0.000	10050423	Mptx2	chr1	-	NM_0012	P25738	protein_cc	ENSMUSG	mucosal p	Mptx2
1107	Ackr1	21.887	0.332	7.273	0.000	0.010	0.264	4.090	8.542	6.953	13349	Ackr1	chr1	-	NM_0100	P2779	protein_cc	AA162249	atypical ch	Ackr1
1108	Cadm3	0.134	0.576	0.077	0.016	0.063	0.120	4.955	2.060	1.263	94332	Cadm3	chr1	-	NM_0531	P2884	protein_cc	BIgR| Igsf4	cell adhesi	Cadm3
1109	Aim2	1.593	1.091	1.739	0.112	0.706	0.233	3.338	4.010	4.136	383619	Aim2	chr1	+	NM_0010	P25879	protein_cc	Gm1313|	absent in	Aim2
1110	BC094916	14.681	0.190	2.796	0.020	0.032	0.235	0.002	3.878	1.485	98256	BC094916	chr1	-	NM_0010	P16962	protein_cc	Ifi214| p2	cDNA sequ	BC094916
1111	Pydc4	22.628	0.273	6.185	0.003	0.008	0.011	1.981	6.481	4.610	623121	Pydc4	chr1	-	NM_0011	P21676	protein_cc	E030037K(	pyrin dom	Pydc4
1112	Pyhin1	15.042	0.321	4.832	0.000	0.000	0.006	4.188	8.099	6.461	236312	Pyhin1	chr1	+	NM_1750	P613	protein_cc	4930422C	pyrin and	Pyhin1
1113	Pydc3	10.742	0.304	3.261	0.001	0.002	0.103	4.716	8.141	6.421	10003345	Pydc3	chr1	+	NM_0011	P21790	protein_cc	9830148g	pyrin dom	Pydc3
1114	AI607873	6.011	0.338	2.030	0.000	0.001	0.014	5.478	8.066	6.500	226691	AI607873	chr1	-	NM_0012	P14359	protein_cc	.	expressed	AI607873
1115	Ifi204	9.923	0.324	3.211	0.000	0.000	0.066	6.419	9.730	8.102	15951	Ifi204	chr1	-	NM_0083	P11187	protein_cc	Ifi16| p20	interferon	Ifi204
1116	Mndal	4.956	0.219	1.087	0.000	0.000	0.545	8.198	10.507	8.318	10004046	Mndal	chr1	-	NM_0011	P12310	protein_cc	10004046	myeloid nu	Mndal
1117	Mnda	19.087	0.309	5.906	0.000	0.000	0.011	6.180	10.435	8.742	381308	Mnda	chr1	-	NM_0010	P21231	protein_cc	A730048F	myeloid ce	Mnda
1118	Ifi203	6.028	0.287	1.731	0.000	0.000	0.097	7.832	10.424	8.624	15950	Ifi203	chr1	-	NM_0013	P15911	protein_cc	.	interferon	Ifi203
1119	Ifi202b	1.000	1.000	1.000	1.000	1.000	1.000	0.000	0.000	0.000	26388	Ifi202b	chr1	-	NM_0119	P19363	protein_cc	Ifbip-1| Ifi	interferon	Ifi202b
1120	Ifi205	51.528	0.283	14.573	0.000	0.001	0.002	4.632	10.320	8.498	226695	Ifi205	chr1	-	NM_1726	P18674	protein_cc	D3| D3cDI	interferon	Ifi205
1121	Olfr433	1.572	0.636	1.000	0.304	1.000	0.423	0.000	0.653	0.000	258712	Olfr433	chr1	+	NM_1467	P24082	protein_cc	MOR123-1	olfactory r	Olfr433
1122	Olfr432	1.000	1.000	1.000	1.000	1.000	1.000	0.000	0.000	0.000	258711	Olfr432	chr1	+	NM_1467	P7333	protein_cc	MOR123-2	olfactory r	Olfr432
1123	Olfr430	1.000	1.000	1.000	1.000	1.000	1.000	0.000	0.000	0.000	258713	Olfr430	chr1	+	NM_1467	P23335	protein_cc	MOR105-5	olfactory r	Olfr430
1124	Olfr429	1.000	1.000	1.000	1.000	1.000	1.000	0.000	0.000	0.000	258717	Olfr429	chr1	+	NM_1467	P26821	protein_cc	MOR105-1	olfactory r	Olfr429
1125	Olfr426	1.000	1.000	1.000	1.000	1.000	1.000	0.000	0.000	0.000	.	Olfr426	chr1	+	NM_0012	P6781	.	.	.	Olfr426
1126	Olfr427	1.000	1.000	1.000	1.000	1.000	1.000	0.000	0.000	0.000	259162	Olfr427	chr1	+	NM_2071	P25443	protein_cc	MOR105-7	olfactory r	Olfr427
1127	Olfr231	1.000	1.000	1.000	1.000	1.000	1.000	0.000	0.000	0.000	404222	Olfr231	chr1	-	NM_0010	P7069	protein_cc	MOR105-4	olfactory r	Olfr231
1128	Olfr424	1.000	1.000	1.000	1.000	1.000	1.000	0.000	0.000	0.000	258716	Olfr424	chr1	+	NM_1467	P1745	protein_cc	MOR105-2	olfactory r	Olfr424
1129	Olfr421-ps	1.000	1.000	1.000	1.000	1.000	1.000	0.000	0.000	0.000	258715	Olfr421-ps	chr1	+	NR_04766	.	misc_RNA	MOR105-3	olfactory r	Olfr421-ps
1130	Olfr420	1.000	1.000	1.000	1.000	1.000	1.000	0.000	0.000	0.000	258302	Olfr420	chr1	+	NM_1463	P16411	protein_cc	MOR105-1	olfactory r	Olfr420
1131	Spta1	1.603	1.647	2.641	0.292	0.212	0.178	0.001	0.681	1.402	20739	Spta1	chr1	+	NM_0114	P21412	protein_cc	AF093576	spectrin al	Spta1
1132	Olfr419	1.000	1.000	1.000	1.000	1.000	1.000	0.000	0.000	0.000	258710	Olfr419	chr1	-	NM_1467	P16295	protein_cc	MOR267-6	olfactory r	Olfr419
1133	Mptx1	1.000	1.000	1.000	1.000	1.000	1.000	0.000	0.000	0.000	66289	Mptx1	chr1	+	NM_0254	P22413	protein_cc	1810030J1	mucosal p	Mptx1
1134	Olfr417	1.000	1.000	1.000	1.000	1.000	1.000	0.000	0.000	0.000	258238	Olfr417	chr1	+	NM_2071	P14124	protein_cc	GA_x5J8B	olfactory r	Olfr417
1135	Olfr248	1.000	1.000	1.000	1.000	1.000	1.000	0.000	0.000	0.000	258709	Olfr248	chr1	+	NM_1467	P20963	protein_cc	MOR267-7	olfactory r	Olfr248
1136	Olfr414	1.000	1.000	1.000	1.000	1.000	1.000	0.000	0.000	0.000	258756	Olfr414	chr1	+	NM_1467	P15223	protein_cc	MOR103-1	olfactory r	Olfr414
1137	Olfr220	1.000	1.000	1.000	1.000	1.000	1.000	0.000	0.000	0.000	546747	Olfr220	chr1	+	NM_2076	P5205	protein_cc	EG546747	olfactory r	Olfr220
1138	Fmn2	1.727	4.545	7.848	0.096	0.144	0.342	3.285	4.073	6.258	54418	Fmn2	chr1	+	NM_0194	P24180	protein_cc	AU024104	formin 2	Fmn2
1139	Grem2	0.281	1.909	0.537	0.000	0.036	0.072	6.053	4.224	5.157	23893	Grem2	chr1	-	NM_0118	P24472	protein_cc	Gremlin2|	gremlin 2,	Grem2
1140	Rgs7	1.036	4.591	4.757	0.000	0.108	0.329	0.001	0.052	2.251	24012	Rgs7	chr1	-	NM_0011	P24698	protein_cc	.	regulator	Rgs7
1141	Fh1	1.819	1.017	1.849	0.017	0.947	0.297	10.907	11.770	11.794	14194	Fh1	chr1	-	NM_0102	P5816	protein_cc	Fh| Fh-1	fumarate	Fh1
1142	Kmo	0.706	1.357	0.958	0.004	0.001	0.701	10.751	10.248	10.689	98256	Kmo	chr1	+	NM_1338	P21941	protein_cc	AI046660	kynurenin	Kmo
1143	Opn3	1.472	1.683	2.477	0.138	0.017	0.001	3.948	4.506	5.257	13603	Opn3	chr1	-	NM_0100	P625	protein_cc	Ecpn| ERC	opsin 3	Opn3
1144	Chml	2.769	0.585	1.619	0.069	0.199	0.378	3.101	4.571	3.796	12663	Chml	chr1	-	NM_0213	P17880	protein_cc	AI450814|	choroidere	Chml
1145	Wdr64	1.000	1.000	1.000	1.000	1.000	1.000	0.000	0.000	0.000	75820	Wdr64	chr1	+	NM_0294	P13751	protein_cc	49304150	WD repea	Wdr64
1146	Exo1	1.005	1.439	1.446	0.000	0.182	0.423	0.000	0.008	0.532	26909	Exo1	chr1	+	NM_0120	P162	protein_cc	5730442G	exonuclea	Exo1
1147	Becn2	1.000	1.000	1.000	1.000	1.000	1.000	0.000	0.000	0.000	226720	Becn2	chr1	+	NM_0012	P13141	protein_cc	Beclin-2| (	beclin 2	Becn2
1148	Pld5	0.726	1.387	1.007	0.187	0.187	0.988	0.477	0.015	0.487	319455	Pld5	chr1	-	NM_0011	P22543	protein_cc	B230365F	phospholi	Pld5
1149	Cep170	6.699	0.525	3.518	0.000	0.000	0.005	5.057	7.801	6.871	545389	Cep170	chr1	-	NM_0010	P12167	protein_cc	4933426L	centrosom	Cep170
1150	Sdccag8	1.854	0.832	1.543	0.019	0.282	0.098	6.463	7.354	7.088	76816	Sdccag8	chr1	+	NM_0297	P24983	protein_cc	2700048G	serologica	Sdccag8
1151	Hmga2-ps	0.609	2.586	1.575	0.252	0.002	0.387	2.525	1.810	3.180	15365	Hmga2-ps	chr1	-	NR_03799	.	misc_RNA	EG666235	high mobi	Hmga2-ps
1152	Akt3	7.390	0.220	1.627	0.000	0.000	0.042	4.075	6.961	4.777	23797	Akt3	chr1	-	NM_0117	P7587	protein_cc	AI851531|	thymoma	Akt3
1153	Zbtb18	0.584	1.443	0.842	0.000	0.008	0.151	7.788	7.011	7.539	30928	Zbtb18	chr1	+	NM_0010	P24895	protein_cc	RP58| Zfp	zinc finger	Zbtb18
1154	2310043L	1.000	1.000	1.000	1.000	1.000	1.000	0.000	0.000	0.000	75589	2310043L	chr1	-	NR_03799	.	lncRNA	.	RIKEN cDN	2310043L1
1155	1700016C	1.000	1.000	1.000	1.000	1.000	1.000	0.000	0.000	0.000	69428	1700016C	chr1	+	NM_0270	P8528	protein_cc	.	RIKEN cDN	1700016C
1156	Adss	0.904	1.092	0.988	0.037	0.136	0.866	8.301	8.156	8.283	11566	Adss	chr1	-	NM_0074	P9057	protein_cc	Adss2| AI	adenylosu	Adss
1157	Gm16432	0.716	0.995	0.712	0.182	1.000	0.423	0.490	0.008	0.000	545391	Gm16432	chr1	+	NM_0010	P6421	protein_cc	EG545391	predicted	Gm16432
1158	Desi2	1.686	0.936	1.579	0.001	0.629	0.221	7.255	8.009	7.914	78825	Desi2	chr1	+	NM_0242	P3374	protein_cc	5830417C	desumoyla	Desi2
1159	Cox20	0.449	1.322	0.593	0.001	0.002	0.059	10.562	9.406	9.809	66359	Cox20	chr1	+	NM_0255	P3518	protein_cc	2310005N	COX20 Co	Cox20
1160	Hnrnpu	1.282	0.852	1.093	0.001	0.005	0.131	10.697	11.056	10.826	51810	Hnrnpu	chr1	-	NM_0168	P14752	protein_cc	AA408410	heterogen	Hnrnpu
1161	Efcab2	1.079	1.741	1.879	0.829	0.028	0.285	4.059	4.169	4.969	68226	Efcab2	chr1	+	NM_0266	P9531	protein_cc	1700073K	EF-hand ca	Efcab2
1162	Kif26b	3.974	0.744	2.957	0.001	0.252	0.224	0.378	2.368	1.942	269152	Kif26b	chr1	+	NM_0011	P10339	protein_cc	4832420M	kinesin far	Kif26b
1163	Smyd3	0.128	2.605	0.333	0.000	0.007	0.037	6.322	3.353	4.734	69726	Smyd3	chr1	-	NM_0271	P26568	protein_cc	2410008A	SET and M	Smyd3
1164	Tfb2m	0.712	1.038	0.739	0.091	0.685	0.318	7.750	7.260	7.314	15278	Tfb2m	chr1	-	NM_0082	P26766	protein_cc	Hkp1	transcripti	Tfb2m
1165	Cnst	0.720	0.841	0.605	0.480	0.663	0.489	5.123	4.648	4.398	226744	Cnst	chr1	+	NM_1461	P20112	protein_cc	9630058J	consortin,	Cnst
1166	Sccpdh	2.500	1.295	3.238	0.008	0.236	0.100	4.245	5.567	5.940	109232	Sccpdh	chr1	+	NM_1786	P24242	protein_cc	AW21450	saccharop	Sccpdh
1167	Ahctf1	0.853	0.931	0.793	0.000	0.555	0.303	7.660	7.430	7.326	226747	Ahctf1	chr1	-	NM_0263	P24585	protein_cc	6230412P	AT hook c	Ahctf1
1168	Cdc42bpa	0.202	1.323	0.267	0.001	0.417	0.036	8.985	6.676	7.080	226751	Cdc42bpa	chr1	+	NM_0010	P21583	protein_cc	A930014J	CDC42 bin	Cdc42bpa
1169	Adck3	0.017	2.262	0.038	0.000	0.074	0.014	11.040	5.132	6.310	67426	Adck3	chr1	-	NM_0233	P17610	protein_cc	4632432J1	aarF doma	Adck3
1170	Psen2	0.087	1.434	0.125	0.000	0.190	0.002	12.731	9.213	9.733	19165	Psen2	chr1	-	NM_0011	P4157	protein_cc	Ad4h| ALC	presenilin	Psen2

1171	Gm5069	0.967	0.947	0.916	0.215	0.565	0.599	4.865	4.816	4.738	277333	Gm5069	chr1	-	NR_00362	.	misc_RNA	EG277333	predicted	Gm5069
1172	Itpkb	0.789	1.084	0.855	0.724	0.878	0.853	2.739	2.397	2.513	320404	Itpkb	chr1	+	NM_0010	P11375	protein_cc	1110033J(	inositol 1,4	Itpkb
1173	6330403A	0.743	2.306	1.713	0.196	0.183	0.557	0.456	0.027	1.232	.	6330403A	chr1	-	NM_0010	P6732	.			6330403A(
1174	Parp1	0.333	1.332	0.443	0.000	0.024	0.007	8.551	6.963	7.377	11545	Parp1	chr1	+	NM_0074	P2397	protein_cc	5830444G	poly (ADP-	Parp1
1175	Lin9	0.361	1.168	0.421	0.023	0.685	0.253	5.643	4.172	4.396	72568	Lin9	chr1	+	NR_12049	.	protein_cc	2700022J2	lin-9 homo	Lin9
1176	Mixl1	1.146	0.632	0.725	0.729	1.000	0.422	0.465	0.661	0.000	27217	Mixl1	chr1	-	NM_0137	P1001	protein_cc	Mm1| Mn	Mix1 hom	Mixl1
1177	Acbd3	2.298	0.597	1.373	0.001	0.001	0.208	8.855	10.055	9.312	170760	Acbd3	chr1	+	NM_1332	P257	protein_cc	60kDa| 84	acyl-Coen	Acbd3
1178	H3f3a	1.000	1.240	1.241	0.997	0.053	0.355	8.893	8.894	9.204	15078	H3f3a	chr1	-	NM_0082	P12472	protein_cc	H3.3A	H3 histone	H3f3a
1179	Sde2	1.824	0.839	1.531	0.023	0.321	0.130	7.915	8.783	8.530	208768	Sde2	chr1	+	NM_1459	P18095	protein_cc	.	SDE2 telor	Sde2
1180	Lefty2	1.000	1.000	1.000	1.000	1.000	1.000	0.000	0.000	0.000	320202	Lefty2	chr1	+	NM_1770	P25690	protein_cc	6030463A	left-right c	Lefty2
1181	Pycr2	2.424	1.038	2.516	0.019	0.802	0.079	6.661	7.938	7.992	69051	Pycr2	chr1	+	NM_1337	P21668	protein_cc	1810018M	pyrroline-5	Pycr2
1182	Lefty1	0.877	2.837	2.487	0.747	0.145	0.432	1.723	1.533	3.037	13590	Lefty1	chr1	+	NM_0100	P4087	protein_cc	AI450052|	left right c	Lefty1
1183	Tmem63a	0.596	1.854	1.105	0.034	0.013	0.441	8.145	7.398	8.289	208795	Tmem63a	chr1	+	NM_1447	P394	protein_cc	BC014795	transmem	Tmem63a
1184	Ephx1	0.273	1.395	0.381	0.000	0.000	0.001	10.955	9.080	9.561	13849	Ephx1	chr1	-	NM_0101	P4668	protein_cc	EH| Eph-1	epoxide h	Ephx1
1185	9130409I2	0.102	1.269	0.130	0.036	0.664	0.206	4.500	1.209	1.552	619326	9130409I2	chr1	+	NM_0010	P16353	protein_cc	.	RIKEN cDN	9130409I2
1186	Nvl	1.502	0.548	0.823	0.023	0.003	0.398	6.276	6.862	5.994	67459	Nvl	chr1	-	NM_0261	P12279	protein_cc	1200009I2	nuclear VC	Nvl
1187	Cnih4	1.490	0.992	1.477	0.000	0.487	0.004	8.410	8.985	8.973	98417	Cnih4	chr1	+	NM_0301	P13730	protein_cc	AI647760|	cornichon	Cnih4
1188	Wdr26	1.234	1.091	1.346	0.041	0.488	0.231	10.775	11.078	11.203	226757	Wdr26	chr1	-	NM_1455	P21952	protein_cc	1600024A	WD repea	Wdr26
1189	Cnih3	1.000	1.000	1.000	1.000	1.000	1.000	0.000	0.000	0.000	72978	Cnih3	chr1	+	NM_0284	P22212	protein_cc	2900075G	cornichon	Cnih3
1190	Ccdc121	0.716	0.995	0.712	0.182	1.000	0.423	0.490	0.008	0.000	403180	Ccdc121	chr1	-	NM_2072	P1063	protein_cc	6530421E	coiled-coil	Ccdc121
1191	Lbr	2.469	1.005	2.481	0.000	0.975	0.129	7.470	8.774	8.781	98386	Lbr	chr1	-	NM_1338	P14318	protein_cc	AI505894|	lamin B re	Lbr
1192	Enah	1.294	0.430	0.557	0.192	0.018	0.265	4.745	5.117	3.899	13800	Enah	chr1	-	NM_0086	P26823	protein_cc	Mena| NE	enabled h	Enah
1193	Srp9	1.592	1.064	1.694	0.037	0.754	0.238	9.685	10.356	10.446	27058	Srp9	chr1	+	NM_0120	P17104	protein_cc	9kDa	signal rec	Srp9
1194	Vmn1r1	1.000	1.000	1.000	1.000	1.000	1.000	0.000	0.000	0.000	625823	Vmn1r1	chr1	-	NM_0011	P19668	protein_cc	EG625823	vomerona	Vmn1r1
1195	Degs1	1.246	1.098	1.368	0.116	0.376	0.043	9.908	10.225	10.360	13244	Degs1	chr1	-	NM_0078	P16572	protein_cc	AA536663	delta(4)-de	Degs1
1196	1700047M	0.319	2.506	0.800	0.009	0.031	0.611	1.701	0.053	1.378	67330	1700047M	chr1	+	NR_01545	.	lncRNA	.	RIKEN cDN	1700047M
1197	Fbxo28	2.089	0.786	1.643	0.004	0.102	0.068	6.636	7.699	7.352	67948	Fbxo28	chr1	-	NM_1751	P19178	protein_cc	4833428J1	F-box prot	Fbxo28
1198	Trp53bp2	0.932	0.716	0.668	0.644	0.042	0.255	6.039	5.938	5.456	209456	Trp53bp2	chr1	+	NM_1733	P332	protein_cc	53BP2| AI	transform	Trp53bp2
1199	Capn2	0.606	1.781	1.079	0.034	0.000	0.732	8.517	7.795	8.627	12334	Capn2	chr1	-	NM_0097	P21094	protein_cc	AI326419|	calpain 2	Capn2
1200	Capn8	1.309	6.634	8.681	0.521	0.022	0.143	0.373	0.761	3.491	170725	Capn8	chr1	+	NM_0011	P12650	protein_cc	nCL-2| nC	calpain 8	Capn8
1201	Ccdc185	1.000	1.000	1.000	1.000	1.000	1.000	0.000	0.000	0.000	433386	Ccdc185	chr1	-	NM_0010	P9678	protein_cc	4922505E	coiled-coil	Ccdc185
1202	Susd4	0.432	4.171	1.800	0.041	0.135	0.572	1.953	0.741	2.801	96935	Susd4	chr1	+	NM_1447	P1828	protein_cc	AI848994|	sushi dom	Susd4
1203	Tlr5	0.250	2.741	0.684	0.018	0.341	0.673	3.802	1.799	3.254	53791	Tlr5	chr1	+	NM_0169	P11526	protein_cc	.	toll-like re	Tlr5
1204	Disp1	0.928	0.648	0.601	0.765	0.231	0.051	4.950	4.841	4.216	68897	Disp1	chr1	-	NM_0268	P9973	protein_cc	1190008H	dispatche	Disp1
1205	Brox	1.155	1.025	1.183	0.444	0.909	0.528	8.083	8.290	8.325	71678	Brox	chr1	-	NM_0278	P7586	protein_cc	0610010K	BRO1 dom	Brox
1206	Aida	2.078	0.796	1.655	0.020	0.154	0.258	6.503	7.558	7.229	108909	Aida	chr1	+	NM_1817	P17379	protein_cc	2610208M	axin intera	Aida
1207	Mia3	0.485	0.752	0.365	0.002	0.074	0.040	11.414	10.372	9.961	338366	Mia3	chr1	-	NM_1773	P2193	protein_cc	9130229H	melanoma	Mia3
1208	Taf1a	1.623	0.893	1.449	0.024	0.395	0.065	5.056	5.755	5.591	21339	Taf1a	chr1	+	NM_0214	P25160	protein_cc	mTAFI48|	TATA-box	Taf1a
1209	Hhipl2	2.799	1.215	3.401	0.019	0.610	0.273	0.001	1.486	1.767	78772	Hhipl2	chr1	+	NM_0301	P19292	protein_cc	4930507C	hedgehog	Hhipl2
1210	1700056E	1.012	1.813	1.834	0.977	0.324	0.524	4.144	4.161	5.019	73363	1700056E	chr1	-	NM_0285	P5051	protein_cc	.	RIKEN cDN	1700056E
1211	Dusp10	2.846	0.749	2.132	0.007	0.404	0.454	4.041	5.550	5.133	63953	Dusp10	chr1	+	NM_0220	P20955	protein_cc	2610306G	dual speci	Dusp10
1212	1700112H	1.000	1.000	1.000	1.000	1.000	1.000	0.000	0.000	0.000	73590	1700112H	chr1	-	NR_04047	.	lncRNA	.	RIKEN cDN	1700112H
1213	4930488B	1.000	1.000	1.000	1.000	1.000	1.000	0.000	0.000	0.000	74971	4930488B	chr1	+	NR_04062	.	lncRNA	.	RIKEN cDN	4930488B
1214	Hlx	0.564	1.506	0.849	0.012	0.181	0.641	5.650	4.823	5.413	15284	Hlx	chr1	-	NM_0082	P12897	protein_cc	Hlx1	H2.0-like	Hlx
1215	Marc1	0.076	2.345	0.178	0.000	0.024	0.003	11.622	7.904	9.133	66112	Marc1	chr1	-	NM_0012	P9371	protein_cc	1300013F	mitochond	Marc1
1216	Marc2	0.262	1.156	0.303	0.000	0.000	0.000	13.131	11.200	11.409	67247	Marc2	chr1	-	NM_1336	P22340	protein_cc	2810484M	mitochond	Marc2
1217	C130074G	0.183	1.879	0.344	0.000	0.032	0.018	10.342	7.893	8.803	226777	C130074G	chr1	-	NM_1786	P9306	protein_cc	.	RIKEN cDN	C130074G
1218	Mark1	1.097	0.980	1.075	0.711	0.960	0.906	3.510	3.645	3.615	226778	Mark1	chr1	-	NM_1455	P22145	protein_cc	AW49115(	MAP/micr	Mark1
1219	Rab3gap2	0.494	1.885	0.931	0.004	0.012	0.722	8.770	7.753	8.667	98732	Rab3gap2	chr1	+	NM_0011	P1098	protein_cc	1110059F	RAB3 GTP	Rab3gap2
1220	Snora36b	1.005	1.405	1.412	0.000	0.182	0.423	0.000	0.008	0.498	10021744	Snora36b	chr1	+	NR_03404	.	snoRNA	MBI-87	small nucl	Snora36b
1221	Iars2	0.584	1.736	1.014	0.013	0.008	0.882	9.021	8.245	9.041	381314	Iars2	chr1	-	NM_1986	P17462	protein_cc	2010002H	isoleucine	Iars2
1222	Bpnt1	0.637	1.245	0.793	0.119	0.443	0.176	8.801	8.150	8.466	23827	Bpnt1	chr1	+	NM_0117	P8759	protein_cc	BPntase	bisphosph	Bpnt1
1223	Eprs	1.228	0.585	0.719	0.049	0.004	0.021	10.738	11.035	10.262	107508	Eprs	chr1	+	NM_0297	P11739	protein_cc	2410081F	glutamyl-p	Eprs
1224	9630028B	0.504	0.990	0.499	0.181	1.000	0.423	1.002	0.015	0.000	319562	9630028B	chr1	-	NR_03800	.	lncRNA	.	RIKEN cDN	9630028B
1225	Gm2061	2.269	0.486	1.104	0.021	0.026	0.598	4.006	5.188	4.148	10003913	Gm2061	chr1	-	NR_04030	.	lncRNA	10003913	predicted	Gm2061
1226	Slc30a10	3.206	0.508	1.630	0.000	0.003	0.262	7.993	9.674	8.698	226781	Slc30a10	chr1	+	NM_0010	P4502	protein_cc	E130106K	solute car	Slc30a10
1227	5033404E	1.000	1.000	1.000	1.000	1.000	1.000	0.000	0.000	0.000	114668	5033404E	chr1	+	NR_03360	.	misc_RNA	.	RIKEN cDN	5033404E
1228	Lyplal1	0.433	1.018	0.440	0.000	0.697	0.007	7.688	6.480	6.505	226791	Lyplal1	chr1	-	NM_1461	P7404	protein_cc	BC027340	lysophosp	Lyplal1
1229	A730004F	1.000	1.000	1.000	1.000	1.000	1.000	0.000	0.000	0.000	320449	A730004F	chr1	-	NR_13115	.	lncRNA	.	RIKEN cDN	A730004F
1230	Tgfb2	1.308	0.440	0.575	0.458	0.003	0.556	3.314	3.701	2.516	21808	Tgfb2	chr1	-	NM_0093	P20330	protein_cc	BB105277	transform	Tgfb2
1231	Rrp15	2.823	1.063	3.001	0.002	0.799	0.203	6.183	7.680	7.769	67223	Rrp15	chr1	-	NM_0260	P19628	protein_cc	2810430M	ribosomal	Rrp15
1232	D1Pas1	1.572	0.636	1.000	0.304	1.000	0.423	0.000	0.653	0.000	110957	D1Pas1	chr1	+	NM_0330	P21228	protein_cc	Pl10	DNA segm	D1Pas1
1233	Spata17	0.429	1.357	0.582	0.025	0.196	0.312	1.248	0.027	0.467	74717	Spata17	chr1	-	NM_0288	P17093	protein_cc	1700065F	spermatog	Spata17
1234	1700007P	1.000	1.000	1.000	1.000	1.000	1.000	0.000	0.000	0.000	69308	1700007P	chr1	+	NR_04055	.	lncRNA	.	RIKEN cDN	1700007P
1235	Gpatch2	0.930	0.894	0.831	0.100	0.016	0.128	6.471	6.366	6.204	67769	Gpatch2	chr1	+	NM_0263	P5612	protein_cc	5830433G	G patch do	Gpatch2
1236	Esrrg	0.080	0.976	0.078	0.000	0.954	0.016	4.429	0.777	0.742	26381	Esrrg	chr1	+	NM_0012	P5224	protein_cc	ERR3| Errg	estrogen-r	Esrrg
1237	9330162B	1.000	1.000	1.000	1.000	1.000	1.000	0.000	0.000	0.000	320272	9330162B	chr1	-	NR_03800	.	lncRNA	.	RIKEN cDN	9330162B
1238	Ush2a	1.000	1.000	1.000	1.000	1.000	1.000	0.000	0.000	0.000	22283	Ush2a	chr1	+	NM_0214	P24622	protein_cc	A930011D	Usher syn	Ush2a
1239	Kctd3	3.385	0.724	2.451	0.002	0.055	0.005	5.321	7.080	6.615	226823	Kctd3	chr1	-	NM_1726	P25000	protein_cc	4930438A	potassium	Kctd3
1240	Kcnk2	0.264	0.971	0.256	0.181	1.000	0.423	1.965	0.043	0.000	16526	Kcnk2	chr1	-	NM_0106	P21999	protein_cc	A430027H	potassium	Kcnk2
1241	Cenpf	1.353	2.202	2.978	0.494	0.018	0.116	2.259	2.695	3.834	108000	Cenpf	chr1	-	NM_0010	P25186	protein_cc	6530404A	centrome	Cenpf
1242	Ptpn14	0.554	1.111	0.616	0.019	0.764	0.350	4.813	3.962	4.113	19250	Ptpn14	chr1	+	NM_0089	P18110	protein_cc	C130080N	protein ty	Ptpn14
1243	Smyd2	0.530	1.952	1.035	0.127	0.080	0.945	6.520	5.604	6.569	226830	Smyd2	chr1	-	NM_0267	P3281	protein_cc	1110020E	SET and M	Smyd2
1244	Prox1	0.947	1.178	1.116	0.303	0.016	0.040	7.517	7.439	7.675	19130	Prox1	chr1	-	NM_0089	P2108	protein_cc	A230003G	prospero	Prox1
1245	Rps6kc1	1.260	0.878	1.106	0.192	0.422	0.695	6.209	6.542	6.354	320119	Rps6kc1	chr1	-	NM_1787	P10865	protein_cc	AA682037	ribosomal	Rps6kc1
1246	Angel2	1.236	0.754	0.932	0.012	0.013	0.629	7.326	7.631	7.225	52477	Angel2	chr1	+	NM_0011	P8452	protein_cc	2610307I2	angel hom	Angel2
1247	Vash2	1.023	3.048	3.117	0.000	0.001	0.028	0.000	0.033	1.641	226841	Vash2	chr1	-	NM_1448	P25458	protein_cc	B130052G	vasohibin	Vash2
1248	Mfsd7b	7.368	0.542	3.997	0.002	0.019	0.005	4.455	7.337	6.454	226844	Mfsd7b	chr1	-	NM_0010	P16999	protein_cc	9630055N	major faci	Mfsd7b
1249	A230020J	1.000	1.000	1.000	1.000	1.000	1.000	0.000	0.000	0.000	10012533	A230020J	chr1	+	NR_02729	.	lncRNA	ENSMUSG	RIKEN cDN	A230020J
1250	Spata45	1.000	1.000	1.000	1.000	1.000	1.000	0.000	0.000	0.000	75558	Spata45	chr1	+	NM_0293	P6142	protein_cc	1700022P	spermatog	Spata45
1251	Tatdn3	0.879	1.949	1.713	0.371	0.071	0.319	5.038	4.852	5.815	68972	Tatdn3	chr1	-	NM_0011	P24742	protein_cc	1500010M	TatD DNas	Tatdn3
1252	Nsl1	0.671	1.142	0.766	0.486	0.756	0.808	1.744	1.169	1.360	381318	Nsl1	chr1	+	NM_1986	P8590	protein_cc	4833432M	NSL1, MIS	Nsl1
1253	Batf3	7.846	0.135	1.060	0.047	0.048	0.877	3.076	6.048	3.161	381319	Batf3	chr1	+	NM_0300	P12691	protein_cc	9130211I(	basic leuci	Batf3
1254	Fam71a	1.000	1.000	1.000	1.000	1.000	1.000	0.000	0.000	0.000	619288	Fam71a	chr1	-	NM_0011	P21334	protein_cc	4933417M	family wit	Fam71a
1255	Atf3	15.483	0.186	2.879	0.000	0.001	0.381	3.552	7.505	5.078	11910	Atf3	chr1	-	NM_0074	P11345	protein_cc	LRG-21	activating	Atf3
1256	Nenf	0.948	1.330	1.262	0.866	0.291	0.354	6.108	6.031	6.443	66208	Nenf	chr1	-	NM_0254	P5074	protein_cc	1110060M	neuron de	Nenf
1257	Tmem206	0.305	1.118	0.341	0.001	0.263	0.036	9.624	7.910	8.071	66950	Tmem206	chr1	+	NM_0258	P6537	protein_cc	2310028N	transmem	Tmem206
1258	Ppp2r5a	0.300	1.214	0.365	0.002	0.075	0.059	9.823	8.087	8.367	226849	Ppp2r5a	chr1	-	NM_1448	P15345	protein_cc	PR61alpha	protein ph	Ppp2r5a
1259	Dtl	3.103	1.493	4.631	0.008	0.331	0.237	0.377	2.011	2.589	76843	Dtl	chr1	-	NM_0013	P17173	protein_cc	2810047L(	denticlele	Dtl
1260	Ints7	1.923	1.083	2.084	0.000	0.251	0.034	6.757	7.701	7.817	77065	Ints7	chr1	+	NM_0013	P23551	protein_cc	5930412E	integrator	Ints7
1261	Lpgat1	4.097	0.618	2.531	0.000	0.000	0.005	10.912	12.947	12.252	226856	Lpgat1	chr1	+	NM_1722	P9965	protein_cc	AI649174|	lysophosp	Lpgat1

1262	Nek2	3.532	0.661	2.336	0.006	0.013	0.235	1.811	3.631	3.035	18005	Nek2	chr1	+	NM_0108	P11966	protein_cc	AA617254	NIMA (ne	Nek2
1263	1700034H	0.441	0.829	0.366	0.351	0.754	0.519	2.326	1.146	0.876	98736	1700034H	chr1	-	NR_03066	.	lncRNA	4930557M	RIKEN cDN	1700034H
1264	Slc30a1	0.619	1.929	1.194	0.002	0.015	0.509	9.058	8.366	9.314	22782	Slc30a1	chr1	+	NM_0095	P20312	protein_cc	AI839647 |	solute car	Slc30a1
1265	Rd3	0.223	3.047	0.679	0.000	0.011	0.200	3.718	1.552	3.159	74023	Rd3	chr1	+	NM_0011	P21507	protein_cc	3322402L(	retinal deg	Rd3
1266	Traf5	0.373	1.563	0.584	0.078	0.316	0.370	5.066	3.644	4.289	22033	Traf5	chr1	-	NM_0116	P22131	protein_cc	.	TNF recep	Traf5
1267	Rcor3	0.767	1.067	0.818	0.213	0.252	0.551	6.405	6.022	6.116	214742	Rcor3	chr1	-	NM_1448	P21308	protein_cc	4921514E	REST core	Rcor3
1268	Gm10516	0.245	0.622	0.153	0.002	0.333	0.011	4.589	2.563	1.877	10003871	Gm10516	chr1	+	NR_03353	.	lncRNA	ENSMUSG	predicted	Gm10516
1269	Kcnh1	1.000	1.000	1.000	1.000	1.000	1.000	0.000	0.000	0.000	16510	Kcnh1	chr1	+	NM_0106	P10278	protein_cc	EAG1| Kv:	potassium	Kcnh1
1270	Hhat	0.390	1.748	0.682	0.183	0.190	0.690	1.395	0.038	0.843	226861	Hhat	chr1	-	NM_1448	P24845	protein_cc	2810432O	hedgehog	Hhat
1271	1700065J1	1.000	1.000	1.000	1.000	1.000	1.000	0.000	0.000	0.000	73455	1700065J1	chr1	+	NR_04046	.	lncRNA	.	RIKEN cDN	1700065J1
1272	Sertad4	1.070	0.687	0.735	0.898	0.608	0.666	2.274	2.371	1.829	214791	Sertad4	chr1	-	NM_1982	P15997	protein_cc	4833404A	SERTA dor	Sertad4
1273	Gm15867	0.735	1.867	1.372	0.191	0.184	0.650	0.465	0.021	0.922	10050347	Gm15867	chr1	+	NR_13174	.	lncRNA	OTTMUSG	predicted	Gm15867
1274	Syt14	2.170	0.461	1.000	0.000	1.000	0.423	0.000	1.118	0.000	329324	Syt14	chr1	-	NM_1815	P23138	protein_cc	B230320I(	synaptota	Syt14
1275	Diexf	0.986	0.722	0.712	0.664	0.005	0.060	6.299	6.278	5.809	215193	Diexf	chr1	-	NM_1454	P7007	protein_cc	AA408296	digestive	Diexf
1276	Irf6	0.372	2.759	1.027	0.013	0.002	0.933	7.491	6.065	7.529	54139	Irf6	chr1	+	NM_0168	P8828	protein_cc	AI876454 |	interferon	Irf6
1277	A130010J1	0.797	1.284	1.023	0.020	0.068	0.895	6.853	6.526	6.886	319266	A130010J1	chr1	+	NM_0011	P12245	protein_cc	.	RIKEN cDN	A130010J1
1278	Traf3ip3	0.654	1.459	0.955	0.327	0.418	0.842	4.025	3.413	3.958	215243	Traf3ip3	chr1	-	NM_1531	P7431	protein_cc	6030423D	TRAF3 inte	Traf3ip3
1279	Hsd11b1	0.393	0.726	0.285	0.000	0.054	0.009	12.621	11.272	10.811	15483	Hsd11b1	chr1	-	NM_0010	P5300	protein_cc	.	hydroxyst	Hsd11b1
1280	G0s2	0.116	5.468	0.633	0.000	0.000	0.064	9.660	6.549	9.000	14373	G0s2	chr1	-	NM_0080	P13780	protein_cc	AI255151 |	G0/G1 sw	G0s2
1281	Lamb3	0.245	2.966	0.728	0.014	0.048	0.099	4.582	2.556	4.124	16780	Lamb3	chr1	+	NM_0084	P21283	protein_cc	.	laminin, b	Lamb3
1282	Camk1g	2.170	1.210	2.626	0.299	0.761	0.423	0.001	1.118	1.393	215303	Camk1g	chr1	-	NM_1448	P25704	protein_cc	CaMKIgan	calcium/ca	Camk1g
1283	4930503O	1.000	1.000	1.000	1.000	1.000	1.000	0.000	0.000	0.000	74942	4930503O	chr1	-	NR_04047	.	lncRNA	.	RIKEN cDN	4930503O
1284	Plxna2	0.783	0.605	0.474	0.044	0.002	0.039	8.410	8.058	7.333	18845	Plxna2	chr1	+	NM_0088	P9950	protein_cc	2810428A	plexin A2	Plxna2
1285	Cd34	0.667	6.808	4.543	0.166	0.003	0.075	4.609	4.026	6.793	12490	Cd34	chr1	+	NM_0011	P21962	protein_cc	AU04096C	CD34 anti	Cd34
1286	Gm16897	1.014	2.240	2.272	0.000	0.182	0.423	0.000	0.021	1.185	320400	Gm16897	chr1	-	NR_03356	.	lncRNA	C030002c	predicted	Gm16897
1287	A330023F	1.557	0.890	1.386	0.128	0.730	0.591	4.269	4.907	4.739	320977	A330023F	chr1	+	NR_01556	.	lncRNA	C030002C	RIKEN cDN	A330023F
1288	Cd46	0.640	1.144	0.732	0.457	0.753	0.781	1.811	1.167	1.362	17221	Cd46	chr1	-	NM_0107	P14092	protein_cc	Mcp	CD46 anti	Cd46
1289	Cr1l	1.375	1.084	1.490	0.190	0.672	0.083	8.363	8.823	8.939	12946	Cr1l	chr1	-	NM_0134	P8503	protein_cc	Crry| Mcp	compleme	Cr1l
1290	Cr2	0.624	0.712	0.444	0.348	0.585	0.408	3.734	3.054	2.563	12902	Cr2	chr1	-	NM_0077	P993	protein_cc	C3DR| CD	compleme	Cr2
1291	B020014A	1.000	1.000	1.000	1.000	1.000	1.000	0.000	0.000	0.000	619314	B020014A	chr10	-	NR_04594	.	lncRNA	.	RIKEN cDN	B020014A
1292	H60c	1.000	1.000	1.000	1.000	1.000	1.000	0.000	0.000	0.000	670558	H60c	chr10	-	NM_0012	P1091	protein_cc	4632413I2	histocomp	H60c
1293	Ppp1r14c	0.716	0.995	0.712	0.182	1.000	0.423	0.490	0.008	0.000	76142	Ppp1r14c	chr10	+	NM_1334	P3111	protein_cc	6330514J(	protein ph	Ppp1r14c
1294	Iyd	0.334	1.133	0.378	0.000	0.240	0.007	10.620	9.037	9.216	70337	Iyd	chr10	+	NM_0273	P19510	protein_cc	0610009A	iodotyrosi	Iyd
1295	Plekhg1	1.576	0.551	0.868	0.021	0.002	0.700	5.467	6.124	5.263	213783	Plekhg1	chr10	+	NM_0011	P26697	protein_cc	D10Ertd7	pleckstrin	Plekhg1
1296	Mthfd1l	7.628	1.154	8.803	0.000	0.287	0.030	4.023	6.954	7.161	270685	Mthfd1l	chr10	+	NM_0011	P14542	protein_cc	2410004L	methylene	Mthfd1l
1297	Akap12	29.102	0.671	19.516	0.000	0.002	0.001	6.546	11.409	10.833	83397	Akap12	chr10	+	NM_0311	P2226	protein_cc	AI317366 |	A kinase (	Akap12
1298	Zbtb2	0.805	1.258	1.013	0.349	0.428	0.966	5.513	5.200	5.532	381990	Zbtb2	chr10	-	NM_0010	P10676	protein_cc	Gm1103	zinc finger	Zbtb2
1299	Rmnd1	0.518	0.792	0.411	0.000	0.127	0.023	6.573	5.625	5.289	66084	Rmnd1	chr10	-	NM_0253	P22875	protein_cc	0610042C	required f	Rmnd1
1300	Gm5512	0.511	0.799	0.408	0.000	0.130	0.022	6.610	5.643	5.318	433224	Gm5512	chr10	-	NR_00289	.	misc_RNA	EG433224	predicted	Gm5512
1301	Armt1	1.334	0.582	0.777	0.222	0.042	0.516	6.623	7.039	6.258	73419	Armt1	chr10	+	NM_0242	P26136	protein_cc	1700052N	acidic resi	Armt1
1302	Ccdc170	1.000	1.000	1.000	1.000	1.000	1.000	0.000	0.000	0.000	10050423	Ccdc170	chr10	+	NM_0011	P3221	protein_cc	Gm221	coiled-coil	Ccdc170
1303	Esr1	0.390	1.262	0.492	0.000	0.340	0.073	8.159	6.800	7.136	13982	Esr1	chr10	+	NM_0079	P7636	protein_cc	ER| ER-alp	estrogen r	Esr1
1304	Syne1	0.129	1.063	0.137	0.000	0.804	0.004	8.829	5.871	5.959	64009	Syne1	chr10	-	NM_0010	P10262	protein_cc	8B| A330(	spectrin re	Syne1
1305	Myct1	0.642	1.333	0.856	0.105	0.076	0.685	5.328	4.689	5.104	68632	Myct1	chr10	+	NM_0267	P8691	protein_cc	1110020B	myc targe	Myct1
1306	Vip	1.000	1.000	1.000	1.000	1.000	1.000	0.000	0.000	0.000	22353	Vip	chr10	+	NM_0117	P23362	protein_cc	.	vasoactive	Vip
1307	Fbxo5	0.669	0.379	0.254	0.498	0.321	0.219	3.827	3.246	1.847	67141	Fbxo5	chr10	-	NM_0259	P22139	protein_cc	2510044I1	F-box prot	Fbxo5
1308	Mtrf1l	1.316	0.669	0.880	0.126	0.020	0.630	6.357	6.754	6.174	108853	Mtrf1l	chr10	-	NM_1753	P13517	protein_cc	9130004K	mitochon	Mtrf1l
1309	Rgs17	0.442	1.555	0.687	0.092	0.262	0.516	1.900	0.721	1.358	56533	Rgs17	chr10	-	NM_0199	P8169	protein_cc	6430507P	regulator	Rgs17
1310	Oprm1	0.658	0.455	0.299	0.469	1.000	0.296	1.742	1.137	0.000	18390	Oprm1	chr10	+	NM_0013	P243	protein_cc	M-OR-1| N	opioid rec	Oprm1
1311	Ipcef1	0.489	0.352	0.172	0.005	1.000	0.002	2.541	1.508	0.000	320495	Ipcef1	chr10	-	NM_0011	P12502	protein_cc	A130090K	interactio	Ipcef1
1312	Cnksr3	0.828	1.054	0.872	0.326	0.549	0.687	5.932	5.659	5.735	215748	Cnksr3	chr10	-	NM_1725	P15166	protein_cc	6820402C	Cnksr fam	Cnksr3
1313	Ulbp1	1.000	1.000	1.000	1.000	1.000	1.000	0.000	0.000	0.000	77777	Ulbp1	chr10	-	NM_0299	P14495	protein_cc	A430108B	UL16 bind	Ulbp1
1314	Lrp11	0.806	0.987	0.796	0.629	0.960	0.775	3.811	3.500	3.481	237253	Lrp11	chr10	+	NM_1727	P6517	protein_cc	1700034J1	low densit	Lrp11
1315	Pcmt1	0.846	0.916	0.775	0.144	0.278	0.309	7.697	7.455	7.328	18537	Pcmt1	chr10	-	NM_0087	P11880	protein_cc	C79501| F	protein-L-	Pcmt1
1316	A630066F	3.696	1.202	4.442	0.002	0.633	0.269	2.578	4.464	4.729	320642	A630066F	chr10	+	NR_03069	.	lncRNA	.	RIKEN cDN	A630066F
1317	Nup43	1.249	0.699	0.873	0.320	0.035	0.714	4.985	5.306	4.789	69912	Nup43	chr10	+	NM_1457	P23758	protein_cc	2610016K	nucleopor	Nup43
1318	BC020402	0.616	3.689	2.273	0.412	0.122	0.416	2.741	2.043	3.926	407824	BC020402	chr10	-	NR_03321	.	lncRNA	.	cDNA seq	BC020402
1319	Lats1	1.545	0.981	1.515	0.070	0.874	0.216	6.893	7.521	7.493	16798	Lats1	chr10	+	NM_0106	P13479	protein_cc	AW20859	large tum	Lats1
1320	Katna1	1.612	0.514	0.829	0.009	0.000	0.448	7.913	8.602	7.642	23924	Katna1	chr10	+	NM_0118	P8464	protein_cc	.	katanin p6	Katna1
1321	Ginm1	1.212	1.289	1.563	0.254	0.081	0.096	8.160	8.438	8.804	215751	Ginm1	chr10	-	NM_1454	P14036	protein_cc	AA589616	glycoprote	Ginm1
1322	Ppil4	1.296	0.798	1.034	0.187	0.111	0.892	8.082	8.456	8.130	67418	Ppil4	chr10	+	NM_0261	P2634	protein_cc	3732410E	peptidylpr	Ppil4
1323	Zc3h12d	0.522	2.010	1.048	0.150	0.302	0.948	4.836	3.897	4.904	237256	Zc3h12d	chr10	+	NM_1727	P7544	protein_cc	D730019B	zinc finger	Zc3h12d
1324	Tab2	0.986	1.086	1.071	0.751	0.504	0.737	9.898	9.877	9.996	68652	Tab2	chr10	+	NM_1386	P11354	protein_cc	1110030N	TGF-beta a	Tab2
1325	Ust	1.478	1.398	2.066	0.408	0.223	0.366	1.983	2.546	3.029	338362	Ust	chr10	-	NM_1773	P23061	protein_cc	D930010C	uronyl-2-s	Ust
1326	Sash1	0.964	1.250	1.205	0.155	0.105	0.395	8.338	8.285	8.607	70097	Sash1	chr10	-	NM_1751	P17628	protein_cc	1100001C	SAM and S	Sash1
1327	Samd5	0.858	0.991	0.850	0.805	0.988	0.862	1.353	1.132	1.119	320825	Samd5	chr10	-	NM_1772	P18361	protein_cc	E130306M	sterile alp	Samd5
1328	Gm6150	1.000	1.000	1.000	1.000	1.000	1.000	0.000	0.000	0.000	620417	Gm6150	chr10	+	NR_03803	.	lncRNA	620417	predicted	Gm6150
1329	Stxbp5	1.790	0.973	1.743	0.021	0.836	0.001	6.524	7.365	7.326	78808	Stxbp5	chr10	-	NM_0010	P4872	protein_cc	0710001E	syntaxin b	Stxbp5
1330	Adgb	0.954	0.997	0.952	0.807	0.996	0.947	3.481	3.413	3.410	215772	Adgb	chr10	-	NM_0011	P12982	protein_cc	9130014G	androglob	Adgb
1331	Rab32	0.678	1.072	0.727	0.002	0.606	0.202	8.899	8.338	8.439	67844	Rab32	chr10	-	NM_0264	P9380	protein_cc	2810011A	RAB32, me	Rab32
1332	Gm5177	0.338	2.900	0.981	0.036	0.055	0.975	1.619	0.056	1.592	382450	Gm5177	chr10	+	NR_03363	.	misc_RNA	EG382450	predicted	Gm5177
1333	Grm1	1.005	1.405	1.412	0.000	0.182	0.423	0.000	0.008	0.498	14816	Grm1	chr10	-	NM_0169	P9234	protein_cc	.	glutamate	Grm1
1334	4930567K	1.000	1.000	1.000	1.000	1.000	1.000	0.000	0.000	0.000	78056	4930567K	chr10	+	NR_04601	.	lncRNA	1700038H	RIKEN cDN	4930567K
1335	Shprh	0.829	0.630	0.522	0.532	0.081	0.319	6.254	5.984	5.317	268281	Shprh	chr10	+	NM_1729	P17580	protein_cc	2610103K	SNF2 histo	Shprh
1336	Fbxo30	1.712	0.653	1.119	0.017	0.042	0.479	6.298	7.074	6.460	71865	Fbxo30	chr10	+	NM_0279	P14675	protein_cc	1700026A	F-box prot	Fbxo30
1337	Epm2a	1.000	1.000	1.000	1.000	1.000	1.000	0.000	0.000	0.000	13853	Epm2a	chr10	+	NM_0101	P7183	protein_cc	TcrbK)TG-	epilepsy, p	Epm2a
1338	Utrn	0.628	0.810	0.509	0.000	0.180	0.052	8.454	7.783	7.480	22288	Utrn	chr10	-	NM_0116	P263	protein_cc	AA589569	utrophin	Utrn
1339	B230208H	1.318	1.973	2.601	0.469	0.210	0.395	3.563	3.961	4.942	320273	B230208H	chr10	-	NR_03802	.	lncRNA	.	RIKEN cDN	B230208H
1340	Stx11	11.663	0.324	3.776	0.001	0.003	0.083	2.882	6.426	4.799	74732	Stx11	chr10	-	NM_0290	P8165	protein_cc	5830405C	syntaxin 1	Stx11
1341	Sf3b5	1.292	1.253	1.619	0.051	0.005	0.065	8.667	9.037	9.362	66125	Sf3b5	chr10	+	NM_1751	P25369	protein_cc	10kDa| 11	splicing fa	Sf3b5
1342	Plagl1	4.853	0.512	2.487	0.008	0.044	0.060	3.883	6.162	5.198	22634	Plagl1	chr10	+	NM_0095	P24132	protein_cc	Lot1| Zac1	pleiomorp	Plagl1
1343	Hymai	1.000	1.000	1.000	1.000	1.000	1.000	0.000	0.000	0.000	338438	Hymai	chr10	+	NR_13191	.	lncRNA	.	hydatidifo	Hymai
1344	Zc2hc1b	1.000	1.000	1.000	1.000	1.000	1.000	0.000	0.000	0.000	75122	Zc2hc1b	chr10	-	NM_0291	P6124	protein_cc	4930519B	zinc finger	Zc2hc1b
1345	Ltv1	2.443	0.665	1.624	0.009	0.055	0.024	7.319	8.608	8.019	353258	Ltv1	chr10	-	NM_1814	P21500	protein_cc	2610020N	LTV1 ribos	Ltv1
1346	Phactr2	0.577	1.935	1.116	0.045	0.001	0.655	6.600	5.806	6.759	215789	Phactr2	chr10	-	NM_0010	P21664	protein_cc	AV158170	phosphata	Phactr2
1347	Fuca2	0.848	1.436	1.217	0.041	0.025	0.369	6.106	5.868	6.389	66848	Fuca2	chr10	+	NM_0257	P13813	protein_cc	0610025O	fucosidase	Fuca2
1348	Pex3	0.893	1.421	1.269	0.544	0.030	0.443	8.346	8.182	8.689	56535	Pex3	chr10	-	NM_0011	P25344	protein_cc	1700014F	peroxisom	Pex3
1349	Adat2	1.262	1.105	1.394	0.011	0.277	0.138	6.834	7.170	7.313	66757	Adat2	chr10	+	NM_0257	P1722	protein_cc	4933426M	adenosine	Adat2
1350	Aig1	1.558	0.968	1.508	0.059	0.821	0.076	5.199	5.838	5.792	66253	Aig1	chr10	-	NM_0254	P13381	protein_cc	1500031O	androgen-	Aig1
1351	Hivep2	1.854	0.730	1.352	0.017	0.001	0.412	7.042	7.932	7.478	15273	Hivep2	chr10	+	NM_0104	P5897	protein_cc	Gm20114	human im	Hivep2
1352	Adgrg6	0.177	1.934	0.342	0.002	0.332	0.122	3.691	1.194	2.145	215798	Adgrg6	chr10	-	NM_0010	P21635	protein_cc	1190004A	adhesion G	Adgrg6

1353	Vta1	1.773	0.977	1.731	0.008	0.838	0.049	6.785	7.611	7.577	66201	Vta1	chr10	-	NM_0254	P2421	protein_cc	1110001D	vesicle (m	Vta1
1354	1700016L(	1.000	1.000	1.000	1.000	1.000	1.000	0.000	0.000	0.000	69412	1700016L(	chr10	+	NR_04582	.	lncRNA	.	RIKEN cDN	1700016L(
1355	Gje1	0.856	0.861	0.737	0.623	0.705	0.345	0.899	0.676	0.459	76743	Gje1	chr10	-	NM_0297	P5693	protein_cc	AEY12| Cx	gap juncti	Gje1
1356	Nmbr	1.000	1.000	1.000	1.000	1.000	1.000	0.000	0.000	0.000	18101	Nmbr	chr10	+	NM_0087	P3205	protein_cc	BB182387	neuromed	Nmbr
1357	C330004P	1.000	1.000	1.000	1.000	1.000	1.000	0.000	0.000	0.000	77538	C330004P	chr10	+	NR_13108	.	lncRNA	.	RIKEN cDN	C330004P
1358	Gm20125	1.000	1.000	1.000	1.000	1.000	1.000	0.000	0.000	0.000	10050423	Gm20125	chr10	-	NR_03802	.	lncRNA	.	predicted	Gm20125
1359	Cited2	1.166	0.580	0.677	0.075	0.002	0.005	7.876	8.098	7.312	17684	Cited2	chr10	+	NM_0108	P1138	protein_cc	AI835299|	Cbp/p300	Cited2
1360	Txlnb	1.000	1.000	1.000	1.000	1.000	1.000	0.000	0.000	0.000	378431	Txlnb	chr10	+	NM_1386	P24388	protein_cc	2310001N	taxilin bet	Txlnb
1361	Heca	0.880	1.063	0.935	0.448	0.830	0.860	7.346	7.162	7.249	380629	Heca	chr10	-	NM_0010	P18127	protein_cc	Gm869| H	hdc homo	Heca
1362	Abracl	2.646	0.954	2.524	0.001	0.795	0.138	6.766	8.170	8.102	73112	Abracl	chr10	-	NM_0284	P22850	protein_cc	1700028I(	ABRA C-te	Abracl
1363	Reps1	0.874	0.655	0.572	0.201	0.051	0.081	6.796	6.601	5.990	19707	Reps1	chr10	+	NM_0011	P4243	protein_cc	BB161292	RalBP1 as	Reps1
1364	Ect2l	2.134	0.469	1.000	0.305	1.000	0.423	0.000	1.094	0.000	10004579	Ect2l	chr10	-	NM_0011	P9429	protein_cc	C330021H	epithelial	Ect2l
1365	Ccdc28a	0.422	1.607	0.678	0.001	0.019	0.004	7.211	5.967	6.652	215814	Ccdc28a	chr10	-	NM_1448	P7891	protein_cc	1700009P	coiled-coil	Ccdc28a
1366	Nhsl1	0.953	0.843	0.803	0.798	0.446	0.094	5.213	5.143	4.896	215819	Nhsl1	chr10	+	NM_1733	P24573	protein_cc	5730409E	NHS-like 1	Nhsl1
1367	Hebp2	0.309	5.677	1.755	0.177	0.002	0.420	3.251	1.557	4.062	56016	Hebp2	chr10	-	NM_0194	P21558	protein_cc	SOUL	heme bind	Hebp2
1368	Arfgef3	1.572	0.636	1.000	0.304	1.000	0.423	0.000	0.653	0.000	215821	Arfgef3	chr10	-	NM_0010	P25518	protein_cc	9630004D	ARFGEF fa	Arfgef3
1369	Gm4922	1.000	1.000	1.000	1.000	1.000	1.000	0.000	0.000	0.000	237300	Gm4922	chr10	-	NM_1777	P16586	protein_cc	4933423E	predicted	Gm4922
1370	4930444F(	1.000	1.000	1.000	1.000	1.000	1.000	0.000	0.000	0.000	73968	4930444F(	chr10	-	NR_10272	.	lncRNA	1700010L	RIKEN cDN	4930444F(
1371	Perp	0.275	1.516	0.417	0.004	0.096	0.088	9.665	7.803	8.402	64058	Perp	chr10	+	NM_0220	P11617	protein_cc	1110017A	PERP, TP5	Perp
1372	Tnfaip3	75.834	0.544	41.260	0.001	0.101	0.215	2.684	8.929	8.051	21929	Tnfaip3	chr10	-	NM_0093	P24836	protein_cc	A20| Tnfip	tumor nec	Tnfaip3
1373	Gm20139	1.580	0.633	1.000	0.305	1.000	0.423	0.000	0.660	0.000	10050426	Gm20139	chr10	+	NR_03802	.	lncRNA	.	predicted	Gm20139
1374	Olig3	1.000	1.000	1.000	1.000	1.000	1.000	0.000	0.000	0.000	94222	Olig3	chr10	+	NM_0530	P2172	protein_cc	Bhlhb7| b	oligodend	Olig3
1375	Ifngr1	1.387	0.962	1.334	0.033	0.686	0.055	7.768	8.241	8.185	15979	Ifngr1	chr10	+	NM_0105	P19423	protein_cc	CD119| Ifg	interferon	Ifngr1
1376	Il22ra2	1.000	1.000	1.000	1.000	1.000	1.000	0.000	0.000	0.000	237310	Il22ra2	chr10	+	NM_1782	P16184	protein_cc	CRF2-10|	interleukin	Il22ra2
1377	Il20ra	0.716	0.995	0.712	0.182	1.000	0.423	0.490	0.008	0.000	237313	Il20ra	chr10	+	NM_1727	P9242	protein_cc	E230031K	interleukin	Il20ra
1378	Slc35d3	1.000	1.000	1.000	1.000	1.000	1.000	0.000	0.000	0.000	76157	Slc35d3	chr10	-	NM_0295	P14531	protein_cc	6230421J1	solute car	Slc35d3
1379	Pex7	0.280	1.439	0.402	0.000	0.029	0.011	8.911	7.073	7.598	18634	Pex7	chr10	-	NM_0088	P17741	protein_cc	MmPEX7	peroxisom	Pex7
1380	Map3k5	0.427	0.931	0.398	0.007	0.639	0.063	8.659	7.433	7.330	26408	Map3k5	chr10	+	NM_0085	P3408	protein_cc	7420452D	mitogen-a	Map3k5
1381	Map7	0.760	1.023	0.778	0.052	0.870	0.181	7.364	6.968	7.001	17761	Map7	chr10	+	NM_0086	P11243	protein_cc	E-MAP-11	microtubu	Map7
1382	4930405J1	1.000	1.000	1.000	1.000	1.000	1.000	0.000	0.000	0.000	73811	4930405J1	chr10	-	NR_04535	.	lncRNA	.	RIKEN cDN	4930405J1
1383	Gm6251	1.120	1.192	1.335	0.551	0.213	0.228	9.282	9.445	9.699	.	Gm6251	chr10	+	NM_0011	P867	.	.	.	Gm6251
1384	Bclaf1	1.161	0.897	1.041	0.031	0.610	0.917	9.908	10.124	9.966	72567	Bclaf1	chr10	+	NM_0010	P1954	protein_cc	2610102K	BCL2-asso	Bclaf1
1385	Mtfr2	4.994	0.200	0.999	0.306	1.000	0.423	0.001	2.321	0.000	71804	Mtfr2	chr10	+	NM_0279	P17552	protein_cc	2610016C	mitochond	Mtfr2
1386	Pde7b	0.359	1.181	0.424	0.020	0.007	0.176	3.285	1.807	2.048	29863	Pde7b	chr10	-	NM_0138	P12111	protein_cc	.	phosphod	Pde7b
1387	Ahi1	4.627	0.563	2.606	0.000	0.000	0.011	4.021	6.231	5.403	52906	Ahi1	chr10	+	NM_0262	P17358	protein_cc	1700015F(	Abelson h	Ahi1
1388	Myb	4.466	0.224	0.999	0.307	1.000	0.423	0.001	2.160	0.000	17863	Myb	chr10	-	NM_0011	P21019	protein_cc	AI550390|	myeloblas	Myb
1389	Hbs1l	1.071	0.709	0.759	0.218	0.001	0.106	9.105	9.204	8.707	56422	Hbs1l	chr10	+	NM_0010	P13375	protein_cc	2810035F	Hbs1-like	Hbs1l
1390	4930455C	2.162	1.021	2.208	0.300	0.972	0.423	0.001	1.113	1.143	73990	4930455C	chr10	-	NR_04535	.	lncRNA	.	RIKEN cDN	4930455C
1391	Aldh8a1	0.125	2.094	0.262	0.000	0.014	0.029	11.871	8.871	9.937	237320	Aldh8a1	chr10	+	NM_1787	P9481	protein_cc	Raldh4	aldehyde	Aldh8a1
1392	1700020N	1.000	1.000	1.000	1.000	1.000	1.000	0.000	0.000	0.000	67692	1700020N	chr10	+	NR_02796	.	lncRNA	.	RIKEN cDN	1700020N
1393	Gm5420	1.000	1.000	1.000	1.000	1.000	1.000	0.000	0.000	0.000	432436	Gm5420	chr10	-	NR_04584	.	lncRNA	EG432436	predicted	Gm5420
1394	Sgk1	1.176	1.050	1.235	0.027	0.027	0.118	8.065	8.299	8.369	20393	Sgk1	chr10	+	NM_0011	P23028	protein_cc	Sgk	serum/glu	Sgk1
1395	4930444G	1.000	1.000	1.000	1.000	1.000	1.000	0.000	0.000	0.000	114671	4930444G	chr10	-	NM_0532	P11254	protein_cc	.	RIKEN cDN	4930444G
1396	E030030I(	1.108	0.759	0.841	0.138	0.006	0.085	4.107	4.255	3.857	319887	E030030I(	chr10	-	NM_0012	P7719	protein_cc	.	RIKEN cDN	E030030I0
1397	Raet1a	1.000	1.000	1.000	1.000	1.000	1.000	0.000	0.000	0.000	.	Raet1a	chr10	+	NM_0090	P25389	.	.	.	Raet1a
1398	Raet1e	0.543	0.643	0.349	0.003	0.052	0.004	5.786	4.906	4.268	379043	Raet1e	chr10	+	NM_1981	P2416	protein_cc	.	retinoic ac	Raet1e
1399	Raet1b	0.644	0.433	0.279	0.003	0.020	0.035	7.200	6.565	5.357	.	Raet1b	chr10	+	NM_0090	P25389	.	.	.	Raet1b
1400	Raet1c	1.787	0.491	0.878	0.156	0.185	0.876	2.650	3.487	2.463	.	Raet1c	chr10	+	NM_0090	P2416	.	.	.	Raet1c
1401	H60b	1.572	0.636	1.000	0.304	1.000	0.423	0.000	0.653	0.000	667281	H60b	chr10	+	NM_0011	P25477	protein_cc	EG667281	histocomp	H60b
1402	C920009B	2.728	1.111	3.032	0.149	0.840	0.398	1.476	2.923	3.076	606736	C920009B	chr10	+	NR_01546	.	lncRNA	.	RIKEN cDN	C920009B
1403	Raet1d	1.000	1.000	1.000	1.000	1.000	1.000	0.000	0.000	0.000	56554	Raet1d	chr10	+	NM_0200	P6567	protein_cc	RAE-1delta	retinoic ac	Raet1d
1404	Gm10825	0.885	0.629	0.557	0.781	1.000	0.422	0.844	0.668	0.000	10003875	Gm10825	chr10	+	NR_02858	.	lncRNA	ENSMUSG	predicted	Gm10825
1405	Slc2a12	1.596	1.020	1.628	0.397	0.957	0.370	3.874	4.549	4.577	353169	Slc2a12	chr10	+	NM_1789	P24182	protein_cc	GLUT-12|	solute car	Slc2a12
1406	Tbpl1	2.518	0.690	1.737	0.001	0.038	0.188	6.733	8.065	7.530	237336	Tbpl1	chr10	-	NM_0116	P9839	protein_cc	4732475G	TATA box	Tbpl1
1407	Tcf21	0.050	9.642	0.487	0.000	0.049	0.181	4.467	0.158	3.427	21412	Tcf21	chr10	-	NM_0115	P16403	protein_cc	bHLHa23|	transcripti	Tcf21
1408	Eya4	1.000	1.000	1.000	1.000	1.000	1.000	0.000	0.000	0.000	14051	Eya4	chr10	-	NM_0101	P12298	protein_cc	B130023L	EYA transc	Eya4
1409	Rps12	1.618	1.593	2.577	0.006	0.009	0.036	8.812	9.506	10.178	20042	Rps12	chr10	-	NM_0112	P13303	protein_cc	.	ribosomal	Rps12
1410	Snora33	1.575	0.466	0.734	0.475	1.000	0.422	0.446	1.101	0.000	10052907	Snora33	chr10	-	NR_03768	.	snoRNA	ACA33| M	small nucl	Snora33
1411	Snord100	1.708	0.940	1.605	0.016	0.844	0.504	1.611	2.383	2.294	10052907	Snord100	chr10	-	NR_03768	.	snoRNA	MBII-429|	small nucl	Snord100
1412	Slc18b1	0.239	4.138	0.988	0.021	0.022	0.975	3.873	1.806	3.855	76306	Slc18b1	chr10	+	NM_1831	P16338	protein_cc	1110021L(	solute car	Slc18b1
1413	Vnn3	0.988	0.569	0.562	0.752	0.000	0.025	9.291	9.274	8.461	26464	Vnn3	chr10	+	NM_0119	P24966	protein_cc	.	vanin 3	Vnn3
1414	Vnn1	0.294	0.708	0.208	0.000	0.022	0.005	9.043	7.277	6.778	22361	Vnn1	chr10	+	NM_0117	P3139	protein_cc	V-1	vanin 1	Vnn1
1415	Taar1	1.000	1.000	1.000	1.000	1.000	1.000	0.000	0.000	0.000	111174	Taar1	chr10	+	NM_0532	P11522	protein_cc	taR-1| Tar	trace amir	Taar1
1416	Taar2	1.000	1.000	1.000	1.000	1.000	1.000	0.000	0.000	0.000	209512	Taar2	chr10	+	NM_0010	P14997	protein_cc	Gm224| G	trace amir	Taar2
1417	Taar3	1.000	1.000	1.000	1.000	1.000	1.000	0.000	0.000	0.000	493809	Taar3	chr10	+	NM_0010	P16486	protein_cc	.	trace amir	Taar3
1418	Taar4	1.000	1.000	1.000	1.000	1.000	1.000	0.000	0.000	0.000	209513	Taar4	chr10	+	NM_0010	P26060	protein_cc	Gm226| T	trace amir	Taar4
1419	Taar5	1.000	1.000	1.000	1.000	1.000	1.000	0.000	0.000	0.000	215854	Taar5	chr10	+	NM_0010	P9799	protein_cc	Gm227| ta	trace amir	Taar5
1420	Taar6	1.000	1.000	1.000	1.000	1.000	1.000	0.000	0.000	0.000	215855	Taar6	chr10	-	NM_0010	P23242	protein_cc	Gm228| ta	trace amir	Taar6
1421	Taar7a	1.000	1.000	1.000	1.000	1.000	1.000	0.000	0.000	0.000	215856	Taar7a	chr10	-	NM_0010	P5851	protein_cc	Gm229| ta	trace amir	Taar7a
1422	Taar7b	1.000	1.000	1.000	1.000	1.000	1.000	0.000	0.000	0.000	209517	Taar7b	chr10	-	NM_0010	P18459	protein_cc	Gm698| ta	trace amir	Taar7b
1423	Taar7d	1.000	1.000	1.000	1.000	1.000	1.000	0.000	0.000	0.000	435206	Taar7d	chr10	+	NM_0010	P2879	protein_cc	taR-7d	trace amir	Taar7d
1424	Taar7e	1.000	1.000	1.000	1.000	1.000	1.000	0.000	0.000	0.000	276742	Taar7e	chr10	+	NM_0010	P15817	protein_cc	Gm697| ta	trace amir	Taar7e
1425	Taar7f	1.000	1.000	1.000	1.000	1.000	1.000	0.000	0.000	0.000	435207	Taar7f	chr10	+	NM_0010	P4212	protein_cc	taR-7f	trace amir	Taar7f
1426	Taar8a	1.000	1.000	1.000	1.000	1.000	1.000	0.000	0.000	0.000	215859	Taar8a	chr10	+	NM_0010	P9769	protein_cc	Gm230| ta	trace amir	Taar8a
1427	Taar8b	0.716	0.995	0.712	0.182	1.000	0.423	0.490	0.008	0.000	382348	Taar8b	chr10	+	NM_0010	P5482	protein_cc	Gm1149|	trace amir	Taar8b
1428	Taar8c	1.000	1.000	1.000	1.000	1.000	1.000	0.000	0.000	0.000	494546	Taar8c	chr10	+	NM_0010	P25224	protein_cc	taR-8c	trace amir	Taar8c
1429	Taar9	1.000	1.000	1.000	1.000	1.000	1.000	0.000	0.000	0.000	503558	Taar9	chr10	+	NM_0010	P11459	protein_cc	Gm231| ta	trace amir	Taar9
1430	Stx7	1.280	1.094	1.400	0.008	0.391	0.157	7.764	8.120	8.249	53331	Stx7	chr10	+	NM_0167	P11786	protein_cc	AI315064|	syntaxin 7	Stx7
1431	Moxd1	1.075	11.787	12.673	0.000	0.116	0.343	0.001	0.106	3.665	59012	Moxd1	chr10	+	NM_0215	P7176	protein_cc	3230402N	monooxyg	Moxd1
1432	Ctgf	1.296	2.030	2.632	0.650	0.255	0.346	7.660	8.034	9.056	14219	Ctgf	chr10	+	NM_0102	P24272	protein_cc	Ccn2| fisp	connective	Ctgf
1433	Enpp1	0.322	1.002	0.322	0.000	0.990	0.010	7.753	6.116	6.119	18605	Enpp1	chr10	-	NM_0088	P4299	protein_cc	4833416E	ectonucle	Enpp1
1434	Enpp3	0.362	0.855	0.309	0.001	0.731	0.082	8.515	7.048	6.822	209558	Enpp3	chr10	-	NM_1340	P6093	protein_cc	AI876438|	ectonucle	Enpp3
1435	Med23	1.442	0.723	1.043	0.009	0.068	0.892	5.815	6.343	5.875	70208	Med23	chr10	+	NM_0011	P517	protein_cc	130kDa| 3	mediator	Med23
1436	Arg1	0.618	1.760	1.088	0.000	0.000	0.224	14.013	13.319	14.134	11846	Arg1	chr10	-	NM_0074	P12448	protein_cc	AI| AI256	arginase, l	Arg1
1437	Akap7	0.722	0.792	0.572	0.014	0.016	0.048	7.507	7.037	6.701	432442	Akap7	chr10	-	NM_0187	P26603	protein_cc	6430401D	A kinase (I	Akap7
1438	Epb4.1l2	0.889	1.085	0.964	0.212	0.698	0.920	8.123	7.953	8.071	.	Epb4.1l2	chr10	+	NM_0011	P2406	.	.	.	Epb4.1l2
1439	Smlr1	1.332	1.300	1.733	0.011	0.509	0.500	8.980	9.394	9.773	10050447	Smlr1	chr10	-	NM_0011	P3143	protein_cc	2010003K	small leuc	Smlr1
1440	4930401C	0.890	0.626	0.557	0.792	1.000	0.422	0.844	0.677	0.000	73803	4930401C	chr10	-	NR_04534	.	lncRNA	.	RIKEN cDN	4930401C
1441	Tmem200	1.000	1.000	1.000	1.000	1.000	1.000	0.000	0.000	0.000	77220	Tmem200	chr10	-	NM_0298	P24212	protein_cc	C030003D	transmem	Tmem200
1442	Gm9767	1.000	1.000	1.000	1.000	1.000	1.000	0.000	0.000	0.000	10004085	Gm9767	chr10	+	NR_02803	.	lncRNA	ENSMUSG	predicted	Gm9767
1443	Samd3	0.272	0.973	0.265	0.022	1.000	0.154	1.917	0.039	0.000	268288	Samd3	chr10	+	NM_0010	P19121	protein_cc	Gm623	sterile alp	Samd3

1444	L3mbtl3	1.454	0.842	1.224	0.354	0.266	0.761	5.574	6.114	5.866	237339	L3mbtl3	chr10	-	NM_1727	P16758	protein_cc	AI481284	l(3)mbt-lik	L3mbtl3
1445	Gm8709	1.068	1.452	1.550	0.870	0.490	0.620	0.587	0.682	1.220	667572	Gm8709	chr10	-	NR_03363	.	misc_RNA	EG667572	predicted	Gm8709
1446	Arhgap18	0.231	2.189	0.506	0.000	0.012	0.004	7.432	5.319	6.450	73910	Arhgap18	chr10	+	NM_1768	P906	protein_cc	4833419J(	Rho GTPas	Arhgap18
1447	Lama2	0.564	1.516	0.855	0.135	0.409	0.810	3.650	2.825	3.425	16773	Lama2	chr10	-	NM_0084	P24797	protein_cc	5830440B	laminin, al	Lama2
1448	4930579H	1.000	1.000	1.000	1.000	1.000	1.000	0.000	0.000	0.000	75855	4930579H	chr10	+	NR_13103	.	lncRNA	.	RIKEN cDN	4930579H
1449	Ptprk	0.983	0.806	0.792	0.795	0.035	0.032	9.240	9.215	8.903	19272	Ptprk	chr10	+	NM_0089	P13568	protein_cc	AI853699|	protein ty	Ptprk
1450	Themis	0.674	4.586	3.089	0.480	0.000	0.075	1.308	0.738	2.935	210757	Themis	chr10	+	NM_0013	P12605	protein_cc	E430004N	thymocyte	Themis
1451	4930519F	0.979	0.301	0.295	0.970	0.188	0.296	2.231	2.200	0.469	114676	4930519F	chr10	-	NR_03360	.	misc_RNA	.	RIKEN cDN	4930519F
1452	2310057J	1.000	1.000	1.000	1.000	1.000	1.000	0.000	0.000	0.000	67719	2310057J	chr10	-	NM_0263	P9686	protein_cc	2210414P	RIKEN cDN	2310057J
1453	Soga3	0.560	1.371	0.768	0.184	0.191	0.687	0.857	0.021	0.477	67412	Soga3	chr10	+	NM_0261	P22531	protein_cc	6330407J	SOGA fam	Soga3
1454	9330159F	0.380	1.742	0.662	0.017	0.000	0.321	1.433	0.038	0.839	212448	9330159F	chr10	+	NM_0011	P21656	protein_cc	.	RIKEN cDN	9330159F
1455	Echdc1	0.927	0.995	0.923	0.487	0.974	0.660	9.328	9.218	9.212	52665	Echdc1	chr10	+	NM_0011	P13737	protein_cc	1700028A	enoyl Coe	Echdc1
1456	Rnf146	0.895	1.036	0.928	0.244	0.797	0.701	9.459	9.299	9.351	68031	Rnf146	chr10	-	NM_0265	P12418	protein_cc	2610509H	ring finger	Rnf146
1457	Rspo3	0.364	1.666	0.606	0.001	0.106	0.214	7.947	6.489	7.225	72780	Rspo3	chr10	-	NM_0283	P14683	protein_cc	2810459H	R-spondin	Rspo3
1458	Cenpw	0.546	3.043	1.660	0.061	0.071	0.439	6.435	5.561	7.166	66311	Cenpw	chr10	-	NM_0011	P22797	protein_cc	2610036L	centromer	Cenpw
1459	Trmt11	1.042	1.277	1.331	0.812	0.153	0.048	5.508	5.567	5.920	73681	Trmt11	chr10	-	NM_0286	P20352	protein_cc	2410075D	tRNA met	Trmt11
1460	Gm20300	0.224	1.544	0.345	0.001	0.256	0.082	7.770	5.609	6.235	10050458	Gm20300	chr10	-	NR_04500	.	lncRNA	.	predicted	Gm20300
1461	Hint3	0.530	1.315	0.696	0.004	0.146	0.183	7.792	6.876	7.270	66847	Hint3	chr10	-	NM_0257	P14996	protein_cc	0610010I1	histidine t	Hint3
1462	Ncoa7	3.007	0.429	1.291	0.001	0.001	0.528	5.871	7.460	6.240	211329	Ncoa7	chr10	-	NM_0011	P4411	protein_cc	9030406N	nuclear re	Ncoa7
1463	Hey2	6.063	0.470	2.850	0.026	0.094	0.002	1.097	3.697	2.608	15214	Hey2	chr10	-	NM_0139	P14201	protein_cc	bHLHb32|	hairy/enh	Hey2
1464	Hddc2	1.818	1.488	2.704	0.214	0.253	0.130	2.290	3.152	3.725	69692	Hddc2	chr10	+	NM_0271	P7579	protein_cc	2310057G	HD domai	Hddc2
1465	Tpd52l1	0.624	1.632	1.018	0.035	0.105	0.964	6.757	6.076	6.783	21987	Tpd52l1	chr10	-	NM_0094	P3973	protein_cc	D53	tumor pro	Tpd52l1
1466	Gm3258	2.584	1.066	2.753	0.000	0.100	0.017	2.523	3.892	3.984	.	Gm3258	chr10	-	NM_0115	P10125	.	.	.	Gm3258
1467	Rnf217	1.318	0.894	1.178	0.223	0.667	0.646	7.274	7.672	7.511	268291	Rnf217	chr10	-	NM_0011	P25811	protein_cc	AU016819	ring finger	Rnf217
1468	Nkain2	2.134	0.469	1.000	0.305	1.000	0.423	0.000	1.094	0.000	432450	Nkain2	chr10	-	NM_0010	P16106	protein_cc	6330571D	Na+/K+ tr	Nkain2
1469	Trdn	0.716	0.995	0.712	0.182	1.000	0.423	0.490	0.008	0.000	76757	Trdn	chr10	+	NM_0297	P25774	protein_cc	2310045H	triadin	Trdn
1470	D830005E	1.000	1.000	1.000	1.000	1.000	1.000	0.000	0.000	0.000	442820	D830005E	chr10	-	NR_04065	.	lncRNA	.	RIKEN cDN	D830005E
1471	Clvs2	1.000	1.000	1.000	1.000	1.000	1.000	0.000	0.000	0.000	215890	Clvs2	chr10	-	NM_1754	P7376	protein_cc	A330019N	clavesin 2	Clvs2
1472	Gm4794	0.086	1.219	0.105	0.015	0.285	0.136	3.641	0.106	0.392	.	Gm4794	chr10	-	NM_0011	P11037	.	.	.	Gm4794
1473	Sult3a1	0.335	0.012	0.004	0.020	1.000	0.062	8.013	6.434	0.000	57430	Sult3a1	chr10	+	NM_0205	P9571	protein_cc	ST3A1| Su	sulfotrans	Sult3a1
1474	Rsph4a	0.051	5.117	0.259	0.000	0.186	0.078	4.454	0.148	2.503	212892	Rsph4a	chr10	-	NM_0011	P16671	protein_cc	A230081C	radial spo	Rsph4a
1475	Zufsp	3.728	0.209	0.778	0.014	0.011	0.449	3.921	5.819	3.558	72580	Zufsp	chr10	-	NM_0282	P22710	protein_cc	2700019D	zinc finger	Zufsp
1476	A830082N	1.110	0.682	0.757	0.768	0.467	0.689	2.665	2.816	2.263	414093	A830082N	chr10	-	NR_01552	.	lncRNA	Gm17567	RIKEN cDN	A830082N
1477	Rwdd1	1.204	1.136	1.368	0.008	0.016	0.003	8.515	8.783	8.968	66521	Rwdd1	chr10	-	NM_0256	P14596	protein_cc	0710001K	RWD dom	Rwdd1
1478	Trappc3l	1.000	1.000	1.000	1.000	1.000	1.000	0.000	0.000	0.000	692132	Trappc3l	chr10	+	NM_0011	P16092	protein_cc	Bet3l| EN	trafficking	Trappc3l
1479	Fam26d	1.000	1.000	1.000	1.000	1.000	1.000	0.000	0.000	0.000	270711	Fam26d	chr10	-	NM_0010	P24793	protein_cc	4732454E	family wit	Fam26d
1480	Fam26e	5.360	0.226	1.211	0.013	0.017	0.745	2.284	4.706	2.560	103511	Fam26e	chr10	-	NM_1789	P25362	protein_cc	BB146404	family wit	Fam26e
1481	Fam26f	68.904	0.155	10.705	0.000	0.001	0.348	4.196	10.303	7.616	215900	Fam26f	chr10	-	NM_1754	P8728	protein_cc	A630077B	family wit	Fam26f
1482	Dse	2.082	0.398	0.829	0.022	0.012	0.475	6.095	7.153	5.825	212898	Dse	chr10	-	NM_1725	P845	protein_cc	6030499O	dermatan	Dse
1483	Tspyl1	1.575	0.681	1.072	0.002	0.002	0.351	8.018	8.673	8.118	22110	Tspyl1	chr10	+	NM_0094	P12264	protein_cc	Tspyl	testis-spe	Tspyl1
1484	Tspyl4	0.569	1.591	0.905	0.384	0.305	0.913	2.607	1.794	2.463	72480	Tspyl4	chr10	+	NM_0302	P5127	protein_cc	2610102M	TSPY-like	Tspyl4
1485	Nt5dc1	0.661	2.097	1.386	0.092	0.077	0.480	6.860	6.262	7.331	319638	Nt5dc1	chr10	-	NM_1769	P12052	protein_cc	6030401B	5'-nucleot	Nt5dc1
1486	Col10a1	1.621	0.951	1.542	0.205	0.757	0.487	1.306	2.003	1.930	12813	Col10a1	chr10	+	NM_0099	P21888	protein_cc	Col10| Co	collagen, t	Col10a1
1487	Frk	1.503	0.895	1.346	0.015	0.334	0.103	6.088	6.676	6.516	14302	Frk	chr10	+	NM_0102	P1634	protein_cc	BSK| BSK/	fyn-relate	Frk
1488	Hs3st5	1.000	1.000	1.000	1.000	1.000	1.000	0.000	0.000	0.000	319415	Hs3st5	chr10	+	NM_0012	P2225	protein_cc	D930005L	heparan s	Hs3st5
1489	Hdac2	1.759	0.795	1.398	0.059	0.282	0.051	7.421	8.236	7.904	15182	Hdac2	chr10	+	NM_0082	P26004	protein_cc	D10Wsu1	histone de	Hdac2
1490	Marcks	3.282	0.567	1.862	0.000	0.000	0.004	9.222	10.937	10.119	17118	Marcks	chr10	-	NM_0085	P25202	protein_cc	Macs| PK	myristoyla	Marcks
1491	4930591E	1.000	1.000	1.000	1.000	1.000	1.000	0.000	0.000	0.000	75877	4930591E	chr10	+	NR_13103	.	lncRNA	.	RIKEN cDN	4930591E
1492	Rfpl4b	1.000	1.000	1.000	1.000	1.000	1.000	0.000	0.000	0.000	215919	Rfpl4b	chr10	-	NM_0011	P7451	protein_cc	10004091	ret finger	Rfpl4b
1493	Lama4	2.870	0.858	2.463	0.024	0.658	0.317	3.908	5.429	5.208	16775	Lama4	chr10	+	NM_0106	P4792	protein_cc	.	laminin, al	Lama4
1494	Fam229b	0.359	2.898	1.041	0.102	0.057	0.926	3.501	2.023	3.558	66337	Fam229b	chr10	-	NM_1832	P15860	protein_cc	1700025K	family wit	Fam229b
1495	Tube1	0.643	0.844	0.543	0.404	0.670	0.591	2.424	1.787	1.543	71924	Tube1	chr10	+	NM_0280	P346	protein_cc	2310061K	epsilon-tu	Tube1
1496	Wisp3	1.000	1.000	1.000	1.000	1.000	1.000	0.000	0.000	0.000	327743	Wisp3	chr10	-	NM_0011	P13139	protein_cc	CCN6| EN	WNT1 ind	Wisp3
1497	Fyn	0.935	0.526	0.492	0.563	0.012	0.032	7.142	7.046	6.120	14360	Fyn	chr10	+	NM_0080	P25868	protein_cc	AI448320|	Fyn proto	Fyn
1498	Traf3ip2	5.764	1.225	7.062	0.029	0.507	0.074	2.484	5.011	5.304	103213	Traf3ip2	chr10	+	NM_1340	P9640	protein_cc	Act1| AI4	TRAF3 inte	Traf3ip2
1499	E130307A	1.001	0.823	0.824	0.998	0.730	0.742	2.210	2.212	1.931	327744	E130307A	chr10	-	NR_03803	.	lncRNA	.	RIKEN cDN	E130307A
1500	Rev3l	0.552	0.955	0.527	0.000	0.279	0.006	6.958	6.101	6.034	19714	Rev3l	chr10	+	NM_0112	P1052	protein_cc	Rev| Rev3	REV3 like,	Rev3l
1501	AA474331	1.000	1.000	1.000	1.000	1.000	1.000	0.000	0.000	0.000	.	AA474331	chr10	-	NR_03362	.	.	.	.	AA474331
1502	2010001E	0.487	0.877	0.427	0.046	0.668	0.145	4.544	3.506	3.317	.	2010001E	chr10	-	NM_0011	P22136	.	.	.	2010001E
1503	G630090E	0.676	0.774	0.524	0.042	0.349	0.159	7.558	6.993	6.624	.	G630090E	chr10	-	NM_0011	P14827	.	.	.	G630090E
1504	BC021785	1.232	0.319	0.393	0.787	0.313	0.438	2.504	2.805	1.158	.	BC021785	chr10	-	NM_0011	P18198	.	.	.	BC021785
1505	AI317395	0.208	1.398	0.290	0.000	0.040	0.016	9.365	7.098	7.581	.	AI317395	chr10	-	NM_1448	P20185	.	.	.	AI317395
1506	Slc16a10	0.304	0.611	0.186	0.000	0.068	0.015	9.815	8.097	7.387	72472	Slc16a10	chr10	-	NM_0011	P21498	protein_cc	2610103N	solute car	Slc16a10
1507	Rpf2	2.476	0.934	2.313	0.001	0.435	0.022	7.295	8.603	8.504	67239	Rpf2	chr10	-	NM_0233	P22039	protein_cc	2810470K	ribosome	Rpf2
1508	Gtf3c6	1.417	1.167	1.653	0.026	0.331	0.173	8.166	8.668	8.891	67371	Gtf3c6	chr10	-	NM_0261	P148	protein_cc	2410016F	general tra	Gtf3c6
1509	Amd1	0.825	1.208	0.996	0.043	0.000	0.974	8.163	7.885	8.158	11702	Amd1	chr10	-	NM_0096	P3181	protein_cc	AdoMetD(	S-adenosy	Amd1
1510	Amd2	0.823	1.171	0.964	0.035	0.051	0.667	8.632	8.351	8.579	10004158	Amd2	chr10	-	NM_0074	P3181	protein_cc	AdoMetD(	S-adenosy	Amd2
1511	Cdk19	0.465	1.250	0.581	0.000	0.008	0.044	7.033	5.927	6.249	78334	Cdk19	chr10	+	NM_0012	P13489	protein_cc	2700084L(	cyclin-dep	Cdk19
1512	Slc22a16	1.000	1.000	1.000	1.000	1.000	1.000	0.000	0.000	0.000	70840	Slc22a16	chr10	+	NM_0275	P23750	protein_cc	4921504E	solute car	Slc22a16
1513	Ddo	0.185	2.318	0.430	0.007	0.068	0.196	5.607	3.174	4.387	70503	Ddo	chr10	+	NM_0274	P12671	protein_cc	5330420D	D-asparta	Ddo
1514	Mettl24	0.552	0.990	0.547	0.182	1.000	0.423	0.871	0.015	0.000	327747	Mettl24	chr10	+	NM_1777	P701	protein_cc	9030224M	methyltra	Mettl24
1515	Cdc40	0.776	0.892	0.692	0.010	0.261	0.129	7.709	7.342	7.178	71713	Cdc40	chr10	-	NM_0278	P7653	protein_cc	1200003H	cell divisio	Cdc40
1516	Wasf1	1.369	0.412	0.564	0.334	0.109	0.442	3.380	3.833	2.553	83767	Wasf1	chr10	+	NM_0318	P11462	protein_cc	AI195380|	WAS prote	Wasf1
1517	Gpr6	1.000	1.000	1.000	1.000	1.000	1.000	0.000	0.000	0.000	140741	Gpr6	chr10	-	NM_1990	P5200	protein_cc	AI852874|	G protein-	Gpr6
1518	Fig4	0.374	2.389	0.893	0.002	0.000	0.508	6.045	4.626	5.882	103199	Fig4	chr10	-	NM_1339	P3744	protein_cc	A530089I1	FIG4 phos	Fig4
1519	Zbtb24	0.605	1.018	0.616	0.082	0.867	0.281	5.574	4.848	4.874	268294	Zbtb24	chr10	+	NM_0012	P11950	protein_cc	BC055367	zinc finger	Zbtb24
1520	Mical1	0.799	0.919	0.734	0.428	0.224	0.545	3.473	3.149	3.027	171580	Mical1	chr10	+	NM_0011	P14128	protein_cc	MICAL| M	microtubu	Mical1
1521	Smpd2	0.379	1.222	0.463	0.000	0.306	0.053	7.790	6.389	6.678	20598	Smpd2	chr10	-	NM_0092	P14292	protein_cc	AW10828	sphingom	Smpd2
1522	Ppil6	0.130	8.135	1.058	0.024	0.007	0.918	3.065	0.122	3.146	73075	Ppil6	chr10	+	NM_0284	P18719	protein_cc	2900084F	peptidylpr	Ppil6
1523	Cd164	0.763	0.910	0.694	0.015	0.486	0.203	11.162	10.772	10.636	53599	Cd164	chr10	+	NM_0168	P25059	protein_cc	A115| A24	CD164 ant	Cd164
1524	Ccdc162	0.532	0.810	0.431	0.063	0.293	0.184	4.559	3.649	3.345	75973	Ccdc162	chr10	-	NM_0013	P4271	protein_cc	5033413D	coiled-coil	Ccdc162
1525	5730435O	0.280	1.499	0.420	0.010	0.559	0.170	3.349	1.514	2.098	70538	5730435O	chr10	+	NR_04534	.	lncRNA	.	RIKEN cDN	5730435O
1526	Cep57l1	0.502	0.667	0.335	0.008	0.032	0.059	4.835	3.841	3.255	103268	Cep57l1	chr10	-	NM_0291	P10146	protein_cc	2410017P	centrosom	Cep57l1
1527	Sesn1	0.278	2.014	0.559	0.000	0.081	0.151	6.578	4.730	5.739	140742	Sesn1	chr10	+	NM_0011	P12504	protein_cc	1110002G	sestrin 1	Sesn1
1528	Armc2	0.885	0.629	0.557	0.781	1.000	0.422	0.844	0.668	0.000	213402	Armc2	chr10	-	NM_0010	P15614	protein_cc	2610018I(	armadillo	Armc2
1529	Foxo3	0.297	1.053	0.313	0.020	0.874	0.125	2.468	0.718	0.792	56484	Foxo3	chr10	-	NM_0197	P17457	protein_cc	1110048B	forkhead	Foxo3
1530	Lace1	0.618	0.888	0.549	0.051	0.590	0.266	5.767	5.072	4.901	215951	Lace1	chr10	+	NM_1457	P13571	protein_cc	CG8520	lactation e	Lace1
1531	Snx3	0.785	0.962	0.755	0.066	0.753	0.107	10.487	10.137	10.081	54198	Snx3	chr10	+	NM_0174	P16994	protein_cc	SDP3	sorting ne	Snx3
1532	Nr2e1	1.000	1.000	1.000	1.000	1.000	1.000	0.000	0.000	0.000	21907	Nr2e1	chr10	-	NM_1522	P17169	protein_cc	fierce| frc	nuclear re	Nr2e1
1533	Ostm1	1.224	0.899	1.100	0.148	0.356	0.292	5.990	6.281	6.127	14628	Ostm1	chr10	+	NM_1724	P10007	protein_cc	1200002H	osteopetr	Ostm1
1534	Sec63	0.894	0.885	0.791	0.003	0.001	0.009	10.881	10.719	10.544	140740	Sec63	chr10	+	NM_1530	P15946	protein_cc	5730478J1	SEC63-like	Sec63

1535	Scml4	1.010	1.885	1.904	0.000	0.182	0.423	0.000	0.015	0.929	268297	Scml4	chr10	+	NM_1729	P19690	protein_cc	9330161D	sex comb	Scml4
1536	Sobp	0.810	1.792	1.452	0.738	0.339	0.490	2.303	2.000	2.842	109205	Sobp	chr10	-	NM_1754	P10607	protein_cc	2900009C	sine oculis	Sobp
1537	9030612E	0.716	0.995	0.712	0.182	1.000	0.423	0.490	0.008	0.000	74530	9030612E	chr10	+	NR_10236	.	lncRNA	.	RIKEN cDN	9030612E
1538	Pdss2	0.595	0.781	0.464	0.250	0.676	0.058	3.148	2.399	2.042	71365	Pdss2	chr10	+	NM_0277	P3877	protein_cc	5430420P	prenyl (so	Pdss2
1539	Bend3	1.334	1.511	2.015	0.562	0.520	0.530	3.489	3.904	4.500	331623	Bend3	chr10	+	NM_1990	P13424	protein_cc	mKIAA155	BEN doma	Bend3
1540	1700021F	0.633	1.274	0.806	0.020	0.263	0.377	7.934	7.273	7.622	67851	1700021F	chr10	-	NM_0264	P21237	protein_cc	.	RIKEN cDN	1700021F
1541	Cd24a	2.167	0.774	1.678	0.000	0.101	0.206	5.553	6.669	6.301	12484	Cd24a	chr10	+	NM_0098	P8412	protein_cc	Cd24 | HSA	CD24a ant	Cd24a
1542	F830002L	2.134	0.469	1.000	0.305	1.000	0.423	0.000	1.094	0.000	414125	F830002L	chr10	+	NR_03355	.	lncRNA	F930017D	RIKEN cDN	F830002L2
1543	1700027J	1.000	1.000	1.000	1.000	1.000	1.000	0.000	0.000	0.000	69443	1700027J	chr10	-	NR_04058	.	lncRNA	.	RIKEN cDN	1700027J0
1544	4933404K	1.005	1.439	1.446	0.000	0.182	0.423	0.000	0.008	0.532	71018	4933404K	chr10	+	NR_13105	.	lncRNA	.	RIKEN cDN	4933404K
1545	Qrsl1	1.729	0.858	1.483	0.000	0.395	0.363	6.201	6.991	6.769	76563	Qrsl1	chr10	-	NM_0010	P9438	protein_cc	2700038P	glutaminy	Qrsl1
1546	Rtn4ip1	0.567	1.035	0.586	0.005	0.393	0.085	7.269	6.450	6.499	170728	Rtn4ip1	chr10	+	NM_1308	P13428	protein_cc	D10Ertd69	reticulon 4	Rtn4ip1
1547	Aim1	5.615	0.397	2.230	0.001	0.007	0.213	7.248	9.737	8.405	11630	Aim1	chr10	-	NM_1723	P3303	protein_cc	AI462491 |	absent in	Aim1
1548	Speer5-ps	1.000	1.000	1.000	1.000	1.000	1.000	0.000	0.000	0.000	70365	Speer5-ps	chr10	+	NR_00158	.	misc_RNA	1700007C	spermatog	Speer5-ps1
1549	Atg5	0.790	0.990	0.783	0.009	0.846	0.144	9.922	9.583	9.569	11793	Atg5	chr10	+	NM_0530	P718	protein_cc	2010107M	autophagy	Atg5
1550	Prdm1	22.964	0.219	5.021	0.000	0.000	0.089	1.981	6.502	4.309	12142	Prdm1	chr10	-	NM_0075	P19863	protein_cc	b2b1765C	PR domain	Prdm1
1551	Prep	0.594	1.213	0.720	0.010	0.102	0.118	7.520	6.768	7.046	19072	Prep	chr10	+	NM_0111	P1908	protein_cc	AI047692 |	prolyl end	Prep
1552	Popdc3	1.005	1.439	1.446	0.000	0.182	0.423	0.000	0.008	0.532	78977	Popdc3	chr10	+	NM_0242	P2006	protein_cc	AA682104	popeye do	Popdc3
1553	Bves	1.146	0.632	0.725	0.729	1.000	0.422	0.465	0.661	0.000	23828	Bves	chr10	+	NM_0242	P3429	protein_cc	mBVES | P	blood vess	Bves
1554	Lin28b	1.000	1.000	1.000	1.000	1.000	1.000	0.000	0.000	0.000	380669	Lin28b	chr10	-	NM_0010	P9497	protein_cc	2810403D	lin-28 hom	Lin28b
1555	Hace1	2.628	0.964	2.533	0.019	0.817	0.039	3.702	5.096	5.043	209462	Hace1	chr10	+	NM_1724	P25759	protein_cc	1700042J1	HECT dom	Hace1
1556	D030045P	1.000	1.000	1.000	1.000	1.000	1.000	0.000	0.000	0.000	320363	D030045P	chr10	+	NR_04062	.	lncRNA	.	RIKEN cDN	D030045P
1557	Snord14d	1.000	1.000	1.000	1.000	1.000	1.000	0.000	0.000	0.000	10030259	Snord14d	chr10	+	NR_02827	.	snoRNA	Gm23710	small nucl	Snord14d
1558	Snord14c	1.000	1.000	1.000	1.000	1.000	1.000	0.000	0.000	0.000	108637	Snord14c	chr10	+	NR_02827	.	snoRNA	Gm23107	small nucl	Snord14c
1559	C130030K	1.010	1.825	1.844	0.000	0.182	0.423	0.000	0.015	0.883	10041823	C130030K	chr10	-	NR_04621	.	misc_RNA	.	RIKEN cDN	C130030K
1560	Grik2	1.000	1.000	1.000	1.000	1.000	1.000	0.000	0.000	0.000	14806	Grik2	chr10	-	NM_0011	P23715	protein_cc	AW12449	glutamate	Grik2
1561	Ascc3	1.912	0.581	1.111	0.001	0.001	0.570	6.973	7.907	7.124	77987	Ascc3	chr10	+	NM_1980	P2944	protein_cc	ASC1p200	activating	Ascc3
1562	Sim1	1.000	1.000	1.000	1.000	1.000	1.000	0.000	0.000	0.000	20464	Sim1	chr10	+	NM_0113	P7016	protein_cc	bHLHe14 |	single-min	Sim1
1563	Lilr4b	56.453	0.409	23.111	0.000	0.001	0.020	1.687	7.506	6.217	14727	Lilr4b	chr10	+	NM_0081	P19730	protein_cc	gp49 | Gp4	leukocyte	Lilr4b
1564	Lilrb4a	10.160	0.465	4.722	0.001	0.009	0.007	5.055	8.400	7.294	14728	Lilrb4a	chr10	+	NM_0135	P24145	protein_cc	CD85K | gp	leukocyte	Lilrb4a
1565	Fam162b	1.000	1.000	1.000	1.000	1.000	1.000	0.000	0.000	0.000	77296	Fam162b	chr10	-	NM_0298	P18842	protein_cc	9430073N	family wit	Fam162b
1566	Gprc6a	1.000	1.000	1.000	1.000	1.000	1.000	0.000	0.000	0.000	210198	Gprc6a	chr10	-	NM_1530	P7269	protein_cc	.	G protein-	Gprc6a
1567	Rfx6	1.000	1.000	1.000	1.000	1.000	1.000	0.000	0.000	0.000	320995	Rfx6	chr10	+	NM_0011	P6761	protein_cc	4930572O	regulatory	Rfx6
1568	4933411G	1.000	1.000	1.000	1.000	1.000	1.000	0.000	0.000	0.000	71090	4933411G	chr10	+	NR_04515	.	misc_RNA	.	RIKEN cDN	4933411G
1569	Vgll2	1.000	1.000	1.000	1.000	1.000	1.000	0.000	0.000	0.000	215031	Vgll2	chr10	+	NM_1537	P25131	protein_cc	C130057C	vestigial li	Vgll2
1570	Ros1	0.552	0.990	0.547	0.182	1.000	0.423	0.871	0.015	0.000	19886	Ros1	chr10	-	NM_0112	P9878	protein_cc	c-ros | Ros	Ros1 prote	Ros1
1571	Dcbld1	1.574	0.971	1.528	0.040	0.769	0.295	5.326	5.980	5.938	66686	Dcbld1	chr10	+	NM_0257	P4358	protein_cc	4631413K	discoidin,	Dcbld1
1572	Gopc	1.380	1.065	1.469	0.000	0.210	0.030	7.629	8.093	8.184	94221	Gopc	chr10	-	NM_0531	P4003	protein_cc	2210402P	golgi asso	Gopc
1573	Nepn	1.000	1.000	1.000	1.000	1.000	1.000	0.000	0.000	0.000	66650	Nepn	chr10	+	NM_0256	P24557	protein_cc	5730521E	nephrocan	Nepn
1574	Nus1	1.751	1.099	1.924	0.000	0.190	0.031	9.402	10.210	10.346	52014	Nus1	chr10	+	NM_0302	P18041	protein_cc	1600027K	NUS1 dehy	Nus1
1575	Zfa-ps	1.004	0.496	0.498	0.982	0.036	0.035	4.458	4.463	3.451	22639	Zfa-ps	chr10	-	NR_03792	.	misc_RNA	Zfa | Zfx	zinc finger	Zfa-ps
1576	Slc35f1	0.539	1.371	0.739	0.000	0.191	0.353	0.913	0.021	0.476	215085	Slc35f1	chr10	+	NM_1786	P24748	protein_cc	Duf6 | E13	solute car	Slc35f1
1577	Cep85l	0.498	0.724	0.361	0.007	0.203	0.032	7.731	6.726	6.260	10003872	Cep85l	chr10	-	NM_0012	P11145	protein_cc	ENSMUSG	centrosom	Cep85l
1578	Gm19395	1.000	1.000	1.000	1.000	1.000	1.000	0.000	0.000	0.000	10050282	Gm19395	chr10	+	NR_03801	.	lncRNA	.	predicted	Gm19395
1579	Pln	0.518	0.456	0.236	0.238	1.000	0.211	2.083	1.134	0.000	18821	Pln	chr10	+	NM_0231	P25371	protein_cc	Plb	phospholar	Pln
1580	Gm20597	0.716	0.995	0.712	0.182	1.000	0.423	0.490	0.008	0.000	10050286	Gm20597	chr10	-	NR_03797	.	lncRNA	B230314j1	predicted	Gm20597
1581	Mcm9	0.923	0.517	0.477	0.828	0.014	0.451	3.443	3.327	2.376	71567	Mcm9	chr10	-	NM_0278	P23493	protein_cc	9030408O	minichrom	Mcm9
1582	Asf1a	1.226	1.154	1.415	0.309	0.241	0.364	6.625	6.919	7.125	66403	Asf1a	chr10	+	NM_0255	P1007	protein_cc	2310079C	anti-silenc	Asf1a
1583	Fam184a	2.731	0.822	2.246	0.155	0.637	0.115	1.866	3.316	3.033	75906	Fam184a	chr10	-	NM_0010	P7433	protein_cc	3110012E	family wit	Fam184a
1584	Man1a	1.353	1.058	1.431	0.057	0.592	0.183	9.472	9.908	9.989	17155	Man1a	chr10	-	NM_0085	P12423	protein_cc	Man1a1 |	mannosid	Man1a
1585	Gm16998	0.345	0.619	0.214	0.083	0.464	0.182	2.703	1.169	0.476	10050292	Gm16998	chr10	+	NR_03801	.	lncRNA	.	predicted	Gm16998
1586	Tbc1d32	0.150	3.077	0.461	0.001	0.183	0.209	5.690	2.951	4.573	544696	Tbc1d32	chr10	-	NM_0010	P24378	protein_cc	b2b2284C	TBC1 dom	Tbc1d32
1587	Msl3l2	7.329	1.011	7.409	0.020	0.978	0.199	2.240	5.114	5.130	73390	Msl3l2	chr10	+	NM_0011	P16110	protein_cc	1700060H	male-spec	Msl3l2
1588	Gja1	6.273	0.800	5.019	0.044	0.527	0.002	5.340	7.989	7.668	14609	Gja1	chr10	+	NM_0102	P19437	protein_cc	AU04204S	gap juncti	Gja1
1589	4930467K	1.000	1.000	1.000	1.000	1.000	1.000	0.000	0.000	0.000	74928	4930467K	chr10	-	NR_04535	.	lncRNA	.	RIKEN cDN	4930467K
1590	Hsf2	0.380	1.400	0.532	0.000	0.007	0.007	8.331	6.935	7.421	15500	Hsf2	chr10	+	NM_0082	P14834	protein_cc	AI661205	heat shock	Hsf2
1591	Serinc1	1.078	0.998	1.075	0.477	0.967	0.711	9.512	9.619	9.616	56442	Serinc1	chr10	-	NM_0197	P908	protein_cc	1500011D	serine inc	Serinc1
1592	Pkib	1.168	0.931	1.088	0.198	0.440	0.681	3.270	3.494	3.392	18768	Pkib	chr10	+	NM_0088	P8308	protein_cc	Pkibeta | P	protein kin	Pkib
1593	Fabp7	0.833	0.181	0.151	0.187	0.000	0.035	5.655	5.392	2.924	12140	Fabp7	chr10	+	NM_0212	P7221	protein_cc	B-FABP | B	fatty acid	Fabp7
1594	Smpdl3a	0.410	1.336	0.548	0.002	0.165	0.015	8.334	7.048	7.466	57319	Smpdl3a	chr10	+	NM_0205	P23382	protein_cc	0610010C	sphingom	Smpdl3a
1595	Dux	1.000	1.000	1.000	1.000	1.000	1.000	0.000	0.000	0.000	.	Dux	chr10	+	NM_0010	P22094	.	.	.	Dux
1596	Gm4981	1.000	1.000	1.000	1.000	1.000	1.000	0.000	0.000	0.000	245263	Gm4981	chr10	-	NM_0010	P25855	protein_cc	EG245263	predicted	Gm4981
1597	Gcc2	2.362	0.800	1.890	0.000	0.002	0.043	8.814	10.055	9.732	70297	Gcc2	chr10	+	NM_0273	P22369	protein_cc	0610043A	GRIP and	Gcc2
1598	Lims1	1.597	0.924	1.476	0.018	0.456	0.132	6.792	7.468	7.354	110829	Lims1	chr10	+	NM_0261	P25446	protein_cc	2310016J	LIM and se	Lims1
1599	Ranbp2	2.103	0.756	1.589	0.002	0.133	0.259	7.918	8.990	8.586	19386	Ranbp2	chr10	+	NM_0112	P9562	protein_cc	A430087B	RAN bindi	Ranbp2
1600	Ccdc138	0.802	1.292	1.036	0.684	0.691	0.975	2.108	1.789	2.158	76138	Ccdc138	chr10	+	NM_0011	P1222	protein_cc	6230424H	coiled-coil	Ccdc138
1601	Edar	0.594	0.623	0.371	0.170	1.000	0.134	1.432	0.682	0.000	13608	Edar	chr10	-	NM_0101	P15553	protein_cc	dl | ED1R |	ectodyspla	Edar
1602	BB019430	1.000	1.000	1.000	1.000	1.000	1.000	0.000	0.000	0.000	103505	BB019430	chr10	+	NR_03356	.	lncRNA	.	expressed	BB019430
1603	Sh3rf3	1.575	0.466	0.734	0.475	1.000	0.422	0.446	1.101	0.000	237353	Sh3rf3	chr10	+	NM_1727	P17949	protein_cc	4831416G	SH3 doma	Sh3rf3
1604	Sept10	1.853	0.821	1.521	0.001	0.177	0.231	7.201	8.091	7.807	103080	Sept10	chr10	-	NM_0010	P11392	protein_cc	4921515A	septin 10	Sept10
1605	Sowahc	20.065	0.235	4.711	0.000	0.001	0.242	5.779	10.106	8.016	268301	Sowahc	chr10	+	NM_1729	P10519	protein_cc	4921515A	sosondow	Sowahc
1606	P4ha1	2.806	0.309	0.866	0.001	0.004	0.824	5.758	7.246	5.551	18451	P4ha1	chr10	+	NM_0110	P5939	protein_cc	AL022634	procollage	P4ha1
1607	Pla2g12b	1.624	0.843	1.369	0.005	0.109	0.078	9.424	10.124	9.877	69836	Pla2g12b	chr10	+	NM_0235	P13460	protein_cc	2010002E	phospholi	Pla2g12b
1608	Oit3	0.328	1.275	0.418	0.001	0.002	0.045	8.377	6.770	7.120	18302	Oit3	chr10	-	NM_0109	P15994	protein_cc	EF-9 | LZP	oncoprote	Oit3
1609	Mcu	0.305	1.614	0.492	0.004	0.019	0.116	9.681	7.967	8.658	215999	Mcu	chr10	-	NM_0010	P1413	protein_cc	20100120	mitochond	Mcu
1610	Micu1	0.706	0.968	0.684	0.095	0.879	0.033	8.202	7.700	7.654	216001	Micu1	chr10	+	NM_0012	P11853	protein_cc	C730016L	mitochond	Micu1
1611	Dnajb12	0.605	0.950	0.575	0.060	0.799	0.274	6.369	5.644	5.570	56709	Dnajb12	chr10	+	NM_0199	P11903	protein_cc	Dj10 | mDj	DnaJ heat	Dnajb12
1612	Ddit4	2.306	0.780	1.799	0.181	0.583	0.303	9.463	10.668	10.310	74747	Ddit4	chr10	-	NM_0290	P133	protein_cc	5830413E	DNA-dama	Ddit4
1613	Anapc16	1.909	1.083	2.068	0.003	0.466	0.050	8.418	9.351	9.466	52717	Anapc16	chr10	-	NM_0255	P9158	protein_cc	2310005G	anaphase	Anapc16
1614	Ascc1	0.362	1.366	0.495	0.005	0.318	0.040	6.938	5.473	5.923	69090	Ascc1	chr10	+	NM_0269	P6340	protein_cc	1810015P	activating	Ascc1
1615	Spock2	1.580	0.633	1.000	0.305	1.000	0.423	0.000	0.660	0.000	94214	Spock2	chr10	+	NM_0529	P22842	protein_cc	AA407235	sparc/oste	Spock2
1616	Chst3	1.271	0.219	0.278	0.654	1.000	0.026	1.845	2.191	0.000	53374	Chst3	chr10	-	NM_0168	P22003	protein_cc	C6ST | C6S	carbohydr	Chst3
1617	Psap	0.674	0.920	0.620	0.000	0.348	0.036	12.248	11.678	11.558	19156	Psap	chr10	+	NM_0011	P23134	protein_cc	AI037048 |	prosaposi	Psap
1618	Cdh23	2.803	2.525	7.078	0.296	0.347	0.423	0.001	1.488	2.825	22295	Cdh23	chr10	-	NM_0233	P18143	protein_cc	4930542A	cadherin 2	Cdh23
1619	4632428N	0.936	1.145	1.071	0.337	0.213	0.727	6.197	6.101	6.296	.	4632428N	chr10	+	NM_0011	P19897	.	.	.	4632428N
1620	Gm17455	2.722	0.367	1.000	0.306	1.000	0.423	0.001	1.445	0.000	10030268	Gm17455	chr10	+	NM_0011	P8433	protein_cc	.	predicted	Gm17455
1621	Slc29a3	0.986	0.867	0.855	0.924	0.348	0.247	6.053	6.033	5.827	71279	Slc29a3	chr10	-	NM_0235	P20817	protein_cc	4933435C	solute car	Slc29a3
1622	Unc5b	0.170	3.031	0.514	0.000	0.024	0.091	6.144	3.585	5.185	107449	Unc5b	chr10	-	NM_0297	P17852	protein_cc	6330415E	unc-5 netr	Unc5b
1623	Pldi	1.000	1.000	1.000	1.000	1.000	1.000	0.000	0.000	0.000	73616	Pldi	chr10	+	NR_03361	.	lncRNA	1700125F	polymorpl	Pldi
1624	Gm20611	1.000	1.000	1.000	1.000	1.000	1.000	0.000	0.000	0.000	327760	Gm20611	chr10	+	NR_04063	.	lncRNA	4930565A	predicted	Gm20611
1625	Pcbd1	0.288	1.133	0.326	0.001	0.355	0.025	11.877	10.080	10.259	13180	Pcbd1	chr10	+	NM_0252	P12050	protein_cc	Dcoh | Pcb	pterin 4 al	Pcbd1

1626	Sgpl1	0.468	0.877	0.410	0.001	0.356	0.005	9.236	8.139	7.949	20397	Sgpl1	chr10	-	NM_0091	P11014	protein_cc AI428538|	sphingosir	Sgpl1
1627	Tbata	1.000	1.000	1.000	1.000	1.000	1.000	0.000	0.000	0.000	65971	Tbata	chr10	+	NM_0010	P16452	protein_cc 1700021K	thymus, b	Tbata
1628	Adamts14	1.119	0.908	1.016	0.792	0.824	0.987	1.335	1.497	1.359	237360	Adamts14	chr10	-	NM_0010	P3777	protein_cc Adamts-14	a disinteg	Adamts14
1629	Prf1	0.339	0.981	0.333	0.181	1.000	0.423	1.588	0.028	0.000	18646	Prf1	chr10	+	NM_0110	P23898	protein_cc Pfn| Pfp|	perforin 1	Prf1
1630	Pald1	1.003	1.120	1.123	0.992	0.725	0.580	5.607	5.611	5.775	27355	Pald1	chr10	-	NM_0137	P8871	protein_cc mKIAA127	phosphata	Pald1
1631	Nodal	7.983	0.937	7.477	0.006	0.880	0.299	0.706	3.703	3.609	18119	Nodal	chr10	+	NM_0136	P6636	protein_cc Tg.413d	nodal	Nodal
1632	Eif4ebp2	0.280	1.089	0.305	0.005	0.828	0.055	10.755	8.919	9.042	13688	Eif4ebp2	chr10	-	NM_0101	P20411	protein_cc 2810011I1	eukaryotic	Eif4ebp2
1633	Lrrc20	0.856	1.549	1.326	0.460	0.001	0.384	5.599	5.375	6.006	216011	Lrrc20	chr10	+	NM_1535	P17181	protein_cc BC036304	leucine ric	Lrrc20
1634	Npffr1	1.000	1.000	1.000	1.000	1.000	1.000	0.000	0.000	0.000	237362	Npffr1	chr10	+	NM_0011	P4462	protein_cc Gm236| G	neuropept	Npffr1
1635	Ppa1	2.048	0.820	1.678	0.000	0.025	0.059	10.885	11.919	11.632	67895	Ppa1	chr10	+	NM_0264	P4732	protein_cc 2010317E	pyrophosp	Ppa1
1636	1700022H	1.000	0.459	0.459	0.999	1.000	0.422	1.125	1.124	0.000	75539	1700022H	chr10	-	NR_12647	.	lncRNA .	RIKEN cDN	1700022H
1637	Sar1a	2.631	0.763	2.009	0.000	0.006	0.033	8.902	10.298	9.909	20224	Sar1a	chr10	+	NM_0091	P3285	protein_cc 1600019H	secretion	Sar1a
1638	Tysnd1	0.283	2.002	0.567	0.001	0.007	0.078	8.524	6.703	7.705	71767	Tysnd1	chr10	+	NM_0279	P26233	protein_cc 1300019N	trypsin do	Tysnd1
1639	Aifm2	0.380	2.187	0.832	0.000	0.047	0.588	7.653	6.258	7.387	71361	Aifm2	chr10	+	NM_1780	P5291	protein_cc 5430437E	apoptosis-	Aifm2
1640	H2afy2	0.382	2.910	1.111	0.004	0.006	0.705	5.908	4.518	6.059	404634	H2afy2	chr10	-	NM_2070	P22065	protein_cc macroH2A	H2A histo	H2afy2
1641	Col13a1	0.971	1.360	1.320	0.824	0.300	0.560	7.115	7.073	7.516	12817	Col13a1	chr10	-	NM_0013	P12998	protein_cc .	collagen, t	Col13a1
1642	2010107G	1.343	1.181	1.586	0.160	0.426	0.394	3.877	4.302	4.543	69894	2010107G	chr10	-	NM_0272	P16951	protein_cc AA545165	RIKEN cDN	2010107G
1643	Neurog3	1.000	1.000	1.000	1.000	1.000	1.000	0.000	0.000	0.000	11925	Neurog3	chr10	+	NM_0097	P20157	protein_cc Atoh5| bH	neurogeni	Neurog3
1644	Tspan15	0.172	2.083	0.358	0.001	0.010	0.048	4.968	2.429	3.487	70423	Tspan15	chr10	-	NM_1979	P11322	protein_cc 1110036D	tetraspani	Tspan15
1645	Tacr2	1.000	1.000	1.000	1.000	1.000	1.000	0.000	0.000	0.000	21337	Tacr2	chr10	+	NM_0093	P6001	protein_cc Nk2r| Skr|	tachykinin	Tacr2
1646	Hk1	6.553	0.370	2.423	0.011	0.030	0.104	4.796	7.508	6.073	15275	Hk1	chr10	-	NM_0104	P7216	protein_cc BB404130	hexokinas	Hk1
1647	Hk1os	1.572	0.636	1.000	0.304	1.000	0.423	0.000	0.653	0.000	70162	Hk1os	chr10	+	NR_03803	.	lncRNA 2210417K	hexokinas	Hk1os
1648	Hkdc1	0.676	1.867	1.261	0.188	0.184	0.739	0.587	0.021	0.922	216019	Hkdc1	chr10	-	NM_1454	P9313	protein_cc BC016235	hexokinas	Hkdc1
1649	Supv3l1	1.047	1.202	1.258	0.297	0.024	0.147	7.286	7.352	7.618	338359	Supv3l1	chr10	-	NM_1814	P17708	protein_cc 6330443E	suppresso	Supv3l1
1650	4930507D	0.761	0.529	0.402	0.275	0.016	0.168	4.741	4.347	3.428	74706	4930507D	chr10	+	NR_02792	.	lncRNA .	RIKEN cDN	4930507D
1651	Vps26a	1.172	0.956	1.121	0.109	0.642	0.435	8.262	8.491	8.427	30930	Vps26a	chr10	-	NM_0011	P20348	protein_cc AA407240	VPS26 ret	Vps26a
1652	Srgn	10.543	0.712	7.511	0.000	0.011	0.007	7.660	11.058	10.569	19073	Srgn	chr10	-	NM_0111	P8036	protein_cc Prg| Prg1|	serglycin	Srgn
1653	Kif1bp	1.453	0.710	1.031	0.008	0.011	0.857	6.523	7.063	6.568	72320	Kif1bp	chr10	-	NM_0281	P408	protein_cc 0710007C	KIF1 bindi	Kif1bp
1654	Ddx21	2.791	0.803	2.240	0.000	0.054	0.069	9.327	10.808	10.491	56200	Ddx21	chr10	-	NM_0195	P19891	protein_cc AI255159|	DEAD (Asp	Ddx21
1655	Ddx50	1.220	0.926	1.129	0.052	0.491	0.488	8.963	9.250	9.139	94213	Ddx50	chr10	-	NM_0531	P24914	protein_cc 4933429B	DEAD (Asp	Ddx50
1656	Stox1	0.451	0.986	0.444	0.182	1.000	0.423	1.170	0.021	0.000	216021	Stox1	chr10	-	NM_0010	P18328	protein_cc 4732470K	storkhead	Stox1
1657	Ccar1	2.268	0.730	1.655	0.003	0.032	0.036	9.401	10.583	10.128	67500	Ccar1	chr10	-	NM_0262	P14653	protein_cc 2610511G	cell divisio	Ccar1
1658	Snord98	1.000	1.000	1.000	1.000	1.000	1.000	0.000	0.000	0.000	10021746	Snord98	chr10	-	NR_02855	.	snoRNA MBII-419	small nucl	Snord98
1659	Tet1	0.337	0.441	0.149	0.000	1.000	0.003	2.751	1.180	0.000	52463	Tet1	chr10	-	NM_0273	P6709	protein_cc 2510010B	tet methy	Tet1
1660	Slc25a16	0.536	1.216	0.652	0.002	0.211	0.033	7.978	7.080	7.362	73132	Slc25a16	chr10	+	NM_1751	P11983	protein_cc 3110021G	solute car	Slc25a16
1661	Dna2	0.739	0.903	0.668	0.002	0.464	0.128	5.891	5.455	5.308	327762	Dna2	chr10	+	NM_1773	P22583	protein_cc Dna2l| E1	DNA repli	Dna2
1662	Rufy2	1.042	0.561	0.584	0.411	0.000	0.021	7.829	7.889	7.055	70432	Rufy2	chr10	+	NM_0274	P14932	protein_cc 2610111N	RUN and F	Rufy2
1663	Hnrnph3	0.980	0.352	0.345	0.865	0.003	0.046	7.398	7.369	5.863	432467	Hnrnph3	chr10	-	NM_0010	P22936	protein_cc AA693301	heteroger	Hnrnph3
1664	Pbld2	0.085	1.312	0.111	0.000	0.282	0.004	10.066	6.504	6.896	67307	Pbld2	chr10	+	NM_0260	P6785	protein_cc 3110049J	phenazine	Pbld2
1665	Pbld1	0.056	1.645	0.092	0.000	0.191	0.021	7.952	3.796	4.514	68371	Pbld1	chr10	+	NM_0267	P12828	protein_cc 0610038K	phenazine	Pbld1
1666	Atoh7	1.000	1.000	1.000	1.000	1.000	1.000	0.000	0.000	0.000	53404	Atoh7	chr10	+	NM_0168	P3190	protein_cc bHLHa13|	atonal bH	Atoh7
1667	Mypn	0.318	0.977	0.311	0.000	1.000	0.009	1.685	0.033	0.000	68802	Mypn	chr10	-	NM_1829	P14222	protein_cc 1110056A	myopallad	Mypn
1668	Herc4	1.063	0.952	1.011	0.276	0.710	0.960	8.372	8.460	8.388	67345	Herc4	chr10	+	NM_0261	P19641	protein_cc 1700056O	hect doma	Herc4
1669	Sirt1	0.884	1.139	1.007	0.091	0.537	0.982	6.812	6.634	6.823	93759	Sirt1	chr10	-	NM_0011	P3285	protein_cc AA673258	sirtuin 1	Sirt1
1670	Dnajc12	0.713	3.007	2.143	0.086	0.173	0.505	7.055	6.567	8.155	30045	Dnajc12	chr10	+	NM_0012	P2299	protein_cc Jdp1| mJD	DnaJ heat	Dnajc12
1671	Ctnna3	1.000	1.000	1.000	1.000	1.000	1.000	0.000	0.000	0.000	216033	Ctnna3	chr10	+	NM_1776	P16728	protein_cc 4930429L	catenin (c	Ctnna3
1672	Lrrtm3	0.716	0.995	0.712	0.182	1.000	0.423	0.490	0.008	0.000	216028	Lrrtm3	chr10	-	NM_1786	P12642	protein_cc 9630044H	leucine ric	Lrrtm3
1673	1700023F	1.000	1.000	1.000	1.000	1.000	1.000	0.000	0.000	0.000	69433	1700023F	chr10	-	NR_03803	.	lncRNA AV205046	RIKEN cDN	1700023F
1674	4930407I1	1.000	1.000	1.000	1.000	1.000	1.000	0.000	0.000	0.000	77198	4930407I1	chr10	+	NR_13107	.	lncRNA .	RIKEN cDN	4930407I1
1675	Gm31763	0.382	0.982	0.375	0.182	1.000	0.423	1.416	0.027	0.000	10263410	Gm31763	chr10	+	NR_11051	.	lncRNA .	predicted	Gm31763
1676	Reep3	1.081	0.826	0.893	0.402	0.003	0.514	10.228	10.341	10.065	28193	Reep3	chr10	-	NM_1786	P2280	protein_cc D10Ucla1	receptor a	Reep3
1677	Jmjd1c	3.907	0.373	1.458	0.000	0.001	0.304	7.413	9.379	7.957	108829	Jmjd1c	chr10	+	NM_2072	P10656	protein_cc 5430433L	jumonji do	Jmjd1c
1678	Nrbf2	1.348	0.822	1.108	0.167	0.292	0.497	7.194	7.625	7.343	641340	Nrbf2	chr10	-	NM_0010	P1042	protein_cc NRBF-2	nuclear re	Nrbf2
1679	Egr2	13.773	0.764	10.525	0.034	0.543	0.172	0.989	4.773	4.385	13654	Egr2	chr10	+	NM_0101	P18156	protein_cc Egr-2| Kro	early grow	Egr2
1680	Ado	1.173	0.913	1.071	0.482	0.666	0.461	7.091	7.322	7.191	211488	Ado	chr10	-	NM_0010	P22117	protein_cc Gm237	2-aminoet	Ado
1681	Zfp365	3.991	1.162	4.639	0.003	0.255	0.004	0.001	1.998	2.215	216049	Zfp365	chr10	-	NM_1786	P12840	protein_cc AI839779|	zinc finger	Zfp365
1682	Rtkn2	2.722	0.367	1.000	0.306	1.000	0.423	0.001	1.445	0.000	170799	Rtkn2	chr10	+	NM_0010	P10782	protein_cc B130039D	rhotekin 2	Rtkn2
1683	Arid5b	0.604	0.622	0.376	0.003	0.021	0.001	10.457	9.730	9.045	71371	Arid5b	chr10	-	NM_0235	P957	protein_cc 4930580B	AT rich int	Arid5b
1684	4930545H	1.000	1.000	1.000	1.000	1.000	1.000	0.000	0.000	0.000	75148	4930545H	chr10	-	NR_04535	.	lncRNA .	RIKEN cDN	4930545H
1685	1700040L	0.090	4.871	0.436	0.003	0.054	0.218	4.722	1.241	3.525	73287	1700040L	chr10	-	NM_0284	P18165	protein_cc .	RIKEN cDN	1700040L
1686	Tmem26	0.549	0.939	0.515	0.118	0.886	0.041	3.568	2.702	2.611	327766	Tmem26	chr10	+	NM_1777	P3056	protein_cc 3230401A	transmem	Tmem26
1687	Rhobtb1	0.215	3.560	0.765	0.000	0.013	0.374	8.795	6.577	8.409	69288	Rhobtb1	chr10	+	NM_0012	P20606	protein_cc 1700008H	Rho-relate	Rhobtb1
1688	Cdk1	2.047	1.616	3.307	0.095	0.088	0.041	4.426	5.460	6.152	12534	Cdk1	chr10	-	NM_0076	P2408	protein_cc Cdc2| Cdc	cyclin-dep	Cdk1
1689	Ank3	0.080	1.577	0.126	0.000	0.319	0.009	8.750	5.107	5.764	11735	Ank3	chr10	+	NM_1706	P15190	protein_cc 2900054D	ankyrin 3,	Ank3
1690	Ccdc6	1.244	0.795	0.989	0.156	0.121	0.943	7.906	8.221	7.890	76551	Ccdc6	chr10	+	NM_0011	P24362	protein_cc 2810012H	coiled-coil	Ccdc6
1691	Mrln	1.000	1.000	1.000	1.000	1.000	1.000	0.000	0.000	0.000	69563	Mrln	chr10	-	NM_0013	P25653	protein_cc 2310015B	myoreguli	Mrln
1692	Slc16a9	0.403	1.802	0.726	0.021	0.001	0.400	4.023	2.710	3.560	66859	Slc16a9	chr10	+	NM_0258	P18122	protein_cc 1200003C	solute car	Slc16a9
1693	Fam13c	0.135	0.948	0.128	0.017	1.000	0.136	2.968	0.078	0.000	71721	Fam13c	chr10	+	NM_0011	P15800	protein_cc 1200015N	family wit	Fam13c
1694	Phyhipl	0.142	0.950	0.135	0.012	1.000	0.117	2.893	0.075	0.000	70911	Phyhipl	chr10	-	NM_0011	P8392	protein_cc 4921522K	phytanoyl	Phyhipl
1695	D630013N	1.000	1.000	1.000	1.000	1.000	1.000	0.000	0.000	0.000	319646	D630013N	chr10	-	NR_04529	.	lncRNA .	RIKEN cDN	D630013N
1696	Bicc1	0.243	2.109	0.513	0.001	0.006	0.071	5.211	3.172	4.248	83675	Bicc1	chr10	-	NM_0313	P16060	protein_cc Bic-C| bpk	BicC family	Bicc1
1697	C730027H	0.160	3.274	0.524	0.000	0.057	0.158	4.470	1.827	3.538	319572	C730027H	chr10	-	NR_03804	.	lncRNA .	RIKEN cDN	C730027H
1698	1700113B	1.000	1.000	1.000	1.000	1.000	1.000	0.000	0.000	0.000	78638	1700113B	chr10	+	NR_13100	.	lncRNA .	RIKEN cDN	1700113B
1699	Tfam	0.692	1.071	0.741	0.048	0.656	0.113	8.977	8.446	8.545	21780	Tfam	chr10	-	NM_0093	P21047	protein_cc AI661103|	transcripti	Tfam
1700	Ube2d1	1.045	0.908	0.949	0.834	0.700	0.919	6.009	6.073	5.934	216080	Ube2d1	chr10	-	NM_1454	P17690	protein_cc UBCH5	ubiquitin-	Ube2d1
1701	Cisd1	0.075	2.199	0.164	0.000	0.001	0.011	11.417	7.671	8.808	52637	Cisd1	chr10	-	NM_1340	P15380	protein_cc AU043990	CDGSH iro	Cisd1
1702	Ipmk	0.233	1.424	0.332	0.000	0.062	0.010	8.968	6.868	7.378	69718	Ipmk	chr10	-	NM_0271	P7179	protein_cc 2410017C	inositol po	Ipmk
1703	1700049L	1.000	1.000	1.000	1.000	1.000	1.000	0.000	0.000	0.000	108950	1700049L	chr10	-	NR_00364	.	misc_RNA .	RIKEN cDN	1700049L1
1704	Zwint	1.124	1.589	1.786	0.205	0.055	0.186	9.275	9.444	10.112	52696	Zwint	chr10	+	NM_0012	P17870	protein_cc 2010007E	ZW10 inte	Zwint
1705	A330049N	1.000	1.000	1.000	1.000	1.000	1.000	0.000	0.000	0.000	327768	A330049N	chr10	-	NR_04064	.	lncRNA .	RIKEN cDN	A330049N
1706	Pcdh15	4.798	0.160	0.768	0.333	1.000	0.420	0.382	2.644	0.000	11994	Pcdh15	chr10	+	NM_0011	P5237	protein_cc av| BB078	protocadh	Pcdh15
1707	Rsph14	1.000	1.000	1.000	1.000	1.000	1.000	0.000	0.000	0.000	71236	Rsph14	chr10	-	NM_0011	P14652	protein_cc 4933431K	radial spo	Rsph14
1708	Gnaz	2.134	0.469	1.000	0.305	1.000	0.423	0.000	1.094	0.000	14687	Gnaz	chr10	+	NM_0103	P17982	protein_cc AI847979|	guanine n	Gnaz
1709	Rab36	0.405	3.006	1.218	0.231	0.001	0.782	2.844	1.540	3.128	76877	Rab36	chr10	+	NM_0297	P9643	protein_cc 6330593L	RAB36, m	Rab36
1710	Bcr	0.544	0.825	0.449	0.018	0.345	0.065	7.691	6.814	6.536	110279	Bcr	chr10	+	NM_0010	P12771	protein_cc 51334000	breakpoin	Bcr
1711	Speccl1	1.040	0.881	0.916	0.852	0.543	0.635	7.331	7.387	7.204	74392	Speccl1	chr10	+	NM_1534	P20402	protein_cc 4930470P	sperm ant	Speccl1
1712	Adora2a	4.877	0.536	2.615	0.000	0.000	0.011	3.500	5.786	4.887	11540	Adora2a	chr10	+	NM_0096	P18152	protein_cc A2AAR| A	adenosine	Adora2a
1713	Gm5779	2.263	1.004	2.272	0.145	0.990	0.067	0.588	1.767	1.772	544707	Gm5779	chr10	+	NR_03360	.	misc_RNA EG544707	predicted	Gm5779
1714	Upb1	0.247	1.170	0.289	0.000	0.116	0.004	12.379	10.360	10.586	103149	Upb1	chr10	+	NM_1339	P3489	protein_cc AI195023	ureidopro	Upb1
1715	4933407G	1.000	1.000	1.000	1.000	1.000	1.000	0.000	0.000	0.000	71125	4933407G	chr10	+	NR_04584	.	lncRNA .	RIKEN cDN	4933407G
1716	Gucd1	0.440	1.559	0.686	0.000	0.000	0.067	9.557	8.372	9.013	68778	Gucd1	chr10	-	NM_1751	P10743	protein_cc 1110038D	guanylyl c	Gucd1

1717	Snrpd3	1.040	0.839	0.873	0.860	0.473	0.177	9.328	9.385	9.132	67332	Snrpd3	chr10	+	NM_0260	P19858	protein_cc	1700043E	small nucl	Snrpd3
1718	Lrrc75b	2.074	0.794	1.648	0.396	0.728	0.288	0.415	1.468	1.136	192734	Lrrc75b	chr10	-	NM_1988	P9856	protein_cc	AI646023	leucine ric	Lrrc75b
1719	Ggt1	1.620	2.413	3.909	0.285	0.284	0.423	0.001	0.697	1.968	14598	Ggt1	chr10	+	NM_0013	P14926	protein_cc	CD224| d	gamma-gl	Ggt1
1720	Ggt5	0.366	0.351	0.129	0.052	0.001	0.150	4.278	2.829	1.320	23887	Ggt5	chr10	+	NM_0118	P6963	protein_cc	AI551243	gamma-gl	Ggt5
1721	Susd2	4.057	0.987	4.003	0.005	0.956	0.135	2.038	4.059	4.040	71733	Susd2	chr10	-	NM_0278	P21204	protein_cc	1200011D	sushi dom	Susd2
1722	Cabin1	1.051	0.822	0.864	0.751	0.350	0.473	6.949	7.022	6.738	104248	Cabin1	chr10	-	NM_1725	P1440	protein_cc	A330070N	calcineurin	Cabin1
1723	Ddt	0.448	1.141	0.511	0.000	0.058	0.001	12.663	11.504	11.694	13202	Ddt	chr10	-	NM_0100	P25660	protein_cc	C78655	D-dopachr	Ddt
1724	Gstt3	0.175	0.715	0.125	0.000	0.015	0.001	12.080	9.568	9.084	103140	Gstt3	chr10	-	NM_1339	P5055	protein_cc	AI118089	glutathion	Gstt3
1725	Gstt1	0.418	0.697	0.292	0.000	0.009	0.003	11.738	10.481	9.960	14871	Gstt1	chr10	-	NM_0081	P2810	protein_cc	AI255817	glutathion	Gstt1
1726	Gstt4	1.000	1.000	1.000	1.000	1.000	1.000	0.000	0.000	0.000	75886	Gstt4	chr10	-	NM_0294	P112	protein_cc	4930583C	glutathion	Gstt4
1727	Gstt2	0.270	1.224	0.330	0.001	0.130	0.031	9.628	7.736	8.027	14872	Gstt2	chr10	-	NM_0103	P18340	protein_cc	AI266894	glutathion	Gstt2
1728	Mif	1.224	0.779	0.953	0.082	0.035	0.650	10.927	11.219	10.858	17319	Mif	chr10	-	NM_0107	P13116	protein_cc	GIF| Glif	macropha	Mif
1729	Derl3	2.860	1.504	4.300	0.030	0.408	0.301	4.308	5.825	6.413	70377	Derl3	chr10	+	NM_0244	P776	protein_cc	1810006I2	Der1-like	Derl3
1730	Smarcb1	0.988	0.926	0.915	0.953	0.799	0.800	7.489	7.471	7.361	20587	Smarcb1	chr10	-	NM_0110	P9238	protein_cc	AU020204	SWI/SNF r	Smarcb1
1731	Mmp11	0.238	1.617	0.385	0.017	0.419	0.204	3.253	1.181	1.875	17385	Mmp11	chr10	-	NM_0130	P20220	protein_cc	MMP-11|	matrix me	Mmp11
1732	Chchd10	0.362	1.230	0.446	0.000	0.013	0.003	13.925	12.461	12.759	103172	Chchd10	chr10	+	NM_1753	P14855	protein_cc	1620401E	coiled-coil	Chchd10
1733	Gm867	0.403	1.685	0.680	0.006	0.038	0.132	12.278	10.969	11.721	333670	Gm867	chr10	-	NM_0012	P13609	protein_cc	Gm736	predicted	Gm867
1734	Vpreb3	2.601	0.071	0.184	0.033	1.000	0.422	2.440	3.819	0.000	22364	Vpreb3	chr10	+	NM_0095	P25580	protein_cc	8HS-20| A	pre-B lymp	Vpreb3
1735	Gm5134	1.000	1.000	1.000	1.000	1.000	1.000	0.000	0.000	0.000	333669	Gm5134	chr10	+	NM_1986	P18437	protein_cc	EG333669	predicted	Gm5134
1736	Zfp280b	1.009	1.514	1.528	0.985	0.344	0.049	5.414	5.427	6.026	64453	Zfp280b	chr10	+	NM_1774	P23890	protein_cc	D10Jhu82	zinc finger	Zfp280b
1737	Slc5a4b	1.000	1.000	1.000	1.000	1.000	1.000	0.000	0.000	0.000	64454	Slc5a4b	chr10	-	NM_0232	P6953	protein_cc	2010104G	solute car	Slc5a4b
1738	Slc5a4a	1.000	1.000	1.000	1.000	1.000	1.000	0.000	0.000	0.000	64452	Slc5a4a	chr10	+	NM_1331	P13473	protein_cc	SGLT3-a	solute car	Slc5a4a
1739	Prmt2	2.365	0.686	1.622	0.004	0.086	0.369	3.797	5.040	4.495	15468	Prmt2	chr10	-	NM_1331	P587	protein_cc	AI504737	protein ar	Prmt2
1740	S100b	0.383	4.079	1.561	0.001	0.099	0.599	1.450	0.064	2.092	20203	S100b	chr10	+	NM_0091	P834	protein_cc	AI850290	S100 prot	S100b
1741	Dip2a	1.059	1.176	1.245	0.650	0.356	0.490	4.031	4.113	4.347	64451	Dip2a	chr10	-	NM_0010	P24169	protein_cc	4931420H	disco inter	Dip2a
1742	Pcnt	0.650	1.204	0.782	0.000	0.042	0.110	7.751	7.129	7.396	18541	Pcnt	chr10	-	NM_0087	P3192	protein_cc	AW47609	pericentri	Pcnt
1743	1700094J0	0.671	0.948	0.636	0.041	0.769	0.080	6.055	5.479	5.402	67523	1700094J0	chr10	+	NR_04058	.	lncRNA	.	RIKEN cDN	1700094J0
1744	2610028H	1.290	1.804	2.327	0.499	0.455	0.568	1.423	1.790	2.642	76964	2610028H	chr10	+	NM_0298	P12871	protein_cc	ORF67	RIKEN cDN	2610028H
1745	Ybey	0.693	1.709	1.184	0.086	0.032	0.250	5.574	5.044	5.818	216119	Ybey	chr10	-	NM_1725	P10457	protein_cc	1110068N	ybeY meta	Ybey
1746	Mcm3ap	1.011	0.858	0.867	0.895	0.210	0.356	7.040	7.056	6.834	54387	Mcm3ap	chr10	+	NM_0194	P5397	protein_cc	GANP| mh	minichrom	Mcm3ap
1747	Lss	1.463	2.133	3.121	0.015	0.000	0.007	7.362	7.911	9.004	16987	Lss	chr10	+	NM_1460	P392	protein_cc	2810025N	lanosterol	Lss
1748	4930483K	1.717	3.720	6.386	0.539	0.016	0.036	1.023	1.803	3.698	74953	4930483K	chr10	-	NR_04535	.	lncRNA	.	RIKEN cDN	4930483K
1749	Spatc1l	1.000	1.000	1.000	1.000	1.000	1.000	0.000	0.000	0.000	76573	Spatc1l	chr10	+	NM_0296	P4276	protein_cc	1700022B	spermatog	Spatc1l
1750	Ftcd	0.293	2.123	0.623	0.000	0.000	0.077	11.956	10.186	11.272	14317	Ftcd	chr10	+	NM_0808	P1367	protein_cc	.	formimino	Ftcd
1751	Col6a2	0.130	2.286	0.298	0.001	0.001	0.045	5.197	2.258	3.451	12834	Col6a2	chr10	-	NM_1460	P24285	protein_cc	Col6a-2	collagen, t	Col6a2
1752	Col6a1	0.341	1.162	0.396	0.000	0.306	0.049	4.713	3.161	3.378	12833	Col6a1	chr10	-	NM_0099	P24309	protein_cc	AI747156	collagen, t	Col6a1
1753	Pcbp3	1.174	0.994	1.168	0.608	0.979	0.658	3.601	3.833	3.824	59093	Pcbp3	chr10	-	NM_0215	P6770	protein_cc	AlphaCP-3	poly(rC) bi	Pcbp3
1754	Gm10787	1.026	3.463	3.555	0.000	0.000	0.002	0.001	0.038	1.830	10003847	Gm10787	chr10	+	NR_04588	.	lncRNA	ENSMUSG	predicted	Gm10787
1755	Slc19a1	0.520	2.697	1.403	0.204	0.006	0.456	5.596	4.653	6.085	20509	Slc19a1	chr10	+	NM_0011	P6750	protein_cc	AI323572	solute car	Slc19a1
1756	Col18a1	0.180	1.078	0.194	0.000	0.315	0.001	11.949	9.474	9.582	12822	Col18a1	chr10	-	NM_0099	P22877	protein_cc	.	collagen, t	Col18a1
1757	Gm10941	0.383	2.148	0.824	0.017	0.187	0.766	1.426	0.043	1.146	10016987	Gm10941	chr10	-	NR_02694	.	lncRNA	ENSMUSG	predicted	Gm10941
1758	Pofut2	1.564	0.678	1.060	0.004	0.003	0.616	7.139	7.785	7.224	80294	Pofut2	chr10	+	NM_0302	P6318	protein_cc	2310011G	protein O-	Pofut2
1759	Adarb1	1.124	0.714	0.802	0.495	0.109	0.221	5.571	5.739	5.253	110532	Adarb1	chr10	-	NR_00442	.	protein_cc	1700057H	adenosine	Adarb1
1760	Gm17769	2.755	0.836	2.304	0.304	0.803	0.423	0.001	1.463	1.205	10004565	Gm17769	chr10	+	NR_02737	.	lncRNA	.	predicted	Gm17769
1761	Fam207a	0.979	1.165	1.140	0.909	0.387	0.506	8.172	8.141	8.361	108707	Fam207a	chr10	-	NM_1339	P10949	protein_cc	1810008A	family wit	Fam207a
1762	Itgb2	3.954	0.779	3.079	0.097	0.562	0.080	5.112	7.095	6.735	16414	Itgb2	chr10	+	NM_0084	P11638	protein_cc	2E6| AI52	integrin be	Itgb2
1763	Pttg1ip	0.416	1.138	0.473	0.000	0.063	0.000	11.779	10.514	10.700	108705	Pttg1ip	chr10	+	NM_1459	P10878	protein_cc	1810010L	pituitary t	Pttg1ip
1764	Sumo3	0.547	1.628	0.890	0.026	0.008	0.613	12.769	11.898	12.601	20610	Sumo3	chr10	+	NM_0199	P23089	protein_cc	2810014B	small ubiq	Sumo3
1765	Ube2g2	1.810	1.096	1.984	0.028	0.599	0.116	8.335	9.191	9.323	22213	Ube2g2	chr10	+	NM_0198	P12316	protein_cc	1110003O	ubiquitin-	Ube2g2
1766	Tspear	1.000	1.000	1.000	1.000	1.000	1.000	0.000	0.000	0.000	252974	Tspear	chr10	+	NM_0012	P21799	protein_cc	C330046G	thrombos	Tspear
1767	Gm19402	1.000	1.000	1.000	1.000	1.000	1.000	0.000	0.000	0.000	10050283	Gm19402	chr10	-	NM_0012	P16977	protein_cc	.	predicted	Gm19402
1768	Gm10272	1.000	1.000	1.000	1.000	1.000	1.000	0.000	0.000	0.000	16697	Gm10272	chr10	+	NR_02683	.	misc_RNA	ENSMUSG	predicted	Gm10272
1769	Gm10024	1.000	1.000	1.000	1.000	1.000	1.000	0.000	0.000	0.000	10000961	Gm10024	chr10	+	NM_0010	P14660	protein_cc	ENSMUSG	predicted	Gm10024
1770	Gm10142	1.000	1.000	1.000	1.000	1.000	1.000	0.000	0.000	0.000	10050292	Gm10142	chr10	+	NM_0012	P20665	protein_cc	ENSMUSG	predicted	Gm10142
1771	Krtap12-1	1.000	1.000	1.000	1.000	1.000	1.000	0.000	0.000	0.000	16694	Krtap12-1	chr10	+	NM_0106	P25403	protein_cc	D10Jhu14	keratin as	Krtap12-1
1772	Gm10100	1.000	1.000	1.000	1.000	1.000	1.000	0.000	0.000	0.000	10050295	Gm10100	chr10	+	NM_0012	P21044	protein_cc	ENSMUSG	predicted	Gm10100
1773	Gm3238	1.000	1.000	1.000	1.000	1.000	1.000	0.000	0.000	0.000	10004126	Gm3238	chr10	+	NM_0011	P21509	protein_cc	10004126	predicted	Gm3238
1774	Gm19668	1.000	1.000	1.000	1.000	1.000	1.000	0.000	0.000	0.000	10050338	Gm19668	chr10	+	NM_0012	P8185	protein_cc	.	predicted	Gm19668
1775	Gm2696	1.000	1.000	1.000	1.000	1.000	1.000	0.000	0.000	0.000	10004029	Gm2696	chr10	+	NM_0012	P23658	protein_cc	10004029	predicted	Gm2696
1776	Krtap10-4	1.000	1.000	1.000	1.000	1.000	1.000	0.000	0.000	0.000	10019103	Krtap10-4	chr10	-	NM_0011	P13936	protein_cc	675238| A	keratin as	Krtap10-4
1777	Krtap10-1	1.000	1.000	1.000	1.000	1.000	1.000	0.000	0.000	0.000	544710	Krtap10-1	chr10	+	NM_0010	P4437	protein_cc	EG544710	keratin as	Krtap10-1
1778	Gm10318	1.000	1.000	1.000	1.000	1.000	1.000	0.000	0.000	0.000	622629	Gm10318	chr10	+	NM_0011	P3224	protein_cc	EG622629	predicted	Gm10318
1779	Gm3285	1.000	1.000	1.000	1.000	1.000	1.000	0.000	0.000	0.000	10004135	Gm3285	chr10	+	NM_0011	P13192	protein_cc	10004135	predicted	Gm3285
1780	1700009J0	0.716	0.995	0.712	0.182	1.000	0.423	0.490	0.008	0.000	75188	1700009J0	chr10	+	NR_01554	.	lncRNA	4930535B	RIKEN cDN	1700009J0
1781	Lrrc3	0.080	3.357	0.270	0.000	0.003	0.003	8.971	5.334	7.081	237387	Lrrc3	chr10	-	NM_1451	P26547	protein_cc	1300011L	leucine ric	Lrrc3
1782	Trpm2	0.462	0.194	0.090	0.126	0.085	0.134	4.946	3.833	1.468	28240	Trpm2	chr10	-	NM_1383	P4038	protein_cc	9830168K	transient r	Trpm2
1783	1810043G	0.351	3.865	1.356	0.028	0.010	0.440	5.162	3.651	5.601	67884	1810043G	chr10	-	NM_0264	P7208	protein_cc	AV026620	RIKEN cDN	1810043G
1784	Pfkl	3.607	0.375	1.352	0.003	0.005	0.351	7.822	9.673	8.258	18641	Pfkl	chr10	-	NM_0088	P15775	protein_cc	AA407869	phosphofr	Pfkl
1785	Aire	1.000	1.000	1.000	1.000	1.000	1.000	0.000	0.000	0.000	11634	Aire	chr10	-	NM_0012	P24691	protein_cc	.	autoimmu	Aire
1786	Dnmt3l	2.191	0.817	1.790	0.000	0.453	0.423	0.001	1.132	0.840	54427	Dnmt3l	chr10	+	NM_0010	P8041	protein_cc	D6Ertd14e	DNA (cyto	Dnmt3l
1787	Icosl	2.487	0.435	1.081	0.070	0.057	0.935	2.936	4.251	3.048	50723	Icosl	chr10	+	NM_0157	P13750	protein_cc	AU044799	icos ligand	Icosl
1788	D10Jhu81	0.334	1.214	0.406	0.000	0.021	0.008	10.995	9.414	9.694	28295	D10Jhu81	chr10	-	NM_1386	P25686	protein_cc	C21orf33	DNA segm	D10Jhu81e
1789	Pwp2	1.255	1.178	1.479	0.086	0.657	0.602	5.882	6.210	6.446	110816	Pwp2	chr10	-	NM_0295	P9754	protein_cc	6530411D	PWP2 per	Pwp2
1790	Trappc10	1.524	0.832	1.268	0.032	0.210	0.198	6.977	7.585	7.319	216131	Trappc10	chr10	-	NM_0010	P19545	protein_cc	B230307C	trafficking	Trappc10
1791	Agpat3	0.361	1.316	0.475	0.000	0.011	0.013	10.130	8.660	9.057	28169	Agpat3	chr10	-	NM_0530	P5185	protein_cc	AW06125	1-acylglyc	Agpat3
1792	Rrp1	1.178	0.897	1.057	0.379	0.548	0.813	8.638	8.874	8.718	18114	Rrp1	chr10	-	NM_0109	P2673	protein_cc	AL033305	ribosomal	Rrp1
1793	Cstb	1.693	1.115	1.888	0.048	0.447	0.021	9.508	10.267	10.425	13014	Cstb	chr10	+	NM_0077	P4962	protein_cc	AA960480	cystatin B	Cstb
1794	Pdxk	0.469	1.656	0.776	0.010	0.006	0.313	8.858	7.765	8.493	216134	Pdxk	chr10	-	NM_1721	P14804	protein_cc	2310030D	pyridoxal	Pdxk
1795	Ilvbl	0.272	1.449	0.394	0.000	0.180	0.027	8.774	6.896	7.431	216136	Ilvbl	chr10	+	NM_1737	P21920	protein_cc	5830463I2	ilvB (bacte	Ilvbl
1796	Syde1	1.689	0.767	1.296	0.346	0.565	0.359	4.331	5.087	4.705	71709	Syde1	chr10	-	NM_0278	P11547	protein_cc	1200008N	synapse d	Syde1
1797	Olfr1357	1.000	1.000	1.000	1.000	1.000	1.000	0.000	0.000	0.000	257883	Olfr1357	chr10	-	NM_0010	P9147	protein_cc	MOR128-3	olfactory r	Olfr1357
1798	Casp14	1.000	1.000	1.000	1.000	1.000	1.000	0.000	0.000	0.000	12365	Casp14	chr10	-	NM_0098	P9573	protein_cc	MICE| mir	caspase 14	Casp14
1799	Ccdc105	1.005	1.405	1.412	0.000	0.182	0.423	0.000	0.008	0.498	70976	Ccdc105	chr10	-	NM_0276	P15065	protein_cc	4931413A	coiled-coil	Ccdc105
1800	Slc1a6	1.000	1.000	1.000	1.000	1.000	1.000	0.000	0.000	0.000	20513	Slc1a6	chr10	+	NM_0092	P24994	protein_cc	EAAT4	solute car	Slc1a6
1801	Olfr1356	1.000	1.000	1.000	1.000	1.000	1.000	0.000	0.000	0.000	258305	Olfr1356	chr10	-	NM_1463	P23912	protein_cc	MOR142-1	olfactory r	Olfr1356
1802	Olfr1355	1.000	1.000	1.000	1.000	1.000	1.000	0.000	0.000	0.000	257734	Olfr1355	chr10	+	NM_2075	P25895	protein_cc	GA_x5J8B	olfactory r	Olfr1355
1803	Olfr1354	1.000	1.000	1.000	1.000	1.000	1.000	0.000	0.000	0.000	259163	Olfr1354	chr10	+	NM_0011	P2858	protein_cc	EG257869	olfactory r	Olfr1354
1804	Olfr8	1.000	1.000	1.000	1.000	1.000	1.000	0.000	0.000	0.000	18372	Olfr8	chr10	+	NM_2072	P8986	protein_cc	GA_x5J8B	olfactory r	Olfr8
1805	Olfr1353	1.000	1.000	1.000	1.000	1.000	1.000	0.000	0.000	0.000	259044	Olfr1353	chr10	+	NM_1470	P7264	protein_cc	MOR139-2	olfactory r	Olfr1353
1806	Olfr1352	1.000	1.000	1.000	1.000	1.000	1.000	0.000	0.000	0.000	259074	Olfr1352	chr10	+	NM_1470	P24713	protein_cc	MOR139-1	olfactory r	Olfr1352
1807	Olfr1351	1.000	1.000	1.000	1.000	1.000	1.000	0.000	0.000	0.000	259042	Olfr1351	chr10	+	NM_1470	P14795	protein_cc	MOR139-4	olfactory r	Olfr1351

1808	Olfr57	1.000	1.000	1.000	1.000	1.000	1.000	0.000	0.000	0.000	18357	Olfr57	chr10	+	NM_1470	P3396	protein_cc	IF12| MOI	olfactory r	Olfr57
1809	2610008E	0.913	1.180	1.077	0.858	0.760	0.844	5.746	5.615	5.853	72128	2610008E	chr10	-	NM_0010	P18581	protein_cc	.	RIKEN cDN	2610008E1
1810	Vmn2r80	1.000	1.000	1.000	1.000	1.000	1.000	0.000	0.000	0.000	624765	Vmn2r80	chr10	+	NM_0011	P3009	protein_cc	EG624765	vomerona	Vmn2r80
1811	Vmn2r81	1.000	1.000	1.000	1.000	1.000	1.000	0.000	0.000	0.000	216144	Vmn2r81	chr10	+	NM_1759	P21336	protein_cc	EC1-V2R|	vomerona	Vmn2r81
1812	Vmn2r82	1.000	1.000	1.000	1.000	1.000	1.000	0.000	0.000	0.000	624845	Vmn2r82	chr10	+	NM_0011	P8899	protein_cc	EG624845	vomerona	Vmn2r82
1813	Vmn2r83	0.716	0.995	0.712	0.182	1.000	0.423	0.490	0.008	0.000	625029	Vmn2r83	chr10	+	NM_0011	P24635	protein_cc	EG625029	vomerona	Vmn2r83
1814	Ppap2c	0.796	1.863	1.482	0.019	0.005	0.131	7.972	7.642	8.540	.	Ppap2c	chr10	-	NM_0158	P13480	.	.	.	Ppap2c
1815	Mier2	2.169	0.904	1.961	0.044	0.652	0.052	5.806	6.923	6.778	70427	Mier2	chr10	-	NM_0274	P8699	protein_cc	2700087H	MIER fami	Mier2
1816	Theg	1.000	1.000	1.000	1.000	1.000	1.000	0.000	0.000	0.000	21830	Theg	chr10	-	NM_1992	P20216	protein_cc	.	testicular	Theg
1817	C2cd4c	0.467	2.184	1.020	0.185	0.017	0.976	1.137	0.038	1.165	237397	C2cd4c	chr10	-	NM_1986	P9515	protein_cc	4932409I2	C2 calcium	C2cd4c
1818	Shc2	1.289	0.508	0.655	0.726	0.420	0.604	1.101	1.467	0.491	216148	Shc2	chr10	-	NM_0010	P24206	protein_cc	6720466E	SHC (Src h	Shc2
1819	Odf3l2	1.000	1.000	1.000	1.000	1.000	1.000	0.000	0.000	0.000	382384	Odf3l2	chr10	-	NM_0010	P9682	protein_cc	Gm1157	outer den	Odf3l2
1820	Madcam1	2.180	1.064	2.319	0.302	0.919	0.423	0.001	1.125	1.214	17123	Madcam1	chr10	+	NM_0135	P13976	protein_cc	AV211525	mucosal v	Madcam1
1821	Tpgs1	1.070	1.104	1.181	0.232	0.062	0.145	7.525	7.623	7.765	110012	Tpgs1	chr10	+	NM_1489	P19143	protein_cc	AV005629	tubulin po	Tpgs1
1822	Cdc34	1.632	1.008	1.646	0.000	0.830	0.024	9.292	9.998	10.010	216150	Cdc34	chr10	+	NM_1776	P5798	protein_cc	AI327276|	cell divisio	Cdc34
1823	Gzmm	0.552	0.990	0.547	0.182	1.000	0.423	0.871	0.015	0.000	16904	Gzmm	chr10	+	NR_12632	.	protein_cc	Lmet1| M	granzyme	Gzmm
1824	Bc1	1.005	1.405	1.412	0.000	0.182	0.423	0.000	0.008	0.498	10056845	Bc1	chr10	-	NR_03808	.	lncRNA	Gm24367	brain cyto	Bc1
1825	Bsg	0.638	0.788	0.502	0.065	0.343	0.057	10.942	10.293	9.949	12215	Bsg	chr10	+	NM_0010	P15184	protein_cc	AI115436|	basigin	Bsg
1826	Hcn2	0.581	1.523	0.884	0.394	0.548	0.904	1.935	1.151	1.757	15166	Hcn2	chr10	+	NM_0082	P4505	protein_cc	BCNG2| H	hyperpola	Hcn2
1827	Polrmt	0.608	1.316	0.800	0.001	0.112	0.306	6.629	5.911	6.307	216151	Polrmt	chr10	-	NM_1725	P2154	protein_cc	1110018N	polymeras	Polrmt
1828	Fgf22	1.000	1.000	1.000	1.000	1.000	1.000	0.000	0.000	0.000	67112	Fgf22	chr10	+	NM_0233	P13998	protein_cc	2210414E	fibroblast	Fgf22
1829	Rnf126	0.848	1.289	1.093	0.078	0.181	0.747	6.803	6.565	6.931	70294	Rnf126	chr10	-	NR_02750	.	protein_cc	2610010O	ring finger	Rnf126
1830	Fstl3	3.490	1.745	6.090	0.011	0.018	0.020	2.037	3.840	4.644	83554	Fstl3	chr10	+	NM_0313	P18638	protein_cc	E030038F	follistatin-	Fstl3
1831	Prss57	2.746	1.797	4.934	0.024	0.021	0.039	2.384	3.841	4.687	73106	Prss57	chr10	-	NM_0010	P4499	protein_cc	2900092M	protease,	Prss57
1832	Palm	0.815	1.042	0.849	0.374	0.933	0.808	2.515	2.221	2.280	18483	Palm	chr10	+	NM_0231	P16563	protein_cc	.	paralemm	Palm
1833	Misp	6.772	0.592	4.010	0.172	0.514	0.391	0.357	3.117	2.360	78906	Misp	chr10	+	NM_0302	P18129	protein_cc	9130017N	mitotic sp	Misp
1834	E130317F	7.614	1.672	12.728	0.010	0.377	0.283	0.002	2.931	3.672	414101	E130317F	chr10	-	NR_02944	.	lncRNA	.	RIKEN cDN	E130317F2
1835	Ptbp1	1.785	0.948	1.692	0.000	0.295	0.040	8.095	8.931	8.854	19205	Ptbp1	chr10	+	NM_0010	P3584	protein_cc	AA407203	polypyrim	Ptbp1
1836	Lppr3	2.170	0.461	1.000	0.000	1.000	0.423	0.000	1.118	0.000	.	Lppr3	chr10	-	NM_0011	P12696	.	.	.	Lppr3
1837	Prtn3	3.405	0.699	2.381	0.237	0.580	0.158	1.267	3.035	2.519	19152	Prtn3	chr10	+	NM_0111	P23150	protein_cc	mPR3| PR	proteinase	Prtn3
1838	Elane	0.504	0.990	0.499	0.181	1.000	0.423	1.002	0.015	0.000	50701	Elane	chr10	+	NM_0157	P208	protein_cc	Ela2| F43	elastase, r	Elane
1839	Cfd	0.098	31.570	3.097	0.008	0.033	0.309	4.158	0.809	5.789	11537	Cfd	chr10	+	NM_0134	P22465	protein_cc	Adn| DF	compleme	Cfd
1840	Med16	0.549	1.896	1.042	0.006	0.001	0.784	7.342	6.478	7.401	216154	Med16	chr10	-	NM_0011	P23108	protein_cc	95kDa| A	mediator	Med16
1841	R3hdm4	0.358	2.037	0.728	0.000	0.003	0.006	8.313	6.830	7.856	109284	R3hdm4	chr10	-	NM_1779	P23969	protein_cc	AI503502|	R3H doma	R3hdm4
1842	Kiss1r	0.970	1.049	1.018	0.948	0.920	0.987	1.544	1.499	1.569	114229	Kiss1r	chr10	+	NM_0532	P1813	protein_cc	Gpr54| Ki	KISS1 rece	Kiss1r
1843	Arid3a	2.795	1.031	2.883	0.007	0.942	0.400	4.410	5.893	5.937	13496	Arid3a	chr10	+	NM_0078	P26715	protein_cc	Bright| Dr	AT rich int	Arid3a
1844	Wdr18	0.548	1.630	0.892	0.003	0.003	0.486	7.784	6.915	7.620	216156	Wdr18	chr10	+	NM_1754	P12426	protein_cc	2310012I1	WD repea	Wdr18
1845	Grin3b	0.676	1.994	1.348	0.001	0.023	0.370	7.487	6.922	7.918	170483	Grin3b	chr10	+	NM_1304	P18275	protein_cc	GluN3B| N	glutamate	Grin3b
1846	Tmem259	0.691	2.040	1.409	0.000	0.026	0.349	7.593	7.059	8.087	216157	Tmem259	chr10	-	NM_0010	P19820	protein_cc	AC087114	transmem	Tmem259
1847	Cnn2	2.752	0.722	1.986	0.000	0.159	0.319	5.190	6.651	6.180	12798	Cnn2	chr10	+	NM_0077	P21226	protein_cc	AA408047	calponin 2	Cnn2
1848	Abca7	0.860	0.704	0.606	0.448	0.235	0.096	4.522	4.305	3.799	27403	Abca7	chr10	+	NM_0138	P25032	protein_cc	Abc51| AE	ATP-bindi	Abca7
1849	Hmha1	0.469	1.009	0.473	0.005	0.914	0.072	6.001	4.908	4.921	70719	Hmha1	chr10	+	NM_0011	P3274	protein_cc	6330406L	histocomp	Hmha1
1850	Polr2e	0.646	1.761	1.137	0.009	0.019	0.610	8.854	8.223	9.040	66420	Polr2e	chr10	-	NM_0255	P14883	protein_cc	2410021N	polymeras	Polr2e
1851	Gpx4	0.574	1.947	1.118	0.002	0.001	0.436	9.083	8.283	9.244	625249	Gpx4	chr10	+	NR_11034	.	protein_cc	GPx-4| GS	glutathion	Gpx4
1852	Sbno2	6.967	0.681	4.746	0.000	0.001	0.004	7.007	9.808	9.254	216161	Sbno2	chr10	-	NM_1834	P8306	protein_cc	BC019206	strawberr	Sbno2
1853	Stk11	0.567	0.842	0.477	0.000	0.227	0.037	9.944	9.125	8.877	20869	Stk11	chr10	+	NM_0013	P26193	protein_cc	AA408040	serine/thr	Stk11
1854	Dos	0.519	0.898	0.466	0.000	0.564	0.068	9.753	8.807	8.651	.	Dos	chr10	-	NM_0011	P20728	.	.	.	Dos
1855	Atp5d	0.529	1.218	0.644	0.001	0.060	0.021	11.195	10.275	10.559	66043	Atp5d	chr10	+	NM_0253	P4028	protein_cc	0610008F	ATP synth	Atp5d
1856	Midn	1.213	0.892	1.082	0.507	0.645	0.733	10.674	10.952	10.788	59090	Midn	chr10	+	NM_0215	P7896	protein_cc	3000003C	midnolin	Midn
1857	Cirbp	0.501	5.917	2.965	0.002	0.048	0.302	7.627	6.630	9.195	12696	Cirbp	chr10	+	NM_0077	P81	protein_cc	Cirp| R74	cold induc	Cirbp
1858	1600002K	1.673	1.206	2.017	0.141	0.407	0.024	5.283	6.026	6.296	69770	1600002K	chr10	+	NM_0272	P9032	protein_cc	.	RIKEN cDN	1600002K
1859	Efna2	0.613	0.969	0.594	0.251	0.958	0.350	1.859	1.154	1.108	13637	Efna2	chr10	+	NM_0079	P17139	protein_cc	CEK7L| Elf	ephrin A2	Efna2
1860	Mum1	0.602	0.890	0.536	0.025	0.610	0.023	6.655	5.923	5.754	68114	Mum1	chr10	+	NM_0234	P8019	protein_cc	2610019J	melanoma	Mum1
1861	Ndufs7	0.375	1.199	0.450	0.001	0.103	0.035	9.581	8.168	8.430	75406	Ndufs7	chr10	+	NM_0292	P9610	protein_cc	1010001M	NADH deh	Ndufs7
1862	Gamt	0.243	0.717	0.174	0.001	0.377	0.044	11.004	8.962	8.482	14431	Gamt	chr10	-	NM_0102	P3409	protein_cc	AA571402	guanidino	Gamt
1863	Dazap1	0.823	1.021	0.841	0.095	0.837	0.196	9.736	9.455	9.485	70248	Dazap1	chr10	+	NM_1331	P25194	protein_cc	2410042M	DAZ assoc	Dazap1
1864	Rps15	1.078	1.293	1.394	0.019	0.001	0.011	10.481	10.590	10.961	20054	Rps15	chr10	+	NM_0090	P2742	protein_cc	rig	ribosomal	Rps15
1865	Apc2	4.603	0.357	1.645	0.298	0.382	0.454	1.020	3.223	1.739	23805	Apc2	chr10	+	NM_0117	P23483	protein_cc	AI852447|	adenomat	Apc2
1866	2310011J	1.297	1.421	1.843	0.006	0.133	0.215	6.940	7.315	7.822	66374	2310011J	chr10	-	NM_0255	P6967	protein_cc	AI452186|	RIKEN cDN	2310011J0
1867	Pcsk4	0.015	19.628	0.298	0.000	0.000	0.001	6.858	0.817	5.112	18551	Pcsk4	chr10	-	NM_0087	P23488	protein_cc	AI647044|	proprotei	Pcsk4
1868	Reep6	0.060	2.389	0.144	0.000	0.014	0.004	12.768	8.719	9.976	70335	Reep6	chr10	+	NM_0012	P15583	protein_cc	0610011M	receptor a	Reep6
1869	Adamtsl5	0.390	0.616	0.240	0.025	1.000	0.065	2.058	0.698	0.000	66548	Adamtsl5	chr10	-	NR_10434	.	protein_cc	2010109H	ADAMTS-l	Adamtsl5
1870	Plk5	0.392	1.052	0.413	0.012	0.929	0.214	3.155	1.805	1.878	216166	Plk5	chr10	+	NM_1831	P25923	protein_cc	6330514A	polo-like k	Plk5
1871	Mex3d	1.253	0.903	1.132	0.002	0.668	0.789	3.974	4.300	4.153	237400	Mex3d	chr10	-	NM_1986	P14908	protein_cc	BC059858	mex3 RNA	Mex3d
1872	Mbd3	0.876	1.209	1.059	0.129	0.302	0.841	7.615	7.424	7.698	17192	Mbd3	chr10	-	NM_0013	P9168	protein_cc	AI181826|	methyl-Cp	Mbd3
1873	Uqcr11	0.781	1.126	0.879	0.000	0.000	0.030	10.725	10.368	10.539	66594	Uqcr11	chr10	-	NM_0256	P22564	protein_cc	0710008D	ubiquinol-	Uqcr11
1874	Tcf3	0.781	1.142	0.892	0.139	0.478	0.639	6.679	6.322	6.514	21423	Tcf3	chr10	-	NM_0011	P11863	protein_cc	A1| AA40	transcripti	Tcf3
1875	Onecut3	1.000	1.000	1.000	1.000	1.000	1.000	0.000	0.000	0.000	246086	Onecut3	chr10	+	NM_1392	P5601	protein_cc	OC-3| Oc3	one cut do	Onecut3
1876	Atp8b3	1.000	1.000	1.000	1.000	1.000	1.000	0.000	0.000	0.000	67331	Atp8b3	chr10	-	NM_0260	P10520	protein_cc	1700042F	ATPase, cl	Atp8b3
1877	Rexo1	1.766	0.596	1.054	0.003	0.000	0.761	7.092	7.913	7.168	66932	Rexo1	chr10	-	NM_0258	P9877	protein_cc	1700021P	REX1, RNA	Rexo1
1878	Klf16	1.223	1.457	1.782	0.419	0.058	0.078	4.829	5.119	5.662	118445	Klf16	chr10	-	NM_0784	P21488	protein_cc	AI843742|	Kruppel-lik	Klf16
1879	Abhd17a	0.506	1.717	0.869	0.000	0.014	0.410	8.415	7.432	8.212	216169	Abhd17a	chr10	-	NM_1454	P13787	protein_cc	1700013O	abhydrola	Abhd17a
1880	Adat3	1.117	1.370	1.530	0.382	0.109	0.228	3.554	3.714	4.168	10011339	Adat3	chr10	+	NM_0011	P15474	protein_cc	A430024H	adenosine	Adat3
1881	Scamp4	1.246	1.261	1.572	0.006	0.066	0.104	7.928	8.246	8.580	56214	Scamp4	chr10	+	NM_0195	P23815	protein_cc	2410022D	secretory	Scamp4
1882	Csnk1g2	0.786	1.459	1.146	0.004	0.000	0.110	8.825	8.478	9.022	103236	Csnk1g2	chr10	+	NM_1340	P7013	protein_cc	2810429I1	casein kin	Csnk1g2
1883	Btbd2	0.394	0.694	0.274	0.005	0.210	0.036	6.701	5.358	4.831	208198	Btbd2	chr10	-	NM_1453	P4510	protein_cc	2610037C	BTB (POZ)	Btbd2
1884	Mknk2	0.426	0.882	0.375	0.007	0.661	0.095	9.701	8.468	8.287	17347	Mknk2	chr10	-	NM_0214	P20039	protein_cc	2010016G	MAP kinas	Mknk2
1885	Mob3a	5.121	0.527	2.698	0.001	0.005	0.009	3.688	6.044	5.119	208228	Mob3a	chr10	-	NM_1724	P1489	protein_cc	5330417K	MOB kinas	Mob3a
1886	Izumo4	0.215	1.666	0.358	0.004	0.040	0.109	5.061	2.844	3.580	71564	Izumo4	chr10	+	NM_0278	P20395	protein_cc	9030607L	IZUMO fa	Izumo4
1887	Ap3d1	1.412	0.790	1.115	0.006	0.013	0.230	9.078	9.575	9.235	11776	Ap3d1	chr10	-	NM_0074	P1847	protein_cc	AA407035	adaptor-re	Ap3d1
1888	Dot1l	2.145	0.528	1.132	0.002	0.003	0.348	7.083	8.184	7.262	208266	Dot1l	chr10	+	NM_1993	P25495	protein_cc	A630076C	DOT1-like,	Dot1l
1889	Plekhj1	1.117	1.015	1.133	0.526	0.932	0.747	6.026	6.185	6.206	78670	Plekhj1	chr10	-	NM_0239	P1405	protein_cc	9530063M	pleckstrin	Plekhj1
1890	Sf3a2	1.268	0.780	0.989	0.549	0.523	0.961	6.420	6.763	6.404	20222	Sf3a2	chr10	+	NM_0136	P21479	protein_cc	66kDa| A	splicing fa	Sf3a2
1891	Amh	1.000	1.000	1.000	1.000	1.000	1.000	0.000	0.000	0.000	11705	Amh	chr10	+	NM_0074	P26218	protein_cc	MIS	anti-Mulle	Amh
1892	Jsrp1	1.146	0.632	0.725	0.729	1.000	0.422	0.465	0.661	0.000	71912	Jsrp1	chr10	-	NM_0280	P3885	protein_cc	2300003C	junctional	Jsrp1
1893	Oaz1	0.332	1.997	0.664	0.061	0.002	0.515	3.404	1.815	2.813	18245	Oaz1	chr10	+	NM_0087	P8976	protein_cc	Antizyme|	ornithine	Oaz1
1894	Oaz1-ps	0.487	1.095	0.533	0.029	0.672	0.136	11.875	10.836	10.966	10004196	Oaz1-ps	chr10	+	NR_02765	.	misc_RNA	ENSMUSG	ornithine	Oaz1-ps
1895	Lingo3	1.005	1.405	1.412	0.000	0.182	0.423	0.000	0.008	0.498	237403	Lingo3	chr10	-	NM_0010	P23255	protein_cc	AI851712|	leucine ric	Lingo3
1896	Lsm7	1.128	1.052	1.187	0.131	0.640	0.454	7.769	7.942	8.016	66094	Lsm7	chr10	-	NM_0253	P25895	protein_cc	0910001B	LSM7 hom	Lsm7
1897	Sppl2b	0.595	0.722	0.430	0.032	0.185	0.039	6.162	5.413	4.943	73218	Sppl2b	chr10	+	NM_1751	P2109	protein_cc	3110056O	signal pep	Sppl2b
1898	Tmprss9	0.594	3.991	2.371	0.195	0.182	0.536	0.804	0.053	2.049	432478	Tmprss9	chr10	+	NM_0010	P7009	protein_cc	Serase-1B	transmem	Tmprss9

1899	Timm13	0.575	1.339	0.770	0.018	0.145	0.310	9.609	8.809	9.231	30055	Timm13	chr10	-	NM_0138	P12521	protein_cc	D10Ertd37	translocas	Timm13
1900	Lmnb2	1.533	0.766	1.175	0.427	0.570	0.650	5.655	6.271	5.887	16907	Lmnb2	chr10	-	NM_0107	P6084	protein_cc	.	lamin B2	Lmnb2
1901	Gadd45b	8.175	0.213	1.744	0.000	0.001	0.179	5.330	8.361	6.132	17873	Gadd45b	chr10	+	NM_0086	P5233	protein_cc	AI323528	growth ar	Gadd45b
1902	Gng7	0.522	0.874	0.456	0.151	0.735	0.285	1.630	0.692	0.497	14708	Gng7	chr10	-	NM_0010	P933	protein_cc	AI840417	guanine n	Gng7
1903	Diras1	1.005	1.405	1.412	0.000	0.182	0.423	0.000	0.008	0.498	208666	Diras1	chr10	-	NM_1452	P20327	protein_cc	Di-Ras1 | C	DIRAS fam	Diras1
1904	Slc39a3	1.107	0.932	1.032	0.475	0.599	0.790	6.263	6.410	6.308	106947	Slc39a3	chr10	-	NM_1341	P23190	protein_cc	AI845814 |	solute car	Slc39a3
1905	Sgta	0.809	1.242	1.004	0.004	0.003	0.826	9.904	9.597	9.910	52551	Sgta	chr10	-	NM_0244	P649	protein_cc	5330427H	small gluta	Sgta
1906	Thop1	0.738	1.978	1.459	0.263	0.033	0.344	4.397	3.958	4.942	50492	Thop1	chr10	+	NM_0226	P17886	protein_cc	AI131655 |	thimet olig	Thop1
1907	Creb3l3	0.512	2.054	1.051	0.000	0.001	0.624	11.597	10.631	11.669	208677	Creb3l3	chr10	-	NM_1453	P16115	protein_cc	BC010786	cAMP resp	Creb3l3
1908	Map2k2	0.823	1.413	1.163	0.011	0.008	0.225	9.761	9.480	9.979	26396	Map2k2	chr10	+	NM_0231	P18668	protein_cc	AA589381	mitogen-a	Map2k2
1909	Zbtb7a	0.786	1.257	0.989	0.024	0.056	0.921	9.665	9.318	9.648	16969	Zbtb7a	chr10	+	NM_0107	P26422	protein_cc	9030619K	zinc finger	Zbtb7a
1910	Pias4	1.521	0.974	1.482	0.039	0.899	0.360	4.766	5.371	5.333	59004	Pias4	chr10	-	NM_0215	P11656	protein_cc	Pias-gamn	protein in	Pias4
1911	Eef2	0.795	1.125	0.894	0.000	0.001	0.023	13.250	12.918	13.087	13629	Eef2	chr10	+	NM_0079	P2878	protein_cc	Ef-2	eukaryotic	Eef2
1912	Snord37	8.336	0.663	5.525	0.005	0.429	0.443	1.747	4.807	4.213	10021745	Snord37	chr10	+	NR_02854	.	snoRNA	MBII-376	small nucl	Snord37
1913	Dapk3	0.990	1.184	1.172	0.942	0.304	0.626	4.669	4.655	4.898	13144	Dapk3	chr10	+	NM_0011	P9205	protein_cc	dlk | ZIPK	death-ass	Dapk3
1914	Atcayos	1.000	1.000	1.000	1.000	1.000	1.000	0.000	0.000	0.000	69678	Atcayos	chr10	+	NR_01547	.	lncRNA	2310050B	ataxia, cer	Atcayos
1915	Nmrk2	1.580	0.633	1.000	0.305	1.000	0.423	0.000	0.660	0.000	69564	Nmrk2	chr10	-	NM_0271	P5579	protein_cc	3110015C	nicotinam	Nmrk2
1916	Atcay	1.000	1.000	1.000	1.000	1.000	1.000	0.000	0.000	0.000	16467	Atcay	chr10	-	NM_1786	P18166	protein_cc	3322401A	ataxia, cer	Atcay
1917	Zfr2	0.594	0.623	0.371	0.170	1.000	0.134	1.432	0.682	0.000	103406	Zfr2	chr10	+	NM_0010	P10128	protein_cc	2010013I2	zinc finger	Zfr2
1918	Matk	1.641	1.210	1.987	0.450	0.711	0.322	0.421	1.136	1.411	17179	Matk	chr10	+	NM_0012	P5475	protein_cc	CHK | HYL |	megakary	Matk
1919	Mrpl54	1.788	0.994	1.777	0.000	0.796	0.007	8.814	9.653	9.644	66047	Mrpl54	chr10	-	NM_0253	P22742	protein_cc	0610008M	mitochond	Mrpl54
1920	Apba3	1.269	0.809	1.027	0.693	0.718	0.925	8.394	8.737	8.432	57267	Apba3	chr10	+	NM_0187	P14286	protein_cc	AW20879	amyloid be	Apba3
1921	Tjp3	0.886	0.887	0.786	0.855	0.864	0.599	8.212	8.037	7.864	27375	Tjp3	chr10	-	NM_0137	P6161	protein_cc	.	tight junct	Tjp3
1922	Pip5k1c	0.546	1.142	0.624	0.001	0.088	0.063	6.922	6.050	6.241	18717	Pip5k1c	chr10	+	NM_0011	P21207	protein_cc	AI115456 |	phosphati	Pip5k1c
1923	Cactin	1.299	1.109	1.441	0.017	0.242	0.062	6.468	6.845	6.995	70312	Cactin	chr10	+	NM_0273	P19685	protein_cc	2510012J0	cactin, spl	Cactin
1924	Tbxa2r	0.145	1.075	0.156	0.004	0.906	0.068	3.976	1.195	1.299	21390	Tbxa2r	chr10	+	NM_0012	P12232	protein_cc	TP | TXA2-	thrombox	Tbxa2r
1925	Gipc3	1.000	1.000	1.000	1.000	1.000	1.000	0.000	0.000	0.000	209047	Gipc3	chr10	-	NM_1489	P11569	protein_cc	Ahl5 | Rgs	GIPC PDZ	Gipc3
1926	Hmg20b	0.586	1.709	1.001	0.030	0.145	0.999	8.125	7.352	8.125	15353	Hmg20b	chr10	-	NM_0011	P17717	protein_cc	AW61068	high mobi	Hmg20b
1927	Mfsd12	1.572	0.636	1.000	0.304	1.000	0.423	0.000	0.653	0.000	73822	Mfsd12	chr10	+	NM_0286	P10260	protein_cc	C19orf28 |	major faci	Mfsd12
1928	4930404N	0.560	1.371	0.768	0.184	0.191	0.687	0.857	0.021	0.477	432479	4930404N	chr10	-	NM_0010	P18374	protein_cc	2210409H	RIKEN cDN	4930404N
1929	Fzr1	1.691	0.878	1.484	0.005	0.491	0.349	6.979	7.736	7.548	56371	Fzr1	chr10	-	NM_0197	P6333	protein_cc	AW10840	fizzy/cell	Fzr1
1930	Dohh	0.975	1.485	1.448	0.939	0.051	0.387	7.786	7.750	8.320	102115	Dohh	chr10	+	NM_1339	P1336	protein_cc	1110033C	deoxyhyp	Dohh
1931	Smim24	1.581	0.906	1.432	0.301	0.802	0.423	0.000	0.661	0.519	72273	Smim24	chr10	+	NM_0010	P17223	protein_cc	2210404O	small integ	Smim24
1932	Nfic	0.171	2.700	0.461	0.000	0.085	0.126	10.469	7.920	9.353	18029	Nfic	chr10	-	NM_0086	P18123	protein_cc	1110019L	nuclear fa	Nfic
1933	Celf5	1.005	1.405	1.412	0.000	0.182	0.423	0.000	0.008	0.498	319586	Celf5	chr10	-	NM_1769	P7470	protein_cc	4930565N	CUGBP, El	Celf5
1934	Ncln	0.575	1.706	0.981	0.003	0.062	0.951	8.374	7.576	8.346	103425	Ncln	chr10	-	NM_1340	P23142	protein_cc	3100002P	nicalin	Ncln
1935	S1pr4	0.820	1.073	0.879	0.287	0.775	0.719	3.619	3.332	3.434	13611	S1pr4	chr10	-	NM_0101	P14906	protein_cc	Edg6 | Lpc	sphingosin	S1pr4
1936	Gna15	1.332	1.088	1.449	0.446	0.812	0.648	1.080	1.494	1.615	14676	Gna15	chr10	-	NM_0103	P317	protein_cc	Galpha15 |	guanine n	Gna15
1937	Gna11	0.587	1.187	0.697	0.000	0.316	0.191	9.061	8.293	8.540	14672	Gna11	chr10	-	NM_0103	P5715	protein_cc	Dsk7 | E43	guanine n	Gna11
1938	Aes	0.156	1.828	0.285	0.000	0.019	0.007	12.657	9.976	10.846	14797	Aes	chr10	+	NM_0103	P15302	protein_cc	AL024115	amino-ter	Aes
1939	Tle2	0.398	2.553	1.016	0.185	0.000	0.981	1.377	0.048	1.400	21886	Tle2	chr10	+	NM_0012	P12611	protein_cc	Grg2 | mKI	transducin	Tle2
1940	Tle6	0.186	5.020	0.936	0.009	0.008	0.881	3.652	1.229	3.557	114606	Tle6	chr10	-	NM_0532	P25636	protein_cc	1810057E	transducin	Tle6
1941	BC025920	2.109	0.775	1.636	0.185	0.647	0.687	1.293	2.370	2.003	268319	BC025920	chr10	+	NR_03067	.	misc_RNA	.	cDNA seq	BC025920
1942	Sirt6	0.802	1.824	1.463	0.583	0.228	0.578	3.574	3.256	4.123	50721	Sirt6	chr10	-	NM_0011	P9387	protein_cc	2810449N	sirtuin 6	Sirt6
1943	Ankrd24	0.257	1.722	0.442	0.016	0.378	0.006	5.855	3.892	4.677	70615	Ankrd24	chr10	+	NM_0274	P20647	protein_cc	4631433D	ankyrin re	Ankrd24
1944	Gm10778	0.681	1.667	1.135	0.326	0.188	0.139	5.218	4.664	5.401	10023320	Gm10778	chr10	+	NM_0011	P25914	protein_cc	ENSMUSG	predicted	Gm10778
1945	Zfp433	0.616	2.178	1.341	0.152	0.028	0.071	4.710	4.011	5.134	73610	Zfp433	chr10	+	NM_0012	P2197	protein_cc	1700123A	zinc finger	Zfp433
1946	Zfp781	1.349	1.074	1.450	0.464	0.539	0.606	3.341	3.774	3.877	331188	Zfp781	chr10	-	NM_1990	P18833	protein_cc	A130081B	zinc finger	Zfp781
1947	Zfp873	0.191	2.060	0.393	0.014	0.042	0.189	4.211	1.820	2.862	408062	Zfp873	chr10	+	NM_0010	P6819	protein_cc	.	zinc finger	Zfp873
1948	AU041133	2.669	0.769	2.052	0.140	0.534	0.011	3.873	5.289	4.910	216177	AU041133	chr10	+	NM_0011	P13734	protein_cc	.	expressed	AU041133
1949	Zfp938	1.515	0.884	1.338	0.002	0.633	0.603	5.367	5.966	5.787	237411	Zfp938	chr10	-	NM_0011	P25367	protein_cc	B230315N	zinc finger	Zfp938
1950	Gm1553	1.000	1.000	1.000	1.000	1.000	1.000	0.000	0.000	0.000	432480	Gm1553	chr10	-	NM_0012	P26574	protein_cc	Lcrn	predicted	Gm1553
1951	1190007I0	0.399	1.088	0.434	0.003	0.578	0.047	8.243	6.917	7.038	544717	1190007I0	chr10	-	NM_0011	P15894	protein_cc	.	RIKEN cDN	1190007I0
1952	Tdg	2.158	0.853	1.842	0.217	0.706	0.054	1.920	3.030	2.801	21665	Tdg	chr10	+	NM_0115	P6861	protein_cc	E130317C	thymine D	Tdg
1953	Gm9855	1.000	1.000	1.000	1.000	1.000	1.000	0.000	0.000	0.000	624784	Gm9855	chr10	-	NR_03719	.	misc_RNA	ENSMUSG	predicted	Gm9855
1954	Glt8d2	1.393	0.742	1.034	0.584	0.541	0.965	1.525	2.003	1.573	74782	Glt8d2	chr10	-	NM_0291	P13944	protein_cc	1110021D	glycosyltra	Glt8d2
1955	Hcfc2	2.672	0.555	1.482	0.001	0.004	0.124	4.869	6.287	5.436	67933	Hcfc2	chr10	+	NM_0010	P685	protein_cc	1700129L	host cell fa	Hcfc2
1956	Nfyb	1.817	0.470	0.853	0.000	0.000	0.073	9.730	10.591	9.501	18045	Nfyb	chr10	-	NM_0109	P26839	protein_cc	AA985999	nuclear tra	Nfyb
1957	1700028I1	1.000	1.000	1.000	1.000	1.000	1.000	0.000	0.000	0.000	70003	1700028I1	chr10	+	NR_03804	.	lncRNA	.	RIKEN cDN	1700028I1
1958	Txnrd1	2.025	0.572	1.158	0.001	0.006	0.499	9.317	10.336	9.530	50493	Txnrd1	chr10	+	NM_0010	P24630	protein_cc	TR | TR1 | ⁻	thioredoxi	Txnrd1
1959	Eid3	8.534	0.071	0.610	0.035	1.000	0.421	0.714	3.808	0.000	66341	Eid3	chr10	+	NM_0254	P7651	protein_cc	1700027M	EP300 inte	Eid3
1960	Chst11	14.731	0.596	8.776	0.000	0.037	0.111	0.989	4.870	4.123	58250	Chst11	chr10	+	NM_0214	P6292	protein_cc	1110020P	carbohydr	Chst11
1961	Slc41a2	23.195	0.872	20.226	0.000	0.644	0.186	3.851	8.386	8.189	338365	Slc41a2	chr10	-	NM_1773	P24931	protein_cc	A230035L	solute car	Slc41a2
1962	D10Wsu10	7.613	0.777	5.915	0.000	0.486	0.300	7.388	10.317	9.953	28109	D10Wsu10	chr10	+	NM_0265	P26771	protein_cc	AI747614 |	DNA segm	D10Wsu10
1963	Aldh1l2	1.437	0.639	0.918	0.549	0.370	0.928	2.005	2.528	1.882	216188	Aldh1l2	chr10	-	NM_1535	P21893	protein_cc	D330038I	aldehyde	Aldh1l2
1964	A230046K	2.817	0.532	1.498	0.000	0.000	0.098	7.262	8.756	7.844	319277	A230046K	chr10	+	NM_0010	P24832	protein_cc	AA589518	RIKEN cDN	A230046K
1965	Appl2	0.687	1.153	0.792	0.022	0.172	0.252	7.294	6.752	6.958	216190	Appl2	chr10	-	NM_1452	P772	protein_cc	Dip3b	adaptor p	Appl2
1966	1500009L1	0.716	0.995	0.712	0.182	1.000	0.423	0.490	0.008	0.000	69784	1500009L1	chr10	+	NM_0011	P12715	protein_cc	2700050C	RIKEN cDN	1500009L1
1967	Nuak1	0.609	1.352	0.824	0.037	0.225	0.620	5.682	4.967	5.403	77976	Nuak1	chr10	-	NM_0010	P3389	protein_cc	AU014801	NUAK fam	Nuak1
1968	4930463O	1.000	1.000	1.000	1.000	1.000	1.000	0.000	0.000	0.000	75804	4930463O	chr10	+	NR_10805	.	lncRNA	.	RIKEN cDN	4930463O
1969	Ckap4	3.455	0.814	2.811	0.009	0.308	0.033	5.016	6.804	6.507	216197	Ckap4	chr10	-	NM_1754	P16786	protein_cc	5630400A	cytoskelet	Ckap4
1970	Platr7	1.000	1.000	1.000	1.000	1.000	1.000	0.000	0.000	0.000	442847	Platr7	chr10	-	NR_04065	.	lncRNA	C330046G	pluripoter	Platr7
1971	Tcp11l2	0.687	0.917	0.630	0.001	0.638	0.179	7.342	6.800	6.675	216198	Tcp11l2	chr10	+	NM_1460	P24371	protein_cc	E430026E	t-complex	Tcp11l2
1972	Polr3b	1.089	0.766	0.834	0.317	0.041	0.222	6.684	6.807	6.423	70428	Polr3b	chr10	+	NM_0274	P1464	protein_cc	2700078H	polymeras	Polr3b
1973	Rfx4	0.038	1.158	0.044	0.019	0.375	0.145	4.856	0.153	0.365	71137	Rfx4	chr10	+	NM_0010	P12206	protein_cc	4933412G	regulatory	Rfx4
1974	Ric8b	1.913	0.800	1.531	0.011	0.057	0.319	5.792	6.728	6.406	237422	Ric8b	chr10	+	NM_0010	P2077	protein_cc	BC051080	RIC8 guan	Ric8b
1975	Fhl4	1.005	1.405	1.412	0.000	0.182	0.423	0.000	0.008	0.498	14202	Fhl4	chr10	-	NM_0102	P8349	protein_cc	.	four and a	Fhl4
1976	Tmem263	4.066	0.605	2.459	0.000	0.000	0.001	6.771	8.795	8.070	103266	Tmem263	chr10	+	NM_0010	P19899	protein_cc	AI597468	transmem	Tmem263
1977	Mterf2	1.039	1.044	1.085	0.747	0.817	0.806	4.526	4.581	4.643	74238	Mterf2	chr10	-	NM_0288	P23430	protein_cc	1700007D	mitochond	Mterf2
1978	Cry1	0.723	1.953	1.412	0.109	0.240	0.656	5.987	5.519	6.485	12952	Cry1	chr10	-	NM_0077	P18045	protein_cc	AU020726	cryptochr	Cry1
1979	Btbd11	0.362	0.932	0.338	0.177	0.900	0.401	3.502	2.037	1.936	74007	Btbd11	chr10	+	NM_0287	P10062	protein_cc	6330404E	BTB (POZ)	Btbd11
1980	Pwp1	2.310	0.971	2.244	0.001	0.844	0.130	6.970	8.178	8.136	103136	Pwp1	chr10	+	NM_1339	P17391	protein_cc	2310058A	PWP1 hor	Pwp1
1981	Prdm4	2.783	0.675	1.878	0.014	0.148	0.220	5.371	6.848	6.281	72843	Prdm4	chr10	-	NM_1816	P19937	protein_cc	1700031E	PR domain	Prdm4
1982	Ascl4	1.000	1.000	1.000	1.000	1.000	1.000	0.000	0.000	0.000	67341	Ascl4	chr10	+	NM_0011	P12221	protein_cc	1700054F	achaete-sc	Ascl4
1983	Rtcb	1.064	1.171	1.246	0.478	0.089	0.057	9.199	9.288	9.516	28088	Rtcb	chr10	-	NM_1454	P14460	protein_cc	AI255213 |	RNA 2',3'-	Rtcb
1984	Bpifc	1.005	1.439	1.446	0.000	0.182	0.423	0.000	0.008	0.532	270757	Bpifc	chr10	-	NM_1777	P17250	protein_cc	4732454E	BPI fold co	Bpifc
1985	Fbxo7	0.796	0.972	0.774	0.105	0.738	0.304	8.026	7.698	7.657	69754	Fbxo7	chr10	+	NM_1531	P25118	protein_cc	2410015K	F-box prot	Fbxo7
1986	Syn3	0.294	1.862	0.547	0.002	0.000	0.106	5.115	3.348	4.245	27204	Syn3	chr10	-	NM_0137	P4416	protein_cc	.	synapsin I	Syn3
1987	4930486F	1.572	0.636	1.000	0.304	1.000	0.423	0.000	0.653	0.000	78170	4930486F	chr10	+	NR_03802	.	lncRNA	.	RIKEN cDN	4930486F
1988	Timp3	4.894	0.850	4.159	0.001	0.338	0.055	8.668	10.959	10.725	21859	Timp3	chr10	+	NM_0115	P4290	protein_cc	Timp-3	tissue inhi	Timp3
1989	1810014B	0.147	2.847	0.420	0.000	0.037	0.065	6.119	3.358	4.867	66263	1810014B	chr10	+	NR_01557	.	misc_RNA	.	RIKEN cDN	1810014B

1990	Hsp90b1	2.077	0.615	1.278	0.008	0.024	0.120	13.336	14.391	13.690	22027	Hsp90b1	chr10	-	NM_0116	P14380	protein_cc	endoplasn	heat shocl	Hsp90b1
1991	Ttc41	0.638	0.456	0.291	0.026	0.086	0.060	3.193	2.545	1.411	103220	Ttc41	chr10	+	NM_0010	P5297	protein_cc	AI449705	tetratricop	Ttc41
1992	Nt5dc3	0.660	0.900	0.594	0.000	0.400	0.060	6.763	6.164	6.011	103466	Nt5dc3	chr10	+	NM_1753	P8896	protein_cc	AU040402	5'-nucleot	Nt5dc3
1993	Stab2	0.619	0.712	0.441	0.000	0.032	0.022	7.723	7.031	6.541	192188	Stab2	chr10	-	NM_1386	P16681	protein_cc	FEEL-2| FE	stabilin 2	Stab2
1994	1700113H	1.000	1.000	1.000	1.000	1.000	1.000	0.000	0.000	0.000	76640	1700113H	chr10	+	NM_0296	P17274	protein_cc	.	RIKEN cDN	1700113H
1995	Ascl1	1.000	1.000	1.000	1.000	1.000	1.000	0.000	0.000	0.000	17172	Ascl1	chr10	-	NM_0085	P9517	protein_cc	AI225900	achaete-sc	Ascl1
1996	Pah	0.143	0.711	0.102	0.000	0.218	0.017	13.058	10.253	9.762	18478	Pah	chr10	+	NM_0087	P2486	protein_cc	AW10692	phenylalar	Pah
1997	Igf1	0.495	1.250	0.618	0.000	0.027	0.025	13.424	12.408	12.730	16000	Igf1	chr10	+	NM_0105	P583	protein_cc	C730016P	insulin-like	Igf1
1998	Tyms-ps	0.697	1.655	1.154	0.012	0.007	0.489	3.350	2.829	3.556	22172	Tyms-ps	chr10	+	NR_00004	.	misc_RNA	.	thymidyla	Tyms-ps
1999	Pmch	1.000	1.000	1.000	1.000	1.000	1.000	0.000	0.000	0.000	110312	Pmch	chr10	+	NM_0299	P8022	protein_cc	A230109K	pro-melar	Pmch
2000	Parpbp	1.000	1.000	1.000	1.000	1.000	1.000	0.000	0.000	0.000	75317	Parpbp	chr10	-	NM_0292	P19034	protein_cc	4930547N	PARP1 bin	Parpbp
2001	Nup37	0.964	1.194	1.151	0.641	0.255	0.553	5.873	5.821	6.076	69736	Nup37	chr10	+	NM_0271	P8889	protein_cc	2410003L	nucleopor	Nup37
2002	Ccdc53	1.087	1.312	1.427	0.687	0.163	0.103	8.377	8.498	8.890	67282	Ccdc53	chr10	-	NM_0274	P22319	protein_cc	2900091E	coiled-coil	Ccdc53
2003	Dram1	12.202	0.374	4.558	0.000	0.000	0.060	3.205	6.814	5.393	71712	Dram1	chr10	-	NM_0278	P19044	protein_cc	1200002N	DNA-dama	Dram1
2004	Gnptab	0.741	1.426	1.057	0.000	0.017	0.704	6.239	5.808	6.320	432486	Gnptab	chr10	+	NM_0010	P3574	protein_cc	EG432486	N-acetylgl	Gnptab
2005	Sycp3	0.264	2.023	0.534	0.001	0.002	0.070	9.255	7.335	8.351	20962	Sycp3	chr10	+	NM_0115	P11879	protein_cc	Cor1| Scp	synaptone	Sycp3
2006	Chpt1	0.253	2.247	0.570	0.001	0.001	0.095	10.507	8.527	9.695	212862	Chpt1	chr10	-	NR_02747	.	protein_cc	.	choline ph	Chpt1
2007	Mybpc1	1.000	1.000	1.000	1.000	1.000	1.000	0.000	0.000	0.000	109272	Mybpc1	chr10	-	NM_0012	P19003	protein_cc	8030451F	myosin bi	Mybpc1
2008	Spic	27.325	0.371	10.141	0.004	0.019	0.048	2.903	7.675	6.245	20728	Spic	chr10	-	NM_0114	P14364	protein_cc	AU019198	Spi-C trans	Spic
2009	Gm4925	10.128	0.454	4.603	0.001	0.050	0.318	0.995	4.335	3.197	237433	Gm4925	chr10	-	NM_0010	P19503	protein_cc	EG237433	predicted	Gm4925
2010	Arl1	8.034	0.605	4.860	0.001	0.171	0.305	2.575	5.581	4.856	104303	Arl1	chr10	+	NM_0258	P8490	protein_cc	2310008D	ADP-ribos	Arl1
2011	Utp20	2.307	1.026	2.368	0.001	0.877	0.132	5.722	6.928	6.966	70683	Utp20	chr10	-	NM_1751	P3624	protein_cc	3830408P	UTP20 sm	Utp20
2012	Slc5a8	1.000	1.000	1.000	1.000	1.000	1.000	0.000	0.000	0.000	216225	Slc5a8	chr10	+	NM_1454	P20263	protein_cc	Ait| SMCT	solute car	Slc5a8
2013	Ano4	0.716	0.995	0.712	0.182	1.000	0.423	0.490	0.008	0.000	320091	Ano4	chr10	-	NM_0012	P13675	protein_cc	A330096C	anoctamin	Ano4
2014	Gas2l3	0.662	0.990	0.655	0.092	0.970	0.261	4.806	4.210	4.196	237436	Gas2l3	chr10	-	NM_0012	P8051	protein_cc	8430435B	growth ar	Gas2l3
2015	Nr1h4	0.169	4.310	0.728	0.000	0.003	0.216	9.458	6.893	9.000	20186	Nr1h4	chr10	-	NM_0091	P12242	protein_cc	AI957360	nuclear re	Nr1h4
2016	Slc17a8	0.241	0.534	0.129	0.016	0.067	0.108	5.487	3.432	2.528	216227	Slc17a8	chr10	-	NM_1829	P16412	protein_cc	BC042593	solute car	Slc17a8
2017	Scyl2	2.021	0.936	1.893	0.000	0.399	0.063	5.500	6.515	6.421	213326	Scyl2	chr10	-	NM_1980	P20065	protein_cc	AU015688	SCY1-like	Scyl2
2018	1500026H	0.206	2.559	0.528	0.050	0.267	0.517	4.697	2.420	3.775	69002	1500026H	chr10	+	NR_13095	.	lncRNA	.	RIKEN cDN	1500026H
2019	Actr6	0.375	1.162	0.435	0.005	0.662	0.098	6.938	5.521	5.737	67019	Actr6	chr10	-	NM_0259	P21748	protein_cc	2010200J	ARP6 actir	Actr6
2020	Uhrf1bp1l	0.479	1.592	0.763	0.000	0.015	0.198	9.360	8.299	8.970	75089	Uhrf1bp1l	chr10	+	NM_0291	P20796	protein_cc	2010319N	UHRF1 (IC	Uhrf1bp1l
2021	Anks1b	1.058	7.963	8.424	0.000	0.181	0.423	0.001	0.082	3.076	77531	Anks1b	chr10	+	NM_0011	P18232	protein_cc	AIDA-1b|	ankyrin re	Anks1b
2022	Apaf1	16.914	0.386	6.527	0.000	0.005	0.203	4.555	8.635	7.261	11783	Apaf1	chr10	-	NM_0010	P23944	protein_cc	6230400I(	apoptotic	Apaf1
2023	Ikbip	3.149	0.594	1.872	0.013	0.100	0.241	5.715	7.370	6.619	67454	Ikbip	chr10	+	NM_0261	P10930	protein_cc	1200009F	IKBKB inte	Ikbip
2024	Slc25a3	0.845	1.248	1.054	0.116	0.087	0.582	11.896	11.653	11.972	18674	Slc25a3	chr10	-	NM_1336	P5997	protein_cc	5730556H	solute car	Slc25a3
2025	Tmpo	0.716	0.995	0.712	0.005	0.984	0.372	8.301	7.819	7.812	21917	Tmpo	chr10	-	NM_0010	P2871	protein_cc	5630400D	thymopoi	Tmpo
2026	Rmst	0.439	0.985	0.432	0.002	1.000	0.051	1.210	0.021	0.000	110333	Rmst	chr10	-	NR_02826	.	lncRNA	10004179	rhabdomy	Rmst
2027	Gm20757	1.000	1.000	1.000	1.000	1.000	1.000	0.000	0.000	0.000	627800	Gm20757	chr10	-	NR_04602	.	lncRNA	.	predicted	Gm20757
2028	Nedd1	1.597	0.739	1.180	0.004	0.022	0.210	6.001	6.676	6.240	17997	Nedd1	chr10	-	NM_0086	P17565	protein_cc	.	neural pre	Nedd1
2029	Cdk17	2.805	0.714	2.003	0.001	0.049	0.137	6.834	8.322	7.836	237459	Cdk17	chr10	+	NM_1462	P2347	protein_cc	6430598J1	cyclin-dep	Cdk17
2030	Elk3	0.416	1.763	0.734	0.027	0.392	0.676	6.151	4.887	5.705	13713	Elk3	chr10	-	NM_0012	P12710	protein_cc	D430049E	ELK3, mer	Elk3
2031	Gm17745	0.669	0.995	0.666	0.181	1.000	0.423	0.587	0.008	0.000	432488	Gm17745	chr10	+	NR_03801	.	lncRNA	.	predicted	Gm17745
2032	Lta4h	0.710	1.068	0.758	0.302	0.855	0.252	7.519	7.024	7.119	16993	Lta4h	chr10	+	NM_0085	P6655	protein_cc	.	leukotrien	Lta4h
2033	Hal	0.091	1.704	0.156	0.000	0.043	0.001	12.015	8.564	9.333	15109	Hal	chr10	+	NM_0104	P26133	protein_cc	his| histid	histidine a	Hal
2034	Amdhd1	0.108	1.259	0.135	0.000	0.376	0.004	11.616	8.400	8.732	71761	Amdhd1	chr10	-	NM_0279	P7886	protein_cc	1300019J(	amidohyd	Amdhd1
2035	Ccdc38	1.786	1.060	1.893	0.424	0.916	0.251	6.447	7.284	7.368	237465	Ccdc38	chr10	+	NM_1754	P19694	protein_cc	4933417K	coiled-coil	Ccdc38
2036	Snrpf	1.821	1.061	1.932	0.416	0.915	0.237	6.480	7.345	7.430	69878	Snrpf	chr10	-	NM_0272	P26847	protein_cc	2010013O	small nucl	Snrpf
2037	4933408J1	1.030	4.041	4.164	0.000	0.181	0.423	0.001	0.044	2.058	74454	4933408J1	chr10	+	NR_04581	.	lncRNA	.	RIKEN cDN	4933408J1
2038	Ntn4	1.586	0.463	0.734	0.473	1.000	0.422	0.446	1.111	0.000	57764	Ntn4	chr10	+	NM_0213	P8531	protein_cc	.	netrin 4	Ntn4
2039	Gm8580	0.618	1.343	0.830	0.196	0.322	0.741	3.251	2.556	2.981	667338	Gm8580	chr10	-	NR_02747	.	misc_RNA	EG667338	predicted	Gm8580
2040	Gm15915	1.000	1.000	1.000	1.000	1.000	1.000	0.000	0.000	0.000	10050406	Gm15915	chr10	-	NR_03801	.	lncRNA	OTTMUSG	predicted	Gm15915
2041	Usp44	1.000	1.000	1.000	1.000	1.000	1.000	0.000	0.000	0.000	327799	Usp44	chr10	+	NM_0012	P9270	protein_cc	E430004F	ubiquitin s	Usp44
2042	Metap2	1.522	0.849	1.293	0.002	0.036	0.001	10.018	10.624	10.388	56307	Metap2	chr10	-	NM_0196	P22274	protein_cc	4930584B	methionin	Metap2
2043	Vezt	0.811	0.658	0.534	0.050	0.104	0.155	6.820	6.518	5.914	215008	Vezt	chr10	-	NM_0013	P6987	protein_cc	A630071D	vezatin, ac	Vezt
2044	Fgd6	0.682	0.711	0.485	0.001	0.016	0.008	7.282	6.730	6.238	13998	Fgd6	chr10	+	NM_0530	P14226	protein_cc	AA123052	FYVE, Rho	Fgd6
2045	Nr2c1	0.494	1.236	0.611	0.002	0.089	0.048	6.661	5.645	5.951	22025	Nr2c1	chr10	+	NM_0116	P11795	protein_cc	4831444H	nuclear re	Nr2c1
2046	Ndufa12	0.774	1.088	0.842	0.097	0.468	0.277	10.382	10.012	10.134	66414	Ndufa12	chr10	+	NM_0255	P19449	protein_cc	2410011G	NADH deh	Ndufa12
2047	Gm4792	1.000	1.000	1.000	1.000	1.000	1.000	0.000	0.000	0.000	215472	Gm4792	chr10	-	NR_03320	.	lncRNA	E130012K	predicted	Gm4792
2048	Tmcc3	0.667	1.591	1.062	0.034	0.026	0.541	7.752	7.168	7.838	319880	Tmcc3	chr10	+	NM_1720	P2163	protein_cc	AW48809	transmem	Tmcc3
2049	Cep83os	1.013	1.112	1.126	0.974	0.771	0.832	3.987	4.005	4.159	67723	Cep83os	chr10	-	NR_01552	.	lncRNA	4932416G	centrosom	Cep83os
2050	Cep83	1.533	0.790	1.211	0.052	0.172	0.409	6.362	6.978	6.638	77048	Cep83	chr10	+	NM_0298	P2662	protein_cc	2600001G	centrosom	Cep83
2051	Plxnc1	0.368	1.020	0.375	0.003	0.930	0.046	7.067	5.623	5.651	54712	Plxnc1	chr10	-	NM_0187	P15159	protein_cc	2510048K	plexin C1	Plxnc1
2052	Gm29684	2.134	0.469	1.000	0.305	1.000	0.423	0.000	1.094	0.000	628062	Gm29684	chr10	-	NR_13114	.	lncRNA	.	predicted	Gm29684
2053	Cradd	0.802	1.289	1.033	0.143	0.174	0.875	7.657	7.339	7.705	12905	Cradd	chr10	-	NM_0099	P14203	protein_cc	RAIDD	CASP2 and	Cradd
2054	2310039L1	1.005	1.439	1.446	0.000	0.182	0.423	0.000	0.008	0.532	69629	2310039L1	chr10	-	NR_04533	.	lncRNA	.	RIKEN cDN	2310039L1
2055	Socs2	6.043	0.570	3.444	0.000	0.178	0.447	8.522	11.117	10.306	216233	Socs2	chr10	-	NM_0011	P19515	protein_cc	8030460N	suppresso	Socs2
2056	5730420D	5.118	1.499	7.670	0.303	0.573	0.214	0.002	2.357	2.941	70523	5730420D	chr10	+	NR_04533	.	lncRNA	.	RIKEN cDN	5730420D
2057	Mrpl42	0.517	1.145	0.592	0.000	0.506	0.128	9.265	8.314	8.509	67270	Mrpl42	chr10	-	NM_0260	P23117	protein_cc	2700009F	mitochond	Mrpl42
2058	Ube2n	0.992	1.191	1.182	0.978	0.570	0.577	8.502	8.490	8.743	93765	Ube2n	chr10	+	NM_0805	P7282	protein_cc	1500026J1	ubiquitin-c	Ube2n
2059	Nudt4	0.742	1.495	1.109	0.023	0.083	0.715	10.966	10.536	11.116	71207	Nudt4	chr10	-	NM_0277	P22751	protein_cc	4933436C	nudix (nuc	Nudt4
2060	Eea1	0.826	0.779	0.643	0.021	0.116	0.139	9.583	9.307	8.946	216238	Eea1	chr10	+	NM_0010	P19724	protein_cc	A430109N	early endc	Eea1
2061	4930459C	1.858	0.306	0.569	0.210	0.093	0.190	3.791	4.685	2.978	74881	4930459C	chr10	+	NR_11044	.	lncRNA	.	RIKEN cDN	4930459C
2062	Btg1	1.574	0.667	1.049	0.000	0.138	0.925	8.623	9.277	8.692	12226	Btg1	chr10	+	NM_0075	P25603	protein_cc	AI426953	B cell tran	Btg1
2063	4930556N	1.000	1.000	1.000	1.000	1.000	1.000	0.000	0.000	0.000	75301	4930556N	chr10	+	NR_04535	.	lncRNA	.	RIKEN cDN	4930556N
2064	Dcn	1.533	0.879	1.347	0.001	0.127	0.128	11.910	12.526	12.340	13179	Dcn	chr10	+	NM_0011	P9016	protein_cc	DC| DSPG	decorin	Dcn
2065	Lum	0.199	1.485	0.296	0.000	0.002	0.013	6.549	4.222	4.792	17022	Lum	chr10	+	NM_0085	P10455	protein_cc	Ldc| SLRR	lumican	Lum
2066	4930473O	1.000	1.000	1.000	1.000	1.000	1.000	0.000	0.000	0.000	75025	4930473O	chr10	-	NR_04535	.	lncRNA	.	RIKEN cDN	4930473O
2067	Kera	1.000	1.000	1.000	1.000	1.000	1.000	0.000	0.000	0.000	16545	Kera	chr10	+	NM_0084	P25979	protein_cc	CNA2| SLF	keratocan	Kera
2068	Epyc	1.000	1.000	1.000	1.000	1.000	1.000	0.000	0.000	0.000	13516	Epyc	chr10	+	NM_0078	P24009	protein_cc	Dspg3| PC	epiphycan	Epyc
2069	Gm10754	1.000	1.000	1.000	1.000	1.000	1.000	0.000	0.000	0.000	10003869	Gm10754	chr10	-	NR_03353	.	lncRNA	ENSMUSG	predicted	Gm10754
2070	Ccer1	1.000	1.000	1.000	1.000	1.000	1.000	0.000	0.000	0.000	66716	Ccer1	chr10	+	NM_0257	P23014	protein_cc	4921510H	coiled-coil	Ccer1
2071	4930525C	1.000	1.000	1.000	1.000	1.000	1.000	0.000	0.000	0.000	78127	4930525C	chr10	+	NR_13099	.	lncRNA	.	RIKEN cDN	4930525C
2072	Gm8633	1.000	1.000	1.000	1.000	1.000	1.000	0.000	0.000	0.000	667438	Gm8633	chr10	-	NR_04517	.	lncRNA	EG667438	predicted	Gm8633
2073	Atp2b1	0.937	0.899	0.842	0.076	0.069	0.074	10.040	9.945	9.791	67972	Atp2b1	chr10	+	NM_0264	P22315	protein_cc	2810442I2	ATPase, Ca	Atp2b1
2074	Poc1b	0.655	1.201	0.786	0.009	0.217	0.320	7.021	6.410	6.675	382406	Poc1b	chr10	+	NM_0277	P22544	protein_cc	4933430F	POC1 cent	Poc1b
2075	Galnt4	0.849	1.049	0.890	0.310	0.765	0.010	7.531	7.295	7.364	14426	Galnt4	chr10	+	NM_0157	P1354	protein_cc	AV011803	UDP-N-ac	Galnt4
2076	Dusp6	0.231	1.018	0.235	0.000	0.918	0.011	10.877	8.763	8.788	67603	Dusp6	chr10	+	NM_0262	P5223	protein_cc	1300019I(	dual speci	Dusp6
2077	Gm20110	1.615	0.921	1.487	0.536	0.895	0.508	0.785	1.476	1.357	10050420	Gm20110	chr10	-	NR_03801	.	lncRNA	.	predicted	Gm20110
2078	Gm18409	1.000	1.000	1.000	1.000	1.000	1.000	0.000	0.000	0.000	10041712	Gm18409	chr10	-	NR_03801	.	misc_RNA	.	predicted	Gm18409
2079	Csl	1.285	0.636	0.817	0.727	0.534	0.764	1.096	1.458	0.805	71832	Csl	chr10	-	NM_0279	P2148	protein_cc	1700007H	citrate syr	Csl
2080	Kitl	1.781	0.400	0.713	0.182	0.094	0.572	5.155	5.988	4.666	17311	Kitl	chr10	+	NM_0135	P23606	protein_cc	blz| Clo| C	kit ligand	Kitl

2081	Gm4301	1.000	1.000	1.000	1.000	1.000	1.000	0.000	0.000	0.000	10004322	Gm4301	chr10	+	NM_0011	P415	protein_cc	10004322	predicted	Gm4301
2082	Gm4312	1.000	1.000	1.000	1.000	1.000	1.000	0.000	0.000	0.000	10004324	Gm4312	chr10	+	NM_0011	P415	protein_cc	10004324	predicted	Gm4312
2083	Gm4303	1.000	1.000	1.000	1.000	1.000	1.000	0.000	0.000	0.000	10004323	Gm4303	chr10	+	NM_0011	P13438	protein_cc	10004323	predicted	Gm4303
2084	Gm4307	1.000	1.000	1.000	1.000	1.000	1.000	0.000	0.000	0.000	10004323	Gm4307	chr10	+	NM_0011	P13438	protein_cc	10004323	predicted	Gm4307
2085	Gm4305	1.000	1.000	1.000	1.000	1.000	1.000	0.000	0.000	0.000	10004323	Gm4305	chr10	+	NM_0011	P13438	protein_cc	10004323	predicted	Gm4305
2086	Gm4302	1.000	1.000	1.000	1.000	1.000	1.000	0.000	0.000	0.000	10004322	Gm4302	chr10	+	NM_0011	P22926	protein_cc	10004322	predicted	Gm4302
2087	Tmtc3	1.444	0.530	0.765	0.037	0.012	0.357	5.424	5.953	5.037	237500	Tmtc3	chr10	-	NM_0011	P539	protein_cc	9130014E	transmem	Tmtc3
2088	Cep290	3.096	0.493	1.526	0.004	0.014	0.238	5.518	7.149	6.128	216274	Cep290	chr10	+	NM_1460	P7063	protein_cc	b2b1454C	centrosom	Cep290
2089	4930430F	0.691	1.339	0.925	0.195	0.003	0.836	6.490	5.956	6.377	68281	4930430F	chr10	-	NM_1751	P18443	protein_cc	4930571E	RIKEN cDN	4930430F
2090	1700017N	1.000	1.000	1.000	1.000	1.000	1.000	0.000	0.000	0.000	66605	1700017N	chr10	+	NM_0010	P26087	protein_cc	.	RIKEN cDN	1700017N
2091	Mgat4c	1.000	1.000	1.000	1.000	1.000	1.000	0.000	0.000	0.000	67569	Mgat4c	chr10	+	NM_0012	P12852	protein_cc	9130411I1	MGAT4 fa	Mgat4c
2092	Nts	16.126	0.419	6.761	0.022	0.087	0.002	1.947	5.958	4.704	67405	Nts	chr10	-	NM_0244	P20909	protein_cc	5033428E	neurotens	Nts
2093	Rassf9	0.835	0.963	0.804	0.773	0.946	0.867	2.480	2.219	2.165	217504	Rassf9	chr10	+	NM_1462	P15470	protein_cc	AW32210	Ras associ	Rassf9
2094	Alx1	1.000	1.000	1.000	1.000	1.000	1.000	0.000	0.000	0.000	216285	Alx1	chr10	-	NM_1725	P18267	protein_cc	AI314867	ALX home	Alx1
2095	Lrriq1	1.000	1.000	1.000	1.000	1.000	1.000	0.000	0.000	0.000	74978	Lrriq1	chr10	-	NM_0011	P6612	protein_cc	4930503E	leucine-ric	Lrriq1
2096	Slc6a15	1.000	1.000	1.000	1.000	1.000	1.000	0.000	0.000	0.000	103098	Slc6a15	chr10	+	NM_0012	P9901	protein_cc	AA536730	solute car	Slc6a15
2097	Gm4340	1.000	1.000	1.000	1.000	1.000	1.000	0.000	0.000	0.000	10004329	Gm4340	chr10	+	NM_0011	P24571	protein_cc	10004329	predicted	Gm4340
2098	Gm6763	1.000	1.000	1.000	1.000	1.000	1.000	0.000	0.000	0.000	627488	Gm6763	chr10	+	NM_0012	P19612	protein_cc	EG627488	predicted	Gm6763
2099	Gm20765	1.000	1.000	1.000	1.000	1.000	1.000	0.000	0.000	0.000	637578	Gm20765	chr10	+	NM_0012	P19612	protein_cc	.	predicted	Gm20765
2100	Gm21304	1.000	1.000	1.000	1.000	1.000	1.000	0.000	0.000	0.000	10086189	Gm21304	chr10	+	NM_0012	P19612	protein_cc	.	predicted	Gm21304
2101	Gm8764	0.716	0.995	0.712	0.182	1.000	0.423	0.490	0.008	0.000	667692	Gm8764	chr10	+	NM_0012	P19612	protein_cc	EG667692	predicted	Gm8764
2102	Gm21293	1.000	1.000	1.000	1.000	1.000	1.000	0.000	0.000	0.000	10086188	Gm21293	chr10	+	NM_0012	P19612	protein_cc	.	predicted	Gm21293
2103	Gm21312	1.000	1.000	1.000	1.000	1.000	1.000	0.000	0.000	0.000	10086190	Gm21312	chr10	+	NM_0012	P19612	protein_cc	.	predicted	Gm21312
2104	Tmtc2	0.902	0.669	0.604	0.072	0.017	0.104	6.171	6.022	5.442	278279	Tmtc2	chr10	-	NM_1773	P23423	protein_cc	8430438D	transmem	Tmtc2
2105	Gm15663	0.976	0.997	0.973	0.960	0.996	0.965	1.163	1.128	1.124	327812	Gm15663	chr10	+	NR_03803	.	lncRNA	9330159K	predicted	Gm15663
2106	Mettl25	2.069	0.548	1.133	0.040	0.074	0.852	4.463	5.512	4.644	216292	Mettl25	chr10	-	NM_2075	P6481	protein_cc	.	methyltra	Mettl25
2107	Ccdc59	2.144	0.746	1.599	0.000	0.001	0.026	8.186	9.286	8.864	52713	Ccdc59	chr10	+	NM_0256	P16200	protein_cc	2300004H	coiled-coil	Ccdc59
2108	Ppfia2	0.083	3.456	0.288	0.032	0.002	0.266	3.704	0.119	1.908	327814	Ppfia2	chr10	+	NM_1773	P6015	protein_cc	5330438O	protein ty	Ppfia2
2109	4930532I	1.152	0.629	0.725	0.722	1.000	0.422	0.465	0.669	0.000	75833	4930532I	chr10	-	NR_13102	.	lncRNA	.	RIKEN cDN	4930532I0
2110	Acss3	0.473	1.462	0.692	0.409	0.443	0.748	2.597	1.517	2.065	380660	Acss3	chr10	-	NM_0011	P11215	protein_cc	8430416H	acyl-CoA s	Acss3
2111	Lin7a	0.015	7.085	0.109	0.000	0.023	0.007	8.996	2.969	5.793	108030	Lin7a	chr10	+	NM_0010	P22050	protein_cc	AI848705	lin-7 hom	Lin7a
2112	Myf5	1.000	1.000	1.000	1.000	1.000	1.000	0.000	0.000	0.000	17877	Myf5	chr10	-	NM_0086	P14191	protein_cc	B130010J	myogenic	Myf5
2113	Myf6	1.000	1.000	1.000	1.000	1.000	1.000	0.000	0.000	0.000	17878	Myf6	chr10	-	NM_0086	P26498	protein_cc	bHLHc4	myogenic	Myf6
2114	Ptprq	1.000	1.000	1.000	1.000	1.000	1.000	0.000	0.000	0.000	237523	Ptprq	chr10	-	NM_0010	P11722	protein_cc	.	protein ty	Ptprq
2115	Otogl	1.000	1.000	1.000	1.000	1.000	1.000	0.000	0.000	0.000	628870	Otogl	chr10	-	NM_0011	P16311	protein_cc	EG628870	otogelin-li	Otogl
2116	Gm29685	9.124	0.055	0.500	0.000	1.000	0.421	1.001	4.191	0.000	628877	Gm29685	chr10	+	NR_13114	.	lncRNA	.	predicted	Gm29685
2117	Ppp1r12a	1.669	0.592	0.987	0.003	0.000	0.932	8.901	9.640	8.883	17931	Ppp1r12a	chr10	+	NM_0278	P886	protein_cc	1200015F	protein ph	Ppp1r12a
2118	Pawr	4.874	0.556	2.711	0.000	0.003	0.053	4.996	7.281	6.434	114774	Pawr	chr10	+	NM_0540	P8802	protein_cc	2310001G	PRKC, apo	Pawr
2119	Syt1	0.055	3.022	0.165	0.000	0.011	0.012	8.377	4.184	5.779	20979	Syt1	chr10	-	NM_0093	P18537	protein_cc	AW12471	synaptota	Syt1
2120	Gm5136	1.005	1.439	1.446	0.000	0.182	0.423	0.000	0.008	0.532	368203	Gm5136	chr10	-	NM_2036	P15663	protein_cc	C6.1AL	E	predicted	Gm5136
2121	Nav3	8.309	0.715	5.940	0.012	0.461	0.335	0.349	3.403	2.919	260315	Nav3	chr10	-	NM_0010	P2711	protein_cc	4732483H	neuron na	Nav3
2122	9230102K	1.010	1.821	1.840	0.000	0.000	0.002	0.000	0.015	0.880	109335	9230102K	chr10	+	NR_02843	.	lncRNA	.	RIKEN cDN	9230102K
2123	E2f7	0.456	2.906	1.324	0.075	0.040	0.599	2.943	1.809	3.348	52679	E2f7	chr10	+	NM_1786	P25974	protein_cc	A630014C	E2F transc	E2f7
2124	1700020G	1.000	1.000	1.000	1.000	1.000	1.000	0.000	0.000	0.000	75630	1700020G	chr10	-	NR_04597	.	lncRNA	4930480H	RIKEN cDN	1700020G
2125	Csrp2	0.386	1.362	0.526	0.005	0.164	0.115	8.546	7.174	7.620	13008	Csrp2	chr10	+	NM_0077	P7613	protein_cc	AW55186	cysteine a	Csrp2
2126	Zdhhc17	2.482	0.657	1.631	0.000	0.017	0.269	5.074	6.386	5.780	320150	Zdhhc17	chr10	-	NM_1725	P9663	protein_cc	A230053P	zinc finger	Zdhhc17
2127	Osbpl8	0.622	0.800	0.498	0.024	0.302	0.016	7.765	7.080	6.758	237542	Osbpl8	chr10	+	NM_0010	P91	protein_cc	AA536976	oxysterol	Osbpl8
2128	Bbs10	1.420	0.599	0.850	0.049	0.040	0.522	5.004	5.510	4.770	71769	Bbs10	chr10	+	NM_0279	P9894	protein_cc	1300007O	Bardet-Bie	Bbs10
2129	Nap1l1	2.398	0.879	2.109	0.012	0.562	0.179	8.097	9.359	9.173	53605	Nap1l1	chr10	+	NM_0157	P1672	protein_cc	AA407126	nucleoson	Nap1l1
2130	Gm5176	0.236	2.292	0.540	0.020	0.202	0.362	3.288	1.204	2.400	382421	Gm5176	chr10	+	NR_03360	.	misc_RNA	EG382421	predicted	Gm5176
2131	Phlda1	0.481	0.405	0.195	0.000	0.000	0.007	11.331	10.274	8.970	21664	Phlda1	chr10	+	NM_0093	P3185	protein_cc	DT1P1B11	pleckstrin	Phlda1
2132	4933440J	5.807	0.251	1.458	0.011	0.018	0.640	1.013	3.551	1.557	71321	4933440J	chr10	-	NR_04534	.	lncRNA	.	RIKEN cDN	4933440J0
2133	Gm20758	1.000	1.000	1.000	1.000	1.000	1.000	0.000	0.000	0.000	629206	Gm20758	chr10	-	NR_04602	.	lncRNA	.	predicted	Gm20758
2134	Krr1	1.151	0.778	0.896	0.025	0.016	0.478	7.637	7.840	7.478	52705	Krr1	chr10	+	NM_1786	P16964	protein_cc	2610511F	KRR1, sma	Krr1
2135	Glipr1	1.083	0.792	0.858	0.676	0.265	0.758	4.391	4.505	4.169	73690	Glipr1	chr10	-	NM_0286	P23076	protein_cc	2410114O	GLI pathog	Glipr1
2136	Glipr1l1	1.000	1.000	1.000	1.000	1.000	1.000	0.000	0.000	0.000	69286	Glipr1l1	chr10	+	NM_0270	P22375	protein_cc	1700011E	GLI pathog	Glipr1l1
2137	Glipr1l2	1.005	1.405	1.412	0.000	0.182	0.423	0.000	0.008	0.498	67537	Glipr1l2	chr10	+	NM_0262	P7034	protein_cc	4921508O	GLI pathog	Glipr1l2
2138	Caps2	1.000	1.000	1.000	1.000	1.000	1.000	0.000	0.000	0.000	353025	Caps2	chr10	+	NM_1782	P7995	protein_cc	D630005B	calcyphos	Caps2
2139	Kcnc2	1.000	1.000	1.000	1.000	1.000	1.000	0.000	0.000	0.000	268345	Kcnc2	chr10	+	NM_0010	P7846	protein_cc	AW04732	potassium	Kcnc2
2140	1700010J	1.000	1.000	1.000	1.000	1.000	1.000	0.000	0.000	0.000	69355	1700010J	chr10	+	NR_04057	.	lncRNA	.	RIKEN cDN	1700010J1
2141	Atxn7l3b	1.070	0.933	0.998	0.653	0.590	0.992	11.741	11.839	11.739	382423	Atxn7l3b	chr10	-	NM_0010	P7500	protein_cc	4921506J	ataxin 7-li	Atxn7l3b
2142	Trhde	1.030	4.041	4.164	0.000	0.181	0.423	0.001	0.044	2.058	237553	Trhde	chr10	-	NM_1462	P17896	protein_cc	9330155P	TRH-degra	Trhde
2143	Tph2	1.000	1.000	1.000	1.000	1.000	1.000	0.000	0.000	0.000	216343	Tph2	chr10	-	NM_1733	P282	protein_cc	AU043594	tryptopha	Tph2
2144	Tbc1d15	1.588	0.930	1.477	0.009	0.610	0.224	8.826	9.493	9.388	66687	Tbc1d15	chr10	-	NM_0257	P19819	protein_cc	4432405K	TBC1 dom	Tbc1d15
2145	Rab21	1.362	0.500	0.681	0.007	0.002	0.099	8.295	8.741	7.741	216344	Rab21	chr10	-	NM_0244	P8313	protein_cc	9630024B	RAB21, me	Rab21
2146	Tmem19	0.537	1.456	0.782	0.001	0.051	0.285	9.445	8.548	9.091	67226	Tmem19	chr10	-	NM_1336	P16194	protein_cc	2810428F	transmem	Tmem19
2147	Thap2	2.601	0.638	1.659	0.000	0.010	0.166	5.290	6.669	6.020	66816	Thap2	chr10	-	NM_0257	P23748	protein_cc	2900040O	THAP dom	Thap2
2148	Zfc3h1	1.451	0.361	0.524	0.002	0.000	0.023	7.568	8.106	6.635	216345	Zfc3h1	chr10	+	NM_0010	P25044	protein_cc	BC033596	zinc finger	Zfc3h1
2149	Lgr5	0.078	0.439	0.034	0.002	0.169	0.043	6.527	2.846	1.657	14160	Lgr5	chr10	-	NM_0101	P25827	protein_cc	FEX	Gpr4	leucine ric	Lgr5
2150	A930009A	1.000	1.000	1.000	1.000	1.000	1.000	0.000	0.000	0.000	77798	A930009A	chr10	+	NM_0299	P1809	protein_cc	.	RIKEN cDN	A930009A
2151	Tspan8	0.083	5.977	0.496	0.010	0.007	0.283	4.379	0.788	3.367	216350	Tspan8	chr10	+	NM_0011	P22306	protein_cc	C76990	E	tetraspani	Tspan8
2152	Ptprr	0.511	2.187	1.117	0.186	0.184	0.897	1.003	0.033	1.162	19279	Ptprr	chr10	+	NM_0112	P23661	protein_cc	Gmcp1	m	protein ty	Ptprr
2153	4933416C	1.000	1.000	1.000	1.000	1.000	1.000	0.000	0.000	0.000	619332	4933416C	chr10	-	NM_0011	P17094	protein_cc	.	RIKEN cDN	4933416C
2154	Ptprb	0.088	2.428	0.214	0.000	0.034	0.010	9.740	6.238	7.517	19263	Ptprb	chr10	+	NM_0299	P19640	protein_cc	3230402H	protein ty	Ptprb
2155	Kcnmb4	1.572	0.636	1.000	0.304	1.000	0.423	0.000	0.653	0.000	58802	Kcnmb4	chr10	-	NM_0214	P14744	protein_cc	1700058G	potassium	Kcnmb4
2156	Kcnmb4os	1.000	1.000	1.000	1.000	1.000	1.000	0.000	0.000	0.000	67342	Kcnmb4os	chr10	+	NR_02810	.	lncRNA	1700058G	potassium	Kcnmb4os
2157	Kcnmb4os	1.000	1.000	1.000	1.000	1.000	1.000	0.000	0.000	0.000	10050441	Kcnmb4os	chr10	+	NR_13064	.	lncRNA	4933400F	potassium	Kcnmb4os
2158	Cnot2	1.214	0.847	1.028	0.113	0.421	0.933	7.683	7.962	7.722	72068	Cnot2	chr10	-	NM_0280	P8171	protein_cc	2600016N	CCR4-NOT	Cnot2
2159	1700030O	1.000	1.000	1.000	1.000	1.000	1.000	0.000	0.000	0.000	72286	1700030O	chr10	-	NR_04534	.	lncRNA	.	RIKEN cDN	1700030O
2160	4930579P	1.000	1.000	1.000	1.000	1.000	1.000	0.000	0.000	0.000	75878	4930579P	chr10	+	NR_13103	.	lncRNA	.	RIKEN cDN	4930579P
2161	Myrfl	1.000	1.000	1.000	1.000	1.000	1.000	0.000	0.000	0.000	237558	Myrfl	chr10	-	NM_0010	P23764	protein_cc	Gm239	myelin reg	Myrfl
2162	Rab3ip	0.701	0.692	0.486	0.001	0.001	0.002	8.112	7.601	7.070	216363	Rab3ip	chr10	-	NM_0010	P16630	protein_cc	B230311A	RAB3A int	Rab3ip
2163	4933412E	10.779	0.421	4.541	0.014	0.066	0.132	1.687	5.117	3.870	71086	4933412E	chr10	+	NR_03802	.	lncRNA	.	RIKEN cDN	4933412E
2164	D630029K	1.000	1.000	1.000	1.000	1.000	1.000	0.000	0.000	0.000	103175	D630029K	chr10	+	NR_02784	.	lncRNA	AI314111	RIKEN cDN	D630029K
2165	Best3	1.000	1.000	1.000	1.000	1.000	1.000	0.000	0.000	0.000	382427	Best3	chr10	+	NM_0010	P24473	protein_cc	mBest4	\	bestrophi	Best3
2166	Lrrc10	1.000	1.000	1.000	1.000	1.000	1.000	0.000	0.000	0.000	237560	Lrrc10	chr10	+	NM_1462	P16344	protein_cc	D330003I	leucine ric	Lrrc10
2167	Cct2	1.164	0.985	1.146	0.297	0.894	0.262	9.665	9.884	9.862	12461	Cct2	chr10	-	NM_0076	P13299	protein_cc	Cctb	chaperoni	Cct2
2168	Frs2	2.589	0.850	2.200	0.006	0.250	0.019	4.464	5.837	5.602	327826	Frs2	chr10	-	NM_1777	P9717	protein_cc	4732458E	fibroblast	Frs2
2169	Yeats4	1.357	1.084	1.471	0.001	0.612	0.294	9.245	9.685	9.802	64050	Yeats4	chr10	-	NM_0265	P16751	protein_cc	4930573H	YEATS dor	Yeats4
2170	9530003J	1.000	1.000	1.000	1.000	1.000	1.000	0.000	0.000	0.000	77397	9530003J	chr10	-	NM_0299	P16499	protein_cc	Lyzf1	RIKEN cDN	9530003J
2171	Lyz2	0.401	0.998	0.400	0.000	0.996	0.108	6.980	5.661	5.658	17105	Lyz2	chr10	-	NM_0173	P16198	protein_cc	AI326280	lysozyme	Lyz2

2172	Lyz1	0.535	1.149	0.615	0.090	0.776	0.315	4.317	3.416	3.616	17110	Lyz1	chr10	-	NM_0135	P17593	protein_cc	Lyz| Lyzf3	lysozyme	Lyz1
2173	Cpsf6	1.609	0.842	1.355	0.020	0.279	0.413	7.216	7.902	7.654	432508	Cpsf6	chr10	-	NM_0010	P21040	protein_cc	4733401N	cleavage a	Cpsf6
2174	Cpm	0.110	1.126	0.124	0.001	0.862	0.023	5.417	2.236	2.408	70574	Cpm	chr10	+	NM_0274	P7015	protein_cc	1110060IC	carboxype	Cpm
2175	Mdm2	1.975	0.924	1.825	0.009	0.488	0.012	8.457	9.439	9.325	17246	Mdm2	chr10	-	NM_0012	P3869	protein_cc	1700007J1	transform	Mdm2
2176	Slc35e3	1.266	1.023	1.296	0.113	0.924	0.566	6.861	7.202	7.235	215436	Slc35e3	chr10	-	NM_0298	P23011	protein_cc	9330166G	solute car	Slc35e3
2177	Nup107	1.515	1.027	1.557	0.366	0.949	0.432	5.874	6.474	6.513	103468	Nup107	chr10	-	NM_1340	P13684	protein_cc	AW54113	nucleopor	Nup107
2178	Rap1b	2.679	0.819	2.196	0.000	0.116	0.085	9.042	10.464	10.176	215449	Rap1b	chr10	-	NM_0244	P11830	protein_cc	2810443E	RAS relate	Rap1b
2179	4933411E	1.000	1.000	1.000	1.000	1.000	1.000	0.000	0.000	0.000	71067	4933411E	chr10	+	NR_04534	.	lncRNA		RIKEN cDN	4933411E
2180	Mdm1	1.254	1.216	1.524	0.662	0.538	0.666	2.719	3.045	3.326	17245	Mdm1	chr10	+	NM_1489	P16703	protein_cc	Arrd2| Mc	transform	Mdm1
2181	Il22	2.134	0.469	1.000	0.305	1.000	0.423	0.000	1.094	0.000	50929	Il22	chr10	+	NM_0169	P16450	protein_cc	IL-22| IL-2	interleukin	Il22
2182	Iltifb	1.000	1.000	1.000	1.000	1.000	1.000	0.000	0.000	0.000	116849	Iltifb	chr10	-	NM_0540	P13702	protein_cc	IL-22b| IL-	interleukin	Iltifb
2183	Ifng	132.981	0.013	1.771	0.001	0.001	0.424	0.003	7.058	0.828	15978	Ifng	chr10	+	NM_0083	P4694	protein_cc	Ifg| IFN-g	interferon	Ifng
2184	Tmevpg1	0.382	0.982	0.375	0.182	1.000	0.423	1.416	0.027	0.000	103214	Tmevpg1	chr10	-	NR_10412	.	lncRNA	AI447422	Theiler's n	Tmevpg1
2185	Dyrk2	1.110	1.684	1.869	0.535	0.395	0.572	5.284	5.435	6.186	69181	Dyrk2	chr10	-	NM_0010	P25271	protein_cc	1810038L	dual-speci	Dyrk2
2186	Gm38403	1.000	1.000	1.000	1.000	1.000	1.000	0.000	0.000	0.000	216377	Gm38403	chr10	-	NR_11048	.	lncRNA	C230029N	predicted	Gm38403
2187	1700064J	1.000	1.000	1.000	1.000	1.000	1.000	0.000	0.000	0.000	73400	1700064J	chr10	+	NR_04534	.	lncRNA	.	RIKEN cDN	1700064J0
2188	Cand1	1.491	0.943	1.407	0.142	0.802	0.342	8.142	8.719	8.635	71902	Cand1	chr10	-	NM_0279	P19383	protein_cc	2310038O	cullin asso	Cand1
2189	4930477N	1.000	1.000	1.000	1.000	1.000	1.000	0.000	0.000	0.000	74905	4930477N	chr10	+	NR_12608	.	lncRNA	.	RIKEN cDN	4930477N
2190	1700025F	1.000	1.000	1.000	1.000	1.000	1.000	0.000	0.000	0.000	67081	1700025F	chr10	+	NR_04057	.	lncRNA	.	RIKEN cDN	1700025F2
2191	Grip1	1.019	2.648	2.698	0.000	0.182	0.423	0.000	0.027	1.432	74053	Grip1	chr10	+	NM_1334	P18030	protein_cc	4931400F	glutamate	Grip1
2192	Grip1os2	1.000	1.000	1.000	1.000	1.000	1.000	0.000	0.000	0.000	75321	Grip1os2	chr10	-	NR_04535	.	lncRNA	4930564G	glutamate	Grip1os2
2193	Grip1os1	1.000	1.000	1.000	1.000	1.000	1.000	0.000	0.000	0.000	78105	Grip1os1	chr10	-	NR_13176	.	lncRNA	4930404E	glutamate	Grip1os1
2194	Helb	1.565	0.974	1.525	0.036	0.902	0.375	6.307	6.954	6.916	117599	Helb	chr10	-	NM_0804	P12047	protein_cc	AI447783|	helicase (D	Helb
2195	Irak3	90.215	0.676	60.984	0.000	0.168	0.139	2.271	8.766	8.201	73914	Irak3	chr10	-	NM_0286	P24028	protein_cc	4833428C	interleukin	Irak3
2196	Tmbim4	1.228	1.148	1.410	0.072	0.253	0.147	9.809	10.105	10.304	68212	Tmbim4	chr10	+	NM_0266	P23126	protein_cc	0610007H	transmem	Tmbim4
2197	Llph	1.355	1.138	1.542	0.196	0.511	0.139	7.812	8.251	8.437	66225	Llph	chr10	+	NM_0254	P25649	protein_cc	1190005P	LLP homol	Llph
2198	Hmga2	5.285	0.816	4.310	0.073	0.613	0.029	2.273	4.675	4.381	15364	Hmga2	chr10	-	NM_0104	P5338	protein_cc	9430083A	high mobi	Hmga2
2199	9230105E	1.000	1.000	1.000	1.000	1.000	1.000	0.000	0.000	0.000	320626	9230105E	chr10	+	NR_04062	.	lncRNA	.	RIKEN cDN	9230105E
2200	4921513I	1.572	0.636	1.000	0.304	1.000	0.423	0.000	0.653	0.000	70874	4921513I	chr10	+	NR_03800	.	lncRNA	.	RIKEN cDN	4921513I0
2201	Msrb3	2.124	0.834	1.772	0.005	0.203	0.106	7.145	8.232	7.970	320183	Msrb3	chr10	-	NM_1770	P18222	protein_cc	D430026P	methionin	Msrb3
2202	Gm15910	1.963	1.031	2.024	0.463	0.962	0.287	0.768	1.741	1.785	10050461	Gm15910	chr10	+	NR_03802	.	lncRNA	OTTMUSG	predicted	Gm15910
2203	Lemd3	1.364	1.047	1.428	0.001	0.152	0.015	6.877	7.325	7.391	380664	Lemd3	chr10	-	NM_0010	P16914	protein_cc	AI316861|	LEM doma	Lemd3
2204	Wif1	0.134	0.949	0.128	0.149	1.000	0.385	2.971	0.075	0.000	24117	Wif1	chr10	+	NM_0119	P10522	protein_cc	AW10779	Wnt inhib	Wif1
2205	Tbc1d30	0.190	0.533	0.101	0.004	0.026	0.058	6.414	4.020	3.112	74694	Tbc1d30	chr10	-	NM_0290	P8685	protein_cc	4930505D	TBC1 dom	Tbc1d30
2206	Gns	0.625	0.914	0.572	0.006	0.105	0.059	10.180	9.502	9.373	75612	Gns	chr10	+	NM_0293	P21297	protein_cc	2610016K	glucosami	Gns
2207	Rassf3	0.278	1.834	0.510	0.002	0.043	0.050	9.360	7.515	8.390	192678	Rassf3	chr10	-	NM_1389	P1630	protein_cc	AW21202	Ras associ	Rassf3
2208	Tbk1	3.289	0.662	2.177	0.000	0.009	0.094	6.966	8.683	8.089	56480	Tbk1	chr10	-	NM_0197	P5101	protein_cc	1200008B	TANK-bind	Tbk1
2209	Xpot	0.545	1.297	0.706	0.003	0.112	0.107	8.753	7.877	8.252	73192	Xpot	chr10	-	NM_0010	P10114	protein_cc	1110004L	exportin, t	Xpot
2210	D930020B	1.000	1.000	1.000	1.000	1.000	1.000	0.000	0.000	0.000	216393	D930020B	chr10	+	NM_1773	P5451	protein_cc	1500017I1	RIKEN cDN	D930020B
2211	BC048403	0.899	1.262	1.134	0.666	0.473	0.740	4.696	4.543	4.878	270802	BC048403	chr10	+	NM_1730	P4135	protein_cc	E030027L	cDNA seq	BC048403
2212	Srgap1	1.210	0.875	1.059	0.130	0.677	0.928	4.540	4.815	4.623	117600	Srgap1	chr10	-	NM_0012	P20493	protein_cc	4930572H	SLIT-ROBO	Srgap1
2213	Gm9079	3.413	0.525	1.791	0.111	0.267	0.478	0.746	2.517	1.586	668272	Gm9079	chr10	-	NR_00405	.	misc_RNA	668272	predicted	Gm9079
2214	Tmem5	1.767	1.143	2.020	0.166	0.631	0.096	6.965	7.787	7.980	216395	Tmem5	chr10	-	NM_1530	P12156	protein_cc	6330415D	transmem	Tmem5
2215	Gm4489	1.000	1.000	1.000	1.000	1.000	1.000	0.000	0.000	0.000	10004351	Gm4489	chr10	+	NR_02763	.	lncRNA	10004351	predicted	Gm4489
2216	Avpr1a	0.494	1.091	0.539	0.000	0.772	0.184	8.774	7.757	7.883	54140	Avpr1a	chr10	+	NM_0168	P23858	protein_cc	AVPR| Av	arginine v	Avpr1a
2217	A130077B	0.560	1.371	0.768	0.184	0.191	0.687	0.857	0.021	0.477	319272	A130077B	chr10	-	NR_04061	.	lncRNA	.	RIKEN cDN	A130077B
2218	Ppm1h	2.036	0.831	1.693	0.150	0.624	0.314	3.593	4.619	4.353	319468	Ppm1h	chr10	+	NM_0011	P799	protein_cc	A430075L	protein ph	Ppm1h
2219	Mon2	1.291	0.689	0.889	0.004	0.001	0.150	8.338	8.707	8.169	67074	Mon2	chr10	-	NM_0011	P10325	protein_cc	2610528O	MON2 hor	Mon2
2220	Usp15	0.659	0.704	0.464	0.003	0.010	0.010	8.264	7.662	7.155	14479	Usp15	chr10	-	NM_0013	P2013	protein_cc	4921514G	ubiquitin s	Usp15
2221	Fam19a2	0.302	0.875	0.264	0.193	0.851	0.402	3.236	1.506	1.314	268354	Fam19a2	chr10	+	NM_0012	P14121	protein_cc	6330575N	family wit	Fam19a2
2222	4930503E	1.182	1.148	1.357	0.696	0.707	0.008	5.389	5.630	5.829	75030	4930503E	chr10	+	NR_02831	.	misc_RNA	.	RIKEN cDN	4930503E2
2223	Slc16a7	0.140	0.750	0.105	0.000	0.332	0.005	8.428	5.591	5.177	20503	Slc16a7	chr10	-	NM_0113	P905	protein_cc	4921534N	solute car	Slc16a7
2224	Lrig3	0.418	1.986	0.831	0.009	0.002	0.518	6.883	5.626	6.616	320398	Lrig3	chr10	+	NM_1771	P7711	protein_cc	9030421L	leucine-ric	Lrig3
2225	4930484H	1.000	1.000	1.000	1.000	1.000	1.000	0.000	0.000	0.000	75813	4930484H	chr10	-	NR_13101	.	lncRNA	.	RIKEN cDN	4930484H
2226	Xrcc6bp1	0.522	0.517	0.270	0.092	0.028	0.183	6.290	5.352	4.401	68876	Xrcc6bp1	chr10	-	NM_0268	P2856	protein_cc	1110068E	XRCC6 bin	Xrcc6bp1
2227	Ctdsp2	0.661	1.174	0.776	0.013	0.427	0.392	8.300	7.704	7.935	52468	Ctdsp2	chr10	+	NM_0011	P12829	protein_cc	AI586070|	CTD (carbo	Ctdsp2
2228	Avil	0.646	0.614	0.397	0.535	0.451	0.486	1.762	1.132	0.428	11567	Avil	chr10	+	NM_0096	P22894	protein_cc	Advil| DO	advillin	Avil
2229	Tsfm	0.817	1.199	0.980	0.604	0.144	0.972	6.127	5.835	6.097	66399	Tsfm	chr10	-	NM_0255	P3974	protein_cc	2310050B	Ts translat	Tsfm
2230	Mettl21b	1.000	1.000	1.000	1.000	1.000	1.000	0.000	0.000	0.000	10050460	Mettl21b	chr10	-	NM_0012	P23270	protein_cc	.	methyltra	Mettl21b
2231	Mettl1	2.794	1.556	4.347	0.027	0.039	0.005	5.002	6.484	7.122	17299	Mettl1	chr10	+	NM_0107	P12000	protein_cc	2810012D	methyltra	Mettl1
2232	Cyp27b1	1.152	0.629	0.725	0.722	1.000	0.422	0.465	0.669	0.000	13115	Cyp27b1	chr10	+	NM_0100	P23935	protein_cc	Cp2b| Cyp	cytochrom	Cyp27b1
2233	March9	1.869	1.206	2.255	0.082	0.621	0.376	0.588	1.490	1.761	216438	March9	chr10	-	NM_0010	P26280	protein_cc	AI608492|	membrane	March9
2234	Cdk4	1.280	1.466	1.877	0.450	0.220	0.166	6.948	7.304	7.856	12567	Cdk4	chr10	+	NM_0098	P11236	protein_cc	Crk3	cyclin-dep	Cdk4
2235	Tspan31	0.702	1.159	0.814	0.134	0.110	0.566	9.409	8.898	9.111	67125	Tspan31	chr10	-	NM_0259	P11597	protein_cc	2700085A	tetraspani	Tspan31
2236	LOC10050	0.186	3.118	0.580	0.004	0.003	0.221	5.393	2.966	4.606	.	LOC10050	chr10	-	NR_04066	.	.	.	.	LOC10050
2237	Agap2	0.677	2.141	1.449	0.525	0.422	0.764	2.338	1.775	2.874	216439	Agap2	chr10	+	NM_0013	P2570	protein_cc	AGAP-2| (	ArfGAP wi	Agap2
2238	Os9	0.681	0.774	0.527	0.000	0.020	0.023	10.816	10.262	9.893	216440	Os9	chr10	-	NM_1776	P4127	protein_cc	4632413K	amplified	Os9
2239	B4galnt1	0.983	1.035	1.017	0.917	0.882	0.961	9.066	9.042	9.091	14421	B4galnt1	chr10	+	NR_04516	.	protein_cc	4933429D	beta-1,4-N	B4galnt1
2240	Slc26a10	0.070	0.922	0.064	0.007	1.000	0.086	3.956	0.117	0.000	216441	Slc26a10	chr10	-	NM_1776	P17331	protein_cc	C78409| [	solute car	Slc26a10
2241	Arhgef25	1.195	1.174	1.403	0.847	0.820	0.690	2.128	2.385	2.616	52666	Arhgef25	chr10	-	NM_0011	P25920	protein_cc	2410008H	Rho guani	Arhgef25
2242	Dtx3	1.073	1.547	1.659	0.820	0.043	0.259	3.615	3.716	4.345	80904	Dtx3	chr10	-	NM_0307	P25117	protein_cc	Deltex3| r	deltex 3, E	Dtx3
2243	F420014N	1.238	0.728	0.901	0.100	0.012	0.699	7.690	7.998	7.540	10003859	F420014N	chr10	+	NR_04571	.	lncRNA	ENSMUSG	RIKEN cDN	F420014N
2244	Pip4k2c	1.296	0.727	0.942	0.057	0.010	0.812	7.836	8.210	7.750	117150	Pip4k2c	chr10	-	NM_0540	P20968	protein_cc	Pip5k2c	phosphati	Pip4k2c
2245	Kif5a	3.417	1.462	4.997	0.000	0.468	0.340	0.001	1.774	2.322	16572	Kif5a	chr10	-	NM_0010	P16911	protein_cc	D10Bwg0	kinesin fa	Kif5a
2246	Dctn2	0.657	1.591	1.045	0.013	0.002	0.715	9.944	9.338	10.007	69654	Dctn2	chr10	+	NM_0011	P12604	protein_cc	2310042E	dynactin 2	Dctn2
2247	Mbd6	1.200	0.651	0.781	0.002	0.000	0.026	7.463	7.725	7.107	110962	Mbd6	chr10	-	NM_0330	P10868	protein_cc	D10Wsu9	methyl-Cp	Mbd6
2248	Ddit3	2.661	0.902	2.400	0.000	0.046	0.008	8.075	9.487	9.338	13198	Ddit3	chr10	+	NM_0078	P20763	protein_cc	chop| CHC	DNA-dama	Ddit3
2249	Mars	1.511	0.914	1.380	0.000	0.064	0.057	9.136	9.732	9.601	216443	Mars	chr10	-	NM_0010	P19008	protein_cc	Metrs| M	methionin	Mars
2250	Arhgap9	0.579	2.636	1.526	0.171	0.090	0.479	3.944	3.155	4.553	216445	Arhgap9	chr10	+	NM_0012	P5032	protein_cc	AU043488	Rho GTPa	Arhgap9
2251	Gli1	0.322	4.992	1.606	0.004	0.011	0.312	3.470	1.833	4.153	14632	Gli1	chr10	-	NM_0102	P6456	protein_cc	AV235269	GLI-Krupp	Gli1
2252	Inhbe	0.539	8.364	4.510	0.038	0.018	0.169	5.720	4.829	7.893	16326	Inhbe	chr10	-	NM_0083	P4490	protein_cc	.	inhibin be	Inhbe
2253	Inhbc	0.194	1.934	0.375	0.000	0.108	0.049	8.373	6.007	6.959	16325	Inhbc	chr10	-	NM_0105	P859	protein_cc	.	inhibin be	Inhbc
2254	R3hdm2	0.476	0.788	0.375	0.000	0.007	0.006	9.484	8.414	8.070	71750	R3hdm2	chr10	+	NM_0011	P17290	protein_cc	1300003K	R3H doma	R3hdm2
2255	Stac3	0.726	1.425	1.034	0.187	0.186	0.943	0.476	0.015	0.525	237611	Stac3	chr10	+	NM_1777	P11885	protein_cc	9830125E	SH3 and c	Stac3
2256	Ndufa4l2	0.447	0.767	0.343	0.001	0.287	0.013	6.875	5.714	5.331	407790	Ndufa4l2	chr10	+	NM_0010	P2794	protein_cc	BC064011	NADH deh	Ndufa4l2
2257	Shmt2	0.293	0.853	0.250	0.000	0.054	0.018	10.367	8.595	8.365	108037	Shmt2	chr10	-	NM_0282	P18099	protein_cc	2700043D	serine hyd	Shmt2
2258	Nxph4	1.010	1.821	1.840	0.000	0.000	0.002	0.000	0.015	0.880	104080	Nxph4	chr10	-	NM_1832	P16196	protein_cc	1110036N	neurexoph	Nxph4
2259	Lrp1	0.388	0.845	0.328	0.001	0.344	0.010	11.506	10.140	9.897	16971	Lrp1	chr10	-	NM_0085	P11301	protein_cc	A2mr| AI3	low densit	Lrp1
2260	Stat6	0.800	1.251	1.001	0.002	0.053	0.994	8.128	7.806	8.129	20852	Stat6	chr10	+	NM_0092	P20222	protein_cc	.	signal tran	Stat6
2261	Nab2	0.674	1.217	0.821	0.000	0.004	0.069	7.408	6.840	7.123	17937	Nab2	chr10	-	NM_0011	P18993	protein_cc	AI451907	Ngfi-A bin	Nab2
2262	1700012D	0.320	1.037	0.332	0.000	0.717	0.018	6.585	4.940	4.992	72243	1700012D	chr10	+	NR_04517	.	lncRNA	.	RIKEN cDN	1700012D

2263	Tmem194	1.059	1.301	1.378	0.826	0.414	0.456	4.707	4.790	5.170	210035	Tmem194	chr10	+	NM_0011	P2946	protein_cc	Nemp1| T	transmem	Tmem194
2264	Myo1a	0.284	2.488	0.708	0.000	0.031	0.337	1.870	0.057	1.372	432516	Myo1a	chr10	+	NM_0010	P19026	protein_cc	BBM-I| M	myosin IA	Myo1a
2265	Tac2	0.534	0.990	0.528	0.000	1.000	0.015	0.921	0.015	0.000	21334	Tac2	chr10	+	NM_0011	P5392	protein_cc	PPT-B| Ta	tachykinin	Tac2
2266	Zbtb39	0.392	1.468	0.576	0.037	0.244	0.258	5.857	4.508	5.061	320080	Zbtb39	chr10	+	NM_1980	P26213	protein_cc	7030401O	zinc finger	Zbtb39
2267	Gpr182	0.483	1.557	0.753	0.006	0.394	0.660	4.701	3.652	4.291	11536	Gpr182	chr10	-	NM_0074	P14685	protein_cc	Admr| AN	G protein-	Gpr182
2268	Rdh1	0.257	1.108	0.285	0.012	0.817	0.126	2.684	0.725	0.873	107605	Rdh1	chr10	+	NM_0804	P23747	protein_cc	.	retinol del	Rdh1
2269	Rdh9	0.044	0.582	0.026	0.000	0.113	0.008	10.237	5.735	4.955	103142	Rdh9	chr10	+	NM_1531	P22154	protein_cc	AI121090|	retinol del	Rdh9
2270	Rdh16	0.229	0.614	0.141	0.000	0.015	0.004	9.953	7.827	7.123	19683	Rdh16	chr10	+	NM_0090	P21187	protein_cc	CRAD| CR	retinol del	Rdh16
2271	Rdh18-ps	0.069	1.199	0.083	0.007	0.309	0.093	3.980	0.120	0.382	380674	Rdh18-ps	chr10	+	NR_03760	.	misc_RNA	Rdh18	retinol del	Rdh18-ps
2272	Rdh19	0.325	0.978	0.318	0.039	1.000	0.201	1.655	0.033	0.000	216453	Rdh19	chr10	+	NM_1472	P19894	protein_cc	RDH-S| RI	retinol del	Rdh19
2273	BC089597	0.289	0.253	0.073	0.000	0.000	0.003	11.256	9.463	7.482	216454	BC089597	chr10	+	NM_1454	P22259	protein_cc	CRAD-L	cDNA seq	BC089597
2274	Rdh7	0.154	1.177	0.181	0.000	0.015	0.012	14.077	11.379	11.614	54150	Rdh7	chr10	-	NM_0174	P8108	protein_cc	AI194929|	retinol del	Rdh7
2275	Sdr9c7	0.010	6.464	0.062	0.000	0.152	0.005	7.538	0.831	3.523	70061	Sdr9c7	chr10	+	NM_0273	P923	protein_cc	1810054F	4short cha	Sdr9c7
2276	Hsd17b6	0.050	0.506	0.025	0.000	0.052	0.002	12.599	8.273	7.290	27400	Hsd17b6	chr10	-	NM_0137	P5041	protein_cc	17betaHSI	hydroxyst	Hsd17b6
2277	Prim1	0.833	1.529	1.273	0.070	0.015	0.298	4.480	4.215	4.827	19075	Prim1	chr10	+	NM_0089	P353	protein_cc	AI324982	DNA prima	Prim1
2278	Naca	0.985	0.856	0.843	0.893	0.150	0.308	9.853	9.831	9.606	17938	Naca	chr10	+	NM_0012	P8324	protein_cc	AL022831	nascent p	Naca
2279	Ptges3	1.230	0.870	1.070	0.050	0.135	0.287	10.555	10.853	10.652	56351	Ptges3	chr10	+	NM_0197	P25251	protein_cc	5730442A	prostaglar	Ptges3
2280	Atp5b	0.696	0.872	0.607	0.000	0.037	0.014	12.476	11.954	11.757	11947	Atp5b	chr10	+	NM_0167	P6789	protein_cc	.	ATP synth	Atp5b
2281	Baz2a	1.127	0.761	0.857	0.166	0.024	0.205	8.125	8.297	7.903	116848	Baz2a	chr10	+	NM_0540	P18285	protein_cc	AA415431	bromodor	Baz2a
2282	Rbms2	0.941	1.308	1.231	0.835	0.324	0.342	7.055	6.968	7.355	56516	Rbms2	chr10	-	NM_0010	P21599	protein_cc	2610315E	RNA bindi	Rbms2
2283	Gls2	0.171	0.866	0.148	0.000	0.429	0.005	12.441	9.896	9.689	216456	Gls2	chr10	+	NM_0012	P8834	protein_cc	A330047B	glutamina	Gls2
2284	Spryd4	0.322	1.196	0.385	0.002	0.226	0.070	10.661	9.024	9.283	66701	Spryd4	chr10	-	NM_0257	P25534	protein_cc	4633402N	SPRY dom	Spryd4
2285	Mip	2.067	0.960	1.985	0.394	0.948	0.323	0.411	1.458	1.400	17339	Mip	chr10	+	NM_0086	P7438	protein_cc	Aqp0| Cat	major intr	Mip
2286	Timeless	1.886	1.039	1.960	0.027	0.817	0.139	4.674	5.589	5.645	21853	Timeless	chr10	+	NM_0011	P8737	protein_cc	C77407| [	timeless c	Timeless
2287	Apon	0.940	1.331	1.251	0.637	0.046	0.118	12.183	12.093	12.506	28194	Apon	chr10	+	NM_1339	P21255	protein_cc	AI195275|	apolipopro	Apon
2288	Apof	0.586	1.219	0.715	0.007	0.037	0.132	13.915	13.145	13.431	103161	Apof	chr10	+	NM_1339	P18534	protein_cc	AI255964	apolipopro	Apof
2289	Stat2	2.555	0.628	1.605	0.002	0.021	0.108	7.365	8.718	8.047	20847	Stat2	chr10	+	NM_0199	P24720	protein_cc	1600010G	signal trar	Stat2
2290	Il23a	2.742	0.364	1.000	0.020	1.000	0.423	0.001	1.456	0.000	83430	Il23a	chr10	-	NM_0312	P9209	protein_cc	IL-23| p19	interleukir	Il23a
2291	Pan2	1.054	0.686	0.723	0.652	0.006	0.220	6.514	6.589	6.046	103135	Pan2	chr10	+	NM_1339	P5357	protein_cc	1200014O	PAN2 poly	Pan2
2292	Cnpy2	0.542	1.166	0.632	0.003	0.378	0.112	10.263	9.381	9.602	56530	Cnpy2	chr10	+	NM_0199	P1015	protein_cc	5330432A	canopy FG	Cnpy2
2293	Cs	0.432	1.080	0.467	0.000	0.541	0.002	9.884	8.674	8.786	12974	Cs	chr10	+	NM_0264	P18956	protein_cc	2610511A	citrate syr	Cs
2294	Coq10a	0.187	2.281	0.427	0.000	0.000	0.041	7.212	4.794	5.984	210582	Coq10a	chr10	-	NM_0010	P18218	protein_cc	Gm1	coenzyme	Coq10a
2295	Ankrd52	3.455	0.639	2.209	0.001	0.032	0.188	3.580	5.368	4.723	237615	Ankrd52	chr10	+	NM_1727	P9860	protein_cc	6430544C	ankyrin re	Ankrd52
2296	Slc39a5	0.716	1.639	1.173	0.061	0.017	0.285	5.605	5.122	5.834	72002	Slc39a5	chr10	-	NM_0280	P805	protein_cc	1810013D	solute car	Slc39a5
2297	Nabp2	0.663	1.492	0.989	0.039	0.117	0.962	7.546	6.953	7.530	69917	Nabp2	chr10	-	NM_0272	P16437	protein_cc	2610036N	nucleic aci	Nabp2
2298	Rnf41	1.821	1.154	2.102	0.003	0.218	0.041	5.370	6.235	6.441	67588	Rnf41	chr10	+	NM_0262	P7273	protein_cc	2210404G	ring finger	Rnf41
2299	Smarcc2	0.482	0.638	0.308	0.002	0.120	0.013	9.286	8.234	7.585	68094	Smarcc2	chr10	+	NM_0011	P26202	protein_cc	5930405J(	SWI/SNF r	Smarcc2
2300	Myl6	1.279	1.189	1.522	0.000	0.000	0.002	10.888	11.243	11.494	17904	Myl6	chr10	-	NM_0108	P13630	protein_cc	ESMLC| L(	myosin, lig	Myl6
2301	Myl6b	0.381	0.757	0.289	0.110	0.000	0.287	2.573	1.181	0.780	216459	Myl6b	chr10	-	NM_1722	P2075	protein_cc	5730437E	myosin, lig	Myl6b
2302	A430046D	0.304	2.605	0.791	0.147	0.025	0.784	3.767	2.048	3.429	654440	A430046D	chr10	+	NR_13115	.	lncRNA	.	Riken cDN	A430046D
2303	Esyt1	1.425	0.799	1.138	0.091	0.167	0.513	7.316	7.827	7.503	23943	Esyt1	chr10	-	NM_0118	P9563	protein_cc	Fam62a| I	extended	Esyt1
2304	Zc3h10	1.359	0.853	1.160	0.003	0.046	0.135	6.341	6.784	6.554	103284	Zc3h10	chr10	-	NM_1340	P16970	protein_cc	AI790326	zinc finger	Zc3h10
2305	Rpl41	1.432	1.387	1.987	0.002	0.015	0.041	12.076	12.595	13.067	67945	Rpl41	chr10	-	NM_0188	P8909	protein_cc	1810055P	ribosomal	Rpl41
2306	Pa2g4	1.422	1.148	1.632	0.000	0.204	0.104	10.445	10.953	11.152	18813	Pa2g4	chr10	-	NM_0111	P22705	protein_cc	38kDa| A/	proliferati	Pa2g4
2307	Erbb3	0.243	1.312	0.319	0.040	0.214	0.247	2.091	0.049	0.441	13867	Erbb3	chr10	-	NM_0101	P21984	protein_cc	C76256| E	erb-b2 rec	Erbb3
2308	Rps26	1.232	1.457	1.796	0.001	0.012	0.064	10.703	11.004	11.547	27370	Rps26	chr10	-	NM_0137	P8167	protein_cc	.	ribosomal	Rps26
2309	Ikzf4	11.233	0.121	1.363	0.011	0.013	0.556	2.671	6.160	3.117	22781	Ikzf4	chr10	-	NM_0117	P26540	protein_cc	A630026H	IKAROS fa	Ikzf4
2310	Suox	0.222	0.870	0.193	0.000	0.078	0.013	9.769	7.597	7.396	211389	Suox	chr10	-	NM_1737	P25269	protein_cc	SO	sulfite oxi	Suox
2311	Rab5b	0.547	1.255	0.686	0.017	0.294	0.298	8.796	7.924	8.252	19344	Rab5b	chr10	-	NM_1774	P22437	protein_cc	C030027M	RAB5B, m	Rab5b
2312	Cdk2	1.086	1.180	1.281	0.735	0.265	0.484	6.612	6.731	6.969	12566	Cdk2	chr10	-	NM_0167	P5208	protein_cc	A630093N	cyclin-dep	Cdk2
2313	Pmel	0.356	1.327	0.472	0.001	0.336	0.068	6.645	5.154	5.562	20431	Pmel	chr10	+	NM_0218	P7095	protein_cc	D10H12S5	premelano	Pmel
2314	Dgka	0.333	1.210	0.403	0.000	0.508	0.063	6.878	5.292	5.567	13139	Dgka	chr10	-	NM_0168	P17041	protein_cc	80kDa| A\	diacylglyc	Dgka
2315	Wibg	2.619	0.584	1.528	0.090	0.161	0.469	1.284	2.673	1.896	.	Wibg	chr10	+	NM_0012	P11673	.	.	.	Wibg
2316	Mmp19	0.167	2.099	0.350	0.000	0.038	0.021	10.002	7.418	8.487	58223	Mmp19	chr10	+	NM_0011	P26614	protein_cc	.	matrix me	Mmp19
2317	Tmem198	0.163	2.120	0.345	0.000	0.037	0.021	9.933	7.312	8.396	73827	Tmem198	chr10	-	NM_1780	P16287	protein_cc	1110012D	transmem	Tmem198
2318	Dnajc14	1.118	0.917	1.025	0.021	0.158	0.782	9.510	9.671	9.546	74330	Dnajc14	chr10	+	NM_0288	P8375	protein_cc	5730551F	DnaJ heat	Dnajc14
2319	Ormdl2	1.111	0.908	1.009	0.074	0.212	0.927	9.428	9.580	9.441	66844	Ormdl2	chr10	-	NM_0241	P5871	protein_cc	0610012C	ORM1-like	Ormdl2
2320	Sarnp	1.826	0.667	1.218	0.000	0.001	0.002	8.309	9.177	8.594	66118	Sarnp	chr10	+	NM_0253	P26414	protein_cc	1110005A	SAP doma	Sarnp
2321	Gdf11	0.750	2.622	1.967	0.200	0.000	0.071	0.448	0.033	1.424	14561	Gdf11	chr10	-	NM_0102	P12750	protein_cc	BMP-11| I	growth dif	Gdf11
2322	Cd63	9.736	1.646	16.029	0.000	0.005	0.012	7.015	10.299	11.018	12512	Cd63	chr10	+	NM_0010	P2169	protein_cc	C75951| N	CD63 anti	Cd63
2323	Rdh5	0.493	0.773	0.381	0.010	0.169	0.052	7.681	6.660	6.287	19682	Rdh5	chr10	-	NM_1340	P3165	protein_cc	9-cis| AI9	retinol del	Rdh5
2324	Bloc1s1	0.742	1.359	1.008	0.011	0.014	0.881	10.121	9.690	10.132	14533	Bloc1s1	chr10	-	NM_0157	P17060	protein_cc	AI839753|	biogenesis	Bloc1s1
2325	Itga7	0.089	3.418	0.303	0.000	0.094	0.061	10.733	7.239	9.012	16404	Itga7	chr10	+	NM_0083	P6998	protein_cc	alpha7| [a	integrin al	Itga7
2326	Mettl7b	0.093	2.481	0.231	0.000	0.121	0.024	12.090	8.667	9.978	71664	Mettl7b	chr10	-	NM_0278	P18882	protein_cc	0610006F	methyltra	Mettl7b
2327	Olfr9	1.000	1.000	1.000	1.000	1.000	1.000	0.000	0.000	0.000	18373	Olfr9	chr10	+	NM_1468	P11013	protein_cc	MOR269-3	olfactory r	Olfr9
2328	Olfr763	1.000	1.000	1.000	1.000	1.000	1.000	0.000	0.000	0.000	258861	Olfr763	chr10	+	NM_1468	P11910	protein_cc	MOR269-2	olfactory r	Olfr763
2329	Olfr765	1.000	1.000	1.000	1.000	1.000	1.000	0.000	0.000	0.000	544748	Olfr765	chr10	-	NM_0010	P6513	protein_cc	.	olfactory r	Olfr765
2330	Olfr767	1.000	1.000	1.000	1.000	1.000	1.000	0.000	0.000	0.000	258315	Olfr767	chr10	-	NM_1463	P25547	protein_cc	MOR115-1	olfactory r	Olfr767
2331	Olfr768	1.000	1.000	1.000	1.000	1.000	1.000	0.000	0.000	0.000	258863	Olfr768	chr10	-	NM_1468	P26674	protein_cc	MOR114-4	olfactory r	Olfr768
2332	Olfr769	1.000	1.000	1.000	1.000	1.000	1.000	0.000	0.000	0.000	257667	Olfr769	chr10	-	NM_1462	P20539	protein_cc	MOR114-1	olfactory r	Olfr769
2333	Olfr770	1.000	1.000	1.000	1.000	1.000	1.000	0.000	0.000	0.000	258862	Olfr770	chr10	-	NM_1468	P3269	protein_cc	MOR114-5	olfactory r	Olfr770
2334	Olfr771	1.000	1.000	1.000	1.000	1.000	1.000	0.000	0.000	0.000	258540	Olfr771	chr10	-	NM_1465	P5241	protein_cc	MOR114-3	olfactory r	Olfr771
2335	Olfr772	1.000	1.000	1.000	1.000	1.000	1.000	0.000	0.000	0.000	257666	Olfr772	chr10	-	NM_1462	P24458	protein_cc	GA_x5J8B	olfactory r	Olfr772
2336	Olfr773	1.000	1.000	1.000	1.000	1.000	1.000	0.000	0.000	0.000	257664	Olfr773	chr10	-	NM_2070	P16696	protein_cc	GA_x6K02	olfactory r	Olfr773
2337	Olfr774	1.000	1.000	1.000	1.000	1.000	1.000	0.000	0.000	0.000	258232	Olfr774	chr10	+	NM_2076	P6976	protein_cc	MOR111-1	olfactory r	Olfr774
2338	Olfr775	1.000	1.000	1.000	1.000	1.000	1.000	0.000	0.000	0.000	258538	Olfr775	chr10	+	NM_1465	P3833	protein_cc	MOR111-6	olfactory r	Olfr775
2339	Olfr776	0.716	0.995	0.712	0.182	1.000	0.423	0.490	0.008	0.000	404321	Olfr776	chr10	+	NM_2075	P19941	protein_cc	MOR111-1	olfactory r	Olfr776
2340	Olfr777	1.000	1.000	1.000	1.000	1.000	1.000	0.000	0.000	0.000	258537	Olfr777	chr10	-	NM_1465	P8974	protein_cc	MOR114-9	olfactory r	Olfr777
2341	Olfr780	1.000	1.000	1.000	1.000	1.000	1.000	0.000	0.000	0.000	258281	Olfr780	chr10	+	NM_1462	P16554	protein_cc	MOR114-1	olfactory r	Olfr780
2342	Olfr781	1.000	1.000	1.000	1.000	1.000	1.000	0.000	0.000	0.000	258723	Olfr781	chr10	+	NM_1467	P19524	protein_cc	MOR114-6	olfactory r	Olfr781
2343	Olfr782	1.000	1.000	1.000	1.000	1.000	1.000	0.000	0.000	0.000	257985	Olfr782	chr10	+	NM_0010	P10573	protein_cc	MOR110-8	olfactory r	Olfr782
2344	Olfr784	1.000	1.000	1.000	1.000	1.000	1.000	0.000	0.000	0.000	258724	Olfr784	chr10	+	NM_1467	P14746	protein_cc	MOR110-3	olfactory r	Olfr784
2345	Olfr785	1.000	1.000	1.000	1.000	1.000	1.000	0.000	0.000	0.000	546488	Olfr785	chr10	+	NM_0013	P17775	protein_cc	EG546488	olfactory r	Olfr785
2346	Olfr786	1.000	1.000	1.000	1.000	1.000	1.000	0.000	0.000	0.000	258542	Olfr786	chr10	+	NM_1465	P17810	protein_cc	MOR111-5	olfactory r	Olfr786
2347	Olfr787	1.000	1.000	1.000	1.000	1.000	1.000	0.000	0.000	0.000	258069	Olfr787	chr10	+	NM_0010	P3480	protein_cc	MOR111-1	olfactory r	Olfr787
2348	Olfr788	1.000	1.000	1.000	1.000	1.000	1.000	0.000	0.000	0.000	258544	Olfr788	chr10	+	NM_1465	P19962	protein_cc	MOR111-4	olfactory r	Olfr788
2349	Olfr789	1.000	1.000	1.000	1.000	1.000	1.000	0.000	0.000	0.000	258148	Olfr789	chr10	+	NM_0013	P11204	protein_cc	MOR111-1	olfactory r	Olfr789
2350	Olfr790	1.000	1.000	1.000	1.000	1.000	1.000	0.000	0.000	0.000	258935	Olfr790	chr10	+	NM_1469	P3672	protein_cc	MOR112-1	olfactory r	Olfr790
2351	Olfr791	1.000	1.000	1.000	1.000	1.000	1.000	0.000	0.000	0.000	258932	Olfr791	chr10	+	NM_1469	P6991	protein_cc	MOR114-1	olfactory r	Olfr791
2352	Olfr792	1.000	1.000	1.000	1.000	1.000	1.000	0.000	0.000	0.000	258150	Olfr792	chr10	+	NM_0010	P5769	protein_cc	MOR108-2	olfactory r	Olfr792
2353	Olfr794	1.000	1.000	1.000	1.000	1.000	1.000	0.000	0.000	0.000	258375	Olfr794	chr10	+	NM_1463	P23236	protein_cc	MOR114-1	olfactory r	Olfr794

2354	Olfr796	1.000	1.000	1.000	1.000	1.000	1.000	0.000	0.000	0.000	258933	Olfr796	chr10	-	NM_1469	P15469	protein_cc	MOR269-1	olfactory	Olfr796
2355	Olfr798	1.000	1.000	1.000	1.000	1.000	1.000	0.000	0.000	0.000	258549	Olfr798	chr10	-	NM_1465	P9236	protein_cc	MOR108-1	olfactory	Olfr798
2356	Olfr799	1.000	1.000	1.000	1.000	1.000	1.000	0.000	0.000	0.000	258929	Olfr799	chr10	+	NM_1469	P851	protein_cc	MOR114-2	olfactory	Olfr799
2357	Olfr800	1.000	1.000	1.000	1.000	1.000	1.000	0.000	0.000	0.000	258541	Olfr800	chr10	+	NM_1465	P9469	protein_cc	MOR114-7	olfactory	Olfr800
2358	Olfr801	1.000	1.000	1.000	1.000	1.000	1.000	0.000	0.000	0.000	258282	Olfr801	chr10	-	NM_1462	P9044	protein_cc	MOR110-1	olfactory	Olfr801
2359	Olfr802	1.000	1.000	1.000	1.000	1.000	1.000	0.000	0.000	0.000	258934	Olfr802	chr10	-	NM_1469	P12889	protein_cc	MOR111-1	olfactory	Olfr802
2360	Olfr803	1.000	1.000	1.000	1.000	1.000	1.000	0.000	0.000	0.000	258547	Olfr803	chr10	-	NM_1465	P17062	protein_cc	MOR111-3	olfactory	Olfr803
2361	Olfr804	1.000	1.000	1.000	1.000	1.000	1.000	0.000	0.000	0.000	258068	Olfr804	chr10	+	NM_0010	P25985	protein_cc	MOR110-7	olfactory	Olfr804
2362	Olfr805	1.000	1.000	1.000	1.000	1.000	1.000	0.000	0.000	0.000	258548	Olfr805	chr10	-	NM_1465	P19967	protein_cc	MOR110-4	olfactory	Olfr805
2363	Olfr806	1.000	1.000	1.000	1.000	1.000	1.000	0.000	0.000	0.000	258546	Olfr806	chr10	-	NM_1465	P19267	protein_cc	MOR110-1	olfactory	Olfr806
2364	Olfr807	1.000	1.000	1.000	1.000	1.000	1.000	0.000	0.000	0.000	258931	Olfr807	chr10	-	NM_1469	P25968	protein_cc	MOR117-1	olfactory	Olfr807
2365	Olfr808	1.000	1.000	1.000	1.000	1.000	1.000	0.000	0.000	0.000	258930	Olfr808	chr10	+	NM_1469	P7524	protein_cc	MOR112-2	olfactory	Olfr808
2366	Olfr809	1.000	1.000	1.000	1.000	1.000	1.000	0.000	0.000	0.000	258321	Olfr809	chr10	+	NM_1463	P5511	protein_cc	MOR108-4	olfactory	Olfr809
2367	Olfr810	1.000	1.000	1.000	1.000	1.000	1.000	0.000	0.000	0.000	258543	Olfr810	chr10	-	NM_1465	P16757	protein_cc	MOR113-4	olfactory	Olfr810
2368	Olfr811	1.000	1.000	1.000	1.000	1.000	1.000	0.000	0.000	0.000	258545	Olfr811	chr10	-	NM_1465	P279	protein_cc	MOR110-6	olfactory	Olfr811
2369	Olfr812	1.000	1.000	1.000	1.000	1.000	1.000	0.000	0.000	0.000	258791	Olfr812	chr10	-	NM_1467	P3517	protein_cc	MOR110-1	olfactory	Olfr812
2370	Olfr813	1.000	1.000	1.000	1.000	1.000	1.000	0.000	0.000	0.000	258252	Olfr813	chr10	+	NM_2071	P20999	protein_cc	MOR108-3	olfactory	Olfr813
2371	Olfr814	1.000	1.000	1.000	1.000	1.000	1.000	0.000	0.000	0.000	259165	Olfr814	chr10	-	NM_2071	P9348	protein_cc	MOR113-5	olfactory	Olfr814
2372	Olfr815	1.000	1.000	1.000	1.000	1.000	1.000	0.000	0.000	0.000	258665	Olfr815	chr10	-	NM_1466	P20913	protein_cc	MOR113-3	olfactory	Olfr815
2373	Olfr816	1.000	1.000	1.000	1.000	1.000	1.000	0.000	0.000	0.000	258667	Olfr816	chr10	-	NM_1466	P24233	protein_cc	MOR113-1	olfactory	Olfr816
2374	Olfr818	1.000	1.000	1.000	1.000	1.000	1.000	0.000	0.000	0.000	258773	Olfr818	chr10	-	NM_1467	P5350	protein_cc	MOR110-2	olfactory	Olfr818
2375	Olfr819	1.000	1.000	1.000	1.000	1.000	1.000	0.000	0.000	0.000	10004320	Olfr819	chr10	-	NM_0011	P3896	protein_cc	MOR265-2	olfactory	Olfr819
2376	Olfr247	1.000	1.000	1.000	1.000	1.000	1.000	0.000	0.000	0.000	258266	Olfr247	chr10	-	NM_1462	P24061	protein_cc	GA_x5J8B	olfactory	Olfr247
2377	Olfr820	1.000	1.000	1.000	1.000	1.000	1.000	0.000	0.000	0.000	258670	Olfr820	chr10	+	NM_1466	P15247	protein_cc	MOR116-1	olfactory	Olfr820
2378	Olfr821	1.000	1.000	1.000	1.000	1.000	1.000	0.000	0.000	0.000	258772	Olfr821	chr10	+	NM_1467	P8617	protein_cc	MOR109-1	olfactory	Olfr821
2379	Olfr822	1.000	1.000	1.000	1.000	1.000	1.000	0.000	0.000	0.000	258666	Olfr822	chr10	+	NM_1466	P7621	protein_cc	MOR113-2	olfactory	Olfr822
2380	Olfr823	1.000	1.000	1.000	1.000	1.000	1.000	0.000	0.000	0.000	258668	Olfr823	chr10	-	NM_1466	P4268	protein_cc	MOR210-4	olfactory	Olfr823
2381	Olfr824	1.000	1.000	1.000	1.000	1.000	1.000	0.000	0.000	0.000	258669	Olfr824	chr10	-	NM_1466	P7907	protein_cc	MOR210-3	olfactory	Olfr824
2382	Olfr825	1.000	1.000	1.000	1.000	1.000	1.000	0.000	0.000	0.000	258672	Olfr825	chr10	-	NM_1466	P1484	protein_cc	MOR210-1	olfactory	Olfr825
2383	Olfr826	1.000	1.000	1.000	1.000	1.000	1.000	0.000	0.000	0.000	258671	Olfr826	chr10	-	NM_1466	P19522	protein_cc	MOR210-2	olfactory	Olfr826
2384	Olfr827	1.000	1.000	1.000	1.000	1.000	1.000	0.000	0.000	0.000	258297	Olfr827	chr10	-	NM_1463	P18424	protein_cc	MOR113-5	olfactory	Olfr827
2385	Neurod4	1.000	1.000	1.000	1.000	1.000	1.000	0.000	0.000	0.000	11923	Neurod4	chr10	-	NM_0075	P13216	protein_cc	AI846749 |	neurogeni	Neurod4
2386	Tespa1	1.572	0.636	1.000	0.304	1.000	0.423	0.000	0.653	0.000	67596	Tespa1	chr10	+	NM_1832	P16629	protein_cc	5830405N	thymocyte	Tespa1
2387	Vmn2r84	1.000	1.000	1.000	1.000	1.000	1.000	0.000	0.000	0.000	625068	Vmn2r84	chr10	-	NM_0010	P11802	protein_cc	EG625068	vomerona	Vmn2r84
2388	Vmn2r85	1.000	1.000	1.000	1.000	1.000	1.000	0.000	0.000	0.000	623734	Vmn2r85	chr10	-	NM_0011	P5011	protein_cc	EG623734	vomerona	Vmn2r85
2389	Vmn2r86	1.000	1.000	1.000	1.000	1.000	1.000	0.000	0.000	0.000	625109	Vmn2r86	chr10	-	NM_0011	P25967	protein_cc	EG625109	vomerona	Vmn2r86
2390	Vmn2r87	1.000	1.000	1.000	1.000	1.000	1.000	0.000	0.000	0.000	625131	Vmn2r87	chr10	-	NM_0011	P13725	protein_cc	EG625131	vomerona	Vmn2r87
2391	Pisd-ps1	0.940	0.904	0.850	0.434	0.483	0.554	8.205	8.116	7.970	236604	Pisd-ps1	chr11	+	NR_00351	.	misc_RNA	.	phosphati	Pisd-ps1
2392	Sfi1	0.721	1.341	0.966	0.104	0.061	0.911	7.742	7.270	7.693	78887	Sfi1	chr11	-	NM_0302	P14239	protein_cc	.	Sfi1 homo	Sfi1
2393	Eif4enif1	1.524	0.902	1.374	0.016	0.251	0.231	6.911	7.519	7.369	74203	Eif4enif1	chr11	+	NM_0011	P2249	protein_cc	2610509L0	eukaryoti	Eif4enif1
2394	Drg1	1.604	1.124	1.803	0.001	0.044	0.011	8.289	8.971	9.140	13494	Drg1	chr11	-	NM_0078	P24667	protein_cc	AA408859	developm	Drg1
2395	Patz1	0.567	1.484	0.841	0.162	0.125	0.719	5.174	4.354	4.924	56218	Patz1	chr11	+	NR_04510	.	protein_cc	8430401L	POZ (BTB)	Patz1
2396	Gm11944	1.082	1.174	1.271	0.834	0.684	0.583	3.134	3.248	3.480	10030264	Gm11944	chr11	+	NR_04570	.	lncRNA	OTTMUSG	predicted	Gm11944
2397	Pik3ip1	1.475	0.986	1.454	0.460	0.975	0.345	3.340	3.900	3.880	216505	Pik3ip1	chr11	+	NM_1781	P20182	protein_cc	1500004A	phosphoir	Pik3ip1
2398	Limk2	1.588	0.847	1.345	0.104	0.372	0.421	6.079	6.747	6.507	16886	Limk2	chr11	-	NM_0107	P17501	protein_cc	Limk-2| w	LIM motif-	Limk2
2399	Rnf185	2.242	1.018	2.284	0.000	0.880	0.086	7.654	8.819	8.845	193670	Rnf185	chr11	-	NR_11095	.	protein_cc	.	ring finger	Rnf185
2400	8430429K	2.018	1.313	2.651	0.000	0.039	0.038	4.897	5.910	6.303	71523	8430429K	chr11	+	NR_02831	.	lncRNA	.	RIKEN cDN	8430429K
2401	Pla2g3	1.000	1.000	1.000	1.000	1.000	1.000	0.000	0.000	0.000	237625	Pla2g3	chr11	+	NM_1727	P791	protein_cc	.	phospholi	Pla2g3
2402	Inpp5j	1.572	0.636	1.000	0.304	1.000	0.423	0.000	0.653	0.000	170835	Inpp5j	chr11	-	NM_1724	P14718	protein_cc	Pib5pa| Pi	inositol po	Inpp5j
2403	Selm	0.319	2.104	0.671	0.005	0.023	0.194	4.717	3.070	4.142	114679	Selm	chr11	+	NM_0532	P24771	protein_cc	1500040L0	selenopro	Selm
2404	Smtn	0.845	1.682	1.422	0.820	0.281	0.697	4.583	4.340	5.090	29856	Smtn	chr11	-	NM_0012	P8149	protein_cc	smsmo	smoothel	Smtn
2405	Tug1	2.011	0.760	1.528	0.000	0.006	0.095	6.255	7.263	6.868	544752	Tug1	chr11	-	NR_11049	.	lncRNA	AI316828	taurine up	Tug1
2406	Morc2a	3.136	0.435	1.365	0.000	0.003	0.477	4.089	5.738	4.538	74522	Morc2a	chr11	+	NM_0011	P4669	protein_cc	8430403M	microrchid	Morc2a
2407	Osbp2	0.036	3.594	0.128	0.024	0.001	0.188	4.969	0.161	2.006	74309	Osbp2	chr11	-	NM_0013	P15984	protein_cc	1700095P	oxysterol	Osbp2
2408	4921536K	0.552	0.990	0.547	0.182	1.000	0.423	0.871	0.015	0.000	67430	4921536K	chr11	-	NM_0261	P15204	protein_cc	AV043724	RIKEN cDN	4921536K
2409	Dusp18	4.307	0.463	1.994	0.001	0.074	0.595	1.021	3.128	2.017	75219	Dusp18	chr11	+	NM_1737	P11868	protein_cc	4930527G	dual speci	Dusp18
2410	Slc35e4	4.713	0.773	3.644	0.022	0.413	0.156	1.711	3.948	3.577	103710	Slc35e4	chr11	-	NM_1531	P24445	protein_cc	A330108F	solute car	Slc35e4
2411	Tcn2	0.404	1.153	0.466	0.000	0.192	0.015	10.129	8.822	9.027	21452	Tcn2	chr11	-	NM_0011	P26582	protein_cc	AW20875	transcoba	Tcn2
2412	Pes1	1.674	0.975	1.631	0.000	0.443	0.006	7.342	8.085	8.047	64934	Pes1	chr11	+	NM_0228	P26381	protein_cc	.	pescadillo	Pes1
2413	Gal3st1	0.484	1.571	0.760	0.321	0.248	0.776	1.757	0.709	1.361	53897	Gal3st1	chr11	+	NM_0011	P11843	protein_cc	Cst| Gcst	galactose-	Gal3st1
2414	Sec14l4	0.228	1.459	0.332	0.000	0.374	0.064	10.860	8.725	9.269	103655	Sec14l4	chr11	+	NM_1460	P2948	protein_cc	AI256582|	SEC14-like	Sec14l4
2415	Sec14l3	1.005	1.405	1.412	0.000	0.182	0.423	0.000	0.008	0.498	380683	Sec14l3	chr11	+	NM_0010	P21162	protein_cc	11100690	SEC14-like	Sec14l3
2416	Mtfp1	0.105	2.319	0.243	0.000	0.034	0.018	7.615	4.359	5.573	67900	Mtfp1	chr11	-	NM_0264	P5886	protein_cc	2700020C	mitochonc	Mtfp1
2417	Sec14l2	0.062	2.125	0.131	0.000	0.110	0.021	12.418	8.396	9.483	67815	Sec14l2	chr11	-	NM_1445	P11639	protein_cc	1300013M	SEC14-like	Sec14l2
2418	Rnf215	1.657	0.609	1.009	0.017	0.043	0.974	6.570	7.299	6.583	71673	Rnf215	chr11	+	NM_0278	P6923	protein_cc	0610009J	ring finger	Rnf215
2419	Ccdc157	1.236	0.578	0.715	0.238	0.001	0.404	6.541	6.847	6.057	216516	Ccdc157	chr11	-	NM_1776	P11637	protein_cc	4930562D	coiled-coil	Ccdc157
2420	Sf3a1	1.974	0.735	1.450	0.001	0.023	0.181	7.273	8.254	7.809	67465	Sf3a1	chr11	+	NM_0261	P4553	protein_cc	1200014H	splicing fa	Sf3a1
2421	Tbc1d10a	0.529	1.128	0.596	0.040	0.667	0.130	5.998	5.078	5.252	103724	Tbc1d10a	chr11	+	NM_1340	P22364	protein_cc	AI447804|	TBC1 dom	Tbc1d10a
2422	Gatsl3	0.212	10.269	2.180	0.025	0.025	0.352	3.030	0.794	4.155	71962	Gatsl3	chr11	+	NM_0280	P12851	protein_cc	2410008K	GATS prot	Gatsl3
2423	Osm	10.404	0.197	2.049	0.074	0.100	0.076	0.002	3.381	1.037	18413	Osm	chr11	+	NM_0010	P9191	protein_cc	OncoM	oncostatir	Osm
2424	Lif	9.881	0.313	3.090	0.000	0.008	0.423	0.002	3.306	1.630	16878	Lif	chr11	+	NM_0085	P9315	protein_cc	.	leukemia i	Lif
2425	Hormad2	1.014	2.240	2.272	0.000	0.182	0.423	0.000	0.021	1.185	75828	Hormad2	chr11	-	NM_0294	P19525	protein_cc	4930529M	HORMA d	Hormad2
2426	Mtmr3	0.805	1.029	0.828	0.310	0.916	0.511	7.754	7.441	7.483	74302	Mtmr3	chr11	-	NM_0288	P17237	protein_cc	1700092A	myotubula	Mtmr3
2427	Gm11961	1.000	1.000	1.000	1.000	1.000	1.000	0.000	0.000	0.000	327860	Gm11961	chr11	-	NR_02779	.	lncRNA	OTTMUSG	predicted	Gm11961
2428	Ascc2	1.924	0.660	1.269	0.055	0.137	0.167	5.370	6.314	5.714	75452	Ascc2	chr11	+	NM_0292	P16226	protein_cc	1700011I1	activating	Ascc2
2429	Uqcr10	0.755	1.318	0.995	0.000	0.004	0.946	11.259	10.854	11.252	66152	Uqcr10	chr11	-	NM_1979	P44	protein_cc	1110020P	ubiquinol-	Uqcr10
2430	Zmat5	0.669	1.119	0.748	0.197	0.722	0.288	6.051	5.471	5.633	67178	Zmat5	chr11	+	NM_0260	P26306	protein_cc	2610510L0	zinc finger	Zmat5
2431	Cabp7	1.000	1.000	1.000	1.000	1.000	1.000	0.000	0.000	0.000	192650	Cabp7	chr11	-	NM_1389	P24990	protein_cc	.	calcium bi	Cabp7
2432	Nf2	1.304	0.862	1.124	0.004	0.024	0.338	7.335	7.718	7.503	18016	Nf2	chr11	-	NM_0012	P10698	protein_cc	merlin	neurofibro	Nf2
2433	Nipsnap1	0.181	1.327	0.240	0.000	0.121	0.007	11.569	9.104	9.512	18082	Nipsnap1	chr11	+	NM_0086	P6698	protein_cc	.	nipsnap h	Nipsnap1
2434	Thoc5	1.411	0.862	1.215	0.351	0.629	0.476	7.434	7.931	7.716	107829	Thoc5	chr11	+	NM_1724	P8494	protein_cc	1700060C	THO comp	Thoc5
2435	AA413626	1.000	1.000	1.000	1.000	1.000	1.000	0.000	0.000	0.000	10035941	AA413626	chr11	+	NR_10268	.	misc_RNA	.	expressed	AA413626
2436	Nefh	0.403	0.414	0.167	0.102	0.210	0.169	3.340	2.028	0.756	380684	Nefh	chr11	-	NM_0109	P11674	protein_cc	mKIAA084	neurofilan	Nefh
2437	Ap1b1	0.689	1.091	0.752	0.003	0.219	0.017	8.992	8.455	8.581	11764	Ap1b1	chr11	+	NM_0012	P5610	protein_cc	Adtb1| b2	adaptor pr	Ap1b1
2438	Gas2l1	1.279	0.803	1.027	0.393	0.479	0.939	7.178	7.533	7.216	78926	Gas2l1	chr11	-	NM_0011	P16056	protein_cc	4930500E	growth ar	Gas2l1
2439	Rasl10a	0.877	2.576	2.258	0.618	0.108	0.389	3.250	3.060	4.425	75668	Rasl10a	chr11	+	NM_1452	P15186	protein_cc	2210403B	RAS-like, f	Rasl10a
2440	Ewsr1	1.456	0.742	1.081	0.201	0.270	0.439	7.882	8.424	7.994	14030	Ewsr1	chr11	-	NM_0079	P12190	protein_cc	Ews| Ewsl	Ewing sarc	Ewsr1
2441	Rhbdd3	0.379	1.172	0.444	0.007	0.659	0.041	7.389	5.989	6.219	279766	Rhbdd3	chr11	+	NM_0012	P9700	protein_cc	Ptag	rhomboid	Rhbdd3
2442	Emid1	0.245	2.095	0.514	0.042	0.190	0.434	2.086	0.058	1.125	140703	Emid1	chr11	-	NM_0805	P18697	protein_cc	AW12207	EMI doma	Emid1
2443	Kremen1	0.791	1.197	0.946	0.028	0.006	0.637	6.032	5.693	5.952	84035	Kremen1	chr11	-	NM_0323	P11182	protein_cc	AV002070	kringle co	Kremen1
2444	Znrf3	0.310	1.186	0.368	0.001	0.375	0.037	6.477	4.789	5.035	407821	Znrf3	chr11	-	NM_0010	P5865	protein_cc	Gm1167	zinc and ri	Znrf3

2445	Xbp1	2.291	1.237	2.835	0.000	0.346	0.154	10.426	11.622	11.930	22433	Xbp1	chr11	+	NM_0138	P15191	protein_cc	D11Ertd39	X-box bind	Xbp1
2446	Ccdc117	1.062	1.241	1.317	0.689	0.196	0.244	7.887	7.973	8.284	104479	Ccdc117	chr11	-	NM_1340	P17384	protein_cc	1110004K	coiled-coil	Ccdc117
2447	Ankrd36	1.000	1.000	1.000	1.000	1.000	1.000	0.000	0.000	0.000	76389	Ankrd36	chr11	+	NM_0238	P9254	protein_cc	1700008J(	ankyrin re	Ankrd36
2448	Mrps24	0.818	1.222	1.000	0.001	0.151	0.999	9.074	8.785	9.074	64660	Mrps24	chr11	-	NM_0260	P24006	protein_cc	3110030K	mitochond	Mrps24
2449	Urgcp	0.781	1.296	1.012	0.034	0.016	0.874	7.124	6.768	7.142	72046	Urgcp	chr11	-	NM_1786	P4047	protein_cc	2010005J(	upregulate	Urgcp
2450	2210015D	0.952	1.775	1.689	0.378	0.000	0.022	6.295	6.223	7.051	.	2210015D	chr11	+	NR_01557	.	.	.	.	2210015D
2451	Dbnl	2.218	0.849	1.884	0.000	0.255	0.162	7.801	8.950	8.714	13169	Dbnl	chr11	+	NM_0011	P18859	protein_cc	Abp1| mA	drebrin-lik	Dbnl
2452	Pgam2	0.552	0.990	0.547	0.182	1.000	0.423	0.871	0.015	0.000	56012	Pgam2	chr11	-	NM_0188	P11130	protein_cc	.	phosphog	Pgam2
2453	Polm	2.864	0.571	1.635	0.000	0.080	0.536	4.011	5.529	4.721	54125	Polm	chr11	-	NM_0174	P25503	protein_cc	AI462166|	polymeras	Polm
2454	Aebp1	0.650	1.653	1.074	0.063	0.016	0.679	6.057	5.435	6.160	11568	Aebp1	chr11	+	NM_0012	P13175	protein_cc	ACLP	AE binding	Aebp1
2455	Pold2	0.684	1.242	0.850	0.074	0.198	0.392	6.239	5.691	6.004	18972	Pold2	chr11	-	NM_0088	P21749	protein_cc	50kDa| p5	polymeras	Pold2
2456	Myl7	0.451	0.986	0.444	0.182	1.000	0.423	1.170	0.021	0.000	17898	Myl7	chr11	-	NM_0228	P4634	protein_cc	MLC-2alpl	myosin, lig	Myl7
2457	Gck	0.022	1.436	0.032	0.000	0.266	0.003	10.725	5.223	5.745	103988	Gck	chr11	-	NM_0012	P7398	protein_cc	Gk| GLK|	glucokinas	Gck
2458	Ykt6	1.488	1.064	1.584	0.026	0.537	0.059	8.950	9.523	9.614	56418	Ykt6	chr11	+	NM_0196	P11772	protein_cc	0610042I1	YKT6 v-SN	Ykt6
2459	Camk2b	1.430	1.755	2.510	0.016	0.105	0.219	6.858	7.374	8.186	12323	Camk2b	chr11	-	NM_0011	P6559	protein_cc	.	calcium/ca	Camk2b
2460	Nudcd3	0.530	1.424	0.755	0.030	0.245	0.215	8.130	7.214	7.724	209586	Nudcd3	chr11	-	NM_1737	P17879	protein_cc	AI427847|	NudC dom	Nudcd3
2461	Rps15a-ps	1.643	1.255	2.062	0.000	0.000	0.001	8.776	9.493	9.820	10004217	Rps15a-ps	chr11	-	NR_02947	.	misc_RNA	Gm11968	ribosomal	Rps15a-ps
2462	Npc1l1	1.000	1.000	1.000	1.000	1.000	1.000	0.000	0.000	0.000	237636	Npc1l1	chr11	-	NM_2072	P19692	protein_cc	9130221N	NPC1-like	Npc1l1
2463	Ddx56	1.059	1.658	1.756	0.337	0.000	0.008	7.613	7.696	8.425	52513	Ddx56	chr11	-	NM_0265	P26564	protein_cc	2600001H	DEAD (Asp	Ddx56
2464	Tmed4	1.023	1.382	1.414	0.667	0.166	0.388	8.893	8.925	9.392	103694	Tmed4	chr11	-	NM_1340	P24230	protein_cc	1110014L	transmem	Tmed4
2465	Ogdh	0.627	0.895	0.561	0.017	0.583	0.111	10.182	9.509	9.349	18293	Ogdh	chr11	+	NM_0109	P19787	protein_cc	2210403E	oxoglutara	Ogdh
2466	Zmiz2	0.709	1.150	0.815	0.065	0.266	0.328	7.002	6.506	6.708	52915	Zmiz2	chr11	+	NM_0010	P47	protein_cc	Zimp7	zinc finger	Zmiz2
2467	Ppia	1.038	1.134	1.178	0.230	0.123	0.289	10.411	10.465	10.647	268373	Ppia	chr11	+	NM_0089	P25628	protein_cc	2700098C	peptidylpr	Ppia
2468	H2afv	0.230	2.298	0.530	0.000	0.001	0.013	10.296	8.178	9.379	77605	H2afv	chr11	-	NM_0299	P25947	protein_cc	C530002L	H2A histo	H2afv
2469	Purb	1.497	0.778	1.165	0.002	0.067	0.469	9.577	10.159	9.797	19291	Purb	chr11	-	NM_0112	P23839	protein_cc	2310015K	purine rich	Purb
2470	Myo1g	1.357	0.866	1.176	0.416	0.515	0.795	4.023	4.464	4.257	246177	Myo1g	chr11	-	NM_1784	P15183	protein_cc	E430002D	myosin IG	Myo1g
2471	Gm11974	7.339	0.942	6.911	0.001	0.764	0.086	4.221	7.097	7.010	.	Gm11974	chr11	-	NR_04589	.	.	.	.	Gm11974
2472	Ccm2	1.404	1.022	1.435	0.009	0.759	0.098	6.852	7.342	7.373	216527	Ccm2	chr11	+	NM_0011	P6097	protein_cc	BC029157	cerebral ca	Ccm2
2473	Nacad	0.750	0.523	0.392	0.560	0.080	0.454	2.423	2.008	1.073	192950	Nacad	chr11	-	NM_0010	P23810	protein_cc	D230024G	NAC alpha	Nacad
2474	Tbrg4	0.909	1.246	1.132	0.454	0.070	0.501	7.890	7.752	8.069	21379	Tbrg4	chr11	-	NM_0011	P21904	protein_cc	2310042P	transform	Tbrg4
2475	Snora5c	0.883	0.536	0.474	0.849	0.283	0.572	2.175	1.996	1.096	100217460	Snora5c	chr11	-	NR_03404	.	snoRNA	MBI-80	small nucl	Snora5c
2476	Wap	1.000	1.000	1.000	1.000	1.000	1.000	0.000	0.000	0.000	22373	Wap	chr11	-	NM_0117	P12908	protein_cc	.	whey acid	Wap
2477	Gm11978	1.058	0.869	0.920	0.888	0.723	0.866	0.587	0.669	0.467	10004131	Gm11978	chr11	-	NR_02858	.	misc_RNA	10004131	predicted	Gm11978
2478	Ramp3	2.507	5.096	12.775	0.026	0.000	0.011	1.631	2.957	5.307	56089	Ramp3	chr11	+	NM_0195	P21861	protein_cc	AI850306	receptor (	Ramp3
2479	Gm11981	0.552	0.990	0.547	0.182	1.000	0.423	0.871	0.015	0.000	626419	Gm11981	chr11	-	NR_04602	.	lncRNA	OTTMUSG	predicted	Gm11981
2480	Adcy1	110.652	0.469	51.863	0.002	0.074	0.245	0.589	7.379	6.286	432530	Adcy1	chr11	+	NM_0096	P5750	protein_cc	AC1| brl|	adenylate	Adcy1
2481	Gm11985	13.440	0.074	0.999	0.009	1.000	0.423	0.002	3.750	0.000	10004134	Gm11985	chr11	-	NR_04510	.	lncRNA	OTTMUSG	predicted	Gm11985
2482	Igfbp1	1.641	0.387	0.636	0.109	0.061	0.504	13.157	13.871	12.503	16006	Igfbp1	chr11	+	NM_0083	P6877	protein_cc	.	insulin-like	Igfbp1
2483	Igfbp3	0.674	1.397	0.941	0.057	0.293	0.870	7.164	6.595	7.077	16009	Igfbp3	chr11	-	NM_0083	P19133	protein_cc	AI649005|	insulin-like	Igfbp3
2484	Tns3	1.431	0.648	0.927	0.178	0.145	0.782	6.960	7.477	6.851	319939	Tns3	chr11	-	NM_0010	P6000	protein_cc	BC023928	tensin 3	Tns3
2485	Hus1	1.387	0.632	0.876	0.213	0.140	0.730	5.820	6.292	5.630	15574	Hus1	chr11	-	NR_13017	.	protein_cc	.	HUS1 chec	Hus1
2486	Sun3	1.580	0.633	1.000	0.305	1.000	0.423	0.000	0.660	0.000	194974	Sun3	chr11	-	NM_0012	P21165	protein_cc	Sunc1	Sad1 and	Sun3
2487	Gm11992	0.203	0.480	0.097	0.010	0.390	0.055	3.821	1.517	0.458	626870	Gm11992	chr11	+	NM_0010	P2657	protein_cc	OTTMUSG	predicted	Gm11992
2488	Upp1	60.833	0.460	27.993	0.003	0.038	0.082	3.308	9.235	8.115	22271	Upp1	chr11	+	NM_0011	P21854	protein_cc	AI325217|	uridine ph	Upp1
2489	Abca13	5.125	0.411	2.108	0.051	0.117	0.079	0.001	2.359	1.077	268379	Abca13	chr11	+	NM_1782	P11832	protein_cc	9830132L	ATP-bindi	Abca13
2490	Vwc2	0.669	0.995	0.666	0.181	1.000	0.423	0.587	0.008	0.000	319922	Vwc2	chr11	+	NM_1770	P1037	protein_cc	A930041G	von Willeb	Vwc2
2491	Zpbp	0.716	0.995	0.712	0.182	1.000	0.423	0.490	0.008	0.000	53604	Zpbp	chr11	-	NM_0157	P11689	protein_cc	4930486K	zona pellu	Zpbp
2492	4930415F	0.355	0.981	0.348	0.056	1.000	0.240	1.524	0.028	0.000	73862	4930415F	chr11	+	NM_0286	P21205	protein_cc	.	RIKEN cDN	4930415F1
2493	4930512M	1.000	1.000	1.000	1.000	1.000	1.000	0.000	0.000	0.000	10263173	4930512M	chr11	-	NM_0013	P14644	protein_cc	.	RIKEN cDN	4930512M
2494	Ikzf1	3.555	0.834	2.963	0.038	0.475	0.128	4.046	5.876	5.613	22778	Ikzf1	chr11	+	NM_0095	P21535	protein_cc	5832432G	IKAROS fa	Ikzf1
2495	Fignl1	1.891	1.147	2.170	0.183	0.791	0.533	1.291	2.210	2.409	60530	Fignl1	chr11	-	NM_0218	P26365	protein_cc	.	fidgetin-lik	Fignl1
2496	Ddc	0.078	1.036	0.080	0.000	0.932	0.006	9.899	6.212	6.263	13195	Ddc	chr11	-	NM_0011	P22041	protein_cc	Aadc	dopa deca	Ddc
2497	1700042O	1.000	1.000	1.000	1.000	1.000	1.000	0.000	0.000	0.000	73321	1700042O	chr11	+	NR_04517	.	lncRNA	.	RIKEN cDN	1700042O
2498	Grb10	1.451	0.620	0.900	0.004	0.007	0.501	5.613	6.150	5.461	14783	Grb10	chr11	-	NM_0103	P9424	protein_cc	5730571D	growth fac	Grb10
2499	Cobl	0.806	2.595	2.091	0.074	0.001	0.029	6.850	6.539	7.914	12808	Cobl	chr11	-	NM_1724	P12945	protein_cc	.	cordon-ble	Cobl
2500	4930554G	1.000	1.000	1.000	1.000	1.000	1.000	0.000	0.000	0.000	75280	4930554G	chr11	-	NR_13101	.	lncRNA	.	RIKEN cDN	4930554G
2501	1700046C	1.000	1.000	1.000	1.000	1.000	1.000	0.000	0.000	0.000	73307	1700046C	chr11	-	NR_04591	.	lncRNA	.	RIKEN cDN	1700046C(
2502	Pom121l1	1.000	1.000	1.000	1.000	1.000	1.000	0.000	0.000	0.000	432536	Pom121l1	chr11	+	NM_0011	P11585	protein_cc	1700054H	POM121 r	Pom121l1
2503	Vstm2a	1.000	1.000	1.000	1.000	1.000	1.000	0.000	0.000	0.000	211739	Vstm2a	chr11	+	NM_0012	P20779	protein_cc	Vstm2	V-set and	Vstm2a
2504	Sec61g	4.231	1.260	5.332	0.000	0.068	0.016	10.529	12.611	12.944	20335	Sec61g	chr11	-	NM_0011	P16389	protein_cc	.	SEC61, ga	Sec61g
2505	Egfr	5.172	0.941	4.865	0.001	0.854	0.257	8.171	10.541	10.453	13649	Egfr	chr11	+	NM_2076	P25125	protein_cc	9030024J1	epidermal	Egfr
2506	Eldr	1.572	0.636	1.000	0.304	1.000	0.423	0.000	0.653	0.000	72735	Eldr	chr11	-	NR_11042	.	lncRNA	2810442I2	Egfr long r	Eldr
2507	Fbxo48	1.010	1.885	1.904	0.000	0.182	0.423	0.000	0.015	0.929	319701	Fbxo48	chr11	+	NM_1769	P17702	protein_cc	A630050E	F-box prot	Fbxo48
2508	Plek	19.703	0.315	6.212	0.000	0.000	0.030	3.972	8.272	6.607	56193	Plek	chr11	-	NM_0195	P19966	protein_cc	2010300B	pleckstrin	Plek
2509	Cnrip1	0.488	1.534	0.749	0.018	0.214	0.368	4.813	3.779	4.396	380686	Cnrip1	chr11	+	NM_0298	P13065	protein_cc	1500041B	cannabino	Cnrip1
2510	Ppp3r1	0.911	1.191	1.086	0.355	0.062	0.541	9.976	9.842	10.095	19058	Ppp3r1	chr11	+	NM_0244	P13584	protein_cc	CaNB1| Cı	protein ph	Ppp3r1
2511	Pno1	1.456	1.174	1.709	0.005	0.028	0.007	8.402	8.944	9.176	66249	Pno1	chr11	-	NM_0254	P7804	protein_cc	1810003N	partner of	Pno1
2512	Wdr92	1.792	1.215	2.178	0.039	0.368	0.129	6.491	7.333	7.614	103784	Wdr92	chr11	+	NM_1789	P7106	protein_cc	AI553587|	WD repea	Wdr92
2513	C1d	0.864	1.026	0.886	0.092	0.617	0.371	9.138	8.927	8.963	57316	C1d	chr11	+	NM_0205	P1140	protein_cc	1110036E	C1D nucle	C1d
2514	Etaa1	0.863	0.559	0.482	0.600	0.057	0.346	6.106	5.894	5.054	68145	Etaa1	chr11	-	NM_0265	P2117	protein_cc	5730466H	Ewing tum	Etaa1
2515	Etaa1os	0.156	7.303	1.142	0.026	0.000	0.758	3.913	1.237	4.105	10263367	Etaa1os	chr11	+	NR_13191	.	lncRNA	2900053O	Ewing tum	Etaa1os
2516	Gm12018	1.000	1.000	1.000	1.000	1.000	1.000	0.000	0.000	0.000	327872	Gm12018	chr11	+	NR_13112	.	lncRNA	OTTMUSG	predicted	Gm12018
2517	Meis1	1.268	0.309	0.391	0.345	0.004	0.300	4.799	5.142	3.446	17268	Meis1	chr11	-	NM_0011	P4473	protein_cc	C530044H	Meis hom	Meis1
2518	4933406G	1.000	1.000	1.000	1.000	1.000	1.000	0.000	0.000	0.000	74059	4933406G	chr11	+	NR_04603	.	lncRNA	.	RIKEN cDN	4933406G
2519	Spred2	0.454	1.029	0.467	0.292	0.963	0.496	2.632	1.493	1.534	114716	Spred2	chr11	+	NM_0335	P24909	protein_cc	C79158	sprouty-re	Spred2
2520	Actr2	0.920	1.177	1.083	0.259	0.141	0.550	9.262	9.141	9.376	66713	Actr2	chr11	-	NM_1462	P17749	protein_cc	4921510D	ARP2 actir	Actr2
2521	Rab1	1.762	0.850	1.498	0.000	0.265	0.265	10.186	11.003	10.769	.	Rab1	chr11	+	NM_0089	P18676	.	.	.	Rab1
2522	Cep68	0.715	0.696	0.498	0.056	0.075	0.042	6.000	5.516	4.994	216543	Cep68	chr11	-	NM_1722	P19348	protein_cc	6030463E	centrosom	Cep68
2523	Slc1a4	0.496	0.547	0.271	0.001	0.000	0.012	6.084	5.072	4.202	55963	Slc1a4	chr11	-	NM_0188	P3454	protein_cc	ASCT-1| A	solute car	Slc1a4
2524	Sertad2	0.609	0.991	0.604	0.080	0.972	0.118	8.951	8.235	8.222	58172	Sertad2	chr11	+	NM_0213	P22427	protein_cc	AB041541	SERTA dor	Sertad2
2525	Aftph	1.912	0.671	1.283	0.012	0.053	0.270	9.045	9.980	9.404	216549	Aftph	chr11	-	NM_0012	P6793	protein_cc	.	aftiphilin	Aftph
2526	Lgalsl	0.308	1.822	0.561	0.006	0.001	0.170	6.426	4.726	5.592	216551	Lgalsl	chr11	-	NM_1737	P12676	protein_cc	1110067D	lectin, gal	Lgalsl
2527	Peli1	1.016	0.870	0.885	0.868	0.190	0.225	8.950	8.973	8.773	67245	Peli1	chr11	+	NM_0233	P12238	protein_cc	2810468L(	pellino 1	Peli1
2528	Vps54	1.038	1.119	1.162	0.754	0.036	0.463	7.016	7.070	7.232	245944	Vps54	chr11	+	NM_1390	P24351	protein_cc	Hcc8| mSI	vacuolar p	Vps54
2529	Ugp2	0.163	0.977	0.160	0.000	0.791	0.014	11.544	8.931	8.898	216558	Ugp2	chr11	-	NM_0012	P2451	protein_cc	UDPGP| U	UDP-gluco	Ugp2
2530	4932414J(	1.000	1.000	1.000	1.000	1.000	1.000	0.000	0.000	0.000	237694	4932414J(	chr11	-	NR_02825	.	lncRNA	.	RIKEN cDN	4932414J0
2531	Mdh1	0.515	0.931	0.480	0.000	0.544	0.038	12.522	11.566	11.463	17449	Mdh1	chr11	-	NM_0086	P3577	protein_cc	B230377B	malate de	Mdh1
2532	Wdpcp	0.454	0.521	0.237	0.023	0.030	0.080	3.961	2.824	1.882	216560	Wdpcp	chr11	+	NM_1454	P10440	protein_cc	AV249152	WD repea	Wdpcp
2533	Otx1	1.000	1.000	1.000	1.000	1.000	1.000	0.000	0.000	0.000	18423	Otx1	chr11	-	NM_0110	P4724	protein_cc	A730044F	orthodent	Otx1
2534	Ehbp1	0.453	0.844	0.383	0.001	0.378	0.038	6.597	5.456	5.211	216565	Ehbp1	chr11	-	NM_1530	P1437	protein_cc	AF424697	EH domair	Ehbp1
2535	Tmem17	0.869	1.798	1.563	0.796	0.215	0.259	3.768	3.566	4.412	103765	Tmem17	chr11	+	NM_1535	P10172	protein_cc	AI503894|	transmem	Tmem17

2536	B3gnt2	2.258	0.465	1.051	0.000	0.002	0.855	7.633	8.809	7.705	53625	B3gnt2	chr11	-	NM_0168	P3399	protein_cc	AA408337	UDP-GlcN	B3gnt2
2537	Commd1	0.637	1.268	0.808	0.003	0.132	0.244	8.526	7.876	8.219	17846	Commd1	chr11	-	NM_1445	P8897	protein_cc	AI256843 |	COMM dc	Commd1
2538	Zrsr1	0.859	0.905	0.778	0.023	0.080	0.063	8.531	8.312	8.168	22183	Zrsr1	chr11	+	NM_0116	P19037	protein_cc	D11Ncvs7	zinc finger	Zrsr1
2539	Cct4	0.947	0.958	0.907	0.212	0.292	0.136	9.397	9.318	9.256	12464	Cct4	chr11	+	NM_0098	P10521	protein_cc	2610204B	chaperoni	Cct4
2540	Fam161a	0.298	2.018	0.601	0.017	0.003	0.302	4.811	3.064	4.077	73873	Fam161a	chr11	+	NM_0286	P13444	protein_cc	4930430E	family wit	Fam161a
2541	1700061J2	1.000	1.000	1.000	1.000	1.000	1.000	0.000	0.000	0.000	76993	1700061J2	chr11	+	NR_13106	.	lncRNA	.	RIKEN cDN	1700061J2
2542	Xpo1	1.868	0.882	1.648	0.000	0.120	0.061	7.557	8.458	8.277	103573	Xpo1	chr11	+	NM_0010	P14016	protein_cc	AA420417	exportin 1	Xpo1
2543	Usp34	0.891	0.839	0.748	0.115	0.100	0.123	9.896	9.730	9.476	17847	Usp34	chr11	+	NM_0011	P9982	protein_cc	A530081C	ubiquitin s	Usp34
2544	Ahsa2	0.894	0.877	0.783	0.103	0.240	0.267	8.920	8.758	8.568	268390	Ahsa2	chr11	-	NM_0012	P2147	protein_cc		AHA1, act	Ahsa2
2545	1700093K	1.000	1.000	1.000	1.000	1.000	1.000	0.000	0.000	0.000	67358	1700093K	chr11	-	NM_0011	P14023	protein_cc	.	RIKEN cDN	1700093K
2546	Gm12060	1.628	0.722	1.176	0.603	0.710	0.824	1.489	2.191	1.722	10013499	Gm12060	chr11	-	NR_00485	.	misc_RNA	OTTMUSG	predicted	Gm12060
2547	0610010F	1.348	1.055	1.422	0.029	0.737	0.371	6.962	7.394	7.471	71675	0610010F	chr11	-	NM_0278	P4398	protein_cc	Kiaa1841 |	RIKEN cDN	0610010F
2548	Pex13	0.880	1.028	0.905	0.405	0.899	0.692	8.370	8.186	8.226	72129	Pex13	chr11	-	NM_0236	P2880	protein_cc	2610008O	peroxisom	Pex13
2549	Pus10	0.808	1.364	1.102	0.137	0.024	0.660	5.842	5.535	5.982	74467	Pus10	chr11	+	NM_0289	P2265	protein_cc	2810013G	pseudouri	Pus10
2550	Rel	4.822	0.920	4.434	0.032	0.851	0.329	2.538	4.808	4.687	19696	Rel	chr11	-	NM_0090	P8234	protein_cc	c-Rel	reticuloen	Rel
2551	Papolg	2.085	0.736	1.534	0.021	0.132	0.005	5.061	6.121	5.678	216578	Papolg	chr11	-	NM_1725	P3813	protein_cc	9630006B	poly(A) po	Papolg
2552	Bcl11a	0.592	0.498	0.295	0.075	0.152	0.004	2.532	1.776	0.769	14025	Bcl11a	chr11	+	NM_0012	P11660	protein_cc	2810047E	B cell CLL/	Bcl11a
2553	4933430M	1.000	1.000	1.000	1.000	1.000	1.000	0.000	0.000	0.000	71316	4933430M	chr11	+	NR_04585	.	lncRNA		RIKEN cDN	4933430M
2554	Gm10466	1.000	1.000	1.000	1.000	1.000	1.000	0.000	0.000	0.000	10003861	Gm10466	chr11	-	NR_03349	.	lncRNA	ENSMUSG	predicted	Gm10466
2555	4933427E	1.000	1.000	1.000	1.000	1.000	1.000	0.000	0.000	0.000	71234	4933427E	chr11	+	NR_04585	.	lncRNA	RP23-117I	RIKEN cDN	4933427E1
2556	5730522E	0.885	0.629	0.557	0.781	1.000	0.422	0.844	0.668	0.000	70626	5730522E	chr11	-	NR_02797	.	lncRNA	RP23-306I	RIKEN cDN	5730522E0
2557	Fancl	1.100	1.266	1.393	0.523	0.013	0.222	6.678	6.815	7.156	67030	Fancl	chr11	+	NM_0259	P26085	protein_cc	2010322C	Fanconi ar	Fancl
2558	Vrk2	1.119	1.192	1.334	0.523	0.182	0.206	6.399	6.561	6.814	69922	Vrk2	chr11	-	NM_0272	P2670	protein_cc	2810003O	vaccinia re	Vrk2
2559	Gm12070	1.023	0.904	0.925	0.946	0.754	0.783	7.215	7.247	7.103	654472	Gm12070	chr11	+	NR_00289	.	misc_RNA	OTTMUSG	predicted	Gm12070
2560	Ccdc85a	1.000	1.000	1.000	1.000	1.000	1.000	0.000	0.000	0.000	216613	Ccdc85a	chr11	-	NM_0011	P15929	protein_cc	.	coiled-coil	Ccdc85a
2561	2810471M	3.363	1.078	3.624	0.303	0.924	0.423	0.001	1.751	1.859	72818	2810471M	chr11	+	NR_04590	.	lncRNA		RIKEN cDN	2810471M
2562	Efemp1	0.429	1.089	0.467	0.000	0.681	0.048	5.658	4.436	4.559	216616	Efemp1	chr11	+	NM_1460	P25053	protein_cc	.	epidermal	Efemp1
2563	Pnpt1	4.401	0.631	2.779	0.003	0.111	0.209	6.603	8.740	8.077	71701	Pnpt1	chr11	+	NM_0278	P10938	protein_cc	1200003F	polyribonu	Pnpt1
2564	Smek2	2.080	0.720	1.498	0.001	0.035	0.262	8.881	9.938	9.464	104570	Smek2	chr11	+	NM_1340	P13607	protein_cc	AW01175	SMEK hom	Smek2
2565	Cfap36	1.342	1.352	1.814	0.089	0.012	0.059	7.473	7.898	8.333	216618	Cfap36	chr11	-	NM_0257	P4236	protein_cc	2300003H	cilia and fl	Cfap36
2566	Ccdc88a	3.913	0.637	2.493	0.000	0.000	0.011	6.193	8.162	7.511	108686	Ccdc88a	chr11	+	NM_1768	P12692	protein_cc	A430106J	coiled coil	Ccdc88a
2567	Prorsd1	1.356	1.027	1.392	0.008	0.779	0.128	9.067	9.506	9.544	67939	Prorsd1	chr11	-	NM_0011	P21131	protein_cc	2010316F	prolyl-tRN	Prorsd1
2568	Mtif2	0.735	1.396	1.027	0.005	0.001	0.682	8.664	8.220	8.702	76784	Mtif2	chr11	+	NM_0012	P18133	protein_cc	2310038D	mitochond	Mtif2
2569	Rps27a	1.388	1.281	1.777	0.001	0.004	0.010	11.540	12.013	12.370	78294	Rps27a	chr11	-	NM_0010	P1303	protein_cc	0610006J1	ribosomal	Rps27a
2570	Clhc1	1.000	1.000	1.000	1.000	1.000	1.000	0.000	0.000	0.000	73324	Clhc1	chr11	+	NM_0011	P8905	protein_cc	1700034F	clathrin he	Clhc1
2571	Rtn4	0.694	0.577	0.401	0.005	0.007	0.006	9.949	9.423	8.631	68585	Rtn4	chr11	+	NM_1940	P18383	protein_cc	1110020G	reticulon 4	Rtn4
2572	Eml6	0.849	0.629	0.534	0.601	1.000	0.022	0.905	0.669	0.000	237711	Eml6	chr11	-	NM_1460	P18093	protein_cc	2900083P	echinoder	Eml6
2573	4931440F	1.166	0.865	1.008	0.701	0.716	0.985	0.455	0.677	0.467	216622	4931440F	chr11	-	NM_1768	P23732	protein_cc	Fem1ab	RIKEN cDN	4931440F1
2574	Sptbn1	1.363	0.969	1.321	0.007	0.744	0.291	9.625	10.071	10.026	20742	Sptbn1	chr11	-	NM_1758	P20386	protein_cc	9930031C	spectrin b	Sptbn1
2575	4930505A	1.000	1.000	1.000	1.000	1.000	1.000	0.000	0.000	0.000	75088	4930505A	chr11	-	NM_0011	P25312	protein_cc	.	RIKEN cDN	4930505A
2576	Acyp2	2.021	1.215	2.456	0.097	0.680	0.410	2.996	4.011	4.292	75572	Acyp2	chr11	-	NM_0293	P1826	protein_cc	2310004B	acylphosp	Acyp2
2577	Psme4	0.648	1.214	0.786	0.000	0.011	0.055	9.964	9.338	9.617	103554	Psme4	chr11	+	NM_1340	P6367	protein_cc	AA409398	proteason	Psme4
2578	Gpr75	1.000	1.000	1.000	1.000	1.000	1.000	0.000	0.000	0.000	237716	Gpr75	chr11	+	NM_1754	P11320	protein_cc	.	G protein-	Gpr75
2579	Erlec1	0.700	1.212	0.848	0.003	0.003	0.172	8.160	7.645	7.922	66753	Erlec1	chr11	-	NM_0257	P15699	protein_cc	4933407N	endoplasn	Erlec1
2580	Asb3	2.335	0.774	1.807	0.004	0.145	0.284	6.408	7.631	7.261	65257	Asb3	chr11	+	NM_0239	P24361	protein_cc	2400011J	ankyrin re	Asb3
2581	Chac2	0.169	1.071	0.181	0.001	0.689	0.040	6.589	4.021	4.120	68044	Chac2	chr11	-	NM_0265	P16848	protein_cc	.	ChaC, cati	Chac2
2582	Stc2	1.539	0.726	1.118	0.307	0.448	0.883	2.059	2.682	2.220	20856	Stc2	chr11	-	NM_0114	P22040	protein_cc	AW12585	stanniocal	Stc2
2583	Bod1	0.831	1.199	0.996	0.082	0.085	0.929	8.770	8.503	8.765	69556	Bod1	chr11	-	NM_0010	P18798	protein_cc	2310022M	biorientat	Bod1
2584	D630024D	0.046	1.258	0.057	0.009	0.649	0.105	5.266	0.809	1.140	414116	D630024D	chr11	-	NR_10231	.	lncRNA		RIKEN cDN	D630024D
2585	Cpeb4	0.682	0.769	0.525	0.028	0.018	0.089	9.203	8.652	8.273	67579	Cpeb4	chr11	+	NM_0262	P24617	protein_cc	Cpe-bp4 |	cytoplasm	Cpeb4
2586	4930524B	1.572	0.636	1.000	0.304	1.000	0.423	0.000	0.653	0.000	67592	4930524B	chr11	+	NM_0262	P5969	protein_cc	.	RIKEN cDN	4930524B1
2587	Nsg2	1.171	1.129	1.322	0.689	0.783	0.667	0.447	0.675	0.850	18197	Nsg2	chr11	+	NM_0087	P8881	protein_cc	.	neuron sp	Nsg2
2588	Gm12108	1.000	1.000	1.000	1.000	1.000	1.000	0.000	0.000	0.000	104692	Gm12108	chr11	-	NR_13063	.	lncRNA	BB052462	predicted	Gm12108
2589	Il9r	1.000	1.000	1.000	1.000	1.000	1.000	0.000	0.000	0.000	16199	Il9r	chr11	-	NM_0011	P11598	protein_cc	.	interleukir	Il9r
2590	Snrnp25	0.626	1.779	1.114	0.000	0.000	0.092	5.644	4.968	5.799	78372	Snrnp25	chr11	+	NM_0300	P23499	protein_cc	3300001G	small nucl	Snrnp25
2591	Rhbdf1	1.905	0.933	1.776	0.012	0.612	0.047	5.007	5.936	5.836	13650	Rhbdf1	chr11	-	NM_0012	P22596	protein_cc	C16ORF8 |	rhomboid	Rhbdf1
2592	Mpg	1.772	1.010	1.789	0.001	0.938	0.123	7.419	8.244	8.258	268395	Mpg	chr11	+	NM_0108	P25398	protein_cc	9830006D	N-methylp	Mpg
2593	Nprl3	1.442	1.031	1.487	0.001	0.819	0.256	7.533	8.062	8.106	17168	Nprl3	chr11	-	NM_1815	P17084	protein_cc	Aag | CGTI	nitrogen p	Nprl3
2594	Hba-x	1.000	1.000	1.000	1.000	1.000	1.000	0.000	0.000	0.000	15126	Hba-x	chr11	+	NM_0104	P4543	protein_cc	AI450015 |	hemoglob	Hba-x
2595	Hba-a1	2.492	0.510	1.271	0.000	0.007	0.485	14.251	15.568	14.597	15122	Hba-a1	chr11	+	NM_0082	P12996	protein_cc	Hba | Hba	hemoglob	Hba-a1
2596	Hba-a2	2.532	0.244	0.618	0.003	0.001	0.024	8.959	10.299	8.265	110257	Hba-a2	chr11	+	NM_0010	P12996	protein_cc	.	hemoglob	Hba-a2
2597	Hbq1b	1.080	0.653	0.706	0.897	0.499	0.641	1.003	1.114	0.500	544763	Hbq1b	chr11	+	NM_0010	P2385	protein_cc	F830116E	hemoglob	Hbq1b
2598	Hbq1a	1.286	0.672	0.864	0.727	0.595	0.856	1.096	1.458	0.885	216635	Hbq1a	chr11	+	NM_1750	P18681	protein_cc	A230090E	hemoglob	Hbq1a
2599	Sh3pxd2b	19.388	0.270	5.238	0.008	0.021	0.052	2.901	7.178	5.290	268396	Sh3pxd2b	chr11	+	NM_1773	P2344	protein_cc	fad49 | G4	SH3 and P	Sh3pxd2b
2600	Ubtd2	2.674	0.740	1.979	0.028	0.401	0.413	5.252	6.672	6.237	327900	Ubtd2	chr11	+	NM_1737	P12745	protein_cc	4930571L	ubiquitin c	Ubtd2
2601	Efcab9	1.005	1.439	1.446	0.000	0.182	0.423	0.000	0.008	0.532	69306	Efcab9	chr11	-	NM_0270	P24403	protein_cc	1700007I0	EF-hand ca	Efcab9
2602	Stk10	3.031	1.324	4.013	0.041	0.219	0.030	3.726	5.326	5.731	20868	Stk10	chr11	+	NM_0092	P18176	protein_cc	Gek1 | Lok	serine/thr	Stk10
2603	Fbxw11	1.515	0.939	1.423	0.003	0.687	0.364	7.790	8.389	8.299	103583	Fbxw11	chr11	+	NM_1340	P3838	protein_cc	2310065A	F-box and	Fbxw11
2604	Smim23	1.000	1.000	1.000	1.000	1.000	1.000	0.000	0.000	0.000	69351	Smim23	chr11	-	NM_0270	P25936	protein_cc	1700008A	small integ	Smim23
2605	Fgf18	1.023	3.051	3.120	0.000	0.182	0.423	0.000	0.033	1.642	14172	Fgf18	chr11	-	NR_10239	.	protein_cc	D130055P	fibroblast	Fgf18
2606	Npm1	1.620	1.034	1.674	0.000	0.505	0.021	10.148	10.844	10.891	18148	Npm1	chr11	-	NM_0087	P9662	protein_cc	B23 | NO3	nucleopho	Npm1
2607	Tlx3	1.000	1.000	1.000	1.000	1.000	1.000	0.000	0.000	0.000	27140	Tlx3	chr11	-	NM_0199	P14089	protein_cc	Hox11l2 |	T cell leuk	Tlx3
2608	Ranbp17	1.010	1.885	1.904	0.000	0.182	0.423	0.000	0.015	0.929	66011	Ranbp17	chr11	-	NM_0231	P25448	protein_cc	.	RAN bindi	Ranbp17
2609	Gabrp	0.115	7.643	0.876	0.006	0.000	0.692	3.249	0.123	3.058	216643	Gabrp	chr11	-	NM_1460	P4538	protein_cc	.	gamma-ar	Gabrp
2610	Kcnip1	1.010	1.825	1.844	0.000	0.182	0.423	0.000	0.015	0.883	70357	Kcnip1	chr11	-	NM_0273	P4948	protein_cc	KCHIP1 | K	Kv channe	Kcnip1
2611	Kcnmb1	1.572	0.636	1.000	0.304	1.000	0.423	0.000	0.653	0.000	16533	Kcnmb1	chr11	+	NM_0311	P10460	protein_cc	BKbeta1	potassium	Kcnmb1
2612	Lcp2	6.755	0.608	4.109	0.000	0.000	0.004	4.953	7.708	6.991	16822	Lcp2	chr11	+	NM_0106	P21360	protein_cc	AI323664 |	lymphocyt	Lcp2
2613	Foxi1	1.000	1.000	1.000	1.000	1.000	1.000	0.000	0.000	0.000	14233	Foxi1	chr11	-	NM_0239	P17360	protein_cc	5830401E	forkhead	Foxi1
2614	Dock2	1.332	0.680	0.905	0.037	0.024	0.735	4.165	4.579	4.022	94176	Dock2	chr11	-	NM_0333	P1122	protein_cc	AI662014 |	dedicator	Dock2
2615	Fam196b	1.000	1.000	1.000	1.000	1.000	1.000	0.000	0.000	0.000	574403	Fam196b	chr11	+	NM_0010	P23281	protein_cc	EG574403	family wit	Fam196b
2616	Spdl1	1.287	1.706	2.196	0.019	0.000	0.016	1.424	1.788	2.559	70385	Spdl1	chr11	-	NM_0274	P10591	protein_cc	1700018I0	spindle ap	Spdl1
2617	Slit3	1.172	1.126	1.319	0.689	0.707	0.357	0.447	0.675	0.846	20564	Slit3	chr11	+	NM_0114	P5277	protein_cc	b2b2362.1	slit homol	Slit3
2618	Pank3	0.662	1.213	0.803	0.030	0.200	0.447	9.912	9.316	9.595	211347	Pank3	chr11	+	NM_1459	P7254	protein_cc	.	pantother	Pank3
2619	Fbll1	1.000	1.000	1.000	1.000	1.000	1.000	0.000	0.000	0.000	237730	Fbll1	chr11	-	NM_0010	P7851	protein_cc	AI595406	fibrillarin-	Fbll1
2620	Rars	2.538	0.773	1.962	0.000	0.021	0.069	8.179	9.522	9.151	104458	Rars	chr11	-	NM_0259	P5565	protein_cc	2610011N	arginyl-tR	Rars
2621	Wwc1	0.199	1.712	0.340	0.036	0.241	0.265	3.086	0.756	1.532	211652	Wwc1	chr11	-	NM_1707	P14089	protein_cc	AA408228	WW, C2 a	Wwc1
2622	Tenm2	1.005	1.439	1.446	0.000	0.182	0.423	0.000	0.008	0.532	23964	Tenm2	chr11	-	NM_0012	P7880	protein_cc	Odz2 | Od	teneurin t	Tenm2
2623	Gm12128	1.053	0.632	0.666	0.897	1.000	0.423	0.587	0.661	0.000	10003914	Gm12128	chr11	+	NR_13115	.	lncRNA	OTTMUSG	predicted	Gm12128
2624	Gm12130	1.000	1.000	1.000	1.000	1.000	1.000	0.000	0.000	0.000	10050291	Gm12130	chr11	-	NR_04029	.	lncRNA	OTTMUSG	predicted	Gm12130
2625	4930553C	1.000	1.000	1.000	1.000	1.000	1.000	0.000	0.000	0.000	75315	4930553C	chr11	-	NR_12643	.	lncRNA	.	RIKEN cDN	4930553C1
2626	Mat2b	0.810	1.367	1.107	0.224	0.096	0.574	8.040	7.736	8.187	108645	Mat2b	chr11	-	NM_0011	P21730	protein_cc	1110064C	methionin	Mat2b

2627	Hmmr	1.305	0.672	0.877	0.211	0.321	0.874	2.544	2.928	2.355	15366	Hmmr	chr11	-	NM_0135	P8163	protein_cc	AA386826	hyalurona	Hmmr
2628	Nudcd2	1.902	1.212	2.306	0.002	0.012	0.016	8.425	9.353	9.630	52653	Nudcd2	chr11	+	NM_0260	P20926	protein_cc	.	NudC dom	Nudcd2
2629	Ccng1	3.958	0.665	2.630	0.000	0.003	0.038	8.050	10.035	9.445	12450	Ccng1	chr11	-	NM_0098	P3805	protein_cc	AI314029	cyclin G1	Ccng1
2630	Gabrg2	1.000	1.000	1.000	1.000	1.000	1.000	0.000	0.000	0.000	14406	Gabrg2	chr11	-	NM_0080	P21089	protein_cc	GABAA-R|	gamma-ar	Gabrg2
2631	Gabra1	0.560	1.371	0.768	0.184	0.191	0.687	0.857	0.021	0.477	14394	Gabra1	chr11	-	NM_0102	P12814	protein_cc	Gabra-1	gamma-ar	Gabra1
2632	Gabra6	0.716	0.995	0.712	0.182	1.000	0.423	0.490	0.008	0.000	14399	Gabra6	chr11	-	NM_0010	P2678	protein_cc	alpha6| G	gamma-ar	Gabra6
2633	Gabrb2	1.026	3.449	3.539	0.000	0.181	0.423	0.001	0.038	1.824	14401	Gabrb2	chr11	+	NM_0080	P17208	protein_cc	AI834970|	gamma-ar	Gabrb2
2634	Atp10b	0.943	2.239	2.111	0.892	0.302	0.558	0.797	0.712	1.875	319767	Atp10b	chr11	+	NM_1769	P23654	protein_cc	5930426O	ATPase, cl	Atp10b
2635	Pttg1	1.001	0.943	0.944	0.981	0.378	0.580	9.913	9.914	9.829	30939	Pttg1	chr11	-	NM_0011	P15063	protein_cc	AW55509	pituitary t	Pttg1
2636	Slu7	1.489	0.810	1.205	0.000	0.000	0.002	8.845	9.420	9.115	193116	Slu7	chr11	+	NM_1989	P4696	protein_cc	AU018913	SLU7 splic	Slu7
2637	C1qtnf2	0.114	1.243	0.142	0.000	0.261	0.003	3.224	0.090	0.403	69183	C1qtnf2	chr11	+	NM_0269	P14741	protein_cc	1810033K	C1q and tu	C1qtnf2
2638	Ccnjl	7.387	0.660	4.874	0.000	0.013	0.003	3.899	6.784	6.184	380694	Ccnjl	chr11	+	NM_0010	P2965	protein_cc	Gm877	cyclin J-lik	Ccnjl
2639	Fabp6	1.000	1.000	1.000	1.000	1.000	1.000	0.000	0.000	0.000	16204	Fabp6	chr11	-	NM_0083	P25857	protein_cc	GT| I-15P|	fatty acid	Fabp6
2640	Pwwp2a	0.614	0.970	0.595	0.000	0.941	0.395	6.527	5.823	5.779	70802	Pwwp2a	chr11	+	NM_0011	P5793	protein_cc	4631424J1	PWWP do	Pwwp2a
2641	Ttc1	2.434	1.000	2.434	0.000	0.999	0.056	8.425	9.709	9.709	66827	Ttc1	chr11	-	NM_1337	P17429	protein_cc	4833412C	tetratricop	Ttc1
2642	Adra1b	0.130	1.355	0.176	0.000	0.561	0.030	10.098	7.151	7.589	11548	Adra1b	chr11	-	NM_0012	P21948	protein_cc	[a]1b	adrenergi	Adra1b
2643	Il12b	10.101	0.124	1.251	0.024	0.027	0.769	0.708	4.045	1.031	16160	Il12b	chr11	+	NM_0013	P339	protein_cc	Il-12b| Il-1	interleuki	Il12b
2644	Ublcp1	1.034	1.349	1.395	0.712	0.030	0.077	6.629	6.677	7.109	79560	Ublcp1	chr11	-	NM_0244	P18995	protein_cc	4930527B	ubiquitin-l	Ublcp1
2645	Rnf145	0.791	0.677	0.536	0.017	0.114	0.163	7.812	7.474	6.912	74315	Rnf145	chr11	+	NM_0288	P3493	protein_cc	3732413I1	ring finger	Rnf145
2646	4930597A	1.005	1.439	1.446	0.000	0.182	0.423	0.000	0.008	0.532	75389	4930597A	chr11	+	NR_12646	.	lncRNA	.	RIKEN cDN	4930597A
2647	Ebf1	1.882	0.685	1.290	0.004	0.002	0.386	2.978	3.890	3.345	13591	Ebf1	chr11	+	NM_0012	P26787	protein_cc	Ebf| O/E-1	early B cel	Ebf1
2648	Gm12159	0.405	0.985	0.399	0.181	1.000	0.423	1.324	0.022	0.000	10050425	Gm12159	chr11	-	NR_04510	.	lncRNA	OTTMUSG	predicted	Gm12159
2649	F630206G	0.716	0.995	0.712	0.182	1.000	0.423	0.490	0.008	0.000	10000966	F630206G	chr11	+	NR_04587	.	lncRNA	.	RIKEN cDN	F630206G
2650	Clint1	1.417	0.724	1.026	0.001	0.000	0.699	9.163	9.666	9.200	216705	Clint1	chr11	+	NM_0010	P16085	protein_cc	AI642036|	clathrin in	Clint1
2651	Lsm11	1.516	0.860	1.304	0.030	0.179	0.242	5.279	5.879	5.662	72290	Lsm11	chr11	-	NM_0281	P18399	protein_cc	2210404M	U7 snRNP	Lsm11
2652	Thg1l	2.408	0.595	1.432	0.024	0.083	0.415	3.577	4.844	4.095	66628	Thg1l	chr11	-	NM_0010	P1005	protein_cc	.	tRNA-histi	Thg1l
2653	Sox30	1.572	0.636	1.000	0.304	1.000	0.423	0.000	0.653	0.000	214105	Sox30	chr11	+	NM_1733	P11862	protein_cc	.	SRY (sex d	Sox30
2654	Adam19	1.453	0.796	1.157	0.279	0.495	0.608	3.358	3.897	3.568	11492	Adam19	chr11	+	NM_0012	P4270	protein_cc	AL024287	a disinteg	Adam19
2655	Nipal4	1.000	1.000	1.000	1.000	1.000	1.000	0.000	0.000	0.000	214112	Nipal4	chr11	-	NM_1725	P19296	protein_cc	9530066K	NIPA-like	Nipal4
2656	Cyfip2	3.570	0.646	2.306	0.049	0.247	0.050	3.874	5.710	5.080	76884	Cyfip2	chr11	-	NM_1337	P7028	protein_cc	1500004I0	cytoplasm	Cyfip2
2657	Fndc9	2.134	0.469	1.000	0.305	1.000	0.423	0.000	1.094	0.000	320116	Fndc9	chr11	+	NM_1770	P3810	protein_cc	C030019I0	fibronecti	Fndc9
2658	Itk	4.138	0.570	2.357	0.003	0.237	0.592	1.646	3.696	2.883	16428	Itk	chr11	-	NM_0105	P79	protein_cc	Emt| Tcsk	IL2 induci	Itk
2659	Fam71b	1.000	1.000	1.000	1.000	1.000	1.000	0.000	0.000	0.000	432552	Fam71b	chr11	+	NM_0010	P15776	protein_cc	Gari-l3| O	family wit	Fam71b
2660	Med7	2.386	0.733	1.748	0.000	0.051	0.178	7.068	8.322	7.873	66213	Med7	chr11	+	NM_0011	P26785	protein_cc	1110063B	mediator	Med7
2661	Havcr2	1.638	3.405	5.579	0.278	0.010	0.054	0.001	0.713	2.481	171285	Havcr2	chr11	+	NM_1342	P16940	protein_cc	TIM-3| Tir	hepatitis A	Havcr2
2662	Gm12169	1.000	1.000	1.000	1.000	1.000	1.000	0.000	0.000	0.000	210535	Gm12169	chr11	+	NM_0011	P10619	protein_cc	OTTMUSG	predicted	Gm12169
2663	Gm12171	1.000	1.000	1.000	1.000	1.000	1.000	0.000	0.000	0.000	675689	Gm12171	chr11	+	NM_0011	P15827	protein_cc	OTTMUSG	predicted	Gm12171
2664	BC053393	1.000	1.000	1.000	1.000	1.000	1.000	0.000	0.000	0.000	407814	BC053393	chr11	+	NM_0010	P26383	protein_cc	RP23-248I	cDNA seq	BC053393
2665	Dppa1	3.269	0.306	1.000	0.305	1.000	0.423	0.001	1.710	0.000	347708	Dppa1	chr11	-	NM_0011	P3906	protein_cc	C77727	developm	Dppa1
2666	Gm4926	1.000	1.000	1.000	1.000	1.000	1.000	0.000	0.000	0.000	237749	Gm4926	chr11	-	NR_02806	.	misc_RNA	EG237749	predicted	Gm4926
2667	Timd2	0.556	0.775	0.431	0.006	0.042	0.038	9.477	8.629	8.262	171284	Timd2	chr11	-	NM_0011	P6564	protein_cc	AA816106	T cell imm	Timd2
2668	Havcr1	0.293	1.380	0.404	0.182	0.202	0.498	1.810	0.039	0.504	171283	Havcr1	chr11	+	NM_0011	P24538	protein_cc	AI503787|	hepatitis A	Havcr1
2669	Timd4	1.362	0.912	1.242	0.345	0.764	0.795	5.160	5.605	5.473	276891	Timd4	chr11	+	NM_1787	P3830	protein_cc	B430010N	T cell imm	Timd4
2670	Sgcd	1.000	1.000	1.000	1.000	1.000	1.000	0.000	0.000	0.000	24052	Sgcd	chr11	-	NM_0118	P9623	protein_cc	35kDa| de	sarcoglyca	Sgcd
2671	Gnb2l1	1.328	1.310	1.740	0.010	0.067	0.099	11.437	11.847	12.236		Gnb2l1	chr11	+	NM_0081	P5990		.	.	Gnb2l1
2672	Snord96a	0.628	0.802	0.503	0.488	0.572	0.609	2.174	1.502	1.184	10021653	Snord96a	chr11	+	NR_02856	.	snoRNA	U96	small nucl	Snord96a
2673	Snord95	1.010	1.825	1.844	0.000	0.182	0.423	0.000	0.015	0.883	10021654	Snord95	chr11	+	NR_02856	.	snoRNA	.	small nucl	Snord95
2674	Trim41	0.874	1.095	0.957	0.083	0.344	0.790	7.640	7.446	7.576	211007	Trim41	chr11	-	NM_1453	P22098	protein_cc	AW55270	tripartite	Trim41
2675	Trim7	0.750	2.739	2.055	0.200	0.182	0.516	0.448	0.033	1.487	94089	Trim7	chr11	+	NM_0531	P9478	protein_cc	AI790312	tripartite	Trim7
2676	Irgm1	11.569	0.373	4.310	0.002	0.035	0.389	6.576	10.108	8.683	15944	Irgm1	chr11	-	NM_0083	P25584	protein_cc	Ifggd3| Ifi	immunity-	Irgm1
2677	Gm5431	4.155	0.242	1.007	0.067	0.068	0.993	3.917	5.971	3.926	432555	Gm5431	chr11	-	NM_0010	P4806	protein_cc	EG432555	predicted	Gm5431
2678	Gm12185	56.488	0.063	3.534	0.010	0.012	0.343	0.988	6.808	2.809	620913	Gm12185	chr11	-	NM_0010	P5123	protein_cc	OTTMUSG	predicted	Gm12185
2679	Psme2b	2.298	0.706	1.623	0.006	0.043	0.029	9.932	11.132	10.631	621823	Psme2b	chr11	-	NM_0012	P11894	protein_cc	PA28b-p|	protease (	Psme2b
2680	9930111J	4.584	0.379	1.739	0.023	0.080	0.638	1.020	3.217	1.819	667214	9930111J	chr11	-	NM_0011	P3721	protein_cc	9930111J	RIKEN cDN	9930111J
2681	9930111J	3.358	0.176	0.591	0.113	1.000	0.422	0.758	2.505	0.000	245240	9930111J	chr11	-	NM_1734	P15350	protein_cc	9930111J	RIKEN cDN	9930111J
2682	Tgtp1	78.585	0.135	10.577	0.000	0.000	0.269	5.440	11.736	8.843	21822	Tgtp1	chr11	-	NM_0115	P26222	protein_cc	Gtp2| Irgb	T cell spec	Tgtp1
2683	Olfr56	112.528	0.528	59.444	0.013	0.145	0.121	0.003	6.817	5.896	18356	Olfr56	chr11	+	NM_0109	P22355	protein_cc	IF7| MOR	olfactory	Olfr56
2684	Tgtp2	65.884	0.143	9.425	0.000	0.000	0.261	5.709	11.750	8.945	10003979	Tgtp2	chr11	-	NM_0011	P5107	protein_cc	10003979	T cell spec	Tgtp2
2685	Ifi47	34.632	0.208	7.216	0.001	0.005	0.288	8.270	13.384	11.121	15953	Ifi47	chr11	+	NM_0012	P1102	protein_cc	47kDa| Ifg	interferon	Ifi47
2686	Olfr1396	1.572	0.636	1.000	0.304	1.000	0.423	0.000	0.653	0.000	258334	Olfr1396	chr11	-	NM_1463	P17057	protein_cc	MOR276-2	olfactory	Olfr1396
2687	Olfr1395	1.000	1.000	1.000	1.000	1.000	1.000	0.000	0.000	0.000	258877	Olfr1395	chr11	+	NM_1468	P23683	protein_cc	MOR277-1	olfactory	Olfr1395
2688	Olfr1394	1.000	1.000	1.000	1.000	1.000	1.000	0.000	0.000	0.000	258273	Olfr1394	chr11	+	NM_1462	P15544	protein_cc	MOR280-1	olfactory	Olfr1394
2689	Btnl9	0.113	1.708	0.193	0.000	0.208	0.018	3.236	0.093	0.865	237754	Btnl9	chr11	-	NM_1727	P24236	protein_cc	B430208I0	butyrophi	Btnl9
2690	Zfp62	2.687	0.539	1.449	0.044	0.121	0.372	4.945	6.371	5.480	22720	Zfp62	chr11	+	NM_0095	P26110	protein_cc	.	zinc finger	Zfp62
2691	Mgat1	0.806	0.778	0.627	0.018	0.078	0.081	9.653	9.342	8.980	17308	Mgat1	chr11	+	NM_0107	P10699	protein_cc	Mgat-1	mannosid	Mgat1
2692	Olfr1393	1.000	1.000	1.000	1.000	1.000	1.000	0.000	0.000	0.000	258463	Olfr1393	chr11	+	NM_1464	P20339	protein_cc	MOR256-2	olfactory	Olfr1393
2693	Olfr1392	1.000	1.000	1.000	1.000	1.000	1.000	0.000	0.000	0.000	258462	Olfr1392	chr11	+	NM_1464	P3512	protein_cc	MOR256-2	olfactory	Olfr1392
2694	Olfr10	1.000	1.000	1.000	1.000	1.000	1.000	0.000	0.000	0.000	18307	Olfr10	chr11	+	NM_2068	P13587	protein_cc	GA_x6K02	olfactory	Olfr10
2695	Olfr1391	1.000	1.000	1.000	1.000	1.000	1.000	0.000	0.000	0.000	258460	Olfr1391	chr11	+	NM_1464	P6830	protein_cc	MOR256-2	olfactory	Olfr1391
2696	Olfr1390	1.000	1.000	1.000	1.000	1.000	1.000	0.000	0.000	0.000	259068	Olfr1390	chr11	+	NM_1470	P26899	protein_cc	MOR256-2	olfactory	Olfr1390
2697	Olfr1389	1.000	1.000	1.000	1.000	1.000	1.000	0.000	0.000	0.000	259069	Olfr1389	chr11	+	NM_1470	P14951	protein_cc	MOR256-1	olfactory	Olfr1389
2698	Olfr1388	1.000	1.000	1.000	1.000	1.000	1.000	0.000	0.000	0.000	258459	Olfr1388	chr11	+	NM_1464	P8354	protein_cc	MOR256-2	olfactory	Olfr1388
2699	Olfr1387	1.000	1.000	1.000	1.000	1.000	1.000	0.000	0.000	0.000	258465	Olfr1387	chr11	+	NM_1464	P26291	protein_cc	MOR256-2	olfactory	Olfr1387
2700	Olfr1386	1.000	1.000	1.000	1.000	1.000	1.000	0.000	0.000	0.000	257888	Olfr1386	chr11	+	NM_0010	P21885	protein_cc	MOR256-5	olfactory	Olfr1386
2701	Olfr1385	1.000	1.000	1.000	1.000	1.000	1.000	0.000	0.000	0.000	258027	Olfr1385	chr11	+	NM_0010	P2613	protein_cc	MOR256-4	olfactory	Olfr1385
2702	Olfr1384	1.000	1.000	1.000	1.000	1.000	1.000	0.000	0.000	0.000	258464	Olfr1384	chr11	+	NM_1464	P13081	protein_cc	MOR256-2	olfactory	Olfr1384
2703	Olfr1383	1.000	1.000	1.000	1.000	1.000	1.000	0.000	0.000	0.000	404337	Olfr1383	chr11	+	NM_2075	P7803	protein_cc	MOR256-5	olfactory	Olfr1383
2704	Olfr1382	1.000	1.000	1.000	1.000	1.000	1.000	0.000	0.000	0.000	257971	Olfr1382	chr11	+	NM_0010	P14811	protein_cc	GA_x5J8B	olfactory	Olfr1382
2705	Olfr1381	1.000	1.000	1.000	1.000	1.000	1.000	0.000	0.000	0.000	258461	Olfr1381	chr11	+	NM_1464	P2967	protein_cc	MOR256-2	olfactory	Olfr1381
2706	Olfr1380	1.000	1.000	1.000	1.000	1.000	1.000	0.000	0.000	0.000	404336	Olfr1380	chr11	+	NM_2075	P24015	protein_cc	GA_x5J8B	olfactory	Olfr1380
2707	Flt4	0.367	2.430	0.892	0.010	0.108	0.837	7.443	5.996	7.277	14257	Flt4	chr11	+	NM_0080	P2536	protein_cc	AI323512|	FMS-like t	Flt4
2708	Scgb3a1	1.005	1.439	1.446	0.000	0.182	0.423	0.000	0.008	0.532	68662	Scgb3a1	chr11	+	NM_1707	P8241	protein_cc	HIN-1| Lul	secretoglo	Scgb3a1
2709	Cnot6	0.928	1.175	1.090	0.530	0.354	0.723	8.869	8.761	8.994	104625	Cnot6	chr11	-	NM_2124	P10561	protein_cc	CCR4	CCR4-NOT	Cnot6
2710	Gfpt2	11.797	0.499	5.883	0.004	0.050	0.151	1.625	5.185	4.181	14584	Gfpt2	chr11	+	NM_0135	P11278	protein_cc	AI480523|	glutamine	Gfpt2
2711	Mapk9	0.331	1.530	0.506	0.000	0.040	0.048	9.578	7.982	8.596	26420	Mapk9	chr11	+	NM_0011	P12950	protein_cc	AI851083|	mitogen-a	Mapk9
2712	Rasgef1c	0.669	0.995	0.666	0.181	1.000	0.423	0.587	0.008	0.000	74563	Rasgef1c	chr11	+	NM_0290	P4049	protein_cc	9130006A	RasGEF do	Rasgef1c
2713	Rnf130	0.420	1.829	0.768	0.003	0.010	0.168	9.066	7.813	8.685	59044	Rnf130	chr11	+	NM_0012	P21482	protein_cc	2510042A	ring finger	Rnf130
2714	Tbc1d9b	0.605	0.959	0.580	0.006	0.795	0.046	8.930	8.204	8.144	76795	Tbc1d9b	chr11	+	NM_0012	P24525	protein_cc	.	TBC1 dom	Tbc1d9b
2715	3010026O	0.063	3.270	0.207	0.000	0.204	0.030	6.224	2.246	3.955	68067	3010026O	chr11	+	NM_0265	P5078	protein_cc	2900016G	RIKEN cDN	3010026O
2716	Sqstm1	0.795	1.110	0.883	0.001	0.373	0.507	11.484	11.154	11.304	18412	Sqstm1	chr11	-	NM_0012	P3864	protein_cc	A170| OSI	sequestos	Sqstm1
2717	Mgat4b	0.356	1.402	0.499	0.000	0.222	0.107	8.471	6.979	7.467	103534	Mgat4b	chr11	+	NM_1459	P22135	protein_cc	AA407995	mannosid	Mgat4b

2718	Ltc4s	1.524	0.648	0.987	0.649	0.657	0.989	1.742	2.350	1.724	17001	Ltc4s	chr11	-	NM_0085	P22610	protein_cc	.	leukotrien	Ltc4s
2719	Maml1	1.150	0.900	1.035	0.381	0.438	0.925	6.398	6.600	6.448	103806	Maml1	chr11	-	NM_1753	P3853	protein_cc	AI644666|	mastermi	Maml1
2720	Canx	0.852	0.970	0.826	0.001	0.726	0.251	11.625	11.393	11.348	12330	Canx	chr11	-	NM_0011	P2969	protein_cc	1110069N	calnexin	Canx
2721	Hnrnph1	2.129	0.461	0.982	0.000	0.000	0.694	7.863	8.953	7.836	59013	Hnrnph1	chr11	+	NM_0215	P7889	protein_cc	AI642080|	heterogen	Hnrnph1
2722	Rufy1	1.205	0.702	0.846	0.299	0.141	0.484	7.163	7.432	6.921	216724	Rufy1	chr11	-	NM_1725	P22549	protein_cc	3000002E	RUN and F	Rufy1
2723	Adamts2	0.250	2.210	0.553	0.000	0.001	0.016	6.314	4.315	5.459	216725	Adamts2	chr11	+	NM_1756	P8385	protein_cc	ADAM-TS	a disinteg	Adamts2
2724	Zfp354c	0.569	3.139	1.786	0.169	0.033	0.229	1.986	1.173	2.823	30944	Zfp354c	chr11	-	NM_0139	P15167	protein_cc	5330421P	zinc finger	Zfp354c
2725	Zfp879	0.586	1.268	0.743	0.215	0.650	0.742	2.796	2.025	2.368	214779	Zfp879	chr11	-	NM_0012	P5250	protein_cc	Znf879	zinc finger	Zfp879
2726	Grm6	1.572	0.636	1.000	0.304	1.000	0.423	0.000	0.653	0.000	108072	Grm6	chr11	+	NM_1733	P9614	protein_cc	BC021919	glutamate	Grm6
2727	Zfp454	0.368	1.660	0.610	0.020	0.229	0.015	2.627	1.183	1.914	237758	Zfp454	chr11	-	NM_1727	P2869	protein_cc	B230342N	zinc finger	Zfp454
2728	Zfp2	0.357	1.397	0.499	0.007	0.099	0.107	6.750	5.265	5.748	22678	Zfp2	chr11	-	NM_0012	P26758	protein_cc	9930007F	zinc finger	Zfp2
2729	Zfp354b	0.518	1.221	0.632	0.399	0.630	0.718	3.508	2.558	2.845	27274	Zfp354b	chr11	-	NM_0137	P3253	protein_cc	Kid2| Tcf1	zinc finger	Zfp354b
2730	Prop1	1.000	1.000	1.000	1.000	1.000	1.000	0.000	0.000	0.000	19127	Prop1	chr11	-	NM_0089	P18535	protein_cc	df| Prop-1	paired like	Prop1
2731	Olfr1378	1.000	1.000	1.000	1.000	1.000	1.000	0.000	0.000	0.000	258912	Olfr1378	chr11	+	NM_1469	P12162	protein_cc	MOR129-2	olfactory r	Olfr1378
2732	Olfr1377	1.000	1.000	1.000	1.000	1.000	1.000	0.000	0.000	0.000	258913	Olfr1377	chr11	+	NM_1469	P20528	protein_cc	MOR129-1	olfactory r	Olfr1377
2733	Olfr51	1.000	1.000	1.000	1.000	1.000	1.000	0.000	0.000	0.000	18351	Olfr51	chr11	+	NM_1469	P11700	protein_cc	ID7| MOR	olfactory r	Olfr51
2734	Olfr54	0.716	0.995	0.712	0.182	1.000	0.423	0.490	0.008	0.000	18354	Olfr54	chr11	+	NM_0109	P6363	protein_cc	F3| MOR1	olfactory r	Olfr54
2735	Olfr1375	1.000	1.000	1.000	1.000	1.000	1.000	0.000	0.000	0.000	258509	Olfr1375	chr11	+	NM_1465	P557	protein_cc	MOR126-2	olfactory r	Olfr1375
2736	Zfp354a	0.785	0.915	0.719	0.286	0.626	0.506	4.738	4.389	4.261	21408	Zfp354a	chr11	+	NM_0093	P21520	protein_cc	AW48848	zinc finger	Zfp354a
2737	9230009I0	1.000	1.000	1.000	1.000	1.000	1.000	0.000	0.000	0.000	619293	9230009I0	chr11	+	NR_04586	.	lncRNA	.	RIKEN cDN	9230009I0
2738	Platr20	1.000	1.000	1.000	1.000	1.000	1.000	0.000	0.000	0.000	71297	Platr20	chr11	-	NR_11044	.	lncRNA	5133400J0	pluripoten	Platr20
2739	BC049762	2.682	0.492	1.319	0.003	0.021	0.561	2.135	3.558	2.534	193286	BC049762	chr11	-	NM_1775	P10610	protein_cc	4930503F	cDNA sequ	BC049762
2740	Clk4	1.506	0.983	1.480	0.007	0.900	0.219	6.855	7.446	7.421	12750	Clk4	chr11	+	NM_0077	P12555	protein_cc	AI987988|	CDC like k	Clk4
2741	Col23a1	2.017	1.226	2.473	0.191	0.743	0.525	0.762	1.775	2.068	237759	Col23a1	chr11	+	NM_1533	P26860	protein_cc	2810458L	collagen, t	Col23a1
2742	Phykpl	0.578	1.530	0.885	0.010	0.082	0.643	7.319	6.529	7.143	72947	Phykpl	chr11	+	NM_0283	P26708	protein_cc	2900006B	5-phospho	Phykpl
2743	Hnrnpab	1.737	1.031	1.791	0.001	0.837	0.175	10.410	11.206	11.250	15384	Hnrnpab	chr11	-	NM_0104	P16129	protein_cc	3010025C	heterogen	Hnrnpab
2744	Nhp2	1.647	1.721	2.835	0.042	0.013	0.022	7.007	7.727	8.511	52530	Nhp2	chr11	+	NM_0266	P10688	protein_cc	2410130M	NHP2 ribo	Nhp2
2745	Rmnd5b	1.057	1.174	1.241	0.318	0.025	0.119	6.931	7.011	7.242	66089	Rmnd5b	chr11	-	NM_0253	P21584	protein_cc	0610039K	required f	Rmnd5b
2746	N4bp3	1.463	0.702	1.028	0.136	0.393	0.977	4.545	5.094	4.584	212706	N4bp3	chr11	-	NM_1459	P12270	protein_cc	C330016O	NEDD4 bi	N4bp3
2747	D930048N	0.231	2.711	0.625	0.019	0.268	0.583	2.862	0.746	2.184	97775	D930048N	chr11	+	NR_02795	.	lncRNA	AI315703|	RIKEN cDN	D930048N
2748	0610009B	1.117	0.844	0.943	0.555	0.349	0.794	7.682	7.843	7.598	66050	0610009B	chr11	-	NM_0253	P13320	protein_cc	RP23-79E1	RIKEN cDN	0610009B2
2749	Sec24a	0.579	1.229	0.711	0.001	0.191	0.184	9.703	8.914	9.212	77371	Sec24a	chr11	-	NM_0012	P6572	protein_cc	.	Sec24 rela	Sec24a
2750	Sar1b	0.288	1.279	0.369	0.004	0.111	0.072	11.024	9.230	9.585	66397	Sar1b	chr11	+	NM_0255	P8159	protein_cc	2310075M	secretion	Sar1b
2751	Jade2	5.736	0.490	2.811	0.000	0.003	0.038	6.434	8.954	7.925	76901	Jade2	chr11	-	NM_1992	P11363	protein_cc	1200017K	jade famil	Jade2
2752	Cdkn2aipn	2.078	1.214	2.522	0.001	0.030	0.036	6.855	7.910	8.190	52626	Cdkn2aipn	chr11	+	NM_0299	P1291	protein_cc	A430101B	CDKN2A in	Cdkn2aipn
2753	Ube2b	0.988	0.928	0.917	0.647	0.536	0.674	10.403	10.385	10.278	22210	Ube2b	chr11	-	NM_0094	P25187	protein_cc	2610301N	ubiquitin-	Ube2b
2754	Cdkl3	0.402	3.070	1.234	0.036	0.175	0.791	2.494	1.179	2.798	213084	Cdkl3	chr11	+	NM_0011	P9671	protein_cc	AI852479|	cyclin-dep	Cdkl3
2755	Ppp2ca	1.481	0.861	1.276	0.006	0.087	0.088	9.423	9.990	9.774	19052	Ppp2ca	chr11	+	NM_0194	P11062	protein_cc	PP2A| R75	protein ph	Ppp2ca
2756	Olfr1373	1.000	1.000	1.000	1.000	1.000	1.000	0.000	0.000	0.000	211472	Olfr1373	chr11	-	NM_2072	P6528	protein_cc	GA_x6K02	olfactory r	Olfr1373
2757	Olfr1372-p	0.363	2.544	0.923	0.063	0.185	0.922	1.511	0.048	1.395	257871	Olfr1372-p	chr11	-	NR_03415	.	misc_RNA	GA_x5J8B	olfactory r	Olfr1372-p
2758	Olfr1371	1.000	1.000	1.000	1.000	1.000	1.000	0.000	0.000	0.000	276865	Olfr1371	chr11	-	NM_2072	P3228	protein_cc	GA_x6K02	olfactory r	Olfr1371
2759	Skp1a	0.532	1.353	0.720	0.001	0.009	0.112	11.026	10.115	10.551	21402	Skp1a	chr11	+	NM_0115	P10918	protein_cc	15kDa| 26	S-phase ki	Skp1a
2760	Tcf7	0.208	2.085	0.433	0.182	0.021	0.555	2.336	0.067	1.127	21414	Tcf7	chr11	-	NM_0093	P16533	protein_cc	AI465550|	transcripti	Tcf7
2761	Vdac1	0.402	1.060	0.426	0.000	0.300	0.002	10.891	9.575	9.659	22333	Vdac1	chr11	+	NM_0116	P16351	protein_cc	AL033343	voltage-de	Vdac1
2762	9530068E	0.821	1.041	0.855	0.170	0.775	0.188	9.802	9.518	9.577	213673	9530068E	chr11	+	NM_1531	P7942	protein_cc	C85061| K	RIKEN cDN	9530068E0
2763	Fstl4	0.716	0.995	0.712	0.182	1.000	0.423	0.490	0.008	0.000	320027	Fstl4	chr11	+	NM_1770	P1370	protein_cc	B230374F	follistatin-	Fstl4
2764	Gm11186	1.000	1.000	1.000	1.000	1.000	1.000	0.000	0.000	0.000	790909	Gm11186	chr11	-	NR_04604	.	lncRNA	OTTMUSG	predicted	Gm11186
2765	Hspa4	1.116	0.828	0.924	0.102	0.063	0.495	10.604	10.762	10.490	15525	Hspa4	chr11	-	NM_0083	P22083	protein_cc	70kDa| AI	heat shock	Hspa4
2766	Zcchc10	1.626	0.853	1.388	0.003	0.164	0.153	6.759	7.461	7.232	67966	Zcchc10	chr11	+	NM_0264	P25847	protein_cc	2410141K	zinc finger	Zcchc10
2767	Aff4	2.618	0.796	2.083	0.000	0.043	0.066	8.649	10.037	9.708	93736	Aff4	chr11	+	NM_0335	P22750	protein_cc	AF5Q31| A	AF4/FMR2	Aff4
2768	Leap2	3.069	0.664	2.038	0.000	0.017	0.120	11.218	12.836	12.245	259301	Leap2	chr11	-	NM_1530	P16339	protein_cc	leap-2	liver-expre	Leap2
2769	Uqcrq	0.803	1.144	0.918	0.032	0.058	0.365	13.190	12.874	13.068	22272	Uqcrq	chr11	-	NM_0253	P18791	protein_cc	1100001F	ubiquinol-	Uqcrq
2770	Gdf9	5.150	0.396	2.040	0.057	0.114	0.324	1.461	3.825	2.489	14566	Gdf9	chr11	+	NM_0081	P5248	protein_cc	Gdf-9	growth dif	Gdf9
2771	Shroom1	1.436	0.901	1.294	0.102	0.687	0.590	7.696	8.218	8.068	71774	Shroom1	chr11	+	NM_0279	P17468	protein_cc	Apx| Shrm	shroom fa	Shroom1
2772	Sowaha	1.160	0.898	1.041	0.707	0.786	0.930	0.455	0.669	0.513	237761	Sowaha	chr11	-	NM_1831	P17009	protein_cc	A830006N	sosondow	Sowaha
2773	Sept8	0.785	0.921	0.723	0.277	0.776	0.335	6.801	6.452	6.334	20362	Sept8	chr11	+	NM_0331	P3331	protein_cc	AW04616	septin 8	Sept8
2774	Kif3a	1.131	1.614	1.825	0.447	0.112	0.277	8.722	8.899	9.589	16568	Kif3a	chr11	+	NM_0084	P12561	protein_cc	Kif3| Kifl|	kinesin fa	Kif3a
2775	Il4	1.000	1.000	1.000	1.000	1.000	1.000	0.000	0.000	0.000	16189	Il4	chr11	-	NR_02749	.	protein_cc	BSF-1| Il-4	interleukin	Il4
2776	Il13	1.000	1.000	1.000	1.000	1.000	1.000	0.000	0.000	0.000	16163	Il13	chr11	-	NM_0083	P23959	protein_cc	Il-13	interleukin	Il13
2777	Rad50	0.731	0.760	0.555	0.092	0.114	0.182	8.057	7.605	7.208	19360	Rad50	chr11	-	NM_0090	P3338	protein_cc	Mrell| Rac	RAD50 do	Rad50
2778	Il5	1.000	1.000	1.000	1.000	1.000	1.000	0.000	0.000	0.000	16191	Il5	chr11	+	NM_0105	P7535	protein_cc	Il-5	interleukin	Il5
2779	Irf1	9.736	0.199	1.941	0.027	0.038	0.057	8.460	11.743	9.416	16362	Irf1	chr11	+	NM_0011	P7286	protein_cc	AU020929	interferon	Irf1
2780	Gm12216	4.169	0.160	0.665	0.031	1.000	0.423	0.588	2.648	0.000	622459	Gm12216	chr11	-	NR_03333	.	lncRNA	602-19-11	predicted	Gm12216
2781	Slc22a5	0.895	0.747	0.669	0.082	0.038	0.143	6.209	6.049	5.629	20520	Slc22a5	chr11	-	NM_0113	P20297	protein_cc	jvs| Lstpl|	solute car	Slc22a5
2782	Slc22a21	3.274	0.920	3.011	0.082	0.788	0.078	1.918	3.629	3.508	56517	Slc22a21	chr11	-	NM_0197	P1419	protein_cc	Octn3| Slc	solute car	Slc22a21
2783	Slc22a4	0.155	1.085	0.168	0.002	0.859	0.034	3.883	1.193	1.311	30805	Slc22a4	chr11	-	NM_0196	P16877	protein_cc	Octn1	solute car	Slc22a4
2784	Pdlim4	0.348	1.146	0.399	0.155	0.238	0.426	3.935	2.413	2.609	30794	Pdlim4	chr11	-	NM_0194	P9689	protein_cc	Ril	PDZ and L	Pdlim4
2785	P4ha2	7.834	0.542	4.248	0.001	0.025	0.091	3.327	6.297	5.414	18452	P4ha2	chr11	+	NM_0110	P25349	protein_cc	AA407196	procollage	P4ha2
2786	4933405E	1.000	1.000	1.000	1.000	1.000	1.000	0.000	0.000	0.000	71056	4933405E	chr11	-	NR_04550	.	lncRNA	.	RIKEN cDN	4933405E2
2787	Csf2	1.000	1.000	1.000	1.000	1.000	1.000	0.000	0.000	0.000	12981	Csf2	chr11	-	NM_0099	P18221	protein_cc	Csfgm| Gr	colony stin	Csf2
2788	Il3	1.580	0.633	1.000	0.305	1.000	0.423	0.000	0.660	0.000	16187	Il3	chr11	-	NM_0105	P14294	protein_cc	BPA| Csfm	interleukin	Il3
2789	Acsl6	0.716	0.995	0.712	0.182	1.000	0.423	0.490	0.008	0.000	216739	Acsl6	chr11	+	NM_0010	P16218	protein_cc	A330035H	acyl-CoA s	Acsl6
2790	Meikin	0.716	0.995	0.712	0.182	1.000	0.423	0.490	0.008	0.000	74847	Meikin	chr11	+	NM_0291	P16100	protein_cc	4930404A	meiotic kin	Meikin
2791	Fnip1	0.697	0.912	0.635	0.000	0.170	0.027	9.503	8.982	8.849	216742	Fnip1	chr11	+	NM_1737	P4955	protein_cc	A730024A	folliculin in	Fnip1
2792	Rapgef6	1.371	0.568	0.779	0.034	0.004	0.249	7.765	8.220	7.405	192786	Rapgef6	chr11	+	NM_0012	P6094	protein_cc	A530068K	Rap guani	Rapgef6
2793	Cdc42se2	1.873	1.225	2.294	0.012	0.263	0.098	6.438	7.343	7.636	72729	Cdc42se2	chr11	-	NM_1786	P26879	protein_cc	2810404F	CDC42 sm	Cdc42se2
2794	Lyrm7os	1.000	1.000	1.000	1.000	1.000	1.000	0.000	0.000	0.000	432562	Lyrm7os	chr11	+	NR_04028	.	lncRNA	Gm12228	LYR motif	Lyrm7os
2795	Lyrm7	0.395	1.553	0.614	0.022	0.338	0.375	4.496	3.157	3.792	75530	Lyrm7	chr11	-	NM_0293	P2543	protein_cc	1700024C	LYR motif	Lyrm7
2796	Hint1	0.751	1.262	0.948	0.004	0.058	0.708	12.863	12.450	12.786	15254	Hint1	chr11	+	NM_0082	P19302	protein_cc	AA673479	histidine t	Hint1
2797	Gpx3	2.532	1.453	3.679	0.001	0.338	0.264	5.861	7.201	7.740	14778	Gpx3	chr11	+	NM_0081	P24373	protein_cc	AA960521	glutathion	Gpx3
2798	Tnip1	10.723	0.644	6.911	0.001	0.044	0.055	6.573	9.996	9.362	57783	Tnip1	chr11	-	NM_0012	P1159	protein_cc	ABIN| ABI	TNFAIP3 in	Tnip1
2799	Anxa6	0.387	1.002	0.387	0.000	0.991	0.018	10.660	9.289	9.291	11749	Anxa6	chr11	-	NM_0134	P11625	protein_cc	Anx6| Anx	annexin A	Anxa6
2800	Ccdc69	3.207	0.580	1.861	0.122	0.298	0.027	0.847	2.528	1.742	52570	Ccdc69	chr11	-	NM_1774	P16485	protein_cc	2210021E	coiled-coil	Ccdc69
2801	Gm2a	0.307	1.188	0.364	0.000	0.084	0.008	10.651	8.945	9.194	14667	Gm2a	chr11	+	NM_0102	P11534	protein_cc	AA408702	GM2 gang	Gm2a
2802	Slc36a3	1.580	0.633	1.000	0.305	1.000	0.423	0.000	0.660	0.000	215332	Slc36a3	chr11	-	NM_1722	P24357	protein_cc	PAT3| TRA	solute car	Slc36a3
2803	Slc36a2	1.039	4.999	5.193	0.000	0.181	0.423	0.001	0.056	2.377	246049	Slc36a2	chr11	-	NM_1531	P4085	protein_cc	A530067G	solute car	Slc36a2
2804	Slc36a1os	1.000	1.000	1.000	1.000	1.000	1.000	0.000	0.000	0.000	74045	Slc36a1os	chr11	-	NR_04603	.	lncRNA	4921508A	solute car	Slc36a1os
2805	Slc36a1	0.381	2.252	0.858	0.006	0.059	0.703	7.904	6.512	7.683	215335	Slc36a1	chr11	+	NM_1531	P23876	protein_cc	5830411H	solute car	Slc36a1
2806	Fat2	1.000	1.000	1.000	1.000	1.000	1.000	0.000	0.000	0.000	245827	Fat2	chr11	-	NM_0010	P18115	protein_cc	EMI2| Fat	FAT atypic	Fat2
2807	Sparc	0.707	1.184	0.837	0.000	0.455	0.591	10.481	9.981	10.225	20692	Sparc	chr11	-	NM_0012	P12274	protein_cc	BM-40| O	secreted a	Sparc
2808	Atox1	0.915	1.079	0.987	0.583	0.551	0.945	10.406	10.278	10.387	11927	Atox1	chr11	-	NM_0097	P12526	protein_cc	AI256639|	antioxidan	Atox1

2809	G3bp1	1.542	0.739	1.139	0.007	0.006	0.399	9.420	10.044	9.608	27041	G3bp1	chr11	+	NM_0137	P23088	protein_cc	AI849976|	GTPase ac	G3bp1
2810	Gm12238	0.691	1.174	0.811	0.631	0.798	0.844	2.036	1.502	1.734	10030374	Gm12238	chr11	+	NR_02848	.	snoRNA	Mbi-39| C	predicted	Gm12238
2811	Glra1	1.000	1.000	1.000	1.000	1.000	1.000	0.000	0.000	0.000	14654	Glra1	chr11	-	NM_0204	P19644	protein_cc	nmf11| os	glycine re	Glra1
2812	Nmur2	1.000	1.000	1.000	1.000	1.000	1.000	0.000	0.000	0.000	216749	Nmur2	chr11	-	NM_1530	P13143	protein_cc	Nmu2r	neuromed	Nmur2
2813	Gria1	0.726	1.387	1.007	0.187	0.187	0.988	0.477	0.015	0.487	14799	Gria1	chr11	+	NM_0011	P21014	protein_cc	2900051M	glutamate	Gria1
2814	Fam114a2	1.217	0.779	0.948	0.174	0.021	0.815	9.379	9.663	9.302	67726	Fam114a2	chr11	-	NM_0011	P26158	protein_cc	1810073G	family wit	Fam114a2
2815	Mfap3	2.063	0.871	1.796	0.000	0.007	0.024	7.256	8.301	8.101	216760	Mfap3	chr11	+	NM_1805	P22763	protein_cc	2610509F	microfibril	Mfap3
2816	Galnt10	1.015	0.812	0.824	0.913	0.292	0.507	5.735	5.756	5.455	171212	Galnt10	chr11	+	NM_1341	P454	protein_cc	AU018154	UDP-N-ac	Galnt10
2817	2010001A	0.424	2.177	0.923	0.051	0.100	0.858	4.826	3.588	4.711	76516	2010001A	chr11	-	NR_13104	.	lncRNA	.	RIKEN cDN	2010001A
2818	Sap30l	0.806	1.471	1.185	0.010	0.002	0.129	8.394	8.082	8.639	50724	Sap30l	chr11	+	NM_0010	P5978	protein_cc	2310079P	SAP30-like	Sap30l
2819	Hand1	1.000	1.000	1.000	1.000	1.000	1.000	0.000	0.000	0.000	15110	Hand1	chr11	-	NM_0082	P14487	protein_cc	bHLHa27|	heart and	Hand1
2820	Larp1	0.531	0.761	0.404	0.019	0.184	0.061	11.481	10.567	10.173	73158	Larp1	chr11	+	NM_0284	P3889	protein_cc	1810024J1	La ribonuc	Larp1
2821	Cnot8	0.620	0.929	0.576	0.037	0.187	0.176	8.561	7.870	7.764	69125	Cnot8	chr11	+	NM_0269	P12447	protein_cc	1500015I0	CCR4-NOT	Cnot8
2822	Gemin5	1.142	0.560	0.640	0.608	0.139	0.190	5.942	6.133	5.298	216766	Gemin5	chr11	-	NM_1725	P19280	protein_cc	AA407055	gem (nucl	Gemin5
2823	Mrpl22	0.679	1.140	0.774	0.000	0.395	0.297	8.242	7.684	7.873	216767	Mrpl22	chr11	+	NM_1750	P5117	protein_cc	E030011D	mitochond	Mrpl22
2824	Gm12250	114.897	0.050	10.390	0.000	0.000	0.331	4.651	11.495	8.028	631323	Gm12250	chr11	+	NM_0011	P498	protein_cc	Irgb10| O	predicted	Gm12250
2825	Igtp	12.270	0.189	2.321	0.001	0.003	0.408	8.570	12.187	9.785	16145	Igtp	chr11	+	NM_0187	P17901	protein_cc	AW55844	interferon	Igtp
2826	Irgm2	6.223	0.238	1.480	0.005	0.008	0.424	8.535	11.173	9.101	54396	Irgm2	chr11	+	NM_0194	P15368	protein_cc	AI481100|	immunity-	Irgm2
2827	Zfp692	0.493	0.886	0.437	0.121	0.858	0.228	4.790	3.771	3.596	103836	Zfp692	chr11	+	NM_0010	P10219	protein_cc	AI746306|	zinc finger	Zfp692
2828	Zfp672	0.908	1.356	1.231	0.416	0.003	0.257	7.068	6.929	7.368	319475	Zfp672	chr11	-	NM_1787	P16254	protein_cc	4930488P	zinc finger	Zfp672
2829	Sh3bp5l	0.592	0.851	0.504	0.020	0.383	0.039	8.673	7.917	7.684	79566	Sh3bp5l	chr11	+	NM_0013	P6335	protein_cc	2310074E	SH3 bindin	Sh3bp5l
2830	Lypd8	1.616	2.282	3.687	0.287	0.295	0.423	0.001	0.693	1.883	70163	Lypd8	chr11	+	NM_0010	P6931	protein_cc	2210415F	LY6/PLAU	Lypd8
2831	1810065E	1.000	1.000	1.000	1.000	1.000	1.000	0.000	0.000	0.000	69864	1810065E	chr11	+	NM_0272	P25590	protein_cc	AV047390	RIKEN cDN	1810065E
2832	Gm12253	0.716	0.995	0.712	0.182	1.000	0.423	0.490	0.008	0.000	624860	Gm12253	chr11	+	NM_0010	P24073	protein_cc	Gm248| C	predicted	Gm12253
2833	4930504O	1.000	1.000	1.000	1.000	1.000	1.000	0.000	0.000	0.000	403200	4930504O	chr11	-	NM_2075	P4305	protein_cc	Gm524	RIKEN cDN	4930504O
2834	Olfr30	1.000	1.000	1.000	1.000	1.000	1.000	0.000	0.000	0.000	18329	Olfr30	chr11	-	NM_1468	P18604	protein_cc	MOR2811	olfactory	Olfr30
2835	Olfr332	1.000	1.000	1.000	1.000	1.000	1.000	0.000	0.000	0.000	257932	Olfr332	chr11	-	NM_0010	P10107	protein_cc	MOR284-2	olfactory	Olfr332
2836	Olfr331	1.000	1.000	1.000	1.000	1.000	1.000	0.000	0.000	0.000	10050288	Olfr331	chr11	-	NM_0010	P13714	protein_cc	MOR275-4	olfactory	Olfr331
2837	Olfr330	0.716	0.995	0.712	0.182	1.000	0.423	0.490	0.008	0.000	258879	Olfr330	chr11	-	NM_1468	P2773	protein_cc	MOR275-1	olfactory	Olfr330
2838	Olfr329-ps	1.000	1.000	1.000	1.000	1.000	1.000	0.000	0.000	0.000	259148	Olfr329-ps	chr11	-	NM_0010	P6837	protein_cc	MOR275-6	olfactory	Olfr329-ps
2839	Olfr328	1.000	1.000	1.000	1.000	1.000	1.000	0.000	0.000	0.000	258495	Olfr328	chr11	-	NM_1465	P8301	protein_cc	MOR275-2	olfactory	Olfr328
2840	Olfr224	1.000	1.000	1.000	1.000	1.000	1.000	0.000	0.000	0.000	258198	Olfr224	chr11	-	NM_2076	P12048	protein_cc	MOR275-3	olfactory	Olfr224
2841	Olfr325	1.000	1.000	1.000	1.000	1.000	1.000	0.000	0.000	0.000	258261	Olfr325	chr11	+	NM_2071	P26468	protein_cc	MOR275-5	olfactory	Olfr325
2842	Olfr324	1.000	1.000	1.000	1.000	1.000	1.000	0.000	0.000	0.000	257892	Olfr324	chr11	+	NM_0010	P2372	protein_cc	MOR102-2	olfactory	Olfr324
2843	2210407C	1.000	1.000	1.000	1.000	1.000	1.000	0.000	0.000	0.000	78354	2210407C	chr11	-	NM_1445	P10719	protein_cc	Ep1| RP23	RIKEN cDN	2210407C
2844	Olfr323	1.000	1.000	1.000	1.000	1.000	1.000	0.000	0.000	0.000	258373	Olfr323	chr11	-	NM_1463	P25364	protein_cc	MOR107-1	olfactory	Olfr323
2845	Trim58	1.590	0.875	1.390	0.302	0.737	0.423	0.000	0.669	0.476	216781	Trim58	chr11	+	NM_0010	P3852	protein_cc	Gm16| Gr	tripartite	Trim58
2846	Olfr322	1.000	1.000	1.000	1.000	1.000	1.000	0.000	0.000	0.000	404346	Olfr322	chr11	+	NM_2076	P11753	protein_cc	GA_x5J8B	olfactory	Olfr322
2847	Olfr320	1.000	1.000	1.000	1.000	1.000	1.000	0.000	0.000	0.000	216783	Olfr320	chr11	+	NM_2072	P19648	protein_cc	GA_x6K02	olfactory	Olfr320
2848	Olfr319	1.000	1.000	1.000	1.000	1.000	1.000	0.000	0.000	0.000	258493	Olfr319	chr11	-	NM_1465	P26178	protein_cc	MOR285-2	olfactory	Olfr319
2849	Olfr318	1.000	1.000	1.000	1.000	1.000	1.000	0.000	0.000	0.000	258494	Olfr318	chr11	-	NM_1465	P3515	protein_cc	MOR285-1	olfactory	Olfr318
2850	Olfr317	1.000	1.000	1.000	1.000	1.000	1.000	0.000	0.000	0.000	257931	Olfr317	chr11	-	NM_0010	P26886	protein_cc	MOR256-4	olfactory	Olfr317
2851	Olfr316	1.000	1.000	1.000	1.000	1.000	1.000	0.000	0.000	0.000	258064	Olfr316	chr11	+	NM_0010	P12898	protein_cc	MOR285-3	olfactory	Olfr316
2852	Olfr315	1.580	0.633	1.000	0.305	1.000	0.423	0.000	0.660	0.000	258531	Olfr315	chr11	+	NM_1465	P17096	protein_cc	MOR278-1	olfactory	Olfr315
2853	Olfr314	1.000	1.000	1.000	1.000	1.000	1.000	0.000	0.000	0.000	257917	Olfr314	chr11	+	NM_0010	P22743	protein_cc	MOR278-2	olfactory	Olfr314
2854	Fam183b	1.005	1.439	1.446	0.000	0.182	0.423	0.000	0.008	0.532	75429	Fam183b	chr11	-	NM_0292	P14035	protein_cc	3100002J2	family wit	Fam183b
2855	Olfr313	1.000	1.000	1.000	1.000	1.000	1.000	0.000	0.000	0.000	258529	Olfr313	chr11	+	NM_1465	P4468	protein_cc	MOR222-2	olfactory	Olfr313
2856	Olfr312	1.000	1.000	1.000	1.000	1.000	1.000	0.000	0.000	0.000	258065	Olfr312	chr11	+	NM_0010	P8925	protein_cc	GA_x5J8B	olfactory	Olfr312
2857	Olfr311	1.000	1.000	1.000	1.000	1.000	1.000	0.000	0.000	0.000	258530	Olfr311	chr11	+	NM_1465	P18390	protein_cc	MOR222-1	olfactory	Olfr311
2858	2810021J	0.974	1.001	0.975	0.949	0.999	0.950	3.602	3.564	3.565	69944	2810021J2	chr11	+	NM_1724	P17053	protein_cc	AI449137|	RIKEN cDN	2810021J2
2859	Zfp39	0.791	1.335	1.057	0.287	0.102	0.868	4.684	4.347	4.764	22698	Zfp39	chr11	-	NM_0117	P6505	protein_cc	CTfin33| Z	zinc finger	Zfp39
2860	Btnl10	1.000	1.000	1.000	1.000	1.000	1.000	0.000	0.000	0.000	192194	Btnl10	chr11	+	NM_1386	P2374	protein_cc	BUTR-1| B	butyrophi	Btnl10
2861	Rnf187	0.479	1.687	0.808	0.000	0.039	0.401	10.107	9.044	9.799	108660	Rnf187	chr11	-	NM_0224	P8743	protein_cc	2410016F	ring finger	Rnf187
2862	Hist3h2ba	1.005	1.405	1.412	0.000	0.182	0.423	0.000	0.008	0.498	78303	Hist3h2ba	chr11	+	NM_0300	P22148	protein_cc	1500011O	histone cl	Hist3h2ba
2863	Hist3h2bb	1.000	1.000	1.000	1.000	1.000	1.000	0.000	0.000	0.000	382522	Hist3h2bb	chr11	+	NM_2068	P8620	misc_RNA	4930534G	histone cl	Hist3h2bb
2864	Hist3h2a	1.605	2.492	3.999	0.056	0.064	0.188	1.345	2.027	3.345	319162	Hist3h2a	chr11	+	NM_1782	P7852	protein_cc	.	histone cl	Hist3h2a
2865	Trim17	3.424	1.606	5.499	0.000	0.000	0.002	0.001	1.777	2.460	56631	Trim17	chr11	+	NM_0311	P25721	protein_cc	Rnf16| ter	tripartite	Trim17
2866	Trim11	2.503	0.517	1.294	0.000	0.001	0.356	7.772	9.096	8.144	94091	Trim11	chr11	+	NM_0012	P5284	protein_cc	.	tripartite	Trim11
2867	Obscn	0.552	0.990	0.547	0.182	1.000	0.423	0.871	0.015	0.000	380698	Obscn	chr11	-	NM_0011	P19854	protein_cc	BC046431	obscurin,	Obscn
2868	Iba57	1.244	0.754	0.937	0.378	0.268	0.602	8.437	8.751	8.343	216792	Iba57	chr11	-	NM_1737	P21432	protein_cc	4930543L	IBA57 hor	Iba57
2869	Gjc2	0.059	1.536	0.091	0.001	0.232	0.046	4.204	0.130	0.749	118454	Gjc2	chr11	-	NM_0804	P17161	protein_cc	B230382L	gap juncti	Gjc2
2870	Guk1	0.958	1.538	1.473	0.435	0.023	0.174	8.846	8.784	9.405	14923	Guk1	chr11	-	NM_0081	P16621	protein_cc	AA409738	guanylate	Guk1
2871	2610507I0	0.348	1.016	0.353	0.000	0.932	0.017	7.576	6.053	6.075	72203	2610507I0	chr11	-	NR_03796	.	lncRNA	5033414D	RIKEN cDN	2610507I0
2872	Mrpl55	0.759	1.597	1.212	0.041	0.026	0.452	8.062	7.664	8.339	67212	Mrpl55	chr11	+	NM_0013	P13829	protein_cc	2810038N	mitochond	Mrpl55
2873	2310033P	1.429	1.296	1.851	0.001	0.005	0.013	6.788	7.303	7.677	67862	2310033P	chr11	+	NM_0242	P17653	protein_cc	Mmtag2	RIKEN cDN	2310033P
2874	Arf1	1.437	0.792	1.139	0.004	0.036	0.415	10.895	11.418	11.082	11840	Arf1	chr11	-	NM_0011	P26209	protein_cc	.	ADP-ribos	Arf1
2875	Wnt3a	1.000	1.000	1.000	1.000	1.000	1.000	0.000	0.000	0.000	22416	Wnt3a	chr11	-	NM_0095	P22848	protein_cc	vt| Wnt-3	wingless-t	Wnt3a
2876	Wnt9a	0.761	0.578	0.439	0.130	0.009	0.106	3.081	2.687	1.895	216795	Wnt9a	chr11	+	NM_1392	P8767	protein_cc	Wnt14	wingless-t	Wnt9a
2877	Prss38	1.000	1.000	1.000	1.000	1.000	1.000	0.000	0.000	0.000	216797	Prss38	chr11	-	NM_0010	P19833	protein_cc	Gm249| N	protease,	Prss38
2878	Snap47	0.403	1.830	0.737	0.000	0.054	0.320	9.966	8.654	9.526	67826	Snap47	chr11	-	NM_1445	P21767	protein_cc	1110031B	synaptoso	Snap47
2879	Jmjd4	1.694	0.725	1.228	0.003	0.049	0.501	5.351	6.111	5.647	194952	Jmjd4	chr11	+	NM_0012	P24254	protein_cc	6430559I2	jumonji do	Jmjd4
2880	Zfp867	2.139	0.721	1.542	0.022	0.212	0.579	4.021	5.118	4.646	237775	Zfp867	chr11	-	NM_1784	P2358	protein_cc	.	zinc finger	Zfp867
2881	Zkscan17	0.716	1.958	1.401	0.017	0.028	0.328	6.743	6.260	7.229	268417	Zkscan17	chr11	-	NM_1729	P911	protein_cc	Nizp1| Zfp	zinc finger	Zkscan17
2882	4933439C	0.664	0.396	0.263	0.162	0.132	0.033	2.981	2.391	1.055	74476	4933439C	chr11	+	NR_01558	.	lncRNA	2610028M	RIKEN cDN	4933439C
2883	Nlrp3	15.316	0.274	4.204	0.000	0.001	0.017	2.197	6.134	4.269	216799	Nlrp3	chr11	+	NM_1458	P17950	protein_cc	AGTAVPRI	NLR family	Nlrp3
2884	Olfr222	1.000	1.000	1.000	1.000	1.000	1.000	0.000	0.000	0.000	257962	Olfr222	chr11	-	NM_0010	P19544	protein_cc	MOR256-4	olfactory	Olfr222
2885	Olfr223	1.000	1.000	1.000	1.000	1.000	1.000	0.000	0.000	0.000	258421	Olfr223	chr11	-	NM_1464	P26489	protein_cc	MOR256-3	olfactory	Olfr223
2886	Olfr225	1.000	1.000	1.000	1.000	1.000	1.000	0.000	0.000	0.000	257886	Olfr225	chr11	+	NM_0010	P7350	protein_cc	MOR256-5	olfactory	Olfr225
2887	Mprip	0.887	0.884	0.784	0.183	0.323	0.362	8.646	8.473	8.294	26936	Mprip	chr11	+	NM_2012	P6571	protein_cc	9530046C	myosin ph	Mprip
2888	1700007J1	1.200	0.897	1.077	0.520	0.674	0.806	1.942	2.205	2.049	69320	1700007J1	chr11	-	NR_04547	.	lncRNA	4930573L	RIKEN cDN	1700007J1
2889	Pld6	0.185	1.351	0.250	0.211	0.561	0.492	3.175	0.740	1.174	194908	Pld6	chr11	-	NM_1831	P24341	protein_cc	4933433K	phospholi	Pld6
2890	Flcn	0.727	0.798	0.580	0.123	0.345	0.059	7.982	7.521	7.196	216805	Flcn	chr11	-	NM_0012	P631	protein_cc	AU014660	folliculin	Flcn
2891	Gm16062	1.268	0.750	0.951	0.018	0.001	0.739	8.456	8.798	8.383	10050410	Gm16062	chr11	+	NR_04568	.	lncRNA	ENSMUSG	predicted	Gm16062
2892	Cops3	1.273	0.799	1.018	0.033	0.008	0.913	8.565	8.913	8.590	26572	Cops3	chr11	-	NM_0119	P12659	protein_cc	Csn3| Sgn	COP9 sign	Cops3
2893	Nt5m	1.388	0.999	1.387	0.046	0.995	0.002	6.051	6.524	6.523	103850	Nt5m	chr11	+	NM_1340	P7219	protein_cc	2010013E	5',3'-nucle	Nt5m
2894	1810063I0	1.000	1.000	1.000	1.000	1.000	1.000	0.000	0.000	0.000	69854	1810063I0	chr11	+	NR_13098	.	lncRNA	.	RIKEN cDN	1810063I0
2895	Med9os	2.134	0.469	1.000	0.305	1.000	0.423	0.000	1.094	0.000	10050412	Med9os	chr11	-	NR_04527	.	lncRNA	1700013G	mediator	Med9os
2896	Med9	3.013	1.305	3.933	0.032	0.315	0.075	6.117	7.708	8.093	192191	Med9	chr11	+	NM_1386	P16171	protein_cc	BC019367	mediator	Med9
2897	Rasd1	12.967	1.720	22.301	0.074	0.290	0.154	2.448	6.145	6.927	19416	Rasd1	chr11	-	NM_0090	P24471	protein_cc	Dexras1	RAS, dexa	Rasd1
2898	Pemt	0.364	1.085	0.396	0.000	0.633	0.026	11.474	10.017	10.136	18618	Pemt	chr11	-	NM_0088	P25164	protein_cc	PEAMT| P	phosphati	Pemt
2899	Rai1	0.398	1.046	0.417	0.006	0.793	0.099	6.828	5.499	5.564	19377	Rai1	chr11	+	NM_0090	P18466	protein_cc	Gt1	retinoic ac	Rai1

2900	Srebf1	0.155	1.022	0.159	0.001	0.948	0.035	9.647	6.962	6.992	20787	Srebf1	chr11	-	NM_0114	P12753	protein_cc	ADD1| bH	sterol regu	Srebf1
2901	Tom1l2	0.714	0.803	0.574	0.026	0.185	0.017	8.320	7.835	7.519	216810	Tom1l2	chr11	-	NM_0010	P6997	protein_cc	2900016IC	target of r	Tom1l2
2902	Lrrc48	0.845	0.647	0.547	0.787	0.491	0.547	1.366	1.123	0.494	74665	Lrrc48	chr11	+	NM_0290	P15476	protein_cc	4930449EI	leucine ric	Lrrc48
2903	Atpaf2	0.720	1.404	1.011	0.144	0.024	0.967	6.482	6.008	6.498	246782	Atpaf2	chr11	-	NM_1454	P9359	protein_cc	ATP12| A1	ATP synth	Atpaf2
2904	Gid4	0.566	1.167	0.660	0.003	0.017	0.102	8.458	7.637	7.860	66771	Gid4	chr11	+	NM_0257	P25786	protein_cc	4933439F:	GID comp	Gid4
2905	Drg2	1.040	1.090	1.134	0.777	0.303	0.624	7.064	7.121	7.245	13495	Drg2	chr11	+	NM_0213	P21723	protein_cc	AI255295|	developm	Drg2
2906	Myo15	1.000	1.000	1.000	1.000	1.000	1.000	0.000	0.000	0.000	17910	Myo15	chr11	+	NM_0108	P13749	protein_cc	Myo15a| :	myosin XV	Myo15
2907	Alkbh5	1.756	0.727	1.276	0.021	0.077	0.053	9.109	9.922	9.461	268420	Alkbh5	chr11	+	NM_1729	P13329	protein_cc	Abh5| AW	alkB homo	Alkbh5
2908	Llgl1	1.018	0.990	1.008	0.879	0.927	0.975	7.703	7.729	7.715	16897	Llgl1	chr11	+	NM_0085	P21438	protein_cc	AI325176|	lethal gian	Llgl1
2909	Flii	0.816	0.905	0.739	0.025	0.106	0.050	8.235	7.942	7.798	14248	Flii	chr11	-	NM_0013	P13395	protein_cc	3632430F(	flightless I	Flii
2910	Mief2	0.326	1.678	0.548	0.003	0.120	0.032	6.944	5.329	6.076	237781	Mief2	chr11	+	NM_0010	P14117	protein_cc	AI482195|	mitochond	Mief2
2911	Top3a	0.502	1.870	0.938	0.024	0.013	0.755	4.644	3.648	4.552	21975	Top3a	chr11	-	NM_0094	P16314	protein_cc	.	topoisome	Top3a
2912	Smcr8	0.259	0.874	0.226	0.003	0.160	0.053	8.858	6.907	6.713	237782	Smcr8	chr11	+	NM_0010	P15236	protein_cc	2310076G	Smith-Ma	Smcr8
2913	Shmt1	0.078	1.105	0.083	0.000	0.337	0.017	10.591	6.863	7.006	20425	Shmt1	chr11	-	NM_0091	P12070	protein_cc	AI324848|	serine hyd	Shmt1
2914	Dhrs7b	0.360	1.624	0.585	0.001	0.019	0.090	8.217	6.744	7.444	216820	Dhrs7b	chr11	+	NM_1454	P19953	protein_cc	BC003479	dehydroge	Dhrs7b
2915	Tmem11	1.331	0.882	1.174	0.072	0.491	0.629	8.912	9.324	9.143	216821	Tmem11	chr11	-	NM_0011	P12776	protein_cc	5730466P	transmem	Tmem11
2916	Natd1	1.949	0.723	1.410	0.010	0.397	0.700	4.948	5.911	5.443	24083	Natd1	chr11	-	NM_0252	P20087	protein_cc	AI256713|	N-acetyltr	Natd1
2917	Map2k3os	0.556	1.540	0.855	0.262	0.472	0.699	2.639	1.791	2.414	24082	Map2k3os	chr11	-	NR_02780	.	lncRNA	F3-1| Gm:	mitogen-a	Map2k3os
2918	Map2k3	0.679	0.923	0.627	0.001	0.400	0.037	10.023	9.465	9.349	26397	Map2k3	chr11	+	NM_0089	P6092	protein_cc	AW21214:	mitogen-a	Map2k3
2919	Kcnj12	1.000	1.000	1.000	1.000	1.000	1.000	0.000	0.000	0.000	16515	Kcnj12	chr11	+	NM_0012	P9286	protein_cc	IRK-2| IRK	potassium	Kcnj12
2920	Tnfrsf13b	1.572	0.636	1.000	0.304	1.000	0.423	0.000	0.653	0.000	57916	Tnfrsf13b	chr11	+	NM_0213	P12500	protein_cc	1200009EI	tumor nec	Tnfrsf13b
2921	Usp22	1.069	1.232	1.318	0.624	0.298	0.367	8.190	8.287	8.588	216825	Usp22	chr11	-	NM_0010	P12634	protein_cc	AI427806	ubiquitin s	Usp22
2922	Aldh3a1	0.895	1.694	1.516	0.790	0.416	0.724	1.332	1.172	1.933	11670	Aldh3a1	chr11	+	NM_0011	P2138	protein_cc	Ahd-4| Ah	aldehyde	Aldh3a1
2923	Aldh3a2	0.068	0.972	0.066	0.000	0.847	0.004	10.733	6.859	6.818	11671	Aldh3a2	chr11	-	NM_0074	P14961	protein_cc	Ahd-3| Ah	aldehyde	Aldh3a2
2924	Slc47a2	1.000	1.000	1.000	1.000	1.000	1.000	0.000	0.000	0.000	380701	Slc47a2	chr11	-	NM_0010	P17154	protein_cc	4933429E:	solute car	Slc47a2
2925	Slc47a1	0.168	0.841	0.142	0.000	0.351	0.005	10.582	8.012	7.761	67473	Slc47a1	chr11	-	NM_0261	P15113	protein_cc	1300013J1	solute car	Slc47a1
2926	Rnf112	1.000	1.000	1.000	1.000	1.000	1.000	0.000	0.000	0.000	22671	Rnf112	chr11	-	NM_0012	P26812	protein_cc	bfp| Zfp17	ring finger	Rnf112
2927	Mfap4	0.235	5.188	1.220	0.184	0.000	0.805	2.177	0.088	2.463	76293	Mfap4	chr11	+	NM_0295	P7394	protein_cc	1110007F:	microfibri	Mfap4
2928	Mapk7	4.275	0.857	3.662	0.006	0.329	0.060	4.256	6.352	6.129	23939	Mapk7	chr11	-	NM_0118	P15214	protein_cc	BMK-1| BI	mitogen-a	Mapk7
2929	B9d1	1.108	1.028	1.139	0.674	0.959	0.889	3.271	3.418	3.459	27078	B9d1	chr11	+	NM_0137	P4181	protein_cc	AW04599	B9 protein	B9d1
2930	Epn2	1.428	1.284	1.833	0.084	0.223	0.138	6.184	6.698	7.058	13855	Epn2	chr11	-	NM_0101	P10043	protein_cc	9530051D	epsin 2	Epn2
2931	Grap	1.403	0.624	0.876	0.225	0.001	0.808	5.116	5.605	4.925	71520	Grap	chr11	+	NM_0278	P26737	protein_cc	8430435N	GRB2-rela	Grap
2932	Slc5a10	2.268	0.957	2.171	0.289	0.939	0.337	2.460	3.642	3.579	109342	Slc5a10	chr11	-	NM_0010	P24274	protein_cc	C330021F	solute car	Slc5a10
2933	Fam83g	1.358	0.443	0.602	0.133	0.034	0.257	5.086	5.527	4.354	69640	Fam83g	chr11	+	NM_1786	P18351	protein_cc	2310040C	family wit	Fam83g
2934	Prpsap2	1.205	0.720	0.868	0.079	0.153	0.702	6.087	6.356	5.883	212627	Prpsap2	chr11	-	NM_0011	P2708	protein_cc	AI427446	phosphori	Prpsap2
2935	Ulk2	0.351	0.871	0.306	0.000	0.552	0.026	9.139	7.629	7.430	29869	Ulk2	chr11	-	NM_0138	P542	protein_cc	A830085I2	unc-51 like	Ulk2
2936	Akap10	1.245	0.659	0.820	0.661	0.444	0.845	4.115	4.430	3.828	56697	Akap10	chr11	-	NM_0199	P95	protein_cc	1500031L:	A kinase (	Akap10
2937	Specc1	2.415	0.955	2.307	0.117	0.888	0.141	3.655	4.927	4.861	432572	Specc1	chr11	+	NM_0012	P7668	protein_cc	2810012G	sperm ant	Specc1
2938	Adora2b	33.140	0.379	12.551	0.028	0.104	0.094	0.331	5.382	3.981	11541	Adora2b	chr11	+	NM_0074	P8592	protein_cc	A2b| A2B/	adenosine	Adora2b
2939	Zswim7	0.611	1.614	0.987	0.048	0.059	0.906	6.191	5.481	6.172	69747	Zswim7	chr11	-	NM_0271	P5097	protein_cc	2410012H	zinc finge	Zswim7
2940	Ttc19	1.010	1.105	1.116	0.951	0.488	0.459	7.269	7.284	7.427	72795	Ttc19	chr11	+	NM_0297	P22460	protein_cc	2010204O	tetratrico	Ttc19
2941	Ncor1	0.652	0.961	0.627	0.000	0.732	0.067	11.144	10.528	10.471	20185	Ncor1	chr11	-	NM_0012	P19647	protein_cc	5730405N	nuclear re	Ncor1
2942	Pigl	1.057	1.099	1.162	0.862	0.815	0.805	3.870	3.950	4.086	327942	Pigl	chr11	+	NM_0010	P12420	protein_cc	Gm737	phosphati	Pigl
2943	Cenpv	0.528	1.179	0.623	0.005	0.005	0.114	8.440	7.518	7.756	73139	Cenpv	chr11	-	NM_0284	P9659	protein_cc	3110013H	centrome	Cenpv
2944	Ubb	0.486	4.112	1.998	0.191	0.099	0.515	1.101	0.060	2.099	22187	Ubb	chr11	+	NM_0116	P7023	protein_cc	AL033289	ubiquitin E	Ubb
2945	Gm1821	2.664	0.825	2.199	0.006	0.237	0.048	12.148	13.562	13.285	218963	Gm1821	chr11	+	NR_00287	.	misc_RNA	.	predicted	Gm1821
2946	Trpv2	1.363	1.275	1.737	0.334	0.237	0.325	3.047	3.493	3.843	22368	Trpv2	chr11	+	NM_0117	P1771	protein_cc	GRC| OTR	transient r	Trpv2
2947	2410006H	1.347	1.205	1.623	0.024	0.582	0.494	8.404	8.834	9.103	69221	2410006H	chr11	+	NR_03073	.	lncRNA	.	RIKEN cDN	2410006H:
2948	Snord49b	2.142	0.906	1.940	0.051	0.640	0.188	3.649	4.747	4.605	10021742	Snord49b	chr11	+	NR_02852	.	snoRNA	MBII-252|	small nucl	Snord49b
2949	Snord49a	2.358	0.562	1.325	0.110	0.232	0.597	1.126	2.364	1.532	10021745	Snord49a	chr11	+	NR_02855	.	snoRNA	MBII-243	small nucl	Snord49a
2950	Snord65	8.566	0.567	4.854	0.005	0.051	0.021	1.005	4.104	3.284	10021744	Snord65	chr11	+	NR_02854	.	snoRNA	MBII-135|	small nucl	Snord65
2951	Lrrc75a	18.792	0.429	8.065	0.008	0.052	0.095	0.697	4.929	3.708	192976	Lrrc75a	chr11	-	NM_1988	P6234	protein_cc	Fam211a	leucine ric	Lrrc75a
2952	Mmgt2	1.975	0.857	1.692	0.000	0.517	0.400	4.162	5.144	4.921	216829	Mmgt2	chr11	+	NM_1750	P20686	protein_cc	AI852046	membran	Mmgt2
2953	Zfp287	0.273	2.494	0.680	0.183	0.187	0.743	1.934	0.060	1.378	170740	Zfp287	chr11	-	NM_1332	P1422	protein_cc	B230333C	zinc finge	Zfp287
2954	Zfp286	1.000	1.000	1.000	1.000	1.000	1.000	0.000	0.000	0.000	192651	Zfp286	chr11	-	NM_1389	P2749	protein_cc	BC027023	zinc finge	Zfp286
2955	Trim16	0.694	3.177	2.204	0.359	0.000	0.118	2.061	1.534	3.201	94092	Trim16	chr11	+	NM_0531	P573	protein_cc	9130006N	tripartite	Trim16
2956	Fbxw10	1.000	1.000	1.000	1.000	1.000	1.000	0.000	0.000	0.000	213980	Fbxw10	chr11	+	NM_0010	P18648	protein_cc	Fbw10| H	F-box and	Fbxw10
2957	Tvp23b	1.961	0.621	1.217	0.000	0.000	0.353	8.031	9.002	8.314	67510	Tvp23b	chr11	+	NM_0262	P7443	protein_cc	1810036I2	trans-golg	Tvp23b
2958	Cdrt4	1.000	1.000	1.000	1.000	1.000	1.000	0.000	0.000	0.000	66338	Cdrt4	chr11	+	NM_0254	P25552	protein_cc	1700019I2	CMT1A du	Cdrt4
2959	Cdrt4os1	1.000	1.000	1.000	1.000	1.000	1.000	0.000	0.000	0.000	74884	Cdrt4os1	chr11	-	NR_12654	.	lncRNA	4930452L(	CMT1A du	Cdrt4os1
2960	Tekt3	1.000	1.000	1.000	1.000	1.000	1.000	0.000	0.000	0.000	71062	Tekt3	chr11	+	NM_0276	P23472	protein_cc	4933407G	tektin 3	Tekt3
2961	Pmp22	1.979	1.415	2.800	0.088	0.175	0.071	5.020	6.005	6.505	18858	Pmp22	chr11	+	NM_0013	P11672	protein_cc	22kDa| Ga	periphera	Pmp22
2962	Hs3st3b1	0.727	0.819	0.596	0.337	0.599	0.254	8.755	8.296	8.008	54710	Hs3st3b1	chr11	-	NM_0188	P3900	protein_cc	3-OST-3B|	heparan s	Hs3st3b1
2963	Cox10	0.663	1.437	0.953	0.048	0.037	0.788	8.687	8.094	8.617	70383	Cox10	chr11	-	NM_1783	P17987	protein_cc	2410004F(	cytochron	Cox10
2964	9630013K	0.990	1.321	1.308	0.937	0.222	0.479	2.408	2.394	2.796	78883	9630013K	chr11	+	NR_13111	.	lncRNA	.	RIKEN cDN	9630013K:
2965	2810001G	1.290	1.069	1.379	0.067	0.034	0.189	5.184	5.551	5.648	66456	2810001G	chr11	+	NR_03378	.	lncRNA	AW04551	RIKEN cDN	2810001G:
2966	F930015N	0.297	1.325	0.393	0.182	0.208	0.488	1.796	0.043	0.448	654805	F930015N	chr11	+	NR_02844	.	lncRNA	.	RIKEN cDN	F930015N(
2967	Hs3st3a1	0.196	2.723	0.534	0.094	0.149	0.559	3.904	1.553	2.998	15478	Hs3st3a1	chr11	+	NM_1788	P5178	protein_cc	3Ost3a| H	heparan s	Hs3st3a1
2968	Elac2	1.420	0.841	1.194	0.217	0.483	0.310	5.408	5.913	5.663	68626	Elac2	chr11	+	NM_0234	P2882	protein_cc	1110017O	elaC ribon	Elac2
2969	Arhgap44	2.358	1.057	2.491	0.152	0.871	0.009	3.863	5.100	5.179	216831	Arhgap44	chr11	-	NM_1750	P11892	protein_cc	6330543G	Rho GTPa	Arhgap44
2970	Myocd	1.000	1.000	1.000	1.000	1.000	1.000	0.000	0.000	0.000	214384	Myocd	chr11	-	NM_1451	P13613	protein_cc	BSAC2A| !	myocardin	Myocd
2971	Gm12295	1.000	1.000	1.000	1.000	1.000	1.000	0.000	0.000	0.000	327946	Gm12295	chr11	-	NR_04028	.	lncRNA	A730013G	predicted	Gm12295
2972	Map2k4	1.952	0.721	1.406	0.000	0.013	0.187	8.250	9.215	8.742	26398	Map2k4	chr11	-	NM_0091	P6074	protein_cc	JNKK1| M	mitogen-a	Map2k4
2973	Zkscan6	2.716	0.727	1.973	0.016	0.234	0.277	4.910	6.351	5.891	52712	Zkscan6	chr11	+	NM_0013	P23294	protein_cc	1700128E:	zinc finge	Zkscan6
2974	Dnah9	0.552	0.990	0.547	0.182	1.000	0.423	0.871	0.015	0.000	237806	Dnah9	chr11	-	NM_0010	P22660	protein_cc	9030022N	dynein, ax	Dnah9
2975	Shisa6	0.716	0.995	0.712	0.182	1.000	0.423	0.490	0.008	0.000	380702	Shisa6	chr11	-	NM_0010	P15686	protein_cc	ckamp52|	shisa fami	Shisa6
2976	Gm12298	1.000	1.000	1.000	1.000	1.000	1.000	0.000	0.000	0.000	10003866	Gm12298	chr11	-	NR_03353	.	lncRNA	OTTMUSG	predicted	Gm12298
2977	Pirt	1.005	1.439	1.446	0.000	0.182	0.423	0.000	0.008	0.532	193003	Pirt	chr11	+	NM_1786	P11207	protein_cc	A530088H	phosphoir	Pirt
2978	9130409J2	1.000	1.000	1.000	1.000	1.000	1.000	0.000	0.000	0.000	71576	9130409J2	chr11	+	NR_13064	.	lncRNA	.	RIKEN cDN	9130409J2
2979	Tmem220	0.176	1.713	0.302	0.000	0.072	0.010	9.453	6.948	7.724	338369	Tmem220	chr11	+	NM_0012	P15464	protein_cc	.	transmem	Tmem220
2980	Adprm	0.766	1.165	0.892	0.057	0.558	0.746	7.385	7.000	7.221	66358	Adprm	chr11	-	NM_0255	P10402	protein_cc	2310004I2	ADP-ribos	Adprm
2981	Sco1	0.897	0.843	0.757	0.127	0.062	0.188	7.339	7.182	6.937	52892	Sco1	chr11	+	NM_0010	P7082	protein_cc	2610001C	SCO1 cyto	Sco1
2982	Myh3	0.560	1.371	0.768	0.184	0.191	0.687	0.857	0.021	0.477	17883	Myh3	chr11	+	NM_0010	P18010	protein_cc	MyHC-em	myosin, he	Myh3
2983	2310065F	1.000	1.000	1.000	1.000	1.000	1.000	0.000	0.000	0.000	74184	2310065F	chr11	-	NR_03805	.	lncRNA	linc-Myh	RIKEN cDN	2310065F(
2984	Myh2	0.134	0.948	0.127	0.007	1.000	0.088	2.973	0.077	0.000	17882	Myh2	chr11	+	NM_0010	P17607	protein_cc	MHC2A| M	myosin, he	Myh2
2985	Myh1	0.509	1.780	0.906	0.184	0.186	0.897	1.002	0.028	0.859	17879	Myh1	chr11	+	NM_0306	P289	protein_cc	AI325106|	myosin, he	Myh1
2986	Myh4	1.000	1.000	1.000	1.000	1.000	1.000	0.000	0.000	0.000	17884	Myh4	chr11	+	NM_0108	P19591	protein_cc	AI506973|	myosin, he	Myh4
2987	Myh8	1.000	1.000	1.000	1.000	1.000	1.000	0.000	0.000	0.000	17885	Myh8	chr11	+	NM_1773	P11866	protein_cc	4832426G	myosin, he	Myh8
2988	Myh13	1.000	1.000	1.000	1.000	1.000	1.000	0.000	0.000	0.000	544791	Myh13	chr11	+	NM_0010	P23306	protein_cc	MyHC-eo	myosin, he	Myh13
2989	Gas7	1.084	0.999	1.083	0.113	0.997	0.786	6.082	6.199	6.198	14457	Gas7	chr11	+	NM_0080	P26644	protein_cc	AW12476	growth ar	Gas7
2990	Rcvrn	1.000	1.000	1.000	1.000	1.000	1.000	0.000	0.000	0.000	19674	Rcvrn	chr11	+	NM_0090	P26015	protein_cc	CAR| S-mc	recoverin	Rcvrn

2991	Glp2r	0.762	3.395	2.588	0.209	0.073	0.335	0.434	0.043	1.806	93896	Glp2r	chr11	-	NM_1756	P3481	protein_cc	9530092J(	glucagon-	Glp2r
2992	Dhrs7c	1.000	1.000	1.000	1.000	1.000	1.000	0.000	0.000	0.000	68460	Dhrs7c	chr11	+	NM_0010	P14968	protein_cc	1110001P	dehydroge	Dhrs7c
2993	Usp43	0.727	1.692	1.230	0.318	0.399	0.799	1.168	0.708	1.466	216835	Usp43	chr11	-	NM_0012	P21614	protein_cc	.	ubiquitin s	Usp43
2994	Cfap52	1.000	1.000	1.000	1.000	1.000	1.000	0.000	0.000	0.000	71860	Cfap52	chr11	-	NM_0279	P20714	protein_cc	1700019F(	cilia and fl	Cfap52
2995	Stx8	0.901	1.332	1.200	0.443	0.104	0.492	7.385	7.234	7.648	55943	Stx8	chr11	+	NM_0187	P12975	protein_cc	0610007H	syntaxin 8	Stx8
2996	Ntn1	0.155	1.508	0.234	0.000	0.349	0.002	5.101	2.415	3.007	18208	Ntn1	chr11	-	NM_0087	P14289	protein_cc	AI561871 |	netrin 1	Ntn1
2997	Pik3r5	17.850	0.454	8.096	0.019	0.093	0.006	1.708	5.866	4.725	320207	Pik3r5	chr11	+	NM_1773	P621	protein_cc	AV230647	phosphoir	Pik3r5
2998	Pik3r6	2.955	0.490	1.448	0.057	0.072	0.587	2.258	3.822	2.793	104709	Pik3r6	chr11	+	NM_0010	P9970	protein_cc	BB220380	phosphoir	Pik3r6
2999	Mfsd6l	2.149	0.465	1.000	0.306	1.000	0.423	0.000	1.104	0.000	215723	Mfsd6l	chr11	+	NM_1460	P8657	protein_cc	.	major faci	Mfsd6l
3000	Ccdc42	1.000	1.000	1.000	1.000	1.000	1.000	0.000	0.000	0.000	276920	Ccdc42	chr11	+	NM_1777	P14528	protein_cc	A530001H	coiled-coil	Ccdc42
3001	Myh10	0.308	1.453	0.447	0.003	0.221	0.078	7.214	5.513	6.052	77579	Myh10	chr11	+	NM_1752	P13771	protein_cc	5730504C	myosin, he	Myh10
3002	Ndel1	1.103	0.831	0.916	0.381	0.165	0.337	8.301	8.442	8.175	83431	Ndel1	chr11	-	NM_0236	P16230	protein_cc	2600006O	nudE neur	Ndel1
3003	Rnf222	0.506	0.654	0.331	0.435	0.574	0.506	2.464	1.482	0.869	320040	Rnf222	chr11	+	NM_1770	P10358	protein_cc	9930039A	ring finger	Rnf222
3004	Rpl26	1.307	1.157	1.512	0.045	0.240	0.100	11.713	12.100	12.310	19941	Rpl26	chr11	+	NM_0090	P11851	protein_cc	SIG-20	ribosomal	Rpl26
3005	Gm15772	1.000	1.000	1.000	1.000	1.000	1.000	0.000	0.000	0.000	10003472	Gm15772	chr11	+	NR_00337	.	misc_RNA	ENSMUSG	predicted	Gm15772
3006	Odf4	1.000	1.000	1.000	1.000	1.000	1.000	0.000	0.000	0.000	252868	Odf4	chr11	-	NM_1457	P7332	protein_cc	Oppo1	outer den	Odf4
3007	Arhgef15	0.598	1.298	0.776	0.039	0.381	0.431	5.294	4.552	4.929	442801	Arhgef15	chr11	-	NM_1775	P26695	protein_cc	D130071N	Rho guani	Arhgef15
3008	Slc25a35	0.341	1.016	0.347	0.027	0.959	0.147	4.380	2.829	2.852	71998	Slc25a35	chr11	+	NM_0280	P18382	protein_cc	1810012H	solute car	Slc25a35
3009	Rangrf	0.810	1.071	0.867	0.370	0.816	0.607	4.200	3.895	3.994	57785	Rangrf	chr11	-	NM_0012	P21711	protein_cc	2400006H	RAN guani	Rangrf
3010	Pfas	1.189	1.024	1.217	0.415	0.899	0.029	5.649	5.899	5.933	237823	Pfas	chr11	-	NM_0011	P11954	protein_cc	4432409B	phosphori	Pfas
3011	Ctc1	0.644	1.110	0.715	0.237	0.550	0.587	4.476	3.841	3.992	68964	Ctc1	chr11	+	NM_0012	P3701	protein_cc	1500010J(	CTS telom	Ctc1
3012	Aurkb	0.242	3.259	0.789	0.117	0.185	0.824	2.116	0.069	1.774	20877	Aurkb	chr11	+	NM_0114	P8130	protein_cc	Aik2| AIM	aurora kin	Aurkb
3013	2310047M	1.660	0.978	1.624	0.005	0.761	0.092	7.607	8.339	8.307	.	2310047M	chr11	+	NM_0280	P10152	.			2310047M
3014	Tmem107	0.780	1.575	1.230	0.674	0.474	0.837	4.079	3.721	4.377	66910	Tmem107	chr11	+	NM_0258	P3305	protein_cc	1110004B	transmem	Tmem107
3015	Vamp2	1.268	1.413	1.791	0.002	0.184	0.277	6.466	6.808	7.307	22318	Vamp2	chr11	+	NM_0094	P15941	protein_cc	Syb-2| Syb	vesicle-ass	Vamp2
3016	Per1	1.470	0.939	1.381	0.002	0.083	0.071	7.161	7.717	7.626	18626	Per1	chr11	+	NM_0110	P25666	protein_cc	m-rigui| m	period circ	Per1
3017	Hes7	1.603	1.647	2.641	0.292	0.212	0.178	0.001	0.681	1.402	84653	Hes7	chr11	+	NM_0330	P10424	protein_cc	bHLHb37	hairy and e	Hes7
3018	Aloxe3	0.726	1.425	1.034	0.187	0.186	0.943	0.476	0.015	0.525	23801	Aloxe3	chr11	+	NM_0117	P19417	protein_cc	e-LOX-3| e	arachidon	Aloxe3
3019	Alox12b	1.000	1.000	1.000	1.000	1.000	1.000	0.000	0.000	0.000	11686	Alox12b	chr11	+	NM_0096	P324	protein_cc	12R-LOX|	arachidon	Alox12b
3020	Alox8	1.014	2.240	2.272	0.000	0.182	0.423	0.000	0.021	1.185	11688	Alox8	chr11	-	NM_0096	P13144	protein_cc	15-LOX-2|	arachidon	Alox8
3021	Gucy2e	1.000	1.000	1.000	1.000	1.000	1.000	0.000	0.000	0.000	14919	Gucy2e	chr11	-	NM_0081	P9491	protein_cc	GC-E| GC1	guanylate	Gucy2e
3022	Cntrob	0.988	0.611	0.603	0.870	0.000	0.084	4.703	4.685	3.974	216846	Cntrob	chr11	-	NM_1725	P23958	protein_cc	9830165K	centrobin,	Cntrob
3023	Trappc1	0.730	1.290	0.942	0.014	0.095	0.762	9.246	8.792	9.160	245828	Trappc1	chr11	+	NM_0010	P5868	protein_cc	BET5| D11	trafficking	Trappc1
3024	Kcnab3	0.609	1.317	0.802	0.297	0.511	0.582	2.945	2.229	2.626	16499	Kcnab3	chr11	+	NM_0105	P5393	protein_cc	C330022D	potassium	Kcnab3
3025	Chd3os	1.970	0.234	0.462	0.231	0.079	0.348	2.957	3.936	1.843	80515	Chd3os	chr11	+	NR_02782	.	lncRNA	2310043C	chromodo	Chd3os
3026	Chd3	0.336	1.480	0.498	0.000	0.073	0.044	9.944	8.372	8.937	216848	Chd3	chr11	-	NM_1460	P5668	protein_cc	2600010P	chromodo	Chd3
3027	Cyb5d1	1.604	0.862	1.383	0.462	0.802	0.636	0.432	1.113	0.899	327951	Cyb5d1	chr11	-	NM_0010	P19279	protein_cc	Gm740	cytochrom	Cyb5d1
3028	Naa38	0.719	1.173	0.844	0.005	0.478	0.602	9.238	8.762	8.993	78304	Naa38	chr11	+	NM_0300	P19179	protein_cc	1500034E	N(alpha)-a	Naa38
3029	Tmem88	0.685	1.254	0.858	0.003	0.324	0.640	9.030	8.484	8.810	67020	Tmem88	chr11	-	NM_0259	P9474	protein_cc	2600017H	transmem	Tmem88
3030	Kdm6b	2.424	0.474	1.149	0.022	0.031	0.251	5.971	7.249	6.172	216850	Kdm6b	chr11	-	NM_0010	P15910	protein_cc	1700064E	KDM1 lysi	Kdm6b
3031	Dnah2	10.743	0.991	10.647	0.000	0.983	0.303	0.340	3.766	3.753	327954	Dnah2	chr11	-	NM_0010	P10241	protein_cc	2900022L	dynein, ax	Dnah2
3032	Efnb3	1.005	1.405	1.412	0.000	0.182	0.423	0.000	0.008	0.498	13643	Efnb3	chr11	-	NM_0079	P4588	protein_cc	EFL-6| ELF	ephrin B3	Efnb3
3033	Wrap53	0.774	1.409	1.090	0.380	0.055	0.849	5.960	5.590	6.084	216853	Wrap53	chr11	-	NM_1448	P22196	protein_cc	BC021790	WD repeat	Wrap53
3034	Trp53	1.354	0.613	0.830	0.310	0.140	0.495	6.166	6.603	5.898	22059	Trp53	chr11	+	NM_0011	P22324	protein_cc	bbl| bfy| b	transform	Trp53
3035	Atp1b2	0.143	0.951	0.136	0.003	1.000	0.062	2.880	0.073	0.000	11932	Atp1b2	chr11	-	NM_0134	P18817	protein_cc	Amog| Atp	ATPase, N	Atp1b2
3036	Shbg	1.010	1.825	1.844	0.000	0.182	0.423	0.000	0.015	0.883	20415	Shbg	chr11	-	NM_0113	P19990	protein_cc	ABP	sex hormo	Shbg
3037	Sat2	0.400	1.408	0.564	0.005	0.167	0.121	7.148	5.827	6.321	69215	Sat2	chr11	+	NM_0269	P24565	protein_cc	2610016A	spermidin	Sat2
3038	Fxr2	1.065	0.848	0.903	0.824	0.570	0.786	8.681	8.773	8.535	23879	Fxr2	chr11	+	NM_0118	P16864	protein_cc	Fxr2h	fragile X m	Fxr2
3039	Sox15	0.636	1.602	1.019	0.002	0.002	0.805	9.737	9.085	9.765	20670	Sox15	chr11	+	NM_0092	P15340	protein_cc	.	SRY (sex d	Sox15
3040	Mpdu1	0.628	1.573	0.988	0.008	0.003	0.907	9.973	9.302	9.956	24070	Mpdu1	chr11	-	NM_0119	P11076	protein_cc	LEC35| SL	mannose-	Mpdu1
3041	Cd68	1.833	0.766	1.404	0.092	0.304	0.140	6.474	7.348	6.963	12514	Cd68	chr11	-	NR_11099	.	protein_cc	gp110| La	CD68 anti	Cd68
3042	Eif4a1	2.360	0.963	2.273	0.001	0.768	0.069	9.766	11.005	10.951	13681	Eif4a1	chr11	-	NM_0011	P24007	protein_cc	BM-010| I	eukaryotic	Eif4a1
3043	Senp3	1.481	1.551	2.296	0.216	0.255	0.287	4.769	5.335	5.968	80886	Senp3	chr11	-	NM_0307	P5070	protein_cc	AA408656	SUMO/sen	Senp3
3044	Tnfsfm13	0.602	1.132	0.682	0.276	0.674	0.566	4.950	4.219	4.398	619441	Tnfsfm13	chr11	-	NM_0010	P1055	protein_cc	Tnfsf12-Tr	tumor nec	Tnfsfm13
3045	Tnfsf13	0.615	1.086	0.668	0.285	0.751	0.545	4.873	4.171	4.290	69583	Tnfsf13	chr11	-	NM_0011	P17685	protein_cc	2310026N	tumor nec	Tnfsf13
3046	Tnfsf12	0.852	1.504	1.282	0.506	0.081	0.170	6.518	6.287	6.876	21944	Tnfsf12	chr11	-	NM_0116	P4947	protein_cc	Apo3l| Dr	tumor nec	Tnfsf12
3047	Polr2a	2.009	0.597	1.199	0.000	0.026	0.646	7.961	8.967	8.222	20020	Polr2a	chr11	-	NM_0012	P10560	protein_cc	220kDa| F	polymeras	Polr2a
3048	Slc35g3	1.000	1.000	1.000	1.000	1.000	1.000	0.000	0.000	0.000	56293	Slc35g3	chr11	-	NM_0198	P13644	protein_cc	Amac1	solute car	Slc35g3
3049	Zbtb4	1.188	0.820	0.974	0.355	0.251	0.936	7.450	7.699	7.412	75580	Zbtb4	chr11	+	NM_0293	P5001	protein_cc	2310026P	zinc finger	Zbtb4
3050	Chrnb1	0.434	3.046	1.322	0.208	0.033	0.690	1.934	0.730	2.336	11443	Chrnb1	chr11	-	NM_0096	P15427	protein_cc	Achr-2| Ac	cholinergi	Chrnb1
3051	Fgf11	0.212	0.604	0.128	0.013	1.000	0.090	2.969	0.728	0.000	14166	Fgf11	chr11	-	NM_0012	P19520	protein_cc	Fgf-11| Fh	fibroblast	Fgf11
3052	Tmem102	0.644	1.382	0.890	0.018	0.243	0.733	3.887	3.252	3.719	380705	Tmem102	chr11	-	NM_0010	P24611	protein_cc	4632403M	transmem	Tmem102
3053	G630025P	0.723	1.006	0.728	0.153	0.975	0.206	3.622	3.154	3.163	74341	G630025P	chr11	+	NR_02791	.	lncRNA	4632403M	RIKEN cDN	G630025P
3054	4933402P	1.000	1.000	1.000	1.000	1.000	1.000	0.000	0.000	0.000	108803	4933402P	chr11	-	NM_1753	P8795	protein_cc	.	RIKEN cDN	4933402P
3055	Spem1	1.000	1.000	1.000	1.000	1.000	1.000	0.000	0.000	0.000	74288	Spem1	chr11	-	NM_0288	P23621	protein_cc	1700095G	sperm ma	Spem1
3056	Nlgn2	1.587	0.830	1.317	0.093	0.496	0.707	3.393	4.059	3.790	216856	Nlgn2	chr11	-	NM_1988	P25120	protein_cc	NL2	neuroligin	Nlgn2
3057	Tmem256	0.759	1.092	0.828	0.015	0.512	0.422	11.645	11.246	11.372	69186	Tmem256	chr11	+	NM_0269	P11006	protein_cc	1810027O	transmem	Tmem256
3058	Plscr3	0.830	1.505	1.249	0.642	0.287	0.362	5.955	5.686	6.276	70310	Plscr3	chr11	+	NM_0011	P19497	protein_cc	2210403O	phospholi	Plscr3
3059	Tnk1	0.830	1.460	1.212	0.642	0.322	0.392	5.955	5.686	6.232	83813	Tnk1	chr11	-	NM_0318	P6217	protein_cc	Kos1| Tnk	tyrosine k	Tnk1
3060	Tmem95	1.000	1.000	1.000	1.000	1.000	1.000	0.000	0.000	0.000	10050336	Tmem95	chr11	-	NM_0011	P25273	protein_cc	432576	transmem	Tmem95
3061	Kctd11	1.785	0.800	1.428	0.000	0.007	0.024	3.668	4.503	4.181	216858	Kctd11	chr11	-	NM_1531	P12194	protein_cc	AF465352	potassium	Kctd11
3062	Acap1	0.226	1.033	0.233	0.020	0.921	0.137	2.878	0.731	0.777	216859	Acap1	chr11	-	NM_1537	P19821	protein_cc	Centb1	ArfGAP wi	Acap1
3063	2810408A	0.266	0.973	0.259	0.098	1.000	0.315	1.950	0.039	0.000	70419	2810408A	chr11	-	NM_0274	P12918	protein_cc	1700019L	RIKEN cDN	2810408A
3064	Neurl4	0.568	1.407	0.799	0.059	0.269	0.252	6.078	5.262	5.755	216860	Neurl4	chr11	+	NM_0012	P25025	protein_cc	.	neuralized	Neurl4
3065	Gps2	1.137	0.794	0.902	0.531	0.303	0.389	8.273	8.457	8.124	56310	Gps2	chr11	+	NM_0197	P7035	protein_cc	AI505953	G protein	Gps2
3066	Eif5a	1.293	1.172	1.515	0.181	0.302	0.033	10.869	11.239	11.468	276770	Eif5a	chr11	-	NM_0011	P11586	protein_cc	AA410058	eukaryotic	Eif5a
3067	Ybx2	1.000	1.000	1.000	1.000	1.000	1.000	0.000	0.000	0.000	53422	Ybx2	chr11	+	NM_0168	P2279	protein_cc	Msy2	Y box prot	Ybx2
3068	Slc2a4	0.373	0.981	0.366	0.018	1.000	0.140	1.450	0.027	0.000	20528	Slc2a4	chr11	-	NM_0092	P8709	protein_cc	Glut-4| Gl	solute car	Slc2a4
3069	Cldn7	1.065	1.595	1.698	0.908	0.211	0.491	1.700	1.791	2.464	53624	Cldn7	chr11	+	NM_0011	P17989	protein_cc	.	claudin 7	Cldn7
3070	Elp5	1.456	1.206	1.756	0.003	0.021	0.010	7.802	8.344	8.614	54351	Elp5	chr11	-	NM_0012	P8290	protein_cc	Derp6| Ra	elongator	Elp5
3071	Ctdnep1	1.911	0.979	1.870	0.048	0.909	0.031	7.828	8.762	8.731	67181	Ctdnep1	chr11	+	NM_0260	P11432	protein_cc	2610507E	CTD nucle	Ctdnep1
3072	Gabarap	1.148	0.966	1.109	0.131	0.636	0.394	10.225	10.424	10.374	56486	Gabarap	chr11	+	NM_0197	P3868	protein_cc	.	gamma-am	Gabarap
3073	Phf23	2.115	0.882	1.865	0.000	0.133	0.041	8.123	9.204	9.023	78246	Phf23	chr11	-	NM_0012	P24022	protein_cc	JUNE-1	PHD finge	Phf23
3074	Dvl2	0.916	0.863	0.791	0.602	0.491	0.247	5.716	5.591	5.377	13543	Dvl2	chr11	+	NM_0078	P6922	protein_cc	.	dishevelle	Dvl2
3075	Acadvl	0.541	1.239	0.671	0.004	0.062	0.081	11.344	10.459	10.769	11370	Acadvl	chr11	-	NM_0173	P17381	protein_cc	vlcad	acyl-Coen	Acadvl
3076	Dlg4	1.185	0.923	1.094	0.666	0.830	0.872	2.565	2.810	2.694	13385	Dlg4	chr11	+	NM_0078	P831	protein_cc	Dlgh4| PSI	discs, larg	Dlg4
3077	Asgr1	0.264	1.660	0.438	0.003	0.029	0.072	13.892	11.968	12.700	11889	Asgr1	chr11	+	NM_0012	P2872	protein_cc	ASGPR1|	asialoglyc	Asgr1
3078	Asgr2	0.268	0.865	0.232	0.000	0.422	0.012	10.774	8.875	8.666	11890	Asgr2	chr11	+	NM_0074	P9510	protein_cc	ASGPR2|	asialoglyc	Asgr2
3079	Mgl2	1.260	1.050	1.324	0.702	0.936	0.744	0.802	1.136	1.206	216864	Mgl2	chr11	+	NM_1451	P10819	protein_cc	CD301b	macropha	Mgl2
3080	Clec10a	0.127	2.620	0.333	0.014	0.066	0.189	4.183	1.207	2.596	17312	Clec10a	chr11	+	NM_0107	P10414	protein_cc	CD301a| M	C-type lec	Clec10a
3081	Slc16a11	0.388	0.943	0.366	0.001	0.721	0.017	6.590	5.224	5.139	216867	Slc16a11	chr11	-	NM_1530	P9338	protein_cc	AB041591	solute car	Slc16a11

3082	Slc16a13	1.484	1.622	2.407	0.592	0.536	0.562	1.813	2.382	3.080	69309	Slc16a13	chr11	-	NM_1723	P209	protein_cc	1700007D	solute car	Slc16a13
3083	Bcl6b	5.780	0.451	2.609	0.000	0.000	0.058	4.472	7.003	5.855	12029	Bcl6b	chr11	-	NM_0075	P5217	protein_cc	Bazf	B cell CLL/	Bcl6b
3084	0610010K	1.075	1.079	1.160	0.121	0.053	0.177	8.147	8.251	8.361	104457	0610010K	chr11	-	NM_1457	P18826	protein_cc	1110020A	RIKEN cDN	0610010K1
3085	Rnasek	0.759	1.214	0.922	0.016	0.007	0.551	9.636	9.238	9.518	52898	Rnasek	chr11	-	NM_1737	P17768	protein_cc	2310033H	ribonuclea	Rnasek
3086	Alox12	11.877	0.269	3.190	0.001	0.006	0.345	2.692	6.262	4.365	11684	Alox12	chr11	-	NM_0074	P12376	protein_cc	9930022G	arachidon	Alox12
3087	Alox12e	1.589	1.141	1.813	0.298	0.765	0.423	0.000	0.669	0.859	11685	Alox12e	chr11	-	NM_1456	P23646	protein_cc	8-LOX| Alc	arachidon	Alox12e
3088	Alox15	2.560	0.759	1.944	0.113	0.588	0.518	0.403	1.759	1.361	11687	Alox15	chr11	-	NM_0096	P3104	protein_cc	12-LO| 12	arachidon	Alox15
3089	Pelp1	1.360	0.614	0.835	0.095	0.035	0.123	7.177	7.621	6.918	75273	Pelp1	chr11	-	NM_0292	P26080	protein_cc	4930563C	proline, gl	Pelp1
3090	Arrb2	0.234	3.544	0.829	0.010	0.006	0.583	5.454	3.359	5.184	216869	Arrb2	chr11	+	NM_1454	P10641	protein_cc	AI326910|	arrestin, b	Arrb2
3091	Med11	1.700	1.232	2.095	0.056	0.246	0.037	6.484	7.250	7.551	66172	Med11	chr11	+	NM_0253	P21784	protein_cc	1110030J	mediator	Med11
3092	Cxcl16	3.094	0.454	1.404	0.000	0.000	0.097	6.805	8.434	7.295	66102	Cxcl16	chr11	-	NM_0231	P11316	protein_cc	0910001K	chemokin	Cxcl16
3093	Zmynd15	1.058	0.663	0.701	0.848	0.092	0.609	3.872	3.953	3.359	574428	Zmynd15	chr11	+	NM_0010	P10970	protein_cc	.	zinc finger	Zmynd15
3094	Tm4sf5	0.743	2.201	1.635	0.196	0.007	0.246	0.456	0.027	1.165	75604	Tm4sf5	chr11	+	NM_0293	P25573	protein_cc	2010003F	transmem	Tm4sf5
3095	Vmo1	0.324	4.787	1.552	0.002	0.039	0.489	5.696	4.071	6.330	327956	Vmo1	chr11	-	NM_0010	P6230	protein_cc	Gm741	vitelline m	Vmo1
3096	Gltpd2	0.344	1.685	0.580	0.000	0.069	0.124	9.135	7.598	8.350	216871	Gltpd2	chr11	+	NM_1460	P13711	protein_cc	C730027E	glycolipid	Gltpd2
3097	Psmb6	1.123	1.268	1.424	0.001	0.132	0.245	10.419	10.586	10.928	19175	Psmb6	chr11	+	NM_0089	P20511	protein_cc	Lmp19| M	proteason	Psmb6
3098	Pld2	1.547	1.090	1.685	0.499	0.868	0.295	3.789	4.418	4.542	18806	Pld2	chr11	+	NM_0088	P3815	protein_cc	PLD1C	phospholi	Pld2
3099	Mink1	0.396	0.983	0.390	0.039	0.961	0.197	6.939	5.604	5.579	50932	Mink1	chr11	+	NM_0010	P12609	protein_cc	B55| Map	misshaper	Mink1
3100	Chrne	0.304	0.977	0.297	0.080	1.000	0.286	1.752	0.034	0.000	11448	Chrne	chr11	-	NM_0096	P15144	protein_cc	AChrepsilo	cholinergi	Chrne
3101	4930544D	1.000	1.000	1.000	1.000	1.000	1.000	0.000	0.000	0.000	668433	4930544D	chr11	+	NM_0011	P22418	protein_cc	.	RIKEN cDN	4930544D
3102	Gp1ba	0.647	0.894	0.579	0.052	0.593	0.044	2.138	1.511	1.349	14723	Gp1ba	chr11	+	NM_0103	P17307	protein_cc	GPIba| GF	glycoprote	Gp1ba
3103	Slc25a11	0.313	1.476	0.461	0.000	0.099	0.065	9.840	8.163	8.725	67863	Slc25a11	chr11	-	NM_0242	P6220	protein_cc	2310022P	solute car	Slc25a11
3104	Rnf167	0.584	0.497	0.290	0.000	0.000	0.002	7.989	7.212	6.205	70510	Rnf167	chr11	+	NM_0274	P26643	protein_cc	0610010G	ring finger	Rnf167
3105	Pfn1	1.038	1.347	1.397	0.857	0.016	0.376	8.370	8.423	8.852	18643	Pfn1	chr11	-	NM_0110	P17353	protein_cc	Pfn	profilin 1	Pfn1
3106	Eno3	0.581	2.037	1.183	0.069	0.025	0.606	3.732	2.948	3.974	13808	Eno3	chr11	+	NM_0011	P22359	protein_cc	Eno-3| MS	enolase 3,	Eno3
3107	Spag7	0.416	1.360	0.567	0.002	0.108	0.102	9.607	8.344	8.788	216873	Spag7	chr11	-	NM_0110	P13017	protein_cc	5730443G	sperm ass	Spag7
3108	Camta2	0.921	1.287	1.185	0.337	0.231	0.609	6.247	6.127	6.491	216874	Camta2	chr11	-	NM_1781	P16108	protein_cc	Kiaa0909-l	calmoduli	Camta2
3109	Inca1	0.103	3.689	0.378	0.000	0.004	0.035	8.201	4.916	6.799	103844	Inca1	chr11	-	NM_0012	P14681	protein_cc	AI842396	inhibitor o	Inca1
3110	Kif1c	0.209	1.781	0.372	0.003	0.115	0.102	10.697	8.438	9.271	16562	Kif1c	chr11	+	NM_1531	P15498	protein_cc	B430105J	kinesin fa	Kif1c
3111	Zfp3	1.345	1.159	1.559	0.344	0.610	0.260	3.739	4.167	4.380	193043	Zfp3	chr11	+	NM_1775	P11763	protein_cc	AI854635|	zinc finger	Zfp3
3112	Scimp	4.068	0.214	0.870	0.198	0.182	0.786	2.608	4.632	2.407	327957	Scimp	chr11	-	NM_0010	P24128	protein_cc	A430084P	SLP adapt	Scimp
3113	Rabep1	0.556	1.011	0.562	0.000	0.897	0.034	9.517	8.670	8.686	54189	Rabep1	chr11	+	NM_0012	P802	protein_cc	Rab5ep| r	rabaptin,	Rabep1
3114	Nup88	0.856	1.219	1.043	0.302	0.202	0.257	8.309	8.084	8.369	19069	Nup88	chr11	-	NM_0010	P5447	protein_cc	Nup84| Pr	nucleopor	Nup88
3115	Rpain	0.591	1.241	0.734	0.063	0.129	0.374	10.083	9.324	9.636	69723	Rpain	chr11	+	NM_0012	P10182	protein_cc	2400006N	RPA intera	Rpain
3116	C1qbp	0.589	1.342	0.791	0.011	0.005	0.315	10.361	9.597	10.022	12261	C1qbp	chr11	-	NM_0075	P24352	protein_cc	AA407365	compleme	C1qbp
3117	Dhx33	1.071	1.246	1.335	0.567	0.170	0.390	6.813	6.913	7.230	216877	Dhx33	chr11	-	NM_1783	P15150	protein_cc	3110057P	DEAH (Asp	Dhx33
3118	Derl2	0.786	0.987	0.776	0.126	0.924	0.216	9.863	9.517	9.497	116891	Derl2	chr11	-	NM_0012	P5150	protein_cc	CGI-101| I	Der1-like	Derl2
3119	Mis12	1.707	0.813	1.388	0.115	0.454	0.268	5.804	6.575	6.277	67139	Mis12	chr11	+	NM_0259	P13076	protein_cc	2510025F	MIS12 kin	Mis12
3120	6330403K	1.603	1.658	2.658	0.292	0.404	0.423	0.001	0.681	1.411	103712	6330403K	chr11	-	NM_1340	P739	protein_cc	AI415455|	RIKEN cDN	6330403K
3121	Nlrp1a	1.829	0.298	0.545	0.200	1.000	0.040	0.875	1.747	0.000	195046	Nlrp1a	chr11	-	NM_0010	P6303	protein_cc	CARD7| D	NLR family	Nlrp1a
3122	Nlrp1b	0.332	0.978	0.324	0.181	1.000	0.423	1.625	0.032	0.000	637515	Nlrp1b	chr11	-	NM_0010	P22085	protein_cc	ENSMUSG	NLR family	Nlrp1b
3123	Nlrp1c-ps	1.005	1.439	1.446	0.000	0.182	0.423	0.000	0.008	0.532	627984	Nlrp1c-ps	chr11	-	NR_02785	.	misc_RNA	Nalp1c| N	NLR family	Nlrp1c-ps
3124	Wscd1	0.266	1.316	0.350	0.182	0.212	0.477	1.956	0.047	0.443	216881	Wscd1	chr11	+	NM_1776	P3231	protein_cc	mKIAA052	WSC dom	Wscd1
3125	4930401O	1.000	1.000	1.000	1.000	1.000	1.000	0.000	0.000	0.000	73816	4930401O	chr11	-	NR_04594	.	lncRNA	.	RIKEN cDN	4930401O
3126	Aipl1	1.000	1.000	1.000	1.000	1.000	1.000	0.000	0.000	0.000	114230	Aipl1	chr11	-	NM_0532	P17875	protein_cc	A930007I	aryl hydro	Aipl1
3127	Fam64a	1.015	2.253	2.285	0.000	0.015	0.125	0.000	0.021	1.193	109212	Fam64a	chr11	+	NM_1445	P10532	protein_cc	2610008F	family wit	Fam64a
3128	Pitpnm3	1.000	1.000	1.000	1.000	1.000	1.000	0.000	0.000	0.000	327958	Pitpnm3	chr11	-	NM_0010	P7574	protein_cc	A330068P	PITPNM fa	Pitpnm3
3129	4933427D	1.862	0.692	1.289	0.005	0.006	0.443	4.495	5.392	4.861	74477	4933427D	chr11	-	NM_0289	P1022	protein_cc	C85113| K	RIKEN cDN	4933427D
3130	Txndc17	0.654	1.515	0.991	0.001	0.003	0.915	9.811	9.198	9.798	52700	Txndc17	chr11	+	NM_0265	P15828	protein_cc	48314430	thioredoxi	Txndc17
3131	Med31	2.119	1.159	2.455	0.023	0.582	0.199	5.810	6.894	7.106	67279	Med31	chr11	-	NM_0260	P19914	protein_cc	3110004H	mediator	Med31
3132	4930563E	4.885	0.397	1.940	0.116	0.204	0.061	0.375	2.663	1.331	75304	4930563E	chr11	+	NM_0011	P20033	protein_cc	.	RIKEN cDN	4930563E
3133	Slc13a5	38.326	1.367	52.387	0.005	0.208	0.043	2.592	7.852	8.303	237831	Slc13a5	chr11	-	NM_0010	P12163	protein_cc	Indy| mIN	solute car	Slc13a5
3134	1700051A	2.829	1.670	4.725	0.023	0.041	0.026	0.001	1.501	2.241	73366	1700051A	chr11	-	NR_04592	.	lncRNA	.	RIKEN cDN	1700051A
3135	Xaf1	9.920	0.584	5.792	0.002	0.133	0.257	5.208	8.518	7.742	327959	Xaf1	chr11	+	NM_0012	P3602	protein_cc	Fbxo39	XIAP asso	Xaf1
3136	Fbxo39	1.000	1.000	1.000	1.000	1.000	1.000	0.000	0.000	0.000	628100	Fbxo39	chr11	+	NM_0010	P12817	protein_cc	1700010H	F-box prot	Fbxo39
3137	Tekt1	1.000	1.000	1.000	1.000	1.000	1.000	0.000	0.000	0.000	21689	Tekt1	chr11	-	NM_0012	P10798	protein_cc	MT14	tektin 1	Tekt1
3138	Smtnl2	4.667	0.170	0.794	0.005	0.001	0.840	2.962	5.184	2.628	276829	Smtnl2	chr11	-	NM_1777	P10079	protein_cc	D130058I	smootheli	Smtnl2
3139	Ggt6	3.463	0.558	1.931	0.004	0.037	0.185	7.194	8.986	8.143	71522	Ggt6	chr11	+	NM_0278	P22959	protein_cc	9030405D	gamma-gl	Ggt6
3140	Mybbp1a	2.054	0.970	1.993	0.001	0.596	0.006	8.219	9.257	9.214	18432	Mybbp1a	chr11	+	NM_0167	P3120	protein_cc	AL024407	MYB bindi	Mybbp1a
3141	Spns2	0.351	1.696	0.595	0.007	0.003	0.175	7.511	6.000	6.762	216892	Spns2	chr11	-	NM_0012	P17884	protein_cc	.	spinster h	Spns2
3142	Spns3	1.010	1.825	1.844	0.000	0.182	0.423	0.000	0.015	0.883	77577	Spns3	chr11	-	NM_0299	P5228	protein_cc	9830002I1	spinster h	Spns3
3143	Ube2g1	0.906	1.089	0.987	0.013	0.519	0.951	8.563	8.421	8.544	67128	Ube2g1	chr11	+	NM_0259	P4382	protein_cc	2700059C	ubiquitin-	Ube2g1
3144	Ankfy1	1.679	0.719	1.207	0.005	0.015	0.382	7.604	8.352	7.875	11736	Ankfy1	chr11	+	NM_0096	P10242	protein_cc	Ankhzn| n	ankyrin re	Ankfy1
3145	Cyb5d2	0.785	0.603	0.473	0.060	0.030	0.065	6.845	6.496	5.765	192986	Cyb5d2	chr11	-	NM_0010	P19998	protein_cc	9330151E	cytochrom	Cyb5d2
3146	Zzef1	0.701	0.790	0.554	0.002	0.053	0.023	7.859	7.346	7.006	195018	Zzef1	chr11	+	NM_0010	P17595	protein_cc	8430405D	zinc finger	Zzef1
3147	Atp2a3	0.211	1.735	0.365	0.046	0.283	0.324	3.794	1.546	2.341	53313	Atp2a3	chr11	+	NM_0011	P4726	protein_cc	Serca3| SE	ATPase, C	Atp2a3
3148	P2rx1	2.700	0.370	1.000	0.305	1.000	0.423	0.001	1.433	0.000	18436	P2rx1	chr11	+	NM_0087	P3400	protein_cc	AI323649|	purinergic	P2rx1
3149	Camkk1	5.815	0.242	1.405	0.189	0.226	0.734	1.013	3.552	1.504	55984	Camkk1	chr11	+	NM_0188	P15546	protein_cc	AI846603|	calcium/ca	Camkk1
3150	1200014J	1.117	0.936	1.045	0.473	0.729	0.879	7.855	8.014	7.919		1200014J1	chr11	+	NM_0258	P14606				1200014J1
3151	Itgae	0.269	0.803	0.216	0.007	0.594	0.057	2.626	0.731	0.415	16407	Itgae	chr11	+	NM_1729	P15122	protein_cc	A530055J	integrin al	Itgae
3152	Gsg2	1.138	0.826	0.940	0.804	0.734	0.899	2.024	2.210	1.934	14841	Gsg2	chr11	-	NM_0103	P4573	protein_cc	Haspin	germ cell-	Gsg2
3153	P2rx5	1.023	3.051	3.120	0.000	0.182	0.423	0.000	0.033	1.642	94045	P2rx5	chr11	+	NM_0333	P12199	protein_cc		purinergic	P2rx5
3154	Emc6	0.763	1.719	1.312	0.093	0.006	0.038	10.960	10.570	11.352	66048	Emc6	chr11	-	NM_0253	P21867	protein_cc	0610009E	ER membr	Emc6
3155	Tax1bp3	2.002	1.148	2.298	0.013	0.448	0.108	5.569	6.570	6.769	76281	Tax1bp3	chr11	+	NM_0295	P3388	protein_cc	1300011C	Tax1 (hum	Tax1bp3
3156	Ctns	0.594	1.459	0.867	0.024	0.090	0.473	6.959	6.209	6.753	83429	Ctns	chr11	-	NM_0312	P17004	protein_cc	AI195360|	cystinosis,	Ctns
3157	Shpk	0.240	1.858	0.445	0.000	0.002	0.006	7.825	5.763	6.658	74637	Shpk	chr11	-	NM_0290	P15718	protein_cc	4930431K	sedohept	Shpk
3158	Trpv1	0.294	1.809	0.532	0.182	0.191	0.599	1.811	0.045	0.899	193034	Trpv1	chr11	+	NM_0010	P15602	protein_cc	OTRPC1|	transient	Trpv1
3159	Trpv3	0.552	0.990	0.547	0.182	1.000	0.423	0.871	0.015	0.000	246788	Trpv3	chr11	+	NM_1450	P15377	protein_cc	1110036I1	transient	Trpv3
3160	Aspa	0.162	1.327	0.215	0.001	0.599	0.050	5.979	3.357	3.765	11484	Aspa	chr11	-	NM_0231	P10371	protein_cc	Acy-2| Ac	aspartoac	Aspa
3161	Spata22	1.000	1.000	1.000	1.000	1.000	1.000	0.000	0.000	0.000	380709	Spata22	chr11	+	NM_0010	P16084	protein_cc	Gm882	spermatog	Spata22
3162	Olfr20	1.000	1.000	1.000	1.000	1.000	1.000	0.000	0.000	0.000	258925	Olfr20	chr11	+	NM_1469	P13510	protein_cc	MOR135-1	olfactory	Olfr20
3163	Olfr376	1.000	1.000	1.000	1.000	1.000	1.000	0.000	0.000	0.000	258924	Olfr376	chr11	+	NM_1469	P9486	protein_cc	MOR135-1	olfactory	Olfr376
3164	Olfr1	1.000	1.000	1.000	1.000	1.000	1.000	0.000	0.000	0.000	258923	Olfr1	chr11	-	NM_1469	P24712	protein_cc	I54| MOR	olfactory	Olfr1
3165	Olfr75-ps1	1.000	1.000	1.000	1.000	1.000	1.000	0.000	0.000	0.000	258186	Olfr75-ps1	chr11	-	NR_00285	.	misc_RNA	MOR135-2	olfactory	Olfr75-ps1
3166	Olfr378	0.716	0.995	0.712	0.182	1.000	0.423	0.490	0.008	0.000	259026	Olfr378	chr11	-	NM_1470	P4519	protein_cc	MOR135-2	olfactory	Olfr378
3167	Olfr380	1.000	1.000	1.000	1.000	1.000	1.000	0.000	0.000	0.000	259027	Olfr380	chr11	-	NM_1470	P10586	protein_cc	MOR135-1	olfactory	Olfr380
3168	Olfr381	1.000	1.000	1.000	1.000	1.000	1.000	0.000	0.000	0.000	259024	Olfr381	chr11	-	NM_1470	P20139	protein_cc	MOR135-4	olfactory	Olfr381
3169	Olfr382	1.000	1.000	1.000	1.000	1.000	1.000	0.000	0.000	0.000	258435	Olfr382	chr11	-	NM_1464	P23660	protein_cc	MOR135-1	olfactory	Olfr382
3170	Olfr385	0.716	0.995	0.712	0.182	1.000	0.423	0.490	0.008	0.000	259025	Olfr385	chr11	-	NM_1470	P4119	protein_cc	MOR135-3	olfactory	Olfr385
3171	Olfr384	1.000	1.000	1.000	1.000	1.000	1.000	0.000	0.000	0.000	193053	Olfr384	chr11	+	NM_2072	P12927	protein_cc	GA_x5J8B	olfactory	Olfr384
3172	Zfp735	1.000	1.000	1.000	1.000	1.000	1.000	0.000	0.000	0.000	76390	Zfp735	chr11	+	NM_0011	P23798	protein_cc	1700012C	zinc finger	Zfp735

3173	Olfr389	1.000	1.000	1.000	1.000	1.000	1.000	0.000	0.000	0.000	259011	Olfr389	chr11	-	NM_1470	P20672	protein_cc	MOR135-€	olfactory r	Olfr389
3174	Olfr390	1.000	1.000	1.000	1.000	1.000	1.000	0.000	0.000	0.000	258344	Olfr390	chr11	+	NM_1463	P15121	protein_cc	MOR135-2	olfactory r	Olfr390
3175	Olfr391-ps	1.000	1.000	1.000	1.000	1.000	1.000	0.000	0.000	0.000	258236	Olfr391-ps	chr11	-	NM_0011	P10732	protein_cc	MOR135-2	olfactory r	Olfr391-ps
3176	Olfr392	1.000	1.000	1.000	1.000	1.000	1.000	0.000	0.000	0.000	259008	Olfr392	chr11	-	NM_1470	P16729	protein_cc	MOR135-9	olfactory r	Olfr392
3177	Olfr393	1.000	1.000	1.000	1.000	1.000	1.000	0.000	0.000	0.000	259010	Olfr393	chr11	-	NM_1470	P9927	protein_cc	MOR135-7	olfactory r	Olfr393
3178	Olfr394	1.000	1.000	1.000	1.000	1.000	1.000	0.000	0.000	0.000	259009	Olfr394	chr11	-	NM_1470	P12552	protein_cc	MOR135-8	olfactory r	Olfr394
3179	Olfr395	1.000	1.000	1.000	1.000	1.000	1.000	0.000	0.000	0.000	259007	Olfr395	chr11	-	NM_1470	P21252	protein_cc	MOR135-1	olfactory r	Olfr395
3180	Olfr23	1.000	1.000	1.000	1.000	1.000	1.000	0.000	0.000	0.000	18321	Olfr23	chr11	+	NM_0109	P4925	protein_cc	MOR135-2	olfactory r	Olfr23
3181	Olfr397	1.000	1.000	1.000	1.000	1.000	1.000	0.000	0.000	0.000	258343	Olfr397	chr11	+	NM_1463	P24699	protein_cc	MOR135-2	olfactory r	Olfr397
3182	Olfr398	1.000	1.000	1.000	1.000	1.000	1.000	0.000	0.000	0.000	258705	Olfr398	chr11	-	NM_1467	P12596	protein_cc	MOR157-1	olfactory r	Olfr398
3183	Olfr139	1.000	1.000	1.000	1.000	1.000	1.000	0.000	0.000	0.000	259005	Olfr139	chr11	-	NM_1470	P5479	protein_cc	M5| MOR	olfactory r	Olfr139
3184	Olfr399	1.000	1.000	1.000	1.000	1.000	1.000	0.000	0.000	0.000	259006	Olfr399	chr11	-	NM_1470	P20960	protein_cc	MOR135-2	olfactory r	Olfr399
3185	Zfp616	1.000	1.000	1.000	1.000	1.000	1.000	0.000	0.000	0.000	327963	Zfp616	chr11	+	NM_0011	P22672	protein_cc	Gm12330	zinc finger	Zfp616
3186	Olfr401	1.000	1.000	1.000	1.000	1.000	1.000	0.000	0.000	0.000	258701	Olfr401	chr11	+	NM_1467	P4606	protein_cc	MOR255-€	olfactory r	Olfr401
3187	Olfr402	1.000	1.000	1.000	1.000	1.000	1.000	0.000	0.000	0.000	258703	Olfr402	chr11	+	NM_1467	P12485	protein_cc	MOR255-4	olfactory r	Olfr402
3188	Olfr403	1.000	1.000	1.000	1.000	1.000	1.000	0.000	0.000	0.000	404316	Olfr403	chr11	+	NM_2076	P17160	protein_cc	IA7| MOR	olfactory r	Olfr403
3189	Olfr43	1.000	1.000	1.000	1.000	1.000	1.000	0.000	0.000	0.000	258706	Olfr43	chr11	-	NM_1467	P16245	protein_cc	IA7| MOR	olfactory r	Olfr43
3190	Olfr406	1.000	1.000	1.000	1.000	1.000	1.000	0.000	0.000	0.000	258181	Olfr406	chr11	+	NM_0010	P8553	protein_cc	MOR133-1	olfactory r	Olfr406
3191	Olfr59	1.000	1.000	1.000	1.000	1.000	1.000	0.000	0.000	0.000	18359	Olfr59	chr11	+	NM_0110	P8715	protein_cc	IH3| MOR	olfactory r	Olfr59
3192	Olfr410	1.000	1.000	1.000	1.000	1.000	1.000	0.000	0.000	0.000	258702	Olfr410	chr11	-	NM_1467	P2423	protein_cc	MOR255-5	olfactory r	Olfr410
3193	Olfr411	1.000	1.000	1.000	1.000	1.000	1.000	0.000	0.000	0.000	258704	Olfr411	chr11	-	NM_1467	P15664	protein_cc	MOR255-3	olfactory r	Olfr411
3194	Olfr412	1.000	1.000	1.000	1.000	1.000	1.000	0.000	0.000	0.000	258153	Olfr412	chr11	+	NM_0010	P24013	protein_cc	MOR127-5	olfactory r	Olfr412
3195	Rap1gap2	5.368	0.741	3.979	0.083	0.517	0.064	2.609	5.033	4.601	380711	Rap1gap2	chr11	-	NM_0010	P2377	protein_cc	AU067654	RAP1 GTP	Rap1gap2
3196	E130309D	1.000	1.000	1.000	1.000	1.000	1.000	0.000	0.000	0.000	.	E130309D	chr11	+	NM_0010	P20952	.	.	.	E130309D
3197	Cluh	0.226	1.740	0.392	0.000	0.001	0.010	11.763	9.614	10.413	74148	Cluh	chr11	+	NM_0010	P16894	protein_cc	1300001I(	clustered	Cluh
3198	Pafah1b1	1.439	0.857	1.233	0.008	0.264	0.414	9.566	10.091	9.868	18472	Pafah1b1	chr11	-	NR_03761	.	protein_cc	LIS-1| Lis1	platelet-ac	Pafah1b1
3199	Mettl16	1.327	1.142	1.516	0.000	0.497	0.339	7.808	8.216	8.408	67493	Mettl16	chr11	+	NM_0261	P18338	protein_cc	2610100D	methyltra	Mettl16
3200	Mnt	0.837	1.141	0.954	0.008	0.008	0.568	6.548	6.291	6.481	17428	Mnt	chr11	+	NM_0108	P1949	protein_cc	bHLHd3| I	max bindi	Mnt
3201	Sgsm2	0.096	1.828	0.176	0.000	0.147	0.030	6.547	3.172	4.042	97761	Sgsm2	chr11	-	NM_1979	P12587	protein_cc	D630003G	small G pr	Sgsm2
3202	Tsr1	1.907	0.779	1.486	0.000	0.003	0.083	8.131	9.062	8.702	104662	Tsr1	chr11	+	NM_1773	P21160	protein_cc	AU040765	TSR1 20S r	Tsr1
3203	Snord91a	1.023	3.048	3.117	0.000	0.001	0.028	0.000	0.033	1.641	10021746	Snord91a	chr11	+	NR_02856	.	snoRNA	MBII-296	small nucl	Snord91a
3204	Srr	0.535	0.891	0.477	0.001	0.253	0.022	9.932	9.031	8.865	27364	Srr	chr11	-	NM_0137	P9819	protein_cc	M100034|	serine rac	Srr
3205	Smg6	0.403	1.256	0.506	0.001	0.062	0.068	7.638	6.325	6.654	103677	Smg6	chr11	+	NM_0010	P2890	protein_cc	AI317223|	Smg-6 hor	Smg6
3206	Hic1	0.671	1.030	0.691	0.142	0.949	0.624	4.531	3.955	3.998	15248	Hic1	chr11	-	NM_0010	P23317	protein_cc	AA408311	hypermet	Hic1
3207	Ovca2	2.135	1.057	2.256	0.003	0.640	0.050	7.192	8.286	8.366	246257	Ovca2	chr11	-	NM_0271	P14717	protein_cc	9030414M	candidate	Ovca2
3208	Dph1	2.044	1.380	2.821	0.001	0.139	0.121	6.025	7.057	7.522	116905	Dph1	chr11	-	NM_1444	P25859	protein_cc	2310011M	diphthami	Dph1
3209	Rtn4rl1	0.058	4.808	0.281	0.000	0.153	0.070	6.833	2.736	5.002	237847	Rtn4rl1	chr11	+	NM_1777	P14287	protein_cc	Ngr3| Ngr	reticulon 4	Rtn4rl1
3210	Rpa1	0.269	1.905	0.512	0.000	0.088	0.135	7.627	5.731	6.661	68275	Rpa1	chr11	-	NM_0266	P19407	protein_cc	5031405K	replicatior	Rpa1
3211	Smyd4	0.587	1.452	0.852	0.010	0.529	0.840	3.925	3.157	3.695	319822	Smyd4	chr11	+	NM_0011	P12906	protein_cc	G430029E	SET and M	Smyd4
3212	Serpinf1	0.447	0.897	0.401	0.000	0.295	0.012	11.398	10.237	10.081	20317	Serpinf1	chr11	-	NM_0113	P22287	protein_cc	AI195227|	serine (or	Serpinf1
3213	Serpinf2	0.831	0.724	0.602	0.456	0.244	0.174	12.778	12.511	12.045	18816	Serpinf2	chr11	-	NM_0088	P11779	protein_cc	AI747498|	serine (or	Serpinf2
3214	Wdr81	0.467	0.695	0.324	0.004	0.196	0.012	7.849	6.749	6.224	192652	Wdr81	chr11	-	NM_1389	P9827	protein_cc	BC054822	WD repea	Wdr81
3215	Tlcd2	0.151	1.019	0.154	0.000	0.790	0.010	10.981	8.249	8.277	380712	Tlcd2	chr11	+	NM_0272	P3208	protein_cc	.	TLC doma	Tlcd2
3216	Prpf8	0.528	0.947	0.500	0.012	0.764	0.041	9.610	8.690	8.612	192159	Prpf8	chr11	+	NM_1386	P14862	protein_cc	AU019467	pre-mRNA	Prpf8
3217	Rilp	0.017	8.475	0.143	0.000	0.073	0.011	7.956	2.070	5.153	280408	Rilp	chr11	+	NM_0010	P5717	protein_cc	AI835553|	Rab intera	Rilp
3218	Scarf1	1.053	0.749	0.788	0.884	0.446	0.460	5.688	5.763	5.345	380713	Scarf1	chr11	+	NM_0010	P10712	protein_cc	AA986099	scavenger	Scarf1
3219	Slc43a2	0.171	3.741	0.639	0.000	0.040	0.289	6.730	4.182	6.085	215113	Slc43a2	chr11	+	NM_1733	P653	protein_cc	7630402D	solute car	Slc43a2
3220	Pitpna	1.199	1.214	1.456	0.013	0.192	0.239	8.623	8.886	9.165	18738	Pitpna	chr11	+	NM_0088	P24269	protein_cc	Pitpn| vb|	phosphati	Pitpna
3221	Gm12338	0.770	1.090	0.839	0.247	0.690	0.299	12.137	11.760	11.884	654359	Gm12338	chr11	.	NR_11047	.	misc_RNA	OTTMUSG	predicted	Gm12338
3222	Inpp5k	0.756	1.653	1.249	0.079	0.088	0.595	5.920	5.516	6.241	19062	Inpp5k	chr11	+	NM_0089	P10433	protein_cc	C62| Pps|	inositol po	Inpp5k
3223	Myo1c	1.116	1.242	1.386	0.307	0.293	0.365	7.378	7.535	7.849	17913	Myo1c	chr11	+	NM_0086	P11173	protein_cc	C80397| r	myosin IC	Myo1c
3224	Crk	1.687	0.546	0.921	0.006	0.014	0.748	7.394	8.148	7.275	12928	Crk	chr11	+	NM_0012	P11535	protein_cc	c-Crk| Crk	v-crk aviar	Crk
3225	Ywhae	1.005	0.997	1.002	0.799	0.966	0.989	12.219	12.226	12.221	22627	Ywhae	chr11	+	NM_0095	P11791	protein_cc	AU019196	tyrosine 3	Ywhae
3226	Doc2b	1.025	1.254	1.285	0.967	0.722	0.798	1.097	1.132	1.459	13447	Doc2b	chr11	+	NM_0078	P3204	protein_cc	.	double C2	Doc2b
3227	Rph3al	0.352	3.536	1.245	0.037	0.163	0.815	3.928	2.421	4.243	380714	Rph3al	chr11	-	NM_0295	P6061	protein_cc	Gm1753|	rabphilin 3	Rph3al
3228	1700016K	1.000	1.000	1.000	1.000	1.000	1.000	0.000	0.000	0.000	74230	1700016K	chr11	+	NM_1986	P7693	protein_cc	Liat1	RIKEN cDN	1700016K1
3229	Fam101b	0.762	1.363	1.038	0.309	0.149	0.878	4.611	4.218	4.664	76566	Fam101b	chr11	+	NM_0296	P22289	protein_cc	1500005K	family wit	Fam101b
3230	Vps53	0.906	1.151	1.043	0.059	0.031	0.678	8.197	8.055	8.258	68299	Vps53	chr11	-	NM_0266	P2256	protein_cc	2010002A	vacuolar p	Vps53
3231	Fam57a	0.552	0.990	0.547	0.182	1.000	0.423	0.871	0.015	0.000	116972	Fam57a	chr11	+	NM_0277	P15502	protein_cc	2310047D	family wit	Fam57a
3232	Gemin4	1.938	0.801	1.552	0.028	0.196	0.187	4.497	5.451	5.130	276919	Gemin4	chr11	-	NM_1773	P8215	protein_cc	4932415L(	gem (nucl	Gemin4
3233	Dbil5	0.291	1.374	0.400	0.056	0.205	0.313	1.825	0.043	0.501	13168	Dbil5	chr11	+	NM_0212	P4296	protein_cc	ELP	diazepam	Dbil5
3234	Glod4	0.850	1.123	0.954	0.003	0.347	0.797	9.520	9.285	9.452	67201	Glod4	chr11	-	NM_0260	P7417	protein_cc	1700082G	glyoxalase	Glod4
3235	Rnmtl1	1.225	1.199	1.468	0.096	0.014	0.080	5.905	6.198	6.460	67390	Rnmtl1	chr11	+	NM_1832	P10340	protein_cc	4833420N	RNA meth	Rnmtl1
3236	Nxn	1.677	1.140	1.911	0.154	0.589	0.055	5.154	5.900	6.089	18230	Nxn	chr11	-	NM_0087	P20513	protein_cc	l11Jus13	nucleored	Nxn
3237	Timm22	1.301	1.456	1.894	0.343	0.168	0.171	8.680	9.060	9.602	56322	Timm22	chr11	+	NM_0012	P11072	protein_cc	Tim22	translocas	Timm22
3238	Abr	2.641	0.419	1.107	0.083	0.098	0.724	4.935	6.336	5.082	109934	Abr	chr11	-	NM_0012	P5968	protein_cc	.	active BCR	Abr
3239	Bhlha9	1.000	1.000	1.000	1.000	1.000	1.000	0.000	0.000	0.000	320522	Bhlha9	chr11	+	NM_1771	P11846	protein_cc	A830053C	basic helix	Bhlha9
3240	Tusc5	0.502	1.173	0.589	0.100	0.756	0.220	2.144	1.150	1.381	237858	Tusc5	chr11	+	NM_1777	P23830	protein_cc	C130069F	tumor sup	Tusc5
3241	Gosr1	3.008	1.102	3.314	0.003	0.813	0.352	5.437	7.026	7.165	53334	Gosr1	chr11	-	NM_0168	P23517	protein_cc	AI414660|	golgi SNAF	Gosr1
3242	Cpd	1.016	1.214	1.233	0.882	0.047	0.157	7.689	7.712	7.991	12874	Cpd	chr11	-	NM_0077	P10540	protein_cc	AA960140	carboxype	Cpd
3243	Tmigd1	1.026	3.449	3.539	0.000	0.181	0.423	0.001	0.038	1.824	66601	Tmigd1	chr11	+	NM_0256	P3579	protein_cc	2010002A	transmem	Tmigd1
3244	Blmh	0.843	1.450	1.222	0.067	0.011	0.243	9.271	9.024	9.560	104184	Blmh	chr11	+	NM_1786	P14664	protein_cc	AI035728|	bleomycin	Blmh
3245	Slc6a4	1.565	0.525	0.821	0.595	0.477	0.804	1.084	1.731	0.800	15567	Slc6a4	chr11	+	NM_0104	P22176	protein_cc	5-HTT| AI	solute car	Slc6a4
3246	Ccdc55	2.531	0.630	1.594	0.000	0.002	0.027	8.698	10.038	9.370	.	Ccdc55	chr11	-	NM_0010	P15527	.	.	.	Ccdc55
3247	Efcab5	1.000	1.000	1.000	1.000	1.000	1.000	0.000	0.000	0.000	319634	Efcab5	chr11	-	NM_1769	P15073	protein_cc	4930563A	EF-hand ca	Efcab5
3248	Ssh2	1.053	0.969	1.020	0.037	0.663	0.881	8.550	8.624	8.578	237860	Ssh2	chr11	+	NM_1777	P6600	protein_cc	mSSH-2L|	slingshot h	Ssh2
3249	Coro6	1.000	1.000	1.000	1.000	1.000	1.000	0.000	0.000	0.000	216961	Coro6	chr11	+	NM_1391	P7469	protein_cc	.	coronin 6	Coro6
3250	Ankrd13b	0.145	4.477	0.651	0.000	0.049	0.348	3.564	0.783	2.945	268445	Ankrd13b	chr11	-	NM_1729	P8698	protein_cc	AW12458	ankyrin re	Ankrd13b
3251	Git1	1.924	1.463	2.815	0.072	0.071	0.010	5.547	6.491	7.040	216963	Git1	chr11	+	NM_0010	P21508	protein_cc	Cat-1| p9	G protein-	Git1
3252	Trp53i13	0.873	1.324	1.156	0.690	0.383	0.284	3.447	3.252	3.656	216964	Trp53i13	chr11	-	NM_0010	P11521	protein_cc	2410019G	transform	Trp53i13
3253	Abhd15	0.113	1.527	0.173	0.000	0.000	0.003	9.220	6.079	6.690	67477	Abhd15	chr11	+	NM_0261	P17479	protein_cc	1300007F(	abhydrola	Abhd15
3254	Taok1	1.363	0.914	1.247	0.014	0.252	0.007	10.326	10.773	10.644	216965	Taok1	chr11	-	NM_1448	P26075	protein_cc	2810468K	TAO kinas	Taok1
3255	Nufip2	1.695	0.769	1.303	0.000	0.177	0.518	10.016	10.777	10.398	68564	Nufip2	chr11	+	NM_0010	P22479	protein_cc	1110001M	nuclear fra	Nufip2
3256	Cryba1	1.000	1.000	1.000	1.000	1.000	1.000	0.000	0.000	0.000	12957	Cryba1	chr11	-	NM_0099	P4969	protein_cc	BA3/A1| C	crystallin,	Cryba1
3257	Myo18a	0.806	1.287	1.038	0.012	0.088	0.853	9.626	9.315	9.680	360013	Myo18a	chr11	+	NM_0012	P14532	protein_cc	MAJN| My	myosin XV	Myo18a
3258	Pipox	0.233	1.118	0.261	0.002	0.535	0.052	11.506	9.407	9.568	19193	Pipox	chr11	-	NM_0089	P6666	protein_cc	LPIPOX| P	pipecolic a	Pipox
3259	Gm11190	1.000	1.000	1.000	1.000	1.000	1.000	0.000	0.000	0.000	791423	Gm11190	chr11	-	NR_03354	.	lncRNA	OTTMUSG	predicted	Gm11190
3260	Sez6	1.000	1.000	1.000	1.000	1.000	1.000	0.000	0.000	0.000	20370	Sez6	chr11	+	NM_0012	P16435	protein_cc	BSRP-C| D	seizure re	Sez6
3261	Phf12	1.254	0.801	1.004	0.003	0.084	0.984	7.420	7.746	7.426	268448	Phf12	chr11	+	NM_1748	P7485	protein_cc	2410142K	PHD finge	Phf12
3262	Dhrs13	2.328	0.929	2.164	0.000	0.761	0.298	5.380	6.599	6.493	70451	Dhrs13	chr11	+	NM_1832	P5700	protein_cc	2610209N	dehydrog	Dhrs13
3263	Flot2	2.479	1.248	3.093	0.004	0.071	0.003	7.905	9.215	9.534	14252	Flot2	chr11	+	NM_0012	P3362	protein_cc	AI573412|	flotillin 2	Flot2

3264	Eral1	0.682	1.300	0.887	0.004	0.017	0.387	6.832	6.281	6.660	57837	Eral1	chr11	-	NM_0223	P16112	protein_cc	2610524P	Era (G-pro	Eral1
3265	Fam222b	1.058	0.890	0.942	0.395	0.014	0.585	8.287	8.368	8.201	216971	Fam222b	chr11	+	NM_1454	P21875	protein_cc	96300201	family wit	Fam222b
3266	Traf4	0.703	0.755	0.530	0.123	0.044	0.177	7.555	7.046	6.640	22032	Traf4	chr11	-	NM_0094	P22552	protein_cc	A530032N	TNF recep	Traf4
3267	Nek8	0.514	1.482	0.761	0.182	0.280	0.717	4.609	3.648	4.216	140859	Nek8	chr11	-	NM_0808	P22520	protein_cc	4632401F	NIMA (ne	Nek8
3268	Tlcd1	0.272	1.736	0.472	0.000	0.014	0.020	7.835	5.956	6.751	68385	Tlcd1	chr11	+	NM_0012	P26159	protein_cc	.	TLC doma	Tlcd1
3269	Rpl23a	1.136	1.138	1.294	0.011	0.003	0.043	8.628	8.812	8.999	268449	Rpl23a	chr11	-	NM_2075	P17799	protein_cc	BC029892	ribosomal	Rpl23a
3270	Snord42a	2.827	2.196	6.210	0.292	0.344	0.377	1.642	3.141	4.276	10052907	Snord42a	chr11	-	NR_03768	.	snoRNA	MBII-292	small nucl	Snord42a
3271	Snord4a	0.364	0.981	0.357	0.000	1.000	0.013	1.485	0.027	0.000	10021653	Snord4a	chr11	-	NR_03070	.	snoRNA	.	small nucl	Snord4a
3272	Snord42b	2.754	0.793	2.184	0.304	0.748	0.423	0.001	1.463	1.128	10052907	Snord42b	chr11	-	NR_03768	.	snoRNA	MBII-287	small nucl	Snord42b
3273	Rab34	0.712	2.718	1.935	0.262	0.099	0.411	5.046	4.556	5.998	19376	Rab34	chr11	+	NM_0011	P17503	protein_cc	AI326479	RAB34, m	Rab34
3274	Proca1	0.409	2.291	0.937	0.131	0.033	0.923	4.354	3.064	4.260	216974	Proca1	chr11	+	NM_0010	P12397	protein_cc	4933404M	protein in	Proca1
3275	Supt6	1.916	0.624	1.195	0.001	0.001	0.219	8.009	8.948	8.266	20926	Supt6	chr11	-	NM_0092	P19935	protein_cc	5131400N	suppresso	Supt6
3276	Sdf2	0.877	0.811	0.711	0.429	0.282	0.049	8.918	8.729	8.426	20316	Sdf2	chr11	+	NM_0091	P11782	protein_cc	AI853825	stromal ce	Sdf2
3277	2610507B	0.498	0.947	0.471	0.029	0.818	0.108	9.954	8.947	8.869	72503	2610507B	chr11	+	NM_0010	P419	protein_cc	AU022687	RIKEN cDN	2610507B
3278	BC030499	1.281	1.170	1.499	0.340	0.719	0.672	0.797	1.153	1.380	216976	BC030499	chr11	+	NM_0012	P2918	protein_cc	BC037104	cDNA seq	BC030499
3279	Spag5	0.278	2.648	0.736	0.025	0.186	0.693	1.905	0.058	1.463	54141	Spag5	chr11	+	NM_0174	P5529	protein_cc	AI874642	sperm ass	Spag5
3280	Aldoc	0.711	1.510	1.075	0.132	0.091	0.838	6.898	6.407	7.002	11676	Aldoc	chr11	+	NM_0091	P13014	protein_cc	AI847350	aldolase C	Aldoc
3281	Pigs	0.743	1.676	1.245	0.062	0.021	0.346	7.080	6.652	7.396	276846	Pigs	chr11	+	NM_2014	P24064	protein_cc	BC058979	phosphati	Pigs
3282	Unc119	0.541	1.956	1.059	0.000	0.138	0.910	5.578	4.693	5.661	22248	Unc119	chr11	+	NM_0116	P14673	protein_cc	D11Bhm5	unc-119 li	Unc119
3283	Foxn1	1.000	1.000	1.000	1.000	1.000	1.000	0.000	0.000	0.000	15218	Foxn1	chr11	-	NM_0012	P1526	protein_cc	D11Bhm1	forkhead b	Foxn1
3284	Slc13a2os	1.244	0.662	0.823	0.556	0.317	0.823	2.225	2.540	1.944	790911	Slc13a2os	chr11	+	NR_00328	.	lncRNA	Gm11194	solute car	Slc13a2os
3285	Slc13a2	0.138	1.462	0.201	0.002	0.325	0.038	6.039	3.179	3.727	20500	Slc13a2	chr11	-	NM_0224	P23609	protein_cc	mNaDC-1	solute car	Slc13a2
3286	Slc46a1	0.309	1.392	0.431	0.000	0.070	0.019	8.609	6.916	7.394	52466	Slc46a1	chr11	+	NM_0267	P5629	protein_cc	1110002C	solute car	Slc46a1
3287	Sarm1	17.810	1.008	17.945	0.000	0.970	0.082	0.002	4.157	4.168	237868	Sarm1	chr11	-	NM_1727	P16754	protein_cc	A830091I	sterile alp	Sarm1
3288	Vtn	1.270	1.122	1.425	0.052	0.397	0.224	13.323	13.668	13.834	22370	Vtn	chr11	+	NM_0117	P20095	protein_cc	AI256434	vitronecti	Vtn
3289	Sebox	18.417	1.438	26.487	0.002	0.066	0.018	0.002	4.205	4.730	18292	Sebox	chr11	+	NM_0087	P5671	protein_cc	OG9 | Og9	SEBOX ho	Sebox
3290	Tmem199	2.494	0.691	1.723	0.026	0.149	0.009	5.919	7.237	6.704	195040	Tmem199	chr11	-	NM_1991	P24943	protein_cc	AI316787	transmem	Tmem199
3291	Poldip2	0.572	1.359	0.777	0.074	0.196	0.451	9.163	8.357	8.800	67811	Poldip2	chr11	+	NM_0263	P18223	protein_cc	1300003F	polymeras	Poldip2
3292	Tnfaip1	2.050	0.732	1.500	0.001	0.025	0.078	9.168	10.204	9.754	21927	Tnfaip1	chr11	-	NM_0011	P4452	protein_cc	Bacurd2 |	tumor nec	Tnfaip1
3293	Ift20	1.523	0.950	1.447	0.002	0.600	0.137	10.600	11.207	11.134	55978	Ift20	chr11	+	NM_0188	P2170	protein_cc	0610009H	intraflagel	Ift20
3294	Tmem97	0.739	1.808	1.336	0.004	0.008	0.225	9.466	9.030	9.884	69071	Tmem97	chr11	-	NM_1337	P3807	protein_cc	1810014L	transmem	Tmem97
3295	Nlk	1.111	0.967	1.074	0.574	0.844	0.850	7.562	7.715	7.666	18099	Nlk	chr11	-	NM_0087	P12148	protein_cc	AI194375	nemo like	Nlk
3296	Fam58b	1.413	0.864	1.221	0.021	0.487	0.644	6.684	7.183	6.972	69109	Fam58b	chr11	-	NM_1979	P876	protein_cc	1810009O	family wit	Fam58b
3297	Lyrm9	0.675	2.095	1.414	0.136	0.004	0.182	6.483	5.916	6.983	66274	Lyrm9	chr11	+	NM_0010	P7943	protein_cc	1810012P	LYR motif	Lyrm9
3298	Nos2	75.972	0.259	19.679	0.000	0.006	0.343	0.589	6.837	4.888	18126	Nos2	chr11	+	NM_0109	P12990	protein_cc	i-NOS | iN	nitric oxid	Nos2
3299	Lgals9	1.702	0.935	1.591	0.000	0.539	0.144	12.174	12.941	12.843	16859	Lgals9	chr11	-	NM_0107	P7312	protein_cc	AA407335	lectin, gala	Lgals9
3300	Ksr1	0.716	0.995	0.712	0.182	1.000	0.423	0.490	0.008	0.000	16706	Ksr1	chr11	-	NM_0135	P745	protein_cc	AW49249	kinase sup	Ksr1
3301	Gm11201	0.885	0.629	0.557	0.781	1.000	0.422	0.844	0.668	0.000	790910	Gm11201	chr11	+	NR_04587	.	lncRNA	ENSMUSG	predicted	Gm11201
3302	Wsb1	0.845	0.531	0.449	0.141	0.005	0.084	9.331	9.087	8.174	78889	Wsb1	chr11	-	NM_0196	P7617	protein_cc	1110056B	WD repea	Wsb1
3303	Nf1	1.789	1.433	2.562	0.045	0.124	0.104	5.277	6.116	6.634	18015	Nf1	chr11	+	NM_0108	P16391	protein_cc	AW49427	neurofibro	Nf1
3304	AU040972	1.000	1.000	1.000	1.000	1.000	1.000	0.000	0.000	0.000	104522	AU040972	chr11	-	NR_04530	.	lncRNA	AU040954	expressed	AU040972
3305	Omg	3.913	0.255	0.999	0.119	1.000	0.423	0.001	1.969	0.000	18377	Omg	chr11	-	NM_0194	P7824	protein_cc	.	oligodend	Omg
3306	Gm21975	1.214	0.551	0.669	0.787	0.452	0.617	3.361	3.642	2.782	10148821	Gm21975	chr11	-	NM_1460	P10356	protein_cc	Evi2a-evi2	predicted	Gm21975
3307	Evi2b	1.214	0.551	0.669	0.787	0.452	0.617	3.361	3.642	2.782	216984	Evi2b	chr11	-	NM_0010	P10356	protein_cc	Evi-2 | Evi2	ecotropic	Evi2b
3308	Evi2a	1.485	0.918	1.363	0.298	0.799	0.478	3.777	4.347	4.223	14017	Evi2a	chr11	-	NM_0101	P17691	protein_cc	AW49189	ecotropic	Evi2a
3309	Rab11fip4	2.318	0.488	1.131	0.054	0.002	0.881	2.106	3.319	2.284	268451	Rab11fip4	chr11	+	NM_1755	P25504	protein_cc	A730072L	RAB11 fan	Rab11fip4
3310	Rab11fip4	1.000	1.000	1.000	1.000	1.000	1.000	0.000	0.000	0.000	790912	Rab11fip4	chr11	-	NR_00328	.	lncRNA	Gm11202	RAB11 fan	Rab11fip4
3311	Rab11fip4	1.000	1.000	1.000	1.000	1.000	1.000	0.000	0.000	0.000	72218	Rab11fip4	chr11	-	NR_04589	.	lncRNA	1700020F	RAB11 fan	Rab11fip4
3312	Utp6	1.554	1.026	1.593	0.001	0.625	0.006	7.841	8.476	8.513	216987	Utp6	chr11	-	NM_1448	P13784	protein_cc	4732497O	UTP6 sma	Utp6
3313	Suz12	1.258	0.949	1.194	0.000	0.004	0.008	8.456	8.787	8.712	52615	Suz12	chr11	+	NM_1991	P16793	protein_cc	2610028O	suppresso	Suz12
3314	Crlf3	1.322	0.686	0.907	0.001	0.011	0.585	6.412	6.815	6.271	54394	Crlf3	chr11	-	NM_0012	P384	protein_cc	BB164954	cytokine r	Crlf3
3315	Atad5	0.533	1.308	0.698	0.030	0.221	0.211	4.335	3.428	3.815	237877	Atad5	chr11	+	NM_0010	P17453	protein_cc	C130052G	ATPase fa	Atad5
3316	Tefm	1.343	0.716	0.962	0.036	0.010	0.852	6.473	6.899	6.418	68550	Tefm	chr11	-	NM_1832	P10825	protein_cc	1110002N	transcripti	Tefm
3317	Adap2	0.253	1.190	0.301	0.000	0.663	0.065	7.913	5.930	6.180	216991	Adap2	chr11	+	NM_1721	P21871	protein_cc	Centa2	ArfGAP wi	Adap2
3318	Rnf135	2.092	0.372	0.779	0.000	0.000	0.262	6.812	7.877	6.452	71956	Rnf135	chr11	+	NM_0280	P16501	protein_cc	0610037N	ring finger	Rnf135
3319	Rhot1	0.767	0.937	0.719	0.002	0.320	0.085	8.563	8.181	8.087	59040	Rhot1	chr11	+	NM_0011	P881	protein_cc	2210403N	ras homol	Rhot1
3320	Rhbdl3	4.510	0.296	1.334	0.000	0.000	0.423	0.001	2.174	0.416	246104	Rhbdl3	chr11	+	NM_1392	P22232	protein_cc	AI847581	rhomboid	Rhbdl3
3321	5730455P	0.669	1.175	0.787	0.031	0.403	0.266	7.812	7.233	7.466	70591	5730455P	chr11	+	NM_0274	P5920	protein_cc	.	RIKEN cDN	5730455P
3322	Zfp207	2.705	0.521	1.408	0.014	0.061	0.475	6.865	8.301	7.359	22680	Zfp207	chr11	+	NM_0011	P22819	protein_cc	8430401D	zinc finger	Zfp207
3323	Psmd11	1.037	1.037	1.075	0.758	0.750	0.234	10.436	10.490	10.541	69077	Psmd11	chr11	+	NM_1786	P10377	protein_cc	1700089D	proteasom	Psmd11
3324	Cdk5r1	1.640	0.261	0.429	0.000	0.000	0.044	4.244	4.958	3.021	12569	Cdk5r1	chr11	+	NM_0098	P23372	protein_cc	Cdk5r | D1	cyclin-dep	Cdk5r1
3325	Myo1d	0.646	1.302	0.842	0.044	0.153	0.573	7.223	6.593	6.974	338367	Myo1d	chr11	-	NM_1773	P19136	protein_cc	9930104H	myosin ID	Myo1d
3326	C030013C	2.170	0.461	1.000	0.000	1.000	0.423	0.000	1.118	0.000	77417	C030013C	chr11	-	NR_12655	.	lncRNA	.	RIKEN cDN	C030013C
3327	Tmem98	0.172	1.755	0.302	0.002	0.275	0.097	7.696	5.158	5.969	103743	Tmem98	chr11	+	NM_0295	P2146	protein_cc	6530411B	transmem	Tmem98
3328	4930507D	1.000	1.000	1.000	1.000	1.000	1.000	0.000	0.000	0.000	75043	4930507D	chr11	-	NR_11044	.	lncRNA	.	RIKEN cDN	4930507D
3329	Spaca3	1.000	1.000	1.000	1.000	1.000	1.000	0.000	0.000	0.000	75622	Spaca3	chr11	+	NM_0293	P13345	protein_cc	1700025M	sperm acr	Spaca3
3330	Asic2	0.589	3.388	1.994	0.193	0.182	0.573	0.812	0.047	1.808	11418	Asic2	chr11	-	NM_0073	P18145	protein_cc	Accn1 | AC	acid-sensi	Asic2
3331	1700071K	1.005	1.439	1.446	0.000	0.182	0.423	0.000	0.008	0.532	237880	1700071K	chr11	+	NM_0010	P12550	protein_cc	.	RIKEN cDN	1700071K
3332	5530401A	1.000	1.000	1.000	1.000	1.000	1.000	0.000	0.000	0.000	71388	5530401A	chr11	+	NR_03801	.	lncRNA	.	RIKEN cDN	5530401A
3333	Ccl2	192.476	0.274	52.804	0.001	0.004	0.129	3.394	10.982	9.116	20296	Ccl2	chr11	+	NM_0113	P20307	protein_cc	AI323594	chemokin	Ccl2
3334	Ccl7	300.671	0.140	42.221	0.001	0.002	0.064	1.328	9.560	6.727	20306	Ccl7	chr11	+	NM_0136	P6990	protein_cc	fic | marc |	chemokin	Ccl7
3335	Ccl11	27.968	0.368	10.305	0.005	0.037	0.203	0.002	4.808	3.368	20292	Ccl11	chr11	+	NM_0113	P2558	protein_cc	eotaxin | S	chemokin	Ccl11
3336	Ccl12	12.003	0.145	1.741	0.000	0.000	0.527	0.346	3.931	1.146	20293	Ccl12	chr11	+	NM_0113	P12304	protein_cc	MCP-5 | Sc	chemokin	Ccl12
3337	Ccl8	59.372	0.292	17.348	0.016	0.057	0.261	0.003	5.894	4.119	20307	Ccl8	chr11	+	NM_0214	P19974	protein_cc	1810063B	chemokin	Ccl8
3338	Ccl1	1.000	1.000	1.000	1.000	1.000	1.000	0.000	0.000	0.000	20290	Ccl1	chr11	-	NM_0113	P2447	protein_cc	BF534335	chemokin	Ccl1
3339	Tmem132	1.857	0.551	1.023	0.028	0.020	0.931	4.370	5.263	4.402	270893	Tmem132	chr11	-	NM_0013	P1572	protein_cc	Gm644	transmem	Tmem132
3340	Gm11426	1.000	1.000	1.000	1.000	1.000	1.000	0.000	0.000	0.000	791422	Gm11426	chr11	-	NR_03358	.	lncRNA	OTTMUSG	predicted	Gm11426
3341	Cct6b	1.375	0.458	0.630	0.441	0.041	0.656	1.536	1.995	0.870	12467	Cct6b	chr11	-	NM_0098	P24672	protein_cc	CCT-zeta-2	chaperoni	Cct6b
3342	Zfp830	1.092	0.831	0.907	0.381	0.062	0.580	7.772	7.899	7.631	66983	Zfp830	chr11	+	NM_0258	P25549	protein_cc	2410003C	zinc finger	Zfp830
3343	Gm11423	0.588	3.230	1.898	0.279	0.084	0.364	4.542	3.775	5.467	10050327	Gm11423	chr11	-	NR_12606	.	lncRNA	.	predicted	Gm11423
3344	Lig3	1.794	0.675	1.211	0.000	0.002	0.227	8.001	8.845	8.278	16882	Lig3	chr11	+	NM_0012	P16149	protein_cc	D11Wsu78	ligase III, D	Lig3
3345	Rffl	1.725	0.584	1.008	0.000	0.002	0.966	7.767	8.554	7.779	67338	Rffl	chr11	-	NM_0260	P22291	protein_cc	1700051E	ring finger	Rffl
3346	Rad51d	0.845	0.570	0.482	0.006	0.000	0.007	6.577	6.334	5.523	19364	Rad51d	chr11	-	NM_0012	P19014	protein_cc	R51H3 | Ra	RAD51 pa	Rad51d
3347	Fndc8	2.800	1.087	3.044	0.023	0.707	0.064	0.001	1.486	1.607	78919	Fndc8	chr11	+	NM_0302	P18322	protein_cc	4930466G	fibronecti	Fndc8
3348	Nle1	1.242	2.174	2.701	0.044	0.045	0.175	5.084	5.398	6.518	217011	Nle1	chr11	-	NM_1454	P18126	protein_cc	AL022765	notchless	Nle1
3349	Unc45b	2.890	0.712	2.057	0.002	0.104	0.178	5.443	6.974	6.483	217012	Unc45b	chr11	+	NM_1786	P5380	protein_cc	AA445617	unc-45 my	Unc45b
3350	Slfn5os	1.596	1.401	2.237	0.295	0.518	0.423	0.000	0.675	1.162	76392	Slfn5os	chr11	-	NR_04593	.	lncRNA	1700003F	schlafen 5	Slfn5os
3351	Slfn5	8.241	0.416	3.428	0.000	0.002	0.145	7.578	10.621	9.355	327978	Slfn5	chr11	+	NM_1832	P9466	protein_cc	AI956840	schlafen 5	Slfn5
3352	Slfn9	11.723	0.128	1.499	0.005	0.007	0.641	3.752	7.303	4.336	237886	Slfn9	chr11	-	NM_1727	P7809	protein_cc	9830137M	schlafen 9	Slfn9
3353	Slfn8	15.451	0.261	4.037	0.000	0.000	0.139	3.741	7.691	5.755	276950	Slfn8	chr11	-	NM_0011	P18704	protein_cc	.	schlafen 8	Slfn8
3354	Slfn10-ps	36.245	0.650	23.541	0.001	0.053	0.042	0.330	5.510	4.888	237887	Slfn10-ps	chr11	-	NR_07352	.	misc_RNA	mFLJ0025	schlafen 1	Slfn10-ps

3355	Slfn2	15.194	0.489	7.431	0.000	0.000	0.001	5.532	9.457	8.425	20556	Slfn2	chr11	+	NM_0114	P16538	protein_cc	Shlf2	schlafen 2	Slfn2
3356	Slfn1	17.299	0.540	9.338	0.002	0.055	0.110	3.202	7.315	6.426	20555	Slfn1	chr11	+	NM_0114	P697	protein_cc	AV316259	schlafen 1	Slfn1
3357	Slfn4	445.473	0.120	53.548	0.000	0.000	0.003	0.003	8.802	5.746	20558	Slfn4	chr11	+	NM_0013	P25396	protein_cc	AI450778	schlafen 4	Slfn4
3358	Slfn3	5.393	1.262	6.805	0.052	0.458	0.038	0.713	3.145	3.480	20557	Slfn3	chr11	+	NM_0114	P9396	protein_cc	.	schlafen 3	Slfn3
3359	AI662270	2.344	0.782	1.834	0.000	0.009	0.001	6.497	7.726	7.372	10004363	AI662270	chr11	+	NR_01551	.	lncRNA	AW49647	expressed	AI662270
3360	Slfn14	1.580	0.633	1.000	0.305	1.000	0.423	0.000	0.660	0.000	237890	Slfn14	chr11	-	NM_0011	P13319	protein_cc	Gm20| Slf	schlafen 1	Slfn14
3361	AA465934	0.267	1.858	0.496	0.001	0.235	0.170	4.740	2.836	3.730	613254	AA465934	chr11	+	NR_02836	.	lncRNA	.	expressed	AA465934
3362	AI450353	0.311	2.125	0.661	0.001	0.015	0.109	4.854	3.170	4.257	103729	AI450353	chr11	+	NR_02836	.	lncRNA	.	expressed	AI450353
3363	Snord7	1.909	1.496	2.856	0.376	0.478	0.352	1.284	2.216	2.798	10030273	Snord7	chr11	+	NR_02836	.	snoRNA	.	small nucl	Snord7
3364	Pex12	1.797	1.159	2.083	0.087	0.598	0.296	5.742	6.588	6.801	103737	Pex12	chr11	-	NM_1340	P135	protein_cc	AI451906	peroxisom	Pex12
3365	Ap2b1	1.188	0.963	1.144	0.009	0.450	0.122	7.823	8.071	8.017	71770	Ap2b1	chr11	+	NM_0010	P14630	protein_cc	1300012O	adaptor-re	Ap2b1
3366	Rasl10b	5.331	6.226	33.188	0.044	0.130	0.309	0.002	2.417	5.055	276952	Rasl10b	chr11	+	NM_0010	P11218	protein_cc	B230331P	RAS-like, f	Rasl10b
3367	Gas2l2	1.572	0.636	1.000	0.304	1.000	0.423	0.000	0.653	0.000	237891	Gas2l2	chr11	-	NM_0010	P13157	protein_cc	.	growth ar	Gas2l2
3368	1700020L	1.159	0.870	1.008	0.708	0.723	0.985	0.455	0.668	0.467	66330	1700020L	chr11	+	NM_0254	P4838	protein_cc	.	RIKEN cDN	1700020L2
3369	Mmp28	1.000	1.000	1.000	1.000	1.000	1.000	0.000	0.000	0.000	118453	Mmp28	chr11	-	NM_1727	P25457	protein_cc	D130023P	matrix me	Mmp28
3370	Taf15	1.857	0.507	0.942	0.000	0.006	0.849	8.052	8.945	7.966	70439	Taf15	chr11	+	NM_0274	P7314	protein_cc	2610111C	TATA-box	Taf15
3371	Heatr9	1.000	1.000	1.000	1.000	1.000	1.000	0.000	0.000	0.000	629303	Heatr9	chr11	-	NM_0010	P25654	protein_cc	Gm11435	HEAT repe	Heatr9
3372	Ccl5	47.570	0.538	25.611	0.000	0.002	0.032	5.218	10.790	9.896	20304	Ccl5	chr11	-	NM_0136	P11122	protein_cc	MuRantes	chemokin	Ccl5
3373	Ccl9	1.049	0.778	0.816	0.838	0.322	0.348	9.753	9.822	9.460	20308	Ccl9	chr11	-	NM_0113	P9875	protein_cc	CCF18| M	chemokin	Ccl9
3374	E230016K	1.000	1.000	1.000	1.000	1.000	1.000	0.000	0.000	0.000	10050446	E230016K	chr11	+	NR_03645	.	lncRNA	.	RIKEN cDN	E230016K2
3375	Ccl6	0.404	0.831	0.336	0.001	0.342	0.035	7.942	6.635	6.368	20305	Ccl6	chr11	-	NM_0091	P7733	protein_cc	c10| MRP	chemokin	Ccl6
3376	Ccl3	85.443	0.108	9.267	0.003	0.004	0.038	2.032	8.449	5.244	20302	Ccl3	chr11	-	NM_0113	P23651	protein_cc	AI323804|	chemokin	Ccl3
3377	Ccl4	271.792	0.132	35.834	0.004	0.006	0.000	0.003	8.089	5.166	20303	Ccl4	chr11	+	NM_0136	P712	protein_cc	Act-2| AT	chemokin	Ccl4
3378	Wfdc17	4.442	0.435	1.931	0.006	0.017	0.016	8.434	10.585	9.384	10003425	Wfdc17	chr11	+	NM_0010	P2402	protein_cc	Amwap| (	WAP four-	Wfdc17
3379	Wfdc18	3.050	0.668	2.037	0.001	0.418	0.623	2.090	3.699	3.116	14038	Wfdc18	chr11	+	NM_0079	P19861	protein_cc	Expi| Kal1	WAP four-	Wfdc18
3380	Wfdc21	2.220	1.212	2.692	0.000	0.052	0.024	14.059	15.210	15.488	66107	Wfdc21	chr11	+	NM_1832	P7305	protein_cc	1100001G	WAP four-	Wfdc21
3381	Heatr6	1.820	0.596	1.085	0.031	0.064	0.772	7.208	8.072	7.325	217026	Heatr6	chr11	+	NM_1454	P7328	protein_cc	2700008B	HEAT repe	Heatr6
3382	Hnf1b	0.701	1.125	0.789	0.091	0.569	0.129	5.797	5.285	5.455	21410	Hnf1b	chr11	+	NM_0012	P13327	protein_cc	HNF-1-bet	HNF1 hom	Hnf1b
3383	Ddx52	2.062	0.907	1.869	0.000	0.589	0.253	7.477	8.521	8.380	78394	Ddx52	chr11	+	NM_0300	P4306	protein_cc	2700029C	DEAD (Asp	Ddx52
3384	Synrg	1.377	0.887	1.221	0.230	0.610	0.289	7.905	8.367	8.194	217030	Synrg	chr11	+	NM_1943	P3589	protein_cc	Ap1gbp1|	synergin,	Synrg
3385	Dusp14	2.970	0.679	2.016	0.014	0.393	0.568	4.369	5.940	5.381	56405	Dusp14	chr11	-	NM_0198	P1867	protein_cc	1110014C	dual speci	Dusp14
3386	Tada2a	1.417	0.970	1.375	0.215	0.729	0.523	6.060	6.562	6.519	217031	Tada2a	chr11	-	NM_1725	P24491	protein_cc	AV319371	transcripti	Tada2a
3387	Gm11437	0.347	1.696	0.589	0.026	0.520	0.474	3.054	1.528	2.291	628813	Gm11437	chr11	-	NM_0010	P15358	protein_cc	AI463690|	predicted	Gm11437
3388	Acaca	0.200	1.933	0.387	0.000	0.001	0.011	8.679	6.360	7.311	107476	Acaca	chr11	+	NM_1333	P26221	protein_cc	A530025K	acetyl-Coe	Acaca
3389	Aatf	0.927	1.038	0.963	0.701	0.837	0.793	6.269	6.160	6.214	56321	Aatf	chr11	-	NM_0198	P1867	protein_cc	4933415H	apoptosis	Aatf
3390	Lhx1	2.722	0.367	1.000	0.306	1.000	0.423	0.001	1.445	0.000	16869	Lhx1	chr11	-	NM_0084	P5452	protein_cc	Lim1	LIM home	Lhx1
3391	Lhx1os	0.716	0.995	0.712	0.182	1.000	0.423	0.490	0.008	0.000	78365	Lhx1os	chr11	+	NR_03805	.	lncRNA	1500016L	LIM home	Lhx1os
3392	1700109G	1.000	1.000	1.000	1.000	1.000	1.000	0.000	0.000	0.000	78471	1700109G	chr11	-	NR_04619	.	lncRNA	.	RIKEN cDN	1700109G
3393	Mrm1	0.724	1.222	0.884	0.474	0.599	0.768	4.931	4.464	4.753	217038	Mrm1	chr11	-	NM_1454	P2994	protein_cc	A530065E	mitochon	Mrm1
3394	Dhrs11	0.354	1.261	0.447	0.002	0.479	0.154	6.437	4.940	5.275	192970	Dhrs11	chr11	-	NM_1775	P3549	protein_cc	.	dehydrog	Dhrs11
3395	4930502E	0.339	0.981	0.333	0.181	1.000	0.423	1.588	0.028	0.000	74995	4930502E	chr11	+	NR_04628	.	lncRNA	.	RIKEN cDN	4930502E
3396	Ggnbp2	0.821	1.033	0.848	0.002	0.804	0.443	11.423	11.139	11.185	217039	Ggnbp2	chr11	-	NM_1531	P22880	protein_cc	AI451896|	gametoge	Ggnbp2
3397	Ggnbp2os	0.485	0.957	0.464	0.014	0.888	0.074	4.943	3.898	3.835	10263556	Ggnbp2os	chr11	+	NR_13120	.	lncRNA	.	gametoge	Ggnbp2os
3398	Pigw	1.353	0.869	1.176	0.068	0.648	0.796	5.386	5.822	5.619	70325	Pigw	chr11	-	NM_0273	P13369	protein_cc	2610044A	phosphati	Pigw
3399	Myo19	2.555	0.801	2.048	0.011	0.309	0.148	5.873	7.227	6.907	66196	Myo19	chr11	+	NM_0254	P18200	protein_cc	1110055A	myosin XI	Myo19
3400	Znhit3	2.346	0.830	1.946	0.002	0.388	0.263	6.328	7.558	7.289	244850	Znhit3	chr11	-	NM_0010	P10751	protein_cc	Myohd1|	zinc finger	Znhit3
3401	Car4	896.251	0.481	431.422	0.002	0.029	0.058	0.003	9.811	8.756	12351	Car4	chr11	+	NM_0076	P18754	protein_cc	AW45671	carbonic a	Car4
3402	Usp32	0.963	0.814	0.784	0.699	0.108	0.023	7.394	7.340	7.042	237898	Usp32	chr11	-	NM_0010	P8037	protein_cc	2900074J	ubiquitin s	Usp32
3403	1700125H	1.000	1.000	1.000	1.000	1.000	1.000	0.000	0.000	0.000	73634	1700125H	chr11	+	NM_0285	P4527	protein_cc	.	RIKEN cDN	1700125H
3404	Appbp2	1.648	0.746	1.229	0.008	0.146	0.556	6.057	6.777	6.355	66884	Appbp2	chr11	-	NM_0258	P10622	protein_cc	1300003O	amyloid be	Appbp2
3405	Appbp2os	0.588	0.405	0.238	0.170	0.033	0.222	3.914	3.148	1.844	654810	Appbp2os	chr11	+	NR_02832	.	lncRNA	D630032N	amyloid be	Appbp2os
3406	Ppm1d	2.141	0.798	1.708	0.001	0.014	0.054	5.483	6.581	6.256	53892	Ppm1d	chr11	+	NM_0169	P13899	protein_cc	AV338790	protein ph	Ppm1d
3407	Bcas3	0.381	1.449	0.552	0.002	0.001	0.087	7.054	5.662	6.197	192197	Bcas3	chr11	+	NM_1386	P16530	protein_cc	1500019F	breast car	Bcas3
3408	Bcas3os1	1.146	0.632	0.725	0.729	1.000	0.422	0.465	0.661	0.000	71489	Bcas3os1	chr11	-	NR_04587	.	lncRNA	8430403D	breast car	Bcas3os1
3409	Bcas3os2	1.000	1.000	1.000	1.000	1.000	1.000	0.000	0.000	0.000	78420	Bcas3os2	chr11	-	NR_04619	.	lncRNA	9530048J	breast car	Bcas3os2
3410	2610027K	0.338	1.356	0.459	0.011	0.546	0.242	5.141	3.578	4.017	69909	2610027K	chr11	-	NR_07705	.	lncRNA	.	RIKEN cDN	2610027K
3411	Tbx2	0.503	1.207	0.607	0.211	0.791	0.448	4.502	3.510	3.781	21385	Tbx2	chr11	+	NM_0093	P21379	protein_cc	.	T-box 2	Tbx2
3412	Tbx4	1.010	1.885	1.904	0.000	0.182	0.423	0.000	0.015	0.929	21387	Tbx4	chr11	+	NM_0115	P16692	protein_cc	3930401C	T-box 4	Tbx4
3413	Gm20759	1.000	1.000	1.000	1.000	1.000	1.000	0.000	0.000	0.000	629393	Gm20759	chr11	-	NR_04603	.	lncRNA	.	predicted	Gm20759
3414	Brip1	1.160	0.566	0.657	0.402	0.000	0.331	6.293	6.507	5.686	237911	Brip1	chr11	-	NM_1783	P5557	protein_cc	3110009N	BRCA1 int	Brip1
3415	Brip1os	0.750	2.574	1.932	0.230	0.089	0.380	4.969	4.555	5.919	74038	Brip1os	chr11	+	NR_13095	.	lncRNA	4632419I2	BRCA1 int	Brip1os
3416	Ints2	1.794	0.639	1.146	0.007	0.013	0.417	5.989	6.832	6.185	70422	Ints2	chr11	-	NM_0274	P469	protein_cc	2810417D	integrator	Ints2
3417	Med13	1.395	0.954	1.330	0.049	0.475	0.322	7.862	8.343	8.274	327987	Med13	chr11	-	NM_0010	P14750	protein_cc	1110067M	mediator	Med13
3418	Rnft1	0.965	1.126	1.086	0.777	0.338	0.277	7.157	7.105	7.276	76892	Rnft1	chr11	+	NM_0297	P4343	protein_cc	0610013E	ring finger	Rnft1
3419	Rps6kb1	1.429	1.177	1.682	0.001	0.084	0.066	8.657	9.172	9.407	72508	Rps6kb1	chr11	-	NM_0011	P6984	protein_cc	2610318I1	ribosomal	Rps6kb1
3420	Tubd1	2.449	0.301	0.737	0.018	0.011	0.315	3.748	5.040	3.307	56427	Tubd1	chr11	+	NM_0197	P1895	protein_cc	4930550G	tubulin, de	Tubd1
3421	Vmp1	1.828	0.887	1.621	0.009	0.325	0.073	10.526	11.396	11.223	75909	Vmp1	chr11	-	NM_0294	P7415	protein_cc	3110098I	vacuole m	Vmp1
3422	Ptrh2	1.214	1.647	1.999	0.237	0.008	0.005	7.020	7.300	8.020	217057	Ptrh2	chr11	+	NM_1750	P1800	protein_cc	A230072I	peptidyl-t	Ptrh2
3423	Cltc	1.120	1.004	1.125	0.005	0.955	0.431	10.854	11.018	11.024	67300	Cltc	chr11	-	NM_0010	P14382	protein_cc	3110065L	clathrin, h	Cltc
3424	Dhx40	1.320	1.123	1.483	0.000	0.334	0.181	8.500	8.901	9.068	67487	Dhx40	chr11	-	NM_0261	P20323	protein_cc	2410016C	DEAH (Asp	Dhx40
3425	Ypel2	0.564	0.570	0.322	0.003	0.016	0.014	8.016	7.191	6.379	77864	Ypel2	chr11	+	NM_0010	P16286	protein_cc	6430570G	yippee-like	Ypel2
3426	Gdpd1	12.340	0.959	11.835	0.002	0.791	0.017	2.763	6.388	6.328	66569	Gdpd1	chr11	-	NM_0256	P341	protein_cc	1200020H	glycerophe	Gdpd1
3427	Smg8	2.954	0.680	2.008	0.026	0.161	0.045	4.946	6.509	5.952	74133	Smg8	chr11	-	NM_0242	P20078	protein_cc	1200011M	smg-8 hor	Smg8
3428	Prr11	1.385	0.355	0.492	0.586	0.214	0.325	2.336	2.806	1.313	270906	Prr11	chr11	-	NM_1755	P2135	protein_cc	B930067F	proline ric	Prr11
3429	Ska2	2.544	0.734	1.867	0.002	0.510	0.622	2.213	3.560	3.114	66140	Ska2	chr11	+	NM_0253	P2503	protein_cc	1110001A	spindle an	Ska2
3430	Trim37	1.042	0.972	1.013	0.857	0.924	0.971	6.843	6.902	6.861	68729	Trim37	chr11	+	NM_1979	P21054	protein_cc	1110032A	tripartite	Trim37
3431	Ppm1e	3.371	0.324	1.092	0.002	0.004	0.918	1.038	2.791	1.165	320472	Ppm1e	chr11	-	NM_1771	P9645	protein_cc	AW049260	protein ph	Ppm1e
3432	Rad51c	2.282	0.190	0.433	0.017	0.000	0.350	2.503	3.693	1.294	114714	Rad51c	chr11	-	NM_0532	P9740	protein_cc	R51H3| Ra	RAD51 pa	Rad51c
3433	Tex14	0.894	2.629	2.350	0.727	0.016	0.079	0.872	0.710	2.104	83560	Tex14	chr11	+	NM_0313	P3910	protein_cc	C85585	testis exp	Tex14
3434	Sept4	0.122	1.012	0.123	0.002	0.968	0.045	7.062	4.021	4.039	18952	Sept4	chr11	+	NM_0012	P8854	protein_cc	ARTS| Bh	septin 4	Sept4
3435	Mtmr4	0.736	1.051	0.773	0.120	0.771	0.303	9.364	8.921	8.993	170749	Mtmr4	chr11	+	NM_1332	P12356	protein_cc	AA596759	myotubula	Mtmr4
3436	Hsf5	0.253	2.416	0.612	0.001	0.287	0.494	2.720	0.739	2.012	327992	Hsf5	chr11	+	NM_0010	P26044	protein_cc	Gm739| N	heat shoc	Hsf5
3437	Rnf43	0.108	1.952	0.211	0.000	0.008	0.003	6.390	3.178	4.143	207742	Rnf43	chr11	+	NM_1724	P24293	protein_cc	4732452J1	ring finger	Rnf43
3438	1110028F	0.290	0.663	0.193	0.021	0.616	0.029	4.742	2.958	2.366	68690	1110028F	chr11	+	NR_04513	.	lncRNA	.	RIKEN cDN	1110028F1
3439	Supt4a	1.424	1.055	1.501	0.014	0.538	0.049	7.912	8.422	8.498	20922	Supt4a	chr11	+	NM_0092	P10847	protein_cc	AL022777	suppresso	Supt4a
3440	Bzrap1	0.669	0.995	0.666	0.181	1.000	0.423	0.587	0.008	0.000	207777	Bzrap1	chr11	+	NM_1724	P7146	protein_cc	D230016K	benzodiaz	Bzrap1
3441	Mpo	9.292	0.267	2.482	0.053	0.090	0.006	0.002	3.218	1.313	17523	Mpo	chr11	+	NM_0108	P25395	protein_cc	mKIAA403	myeloper	Mpo
3442	Lpo	1.000	1.000	1.000	1.000	1.000	1.000	0.000	0.000	0.000	76113	Lpo	chr11	-	NM_0804	P1117	protein_cc	5830499B	lactoperox	Lpo
3443	Mks1	0.782	2.073	1.620	0.615	0.003	0.463	1.536	1.181	2.233	380718	Mks1	chr11	+	NM_0010	P1204	protein_cc	AK190930	Meckel sy	Mks1
3444	Epx	1.000	1.000	1.000	1.000	1.000	1.000	0.000	0.000	0.000	13861	Epx	chr11	-	NM_0079	P3021	protein_cc	EPO	eosinophi	Epx
3445	Olfr462	1.000	1.000	1.000	1.000	1.000	1.000	0.000	0.000	0.000	258406	Olfr462	chr11	-	NM_1464	P13086	protein_cc	MOR240-	olfactory	Olfr462

3446	Olfr463	1.000	1.000	1.000	1.000	1.000	1.000	0.000	0.000	0.000	258408	Olfr463	chr11	-	NM_1464	P23316	protein_cc	MOR240-1	olfactory r	Olfr463
3447	Olfr464	1.000	1.000	1.000	1.000	1.000	1.000	0.000	0.000	0.000	258407	Olfr464	chr11	-	NM_1464	P21454	protein_cc	MOR240-2	olfactory r	Olfr464
3448	Dynll2	0.216	1.263	0.273	0.001	0.100	0.034	11.654	9.446	9.782	68097	Dynll2	chr11	-	NM_0265	P24067	protein_cc	1700064A	dynein ligl	Dynll2
3449	Srsf1	2.325	0.608	1.413	0.007	0.025	0.105	9.183	10.401	9.682	110809	Srsf1	chr11	+	NM_1733	P17689	protein_cc	1110054N	serine/arg	Srsf1
3450	Vezf1	1.262	0.751	0.948	0.024	0.016	0.521	7.608	7.944	7.531	22344	Vezf1	chr11	+	NM_0166	P15647	protein_cc	AI848691	vascular e	Vezf1
3451	2210416O	1.000	1.000	1.000	1.000	1.000	1.000	0.000	0.000	0.000	70393	2210416O	chr11	+	NR_04549	.	lncRNA	.	RIKEN cDN	2210416O
3452	Cuedc1	0.676	1.116	0.754	0.179	0.783	0.602	4.069	3.504	3.662	103841	Cuedc1	chr11	+	NM_1980	P236	protein_cc	AI841487	CUE doma	Cuedc1
3453	Mrps23	1.330	1.347	1.792	0.000	0.000	0.003	8.213	8.624	9.054	64656	Mrps23	chr11	+	NM_0012	P4590	protein_cc	D11Bwg1	mitochond	Mrps23
3454	1700106J1	1.000	1.000	1.000	1.000	1.000	1.000	0.000	0.000	0.000	.	1700106J1	chr11	-	NM_0288	P13130	protein_cc	.	.	1700106J1
3455	Msi2	0.912	1.663	1.518	0.245	0.002	0.038	7.843	7.711	8.445	76626	Msi2	chr11	-	NM_0540	P2663	protein_cc	1700105C	musashi R	Msi2
3456	0610039H	2.455	0.305	0.748	0.375	1.000	0.421	0.419	1.714	0.000	68397	0610039H	chr11	+	NR_13102	.	lncRNA	.	RIKEN cDN	0610039H
3457	C030037D	0.674	0.375	0.253	0.352	0.245	0.002	3.493	2.924	1.508	193280	C030037D	chr11	+	NR_03805	.	lncRNA	9330160N	RIKEN cDN	C030037D
3458	Akap1	0.742	0.886	0.657	0.093	0.472	0.050	8.781	8.350	8.175	11640	Akap1	chr11	-	NM_0010	P25987	protein_cc	Akap| AKA	A kinase (	Akap1
3459	4930556N	0.716	0.995	0.712	0.182	1.000	0.423	0.490	0.008	0.000	75285	4930556N	chr11	+	NR_12643	.	lncRNA	.	RIKEN cDN	4930556N
3460	Scpep1os	1.606	1.455	2.338	0.297	0.490	0.423	0.000	0.684	1.226	73921	Scpep1os	chr11	+	NR_04595	.	lncRNA	4930405P	serine car	Scpep1os
3461	Scpep1	0.624	1.192	0.744	0.015	0.263	0.033	9.607	8.927	9.180	74617	Scpep1	chr11	-	NM_0290	P23392	protein_cc	2410018F	serine car	Scpep1
3462	Gm15698	1.000	1.000	1.000	1.000	1.000	1.000	0.000	0.000	0.000	217066	Gm15698	chr11	-	NR_00356	.	misc_RNA	OTTMUSG	predicted	Gm15698
3463	2210409E	1.000	1.000	1.000	1.000	1.000	1.000	0.000	0.000	0.000	72381	2210409E	chr11	+	NR_00349	.	misc_RNA	AU021711	RIKEN cDN	2210409E1
3464	Coil	1.185	0.794	0.941	0.104	0.135	0.806	5.652	5.897	5.564	12812	Coil	chr11	+	NM_0167	P5167	protein_cc	C79982| C	coilin	Coil
3465	Trim25	1.151	1.055	1.214	0.006	0.376	0.162	8.997	9.200	9.278	217069	Trim25	chr11	+	NM_0095	P21892	protein_cc	AA960166	tripartite r	Trim25
3466	Dgke	3.019	1.168	3.527	0.037	0.560	0.209	2.514	4.108	4.332	56077	Dgke	chr11	-	NM_0195	P5308	protein_cc	C87606| D	diacylglyc	Dgke
3467	Dgkeos	1.146	0.632	0.725	0.729	1.000	0.422	0.465	0.661	0.000	414094	Dgkeos	chr11	+	NR_11033	.	lncRNA	A930013B	diacylglyc	Dgkeos
3468	Gm525	0.716	0.995	0.712	0.182	1.000	0.423	0.490	0.008	0.000	217071	Gm525	chr11	+	NM_0010	P11326	protein_cc	.	predicted	Gm525
3469	Nog	1.000	1.000	1.000	1.000	1.000	1.000	0.000	0.000	0.000	18121	Nog	chr11	-	NM_0087	P14745	protein_cc	.	noggin	Nog
3470	4932411E	0.552	0.990	0.547	0.182	1.000	0.423	0.871	0.015	0.000	.	4932411E	chr11	-	NM_1725	P16717	.	.	.	4932411E2
3471	Ankfn1	1.000	1.000	1.000	1.000	1.000	1.000	0.000	0.000	0.000	382543	Ankfn1	chr11	-	NM_0010	P14925	protein_cc	4932411E	ankyrin-re	Ankfn1
3472	Pctp	0.035	3.958	0.138	0.000	0.004	0.003	9.992	5.150	7.135	18559	Pctp	chr11	-	NM_0087	P8468	protein_cc	PC-TP| Sta	phosphati	Pctp
3473	Tmem100	0.225	14.882	3.352	0.053	0.015	0.214	3.394	1.243	5.139	67888	Tmem100	chr11	+	NM_0264	P3243	protein_cc	1810057C	transmem	Tmem100
3474	Mmd	0.069	1.809	0.125	0.000	0.095	0.006	10.777	6.924	7.780	67468	Mmd	chr11	+	NM_0261	P14113	protein_cc	1200017E	monocyte	Mmd
3475	Hlf	0.146	2.522	0.368	0.000	0.004	0.013	9.477	6.699	8.034	217082	Hlf	chr11	-	NM_1725	P14552	protein_cc	E230015K	hepatic le	Hlf
3476	Stxbp4	0.516	0.457	0.236	0.065	0.112	0.123	4.197	3.243	2.113	20913	Stxbp4	chr11	-	NM_0115	P11343	protein_cc	6030470N	syntaxin b	Stxbp4
3477	Cox11	1.152	0.781	0.900	0.400	0.062	0.760	7.542	7.747	7.391	69802	Cox11	chr11	+	NM_1990	P3966	protein_cc	2010004IC	cytochron	Cox11
3478	Tom1l1	0.885	0.958	0.848	0.602	0.803	0.674	6.607	6.431	6.369	71943	Tom1l1	chr11	-	NM_0280	P16585	protein_cc	2310045L	target of r	Tom1l1
3479	4930405D	1.000	1.000	1.000	1.000	1.000	1.000	0.000	0.000	0.000	73922	4930405D	chr11	+	NR_04595	.	lncRNA	.	RIKEN cDN	4930405D
3480	Kif2b	1.000	1.000	1.000	1.000	1.000	1.000	0.000	0.000	0.000	73470	Kif2b	chr11	-	NM_0285	P17328	protein_cc	1700063D	kinesin fa	Kif2b
3481	Car10	0.716	0.995	0.712	0.182	1.000	0.423	0.490	0.008	0.000	72605	Car10	chr11	+	NM_0282	P15337	protein_cc	2700029L	carbonic a	Car10
3482	Utp18	1.811	0.728	1.319	0.048	0.190	0.438	5.801	6.658	6.200	217109	Utp18	chr11	-	NM_0010	P4859	protein_cc	6230425C	UTP18 sm	Utp18
3483	Mbtd1	0.860	1.110	0.954	0.075	0.510	0.834	8.178	7.959	8.110	103537	Mbtd1	chr11	+	NM_1340	P16140	protein_cc	AA408199	mbt doma	Mbtd1
3484	Nme2	1.429	1.292	1.846	0.022	0.025	0.013	11.767	12.283	12.652	18103	Nme2	chr11	-	NM_0010	P10585	protein_cc	NM23-H2	NME/NM2	Nme2
3485	Nme1	1.545	1.118	1.727	0.001	0.286	0.102	9.750	10.378	10.538	18102	Nme1	chr11	-	NM_0087	P18141	protein_cc	AL024257	NME/NM2	Nme1
3486	Spag9	0.560	1.235	0.692	0.001	0.061	0.058	8.625	7.788	8.093	70834	Spag9	chr11	+	NM_0275	P4046	protein_cc	3110018C	sperm ass	Spag9
3487	Tob1	0.246	1.355	0.334	0.000	0.045	0.015	9.415	7.393	7.831	22057	Tob1	chr11	+	NM_0094	P12858	protein_cc	Tob| Trob	transduce	Tob1
3488	Wfikkn2	0.507	1.370	0.694	0.183	0.191	0.627	1.002	0.021	0.476	278507	Wfikkn2	chr11	-	NM_1818	P12798	protein_cc	2610304F	WAP, folli	Wfikkn2
3489	Luc7l3	1.474	0.684	1.007	0.000	0.000	0.749	10.119	10.678	10.129	67684	Luc7l3	chr11	-	NM_0263	P2014	protein_cc	3300001P	LUC7-like	Luc7l3
3490	Ankrd40	0.537	1.512	0.812	0.009	0.074	0.461	8.987	8.090	8.686	71452	Ankrd40	chr11	+	NM_1460	P24438	protein_cc	1110011C	ankyrin re	Ankrd40
3491	Abcc3	0.927	0.781	0.724	0.403	0.285	0.410	9.121	9.011	8.654	76408	Abcc3	chr11	-	NM_0296	P24888	protein_cc	1700019L	ATP-bindi	Abcc3
3492	Cacna1g	1.005	1.405	1.412	0.000	0.182	0.423	0.000	0.008	0.498	12291	Cacna1g	chr11	-	NM_0011	P8600	protein_cc	a1G| alph	calcium ch	Cacna1g
3493	Spata20	1.000	1.000	1.000	1.000	1.000	1.000	0.000	0.000	0.000	217116	Spata20	chr11	-	NM_1448	P15713	protein_cc	Tisp78	spermatog	Spata20
3494	Epn3	0.439	0.985	0.432	0.002	1.000	0.051	1.210	0.021	0.000	71889	Epn3	chr11	-	NM_0279	P19272	protein_cc	2310022G	epsin 3	Epn3
3495	Mycbpap	1.000	1.000	1.000	1.000	1.000	1.000	0.000	0.000	0.000	104601	Mycbpap	chr11	-	NM_1706	P18758	protein_cc	4932408B	MYCBP as	Mycbpap
3496	Rsad1	0.595	1.920	1.143	0.191	0.001	0.773	5.600	4.852	5.793	237926	Rsad1	chr11	-	NM_0010	P7304	protein_cc	B430319G	radical S-a	Rsad1
3497	Acsf2	0.233	2.217	0.517	0.002	0.053	0.127	7.506	5.405	6.553	264895	Acsf2	chr11	-	NM_1538	P6966	protein_cc	.	acyl-CoA s	Acsf2
3498	Chad	1.000	1.000	1.000	1.000	1.000	1.000	0.000	0.000	0.000	12643	Chad	chr11	+	NM_0076	P8729	protein_cc	SLRR4A	chondroa	Chad
3499	Lrrc59	4.801	0.797	3.829	0.000	0.156	0.066	8.312	10.576	10.249	98238	Lrrc59	chr11	+	NM_1338	P20691	protein_cc	AA959742	leucine ric	Lrrc59
3500	Eme1	4.472	0.821	3.671	0.000	0.199	0.067	7.935	10.096	9.811	268465	Eme1	chr11	-	NM_1777	P1307	protein_cc	6820428D	essential r	Eme1
3501	Mrpl27	0.867	1.566	1.357	0.562	0.150	0.371	9.655	9.449	10.096	94064	Mrpl27	chr11	+	NM_0531	P14984	protein_cc	A630055F	mitochond	Mrpl27
3502	Xylt2	0.402	1.256	0.506	0.000	0.458	0.158	6.810	5.497	5.826	217119	Xylt2	chr11	-	NM_1458	P15762	protein_cc	E030002B	xylosyltra	Xylt2
3503	Gm11541	1.000	1.000	1.000	1.000	1.000	1.000	0.000	0.000	0.000	432589	Gm11541	chr11	-	NM_0010	P7392	protein_cc	OTTMUSG	predicted	Gm11541
3504	Gm11545	0.154	1.744	0.269	0.143	0.201	0.443	2.773	0.075	0.877	217122	Gm11545	chr11	-	NM_0011	P9262	protein_cc	A430060F	predicted	Gm11545
3505	Tmem92	0.669	0.995	0.666	0.181	1.000	0.423	0.587	0.008	0.000	544806	Tmem92	chr11	-	NM_0010	P14268	protein_cc	AU01643C	transmem	Tmem92
3506	Gm11544	1.000	1.000	1.000	1.000	1.000	1.000	0.000	0.000	0.000	432591	Gm11544	chr11	-	NM_0012	P23832	protein_cc	OTTMUSG	predicted	Gm11544
3507	Col1a1	0.247	2.961	0.731	0.001	0.002	0.162	7.422	5.404	6.970	12842	Col1a1	chr11	+	NM_0077	P1834	protein_cc	Col1a-1| C	collagen, t	Col1a1
3508	Sgca	1.000	1.000	1.000	1.000	1.000	1.000	0.000	0.000	0.000	20391	Sgca	chr11	-	NM_0091	P1916	protein_cc	50DAG| A	sarcoglyca	Sgca
3509	Hils1	1.000	1.000	1.000	1.000	1.000	1.000	0.000	0.000	0.000	54388	Hils1	chr11	+	NM_0187	P24847	protein_cc	H1.9| TISF	histone H1	Hils1
3510	Ppp1r9b	2.967	0.422	1.253	0.009	0.012	0.700	4.739	6.308	5.064	217124	Ppp1r9b	chr11	+	NM_1722	P9866	protein_cc	SPL| Spn	protein ph	Ppp1r9b
3511	Samd14	0.600	0.625	0.375	0.473	0.567	0.401	2.511	1.773	1.094	217125	Samd14	chr11	+	NM_1460	P8388	protein_cc	AI839049	sterile alp	Samd14
3512	Pdk2	0.028	3.316	0.092	0.000	0.008	0.000	9.859	4.690	6.420	18604	Pdk2	chr11	-	NM_1336	P1510	protein_cc	.	pyruvate c	Pdk2
3513	Itga3	1.227	1.321	1.620	0.444	0.340	0.479	4.992	5.286	5.688	16400	Itga3	chr11	-	NM_0013	P17812	protein_cc	AA407068	integrin al	Itga3
3514	Dlx3	2.149	0.465	1.000	0.306	1.000	0.423	0.000	1.104	0.000	13393	Dlx3	chr11	+	NM_0100	P1173	protein_cc	AV237891	distal-less	Dlx3
3515	Dlx4	1.000	1.000	1.000	1.000	1.000	1.000	0.000	0.000	0.000	13394	Dlx4	chr11	-	NM_0078	P16425	protein_cc	Dlx-4| Dlx	distal-less	Dlx4
3516	A730090H	0.726	1.387	1.007	0.187	0.187	0.988	0.477	0.015	0.487	.	A730090H	chr11	+	NR_04027	.	.	.	.	A730090H
3517	Tac4	1.005	1.405	1.412	0.000	0.182	0.423	0.000	0.008	0.498	93670	Tac4	chr11	+	NM_0530	P7790	protein_cc	AW48937	tachykinin	Tac4
3518	Kat7	1.063	1.415	1.504	0.528	0.200	0.412	7.597	7.685	8.185	217127	Kat7	chr11	-	NM_1776	P26600	protein_cc	Hbo1| Hb	K(lysine) a	Kat7
3519	Fam117a	0.452	1.541	0.696	0.002	0.510	0.643	5.542	4.395	5.018	215512	Fam117a	chr11	+	NM_1725	P12890	protein_cc	5730593F	family wit	Fam117a
3520	Slc35b1	1.761	1.111	1.957	0.000	0.092	0.018	9.740	10.557	10.708	110172	Slc35b1	chr11	+	NM_0167	P2259	protein_cc	Ugalt2	solute car	Slc35b1
3521	Spop	0.660	0.900	0.595	0.024	0.539	0.102	10.053	9.455	9.303	20747	Spop	chr11	+	NM_0252	P17215	protein_cc	AI315626	speckle-ty	Spop
3522	Nxph3	1.010	1.885	1.904	0.000	0.182	0.423	0.000	0.015	0.929	104079	Nxph3	chr11	-	NM_1308	P1721	protein_cc	.	neurexoph	Nxph3
3523	Ngfr	0.238	0.451	0.107	0.027	0.005	0.133	5.978	3.906	2.756	18053	Ngfr	chr11	-	NM_0332	P22076	protein_cc	LNGFR| p	nerve grov	Ngfr
3524	Phb	0.635	1.225	0.778	0.000	0.004	0.018	9.596	8.941	9.234	18673	Phb	chr11	+	NM_0088	P12387	protein_cc	Bap32	prohibitin	Phb
3525	Zfp652os	0.517	0.730	0.378	0.033	0.397	0.009	2.735	1.784	1.330	432396	Zfp652os	chr11	-	NR_04578	.	lncRNA	1110035M	zinc finger	Zfp652os
3526	B130006D	1.770	0.239	0.422	0.249	0.061	0.290	2.309	3.133	1.065	320616	B130006D	chr11	+	NR_02826	.	lncRNA	.	RIKEN cDN	B130006D
3527	Zfp652	0.859	0.973	0.836	0.035	0.506	0.089	6.294	6.075	6.035	268469	Zfp652	chr11	+	NM_2016	P4923	protein_cc	9530033F	zinc finger	Zfp652
3528	Phospho1	0.292	2.299	0.672	0.009	0.178	0.398	4.200	2.424	3.626	237928	Phospho1	chr11	+	NM_1531	P16454	protein_cc	D11Moh3	phosphata	Phospho1
3529	Abi3	0.921	0.903	0.832	0.394	0.503	0.553	6.871	6.753	6.606	66610	Abi3	chr11	-	NM_0011	P20733	protein_cc	2210414K	ABI gene f	Abi3
3530	Gngt2	1.545	1.099	1.699	0.000	0.462	0.157	6.857	7.484	7.621	14710	Gngt2	chr11	+	NM_0012	P2025	protein_cc	AV096488	guanine n	Gngt2
3531	4833417C	0.976	1.189	1.161	0.975	0.798	0.759	2.423	2.388	2.638	73906	4833417C	chr11	+	NR_04518	.	lncRNA	.	RIKEN cDN	4833417C
3532	B4galnt2	1.714	1.877	3.218	0.383	0.148	0.085	2.460	3.238	4.146	14422	B4galnt2	chr11	-	NM_0080	P8483	protein_cc	AI593864	beta-1,4-N	B4galnt2
3533	Igf2bp1	3.119	2.700	8.419	0.201	0.093	0.150	0.589	2.230	3.663	140486	Igf2bp1	chr11	-	NM_0099	P24353	protein_cc	AL024068	insulin-lik	Igf2bp1
3534	Gip	1.000	1.000	1.000	1.000	1.000	1.000	0.000	0.000	0.000	14607	Gip	chr11	+	NM_0081	P1632	protein_cc	.	gastric inh	Gip
3535	Snf8	1.061	1.024	1.087	0.280	0.666	0.227	8.633	8.719	8.753	27681	Snf8	chr11	+	NM_0335	P10824	protein_cc	D11Moh3	SNF8, ESC	Snf8
3536	Ube2z	1.620	0.800	1.296	0.000	0.014	0.129	8.707	9.403	9.081	268470	Ube2z	chr11	-	NM_1723	P10021	protein_cc	AW04919	ubiquitin-	Ube2z

3537	Atp5g1	0.902	0.946	0.854	0.236	0.621	0.260	10.939	10.791	10.711	11951	Atp5g1	chr11	-	NM_0075	P783	protein_cc	.	ATP synth	Atp5g1
3538	Calcoco2	1.000	1.000	1.000	1.000	1.000	1.000	0.000	0.000	0.000	76815	Calcoco2	chr11	-	NM_0012	P11855	protein_cc	2410154J1	calcium bi	Calcoco2
3539	Ttll6	1.000	1.000	1.000	1.000	1.000	1.000	0.000	0.000	0.000	237930	Ttll6	chr11	+	NM_1727	P4241	protein_cc	4932418K	tubulin ty	Ttll6
3540	Hoxb13	1.000	1.000	1.000	1.000	1.000	1.000	0.000	0.000	0.000	15408	Hoxb13	chr11	+	NM_0082	P6514	protein_cc	.	homeobox	Hoxb13
3541	Gm11538	1.000	1.000	1.000	1.000	1.000	1.000	0.000	0.000	0.000	432593	Gm11538	chr11	+	NR_10802	.	lncRNA	OTTMUSG	predicted	Gm11538
3542	Gm53	1.000	1.000	1.000	1.000	1.000	1.000	0.000	0.000	0.000	193022	Gm53	chr11	+	NR_03797	.	lncRNA	.	predicted	Gm53
3543	Hoxb9	1.000	1.000	1.000	1.000	1.000	1.000	0.000	0.000	0.000	15417	Hoxb9	chr11	+	NM_0082	P24407	protein_cc	Hox-2.5	homeobox	Hoxb9
3544	Hoxb8	1.005	1.405	1.412	0.000	0.182	0.423	0.000	0.008	0.498	15416	Hoxb8	chr11	+	NM_0104	P2502	protein_cc	Hox-2.4	homeobox	Hoxb8
3545	Hoxb7	5.343	0.303	1.619	0.050	0.087	0.553	0.372	2.790	1.068	15415	Hoxb7	chr11	+	NM_0104	P4666	protein_cc	AI325018		homeobox	Hoxb7
3546	Hoxb5os	1.000	1.000	1.000	1.000	1.000	1.000	0.000	0.000	0.000	75395	Hoxb5os	chr11	-	NR_13175	.	lncRNA	0610040B	homeobox	Hoxb5os
3547	Hoxb6	0.750	2.739	2.055	0.200	0.182	0.516	0.448	0.033	1.487	15414	Hoxb6	chr11	+	NM_0082	P16526	protein_cc	Hox-2.2	homeobox	Hoxb6
3548	Hoxb5	0.230	2.320	0.535	0.009	0.406	0.425	4.672	2.554	3.769	15413	Hoxb5	chr11	+	NM_0082	P20050	protein_cc	AI385717		homeobox	Hoxb5
3549	Hoxb4	0.505	1.588	0.802	0.230	0.412	0.497	4.755	3.769	4.436	15412	Hoxb4	chr11	+	NM_0104	P20293	protein_cc	Hox-2.6	homeobox	Hoxb4
3550	Hoxb3	0.975	1.588	1.548	0.928	0.147	0.230	3.377	3.341	4.008	15410	Hoxb3	chr11	+	NM_0010	P20125	protein_cc	Hox-2.7	homeobox	Hoxb3
3551	Hoxb2	4.582	0.990	4.537	0.000	0.980	0.330	0.001	2.197	2.183	103889	Hoxb2	chr11	+	NM_1340	P8415	protein_cc	AI894218		homeobox	Hoxb2
3552	Hoxb1	1.000	1.000	1.000	1.000	1.000	1.000	0.000	0.000	0.000	15407	Hoxb1	chr11	+	NM_0082	P2297	protein_cc	Hox-2.9	homeobox	Hoxb1
3553	Gm11529	1.000	1.000	1.000	1.000	1.000	1.000	0.000	0.000	0.000	10003860	Gm11529	chr11	-	NR_03352	.	lncRNA	OTTMUSG	predicted	Gm11529
3554	Skap1	1.567	0.561	0.879	0.177	0.143	0.890	1.724	2.373	1.538	78473	Skap1	chr11	+	NM_0011	P14782	protein_cc	1700091G	src family	Skap1
3555	Snx11	3.019	1.396	4.215	0.002	0.225	0.130	5.602	7.196	7.677	74479	Snx11	chr11	-	NM_0289	P18271	protein_cc	4933439F	sorting ne	Snx11
3556	Cbx1	0.973	0.633	0.615	0.891	0.164	0.245	7.729	7.689	7.028	12412	Cbx1	chr11	+	NM_0076	P6816	protein_cc	Cbx	Cbx-r	chromobo	Cbx1
3557	Gm11517	0.593	1.457	0.865	0.005	0.060	0.542	3.453	2.700	3.243	629750	Gm11517	chr11	-	NR_03352	.	misc_RNA	OTTMUSG	predicted	Gm11517
3558	Nfe2l1	1.136	0.771	0.875	0.103	0.069	0.456	9.179	9.363	8.987	18023	Nfe2l1	chr11	-	NM_0086	P2778	protein_cc	AA408798	nuclear fa	Nfe2l1
3559	Copz2	0.504	1.035	0.522	0.000	0.896	0.153	6.597	5.608	5.658	56358	Copz2	chr11	+	NM_0198	P22607	protein_cc	1110012D	coatomer	Copz2
3560	Cdk5rap3	0.916	1.646	1.508	0.239	0.000	0.005	7.939	7.813	8.532	80280	Cdk5rap3	chr11	-	NM_0013	P9833	protein_cc	1810007E	CDK5 regu	Cdk5rap3
3561	Prr15l	0.574	1.420	0.814	0.069	0.485	0.721	3.626	2.824	3.330	217138	Prr15l	chr11	+	NM_1460	P6834	protein_cc	Atad4	BC	proline ric	Prr15l
3562	Pnpo	0.493	1.220	0.602	0.000	0.325	0.132	10.524	9.504	9.791	103711	Pnpo	chr11	-	NM_1340	P11564	protein_cc	AI415282	pyridoxine	Pnpo
3563	D030028A	3.079	1.365	4.204	0.000	0.450	0.282	2.085	3.708	4.157	319371	D030028A	chr11	+	NR_0329	.	lncRNA		RIKEN cDN	D030028A
3564	Sp2	1.954	0.708	1.383	0.004	0.069	0.312	5.651	6.618	6.119	78912	Sp2	chr11	-	NM_0302	P17669	protein_cc	4930480I1	Sp2 transc	Sp2
3565	Sp6	0.552	0.990	0.547	0.182	1.000	0.423	0.871	0.015	0.000	83395	Sp6	chr11	+	NM_0311	P20081	protein_cc	1110025J	trans-actir	Sp6
3566	Scrn2	0.271	1.553	0.421	0.000	0.001	0.007	7.855	5.970	6.605	217140	Scrn2	chr11	+	NM_1460	P20492	protein_cc	AV001119	secernin 2	Scrn2
3567	Lrrc46	0.440	0.482	0.212	0.112	0.454	0.005	4.241	3.058	2.005	69297	Lrrc46	chr11	-	NM_0270	P7048	protein_cc	1700006D	leucine ric	Lrrc46
3568	Mrpl10	0.959	1.448	1.389	0.810	0.075	0.116	8.846	8.786	9.320	107732	Mrpl10	chr11	+	NM_0261	P9337	protein_cc	0610040E	mitochond	Mrpl10
3569	Osbpl7	0.844	1.305	1.102	0.386	0.046	0.689	4.677	4.433	4.817	71240	Osbpl7	chr11	+	NM_0010	P15038	protein_cc	4933437E	oxysterol	Osbpl7
3570	Tbx21	3.081	0.115	0.356	0.093	1.000	0.422	1.492	3.115	0.000	57765	Tbx21	chr11	-	NM_0195	P10927	protein_cc	Tbet	Tbly	T-box 21	Tbx21
3571	Tbkbp1	1.730	0.629	1.088	0.003	0.035	0.825	5.333	6.123	5.455	73174	Tbkbp1	chr11	-	NM_1981	P10796	protein_cc	3110043L	TBK1 bind	Tbkbp1
3572	Kpnb1	1.498	1.047	1.568	0.010	0.290	0.106	8.779	9.363	9.428	16211	Kpnb1	chr11	-	NM_0083	P21544	protein_cc	AA409963	karyopher	Kpnb1
3573	Npepps	1.220	0.674	0.822	0.205	0.089	0.459	8.498	8.785	8.215	19155	Npepps	chr11	-	NM_0089	P10173	protein_cc	AAP-S	go	aminopep	Npepps
3574	Mrpl45	1.081	0.898	0.971	0.078	0.097	0.786	8.870	8.983	8.829	67036	Mrpl45	chr11	+	NM_0259	P22600	protein_cc	2600005P	mitochond	Mrpl45
3575	Gpr179	1.000	1.000	1.000	1.000	1.000	1.000	0.000	0.000	0.000	217143	Gpr179	chr11	-	NM_0010	P25938	protein_cc	5330439C	G protein-	Gpr179
3576	Socs7	1.219	0.667	0.813	0.016	0.005	0.238	8.049	8.335	7.750	192157	Socs7	chr11	+	NM_1386	P24323	protein_cc	2310063P	suppresso	Socs7
3577	Arhgap23	0.522	1.462	0.762	0.007	0.000	0.259	7.531	6.591	7.139	58996	Arhgap23	chr11	+	NM_0214	P17576	protein_cc	A330041B	Rho GTPas	Arhgap23
3578	4933428G	0.164	0.955	0.156	0.117	1.000	0.344	2.677	0.067	0.000	10014147	4933428G	chr11	-	NM_0012	P14729	protein_cc	.	RIKEN cDN	4933428G
3579	Srcin1	1.371	0.364	0.499	0.668	1.000	0.423	1.003	1.458	0.000	56013	Srcin1	chr11	-	NM_0188	P21640	protein_cc	mKIAA168	SRC kinase	Srcin1
3580	2410003L	1.111	1.172	1.301	0.737	0.003	0.610	1.003	1.155	1.383	69729	2410003L	chr11	+	NR_04549	.	lncRNA		RIKEN cDN	2410003L1
3581	E130012A	1.804	0.683	1.231	0.002	0.156	0.710	4.680	5.531	4.980	103551	E130012A	chr11	-	NM_1753	P16181	protein_cc	AA409164	RIKEN cDN	E130012A
3582	Mllt6	1.398	0.987	1.380	0.220	0.953	0.229	7.004	7.488	7.469	246198	Mllt6	chr11	+	NM_1393	P18220	protein_cc	Af17	AI3	myeloid/ly	Mllt6
3583	Cisd3	0.499	1.058	0.528	0.001	0.722	0.083	8.901	7.898	7.979	217149	Cisd3	chr11	+	NM_0010	P1021	protein_cc	Mel-13	M	CDGSH iro	Cisd3
3584	Pcgf2	0.798	1.884	1.502	0.445	0.043	0.007	4.389	4.062	4.976	22658	Pcgf2	chr11	-	NM_0011	P24804	protein_cc	mel-18	M	polycomb	Pcgf2
3585	Psmb3	1.192	1.224	1.460	0.251	0.088	0.078	9.194	9.448	9.740	26446	Psmb3	chr11	+	NM_0119	P20748	protein_cc	AL033320	proteason	Psmb3
3586	Pip4k2b	0.526	1.230	0.647	0.001	0.131	0.108	7.462	6.536	6.834	108083	Pip4k2b	chr11	-	NM_0540	P4072	protein_cc	AI848124		phosphati	Pip4k2b
3587	Cwc25	1.190	0.831	0.990	0.134	0.114	0.871	7.769	8.020	7.754	67480	Cwc25	chr11	-	NM_0261	P7336	protein_cc	1300013D	CWC25 sp	Cwc25
3588	1700001P	3.391	1.069	3.624	0.000	0.891	0.423	0.001	1.763	1.859	72215	1700001P	chr11	-	NM_0281	P17809	protein_cc	.	RIKEN cDN	1700001P
3589	Rpl23	1.145	1.379	1.578	0.000	0.000	0.002	11.691	11.886	12.349	65019	Rpl23	chr11	-	NM_0228	P8180	protein_cc	2810009A	ribosomal	Rpl23
3590	Snora21	0.788	1.521	1.199	0.170	0.259	0.714	4.464	4.120	4.726	10030249	Snora21	chr11	-	NR_02807	.	snoRNA	MBI-3	small nucl	Snora21
3591	Lasp1	0.508	1.224	0.621	0.000	0.218	0.088	10.750	9.772	10.064	16796	Lasp1	chr11	+	NM_0106	P4922	protein_cc	AA408629	LIM and S	Lasp1
3592	B230217C	10.600	0.895	9.491	0.135	0.882	0.388	0.002	3.408	3.249	68127	B230217C	chr11	+	NM_0010	P123	protein_cc	AI840637	RIKEN cDN	B230217C
3593	Fbxo47	1.590	0.875	1.390	0.302	0.737	0.423	0.000	0.669	0.476	72973	Fbxo47	chr11	-	NM_0010	P13572	protein_cc	2900052P	F-box prot	Fbxo47
3594	Plxdc1	0.269	0.446	0.120	0.009	1.000	0.030	3.057	1.163	0.000	72324	Plxdc1	chr11	-	NM_0011	P4463	protein_cc	2410003I	plexin dor	Plxdc1
3595	Arl5c	8.313	0.286	2.379	0.076	0.130	0.336	1.470	4.525	2.721	217151	Arl5c	chr11	-	NM_2072	P13959	protein_cc	Arl12	BC	ADP-ribos	Arl5c
3596	Cacnb1	0.970	0.851	0.826	0.950	0.786	0.703	1.167	1.124	0.891	12295	Cacnb1	chr11	-	NM_0012	P6813	protein_cc	CAB1	Cch	calcium ch	Cacnb1
3597	Rpl19	1.402	1.246	1.746	0.036	0.065	0.042	9.793	10.280	10.597	19921	Rpl19	chr11	+	NM_0011	P12402	protein_cc	.	ribosomal	Rpl19
3598	Stac2	1.000	1.000	1.000	1.000	1.000	1.000	0.000	0.000	0.000	217154	Stac2	chr11	-	NM_1460	P26773	protein_cc	24b2	24b	SH3 and c	Stac2
3599	Fbxl20	0.524	0.857	0.449	0.000	0.000	0.015	7.945	7.012	6.790	72194	Fbxl20	chr11	-	NM_0281	P17432	protein_cc	2610511F	F-box and	Fbxl20
3600	Med1	0.998	0.729	0.727	0.966	0.007	0.117	9.030	9.026	8.570	19014	Med1	chr11	-	NM_1340	P11279	protein_cc	AI480703		mediator	Med1
3601	Cdk12	2.487	0.775	1.928	0.001	0.245	0.300	7.473	8.787	8.420	69131	Cdk12	chr11	+	NM_0269	P23550	protein_cc	1810022J1	cyclin-dep	Cdk12
3602	Neurod2	1.000	1.000	1.000	1.000	1.000	1.000	0.000	0.000	0.000	18013	Neurod2	chr11	-	NM_0108	P25126	protein_cc	bHLHa1	N	neurogeni	Neurod2
3603	Ppp1r1b	0.210	2.193	0.460	0.001	0.031	0.073	5.909	3.655	4.788	19049	Ppp1r1b	chr11	+	NM_1448	P3271	protein_cc	AU040756	protein ph	Ppp1r1b
3604	1700003D	1.000	1.000	1.000	1.000	1.000	1.000	0.000	0.000	0.000	69338	1700003D	chr11	-	NR_04547	.	lncRNA		RIKEN cDN	1700003D
3605	Stard3	1.057	1.133	1.197	0.526	0.184	0.329	7.059	7.139	7.319	59045	Stard3	chr11	+	NM_0215	P2567	protein_cc	es64	Mln	START dor	Stard3
3606	Tcap	1.000	1.000	1.000	1.000	1.000	1.000	0.000	0.000	0.000	21393	Tcap	chr11	+	NM_0115	P2499	protein_cc	AI503981		titin-cap	Tcap
3607	Pnmt	1.000	1.000	1.000	1.000	1.000	1.000	0.000	0.000	0.000	18948	Pnmt	chr11	+	NM_0088	P5855	protein_cc	Pent	phenyleth	Pnmt
3608	Pgap3	1.175	1.064	1.250	0.305	0.779	0.608	4.195	4.427	4.517	320655	Pgap3	chr11	-	NM_0010	P21533	protein_cc	CAB2	D4	post-GPI a	Pgap3
3609	Erbb2	1.245	1.516	1.888	0.523	0.143	0.016	2.501	2.817	3.417	13866	Erbb2	chr11	+	NM_0010	P26063	protein_cc	c-erbB2	c	erb-b2 rec	Erbb2
3610	Mien1	0.855	1.084	0.926	0.246	0.512	0.688	9.910	9.683	9.799	103742	Mien1	chr11	-	NM_0255	P2590	protein_cc	1810046J1	migration	Mien1
3611	Grb7	0.203	2.100	0.427	0.000	0.003	0.010	10.246	7.947	9.018	14786	Grb7	chr11	+	NM_0103	P15512	protein_cc	mKIAA402	growth fac	Grb7
3612	Ikzf3	0.698	0.800	0.558	0.394	0.709	0.211	1.652	1.132	0.810	22780	Ikzf3	chr11	-	NM_0117	P13380	protein_cc	5830411O	IKAROS fa	Ikzf3
3613	Zpbp2	0.357	4.500	1.606	0.187	0.000	0.458	1.557	0.071	2.241	69376	Zpbp2	chr11	+	NM_0011	P2808	protein_cc	1700017D	zona pellu	Zpbp2
3614	Ormdl3	0.728	0.976	0.711	0.142	0.859	0.274	9.296	8.839	8.804	66612	Ormdl3	chr11	-	NM_0256	P25750	protein_cc	2810011N	ORM1-like	Ormdl3
3615	Gm12	1.000	1.000	1.000	1.000	1.000	1.000	0.000	0.000	0.000	.	Gm12	chr11	+	NM_0011	P2922	.		.	Gm12
3616	Gsdma3	1.000	1.000	1.000	1.000	1.000	1.000	0.000	0.000	0.000	450219	Gsdma3	chr11	+	NM_0010	P8619	protein_cc	Bsk	Dfl	I	gasdermin	Gsdma3
3617	Gsdma2	1.000	1.000	1.000	1.000	1.000	1.000	0.000	0.000	0.000	76758	Gsdma2	chr11	+	NM_0297	P15564	protein_cc	2200001G	gasdermin	Gsdma2
3618	Gsdma	1.000	1.000	1.000	1.000	1.000	1.000	0.000	0.000	0.000	57911	Gsdma	chr11	+	NM_0213	P21030	protein_cc	BB149167	gasdermin	Gsdma
3619	Psmd3	1.095	1.197	1.311	0.610	0.245	0.176	10.672	10.804	11.063	22123	Psmd3	chr11	+	NM_0094	P20830	protein_cc	AI255837		proteason	Psmd3
3620	Csf3	138.577	0.439	60.820	0.000	0.006	0.036	0.003	7.117	5.929	12985	Csf3	chr11	+	NM_0099	P4339	protein_cc	Csfg	G-CS	colony stir	Csf3
3621	Med24	1.589	0.859	1.366	0.061	0.283	0.293	5.054	5.723	5.504	23989	Med24	chr11	+	NM_0118	P9688	protein_cc	911GSE	A	mediator	Med24
3622	Thra	0.186	1.534	0.285	0.001	0.012	0.043	6.652	4.224	4.842	21833	Thra	chr11	+	NM_1780	P2563	protein_cc	6430529J	thyroid ho	Thra
3623	Nr1d1	0.061	2.563	0.157	0.000	0.027	0.002	9.274	5.245	6.603	217166	Nr1d1	chr11	-	NM_1454	P23669	protein_cc	A530070C	nuclear re	Nr1d1
3624	Msl1	0.869	0.813	0.706	0.207	0.144	0.094	9.139	8.936	8.637	74026	Msl1	chr11	+	NM_0287	P2001	protein_cc	2810017F	male-spec	Msl1
3625	Gm12359	0.553	2.137	1.181	0.424	0.128	0.867	2.383	1.528	2.624	10003726	Gm12359	chr11	-	NR_03355	.	lncRNA	OTTMUSG	predicted	Gm12359
3626	Casc3	1.700	0.719	1.222	0.004	0.113	0.587	7.711	8.476	8.000	192160	Casc3	chr11	+	NM_1386	P9041	protein_cc	Btz	Mln5	cancer sus	Casc3
3627	Rapgefl1	1.320	1.017	1.342	0.082	0.961	0.690	1.374	1.775	1.799	268480	Rapgefl1	chr11	+	NM_0010	P15591	protein_cc	B230105J	Rap guani	Rapgefl1

3628	Wipf2	1.311	0.714	0.936	0.001	0.031	0.764	8.141	8.531	8.045	68524	Wipf2	chr11	+	NM_1979	P10228	protein_cc	1110014J(	WAS/WAS	Wipf2
3629	Cdc6	3.063	1.847	5.656	0.346	0.398	0.283	0.372	1.988	2.872	23834	Cdc6	chr11	+	NM_0010	P12787	protein_cc	CDC18L	cell divisio	Cdc6
3630	Rara	0.340	1.606	0.546	0.000	0.135	0.147	6.935	5.378	6.061	19401	Rara	chr11	+	NM_0090	P11757	protein_cc	Nr1b1 | RA	retinoic ac	Rara
3631	Gjd3	1.000	1.000	1.000	1.000	1.000	1.000	0.000	0.000	0.000	353155	Gjd3	chr11	-	NM_1785	P11609	protein_cc	cx30.2 | Gj	gap juncti	Gjd3
3632	Top2a	2.557	0.788	2.016	0.012	0.125	0.112	4.116	5.470	5.127	21973	Top2a	chr11	-	NM_0116	P20613	protein_cc	Top-2	topoisome	Top2a
3633	Igfbp4	0.235	1.919	0.451	0.000	0.018	0.043	14.430	12.340	13.280	16010	Igfbp4	chr11	+	NM_0105	P6529	protein_cc	AI875747 |	insulin-like	Igfbp4
3634	Tns4	0.864	1.069	0.924	0.703	0.774	0.879	2.431	2.220	2.317	217169	Tns4	chr11	-	NM_1725	P20855	protein_cc	9930017A	tensin 4	Tns4
3635	Ccr7	5.885	0.434	2.554	0.003	0.038	0.395	2.657	5.214	4.009	12775	Ccr7	chr11	-	NM_0077	P12687	protein_cc	CC-CKR-7 |	chemokin	Ccr7
3636	Smarce1	1.631	0.845	1.378	0.002	0.020	0.164	8.035	8.740	8.497	57376	Smarce1	chr11	-	NM_0206	P5545	protein_cc	2810417B	SWI/SNF r	Smarce1
3637	Krt222	5.391	1.338	7.216	0.000	0.050	0.024	0.713	3.143	3.564	268481	Krt222	chr11	-	NM_1729	P4771	protein_cc	6330509G	keratin 22	Krt222
3638	Krt24	1.000	1.000	1.000	1.000	1.000	1.000	0.000	0.000	0.000	75706	Krt24	chr11	-	NM_0293	P8222	protein_cc	2310058N	keratin 24	Krt24
3639	Krt25	1.000	1.000	1.000	1.000	1.000	1.000	0.000	0.000	0.000	70810	Krt25	chr11	-	NM_1337	P23697	protein_cc	4631426H	keratin 25	Krt25
3640	Krt26	1.000	1.000	1.000	1.000	1.000	1.000	0.000	0.000	0.000	320864	Krt26	chr11	-	NM_0010	P12995	protein_cc	4732407F	keratin 26	Krt26
3641	Krt27	1.000	1.000	1.000	1.000	1.000	1.000	0.000	0.000	0.000	16675	Krt27	chr11	-	NM_0106	P24705	protein_cc	CK-27 | Krt	keratin 27	Krt27
3642	Krt28	1.000	1.000	1.000	1.000	1.000	1.000	0.000	0.000	0.000	70843	Krt28	chr11	-	NM_0275	P12627	protein_cc	4733401L	keratin 28	Krt28
3643	Krt10	0.643	0.893	0.574	0.002	0.329	0.085	5.969	5.332	5.168	16661	Krt10	chr11	-	NM_0106	P23589	protein_cc	D130054E	keratin 10	Krt10
3644	Krt12	1.005	1.405	1.412	0.000	0.182	0.423	0.000	0.008	0.498	268482	Krt12	chr11	-	NM_0106	P13436	protein_cc	AI835216 |	keratin 12	Krt12
3645	Krt20	0.716	0.995	0.712	0.182	1.000	0.423	0.490	0.008	0.000	66809	Krt20	chr11	-	NM_0232	P16471	protein_cc	9030623C	keratin 20	Krt20
3646	Krt23	0.009	2.633	0.023	0.000	0.206	0.024	7.054	0.205	1.602	94179	Krt23	chr11	-	NM_0333	P15339	protein_cc	CK23 | Hai	keratin 23	Krt23
3647	Krt39	1.000	1.000	1.000	1.000	1.000	1.000	0.000	0.000	0.000	237934	Krt39	chr11	-	NM_2137	P18432	protein_cc	4732494G	keratin 39	Krt39
3648	Krt40	1.000	1.000	1.000	1.000	1.000	1.000	0.000	0.000	0.000	406221	Krt40	chr11	-	NM_0010	P21581	protein_cc	CK-40 | K4	keratin 40	Krt40
3649	Krtap3-3	1.000	1.000	1.000	1.000	1.000	1.000	0.000	0.000	0.000	66380	Krtap3-3	chr11	-	NM_0255	P21490	protein_cc	2310020D	keratin as	Krtap3-3
3650	Krtap3-2	1.000	1.000	1.000	1.000	1.000	1.000	0.000	0.000	0.000	66708	Krtap3-2	chr11	-	NM_0257	P15798	protein_cc	2310020D	keratin as	Krtap3-2
3651	Krtap3-1	1.000	1.000	1.000	1.000	1.000	1.000	0.000	0.000	0.000	69473	Krtap3-1	chr11	-	NM_0235	P18725	protein_cc	2310003K	keratin as	Krtap3-1
3652	Krtap1-5	1.000	1.000	1.000	1.000	1.000	1.000	0.000	0.000	0.000	69664	Krtap1-5	chr11	-	NM_0271	P6578	protein_cc	2310043L(	keratin as	Krtap1-5
3653	Krtap1-4	1.000	1.000	1.000	1.000	1.000	1.000	0.000	0.000	0.000	629873	Krtap1-4	chr11	-	NM_0010	P18781	protein_cc	OTTMUSG	keratin as	Krtap1-4
3654	Krtap1-3	0.716	0.995	0.712	0.182	1.000	0.423	0.490	0.008	0.000	435273	Krtap1-3	chr11	-	NM_0010	P11409	protein_cc	.	keratin as	Krtap1-3
3655	Krtap9-3	1.000	1.000	1.000	1.000	1.000	1.000	0.000	0.000	0.000	75586	Krtap9-3	chr11	-	NM_0293	P26475	protein_cc	2310040N	keratin as	Krtap9-3
3656	Gm11938	1.000	1.000	1.000	1.000	1.000	1.000	0.000	0.000	0.000	10004141	Gm11938	chr11	-	NM_0011	P20792	protein_cc	.	predicted	Gm11938
3657	Gm11937	1.000	1.000	1.000	1.000	1.000	1.000	0.000	0.000	0.000	10004148	Gm11937	chr11	-	NM_0010	P3937	protein_cc	.	predicted	Gm11937
3658	Krtap2-4	1.000	1.000	1.000	1.000	1.000	1.000	0.000	0.000	0.000	71453	Krtap2-4	chr11	-	NM_0278	P1571	protein_cc	5530401P	keratin as	Krtap2-4
3659	Gm11562	1.000	1.000	1.000	1.000	1.000	1.000	0.000	0.000	0.000	10050280	Gm11562	chr11	-	NM_0011	P1067	protein_cc	OTTMUSG	predicted	Gm11562
3660	Krtap4-1	1.000	1.000	1.000	1.000	1.000	1.000	0.000	0.000	0.000	665891	Krtap4-1	chr11	-	NM_0010	P1177	protein_cc	A030010K	keratin as	Krtap4-1
3661	Krtap4-2	1.000	1.000	1.000	1.000	1.000	1.000	0.000	0.000	0.000	68673	Krtap4-2	chr11	-	NM_0268	P1737	protein_cc	1110033F(	keratin as	Krtap4-2
3662	Krtap4-7	1.000	1.000	1.000	1.000	1.000	1.000	0.000	0.000	0.000	76444	Krtap4-7	chr11	-	NM_0296	P9779	protein_cc	2310037K	keratin as	Krtap4-7
3663	Gm11563	1.000	1.000	1.000	1.000	1.000	1.000	0.000	0.000	0.000	10004024	Gm11563	chr11	-	NM_0011	P7646	protein_cc	2310045F	predicted	Gm11563
3664	Krtap4-6	1.000	1.000	1.000	1.000	1.000	1.000	0.000	0.000	0.000	68768	Krtap4-6	chr11	-	NM_0268	P12764	protein_cc	1110054P	keratin as	Krtap4-6
3665	Gm11595	1.000	1.000	1.000	1.000	1.000	1.000	0.000	0.000	0.000	10004027	Gm11595	chr11	-	NM_0011	P21114	protein_cc	OTTMUSG	predicted	Gm11595
3666	Krtap4-8	1.000	1.000	1.000	1.000	1.000	1.000	0.000	0.000	0.000	665992	Krtap4-8	chr11	-	NM_0010	P16273	protein_cc	OTTMUSG	keratin as	Krtap4-8
3667	Krtap4-9	1.572	0.636	1.000	0.304	1.000	0.423	0.000	0.653	0.000	665998	Krtap4-9	chr11	+	NM_0010	P670	protein_cc	OTTMUSG	keratin as	Krtap4-9
3668	Gm11596	1.000	1.000	1.000	1.000	1.000	1.000	0.000	0.000	0.000	670464	Gm11596	chr11	-	NM_0010	P12043	protein_cc	OTTMUSG	predicted	Gm11596
3669	Gm11554	1.000	1.000	1.000	1.000	1.000	1.000	0.000	0.000	0.000	670482	Gm11554	chr11	-	NM_0010	P26448	protein_cc	OTTMUSG	predicted	Gm11554
3670	Gm11569	1.000	1.000	1.000	1.000	1.000	1.000	0.000	0.000	0.000	670472	Gm11569	chr11	-	NM_0010	P9937	protein_cc	OTTMUSG	predicted	Gm11569
3671	Krtap4-13	1.000	1.000	1.000	1.000	1.000	1.000	0.000	0.000	0.000	69464	Krtap4-13	chr11	-	NM_0270	P19995	protein_cc	2300006N	keratin as	Krtap4-13
3672	Gm11564	1.000	1.000	1.000	1.000	1.000	1.000	0.000	0.000	0.000	670496	Gm11564	chr11	-	NM_0011	P4103	protein_cc	OTTMUSG	predicted	Gm11564
3673	2300003K	1.000	1.000	1.000	1.000	1.000	1.000	0.000	0.000	0.000	10050286	2300003K	chr11	-	NM_0011	P16288	protein_cc	.	RIKEN cDN	2300003K
3674	Krtap4-16	1.000	1.000	1.000	1.000	1.000	1.000	0.000	0.000	0.000	435285	Krtap4-16	chr11	-	NM_0010	P26646	protein_cc	OTTMUSG	keratin as	Krtap4-16
3675	Gm11568	1.000	1.000	1.000	1.000	1.000	1.000	0.000	0.000	0.000	432600	Gm11568	chr11	+	NM_0012	P21642	protein_cc	OTTMUSG	predicted	Gm11568
3676	Gm11559	1.000	1.000	1.000	1.000	1.000	1.000	0.000	0.000	0.000	10041578	Gm11559	chr11	+	NM_0011	P15658	protein_cc	OTTMUSG	predicted	Gm11559
3677	Krtap9-1	1.000	1.000	1.000	1.000	1.000	1.000	0.000	0.000	0.000	16705	Krtap9-1	chr11	+	NM_0157	P10326	protein_cc	.	keratin as	Krtap9-1
3678	Gm11567	1.000	1.000	1.000	1.000	1.000	1.000	0.000	0.000	0.000	670533	Gm11567	chr11	+	NM_0011	P4794	protein_cc	OTTMUSG	predicted	Gm11567
3679	Krtap31-1	1.000	1.000	1.000	1.000	1.000	1.000	0.000	0.000	0.000	70831	Krtap31-1	chr11	+	NM_0275	P26288	protein_cc	4733401H	keratin as	Krtap31-1
3680	Gm11565	1.000	1.000	1.000	1.000	1.000	1.000	0.000	0.000	0.000	670550	Gm11565	chr11	+	NM_0011	P14518	protein_cc	OTTMUSG	predicted	Gm11565
3681	Krtap31-2	1.000	1.000	1.000	1.000	1.000	1.000	0.000	0.000	0.000	432602	Krtap31-2	chr11	+	NM_0010	P1278	protein_cc	OTTMUSG	keratin as	Krtap31-2
3682	Krtap9-5	1.000	1.000	1.000	1.000	1.000	1.000	0.000	0.000	0.000	435286	Krtap9-5	chr11	+	NM_0010	P24750	protein_cc	.	keratin as	Krtap9-5
3683	Krtap16-1	1.000	1.000	1.000	1.000	1.000	1.000	0.000	0.000	0.000	10050418	Krtap16-1	chr11	-	NM_0012	P13892	protein_cc	Gm11570	keratin as	Krtap16-1
3684	Krtap17-1	1.000	1.000	1.000	1.000	1.000	1.000	0.000	0.000	0.000	77914	Krtap17-1	chr11	-	NM_0010	P6772	protein_cc	.	keratin as	Krtap17-1
3685	Krt33a	1.000	1.000	1.000	1.000	1.000	1.000	0.000	0.000	0.000	71888	Krt33a	chr11	-	NM_0279	P3895	protein_cc	2310015J(	keratin 33	Krt33a
3686	Krt33b	1.000	1.000	1.000	1.000	1.000	1.000	0.000	0.000	0.000	16671	Krt33b	chr11	-	NM_0135	P16328	protein_cc	Ha3 | Ha4 |	keratin 33	Krt33b
3687	Krt34	1.005	1.405	1.412	0.000	0.182	0.423	0.000	0.008	0.498	16672	Krt34	chr11	-	NM_0275	P6163	protein_cc	4733401E	keratin 34	Krt34
3688	Krt31	1.000	1.000	1.000	1.000	1.000	1.000	0.000	0.000	0.000	16660	Krt31	chr11	-	NM_0106	P13168	protein_cc	Ha1 | Kha1	keratin 31	Krt31
3689	Krt32	1.000	1.000	1.000	1.000	1.000	1.000	0.000	0.000	0.000	16670	Krt32	chr11	-	NM_0011	P16271	protein_cc	Ha3 | Hka2	keratin 32	Krt32
3690	Krt35	1.000	1.000	1.000	1.000	1.000	1.000	0.000	0.000	0.000	53617	Krt35	chr11	-	NM_0168	P8999	protein_cc	AI662400 |	keratin 35	Krt35
3691	Krt36	1.000	1.000	1.000	1.000	1.000	1.000	0.000	0.000	0.000	16673	Krt36	chr11	-	NM_0011	P8300	protein_cc	HRa-1 | K3	keratin 36	Krt36
3692	Krt13	1.000	1.000	1.000	1.000	1.000	1.000	0.000	0.000	0.000	16663	Krt13	chr11	-	NM_0106	P7876	protein_cc	CK13 | K13	keratin 13	Krt13
3693	Krt15	1.000	1.000	1.000	1.000	1.000	1.000	0.000	0.000	0.000	16665	Krt15	chr11	-	NM_0084	P299	protein_cc	AI528832 |	keratin 15	Krt15
3694	Krt19	0.080	12.533	1.002	0.000	0.060	0.997	4.442	0.798	4.446	16669	Krt19	chr11	-	NM_0084	P6809	protein_cc	AI663979 |	keratin 19	Krt19
3695	Krt9	1.000	1.000	1.000	1.000	1.000	1.000	0.000	0.000	0.000	107656	Krt9	chr11	-	NM_2012	P15867	protein_cc	EPPK | K9 |	keratin 9	Krt9
3696	Krt14	1.000	1.000	1.000	1.000	1.000	1.000	0.000	0.000	0.000	16664	Krt14	chr11	-	NM_0169	P23806	protein_cc	AI626930 |	keratin 14	Krt14
3697	Krt16	1.000	1.000	1.000	1.000	1.000	1.000	0.000	0.000	0.000	16666	Krt16	chr11	-	NM_0084	P18186	protein_cc	AI324768 |	keratin 16	Krt16
3698	Krt17	7.785	0.196	1.528	0.184	0.214	0.521	0.715	3.676	1.327	16667	Krt17	chr11	-	NM_0106	P7909	protein_cc	K17 | Krt1-	keratin 17	Krt17
3699	Krt42	3.316	0.301	1.000	0.070	1.000	0.423	0.001	1.730	0.000	68239	Krt42	chr11	-	NM_2124	P12528	protein_cc	2410039E	keratin 42	Krt42
3700	Eif1	1.054	0.746	0.787	0.095	0.004	0.087	11.645	11.722	11.299	20918	Eif1	chr11	+	NM_0115	P25189	protein_cc	Sui1-rs1	eukaryotic	Eif1
3701	Gast	1.000	1.000	1.000	1.000	1.000	1.000	0.000	0.000	0.000	14459	Gast	chr11	+	NM_0102	P9350	protein_cc	GAS	gastrin	Gast
3702	Hap1	5.478	0.465	2.545	0.003	0.012	0.054	3.094	5.548	4.442	15114	Hap1	chr11	-	NM_1779	P22525	protein_cc	HAP-1	huntingtin	Hap1
3703	Jup	0.669	0.703	0.470	0.029	0.135	0.077	8.486	7.906	7.398	16480	Jup	chr11	-	NM_0105	P10257	protein_cc	Ctnng | D9	junction p	Jup
3704	P3h4	0.392	2.376	0.932	0.001	0.081	0.876	4.412	3.063	4.311	66180	P3h4	chr11	-	NM_1768	P25797	protein_cc	1110036O	prolyl 3-hy	P3h4
3705	Fkbp10	0.255	3.912	0.998	0.038	0.054	0.998	3.503	1.532	3.500	14230	Fkbp10	chr11	+	NM_0102	P798	protein_cc	AI325255 |	FK506 bin	Fkbp10
3706	Nt5c3b	1.251	1.421	1.777	0.380	0.152	0.280	3.844	4.167	4.673	68106	Nt5c3b	chr11	-	NM_0265	P9835	protein_cc	2610037D	5'-nucleot	Nt5c3b
3707	Klhl10	0.373	0.981	0.366	0.018	1.000	0.140	1.450	0.027	0.000	66720	Klhl10	chr11	+	NM_0257	P864	protein_cc	4921517C	kelch-like	Klhl10
3708	Klhl11	1.869	1.385	2.589	0.272	0.379	0.074	2.136	3.038	3.508	217194	Klhl11	chr11	-	NM_1725	P6663	protein_cc	BC011167	kelch-like	Klhl11
3709	Acly	0.301	2.084	0.628	0.000	0.004	0.046	9.778	8.048	9.107	104112	Acly	chr11	-	NM_1340	P4457	protein_cc	A730098H	ATP citrat	Acly
3710	Ttc25	1.580	0.633	1.000	0.305	1.000	0.423	0.000	0.660	0.000	74407	Ttc25	chr11	+	NM_0289	P5633	protein_cc	4933404O	tetratrico	Ttc25
3711	Cnp	0.508	0.895	0.455	0.005	0.640	0.030	6.668	5.691	5.531	12799	Cnp	chr11	+	NM_0099	P11668	protein_cc	Cnp-1 | Cn	2',3'-cyclic	Cnp
3712	Dnajc7	1.778	0.816	1.450	0.001	0.015	0.142	8.198	9.028	8.734	56354	Dnajc7	chr11	-	NM_0197	P18796	protein_cc	2010003F	DnaJ heat	Dnajc7
3713	Nkiras2	1.354	0.937	1.268	0.100	0.712	0.380	6.572	7.008	6.914	71966	Nkiras2	chr11	+	NM_0280	P18040	protein_cc	2410003N	NFKB inhil	Nkiras2
3714	Zfp385c	1.354	0.937	1.268	0.100	0.712	0.380	6.572	7.008	6.914	278304	Zfp385c	chr11	-	NM_1777	P26144	protein_cc	A930006D	zinc finger	Zfp385c
3715	Dhx58	4.467	0.469	2.094	0.001	0.030	0.387	5.954	8.114	7.021	80861	Dhx58	chr11	-	NM_0301	P3124	protein_cc	B430001I(	DEXH (Asp	Dhx58
3716	Kat2a	0.760	0.752	0.572	0.129	0.407	0.256	5.641	5.245	4.834	14534	Kat2a	chr11	-	NM_0010	P7211	protein_cc	1110051E	K(lysine) a	Kat2a
3717	Hspb9	0.552	0.990	0.547	0.182	1.000	0.423	0.871	0.015	0.000	75482	Hspb9	chr11	+	NM_0293	P9351	protein_cc	1700007H	heat shocl	Hspb9
3718	Rab5c	1.557	0.760	1.183	0.167	0.308	0.319	10.278	10.917	10.521	19345	Rab5c	chr11	-	NM_0244	P2516	protein_cc	AI326010 |	RAB5C, m	Rab5c

3719	Kcnh4	1.000	1.000	1.000	1.000	1.000	1.000	0.000	0.000	0.000	380728	Kcnh4	chr11	-	NM_0010	P22913	protein_cc	BEC2	potassium	Kcnh4
3720	Hcrt	1.000	1.000	1.000	1.000	1.000	1.000	0.000	0.000	0.000	15171	Hcrt	chr11	-	NM_0104	P14259	protein_cc	PPOX	hypocretin	Hcrt
3721	Ghdc	0.453	1.137	0.515	0.002	0.702	0.178	7.687	6.543	6.729	80860	Ghdc	chr11	-	NM_0318	P1176	protein_cc	D11Lgp1e	GH3 doma	Ghdc
3722	Stat5b	0.288	0.769	0.221	0.000	0.181	0.002	9.011	7.216	6.836	20851	Stat5b	chr11	-	NM_0114	P14773	protein_cc	.	signal trar	Stat5b
3723	Stat5a	0.856	1.030	0.882	0.026	0.721	0.333	6.459	6.235	6.277	20850	Stat5a	chr11	+	NM_0114	P9059	protein_cc	AA959963	signal trar	Stat5a
3724	Stat3	4.170	0.732	3.053	0.011	0.279	0.151	10.435	12.495	12.045	20848	Stat3	chr11	-	NM_0114	P2627	protein_cc	1110034C	signal trar	Stat3
3725	Ptrf	0.262	2.297	0.602	0.001	0.021	0.135	8.064	6.133	7.332	19285	Ptrf	chr11	-	NM_0089	P2676	protein_cc	2310075E	polymeras	Ptrf
3726	Atp6v0a1	2.236	0.935	2.092	0.000	0.476	0.063	8.344	9.505	9.409	11975	Atp6v0a1	chr11	+	NM_0012	P13913	protein_cc	AA959968	ATPase, H	Atp6v0a1
3727	Naglu	0.323	1.304	0.422	0.000	0.015	0.011	7.967	6.338	6.720	27419	Naglu	chr11	+	NM_0137	P2739	protein_cc		alpha-N-a	Naglu
3728	Hsd17b1	1.238	0.456	0.565	0.421	1.000	0.422	0.824	1.132	0.000	15485	Hsd17b1	chr11	+	NM_0104	P14825	protein_cc	17beta-HS	hydroxyst	Hsd17b1
3729	Coasy	0.134	2.296	0.307	0.000	0.029	0.016	11.127	8.223	9.423	71743	Coasy	chr11	+	NM_0013	P23215	protein_cc	1300003G	Coenzyme	Coasy
3730	Mlx	1.150	0.988	1.136	0.144	0.860	0.222	8.486	8.688	8.670	21428	Mlx	chr11	+	NM_0011	P3841	protein_cc	bHLHd13	MAX-like	Mlx
3731	Psmc3ip	0.954	0.923	0.881	0.801	0.540	0.639	7.144	7.075	6.960	19183	Psmc3ip	chr11	-	NM_0089	P2556	protein_cc	C79099	proteason	Psmc3ip
3732	Fam134c	0.291	1.332	0.387	0.000	0.142	0.012	7.884	6.101	6.515	67998	Fam134c	chr11	-	NM_0289	P7397	protein_cc	1300010N	family wit	Fam134c
3733	Tubg1	0.385	1.950	0.752	0.001	0.153	0.547	7.863	6.488	7.452	103733	Tubg1	chr11	+	NM_1340	P7346	protein_cc	1500010O	tubulin, ga	Tubg1
3734	Tubg2	3.041	0.732	2.226	0.001	0.442	0.523	0.588	2.193	1.743	103768	Tubg2	chr11	+	NM_1340	P8062	protein_cc	AI504772	tubulin, ga	Tubg2
3735	Plekhh3	0.496	0.875	0.434	0.089	0.480	0.241	6.259	5.247	5.054	217198	Plekhh3	chr11	-	NM_1460	P16759	protein_cc	BC020025	pleckstrin	Plekhh3
3736	Ccr10	1.005	1.405	1.412	0.000	0.182	0.423	0.000	0.008	0.498	12777	Ccr10	chr11	-	NM_0077	P382	protein_cc	Cmkbr9	chemokin	Ccr10
3737	Cntnap1	4.253	1.821	7.743	0.036	0.156	0.152	2.071	4.160	5.024	53321	Cntnap1	chr11	+	NM_0167	P21098	protein_cc	AI841080	contactin	Cntnap1
3738	Ezh1	1.185	0.779	0.923	0.010	0.006	0.502	7.025	7.270	6.910	14055	Ezh1	chr11	-	NM_0079	P10738	protein_cc	ENX-2	enhancer	Ezh1
3739	Ramp2	0.594	2.805	1.667	0.007	0.081	0.472	6.543	5.792	7.280	54409	Ramp2	chr11	+	NM_0194	P14148	protein_cc	.	receptor	Ramp2
3740	Vps25	0.719	1.216	0.874	0.003	0.074	0.298	10.231	9.754	10.037	28084	Vps25	chr11	+	NM_0267	P2919	protein_cc	1110020N	vacuolar p	Vps25
3741	Wnk4	0.523	0.488	0.255	0.297	0.062	0.362	2.445	1.510	0.475	69847	Wnk4	chr11	+	NM_1756	P15759	protein_cc	2010002J1	WNK lysin	Wnk4
3742	Coa3	0.512	1.524	0.781	0.004	0.119	0.445	9.379	8.414	9.022	52469	Coa3	chr11	-	NM_0266	P11542	protein_cc	1810033A	cytochron	Coa3
3743	Cntd1	1.111	1.349	1.499	0.582	0.127	0.172	8.972	9.124	9.556	68107	Cntd1	chr11	+	NM_0265	P6028	protein_cc	1700051C	cyclin N-te	Cntd1
3744	Becn1	1.135	1.326	1.505	0.464	0.118	0.116	9.092	9.274	9.681	56208	Becn1	chr11	-	NM_0195	P19201	protein_cc	4921513J1	beclin 1, a	Becn1
3745	Psme3	1.297	1.030	1.335	0.373	0.898	0.289	8.527	8.902	8.944	19192	Psme3	chr11	+	NM_0111	P26621	protein_cc	AA410043	proteaso	Psme3
3746	Aoc2	0.737	0.850	0.626	0.442	0.643	0.410	2.225	1.785	1.550	237940	Aoc2	chr11	+	NM_1789	P19913	protein_cc	RAO	amine oxi	Aoc2
3747	Aoc3	0.250	2.115	0.530	0.004	0.020	0.188	2.055	0.057	1.138	11754	Aoc3	chr11	+	NM_0096	P24893	protein_cc	SSAO	VAI	amine oxi	Aoc3
3748	G6pc	0.004	2.591	0.009	0.000	0.213	0.001	12.681	4.555	5.928	14377	G6pc	chr11	+	NM_0080	P13872	protein_cc	AW10737	glucose-6-	G6pc
3749	Aarsd1	0.499	1.686	0.841	0.002	0.014	0.442	7.483	6.480	7.234	69684	Aarsd1	chr11	-	NM_1448	P11255	protein_cc	1110069E	alanyl-tRN	Aarsd1
3750	Ptges3l	0.515	1.603	0.825	0.059	0.153	0.713	5.778	4.820	5.500	73635	Ptges3l	chr11	-	NM_0268	P26207	protein_cc	1110069E	prostaglar	Ptges3l
3751	Rundc1	1.051	1.103	1.159	0.864	0.731	0.411	6.133	6.206	6.347	217201	Rundc1	chr11	+	NM_1725	P25588	protein_cc	AW12554	RUN doma	Rundc1
3752	Rpl27	1.115	1.437	1.602	0.164	0.024	0.108	9.247	9.404	9.928	19942	Rpl27	chr11	+	NM_0112	P138	protein_cc	.	ribosomal	Rpl27
3753	Ifi35	4.297	0.536	2.301	0.000	0.002	0.094	7.488	9.591	8.690	70110	Ifi35	chr11	-	NM_0273	P4926	protein_cc	2010008K	interferon	Ifi35
3754	Vat1	0.639	1.127	0.720	0.125	0.634	0.256	5.862	5.216	5.388	26949	Vat1	chr11	-	NM_0120	P8426	protein_cc	VAT-1	vesicle am	Vat1
3755	Rnd2	1.005	1.405	1.412	0.000	0.182	0.423	0.000	0.008	0.498	11858	Rnd2	chr11	+	NM_0097	P19232	protein_cc	Arhn	Roh	Rho family	Rnd2
3756	Brca1	0.599	3.583	2.146	0.039	0.067	0.383	1.943	1.203	3.044	12189	Brca1	chr11	-	NM_0097	P1875	protein_cc	.	breast car	Brca1
3757	Nbr1	0.596	0.874	0.521	0.009	0.172	0.064	10.356	9.609	9.415	17966	Nbr1	chr11	+	NM_0012	P3	protein_cc	mKIAA004	neighbor c	Nbr1
3758	Tmem106	1.702	0.741	1.260	0.006	0.001	0.376	6.677	7.444	7.011	217203	Tmem106	chr11	+	NM_1448	P3918	protein_cc	0610008L	transmem	Tmem106
3759	LOC66633	1.000	1.000	1.000	1.000	1.000	1.000	0.000	0.000	0.000	666331	LOC66633	chr11	+	NM_0012	P19479	protein_cc	.	uncharact	LOC66633
3760	Rdm1	0.335	0.830	0.278	0.000	0.116	0.003	6.273	4.694	4.426	66599	Rdm1	chr11	+	NM_0256	P16381	protein_cc	2410008N	RAD52 mo	Rdm1
3761	Arl4d	0.054	2.605	0.140	0.000	0.002	0.015	7.488	3.275	4.656	80981	Arl4d	chr11	+	NM_0254	P11664	protein_cc	1110036H	ADP-ribos	Arl4d
3762	Dhx8	1.419	0.845	1.199	0.062	0.241	0.127	7.787	8.292	8.049	217207	Dhx8	chr11	+	NM_1448	P14726	protein_cc	Ddx8	mD	DEAH (Asp	Dhx8
3763	Etv4	1.019	2.762	2.814	0.000	0.181	0.423	0.000	0.027	1.493	18612	Etv4	chr11	-	NM_0088	P10217	protein_cc	AW41440	ets variant	Etv4
3764	Meox1	0.680	2.374	1.615	0.551	0.154	0.398	2.764	2.209	3.456	17285	Meox1	chr11	-	NM_0107	P18923	protein_cc	AI385561	mesenchy	Meox1
3765	4930417O	1.000	1.000	1.000	1.000	1.000	1.000	0.000	0.000	0.000	73887	4930417O	chr11	+	NR_04580	.	lncRNA	.	RIKEN cDN	4930417O
3766	Sost	1.000	1.000	1.000	1.000	1.000	1.000	0.000	0.000	0.000	74499	Sost	chr11	-	NM_0244	P5361	protein_cc	5430411E	sclerostin	Sost
3767	Dusp3	0.711	1.639	1.165	0.018	0.109	0.708	8.671	8.178	8.891	72349	Dusp3	chr11	-	NM_0282	P19137	protein_cc	2210015O	dual speci	Dusp3
3768	1700006E	1.000	1.000	1.000	1.000	1.000	1.000	0.000	0.000	0.000	75437	1700006E	chr11	+	NM_0292	P8702	protein_cc	.	RIKEN cDN	1700006E
3769	Mpp3	1.015	2.253	2.285	0.000	0.015	0.125	0.000	0.021	1.193	13384	Mpp3	chr11	-	NM_0078	P3386	protein_cc	6430514B	membran	Mpp3
3770	Cd300lg	0.006	11.998	0.074	0.000	0.078	0.021	7.547	0.212	3.797	52685	Cd300lg	chr11	+	NM_0011	P18384	protein_cc	2310016B	CD300 mo	Cd300lg
3771	Mpp2	0.367	1.344	0.494	0.000	0.200	0.083	1.479	0.033	0.460	50997	Mpp2	chr11	-	NM_0166	P3125	protein_cc	D11Bwg06	membran	Mpp2
3772	Ppy	1.000	1.000	1.000	1.000	1.000	1.000	0.000	0.000	0.000	19064	Ppy	chr11	-	NM_0089	P7781	protein_cc	AV061413	pancreatic	Ppy
3773	Pyy	1.000	1.000	1.000	1.000	1.000	1.000	0.000	0.000	0.000	217212	Pyy	chr11	-	NM_1454	P12059	protein_cc	.	peptide YY	Pyy
3774	Nags	1.078	1.027	1.107	0.063	0.583	0.363	8.561	8.670	8.708	217214	Nags	chr11	+	NM_1458	P4903	protein_cc	1700120E	N-acetylgl	Nags
3775	Tmem101	0.865	1.250	1.081	0.574	0.350	0.765	6.125	5.916	6.238	76547	Tmem101	chr11	-	NM_0296	P974	protein_cc	2610511E	transmem	Tmem101
3776	Lsm12	1.241	1.363	1.691	0.002	0.111	0.192	6.588	6.899	7.346	268490	Lsm12	chr11	-	NM_1729	P8120	protein_cc	1110063E	LSM12 ho	Lsm12
3777	G6pc3	0.541	0.884	0.478	0.136	0.531	0.348	6.059	5.172	4.995	68401	G6pc3	chr11	+	NM_1759	P21137	protein_cc	0710001K	glucose 6	G6pc3
3778	Hdac5	0.332	0.928	0.308	0.000	0.805	0.060	7.381	5.792	5.684	15184	Hdac5	chr11	-	NM_0012	P16532	protein_cc	AI426555	histone de	Hdac5
3779	BC030867	0.395	2.256	0.890	0.184	0.186	0.903	1.385	0.043	1.216	217216	BC030867	chr11	-	NM_1535	P14538	protein_cc	.	cDNA seq	BC030867
3780	Asb16	1.000	1.000	1.000	1.000	1.000	1.000	0.000	0.000	0.000	217217	Asb16	chr11	+	NM_1489	P24211	protein_cc	.	ankyrin re	Asb16
3781	Tmub2	1.368	0.889	1.217	0.203	0.482	0.475	7.871	8.323	8.154	72053	Tmub2	chr11	+	NM_0013	P17654	protein_cc	2010008E	transmem	Tmub2
3782	Atxn7l3	1.469	0.897	1.317	0.113	0.392	0.389	7.309	7.864	7.707	217218	Atxn7l3	chr11	-	NM_0010	P3715	protein_cc	E030022H	ataxin 7-li	Atxn7l3
3783	Ubtf	0.718	1.225	0.880	0.122	0.341	0.433	7.706	7.228	7.521	21429	Ubtf	chr11	-	NM_0013	P17571	protein_cc	A930005G	upstream	Ubtf
3784	Slc4a1	6.385	0.358	2.284	0.000	0.001	0.306	0.719	3.394	1.911	20533	Slc4a1	chr11	-	NM_0114	P18595	protein_cc	Ae1	CD2	solute car	Slc4a1
3785	Bloodlinc	1.575	0.466	0.734	0.475	1.000	0.422	0.446	1.101	0.000	10546305	Bloodlinc	chr11	-	NR_13119	.	lncRNA	Gm11616	Bloodlinc,	Bloodlinc
3786	Rundc3a	0.578	2.499	1.444	0.012	0.002	0.104	7.619	6.828	8.149	51799	Rundc3a	chr11	+	NM_0167	P24316	protein_cc	Rap2ip	R	RUN doma	Rundc3a
3787	Slc25a39	0.556	1.326	0.738	0.003	0.070	0.135	12.299	11.453	11.860	68066	Slc25a39	chr11	-	NM_0265	P2700	protein_cc	3010027G	solute car	Slc25a39
3788	Grn	1.347	1.853	2.495	0.000	0.005	0.046	11.140	11.570	12.459	14824	Grn	chr11	+	NM_0081	P21427	protein_cc	epithelin	granulin	Grn
3789	Fam171a2	0.102	1.719	0.175	0.000	0.451	0.037	5.728	2.431	3.212	217219	Fam171a2	chr11	-	NM_1992	P25507	protein_cc	.	family wit	Fam171a2
3790	Itga2b	1.258	0.511	0.643	0.020	0.002	0.059	5.081	5.412	4.443	16399	Itga2b	chr11	-	NM_0105	P2086	protein_cc	AI172977	integrin al	Itga2b
3791	Gpatch8	0.656	0.923	0.606	0.004	0.443	0.036	7.842	7.235	7.119	237943	Gpatch8	chr11	-	NM_0011	P6400	protein_cc	5430405G	G patch do	Gpatch8
3792	Gm11627	1.000	1.000	1.000	1.000	1.000	1.000	0.000	0.000	0.000	10004079	Gm11627	chr11	-	NR_04028	.	lncRNA	OTTMUSG	predicted	Gm11627
3793	Fzd2	0.410	2.181	0.894	0.184	0.017	0.883	1.325	0.039	1.164	57265	Fzd2	chr11	+	NM_0205	P18747	protein_cc	AL033370	frizzled cla	Fzd2
3794	2810433D	0.852	0.641	0.547	0.798	0.489	0.547	1.366	1.135	0.494	77132	2810433D	chr11	-	NR_03347	.	lncRNA	.	RIKEN cDN	2810433D
3795	Gm1564	1.000	1.000	1.000	1.000	1.000	1.000	0.000	0.000	0.000	.	Gm1564	chr11	+	NM_0011	P5540	.	.	.	Gm1564
3796	Ccdc43	1.573	1.045	1.644	0.058	0.762	0.006	6.556	7.209	7.273	52715	Ccdc43	chr11	-	NM_0259	P2799	protein_cc	2610001E	coiled-coil	Ccdc43
3797	Adam11	0.328	0.411	0.135	0.046	0.003	0.152	5.032	3.424	2.141	11488	Adam11	chr11	+	NM_0096	P25301	protein_cc	AW06061	a disinteg	Adam11
3798	Gjc1	2.521	1.024	2.582	0.192	0.955	0.075	2.925	4.259	4.294	14615	Gjc1	chr11	-	NM_0011	P18577	protein_cc	C130009G	gap juncti	Gjc1
3799	Higd1b	1.000	1.000	1.000	1.000	1.000	1.000	0.000	0.000	0.000	75689	Higd1b	chr11	+	NM_0808	P14975	protein_cc	2310056K	HIG1 dom	Higd1b
3800	Eftud2	1.608	0.975	1.567	0.002	0.861	0.261	7.199	7.884	7.847	20624	Eftud2	chr11	-	NM_0110	P1629	protein_cc	116kDa	S	elongation	Eftud2
3801	Ccdc103	0.735	1.867	1.372	0.191	0.184	0.650	0.465	0.021	0.922	73293	Ccdc103	chr11	+	NM_0284	P12957	protein_cc	1700039D	coiled-coil	Ccdc103
3802	Fam187a	1.000	1.000	1.000	1.000	1.000	1.000	0.000	0.000	0.000	66784	Fam187a	chr11	+	NM_0257	P18112	protein_cc	4933439F	family wit	Fam187a
3803	Gfap	0.451	2.155	0.972	0.002	0.007	0.904	1.186	0.038	1.146	14580	Gfap	chr11	-	NM_0102	P26102	protein_cc	AI836096	glial fibrill	Gfap
3804	Kif18b	1.005	1.439	1.446	0.000	0.182	0.423	0.000	0.000	0.532	70218	Kif18b	chr11	-	NM_1979	P10756	protein_cc	3000004C	kinesin fa	Kif18b
3805	C1ql1	1.000	1.000	1.000	1.000	1.000	1.000	0.000	0.000	0.000	23829	C1ql1	chr11	-	NM_0117	P14423	protein_cc	C1qrf	CR	compleme	C1ql1
3806	2410004I	1.000	1.000	1.000	1.000	1.000	1.000	0.000	0.000	0.000	69739	2410004I	chr11	-	NR_03796	.	lncRNA	.	RIKEN cDN	2410004I
3807	Dcakd	1.570	1.073	1.684	0.149	0.737	0.011	7.463	8.113	8.215	68087	Dcakd	chr11	-	NM_0265	P22477	protein_cc	3010024O	dephosph	Dcakd
3808	Nmt1	1.245	1.198	1.491	0.085	0.204	0.140	9.515	9.831	10.092	18107	Nmt1	chr11	+	NM_0087	P21828	protein_cc	AW53659	N-myristo	Nmt1
3809	Plcd3	2.070	2.365	4.897	0.094	0.197	0.309	2.455	3.505	4.747	72469	Plcd3	chr11	-	NM_1528	P15084	protein_cc	2610205J1	phospholi	Plcd3

3810	Acbd4	0.315	1.031	0.324	0.000	0.880	0.021	9.117	7.448	7.493	67131	Acbd4	chr11	+	NM_0259	P19616	protein_cc	2010009P	acyl-Coen	Acbd4
3811	Hexim1	0.829	0.819	0.679	0.018	0.019	0.014	8.782	8.511	8.222	192231	Hexim1	chr11	+	NM_1387	P26242	protein_cc	7330426E	hexameth	Hexim1
3812	Hexim2	1.454	1.029	1.496	0.031	0.817	0.118	4.113	4.653	4.694	71059	Hexim2	chr11	+	NM_0011	P14048	protein_cc	4933402L	hexameth	Hexim2
3813	Fmnl1	6.794	0.763	5.183	0.010	0.341	0.090	2.987	5.752	5.361	57778	Fmnl1	chr11	+	NM_0196	P8784	protein_cc	8030453N	formin-like	Fmnl1
3814	1700023F	1.000	1.000	1.000	1.000	1.000	1.000	0.000	0.000	0.000	69441	1700023F	chr11	-	NM_0012	P18944	protein_cc	.	RIKEN cDN	1700023F0
3815	Spata32	1.000	1.000	1.000	1.000	1.000	1.000	0.000	0.000	0.000	328019	Spata32	chr11	-	NM_1778	P17395	protein_cc	4933400C	spermatog	Spata32
3816	Map3k14	15.021	0.663	9.953	0.000	0.069	0.076	3.004	6.913	6.319	53859	Map3k14	chr11	-	NM_0168	P169	protein_cc	aly| Nik	mitogen-a	Map3k14
3817	Arhgap27	2.460	0.399	0.983	0.049	0.043	0.980	2.857	4.156	2.832	544817	Arhgap27	chr11	-	NM_1337	P18260	protein_cc	2310069I	Rho GTPas	Arhgap27
3818	Arhgap27	1.598	1.135	1.813	0.300	0.776	0.423	0.000	0.677	0.859	653030	Arhgap27	chr11	+	NR_04534	.	lncRNA	F830206d	Rho GTPas	Arhgap27c
3819	Plekhm1	3.444	0.408	1.406	0.000	0.001	0.371	5.882	7.666	6.373	353047	Plekhm1	chr11	-	NM_1830	P9399	protein_cc	AP162| B2	pleckstrin	Plekhm1
3820	Gm884	1.000	1.000	1.000	1.000	1.000	1.000	0.000	0.000	0.000	380730	Gm884	chr11	-	NM_0010	P5085	protein_cc	LRRC37	predicted	Gm884
3821	Lyzl6	1.000	1.000	1.000	1.000	1.000	1.000	0.000	0.000	0.000	69444	Lyzl6	chr11	-	NM_0270	P19665	protein_cc	1700023H	lysozyme-	Lyzl6
3822	1700072I	1.000	1.000	1.000	1.000	1.000	1.000	0.000	0.000	0.000	78629	1700072I	chr11	+	NR_13114	.	lncRNA	.	RIKEN cDN	1700072I2
3823	Rprml	1.580	0.633	1.000	0.305	1.000	0.423	0.000	0.660	0.000	104582	Rprml	chr11	+	NM_0010	P1937	protein_cc	AW04933	reprimo-li	Rprml
3824	Gosr2	2.156	1.350	2.910	0.001	0.052	0.042	7.435	8.543	8.976	56494	Gosr2	chr11	-	NM_0196	P2578	protein_cc	2310032N	golgi SNAF	Gosr2
3825	C130046K	0.542	1.207	0.655	0.441	0.263	0.730	2.913	2.031	2.302	399609	C130046K	chr11	+	NR_10239	.	lncRNA	.	RIKEN cDN	C130046K
3826	Wnt9b	0.488	8.577	4.185	0.001	0.181	0.483	1.130	0.095	3.196	22412	Wnt9b	chr11	-	NM_0117	P16399	protein_cc	clf| clf1| v	wingless-t	Wnt9b
3827	Wnt3	1.572	0.636	1.000	0.304	1.000	0.423	0.000	0.653	0.000	22415	Wnt3	chr11	+	NM_0095	P22794	protein_cc	Int-4| Wnt	wingless-t	Wnt3
3828	Nsf	1.452	0.826	1.199	0.068	0.081	0.515	7.273	7.811	7.535	18195	Nsf	chr11	-	NM_0087	P18339	protein_cc	AI316878|	N-ethylma	Nsf
3829	Arf2	4.237	1.240	5.254	0.000	0.363	0.118	5.561	7.644	7.954	11841	Arf2	chr11	+	NM_0013	P19836	protein_cc	.	ADP-ribos	Arf2
3830	Crhr1	1.000	1.000	1.000	1.000	1.000	1.000	0.000	0.000	0.000	12921	Crhr1	chr11	+	NM_0077	P4929	protein_cc	CRFR1| Cr	corticotro	Crhr1
3831	Sppl2c	1.000	1.000	1.000	1.000	1.000	1.000	0.000	0.000	0.000	237958	Sppl2c	chr11	+	NM_0010	P20116	protein_cc	4933407P	signal pep	Sppl2c
3832	Mapt	0.103	1.009	0.104	0.025	0.990	0.162	5.088	1.813	1.826	17762	Mapt	chr11	+	NM_0012	P23191	protein_cc	AI413597|	microtubu	Mapt
3833	Kansl1	1.579	1.235	1.951	0.073	0.329	0.144	7.073	7.732	8.037	76719	Kansl1	chr11	-	NM_0010	P12638	protein_cc	1700081L	KAT8 regu	Kansl1
3834	Cdc27	1.197	0.952	1.139	0.044	0.833	0.768	7.221	7.480	7.409	217232	Cdc27	chr11	-	NM_0012	P361	protein_cc	AI452358|	cell divisio	Cdc27
3835	Myl4	1.786	0.708	1.263	0.272	0.294	0.751	1.844	2.680	2.181	17896	Myl4	chr11	+	NM_0108	P14154	protein_cc	ALC1| AM	myosin, lig	Myl4
3836	Gm11665	1.000	1.000	1.000	1.000	1.000	1.000	0.000	0.000	0.000	10041591	Gm11665	chr11	-	NR_13017	.	lncRNA	Gm11664	predicted	Gm11665
3837	Itgb3	6.862	0.840	5.763	0.000	0.575	0.240	4.968	7.746	7.495	16416	Itgb3	chr11	+	NM_0167	P3720	protein_cc	CD61| GP	integrin be	Itgb3
3838	Efcab3	0.726	1.387	1.007	0.187	0.187	0.988	0.477	0.015	0.487	70894	Efcab3	chr11	+	NM_0010	P530	protein_cc	4921510J1	EF-hand ca	Efcab3
3839	Mettl2	1.274	0.817	1.041	0.001	0.017	0.714	7.317	7.666	7.375	52686	Mettl2	chr11	+	NM_1725	P20015	protein_cc	2810438F	methyltra	Mettl2
3840	Tlk2	1.708	0.686	1.172	0.000	0.012	0.488	8.192	8.964	8.421	24086	Tlk2	chr11	+	NM_0012	P22156	protein_cc	4933403M	tousled-lik	Tlk2
3841	1700052K	3.417	0.670	2.290	0.000	0.067	0.205	4.122	5.895	5.317	73431	1700052K	chr11	-	NR_02795	.	lncRNA	AI840954	RIKEN cDN	1700052K1
3842	Mrc2	0.148	3.555	0.525	0.106	0.000	0.553	2.850	0.092	1.921	17534	Mrc2	chr11	+	NM_0086	P13256	protein_cc	Endo180|	mannose	Mrc2
3843	March10	1.000	1.000	1.000	1.000	1.000	1.000	0.000	0.000	0.000	632687	March10	chr11	-	NM_1725	P576	protein_cc	4933417C	membran	March10
3844	Tanc2	1.111	1.603	1.782	0.677	0.080	0.271	5.384	5.536	6.217	77097	Tanc2	chr11	+	NM_1810	P17823	protein_cc	3526402J	tetratricop	Tanc2
3845	Cyb561	12.122	1.201	14.554	0.000	0.116	0.015	4.493	8.093	8.356	13056	Cyb561	chr11	-	NM_0078	P18614	protein_cc	.	cytochrom	Cyb561
3846	Gm11651	1.000	1.000	1.000	1.000	1.000	1.000	0.000	0.000	0.000	10037961	Gm11651	chr11	-	NR_10404	.	lncRNA	OTTMUSG	predicted	Gm11651
3847	Ace	0.908	2.878	2.612	0.700	0.019	0.160	1.667	1.528	3.053	11421	Ace	chr11	+	NM_2076	P21086	protein_cc	AW20857	angiotensi	Ace
3848	Ace3	1.000	1.000	1.000	1.000	1.000	1.000	0.000	0.000	0.000	217246	Ace3	chr11	+	NM_0011	P22966	protein_cc	EG217246	angiotensi	Ace3
3849	Kcnh6	1.000	1.000	1.000	1.000	1.000	1.000	0.000	0.000	0.000	192775	Kcnh6	chr11	+	NM_0010	P25426	protein_cc	m-erg2	potassium	Kcnh6
3850	Dcaf7	1.040	1.091	1.134	0.708	0.318	0.273	7.712	7.768	7.893	71833	Dcaf7	chr11	+	NM_0279	P23351	protein_cc	1700012F	DDB1 and	Dcaf7
3851	Taco1	0.894	0.865	0.773	0.724	0.430	0.658	5.805	5.643	5.433	70207	Taco1	chr11	+	NM_0273	P11520	protein_cc	2310066I1	translation	Taco1
3852	Map3k3	1.417	1.073	1.520	0.017	0.602	0.169	7.049	7.552	7.654	26406	Map3k3	chr11	+	NM_0119	P12598	protein_cc	AW54891	mitogen-a	Map3k3
3853	Limd2	0.537	1.118	0.600	0.000	0.429	0.098	8.416	7.518	7.679	67803	Limd2	chr11	-	NM_1723	P3640	protein_cc	0610025L	LIM doma	Limd2
3854	Strada	1.184	0.712	0.843	0.588	0.069	0.782	3.967	4.210	3.721	72149	Strada	chr11	-	NM_0281	P14007	protein_cc	2610019A	STE20-rela	Strada
3855	Ccdc47	1.410	0.755	1.065	0.002	0.002	0.564	9.683	10.179	9.774	67163	Ccdc47	chr11	-	NM_0260	P8742	protein_cc	2610204L	coiled-coil	Ccdc47
3856	Ddx42	1.359	0.819	1.113	0.145	0.270	0.389	8.083	8.526	8.238	72047	Ddx42	chr11	+	NM_0280	P17527	protein_cc	1810047H	DEAD (Asp	Ddx42
3857	Ftsj3	2.079	1.451	3.016	0.011	0.020	0.004	8.438	9.494	10.030	56095	Ftsj3	chr11	-	NM_0253	P20600	protein_cc	AA537063	FtsJ RNA r	Ftsj3
3858	Psmc5	1.221	1.045	1.276	0.012	0.723	0.384	8.768	9.056	9.120	19184	Psmc5	chr11	+	NM_0089	P10178	protein_cc	mSUG1	protease (	Psmc5
3859	Smarcd2	0.464	1.024	0.475	0.005	0.872	0.059	9.778	8.670	8.704	83796	Smarcd2	chr11	-	NM_0318	P24881	protein_cc	AW32245	SWI/SNF r	Smarcd2
3860	Tcam1	1.000	1.000	1.000	1.000	1.000	1.000	0.000	0.000	0.000	75870	Tcam1	chr11	+	NM_0294	P14676	protein_cc	4930570F	testicular	Tcam1
3861	Gh	1.000	1.000	1.000	1.000	1.000	1.000	0.000	0.000	0.000	14599	Gh	chr11	-	NM_0081	P14110	protein_cc	Gh1| Ghb	growth ho	Gh
3862	Cd79b	0.780	0.334	0.261	0.004	0.012	0.068	3.926	3.567	1.986	15985	Cd79b	chr11	-	NM_0083	P26027	protein_cc	B29| Ig-be	CD79B ant	Cd79b
3863	Scn4a	1.000	1.000	1.000	1.000	1.000	1.000	0.000	0.000	0.000	110880	Scn4a	chr11	-	NM_1331	P9986	protein_cc	mH2| Nav	sodium ch	Scn4a
3864	Prr29	0.750	2.619	1.964	0.200	0.183	0.524	0.448	0.033	1.422	75573	Prr29	chr11	+	NM_0293	P24304	protein_cc	2310007L	proline ric	Prr29
3865	Icam2	2.259	0.759	1.715	0.005	0.049	0.245	5.699	6.875	6.477	15896	Icam2	chr11	-	NM_0104	P2149	protein_cc	CD102| Ic	intercellul	Icam2
3866	Ern1	1.305	1.465	1.912	0.488	0.129	0.206	2.864	3.248	3.799	78943	Ern1	chr11	-	NM_0239	P22730	protein_cc	9030414B	endoplasn	Ern1
3867	Snord104	2.553	0.552	1.409	0.000	0.000	0.035	5.736	7.089	6.231	10021653	Snord104	chr11	+	NR_03070	.	snoRNA	.	small nucl	Snord104
3868	Tex2	0.496	0.768	0.381	0.000	0.002	0.011	9.718	8.706	8.325	21763	Tex2	chr11	-	NM_1982	P20791	protein_cc	4930568E	testis expr	Tex2
3869	Pecam1	1.515	0.688	1.042	0.276	0.298	0.936	7.784	8.383	7.843	18613	Pecam1	chr11	-	NM_0088	P20308	protein_cc	C85791| C	platelet/e	Pecam1
3870	Milr1	2.864	0.901	2.579	0.091	0.754	0.049	3.518	5.036	4.885	380732	Milr1	chr11	+	NM_0012	P18456	protein_cc	Allergin-1|	mast cell i	Milr1
3871	Polg2	0.216	0.800	0.173	0.000	0.330	0.006	5.276	3.066	2.745	50776	Polg2	chr11	-	NM_0158	P25070	protein_cc	.	polymeras	Polg2
3872	Ddx5	1.398	0.764	1.069	0.030	0.019	0.675	10.739	11.222	10.834	13207	Ddx5	chr11	-	NM_0078	P24084	protein_cc	2600009A	DEAD (Asp	Ddx5
3873	Cep95	0.767	0.607	0.466	0.151	0.057	0.163	6.093	5.711	4.991	320162	Cep95	chr11	+	NM_0011	P6649	protein_cc	4732496G	centrosom	Cep95
3874	Smurf2	1.212	0.845	1.025	0.158	0.219	0.927	7.882	8.160	7.918	66313	Smurf2	chr11	-	NM_0254	P10419	protein_cc	2810411E	SMAD spe	Smurf2
3875	Kpna2	1.594	1.106	1.764	0.108	0.620	0.178	6.193	6.866	7.012	16647	Kpna2	chr11	-	NM_0106	P17168	protein_cc	2410044B	karyopher	Kpna2
3876	1810010H	0.191	2.602	0.497	0.010	0.043	0.191	4.803	2.414	3.794	69066	1810010H	chr11	+	NM_0011	P6440	protein_cc	OTTMUSG	RIKEN cDN	1810010H
3877	Bptf	1.319	0.595	0.785	0.012	0.002	0.067	9.320	9.719	8.971	207165	Bptf	chr11	-	NM_1768	P20006	protein_cc	9430093H	bromodon	Bptf
3878	Gm11715	1.577	1.303	2.056	0.140	0.208	0.063	6.266	6.924	7.306	10573473	Gm11715	chr11	+	NM_0013	P3103	protein_cc	OTTMUSG	predicted	Gm11715
3879	Nol11	1.846	1.119	2.066	0.048	0.520	0.051	6.912	7.797	7.959	68979	Nol11	chr11	-	NM_0011	P174	protein_cc	1500002M	nucleolar	Nol11
3880	Pitpnc1	0.760	0.750	0.570	0.000	0.003	0.015	9.149	8.753	8.338	71795	Pitpnc1	chr11	-	NM_1458	P3112	protein_cc	1110020B	phosphati	Pitpnc1
3881	Psmd12	1.422	1.015	1.444	0.036	0.882	0.012	9.227	9.735	9.757	66997	Psmd12	chr11	+	NM_0258	P18214	protein_cc	1500002F	proteason	Psmd12
3882	Helz	1.128	0.809	0.912	0.169	0.047	0.034	6.704	6.878	6.571	78455	Helz	chr11	+	NM_1982	P11081	protein_cc	3110078M	helicase w	Helz
3883	Cacng1	1.144	1.676	1.916	0.746	0.271	0.496	1.314	1.508	2.253	12299	Cacng1	chr11	-	NM_0075	P24156	protein_cc	.	calcium ch	Cacng1
3884	Cacng4	1.000	1.000	1.000	1.000	1.000	1.000	0.000	0.000	0.000	54377	Cacng4	chr11	-	NM_0194	P26648	protein_cc	AI413107|	calcium ch	Cacng4
3885	Cacng5	1.000	1.000	1.000	1.000	1.000	1.000	0.000	0.000	0.000	140723	Cacng5	chr11	-	NM_0011	P6152	protein_cc	.	calcium ch	Cacng5
3886	Prkca	0.245	1.668	0.409	0.000	0.092	0.032	7.402	5.374	6.113	18750	Prkca	chr11	-	NM_0111	P23992	protein_cc	AI875142|	protein kir	Prkca
3887	Apoh	0.429	0.778	0.334	0.002	0.228	0.030	14.109	12.889	12.527	11818	Apoh	chr11	+	NM_0134	P4862	protein_cc	B2GPI| be	apolipopro	Apoh
3888	Cep112	0.114	2.204	0.251	0.006	0.114	0.104	4.676	1.543	2.683	76380	Cep112	chr11	+	NM_0296	P9468	protein_cc	1700001M	centrosom	Cep112
3889	Axin2	0.075	0.614	0.046	0.000	0.133	0.002	5.574	1.841	1.137	12006	Axin2	chr11	+	NM_0157	P8406	protein_cc	Axi1| Axil|	axin 2	Axin2
3890	E030025P	1.000	1.000	1.000	1.000	1.000	1.000	0.000	0.000	0.000	268498	E030025P	chr11	-	NR_03797	.	lncRNA	Gm664	RIKEN cDN	E030025P0
3891	Rgs9	2.959	0.165	0.487	0.019	0.010	0.372	3.148	4.713	2.111	19739	Rgs9	chr11	-	NM_0112	P19659	protein_cc	RGS9-1| R	regulator	Rgs9
3892	1700096J	1.000	1.000	1.000	1.000	1.000	1.000	0.000	0.000	0.000	67927	1700096J	chr11	+	NR_02788	.	lncRNA	.	RIKEN cDN	1700096J1
3893	Gm11696	2.277	0.354	0.806	0.000	0.001	0.492	1.609	2.797	1.299	10003676	Gm11696	chr11	-	NR_03809	.	lncRNA	OTTMUSG	predicted	Gm11696
3894	Gna13	4.120	0.480	1.977	0.001	0.009	0.174	8.492	10.535	9.475	14674	Gna13	chr11	+	NM_0103	P16413	protein_cc	AU024132	guanine nu	Gna13
3895	Amz2	1.785	0.787	1.405	0.001	0.031	0.084	6.898	7.734	7.389	13929	Amz2	chr11	+	NM_0252	P839	protein_cc	AA408420	archaelysi	Amz2
3896	LOC10050	7.955	0.557	4.428	0.001	0.062	0.200	6.209	9.201	8.356	10050349	LOC10050	chr11	+	NR_04068	.	lncRNA	.	uncharact	LOC10050
3897	Slc16a6	9.508	0.469	4.463	0.000	0.017	0.214	7.134	10.383	9.292	104681	Slc16a6	chr11	-	NM_1340	P4635	protein_cc	AW74311	solute car	Slc16a6
3898	Arsg	0.618	2.428	1.501	0.005	0.001	0.123	6.320	5.626	6.906	74008	Arsg	chr11	+	NM_0287	P6076	protein_cc	6330406P	arylsulfata	Arsg
3899	Wipi1	1.416	1.658	2.348	0.000	0.054	0.139	6.739	7.241	7.971	52639	Wipi1	chr11	-	NM_1459	P8943	protein_cc	4930533H	WD repea	Wipi1
3900	Prkar1a	1.424	1.007	1.433	0.057	0.960	0.092	10.656	11.166	11.176	19084	Prkar1a	chr11	+	NM_0218	P17912	protein_cc	1300018C	protein kir	Prkar1a

3901	Fam20a	0.070	4.266	0.299	0.000	0.078	0.047	8.062	4.229	6.322	208659	Fam20a	chr11	-	NM_1537	P3437	protein_cc	AI606893	family wit	Fam20a
3902	1700012B	1.000	1.000	1.000	1.000	1.000	1.000	0.000	0.000	0.000	69324	1700012B	chr11	-	NM_0011	P1244	protein_cc	1700069D	RIKEN cDN	1700012B
3903	1700023C	1.000	1.000	1.000	1.000	1.000	1.000	0.000	0.000	0.000	73257	1700023C	chr11	+	NR_04590	.	lncRNA		RIKEN cDN	1700023C
3904	Abca8b	0.237	0.502	0.119	0.000	0.083	0.009	6.554	4.477	3.482	27404	Abca8b	chr11	-	NM_0138	P23240	protein_cc	Abca8	ATP-bindi	Abca8b
3905	Abca8a	0.075	0.341	0.025	0.000	0.003	0.010	10.228	6.483	4.930	217258	Abca8a	chr11	-	NM_1531	P7298	protein_cc	.	ATP-bindi	Abca8a
3906	Abca9	0.063	2.038	0.127	0.004	0.206	0.080	4.128	0.129	1.156	217262	Abca9	chr11	-	NM_1472	P15545	protein_cc	D630040K	ATP-bindi	Abca9
3907	Abca6	0.145	4.672	0.679	0.000	0.020	0.311	9.337	6.556	8.780	76184	Abca6	chr11	-	NM_1472	P15606	protein_cc	6330565N	ATP-bindi	Abca6
3908	Abca5	0.739	1.499	1.107	0.347	0.459	0.908	4.603	4.166	4.749	217265	Abca5	chr11	-	NM_1472	P14802	protein_cc	ABC13| B!	ATP-bindi	Abca5
3909	Map2k6	0.039	2.126	0.083	0.000	0.134	0.007	6.501	1.829	2.917	26399	Map2k6	chr11	+	NM_0119	P3017	protein_cc	MEK6| MI	mitogen-a	Map2k6
3910	Kcnj16	0.151	0.342	0.052	0.065	1.000	0.216	4.270	1.547	0.000	16517	Kcnj16	chr11	+	NM_0106	P12868	protein_cc	6430410F	potassium	Kcnj16
3911	Kcnj2	7.058	0.919	6.484	0.055	0.812	0.026	1.240	4.059	3.937	16518	Kcnj2	chr11	+	NM_0084	P25813	protein_cc	IRK1| Kcni	potassium	Kcnj2
3912	BC006965	0.405	0.985	0.399	0.181	1.000	0.423	1.324	0.022	0.000	217294	BC006965	chr11	-	NR_02408	.	lncRNA		cDNA seq	BC006965
3913	Sox9	8.739	0.625	5.463	0.000	0.118	0.235	6.618	9.745	9.067	20682	Sox9	chr11	+	NM_0114	P25086	protein_cc	2010306G	SRY (sex d	Sox9
3914	Gm11681	1.000	1.000	1.000	1.000	1.000	1.000	0.000	0.000	0.000	10003873	Gm11681	chr11	-	NR_12607	.	lncRNA	.	predicted	Gm11681
3915	1700012H	1.000	1.000	1.000	1.000	1.000	1.000	0.000	0.000	0.000	76393	1700012H	chr11	-	NR_13016	.	lncRNA	.	RIKEN cDN	1700012H
3916	2610035D	0.404	1.753	0.707	0.075	0.218	0.475	3.550	2.241	3.051	72386	2610035D	chr11	-	NR_01555	.	lncRNA	.	RIKEN cDN	2610035D
3917	4732490B	1.000	1.000	1.000	1.000	1.000	1.000	0.000	0.000	0.000	319871	4732490B	chr11	-	NR_04027	.	lncRNA	.	RIKEN cDN	4732490B
3918	Slc39a11	0.438	1.838	0.805	0.000	0.003	0.141	9.134	7.945	8.822	69806	Slc39a11	chr11	-	NM_0011	P23482	protein_cc	1810074D	solute car	Slc39a11
3919	Sstr2	0.386	1.392	0.538	0.182	0.197	0.546	1.405	0.033	0.509	20606	Sstr2	chr11	+	NM_0010	P7882	protein_cc	Smstr-2| S	somatosta	Sstr2
3920	Cog1	1.590	0.751	1.194	0.000	0.040	0.449	5.827	6.496	6.082	16834	Cog1	chr11	+	NM_0135	P18280	protein_cc	6030445I1	componer	Cog1
3921	Fam104a	1.367	0.967	1.323	0.125	0.830	0.244	9.660	10.111	10.064	28081	Fam104a	chr11	-	NM_1385	P6646	protein_cc	D11Wsu9	family wit	Fam104a
3922	D11Wsu4	2.681	0.553	1.483	0.001	0.000	0.231	5.033	6.456	5.602	227620	D11Wsu4	chr11	+	NM_1777	P22833	protein_cc	.	DNA segm	D11Wsu47
3923	Cpsf4l	0.101	0.778	0.078	0.004	0.710	0.065	6.043	2.730	2.367	52670	Cpsf4l	chr11	-	NM_0266	P8847	protein_cc	0610010C	cleavage a	Cpsf4l
3924	Cdc42ep4	0.689	1.594	1.099	0.005	0.000	0.318	10.122	9.585	10.258	56699	Cdc42ep4	chr11	-	NM_0011	P22754	protein_cc	1500041M	CDC42 eff	Cdc42ep4
3925	Sdk2	0.716	0.995	0.712	0.182	1.000	0.423	0.490	0.008	0.000	237979	Sdk2	chr11	-	NM_1728	P19587	protein_cc	4632412F	sidekick c	Sdk2
3926	1700092K	1.000	1.000	1.000	1.000	1.000	1.000	0.000	0.000	0.000	73536	1700092K	chr11	+	NR_04593	.	lncRNA	.	RIKEN cDN	1700092K
3927	4932435O	1.000	1.000	1.000	1.000	1.000	1.000	0.000	0.000	0.000	319819	4932435O	chr11	-	NR_02764	.	lncRNA	.	RIKEN cDN	4932435O
3928	Rpl38	1.262	1.405	1.773	0.020	0.001	0.005	9.533	9.868	10.360	67671	Rpl38	chr11	+	NM_0233	P6495	protein_cc	0610025G	ribosomal	Rpl38
3929	Ttyh2	0.362	1.600	0.580	0.003	0.245	0.307	7.811	6.347	7.025	117160	Ttyh2	chr11	+	NM_0532	P2945	protein_cc	1110001A	tweety fa	Ttyh2
3930	Dnaic2	1.000	1.000	1.000	1.000	1.000	1.000	0.000	0.000	0.000	432611	Dnaic2	chr11	+	NM_0010	P22522	protein_cc	C030015H	dynein, ax	Dnaic2
3931	Kif19a	1.000	1.000	1.000	1.000	1.000	1.000	0.000	0.000	0.000	286942	Kif19a	chr11	+	NM_0011	P5666	protein_cc	Kif19	kinesin fa	Kif19a
3932	Btbd17	1.580	0.633	1.000	0.305	1.000	0.423	0.000	0.660	0.000	72014	Btbd17	chr11	-	NM_0280	P4674	protein_cc	1500005I	BTB (POZ)	Btbd17
3933	Gpr142	1.000	1.000	1.000	1.000	1.000	1.000	0.000	0.000	0.000	217302	Gpr142	chr11	+	NM_1817	P12720	protein_cc	PGR2	G protein-	Gpr142
3934	Gprc5c	0.127	2.921	0.370	0.000	0.000	0.022	7.570	4.590	6.136	70355	Gprc5c	chr11	+	NM_0011	P19318	protein_cc	1110028I	G protein-	Gprc5c
3935	Cd300a	1.183	0.903	1.068	0.509	0.308	0.888	4.663	4.906	4.759	217303	Cd300a	chr11	+	NM_1707	P25459	protein_cc	B230315M	CD300A m	Cd300a
3936	Cd300lb	0.668	0.930	0.621	0.520	0.919	0.436	2.574	1.992	1.887	217304	Cd300lb	chr11	-	NM_1992	P18104	protein_cc	CLM-7| Cl	CD300 mo	Cd300lb
3937	Cd300c	2.134	0.469	1.000	0.305	1.000	0.423	0.000	1.094	0.000	387565	Cd300c	chr11	-	NM_1992	P1664	protein_cc	Clm6	CD300C m	Cd300c
3938	Cd300ld	0.255	0.972	0.248	0.001	0.908	0.047	6.447	4.474	4.434	217305	Cd300ld	chr11	-	NM_1454	P23987	protein_cc	4732429D	CD300 mo	Cd300ld
3939	AF251705	0.308	0.413	0.127	0.001	0.115	0.012	5.039	3.338	2.063	.	AF251705	chr11	-	NM_1341	P6736	.	.	.	AF251705
3940	Gm11710	0.716	0.995	0.712	0.182	1.000	0.423	0.490	0.008	0.000	.	Gm11710	chr11	-	NM_0011	P748	.	.	.	Gm11710
3941	Gm11711	1.000	1.000	1.000	1.000	1.000	1.000	0.000	0.000	0.000	.	Gm11711	chr11	-	NM_0011	P748	.	.	.	Gm11711
3942	Cd300lh	1.000	1.000	1.000	1.000	1.000	1.000	0.000	0.000	0.000	.	Cd300lh	chr11	-	NM_1992	P2983	.	.	.	Cd300lh
3943	Cd300e	2.328	0.620	1.442	0.003	0.128	0.620	3.242	4.461	3.771	217306	Cd300e	chr11	-	NM_1720	P24251	protein_cc	BC034097	CD300E m	Cd300e
3944	Rab37	38.255	0.689	26.365	0.000	0.075	0.049	0.003	5.260	4.723	58222	Rab37	chr11	+	NM_0011	P6435	protein_cc	B230331O	RAB37, m	Rab37
3945	Cd300lf	43.419	1.010	43.849	0.021	0.974	0.027	2.920	8.360	8.374	246746	Cd300lf	chr11	-	NM_0011	P10049	protein_cc	CLIM1| CL	CD300 mo	Cd300lf
3946	Slc9a3r1	3.781	0.669	2.529	0.000	0.014	0.086	9.895	11.813	11.233	26941	Slc9a3r1	chr11	+	NM_0120	P9308	protein_cc	EBP-50| N	solute car	Slc9a3r1
3947	Nat9	0.380	1.644	0.625	0.017	0.104	0.308	6.796	5.401	6.118	66176	Nat9	chr11	-	NM_0254	P13317	protein_cc	1110028N	N-acetyltr	Nat9
3948	Tmem104	0.491	1.467	0.721	0.013	0.067	0.341	6.635	5.610	6.163	320534	Tmem104	chr11	+	NM_0010	P4205	protein_cc	C630005D	transmem	Tmem104
3949	Grin2c	1.000	1.000	1.000	1.000	1.000	1.000	0.000	0.000	0.000	14813	Grin2c	chr11	-	NM_0103	P22308	protein_cc	GluN2C| N	glutamate	Grin2c
3950	Fdxr	0.330	1.117	0.368	0.002	0.684	0.022	7.023	5.423	5.582	14149	Fdxr	chr11	-	NM_0079	P2717	protein_cc	.	ferredoxin	Fdxr
3951	Fads6	0.956	0.689	0.659	0.485	0.411	0.588	3.704	3.639	3.102	328035	Fads6	chr11	-	NM_1780	P3774	protein_cc	BC044804	fatty acid	Fads6
3952	Otop2	1.000	1.000	1.000	1.000	1.000	1.000	0.000	0.000	0.000	237987	Otop2	chr11	+	NM_1728	P9760	protein_cc	4732464P	otopetrin	Otop2
3953	Ush1g	0.271	3.270	0.885	0.069	0.185	0.897	1.953	0.067	1.776	16470	Ush1g	chr11	-	NM_1768	P15610	protein_cc	js| Sans	Usher syn	Ush1g
3954	Otop3	1.000	1.000	1.000	1.000	1.000	1.000	0.000	0.000	0.000	69602	Otop3	chr11	+	NM_0271	P10666	protein_cc	2310011E	otopetrin	Otop3
3955	Hid1	0.634	1.121	0.711	0.033	0.710	0.507	4.672	4.014	4.179	217310	Hid1	chr11	-	NM_1754	P16603	protein_cc	C630004H	HID1 dom	Hid1
3956	Cdr2l	0.768	2.126	1.633	0.519	0.021	0.391	4.857	4.477	5.565	237988	Cdr2l	chr11	+	NM_0010	P22433	protein_cc	D030068L	cerebellar	Cdr2l
3957	Ict1	0.768	1.349	1.035	0.000	0.000	0.418	8.884	8.502	8.934	68572	Ict1	chr11	+	NM_0267	P1932	protein_cc	1110001A	immature	Ict1
3958	Atp5h	0.657	1.125	0.739	0.001	0.107	0.020	11.801	11.195	11.364	71679	Atp5h	chr11	-	NM_0278	P5116	protein_cc	0610009D	ATP synth	Atp5h
3959	Kctd2	0.363	1.462	0.530	0.004	0.000	0.120	8.250	6.786	7.335	70382	Kctd2	chr11	+	NM_1832	P12193	protein_cc	2310012I1	potassium	Kctd2
3960	Trim80	0.134	2.541	0.339	0.085	0.191	0.421	2.995	0.091	1.436	432613	Trim80	chr11	+	NM_0010	P3505	protein_cc	4933422H	tripartite	Trim80
3961	Slc16a5	0.019	1.833	0.035	0.000	0.051	0.000	6.504	0.813	1.686	217316	Slc16a5	chr11	+	NM_0010	P22001	protein_cc	A130015N	solute car	Slc16a5
3962	Armc7	0.921	1.518	1.397	0.600	0.027	0.084	6.818	6.698	7.301	276905	Armc7	chr11	+	NM_1777	P7309	protein_cc	A630084C	armadillo	Armc7
3963	Nt5c	0.569	1.775	1.009	0.021	0.003	0.968	8.134	7.319	8.147	50773	Nt5c	chr11	-	NM_0158	P4486	protein_cc	Dnt| Dnt1	5',3'-nucle	Nt5c
3964	Hn1	2.002	1.246	2.494	0.008	0.294	0.127	6.436	7.438	7.755	15374	Hn1	chr11	-	NM_0082	P11134	protein_cc	.	hematolog	Hn1
3965	Sumo2	1.722	0.750	1.292	0.003	0.019	0.168	6.531	7.315	6.900	170930	Sumo2	chr11	-	NM_1333	P7205	protein_cc	Smt3A| Sr	small ubiq	Sumo2
3966	Nup85	0.703	1.099	0.773	0.005	0.126	0.140	7.611	7.103	7.240	445007	Nup85	chr11	+	NM_0010	P6629	protein_cc	frount| Pc	nucleopor	Nup85
3967	Gga3	2.256	0.474	1.069	0.044	0.050	0.584	5.719	6.893	5.815	260302	Gga3	chr11	-	NM_1730	P1777	protein_cc	C230037M	golgi asso	Gga3
3968	Mrps7	0.910	1.161	1.057	0.128	0.020	0.329	9.043	8.907	9.122	50529	Mrps7	chr11	+	NM_0253	P5141	protein_cc	MRP-S7| F	mitochondr	Mrps7
3969	Mif4gd	1.687	0.888	1.498	0.001	0.051	0.004	9.480	10.235	10.064	69674	Mif4gd	chr11	-	NM_0271	P651	protein_cc	1110014L	MIF4G do	Mif4gd
3970	Slc25a19	1.097	1.202	1.318	0.488	0.374	0.517	6.456	6.590	6.855	67283	Slc25a19	chr11	-	NM_0012	P4579	protein_cc	2900089E	solute car	Slc25a19
3971	Grb2	1.177	0.777	0.915	0.028	0.017	0.397	9.565	9.801	9.437	14784	Grb2	chr11	-	NM_0081	P155	protein_cc	AA408164	growth fa	Grb2
3972	2310067B	1.154	1.020	1.178	0.001	0.947	0.776	6.594	6.801	6.830	.	2310067B	chr11	-	NM_0280	P4082	.	.	.	2310067B
3973	Caskin2	2.538	0.894	2.268	0.008	0.550	0.125	4.844	6.187	6.025	140721	Caskin2	chr11	-	NM_0806	P9651	protein_cc	1600028L	CASK-inte	Caskin2
3974	Tsen54	1.321	1.357	1.793	0.073	0.257	0.330	4.770	5.171	5.612	76265	Tsen54	chr11	+	NM_0295	P19588	protein_cc	0610034P	tRNA splic	Tsen54
3975	Llgl2	0.653	0.832	0.543	0.193	0.621	0.192	7.694	7.078	6.813	217325	Llgl2	chr11	+	NM_0012	P13207	protein_cc	9130006H	lethal gian	Llgl2
3976	Recql5	0.665	1.338	0.889	0.419	0.615	0.746	5.365	4.777	5.196	170472	Recql5	chr11	-	NM_1304	P24335	protein_cc	RecQ5| Re	RecQ prot	Recql5
3977	Smim5	0.219	4.367	0.958	0.127	0.041	0.962	2.271	0.082	2.209	66528	Smim5	chr11	+	NM_1832	P3213	protein_cc	2210020M	small inte	Smim5
3978	Smim6	0.347	1.060	0.368	0.005	0.826	0.038	4.591	3.064	3.148	68528	Smim6	chr11	+	NM_0011	P13382	protein_cc	1110017F	small inte	Smim6
3979	Sap30bp	2.453	1.228	3.013	0.112	0.510	0.067	5.724	7.019	7.315	57230	Sap30bp	chr11	+	NM_0204	P9731	protein_cc	2700016D	SAP30 bin	Sap30bp
3980	Itgb4	0.296	1.172	0.347	0.102	0.822	0.305	3.792	2.037	2.266	192897	Itgb4	chr11	+	NM_0010	P21734	protein_cc	AA407042	integrin b	Itgb4
3981	Galk1	0.448	1.700	0.762	0.003	0.012	0.228	8.640	7.483	8.248	14635	Galk1	chr11	-	NM_0169	P11602	protein_cc	AA409894	galactokin	Galk1
3982	H3f3b	5.188	0.982	5.092	0.000	0.960	0.295	10.909	13.284	13.257	15081	H3f3b	chr11	+	NM_0082	P23782	protein_cc	9430068D	H3 histone	H3f3b
3983	Unk	2.769	0.517	1.431	0.001	0.036	0.574	5.217	6.686	5.734	217331	Unk	chr11	+	NM_1725	P5060	protein_cc	B230379M	unkempt f	Unk
3984	Unc13d	1.631	1.320	2.153	0.368	0.608	0.524	1.678	2.384	2.784	70450	Unc13d	chr11	-	NM_0010	P16374	protein_cc	2610108D	unc-13 ho	Unc13d
3985	Wbp2	0.747	1.162	0.868	0.083	0.191	0.413	10.085	9.665	9.882	22378	Wbp2	chr11	-	NM_0168	P5299	protein_cc	.	WW doma	Wbp2
3986	Trim47	5.568	0.479	2.670	0.000	0.002	0.098	5.837	8.314	7.253	217333	Trim47	chr11	-	NM_0012	P12001	protein_cc	2210023F	tripartite	Trim47
3987	Trim65	0.864	2.150	1.857	0.863	0.278	0.514	2.748	2.536	3.641	338364	Trim65	chr11	-	NM_1788	P1283	protein_cc	4732463G	tripartite	Trim65
3988	Mrpl38	0.831	1.166	0.968	0.000	0.531	0.932	8.586	8.318	8.540	60441	Mrpl38	chr11	-	NM_0241	P7136	protein_cc	1110036N	mitochond	Mrpl38
3989	Fbf1	0.542	2.195	1.190	0.039	0.060	0.617	4.725	3.841	4.976	217335	Fbf1	chr11	-	NM_1725	P25832	protein_cc	1110033G	Fas (TNFR	Fbf1
3990	Acox1	0.707	1.205	0.852	0.017	0.390	0.610	12.543	12.044	12.313	11430	Acox1	chr11	-	NM_0157	P20342	protein_cc	Acox| AO	acyl-Coen	Acox1
3991	Ten1	1.138	1.566	1.782	0.684	0.118	0.131	4.595	4.782	5.429	69535	Ten1	chr11	+	NM_0271	P8396	protein_cc	2310004N	TEN1 telo	Ten1

3992	Cdk3-ps	1.000	1.000	1.000	1.000	1.000	1.000	0.000	0.000	0.000	69681	Cdk3-ps	chr11	+	NR_00485	.	misc_RNA	2310050O	cyclin-dep	Cdk3-ps
3993	Evpl	0.772	0.537	0.415	0.648	0.493	0.106	2.137	1.764	0.868	14027	Evpl	chr11	-	NM_0252	P3591	protein_cc	.	envoplakir	Evpl
3994	Srp68	1.341	0.931	1.248	0.003	0.170	0.025	8.723	9.147	9.043	217337	Srp68	chr11	-	NM_1460	P3599	protein_cc	2610024I0	signal recc	Srp68
3995	Galr2	0.439	0.985	0.432	0.002	1.000	0.051	1.210	0.021	0.000	14428	Galr2	chr11	+	NM_0102	P19441	protein_cc	mGalR	galanin re	Galr2
3996	Exoc7	0.584	1.515	0.884	0.009	0.034	0.353	7.871	7.094	7.693	53413	Exoc7	chr11	-	NM_0168	P1780	protein_cc	Exo70| se	exocyst cc	Exoc7
3997	Foxj1	0.457	1.401	0.640	0.183	0.194	0.600	1.158	0.027	0.514	15223	Foxj1	chr11	-	NM_0082	P15238	protein_cc	FKHL-13|	forkhead b	Foxj1
3998	Rnf157	0.164	0.778	0.127	0.006	0.590	0.070	4.656	2.045	1.683	217340	Rnf157	chr11	-	NM_0272	P2910	protein_cc	2610036E	ring finger	Rnf157
3999	Ubald2	1.000	1.000	1.000	1.000	1.000	1.000	0.000	0.000	0.000	319370	Ubald2	chr11	+	NM_1769	P412	protein_cc	1110014K	UBA-like c	Ubald2
4000	Gm7367	0.571	1.369	0.782	0.005	0.093	0.391	7.694	6.885	7.339	664849	Gm7367	chr11	+	NR_00337	.	misc_RNA	EG664849	predicted	Gm7367
4001	Qrich2	1.000	1.000	1.000	1.000	1.000	1.000	0.000	0.000	0.000	217341	Qrich2	chr11	-	NM_0010	P6576	protein_cc	Gm66	glutamine	Qrich2
4002	Prpsap1	0.458	1.284	0.588	0.000	0.103	0.053	9.098	7.971	8.332	67763	Prpsap1	chr11	-	NM_0263	P765	protein_cc	5730409F	phosphori	Prpsap1
4003	Sphk1	81.842	0.576	47.163	0.000	0.047	0.141	0.330	6.685	5.890	20698	Sphk1	chr11	+	NM_0011	P25357	protein_cc	1110006G	sphingosir	Sphk1
4004	Ube2o	1.147	1.037	1.190	0.264	0.792	0.509	6.239	6.437	6.490	217342	Ube2o	chr11	-	NM_1737	P11861	protein_cc	9630022M	ubiquitin-	Ube2o
4005	Aanat	3.312	0.425	1.406	0.306	0.392	0.423	0.001	1.729	0.492	11298	Aanat	chr11	+	NM_0095	P24217	protein_cc	AA-NAT| N	arylalkylar	Aanat
4006	Rhbdf2	5.605	0.460	2.576	0.009	0.036	0.034	4.477	6.964	5.843	217344	Rhbdf2	chr11	-	NM_1725	P17615	protein_cc	4732465I1	rhomboid	Rhbdf2
4007	Cygb	0.226	2.366	0.535	0.000	0.107	0.197	7.414	5.270	6.512	114886	Cygb	chr11	-	NM_0302	P14081	protein_cc	3110001K	cytoglobir	Cygb
4008	Gm11744	0.558	0.835	0.466	0.204	0.762	0.236	1.981	1.140	0.880	10003857	Gm11744	chr11	-	NM_0011	P17822	protein_cc	OTTMUSG	predicted	Gm11744
4009	1810032O	0.616	1.825	1.125	0.159	0.072	0.620	3.945	3.247	4.115	66293	1810032O	chr11	+	NR_02782	.	lncRNA		RIKEN cDN	1810032O
4010	Snord1c	1.590	0.875	1.390	0.302	0.737	0.423	0.000	0.669	0.476	10021653	Snord1c	chr11	+	NR_02856	.	snoRNA	.	small nucl	Snord1c
4011	Snord1b	3.342	0.404	1.350	0.076	0.119	0.423	0.001	1.741	0.433	10021653	Snord1b	chr11	+	NR_02856	.	snoRNA	.	small nucl	Snord1b
4012	Snord1a	1.580	0.633	1.000	0.305	1.000	0.423	0.000	0.660	0.000	10021653	Snord1a	chr11	+	NR_02857	.	snoRNA	snR38	small nucl	Snord1a
4013	St6galnac	0.751	0.156	0.117	0.305	0.305	0.038	3.096	2.683	0.000	20446	St6galnac	chr11	-	NM_0091	P716	protein_cc	II| Siat7| S	ST6 (alpha	St6galnac2
4014	BC018473	1.000	1.000	1.000	1.000	1.000	1.000	0.000	0.000	0.000	193217	BC018473	chr11	+	NR_00336	.	lncRNA	.	cDNA seq	BC018473
4015	St6galnac	0.552	0.990	0.547	0.182	1.000	0.423	0.871	0.015	0.000	20445	St6galnac	chr11	-	NM_0113	P5782	protein_cc	Siat7a	ST6 (alpha	St6galnac1
4016	Mxra7	0.115	3.050	0.351	0.001	0.103	0.119	4.352	1.233	2.842	67622	Mxra7	chr11	-	NM_0262	P816	protein_cc	1810057P	matrix-rer	Mxra7
4017	Jmjd6	2.730	0.679	1.854	0.001	0.013	0.030	7.643	9.092	8.534	107817	Jmjd6	chr11	-	NM_0333	P18627	protein_cc	5730436I2	jumonji dc	Jmjd6
4018	Mettl23	0.664	0.973	0.646	0.006	0.931	0.402	7.767	7.176	7.136	74319	Mettl23	chr11	+	NM_0288	P21924	protein_cc	1110005A	methyltra	Mettl23
4019	Srsf2	1.434	1.042	1.494	0.002	0.690	0.153	9.547	10.068	10.126	20382	Srsf2	chr11	-	NM_0113	P5812	protein_cc	D11Wsu1	serine/arg	Srsf2
4020	Mfsd11	0.928	0.790	0.733	0.132	0.074	0.203	6.844	6.736	6.396	69900	Mfsd11	chr11	+	NM_1786	P5371	protein_cc	2600014M	major faci	Mfsd11
4021	Mgat5b	1.000	1.000	1.000	1.000	1.000	1.000	0.000	0.000	0.000	268510	Mgat5b	chr11	+	NM_1729	P26329	protein_cc	C330018B	mannosid	Mgat5b
4022	Snhg20	1.214	0.585	0.710	0.671	0.337	0.133	5.085	5.364	4.590	76972	Snhg20	chr11	+	NR_02705	.	lncRNA	2810008D	small nucl	Snhg20
4023	Sec14l1	0.919	0.520	0.478	0.276	0.061	0.213	7.957	7.836	6.892	74136	Sec14l1	chr11	+	NM_0011	P5419	protein_cc	1200017E	SEC14-like	Sec14l1
4024	Sept9	0.361	2.124	0.767	0.001	0.007	0.082	6.287	4.819	5.906	53860	Sept9	chr11	+	NM_0011	P22214	protein_cc	Msf| MSF	septin 9	Sept9
4025	Gm11729	1.000	1.000	1.000	1.000	1.000	1.000	0.000	0.000	0.000	10582774	Gm11729	chr11	+	NR_13209	.	lncRNA	OTTMUSG	predicted	Gm11729
4026	Gm11733	1.000	1.000	1.000	1.000	1.000	1.000	0.000	0.000	0.000	432615	Gm11733	chr11	+	NR_13096	.	lncRNA	OTTMUSG	predicted	Gm11733
4027	2900041M	1.000	1.000	1.000	1.000	1.000	1.000	0.000	0.000	0.000	78403	2900041M	chr11	+	NR_01548	.	lncRNA		RIKEN cDN	2900041M
4028	Tnrc6c	0.781	0.880	0.687	0.128	0.514	0.265	7.493	7.136	6.951	217351	Tnrc6c	chr11	+	NM_1980	P11246	protein_cc	9930033H	trinucleoti	Tnrc6c
4029	Tmc6	0.187	3.450	0.643	0.000	0.001	0.096	5.384	2.961	4.748	217353	Tmc6	chr11	-	NM_1454	P24119	protein_cc	D11Ertd2C	transmem	Tmc6
4030	Tmc8	0.219	1.762	0.385	0.025	0.112	0.226	2.946	0.752	1.569	217356	Tmc8	chr11	+	NM_0011	P16326	protein_cc	Ever2| EV	transmem	Tmc8
4031	6030468B	1.000	1.000	1.000	1.000	1.000	1.000	0.000	0.000	0.000	77727	6030468B	chr11	+	NM_0299	P20889	protein_cc	.	RIKEN cDN	6030468B
4032	Syngr2	0.612	1.389	0.849	0.005	0.030	0.330	9.142	8.432	8.906	20973	Syngr2	chr11	+	NM_0093	P20289	protein_cc	cellugyrin|	synaptogy	Syngr2
4033	Tk1	0.047	1.114	0.053	0.000	0.678	0.009	8.840	4.437	4.593	21877	Tk1	chr11	-	NM_0012	P18460	protein_cc	D530002A	thymidine	Tk1
4034	Afmid	0.133	0.751	0.100	0.000	0.272	0.005	12.216	9.310	8.897	71562	Afmid	chr11	+	NM_0278	P1796	protein_cc	9030621K	arylformar	Afmid
4035	Birc5	0.477	1.259	0.600	0.000	0.390	0.204	4.572	3.504	3.836	11799	Birc5	chr11	+	NM_0010	P20451	protein_cc	AAC-11| A	baculovira	Birc5
4036	Tmem235	1.000	1.000	1.000	1.000	1.000	1.000	0.000	0.000	0.000	546519	Tmem235	chr11	+	NM_0010	P10453	protein_cc	Cldn27| G	transmem	Tmem235
4037	Tha1	2.117	1.880	3.981	0.000	0.043	0.103	4.506	5.589	6.500	71776	Tha1	chr11	-	NM_0279	P8793	protein_cc	1300017K	threonine	Tha1
4038	Socs3	12.382	0.587	7.274	0.017	0.156	0.005	8.563	12.193	11.426	12702	Socs3	chr11	-	NM_0077	P20147	protein_cc	Cis3| Cish	suppresso	Socs3
4039	Pgs1	6.422	0.607	3.900	0.001	0.026	0.087	5.492	8.175	7.456	74451	Pgs1	chr11	+	NM_1337	P15423	protein_cc	2610019F	phosphati	Pgs1
4040	Dnah17	1.000	1.000	1.000	1.000	1.000	1.000	0.000	0.000	0.000	69926	Dnah17	chr11	-	NM_0011	P21506	protein_cc	2810003K	dynein, ax	Dnah17
4041	Cyth1	0.744	1.165	0.866	0.269	0.252	0.737	7.147	6.720	6.941	19157	Cyth1	chr11	-	NM_0111	P98	protein_cc	CLM1| CT	cytohesin	Cyth1
4042	Usp36	1.196	0.877	1.049	0.076	0.074	0.703	7.445	7.704	7.514	72344	Usp36	chr11	-	NM_0010	P26452	protein_cc	2700002L	ubiquitin s	Usp36
4043	Timp2	0.151	1.948	0.294	0.000	0.003	0.002	8.287	5.558	6.520	21858	Timp2	chr11	-	NM_0115	P24918	protein_cc	D11Bwg1	tissue inhi	Timp2
4044	BC100451	1.000	1.000	1.000	1.000	1.000	1.000	0.000	0.000	0.000	58251	BC100451	chr11	-	NM_0214	P11704	protein_cc	Cep295nl|	cDNA seq	BC100451
4045	Lgals3bp	3.367	0.775	2.611	0.000	0.157	0.142	9.208	10.959	10.592	19039	Lgals3bp	chr11	-	NM_0111	P15932	protein_cc	90K| CyCA	lectin, gala	Lgals3bp
4046	Cant1	1.272	0.769	0.978	0.017	0.021	0.776	8.446	8.793	8.414	76025	Cant1	chr11	-	NM_0012	P7703	protein_cc	5830420C	calcium ac	Cant1
4047	C1qtnf1	0.568	0.716	0.407	0.000	0.016	0.007	7.639	6.823	6.341	56745	C1qtnf1	chr11	+	NM_0012	P543	protein_cc	1600017K	C1q and tu	C1qtnf1
4048	Gm11747	0.689	0.926	0.638	0.376	0.871	0.197	4.103	3.565	3.454	10012622	Gm11747	chr11	-	NR_04590	.	lncRNA	OTTMUSG	predicted	Gm11747
4049	Engase	0.951	1.388	1.320	0.398	0.003	0.021	5.091	5.018	5.492	217364	Engase	chr11	+	NM_1725	P5677	protein_cc	C130099A	endo-beta	Engase
4050	Rbfox3	0.188	4.060	0.761	0.016	0.000	0.546	4.672	2.258	4.279	52897	Rbfox3	chr11	-	NM_0012	P10442	protein_cc	Fox-3| Hrr	RNA bindi	Rbfox3
4051	Enpp7	1.000	1.000	1.000	1.000	1.000	1.000	0.000	0.000	0.000	238011	Enpp7	chr11	+	NM_0010	P4121	protein_cc	Alk-SMase	ectonucle	Enpp7
4052	Cbx2	0.121	1.006	0.122	0.001	0.985	0.036	6.396	3.350	3.358	12416	Cbx2	chr11	+	NM_0076	P9770	protein_cc	M33| MO	chromobo	Cbx2
4053	Cbx8	0.375	1.243	0.466	0.013	0.514	0.055	6.178	4.764	5.078	30951	Cbx8	chr11	-	NM_0139	P16840	protein_cc	Pc3	chromobo	Cbx8
4054	Cbx4	0.340	1.014	0.345	0.030	0.973	0.137	7.760	6.206	6.226	12418	Cbx4	chr11	-	NM_0076	P24780	protein_cc	MPc2| PC	chromobo	Cbx4
4055	Tbc1d16	0.050	3.970	0.198	0.000	0.003	0.032	6.397	2.069	4.058	207592	Tbc1d16	chr11	-	NM_1724	P25622	protein_cc	B930087K	TBC1 dom	Tbc1d16
4056	Ccdc40	1.000	1.000	1.000	1.000	1.000	1.000	0.000	0.000	0.000	207607	Ccdc40	chr11	+	NM_1754	P2955	protein_cc	B930008I	coiled-coil	Ccdc40
4057	Gaa	0.315	1.292	0.407	0.000	0.323	0.033	8.614	6.947	7.316	14387	Gaa	chr11	+	NM_0011	P5650	protein_cc	E430018M	glucosidas	Gaa
4058	Eif4a3	1.386	0.917	1.271	0.051	0.417	0.150	9.409	9.879	9.754	192170	Eif4a3	chr11	-	NM_1386	P13182	protein_cc	2400003O	eukaryotic	Eif4a3
4059	Card14	1.371	0.789	1.081	0.665	0.741	0.925	1.003	1.459	1.116	170720	Card14	chr11	+	NM_1308	P900	protein_cc	Bimp2| CA	caspase re	Card14
4060	Sgsh	0.616	1.828	1.125	0.001	0.000	0.147	6.492	5.792	6.662	27029	Sgsh	chr11	-	NM_0188	P3516	protein_cc	4632406A	N-sulfoglu	Sgsh
4061	Slc26a11	0.956	0.469	0.448	0.831	0.025	0.241	4.999	4.935	3.842	268512	Slc26a11	chr11	+	NM_1787	P17063	protein_cc	F630021I0	solute car	Slc26a11
4062	Endov	1.143	1.739	1.987	0.311	0.081	0.247	5.139	5.331	6.130	338371	Endov	chr11	+	NM_0011	P3035	protein_cc	A730011L	endonucle	Endov
4063	Nptx1	1.010	1.825	1.844	0.000	0.182	0.423	0.000	0.015	0.883	18164	Nptx1	chr11	-	NM_0087	P3043	protein_cc	D11Bwg1	neuronal p	Nptx1
4064	Gm11762	1.000	1.000	1.000	1.000	1.000	1.000	0.000	0.000	0.000	10050383	Gm11762	chr11	+	NR_04509	.	lncRNA	OTTMUSG	predicted	Gm11762
4065	Rptor	0.451	1.530	0.690	0.018	0.168	0.058	6.872	5.723	6.336	74370	Rptor	chr11	+	NM_0288	P5388	protein_cc	4932417H	regulatory	Rptor
4066	Rptoros	1.000	1.000	1.000	1.000	1.000	1.000	0.000	0.000	0.000	319454	Rptoros	chr11	-	NR_04531	.	lncRNA	A430071A	regulatory	Rptoros
4067	Chmp6	1.045	1.331	1.391	0.677	0.036	0.046	6.715	6.779	7.191	208092	Chmp6	chr11	+	NM_0010	P17927	protein_cc	2400004G	charged m	Chmp6
4068	Baiap2	0.092	4.424	0.407	0.001	0.000	0.055	7.572	4.131	6.276	108100	Baiap2	chr11	+	NM_1308	P16769	protein_cc	IRSp53| R	brain-spec	Baiap2
4069	Aatk	0.144	1.476	0.213	0.003	0.455	0.088	5.050	2.256	2.817	11302	Aatk	chr11	-	NM_0011	P10651	protein_cc	AATYK| aa	apoptosis-	Aatk
4070	Cep131	0.430	1.062	0.457	0.092	0.850	0.367	4.377	3.160	3.247	12009	Cep131	chr11	-	NM_0097	P15819	protein_cc	AZ1| Azi|	centrosom	Cep131
4071	Enthd2	1.067	0.834	0.890	0.370	0.026	0.257	5.665	5.759	5.498	78777	Enthd2	chr11	-	NM_1831	P23228	protein_cc	2410002I0	ENTH dom	Enthd2
4072	1810043H	1.145	1.168	1.337	0.383	0.329	0.228	9.565	9.759	9.983	208501	1810043H	chr11	+	NM_0011	P742	protein_cc	.	RIKEN cDN	1810043H
4073	Slc38a10	1.404	0.923	1.296	0.184	0.646	0.289	9.917	10.407	10.291	72055	Slc38a10	chr11	-	NM_0011	P2703	protein_cc	1810073N	solute car	Slc38a10
4074	2810410L	0.730	0.590	0.431	0.008	0.006	0.020	3.389	2.935	2.174	10004233	2810410L	chr11	-	NR_03068	.	lncRNA	.	RIKEN cDN	2810410L2
4075	Bahcc1	0.171	3.460	0.592	0.003	0.024	0.070	4.780	2.234	4.025	268515	Bahcc1	chr11	+	NM_1984	P385	protein_cc	B930044J	BAH doma	Bahcc1
4076	Actg1	6.029	0.466	2.813	0.000	0.002	0.131	9.834	12.426	11.325	11465	Actg1	chr11	-	NM_0096	P1494	protein_cc	Actg| Actl	actin, gam	Actg1
4077	0610009L	0.624	1.558	0.972	0.452	0.419	0.970	3.726	3.045	3.684	66838	0610009L	chr11	+	NR_03812	.	lncRNA	.	RIKEN cDN	0610009L1
4078	Fscn2	1.000	1.000	1.000	1.000	1.000	1.000	0.000	0.000	0.000	238021	Fscn2	chr11	+	NM_1728	P3959	protein_cc	A930022G	fascin acti	Fscn2
4079	Faap100	0.325	2.200	0.714	0.003	0.056	0.339	6.472	4.849	5.987	71885	Faap100	chr11	-	NM_0279	P16462	protein_cc	2310003H	Fanconi ar	Faap100
4080	Nploc4	1.235	1.210	1.494	0.044	0.028	0.007	8.362	8.666	8.941	217365	Nploc4	chr11	-	NM_0011	P2783	protein_cc	AK129375	NPL4 hom	Nploc4
4081	Tspan10	0.716	0.995	0.712	0.182	1.000	0.423	0.490	0.008	0.000	208634	Tspan10	chr11	+	NM_1453	P17310	protein_cc	Ocsp	tetraspani	Tspan10
4082	Pde6g	0.273	2.192	0.597	0.228	0.068	0.670	3.056	1.181	2.313	18588	Pde6g	chr11	-	NR_12608	.	protein_cc	p3AT| Pde	phosphod	Pde6g

4083	Oxld1	0.172	1.352	0.233	0.004	0.363	0.068	7.011	4.473	4.908	66431	Oxld1	chr11	-	NM_0255	P6507	protein_cc	1810049H	oxidoredu	Oxld1
4084	Ccdc137	1.857	0.856	1.590	0.001	0.021	0.082	6.552	7.445	7.221	67291	Ccdc137	chr11	+	NM_1528	P4839	protein_cc	3110023B	coiled-coil	Ccdc137
4085	Arl16	1.253	1.917	2.401	0.250	0.013	0.049	6.959	7.284	8.223	70317	Arl16	chr11	-	NM_1979	P13660	protein_cc	2600005N	ADP-ribos	Arl16
4086	Hgs	1.395	0.732	1.021	0.001	0.001	0.853	7.750	8.230	7.780	15239	Hgs	chr11	+	NM_0082	P10357	protein_cc	Hgr|Hrs|	HGF-regul	Hgs
4087	Mrpl12	0.249	1.968	0.491	0.000	0.015	0.068	10.888	8.885	9.861	56282	Mrpl12	chr11	+	NM_0272	P11287	protein_cc	0610034O	mitochon	Mrpl12
4088	Slc25a10	0.092	2.032	0.186	0.000	0.023	0.003	9.939	6.492	7.515	27376	Slc25a10	chr11	+	NM_0137	P22838	protein_cc	Dic	solute car	Slc25a10
4089	Gcgr	0.176	1.374	0.242	0.000	0.051	0.009	9.722	7.215	7.673	14527	Gcgr	chr11	+	NM_0081	P17190	protein_cc	GR	glucagon	Gcgr
4090	Fam195b	2.124	1.018	2.164	0.003	0.897	0.094	7.306	8.393	8.420	192173	Fam195b	chr11	-	NM_0010	P5005	protein_cc	.	family wit	Fam195b
4091	Ppp1r27	1.019	2.633	2.682	0.000	0.030	0.179	0.000	0.027	1.424	68701	Ppp1r27	chr11	-	NM_0268	P1986	protein_cc	1110033I1	protein ph	Ppp1r27
4092	P4hb	1.002	1.047	1.048	0.986	0.775	0.842	12.584	12.586	12.652	18453	P4hb	chr11	-	NM_0110	P3863	protein_cc	ERp59|PD	prolyl 4-hy	P4hb
4093	Arhgdia	2.120	1.047	2.220	0.000	0.261	0.003	7.883	8.967	9.034	192662	Arhgdia	chr11	-	NM_1337	P6878	protein_cc	5330430M	Rho GDP d	Arhgdia
4094	Alyref	2.714	0.943	2.558	0.003	0.722	0.090	6.971	8.412	8.327	21681	Alyref	chr11	-	NM_0115	P17098	protein_cc	ALY|REF1	Aly/REF ex	Alyref
4095	Anapc11	0.568	1.243	0.706	0.001	0.241	0.224	9.413	8.596	8.910	66156	Anapc11	chr11	+	NM_0253	P10449	protein_cc	1110011I1	anaphase	Anapc11
4096	Npb	0.206	1.767	0.364	0.182	0.197	0.519	2.342	0.063	0.885	208990	Npb	chr11	+	NM_1532	P9047	protein_cc	.	neuropept	Npb
4097	Pcyt2	0.564	1.001	0.565	0.006	0.992	0.039	9.817	8.990	8.992	68671	Pcyt2	chr11	-	NM_0242	P569	protein_cc	1110033E	phosphate	Pcyt2
4098	Sirt7	0.479	1.067	0.512	0.002	0.648	0.021	8.311	7.250	7.344	209011	Sirt7	chr11	-	NM_1530	P7944	protein_cc	.	sirtuin 7	Sirt7
4099	Mafg	0.391	1.219	0.476	0.000	0.018	0.002	10.173	8.817	9.102	17134	Mafg	chr11	-	NM_0107	P21795	protein_cc	AA545192	v-maf mus	Mafg
4100	Pycr1	1.620	1.887	3.057	0.293	0.189	0.236	0.001	0.696	1.613	209027	Pycr1	chr11	-	NM_1447	P17083	protein_cc	.	pyrroline-	Pycr1
4101	Myadml2	1.000	1.000	1.000	1.000	1.000	1.000	0.000	0.000	0.000	68515	Myadml2	chr11	-	NM_0012	P1393	protein_cc	1110012N	myeloid-a	Myadml2
4102	Notum	0.017	1.051	0.018	0.000	0.727	0.003	8.704	2.852	2.923	77583	Notum	chr11	-	NM_1752	P24374	protein_cc	5730593N	notum pe	Notum
4103	Aspscr1	0.259	2.443	0.632	0.006	0.019	0.315	5.802	3.851	5.140	68938	Aspscr1	chr11	+	NM_0268	P26229	protein_cc	1190006K	alveolar s	Aspscr1
4104	Stra13	0.421	1.314	0.553	0.007	0.017	0.143	8.205	6.958	7.352	20892	Stra13	chr11	-	NM_0166	P2695	protein_cc	.	stimulated	Stra13
4105	Lrrc45	0.316	0.873	0.276	0.009	0.605	0.106	5.512	3.848	3.653	217366	Lrrc45	chr11	+	NM_1535	P8989	protein_cc	BC023296	leucine ric	Lrrc45
4106	Rac3	0.743	2.201	1.635	0.196	0.007	0.246	0.456	0.027	1.165	170758	Rac3	chr11	+	NM_1332	P21582	protein_cc	Rac1B	RAS-relate	Rac3
4107	Dcxr	0.344	1.292	0.444	0.000	0.309	0.054	9.957	8.417	8.787	67880	Dcxr	chr11	-	NM_0264	P23554	protein_cc	0610038K	dicarbony	Dcxr
4108	Cbr2	0.399	1.817	0.724	0.029	0.069	0.352	2.044	0.718	1.579	12409	Cbr2	chr11	-	NM_0076	P15085	protein_cc	MLCR	carbonyl r	Cbr2
4109	Rfng	0.307	1.582	0.486	0.000	0.003	0.002	6.431	4.729	5.391	19719	Rfng	chr11	-	NM_0090	P635	protein_cc	.	RFNG O-fu	Rfng
4110	Gps1	0.741	1.225	0.907	0.026	0.149	0.426	7.865	7.432	7.725	209318	Gps1	chr11	+	NM_0011	P9915	protein_cc	Cops1|Cs	G protein	Gps1
4111	Dus1l	0.468	1.553	0.727	0.000	0.000	0.069	8.172	7.078	7.713	68730	Dus1l	chr11	-	NM_0268	P17567	protein_cc	1110032N	dihydrour	Dus1l
4112	Fasn	0.582	1.297	0.755	0.000	0.517	0.601	11.120	10.340	10.715	14104	Fasn	chr11	-	NM_0079	P17568	protein_cc	A630082H	fatty acid	Fasn
4113	Ccdc57	0.777	0.445	0.346	0.568	0.055	0.394	3.606	3.242	2.075	71276	Ccdc57	chr11	-	NM_0277	P21028	protein_cc	4933434G	coiled-coil	Ccdc57
4114	Slc16a3	82.022	0.499	40.919	0.000	0.068	0.269	1.178	7.536	6.533	80879	Slc16a3	chr11	+	NM_0306	P16021	protein_cc	Mct3|Mc	solute car	Slc16a3
4115	Csnk1d	1.554	0.797	1.239	0.001	0.174	0.519	9.165	9.801	9.475	104318	Csnk1d	chr11	-	NM_0278	P18061	protein_cc	1200006A	casein kin	Csnk1d
4116	Cd7	0.257	1.314	0.338	0.009	0.213	0.132	2.009	0.048	0.442	12516	Cd7	chr11	-	NM_0098	P15548	protein_cc	.	CD7 antig	Cd7
4117	Sectm1b	1.575	0.466	0.734	0.475	1.000	0.422	0.446	1.101	0.000	58210	Sectm1b	chr11	-	NM_0269	P14569	protein_cc	1810003C	secreted	Sectm1b
4118	Sectm1a	2.027	0.225	0.456	0.031	0.000	0.387	3.132	4.151	2.000	209588	Sectm1a	chr11	-	NM_1453	P2381	protein_cc	BC010462	secreted a	Sectm1a
4119	Tex19.2	1.000	1.000	1.000	1.000	1.000	1.000	0.000	0.000	0.000	70956	Tex19.2	chr11	-	NM_0276	P14498	protein_cc	4921530G	testis exp	Tex19.2
4120	Tex19.1	2.700	0.370	1.000	0.305	1.000	0.423	0.001	1.433	0.000	73679	Tex19.1	chr11	+	NM_0286	P5571	protein_cc	2410081M	testis exp	Tex19.1
4121	Uts2r	1.000	1.000	1.000	1.000	1.000	1.000	0.000	0.000	0.000	217369	Uts2r	chr11	+	NM_1454	P139	protein_cc	Gpr14|UF	urotensin	Uts2r
4122	Ogfod3	0.555	1.001	0.556	0.002	0.994	0.055	6.731	5.883	5.883	66179	Ogfod3	chr11	-	NM_0254	P2049	protein_cc	1110031C	2-oxoglut	Ogfod3
4123	Hexdc	2.096	0.684	1.433	0.001	0.010	0.116	5.962	7.029	6.481	238023	Hexdc	chr11	+	NM_0010	P23140	protein_cc	BC069960	hexosami	Hexdc
4124	BC017643	2.198	0.637	1.401	0.002	0.006	0.250	7.031	8.167	7.517	217370	BC017643	chr11	-	NM_0012	P24549	protein_cc	.	cDNA seq	BC017643
4125	Narf	0.804	1.219	0.979	0.225	0.241	0.935	7.910	7.594	7.880	67608	Narf	chr11	+	NM_0262	P3432	protein_cc	4430402O	nuclear pr	Narf
4126	Foxk2	1.597	0.975	1.557	0.017	0.730	0.089	6.265	6.940	6.904	68837	Foxk2	chr11	+	NM_0010	P25390	protein_cc	1110054H	forkhead	Foxk2
4127	Wdr45b	1.828	1.259	2.300	0.002	0.250	0.156	8.729	9.599	9.931	68215	Wdr45b	chr11	-	NM_0257	P7759	protein_cc	0610008N	WD repea	Wdr45b
4128	Rab40b	0.138	6.051	0.834	0.000	0.136	0.813	2.970	0.110	2.708	217371	Rab40b	chr11	-	NM_1391	P20284	protein_cc	rar|SEC4L	Rab40B, n	Rab40b
4129	Fn3krp	1.146	0.632	0.725	0.729	1.000	0.422	0.465	0.661	0.000	238024	Fn3krp	chr11	+	NM_1814	P13790	protein_cc	FN3K-RP	fructosam	Fn3krp
4130	Fn3k	0.015	6.422	0.096	0.001	0.046	0.050	6.876	0.816	3.499	63828	Fn3k	chr11	+	NM_0010	P15088	protein_cc	2310074G	fructosam	Fn3k
4131	Tbcd	0.919	2.223	2.043	0.423	0.008	0.096	6.593	6.471	7.624	108903	Tbcd	chr11	+	NM_0298	P16067	protein_cc	2310057L	tubulin-sp	Tbcd
4132	Zfp750	1.856	1.644	3.051	0.011	0.002	0.006	5.430	6.322	7.039	319530	Zfp750	chr11	-	NM_1787	P21976	protein_cc	A030007D	zinc finger	Zfp750
4133	B3gntl1	0.954	1.948	1.859	0.717	0.007	0.059	6.808	6.740	7.702	210004	B3gntl1	chr11	-	NM_1786	P6446	protein_cc	6030413G	UDP-GlcN	B3gntl1
4134	Metrnl	4.418	0.596	2.633	0.019	0.127	0.089	4.659	6.802	6.056	210029	Metrnl	chr11	+	NM_1447	P20437	protein_cc	9430048M	meteorin,	Metrnl
4135	Ptchd3	1.000	1.000	1.000	1.000	1.000	1.000	0.000	0.000	0.000	74675	Ptchd3	chr11	+	NM_0290	P3159	protein_cc	4930451E	patched d	Ptchd3
4136	Rab10os	1.348	0.728	0.981	0.273	0.246	0.955	5.709	6.140	5.682	74173	Rab10os	chr12	+	NR_01555	.	lncRNA	1700012B	RAB10, m	Rab10os
4137	Rab10	0.839	0.797	0.668	0.001	0.011	0.026	11.168	10.915	10.587	19325	Rab10	chr12	+	NM_0166	P17055	protein_cc	AW10775	RAB10, m	Rab10
4138	Kif3c	1.442	0.671	0.967	0.324	0.403	0.965	4.684	5.212	4.635	16570	Kif3c	chr12	+	NM_0084	P9257	protein_cc	mKIAA405	kinesin fa	Kif3c
4139	1110002L	1.423	0.705	1.003	0.319	0.434	0.997	4.656	5.165	4.660	10004304	1110002L	chr12	+	NR_03069	.	lncRNA	.	RIKEN cDN	1110002L
4140	Asxl2	0.695	0.925	0.643	0.000	0.676	0.196	8.154	7.629	7.516	75302	Asxl2	chr12	+	NM_1724	P24331	protein_cc	4930556B	additional	Asxl2
4141	Dtnb	0.403	1.731	0.697	0.000	0.344	0.572	5.664	4.352	5.143	13528	Dtnb	chr12	+	NM_0011	P22876	protein_cc	.	dystrobre	Dtnb
4142	Dnmt3a	0.607	1.094	0.664	0.002	0.491	0.053	7.633	6.912	7.042	13435	Dnmt3a	chr12	+	NM_0078	P22795	protein_cc	MmuIIIA	DNA meth	Dnmt3a
4143	Dnmt3aos	0.552	0.990	0.547	0.182	1.000	0.423	0.871	0.015	0.000	10003852	Dnmt3aos	chr12	-	NR_04588	.	lncRNA	ENSMUSG	DNA meth	Dnmt3aos
4144	Pomc	5.540	0.255	1.411	0.009	0.011	0.317	1.525	3.995	2.021	18976	Pomc	chr12	+	NM_0088	P661	protein_cc	ACTH|alp	pro-opiom	Pomc
4145	Efr3b	1.115	2.502	2.790	0.683	0.146	0.367	1.873	2.030	3.353	668212	Efr3b	chr12	-	NM_0010	P16489	protein_cc	AI852640|	EFR3 hom	Efr3b
4146	Dnajc27	3.891	0.888	3.457	0.075	0.763	0.189	2.239	4.199	4.028	217378	Dnajc27	chr12	+	NM_1530	P22721	protein_cc	AI639580|	DnaJ heat	Dnajc27
4147	Adcy3	0.716	1.346	0.964	0.294	0.419	0.898	3.419	2.938	3.366	104111	Adcy3	chr12	+	NM_0011	P20990	protein_cc	AC3|mKI/	adenylate	Adcy3
4148	Cenpo	0.686	1.203	0.825	0.221	0.546	0.358	4.753	4.209	4.475	52504	Cenpo	chr12	-	NM_1340	P26297	protein_cc	2810429O	centrome	Cenpo
4149	Ptrhd1	0.880	1.516	1.334	0.294	0.004	0.131	7.668	7.483	8.083	69709	Ptrhd1	chr12	-	NM_0012	P13285	protein_cc	2410017P	peptidyl-t	Ptrhd1
4150	Ncoa1	0.893	0.963	0.861	0.201	0.622	0.314	7.740	7.578	7.524	17977	Ncoa1	chr12	-	NM_0108	P21088	protein_cc	bHLHe74	nuclear re	Ncoa1
4151	Itsn2	1.557	0.891	1.387	0.013	0.357	0.285	8.525	9.163	8.997	20403	Itsn2	chr12	+	NM_0113	P18769	protein_cc	AI327390|	intersecti	Itsn2
4152	Fam228a	2.877	0.469	1.348	0.078	0.084	0.669	4.081	5.606	4.512	74855	Fam228a	chr12	-	NM_0291	P8894	protein_cc	4930417G	family wit	Fam228a
4153	Fam228b	0.461	0.518	0.239	0.001	0.019	0.027	4.059	2.943	1.995	207921	Fam228b	chr12	-	NM_1754	P23795	protein_cc	A830093I2	family wit	Fam228b
4154	Pfn4	0.149	2.560	0.380	0.000	0.190	0.124	2.835	0.085	1.441	382562	Pfn4	chr12	+	NM_0283	P355	protein_cc	2900024P	profilin fa	Pfn4
4155	Gm6682	1.716	1.038	1.782	0.046	0.608	0.194	2.860	3.640	3.694	626534	Gm6682	chr12	+	NR_03359	.	pseudogene	EG626534	predicted	Gm6682
4156	Sf3b6	2.385	0.972	2.317	0.004	0.875	0.138	9.605	10.859	10.817	66055	Sf3b6	chr12	+	NM_0253	P18816	protein_cc	0610009D	splicing fa	Sf3b6
4157	Fkbp1b	1.169	3.176	3.712	0.772	0.009	0.028	3.022	3.247	4.914	14226	Fkbp1b	chr12	-	NM_0168	P1848	protein_cc	12.6kDa|.	FK506 bin	Fkbp1b
4158	BC068281	1.644	0.253	0.416	0.110	0.017	0.014	4.888	5.605	3.622	238037	BC068281	chr12	+	NM_1734	P2209	protein_cc	A430092C	cDNA seq	BC068281
4159	Mfsd2b	1.547	0.258	0.399	0.627	1.000	0.423	1.326	1.955	0.000	432628	Mfsd2b	chr12	-	NM_0010	P9499	protein_cc	Gm1964	major faci	Mfsd2b
4160	Ubxn2a	0.833	1.291	1.076	0.044	0.039	0.680	7.016	6.753	7.122	217379	Ubxn2a	chr12	-	NM_1454	P24172	protein_cc	6330407P	UBX doma	Ubxn2a
4161	Atad2b	1.100	0.818	0.900	0.021	0.065	0.501	8.574	8.711	8.422	320817	Atad2b	chr12	+	NM_0010	P1941	protein_cc	1110014E	ATPase fa	Atad2b
4162	Klhl29	0.977	2.129	2.080	0.785	0.330	0.576	1.831	1.798	2.888	208439	Klhl29	chr12	-	NM_0011	P26361	protein_cc	A230106N	kelch-like	Klhl29
4163	2810032G	3.300	0.303	1.000	0.306	1.000	0.423	0.001	1.723	0.000	72669	2810032G	chr12	+	NR_01557	.	lncRNA	AI461720	RIKEN cDN	2810032G
4164	1700101O	1.000	1.000	1.000	1.000	1.000	1.000	0.000	0.000	0.000	73575	1700101O	chr12	-	NR_04504	.	lncRNA		RIKEN cDN	1700101O
4165	Apob	0.756	1.294	0.978	0.027	0.208	0.937	11.401	10.997	11.369	238055	Apob	chr12	+	NM_0096	P16235	protein_cc	AI315052|	apolipopro	Apob
4166	Ldah	0.896	0.923	0.827	0.050	0.162	0.053	7.501	7.342	7.226	68832	Ldah	chr12	+	NM_1724	P18287	protein_cc	1110057K	lipid dropl	Ldah
4167	Gdf7	1.000	1.000	1.000	1.000	1.000	1.000	0.000	0.000	0.000	238057	Gdf7	chr12	-	NM_0135	P18609	protein_cc	BMP12	growth dif	Gdf7
4168	Hs1bp3	0.971	0.467	0.453	0.706	0.000	0.039	6.386	6.344	5.244	58240	Hs1bp3	chr12	+	NM_0214	P22014	protein_cc	.	HCLS1 bi	Hs1bp3
4169	Rhob	3.336	0.468	1.562	0.015	0.035	0.105	8.004	9.742	8.647	11852	Rhob	chr12	+	NM_0074	P17445	protein_cc	AA017882	ras homol	Rhob
4170	Slc7a15	4.428	0.301	1.334	0.306	0.350	0.423	0.001	2.148	0.417	328059	Slc7a15	chr12	-	NM_0010	P7704	protein_cc	Arpat	solute car	Slc7a15
4171	Pum2	1.248	0.727	0.908	0.142	0.063	0.219	8.853	9.173	8.714	80913	Pum2	chr12	+	NM_0011	P4512	protein_cc	5730503J2	pumilio R	Pum2
4172	Sdc1	0.927	0.600	0.557	0.019	0.010	0.077	10.691	10.582	9.846	20969	Sdc1	chr12	+	NM_0115	P18177	protein_cc	AA408134	syndecan	Sdc1
4173	Laptm4a	1.510	0.879	1.328	0.002	0.119	0.108	12.381	12.976	12.790	17775	Laptm4a	chr12	+	NM_0086	P25541	protein_cc	AA286466	lysosomal	Laptm4a

4174	Matn3	1.572	0.636	1.000	0.304	1.000	0.423	0.000	0.653	0.000	17182	Matn3	chr12	+	NM_0107	P8948	protein_cc	AV009181	matrilin 3	Matn3
4175	Wdr35	0.950	1.373	1.303	0.811	0.096	0.520	5.295	5.221	5.678	74682	Wdr35	chr12	+	NM_1724	P4482	protein_cc	4930459N	WD repea	Wdr35
4176	Ttc32	1.125	0.625	0.704	0.018	0.008	0.176	8.058	8.228	7.551	75516	Ttc32	chr12	+	NM_0293	P2685	protein_cc	1700013G	tetratricop	Ttc32
4177	1700022H	1.000	1.000	1.000	1.000	1.000	1.000	0.000	0.000	0.000	69420	1700022H	chr12	-	NR_04548	.	lncRNA		RIKEN cDN	1700022H
4178	Osr1	0.297	9.811	2.918	0.188	0.001	0.158	1.860	0.110	3.404	23967	Osr1	chr12	+	NM_0118	P14098	protein_cc	Odd1| Osı	odd-skipp	Osr1
4179	Nt5c1b	1.000	1.000	1.000	1.000	1.000	1.000	0.000	0.000	0.000	70881	Nt5c1b	chr12	+	NM_0275	P2640	protein_cc	4921514H	5'-nucleot	Nt5c1b
4180	Rdh14	1.200	1.081	1.297	0.001	0.171	0.078	8.147	8.410	8.522	105014	Rdh14	chr12	+	NM_0236	P23479	protein_cc	3110030G	retinol del	Rdh14
4181	Gm38407	0.716	0.995	0.712	0.182	1.000	0.423	0.490	0.008	0.000	328061	Gm38407	chr12	+	NR_11048	.	lncRNA	4931412N	predicted	Gm38407
4182	7420701I0	1.000	1.000	1.000	1.000	1.000	1.000	0.000	0.000	0.000	71457	7420701I0	chr12	+	NR_04586	.	lncRNA		RIKEN cDN	7420701I0
4183	Kcns3	0.353	3.744	1.320	0.186	0.001	0.688	1.568	0.064	1.968	238076	Kcns3	chr12	-	NM_0011	P1401	protein_cc	.	potassium	Kcns3
4184	9530020I1	0.716	0.995	0.712	0.182	1.000	0.423	0.490	0.008	0.000	77442	9530020I1	chr12	-	NR_13108	.	lncRNA	.	RIKEN cDN	9530020I1
4185	Msgn1	1.000	1.000	1.000	1.000	1.000	1.000	0.000	0.000	0.000	56184	Msgn1	chr12	-	NM_0195	P5792	protein_cc	Msgn| pM	mesogeni	Msgn1
4186	Gen1	1.068	0.193	0.206	0.898	1.000	0.422	2.279	2.374	0.000	209334	Gen1	chr12	-	NM_1773	P1488	protein_cc	5830483C	GEN1, Hol	Gen1
4187	Smc6	1.083	0.819	0.888	0.025	0.017	0.327	9.248	9.364	9.076	67241	Smc6	chr12	+	NM_0256	P3707	protein_cc	2810489L	structural	Smc6
4188	Vsnl1	1.015	2.253	2.285	0.000	0.015	0.125	0.000	0.021	1.193	26950	Vsnl1	chr12	-	NM_0120	P4997	protein_cc	VILIP| Vns	visinin-like	Vsnl1
4189	4930511A	1.000	1.000	1.000	1.000	1.000	1.000	0.000	0.000	0.000	74712	4930511A	chr12	+	NR_04596	.	lncRNA		RIKEN cDN	4930511A
4190	Rad51ap2	1.000	1.000	1.000	1.000	1.000	1.000	0.000	0.000	0.000	209550	Rad51ap2	chr12	+	NM_0011	P12434	protein_cc	EG209550	RAD51 ass	Rad51ap2
4191	Tubb2a-ps	1.000	1.000	1.000	1.000	1.000	1.000	0.000	0.000	0.000	627110	Tubb2a-ps	chr12	-	NR_00396	.	misc_RNA	C79445	tubulin, be	Tubb2a-ps
4192	Fam49a	1.888	0.738	1.394	0.002	0.025	0.117	4.935	5.851	5.413	76820	Fam49a	chr12	+	NM_0011	P25623	protein_cc	2410157N	family wit	Fam49a
4193	4921511I1	3.334	0.645	2.151	0.305	0.585	0.081	0.001	1.738	1.106	70858	4921511I1	chr12	-	NR_04582	.	lncRNA	.	RIKEN cDN	4921511I1
4194	Mycn	0.412	1.810	0.746	0.286	0.370	0.804	2.004	0.726	1.581	18109	Mycn	chr12	-	NM_0087	P23470	protein_cc	bHLHe37|	v-myc avia	Mycn
4195	Ddx1	1.579	0.855	1.350	0.006	0.102	0.009	8.725	9.384	9.158	104721	Ddx1	chr12	-	NM_1340	P3317	protein_cc	AA409185	DEAD (Asp	Ddx1
4196	Nbas	2.174	0.487	1.059	0.012	0.006	0.857	6.122	7.242	6.205	71169	Nbas	chr12	+	NM_0277	P16048	protein_cc	4933425L	neuroblas	Nbas
4197	Fam84a	0.790	0.661	0.523	0.568	0.487	0.110	3.901	3.561	2.964	105005	Fam84a	chr12	-	NM_0290	P17952	protein_cc	2310003N	family wit	Fam84a
4198	Gm16497	1.000	1.000	1.000	1.000	1.000	1.000	0.000	0.000	0.000	791361	Gm16497	chr12	+	NR_04500	.	lncRNA	ENSMUSG	predicted	Gm16497
4199	4930448C	1.000	1.000	1.000	1.000	1.000	1.000	0.000	0.000	0.000	73972	4930448C	chr12	-	NR_04596	.	lncRNA		RIKEN cDN	4930448C
4200	Trib2	5.725	1.103	6.315	0.000	0.649	0.105	5.046	7.563	7.705	217410	Trib2	chr12	-	NM_1445	P24133	protein_cc	AW31951	tribbles ps	Trib2
4201	Lpin1	0.213	2.076	0.443	0.000	0.005	0.018	10.714	8.485	9.538	14245	Lpin1	chr12	-	NM_0011	P9947	protein_cc	4631420P	lipin 1	Lpin1
4202	Ntsr2	1.000	1.000	1.000	1.000	1.000	1.000	0.000	0.000	0.000	18217	Ntsr2	chr12	+	NM_0087	P12297	protein_cc	NT2R| NTI	neurotens	Ntsr2
4203	Greb1	1.005	1.405	1.412	0.000	0.182	0.423	0.000	0.008	0.498	268527	Greb1	chr12	-	NM_0012	P15873	protein_cc	5730583K	gene regu	Greb1
4204	E2f6	0.797	0.939	0.748	0.029	0.780	0.416	6.901	6.574	6.483	50496	E2f6	chr12	+	NR_02767	.	protein_cc	AI462434|	E2F transc	E2f6
4205	Rock2	1.431	0.659	0.943	0.029	0.025	0.582	8.396	8.913	8.311	19878	Rock2	chr12	+	NM_0090	P1578	protein_cc	B230113H	Rho-assoc	Rock2
4206	Pqlc3	2.700	1.568	4.234	0.008	0.348	0.311	1.714	3.146	3.795	217430	Pqlc3	chr12	-	NM_1725	P14474	protein_cc	C78076| E	PQ loop re	Pqlc3
4207	2410004P	1.000	1.000	1.000	1.000	1.000	1.000	0.000	0.000	0.000	73667	2410004P	chr12	-	NM_0012	P5845	protein_cc	.	RIKEN cDN	2410004P
4208	Kcnf1	1.000	1.000	1.000	1.000	1.000	1.000	0.000	0.000	0.000	382571	Kcnf1	chr12	-	NM_2015	P11504	protein_cc	Gm1182|	potassium	Kcnf1
4209	Pdia6	2.028	0.739	1.499	0.000	0.036	0.184	11.731	12.751	12.315	71853	Pdia6	chr12	+	NM_0279	P12086	protein_cc	1700015E	protein dis	Pdia6
4210	Atp6v1c2	1.689	0.689	1.164	0.003	0.033	0.502	3.626	4.382	3.845	68775	Atp6v1c2	chr12	-	NM_1336	P5517	protein_cc	1110038G	ATPase, H	Atp6v1c2
4211	Nol10	2.604	0.783	2.037	0.000	0.001	0.005	6.269	7.649	7.296	217431	Nol10	chr12	+	NM_0010	P11826	protein_cc	Gm67	nucleolar	Nol10
4212	Odc1	4.300	0.639	2.747	0.000	0.003	0.047	8.160	10.264	9.618	18263	Odc1	chr12	+	NM_0136	P3921	protein_cc	ODC	ornithine	Odc1
4213	Hpcal1	0.749	1.544	1.155	0.072	0.014	0.346	7.397	6.980	7.606	53602	Hpcal1	chr12	+	NM_0166	P21075	protein_cc	NVP-3| N	hippocalci	Hpcal1
4214	5730507C	1.173	0.784	0.920	0.661	0.527	0.799	3.781	4.011	3.661	236366	5730507C	chr12	+	NM_0010	P11332	protein_cc	.	RIKEN cDN	5730507C
4215	1700030C	1.204	2.313	2.785	0.013	0.142	0.333	5.909	6.177	7.386	69513	1700030C	chr12	-	NR_01552	.	lncRNA	.	RIKEN cDN	1700030C
4216	Asap2	0.991	0.630	0.624	0.929	0.019	0.079	6.378	6.364	5.697	211914	Asap2	chr12	+	NM_0010	P12145	protein_cc	6530401G	ArfGAP wi	Asap2
4217	Itgb1bp1	1.193	0.881	1.050	0.204	0.361	0.857	7.739	7.994	7.810	16413	Itgb1bp1	chr12	-	NM_0084	P17418	protein_cc	AI449260|	integrin be	Itgb1bp1
4218	Cpsf3	0.737	1.117	0.823	0.028	0.297	0.071	8.739	8.298	8.458	54451	Cpsf3	chr12	+	NM_0188	P17889	protein_cc	.	cleavage a	Cpsf3
4219	Iah1	0.453	0.977	0.442	0.000	0.603	0.001	10.605	9.461	9.428	67732	Iah1	chr12	+	NM_0263	P7213	protein_cc	1500019E	isoamyl ac	Iah1
4220	Adam17	0.761	0.755	0.575	0.005	0.002	0.021	10.308	9.913	9.508	11491	Adam17	chr12	-	NM_0096	P10333	protein_cc	CD156b| T	a disinteg	Adam17
4221	Ywhaq	0.803	1.296	1.041	0.002	0.086	0.830	9.132	8.816	9.190	22630	Ywhaq	chr12	-	NM_0117	P23727	protein_cc	2700028P	tyrosine 3	Ywhaq
4222	2410018L	1.000	1.000	1.000	1.000	1.000	1.000	0.000	0.000	0.000	69732	2410018L	chr12	-	NR_01550	.	lncRNA	2810480C	RIKEN cDN	2410018L
4223	9030624G	0.716	0.995	0.712	0.182	1.000	0.423	0.490	0.008	0.000	66808	9030624G	chr12	-	NM_0012	P23465	protein_cc	.	RIKEN cDN	9030624G
4224	Taf1b	1.078	1.770	1.908	0.740	0.036	0.126	5.792	5.901	6.724	21340	Taf1b	chr12	+	NM_0206	P13371	protein_cc	4930408G	TATA-box	Taf1b
4225	Grhl1	5.570	0.394	2.195	0.000	0.018	0.453	3.177	5.654	4.311	195733	Grhl1	chr12	+	NM_0011	P16102	protein_cc	LBP-32| M	grainyhea	Grhl1
4226	Klf11	0.976	2.266	2.210	0.202	0.225	0.480	6.371	6.335	7.516	194655	Klf11	chr12	+	NM_1783	P18119	protein_cc	9830142A	Kruppel-lik	Klf11
4227	Cys1	0.111	1.411	0.156	0.000	0.514	0.005	4.717	1.542	2.038	12879	Cys1	chr12	-	NM_0010	P5379	protein_cc	2900006B	cystin 1	Cys1
4228	Rrm2	0.181	3.222	0.582	0.000	0.001	0.020	4.728	2.259	3.947	20135	Rrm2	chr12	+	NM_0091	P16644	protein_cc	AA407299	ribonucleo	Rrm2
4229	Mboat2	2.162	1.021	2.208	0.300	0.972	0.423	0.001	1.113	1.143	67216	Mboat2	chr12	+	NM_0260	P17144	protein_cc	2810049G	membrane	Mboat2
4230	Kidins220	0.788	1.117	0.881	0.057	0.290	0.242	8.343	8.000	8.160	77480	Kidins220	chr12	+	NM_0010	P1397	protein_cc	3110039L	kinase D-ir	Kidins220
4231	Id2	0.942	1.034	0.974	0.008	0.890	0.949	12.203	12.116	12.165	15902	Id2	chr12	-	NM_0104	P7838	protein_cc	AI255428|	inhibitor o	Id2
4232	Gm17746	1.927	0.299	0.577	0.201	1.000	0.422	0.794	1.740	0.000	432641	Gm17746	chr12	+	NR_04584	.	lncRNA		predicted	Gm17746
4233	Gm29687	1.000	1.000	1.000	1.000	1.000	1.000	0.000	0.000	0.000	629481	Gm29687	chr12	-	NR_13114	.	lncRNA	.	predicted	Gm29687
4234	4930480M	1.000	1.000	1.000	1.000	1.000	1.000	0.000	0.000	0.000	74930	4930480M	chr12	-	NR_04627	.	lncRNA	.	RIKEN cDN	4930480M
4235	Rnf144a	2.004	0.353	0.707	0.000	0.000	0.152	7.183	8.186	6.683	108089	Rnf144a	chr12	-	NM_0010	P18288	protein_cc	Rnf144| U	ring finger	Rnf144a
4236	4930549C	1.359	0.367	0.499	0.673	1.000	0.423	1.003	1.445	0.000	75243	4930549C	chr12	-	NR_13098	.	lncRNA	.	RIKEN cDN	4930549C
4237	Rsad2	6.882	0.348	2.393	0.000	0.001	0.232	8.617	11.400	9.876	58185	Rsad2	chr12	-	NM_0213	P19999	protein_cc	2510004L	radical S-a	Rsad2
4238	Cmpk2	3.909	0.229	0.896	0.029	0.027	0.775	5.935	7.902	5.777	22169	Cmpk2	chr12	+	NM_0205	P23994	protein_cc	1200004E	cytidine m	Cmpk2
4239	Gm20187	1.000	1.000	1.000	1.000	1.000	1.000	0.000	0.000	0.000	10050434	Gm20187	chr12	-	NR_04505	.	lncRNA	.	predicted	Gm20187
4240	Gm9866	1.019	2.648	2.698	0.000	0.182	0.423	0.000	0.027	1.432	636791	Gm9866	chr12	-	NR_04586	.	lncRNA	EG636791	predicted	Gm9866
4241	Sox11	1.533	0.398	0.611	0.489	0.254	0.459	1.577	2.193	0.865	20666	Sox11	chr12	-	NM_0092	P5096	protein_cc	1110038H	SRY (sex d	Sox11
4242	Dcdc2c	0.504	0.990	0.499	0.181	1.000	0.423	1.002	0.015	0.000	68511	Dcdc2c	chr12	-	NM_0011	P17569	protein_cc	1110015N	doublecor	Dcdc2c
4243	Allc	1.000	1.000	1.000	1.000	1.000	1.000	0.000	0.000	0.000	94041	Allc	chr12	-	NM_0531	P6798	protein_cc	1700012B	allantoicas	Allc
4244	Colec11	0.205	0.708	0.145	0.000	0.164	0.004	8.477	6.188	5.689	71693	Colec11	chr12	-	NM_0278	P17872	protein_cc	CL-K1	collectin s	Colec11
4245	Rps7	1.322	1.411	1.866	0.000	0.038	0.096	9.208	9.611	10.108	20115	Rps7	chr12	-	NM_0113	P13392	protein_cc	Mtu| Rps7	ribosomal	Rps7
4246	Rnaseh1	1.533	0.748	1.148	0.318	0.546	0.821	4.651	5.268	4.850	19819	Rnaseh1	chr12	+	NM_0112	P9017	protein_cc	.	ribonuclea	Rnaseh1
4247	Adi1	0.093	1.999	0.186	0.001	0.045	0.034	9.784	6.357	7.356	104923	Adi1	chr12	+	NM_1340	P20903	protein_cc	AL024210	acireducto	Adi1
4248	Trappc12	0.946	1.308	1.236	0.662	0.053	0.087	6.633	6.552	6.939	217449	Trappc12	chr12	-	NM_0011	P8327	protein_cc	CGI-87| D	trafficking	Trappc12
4249	Tssc1	0.909	1.212	1.101	0.377	0.030	0.498	7.486	7.348	7.625	380752	Tssc1	chr12	+	NM_2013	P24638	protein_cc	D12Ertd6	tumor sup	Tssc1
4250	Myt1l	2.745	0.643	1.765	0.304	0.545	0.005	0.001	1.457	0.821	17933	Myt1l	chr12	+	NM_0010	P6796	protein_cc	2900046C	myelin tra	Myt1l
4251	C630031E	1.000	1.000	1.000	1.000	1.000	1.000	0.000	0.000	0.000	328075	C630031E	chr12	-	NR_04608	.	lncRNA	C630031E	RIKEN cDN	C630031E
4252	Pxdn	0.215	1.199	0.257	0.022	0.447	0.170	5.942	3.722	3.984	69675	Pxdn	chr12	+	NM_1813	P14778	protein_cc	2310075N	peroxidasi	Pxdn
4253	Tpo	1.000	1.000	1.000	1.000	1.000	1.000	0.000	0.000	0.000	22018	Tpo	chr12	-	NM_0094	P8237	protein_cc	.	thyroid pe	Tpo
4254	Sntg2	1.030	1.374	1.415	0.898	0.537	0.680	3.794	3.836	4.295	268534	Sntg2	chr12	-	NM_1729	P18297	protein_cc	2210008K	syntrophin	Sntg2
4255	2310016D	1.000	1.000	1.000	1.000	1.000	1.000	0.000	0.000	0.000	69566	2310016D	chr12	-	NR_04549	.	lncRNA		RIKEN cDN	2310016D
4256	Tmem18	3.894	0.691	2.691	0.000	0.005	0.067	5.784	7.745	7.213	211986	Tmem18	chr12	+	NM_1720	P3943	protein_cc	BC024093	transmem	Tmem18
4257	Fam150b	0.485	3.174	1.539	0.067	0.016	0.273	3.081	2.037	3.704	10029458	Fam150b	chr12	-	NM_0011	P18169	protein_cc	6230419C	family wit	Fam150b
4258	Acp1	0.877	1.222	1.072	0.031	0.006	0.224	9.840	9.651	9.941	11431	Acp1	chr12	-	NM_0213	P7814	protein_cc	4632432E	acid phosp	Acp1
4259	Sh3yl1	1.016	0.635	0.645	0.912	0.042	0.055	6.528	6.551	5.897	24057	Sh3yl1	chr12	+	NM_0012	P20287	protein_cc	AI314953|	Sh3 doma	Sh3yl1
4260	Fam110c	0.835	0.487	0.406	0.751	0.433	0.051	4.102	3.842	2.802	104943	Fam110c	chr12	+	NM_0278	P10268	protein_cc	9030611O	family wit	Fam110c
4261	6030469F	1.000	1.000	1.000	1.000	1.000	1.000	0.000	0.000	0.000	10004306	6030469F	chr12	+	NR_10271	.	lncRNA	.	RIKEN cDN	6030469F
4262	Lamb1	0.598	1.391	0.831	0.015	0.467	0.745	6.638	5.895	6.371	16777	Lamb1	chr12	+	NM_0084	P9857	protein_cc	C77966| C	laminin B1	Lamb1
4263	Dld	1.159	0.996	1.153	0.006	0.959	0.396	9.725	9.937	9.931	13382	Dld	chr12	-	NM_0078	P947	protein_cc	.	dihydrolip	Dld
4264	Slc26a3	0.387	2.682	1.037	0.016	0.184	0.963	1.419	0.048	1.472	13487	Slc26a3	chr12	+	NM_0213	P16252	protein_cc	9030623B	solute car	Slc26a3

4265	Cbll1	1.499	0.652	0.977	0.180	0.125	0.953	7.097	7.680	7.063	104836	Cbll1	chr12	-	NM_0012	P1212	protein_cc	AI467391|	Casitas B-	Cbll1
4266	Slc26a4	0.051	0.910	0.046	0.003	1.000	0.062	4.438	0.137	0.000	23985	Slc26a4	chr12	-	NM_0118	P3050	protein_cc	Pds| pend	solute car	Slc26a4
4267	Bcap29	1.062	1.497	1.589	0.690	0.004	0.094	6.594	6.680	7.262	12033	Bcap29	chr12	-	NM_0075	P25901	protein_cc	AW20840	B cell rece	Bcap29
4268	Dus4l	0.818	1.095	0.896	0.613	0.772	0.847	4.358	4.069	4.199	71916	Dus4l	chr12	-	NM_0280	P23193	protein_cc	2310069P	dihydrouri	Dus4l
4269	Cog5	0.555	1.536	0.853	0.026	0.064	0.522	3.408	2.559	3.178	238123	Cog5	chr12	+	NM_0011	P7511	protein_cc	5430405C	componer	Cog5
4270	Gpr22	2.170	0.461	1.000	0.000	1.000	0.423	0.000	1.118	0.000	73010	Gpr22	chr12	-	NM_1751	P17283	protein_cc	2900068K	G protein-	Gpr22
4271	Hbp1	0.875	1.191	1.042	0.178	0.102	0.741	10.194	10.001	10.253	73389	Hbp1	chr12	-	NM_1531	P6536	protein_cc	1700058O	high mobi	Hbp1
4272	Prkar2b	1.995	1.285	2.564	0.239	0.464	0.031	2.832	3.829	4.191	19088	Prkar2b	chr12	-	NM_0111	P24664	protein_cc	AI451071|	protein kir	Prkar2b
4273	Pik3cg	1.501	0.523	0.785	0.488	0.348	0.481	3.476	4.062	3.127	30955	Pik3cg	chr12	-	NM_0202	P4535	protein_cc	5830428L	phosphoir	Pik3cg
4274	Ccdc71l	4.651	0.494	2.298	0.000	0.002	0.095	5.855	8.073	7.056	72123	Ccdc71l	chr12	+	NM_0011	P23441	protein_cc	2010109K	coiled-coil	Ccdc71l
4275	Nampt	10.370	1.339	13.881	0.004	0.516	0.233	9.007	12.381	12.802	59027	Nampt	chr12	+	NM_0215	P20302	protein_cc	1110035O	nicotinam	Nampt
4276	Gdap10	5.711	0.336	1.920	0.018	0.036	0.089	4.386	6.900	5.327	10050448	Gdap10	chr12	+	NR_04503	.	lncRNA	Gdap-ps	gangliosid	Gdap10
4277	4933406C	1.010	1.825	1.844	0.000	0.182	0.423	0.000	0.015	0.883	74076	4933406C	chr12	-	NR_04498	.	lncRNA	.	RIKEN cDN	4933406C
4278	Sypl	0.607	1.394	0.846	0.010	0.001	0.379	9.502	8.780	9.260	19027	Sypl	chr12	+	NM_0136	P15514	protein_cc	AI314763|	synaptoph	Sypl
4279	Cdhr3	0.716	0.995	0.712	0.182	1.000	0.423	0.490	0.008	0.000	68764	Cdhr3	chr12	-	NM_0010	P21478	protein_cc	1110049B	cadherin-r	Cdhr3
4280	F730043M	3.301	0.694	2.292	0.304	0.657	0.423	0.001	1.724	1.198	320046	F730043M	chr12	-	NR_01560	.	lncRNA	.	RIKEN cDN	F730043M
4281	Atxn7l1	1.750	0.508	0.889	0.036	0.022	0.581	5.411	6.218	5.241	380753	Atxn7l1	chr12	+	NM_0281	P12993	protein_cc	2810423G	ataxin 7-li	Atxn7l1
4282	Atxn7l1os	1.000	1.000	1.000	1.000	1.000	1.000	0.000	0.000	0.000	75836	Atxn7l1os	chr12	-	NR_13103	.	lncRNA	4930556N	ataxin 7-li	Atxn7l1os
4283	Efcab10	1.000	1.000	1.000	1.000	1.000	1.000	0.000	0.000	0.000	75040	Efcab10	chr12	+	NM_0291	P12572	protein_cc	4930504H	EF-hand ca	Efcab10
4284	Twistnb	1.454	0.845	1.228	0.036	0.257	0.406	8.776	9.315	9.072	28071	Twistnb	chr12	+	NM_1722	P26691	protein_cc	2410173G	twist basi	Twistnb
4285	Ferd3l	1.000	1.000	1.000	1.000	1.000	1.000	0.000	0.000	0.000	114712	Ferd3l	chr12	+	NM_0335	P12467	protein_cc	bHLHa31|	Fer3-like (	Ferd3l
4286	Twist1	1.539	1.646	2.532	0.420	0.291	0.258	2.432	3.054	3.772	22160	Twist1	chr12	+	NM_0116	P20282	protein_cc	bHLHa38|	twist basi	Twist1
4287	Hdac9	1.304	0.764	0.996	0.330	0.050	0.994	3.729	4.111	3.722	79221	Hdac9	chr12	-	NM_0012	P24183	protein_cc	AV022454	histone de	Hdac9
4288	Prps1l1	1.000	1.000	1.000	1.000	1.000	1.000	0.000	0.000	0.000	75456	Prps1l1	chr12	+	NM_0292	P18257	protein_cc	1700011K	phosphori	Prps1l1
4289	Snx13	0.944	0.827	0.780	0.579	0.187	0.346	7.174	7.091	6.816	217463	Snx13	chr12	+	NM_0010	P21728	protein_cc	mKIAA071	sorting ne	Snx13
4290	Ahr	0.738	0.962	0.710	0.063	0.721	0.293	7.714	7.276	7.220	11622	Ahr	chr12	-	NM_0134	P15351	protein_cc	Ah| Ahh|	aryl-hydro	Ahr
4291	9130015A	37.081	1.072	39.759	0.032	0.842	0.055	0.330	5.543	5.643	71581	9130015A	chr12	-	NR_04505	.	lncRNA	10004357	RIKEN cDN	9130015A
4292	Agr3	1.000	1.000	1.000	1.000	1.000	1.000	0.000	0.000	0.000	403205	Agr3	chr12	+	NM_2075	P20982	protein_cc	BCMP11|	anterior g	Agr3
4293	Agr2	0.669	0.995	0.666	0.181	1.000	0.423	0.587	0.008	0.000	23795	Agr2	chr12	+	NM_0117	P10980	protein_cc	Agr2h| Gc	anterior g	Agr2
4294	Tspan13	0.726	0.642	0.466	0.309	0.172	0.221	4.420	3.958	3.320	66109	Tspan13	chr12	-	NM_0253	P9145	protein_cc	1100001I2	tetraspani	Tspan13
4295	Gm5434	4.400	0.518	2.278	0.086	0.233	0.116	0.377	2.515	1.565	432649	Gm5434	chr12	+	NR_00364	.	misc_RNA	EG432649	predicted	Gm5434
4296	Bzw2	5.769	0.947	5.465	0.000	0.866	0.245	7.156	9.684	9.606	66912	Bzw2	chr12	-	NM_0258	P8350	protein_cc	1110001I2	basic leuci	Bzw2
4297	Ankmy2	0.826	0.682	0.564	0.491	0.143	0.345	6.099	5.824	5.272	217473	Ankmy2	chr12	+	NM_1460	P5703	protein_cc	AI035571|	ankyrin re	Ankmy2
4298	Lrrc72	1.000	1.000	1.000	1.000	1.000	1.000	0.000	0.000	0.000	71156	Lrrc72	chr12	-	NM_0276	P2830	protein_cc	1700108M	leucine ric	Lrrc72
4299	Sostdc1	0.461	3.376	1.557	0.001	0.000	0.044	1.169	0.052	1.808	66042	Sostdc1	chr12	+	NM_0253	P8150	protein_cc	0610006G	sclerostin	Sostdc1
4300	Ispd	0.572	2.671	1.527	0.196	0.021	0.177	4.056	3.249	4.666	75847	Ispd	chr12	+	NM_0012	P3538	protein_cc	4930579E	isoprenoid	Ispd
4301	Meox2	0.552	0.990	0.547	0.182	1.000	0.423	0.871	0.015	0.000	17286	Meox2	chr12	+	NM_0085	P8259	protein_cc	AI528662|	mesenchy	Meox2
4302	Agmo	0.256	0.492	0.126	0.000	0.067	0.010	5.814	3.848	2.823	319660	Agmo	chr12	+	NM_1787	P2186	protein_cc	A530016C	alkylglyce	Agmo
4303	Dgkb	1.005	1.405	1.412	0.000	0.182	0.423	0.000	0.008	0.498	217480	Dgkb	chr12	+	NM_1786	P4775	protein_cc	6430574F	diacylglyc	Dgkb
4304	4930428E	1.000	1.000	1.000	1.000	1.000	1.000	0.000	0.000	0.000	73867	4930428E	chr12	-	NR_04594	.	lncRNA	.	RIKEN cDN	4930428E
4305	Etv1	0.094	1.289	0.121	0.000	0.257	0.020	3.512	0.102	0.468	14009	Etv1	chr12	+	NM_0079	P21487	protein_cc	ER81| Etsr	ets variant	Etv1
4306	Arl4a	0.952	1.071	1.020	0.509	0.744	0.954	9.340	9.269	9.368	11861	Arl4a	chr12	-	NM_0074	P13606	protein_cc	AI467555|	ADP-ribos	Arl4a
4307	Scin	1.005	1.439	1.446	0.000	0.182	0.423	0.000	0.008	0.532	20259	Scin	chr12	-	NM_0011	P26906	protein_cc	adseverin	scinderin	Scin
4308	Lsmem1	0.604	0.453	0.274	0.234	1.000	0.018	1.869	1.141	0.000	380755	Lsmem1	chr12	-	NM_0010	P15577	protein_cc	Gm889	leucine-ric	Lsmem1
4309	Ifrd1	1.534	1.360	2.086	0.081	0.289	0.268	9.351	9.969	10.412	15982	Ifrd1	chr12	-	NM_0135	P7287	protein_cc	Ifnl| PC4|	interferon	Ifrd1
4310	Gm7008	0.716	0.995	0.712	0.182	1.000	0.423	0.490	0.008	0.000	629820	Gm7008	chr12	+	NR_04515	.	lncRNA	EG629820	predicted	Gm7008
4311	Zfp277	0.780	1.489	1.161	0.011	0.022	0.465	6.767	6.408	6.982	246196	Zfp277	chr12	-	NM_1788	P24093	protein_cc	2410017E	zinc finger	Zfp277
4312	Dock4	0.470	1.003	0.472	0.016	0.992	0.184	4.146	3.058	3.062	238130	Dock4	chr12	+	NM_1728	P894	protein_cc	5330406C	dedicator	Dock4
4313	Immp2l	0.610	1.202	0.733	0.008	0.003	0.168	8.364	7.651	7.917	93757	Immp2l	chr12	+	NM_0531	P2444	protein_cc	AI853880|	IMP2 inne	Immp2l
4314	Lrrn3	0.167	0.190	0.032	0.015	1.000	0.073	4.975	2.397	0.000	16981	Lrrn3	chr12	-	NM_0012	P25865	protein_cc	NLRR-3	leucine ric	Lrrn3
4315	Dnajb9	2.826	0.694	1.960	0.001	0.046	0.191	7.511	9.010	8.482	27362	Dnajb9	chr12	-	NM_0137	P4801	protein_cc	AA408011	DnaJ heat	Dnajb9
4316	Gm2027	1.114	1.282	1.428	0.744	0.482	0.439	2.232	2.387	2.746	10003906	Gm2027	chr12	+	NR_04511	.	misc_RNA	10003906	predicted	Gm2027
4317	Pnpla8	0.768	1.300	0.999	0.086	0.029	0.995	9.327	8.946	9.325	67452	Pnpla8	chr12	+	NM_0261	P7275	protein_cc	1200006O	patatin-lik	Pnpla8
4318	Nrcam	0.914	1.365	1.247	0.835	0.344	0.657	0.817	0.687	1.136	319504	Nrcam	chr12	+	NM_1769	P6098	protein_cc	Bravo| C0	neuronal c	Nrcam
4319	Stxbp6	0.256	2.453	0.629	0.001	0.001	0.107	7.235	5.272	6.567	217517	Stxbp6	chr12	-	NM_1445	P11424	protein_cc	BC024598	syntaxin b	Stxbp6
4320	Nova1	1.010	1.825	1.844	0.000	0.182	0.423	0.000	0.015	0.883	664883	Nova1	chr12	-	NM_0213	P15328	protein_cc	9430099M	neuro-onc	Nova1
4321	Foxg1	1.000	1.000	1.000	1.000	1.000	1.000	0.000	0.000	0.000	15228	Foxg1	chr12	+	NM_0011	P5777	protein_cc	2900064B	forkhead b	Foxg1
4322	3110039M	1.000	1.000	1.000	1.000	1.000	1.000	0.000	0.000	0.000	67293	3110039M	chr12	+	NR_02673	.	lncRNA	.	RIKEN cDN	3110039M
4323	1810007C	1.000	1.000	1.000	1.000	1.000	1.000	0.000	0.000	0.000	69054	1810007C	chr12	-	NR_04547	.	lncRNA	.	RIKEN cDN	1810007C
4324	Prkd1	2.734	0.517	1.413	0.305	0.427	0.423	0.001	1.452	0.499	18760	Prkd1	chr12	-	NM_0088	P25711	protein_cc	Pkcm| PKI	protein kir	Prkd1
4325	1700008C	1.000	1.000	1.000	1.000	1.000	1.000	0.000	0.000	0.000	75494	1700008C	chr12	+	NR_12646	.	lncRNA	.	RIKEN cDN	1700008C
4326	G2e3	1.606	0.992	1.593	0.011	0.951	0.121	5.715	6.399	6.387	217558	G2e3	chr12	+	NM_0011	P10982	protein_cc	6030408C	G2/M-pha	G2e3
4327	Scfd1	2.293	0.572	1.310	0.000	0.009	0.451	8.618	9.815	9.008	76983	Scfd1	chr12	+	NM_0013	P19779	protein_cc	3110021P	Sec1 fami	Scfd1
4328	Coch	1.005	1.439	1.446	0.000	0.182	0.423	0.000	0.008	0.532	12810	Coch	chr12	+	NM_0011	P20156	protein_cc	AW12293	cochlin	Coch
4329	Strn3	1.598	0.803	1.284	0.005	0.022	0.222	9.273	9.949	9.633	94186	Strn3	chr12	-	NM_0529	P24265	protein_cc	Gs2na| SC	striatin, ca	Strn3
4330	Ap4s1	0.654	1.052	0.688	0.001	0.512	0.016	7.519	6.905	6.978	11782	Ap4s1	chr12	+	NM_0217	P828	protein_cc	AI314282	adaptor-re	Ap4s1
4331	Hectd1	0.798	0.809	0.646	0.002	0.002	0.005	10.846	10.520	10.215	207304	Hectd1	chr12	-	NM_1447	P19804	protein_cc	A630086P	HECT dom	Hectd1
4332	Heatr5a	0.719	1.037	0.746	0.018	0.569	0.149	5.930	5.455	5.508	320487	Heatr5a	chr12	-	NM_1771	P22870	protein_cc	C230081H	HEAT repe	Heatr5a
4333	Dtd2	1.273	1.032	1.313	0.418	0.880	0.492	4.583	4.931	4.976	328092	Dtd2	chr12	-	NM_0295	P2844	protein_cc	4930578F	D-tyrosyl-	Dtd2
4334	Gpr33	1.000	1.000	1.000	1.000	1.000	1.000	0.000	0.000	0.000	14762	Gpr33	chr12	-	NM_0081	P23349	protein_cc	.	G protein-	Gpr33
4335	Nubpl	0.981	1.390	1.364	0.963	0.106	0.607	5.481	5.454	5.929	76826	Nubpl	chr12	+	NM_0297	P9693	protein_cc	2410170E	nucleotide	Nubpl
4336	Arhgap5	1.275	0.660	0.842	0.073	0.017	0.362	10.011	10.361	9.762	11855	Arhgap5	chr12	+	NM_0097	P22528	protein_cc	p190-B| p	Rho GTPas	Arhgap5
4337	1700031P	1.000	1.000	1.000	1.000	1.000	1.000	0.000	0.000	0.000	73278	1700031P	chr12	-	NR_04591	.	lncRNA	.	RIKEN cDN	1700031P
4338	Akap6	0.386	1.346	0.520	0.182	0.200	0.530	1.405	0.033	0.461	238161	Akap6	chr12	+	NM_1981	P9547	protein_cc	AI482140|	A kinase (	Akap6
4339	Npas3	4.532	0.471	2.135	0.149	0.318	0.423	0.001	2.181	1.095	27386	Npas3	chr12	+	NM_0137	P22177	protein_cc	4930423H	neuronal P	Npas3
4340	Egln3	1.638	0.406	0.666	0.000	0.000	0.142	7.462	8.174	6.875	112407	Egln3	chr12	-	NM_0281	P17164	protein_cc	2610021G	egl-9 fami	Egln3
4341	1700104L	1.000	1.000	1.000	1.000	1.000	1.000	0.000	0.000	0.000	73592	1700104L	chr12	+	NR_10803	.	lncRNA	.	RIKEN cDN	1700104L
4342	Gm7550	1.000	1.000	1.000	1.000	1.000	1.000	0.000	0.000	0.000	665239	Gm7550	chr12	-	NR_03368	.	lncRNA	EG665239	predicted	Gm7550
4343	Sptssa	0.335	2.091	0.701	0.000	0.001	0.063	8.743	7.165	8.229	104725	Sptssa	chr12	-	NM_1340	P14566	protein_cc	1110002B	serine pal	Sptssa
4344	Eapp	2.375	0.788	1.871	0.000	0.124	0.149	7.978	9.226	8.881	66266	Eapp	chr12	-	NM_0254	P9015	protein_cc	1810011O	E2F-assoc	Eapp
4345	Snx6	2.135	0.702	1.498	0.002	0.000	0.205	7.534	8.628	8.117	72183	Snx6	chr12	-	NM_0269	P13071	protein_cc	2010006G	sorting ne	Snx6
4346	Cfl2	0.744	1.333	0.992	0.076	0.085	0.955	9.134	8.708	9.122	12632	Cfl2	chr12	-	NM_0076	P11543	protein_cc	.	cofilin 2, n	Cfl2
4347	Baz1a	3.387	0.550	1.863	0.002	0.008	0.004	8.275	10.035	9.173	217578	Baz1a	chr12	-	NM_0138	P20627	protein_cc	Acf1| B93	bromodor	Baz1a
4348	2700097O	1.552	0.608	0.943	0.005	0.087	0.906	5.975	6.609	5.890	72658	2700097O	chr12	-	NR_03076	.	protein_cc	AW55221	RIKEN cDN	2700097O
4349	Srp54a	1.754	0.809	1.419	0.002	0.021	0.053	8.259	9.069	8.764	24067	Srp54a	chr12	+	NM_0118	P22713	protein_cc	54kDa| Sr	signal recc	Srp54a
4350	1700047I1	1.536	1.005	1.543	0.008	0.946	0.024	6.958	7.577	7.584	10010180	1700047I1	chr12	+	NM_0011	P5222	protein_cc	.	RIKEN cDN	1700047I1
4351	Fam177a	1.000	1.000	1.000	1.000	1.000	1.000	0.000	0.000	0.000	73385	Fam177a	chr12	+	NM_0285	P5222	protein_cc	1700047I1	family wit	Fam177a
4352	Srp54b	2.322	0.757	1.759	0.004	0.029	0.096	7.782	8.997	8.596	665155	Srp54b	chr12	+	NM_0011	P7559	protein_cc	.	signal recc	Srp54b
4353	Srp54c	2.173	0.740	1.607	0.002	0.031	0.074	7.808	8.928	8.492	10010180	Srp54c	chr12	+	NM_0011	P11091	protein_cc	E330029E	signal recc	Srp54c
4354	Ppp2r3c	1.381	0.857	1.184	0.012	0.128	0.189	8.523	8.988	8.766	59032	Ppp2r3c	chr12	-	NM_0215	P5685	protein_cc	4930511A	protein ph	Ppp2r3c
4355	1110008L	1.295	0.866	1.121	0.033	0.199	0.379	8.549	8.922	8.714	66132	1110008L	chr12	+	NM_0253	P7366	protein_cc	Kiaa0391|	RIKEN cDN	1110008L

4356	Psma6	1.216	1.361	1.656	0.187	0.078	0.131	9.916	10.199	10.644	26443	Psma6	chr12	+	NM_0119	P23983	protein_cc	IOTA	proteason	Psma6
4357	Nfkbia	4.056	0.560	2.271	0.000	0.004	0.008	11.508	13.529	12.692	18035	Nfkbia	chr12	-	NM_0109	P17447	protein_cc	AI462015	nuclear fa	Nfkbia
4358	Aldoart2	0.380	1.311	0.499	0.194	0.593	0.535	2.117	0.722	1.112	79459	Aldoart2	chr12	+	NM_0012	P4390	protein_cc	4933425L	aldolase 1	Aldoart2
4359	Insm2	2.745	0.645	1.771	0.304	0.565	0.423	0.001	1.457	0.825	56856	Insm2	chr12	+	NM_0202	P13359	protein_cc	Mlt1	insulinoma	Insm2
4360	Ralgapa1	1.802	0.643	1.159	0.000	0.000	0.203	5.919	6.768	6.131	56784	Ralgapa1	chr12	-	NM_0011	P16253	protein_cc	2310003F	Ral GTPas	Ralgapa1
4361	Brms1l	1.637	1.562	2.557	0.065	0.115	0.137	2.998	3.709	4.352	52592	Brms1l	chr12	+	NM_0010	P26257	protein_cc	07100080	breast car	Brms1l
4362	Gm19990	1.000	1.000	1.000	1.000	1.000	1.000	0.000	0.000	0.000	10050396	Gm19990	chr12	+	NR_04504	.	lncRNA	.	predicted	Gm19990
4363	Mbip	1.121	0.464	0.520	0.780	0.188	0.276	4.855	5.021	3.912	217588	Mbip	chr12	-	NM_1454	P25238	protein_cc	4933408E	MAP3K12	Mbip
4364	E030019B	1.000	1.000	1.000	1.000	1.000	1.000	0.000	0.000	0.000	104798	E030019B	chr12	-	NR_04508	.	lncRNA	AI413669	RIKEN cDN	E030019B
4365	Nkx2-1	0.410	2.181	0.894	0.184	0.017	0.883	1.325	0.039	1.164	21869	Nkx2-1	chr12	-	NM_0093	P10503	protein_cc	AV026640	NK2 home	Nkx2-1
4366	C87198	1.000	1.000	1.000	1.000	1.000	1.000	0.000	0.000	0.000	97836	C87198	chr12	+	NR_04600	.	lncRNA	.	expressed	C87198
4367	Nkx2-9	1.000	1.000	1.000	1.000	1.000	1.000	0.000	0.000	0.000	18094	Nkx2-9	chr12	-	NM_0087	P8961	protein_cc	Nkx-2.9	N	NK2 home	Nkx2-9
4368	Pax9	0.155	1.225	0.190	0.039	0.542	0.214	3.454	0.763	1.055	18511	Pax9	chr12	+	NM_0110	P24539	protein_cc	Pax-9	paired box	Pax9
4369	Slc25a21	0.094	2.411	0.224	0.007	0.026	0.117	7.216	3.787	5.057	217593	Slc25a21	chr12	-	NM_0011	P7201	protein_cc	99300336	solute car	Slc25a21
4370	Prps1l3	0.966	1.041	1.005	0.845	0.828	0.978	8.163	8.113	8.171	328099	Prps1l3	chr12	+	NM_0010	P13364	protein_cc	328099	A	phosphori	Prps1l3
4371	Mipol1	0.341	1.508	0.515	0.039	0.251	0.342	4.711	3.161	3.753	73490	Mipol1	chr12	+	NM_0011	P4698	protein_cc	17000810	mirror-ima	Mipol1
4372	Foxa1	0.525	1.069	0.561	0.131	0.860	0.283	6.508	5.578	5.674	15375	Foxa1	chr12	-	NM_0082	P20138	protein_cc	Hnf-3a	H	forkhead b	Foxa1
4373	4921506M	0.726	1.387	1.007	0.187	0.187	0.988	0.477	0.015	0.487	.	4921506M	chr12	+	NM_0010	P2136	.	.	.	4921506M
4374	4921518K	1.015	2.326	2.360	0.000	0.182	0.423	0.000	0.021	1.239	78758	4921518K	chr12	-	NR_13111	.	lncRNA	.	RIKEN cDN	4921518K
4375	BC042761	1.000	1.000	1.000	1.000	1.000	1.000	0.000	0.000	0.000	217601	BC042761	chr12	-	NR_13111	.	lncRNA	.	cDNA seq	BC042761
4376	Sstr1	1.581	0.879	1.390	0.301	0.744	0.423	0.000	0.661	0.476	20605	Sstr1	chr12	+	NM_0092	P7766	protein_cc	Smstr-1	S	somatosta	Sstr1
4377	Clec14a	0.065	5.124	0.333	0.000	0.001	0.008	7.124	3.180	5.538	66864	Clec14a	chr12	-	NM_0258	P2649	protein_cc	1200003C	C-type lec	Clec14a
4378	Sec23a	0.875	1.143	0.999	0.124	0.016	0.996	9.576	9.383	9.576	20334	Sec23a	chr12	-	NM_0091	P3728	protein_cc	Msec23	S	SEC23 hor	Sec23a
4379	Gemin2	0.238	3.585	0.853	0.000	0.004	0.504	5.984	3.913	5.755	66603	Gemin2	chr12	+	NM_0256	P5309	protein_cc	gemin-2		gem (nucl	Gemin2
4380	Trappc6b	1.259	0.913	1.149	0.038	0.187	0.460	6.808	7.140	7.008	78232	Trappc6b	chr12	-	NM_0300	P15148	protein_cc	5830498C	trafficking	Trappc6b
4381	Pnn	2.470	0.564	1.393	0.000	0.011	0.439	7.414	8.718	7.892	18949	Pnn	chr12	+	NM_0088	P10734	protein_cc	AU045199	pinin	Pnn
4382	Mia2	0.412	0.935	0.385	0.000	0.666	0.024	8.319	7.038	6.941	338320	Mia2	chr12	+	NM_1773	P16347	protein_cc	.	melanoma	Mia2
4383	Ctage5	0.560	1.010	0.566	0.000	0.834	0.013	10.918	10.082	10.097	217615	Ctage5	chr12	+	NM_0011	P348	protein_cc	D12Bwg0	CTAGE far	Ctage5
4384	Fbxo33	1.639	1.134	1.858	0.012	0.329	0.059	7.002	7.715	7.896	70611	Fbxo33	chr12	-	NM_0010	P9365	protein_cc	5730501N	F-box prot	Fbxo33
4385	Gm20063	0.405	0.985	0.399	0.181	1.000	0.423	1.324	0.022	0.000	10050410	Gm20063	chr12	-	NR_04504	.	lncRNA	.	predicted	Gm20063
4386	Lrfn5	1.000	1.000	1.000	1.000	1.000	1.000	0.000	0.000	0.000	238205	Lrfn5	chr12	+	NM_1787	P21889	protein_cc	AI427653	leucine ric	Lrfn5
4387	Spanxn4	1.000	1.000	1.000	1.000	1.000	1.000	0.000	0.000	0.000	73309	Spanxn4	chr12	+	NR_13194	.	lncRNA	1700047L	SPANX far	Spanxn4
4388	Fscb	1.000	1.000	1.000	1.000	1.000	1.000	0.000	0.000	0.000	623046	Fscb	chr12	-	NM_0011	P14990	protein_cc	EG623046	fibrous sh	Fscb
4389	Gm527	8.692	0.956	8.310	0.048	0.909	0.102	1.089	4.209	4.144	217648	Gm527	chr12	+	NM_0010	P4086	protein_cc	.	predicted	Gm527
4390	Klhl28	1.735	1.050	1.822	0.035	0.776	0.185	5.174	5.969	6.039	66689	Klhl28	chr12	-	NM_0257	P10893	protein_cc	2810440N	kelch-like	Klhl28
4391	Fam179b	1.435	0.907	1.301	0.032	0.074	0.287	6.953	7.474	7.332	328108	Fam179b	chr12	+	NM_1778	P10749	protein_cc	A430041B	family wit	Fam179b
4392	Prpf39	1.115	0.887	0.989	0.000	0.337	0.958	8.447	8.604	8.431	328110	Prpf39	chr12	+	NM_1778	P18824	protein_cc	Srcs1	pre-mRNA	Prpf39
4393	Fkbp3	0.780	0.941	0.734	0.001	0.581	0.158	8.487	8.128	8.041	30795	Fkbp3	chr12	-	NM_0139	P24882	protein_cc	25kDa	FK	FK506 bin	Fkbp3
4394	Fancm	0.964	1.489	1.436	0.909	0.250	0.406	4.317	4.264	4.839	104806	Fancm	chr12	+	NM_1789	P24851	protein_cc	AI427100	Fanconi ar	Fancm
4395	Mis18bp1	0.497	1.317	0.654	0.141	0.519	0.347	3.243	2.233	2.631	217653	Mis18bp1	chr12	-	NM_1725	P9697	protein_cc	6720403H	MIS18 bin	Mis18bp1
4396	Wdr20rt	6.276	0.159	0.999	0.101	1.000	0.423	0.001	2.651	0.000	70948	Wdr20rt	chr12	+	NM_0276	P11758	protein_cc	4921538B	WD repea	Wdr20rt
4397	Rpl10l	0.676	1.797	1.214	0.188	0.000	0.588	0.587	0.021	0.867	238217	Rpl10l	chr12	-	NM_0011	P13968	protein_cc	EG238217	ribosomal	Rpl10l
4398	Mdga2	0.673	1.424	0.958	0.185	0.186	0.933	0.587	0.015	0.525	320772	Mdga2	chr12	-	NM_2070	P8314	protein_cc	6720489L	MAM dom	Mdga2
4399	Gm17821	3.139	0.541	1.697	0.000	0.001	0.171	3.335	4.985	4.098	10031687	Gm17821	chr12	-	NR_03314	.	pseudoge	.	predicted	Gm17821
4400	Rps29	1.132	1.234	1.397	0.134	0.017	0.015	11.328	11.507	11.810	20090	Rps29	chr12	-	NM_0090	P8230	protein_cc	.	ribosomal	Rps29
4401	Lrr1	0.217	0.966	0.210	0.054	1.000	0.238	2.251	0.049	0.000	69706	Lrr1	chr12	+	NM_0010	P21921	protein_cc	2410005L	leucine ric	Lrr1
4402	Rpl36al	1.212	1.032	1.252	0.006	0.405	0.042	11.194	11.472	11.518	66483	Rpl36al	chr12	-	NM_0255	P19849	protein_cc	2410038A	ribosomal	Rpl36al
4403	Mgat2	1.274	0.992	1.264	0.001	0.953	0.422	8.587	8.936	8.925	217664	Mgat2	chr12	+	NM_1460	P21514	protein_cc	AA407964	mannosid	Mgat2
4404	Dnaaf2	0.493	1.457	0.718	0.001	0.040	0.073	5.192	4.172	4.714	109065	Dnaaf2	chr12	-	NM_0272	P910	protein_cc	kintoun	k	dynein, ax	Dnaaf2
4405	9330151L	0.527	0.944	0.498	0.073	0.885	0.352	2.950	2.027	1.945	414085	9330151L	chr12	+	NR_03322	.	lncRNA	.	RIKEN cDN	9330151L
4406	Pole2	0.680	1.923	1.307	0.475	0.106	0.569	2.791	2.234	3.177	18974	Pole2	chr12	-	NM_0111	P9361	protein_cc	.	polymeras	Pole2
4407	Klhdc1	2.181	0.630	1.373	0.000	0.024	0.423	0.000	1.125	0.458	271005	Klhdc1	chr12	+	NM_1782	P18435	protein_cc	.	kelch dom	Klhdc1
4408	Klhdc2	1.203	1.122	1.349	0.247	0.360	0.149	9.021	9.287	9.453	69554	Klhdc2	chr12	+	NM_0271	P20934	protein_cc	2310022K	kelch dom	Klhdc2
4409	Nemf	1.993	0.842	1.678	0.003	0.044	0.069	8.456	9.451	9.203	66244	Nemf	chr12	-	NM_0254	P11587	protein_cc	15000111	nuclear ex	Nemf
4410	Arf6	2.046	0.778	1.592	0.001	0.048	0.083	9.956	10.988	10.627	11845	Arf6	chr12	+	NM_0074	P24229	protein_cc	AI788669	ADP-ribos	Arf6
4411	5830428M	1.000	1.000	1.000	1.000	1.000	1.000	0.000	0.000	0.000	76062	5830428M	chr12	-	NR_03806	.	lncRNA	10004086	RIKEN cDN	5830428M
4412	Vcpkmt	0.679	2.260	1.535	0.094	0.083	0.444	3.809	3.251	4.427	207965	Vcpkmt	chr12	-	NM_0010	P1520	protein_cc	Gm71	M	valosin co	Vcpkmt
4413	Sos2	1.175	1.364	1.602	0.263	0.265	0.346	6.840	7.073	7.520	20663	Sos2	chr12	-	NM_0011	P9119	protein_cc	mSOS-2	S	son of sev	Sos2
4414	L2hgdh	0.179	2.282	0.408	0.000	0.000	0.017	7.398	4.915	6.105	217666	L2hgdh	chr12	-	NM_1454	P11620	protein_cc	BC016226	L-2-hydrox	L2hgdh
4415	Atp5s	0.856	1.186	1.015	0.057	0.163	0.939	5.968	5.743	5.989	68055	Atp5s	chr12	+	NM_0265	P19876	protein_cc	1110015E	ATP synth	Atp5s
4416	Cdkl1	2.742	0.364	1.000	0.020	1.000	0.423	0.001	1.456	0.000	71091	Cdkl1	chr12	-	NM_1832	P18298	protein_cc	49334110	cyclin-dep	Cdkl1
4417	4930512B	1.000	1.000	1.000	1.000	1.000	1.000	0.000	0.000	0.000	74724	4930512B	chr12	+	NR_03357	.	lncRNA	4930472D	RIKEN cDN	4930512B
4418	Map4k5	2.189	0.826	1.807	0.011	0.171	0.132	6.053	7.184	6.907	399510	Map4k5	chr12	-	NM_2015	P13441	protein_cc	4432415E	mitogen-a	Map4k5
4419	4931403G	0.537	0.619	0.333	0.355	1.000	0.423	1.588	0.692	0.000	70934	4931403G	chr12	+	NR_03817	.	lncRNA	.	RIKEN cDN	4931403G
4420	Atl1	1.728	1.477	2.552	0.005	0.084	0.107	3.421	4.210	4.773	73991	Atl1	chr12	+	NM_1786	P9942	protein_cc	4930435M	atlastin GT	Atl1
4421	Gm3086	2.526	0.684	1.728	0.370	0.647	0.544	0.406	1.742	1.194	10004099	Gm3086	chr12	+	NR_03660	.	misc_RNA	10004099	predicted	Gm3086
4422	Sav1	4.804	0.583	2.802	0.000	0.035	0.175	5.697	7.961	7.183	64010	Sav1	chr12	-	NM_0220	P20741	protein_cc	1700040G	salvador f	Sav1
4423	Nin	1.001	1.454	1.455	0.998	0.319	0.322	4.760	4.761	5.301	18080	Nin	chr12	-	NM_0010	P5713	protein_cc	3110068G	ninein	Nin
4424	Abhd12b	1.023	3.106	3.177	0.000	0.069	0.267	0.000	0.033	1.668	10050428	Abhd12b	chr12	+	NM_0011	P12944	protein_cc	Gm804	abhydrola	Abhd12b
4425	Pygl	0.229	1.315	0.301	0.000	0.303	0.034	12.402	10.275	10.671	110095	Pygl	chr12	-	NM_1331	P6769	protein_cc	.	liver glyco	Pygl
4426	F730035M	0.342	1.739	0.594	0.183	0.000	0.605	1.588	0.039	0.837	414124	F730035M	chr12	+	NR_04517	.	lncRNA	.	RIKEN cDN	F730035M
4427	Trim9	1.000	1.000	1.000	1.000	1.000	1.000	0.000	0.000	0.000	94090	Trim9	chr12	-	NM_0011	P14006	protein_cc	.	tripartite	Trim9
4428	Tmx1	1.106	0.823	0.910	0.089	0.018	0.294	9.704	9.848	9.568	72736	Tmx1	chr12	+	NM_0283	P23977	protein_cc	2810425A	thioredoxi	Tmx1
4429	Frmd6	0.991	0.887	0.879	0.947	0.518	0.615	5.405	5.392	5.219	319710	Frmd6	chr12	+	NM_0281	P1603	protein_cc	2610019N	FERM dom	Frmd6
4430	Actr10	1.271	1.325	1.685	0.101	0.044	0.026	9.042	9.388	9.795	56444	Actr10	chr12	+	NM_0197	P15152	protein_cc	Actr11	A	ARP10 act	Actr10
4431	Psma3	1.365	0.876	1.196	0.156	0.434	0.336	9.736	10.185	9.994	19167	Psma3	chr12	+	NM_0111	P822	protein_cc	Lmpc8	proteason	Psma3
4432	3110056K	1.430	0.630	0.902	0.042	0.140	0.869	2.020	2.536	1.871	73204	3110056K	chr12	-	NR_04505	.	lncRNA	4921536K	RIKEN cDN	3110056K
4433	Arid4a	0.827	0.700	0.579	0.113	0.041	0.065	9.068	8.795	8.280	238247	Arid4a	chr12	+	NM_0010	P1306	protein_cc	A630009N	AT rich int	Arid4a
4434	Tomm20l	0.609	1.281	0.780	0.243	0.580	0.074	5.700	4.984	5.342	75266	Tomm20l	chr12	+	NM_0292	P5464	protein_cc	4930553D	translocas	Tomm20l
4435	Timm9	0.522	1.150	0.600	0.042	0.428	0.218	9.238	8.299	8.501	30056	Timm9	chr12	-	NM_0010	P18085	protein_cc	2810011L	translocas	Timm9
4436	2700049A	0.925	0.641	0.593	0.834	0.143	0.545	4.410	4.298	3.657	76967	2700049A	chr12	+	NM_0298	P16824	protein_cc	mKIAA058	RIKEN cDN	2700049A
4437	Dact1	0.766	2.154	1.651	0.448	0.016	0.181	5.857	5.472	6.579	59036	Dact1	chr12	+	NM_0011	P10784	protein_cc	4921528D	dishevelle	Dact1
4438	4930404H	0.067	1.903	0.127	0.006	0.209	0.097	4.034	0.125	1.053	73815	4930404H	chr12	+	NR_04594	.	lncRNA	.	RIKEN cDN	4930404H
4439	Daam1	0.297	1.037	0.308	0.000	0.839	0.013	8.963	7.212	7.265	208846	Daam1	chr12	+	NM_1724	P10047	protein_cc	.	dishevelle	Daam1
4440	Gpr135	1.027	3.491	3.584	0.000	0.014	0.123	0.001	0.038	1.842	238252	Gpr135	chr12	-	NM_1817	P17780	protein_cc	PAFR	G protein-	Gpr135
4441	L3hypdh	0.228	1.153	0.264	0.003	0.763	0.094	7.652	5.522	5.728	67217	L3hypdh	chr12	-	NM_0260	P7202	protein_cc	1500055F	L-3-hydrox	L3hypdh
4442	Jkamp	0.665	1.016	0.676	0.000	0.937	0.261	8.946	8.358	8.381	104771	Jkamp	chr12	+	NM_0012	P20345	protein_cc	1200003C	JNK1/MAP	Jkamp
4443	Ccdc175	0.695	1.048	0.729	0.010	0.761	0.170	7.557	7.033	7.100	73936	Ccdc175	chr12	-	NM_0286	P13349	protein_cc	4930403N	coiled-coil	Ccdc175
4444	Rtn1	0.441	0.315	0.139	0.001	0.007	0.017	4.753	3.574	1.909	104001	Rtn1	chr12	-	NM_0010	P24962	protein_cc	Nsp	reticulon 1	Rtn1
4445	Lrrc9	0.716	0.995	0.712	0.182	1.000	0.423	0.490	0.008	0.000	78257	Lrrc9	chr12	+	NM_0300	P19517	protein_cc	49215290	leucine ric	Lrrc9
4446	Pcnxl4	0.840	1.644	1.380	0.531	0.128	0.457	6.293	6.041	6.758	.	Pcnxl4	chr12	+	NM_0263	P9272	.	.	.	Pcnxl4

4447	Dhrs7	0.213	0.928	0.198	0.001	0.787	0.019	9.225	6.994	6.887	66375	Dhrs7	chr12	-	NM_0255	P21739	protein_cc	2310016E	dehydroge	Dhrs7
4448	Ppm1a	0.479	1.731	0.829	0.000	0.019	0.329	10.199	9.137	9.929	19042	Ppm1a	chr12	+	NM_0089	P1974	protein_cc	2310003C	protein ph	Ppm1a
4449	4930447C	1.000	1.000	1.000	1.000	1.000	1.000	0.000	0.000	0.000	75801	4930447C	chr12	-	NM_0294	P8914	protein_cc	4921504I0	RIKEN cDN	4930447C
4450	Six6	1.000	1.000	1.000	1.000	1.000	1.000	0.000	0.000	0.000	20476	Six6	chr12	+	NM_0113	P1032	protein_cc	Optx2 | Six	sine oculis	Six6
4451	Six1	1.019	2.651	2.701	0.000	0.000	0.002	0.000	0.027	1.434	20471	Six1	chr12	-	NM_0091	P18679	protein_cc	BB138287	sine oculis	Six1
4452	Six4	1.000	1.000	1.000	1.000	1.000	1.000	0.000	0.000	0.000	20474	Six4	chr12	-	NM_0113	P19128	protein_cc	AI047561 |	sine oculis	Six4
4453	Mnat1	0.667	1.526	1.018	0.011	0.008	0.875	7.270	6.686	7.296	17420	Mnat1	chr12	+	NM_0086	P26508	protein_cc	E130115E	menage a	Mnat1
4454	Trmt5	1.977	0.719	1.422	0.015	0.198	0.438	4.909	5.892	5.417	76357	Trmt5	chr12	-	NM_0295	P25166	protein_cc	2610027O	TRM5 tRN	Trmt5
4455	Slc38a6	0.607	0.725	0.440	0.064	0.350	0.023	5.631	4.912	4.447	625098	Slc38a6	chr12	+	NM_0010	P12899	protein_cc	AW32267	solute car	Slc38a6
4456	D830013O	1.019	2.762	2.814	0.000	0.181	0.423	0.000	0.027	1.493	380765	D830013O	chr12	-	NR_04601	.	lncRNA	Gm891	RIKEN cDN	D830013O
4457	Tmem30b	0.367	1.674	0.614	0.003	0.066	0.222	8.160	6.713	7.456	238257	Tmem30b	chr12	-	NM_1787	P5709	protein_cc	9130011B	transmem	Tmem30b
4458	2210039B	0.373	1.387	0.518	0.014	0.589	0.393	2.950	1.528	2.000	73644	2210039B	chr12	+	NR_04498	.	lncRNA		RIKEN cDN	2210039B
4459	Prkch	6.795	0.876	5.955	0.000	0.319	0.054	4.644	7.409	7.219	18755	Prkch	chr12	+	NM_0088	P13674	protein_cc	Pkch	protein kin	Prkch
4460	Hif1a	7.670	0.877	6.727	0.000	0.308	0.038	7.779	10.718	10.529	15251	Hif1a	chr12	+	NM_0104	P2164	protein_cc	AA959795	hypoxia in	Hif1a
4461	Snapc1	5.093	0.470	2.393	0.002	0.007	0.022	5.866	8.214	7.125	75627	Snapc1	chr12	+	NM_1783	P23075	protein_cc	2700033G	small nucl	Snapc1
4462	Syt16	1.000	1.000	1.000	1.000	1.000	1.000	0.000	0.000	0.000	238266	Syt16	chr12	+	NM_1728	P8198	protein_cc	6430703N	synaptota	Syt16
4463	1700086L	1.000	1.000	1.000	1.000	1.000	1.000	0.000	0.000	0.000	74284	1700086L	chr12	+	NR_03073	.	lncRNA	1700086L	RIKEN cDN	1700086L
4464	Dbpht2	1.000	1.000	1.000	1.000	1.000	1.000	0.000	0.000	0.000	386753	Dbpht2	chr12	+	NM_1988	P6935	protein_cc	Spot2	DNA bindi	Dbpht2
4465	Kcnh5	1.000	1.000	1.000	1.000	1.000	1.000	0.000	0.000	0.000	238271	Kcnh5	chr12	-	NM_1728	P6906	protein_cc	Eag2	potassium	Kcnh5
4466	Rhoj	4.648	1.332	6.189	0.000	0.035	0.023	6.019	8.236	8.649	80837	Rhoj	chr12	+	NM_0232	P4909	protein_cc	1110005O	ras homol	Rhoj
4467	Gphb5	1.000	1.000	1.000	1.000	1.000	1.000	0.000	0.000	0.000	217674	Gphb5	chr12	-	NM_1756	P12173	protein_cc	OGH | Zlut	glycoprote	Gphb5
4468	Ppp2r5e	0.458	1.220	0.559	0.000	0.231	0.053	9.522	8.397	8.683	26932	Ppp2r5e	chr12	-	NM_0120	P16734	protein_cc	4633401M	protein ph	Ppp2r5e
4469	Wdr89	1.125	1.357	1.527	0.327	0.052	0.139	12.425	12.595	13.036	72338	Wdr89	chr12	-	NM_0282	P5498	protein_cc	2600001A	WD repea	Wdr89
4470	Rplp2-ps1	1.126	1.358	1.529	0.322	0.051	0.138	12.422	12.593	13.035	665931	Rplp2-ps1	chr12	-	NR_03806	.	misc_RNA	665931 | G	ribosomal	Rplp2-ps1
4471	Sgpp1	0.478	1.473	0.703	0.001	0.017	0.046	9.319	8.253	8.812	81535	Sgpp1	chr12	-	NM_0307	P8700	protein_cc	AI463453 |	sphingosin	Sgpp1
4472	Syne2	0.351	1.141	0.401	0.000	0.615	0.054	7.507	5.997	6.187	319565	Syne2	chr12	+	NM_0010	P12631	protein_cc	6820443O	spectrin re	Syne2
4473	Esr2	1.000	1.000	1.000	1.000	1.000	1.000	0.000	0.000	0.000	13983	Esr2	chr12	-	NM_0101	P2709	protein_cc	ERbeta | E	estrogen r	Esr2
4474	Tex21	1.000	1.000	1.000	1.000	1.000	1.000	0.000	0.000	0.000	80384	Tex21	chr12	-	NM_0197	P6498	protein_cc	4931406F	testis expr	Tex21
4475	Mthfd1	0.162	0.581	0.094	0.000	0.004	0.007	10.452	7.822	7.038	108156	Mthfd1	chr12	+	NM_1387	P10859	protein_cc	Dcs | E430	methylene	Mthfd1
4476	Akap5	0.926	3.186	2.950	0.545	0.023	0.173	5.935	5.824	7.496	238276	Akap5	chr12	+	NM_0011	P8618	protein_cc	3526401B	A kinase (	Akap5
4477	Zbtb25	1.238	1.144	1.416	0.068	0.355	0.286	4.444	4.752	4.946	109929	Zbtb25	chr12	-	NM_0283	P21470	protein_cc	2810462N	zinc finger	Zbtb25
4478	Zbtb1	1.277	0.886	1.131	0.446	0.712	0.677	5.106	5.459	5.283	268564	Zbtb1	chr12	+	NM_1787	P23779	protein_cc	9430077A	zinc finger	Zbtb1
4479	Hspa2	6.469	0.577	3.731	0.031	0.183	0.055	4.730	7.424	6.630	15512	Hspa2	chr12	+	NM_0010	P14703	protein_cc	70kDa | Hs	heat shoc	Hspa2
4480	Ppp1r36	2.170	0.461	1.000	0.000	1.000	0.423	0.000	1.118	0.000	210762	Ppp1r36	chr12	+	NM_0011	P20813	protein_cc	Gm70	protein ph	Ppp1r36
4481	Gm10451	1.000	1.000	1.000	1.000	1.000	1.000	0.000	0.000	0.000	10004169	Gm10451	chr12	+	NR_02852	.	lncRNA	ENSMUSG	predicted	Gm10451
4482	Plekhg3	0.303	1.159	0.352	0.000	0.318	0.014	8.315	6.594	6.807	263406	Plekhg3	chr12	+	NM_1538	P21870	protein_cc	BC030417	pleckstrin	Plekhg3
4483	AI463170	1.125	2.632	2.960	0.538	0.017	0.110	4.268	4.437	5.833	10050454	AI463170	chr12	-	NR_04604	.	lncRNA	.	expressed	AI463170
4484	Sptb	0.308	1.383	0.426	0.003	0.537	0.146	2.418	0.721	1.189	20741	Sptb	chr12	-	NM_0136	P4799	protein_cc	AI842465 |	spectrin b	Sptb
4485	Churc1	1.278	1.099	1.404	0.487	0.755	0.268	6.498	6.852	6.988	211151	Churc1	chr12	+	NM_2065	P5034	protein_cc	1700030P	churchill d	Churc1
4486	Gpx2	1.046	6.104	6.382	0.000	0.181	0.423	0.001	0.065	2.675	14776	Gpx2	chr12	-	NM_0306	P8950	protein_cc	GI-GPx | G	glutathion	Gpx2
4487	Rab15	0.750	1.615	1.211	0.569	0.420	0.840	1.112	0.697	1.388	104886	Rab15	chr12	-	NM_1340	P1131	protein_cc	2310012G	RAB15, m	Rab15
4488	Fntb	1.696	0.679	1.151	0.066	0.141	0.545	5.106	5.868	5.309	110606	Fntb	chr12	+	NM_1459	P19482	protein_cc	2010013E	farnesyltra	Fntb
4489	Max	3.179	0.463	1.471	0.001	0.003	0.009	8.040	9.708	8.596	17187	Max	chr12	-	NM_0011	P3375	protein_cc	AA960152	Max prote	Max
4490	Fut8	1.319	0.628	0.829	0.274	0.139	0.630	4.837	5.236	4.566	53618	Fut8	chr12	+	NM_0168	P11220	protein_cc	.	fucosyltra	Fut8
4491	Gphn	0.210	1.117	0.235	0.000	0.738	0.020	9.173	6.921	7.080	268566	Gphn	chr12	+	NM_1729	P11304	protein_cc	5730552E	gephyrin	Gphn
4492	Fam71d	1.000	1.000	1.000	1.000	1.000	1.000	0.000	0.000	0.000	70897	Fam71d	chr12	+	NM_0275	P4900	protein_cc	4921509E	family wit	Fam71d
4493	Mpp5	1.631	0.805	1.312	0.000	0.003	0.070	8.014	8.720	8.406	56217	Mpp5	chr12	+	NM_0195	P15023	protein_cc	3830420B	membran	Mpp5
4494	Atp6v1d	1.888	0.794	1.499	0.000	0.000	0.032	9.190	10.106	9.773	73834	Atp6v1d	chr12	-	NM_0237	P18893	protein_cc	1110004P	ATPase, H	Atp6v1d
4495	Eif2s1	2.251	0.990	2.229	0.003	0.923	0.011	8.383	9.554	9.540	13665	Eif2s1	chr12	+	NM_0261	P16262	protein_cc	0910001O	eukaryoti	Eif2s1
4496	Plek2	0.275	0.953	0.262	0.008	0.930	0.016	4.930	3.069	2.999	27260	Plek2	chr12	-	NM_0137	P20745	protein_cc	.	pleckstrin	Plek2
4497	Tmem229	1.661	0.597	0.991	0.155	0.126	0.988	3.767	4.500	3.755	268567	Tmem229	chr12	-	NM_1787	P15967	protein_cc	6330442E	transmem	Tmem229
4498	Plekhh1	1.537	0.422	0.649	0.082	0.000	0.452	6.171	6.791	5.546	211945	Plekhh1	chr12	+	NM_1810	P1016	protein_cc	AV308913	pleckstrin	Plekhh1
4499	Pigh	1.497	0.426	0.638	0.111	0.001	0.445	6.208	6.790	5.559	110417	Pigh	chr12	-	NM_0299	P6429	protein_cc	2210416H	phosphati	Pigh
4500	9230116L	1.000	1.000	1.000	1.000	1.000	1.000	0.000	0.000	0.000	321017	9230116L	chr12	-	NR_11048	.	lncRNA		RIKEN cDN	9230116L
4501	Arg2	1.122	1.372	1.540	0.253	0.003	0.060	8.940	9.106	9.563	11847	Arg2	chr12	+	NM_0097	P3079	protein_cc	AII | AU02	arginase t	Arg2
4502	Vti1b	1.003	1.357	1.361	0.965	0.010	0.098	9.444	9.449	9.889	53612	Vti1b	chr12	-	NM_0168	P3540	protein_cc	AU015348	vesicle tra	Vti1b
4503	Rdh11	0.862	1.052	0.907	0.472	0.162	0.772	5.907	5.694	5.767	17252	Rdh11	chr12	-	NM_0215	P23211	protein_cc	2610319N	retinol de	Rdh11
4504	Rdh12	7.970	0.825	6.577	0.000	0.303	0.105	1.005	4.000	3.722	77974	Rdh12	chr12	+	NM_0300	P17887	protein_cc	.	retinol de	Rdh12
4505	Zfyve26	0.580	1.050	0.609	0.023	0.511	0.193	6.900	6.114	6.184	211978	Zfyve26	chr12	-	NM_0010	P10838	protein_cc	4930465A	zinc finger	Zfyve26
4506	Rad51b	0.415	0.688	0.285	0.000	0.425	0.101	6.490	5.221	4.681	19363	Rad51b	chr12	+	NM_0090	P23518	protein_cc	AI553500 |	RAD51 pa	Rad51b
4507	9430078K	1.000	1.000	1.000	1.000	1.000	1.000	0.000	0.000	0.000	77290	9430078K	chr12	-	NR_13107	.	lncRNA	.	RIKEN cDN	9430078K
4508	Zfp36l1	1.161	0.364	0.422	0.014	0.000	0.013	11.305	11.520	10.061	12192	Zfp36l1	chr12	-	NM_0075	P6806	protein_cc	AW74243	zinc finger	Zfp36l1
4509	2310015A	5.156	0.517	2.668	0.000	0.007	0.135	3.887	6.253	5.302	69548	2310015A	chr12	-	NR_03351	.	lncRNA	5330419I1	RIKEN cDN	2310015A
4510	Actn1	1.424	0.784	1.117	0.004	0.002	0.364	9.302	9.812	9.461	109711	Actn1	chr12	-	NM_1341	P20991	protein_cc	3110023F	actinin, al	Actn1
4511	Dcaf5	1.362	0.860	1.172	0.233	0.526	0.469	8.275	8.721	8.504	320808	Dcaf5	chr12	-	NM_1772	P3490	protein_cc	9430020B	DDB1 and	Dcaf5
4512	Scarna3b	1.000	1.000	1.000	1.000	1.000	1.000	0.000	0.000	0.000	10021744	Scarna3b	chr12	-	NR_02854	.	guide_RN	MBI-100	small Caja	Scarna3b
4513	Exd2	1.154	0.663	0.765	0.645	0.212	0.634	4.096	4.302	3.709	97827	Exd2	chr12	+	NM_1337	P871	protein_cc	4930539P	exonuclea	Exd2
4514	2310002D	1.000	1.000	1.000	1.000	1.000	1.000	0.000	0.000	0.000	69522	2310002D	chr12	-	NR_04549	.	lncRNA		RIKEN cDN	2310002D
4515	Galnt16	1.000	1.000	1.000	1.000	1.000	1.000	0.000	0.000	0.000	108760	Galnt16	chr12	+	NM_0010	P11434	protein_cc	5730405L	UDP-N-ac	Galnt16
4516	Erh	1.849	1.310	2.423	0.001	0.057	0.047	7.361	8.247	8.637	13877	Erh	chr12	-	NM_0079	P20604	protein_cc	Mer | Pre	enhancer	Erh
4517	Slc39a9	0.992	0.811	0.805	0.828	0.171	0.415	9.000	8.989	8.687	328133	Slc39a9	chr12	+	NM_0262	P10345	protein_cc	2010002A	solute car	Slc39a9
4518	Plekhd1os	1.000	1.000	1.000	1.000	1.000	1.000	0.000	0.000	0.000	68354	Plekhd1os	chr12	-	NR_03799	.	lncRNA	0610009B	RIKEN cDN	Plekhd1os
4519	Plekhd1	11.350	1.659	18.826	0.000	0.389	0.295	1.591	5.096	5.826	217682	Plekhd1	chr12	+	NM_0011	P20085	protein_cc	3830431G	pleckstrin	Plekhd1
4520	Ccdc177	8.679	0.475	4.124	0.034	0.134	0.004	0.002	3.119	2.046	380768	Ccdc177	chr12	-	NM_0010	P11952	protein_cc	Gm1568	coiled-coil	Ccdc177
4521	Susd6	5.011	0.398	1.994	0.013	0.033	0.030	7.816	10.141	8.811	217684	Susd6	chr12	+	NM_1786	P22686	protein_cc	4933426M	sushi dom	Susd6
4522	1700052I2	1.000	1.000	1.000	1.000	1.000	1.000	0.000	0.000	0.000	67340	1700052I2	chr12	-	NR_03378	.	lncRNA	.	RIKEN cDN	1700052I2
4523	Gm20337	0.118	1.547	0.183	0.006	0.115	0.094	4.311	1.228	1.857	10050466	Gm20337	chr12	-	NR_04505	.	lncRNA		predicted	Gm20337
4524	Srsf5	0.908	0.963	0.875	0.337	0.855	0.671	10.093	9.953	9.899	20384	Srsf5	chr12	+	NM_0091	P1094	protein_cc	Sfrs5	serine/arg	Srsf5
4525	Slc10a1	0.059	0.972	0.057	0.000	0.875	0.006	12.298	8.206	8.166	20493	Slc10a1	chr12	-	NM_0011	P21321	protein_cc	Ntcp	solute car	Slc10a1
4526	Smoc1	0.891	0.941	0.838	0.037	0.193	0.019	9.457	9.290	9.202	64075	Smoc1	chr12	+	NM_0011	P23218	protein_cc	2600002F	SPARC rel	Smoc1
4527	Slc8a3	0.544	1.781	0.969	0.000	0.186	0.950	0.906	0.027	0.860	110893	Slc8a3	chr12	-	NM_0011	P2796	protein_cc	AW74226	solute car	Slc8a3
4528	Cox16	0.948	1.338	1.268	0.135	0.000	0.006	9.862	9.786	10.205	66272	Cox16	chr12	-	NM_0013	P20337	protein_cc	1810020G	cytochron	Cox16
4529	Synj2bp-c	0.337	2.149	0.725	0.000	0.013	0.189	8.513	6.946	8.049	10594040	Synj2bp-c	chr12	-	NM_0013	P19852	protein_cc	ARIP2a	Synj2bp-c	Synj2bp-c
4530	Gm4787	0.552	0.990	0.547	0.182	1.000	0.423	0.871	0.015	0.000	214321	Gm4787	chr12	-	NM_0010	P25048	protein_cc	Adam4b |	predicted	Gm4787
4531	Adam4	0.913	1.374	1.255	0.834	0.538	0.783	0.817	0.687	1.145	11498	Adam4	chr12	-	NM_0096	P26299	protein_cc	AV274654	a disinteg	Adam4
4532	Synj2bp	0.814	0.896	0.729	0.005	0.404	0.208	9.884	9.587	9.428	24071	Synj2bp	chr12	-	NM_0252	P6215	protein_cc	AA409442	synaptoja	Synj2bp
4533	Adam21	0.552	0.990	0.547	0.182	1.000	0.423	0.871	0.015	0.000	56622	Adam21	chr12	-	NM_0203	P1349	protein_cc	Adam31	a disinteg	Adam21
4534	Med6	2.045	0.891	1.821	0.001	0.577	0.284	6.303	7.334	7.168	69792	Med6	chr12	-	NM_0272	P17233	protein_cc	1500012F	mediator	Med6
4535	Ttc9	0.979	3.885	3.803	0.961	0.012	0.107	0.768	0.737	2.695	69480	Ttc9	chr12	+	NM_0010	P10053	protein_cc	.	tetratricop	Ttc9
4536	Map3k9	0.728	0.533	0.388	0.419	0.034	0.363	2.234	1.776	0.867	338372	Map3k9	chr12	-	NM_0011	P995	protein_cc	E130314H	mitogen-a	Map3k9
4537	Pcnx	0.319	0.902	0.288	0.014	0.636	0.117	7.549	5.901	5.753	54604	Pcnx	chr12	+	NM_0188	P12675	protein_cc	2900024E	pecanex h	Pcnx

4538	Sipa1l1	0.579	0.898	0.520	0.000	0.003	0.009	7.644	6.855	6.699	217692	Sipa1l1	chr12	+	NM_0011	P19667	protein_cc	4931426N	signal-indu	Sipa1l1
4539	Rgs6	1.000	1.000	1.000	1.000	1.000	1.000	0.000	0.000	0.000	50779	Rgs6	chr12	+	NM_0158	P11986	protein_cc	.	regulator	Rgs6
4540	1700085C	1.000	1.000	1.000	1.000	1.000	1.000	0.000	0.000	0.000	74283	1700085C	chr12	-	NR_04604	.	lncRNA		RIKEN cDN	1700085C2
4541	Dpf3	0.796	0.601	0.479	0.458	0.208	0.286	3.973	3.644	2.909	70127	Dpf3	chr12	-	NM_0012	P3036	protein_cc	2810403B	D4, zinc ar	Dpf3
4542	Dcaf4	0.820	2.485	2.038	0.444	0.042	0.226	5.133	4.847	6.160	73828	Dcaf4	chr12	+	NM_0302	P25657	protein_cc	1110018E	DDB1 and	Dcaf4
4543	Zfyve1	0.742	0.694	0.515	0.041	0.099	0.078	5.762	5.333	4.806	217695	Zfyve1	chr12	-	NM_1831	P13902	protein_cc	DFCP1| Gi	zinc finger	Zfyve1
4544	Rbm25	1.257	0.860	1.081	0.028	0.092	0.273	10.443	10.774	10.556	67039	Rbm25	chr12	+	NM_0273	P16307	protein_cc	2600011C	RNA bindi	Rbm25
4545	Psen1	2.768	0.766	2.122	0.000	0.001	0.018	7.139	8.608	8.224	19164	Psen1	chr12	+	NM_0089	P2892	protein_cc	Ad3h| PS-	presenilin	Psen1
4546	Papln	0.612	0.361	0.221	0.311	1.000	0.019	2.179	1.470	0.000	170721	Papln	chr12	+	NM_0012	P17743	protein_cc	E030033C	papilin, pr	Papln
4547	Numb	0.798	1.434	1.144	0.441	0.292	0.719	6.285	5.960	6.480	18222	Numb	chr12	-	NM_0012	P3296	protein_cc	Nb	numb hon	Numb
4548	2410016O	1.369	1.004	1.374	0.012	0.980	0.361	6.778	7.231	7.236	71952	2410016O	chr12	+	NM_0236	P16869	protein_cc	MAPJD| N	RIKEN cDN	2410016O
4549	Acot2	0.111	7.296	0.809	0.000	0.000	0.292	7.733	4.559	7.426	171210	Acot2	chr12	+	NM_1341	P13453	protein_cc	AA571646	acyl-CoA t	Acot2
4550	Acot1	0.068	1.351	0.091	0.000	0.334	0.002	8.973	5.084	5.518	26897	Acot1	chr12	+	NM_0120	P14534	protein_cc	ACH2| CTI	acyl-CoA t	Acot1
4551	Acot4	0.256	1.791	0.458	0.006	0.000	0.146	7.118	5.152	5.993	171282	Acot4	chr12	+	NM_1342	P5746	protein_cc	B430212I	acyl-CoA t	Acot4
4552	Acot3	0.552	1.087	0.600	0.020	0.864	0.483	7.916	7.059	7.180	171281	Acot3	chr12	+	NM_1342	P16219	protein_cc	AW10839	acyl-CoA t	Acot3
4553	Acot5	1.000	1.000	1.000	1.000	1.000	1.000	0.000	0.000	0.000	217698	Acot5	chr12	+	NM_1454	P7590	protein_cc	BC016076	acyl-CoA t	Acot5
4554	Acot6	0.520	3.844	2.000	0.099	0.002	0.096	1.677	0.734	2.677	217700	Acot6	chr12	+	NM_1725	P21912	protein_cc	4632408A	acyl-CoA t	Acot6
4555	Dnal1	1.599	1.113	1.779	0.278	0.728	0.076	2.141	2.818	2.972	105000	Dnal1	chr12	+	NM_0288	P3501	protein_cc	1700010H	dynein, ax	Dnal1
4556	Pnma1	1.238	0.456	0.565	0.421	1.000	0.422	0.824	1.132	0.000	70481	Pnma1	chr12	-	NM_0274	P18225	protein_cc	5730402C	paraneopl	Pnma1
4557	Elmsan1	1.797	0.941	1.692	0.063	0.675	0.234	4.774	5.620	5.533	238317	Elmsan1	chr12	-	NM_0011	P26902	protein_cc	9430029N	ELM2 and	Elmsan1
4558	Ptgr2	0.373	0.993	0.370	0.002	0.969	0.068	9.667	8.243	8.233	77219	Ptgr2	chr12	+	NM_0012	P20732	protein_cc	1810016I2	prostaglar	Ptgr2
4559	Zfp410	1.375	1.125	1.547	0.185	0.496	0.091	6.296	6.756	6.925	52708	Zfp410	chr12	+	NR_04554	.	protein_cc	Apa1| D12	zinc finger	Zfp410
4560	Fam161b	3.028	0.603	1.827	0.080	0.249	0.270	1.196	2.795	2.066	217705	Fam161b	chr12	-	NM_1725	P2931	protein_cc	9330169D	family wit	Fam161b
4561	Coq6	0.778	1.230	0.957	0.079	0.147	0.762	6.257	5.895	6.194	217707	Coq6	chr12	+	NM_1725	P21722	protein_cc	5930427M	coenzyme	Coq6
4562	Entpd5	0.142	1.719	0.243	0.000	0.014	0.012	10.119	7.300	8.081	12499	Entpd5	chr12	-	NM_0076	P21840	protein_cc	AI196558|	ectonucle	Entpd5
4563	Ccdc176	0.623	2.279	1.419	0.368	0.089	0.146	4.098	3.414	4.603	.	Ccdc176	chr12	+	NM_0283	P11372	.	.		Ccdc176
4564	Rnf113a2	2.122	0.656	1.392	0.005	0.032	0.164	6.296	7.381	6.773	66381	Rnf113a2	chr12	+	NM_0255	P7447	protein_cc	2310020H	ring finger	Rnf113a2
4565	Aldh6a1	0.282	1.039	0.293	0.000	0.671	0.019	11.125	9.297	9.352	104776	Aldh6a1	chr12	-	NM_1340	P14901	protein_cc	1110038I	aldehyde	Aldh6a1
4566	Lin52	1.091	0.919	1.003	0.833	0.535	0.997	4.889	5.015	4.893	217708	Lin52	chr12	+	NM_1737	P11003	protein_cc	5830457H	lin-52 hom	Lin52
4567	Vsx2	1.000	1.000	1.000	1.000	1.000	1.000	0.000	0.000	0.000	12677	Vsx2	chr12	+	NM_0013	P6634	protein_cc	Chx10| Hc	visual syst	Vsx2
4568	Abcd4	0.930	1.703	1.584	0.872	0.067	0.371	4.620	4.515	5.284	19300	Abcd4	chr12	-	NM_0089	P4658	protein_cc	P69r| P70	ATP-bindi	Abcd4
4569	Vrtn	1.000	1.000	1.000	1.000	1.000	1.000	0.000	0.000	0.000	432677	Vrtn	chr12	+	NM_0011	P14141	protein_cc	7420416P	vertebrae	Vrtn
4570	Syndig1l	1.000	1.000	1.000	1.000	1.000	1.000	0.000	0.000	0.000	627191	Syndig1l	chr12	-	NM_0010	P14840	protein_cc	DSPC1| Gi	synapse d	Syndig1l
4571	Npc2	1.018	1.049	1.067	0.525	0.219	0.285	10.648	10.673	10.742	67963	Npc2	chr12	-	NM_0234	P4845	protein_cc	2700012J1	Niemann-	Npc2
4572	Isca2	0.815	1.266	1.032	0.271	0.021	0.898	8.536	8.241	8.581	74316	Isca2	chr12	+	NM_0288	P2360	protein_cc	0710001C	iron-sulfu	Isca2
4573	Ltbp2	0.402	0.826	0.332	0.325	0.749	0.507	2.464	1.149	0.873	16997	Ltbp2	chr12	-	NM_0135	P10231	protein_cc	AW20864	latent trar	Ltbp2
4574	D030025P	1.000	1.000	1.000	1.000	1.000	1.000	0.000	0.000	0.000	10030373	D030025P	chr12	+	NR_02857	.	lncRNA		RIKEN cDN	D030025P
4575	Arel1	1.438	0.902	1.297	0.001	0.015	0.093	7.450	7.974	7.825	68497	Arel1	chr12	-	NM_1780	P13064	protein_cc	1110018G	apoptosis	Arel1
4576	Fcf1	1.718	1.128	1.938	0.038	0.446	0.048	8.975	9.756	9.929	73736	Fcf1	chr12	+	NM_0286	P4564	protein_cc	1110008B	FCF1 rRNA	Fcf1
4577	Ylpm1	1.040	0.701	0.729	0.730	0.066	0.115	8.083	8.140	7.626	56531	Ylpm1	chr12	+	NM_1783	P17405	protein_cc	A930013E	YLP motif	Ylpm1
4578	Prox2	0.548	1.597	0.876	0.363	0.129	0.869	1.572	0.705	1.381	73422	Prox2	chr12	-	NM_1751	P21964	protein_cc	1700058C	prospero I	Prox2
4579	Dlst	0.617	1.268	0.783	0.004	0.026	0.152	9.989	9.294	9.637	78920	Dlst	chr12	+	NM_0302	P23101	protein_cc	1600017E	dihydrolip	Dlst
4580	Rps6kl1	0.433	0.870	0.377	0.010	0.714	0.067	6.639	5.433	5.232	238323	Rps6kl1	chr12	-	NM_1462	P20162	protein_cc	A830084F	ribosomal	Rps6kl1
4581	Pgf	4.074	3.402	13.858	0.300	0.045	0.069	0.002	2.028	3.794	18654	Pgf	chr12	-	NM_0088	P14562	protein_cc	AI854365|	placental	Pgf
4582	Eif2b2	2.212	1.247	2.759	0.019	0.281	0.072	7.696	8.842	9.160	217715	Eif2b2	chr12	+	NM_1454	P2574	protein_cc	EIF-2Bbeta	eukaryotic	Eif2b2
4583	Mlh3	0.965	0.821	0.793	0.857	0.264	0.549	6.745	6.693	6.410	217716	Mlh3	chr12	-	NM_1753	P21839	protein_cc	AV125803	mutL hom	Mlh3
4584	Acyp1	0.429	1.376	0.590	0.027	0.292	0.362	6.640	5.418	5.878	66204	Acyp1	chr12	-	NM_0254	P9881	protein_cc	1110039O	acylphosp	Acyp1
4585	Zc2hc1c	0.569	1.528	0.870	0.015	0.023	0.545	4.393	3.580	4.192	72350	Zc2hc1c	chr12	+	NM_1724	P5505	protein_cc	2810002I	zinc finger	Zc2hc1c
4586	Nek9	1.179	1.351	1.594	0.010	0.077	0.174	8.089	8.327	8.761	217718	Nek9	chr12	-	NM_1451	P7241	protein_cc	C130021H	NIMA (ne	Nek9
4587	Tmed10	1.069	1.083	1.158	0.021	0.073	0.144	10.203	10.299	10.414	68581	Tmed10	chr12	-	NM_0267	P19097	protein_cc	1110014C	transmem	Tmed10
4588	Fos	2.005	1.049	2.102	0.021	0.736	0.013	4.068	5.071	5.140	14281	Fos	chr12	+	NM_0102	P18470	protein_cc	c-fos| cFo	FBJ osteos	Fos
4589	Jdp2	4.798	0.635	3.049	0.001	0.027	0.047	4.449	6.712	6.057	81703	Jdp2	chr12	+	NM_0012	P17052	protein_cc	Jundm2| J	Jun dimer	Jdp2
4590	Batf	10.878	0.291	3.164	0.000	0.000	0.129	2.919	6.362	4.580	53314	Batf	chr12	+	NM_0167	P8806	protein_cc	B-ATF| SF	basic leuci	Batf
4591	Mfsd7c	0.093	1.310	0.119	0.000	0.565	0.004	6.437	2.975	3.364	217721	Mfsd7c	chr12	+	NM_1454	P9083	protein_cc	CCT| Flvcr	major faci	Mfsd7c
4592	0610007P	0.462	2.343	1.082	0.002	0.000	0.538	10.543	9.429	10.657	58520	0610007P	chr12	-	NM_0214	P22293	protein_cc	1190004E	RIKEN cDN	0610007P1
4593	Ttll5	0.491	0.747	0.367	0.000	0.142	0.030	6.424	5.396	4.976	320244	Ttll5	chr12	+	NM_0010	P23203	protein_cc	1700048H	tubulin ty	Ttll5
4594	Tgfb3	0.755	1.331	1.005	0.312	0.304	0.783	2.799	2.393	2.806	21809	Tgfb3	chr12	-	NM_0093	P23841	protein_cc	TGF-beta-	transform	Tgfb3
4595	Ift43	2.049	0.962	1.972	0.022	0.807	0.016	5.715	6.750	6.694	76411	Ift43	chr12	+	NM_0011	P883	protein_cc	1700019E	intraflagel	Ift43
4596	Gm805	1.000	1.000	1.000	1.000	1.000	1.000	0.000	0.000	0.000	328136	Gm805	chr12	+	NR_04608	.	lncRNA		predicted	Gm805
4597	Gpatch2l	2.361	0.848	2.002	0.090	0.579	0.193	4.472	5.711	5.474	70373	Gpatch2l	chr12	+	NM_0274	P11150	protein_cc	1700020O	G patch do	Gpatch2l
4598	Esrrb	0.472	0.329	0.155	0.244	0.348	0.173	3.074	1.992	0.387	26380	Esrrb	chr12	+	NM_0011	P11702	protein_cc	Err2| Errb	estrogen r	Esrrb
4599	Vash1	0.813	0.813	0.661	0.681	0.343	0.659	3.867	3.568	3.270	238328	Vash1	chr12	+	NM_1773	P18457	protein_cc	AI834978|	vasohibin	Vash1
4600	Angel1	0.639	0.865	0.553	0.367	0.035	0.523	4.065	3.420	3.211	68737	Angel1	chr12	-	NM_1445	P6659	protein_cc	1110030H	angel hom	Angel1
4601	Lrrc74a	0.716	0.995	0.712	0.182	1.000	0.423	0.490	0.008	0.000	627607	Lrrc74a	chr12	+	NM_0011	P9452	protein_cc	EG627607	leucine ric	Lrrc74a
4602	Irf2bpl	0.737	0.820	0.604	0.009	0.087	0.006	8.883	8.442	8.156	238330	Irf2bpl	chr12	-	NM_1458	P6370	protein_cc	6430527G	interferon	Irf2bpl
4603	Cipc	2.200	0.652	1.436	0.007	0.043	0.228	6.985	8.123	7.507	217732	Cipc	chr12	+	NM_0012	P9332	protein_cc	2310044G	CLOCK int	Cipc
4604	Zdhhc22	1.000	1.000	1.000	1.000	1.000	1.000	0.000	0.000	0.000	238331	Zdhhc22	chr12	-	NM_0010	P10484	protein_cc	Gm262	zinc finger	Zdhhc22
4605	Tmem63c	1.000	1.000	1.000	1.000	1.000	1.000	0.000	0.000	0.000	217733	Tmem63c	chr12	+	NM_1725	P4745	protein_cc	4932420N	transmem	Tmem63c
4606	Ngb	0.750	2.739	2.055	0.200	0.182	0.516	0.448	0.033	1.487	64242	Ngb	chr12	-	NM_0012	P20984	protein_cc	.	neuroglob	Ngb
4607	Pomt2	1.106	1.042	1.153	0.511	0.862	0.755	5.865	6.010	6.070	217734	Pomt2	chr12	-	NM_1534	P4210	protein_cc	.	protein-O-	Pomt2
4608	Gstz1	0.284	1.332	0.378	0.000	0.010	0.003	12.530	10.711	11.125	14874	Gstz1	chr12	+	NM_0012	P17977	protein_cc	MAAI	glutathion	Gstz1
4609	Tmed8	1.027	1.212	1.244	0.942	0.356	0.699	5.010	5.048	5.325	382620	Tmed8	chr12	-	NM_0010	P9992	protein_cc	6430595O	transmem	Tmed8
4610	Samd15	2.191	0.853	1.870	0.000	0.579	0.423	0.001	1.132	0.904	238333	Samd15	chr12	+	NM_0012	P9943	protein_cc	Gm263	sterile alp	Samd15
4611	Noxred1	2.722	0.367	1.000	0.306	1.000	0.423	0.001	1.445	0.000	71275	Noxred1	chr12	-	NM_0277	P9951	protein_cc	4933437F	NADP+ de	Noxred1
4612	Vipas39	1.384	0.874	1.209	0.453	0.717	0.512	5.804	6.273	6.078	104799	Vipas39	chr12	-	NM_1340	P26285	protein_cc	6720456H	VPS33B in	Vipas39
4613	Ahsa1	1.336	1.068	1.426	0.050	0.373	0.085	9.566	9.983	10.078	217737	Ahsa1	chr12	+	NM_1460	P24002	protein_cc	BC023857	AHA1, act	Ahsa1
4614	Ism2	0.716	0.995	0.712	0.182	1.000	0.423	0.490	0.008	0.000	217738	Ism2	chr12	-	NM_0012	P17329	protein_cc	Gm264| T	isthmin 2	Ism2
4615	Sptlc2	4.737	0.902	4.271	0.000	0.061	0.007	6.652	8.896	8.747	20773	Sptlc2	chr12	-	NM_0114	P9235	protein_cc	LCB2| LCB	serine pal	Sptlc2
4616	Alkbh1	2.430	0.960	2.331	0.059	0.906	0.294	4.639	5.920	5.860	211064	Alkbh1	chr12	-	NM_0011	P1690	protein_cc	2700073G	alkB homo	Alkbh1
4617	Nrp	1.773	0.707	1.253	0.099	0.236	0.437	4.409	5.235	4.734	654309	Nrp	chr12	-	NM_0010	P2571	protein_cc	.	neural reg	Nrp
4618	Slirp	1.748	0.927	1.620	0.012	0.496	0.006	8.968	9.774	9.664	380773	Slirp	chr12	+	NM_0269	P3794	protein_cc	1810035L	SRA stem-	Slirp
4619	Snw1	1.817	0.936	1.701	0.000	0.222	0.031	9.253	10.115	10.019	66354	Snw1	chr12	-	NM_0255	P22115	protein_cc	2310008B	SNW dom	Snw1
4620	Gm8300	1.005	1.405	1.412	0.000	0.182	0.423	0.000	0.008	0.498	666806	Gm8300	chr12	+	NM_0011	P25849	protein_cc	EG666806	predicted	Gm8300
4621	Gm2042	1.000	1.000	1.000	1.000	1.000	1.000	0.000	0.000	0.000	10003908	Gm2042	chr12	+	NM_0012	P12009	protein_cc	10003908	predicted	Gm2042
4622	Oog1	1.000	1.000	1.000	1.000	1.000	1.000	0.000	0.000	0.000	193322	Oog1	chr12	+	NM_1786	P12009	protein_cc	c-1| Oog	oogenesir	Oog1
4623	BB287469	1.581	0.879	1.390	0.301	0.744	0.423	0.000	0.661	0.476	544881	BB287469	chr12	+	NM_0011	P22946	protein_cc	.	expressed	BB287469
4624	Gm4027	1.428	0.466	0.666	0.562	1.000	0.423	0.587	1.101	0.000	10004277	Gm4027	chr12	+	NM_0011	P22946	protein_cc	10004277	predicted	Gm4027
4625	Gm16381	0.503	0.976	0.491	0.001	0.820	0.038	5.060	4.069	4.033	10004278	Gm16381	chr12	-	NM_0011	P7980	protein_cc	EG435336	predicted	Gm16381
4626	Gm21319	1.000	1.000	1.000	1.000	1.000	1.000	0.000	0.000	0.000	10086190	Gm21319	chr12	+	NM_0012	P2923	protein_cc	.	predicted	Gm21319
4627	Gm1995	1.146	0.632	0.725	0.729	1.000	0.422	0.465	0.661	0.000	10003898	Gm1995	chr12	+	NR_03356	.	misc_RNA	10003898	predicted	Gm1995
4628	Gm2016	2.754	0.494	1.360	0.020	0.054	0.423	0.001	1.462	0.445	10003904	Gm2016	chr12	+	NM_0011	P15402	protein_cc	10003904	predicted	Gm2016

4629	Gm2022	1.590	0.658	1.046	0.468	0.505	0.919	0.438	1.107	0.504	10003905	Gm2022	chr12	+	NM_0011	P20989	protein_cc	10003905	predicted	Gm2022
4630	Gm10436	1.000	1.000	1.000	1.000	1.000	1.000	0.000	0.000	0.000	10003931	Gm10436	chr12	-	NM_0011	P14008	protein_cc	ENSMUSG	predicted	Gm10436
4631	Gm5662	2.181	0.652	1.422	0.000	0.042	0.423	0.000	1.126	0.508	435337	Gm5662	chr12	-	NM_0010	P17748	protein_cc	EG435337	predicted	Gm5662
4632	Gm7104	0.451	0.986	0.444	0.182	1.000	0.423	1.170	0.021	0.000	633093	Gm7104	chr12	-	NR_03357	.	misc_RNA	633093	predicted	Gm7104
4633	Gm5039	1.010	1.821	1.840	0.000	0.000	0.002	0.000	0.015	0.880	266459	Gm5039	chr12	-	NR_00364	.	misc_RNA	EG266459	predicted	Gm5039
4634	Adck1	0.810	0.713	0.578	0.020	0.025	0.089	6.913	6.609	6.120	72113	Adck1	chr12	+	NM_0012	P21348	protein_cc	2610005A	aarF doma	Adck1
4635	3200001D	0.885	0.629	0.557	0.781	1.000	0.422	0.844	0.668	0.000	71818	3200001D	chr12	-	NR_04588	.	lncRNA	.	RIKEN cDN	3200001D
4636	Nrxn3	1.385	0.619	0.858	0.065	0.020	0.684	4.280	4.749	4.058	18191	Nrxn3	chr12	+	NM_0011	P23237	protein_cc	.	neurexin I	Nrxn3
4637	Dio2	10.160	5.766	58.584	0.003	0.068	0.212	0.003	3.347	5.875	13371	Dio2	chr12	-	NM_0100	P4379	protein_cc	5DII| AI32	deiodinase	Dio2
4638	Cep128	0.860	0.682	0.586	0.812	0.431	0.718	3.036	2.819	2.266	75216	Cep128	chr12	-	NM_1818	P688	protein_cc	4930534B	centrosom	Cep128
4639	5430427M	1.000	1.000	1.000	1.000	1.000	1.000	0.000	0.000	0.000	71349	5430427M	chr12	+	NR_04585	.	lncRNA	.	RIKEN cDN	5430427M
4640	Tshr	1.000	1.000	1.000	1.000	1.000	1.000	0.000	0.000	0.000	22095	Tshr	chr12	+	NM_0116	P15554	protein_cc	AI481368|	thyroid sti	Tshr
4641	Gtf2a1	1.528	0.671	1.025	0.080	0.128	0.921	8.052	8.664	8.088	83602	Gtf2a1	chr12	-	NM_1753	P14582	protein_cc	6330549H	general tra	Gtf2a1
4642	Ston2	1.080	0.629	0.680	0.628	0.016	0.303	6.052	6.163	5.495	108800	Ston2	chr12	-	NM_1753	P23731	protein_cc	4933401N	stonin 2	Ston2
4643	Sel1l	0.820	0.749	0.614	0.004	0.004	0.025	10.806	10.520	10.103	20338	Sel1l	chr12	-	NM_0010	P6563	protein_cc	AW49376	sel-1 supp	Sel1l
4644	4930559C	1.000	1.000	1.000	1.000	1.000	1.000	0.000	0.000	0.000	75295	4930559C	chr12	-	NR_12643	.	lncRNA	.	RIKEN cDN	4930559C
4645	Flrt2	3.229	1.295	4.180	0.000	0.009	0.001	2.775	4.466	4.838	399558	Flrt2	chr12	+	NM_2015	P13526	protein_cc	.	fibronecti	Flrt2
4646	1700019M	1.000	1.000	1.000	1.000	1.000	1.000	0.000	0.000	0.000	69423	1700019M	chr12	-	NR_10380	.	misc_RNA	.	RIKEN cDN	1700019M
4647	Galc	1.377	0.768	1.057	0.283	0.208	0.895	4.758	5.219	4.838	14420	Galc	chr12	-	NM_0080	P20390	protein_cc	2310068B	galactosyl	Galc
4648	Gpr65	2.487	0.411	1.023	0.013	0.022	0.974	4.856	6.170	4.888	14744	Gpr65	chr12	+	NM_0081	P13739	protein_cc	Dig1| Gpc	G-protein	Gpr65
4649	Kcnk10	0.072	1.203	0.087	0.000	0.303	0.001	3.908	0.117	0.384	72258	Kcnk10	chr12	-	NM_0299	P11556	protein_cc	1700024D	potassium	Kcnk10
4650	Spata7	1.052	0.540	0.567	0.903	0.282	0.208	4.275	4.348	3.458	104871	Spata7	chr12	+	NM_1789	P23286	protein_cc	AI661438|	spermatog	Spata7
4651	Ptpn21	0.920	1.740	1.601	0.741	0.043	0.109	6.057	5.937	6.736	24000	Ptpn21	chr12	-	NM_0118	P14340	protein_cc	PTPD1| PT	protein ty	Ptpn21
4652	Zc3h14	0.715	1.009	0.722	0.112	0.913	0.322	7.819	7.335	7.348	75553	Zc3h14	chr12	+	NM_0011	P8545	protein_cc	1010001P	zinc finger	Zc3h14
4653	Eml5	0.753	1.024	0.771	0.118	0.824	0.288	7.749	7.340	7.375	319670	Eml5	chr12	-	NM_0010	P9599	protein_cc	BC027154	echinoder	Eml5
4654	Ttc8	0.584	1.685	0.985	0.057	0.232	0.973	4.358	3.583	4.336	76260	Ttc8	chr12	+	NM_0295	P6719	protein_cc	0610012F	tetratricop	Ttc8
4655	4930474N	1.005	1.439	1.446	0.000	0.182	0.423	0.000	0.008	0.532	67639	4930474N	chr12	-	NR_03813	.	lncRNA	.	RIKEN cDN	4930474N
4656	Foxn3	0.164	1.733	0.284	0.006	0.103	0.121	9.182	6.574	7.367	71375	Foxn3	chr12	-	NM_1831	P16534	protein_cc	5430426H	forkhead b	Foxn3
4657	1700064M	1.000	1.000	1.000	1.000	1.000	1.000	0.000	0.000	0.000	73457	1700064M	chr12	-	NR_04528	.	lncRNA	.	RIKEN cDN	1700064M
4658	Efcab11	1.580	0.633	1.000	0.305	1.000	0.423	0.000	0.660	0.000	78767	Efcab11	chr12	-	NM_0301	P18916	protein_cc	2610021K	EF-hand ca	Efcab11
4659	Tdp1	1.052	1.014	1.066	0.497	0.951	0.872	4.775	4.848	4.868	104884	Tdp1	chr12	+	NM_0283	P5583	protein_cc	2810481F	tyrosyl-DN	Tdp1
4660	Kcnk13	0.206	1.682	0.347	0.002	0.199	0.113	2.340	0.061	0.811	217826	Kcnk13	chr12	+	NM_1460	P23882	protein_cc	BB085247	potassium	Kcnk13
4661	Psmc1	0.993	1.592	1.582	0.381	0.004	0.069	10.243	10.233	10.904	19179	Psmc1	chr12	+	NM_0089	P229	protein_cc	AI325227|	protease (	Psmc1
4662	Nrde2	0.973	1.210	1.177	0.774	0.060	0.199	7.069	7.030	7.304	217827	Nrde2	chr12	-	NM_0012	P4415	protein_cc	6720454P	nrde-2 ne	Nrde2
4663	Gm10433	2.036	1.469	2.990	0.303	0.406	0.185	2.755	3.781	4.335	10003837	Gm10433	chr12	+	NR_04528	.	lncRNA	ENSMUSG	predicted	Gm10433
4664	Calm1	1.260	1.115	1.404	0.000	0.017	0.022	11.861	12.194	12.350	12313	Calm1	chr12	+	NM_0097	P956	protein_cc	AI256814|	calmoduli	Calm1
4665	Gm10432	0.456	1.249	0.569	0.014	0.423	0.014	6.150	5.016	5.338	10003871	Gm10432	chr12	+	NR_04574	.	lncRNA	ENSMUSG	predicted	Gm10432
4666	Ttc7b	0.393	1.294	0.508	0.010	0.417	0.024	6.468	5.120	5.491	104718	Ttc7b	chr12	-	NM_0010	P24045	protein_cc	AA408451	tetratricop	Ttc7b
4667	Rps6ka5	0.661	2.567	1.697	0.221	0.111	0.516	2.638	2.041	3.401	73086	Rps6ka5	chr12	-	NM_1535	P19464	protein_cc	3110005L	ribosomal	Rps6ka5
4668	9030617O	0.735	0.824	0.605	0.005	0.013	0.046	8.604	8.160	7.880	217830	9030617O	chr12	+	NM_1454	P3272	protein_cc	.	RIKEN cDN	9030617O
4669	Gpr68	1.246	0.094	0.118	0.771	1.000	0.236	3.088	3.406	0.000	238377	Gpr68	chr12	-	NM_0011	P24730	protein_cc	BB131428	G protein-	Gpr68
4670	Ccdc88c	0.386	1.346	0.520	0.182	0.200	0.530	1.405	0.033	0.461	68339	Ccdc88c	chr12	-	NM_0266	P20014	protein_cc	0610010D	coiled-coil	Ccdc88c
4671	Smek1	0.676	0.800	0.541	0.001	0.061	0.047	8.935	8.369	8.048	68734	Smek1	chr12	-	NM_0011	P8458	protein_cc	1110034C	SMEK hon	Smek1
4672	D130020L	0.087	1.851	0.162	0.000	0.001	0.004	8.342	4.824	5.713	319760	D130020L	chr12	+	NR_03804	.	lncRNA	9430007L	RIKEN cDN	D130020L
4673	Kif4-ps	1.000	1.000	1.000	1.000	1.000	1.000	0.000	0.000	0.000	74947	Kif4-ps	chr12	+	NR_03365	.	misc_RNA	4930463M	kinesin far	Kif4-ps
4674	Catsperb	1.000	1.000	1.000	1.000	1.000	1.000	0.000	0.000	0.000	271036	Catsperb	chr12	+	NM_1730	P16713	protein_cc	4931410B	catsper ch	Catsperb
4675	Tc2n	0.285	2.479	0.706	0.085	0.115	0.600	3.628	1.816	3.126	74413	Tc2n	chr12	-	NM_0012	P20945	protein_cc	4933406D	tandem C2	Tc2n
4676	Fbln5	0.140	1.318	0.185	0.000	0.482	0.008	5.679	2.842	3.240	23876	Fbln5	chr12	-	NM_0118	P22996	protein_cc	A55| DAN	fibulin 5	Fbln5
4677	Trip11	2.416	0.482	1.165	0.001	0.000	0.567	7.111	8.383	7.331	109181	Trip11	chr12	-	NM_0284	P20872	protein_cc	2610511G	thyroid ho	Trip11
4678	Atxn3	1.035	1.465	1.516	0.714	0.014	0.094	6.784	6.833	7.384	110616	Atxn3	chr12	-	NM_0297	P11097	protein_cc	2210008M	ataxin 3	Atxn3
4679	Cpsf2	2.541	0.718	1.824	0.000	0.005	0.119	7.741	9.086	8.608	51786	Cpsf2	chr12	+	NM_0168	P24940	protein_cc	100kDa| 2	cleavage a	Cpsf2
4680	Slc24a4	1.000	1.000	1.000	1.000	1.000	1.000	0.000	0.000	0.000	238384	Slc24a4	chr12	+	NM_1721	P10679	protein_cc	A930002M	solute car	Slc24a4
4681	Rin3	1.165	0.452	0.527	0.475	0.004	0.249	4.922	5.142	3.998	217835	Rin3	chr12	+	NM_1776	P2343	protein_cc	6430500K	Ras and Ra	Rin3
4682	Lgmn	1.589	0.960	1.525	0.003	0.477	0.055	8.052	8.721	8.661	19141	Lgmn	chr12	-	NM_0111	P12744	protein_cc	AEP| AI74	legumain	Lgmn
4683	Golga5	2.164	0.777	1.682	0.039	0.350	0.251	6.770	7.884	7.521	27277	Golga5	chr12	+	NM_0011	P11481	protein_cc	Ret-II	golgi auto	Golga5
4684	Chga	0.539	1.371	0.739	0.000	0.191	0.353	0.913	0.021	0.476	12652	Chga	chr12	+	NM_0076	P21459	protein_cc	cgA| ChrA	chromogra	Chga
4685	Itpk1	1.443	0.626	0.904	0.010	0.001	0.494	6.739	7.268	6.593	217837	Itpk1	chr12	-	NM_1725	P25567	protein_cc	BC031182	inositol 1,3	Itpk1
4686	Moap1	2.863	1.074	3.075	0.000	0.630	0.096	5.723	7.240	7.344	64113	Moap1	chr12	-	NM_0011	P15035	protein_cc	AA987038	modulator	Moap1
4687	Gm20604	2.863	1.074	3.075	0.000	0.630	0.096	5.723	7.240	7.344	10085993	Gm20604	chr12	-	NM_0011	P12533	protein_cc	AK010878	predicted	Gm20604
4688	Tmem251	2.445	1.128	2.759	0.024	0.517	0.143	5.625	6.915	7.089	320351	Tmem251	chr12	+	NM_1771	P12091	protein_cc	D230037D	transmem	Tmem251
4689	AK010878	1.909	0.672	1.282	0.003	0.012	0.388	5.876	6.808	6.234	10023317	AK010878	chr12	-	NM_0011	P23659	protein_cc	.	cDNA seq	AK010878
4690	Ubr7	1.930	0.736	1.420	0.001	0.046	0.286	6.589	7.537	7.095	66622	Ubr7	chr12	+	NM_0256	P21486	protein_cc	5730410I1	ubiquitin p	Ubr7
4691	Btbd7	1.309	0.661	0.865	0.220	0.018	0.731	5.144	5.532	4.935	238386	Btbd7	chr12	-	NM_1728	P3717	protein_cc	5730507E	BTB (POZ)	Btbd7
4692	Cox8c	1.000	1.000	1.000	1.000	1.000	1.000	0.000	0.000	0.000	75483	Cox8c	chr12	+	NM_0010	P21280	protein_cc	1700007F	cytochrom	Cox8c
4693	Unc79	1.384	1.527	2.113	0.240	0.405	0.468	3.246	3.715	4.325	217843	Unc79	chr12	+	NM_0010	P4338	protein_cc	9030205A	unc-79 ho	Unc79
4694	Prima1	1.000	1.000	1.000	1.000	1.000	1.000	0.000	0.000	0.000	170952	Prima1	chr12	-	NM_1333	P6881	protein_cc	B230212M	proline ric	Prima1
4695	Fam181a	1.000	1.000	1.000	1.000	1.000	1.000	0.000	0.000	0.000	10050415	Fam181a	chr12	+	NM_0011	P23439	protein_cc	EG544888	family wit	Fam181a
4696	Asb2	0.955	0.607	0.580	0.784	0.061	0.084	3.560	3.494	2.774	65256	Asb2	chr12	-	NM_0230	P11661	protein_cc	1110008E	ankyrin re	Asb2
4697	Otub2	2.110	0.306	0.647	0.009	0.002	0.226	4.695	5.772	4.066	68149	Otub2	chr12	+	NR_03358	.	protein_cc	2010015L	OTU doma	Otub2
4698	Ddx24	1.832	1.079	1.978	0.001	0.514	0.082	9.863	10.736	10.847	27225	Ddx24	chr12	-	NM_0011	P1465	protein_cc	1700055J	DEAD (Asp	Ddx24
4699	Ifi27l2a	1.373	0.822	1.128	0.006	0.088	0.535	6.862	7.319	7.035	76933	Ifi27l2a	chr12	-	NM_0012	P16094	protein_cc	2310061N	interferon	Ifi27l2a
4700	Ifi27	2.026	0.572	1.159	0.002	0.007	0.390	9.898	10.917	10.111	52668	Ifi27	chr12	+	NM_1940	P12292	protein_cc	1110013J	interferon	Ifi27
4701	Ifi27l2b	3.595	0.441	1.585	0.020	0.053	0.326	4.987	6.834	5.652	217845	Ifi27l2b	chr12	-	NM_1454	P14391	protein_cc	1810023F	interferon	Ifi27l2b
4702	Ppp4r4	0.615	0.473	0.291	0.090	0.005	0.135	5.608	4.908	3.827	74521	Ppp4r4	chr12	+	NM_0289	P11160	protein_cc	8430415E	protein ph	Ppp4r4
4703	Serpina10	5.149	1.131	5.825	0.001	0.263	0.006	10.859	13.223	13.401	217847	Serpina10	chr12	-	NM_0013	P5144	protein_cc	PZI| ZPI	serine (or	Serpina10
4704	Serpina6	0.376	0.377	0.142	0.000	0.009	0.007	11.558	10.145	8.737	12401	Serpina6	chr12	-	NM_0076	P12648	protein_cc	Cbg	serine (or	Serpina6
4705	Serpina1f	1.010	1.825	1.844	0.000	0.182	0.423	0.000	0.015	0.883	68348	Serpina1f	chr12	-	NM_0266	P10106	protein_cc	0610012A	serine (or	Serpina1f
4706	Serpina1b	0.475	0.696	0.331	0.005	0.110	0.024	16.457	15.383	14.861	20701	Serpina1b	chr12	-	NM_0092	P19605	protein_cc	D12Ucla2|	serine (or	Serpina1b
4707	Serpina1d	0.467	0.654	0.305	0.000	0.000	0.001	16.481	15.381	14.769	20703	Serpina1d	chr12	-	NM_0092	P442	protein_cc	AI118301|	serine (or	Serpina1d
4708	Serpina1a	0.391	1.199	0.469	0.000	0.166	0.014	15.557	14.203	14.465	20700	Serpina1a	chr12	-	NM_0012	P15241	protein_cc	Aat-2| Aat	serine (or	Serpina1a
4709	Serpina1c	0.506	1.279	0.647	0.009	0.104	0.125	16.491	15.509	15.864	20702	Serpina1c	chr12	-	NM_0092	P564	protein_cc	Pi3| Pi6| S	serine (or	Serpina1c
4710	Serpina1e	1.155	10.556	12.188	0.523	0.001	0.026	11.860	12.067	15.467	20704	Serpina1e	chr12	-	NM_0092	P4405	protein_cc	Dom5| PI5	serine (or	Serpina1e
4711	Serpina11	1.861	1.736	3.231	0.000	0.002	0.017	7.828	8.725	9.521	380780	Serpina11	chr12	-	NM_0011	P8076	protein_cc	Gm895	serine (or	Serpina11
4712	Serpina9	0.467	2.169	1.012	0.185	0.185	0.989	1.137	0.038	1.155	71907	Serpina9	chr12	-	NM_0279	P2921	protein_cc	2310014L	serine (or	Serpina9
4713	Serpina12	5.493	2.784	15.290	0.000	0.015	0.067	4.726	7.183	8.660	68054	Serpina12	chr12	-	NM_0265	P22155	protein_cc	4632419J1	serine (or	Serpina12
4714	Serpina4-p	14.056	1.311	18.422	0.000	0.000	0.001	4.491	8.304	8.694	321018	Serpina4-p	chr12	+	NR_00286	.	misc_RNA	KALps| Kls	serine (or	Serpina4-p
4715	Serpina5	9.422	1.603	15.105	0.001	0.004	0.001	0.002	3.238	3.919	268591	Serpina5	chr12	+	NM_1729	P3122	protein_cc	4933415L	serine (or	Serpina5
4716	Serpina3a	1.000	1.000	1.000	1.000	1.000	1.000	0.000	0.000	0.000	74069	Serpina3a	chr12	+	NM_0011	P1674	protein_cc	.	serine (or	Serpina3a
4717	Serpina3b	1.000	1.000	1.000	1.000	1.000	1.000	0.000	0.000	0.000	271047	Serpina3b	chr12	+	NM_1730	P17308	protein_cc	6A1| A030	serine (or	Serpina3b
4718	Serpina3c	3.206	1.453	4.657	0.000	0.091	0.073	8.233	9.913	10.452	16625	Serpina3c	chr12	-	NM_0084	P20169	protein_cc	1A1| Kalb	serine (or	Serpina3c
4719	Serpina3g	29.843	0.358	10.691	0.000	0.003	0.183	5.480	10.379	8.898	20715	Serpina3g	chr12	+	NM_0092	P15558	protein_cc	2A2| AI11	serine (or	Serpina3g

4720	Serpina3f	190.157	0.395	75.148	0.000	0.016	0.234	5.622	13.194	11.854	238393	Serpina3f	chr12	+	NM_0011	P4211	protein_cc	2A1| BC04	serine (or	Serpina3f
4721	Serpina3h	56.054	1.291	72.342	0.000	0.061	0.020	2.310	8.119	8.487	546546	Serpina3h	chr12	+	NR_03345	.	misc_RNA	6C28| Spi2	serine (or	Serpina3h
4722	Serpina3i	256.052	1.550	396.996	0.000	0.158	0.096	0.817	8.817	9.450	628900	Serpina3i	chr12	+	NM_0011	P16747	protein_cc	2B2| EG62	serine (or	Serpina3i
4723	Serpina3j	1.015	2.229	2.261	0.000	0.007	0.086	0.000	0.021	1.177	238395	Serpina3j	chr12	+	NM_0011	P25519	protein_cc	AI506554|	serine (or	Serpina3j
4724	Serpina3k	2.423	1.208	2.926	0.003	0.122	0.017	15.857	17.134	17.406	20714	Serpina3k	chr12	+	NM_0114	P2792	protein_cc	1300001I0	serine (or	Serpina3k
4725	Serpina3m	4.757	1.140	5.424	0.004	0.535	0.070	11.939	14.189	14.378	20717	Serpina3m	chr12	+	NM_0092	P16472	protein_cc	3e46| AI1	serine (or	Serpina3m
4726	Serpina3n	25.508	1.017	25.937	0.000	0.846	0.014	11.948	16.621	16.645	20716	Serpina3n	chr12	+	NM_0092	P17431	protein_cc	Spi2-2| Sp	serine (or	Serpina3n
4727	Gsc	1.010	1.885	1.904	0.000	0.182	0.423	0.000	0.015	0.929	14836	Gsc	chr12	-	NM_0103	P4385	protein_cc	.	goosecoid	Gsc
4728	Dicer1	1.163	1.211	1.408	0.120	0.001	0.098	8.389	8.607	8.883	192119	Dicer1	chr12	-	NM_1489	P21108	protein_cc	1110006F	dicer 1, rib	Dicer1
4729	Clmn	0.121	1.965	0.238	0.000	0.047	0.020	10.301	7.255	8.229	94040	Clmn	chr12	-	NM_0010	P4970	protein_cc	9330188N	calmin	Clmn
4730	Syne3	0.830	0.623	0.517	0.647	0.184	0.406	4.809	4.540	3.856	212073	Syne3	chr12	-	NM_0010	P7308	protein_cc	nesprin-3	spectrin re	Syne3
4731	Snhg10	1.374	1.064	1.463	0.588	0.928	0.743	1.740	2.199	2.289	69434	Snhg10	chr12	-	NR_00314	.	lncRNA	1700026B	small nucl	Snhg10
4732	Scarna13	0.398	0.595	0.237	0.133	0.435	0.220	2.490	1.162	0.413	10030694	Scarna13	chr12	-	NR_02857	.	guide_RN	U93	small Caja	Scarna13
4733	Glrx5	0.386	1.694	0.654	0.002	0.002	0.131	11.173	9.799	10.559	73046	Glrx5	chr12	+	NM_0284	P4626	protein_cc	.	glutaredox	Glrx5
4734	Tcl1b2	1.000	1.000	1.000	1.000	1.000	1.000	0.000	0.000	0.000	27381	Tcl1b2	chr12	+	NM_0137	P25500	protein_cc	.	T cell leuk	Tcl1b2
4735	Tcl1b1	1.000	1.000	1.000	1.000	1.000	1.000	0.000	0.000	0.000	27379	Tcl1b1	chr12	+	NM_0137	P22046	protein_cc	C88214	T cell leuk	Tcl1b1
4736	Tcl1b5	1.000	1.000	1.000	1.000	1.000	1.000	0.000	0.000	0.000	27382	Tcl1b5	chr12	+	NM_0137	P18653	protein_cc	D12Ertd64	T cell leuk	Tcl1b5
4737	Tcl1b3	1.000	1.000	1.000	1.000	1.000	1.000	0.000	0.000	0.000	27378	Tcl1b3	chr12	+	NM_0137	P12415	protein_cc	.	T cell leuk	Tcl1b3
4738	Tcl1b4	1.000	1.000	1.000	1.000	1.000	1.000	0.000	0.000	0.000	27380	Tcl1b4	chr12	+	NM_0137	P17622	protein_cc	AA675342	T cell leuk	Tcl1b4
4739	Tcl1	1.000	1.000	1.000	1.000	1.000	1.000	0.000	0.000	0.000	21432	Tcl1	chr12	-	NM_0013	P1326	protein_cc	Tcl1a	T cell lymp	Tcl1
4740	Tunar	1.000	1.000	1.000	1.000	1.000	1.000	0.000	0.000	0.000	69952	Tunar	chr12	+	NR_04504	.	lncRNA	2810011L	Tcl1 upstr	Tunar
4741	Gm2721	1.000	1.000	1.000	1.000	1.000	1.000	0.000	0.000	0.000	10004033	Gm2721	chr12	-	NR_04508	.	lncRNA	10004033	predicted	Gm2721
4742	D430019H	0.676	0.939	0.635	0.254	0.869	0.348	4.411	3.845	3.754	268595	D430019H	chr12	+	NM_0012	P9673	protein_cc	AI852661	RIKEN cDN	D430019H
4743	AU015791	0.292	0.977	0.285	0.181	1.000	0.423	1.810	0.034	0.000	104932	AU015791	chr12	+	NR_10238	.	lncRNA	.	expressed	AU015791
4744	Bdkrb2	1.000	1.000	1.000	1.000	1.000	1.000	0.000	0.000	0.000	12062	Bdkrb2	chr12	+	NM_0097	P24510	protein_cc	B(2)| B2|	bradykinin	Bdkrb2
4745	Bdkrb1	2.134	0.469	1.000	0.305	1.000	0.423	0.000	1.094	0.000	12061	Bdkrb1	chr12	+	NM_0075	P17666	protein_cc	B1BKR| B1	bradykinin	Bdkrb1
4746	Atg2b	0.561	0.864	0.485	0.002	0.505	0.098	8.110	7.277	7.067	76559	Atg2b	chr12	-	NM_0296	P3367	protein_cc	2410024A	autophagy	Atg2b
4747	Gskip	1.029	1.371	1.411	0.909	0.199	0.099	6.940	6.981	7.437	66787	Gskip	chr12	+	NM_1786	P22405	protein_cc	4933433P	GSK3B int	Gskip
4748	Ak7	1.240	2.857	3.542	0.599	0.085	0.240	0.409	0.720	2.234	78801	Ak7	chr12	+	NM_0301	P18359	protein_cc	4930502N	adenylate	Ak7
4749	Papola	1.590	0.677	1.076	0.018	0.014	0.673	9.748	10.417	9.854	18789	Papola	chr12	+	NM_0111	P24330	protein_cc	Pap| PapII	poly (A) po	Papola
4750	Vrk1	0.963	0.881	0.849	0.859	0.607	0.624	6.455	6.401	6.219	22367	Vrk1	chr12	+	NM_0010	P22533	protein_cc	51PK	vaccinia re	Vrk1
4751	1700121N	1.000	1.000	1.000	1.000	1.000	1.000	0.000	0.000	0.000	76639	1700121N	chr12	-	NR_03659	.	lncRNA	.	RIKEN cDN	1700121N
4752	4933406K	1.000	1.000	1.000	1.000	1.000	1.000	0.000	0.000	0.000	71033	4933406K	chr12	-	NR_01551	.	lncRNA	4930426M	RIKEN cDN	4933406K
4753	4930465M	1.000	1.000	1.000	1.000	1.000	1.000	0.000	0.000	0.000	74968	4930465M	chr12	-	NR_04597	.	lncRNA	4930431H	RIKEN cDN	4930465M
4754	Bcl11b	2.350	0.176	0.415	0.002	0.001	0.062	2.590	3.823	1.320	58208	Bcl11b	chr12	-	NM_0012	P19054	protein_cc	9130430L	B cell leuk	Bcl11b
4755	Setd3	0.489	0.962	0.470	0.000	0.209	0.009	9.330	8.299	8.243	52690	Setd3	chr12	-	NM_0282	P3899	protein_cc	2610102I0	SET domai	Setd3
4756	Ccnk	1.117	0.853	0.953	0.031	0.243	0.834	7.926	8.085	7.855	12454	Ccnk	chr12	+	NM_0098	P24454	protein_cc	AW12319	cyclin K	Ccnk
4757	Ccdc85c	1.000	1.000	1.000	1.000	1.000	1.000	0.000	0.000	0.000	668158	Ccdc85c	chr12	-	NM_0011	P11367	protein_cc	EG668158	coiled-coil	Ccdc85c
4758	Hhipl1	2.149	0.465	1.000	0.306	1.000	0.423	0.000	1.104	0.000	214305	Hhipl1	chr12	+	NM_0010	P13103	protein_cc	1600002O	hedgehog	Hhipl1
4759	Cyp46a1	0.549	0.247	0.136	0.027	1.000	0.041	2.879	2.015	0.000	13116	Cyp46a1	chr12	+	NM_0100	P19212	protein_cc	Cyp46	cytochrom	Cyp46a1
4760	Eml1	0.998	2.127	2.122	0.989	0.172	0.412	3.961	3.958	5.047	68519	Eml1	chr12	+	NM_0012	P3041	protein_cc	1110008N	echinoder	Eml1
4761	Gm16596	0.951	1.780	1.693	0.916	0.208	0.480	3.011	2.938	3.770	10050446	Gm16596	chr12	-	NR_04575	.	lncRNA	.	predicted	Gm16596
4762	Evl	1.685	0.287	0.483	0.261	0.023	0.519	3.890	4.643	2.839	14026	Evl	chr12	+	NM_0079	P2342	protein_cc	AI528774	Ena-vasod	Evl
4763	Degs2	1.568	0.356	0.558	0.337	0.032	0.587	3.995	4.644	3.153	70059	Degs2	chr12	-	NM_0011	P6569	protein_cc	2210008A	delta(4)-de	Degs2
4764	Yy1	1.182	0.689	0.814	0.002	0.000	0.047	9.301	9.542	9.003	22632	Yy1	chr12	+	NM_0095	P25980	protein_cc	AW48867	YY1 transc	Yy1
4765	Slc25a29	1.687	0.862	1.454	0.364	0.722	0.503	3.410	4.165	3.950	214663	Slc25a29	chr12	-	NM_1813	P8748	protein_cc	C030003J	solute carr	Slc25a29
4766	Slc25a47	0.337	3.382	1.139	0.000	0.036	0.772	12.014	10.445	12.202	104910	Slc25a47	chr12	+	NM_0010	P25213	protein_cc	AI132487|	solute carr	Slc25a47
4767	Wars	15.015	0.477	7.159	0.000	0.007	0.126	5.778	9.686	8.618	22375	Wars	chr12	-	NM_0117	P888	protein_cc	TrpRS| WI	tryptopha	Wars
4768	Wdr25	1.002	1.719	1.723	0.991	0.004	0.155	4.581	4.584	5.366	212198	Wdr25	chr12	+	NM_1776	P15027	protein_cc	B930090D	WD repea	Wdr25
4769	Begain	1.000	1.000	1.000	1.000	1.000	1.000	0.000	0.000	0.000	380785	Begain	chr12	-	NM_0011	P25355	protein_cc	BM94837	brain-enri	Begain
4770	Dlk1	0.031	3.877	0.122	0.179	0.191	0.463	5.156	0.166	2.121	13386	Dlk1	chr12	+	NR_03381	.	protein_cc	AW74267	delta-like	Dlk1
4771	Meg3	0.562	1.129	0.634	0.001	0.389	0.076	5.305	4.474	4.648	17263	Meg3	chr12	+	NR_02765	.	lncRNA	2900016C	maternally	Meg3
4772	Rtl1	1.000	1.000	1.000	1.000	1.000	1.000	0.000	0.000	0.000	353326	Rtl1	chr12	-	NM_1841	P11956	protein_cc	6430411K	retrotrans	Rtl1
4773	6430411K	1.000	1.000	1.000	1.000	1.000	1.000	0.000	0.000	0.000	76880	6430411K	chr12	+	NR_00284	.	lncRNA	PEG11as	RIKEN cDN	6430411K
4774	Rian	1.112	1.591	1.768	0.859	0.369	0.254	2.404	2.556	3.226	75745	Rian	chr12	+	NR_02826	.	lncRNA	5530401N	RNA impri	Rian
4775	AF357425	2.179	1.013	2.208	0.302	0.983	0.423	0.001	1.124	1.143	449562	AF357425	chr12	+	NR_04630	.	snoRNA	MBII-48	snoRNA A	AF357425
4776	AF357426	1.000	1.000	1.000	1.000	1.000	1.000	0.000	0.000	0.000	449561	AF357426	chr12	+	NR_04630	.	snoRNA	MBII-49	snoRNA A	AF357426
4777	DQ267100	1.000	1.000	1.000	1.000	1.000	1.000	0.000	0.000	0.000	10086157	DQ267100	chr12	+	NR_04630	.	snoRNA	.	snoRNA D	DQ267100
4778	AF357359	1.005	1.439	1.446	0.000	0.182	0.423	0.000	0.008	0.532	10030364	AF357359	chr12	+	NR_02843	.	snoRNA	MBII-426	snoRNA A	AF357359
4779	DQ267101	1.000	1.000	1.000	1.000	1.000	1.000	0.000	0.000	0.000	10086157	DQ267101	chr12	+	NR_04630	.	snoRNA	.	snoRNA D	DQ267101
4780	AF357355	1.000	1.000	1.000	1.000	1.000	1.000	0.000	0.000	0.000	10030364	AF357355	chr12	+	NR_02843	.	snoRNA	MBII-343	snoRNA A	AF357355
4781	DQ267102	1.000	1.000	1.000	1.000	1.000	1.000	0.000	0.000	0.000	10086157	DQ267102	chr12	+	NR_04630	.	snoRNA	.	snoRNA D	DQ267102
4782	3110009F	0.480	1.334	0.640	0.319	0.570	0.692	1.763	0.705	1.120	67280	3110009F	chr12	-	NR_04546	.	lncRNA	.	RIKEN cDN	3110009F
4783	Dio3os	0.122	0.944	0.115	0.010	1.000	0.103	3.121	0.084	0.000	353504	Dio3os	chr12	-	NR_00286	.	lncRNA	AW55646	deiodinase	Dio3os
4784	Dio3	0.288	0.974	0.281	0.056	1.000	0.242	1.833	0.038	0.000	107585	Dio3	chr12	+	NM_1721	P14471	protein_cc	.	deiodinase	Dio3
4785	Ppp2r5c	0.756	1.284	0.970	0.013	0.226	0.919	8.433	8.029	8.389	26931	Ppp2r5c	chr12	+	NM_0011	P24452	protein_cc	2610043M	protein ph	Ppp2r5c
4786	B930059L	2.064	0.306	0.632	0.000	0.000	0.205	1.139	2.185	0.476	319786	B930059L	chr12	+	NR_03334	.	lncRNA	.	RIKEN cDN	B930059L
4787	Dync1h1	0.926	0.793	0.735	0.461	0.000	0.217	9.414	9.303	8.969	13424	Dync1h1	chr12	+	NM_0302	P9570	protein_cc	9930018I2	dynein cyt	Dync1h1
4788	1700001K	1.000	1.000	1.000	1.000	1.000	1.000	0.000	0.000	0.000	66323	1700001K	chr12	-	NM_0254	P6847	protein_cc	.	RIKEN cDN	1700001K
4789	Hsp90aa1	4.355	0.698	3.039	0.000	0.011	0.028	10.814	12.937	12.418	15519	Hsp90aa1	chr12	-	NM_0104	P6343	protein_cc	86kDa| 89	heat shock	Hsp90aa1
4790	Wdr20	1.653	0.852	1.408	0.000	0.041	0.050	5.771	6.496	6.264	69641	Wdr20	chr12	+	NM_0271	P20541	protein_cc	2310040A	WD repea	Wdr20
4791	Mok	3.032	0.765	2.320	0.152	0.616	0.348	0.392	1.992	1.606	26448	Mok	chr12	-	NM_0119	P2333	protein_cc	Rage| RAC	MOK prote	Mok
4792	Zfp839	0.833	0.828	0.690	0.306	0.345	0.267	7.157	6.894	6.621	72805	Zfp839	chr12	+	NM_0011	P10867	protein_cc	2810455K	zinc finger	Zfp839
4793	Cinp	1.747	1.116	1.949	0.000	0.306	0.074	4.984	5.789	5.947	67236	Cinp	chr12	-	NM_0260	P5923	protein_cc	1810047K	cyclin-dep	Cinp
4794	Tecpr2	1.865	0.703	1.310	0.006	0.032	0.116	5.788	6.687	6.178	104859	Tecpr2	chr12	+	NM_1753	P7049	protein_cc	4930573I1	tectonin b	Tecpr2
4795	Ankrd9	2.144	0.727	1.559	0.117	0.374	0.116	3.707	4.808	4.348	74251	Ankrd9	chr12	-	NM_1752	P19289	protein_cc	2500003O	ankyrin re	Ankrd9
4796	6030440G	1.005	1.439	1.446	0.000	0.182	0.423	0.000	0.008	0.532	77878	6030440G	chr12	+	NR_03659	.	lncRNA	.	RIKEN cDN	6030440G
4797	Rcor1	1.330	0.932	1.239	0.002	0.117	0.003	7.204	7.615	7.513	217864	Rcor1	chr12	+	NM_1980	P20123	protein_cc	5730409O	REST core	Rcor1
4798	4930595D	1.000	1.000	1.000	1.000	1.000	1.000	0.000	0.000	0.000	78062	4930595D	chr12	-	NR_13098	.	lncRNA	.	RIKEN cDN	4930595D
4799	Traf3	3.460	0.815	2.820	0.004	0.183	0.002	6.032	7.822	7.528	22031	Traf3	chr12	+	NM_0116	P24553	protein_cc	AI528849|	TNF recep	Traf3
4800	Amn	1.426	1.926	2.746	0.622	0.390	0.468	3.644	4.156	5.101	93835	Amn	chr12	+	NM_0336	P7135	protein_cc	.	amnionles	Amn
4801	Cdc42bpb	0.675	1.195	0.806	0.069	0.199	0.523	7.684	7.116	7.373	217866	Cdc42bpb	chr12	-	NM_1830	P8210	protein_cc	MRCKb	CDC42 bin	Cdc42bpb
4802	A230065H	1.000	1.000	1.000	1.000	1.000	1.000	0.000	0.000	0.000	380787	A230065H	chr12	+	NM_0011	P16971	protein_cc	Gm898| L	RIKEN cDN	A230065H
4803	Exoc3l4	9.433	0.458	4.316	0.001	0.004	0.007	3.379	6.616	5.488	74190	Exoc3l4	chr12	+	NM_0012	P8156	protein_cc	1200009I0	exocyst co	Exoc3l4
4804	Tnfaip2	4.989	0.306	1.529	0.000	0.000	0.062	8.229	10.547	8.841	21928	Tnfaip2	chr12	+	NM_0093	P21990	protein_cc	B94| Exoc	tumor nec	Tnfaip2
4805	Gm266	1.616	1.066	1.722	0.456	0.916	0.549	0.426	1.119	1.210	212539	Gm266	chr12	-	NM_0010	P10969	protein_cc	.	predicted	Gm266
4806	Eif5	1.018	1.179	1.200	0.591	0.007	0.087	11.350	11.376	11.613	217869	Eif5	chr12	+	NM_1780	P191	protein_cc	2810011H	eukaryotic	Eif5
4807	Snora28	0.484	0.743	0.359	0.023	0.430	0.141	2.559	1.511	1.082	10031693	Snora28	chr12	+	NR_03316	.	snoRNA	MBI-13	small nucl	Snora28
4808	2810029C	0.411	2.569	1.057	0.185	0.000	0.933	1.325	0.044	1.405	10050468	2810029C	chr12	-	NR_04529	.	lncRNA	AU017849	RIKEN cDN	2810029C
4809	Mark3	1.385	0.921	1.276	0.023	0.129	0.290	7.732	8.201	8.083	17169	Mark3	chr12	+	NM_0215	P26638	protein_cc	1600015G	MAP/micr	Mark3
4810	Ckb	0.063	3.717	0.233	0.000	0.110	0.033	6.266	2.269	4.163	12709	Ckb	chr12	-	NM_0212	P3119	protein_cc	B-CK| Bck	creatine ki	Ckb

4811	Trmt61a	4.580	1.119	5.124	0.003	0.703	0.177	5.087	7.283	7.445	328162	Trmt61a	chr12	+	NM_0010	P14938	protein_cc	6720458F	tRNA met	Trmt61a
4812	Bag5	1.242	1.085	1.348	0.007	0.363	0.154	7.035	7.347	7.466	70369	Bag5	chr12	-	NM_0274	P19221	protein_cc	1600025G	BCL2-asso	Bag5
4813	Apopt1	0.340	2.364	0.804	0.027	0.077	0.689	6.998	5.443	6.684	68020	Apopt1	chr12	+	NM_0011	P13862	protein_cc	1700081D	apoptoge	Apopt1
4814	Klc1	1.556	0.722	1.124	0.015	0.030	0.230	7.665	8.303	7.833	16593	Klc1	chr12	+	NM_0084	P13727	protein_cc	AI874768	kinesin lig	Klc1
4815	Xrcc3	0.365	2.023	0.738	0.064	0.062	0.586	3.262	1.807	2.823	74335	Xrcc3	chr12	-	NM_0288	P2395	protein_cc	.	X-ray repa	Xrcc3
4816	Zfyve21	0.180	1.805	0.324	0.000	0.000	0.035	8.725	6.248	7.100	68520	Zfyve21	chr12	+	NM_0267	P25250	protein_cc	1110013H	zinc finger	Zfyve21
4817	Ppp1r13b	1.475	0.592	0.873	0.092	0.051	0.199	5.277	5.837	5.081	21981	Ppp1r13b	chr12	-	NM_0116	P9919	protein_cc	AI449786	protein ph	Ppp1r13b
4818	5033406O	1.472	1.137	1.674	0.006	0.081	0.073	5.629	6.187	6.372	77675	5033406O	chr12	-	NR_02946	.	lncRNA	9130204G	RIKEN cDN	5033406O
4819	2010107E	0.897	1.084	0.972	0.035	0.107	0.707	11.326	11.169	11.285	70257	2010107E	chr12	-	NM_0273	P6980	protein_cc	9430003J	RIKEN cDN	2010107E
4820	Tdrd9	1.000	1.000	1.000	1.000	1.000	1.000	0.000	0.000	0.000	74691	Tdrd9	chr12	+	NM_0290	P2961	protein_cc	4930441E	tudor dom	Tdrd9
4821	Rd3l	1.000	1.000	1.000	1.000	1.000	1.000	0.000	0.000	0.000	217874	Rd3l	chr12	-	NM_0011	P16576	protein_cc	A930011N	retinal deg	Rd3l
4822	Aspg	0.159	2.148	0.342	0.000	0.136	0.063	9.652	7.002	8.105	104816	Aspg	chr12	+	NM_0010	P21224	protein_cc	A530050D	asparagin	Aspg
4823	Kif26a	0.281	1.190	0.334	0.027	0.757	0.253	4.400	2.568	2.819	668303	Kif26a	chr12	+	NM_0010	P18967	protein_cc	mKIAA123	kinesin fa	Kif26a
4824	A730018C	1.000	1.000	1.000	1.000	1.000	1.000	0.000	0.000	0.000	10050473	A730018C	chr12	-	NR_03645	.	lncRNA	.	RIKEN cDN	A730018C
4825	B020018J	1.000	1.000	1.000	1.000	1.000	1.000	0.000	0.000	0.000	10012669	B020018J	chr12	-	NR_04595	.	lncRNA	.	Riken cDN	B020018J
4826	A530016L	1.000	1.000	1.000	1.000	1.000	1.000	0.000	0.000	0.000	319942	A530016L	chr12	+	NM_1770	P5030	protein_cc	Nrac	RIKEN cDN	A530016L
4827	Tmem179	1.000	1.000	1.000	1.000	1.000	1.000	0.000	0.000	0.000	104885	Tmem179	chr12	-	NM_1789	P25060	protein_cc	AI839735	transmem	Tmem179
4828	Inf2	0.379	0.849	0.322	0.001	0.465	0.037	8.325	6.927	6.691	70435	Inf2	chr12	+	NM_1984	P25221	protein_cc	2610204M	inverted fo	Inf2
4829	Adssl1	0.182	0.500	0.091	0.001	0.214	0.015	7.051	4.596	3.595	11565	Adssl1	chr12	+	NM_0074	P5735	protein_cc	Adss| Ads	adenylosu	Adssl1
4830	Siva1	0.470	1.120	0.526	0.018	0.579	0.187	7.139	6.048	6.212	30954	Siva1	chr12	+	NM_0011	P6601	protein_cc	CD27bp| S	SIVA1, apo	Siva1
4831	Akt1	1.044	1.064	1.111	0.714	0.716	0.664	9.563	9.625	9.715	11651	Akt1	chr12	-	NM_0011	P11422	protein_cc	Akt| PKB|	thymoma	Akt1
4832	Zbtb42	1.646	1.264	2.081	0.158	0.306	0.011	4.239	4.959	5.296	382639	Zbtb42	chr12	+	NM_0011	P3935	protein_cc	EG382639	zinc finger	Zbtb42
4833	Cep170b	1.387	0.921	1.277	0.144	0.650	0.142	8.087	8.559	8.440	217882	Cep170b	chr12	+	NM_0010	P8374	protein_cc	AI466840	centrosom	Cep170b
4834	Pld4	0.166	1.424	0.236	0.000	0.270	0.023	5.553	2.959	3.469	104759	Pld4	chr12	+	NM_1789	P21249	protein_cc	AI132321	phospholi	Pld4
4835	BC022687	8.385	1.466	12.291	0.000	0.012	0.018	0.992	4.060	4.612	217887	BC022687	chr12	+	NM_1454	P24777	protein_cc	AW05000	cDNA seq	BC022687
4836	Cdca4	1.736	1.001	1.739	0.030	0.992	0.017	6.559	7.355	7.357	71963	Cdca4	chr12	-	NM_0280	P10135	protein_cc	2410018C	cell divisio	Cdca4
4837	Gpr132	1.192	0.270	0.322	0.832	0.270	0.498	3.138	3.392	1.504	56696	Gpr132	chr12	-	NM_0199	P26181	protein_cc	G2a	G protein-	Gpr132
4838	Jag2	1.572	0.636	1.000	0.304	1.000	0.423	0.000	0.653	0.000	16450	Jag2	chr12	-	NM_0105	P2107	protein_cc	D12Ggc2e	jagged 2	Jag2
4839	Nudt14	0.606	1.472	0.892	0.003	0.006	0.495	6.899	6.177	6.734	66174	Nudt14	chr12	-	NM_0253	P15390	protein_cc	1110030M	nudix (nuc	Nudt14
4840	Brf1	0.750	1.142	0.857	0.013	0.525	0.604	6.220	5.805	5.997	72308	Brf1	chr12	-	NM_0281	P5762	protein_cc	2510002F	BRF1, RNA	Brf1
4841	Btbd6	0.536	1.430	0.766	0.000	0.021	0.151	6.724	5.823	6.339	399566	Btbd6	chr12	+	NM_0011	P9993	protein_cc	.	BTB (POZ)	Btbd6
4842	Pacs2	1.062	0.847	0.899	0.697	0.087	0.679	6.392	6.479	6.239	217893	Pacs2	chr12	+	NM_0012	P7247	protein_cc	6720425G	phosphofu	Pacs2
4843	Tex22	1.019	2.648	2.698	0.000	0.182	0.423	0.000	0.027	1.432	75671	Tex22	chr12	+	NM_0293	P5648	protein_cc	1700028O	testis expr	Tex22
4844	Mta1	0.787	1.226	0.965	0.055	0.005	0.796	8.778	8.433	8.727	116870	Mta1	chr12	+	NM_0540	P20821	protein_cc	.	metastasis	Mta1
4845	Crip2	0.173	1.672	0.289	0.001	0.014	0.049	9.972	7.439	8.181	68337	Crip2	chr12	+	NM_0242	P280	protein_cc	0610010I2	cysteine ri	Crip2
4846	Crip1	0.199	1.787	0.355	0.000	0.093	0.004	5.911	3.581	4.418	12925	Crip1	chr12	+	NM_0077	P16206	protein_cc	CRHP| Cri	cysteine-r	Crip1
4847	4930427A	3.936	0.775	3.049	0.305	0.753	0.279	0.001	1.978	1.609	104732	4930427A	chr12	+	NM_1340	P22475	protein_cc	AA536649	RIKEN cDN	4930427A
4848	Tmem121	0.355	0.981	0.348	0.056	1.000	0.240	1.524	0.028	0.000	69195	Tmem121	chr12	+	NM_1537	P25058	protein_cc	2410008J	transmem	Tmem121
4849	Adam6b	1.000	1.000	1.000	1.000	1.000	1.000	0.000	0.000	0.000	238405	Adam6b	chr12	+	NM_0010	P1277	protein_cc	4930523C	a disintegr	Adam6b
4850	Adam6a	1.000	1.000	1.000	1.000	1.000	1.000	0.000	0.000	0.000	238406	Adam6a	chr12	+	NM_1748	P14565	protein_cc	Adam6| th	a disintegr	Adam6a
4851	Zfp386	1.342	0.799	1.072	0.347	0.458	0.773	6.896	7.321	6.996	56220	Zfp386	chr12	+	NM_0010	P25919	protein_cc	Kzf1| mKI	zinc finger	Zfp386
4852	Vipr2	0.716	0.995	0.712	0.182	1.000	0.423	0.490	0.008	0.000	22355	Vipr2	chr12	+	NM_0095	P20956	protein_cc	Vip2| VPA	vasoactive	Vipr2
4853	Wdr60	1.136	0.768	0.873	0.527	0.236	0.621	6.070	6.253	5.874	217935	Wdr60	chr12	-	NM_1460	P8973	protein_cc	D430033N	WD repea	Wdr60
4854	Esyt2	0.848	0.979	0.830	0.011	0.879	0.460	7.208	6.970	6.939	52635	Esyt2	chr12	+	NM_0287	P13344	protein_cc	2410017M	extended	Esyt2
4855	D430020J	4.175	0.535	2.235	0.000	0.000	0.100	0.735	2.796	1.895	319545	D430020J	chr12	-	NR_02842	.	lncRNA	.	RIKEN cDN	D430020J
4856	Ncapg2	1.318	0.578	0.761	0.322	0.236	0.721	2.743	3.142	2.350	76044	Ncapg2	chr12	+	NM_1337	P21954	protein_cc	5830426I	non-SMC	Ncapg2
4857	Ptprn2	1.000	1.000	1.000	1.000	1.000	1.000	0.000	0.000	0.000	19276	Ptprn2	chr12	+	NM_0112	P3200	protein_cc	4930425H	protein ty	Ptprn2
4858	Gm10421	1.000	1.000	1.000	1.000	1.000	1.000	0.000	0.000	0.000	10003868	Gm10421	chr12	-	NR_03353	.	lncRNA	ENSMUSG	predicted	Gm10421
4859	Gm5441	1.000	1.000	1.000	1.000	1.000	1.000	0.000	0.000	0.000	432713	Gm5441	chr12	-	NR_04498	.	lncRNA	432713	predicted	Gm5441
4860	Rapgef5	3.944	0.910	3.590	0.030	0.710	0.007	4.539	6.519	6.383	217944	Rapgef5	chr12	+	NM_1759	P14	protein_cc	4932413M	Rap guani	Rapgef5
4861	Cdca7l	0.438	0.649	0.284	0.161	0.567	0.211	2.679	1.489	0.864	217946	Cdca7l	chr12	+	NM_1460	P6352	protein_cc	BC006933	cell divisio	Cdca7l
4862	Dnah11	0.822	0.353	0.290	0.752	0.364	0.059	2.259	1.975	0.471	13411	Dnah11	chr12	-	NM_0100	P2036	protein_cc	b2b1203C	dynein, ax	Dnah11
4863	Sp4	0.917	0.408	0.374	0.600	0.006	0.136	7.361	7.236	5.942	20688	Sp4	chr12	-	NM_0092	P13477	protein_cc	5730497N	trans-acti	Sp4
4864	D230030E	1.000	1.000	1.000	1.000	1.000	1.000	0.000	0.000	0.000	10038489	D230030E	chr12	-	NR_04594	.	lncRNA	Npe	Riken cDN	D230030E
4865	Sp8	1.000	1.000	1.000	1.000	1.000	1.000	0.000	0.000	0.000	320145	Sp8	chr12	+	NM_1770	P1638	protein_cc	D930049B	trans-acti	Sp8
4866	Abcb5	1.000	1.000	1.000	1.000	1.000	1.000	0.000	0.000	0.000	77706	Abcb5	chr12	-	NM_0299	P12860	protein_cc	9230106F	ATP-bindi	Abcb5
4867	Itgb8	1.000	1.000	1.000	1.000	1.000	1.000	0.000	0.000	0.000	320910	Itgb8	chr12	-	NM_1772	P17351	protein_cc	4832412O	integrin b	Itgb8
4868	Macc1	1.000	1.000	1.000	1.000	1.000	1.000	0.000	0.000	0.000	238455	Macc1	chr12	+	NM_0011	P4289	protein_cc	4732474O	metastasis	Macc1
4869	Tmem196	1.000	1.000	1.000	1.000	1.000	1.000	0.000	0.000	0.000	217951	Tmem196	chr12	+	NM_0012	P17637	protein_cc	Gm528	transmem	Tmem196
4870	Speer6-ps	1.000	1.000	1.000	1.000	1.000	1.000	0.000	0.000	0.000	73266	Speer6-ps	chr13	+	NR_00158	.	misc_RNA	1700039A	spermatog	Speer6-ps
4871	2810429I	1.000	1.000	1.000	1.000	1.000	1.000	0.000	0.000	0.000	76937	2810429I	chr13	+	NR_01552	.	lncRNA	AI256682	RIKEN cDN	2810429I
4872	1700024F	1.000	1.000	1.000	1.000	1.000	1.000	0.000	0.000	0.000	73270	1700024F	chr13	+	NR_04536	.	lncRNA	.	RIKEN cDN	1700024F
4873	Gdi2	0.793	1.083	0.859	0.003	0.001	0.118	11.171	10.836	10.952	14569	Gdi2	chr13	+	NM_0081	P13404	protein_cc	GDI-B| Gd	guanosine	Gdi2
4874	Fam208b	1.628	0.872	1.420	0.022	0.232	0.174	6.119	6.822	6.624	105203	Fam208b	chr13	-	NM_1340	P5507	protein_cc	AI645998	family wit	Fam208b
4875	Asb13	0.436	1.143	0.499	0.000	0.323	0.030	8.161	6.963	7.157	142688	Asb13	chr13	+	NM_0012	P21392	protein_cc	2210015B	ankyrin re	Asb13
4876	Calml3	0.544	1.776	0.966	0.000	0.000	0.694	0.906	0.027	0.856	70405	Calml3	chr13	-	NM_0274	P8763	protein_cc	2310068O	calmoduli	Calml3
4877	Calm4	1.000	1.000	1.000	1.000	1.000	1.000	0.000	0.000	0.000	80796	Calm4	chr13	-	NM_0200	P20141	protein_cc	2310037J	calmoduli	Calm4
4878	Calm5	1.000	1.000	1.000	1.000	1.000	1.000	0.000	0.000	0.000	494124	Calm5	chr13	-	NM_0010	P779	protein_cc	AY314008	calmoduli	Calm5
4879	Net1	1.018	0.791	0.805	0.917	0.250	0.428	8.556	8.582	8.243	56349	Net1	chr13	-	NM_0196	P6490	protein_cc	mNET1| N	neuroepit	Net1
4880	Tubal3	1.000	1.000	1.000	1.000	1.000	1.000	0.000	0.000	0.000	238463	Tubal3	chr13	+	NM_0010	P342	protein_cc	.	tubulin, al	Tubal3
4881	Ucn3	1.000	1.000	1.000	1.000	1.000	1.000	0.000	0.000	0.000	83428	Ucn3	chr13	-	NM_0312	P9072	protein_cc	.	urocortin	Ucn3
4882	Akr1c14	0.045	0.931	0.042	0.000	0.854	0.011	9.772	5.291	5.188	105387	Akr1c14	chr13	+	NM_1340	P10807	protein_cc	9030611N	aldo-keto	Akr1c14
4883	Akr1c18	1.722	0.357	0.615	0.241	0.093	0.251	2.018	2.803	1.316	105349	Akr1c18	chr13	-	NM_1340	P21529	protein_cc	AW14604	aldo-keto	Akr1c18
4884	Akr1c13	0.425	1.154	0.491	0.000	0.236	0.012	10.391	9.157	9.363	27384	Akr1c13	chr13	+	NM_0137	P4099	protein_cc	mKIAA401	aldo-keto	Akr1c13
4885	Akr1c19	0.140	1.238	0.174	0.000	0.381	0.007	9.069	6.236	6.544	432720	Akr1c19	chr13	+	NM_0010	P18325	protein_cc	1810010N	aldo-keto	Akr1c19
4886	Marcksl1-	0.021	2.809	0.059	0.000	0.006	0.009	5.756	0.182	1.671	111002	Marcksl1-	chr13	-	NR_02840	.	misc_RNA	Gm16264	MARCKS-l	Marcksl1-
4887	Akr1c12	0.399	1.762	0.703	0.001	0.015	0.034	9.180	7.855	8.673	622402	Akr1c12	chr13	+	NM_0137	P7130	protein_cc	AI266976	aldo-keto	Akr1c12
4888	Akr1c6	0.320	0.621	0.199	0.000	0.233	0.029	13.468	11.824	11.135	83702	Akr1c6	chr13	+	NM_0306	P2927	protein_cc	3alpha-HS	aldo-keto	Akr1c6
4889	Akr1c20	0.540	0.369	0.199	0.011	0.015	0.045	7.714	6.826	5.388	116852	Akr1c20	chr13	-	NM_0540	P15154	protein_cc	2610528B	aldo-keto	Akr1c20
4890	Akr1c21	1.023	3.033	3.101	0.000	0.054	0.236	0.000	0.033	1.633	77337	Akr1c21	chr13	+	NM_0299	P6681	protein_cc	9430025F	aldo-keto	Akr1c21
4891	Akr1e1	0.361	1.100	0.397	0.001	0.612	0.050	8.782	7.312	7.449	56043	Akr1e1	chr13	-	NM_0188	P16998	protein_cc	1810061I1	aldo-keto	Akr1e1
4892	1700016G	1.000	1.000	1.000	1.000	1.000	1.000	0.000	0.000	0.000	71867	1700016G	chr13	-	NR_04589	.	lncRNA	.	RIKEN cDN	1700016G
4893	Klf6	4.379	0.575	2.517	0.008	0.092	0.204	8.011	10.142	9.343	23849	Klf6	chr13	+	NM_0118	P20373	protein_cc	AI448727	Kruppel-li	Klf6
4894	Pitrm1	0.962	0.693	0.667	0.822	0.071	0.271	7.333	7.277	6.748	69617	Pitrm1	chr13	+	NM_1451	P8329	protein_cc	2310012C	pitrilysin r	Pitrm1
4895	Pfkp	1.509	0.569	0.859	0.013	0.001	0.446	7.207	7.800	6.988	56421	Pfkp	chr13	-	NM_0197	P5175	protein_cc	1200015H	phosphofr	Pfkp
4896	Adarb2	0.669	0.995	0.666	0.181	1.000	0.423	0.587	0.008	0.000	94191	Adarb2	chr13	+	NM_0012	P2509	protein_cc	Adar3| RE	adenosine	Adarb2
4897	Wdr37	1.751	1.127	1.973	0.009	0.491	0.216	6.627	7.435	7.607	207615	Wdr37	chr13	-	NM_0010	P15250	protein_cc	.	WD repea	Wdr37
4898	Idi1	1.366	2.170	2.965	0.430	0.212	0.353	8.732	9.182	10.300	319554	Idi1	chr13	+	NM_1453	P6817	protein_cc	4832416K	isopenten	Idi1
4899	Idi2	1.000	1.000	1.000	1.000	1.000	1.000	0.000	0.000	0.000	320581	Idi2	chr13	+	NM_1771	P14887	protein_cc	4833405L	isopenten	Idi2
4900	Gtpbp4	1.575	0.664	1.046	0.025	0.037	0.777	8.756	9.411	8.821	69237	Gtpbp4	chr13	-	NM_0270	P25063	protein_cc	2610028C	GTP bindi	Gtpbp4
4901	Larp4b	0.632	1.083	0.685	0.000	0.221	0.028	9.360	8.698	8.813	217980	Larp4b	chr13	+	NM_1725	P4844	protein_cc	A630096F	La ribonuc	Larp4b

4902	Dip2c	0.572	1.091	0.625	0.013	0.451	0.145	6.517	5.712	5.838	208440	Dip2c	chr13	+	NM_0010	P6832	protein_c	2900024P	disco inte	Dip2c
4903	Zmynd11	0.825	0.896	0.740	0.002	0.019	0.017	9.578	9.300	9.142	66505	Zmynd11	chr13	-	NM_1445	P22058	protein_c	2210402G	zinc finge	Zmynd11
4904	Chrm3	0.914	1.442	1.318	0.836	0.496	0.748	0.817	0.687	1.216	12671	Chrm3	chr13	-	NM_0332	P26583	protein_c	Chrm-3 | N	cholinergi	Chrm3
4905	Zp4-ps	1.000	1.000	1.000	1.000	1.000	1.000	0.000	0.000	0.000	664793	Zp4-ps	chr13	+	NR_02781	.	misc_RNA	EG664793	zona pellu	Zp4-ps
4906	Ryr2	1.005	1.405	1.412	0.000	0.182	0.423	0.000	0.008	0.498	20191	Ryr2	chr13	-	NM_0238	P500	protein_c	9330127I2	ryanodine	Ryr2
4907	Gm10336	0.949	0.815	0.774	0.914	0.614	0.782	4.380	4.305	4.010	328186	Gm10336	chr13	-	NR_04517	.	lncRNA	B330003H	predicted	Gm10336
4908	Mtr	0.742	1.071	0.794	0.022	0.529	0.114	5.825	5.394	5.493	238505	Mtr	chr13	-	NM_0010	P14073	protein_c	AI894170 |	5-methylt	Mtr
4909	Actn2	1.000	1.000	1.000	1.000	1.000	1.000	0.000	0.000	0.000	11472	Actn2	chr13	-	NM_0332	P6302	protein_c	1110008F	actinin alp	Actn2
4910	Heatr1	10.501	0.291	3.058	0.000	0.001	0.171	6.914	10.307	8.527	217995	Heatr1	chr13	+	NM_1448	P2970	protein_c	AA517551	HEAT repe	Heatr1
4911	Lgals8	1.916	0.764	1.464	0.002	0.040	0.089	10.195	11.133	10.745	56048	Lgals8	chr13	-	NM_0012	P8855	protein_c	1200015E	lectin, gal	Lgals8
4912	Edaradd	5.023	0.988	4.964	0.070	0.986	0.455	0.589	2.917	2.900	171211	Edaradd	chr13	-	NM_1336	P13174	protein_c	1810032E	EDAR (ect	Edaradd
4913	Ero1lb	0.747	1.021	0.762	0.132	0.928	0.255	7.256	6.835	6.865	67475	Ero1lb	chr13	+	NM_0261	P15400	protein_c	1300013B	ERO1-like	Ero1lb
4914	Gpr137b-p	2.570	1.188	3.054	0.000	0.541	0.220	2.697	4.059	4.308	664862	Gpr137b-p	chr13	-	NR_00356	.	misc_RNA	2310041G	G protein-	Gpr137b-p
4915	Prl2c4	1.000	1.000	1.000	1.000	1.000	1.000	0.000	0.000	0.000	.	Prl2c4	chr13	-	NM_0119	P16650	.	.	.	Prl2c4
4916	Prl2c3	1.000	1.000	1.000	1.000	1.000	1.000	0.000	0.000	0.000	18812	Prl2c3	chr13	-	NM_0111	P16650	protein_c	Ghd1 | MF	prolactin f	Prl2c3
4917	Prl2c2	1.000	1.000	1.000	1.000	1.000	1.000	0.000	0.000	0.000	18811	Prl2c2	chr13	-	NM_0311	P7741	protein_c	MRP-1 | Pl	prolactin f	Prl2c2
4918	Prl2c5	0.716	0.995	0.712	0.182	1.000	0.423	0.490	0.008	0.000	107849	Prl2c5	chr13	+	NM_1818	P7751	protein_c	Ghd3 | MF	prolactin f	Prl2c5
4919	Gpr137b	1.026	0.811	0.832	0.953	0.715	0.775	3.908	3.945	3.642	83924	Gpr137b	chr13	-	NM_0319	P12887	protein_c	AI788917 |	G protein-	Gpr137b
4920	Nid1	2.735	1.266	3.464	0.006	0.088	0.004	6.596	8.047	8.388	18073	Nid1	chr13	+	NM_0109	P9558	protein_c	A630025C	nidogen 1	Nid1
4921	Lyst	0.359	1.350	0.485	0.047	0.624	0.307	5.639	4.162	4.594	17101	Lyst	chr13	+	NM_0107	P8073	protein_c	beige | bg	lysosomal	Lyst
4922	Gng4	1.000	1.000	1.000	1.000	1.000	1.000	0.000	0.000	0.000	14706	Gng4	chr13	-	NM_0013	P20407	protein_c	.	guanine n	Gng4
4923	B3galnt2	1.267	0.637	0.807	0.001	0.001	0.063	7.337	7.679	7.028	97884	B3galnt2	chr13	+	NM_1786	P3080	protein_c	A930105D	UDP-GalN	B3galnt2
4924	Tbce	1.080	1.012	1.093	0.435	0.929	0.729	6.568	6.679	6.696	70430	Tbce	chr13	-	NR_13078	.	protein_c	2610206D	tubulin-sp	Tbce
4925	Ggps1	2.160	0.900	1.944	0.109	0.739	0.119	5.714	6.826	6.674	14593	Ggps1	chr13	-	NM_0102	P25175	protein_c	1810026C	geranylge	Ggps1
4926	Arid4b	1.459	0.565	0.825	0.020	0.005	0.150	9.578	10.122	9.300	94246	Arid4b	chr13	+	NM_1981	P14151	protein_c	BCAA | BR	AT rich int	Arid4b
4927	Hecw1	0.673	1.387	0.933	0.185	0.187	0.889	0.587	0.015	0.486	94253	Hecw1	chr13	-	NM_0010	P26274	protein_c	9330116H	HECT, C2 a	Hecw1
4928	Mrpl32	1.514	0.786	1.190	0.001	0.003	0.192	7.824	8.422	8.075	75398	Mrpl32	chr13	-	NM_0292	P10427	protein_c	0610033O	mitochon	Mrpl32
4929	Psma2	1.660	1.004	1.667	0.000	0.960	0.091	11.229	11.960	11.966	19166	Psma2	chr13	+	NM_0089	P9281	protein_c	Lmpc3	proteason	Psma2
4930	AW20949	1.481	1.645	2.437	0.023	0.001	0.027	7.191	7.758	8.476	105351	AW20949	chr13	+	NM_0011	P19827	protein_c	.	expressed	AW209491
4931	Gli3	1.170	1.071	1.253	0.158	0.431	0.121	2.710	2.937	3.036	14634	Gli3	chr13	-	NM_0081	P10595	protein_c	add | AI85	GLI-Krupp	Gli3
4932	4933412O	2.170	0.461	1.000	0.000	1.000	0.423	0.000	1.118	0.000	71071	4933412O	chr13	-	NR_04550	.	lncRNA		RIKEN cDN	4933412O
4933	A530046N	3.300	0.303	1.000	0.306	1.000	0.423	0.001	1.723	0.000	328190	A530046N	chr13	-	NR_04613	.	lncRNA	A530046N	RIKEN cDN	A530046N
4934	Inhba	0.992	1.249	1.238	0.982	0.562	0.736	5.164	5.152	5.472	16323	Inhba	chr13	+	NM_0083	P12007	protein_c	.	inhibin be	Inhba
4935	Sugct	0.486	0.868	0.422	0.021	0.522	0.128	7.503	6.462	6.258	192136	Sugct	chr13	-	NM_1386	P6857	protein_c	5033411D	succinyl-C	Sugct
4936	Mplkip	0.736	0.840	0.618	0.054	0.040	0.135	7.103	6.661	6.408	66308	Mplkip	chr13	+	NM_0254	P10394	protein_c	2810021B	M-phase s	Mplkip
4937	Cdk13	1.063	0.832	0.884	0.400	0.056	0.431	7.703	7.791	7.524	69562	Cdk13	chr13	-	NM_0010	P3618	protein_c	2310015O	cyclin-dep	Cdk13
4938	Rala	1.455	1.001	1.457	0.004	0.978	0.016	9.790	10.331	10.333	56044	Rala	chr13	-	NM_0194	P12386	protein_c	3010001O	v-ral simia	Rala
4939	Yae1d1	1.137	1.079	1.227	0.151	0.575	0.448	6.213	6.398	6.508	67008	Yae1d1	chr13	-	NM_0259	P25405	protein_c	1600012F	Yae1 dom	Yae1d1
4940	4930448F	1.000	1.000	1.000	1.000	1.000	1.000	0.000	0.000	0.000	73992	4930448F	chr13	-	NR_04603	.	lncRNA		RIKEN cDN	4930448F1
4941	Pou6f2	0.407	1.356	0.552	0.182	0.196	0.545	1.325	0.028	0.467	218030	Pou6f2	chr13	-	NM_1750	P10929	protein_c	D130006K	POU doma	Pou6f2
4942	Vps41	0.715	1.156	0.827	0.011	0.303	0.442	8.016	7.533	7.742	218035	Vps41	chr13	+	NM_1721	P15013	protein_c	AI317346 |	VPS41 HO	Vps41
4943	Amph	1.000	1.000	1.000	1.000	1.000	1.000	0.000	0.000	0.000	218038	Amph	chr13	+	NM_0012	P22577	protein_c	.	amphiphy	Amph
4944	Stard3nl	1.019	0.907	0.924	0.951	0.737	0.751	6.437	6.463	6.322	76205	Stard3nl	chr13	-	NM_0242	P13789	protein_c	0610035N	STARD3 N	Stard3nl
4945	9330199G	1.000	1.000	1.000	1.000	1.000	1.000	0.000	0.000	0.000	319466	9330199G	chr13	-	NR_13103	.	lncRNA		RIKEN cDN	9330199G
4946	Epdr1	0.442	1.161	0.513	0.113	0.788	0.188	2.692	1.514	1.730	105298	Epdr1	chr13	-	NM_1340	P22497	protein_c	AU04095C	ependymi	Epdr1
4947	Sfrp4	1.000	1.000	1.000	1.000	1.000	1.000	0.000	0.000	0.000	20379	Sfrp4	chr13	+	NM_0166	P9303	protein_c	.	secreted f	Sfrp4
4948	Nme8	0.716	0.995	0.712	0.182	1.000	0.423	0.490	0.008	0.000	73412	Nme8	chr13	-	NM_0011	P7117	protein_c	1700056P	NME/NM2	Nme8
4949	A530099J	0.420	0.618	0.260	0.190	1.000	0.319	1.945	0.694	0.000	319293	A530099J	chr13	-	NM_1756	P5947	protein_c	.	RIKEN cDN	A530099J1
4950	Gpr141	23.706	1.079	25.589	0.000	0.290	0.001	0.695	5.262	5.373	353346	Gpr141	chr13	-	NM_1817	P9114	protein_c	PGR13	G protein-	Gpr141
4951	Elmo1	1.545	0.633	0.979	0.145	0.216	0.964	3.879	4.506	3.848	140580	Elmo1	chr13	+	NM_0802	P9601	protein_c	6330578D	engulfmer	Elmo1
4952	Aoah	12.761	0.717	9.153	0.000	0.089	0.083	1.441	5.115	4.636	27052	Aoah	chr13	+	NM_0012	P8399	protein_c	4930433E	acyloxyac	Aoah
4953	Olfr1370	1.000	1.000	1.000	1.000	1.000	1.000	0.000	0.000	0.000	258528	Olfr1370	chr13	-	NM_1465	P1910	protein_c	MOR256-1	olfactory	Olfr1370
4954	Olfr263	1.000	1.000	1.000	1.000	1.000	1.000	0.000	0.000	0.000	18341	Olfr263	chr13	+	NM_0109	P11031	protein_c	GA_x5J8B	olfactory	Olfr263
4955	Olfr1368	1.000	1.000	1.000	1.000	1.000	1.000	0.000	0.000	0.000	258527	Olfr1368	chr13	-	NM_1465	P4797	protein_c	MOR256-1	olfactory	Olfr1368
4956	Trim27	2.069	0.806	1.667	0.000	0.034	0.061	7.506	8.555	8.244	19720	Trim27	chr13	+	NM_0090	P18869	protein_c	AW53889	tripartite	Trim27
4957	Gpx5	1.000	1.000	1.000	1.000	1.000	1.000	0.000	0.000	0.000	14780	Gpx5	chr13	-	NM_0103	P1703	protein_c	Arep | AV3	glutathion	Gpx5
4958	Gpx6	0.220	1.293	0.284	0.114	0.570	0.363	3.366	1.182	1.553	75512	Gpx6	chr13	+	NM_1454	P25416	protein_c	1700020G	glutathion	Gpx6
4959	Olfr1367	1.000	1.000	1.000	1.000	1.000	1.000	0.000	0.000	0.000	258526	Olfr1367	chr13	+	NM_1465	P15000	protein_c	MOR256-1	olfactory	Olfr1367
4960	Zscan12	0.669	1.496	1.001	0.152	0.398	0.999	6.525	5.945	6.526	22758	Zscan12	chr13	+	NM_0166	P21884	protein_c	2510038J	zinc finge	Zscan12
4961	Zkscan3	0.617	0.989	0.610	0.034	0.944	0.130	7.924	7.227	7.211	72739	Zkscan3	chr13	-	NM_0236	P19333	protein_c	2810435N	zinc finge	Zkscan3
4962	Pgbd1	1.238	1.170	1.448	0.645	0.714	0.371	2.372	2.680	2.907	319207	Pgbd1	chr13	-	NM_0011	P14197	protein_c	4921509E	piggyBac t	Pgbd1
4963	Zscan26	1.037	0.667	0.692	0.466	0.001	0.085	6.072	6.125	5.542	432731	Zscan26	chr13	-	NM_0010	P24390	protein_c	BC068174	zinc finge	Zscan26
4964	Nkapl	2.025	0.480	0.971	0.010	0.004	0.933	4.266	5.284	4.224	66707	Nkapl	chr13	-	NM_0257	P15082	protein_c	4921504I	NFKB acti	Nkapl
4965	Zkscan4	0.287	1.442	0.413	0.080	0.515	0.302	3.591	1.788	2.316	544922	Zkscan4	chr13	+	NM_0010	P4683	protein_c	AI841875 |	zinc finge	Zkscan4
4966	Zfp389	3.033	0.795	2.411	0.152	0.704	0.486	0.392	1.993	1.662	10003837	Zfp389	chr13	-	NR_02679	.	misc_RNA	ENSMUSG	zinc finge	Zfp389
4967	Zkscan8	1.457	0.620	0.903	0.073	0.090	0.751	5.059	5.602	4.911	93681	Zkscan8	chr13	-	NM_1391	P3238	protein_c	2510038J	zinc finge	Zkscan8
4968	Olfr1366	1.586	0.463	0.734	0.473	1.000	0.422	0.446	1.111	0.000	258280	Olfr1366	chr13	-	NM_1462	P7540	protein_c	MOR130-2	olfactory	Olfr1366
4969	Olfr1535	1.000	1.000	1.000	1.000	1.000	1.000	0.000	0.000	0.000	404335	Olfr1535	chr13	-	NM_2075	P7439	protein_c	MOR256-1	olfactory	Olfr1535
4970	Olfr1364	1.000	1.000	1.000	1.000	1.000	1.000	0.000	0.000	0.000	258533	Olfr1364	chr13	-	NM_1465	P7748	protein_c	MOR256-1	olfactory	Olfr1364
4971	Olfr1362	1.000	1.000	1.000	1.000	1.000	1.000	0.000	0.000	0.000	258739	Olfr1362	chr13	-	NM_1467	P6178	protein_c	MOR256-8	olfactory	Olfr1362
4972	Olfr11	1.000	1.000	1.000	1.000	1.000	1.000	0.000	0.000	0.000	218066	Olfr11	chr13	-	NM_1465	P3442	protein_c	M49 | MO	olfactory	Olfr11
4973	Olfr1361	1.000	1.000	1.000	1.000	1.000	1.000	0.000	0.000	0.000	258534	Olfr1361	chr13	-	NM_1465	P24820	protein_c	MOR256-1	olfactory	Olfr1361
4974	Olfr1359	1.000	1.000	1.000	1.000	1.000	1.000	0.000	0.000	0.000	258067	Olfr1359	chr13	-	NM_0010	P19568	protein_c	MOR256-3	olfactory	Olfr1359
4975	Olfr1360	1.000	1.000	1.000	1.000	1.000	1.000	0.000	0.000	0.000	258536	Olfr1360	chr13	-	NM_1465	P19568	protein_c	MOR256-1	olfactory	Olfr1360
4976	Hist1h2bl	1.023	3.033	3.101	0.000	0.054	0.236	0.000	0.033	1.633	319185	Hist1h2bl	chr13	-	NM_1781	P376	protein_c	H2b-291a	histone cl	Hist1h2bl
4977	Hist1h2ai	1.910	1.488	2.841	0.129	0.184	0.086	2.395	3.328	3.901	319191	Hist1h2ai	chr13	+	NM_1781	P13133	protein_c	H2a-291A	histone cl	Hist1h2ai
4978	Hist1h3h	2.179	1.013	2.208	0.302	0.983	0.423	0.001	1.124	1.143	319152	Hist1h3h	chr13	-	NM_1782	P26208	protein_c	H3-291	histone cl	Hist1h3h
4979	Hist1h2bn	2.546	0.636	1.619	0.001	0.004	0.225	0.406	1.754	1.101	319186	Hist1h2bn	chr13	+	NM_1782	P10937	protein_c	H2b-291b	histone cl	Hist1h2bm
4980	Hist1h4j	3.307	0.735	2.432	0.032	0.455	0.388	1.909	3.635	3.191	319159	Hist1h4j	chr13	+	NM_1782	P9128	protein_c	.	histone cl	Hist1h4j
4981	Hist1h4k	8.638	0.607	5.241	0.091	0.469	0.410	0.589	3.700	2.979	319160	Hist1h4k	chr13	-	NM_1782	P16862	protein_c	.	histone cl	Hist1h4k
4982	Hist1h2ak	1.805	1.336	2.413	0.000	0.008	0.020	1.965	2.817	3.236	319169	Hist1h2ak	chr13	-	NM_1781	P12396	protein_c	.	histone cl	Hist1h2ak
4983	Hist1h2bn	2.160	0.636	1.373	0.304	0.480	0.423	0.000	1.111	0.458	319187	Hist1h2bn	chr13	+	NM_1782	P24666	protein_c	.	histone cl	Hist1h2bn
4984	Hist1h1b	0.353	3.108	1.097	0.060	0.312	0.933	3.298	1.795	3.431	56702	Hist1h1b	chr13	-	NM_0200	P8005	protein_c	H1.5 | H1E	histone cl	Hist1h1b
4985	Hist1h3i	1.484	3.005	4.459	0.528	0.286	0.460	0.588	1.157	2.745	319153	Hist1h3i	chr13	-	NM_1782	P21480	protein_c	H3-H	histone cl	Hist1h3i
4986	Hist1h2an	1.219	1.258	1.534	0.002	0.134	0.194	2.264	2.549	2.881	319170	Hist1h2an	chr13	-	NM_1781	P11313	protein_c	.	histone cl	Hist1h2an
4987	Hist1h2bp	1.652	1.388	2.294	0.446	0.466	0.113	0.416	1.140	1.614	319188	Hist1h2bp	chr13	-	NM_1782	P11400	protein_c	.	histone cl	Hist1h2bp
4988	Hist1h2bq	1.218	1.439	1.752	0.169	0.044	0.177	1.934	2.218	2.743	665596	Hist1h2bq	chr13	-	NM_0010	P5658	protein_c	Gm13646	histone cl	Hist1h2bq
4989	Hist1h2br	1.993	1.084	2.160	0.169	0.903	0.600	1.820	2.816	2.931	665622	Hist1h2br	chr13	-	NM_0011	P5658	protein_c	Gm11277	histone cl	Hist1h2br
4990	Hist1h2ao	1.514	1.200	1.816	0.140	0.419	0.139	5.447	6.045	6.308	665433	Hist1h2ao	chr13	+	NM_0011	P3322	protein_c	.	histone cl	Hist1h2ao
4991	Hist1h2ap	1.438	1.292	1.858	0.096	0.269	0.201	6.422	6.946	7.316	319171	Hist1h2ap	chr13	-	NM_1781	P3322	protein_c	ENSMUSG	histone cl	Hist1h2ap
4992	Hist1h4m	2.574	2.174	5.596	0.013	0.388	0.461	2.284	3.648	4.769	10004123	Hist1h4m	chr13	+	NM_0011	P26794	protein_c	Gm11275	histone cl	Hist1h4m

4993	Hist1h4n	3.325	1.919	6.381	0.000	0.452	0.465	3.144	4.878	5.818	319161	Hist1h4n	chr13	+	NM_1756	P26794	protein_cc	.	histone cl	Hist1h4n
4994	Zfp184	2.208	2.409	5.320	0.293	0.273	0.358	0.001	1.144	2.412	193452	Zfp184	chr13	+	NM_1830	P24247	protein_cc	4930500C	zinc finger	Zfp184
4995	Pom121l2	1.000	1.000	1.000	1.000	1.000	1.000	0.000	0.000	0.000	195236	Pom121l2	chr13	+	NM_0011	P25018	protein_cc	Gm24	POM121 r	Pom121l2
4996	Prss16	5.075	0.929	4.717	0.304	0.928	0.249	0.001	2.345	2.239	54373	Prss16	chr13	-	NM_0194	P966	protein_cc	AI448615	protease,	Prss16
4997	Hist1h2ah	0.794	1.312	1.042	0.511	0.402	0.936	3.380	3.047	3.439	319168	Hist1h2ah	chr13	-	NM_1756	P14916	protein_cc	.	histone cl	Hist1h2ah
4998	Hist1h2bk	4.570	0.993	4.538	0.020	0.988	0.330	0.001	2.193	2.183	319184	Hist1h2bk	chr13	+	NM_1756	P14265	protein_cc	.	histone cl	Hist1h2bk
4999	Hist1h4i	2.119	1.276	2.704	0.000	0.699	0.536	3.385	4.468	4.820	319158	Hist1h4i	chr13	-	NM_1756	P24741	protein_cc	.	histone cl	Hist1h4i
5000	Hist1h2ag	1.410	0.873	1.231	0.225	0.569	0.508	2.913	3.409	3.214	319167	Hist1h2ag	chr13	-	NM_1781	P10277	protein_cc	.	histone cl	Hist1h2ag
5001	Hist1h2bj	1.513	0.787	1.190	0.042	0.351	0.714	1.785	2.382	2.035	319183	Hist1h2bj	chr13	+	NM_1781	P26360	protein_cc	.	histone cl	Hist1h2bj
5002	Vmn1r188	1.000	1.000	1.000	1.000	1.000	1.000	0.000	0.000	0.000	252912	Vmn1r188	chr13	+	NM_1458	P10546	protein_cc	V1rh17	vomerona	Vmn1r188
5003	Vmn1r189	1.000	1.000	1.000	1.000	1.000	1.000	0.000	0.000	0.000	252906	Vmn1r189	chr13	-	NM_1458	P11529	protein_cc	V1rh2	vomerona	Vmn1r189
5004	Vmn1r191	1.000	1.000	1.000	1.000	1.000	1.000	0.000	0.000	0.000	632534	Vmn1r191	chr13	-	NM_1458	P6678	protein_cc	V1rh15	vomerona	Vmn1r191
5005	Vmn1r192	1.000	1.000	1.000	1.000	1.000	1.000	0.000	0.000	0.000	252907	Vmn1r192	chr13	-	NM_1458	P3936	protein_cc	V1ri1	vomerona	Vmn1r192
5006	Vmn1r193	1.000	1.000	1.000	1.000	1.000	1.000	0.000	0.000	0.000	171259	Vmn1r193	chr13	-	NM_1342	P15716	protein_cc	V1ri9	vomerona	Vmn1r193
5007	Vmn1r194	1.000	1.000	1.000	1.000	1.000	1.000	0.000	0.000	0.000	626299	Vmn1r194	chr13	+	NM_0010	P23728	protein_cc	Gm11294	vomerona	Vmn1r194
5008	Vmn1r195	1.000	1.000	1.000	1.000	1.000	1.000	0.000	0.000	0.000	171257	Vmn1r195	chr13	+	NM_1342	P5359	protein_cc	V1ri6	vomerona	Vmn1r195
5009	Vmn1r196	1.000	1.000	1.000	1.000	1.000	1.000	0.000	0.000	0.000	10031248	Vmn1r196	chr13	+	NM_0011	P22688	protein_cc	V1rh19	vomerona	Vmn1r196
5010	Vmn1r197	1.000	1.000	1.000	1.000	1.000	1.000	0.000	0.000	0.000	171278	Vmn1r197	chr13	+	NM_1342	P25450	protein_cc	V1rh21	vomerona	Vmn1r197
5011	Vmn1r198	1.000	1.000	1.000	1.000	1.000	1.000	0.000	0.000	0.000	171254	Vmn1r198	chr13	+	NM_1342	P23766	protein_cc	V1ri3	vomerona	Vmn1r198
5012	Vmn1r199	1.000	1.000	1.000	1.000	1.000	1.000	0.000	0.000	0.000	171247	Vmn1r199	chr13	+	NM_1342	P3330	protein_cc	V1rh4	vomerona	Vmn1r199
5013	Vmn1r200	1.000	1.000	1.000	1.000	1.000	1.000	0.000	0.000	0.000	171246	Vmn1r200	chr13	+	NM_1342	P14850	protein_cc	V1rh3	vomerona	Vmn1r200
5014	Vmn1r-ps1	1.000	1.000	1.000	1.000	1.000	1.000	0.000	0.000	0.000	171245	Vmn1r-ps1	chr13	+	NM_1342	P9768	protein_cc	V1rh1	vomerona	Vmn1r-ps1
5015	Vmn1r201	1.000	1.000	1.000	1.000	1.000	1.000	0.000	0.000	0.000	171255	Vmn1r201	chr13	+	NM_1342	P9977	protein_cc	V1ri4	vomerona	Vmn1r201
5016	Vmn1r202	1.000	1.000	1.000	1.000	1.000	1.000	0.000	0.000	0.000	171258	Vmn1r202	chr13	-	NM_1342	P9162	protein_cc	V1ri7	vomerona	Vmn1r202
5017	Vmn1r203	1.000	1.000	1.000	1.000	1.000	1.000	0.000	0.000	0.000	171270	Vmn1r203	chr13	+	NM_1342	P3686	protein_cc	V1rh11	vomerona	Vmn1r203
5018	Vmn1r204	1.000	1.000	1.000	1.000	1.000	1.000	0.000	0.000	0.000	632793	Vmn1r204	chr13	+	NM_0010	P4012	protein_cc	Gm11301	vomerona	Vmn1r204
5019	Vmn1r205	1.000	1.000	1.000	1.000	1.000	1.000	0.000	0.000	0.000	171251	Vmn1r205	chr13	-	NM_1342	P26094	protein_cc	V1rh8	vomerona	Vmn1r205
5020	Vmn1r206	1.000	1.000	1.000	1.000	1.000	1.000	0.000	0.000	0.000	171250	Vmn1r206	chr13	-	NM_1342	P9362	protein_cc	V1rh7	vomerona	Vmn1r206
5021	Vmn1r207	1.000	1.000	1.000	1.000	1.000	1.000	0.000	0.000	0.000	432735	Vmn1r207	chr13	-	NM_0011	P9713	protein_cc	Gm11312	vomerona	Vmn1r207
5022	Vmn1r208	1.000	1.000	1.000	1.000	1.000	1.000	0.000	0.000	0.000	171252	Vmn1r208	chr13	-	NM_1342	P26606	protein_cc	V1rh9 | Vn	vomerona	Vmn1r208
5023	Vmn1r209	1.000	1.000	1.000	1.000	1.000	1.000	0.000	0.000	0.000	432736	Vmn1r209	chr13	-	NM_0010	P4431	protein_cc	Gm11315	vomerona	Vmn1r209
5024	Vmn1r210	1.000	1.000	1.000	1.000	1.000	1.000	0.000	0.000	0.000	171269	Vmn1r210	chr13	-	NM_1342	P12511	protein_cc	V1rh10	vomerona	Vmn1r210
5025	Vmn1r211	1.000	1.000	1.000	1.000	1.000	1.000	0.000	0.000	0.000	171277	Vmn1r211	chr13	-	NM_1342	P12196	protein_cc	V1rh20	vomerona	Vmn1r211
5026	Vmn1r212	1.000	1.000	1.000	1.000	1.000	1.000	0.000	0.000	0.000	171275	Vmn1r212	chr13	-	NM_1342	P14650	protein_cc	V1rh18	vomerona	Vmn1r212
5027	Vmn1r213	1.000	1.000	1.000	1.000	1.000	1.000	0.000	0.000	0.000	171249	Vmn1r213	chr13	+	NM_1342	P17944	protein_cc	V1rh6	vomerona	Vmn1r213
5028	Vmn1r214	1.000	1.000	1.000	1.000	1.000	1.000	0.000	0.000	0.000	171248	Vmn1r214	chr13	+	NM_1342	P16509	protein_cc	V1rh5	vomerona	Vmn1r214
5029	Vmn1r215	1.000	1.000	1.000	1.000	1.000	1.000	0.000	0.000	0.000	171253	Vmn1r215	chr13	+	NM_1342	P19234	protein_cc	V1ri2	vomerona	Vmn1r215
5030	Vmn1r216	1.000	1.000	1.000	1.000	1.000	1.000	0.000	0.000	0.000	171279	Vmn1r216	chr13	+	NM_1342	P1453	protein_cc	V1ri10	vomerona	Vmn1r216
5031	Vmn1r217	1.000	1.000	1.000	1.000	1.000	1.000	0.000	0.000	0.000	171273	Vmn1r217	chr13	+	NM_1342	P12751	protein_cc	V1rh14	vomerona	Vmn1r217
5032	Vmn1r218	1.000	1.000	1.000	1.000	1.000	1.000	0.000	0.000	0.000	171256	Vmn1r218	chr13	+	NM_1342	P26105	protein_cc	V1ri5	vomerona	Vmn1r218
5033	Vmn1r219	1.000	1.000	1.000	1.000	1.000	1.000	0.000	0.000	0.000	171272	Vmn1r219	chr13	+	NM_1342	P23988	protein_cc	V1rh13	vomerona	Vmn1r219
5034	Vmn1r220	1.000	1.000	1.000	1.000	1.000	1.000	0.000	0.000	0.000	171271	Vmn1r220	chr13	-	NM_1342	P1456	protein_cc	V1rh12	vomerona	Vmn1r220
5035	Vmn1r221	1.000	1.000	1.000	1.000	1.000	1.000	0.000	0.000	0.000	10031248	Vmn1r221	chr13	+	NM_0011	P11019	protein_cc	Gm11329	vomerona	Vmn1r221
5036	Vmn1r222	1.000	1.000	1.000	1.000	1.000	1.000	0.000	0.000	0.000	171274	Vmn1r222	chr13	-	NM_1342	P15429	protein_cc	V1rh16	vomerona	Vmn1r222
5037	Vmn1r223	1.000	1.000	1.000	1.000	1.000	1.000	0.000	0.000	0.000	10003651	Vmn1r223	chr13	+	NM_0010	P2218	protein_cc	Gm11330	vomerona	Vmn1r223
5038	4930557F	1.000	1.000	1.000	1.000	1.000	1.000	0.000	0.000	0.000	75322	4930557F	chr13	-	NR_12644	.	lncRNA	.	RIKEN cDN	4930557F1
5039	4933404K	1.000	1.000	1.000	1.000	1.000	1.000	0.000	0.000	0.000	74061	4933404K	chr13	+	NR_04603	.	lncRNA	.	RIKEN cDN	4933404K(
5040	4930586N	0.943	1.015	0.957	0.841	0.965	0.895	3.496	3.411	3.433	75887	4930586N	chr13	+	NR_13105	.	lncRNA	.	RIKEN cDN	4930586N
5041	Zfp322a	1.510	0.871	1.315	0.009	0.325	0.317	7.634	8.229	8.029	218100	Zfp322a	chr13	-	NM_0011	P1377	protein_cc	Znf322| Zı	zinc finger	Zfp322a
5042	Abt1	1.175	1.188	1.397	0.733	0.706	0.469	6.908	7.141	7.390	30946	Abt1	chr13	-	NM_0139	P11214	protein_cc	.	activator	Abt1
5043	C230035I1	0.712	1.159	0.824	0.281	0.744	0.703	1.179	0.687	0.900	320842	C230035I1	chr13	-	NR_01549	.	lncRNA	.	RIKEN cDN	C230035I1
5044	Btn1a1	1.580	0.633	1.000	0.305	1.000	0.423	0.000	0.660	0.000	12231	Btn1a1	chr13	-	NM_0134	P1802	protein_cc	Btn	butyrophi	Btn1a1
5045	Btn2a2	1.000	1.000	1.000	1.000	1.000	1.000	0.000	0.000	0.000	238555	Btn2a2	chr13	-	NM_1759	P19024	protein_cc	Btn2| D03	butyrophi	Btn2a2
5046	Hist1h4h	1.182	0.871	1.029	0.160	0.465	0.936	5.485	5.726	5.527	69386	Hist1h4h	chr13	+	NM_1531	P20603	protein_cc	1700024H	histone cl	Hist1h4h
5047	Hist1h2af	0.796	1.422	1.133	0.291	0.054	0.638	2.354	2.026	2.534	319173	Hist1h2af	chr13	+	NM_1756	P13041	protein_cc	H2a-221	histone cl	Hist1h2af
5048	Hist1h3g	0.669	0.995	0.666	0.181	1.000	0.423	0.587	0.008	0.000	97908	Hist1h3g	chr13	+	NM_1450	P18074	protein_cc	H3.1-221|	histone cl	Hist1h3g
5049	Hist1h2bh	0.630	2.817	1.773	0.200	0.175	0.559	1.387	0.720	2.214	319182	Hist1h2bh	chr13	-	NM_1781	P14376	protein_cc	H2b-221	histone cl	Hist1h2bh
5050	Hist1h3f	2.720	0.651	1.771	0.303	0.569	0.423	0.001	1.444	0.825	260423	Hist1h3f	chr13	-	NM_0135	P11810	protein_cc	H3.2-221|	histone cl	Hist1h3f
5051	Hist1h4f	1.200	1.650	1.980	0.488	0.524	0.653	2.678	2.941	3.663	319157	Hist1h4f	chr13	-	NM_1756	P12537	protein_cc	.	histone cl	Hist1h4f
5052	Hist1h1d	1.365	2.253	3.074	0.522	0.393	0.536	3.205	3.654	4.826	14957	Hist1h1d	chr13	+	NM_1457	P12110	protein_cc	H1.3| H1D	histone cl	Hist1h1d
5053	Hist1h3e	2.391	0.948	2.266	0.003	0.917	0.556	1.120	2.377	2.300	319151	Hist1h3e	chr13	-	NM_1782	P544	protein_cc	H3-f| H3.1	histone cl	Hist1h3e
5054	Hist1h2ae	1.709	1.033	1.765	0.003	0.684	0.064	3.000	3.773	3.819	319166	Hist1h2ae	chr13	-	NM_1781	P17831	protein_cc	1190022L(	histone cl	Hist1h2ae
5055	Hist1h2bg	1.128	2.030	2.289	0.882	0.334	0.434	2.026	2.200	3.221	319181	Hist1h2bg	chr13	+	NM_1781	P26117	protein_cc	.	histone cl	Hist1h2bg
5056	Hist1h2bf	0.563	3.767	2.119	0.001	0.017	0.228	0.882	0.052	1.966	319180	Hist1h2bf	chr13	-	NM_1781	P8126	protein_cc	.	histone cl	Hist1h2bf
5057	Hist1h2ad	0.984	0.970	0.954	0.974	0.944	0.927	3.164	3.140	3.096	319165	Hist1h2ad	chr13	+	NM_1781	P23842	protein_cc	.	histone cl	Hist1h2ad
5058	Hist1h3d	0.552	0.990	0.547	0.182	1.000	0.423	0.871	0.015	0.000	319149	Hist1h3d	chr13	+	NM_1782	P25142	protein_cc	H3-b	histone cl	Hist1h3d
5059	Hist1h4d	2.379	1.466	3.487	0.028	0.535	0.448	3.139	4.390	4.941	319156	Hist1h4d	chr13	+	NM_1756	P23241	protein_cc	.	histone cl	Hist1h4d
5060	Hist1h2be	1.020	1.139	1.163	0.922	0.633	0.785	3.541	3.570	3.759	319179	Hist1h2be	chr13	-	NM_1781	P3993	protein_cc	.	histone cl	Hist1h2be
5061	Hist1h1e	0.622	0.502	0.312	0.009	0.033	0.074	7.327	6.643	5.648	50709	Hist1h1e	chr13	-	NM_0157	P381	protein_cc	H1-4| H1.4	histone cl	Hist1h1e
5062	Hist1h2ac	2.366	0.802	1.898	0.011	0.141	0.108	2.524	3.766	3.449	319164	Hist1h2ac	chr13	-	NM_1781	P26252	protein_cc	.	histone cl	Hist1h2ac
5063	Hist1h2bc	0.530	1.361	0.722	0.006	0.262	0.393	11.684	10.769	11.214	68024	Hist1h2bc	chr13	+	NM_0234	P3825	protein_cc	2610022J(	histone cl	Hist1h2bc
5064	Hist1h1t	1.000	1.000	1.000	1.000	1.000	1.000	0.000	0.000	0.000	107970	Hist1h1t	chr13	+	NM_0103	P1006	protein_cc	H1.6| H1ft	histone cl	Hist1h1t
5065	Hist1h4c	1.908	0.871	1.663	0.001	0.795	0.690	3.913	4.845	4.646	319155	Hist1h4c	chr13	-	NM_1782	P2180	protein_cc	.	histone cl	Hist1h4c
5066	Hfe	0.704	1.024	0.721	0.038	0.866	0.025	8.162	7.656	7.690	15216	Hfe	chr13	-	NM_0104	P25805	protein_cc	MR2	hemochro	Hfe
5067	Hist1h1c	0.467	2.985	1.393	0.080	0.318	0.801	4.607	3.508	5.085	50708	Hist1h1c	chr13	+	NM_0157	P4298	protein_cc	0610008C	histone cl	Hist1h1c
5068	Hist1h3c	1.445	1.012	1.462	0.551	0.984	0.646	0.588	1.119	1.136	319148	Hist1h3c	chr13	+	NM_1756	P17830	protein_cc	H3-143	histone cl	Hist1h3c
5069	Hist1h2bb	0.971	1.079	1.047	0.949	0.831	0.959	1.543	1.500	1.609	319178	Hist1h2bb	chr13	+	NM_1756	P17721	protein_cc	H2b-143	histone cl	Hist1h2bb
5070	Hist1h2ab	0.749	1.577	1.182	0.183	0.060	0.220	3.365	2.949	3.606	319172	Hist1h2ab	chr13	-	NM_1756	P9991	protein_cc	H2a-53| H	histone cl	Hist1h2ab
5071	Hist1h3b	2.722	0.367	1.000	0.306	1.000	0.423	0.001	1.445	0.000	319150	Hist1h3b	chr13	+	NM_1782	P8358	protein_cc	H3-53	histone cl	Hist1h3b
5072	Hist1h4b	3.903	1.321	5.154	0.000	0.632	0.402	1.808	3.773	4.174	326620	Hist1h4b	chr13	+	NM_1781	P16440	protein_cc	.	histone cl	Hist1h4b
5073	Hist1h4a	11.604	0.668	7.755	0.073	0.523	0.340	1.998	5.534	4.953	326619	Hist1h4a	chr13	-	NM_1781	P2289	protein_cc	.	histone cl	Hist1h4a
5074	Hist1h3a	6.733	0.299	2.011	0.000	0.000	0.227	1.005	3.756	2.013	360198	Hist1h3a	chr13	-	NM_0135	P14861	protein_cc	.	histone cl	Hist1h3a
5075	Hist1h1a	14.057	0.395	5.548	0.011	0.102	0.423	0.002	3.815	2.474	80838	Hist1h1a	chr13	+	NM_0306	P188	protein_cc	H1.1| H1a	histone cl	Hist1h1a
5076	Trim38	1.023	3.072	3.142	0.000	0.003	0.057	0.000	0.033	1.652	214158	Trim38	chr13	+	NM_0010	P4707	protein_cc	Gm23	tripartite	Trim38
5077	Slc17a2	0.849	1.366	1.159	0.017	0.001	0.039	10.184	9.947	10.397	218103	Slc17a2	chr13	+	NM_1448	P21337	protein_cc	C730032N	solute car	Slc17a2
5078	Slc17a3	0.501	0.997	0.500	0.002	0.992	0.170	8.142	7.145	7.141	105355	Slc17a3	chr13	+	NM_0011	P20165	protein_cc	AW26172	solute car	Slc17a3
5079	Slc17a1	1.109	1.430	1.586	0.574	0.043	0.076	6.618	6.767	7.283	20504	Slc17a1	chr13	+	NR_12161	.	protein_cc	NAPI-1| N	solute car	Slc17a1
5080	Slc17a4	0.046	0.300	0.014	0.000	0.087	0.000	8.613	4.173	2.438	319848	Slc17a4	chr13	-	NM_1770	P16298	protein_cc	9130214H	solute car	Slc17a4
5081	Hist1h2ba	1.653	1.384	2.287	0.158	0.341	0.304	0.772	1.497	1.966	319177	Hist1h2ba	chr13	-	NM_1756	P15688	protein_cc	Th2b	histone cl	Hist1h2ba
5082	Hist1h2aa	1.159	0.870	1.008	0.708	0.723	0.985	0.455	0.668	0.467	319163	Hist1h2aa	chr13	+	NM_1756	P12879	protein_cc	Th2a	histone cl	Hist1h2aa
5083	Scgn	1.000	1.000	1.000	1.000	1.000	1.000	0.000	0.000	0.000	214189	Scgn	chr13	-	NM_1453	P6544	protein_cc	.	secretago	Scgn

5084	Lrrc16a	2.091	1.836	3.838	0.141	0.037	0.044	3.951	5.015	5.891	68732	Lrrc16a	chr13	-	NM_0268	P4054	protein_cc	1110037D	leucine ric	Lrrc16a
5085	Cmah	0.736	0.794	0.584	0.052	0.314	0.257	8.328	7.886	7.553	12763	Cmah	chr13	+	NM_0011	P24096	protein_cc	.	cytidine m	Cmah
5086	Fam65b	5.049	0.754	3.809	0.000	0.017	0.045	3.684	6.020	5.614	193385	Fam65b	chr13	+	NM_0012	P1369	protein_cc	.	family wit	Fam65b
5087	Gm11346	1.000	1.000	1.000	1.000	1.000	1.000	0.000	0.000	0.000	76024	Gm11346	chr13	-	NR_02459	.	misc_RNA	5830431A	predicted	Gm11346
5088	Gmnn	0.149	1.742	0.260	0.001	0.032	0.052	6.874	4.129	4.929	57441	Gmnn	chr13	-	NM_0205	P3412	protein_cc	AW54634	geminin	Gmnn
5089	BC005537	0.756	1.024	0.774	0.069	0.898	0.266	7.214	6.811	6.845	79555	BC005537	chr13	+	NM_0244	P14306	protein_cc	8030460C	cDNA seq	BC005537
5090	Acot13	0.254	1.145	0.291	0.000	0.274	0.005	11.453	9.475	9.670	66834	Acot13	chr13	-	NM_0257	P10117	protein_cc	0610006O	acyl-CoA t	Acot13
5091	Tdp2	0.388	1.443	0.559	0.000	0.032	0.058	7.080	5.713	6.242	56196	Tdp2	chr13	+	NM_0195	P4074	protein_cc	D13Ertd65	tyrosyl-DN	Tdp2
5092	D130043K	0.065	1.492	0.081	0.000	0.001	0.024	6.460	2.266	2.843	210108	D130043K	chr13	+	NM_0010	P19660	protein_cc	4930451E	RIKEN cDN	D130043K
5093	4932702P	0.444	1.228	0.546	0.013	0.281	0.116	8.352	7.181	7.477	73326	4932702P	chr13	+	NR_04518	.	lncRNA	1700047I1	RIKEN cDN	4932702P
5094	Aldh5a1	0.342	1.368	0.469	0.000	0.101	0.004	9.284	7.738	8.190	214579	Aldh5a1	chr13	-	NM_1725	P7289	protein_cc	6330403E	aldhehyde	Aldh5a1
5095	93301620	0.188	2.287	0.431	0.003	0.162	0.168	4.469	2.060	3.254	493800	93301620	chr13	+	NR_10232	.	lncRNA	.	cDNA RIKE	93301620
5096	Gpld1	0.190	1.068	0.203	0.000	0.592	0.001	10.910	8.518	8.613	14756	Gpld1	chr13	+	NM_0081	P5639	protein_cc	6330541J1	glycosylph	Gpld1
5097	Mrs2	1.045	1.376	1.438	0.887	0.084	0.404	4.931	4.995	5.455	380836	Mrs2	chr13	-	NM_0010	P11046	protein_cc	Gm902 | H	MRS2 mag	Mrs2
5098	Dcdc2a	0.067	3.213	0.216	0.004	0.114	0.108	4.680	0.788	2.472	195208	Dcdc2a	chr13	+	NM_0011	P8337	protein_cc	AW49295	doublecor	Dcdc2a
5099	Nrsn1	1.000	1.000	1.000	1.000	1.000	1.000	0.000	0.000	0.000	22360	Nrsn1	chr13	-	NM_0095	P12053	protein_cc	Neurensin	neurensin	Nrsn1
5100	Gm11351	1.000	1.000	1.000	1.000	1.000	1.000	0.000	0.000	0.000	10050353	Gm11351	chr13	-	NR_04506	.	lncRNA	OTTMUSG	predicted	Gm11351
5101	1700092E	1.000	1.000	1.000	1.000	1.000	1.000	0.000	0.000	0.000	73576	1700092E	chr13	-	NR_04593	.	lncRNA	.	RIKEN cDN	1700092E
5102	Hdgfl1	1.000	1.000	1.000	1.000	1.000	1.000	0.000	0.000	0.000	15192	Hdgfl1	chr13	-	NM_0082	P10771	protein_cc	4921520N	hepatoma	Hdgfl1
5103	Prl	0.552	0.990	0.547	0.182	1.000	0.423	0.871	0.015	0.000	19109	Prl	chr13	+	NM_0110	P5135	protein_cc	AV290867	prolactin	Prl
5104	Prl3d1	1.000	1.000	1.000	1.000	1.000	1.000	0.000	0.000	0.000	18775	Prl3d1	chr13	+	NM_0088	P982	protein_cc	AI325057 |	prolactin f	Prl3d1
5105	Prl3d2	1.000	1.000	1.000	1.000	1.000	1.000	0.000	0.000	0.000	215028	Prl3d2	chr13	+	NM_1721	P26203	protein_cc	Ghd5 | PL-	prolactin f	Prl3d2
5106	Prl3d3	1.000	1.000	1.000	1.000	1.000	1.000	0.000	0.000	0.000	215029	Prl3d3	chr13	+	NM_1721	P1967	protein_cc	PL-Ig | Plig	prolactin f	Prl3d3
5107	Prl3c1	1.000	1.000	1.000	1.000	1.000	1.000	0.000	0.000	0.000	27372	Prl3c1	chr13	+	NM_0137	P5935	protein_cc	decidualin	prolactin f	Prl3c1
5108	Prl3b1	1.000	1.000	1.000	1.000	1.000	1.000	0.000	0.000	0.000	18776	Prl3b1	chr13	+	NM_0088	P4731	protein_cc	Csh2 | Ghc	prolactin f	Prl3b1
5109	Prl3a1	1.000	1.000	1.000	1.000	1.000	1.000	0.000	0.000	0.000	67000	Prl3a1	chr13	+	NM_0258	P22540	protein_cc	1600016E	prolactin f	Prl3a1
5110	Prl6a1	1.000	1.000	1.000	1.000	1.000	1.000	0.000	0.000	0.000	19111	Prl6a1	chr13	+	NM_0110	P21261	protein_cc	Ghd10 | Pl	prolactin f	Prl6a1
5111	Prl8a2	0.669	0.995	0.666	0.181	1.000	0.423	0.587	0.008	0.000	13529	Prl8a2	chr13	+	NM_0100	P25348	protein_cc	D/tPRP | D	prolactin f	Prl8a2
5112	Prl2b1	1.000	1.000	1.000	1.000	1.000	1.000	0.000	0.000	0.000	66392	Prl2b1	chr13	-	NM_0255	P24418	protein_cc	2310047B	prolactin f	Prl2b1
5113	Prl8a6	1.000	1.000	1.000	1.000	1.000	1.000	0.000	0.000	0.000	19112	Prl8a6	chr13	-	NM_0012	P17119	protein_cc	Ghd13 | Pl	prolactin f	Prl8a6
5114	Prl8a8	1.000	1.000	1.000	1.000	1.000	1.000	0.000	0.000	0.000	74188	Prl8a8	chr13	-	NM_0237	P2348	protein_cc	1600032B	prolactin f	Prl8a8
5115	Prl8a9	1.000	1.000	1.000	1.000	1.000	1.000	0.000	0.000	0.000	67310	Prl8a9	chr13	-	NM_0233	P14902	protein_cc	1600019F	prolactin f	Prl8a9
5116	Prl8a1	1.000	1.000	1.000	1.000	1.000	1.000	0.000	0.000	0.000	73244	Prl8a1	chr13	-	NM_0284	P11059	protein_cc	1600017L	prolactin f	Prl8a1
5117	Prl7b1	1.000	1.000	1.000	1.000	1.000	1.000	0.000	0.000	0.000	75596	Prl7b1	chr13	-	NM_0293	P12672	protein_cc	1600014J1	prolactin f	Prl7b1
5118	Prl7a1	1.000	1.000	1.000	1.000	1.000	1.000	0.000	0.000	0.000	19113	Prl7a1	chr13	-	NM_0110	P3628	protein_cc	AA407021	prolactin f	Prl7a1
5119	Prl7a2	1.000	1.000	1.000	1.000	1.000	1.000	0.000	0.000	0.000	19114	Prl7a2	chr13	-	NM_0110	P8790	protein_cc	Ghd18 | Pl	prolactin f	Prl7a2
5120	Prl7d1	1.000	1.000	1.000	1.000	1.000	1.000	0.000	0.000	0.000	18814	Prl7d1	chr13	-	NM_0111	P247	protein_cc	Ghd19 | Pl	prolactin f	Prl7d1
5121	Prl7c1	1.000	1.000	1.000	1.000	1.000	1.000	0.000	0.000	0.000	67505	Prl7c1	chr13	-	NM_0262	P23124	protein_cc	1600017N	prolactin f	Prl7c1
5122	Prl2a1	1.000	1.000	1.000	1.000	1.000	1.000	0.000	0.000	0.000	56635	Prl2a1	chr13	+	NM_0199	P4773	protein_cc	Ghd21 | Pl	prolactin f	Prl2a1
5123	Prl2c1	1.000	1.000	1.000	1.000	1.000	1.000	0.000	0.000	0.000	666317	Prl2c1	chr13	+	NM_0010	P17758	protein_cc	Ghd22	Prolactin f	Prl2c1
5124	Prl4a1	1.000	1.000	1.000	1.000	1.000	1.000	0.000	0.000	0.000	19110	Prl4a1	chr13	+	NM_0110	P7430	protein_cc	AA409771	prolactin f	Prl4a1
5125	Prl5a1	1.000	1.000	1.000	1.000	1.000	1.000	0.000	0.000	0.000	28078	Prl5a1	chr13	+	NM_0237	P9611	protein_cc	1600013P	prolactin f	Prl5a1
5126	2610307P	3.904	0.446	1.742	0.306	0.439	0.423	0.001	1.966	0.802	72518	2610307P	chr13	-	NR_04505	.	lncRNA	AI666457	RIKEN cDN	2610307P
5127	Sox4	3.193	0.833	2.660	0.000	0.475	0.276	5.828	7.503	7.240	20677	Sox4	chr13	-	NM_0092	P10035	protein_cc	AA682046	SRY (sex d	Sox4
5128	A330102I	1.589	1.141	1.813	0.298	0.765	0.423	0.000	0.669	0.859	77920	A330102I	chr13	+	NR_04507	.	lncRNA	.	RIKEN cDN	A330102I
5129	Cdkal1	0.880	1.067	0.939	0.144	0.387	0.638	7.275	7.091	7.185	68916	Cdkal1	chr13	-	NR_13189	.	protein_cc	1190005B	CDK5 regu	Cdkal1
5130	E2f3	1.633	1.016	1.659	0.093	0.929	0.055	5.376	6.084	6.107	13557	E2f3	chr13	-	NM_0012	P23533	protein_cc	E2f3a | E2I	E2F transc	E2f3
5131	Mboat1	3.434	0.936	3.216	0.064	0.870	0.251	2.172	3.952	3.857	218121	Mboat1	chr13	+	NM_1535	P4194	protein_cc	9130215M	membran	Mboat1
5132	Agtr1a	0.137	2.552	0.350	0.000	0.013	0.024	8.880	6.013	7.364	11607	Agtr1a	chr13	+	NM_1773	P7426	protein_cc	1810074K	angiotens	Agtr1a
5133	Uqcrfs1	0.514	1.193	0.613	0.000	0.026	0.017	11.713	10.752	11.007	66694	Uqcrfs1	chr13	-	NM_0257	P4492	protein_cc	4430402G	ubiquinol-	Uqcrfs1
5134	4930519D	1.000	1.000	1.000	1.000	1.000	1.000	0.000	0.000	0.000	75090	4930519D	chr13	+	NR_04597	.	lncRNA	.	RIKEN cDN	4930519D
5135	Dusp22	0.664	1.235	0.820	0.010	0.134	0.205	7.887	7.296	7.601	105352	Dusp22	chr13	+	NM_1340	P16487	protein_cc	JKAP | JSP	dual speci	Dusp22
5136	Irf4	6.858	0.373	2.555	0.115	0.221	0.238	0.001	2.779	1.355	16364	Irf4	chr13	+	NM_0136	P20161	protein_cc	AI385587 |	interferon	Irf4
5137	Exoc2	1.784	0.968	1.728	0.001	0.781	0.167	5.488	6.323	6.276	66482	Exoc2	chr13	-	NM_0255	P3957	protein_cc	2410030I2	exocyst cc	Exoc2
5138	Hus1b	0.716	0.995	0.712	0.182	1.000	0.423	0.490	0.008	0.000	210554	Hus1b	chr13	-	NM_1530	P21215	protein_cc	.	HUS1 chec	Hus1b
5139	4930401O	1.000	1.000	1.000	1.000	1.000	1.000	0.000	0.000	0.000	73924	4930401O	chr13	-	NR_04595	.	lncRNA	.	RIKEN cDN	4930401O
5140	Foxq1	0.024	4.882	0.116	0.000	0.025	0.010	10.502	5.102	7.389	15220	Foxq1	chr13	+	NM_0082	P19274	protein_cc	HFH-1 | Hf	forkhead b	Foxq1
5141	1700018A	1.000	1.000	1.000	1.000	1.000	1.000	0.000	0.000	0.000	71307	1700018A	chr13	-	NR_02943	.	lncRNA	4930551G	RIKEN cDN	1700018A
5142	Foxf2	1.000	1.000	1.000	1.000	1.000	1.000	0.000	0.000	0.000	14238	Foxf2	chr13	+	NM_0102	P22285	protein_cc	Fkh20 | FR	forkhead b	Foxf2
5143	Foxc1	3.378	0.894	3.019	0.010	0.465	0.147	1.649	3.405	3.243	17300	Foxc1	chr13	+	NM_0085	P19168	protein_cc	ch | fkh-1 |	forkhead b	Foxc1
5144	Gmds	2.953	1.302	3.844	0.039	0.394	0.217	3.457	5.019	5.400	218138	Gmds	chr13	-	NM_1460	P6965	protein_cc	BC031788	GDP-man	Gmds
5145	Mylk4	0.669	0.995	0.666	0.181	1.000	0.423	0.587	0.008	0.000	238564	Mylk4	chr13	-	NM_0011	P11687	protein_cc	EG238564	myosin lig	Mylk4
5146	Wrnip1	0.753	0.825	0.621	0.138	0.382	0.061	8.599	8.190	7.912	78903	Wrnip1	chr13	+	NM_0302	P4581	protein_cc	4833444L	Werner he	Wrnip1
5147	Serpinb1a	0.208	0.774	0.161	0.000	0.600	0.020	8.405	6.137	5.767	66222	Serpinb1a	chr13	-	NM_0254	P24544	protein_cc	1190005M	serine (or	Serpinb1a
5148	Serpinb1c	6.276	0.159	0.999	0.101	1.000	0.423	0.001	2.651	0.000	380839	Serpinb1c	chr13	-	NM_1730	P21813	protein_cc	EIC | ovalb	serine (or	Serpinb1c
5149	Serpinb6b	4.099	0.443	1.816	0.000	0.000	0.083	4.380	6.415	5.241	20708	Serpinb6b	chr13	+	NM_0114	P20905	protein_cc	NK13 | ova	serine (or	Serpinb6b
5150	Serpinb9	2.691	0.494	1.329	0.045	0.121	0.700	6.499	7.928	6.910	20723	Serpinb9	chr13	+	NM_0092	P16030	protein_cc	BB283241	serine (or	Serpinb9
5151	Serpinb9b	0.548	1.324	0.726	0.365	0.399	0.724	1.579	0.711	1.116	20706	Serpinb9b	chr13	+	NM_0114	P1166	protein_cc	1600019A	serine (or	Serpinb9b
5152	Serpinb1b	1.076	0.464	0.499	0.902	1.000	0.423	1.003	1.108	0.000	282663	Serpinb1b	chr13	+	NM_1730	P16661	protein_cc	6330533H	serine (or	Serpinb1b
5153	Serpinb9c	1.000	1.000	1.000	1.000	1.000	1.000	0.000	0.000	0.000	20707	Serpinb9c	chr13	-	NM_0114	P24768	protein_cc	3830421J	serine (or	Serpinb9c
5154	Serpinb9d	1.000	1.000	1.000	1.000	1.000	1.000	0.000	0.000	0.000	20726	Serpinb9d	chr13	+	NM_0114	P17540	protein_cc	AT2 | ovall	serine (or	Serpinb9d
5155	Serpinb9e	1.010	1.885	1.904	0.000	0.182	0.423	0.000	0.015	0.929	20710	Serpinb9e	chr13	+	NM_0114	P10959	protein_cc	AA540191	serine (or	Serpinb9e
5156	Serpinb9f	0.669	0.995	0.666	0.181	1.000	0.423	0.587	0.008	0.000	20709	Serpinb9f	chr13	+	NM_1831	P24003	protein_cc	BC052216	serine (or	Serpinb9f
5157	Serpinb9g	1.000	1.000	1.000	1.000	1.000	1.000	0.000	0.000	0.000	93806	Serpinb9g	chr13	+	NM_0114	P22708	protein_cc	1600002F	serine (or	Serpinb9g
5158	Gm6093	1.010	1.821	1.840	0.000	0.000	0.002	0.000	0.015	0.880	619715	Gm6093	chr13	-	NR_13114	.	lncRNA	EG619715	predicted	Gm6093
5159	Serpinb6d	1.000	1.000	1.000	1.000	1.000	1.000	0.000	0.000	0.000	238568	Serpinb6d	chr13	+	NM_0010	P26070	protein_cc	Gm11390	serine (or	Serpinb6d
5160	Serpinb6e	1.000	1.000	1.000	1.000	1.000	1.000	0.000	0.000	0.000	435350	Serpinb6e	chr13	-	NM_0010	P16225	protein_cc	Gm11396	serine (or	Serpinb6e
5161	Serpinb6c	1.000	1.000	1.000	1.000	1.000	1.000	0.000	0.000	0.000	97848	Serpinb6c	chr13	-	NM_1489	P5501	protein_cc	C76171 | C	serine (or	Serpinb6c
5162	Serpinb6a	0.546	1.775	0.969	0.079	0.059	0.876	7.622	6.749	7.577	20719	Serpinb6a	chr13	-	NM_0012	P15385	protein_cc	4930482L	serine (or	Serpinb6a
5163	1110046J	1.202	1.587	1.908	0.731	0.331	0.242	0.871	1.137	1.803	68808	1110046J	chr13	+	NR_04070	.	lncRNA	.	RIKEN cDN	1110046J
5164	Nqo2	0.178	0.726	0.129	0.000	0.126	0.002	10.378	7.890	7.427	18105	Nqo2	chr13	+	NM_0011	P5102	protein_cc	NMO2 | N	NAD(P)H d	Nqo2
5165	Ripk1	2.736	0.420	1.149	0.001	0.004	0.644	7.815	9.267	8.015	19766	Ripk1	chr13	+	NM_0090	P14989	protein_cc	D330015H	receptor (	Ripk1
5166	Bphl	0.302	0.879	0.266	0.002	0.525	0.045	10.287	8.560	8.374	68021	Bphl	chr13	+	NM_0265	P23619	protein_cc	2010012D	biphenyl h	Bphl
5167	Tubb2a	1.255	0.876	1.099	0.067	0.164	0.355	9.757	10.085	9.894	22151	Tubb2a	chr13	-	NM_0094	P6144	protein_cc	M(beta)2 |	tubulin, be	Tubb2a
5168	4930447K	1.000	1.000	1.000	1.000	1.000	1.000	0.000	0.000	0.000	432745	4930447K	chr13	-	NR_04618	.	lncRNA	.	RIKEN cDN	4930447K
5169	Tubb2b	1.208	0.804	0.971	0.132	0.247	0.937	4.872	5.145	4.830	73710	Tubb2b	chr13	-	NM_0237	P14369	protein_cc	2410129E	tubulin, be	Tubb2b
5170	Psmg4	1.866	1.016	1.896	0.000	0.887	0.110	7.793	8.693	8.716	69666	Psmg4	chr13	+	NM_0011	P18858	protein_cc	mPAC4 | P	proteason	Psmg4
5171	Slc22a23	0.154	1.361	0.209	0.000	0.187	0.019	9.037	6.335	6.780	73102	Slc22a23	chr13	-	NM_0010	P9192	protein_cc	3110004L	solute car	Slc22a23
5172	Pxdc1	0.300	1.304	0.391	0.000	0.152	0.018	9.183	7.446	7.830	66895	Pxdc1	chr13	-	NM_0258	P6972	protein_cc	1300014I0	PX domair	Pxdc1
5173	Gm15908	1.590	0.875	1.390	0.302	0.737	0.423	0.000	0.669	0.476	10105614	Gm15908	chr13	+	NR_10504	.	lncRNA	OTTMUSG	predicted	Gm15908
5174	Fam50b	0.457	1.401	0.640	0.183	0.194	0.600	1.158	0.027	0.514	108161	Fam50b	chr13	+	NM_1387	P23673	protein_cc	D0H6S265	family wit	Fam50b

5175	Prpf4b	1.295	0.802	1.039	0.002	0.073	0.844	8.876	9.249	8.932	19134	Prpf4b	chr13	+	NM_0138	P16263	protein_cc	2610037H	pre-mRNA	Prpf4b
5176	Fam217a	3.300	0.303	1.000	0.306	1.000	0.423	0.001	1.723	0.000	71864	Fam217a	chr13	-	NM_0279	P22416	protein_cc	1700026J0	family wit	Fam217a
5177	4933417A	1.000	1.000	1.000	1.000	1.000	1.000	0.000	0.000	0.000	66761	4933417A	chr13	+	NM_0257	P6811	protein_cc	.	RIKEN cDN	4933417A
5178	Eci3	0.445	1.870	0.832	0.002	0.000	0.298	6.666	5.497	6.401	69123	Eci3	chr13	-	NM_0269	P15736	protein_cc	1810022C	enoyl-Coe	Eci3
5179	Eci2	0.599	1.691	1.012	0.000	0.050	0.964	10.574	9.834	10.591	23986	Eci2	chr13	-	NR_07342	.	protein_cc	Acbd2| Dr	enoyl-Coe	Eci2
5180	1700011B	2.170	0.862	1.870	0.303	0.803	0.423	0.001	1.118	0.904	10050372	1700011B	chr13	+	NR_04561	.	lncRNA	.	RIKEN cDN	1700011B
5181	1700019C	1.000	1.000	1.000	1.000	1.000	1.000	0.000	0.000	0.000	75543	1700019C	chr13	-	NR_12647	.	lncRNA	.	RIKEN cDN	1700019C
5182	Cdyl	1.235	1.309	1.617	0.507	0.333	0.149	5.481	5.786	6.174	12593	Cdyl	chr13	+	NM_0098	P1020	protein_cc	AI325931	chromodo	Cdyl
5183	Rpp40	0.564	2.443	1.378	0.022	0.000	0.210	5.738	4.912	6.201	208366	Rpp40	chr13	-	NM_1459	P18946	protein_cc	D8Bwg12(	ribonuclea	Rpp40
5184	Ppp1r3g	0.009	0.208	0.002	0.000	0.020	0.000	9.721	2.973	0.707	76487	Ppp1r3g	chr13	+	NM_0296	P26723	protein_cc	1600032L	protein ph	Ppp1r3g
5185	Lyrm4	0.561	1.781	0.999	0.001	0.017	0.997	8.641	7.807	8.640	380840	Lyrm4	chr13	-	NM_2013	P14409	protein_cc	BC034664	LYR motif	Lyrm4
5186	Fars2	0.487	1.293	0.629	0.001	0.164	0.077	7.892	6.853	7.224	69955	Fars2	chr13	+	NM_0242	P2462	protein_cc	Fars1| phe	phenylala	Fars2
5187	Nrn1	0.032	1.089	0.035	0.000	0.650	0.023	11.325	6.373	6.496	68404	Nrn1	chr13	-	NM_1535	P6839	protein_cc	0710008J2	neuritin 1	Nrn1
5188	F13a1	4.219	1.404	5.924	0.219	0.481	0.039	2.074	4.150	4.640	74145	F13a1	chr13	-	NM_0287	P8048	protein_cc	1200014I0	coagulatic	F13a1
5189	Ly86	0.665	0.551	0.367	0.308	0.053	0.282	3.734	3.145	2.287	17084	Ly86	chr13	+	NM_0107	P16662	protein_cc	ly86_tv2|	lymphocyt	Ly86
5190	Rreb1	0.580	0.958	0.556	0.001	0.835	0.105	7.438	6.652	6.590	68750	Rreb1	chr13	+	NM_0268	P12618	protein_cc	1110037N	ras respor	Rreb1
5191	Ssr1	1.469	0.974	1.431	0.000	0.004	0.002	9.853	10.408	10.370	107513	Ssr1	chr13	-	NM_0259	P14304	protein_cc	2510001K	signal seq	Ssr1
5192	Cage1	0.299	2.233	0.667	0.024	0.192	0.537	2.488	0.745	1.904	71213	Cage1	chr13	-	NM_0277	P10678	protein_cc	4933427I0	cancer ant	Cage1
5193	Riok1	2.216	0.758	1.680	0.000	0.000	0.008	6.945	8.093	7.694	71340	Riok1	chr13	+	NM_0242	P25562	protein_cc	3110046C	RIO kinase	Riok1
5194	Dsp	0.224	2.603	0.583	0.000	0.003	0.029	8.895	6.736	8.116	109620	Dsp	chr13	+	NM_0238	P14334	protein_cc	2300002E	desmopla	Dsp
5195	Snrnp48	1.010	0.597	0.603	0.701	0.000	0.018	9.689	9.704	8.960	67797	Snrnp48	chr13	+	NM_0263	P23032	protein_cc	1110050F	small nucl	Snrnp48
5196	Bmp6	1.146	0.632	0.725	0.729	1.000	0.422	0.465	0.661	0.000	12161	Bmp6	chr13	+	NM_0075	P20231	protein_cc	D13Wsu1	bone mor	Bmp6
5197	4930579J1	1.000	1.000	1.000	1.000	1.000	1.000	0.000	0.000	0.000	75854	4930579J1	chr13	-	NR_13103	.	lncRNA	.	RIKEN cDN	4930579J1
5198	Txndc5	0.737	1.061	0.782	0.016	0.172	0.177	8.849	8.408	8.494	105245	Txndc5	chr13	-	NM_0012	P6725	protein_cc	AL022641	thioredox	Txndc5
5199	Bloc1s5	0.400	1.608	0.643	0.000	0.009	0.021	7.974	6.652	7.338	17828	Bloc1s5	chr13	-	NM_1390	P20035	protein_cc	1810074A	biogenesis	Bloc1s5
5200	Eef1e1	2.182	0.593	1.294	0.039	0.067	0.620	6.112	7.238	6.484	66143	Eef1e1	chr13	-	NM_0253	P14761	protein_cc	1110003A	eukaryoti	Eef1e1
5201	Slc35b3	2.158	1.040	2.245	0.001	0.870	0.274	6.269	7.379	7.435	108652	Slc35b3	chr13	-	NM_0011	P458	protein_cc	4921526O	solute car	Slc35b3
5202	5033403F	1.373	1.003	1.378	0.366	0.985	0.651	1.317	1.774	1.779	75958	5033403F	chr13	+	NR_13101	.	lncRNA	.	RIKEN cDN	5033403F
5203	Ofcc1	1.000	1.000	1.000	1.000	1.000	1.000	0.000	0.000	0.000	218165	Ofcc1	chr13	-	NM_1721	P24288	protein_cc	opo	orofacial	Ofcc1
5204	Tfap2a	1.238	0.456	0.565	0.421	1.000	0.422	0.824	1.132	0.000	21418	Tfap2a	chr13	-	NM_0013	P16394	protein_cc	AP-2| Ap-	transcripti	Tfap2a
5205	Gm26688	1.000	1.000	1.000	1.000	1.000	1.000	0.000	0.000	0.000	10263380	Gm26688	chr13	+	NR_12589	.	lncRNA	Tfap2a-as	predicted	Gm26688
5206	Gcnt2	5.367	0.285	1.531	0.000	0.001	0.111	8.055	10.479	8.669	14538	Gcnt2	chr13	+	NM_0238	P4796	protein_cc	5330430K	glucosami	Gcnt2
5207	Pak1ip1	0.870	0.779	0.677	0.029	0.023	0.034	9.266	9.065	8.704	68083	Pak1ip1	chr13	+	NM_0265	P24480	protein_cc	5830431I1	PAK1 inte	Pak1ip1
5208	Tmem14c	0.282	1.596	0.450	0.000	0.024	0.053	11.376	9.549	10.223	66154	Tmem14c	chr13	+	NM_0253	P22393	protein_cc	1110021D	transmem	Tmem14c
5209	Mak	1.005	1.439	1.446	0.000	0.182	0.423	0.000	0.008	0.532	17152	Mak	chr13	-	NM_0085	P20209	protein_cc	A930010C	male gern	Mak
5210	Gcm2	1.000	1.000	1.000	1.000	1.000	1.000	0.000	0.000	0.000	107889	Gcm2	chr13	-	NM_0081	P14599	protein_cc	Gcm-rs1|	glial cells	Gcm2
5211	Elovl2	0.185	1.004	0.186	0.000	0.979	0.004	12.067	9.631	9.637	54326	Elovl2	chr13	-	NM_0194	P23118	protein_cc	AI317360|	elongation	Elovl2
5212	Smim13	0.327	1.513	0.495	0.001	0.082	0.008	8.279	6.667	7.264	108934	Smim13	chr13	+	NM_0011	P7917	protein_cc	2900036K	small inte	Smim13
5213	Nedd9	1.520	1.975	3.002	0.088	0.002	0.035	5.570	6.174	7.156	18003	Nedd9	chr13	-	NM_0011	P20527	protein_cc	Cas-L| Cas	neural pre	Nedd9
5214	Tmem170	0.897	1.065	0.956	0.582	0.594	0.900	6.555	6.399	6.490	621976	Tmem170	chr13	+	NM_0011	P13991	protein_cc	EG621976	transmem	Tmem170
5215	Gm5082	1.572	0.636	1.000	0.304	1.000	0.423	0.000	0.653	0.000	328231	Gm5082	chr13	+	NM_1778	P7728	protein_cc	A030008J	predicted	Gm5082
5216	Adtrp	0.455	1.444	0.657	0.000	0.003	0.021	10.907	9.770	10.300	109254	Adtrp	chr13	-	NM_0011	P2092	protein_cc	9530008L	androgen	Adtrp
5217	Gm10790	1.000	1.000	1.000	1.000	1.000	1.000	0.000	0.000	0.000	10003857	Gm10790	chr13	+	NR_03354	.	lncRNA	ENSMUSG	predicted	Gm10790
5218	Hivep1	2.132	0.490	1.044	0.018	0.024	0.818	6.359	7.451	6.422	110521	Hivep1	chr13	+	NM_0077	P6326	protein_cc	Cryabp1	human im	Hivep1
5219	Edn1	11.302	0.462	5.225	0.004	0.053	0.199	2.969	6.468	5.355	13614	Edn1	chr13	+	NM_0101	P17631	protein_cc	ET-1| PPE	endotheli	Edn1
5220	Phactr1	0.371	2.543	0.942	0.049	0.071	0.832	4.768	3.336	4.682	218194	Phactr1	chr13	+	NM_0013	P15741	protein_cc	9630030F	phosphata	Phactr1
5221	Tbc1d7	0.540	1.653	0.893	0.003	0.006	0.560	5.963	5.075	5.800	67046	Tbc1d7	chr13	-	NM_0259	P24308	protein_cc	2610009C	TBC1 dom	Tbc1d7
5222	Gfod1	0.349	1.278	0.446	0.008	0.502	0.135	4.585	3.065	3.420	328232	Gfod1	chr13	-	NM_0010	P14510	protein_cc	9630032O	glucose-fr	Gfod1
5223	Sirt5	0.368	1.089	0.401	0.002	0.781	0.084	6.800	5.359	5.481	68346	Sirt5	chr13	+	NM_1788	P12823	protein_cc	0610012J0	sirtuin 5	Sirt5
5224	Nol7	1.045	1.098	1.148	0.555	0.445	0.544	9.673	9.736	9.871	70078	Nol7	chr13	+	NM_0235	P19951	protein_cc	2210008F	nucleolar	Nol7
5225	Ranbp9	1.403	1.039	1.458	0.050	0.819	0.277	7.051	7.540	7.595	56705	Ranbp9	chr13	-	NM_0199	P16772	protein_cc	IBAP-1| Ib	RAN bindi	Ranbp9
5226	Mcur1	1.188	1.015	1.206	0.010	0.648	0.098	9.092	9.340	9.362	76137	Mcur1	chr13	-	NM_0010	P10976	protein_cc	6230416A	mitochond	Mcur1
5227	Rnf182	0.716	0.995	0.712	0.182	1.000	0.423	0.490	0.008	0.000	328234	Rnf182	chr13	+	NM_1832	P15503	protein_cc	C630023L	ring finger	Rnf182
5228	Gm20751	1.000	1.000	1.000	1.000	1.000	1.000	0.000	0.000	0.000		Gm20751	chr13	+	NR_04602	.	lncRNA		predicted	Gm20751
5229	Cd83	5.244	0.053	0.277	0.000	0.000	0.224	4.213	6.603	2.363	12522	Cd83	chr13	+	NM_0012	P818	protein_cc	.	CD83 anti	Cd83
5230	Gm5083	1.000	1.000	1.000	1.000	1.000	1.000	0.000	0.000	0.000	328235	Gm5083	chr13	+	NR_04528	.	lncRNA	328235| A	predicted	Gm5083
5231	A330076C	1.005	1.405	1.412	0.000	0.182	0.423	0.000	0.008	0.498	10050359	A330076C	chr13	-	NR_04508	.	lncRNA		RIKEN cDN	A330076C
5232	1700029N	0.716	0.995	0.712	0.182	1.000	0.423	0.490	0.008	0.000	69491	1700029N	chr13	-	NR_04548	.	lncRNA		RIKEN cDN	1700029N
5233	Jarid2	0.735	1.085	0.798	0.028	0.492	0.129	8.159	7.715	7.833	16468	Jarid2	chr13	+	NM_0012	P24024	protein_cc	Jmj| jumo	jumonji, A	Jarid2
5234	Dtnbp1	0.720	1.459	1.050	0.004	0.009	0.664	8.681	8.207	8.751	94245	Dtnbp1	chr13	-	NM_0257	P7384	protein_cc	5430437B	dystrobre	Dtnbp1
5235	Mylip	0.444	0.846	0.376	0.001	0.162	0.012	7.042	5.870	5.629	218203	Mylip	chr13	+	NM_1537	P8635	protein_cc	9430057C	myosin re	Mylip
5236	Gmpr	0.297	0.990	0.294	0.080	0.991	0.218	4.321	2.568	2.554	66355	Gmpr	chr13	+	NM_0255	P21417	protein_cc	2310004P	guanosine	Gmpr
5237	Atxn1	1.232	0.314	0.387	0.126	0.002	0.005	8.010	8.311	6.641	20238	Atxn1	chr13	-	NM_0011	P7427	protein_cc	2900016G	ataxin 1	Atxn1
5238	Stmnd1	1.000	1.000	1.000	1.000	1.000	1.000	0.000	0.000	0.000	380842	Stmnd1	chr13	+	NM_0010	P10272	protein_cc	Gm1574	stathmin	Stmnd1
5239	Rbm24	1.590	0.901	1.432	0.302	0.794	0.423	0.000	0.669	0.519	666794	Rbm24	chr13	+	NM_0010	P9516	protein_cc	6330546B	RNA bindi	Rbm24
5240	Cap2	0.716	0.995	0.712	0.182	1.000	0.423	0.490	0.008	0.000	67252	Cap2	chr13	+	NM_0260	P21824	protein_cc	2810452G	CAP, aden	Cap2
5241	Fam8a1	1.173	1.092	1.281	0.386	0.512	0.525	4.072	4.303	4.430	97863	Fam8a1	chr13	+	NM_0010	P1717	protein_cc	C78339	family wit	Fam8a1
5242	Nup153	1.379	0.815	1.124	0.102	0.271	0.558	6.804	7.267	6.972	218210	Nup153	chr13	-	NM_1757	P7406	protein_cc	B130015D	nucleopor	Nup153
5243	Kif13a	0.808	1.102	0.890	0.113	0.000	0.550	7.192	6.884	7.024	16553	Kif13a	chr13	-	NM_0106	P8896	protein_cc	4930505I0	kinesin fa	Kif13a
5244	Nhlrc1	1.558	1.291	2.012	0.305	0.402	0.310	3.066	3.706	4.074	105193	Nhlrc1	chr13	-	NM_1753	P10158	protein_cc	AI505271|	NHL repea	Nhlrc1
5245	Tpmt	0.465	1.758	0.817	0.013	0.046	0.582	9.154	8.048	8.862	22017	Tpmt	chr13	-	NM_0167	P13306	protein_cc	AW10691	thiopurine	Tpmt
5246	Kdm1b	0.863	1.219	1.051	0.355	0.195	0.858	8.221	8.007	8.293	218214	Kdm1b	chr13	+	NM_1722	P19031	protein_cc	4632428N	lysine (K)-	Kdm1b
5247	Dek	0.920	1.254	1.154	0.412	0.056	0.263	10.487	10.367	10.694	110052	Dek	chr13	-	NM_0259	P22674	protein_cc	1810019E	DEK oncog	Dek
5248	Rnf144b	0.299	1.873	0.561	0.001	0.000	0.060	8.182	6.442	7.347	218215	Rnf144b	chr13	+	NM_1460	P25760	protein_cc	BC025007	ring finger	Rnf144b
5249	A930002C	0.165	0.599	0.099	0.011	1.000	0.091	3.336	0.740	0.000	319467	A930002C	chr13	-	NR_12608	.	lncRNA	.	RIKEN cDN	A930002C
5250	G630093K	1.000	1.000	1.000	1.000	1.000	1.000	0.000	0.000	0.000	619327	G630093K	chr13	+	NR_04515	.	lncRNA	.	RIKEN cDN	G630093K
5251	4931429P	1.000	1.000	1.000	1.000	1.000	1.000	0.000	0.000	0.000	70971	4931429P	chr13	-	NR_03800	.	lncRNA	.	RIKEN cDN	4931429P
5252	A330033J	1.000	1.000	1.000	1.000	1.000	1.000	0.000	0.000	0.000	320614	A330033J	chr13	-	NR_10230	.	lncRNA	.	RIKEN cDN	A330033J
5253	Id4	0.505	1.011	0.511	0.013	0.972	0.168	2.506	1.520	1.536	15904	Id4	chr13	+	NM_0311	P16207	protein_cc	bHLHb27|	inhibitor c	Id4
5254	A330048C	0.716	0.995	0.712	0.182	1.000	0.423	0.490	0.008	0.000	320315	A330048C	chr13	-	NR_04516	.	lncRNA		RIKEN cDN	A330048C
5255	Zfp169	1.983	0.271	0.538	0.355	0.198	0.090	2.402	3.390	1.509	67911	Zfp169	chr13	-	NM_0011	P25669	protein_cc	1700025J1	zinc finger	Zfp169
5256	Ptpdc1	3.327	0.306	1.017	0.004	0.006	0.970	2.640	4.374	2.664	218232	Ptpdc1	chr13	-	NM_2072	P19397	protein_cc	AI843923|	protein ty	Ptpdc1
5257	Barx1	1.250	0.808	1.010	0.712	0.696	0.984	0.809	1.130	0.822	12022	Barx1	chr13	+	NM_0075	P17219	protein_cc	.	BarH-like	Barx1
5258	Phf2	0.888	0.744	0.660	0.034	0.000	0.025	7.993	7.821	7.394	18676	Phf2	chr13	-	NM_0110	P22759	protein_cc	GRC5	PHD finge	Phf2
5259	Fam120a	0.483	1.418	0.686	0.027	0.157	0.228	11.227	10.178	10.682	218236	Fam120a	chr13	-	NM_0010	P26838	protein_cc	C9orf10| (	family wit	Fam120a
5260	Fam120ac	0.217	1.611	0.349	0.000	0.081	0.006	7.304	5.098	5.786	68128	Fam120ac	chr13	-	NR_01560	.	lncRNA	C030044B	family wit	Fam120ao
5261	Wnk2	0.399	1.335	0.533	0.134	0.521	0.403	2.485	1.160	1.576	75607	Wnk2	chr13	-	NM_0012	P7073	protein_cc	1810073P	WNK lysin	Wnk2
5262	Ninj1	1.225	0.889	1.089	0.186	0.338	0.515	10.461	10.754	10.584	18081	Ninj1	chr13	+	NM_0136	P7486	protein_cc	AU024536	ninjurin 1	Ninj1
5263	1110007C	1.301	0.718	0.934	0.194	0.072	0.774	7.043	7.422	6.944	.	1110007C	chr13	-	NM_0267	P8732	.	.	.	1110007C
5264	Susd3	0.674	0.529	0.357	0.541	0.087	0.510	3.813	3.245	2.325	66329	Susd3	chr13	-	NM_0283	P26677	protein_cc	1700017I1	sushi dom	Susd3
5265	Fgd3	1.770	0.365	0.645	0.000	0.002	0.274	1.976	2.800	1.344	30938	Fgd3	chr13	-	NM_0157	P15563	protein_cc	5830461L(	FYVE, Rho	Fgd3

5266	Bicd2	1.121	0.898	1.007	0.298	0.341	0.970	6.586	6.752	6.597	76895	Bicd2	chr13	+	NM_0010	P19986	protein_cc	. bicaudal	Bicd2
5267	Ippk	1.867	0.765	1.428	0.045	0.281	0.423	5.745	6.646	6.259	75678	Ippk	chr13	+	NR_07508	.	protein_cc	1810043M inositol 1,	Ippk
5268	Cenpp	0.441	0.965	0.425	0.152	0.963	0.303	2.953	1.770	1.718	66336	Cenpp	chr13	-	NM_0254	P20306	protein_cc	1700022C centromer	Cenpp
5269	Ecm2	0.228	0.476	0.108	0.004	0.393	0.004	3.665	1.531	0.460	407800	Ecm2	chr13	+	NM_0010	P20233	protein_cc	9030618O extracellu	Ecm2
5270	Aspn	0.132	3.240	0.428	0.000	0.099	0.152	3.017	0.096	1.791	66695	Aspn	chr13	+	NM_0257	P5412	protein_cc	4631401G asporin	Aspn
5271	Omd	1.311	1.895	2.483	0.329	0.093	0.248	2.860	3.251	4.172	27047	Omd	chr13	+	NM_0120	P17632	protein_cc	OSAD| SLI osteomod	Omd
5272	Ogn	0.330	1.041	0.343	0.098	0.286	0.326	3.839	2.238	2.295	18295	Ogn	chr13	+	NM_0087	P26499	protein_cc	3110079A osteoglyci	Ogn
5273	Nol8	2.069	0.653	1.351	0.010	0.055	0.296	7.636	8.684	8.070	70930	Nol8	chr13	+	NM_0012	P7435	protein_cc	4921532D nucleolar	Nol8
5274	Iars	1.282	0.900	1.154	0.130	0.341	0.401	7.510	7.868	7.717	105148	Iars	chr13	+	NM_1720	P12694	protein_cc	2510016L isoleucine	Iars
5275	Gm906	1.000	1.000	1.000	1.000	1.000	1.000	0.000	0.000	0.000	380882	Gm906	chr13	-	NM_0010	P9650	protein_cc	. predicted	Gm906
5276	2310081J2	1.000	1.000	1.000	1.000	1.000	1.000	0.000	0.000	0.000	69211	2310081J2	chr13	+	NR_04547	.	lncRNA	. RIKEN cDN	2310081J2
5277	Fbxw17	5.176	0.518	2.683	0.000	0.009	0.192	3.430	5.802	4.854	109082	Fbxw17	chr13	+	NR_1754	P25542	protein_cc	1110064L F-box and	Fbxw17
5278	Nutm2	1.000	1.000	1.000	1.000	1.000	1.000	0.000	0.000	0.000	328250	Nutm2	chr13	+	NM_0010	P12794	protein_cc	Fam22| G NUT famil	Nutm2
5279	Gm904	1.000	1.000	1.000	1.000	1.000	1.000	0.000	0.000	0.000	380845	Gm904	chr13	+	NM_0010	P15682	protein_cc	Gm1135 predicted	Gm904
5280	Gm8765	1.000	1.000	1.000	1.000	1.000	1.000	0.000	0.000	0.000	667693	Gm8765	chr13	+	NM_0012	P12531	protein_cc	EG667693 predicted	Gm8765
5281	4930518P	1.000	1.000	1.000	1.000	1.000	1.000	0.000	0.000	0.000	71654	4930518P	chr13	-	NR_04536	.	lncRNA	. RIKEN cDN	4930518P
5282	9430083A	2.522	0.492	1.240	0.051	0.037	0.727	3.858	5.193	4.169	77428	9430083A	chr13	-	NR_02946	.	lncRNA	. RIKEN cDN	9430083A
5283	Spin1	2.878	0.713	2.052	0.002	0.061	0.134	8.216	9.741	9.253	20729	Spin1	chr13	+	NM_0012	P9694	protein_cc	Spin spindlin 1	Spin1
5284	Nxnl2	0.451	0.986	0.444	0.182	1.000	0.423	1.170	0.021	0.000	75124	Nxnl2	chr13	+	NM_0291	P2689	protein_cc	4930519N nucleored	Nxnl2
5285	S1pr3	3.317	1.004	3.330	0.016	0.986	0.082	2.923	4.652	4.658	13610	S1pr3	chr13	+	NM_0101	P24243	protein_cc	AI132464| sphingosir	S1pr3
5286	Shc3	1.000	1.000	1.000	1.000	1.000	1.000	0.000	0.000	0.000	20418	Shc3	chr13	-	NM_0091	P20423	protein_cc	N-Shc| Ra src homol	Shc3
5287	Cks2	0.166	1.528	0.253	0.000	0.320	0.043	7.444	4.851	5.462	66197	Cks2	chr13	-	NM_0254	P9042	protein_cc	1110038L CDC28 pro	Cks2
5288	Secisbp2	1.289	0.968	1.248	0.148	0.838	0.303	7.046	7.412	7.365	75420	Secisbp2	chr13	+	NM_0292	P4705	protein_cc	2210413N SECIS bind	Secisbp2
5289	Sema4d	3.902	0.302	1.178	0.035	0.043	0.607	3.547	5.512	3.784	20354	Sema4d	chr13	-	NM_0012	P26658	protein_cc	CD100| cc sema dom	Sema4d
5290	Gadd45g	3.287	0.978	3.215	0.000	0.638	0.002	10.830	12.546	12.515	23882	Gadd45g	chr13	+	NM_0118	P19225	protein_cc	AI327420| growth ar	Gadd45g
5291	4921525O	3.269	0.306	1.000	0.305	1.000	0.423	0.001	1.710	0.000	74050	4921525O	chr13	+	NR_04506	.	lncRNA	. RIKEN cDN	4921525O
5292	Diras2	0.560	1.371	0.768	0.184	0.191	0.687	0.857	0.021	0.477	68203	Diras2	chr13	-	NM_0010	P25683	protein_cc	2900052J1 DIRAS fam	Diras2
5293	Gm2848	0.355	0.981	0.348	0.056	1.000	0.240	1.524	0.028	0.000	10004057	Gm2848	chr13	-	NR_04606	.	lncRNA	10004057 predicted	Gm2848
5294	Syk	4.874	0.604	2.944	0.001	0.123	0.315	4.320	6.605	5.877	20963	Syk	chr13	+	NM_0011	P13580	protein_cc	Sykb spleen tyr	Syk
5295	BB123696	6.437	1.599	10.296	0.025	0.077	0.007	0.002	2.688	3.366	105404	BB123696	chr13	-	NR_02789	.	lncRNA	. expressed	BB123696
5296	Auh	0.272	1.407	0.383	0.000	0.001	0.023	8.811	6.935	7.428	11992	Auh	chr13	-	NM_0167	P5912	protein_cc	C77140| V AU RNA bi	Auh
5297	Nfil3	4.604	0.367	1.691	0.000	0.000	0.125	7.195	9.397	7.952	18030	Nfil3	chr13	-	NM_0173	P16721	protein_cc	AV225605 nuclear fa	Nfil3
5298	Ror2	0.556	2.971	1.652	0.001	0.054	0.433	0.890	0.043	1.614	26564	Ror2	chr13	-	NM_0138	P678	protein_cc	mRor2| N receptor t	Ror2
5299	Sptlc1	1.287	0.618	0.796	0.002	0.000	0.041	8.637	9.002	8.308	268656	Sptlc1	chr13	-	NM_0092	P23984	protein_cc	AW55208 serine pal	Sptlc1
5300	Gm2762	1.000	1.000	1.000	1.000	1.000	1.000	0.000	0.000	0.000	10004042	Gm2762	chr13	+	NR_03799	.	lncRNA	10004042 predicted	Gm2762
5301	Msx2	1.000	1.000	1.000	1.000	1.000	1.000	0.000	0.000	0.000	17702	Msx2	chr13	-	NM_0136	P3945	protein_cc	BB122635 msh home	Msx2
5302	9530014B	1.000	1.000	1.000	1.000	1.000	1.000	0.000	0.000	0.000	77398	9530014B	chr13	-	NR_13107	.	lncRNA	. RIKEN cDN	9530014B
5303	4930555G	1.000	1.000	1.000	1.000	1.000	1.000	0.000	0.000	0.000	75252	4930555G	chr13	+	NR_13101	.	lncRNA	. RIKEN cDN	4930555G
5304	Drd1	1.092	16.816	18.359	0.000	0.000	0.000	0.002	0.128	4.200	13488	Drd1	chr13	-	NM_0100	P21464	protein_cc	C030036C dopamine	Drd1
5305	Sfxn1	0.674	1.469	0.990	0.029	0.119	0.975	11.761	11.192	11.747	14057	Sfxn1	chr13	+	NM_0273	P26778	protein_cc	28100020 sideroflex	Sfxn1
5306	Hrh2	5.098	0.583	2.972	0.167	0.435	0.069	0.001	2.351	1.573	15466	Hrh2	chr13	+	NM_0010	P15952	protein_cc	. histamine	Hrh2
5307	Cplx2	0.283	1.182	0.335	0.050	0.783	0.175	4.780	2.961	3.202	12890	Cplx2	chr13	+	NM_0099	P16215	protein_cc	921-L| AI4 complexin	Cplx2
5308	Thoc3	0.915	1.287	1.178	0.564	0.152	0.365	8.788	8.659	9.024	73666	Thoc3	chr13	-	NM_0285	P7459	protein_cc	2410044K THO comp	Thoc3
5309	Simc1	1.057	1.288	1.361	0.533	0.039	0.062	6.944	7.023	7.388	319719	Simc1	chr13	+	NM_1769	P23632	protein_cc	4732471D SUMO-int	Simc1
5310	4833439L	1.132	1.093	1.237	0.624	0.684	0.472	7.867	8.045	8.174	97820	4833439L	chr13	-	NM_0012	P8472	protein_cc	4930558H RIKEN cDN	4833439L1
5311	Arl10	1.023	3.072	3.142	0.000	0.003	0.057	0.000	0.033	1.652	56795	Arl10	chr13	+	NM_0199	P17036	protein_cc	Arl10a| Ar ADP-ribos	Arl10
5312	Nop16	1.363	1.624	2.213	0.046	0.004	0.008	8.274	8.720	9.419	28126	Nop16	chr13	-	NM_1786	P10098	protein_cc	AA409471 NOP16 nu	Nop16
5313	Higd2a	0.547	1.376	0.753	0.000	0.002	0.046	10.586	9.716	10.176	67044	Higd2a	chr13	+	NM_0259	P4391	protein_cc	2010110M HIG1 dom	Higd2a
5314	Cltb	2.549	0.934	2.381	0.000	0.171	0.023	8.660	10.010	9.912	74325	Cltb	chr13	-	NM_0288	P22129	protein_cc	2310046E clathrin, li	Cltb
5315	Faf2	1.370	0.719	0.985	0.007	0.006	0.796	8.907	9.361	8.884	76577	Faf2	chr13	+	NM_1783	P13008	protein_cc	2210404D Fas associ	Faf2
5316	Rnf44	1.203	0.816	0.982	0.008	0.141	0.931	8.463	8.730	8.437	105239	Rnf44	chr13	-	NM_1340	P11685	protein_cc	AI854545| ring finger	Rnf44
5317	Cdhr2	0.684	3.041	2.079	0.197	0.003	0.159	0.587	0.039	1.643	268663	Cdhr2	chr13	+	NM_0010	P12037	protein_cc	Gm624| P cadherin-r	Cdhr2
5318	Gprin1	0.676	1.801	1.217	0.188	0.184	0.768	0.587	0.021	0.870	26913	Gprin1	chr13	-	NM_0120	P19020	protein_cc	GRIN1| Z1 G protein-	Gprin1
5319	Sncb	1.000	1.000	1.000	1.000	1.000	1.000	0.000	0.000	0.000	104069	Sncb	chr13	-	NM_0336	P12295	protein_cc	AI838531| synuclein,	Sncb
5320	Eif4e1b	1.000	1.000	1.000	1.000	1.000	1.000	0.000	0.000	0.000	218268	Eif4e1b	chr13	+	NM_0012	P13668	protein_cc	Eif4eloo| (eukaryoti	Eif4e1b
5321	Tspan17	0.746	0.386	0.288	0.187	0.043	0.011	4.378	3.956	2.581	74257	Tspan17	chr13	+	NM_0288	P7966	protein_cc	2210021G tetraspani	Tspan17
5322	Unc5a	2.841	0.415	1.179	0.027	0.035	0.299	3.203	4.710	3.440	107448	Unc5a	chr13	+	NM_1531	P14108	protein_cc	mKIAA197 unc-5 netr	Unc5a
5323	Hk3	6.167	0.374	2.304	0.003	0.008	0.166	3.653	6.278	4.858	212032	Hk3	chr13	-	NM_0012	P2243	protein_cc	HK III| HK hexokinas	Hk3
5324	Uimc1	1.066	0.793	0.846	0.727	0.297	0.377	7.892	7.984	7.650	20184	Uimc1	chr13	-	NM_0012	P17716	protein_cc	9430016E ubiquitin i	Uimc1
5325	Zfp346	1.088	0.783	0.851	0.758	0.102	0.766	5.538	5.659	5.305	26919	Zfp346	chr13	+	NM_0120	P21189	protein_cc	Jaz| Znf34 zinc finger	Zfp346
5326	Fgfr4	0.692	0.905	0.626	0.036	0.342	0.169	7.175	6.644	6.500	14186	Fgfr4	chr13	+	NM_0080	P20187	protein_cc	Fgfr-4 fibroblast	Fgfr4
5327	Nsd1	1.165	0.853	0.993	0.100	0.087	0.749	8.890	9.110	8.880	18193	Nsd1	chr13	+	NM_0087	P25599	protein_cc	AI528500| nuclear re	Nsd1
5328	Rab24	0.626	1.371	0.858	0.013	0.087	0.495	9.213	8.537	8.992	19336	Rab24	chr13	-	NM_0090	P17560	protein_cc	6530406O RAB24, m	Rab24
5329	Prelid1	0.750	1.025	0.769	0.002	0.659	0.032	10.068	9.654	9.689	66494	Prelid1	chr13	+	NM_0255	P15098	protein_cc	2610524G PRELI dom	Prelid1
5330	Mxd3	0.747	1.041	0.777	0.012	0.654	0.041	9.700	9.278	9.337	17121	Mxd3	chr13	-	NM_0166	P8068	protein_cc	4631412E Max dime	Mxd3
5331	Lman2	0.912	0.984	0.897	0.501	0.889	0.441	11.260	11.127	11.104	66890	Lman2	chr13	-	NM_0258	P20418	protein_cc	1110003H lectin, ma	Lman2
5332	Rgs14	1.014	1.358	1.376	0.982	0.507	0.724	2.535	2.555	2.996	51791	Rgs14	chr13	+	NM_0167	P7709	protein_cc	RPIP1 regulator	Rgs14
5333	Slc34a1	0.716	0.995	0.712	0.182	1.000	0.423	0.490	0.008	0.000	20505	Slc34a1	chr13	+	NM_0113	P16915	protein_cc	NaPi-IIa| I solute car	Slc34a1
5334	Pfn3	1.000	1.000	1.000	1.000	1.000	1.000	0.000	0.000	0.000	75477	Pfn3	chr13	-	NM_0293	P21528	protein_cc	1700012P profilin 3	Pfn3
5335	F12	0.532	1.259	0.670	0.003	0.120	0.058	12.251	11.340	11.673	58992	F12	chr13	-	NM_0214	P16978	protein_cc	FXII| HAF coagulatio	F12
5336	Grk6	1.158	1.688	1.956	0.590	0.052	0.074	6.906	7.118	7.873	26385	Grk6	chr13	+	NM_0012	P7441	protein_cc	Gprk6 G protein-	Grk6
5337	Prr7	1.000	1.000	1.000	1.000	1.000	1.000	0.000	0.000	0.000	432763	Prr7	chr13	+	NM_0010	P20673	protein_cc	XM_4842(proline ric	Prr7
5338	Dbn1	13.781	0.820	11.304	0.041	0.610	0.039	4.087	7.871	7.585	56320	Dbn1	chr13	-	NM_0011	P20082	protein_cc	. drebrin 1	Dbn1
5339	Pdlim7	3.373	0.695	2.346	0.013	0.150	0.067	5.412	7.166	6.642	67399	Pdlim7	chr13	-	NM_0011	P18422	protein_cc	LMP PDZ and L	Pdlim7
5340	Dok3	3.429	1.022	3.502	0.005	0.897	0.049	3.894	5.672	5.702	27261	Dok3	chr13	-	NM_0137	P13515	protein_cc	AI450713| docking pr	Dok3
5341	Ddx41	1.255	0.960	1.205	0.160	0.756	0.109	7.559	7.887	7.828	72935	Ddx41	chr13	-	NM_1340	P14267	protein_cc	2900024F(DEAD (Asp	Ddx41
5342	Fam193b	0.593	0.546	0.324	0.009	0.002	0.035	7.406	6.652	5.778	212483	Fam193b	chr13	-	NM_1453	P13955	protein_cc	IRIZIO| Kia family wit	Fam193b
5343	Tmed9	3.327	0.965	3.209	0.001	0.717	0.009	10.181	11.915	11.863	67511	Tmed9	chr13	+	NM_0262	P2438	protein_cc	2400003B transmem	Tmed9
5344	B4galt7	1.081	1.184	1.280	0.883	0.726	0.304	2.268	2.380	2.623	218271	B4galt7	chr13	+	NM_1460	P26868	protein_cc	. xylosylpro	B4galt7
5345	Caml	2.078	0.989	2.056	0.103	0.970	0.136	6.686	7.741	7.726	12328	Caml	chr13	+	NM_0075	P10810	protein_cc	AI385748| calcium m	Caml
5346	Ddx46	0.802	1.222	0.980	0.007	0.015	0.595	9.277	8.959	9.248	212880	Ddx46	chr13	+	NM_0012	P21104	protein_cc	2200005K DEAD (Asp	Ddx46
5347	B230219D	0.798	0.841	0.672	0.024	0.182	0.153	9.045	8.720	8.470	78521	B230219D	chr13	+	NM_1812	P16925	protein_cc	AA119856 RIKEN cDN	B230219D
5348	Txndc15	0.300	1.178	0.353	0.000	0.521	0.038	10.411	8.674	8.911	69672	Txndc15	chr13	+	NM_1751	P11001	protein_cc	2310047H thioredoxi	Txndc15
5349	Pcbd2	0.533	1.026	0.546	0.000	0.691	0.012	8.927	8.019	8.055	72562	Pcbd2	chr13	+	NM_0282	P23257	protein_cc	2700061N pterin 4 al	Pcbd2
5350	Catsper3	0.344	2.542	0.874	0.184	0.185	0.897	1.588	0.049	1.395	76856	Catsper3	chr13	+	NM_0012	P20598	protein_cc	4921522D cation cha	Catsper3
5351	Pitx1	0.726	1.387	1.007	0.187	0.187	0.988	0.477	0.015	0.487	18740	Pitx1	chr13	-	NM_0110	P17206	protein_cc	Bft| P-OT) paired-like	Pitx1
5352	H2afy	0.990	0.732	0.724	0.927	0.038	0.110	10.217	10.203	9.752	26914	H2afy	chr13	-	NM_0011	P24393	protein_cc	H2AF12M H2A histo	H2afy
5353	Tifab	0.853	1.465	1.250	0.747	0.580	0.853	2.929	2.700	3.251	212937	Tifab	chr13	-	NM_1459	P21865	protein_cc	. TRAF-inte	Tifab
5354	4930550C	1.000	1.000	1.000	1.000	1.000	1.000	0.000	0.000	0.000	75245	4930550C	chr13	-	NR_13102	.	lncRNA	. RIKEN cDN	4930550C
5355	Neurog1	1.000	1.000	1.000	1.000	1.000	1.000	0.000	0.000	0.000	18014	Neurog1	chr13	-	NM_0108	P53	protein_cc	AKA| bHLI neurogeni	Neurog1
5356	Cxcl14	1.165	3.666	4.272	0.738	0.000	0.046	3.432	3.652	5.526	57266	Cxcl14	chr13	-	NM_0195	P20873	protein_cc	1110031L chemokin	Cxcl14

5357	Gm10782	1.005	1.439	1.446	0.000	0.182	0.423	0.000	0.008	0.532	10003849	Gm10782	chr13	+	NR_04606	.	lncRNA	ENSMUSG	predicted	Gm10782
5358	Slc25a48	0.171	2.025	0.345	0.002	0.007	0.079	2.627	0.075	1.092	328258	Slc25a48	chr13	+	NM_1778	P10754	protein_cc	AU042651	solute car	Slc25a48
5359	Il9	1.000	1.000	1.000	1.000	1.000	1.000	0.000	0.000	0.000	16198	Il9	chr13	-	NM_0083	P18596	protein_cc	Il-9| P40	interleukin	Il9
5360	Fbxl21	0.112	0.761	0.086	0.001	0.527	0.034	5.216	2.064	1.669	213311	Fbxl21	chr13	+	NM_1786	P17051	protein_cc	D630045D	F-box and	Fbxl21
5361	Lect2	0.287	0.695	0.200	0.000	0.010	0.002	11.042	9.242	8.716	16841	Lect2	chr13	-	NM_0107	P15697	protein_cc	.	leukocyte	Lect2
5362	Tgfbi	0.228	1.550	0.353	0.000	0.075	0.013	7.811	5.675	6.308	21810	Tgfbi	chr13	+	NM_0093	P19739	protein_cc	68kDa| AI	transform	Tgfbi
5363	Smad5	2.397	0.415	0.996	0.001	0.001	0.979	8.618	9.879	8.611	17129	Smad5	chr13	+	NM_0085	P8200	protein_cc	1110051N	SMAD fam	Smad5
5364	Trpc7	1.000	1.000	1.000	1.000	1.000	1.000	0.000	0.000	0.000	26946	Trpc7	chr13	-	NM_0120	P10141	protein_cc	TRP7| Trp	transient	Trpc7
5365	Spock1	1.059	0.629	0.666	0.888	1.000	0.423	0.587	0.669	0.000	20745	Spock1	chr13	-	NM_0011	P3418	protein_cc	Spock| tes	sparc/oste	Spock1
5366	Klhl3	1.000	1.000	1.000	1.000	1.000	1.000	0.000	0.000	0.000	10050308	Klhl3	chr13	-	NM_0011	P6077	protein_cc	7530408C	kelch-like	Klhl3
5367	Hnrnpa0	1.377	0.894	1.231	0.000	0.364	0.438	10.241	10.702	10.540	77134	Hnrnpa0	chr13	-	NM_0298	P18885	protein_cc	1110055B	heterogen	Hnrnpa0
5368	1700066J	1.000	1.000	1.000	1.000	1.000	1.000	0.000	0.000	0.000	67339	1700066J	chr13	+	NR_12654	.	lncRNA	.	RIKEN cDN	1700066J0
5369	Idnk	1.310	0.892	1.168	0.007	0.294	0.515	8.767	9.157	8.992	75731	Idnk	chr13	+	NM_1980	P24817	protein_cc	5133401N	idnK gluco	Idnk
5370	Ubqln1	0.660	0.990	0.654	0.001	0.880	0.059	10.588	9.989	9.974	56085	Ubqln1	chr13	-	NM_1522	P24868	protein_cc	1110046H	ubiquilin 1	Ubqln1
5371	Gkap1	0.691	1.229	0.849	0.034	0.041	0.377	8.258	7.725	8.022	56278	Gkap1	chr13	-	NM_0198	P19728	protein_cc	42kDa| 4S	G kinase a	Gkap1
5372	Kif27	1.000	1.000	1.000	1.000	1.000	1.000	0.000	0.000	0.000	75050	Kif27	chr13	-	NM_1752	P24796	protein_cc	4930517I1	kinesin fa	Kif27
5373	2210016F	1.303	0.994	1.294	0.057	0.922	0.240	8.358	8.739	8.730	70153	2210016F	chr13	-	NM_0273	P11285	protein_cc	.	RIKEN cDN	2210016F1
5374	Hnrnpk	0.979	1.230	1.204	0.605	0.007	0.085	11.376	11.346	11.644	15387	Hnrnpk	chr13	-	NM_0013	P6995	protein_cc	Hnrpk| KB	heterogen	Hnrnpk
5375	Rmi1	0.549	1.092	0.599	0.038	0.173	0.231	6.702	5.837	5.964	74386	Rmi1	chr13	+	NR_03176	.	protein_cc	4932432N	RecQ med	Rmi1
5376	Slc28a3	2.162	1.015	2.195	0.300	0.976	0.083	0.001	1.113	1.135	114304	Slc28a3	chr13	-	NM_0223	P13213	protein_cc	Cnt3	solute car	Slc28a3
5377	4930455J1	1.000	1.000	1.000	1.000	1.000	1.000	0.000	0.000	0.000	67585	4930455J1	chr13	-	NR_04546	.	lncRNA	.	RIKEN cDN	4930455J1
5378	Ntrk2	0.186	3.749	0.698	0.001	0.040	0.403	3.185	0.759	2.665	18212	Ntrk2	chr13	+	NM_0012	P1979	protein_cc	GP145-Trk	neurotrop	Ntrk2
5379	Agtpbp1	0.531	0.914	0.485	0.009	0.621	0.022	6.784	5.871	5.741	67269	Agtpbp1	chr13	-	NM_0012	P18717	protein_cc	1700020N	ATP/GTP b	Agtpbp1
5380	A230056J	1.596	1.401	2.237	0.295	0.518	0.423	0.000	0.675	1.162	319469	A230056J	chr13	-	NR_04563	.	lncRNA	.	RIKEN cDN	A230056J0
5381	Naa35	0.594	1.264	0.750	0.002	0.044	0.047	9.246	8.494	8.832	78689	Naa35	chr13	+	NM_0301	P25148	protein_cc	A330021G	N(alpha)-a	Naa35
5382	Golm1	0.552	2.192	1.210	0.032	0.000	0.470	5.211	4.353	5.486	105348	Golm1	chr13	-	NM_0273	P17966	protein_cc	2310001L	golgi mem	Golm1
5383	4921517D	1.005	1.439	1.446	0.000	0.182	0.423	0.000	0.008	0.532	70900	4921517D	chr13	-	NM_1832	P13186	protein_cc	.	RIKEN cDN	4921517D
5384	Spata31d1	1.014	2.240	2.272	0.000	0.182	0.423	0.000	0.021	1.185	72219	Spata31d1	chr13	-	NM_0281	P15973	protein_cc	1700013B	spermatog	Spata31d1
5385	Spata31d1	1.000	1.000	1.000	1.000	1.000	1.000	0.000	0.000	0.000	238662	Spata31d1	chr13	+	NM_0011	P22813	protein_cc	EG238662	spermatog	Spata31d1
5386	Spata31d1	1.000	1.000	1.000	1.000	1.000	1.000	0.000	0.000	0.000	238663	Spata31d1	chr13	-	NM_1777	P25970	protein_cc	4932411G	spermatog	Spata31d1
5387	4930528D	1.000	1.000	1.000	1.000	1.000	1.000	0.000	0.000	0.000	75223	4930528D	chr13	+	NR_04597	.	lncRNA	.	RIKEN cDN	4930528D
5388	Isca1	0.985	1.043	1.027	0.821	0.540	0.571	7.938	7.916	7.976	69046	Isca1	chr13	-	NM_0269	P5290	protein_cc	1810010A	iron-sulfur	Isca1
5389	Etohd2	1.127	0.897	1.011	0.141	0.338	0.961	8.448	8.620	8.464	13996	Etohd2	chr13	+	NR_01534	.	lncRNA	AW49165	ethanol de	Etohd2
5390	Zcchc6	1.061	0.720	0.764	0.260	0.006	0.133	9.612	9.697	9.224	214290	Zcchc6	chr13	-	NM_1535	P9358	protein_cc	6030448M	zinc finger	Zcchc6
5391	A530065N	1.000	1.000	1.000	1.000	1.000	1.000	0.000	0.000	0.000	328263	A530065N	chr13	+	NR_04614	.	lncRNA	A530065N	RIKEN cDN	A530065N
5392	Gas1	0.030	1.119	0.034	0.000	0.824	0.017	7.904	2.862	3.025	14451	Gas1	chr13	-	NM_0080	P13043	protein_cc	AW55419	growth ar	Gas1
5393	Gm5084	0.716	0.995	0.712	0.182	1.000	0.423	0.490	0.008	0.000	10050375	Gm5084	chr13	+	NR_03644	.	lncRNA	A030007L	predicted	Gm5084
5394	Dapk1	0.080	1.380	0.111	0.000	0.271	0.003	8.194	4.557	5.022	69635	Dapk1	chr13	+	NM_0296	P20460	protein_cc	D13Ucla1|	death ass	Dapk1
5395	BC051665	1.000	1.000	1.000	1.000	1.000	1.000	0.000	0.000	0.000	218275	BC051665	chr13	-	NM_1991	P10306	protein_cc	.	cDNA seq	BC051665
5396	Ctsll3	1.000	1.000	1.000	1.000	1.000	1.000	0.000	0.000	0.000	70202	Ctsll3	chr13	-	NM_0273	P24025	protein_cc	2310051M	cathepsin	Ctsll3
5397	4930486L	22.311	0.482	10.748	0.000	0.000	0.010	2.063	6.542	5.489	214639	4930486L	chr13	-	NM_1780	P14564	protein_cc	AW54503	RIKEN cDN	4930486L2
5398	Ctla2b	15.675	1.068	16.741	0.001	0.615	0.015	4.727	8.697	8.792	13025	Ctla2b	chr13	-	NM_0077	P10222	protein_cc	AW54556	cytotoxic	Ctla2b
5399	Tpbpb	1.000	1.000	1.000	1.000	1.000	1.000	0.000	0.000	0.000	116913	Tpbpb	chr13	-	NM_0264	P16269	protein_cc	1600012N	trophobla	Tpbpb
5400	Ctla2a	17.156	1.787	30.666	0.000	0.001	0.003	6.149	10.249	11.087	13024	Ctla2a	chr13	-	NM_0011	P12076	protein_cc	Ctla-2a	cytotoxic	Ctla2a
5401	Tpbpa	1.000	1.000	1.000	1.000	1.000	1.000	0.000	0.000	0.000	21984	Tpbpa	chr13	-	NM_0094	P11397	protein_cc	AV033951	trophobla	Tpbpa
5402	Ctsj	13.649	3.503	47.817	0.127	0.029	0.069	0.003	3.773	5.582	26898	Ctsj	chr13	-	NM_0120	P16127	protein_cc	CATP| Cat	cathepsin	Ctsj
5403	Ctsq	1.000	1.000	1.000	1.000	1.000	1.000	0.000	0.000	0.000	104002	Ctsq	chr13	-	NM_0296	P12876	protein_cc	1600010J	cathepsin	Ctsq
5404	Ctsr	1.000	1.000	1.000	1.000	1.000	1.000	0.000	0.000	0.000	56835	Ctsr	chr13	-	NM_0202	P24746	protein_cc	AI428350	cathepsin	Ctsr
5405	Cts6	1.000	1.000	1.000	1.000	1.000	1.000	0.000	0.000	0.000	58518	Cts6	chr13	-	NM_0214	P1636	protein_cc	1600022N	cathepsin	Cts6
5406	Cts8	0.669	0.995	0.666	0.181	1.000	0.423	0.587	0.008	0.000	56094	Cts8	chr13	-	NM_0195	P1795	protein_cc	CTS2| Epc	cathepsin	Cts8
5407	Cts8-ps	1.000	1.000	1.000	1.000	1.000	1.000	0.000	0.000	0.000	116911	Cts8-ps	chr13	-	NR_02787	.	misc_RNA	Cts2-ps	cathepsin	Cts8-ps
5408	Cts7	1.005	1.439	1.446	0.000	0.182	0.423	0.000	0.008	0.532	56092	Cts7	chr13	-	NM_0195	P3368	protein_cc	AA408230	cathepsin	Cts7
5409	Ctsm	1.000	1.000	1.000	1.000	1.000	1.000	0.000	0.000	0.000	64139	Ctsm	chr13	-	NM_0223	P24340	protein_cc	1600027J1	cathepsin	Ctsm
5410	Cts3	1.000	1.000	1.000	1.000	1.000	1.000	0.000	0.000	0.000	117066	Cts3	chr13	-	NM_0269	P11435	protein_cc	1600000I2	cathepsin	Cts3
5411	BC052688	1.000	1.000	1.000	1.000	1.000	1.000	0.000	0.000	0.000	432812	BC052688	chr13	+	NR_02843	.	misc_RNA	.	cDNA seq	BC052688
5412	Zfp808	1.409	0.414	0.583	0.638	0.289	0.629	1.867	2.361	1.088	630579	Zfp808	chr13	+	NM_0010	P26390	protein_cc	EG630579	zinc finger	Zfp808
5413	Gm3604	1.286	1.476	1.898	0.270	0.432	0.497	1.140	1.503	2.064	10004197	Gm3604	chr13	-	NM_0011	P25290	protein_cc	10004197	predicted	Gm3604
5414	Zfp935	1.174	0.626	0.735	0.114	0.129	0.534	4.889	5.121	4.446	71508	Zfp935	chr13	-	NM_0011	P11191	protein_cc	8430426H	zinc finger	Zfp935
5415	Zfp934	1.322	1.387	1.834	0.700	0.521	0.295	1.073	1.476	1.948	77117	Zfp934	chr13	-	NM_1752	P19467	protein_cc	6720457D	zinc finger	Zfp934
5416	6720489N	1.276	0.492	0.628	0.734	0.401	0.573	1.102	1.454	0.431	211378	6720489N	chr13	-	NM_1733	P23069	protein_cc	9330128I	RIKEN cDN	6720489N
5417	Gm5141	1.631	2.984	4.865	0.281	0.102	0.243	0.001	0.706	2.283	380850	Gm5141	chr13	-	NM_0012	P1604	protein_cc	380850	predicted	Gm5141
5418	Fbp2	0.566	4.152	2.348	0.001	0.029	0.266	0.879	0.056	2.110	14120	Fbp2	chr13	-	NM_0079	P22588	protein_cc	Fbp-1| Fb	fructose b	Fbp2
5419	Fbp1	1.349	1.309	1.767	0.002	0.009	0.029	12.491	12.923	13.312	14121	Fbp1	chr13	-	NM_0193	P24487	protein_cc	Fbp-2| Fb	fructose b	Fbp1
5420	2010111I	1.798	0.709	1.274	0.008	0.019	0.509	7.053	7.900	7.403	72061	2010111I	chr13	+	NM_0012	P15381	protein_cc	2300006M	RIKEN cDN	2010111I0
5421	Gm16907	2.698	0.653	1.762	0.051	0.193	0.280	0.757	2.189	1.574	10050411	Gm16907	chr13	-	NR_04579	.	lncRNA	.	predicted	Gm16907
5422	Fancc	52.582	0.342	17.981	0.000	0.001	0.084	4.653	10.369	8.821	14088	Fancc	chr13	-	NM_0010	P15487	protein_cc	Facc| mir-	Fanconi ar	Fancc
5423	Ptch1	1.183	1.453	1.718	0.672	0.489	0.553	0.439	0.682	1.220	19206	Ptch1	chr13	-	NM_0089	P7899	protein_cc	A230106A	patched 1	Ptch1
5424	1700024I	1.000	1.000	1.000	1.000	1.000	1.000	0.000	0.000	0.000	75612	1700024I	chr13	-	NR_12647	.	lncRNA	.	RIKEN cDN	1700024I0
5425	Ercc6l2	1.076	1.245	1.340	0.809	0.454	0.424	5.756	5.863	6.178	76251	Ercc6l2	chr13	+	NM_0010	P26630	protein_cc	0610007P	excision re	Ercc6l2
5426	Hsd17b3	2.775	0.830	2.305	0.020	0.638	0.423	0.001	1.473	1.205	15487	Hsd17b3	chr13	-	NM_0082	P14350	protein_cc	.	hydroxyst	Hsd17b3
5427	Slc35d2	0.234	3.443	0.806	0.000	0.001	0.137	8.292	6.197	7.981	70484	Slc35d2	chr13	-	NM_0010	P12005	protein_cc	5730408I2	solute car	Slc35d2
5428	Zfp367	0.444	1.780	0.791	0.001	0.008	0.314	7.430	6.259	7.091	238673	Zfp367	chr13	-	NM_1754	P17262	protein_cc	8030486J2	zinc finger	Zfp367
5429	Habp4	0.170	2.627	0.448	0.000	0.030	0.057	9.951	7.399	8.792	56541	Habp4	chr13	+	NM_0199	P7268	protein_cc	4933413D	hyaluronic	Habp4
5430	Cdc14b	0.117	1.655	0.194	0.000	0.003	0.002	9.035	5.944	6.671	218294	Cdc14b	chr13	-	NM_1725	P9719	protein_cc	2810432N	CDC14 cel	Cdc14b
5431	1810034E	0.120	2.079	0.249	0.000	0.002	0.006	6.650	3.591	4.647	66503	1810034E	chr13	+	NR_04579	.	lncRNA	.	RIKEN cDN	1810034E1
5432	Aaed1	1.026	0.639	0.655	0.936	0.280	0.019	5.500	5.536	4.890	66129	Aaed1	chr13	-	NM_0253	P12285	protein_cc	1110018J1	AhpC/TSA	Aaed1
5433	Ctsl	1.088	1.473	1.603	0.320	0.115	0.292	12.391	12.513	13.072	13039	Ctsl	chr13	-	NM_0099	P18874	protein_cc	1190035F	cathepsin	Ctsl
5434	Cdk20	1.382	1.452	2.007	0.000	0.001	0.015	5.514	5.982	6.519	105278	Cdk20	chr13	+	NM_0531	P20166	protein_cc	4932702G	cyclin-dep	Cdk20
5435	1190003K	1.000	1.000	1.000	1.000	1.000	1.000	0.000	0.000	0.000	68880	1190003K	chr13	-	NM_0011	P19089	protein_cc	.	RIKEN cDN	1190003K1
5436	Cntnap3	1.000	1.000	1.000	1.000	1.000	1.000	0.000	0.000	0.000	238680	Cntnap3	chr13	-	NM_0010	P3430	protein_cc	.	contactin	Cntnap3
5437	Spata31	1.000	1.000	1.000	1.000	1.000	1.000	0.000	0.000	0.000	78124	Spata31	chr13	+	NM_0300	P16434	protein_cc	4930458L	spermatog	Spata31
5438	Spata31d1	1.000	1.000	1.000	1.000	1.000	1.000	0.000	0.000	0.000	238683	Spata31d1	chr13	+	NM_0010	P8220	protein_cc	4932441B	spermatog	Spata31d1
5439	Hiatl1	0.968	1.003	0.971	0.726	0.971	0.694	7.762	7.715	7.720	.	Hiatl1	chr13	-	NM_0010	P611	.	.	.	Hiatl1
5440	Olfr466	1.000	1.000	1.000	1.000	1.000	1.000	0.000	0.000	0.000	258816	Olfr466	chr13	+	NM_1468	P15971	protein_cc	MOR209-1	olfactory r	Olfr466
5441	Nlrp4f	1.000	1.000	1.000	1.000	1.000	1.000	0.000	0.000	0.000	97895	Nlrp4f	chr13	-	NM_1752	P1474	protein_cc	C330026N	NLR family	Nlrp4f
5442	Zfp369	1.101	0.416	0.458	0.659	0.008	0.302	3.185	3.324	2.058	170936	Zfp369	chr13	+	NM_1783	P26421	protein_cc	B930030B	zinc finger	Zfp369
5443	Gm10324	0.716	0.995	0.712	0.182	1.000	0.423	0.490	0.008	0.000	628709	Gm10324	chr13	+	NM_0011	P26873	protein_cc	ENSMUSG	predicted	Gm10324
5444	2410141K	1.000	1.000	1.000	1.000	1.000	1.000	0.000	0.000	0.000	76803	2410141K	chr13	+	NM_1831	P26557	protein_cc	4930482J1	RIKEN cDN	2410141K0
5445	Uqcrb	0.785	0.970	0.761	0.000	0.308	0.010	11.182	10.833	10.788	67530	Uqcrb	chr13	-	NM_0262	P1960	protein_cc	2210415M	ubiquinol-	Uqcrb
5446	Gm10767	1.060	0.742	0.786	0.835	0.072	0.680	3.977	4.062	3.630	10003853	Gm10767	chr13	+	NM_0011	P2037	protein_cc	ENSMUSG	predicted	Gm10767
5447	Mterf3	1.356	0.764	1.035	0.067	0.412	0.953	5.383	5.822	5.433	66410	Mterf3	chr13	-	NM_0255	P22619	protein_cc	2410017I1	mitochond	Mterf3

5448	Ptdss1	0.397	1.219	0.484	0.000	0.064	0.013	9.514	8.180	8.466	19210	Ptdss1	chr13	+	NM_0089	P18845	protein_cc	AU044268	phosphati	Ptdss1
5449	4933433G	0.653	0.799	0.522	0.093	0.395	0.070	2.830	2.215	1.891	71226	4933433G	chr13	-	NR_04585	.	lncRNA	.	RIKEN cDN	4933433G
5450	Zfp712	0.440	3.588	1.578	0.153	0.007	0.406	1.921	0.736	2.579	78251	Zfp712	chr13	-	NM_0011	P16479	protein_cc	4921504N	zinc finger	Zfp712
5451	Zfp708	0.357	0.700	0.250	0.000	0.096	0.011	4.911	3.426	2.911	432769	Zfp708	chr13	-	NM_0010	P16720	protein_cc	Rslcan11	zinc finger	Zfp708
5452	Rslcan18	1.669	0.584	0.975	0.054	0.190	0.972	3.210	3.950	3.175	432770	Rslcan18	chr13	-	NM_0012	P22453	protein_cc	mszf51	regulator	Rslcan18
5453	Zfp759	0.664	0.900	0.597	0.288	0.815	0.068	4.082	3.490	3.338	268670	Zfp759	chr13	+	NM_1723	P16928	protein_cc	BC028265	zinc finger	Zfp759
5454	Rsl1	2.991	0.555	1.660	0.000	0.001	0.196	2.049	3.629	2.780	380855	Rsl1	chr13	+	NM_0010	P1399	protein_cc	rslcan-9	regulator	Rsl1
5455	Zfp455	1.831	0.496	0.908	0.308	0.264	0.029	3.582	4.455	3.442	218311	Zfp455	chr13	+	NM_0010	P12818	protein_cc	3732412P	zinc finger	Zfp455
5456	Zfp458	0.897	3.609	3.239	0.860	0.033	0.193	1.326	1.170	3.022	238690	Zfp458	chr13	-	NM_0010	P6726	protein_cc	BC062958	zinc finger	Zfp458
5457	F630042J(	0.716	0.995	0.712	0.182	1.000	0.423	0.490	0.008	0.000	10003862	F630042J(	chr13	+	NR_03354	.	lncRNA	ENSMUSG	RIKEN cDN	F630042J0
5458	Zfp457	1.000	1.000	1.000	1.000	1.000	1.000	0.000	0.000	0.000	431706	Zfp457	chr13	-	NM_0010	P25531	protein_cc	Rslcan-6	zinc finger	Zfp457
5459	Zfp595	1.457	0.863	1.257	0.336	0.641	0.599	4.724	5.267	5.054	218314	Zfp595	chr13	-	NM_1776	P8102	protein_cc	A230042K	zinc finger	Zfp595
5460	Zfp953	0.533	0.678	0.361	0.066	0.446	0.143	5.691	4.782	4.221	629016	Zfp953	chr13	-	NM_0010	P12125	protein_cc	E130120F	zinc finger	Zfp953
5461	Zfp456	3.206	0.514	1.649	0.000	0.021	0.419	3.383	5.064	4.105	408065	Zfp456	chr13	-	NM_0010	P9898	protein_cc	BC065418	zinc finger	Zfp456
5462	Zfp429	2.520	0.433	1.091	0.029	0.040	0.859	4.114	5.448	4.240	72807	Zfp429	chr13	-	NM_0010	P13484	protein_cc	2810487A	zinc finger	Zfp429
5463	Zfp459	2.181	0.164	0.358	0.318	0.141	0.307	2.505	3.630	1.023	328274	Zfp459	chr13	-	NM_1778	P16357	protein_cc	9930025G	zinc finger	Zfp459
5464	Zfp874a	2.048	0.903	1.848	0.041	0.695	0.281	6.630	7.663	7.516	238692	Zfp874a	chr13	-	NM_1777	P487	protein_cc	C330011K	zinc finger	Zfp874a
5465	Zfp874b	1.315	0.926	1.218	0.010	0.365	0.193	5.499	5.895	5.783	408067	Zfp874b	chr13	-	NM_0010	P9685	protein_cc	9630025I2	zinc finger	Zfp874b
5466	Zfp58	1.442	0.533	0.769	0.124	0.034	0.649	4.376	4.904	3.997	238693	Zfp58	chr13	-	NM_0010	P26551	protein_cc	A530094I1	zinc finger	Zfp58
5467	Zfp87	1.263	0.894	1.129	0.145	0.120	0.669	5.484	5.821	5.660	170763	Zfp87	chr13	-	NM_1332	P18952	protein_cc	2210039O	zinc finger	Zfp87
5468	Zfp748	0.913	0.879	0.802	0.616	0.616	0.665	5.096	4.965	4.778	212276	Zfp748	chr13	-	NM_0010	P21798	protein_cc	KRAB-O| r	zinc finger	Zfp748
5469	9430065F	1.887	0.461	0.870	0.093	0.042	0.862	1.074	1.989	0.873	77857	9430065F	chr13	+	NR_13110	.	lncRNA	E130110G	RIKEN cDN	9430065F1
5470	Zfp729b	1.583	0.550	0.870	0.063	0.053	0.680	6.078	6.741	5.878	10041670	Zfp729b	chr13	-	NM_0011	P21272	protein_cc	AA987127	zinc finger	Zfp729b
5471	Zfp729a	1.895	0.623	1.181	0.000	0.000	0.322	5.251	6.173	5.491	212281	Zfp729a	chr13	-	NM_1831	P22119	protein_cc	A530054K	zinc finger	Zfp729a
5472	Zfp738	1.655	0.964	1.595	0.022	0.610	0.160	5.108	5.835	5.782	408068	Zfp738	chr13	-	NM_0010	P5109	protein_cc	3830402I(	zinc finger	Zfp738
5473	Zfp65	1.411	0.875	1.235	0.001	0.126	0.284	5.951	6.448	6.256	235907	Zfp65	chr13	-	NM_1456	P10935	protein_cc	KRAB5| pa	zinc finger	Zfp65
5474	Zfp85os	0.308	2.587	0.795	0.011	0.012	0.479	3.515	1.813	3.184	68271	Zfp85os	chr13	+	NR_02796	.	lncRNA	4930441O	zinc finger	Zfp85os
5475	Zfp85	0.949	1.427	1.354	0.893	0.472	0.740	3.014	2.938	3.451	22746	Zfp85	chr13	-	NM_0010	P2710	protein_cc	KRAB19| F	zinc finger	Zfp85
5476	Zfp493	1.543	0.134	0.207	0.416	0.083	0.095	3.007	3.632	0.735	72958	Zfp493	chr13	+	NM_0284	P2263	protein_cc	2900054J(	zinc finger	Zfp493
5477	4930525G	2.081	0.896	1.864	0.287	0.795	0.400	1.973	3.031	2.872	320163	4930525G	chr13	-	NR_04519	.	lncRNA	.	RIKEN cDN	4930525G
5478	Zfp273	0.301	0.968	0.291	0.010	0.944	0.109	3.545	1.812	1.764	212569	Zfp273	chr13	+	NM_1983	P48	protein_cc	6820416H	zinc finger	Zfp273
5479	Gm10037	1.000	1.000	1.000	1.000	1.000	1.000	0.000	0.000	0.000	10263736	Gm10037	chr13	+	NM_0013	P4930	protein_cc	EG328280	predicted	Gm10037
5480	BC048507	1.010	1.821	1.840	0.000	0.000	0.002	0.000	0.015	0.880	408058	BC048507	chr13	+	NM_0010	P10577	protein_cc	.	cDNA sequ	BC048507
5481	Mtrr	2.163	1.026	2.220	0.000	0.835	0.093	5.477	6.591	6.628	210009	Mtrr	chr13	-	NR_13189	.	protein_cc	4732420G	5-methylt	Mtrr
5482	Fastkd3	2.052	0.775	1.589	0.000	0.225	0.388	6.709	7.745	7.377	69577	Fastkd3	chr13	+	NM_0271	P3872	protein_cc	2310010B	FAST kinas	Fastkd3
5483	1700001L	0.457	1.358	0.620	0.183	0.195	0.578	1.158	0.027	0.468	69315	1700001L	chr13	+	NM_0270	P21182	protein_cc	.	RIKEN cDN	1700001L1
5484	Adcy2	0.275	4.416	1.214	0.068	0.183	0.860	1.940	0.077	2.219	210044	Adcy2	chr13	-	NM_1535	P8526	protein_cc	mKIAA106	adenylate	Adcy2
5485	Papd7	0.845	0.619	0.523	0.173	0.008	0.076	6.521	6.278	5.585	210106	Papd7	chr13	-	NM_0011	P2852	protein_cc	LAK-1| PO	PAP assoc	Papd7
5486	Srd5a1	0.198	1.088	0.216	0.002	0.760	0.065	7.533	5.199	5.322	78925	Srd5a1	chr13	-	NM_1752	P22582	protein_cc	0610031P	steroid 5 a	Srd5a1
5487	Nsun2	0.818	1.594	1.304	0.001	0.000	0.015	9.528	9.238	9.911	28114	Nsun2	chr13	+	NM_1453	P19806	protein_cc	D13Wsu1	NOL1/NOI	Nsun2
5488	Ube2ql1	1.376	0.221	0.304	0.728	1.000	0.422	1.720	2.180	0.000	76980	Ube2ql1	chr13	-	NM_0011	P14594	protein_cc	3110006E	ubiquitin-	Ube2ql1
5489	Med10	1.890	1.043	1.971	0.008	0.838	0.233	7.183	8.102	8.162	28077	Med10	chr13	+	NM_1385	P23566	protein_cc	AA959813	mediator	Med10
5490	1700084F	1.000	1.000	1.000	1.000	1.000	1.000	0.000	0.000	0.000	74280	1700084F	chr13	-	NR_04596	.	lncRNA	1700003E	RIKEN cDN	1700084F2
5491	Gm3772	1.000	1.000	1.000	1.000	1.000	1.000	0.000	0.000	0.000	10004229	Gm3772	chr13	+	NR_13115	.	lncRNA	10004229	predicted	Gm3772
5492	4930520P	1.000	1.000	1.000	1.000	1.000	1.000	0.000	0.000	0.000	75086	4930520P	chr13	+	NR_03659	.	lncRNA	.	RIKEN cDN	4930520P
5493	1700100L	1.000	1.000	1.000	1.000	1.000	1.000	0.000	0.000	0.000	73584	1700100L	chr13	+	NR_04593	.	lncRNA	.	RIKEN cDN	1700100L1
5494	Ice1	1.218	0.815	0.993	0.414	0.401	0.905	6.661	6.945	6.650	218333	Ice1	chr13	-	NM_1448	P3680	protein_cc	C77245| n	interactor	Ice1
5495	Adamts16	0.669	0.995	0.666	0.181	1.000	0.423	0.587	0.008	0.000	271127	Adamts16	chr13	-	NM_1720	P6580	protein_cc	.	a disintegr	Adamts16
5496	8030423J	1.000	1.000	1.000	1.000	1.000	1.000	0.000	0.000	0.000	77166	8030423J	chr13	+	NM_0298	P24911	protein_cc	.	RIKEN cDN	8030423J2
5497	Irx1	0.426	0.985	0.420	0.023	1.000	0.157	1.253	0.021	0.000	16371	Irx1	chr13	-	NM_0105	P17377	protein_cc	.	Iroquois re	Irx1
5498	Gm20554	1.000	1.000	1.000	1.000	1.000	1.000	0.000	0.000	0.000	328287	Gm20554	chr13	-	NR_03070	.	lncRNA	D430050G	predicted	Gm20554
5499	Irx2	1.580	0.633	1.000	0.305	1.000	0.423	0.000	0.660	0.000	16372	Irx2	chr13	+	NM_0105	P6532	protein_cc	.	Iroquois re	Irx2
5500	D730050B	1.000	1.000	1.000	1.000	1.000	1.000	0.000	0.000	0.000	78446	D730050B	chr13	-	NR_04619	.	lncRNA	.	RIKEN cDN	D730050B
5501	4930555K	1.000	1.000	1.000	1.000	1.000	1.000	0.000	0.000	0.000	75361	4930555K	chr13	-	NR_12645	.	lncRNA	.	RIKEN cDN	4930555K(
5502	Irx4	1.000	1.000	1.000	1.000	1.000	1.000	0.000	0.000	0.000	50916	Irx4	chr13	+	NM_0188	P20953	protein_cc	.	Iroquois re	Irx4
5503	Ndufs6	0.789	1.187	0.936	0.092	0.145	0.573	10.752	10.409	10.656	407785	Ndufs6	chr13	-	NR_03330	.	protein_cc	BC059730	NADH deh	Ndufs6
5504	Mrpl36	0.725	1.097	0.795	0.000	0.351	0.203	8.935	8.471	8.604	94066	Mrpl36	chr13	+	NM_0531	P21050	protein_cc	AI646041	mitochond	Mrpl36
5505	D630045N	1.183	0.299	0.353	0.819	1.000	0.262	1.501	1.743	0.000	432777	D630045N	chr13	-	NR_04529	.	lncRNA	AU019512	RIKEN cDN	D630045M
5506	Lpcat1	1.572	0.636	1.000	0.304	1.000	0.423	0.000	0.653	0.000	210992	Lpcat1	chr13	+	NM_1453	P5987	protein_cc	2900035H	lysophosp	Lpcat1
5507	Slc6a3	1.000	1.000	1.000	1.000	1.000	1.000	0.000	0.000	0.000	13162	Slc6a3	chr13	+	NM_0100	P10566	protein_cc	DAT| Dat1	solute car	Slc6a3
5508	Clptm1l	1.876	0.999	1.873	0.002	0.982	0.041	9.606	10.514	10.512	218335	Clptm1l	chr13	+	NM_1460	P16888	protein_cc	C130052I1	CLPTM1-li	Clptm1l
5509	Tert	1.000	1.000	1.000	1.000	1.000	1.000	0.000	0.000	0.000	21752	Tert	chr13	+	NM_0093	P9773	protein_cc	EST2| TCS	telomeras	Tert
5510	Slc6a18	1.000	1.000	1.000	1.000	1.000	1.000	0.000	0.000	0.000	22598	Slc6a18	chr13	-	NM_0011	P19528	protein_cc	B0AT3| D(	solute car	Slc6a18
5511	Slc6a19	0.552	0.990	0.547	0.182	1.000	0.423	0.871	0.015	0.000	74338	Slc6a19	chr13	-	NM_0288	P8550	protein_cc	4632401C	solute car	Slc6a19
5512	Slc6a19os	1.000	1.000	1.000	1.000	1.000	1.000	0.000	0.000	0.000	71390	Slc6a19os	chr13	+	NM_0277	P1626	protein_cc	5430425J1	solute car	Slc6a19os
5513	Slc12a7	0.986	0.656	0.647	0.927	0.121	0.329	8.602	8.581	7.973	20499	Slc12a7	chr13	+	NM_0113	P8466	protein_cc	AA408796	solute car	Slc12a7
5514	Nkd2	1.848	0.570	1.053	0.060	0.024	0.920	1.487	2.373	1.563	72293	Nkd2	chr13	-	NM_0281	P7754	protein_cc	2210403L	naked cuti	Nkd2
5515	Trip13	0.688	2.438	1.676	0.456	0.065	0.287	2.938	2.398	3.683	69716	Trip13	chr13	-	NM_0271	P5418	protein_cc	2410002G	thyroid ho	Trip13
5516	Brd9	1.138	0.955	1.087	0.009	0.542	0.544	7.288	7.475	7.408	105246	Brd9	chr13	+	NM_0010	P22771	protein_cc	AL022779	bromodor	Brd9
5517	Zdhhc11	1.000	1.000	1.000	1.000	1.000	1.000	0.000	0.000	0.000	71164	Zdhhc11	chr13	+	NM_0277	P9050	protein_cc	4933421L	zinc finger	Zdhhc11
5518	Tppp	0.266	0.463	0.123	0.005	0.290	0.019	6.311	4.402	3.292	72948	Tppp	chr13	+	NM_1828	P8883	protein_cc	2900041A	tubulin po	Tppp
5519	Cep72	0.455	2.607	1.186	0.001	0.048	0.713	1.180	0.043	1.426	74470	Cep72	chr13	-	NM_0289	P23600	protein_cc	2610029E	centrosom	Cep72
5520	Slc9a3	1.000	1.000	1.000	1.000	1.000	1.000	0.000	0.000	0.000	105243	Slc9a3	chr13	+	NM_0010	P3116	protein_cc	9030624O	solute car	Slc9a3
5521	Exoc3	1.552	0.771	1.196	0.026	0.109	0.211	7.983	8.617	8.242	211446	Exoc3	chr13	-	NM_1773	P16640	protein_cc	2810050O	exocyst co	Exoc3
5522	Ahrr	0.801	1.166	0.934	0.640	0.734	0.930	1.003	0.682	0.904	11624	Ahrr	chr13	-	NM_0096	P1058	protein_cc	mKIAA123	aryl-hydro	Ahrr
5523	Pdcd6	0.719	1.312	0.944	0.004	0.005	0.357	9.471	8.996	9.388	18570	Pdcd6	chr13	-	NM_0110	P22974	protein_cc	alg-2| Alg	programm	Pdcd6
5524	Sdha	0.517	1.159	0.599	0.000	0.257	0.056	11.714	10.762	10.975	66945	Sdha	chr13	-	NM_0232	P17460	protein_cc	1500032O	succinate	Sdha
5525	Ccdc127	0.705	0.752	0.530	0.289	0.523	0.047	5.388	4.883	4.473	67433	Ccdc127	chr13	+	NM_0011	P18757	protein_cc	0610011N	coiled-coil	Ccdc127
5526	Lrrc14b	1.083	1.141	1.236	0.880	0.739	0.697	4.598	4.714	4.904	432779	Lrrc14b	chr13	-	NM_0010	P2329	protein_cc	AI595366	leucine ric	Lrrc14b
5527	Zfp72	3.892	0.358	1.395	0.340	0.407	0.617	0.388	2.348	0.868	238722	Zfp72	chr13	-	NM_0010	P15031	protein_cc	Zfp74	zinc finger	Zfp72
5528	Zfp825	1.203	0.683	0.822	0.527	0.317	0.566	6.018	6.285	5.735	235956	Zfp825	chr13	-	NM_1462	P24961	protein_cc	.	zinc finger	Zfp825
5529	Erap1	1.471	0.843	1.240	0.066	0.307	0.240	7.753	8.310	8.064	80898	Erap1	chr13	+	NM_0307	P13600	protein_cc	Arts1| ER/	endoplasn	Erap1
5530	Cast	1.755	1.105	1.940	0.008	0.626	0.227	7.725	8.536	8.680	12380	Cast	chr13	-	NM_0013	P2845	protein_cc	.	calpastati	Cast
5531	Pcsk1	3.329	0.300	1.000	0.000	1.000	0.423	0.001	1.736	0.000	18548	Pcsk1	chr13	+	NM_0136	P12759	protein_cc	Nec-1| Ne	proprotei	Pcsk1
5532	Ell2	1.961	0.495	0.970	0.035	0.026	0.914	9.222	10.193	9.179	192657	Ell2	chr13	+	NM_1389	P14393	protein_cc	.	elongatior	Ell2
5533	Glrx	1.996	0.959	1.914	0.000	0.847	0.296	8.791	9.788	9.727	93692	Glrx	chr13	+	NM_0531	P21242	protein_cc	C86710| D	glutaredox	Glrx
5534	Rhobtb3	1.499	1.110	1.663	0.228	0.051	0.371	5.039	5.623	5.773	73296	Rhobtb3	chr13	-	NM_0284	P12323	protein_cc	1700040C	Rho-relate	Rhobtb3
5535	Spata9	2.134	0.469	1.000	0.305	1.000	0.423	0.000	1.094	0.000	75571	Spata9	chr13	+	NM_0293	P21925	protein_cc	1700030K	spermatog	Spata9
5536	Rfesd	0.596	1.082	0.644	0.218	0.828	0.386	6.999	6.251	6.365	218341	Rfesd	chr13	-	NM_0011	P19139	protein_cc	AI256775|	Rieske (Fe	Rfesd
5537	Gpr150	1.581	0.906	1.432	0.301	0.802	0.423	0.000	0.661	0.519	238725	Gpr150	chr13	-	NM_1754	P1388	protein_cc	C030001A	G protein-	Gpr150
5538	Arsk	0.389	1.943	0.755	0.009	0.047	0.020	6.148	4.785	5.743	77041	Arsk	chr13	-	NM_0298	P9649	protein_cc	2810429K	arylsulfata	Arsk

5539	Ttc37	2.097	0.558	1.169	0.018	0.003	0.730	3.773	4.841	3.998	218343	Ttc37	chr13	+	NM_0010	P5509	protein_cc	mKIAA037	tetratricop	Ttc37
5540	Mctp1	2.343	0.907	2.126	0.208	0.873	0.552	0.760	1.989	1.848	78771	Mctp1	chr13	+	NM_0301	P14541	protein_cc	2810465F	multiple C	Mctp1
5541	Ankrd32	2.376	0.492	1.168	0.035	0.042	0.672	4.768	6.016	4.992	.	Ankrd32	chr13	-	NM_1340	P25726	.	.	.	Ankrd32
5542	2210408I2	0.434	3.878	1.682	0.002	0.000	0.001	3.785	2.579	4.535	72371	2210408I2	chr13	+	NM_0011	P2237	protein_cc	.	RIKEN cDN	2210408I2
5543	Fam172a	0.568	1.683	0.955	0.004	0.000	0.724	6.956	6.139	6.890	68675	Fam172a	chr13	+	NM_0011	P22954	protein_cc	1110033N	family wit	Fam172a
5544	Pou5f2	2.681	0.198	0.531	0.419	0.301	0.598	1.975	3.398	1.061	75507	Pou5f2	chr13	+	NM_0293	P20863	protein_cc	1700013G	POU doma	Pou5f2
5545	Nr2f1	0.452	2.398	1.085	0.085	0.094	0.789	4.315	3.170	4.432	13865	Nr2f1	chr13	-	NM_0101	P7455	protein_cc	COUP-TF1	nuclear re	Nr2f1
5546	A830082K	0.162	2.882	0.468	0.181	0.080	0.604	2.705	0.081	1.608	320174	A830082K	chr13	+	NR_04519	.	lncRNA	.	RIKEN cDN	A830082K
5547	Arrdc3	0.101	1.876	0.190	0.000	0.175	0.032	9.317	6.013	6.921	105171	Arrdc3	chr13	+	NM_0010	P12590	protein_cc	AI450344	arrestin do	Arrdc3
5548	5430425K	1.010	1.885	1.904	0.000	0.182	0.423	0.000	0.015	0.929	71416	5430425K	chr13	+	NR_10355	.	lncRNA	.	RIKEN cDN	5430425K
5549	9330111N	1.000	1.000	1.000	1.000	1.000	1.000	0.000	0.000	0.000	319983	9330111N	chr13	+	NR_01558	.	lncRNA	.	RIKEN cDN	9330111N
5550	Adgrv1	0.088	2.679	0.237	0.003	0.000	0.081	3.613	0.113	1.535	110789	Adgrv1	chr13	-	NM_0540	P2085	protein_cc	Frings| Gp	adhesion G	Adgrv1
5551	Lysmd3	2.375	0.568	1.349	0.012	0.029	0.175	7.623	8.871	8.055	80289	Lysmd3	chr13	+	NM_0302	P1031	protein_cc	1110030H	LysM, puta	Lysmd3
5552	Polr3g	0.261	1.750	0.457	0.001	0.302	0.198	7.069	5.130	5.938	67486	Polr3g	chr13	-	NM_0010	P20597	protein_cc	2310047G	polymeras	Polr3g
5553	Mblac2	0.086	4.663	0.400	0.000	0.056	0.101	6.720	3.178	5.399	72852	Mblac2	chr13	+	NM_0283	P13372	protein_cc	2900024O	metallo-be	Mblac2
5554	Cetn3	1.176	1.022	1.203	0.345	0.877	0.140	10.699	10.933	10.965	12626	Cetn3	chr13	+	NM_0076	P14873	protein_cc	MmCEN3	centrin 3	Cetn3
5555	Mef2c	0.480	1.170	0.562	0.008	0.622	0.284	7.236	6.177	6.404	17260	Mef2c	chr13	+	NM_0252	P3397	protein_cc	5430401D	myocyte e	Mef2c
5556	C130071C	1.000	1.000	1.000	1.000	1.000	1.000	0.000	0.000	0.000	320203	C130071C	chr13	+	NR_01556	.	lncRNA	2810449C	RIKEN cDN	C130071C
5557	2810049E	1.000	1.000	1.000	1.000	1.000	1.000	0.000	0.000	0.000	72677	2810049E	chr13	+	NR_03659	.	lncRNA	.	RIKEN cDN	2810049E
5558	Gm17750	1.000	1.000	1.000	1.000	1.000	1.000	0.000	0.000	0.000	553095	Gm17750	chr13	-	NR_13122	.	lncRNA	.	predicted	Gm17750
5559	Tmem161	1.196	0.836	1.000	0.371	0.385	0.997	6.666	6.924	6.666	72745	Tmem161	chr13	+	NM_1751	P15924	protein_cc	2810446P	transmem	Tmem161
5560	Ccnh	0.681	0.985	0.671	0.004	0.801	0.045	7.713	7.160	7.138	66671	Ccnh	chr13	+	NM_0232	P6648	protein_cc	6330408H	cyclin H	Ccnh
5561	Rasa1	1.173	0.903	1.059	0.321	0.464	0.652	8.776	9.007	8.860	218397	Rasa1	chr13	-	NM_1454	P14826	protein_cc	Gap| Rasa	RAS p21 p	Rasa1
5562	Cox7c	0.812	1.133	0.920	0.478	0.662	0.666	11.703	11.403	11.583	12867	Cox7c	chr13	-	NM_0077	P4052	protein_cc	Cox7c1| C	cytochrom	Cox7c
5563	Edil3	1.000	1.000	1.000	1.000	1.000	1.000	0.000	0.000	0.000	13612	Edil3	chr13	+	NM_0101	P3424	protein_cc	Del-1| Del	EGF-like re	Edil3
5564	Hapln1	1.000	1.000	1.000	1.000	1.000	1.000	0.000	0.000	0.000	12950	Hapln1	chr13	+	NM_0135	P11690	protein_cc	BB099155	hyalurona	Hapln1
5565	Vcan	30.549	1.060	32.392	0.001	0.613	0.004	1.440	6.373	6.458	13003	Vcan	chr13	-	NM_0010	P25945	protein_cc	5430420N	versican	Vcan
5566	Xrcc4	2.199	0.689	1.514	0.007	0.033	0.093	5.070	6.207	5.669	108138	Xrcc4	chr13	-	NM_0280	P12100	protein_cc	2310057B	X-ray repa	Xrcc4
5567	Tmem167	2.757	0.860	2.371	0.005	0.312	0.041	8.893	10.356	10.138	66074	Tmem167	chr13	+	NM_0253	P20404	protein_cc	0610041E	transmem	Tmem167
5568	Atp6ap1l	1.000	1.000	1.000	1.000	1.000	1.000	0.000	0.000	0.000	435376	Atp6ap1l	chr13	-	NM_0011	P5895	protein_cc	EG435376	ATPase, H	Atp6ap1l
5569	Rps23	1.044	1.419	1.482	0.564	0.010	0.091	11.865	11.927	12.432	66475	Rps23	chr13	+	NM_0241	P17918	protein_cc	2410044J1	ribosomal	Rps23
5570	Atg10	0.568	1.548	0.879	0.001	0.002	0.349	6.799	5.983	6.613	66795	Atg10	chr13	-	NM_0257	P10667	protein_cc	5330424L	autophagy	Atg10
5571	A830009L	1.000	1.000	1.000	1.000	1.000	1.000	0.000	0.000	0.000	320297	A830009L	chr13	-	NR_04516	.	lncRNA	.	RIKEN cDN	A830009L
5572	Ssbp2	1.301	0.883	1.148	0.689	0.856	0.854	4.226	4.606	4.426	66970	Ssbp2	chr13	+	NM_0241	P23686	protein_cc	1500004K	single-stra	Ssbp2
5573	4833422C	0.175	3.948	0.690	0.000	0.002	0.145	4.946	2.428	4.410	373852	4833422C	chr13	-	NR_01550	.	lncRNA	.	RIKEN cDN	4833422C
5574	Acot12	0.069	3.158	0.217	0.000	0.006	0.000	9.507	5.646	7.305	74156	Acot12	chr13	+	NM_0287	P7169	protein_cc	1300004O	acyl-CoA t	Acot12
5575	Zcchc9	2.494	1.046	2.610	0.002	0.749	0.081	6.519	7.838	7.903	69085	Zcchc9	chr13	-	NM_1454	P21010	protein_cc	1810019C	zinc finger	Zcchc9
5576	Ckmt2	0.552	0.990	0.547	0.182	1.000	0.423	0.871	0.015	0.000	76722	Ckmt2	chr13	-	NM_1984	P7284	protein_cc	2300008A	creatine k	Ckmt2
5577	Rasgrf2	0.118	0.943	0.112	0.000	1.000	0.000	3.164	0.085	0.000	19418	Rasgrf2	chr13	-	NM_0090	P3631	protein_cc	6330417G	RAS prote	Rasgrf2
5578	Msh3	0.315	0.957	0.302	0.003	0.853	0.067	7.040	5.375	5.312	17686	Msh3	chr13	-	NM_0108	P5352	protein_cc	D13Em1|	mutS hom	Msh3
5579	Dhfr	0.151	2.486	0.376	0.000	0.002	0.020	9.565	6.840	8.154	13361	Dhfr	chr13	+	NM_0100	P22786	protein_cc	8430436I	dihydrofol	Dhfr
5580	Ankrd34b	2.700	0.370	1.000	0.305	1.000	0.423	0.001	1.433	0.000	218440	Ankrd34b	chr13	+	NM_1754	P24240	protein_cc	6430502N	ankyrin re	Ankrd34b
5581	Fam151b	0.563	0.840	0.473	0.024	0.588	0.101	6.052	5.224	4.972	73942	Fam151b	chr13	-	NM_0011	P15626	protein_cc	4930405N	family wit	Fam151b
5582	Zfyve16	2.063	0.895	1.846	0.007	0.556	0.296	4.680	5.725	5.565	218441	Zfyve16	chr13	-	NM_1733	P24486	protein_cc	AI035632|	zinc finger	Zfyve16
5583	Spz1	1.000	1.000	1.000	1.000	1.000	1.000	0.000	0.000	0.000	79401	Spz1	chr13	-	NM_0302	P18883	protein_cc	1700027N	spermatog	Spz1
5584	Serinc5	1.189	0.898	1.068	0.002	0.052	0.399	7.489	7.738	7.583	218442	Serinc5	chr13	+	NM_1725	P21883	protein_cc	A130038L	serine inc	Serinc5
5585	Thbs4	1.615	2.150	3.472	0.287	0.175	0.273	0.001	0.692	1.796	21828	Thbs4	chr13	-	NM_0115	P23292	protein_cc	TSP4	thrombos	Thbs4
5586	Mtx3	0.430	1.123	0.483	0.001	0.403	0.018	5.432	4.215	4.381	382793	Mtx3	chr13	+	NM_0011	P24336	protein_cc	4930470O	metaxin 3	Mtx3
5587	Cmya5	1.146	0.632	0.725	0.729	1.000	0.422	0.465	0.661	0.000	76469	Cmya5	chr13	-	NM_0238	P19197	protein_cc	2310076E	cardiomyo	Cmya5
5588	Gm4814	1.000	1.000	1.000	1.000	1.000	1.000	0.000	0.000	0.000	10050294	Gm4814	chr13	+	NR_03645	.	lncRNA	C920025J	predicted	Gm4814
5589	A630019I	0.716	0.995	0.712	0.182	1.000	0.423	0.490	0.008	0.000	408254	A630019I	chr13	+	NR_04618	.	lncRNA	.	RIKEN cDN	A630019I
5590	Papd4	1.844	0.904	1.667	0.177	0.771	0.357	4.566	5.449	5.303	100715	Papd4	chr13	-	NM_1339	P18709	protein_cc	8030446C	PAP assoc	Papd4
5591	Homer1	0.650	1.706	1.109	0.001	0.008	0.562	6.156	5.534	6.305	26556	Homer1	chr13	+	NM_0119	P26425	protein_cc	homer-1|	homer sca	Homer1
5592	Jmy	0.491	1.121	0.550	0.001	0.334	0.012	8.125	7.099	7.263	57748	Jmy	chr13	-	NM_0213	P18982	protein_cc	.	junction-m	Jmy
5593	Bhmt	0.684	0.685	0.469	0.021	0.242	0.178	14.181	13.632	13.087	12116	Bhmt	chr13	-	NM_0166	P4131	protein_cc	.	betaine-h	Bhmt
5594	Bhmt2	0.095	2.831	0.268	0.000	0.023	0.012	10.297	6.894	8.396	64918	Bhmt2	chr13	-	NM_0228	P15119	protein_cc	C81077| D	betaine-h	Bhmt2
5595	Dmgdh	0.473	0.969	0.458	0.002	0.739	0.035	11.386	10.306	10.260	74129	Dmgdh	chr13	+	NM_0287	P20971	protein_cc	1200014D	dimethylg	Dmgdh
5596	AW49522	0.356	1.660	0.590	0.040	0.408	0.484	2.209	0.718	1.449	105364	AW49522	chr13	-	NR_04508	.	lncRNA	.	expressed	AW49522
5597	Arsb	0.449	0.989	0.444	0.001	0.951	0.019	6.631	5.475	5.459	11881	Arsb	chr13	+	NM_0097	P6484	protein_cc	1110007C	arylsulfata	Arsb
5598	Lhfpl2	0.314	0.177	0.056	0.000	0.000	0.011	6.458	4.788	2.293	218454	Lhfpl2	chr13	+	NM_1725	P6006	protein_cc	6030465B	lipoma HN	Lhfpl2
5599	Scamp1	1.172	1.654	1.939	0.012	0.019	0.102	9.523	9.752	10.478	107767	Scamp1	chr13	-	NM_0291	P10390	protein_cc	4930505N	secretory	Scamp1
5600	Gm9776	1.460	1.570	2.292	0.276	0.268	0.277	3.022	3.568	4.219	328309	Gm9776	chr13	-	NR_04561	.	lncRNA	A530089L	predicted	Gm9776
5601	Ap3b1	0.986	0.535	0.528	0.808	0.000	0.036	8.766	8.745	7.843	11774	Ap3b1	chr13	+	NM_0096	P21460	protein_cc	AP-3| AUC	adaptor-re	Ap3b1
5602	Tbca	1.065	1.326	1.412	0.422	0.002	0.051	10.896	10.988	11.394	21371	Tbca	chr13	+	NM_0093	P22783	protein_cc	Tbca13	tubulin co	Tbca
5603	Otp	1.000	1.000	1.000	1.000	1.000	1.000	0.000	0.000	0.000	18420	Otp	chr13	+	NM_0110	P16893	protein_cc	.	orthopedi	Otp
5604	Wdr41	0.791	1.478	1.169	0.161	0.000	0.484	6.865	6.527	7.090	218460	Wdr41	chr13	+	NM_1725	P18067	protein_cc	B830029I	WD repea	Wdr41
5605	Pde8b	0.204	2.034	0.414	0.000	0.001	0.002	4.722	2.426	3.450	218461	Pde8b	chr13	-	NM_0011	P19190	protein_cc	B230331L	phosphod	Pde8b
5606	Zbed3	1.614	0.826	1.333	0.040	0.313	0.563	3.993	4.684	4.407	72114	Zbed3	chr13	-	NM_0013	P4373	protein_cc	0610037K	zinc finger	Zbed3
5607	Snora47	7.118	1.539	10.957	0.240	0.594	0.344	1.237	4.068	4.690	10021745	Snora47	chr13	+	NR_03404	.	snoRNA	MBI-115	small nucl	Snora47
5608	Aggf1	0.964	0.847	0.817	0.181	0.007	0.039	8.536	8.482	8.243	66549	Aggf1	chr13	-	NM_0256	P11621	protein_cc	2010009L	angiogeni	Aggf1
5609	Crhbp	1.000	1.000	1.000	1.000	1.000	1.000	0.000	0.000	0.000	12919	Crhbp	chr13	-	NM_1984	P990	protein_cc	CRH-BP	corticotro	Crhbp
5610	S100z	1.000	1.000	1.000	1.000	1.000	1.000	0.000	0.000	0.000	268686	S100z	chr13	-	NM_0010	P2327	protein_cc	Gm625	S100 calci	S100z
5611	F2rl1	0.646	0.614	0.397	0.535	0.451	0.486	1.762	1.132	0.428	14063	F2rl1	chr13	-	NM_0079	P25101	protein_cc	Gpcr11| P	coagulatio	F2rl1
5612	F2r	1.077	1.982	2.135	0.338	0.018	0.121	8.234	8.341	9.328	14062	F2r	chr13	-	NM_0101	P6694	protein_cc	AI482343|	coagulatio	F2r
5613	Iqgap2	0.912	0.702	0.640	0.124	0.002	0.015	12.373	12.239	11.729	544963	Iqgap2	chr13	-	NM_0277	P16688	protein_cc	4933417J	IQ motif c	Iqgap2
5614	F2rl2	1.453	1.006	1.462	0.502	0.991	0.740	1.849	2.388	2.397	14064	F2rl2	chr13	+	NM_0101	P5790	protein_cc	F730031A	coagulatio	F2rl2
5615	Sv2c	2.180	1.064	2.319	0.302	0.919	0.423	0.001	1.125	1.214	75209	Sv2c	chr13	-	NM_0292	P23256	protein_cc	4930517L	synaptic v	Sv2c
5616	Poc5	1.753	0.965	1.691	0.000	0.834	0.267	4.644	5.453	5.402	67463	Poc5	chr13	+	NM_0261	P25908	protein_cc	1200014N	POC5 cent	Poc5
5617	Ankdd1b	0.177	5.877	1.038	0.183	0.000	0.968	2.603	0.102	2.657	271144	Ankdd1b	chr13	-	NM_0010	P4318	protein_cc	9330128J1	ankyrin re	Ankdd1b
5618	Polk	3.295	0.380	1.252	0.001	0.002	0.458	5.674	7.394	5.998	27015	Polk	chr13	-	NM_0120	P4069	protein_cc	Dinb1	polymeras	Polk
5619	Col4a3bp	0.588	0.735	0.432	0.022	0.235	0.003	8.320	7.555	7.110	68018	Col4a3bp	chr13	+	NM_0011	P1784	protein_cc	2810404O	collagen, t	Col4a3bp
5620	Hmgcr	0.730	1.694	1.237	0.000	0.002	0.159	9.394	8.940	9.701	15357	Hmgcr	chr13	-	NM_0082	P17419	protein_cc	HMG-CoA	3-hydroxy	Hmgcr
5621	Gcnt4	1.000	0.947	0.947	0.998	0.812	0.876	5.639	5.639	5.561	218476	Gcnt4	chr13	+	NM_0011	P1841	protein_cc	C2gnt3| G	glucosami	Gcnt4
5622	1700029F	1.000	1.000	1.000	1.000	1.000	1.000	0.000	0.000	0.000	66479	1700029F	chr13	-	NR_10427	.	protein_cc	.	RIKEN cDN	1700029F
5623	Fam169a	0.891	1.293	1.152	0.325	0.015	0.506	6.665	6.498	6.869	320557	Fam169a	chr13	+	NM_0011	P18823	protein_cc	B230112C	family wit	Fam169a
5624	Nsa2	1.234	0.988	1.218	0.034	0.851	0.074	10.265	10.568	10.550	59050	Nsa2	chr13	-	NM_0215	P13904	protein_cc	5730427N	NSA2 ribo	Nsa2
5625	Gfm2	0.631	0.761	0.480	0.002	0.042	0.047	7.995	7.331	6.937	320806	Gfm2	chr13	+	NM_0011	P14816	protein_cc	6530419G	G elongati	Gfm2
5626	Hexb	0.466	0.645	0.301	0.000	0.001	0.008	7.925	6.825	6.191	15212	Hexb	chr13	-	NM_0104	P23478	protein_cc	.	hexosamin	Hexb
5627	Enc1	16.053	0.394	6.317	0.001	0.010	0.068	6.742	10.747	9.402	13803	Enc1	chr13	+	NM_0079	P15723	protein_cc	Nrpb| PIG	ectoderm	Enc1
5628	Lincenc1	91.005	1.655	150.649	0.013	0.140	0.065	0.003	6.511	7.238	.	Lincenc1	chr13	+	NR_11043	.	.	.	.	Lincenc1
5629	Gm5086	1.000	1.000	1.000	1.000	1.000	1.000	0.000	0.000	0.000	328314	Gm5086	chr13	+	NR_04615	.	lncRNA	B230311A	predicted	Gm5086

5630	Arhgef28	0.293	1.032	0.303	0.223	0.957	0.457	3.277	1.507	1.553	110596	Arhgef28	chr13	-	NM_0120	P1479	protein_cc	9230110L	Rho guani	Arhgef28
5631	Utp15	1.389	0.912	1.267	0.031	0.362	0.385	6.221	6.696	6.563	105372	Utp15	chr13	-	NM_1789	P6640	protein_cc	AW54486	UTP15 sm	Utp15
5632	Ankra2	1.120	0.842	0.944	0.202	0.055	0.738	7.965	8.129	7.882	68558	Ankra2	chr13	+	NM_0012	P15463	protein_cc	1110004N	ankyrin re	Ankra2
5633	Btf3	1.248	1.219	1.521	0.001	0.001	0.014	9.191	9.511	9.796	218490	Btf3	chr13	-	NM_1454	P19934	protein_cc	1700054E	basic trans	Btf3
5634	Foxd1	1.000	1.000	1.000	1.000	1.000	1.000	0.000	0.000	0.000	15229	Foxd1	chr13	+	NM_0082	P13025	protein_cc	AI385632	forkhead	Foxd1
5635	2310005E	1.000	1.000	1.000	1.000	1.000	1.000	0.000	0.000	0.000	70090	2310005E	chr13	-	NR_04549	.	lncRNA	.	RIKEN cDN	2310005E1
5636	Tmem174	1.000	1.000	1.000	1.000	1.000	1.000	0.000	0.000	0.000	68344	Tmem174	chr13	-	NM_0266	P11653	protein_cc	0610009B	transmem	Tmem174
5637	Tmem171	4.859	0.288	1.398	0.117	0.153	0.612	0.378	2.659	0.861	380863	Tmem171	chr13	-	NM_0010	P2939	protein_cc	Gm905	transmem	Tmem171
5638	Fcho2	0.827	0.995	0.824	0.010	0.953	0.243	7.834	7.561	7.554	218503	Fcho2	chr13	-	NM_1725	P6114	protein_cc	5832424M	FCH doma	Fcho2
5639	Tnpo1	1.944	0.606	1.179	0.132	0.194	0.369	5.921	6.880	6.158	238799	Tnpo1	chr13	-	NM_1787	P11307	protein_cc	AU021749	transporti	Tnpo1
5640	1700024P	1.000	1.000	1.000	1.000	1.000	1.000	0.000	0.000	0.000	69382	1700024P	chr13	-	NM_0270	P1227	protein_cc	H2bl1 | Su	RIKEN cDN	1700024P(
5641	2310020H	1.000	1.000	1.000	1.000	1.000	1.000	0.000	0.000	0.000	69603	2310020H	chr13	-	NR_04549	.	lncRNA	.	RIKEN cDN	2310020H(
5642	Zfp366	1.000	1.000	1.000	1.000	1.000	1.000	0.000	0.000	0.000	238803	Zfp366	chr13	+	NM_0010	P23663	protein_cc	DC-SCRIPT	zinc finger	Zfp366
5643	Ptcd2	1.057	1.160	1.227	0.690	0.012	0.399	7.934	8.015	8.229	68927	Ptcd2	chr13	-	NM_0268	P23688	protein_cc	1190005P	pentatrico	Ptcd2
5644	Mrps27	0.580	1.410	0.818	0.073	0.174	0.457	7.774	6.989	7.484	218506	Mrps27	chr13	+	NM_1737	P15694	protein_cc	2610028H	mitochond	Mrps27
5645	6430562O	1.589	1.138	1.809	0.298	0.679	0.003	0.000	0.669	0.855	320893	6430562O	chr13	-	NR_01551	.	lncRNA	.	RIKEN cDN	6430562O
5646	Map1b	2.665	0.774	2.062	0.000	0.003	0.017	4.451	5.865	5.495	17755	Map1b	chr13	-	NM_0086	P2288	protein_cc	A230055D	microtubu	Map1b
5647	Cartpt	1.000	1.000	1.000	1.000	1.000	1.000	0.000	0.000	0.000	27220	Cartpt	chr13	-	NM_0010	P20471	protein_cc	Cart	CART prep	Cartpt
5648	Mccc2	0.446	0.876	0.391	0.014	0.580	0.064	7.577	6.413	6.222	78038	Mccc2	chr13	-	NM_0300	P23720	protein_cc	4930552N	methylcro	Mccc2
5649	Bdp1	1.341	0.522	0.700	0.003	0.001	0.095	8.273	8.696	7.758	544971	Bdp1	chr13	-	NM_0010	P6988	protein_cc	AI662220 |	B double p	Bdp1
5650	Serf1	0.637	0.893	0.569	0.020	0.588	0.191	7.278	6.628	6.466	20365	Serf1	chr13	+	NM_0113	P18714	protein_cc	4F5 | m4F	small EDR	Serf1
5651	Smn1	2.337	0.891	2.082	0.001	0.605	0.277	7.212	8.436	8.270	20595	Smn1	chr13	+	NM_0012	P20636	protein_cc	AI849087 |	survival m	Smn1
5652	Naip2	1.987	1.114	2.213	0.181	0.727	0.114	4.601	5.592	5.747	17948	Naip2	chr13	-	NM_0108	P16006	protein_cc	Birc1b | Na	NLR family	Naip2
5653	Naip5	1.129	1.247	1.408	0.849	0.672	0.710	3.327	3.502	3.820	17951	Naip5	chr13	-	NM_0108	P3022	protein_cc	Birc1e | Lg	NLR family	Naip5
5654	Naip6	1.286	0.897	1.153	0.577	0.820	0.841	2.563	2.926	2.769	17952	Naip6	chr13	-	NM_0108	P10677	protein_cc	Birc1f | Na	NLR family	Naip6
5655	Naip7	0.726	1.425	1.034	0.187	0.186	0.943	0.476	0.015	0.525	.	Naip7	chr13	-	NM_0215	P2654	.	.	.	Naip7
5656	Naip1	4.586	1.032	4.734	0.149	0.955	0.248	0.001	2.199	2.244	17940	Naip1	chr13	-	NM_0086	P7369	protein_cc	AV364616	NLR family	Naip1
5657	Gtf2h2	1.180	1.026	1.210	0.355	0.873	0.151	5.951	6.189	6.226	23894	Gtf2h2	chr13	-	NM_0220	P21074	protein_cc	44kDa | BT	general tra	Gtf2h2
5658	Ocln	0.389	0.472	0.184	0.010	0.086	0.049	5.799	4.436	3.354	18260	Ocln	chr13	-	NM_0087	P12256	protein_cc	AI503564 |	occludin	Ocln
5659	Marveld2	0.642	1.180	0.757	0.004	0.169	0.053	6.966	6.326	6.565	218518	Marveld2	chr13	-	NM_1784	P14320	protein_cc	BC003296	MARVEL (	Marveld2
5660	Rad17	1.896	0.948	1.798	0.000	0.424	0.050	7.716	8.639	8.563	19356	Rad17	chr13	-	NM_0012	P26544	protein_cc	MmRad24	RAD17 che	Rad17
5661	Ak6	1.945	0.593	1.154	0.008	0.014	0.104	7.862	8.822	8.068	10221627	Ak6	chr13	+	NM_0275	P11784	protein_cc	2810046E	adenylate	Ak6
5662	Taf9	2.359	1.412	3.332	0.000	0.029	0.039	8.288	9.526	10.024	108143	Taf9	chr13	+	NM_0271	P7632	protein_cc	2310012N	TATA-box	Taf9
5663	Ccdc125	2.257	0.990	2.234	0.000	0.925	0.081	6.140	7.314	7.299	76041	Ccdc125	chr13	+	NM_0011	P5599	protein_cc	5830436D	coiled-coil	Ccdc125
5664	Cdk7	1.947	0.836	1.628	0.019	0.203	0.106	7.178	8.140	7.882	12572	Cdk7	chr13	-	NM_0098	P7833	protein_cc	AI323415 |	cyclin-dep	Cdk7
5665	Mrps36	0.424	1.225	0.519	0.000	0.514	0.155	6.638	5.398	5.691	66128	Mrps36	chr13	-	NM_0011	P3811	protein_cc	1110018B	mitochond	Mrps36
5666	Cenph	1.098	1.033	1.134	0.852	0.795	0.883	2.254	2.388	2.435	26886	Cenph	chr13	-	NM_0218	P25858	protein_cc	1700021I1	centromer	Cenph
5667	Ccnb1	3.477	0.745	2.589	0.173	0.583	0.025	0.384	2.182	1.757	268697	Ccnb1	chr13	-	NM_1723	P15709	protein_cc	Ccnb1-rs1	cyclin B1	Ccnb1
5668	Slc30a5	2.114	0.799	1.689	0.010	0.165	0.039	6.743	7.823	7.499	69048	Slc30a5	chr13	-	NM_0228	P6236	protein_cc	1810010K	solute car	Slc30a5
5669	5930438M	1.000	1.000	1.000	1.000	1.000	1.000	0.000	0.000	0.000	328324	5930438N	chr13	+	NR_04615	.	lncRNA	5930438M	RIKEN cDN	5930438M
5670	4933416M	1.000	1.000	1.000	1.000	1.000	1.000	0.000	0.000	0.000	71174	4933416N	chr13	+	NR_04584	.	lncRNA	.	RIKEN cDN	4933416M
5671	4922502H	1.000	1.000	1.000	1.000	1.000	1.000	0.000	0.000	0.000	78136	4922502H	chr13	-	NR_04618	.	lncRNA	.	RIKEN cDN	4922502H
5672	Pik3r1	1.269	0.891	1.131	0.331	0.573	0.590	9.103	9.447	9.281	18708	Pik3r1	chr13	-	NM_0010	P20599	protein_cc	p50alpha |	phosphati	Pik3r1
5673	Cd180	0.457	1.401	0.640	0.183	0.194	0.600	1.158	0.027	0.514	17079	Cd180	chr13	+	NM_0085	P19038	protein_cc	F630107B	CD180 ant	Cd180
5674	Mast4	1.583	0.745	1.179	0.138	0.326	0.610	5.519	6.182	5.756	328329	Mast4	chr13	-	NM_1751	P629	protein_cc	4930420O	microtubu	Mast4
5675	1700099I0	3.635	0.110	0.399	0.399	1.000	0.423	1.327	3.189	0.000	76624	1700099I0	chr13	+	NR_13031	.	lncRNA	.	RIKEN cDN	1700099I0
5676	Srek1	1.117	0.425	0.475	0.291	0.000	0.090	8.752	8.912	7.677	218543	Srek1	chr13	-	NM_1725	P20098	protein_cc	8430401B	splicing re	Srek1
5677	Erbb2ip	1.117	0.833	0.931	0.160	0.003	0.597	8.169	8.328	8.065	59079	Erbb2ip	chr13	-	NM_0010	P4122	protein_cc	1700028E	Erbb2 inte	Erbb2ip
5678	Nln	0.983	1.079	1.061	0.896	0.380	0.736	6.626	6.601	6.711	75805	Nln	chr13	-	NM_0294	P19387	protein_cc	4930472G	neurolysin	Nln
5679	Sgtb	0.160	3.823	0.613	0.002	0.007	0.107	5.349	2.710	4.644	218544	Sgtb	chr13	+	NM_1448	P8043	protein_cc	C630001O	small gluta	Sgtb
5680	Trappc13	1.551	0.688	1.067	0.007	0.000	0.741	7.812	8.445	7.906	66975	Trappc13	chr13	-	NM_0010	P16148	protein_cc	2410002O	trafficking	Trappc13
5681	Trim23	0.183	1.265	0.232	0.000	0.297	0.008	4.003	1.554	1.893	81003	Trim23	chr13	+	NM_0307	P4670	protein_cc	6330516O	tripartite	Trim23
5682	Ppwd1	1.500	0.512	0.768	0.007	0.015	0.446	5.692	6.277	5.310	238831	Ppwd1	chr13	-	NM_1728	P7036	protein_cc	4632422N	peptidylpr	Ppwd1
5683	Cenpk	1.059	0.473	0.501	0.797	0.028	0.267	5.431	5.514	4.434	60411	Cenpk	chr13	+	NM_1810	P16050	protein_cc	A930019D	a disintegr	Cenpk
5684	Adamts6	1.130	0.395	0.447	0.857	0.252	0.552	2.024	2.200	0.862	108154	Adamts6	chr13	+	NM_0010	P8714	protein_cc	A930019D	a disintegr	Adamts6
5685	Cwc27	1.183	0.829	0.980	0.055	0.020	0.838	6.922	7.164	6.893	67285	Cwc27	chr13	-	NM_0260	P26239	protein_cc	3110009E	CWC27 sp	Cwc27
5686	Srek1ip1	0.935	0.891	0.834	0.745	0.606	0.248	8.743	8.647	8.481	67288	Srek1ip1	chr13	+	NM_0260	P3105	protein_cc	3110031B	splicing re	Srek1ip1
5687	Fam159b	1.000	1.000	1.000	1.000	1.000	1.000	0.000	0.000	0.000	77803	Fam159b	chr13	-	NM_0299	P4141	protein_cc	A930021C	family wit	Fam159b
5688	Rgs7bp	0.101	2.480	0.250	0.000	0.107	0.046	4.546	1.234	2.545	52882	Rgs7bp	chr13	-	NM_0298	P24971	protein_cc	A930030I	regulator	Rgs7bp
5689	4933425L	1.000	1.000	1.000	1.000	1.000	1.000	0.000	0.000	0.000	66763	4933425L	chr13	+	NM_0257	P8504	protein_cc	.	RIKEN cDN	4933425L
5690	Rnf180	1.010	1.885	1.904	0.000	0.182	0.423	0.000	0.015	0.929	71816	Rnf180	chr13	-	NM_0279	P18158	protein_cc	3110001E	ring finger	Rnf180
5691	Htr1a	1.000	1.000	1.000	1.000	1.000	1.000	0.000	0.000	0.000	15550	Htr1a	chr13	+	NM_0083	P14954	protein_cc	Gpcr18	5-hydroxy	Htr1a
5692	Ipo11	1.981	0.580	1.149	0.001	0.007	0.545	6.141	7.128	6.341	76582	Ipo11	chr13	-	NM_0296	P126	protein_cc	1700081E	importin 1	Ipo11
5693	Dimt1	2.690	1.140	3.066	0.020	0.416	0.170	2.872	4.300	4.489	66254	Dimt1	chr13	+	NM_0254	P9677	protein_cc	1500031N	DIM1 dim	Dimt1
5694	Kif2a	1.384	0.817	1.131	0.003	0.012	0.238	7.082	7.551	7.260	16563	Kif2a	chr13	-	NM_0084	P14159	protein_cc	C530030B	kinesin fa	Kif2a
5695	3830408C	1.000	1.000	1.000	1.000	1.000	1.000	0.000	0.000	0.000	10004032	3830408C	chr13	+	NR_01547	.	lncRNA	.	RIKEN cDN	3830408C
5696	AI197445	0.552	0.990	0.547	0.182	1.000	0.423	0.871	0.015	0.000	105120	AI197445	chr13	+	NR_04508	.	lncRNA	.	expressed	AI197445
5697	1700006H	1.000	1.000	1.000	1.000	1.000	1.000	0.000	0.000	0.000	72257	1700006H	chr13	+	NR_04590	.	lncRNA	.	RIKEN cDN	1700006H
5698	Zswim6	1.231	0.726	0.893	0.001	0.009	0.455	7.052	7.352	6.889	67263	Zswim6	chr13	-	NM_1454	P2731	protein_cc	2900036G	zinc finger	Zswim6
5699	Smim15	1.319	0.780	1.030	0.001	0.019	0.846	8.250	8.650	8.293	75616	Smim15	chr13	+	NM_0010	P1017	protein_cc	2810008N	small integ	Smim15
5700	Ndufaf2	0.702	1.333	0.935	0.006	0.063	0.709	7.522	7.011	7.425	75597	Ndufaf2	chr13	-	NM_0011	P12943	protein_cc	1810058I1	NADH deh	Ndufaf2
5701	Ercc8	2.072	0.577	1.196	0.024	0.020	0.778	4.421	5.472	4.679	71991	Ercc8	chr13	+	NM_0280	P26343	protein_cc	2410022P	excision re	Ercc8
5702	Elovl7	4.158	0.730	3.035	0.079	0.439	0.126	2.630	4.686	4.232	74559	Elovl7	chr13	+	NM_0290	P14420	protein_cc	9130013K	ELOVL fam	Elovl7
5703	Depdc1b	1.000	1.000	1.000	1.000	1.000	1.000	0.000	0.000	0.000	218581	Depdc1b	chr13	+	NM_1786	P18702	protein_cc	9830132O	DEP doma	Depdc1b
5704	Pde4d	0.560	1.501	0.840	0.050	0.138	0.615	5.457	4.619	5.205	238871	Pde4d	chr13	+	NM_0110	P15177	protein_cc	9630011N	phosphod	Pde4d
5705	Rab3c	0.552	2.575	1.422	0.001	0.031	0.439	0.895	0.038	1.403	67295	Rab3c	chr13	-	NM_0238	P16038	protein_cc	2700062I	RAB3C, m	Rab3c
5706	Gapt	1.575	0.466	0.734	0.475	1.000	0.422	0.446	1.101	0.000	238875	Gapt	chr13	-	NM_1777	P10986	protein_cc	9830130N	Grb2-bind	Gapt
5707	Plk2	1.483	0.837	1.241	0.420	0.698	0.603	5.133	5.701	5.444	20620	Plk2	chr13	+	NM_1528	P23765	protein_cc	Snk	polo-like k	Plk2
5708	4930526H	1.000	1.000	1.000	1.000	1.000	1.000	0.000	0.000	0.000	10263614	4930526H	chr13	-	NR_13119	.	lncRNA	.	RIKEN cDN	4930526H
5709	Gm38397	1.000	1.000	1.000	1.000	1.000	1.000	0.000	0.000	0.000	75139	Gm38397	chr13	+	NR_13119	.	lncRNA	.	predicted	Gm38397
5710	3110015C	1.000	1.000	1.000	1.000	1.000	1.000	0.000	0.000	0.000	73129	3110015C	chr13	-	NR_04590	.	lncRNA	.	RIKEN cDN	3110015C
5711	Actbl2	1.000	1.000	1.000	1.000	1.000	1.000	0.000	0.000	0.000	238880	Actbl2	chr13	+	NM_1754	P19557	protein_cc	4732495G	actin, beta	Actbl2
5712	Gpbp1	1.380	0.608	0.839	0.007	0.010	0.395	9.077	9.541	8.824	73274	Gpbp1	chr13	-	NM_0284	P521	protein_cc	1700034P	GC-rich pr	Gpbp1
5713	Mier3	1.595	0.755	1.205	0.003	0.055	0.398	7.929	8.603	8.198	218613	Mier3	chr13	+	NM_1725	P10342	protein_cc	5730509D	MIER fami	Mier3
5714	Map3k1	2.324	0.592	1.376	0.002	0.008	0.212	6.735	7.952	7.196	26401	Map3k1	chr13	-	NM_0119	P7231	protein_cc	MAPKKK1	mitogen-a	Map3k1
5715	Gm15326	1.000	1.000	1.000	1.000	1.000	1.000	0.000	0.000	0.000	10263662	Gm15326	chr13	+	NR_13034	.	lncRNA	OTTMUSG	predicted	Gm15326
5716	Ankrd55	0.361	2.146	0.775	0.061	0.187	0.738	1.514	0.044	1.145	77318	Ankrd55	chr13	+	NM_0298	P12202	protein_cc	C030011J	ankyrin re	Ankrd55
5717	Il6st	2.233	0.616	1.376	0.046	0.115	0.263	7.945	9.103	8.405	16195	Il6st	chr13	+	NM_0105	P8812	protein_cc	5133400A	interleukin	Il6st
5718	Il31ra	1.010	1.885	1.904	0.000	0.182	0.423	0.000	0.015	0.929	218624	Il31ra	chr13	-	NM_1392	P12226	protein_cc	CRL3 | GLN	interleukin	Il31ra
5719	Ddx4	1.014	2.240	2.272	0.000	0.182	0.423	0.000	0.021	1.185	13206	Ddx4	chr13	-	NM_0011	P21688	protein_cc	AV206478	DEAD (Asp	Ddx4
5720	Slc38a9	0.719	1.168	0.840	0.069	0.427	0.378	6.326	5.850	6.075	268706	Slc38a9	chr13	+	NM_1787	P14399	protein_cc	4833412L	solute car	Slc38a9

5721	Ppap2a	1.159	1.277	1.480	0.089	0.065	0.117	8.298	8.511	8.863	.	Ppap2a	chr13	+	NM_0082	P12682	.	.	.	Ppap2a
5722	Skiv2l2	1.346	1.007	1.355	0.000	0.846	0.040	8.332	8.761	8.771	72198	Skiv2l2	chr13	-	NM_0281	P20661	protein_cc	2610528A	superkiller	Skiv2l2
5723	Dhx29	2.056	0.646	1.328	0.020	0.056	0.129	4.201	5.241	4.610	218629	Dhx29	chr13	+	NM_1725	P25074	protein_cc	3732415M	DEAH (Asp	Dhx29
5724	Ccno	0.816	0.732	0.597	0.611	0.227	0.515	4.006	3.713	3.262	218630	Ccno	chr13	+	NM_0010	P10543	protein_cc	C86987 | C	cyclin O	Ccno
5725	Mcidas	1.005	1.405	1.412	0.000	0.182	0.423	0.000	0.008	0.498	622408	Mcidas	chr13	+	NM_0010	P17031	protein_cc	EG622408	multiciliat	Mcidas
5726	Cdc20b	1.000	1.000	1.000	1.000	1.000	1.000	0.000	0.000	0.000	238896	Cdc20b	chr13	+	NM_0012	P4541	protein_cc	EG238896	cell divisio	Cdc20b
5727	Gpx8	0.786	1.310	1.029	0.584	0.513	0.914	4.180	3.832	4.222	69590	Gpx8	chr13	-	NM_0271	P14125	protein_cc	2310016C	glutathion	Gpx8
5728	Gzma	2.886	0.381	1.098	0.020	0.026	0.679	4.285	5.814	4.420	14938	Gzma	chr13	-	NM_0103	P21945	protein_cc	AW49411	granzyme	Gzma
5729	1700084D	1.000	1.000	1.000	1.000	1.000	1.000	0.000	0.000	0.000	76720	1700084D	chr13	+	NR_13104	.	lncRNA	.	RIKEN cDN	1700084D
5730	Gzmk	1.000	1.000	1.000	1.000	1.000	1.000	0.000	0.000	0.000	14945	Gzmk	chr13	-	NM_0081	P8244	protein_cc	.	granzyme	Gzmk
5731	Esm1	3.082	1.569	4.836	0.002	0.227	0.202	2.845	4.469	5.119	71690	Esm1	chr13	+	NM_0236	P11087	protein_cc	0610042H	endothelia	Esm1
5732	4921509O	3.913	0.255	0.999	0.119	1.000	0.423	0.001	1.969	0.000	70917	4921509O	chr13	-	NR_04550	.	lncRNA	.	RIKEN cDN	4921509O
5733	Snx18	2.144	0.833	1.787	0.012	0.264	0.122	7.447	8.547	8.284	170625	Snx18	chr13	-	NM_1307	P5887	protein_cc	Snag1	sorting ne	Snx18
5734	Hspb3	1.000	1.000	1.000	1.000	1.000	1.000	0.000	0.000	0.000	56534	Hspb3	chr13	-	NM_0199	P14426	protein_cc	2310035K	heat shocl	Hspb3
5735	Arl15	3.435	0.586	2.013	0.002	0.002	0.177	5.449	7.229	6.459	218639	Arl15	chr13	+	NM_1725	P16726	protein_cc	A430036I	ADP-ribos	Arl15
5736	A430090L	1.376	0.538	0.740	0.131	0.039	0.148	3.029	3.490	2.595	319803	A430090L	chr13	-	NR_04583	.	lncRNA	.	RIKEN cDN	A430090L
5737	Ndufs4	1.063	1.189	1.264	0.065	0.091	0.227	10.071	10.159	10.409	17993	Ndufs4	chr13	-	NM_0108	P12869	protein_cc	6720411N	NADH deh	Ndufs4
5738	Fst	17.462	0.357	6.239	0.023	0.101	0.339	5.415	9.541	8.057	14313	Fst	chr13	-	NM_0080	P11317	protein_cc	AL033346	follistatin	Fst
5739	4930544M	1.000	1.000	1.000	1.000	1.000	1.000	0.000	0.000	0.000	75161	4930544M	chr13	+	NR_04597	.	lncRNA	.	RIKEN cDN	4930544M
5740	Mocs2	0.477	1.321	0.630	0.000	0.010	0.013	10.481	9.412	9.813	17434	Mocs2	chr13	+	NM_0011	P12958	protein_cc	AI415403	molybden	Mocs2
5741	Itga2	4.612	2.056	9.483	0.141	0.124	0.078	0.002	2.207	3.247	16398	Itga2	chr13	-	NM_0083	P23036	protein_cc	CD49B | D	integrin al	Itga2
5742	Itga1	1.070	0.453	0.485	0.510	0.000	0.090	6.340	6.437	5.296	109700	Itga1	chr13	-	NM_0010	P26896	protein_cc	CD49A | E	integrin al	Itga1
5743	Pelo	2.292	0.891	2.043	0.000	0.014	0.019	7.923	9.119	8.953	105083	Pelo	chr13	-	NM_1340	P20435	protein_cc	AA409897	pelota hor	Pelo
5744	Isl1	1.010	1.885	1.904	0.000	0.182	0.423	0.000	0.015	0.929	16392	Isl1	chr13	-	NM_0214	P1769	protein_cc	.	ISL1 trans	Isl1
5745	Parp8	3.908	0.823	3.215	0.002	0.155	0.056	1.864	3.830	3.548	52552	Parp8	chr13	-	NM_0010	P23941	protein_cc	2810430O	poly (ADP-	Parp8
5746	Gm6416	1.000	1.000	1.000	1.000	1.000	1.000	0.000	0.000	0.000	623312	Gm6416	chr13	+	NR_04602	.	lncRNA	623312	predicted	Gm6416
5747	Emb	3.913	0.821	3.211	0.016	0.396	0.040	2.641	4.609	4.324	13723	Emb	chr13	+	NM_0103	P20616	protein_cc	AL022799	embigin	Emb
5748	Hcn1	0.214	1.303	0.279	0.121	0.219	0.388	2.278	0.054	0.436	15165	Hcn1	chr13	+	NM_0104	P22950	protein_cc	Bcng1 | C6	hyperpola	Hcn1
5749	4933413L	1.000	1.000	1.000	1.000	1.000	1.000	0.000	0.000	0.000	71100	4933413L	chr13	-	NR_04550	.	lncRNA	.	RIKEN cDN	4933413L
5750	Mrps30	1.094	1.088	1.190	0.464	0.519	0.351	8.647	8.777	8.899	59054	Mrps30	chr13	-	NM_0215	P4029	protein_cc	2610020A	mitochon	Mrps30
5751	1700003P	1.000	1.000	1.000	1.000	1.000	1.000	0.000	0.000	0.000	75890	1700003P	chr13	+	NR_04598	.	lncRNA	1700030O	RIKEN cDN	1700003P
5752	Gm10732	1.000	1.000	1.000	1.000	1.000	1.000	0.000	0.000	0.000	10003869	Gm10732	chr13	-	NR_13115	.	lncRNA	ENSMUSG	predicted	Gm10732
5753	Fgf10	0.552	0.990	0.547	0.182	1.000	0.423	0.871	0.015	0.000	14165	Fgf10	chr13	+	NM_0080	P25011	protein_cc	AEY17 | BE	fibroblast	Fgf10
5754	Nnt	0.386	1.192	0.460	0.000	0.125	0.019	10.436	9.063	9.317	18115	Nnt	chr13	-	NM_0087	P24789	protein_cc	4930423F	nicotinam	Nnt
5755	Paip1	1.346	1.128	1.519	0.003	0.026	0.003	8.892	9.320	9.495	218693	Paip1	chr13	+	NM_0010	P7630	protein_cc	PAIP-	polyadeny	Paip1
5756	4833420G	1.473	1.105	1.628	0.168	0.727	0.371	7.503	8.062	8.206	67392	4833420G	chr13	+	NM_0011	P520	protein_cc	2210017J	RIKEN cDN	4833420G
5757	3110070M	1.485	1.038	1.541	0.208	0.547	0.418	3.069	3.639	3.693	67304	3110070M	chr13	-	NR_02797	.	lncRNA	.	RIKEN cDN	3110070M
5758	Gm7120	5.892	0.704	4.149	0.000	0.054	0.060	0.360	2.919	2.413	633640	Gm7120	chr13	+	NM_0010	P3433	protein_cc	EG633640	predicted	Gm7120
5759	Ccl28	0.735	1.867	1.372	0.191	0.184	0.650	0.465	0.021	0.922	56838	Ccl28	chr13	+	NM_0202	P22231	protein_cc	CCK1 | ME	chemokin	Ccl28
5760	1700074H	1.000	1.000	1.000	1.000	1.000	1.000	0.000	0.000	0.000	73480	1700074H	chr13	-	NR_04529	.	lncRNA	CH25-211	RIKEN cDN	1700074H
5761	Hmgcs1	0.787	1.877	1.477	0.001	0.000	0.017	12.236	11.890	12.799	208715	Hmgcs1	chr13	+	NM_1459	P8189	protein_cc	B130032C	3-hydroxy	Hmgcs1
5762	Nim1k	0.332	1.311	0.435	0.003	0.341	0.123	5.899	4.309	4.699	245269	Nim1k	chr13	-	NM_1755	P10275	protein_cc	E130304F	NIM1 seri	Nim1k
5763	Zfp131	1.752	0.863	1.513	0.001	0.015	0.084	7.724	8.533	8.321	72465	Zfp131	chr13	-	NM_0013	P14503	protein_cc	2610109I	zinc finger	Zfp131
5764	AF067063	1.000	1.000	1.000	1.000	1.000	1.000	0.000	0.000	0.000	380878	AF067063	chr13	-	NM_0010	P25508	protein_cc	.	cDNA seq	AF067063
5765	Tcstv1	1.000	1.000	1.000	1.000	1.000	1.000	0.000	0.000	0.000	54382	Tcstv1	chr13	-	NM_0187	P1343	protein_cc	Lo1	2-cell-stag	Tcstv1
5766	B020031M	1.000	1.000	1.000	1.000	1.000	1.000	0.000	0.000	0.000	333467	B020031M	chr13	-	NM_0010	P19968	protein_cc	.	RIKEN cDN	B020031M
5767	Gm21188	10.572	0.096	1.014	0.010	0.010	0.969	0.350	3.752	0.369	10086175	Gm21188	chr13	-	NM_0013	P4950	protein_cc	.	predicted	Gm21188
5768	Gm20767	1.000	1.000	1.000	1.000	1.000	1.000	0.000	0.000	0.000	639910	Gm20767	chr13	+	NM_0010	P10910	protein_cc	.	predicted	Gm20767
5769	AF067061	1.000	1.000	1.000	1.000	1.000	1.000	0.000	0.000	0.000	236546	AF067061	chr13	+	NM_1990	P12064	protein_cc	.	cDNA seq	AF067061
5770	BC147527	5.294	0.145	0.770	0.000	1.000	0.420	0.377	2.782	0.000	625360	BC147527	chr13	+	NM_0010	P7255	protein_cc	.	cDNA seq	BC147527
5771	Tcstv3	1.000	1.000	1.000	1.000	1.000	1.000	0.000	0.000	0.000	236219	Tcstv3	chr13	+	NM_1535	P15531	protein_cc	.	2-cell-stag	Tcstv3
5772	Gm2897	1.000	1.000	1.000	1.000	1.000	1.000	0.000	0.000	0.000	10004067	Gm2897	chr14	+	NM_0011	P14172	protein_cc	10004067	predicted	Gm2897
5773	Gm5795	1.000	1.000	1.000	1.000	1.000	1.000	0.000	0.000	0.000	544990	Gm5795	chr14	+	NM_0012	P6717	protein_cc	544990	predicted	Gm5795
5774	D830030K	0.534	0.990	0.528	0.000	1.000	0.015	0.921	0.015	0.000	320333	D830030K	chr14	+	NM_1771	P19007	protein_cc	.	RIKEN cDN	D830030K
5775	1700110I	0.726	1.425	1.034	0.187	0.186	0.943	0.476	0.015	0.525	73549	1700110I	chr14	+	NR_03805	.	lncRNA	10004074	RIKEN cDN	1700110I
5776	Gm10413	1.000	1.000	1.000	1.000	1.000	1.000	0.000	0.000	0.000	10004177	Gm10413	chr14	+	NM_0292	P11394	protein_cc	10003884	predicted	Gm10413
5777	Gm3020	1.000	1.000	1.000	1.000	1.000	1.000	0.000	0.000	0.000	10004088	Gm3020	chr14	+	NR_03311	.	misc_RNA	10004088	predicted	Gm3020
5778	Gm10409	0.890	0.626	0.557	0.792	1.000	0.422	0.844	0.677	0.000	10004153	Gm10409	chr14	+	NR_03312	.	misc_RNA	EG666890	predicted	Gm10409
5779	Gm10408	0.901	0.856	0.771	0.812	0.699	0.685	0.834	0.684	0.460	10004184	Gm10408	chr14	+	NM_0012	P22188	protein_cc	10004079	predicted	Gm10408
5780	Gm3002	1.005	1.405	1.412	0.000	0.182	0.423	0.000	0.008	0.498	10004085	Gm3002	chr14	+	NR_03338	.	misc_RNA	10004085	predicted	Gm3002
5781	Gm5796	1.000	1.000	1.000	1.000	1.000	1.000	0.000	0.000	0.000	545007	Gm5796	chr14	+	NM_0010	P6374	protein_cc	545007	predicted	Gm5796
5782	2610042L	1.005	1.439	1.446	0.000	0.182	0.423	0.000	0.008	0.532	554327	2610042L	chr14	+	NM_0013	P19036	protein_cc	takusan | T	RIKEN cDN	2610042L
5783	Gm3264	1.000	1.000	1.000	1.000	1.000	1.000	0.000	0.000	0.000	10004130	Gm3264	chr14	+	NM_0012	P13468	protein_cc	10004130	predicted	Gm3264
5784	4930555G	1.000	1.000	1.000	1.000	1.000	1.000	0.000	0.000	0.000	108978	4930555G	chr14	+	NM_1753	P17962	protein_cc	.	RIKEN cDN	4930555G
5785	Gm3488	1.590	0.875	1.390	0.302	0.737	0.423	0.000	0.669	0.476	10004173	Gm3488	chr14	+	NM_0012	P1631	protein_cc	10004173	predicted	Gm3488
5786	Gm3317	1.000	1.000	1.000	1.000	1.000	1.000	0.000	0.000	0.000	666329	Gm3317	chr14	+	NM_0012	P1631	protein_cc	10004140	predicted	Gm3317
5787	Gm3500	1.000	1.000	1.000	1.000	1.000	1.000	0.000	0.000	0.000	10004167	Gm3500	chr14	-	NM_0012	P9605	protein_cc	10004175	predicted	Gm3500
5788	Gm3383	1.005	1.405	1.412	0.000	0.182	0.423	0.000	0.008	0.498	10004151	Gm3383	chr14	-	NM_0012	P4803	protein_cc	10004151	predicted	Gm3383
5789	LOC10086	1.000	1.000	1.000	1.000	1.000	1.000	0.000	0.000	0.000	10086161	LOC10086	chr14	-	NM_0012	P18107	protein_cc	.	alpha taku	LOC10086
5790	Gm3636	1.010	1.885	1.904	0.000	0.182	0.423	0.000	0.015	0.929	10004115	Gm3636	chr14	-	NM_0013	P11420	protein_cc	10004204	predicted	Gm3636
5791	Gm10406	1.572	0.636	1.000	0.304	1.000	0.423	0.000	0.653	0.000	10003884	Gm10406	chr14	-	NM_0011	P11163	protein_cc	ENSMUSG	predicted	Gm10406
5792	Gm3696	1.000	1.000	1.000	1.000	1.000	1.000	0.000	0.000	0.000	10004214	Gm3696	chr14	-	NM_0010	P21843	protein_cc	10004214	predicted	Gm3696
5793	Gm5797	1.000	1.000	1.000	1.000	1.000	1.000	0.000	0.000	0.000	545013	Gm5797	chr14	-	NM_0010	P7542	protein_cc	EG545013	predicted	Gm5797
5794	4933406F	0.716	0.995	0.712	0.182	1.000	0.423	0.490	0.008	0.000	68308	4933406F	chr14	+	NR_01556	.	lncRNA	1700007H	RIKEN cDN	4933406F
5795	Gm3558	1.000	1.000	1.000	1.000	1.000	1.000	0.000	0.000	0.000	10004187	Gm3558	chr14	-	NM_0012	P25929	protein_cc	10004187	predicted	Gm3558
5796	Flnb	1.076	1.087	1.169	0.655	0.636	0.377	7.756	7.862	7.982	286940	Flnb	chr14	+	NM_0010	P14945	protein_cc	AL024016	filamin, be	Flnb
5797	Dnase1l3	0.469	0.534	0.250	0.000	0.017	0.017	10.105	9.013	8.107	13421	Dnase1l3	chr14	-	NM_0078	P13181	protein_cc	Dhp2 | DN	deoxyribo	Dnase1l3
5798	Abhd6	0.259	1.649	0.427	0.003	0.089	0.071	9.830	7.881	8.603	66082	Abhd6	chr14	+	NM_0253	P5787	protein_cc	0610041D	abhydrola	Abhd6
5799	Rpp14	1.125	1.683	1.894	0.564	0.100	0.240	8.103	8.274	9.025	67053	Rpp14	chr14	+	NM_0259	P23129	protein_cc	2610511E	ribonuclea	Rpp14
5800	Pxk	1.837	0.772	1.418	0.004	0.068	0.242	7.021	7.898	7.525	218699	Pxk	chr14	+	NM_1782	P7859	protein_cc	C230080L	PX domair	Pxk
5801	Pdhb	0.590	1.019	0.601	0.035	0.935	0.069	10.436	9.674	9.701	68263	Pdhb	chr14	-	NM_0242	P22033	protein_cc	2610103L	pyruvate c	Pdhb
5802	Kctd6	1.316	0.973	1.280	0.381	0.912	0.308	5.547	5.943	5.903	71393	Kctd6	chr14	+	NM_0277	P13740	protein_cc	5430433B	potassium	Kctd6
5803	Acox2	0.680	1.273	0.866	0.003	0.112	0.453	10.397	9.840	10.189	93732	Acox2	chr14	-	NM_0011	P10645	protein_cc	THCCox	acyl-Coen	Acox2
5804	Fam107a	8.626	0.217	1.875	0.073	0.106	0.576	0.712	3.820	1.619	268709	Fam107a	chr14	-	NM_1831	P17332	protein_cc	DRR1 | RP	family wit	Fam107a
5805	Oit1	0.609	5.415	3.297	0.202	0.008	0.143	0.784	0.068	2.505	18300	Oit1	chr14	-	NM_1460	P20485	protein_cc	2310076N	oncoprote	Oit1
5806	4930452B	0.497	2.609	1.296	0.168	0.042	0.534	2.195	1.185	2.569	74430	4930452B	chr14	-	NM_0289	P5982	protein_cc	4930406L	RIKEN cDN	4930452B
5807	4930455B	0.716	0.995	0.712	0.182	1.000	0.423	0.490	0.008	0.000	74859	4930455B	chr14	+	NR_04596	.	lncRNA	1700027C	RIKEN cDN	4930455B
5808	Fhit	0.485	1.309	0.634	0.003	0.556	0.416	5.168	4.123	4.511	14198	Fhit	chr14	-	NM_0102	P9585	protein_cc	AW04563	fragile hist	Fhit
5809	Mnd1-ps	0.324	1.093	0.354	0.004	0.647	0.077	5.413	3.788	3.916	10004241	Mnd1-ps	chr14	+	NR_03068	.	misc_RNA	10004241	Mnd1 retr	Mnd1-ps
5810	Fhitos	0.165	0.957	0.158	0.139	1.000	0.372	2.666	0.063	0.000	547334	Fhitos	chr14	+	NR_13113	.	lncRNA	Gm16542	fragile hist	Fhitos
5811	Ptprg	0.507	1.011	0.512	0.001	0.933	0.009	8.248	7.267	7.282	19270	Ptprg	chr14	+	NM_0089	P17277	protein_cc	5430405N	protein ty	Ptprg

5812	3830406C	0.760	1.222	0.928	0.012	0.561	0.882	7.475	7.079	7.368	218734	3830406C	chr14	+	NM_1460	P25903	protein_cc	.	RIKEN cDN	3830406C
5813	Fezf2	1.000	1.000	1.000	1.000	1.000	1.000	0.000	0.000	0.000	54713	Fezf2	chr14	-	NM_0804	P19080	protein_cc	AI451466|	Fez family	Fezf2
5814	Cadps	1.000	1.000	1.000	1.000	1.000	1.000	0.000	0.000	0.000	27062	Cadps	chr14	-	NM_0010	P17948	protein_cc	AU067781	Ca2+-depe	Cadps
5815	Gm5087	1.000	1.000	1.000	1.000	1.000	1.000	0.000	0.000	0.000	328354	Gm5087	chr14	-	NR_12158	.	lncRNA	C630025C	predicted	Gm5087
5816	Synpr	1.000	1.000	1.000	1.000	1.000	1.000	0.000	0.000	0.000	72003	Synpr	chr14	+	NM_0280	P6338	protein_cc	1500003F	synaptopo	Synpr
5817	Sntn	1.000	1.000	1.000	1.000	1.000	1.000	0.000	0.000	0.000	218739	Sntn	chr14	+	NM_1776	P9407	protein_cc	A430083B	sentan, cil	Sntn
5818	Thoc7	0.717	1.107	0.794	0.001	0.009	0.046	9.951	9.471	9.618	66231	Thoc7	chr14	-	NM_0254	P8420	protein_cc	Nif3l1bp1	THO comp	Thoc7
5819	Atxn7	1.471	0.500	0.736	0.020	0.002	0.420	8.299	8.855	7.857	246103	Atxn7	chr14	+	NM_1392	P1014	protein_cc	A430107N	ataxin 7	Atxn7
5820	Psmd6	1.457	0.932	1.358	0.005	0.332	0.050	8.916	9.460	9.357	66413	Psmd6	chr14	-	NM_0255	P7067	protein_cc	2400006A	proteason	Psmd6
5821	Olfr720	1.000	1.000	1.000	1.000	1.000	1.000	0.000	0.000	0.000	258387	Olfr720	chr14	-	NM_1463	P17477	protein_cc	MOR274-2	olfactory	Olfr720
5822	Olfr31	1.000	1.000	1.000	1.000	1.000	1.000	0.000	0.000	0.000	18330	Olfr31	chr14	+	NM_1470	P25262	protein_cc	MOR274-1	olfactory r	Olfr31
5823	Il3ra	1.526	0.905	1.381	0.189	0.777	0.646	2.535	3.145	3.001	16188	Il3ra	chr14	+	NM_0083	P17981	protein_cc	CD123| Cl	interleuki	Il3ra
5824	Slc4a7	1.199	0.725	0.869	0.156	0.009	0.601	6.708	6.970	6.505	218756	Slc4a7	chr14	+	NM_0010	P25754	protein_cc	E430014N	solute car	Slc4a7
5825	Nek10	1.000	1.000	1.000	1.000	1.000	1.000	0.000	0.000	0.000	674895	Nek10	chr14	+	NM_0011	P3062	protein_cc	Gm282	NIMA (nev	Nek10
5826	Lrrc3b	1.000	1.000	1.000	1.000	1.000	1.000	0.000	0.000	0.000	218763	Lrrc3b	chr14	-	NM_1460	P25307	protein_cc	BC019794	leucine ric	Lrrc3b
5827	Oxsm	0.559	1.595	0.892	0.026	0.075	0.486	7.848	7.009	7.682	71147	Oxsm	chr14	-	NM_0276	P22164	protein_cc	4933425A	3-oxoacyl-	Oxsm
5828	Ngly1	0.741	1.309	0.969	0.009	0.012	0.260	7.606	7.172	7.561	59007	Ngly1	chr14	+	NM_0215	P23819	protein_cc	1110002C	N-glycana	Ngly1
5829	Top2b	0.808	1.069	0.863	0.030	0.221	0.223	9.716	9.407	9.504	21974	Top2b	chr14	+	NM_0094	P17534	protein_cc	D230016L	topoisome	Top2b
5830	Rarb	0.366	2.309	0.845	0.000	0.272	0.824	4.882	3.431	4.639	218772	Rarb	chr14	-	NM_0012	P7592	protein_cc	A830025K	retinoic ac	Rarb
5831	Thrb	0.254	1.562	0.396	0.000	0.140	0.045	8.789	6.811	7.454	21834	Thrb	chr14	+	NM_0011	P6145	protein_cc	c-erbAbet	thyroid ho	Thrb
5832	Nr1d2	0.186	4.174	0.777	0.000	0.009	0.437	10.088	7.662	9.723	353187	Nr1d2	chr14	-	NM_0115	P5967	protein_cc	Rev-erb| F	nuclear re	Nr1d2
5833	Rpl15	1.339	1.245	1.668	0.000	0.059	0.083	10.209	10.631	10.947	66480	Rpl15	chr14	-	NM_0255	P2426	protein_cc	2510008H	ribosomal	Rpl15
5834	Nkiras1	1.276	0.991	1.264	0.041	0.932	0.234	9.140	9.491	9.478	69721	Nkiras1	chr14	+	NM_0235	P23460	protein_cc	2400004O	NFKB inhib	Nkiras1
5835	Ube2e1	1.152	0.987	1.137	0.076	0.898	0.456	8.758	8.962	8.943	22194	Ube2e1	chr14	-	NM_0094	P11170	protein_cc	Ubce5| ub	ubiquitin-	Ube2e1
5836	Ube2e2	0.943	1.618	1.527	0.860	0.069	0.257	6.972	6.887	7.582	218793	Ube2e2	chr14	-	NM_1448	P536	protein_cc	BC016265	ubiquitin-	Ube2e2
5837	Gm5458	1.000	1.000	1.000	1.000	1.000	1.000	0.000	0.000	0.000	432825	Gm5458	chr14	-	NM_0010	P15675	protein_cc	EG432825	predicted	Gm5458
5838	Nid2	0.471	1.505	0.709	0.012	0.062	0.227	4.808	3.722	4.312	18074	Nid2	chr14	+	NM_0086	P14927	protein_cc	AW54714	nidogen 2	Nid2
5839	2700060E	0.932	1.140	1.063	0.265	0.095	0.350	9.833	9.732	9.921	68045	2700060E	chr14	-	NM_0265	P20090	protein_cc	.	RIKEN cDN	2700060E
5840	D14Ertd67	1.000	1.000	1.000	1.000	1.000	1.000	0.000	0.000	0.000	52512	D14Ertd67	chr14	+	NR_10502	.	lncRNA	.	DNA segm	D14Ertd67
5841	Gng2	1.652	0.620	1.024	0.042	0.090	0.948	3.995	4.719	4.029	14702	Gng2	chr14	-	NM_0012	P18344	protein_cc	.	guanine n	Gng2
5842	Saysd1	1.173	0.966	1.133	0.224	0.894	0.778	6.905	7.136	7.086	67509	Saysd1	chr14	-	NM_0262	P20635	protein_cc	1810063B	SAYSVFN	Saysd1
5843	Kcnk5	0.018	7.499	0.137	0.000	0.000	0.003	7.610	1.833	4.740	16529	Kcnk5	chr14	-	NM_0215	P2854	protein_cc	KCNK5b|	potassium	Kcnk5
5844	Kcnk16	1.000	1.000	1.000	1.000	1.000	1.000	0.000	0.000	0.000	74571	Kcnk16	chr14	-	NM_0290	P5654	protein_cc	4731413G	potassium	Kcnk16
5845	Nudt13	0.764	0.770	0.588	0.069	0.105	0.182	6.755	6.366	5.989	67725	Nudt13	chr14	+	NM_0263	P20385	protein_cc	4933433B	nudix (nuc	Nudt13
5846	Ecd	0.784	1.263	0.990	0.024	0.158	0.958	8.706	8.354	8.691	70601	Ecd	chr14	-	NM_0274	P1436	protein_cc	5730461K	ecdysonel	Ecd
5847	Fam149b	0.564	0.770	0.435	0.000	0.090	0.023	6.722	5.896	5.520	105428	Fam149b	chr14	+	NM_1723	P10204	protein_cc	AA536717	family wit	Fam149b
5848	Dnajc9	1.452	0.572	0.830	0.185	0.083	0.397	6.037	6.575	5.769	108671	Dnajc9	chr14	-	NM_1340	P17734	protein_cc	5330419I	DnaJ heat	Dnajc9
5849	Mrps16	0.499	1.440	0.719	0.000	0.011	0.075	9.227	8.225	8.751	66242	Mrps16	chr14	-	NM_0254	P1918	protein_cc	1500011E	mitochond	Mrps16
5850	Cfap70	1.000	1.000	1.000	1.000	1.000	1.000	0.000	0.000	0.000	76670	Cfap70	chr14	-	NM_0011	P25073	protein_cc	Ttc18	cilia and fl	Cfap70
5851	Anxa7	1.128	1.230	1.387	0.241	0.041	0.018	8.717	8.890	9.189	11750	Anxa7	chr14	-	NM_0011	P5035	protein_cc	AI265384|	annexin A	Anxa7
5852	Mss51	1.647	1.443	2.377	0.296	0.175	0.213	1.497	2.217	2.747	74843	Mss51	chr14	-	NM_0291	P3099	protein_cc	4833444M	MSS51 mi	Mss51
5853	Ppp3cb	0.745	0.808	0.602	0.015	0.163	0.144	6.988	6.564	6.256	19056	Ppp3cb	chr14	-	NM_0089	P22084	protein_cc	1110063J1	protein ph	Ppp3cb
5854	1810062O	0.889	2.376	2.113	0.714	0.288	0.552	0.875	0.706	1.954	75602	1810062O	chr14	+	NR_03357	.	lncRNA	.	RIKEN cDN	1810062O
5855	Usp54	2.614	0.432	1.129	0.041	0.037	0.887	4.062	5.448	4.238	78787	Usp54	chr14	-	NM_0301	P14146	protein_cc	4930429G	ubiquitin s	Usp54
5856	Myoz1	0.321	1.333	0.428	0.000	0.205	0.048	1.679	0.038	0.453	59011	Myoz1	chr14	-	NM_0215	P7989	protein_cc	2310001N	myozenin	Myoz1
5857	Synpo2l	1.000	1.000	1.000	1.000	1.000	1.000	0.000	0.000	0.000	68760	Synpo2l	chr14	-	NM_1751	P16409	protein_cc	1110054M	synaptopo	Synpo2l
5858	Sec24c	1.316	0.750	0.987	0.032	0.021	0.912	8.695	9.092	8.676	218811	Sec24c	chr14	+	NM_1725	P3127	protein_cc	2610204K	Sec24 rela	Sec24c
5859	Fut11	1.182	1.341	1.585	0.607	0.437	0.425	4.263	4.503	4.927	73068	Fut11	chr14	+	NM_0284	P14083	protein_cc	3110009G	fucosyltra	Fut11
5860	6230400D	2.456	2.434	5.978	0.204	0.043	0.063	0.732	2.028	3.311	76133	6230400D	chr14	-	NR_02944	.	lncRNA	.	RIKEN cDN	6230400D
5861	Chchd1	0.861	1.077	0.927	0.393	0.587	0.701	9.999	9.783	9.890	66121	Chchd1	chr14	+	NM_0253	P7537	protein_cc	1110001O	coiled-coil	Chchd1
5862	Zswim8	2.156	0.893	1.925	0.000	0.122	0.044	6.713	7.821	7.657	268721	Zswim8	chr14	+	NM_0279	P14432	protein_cc	2310021P	zinc finger	Zswim8
5863	Ndst2	1.004	1.295	1.300	0.972	0.298	0.537	6.993	6.999	7.371	17423	Ndst2	chr14	-	NM_0108	P23846	protein_cc	Mndns| N	N-deacety	Ndst2
5864	Camk2g	2.160	0.780	1.684	0.001	0.010	0.097	5.167	6.278	5.919	12325	Camk2g	chr14	-	NM_0010	P3225	protein_cc	Camkg	calcium/ca	Camk2g
5865	Plau	1.538	0.403	0.620	0.548	0.333	0.244	2.597	3.218	1.907	18792	Plau	chr14	+	NM_0088	P7901	protein_cc	u-PA| uPA	plasminog	Plau
5866	Vcl	1.530	0.585	0.895	0.016	0.003	0.571	7.523	8.136	7.363	22330	Vcl	chr14	-	NM_0095	P1423	protein_cc	9430097D	vinculin	Vcl
5867	Ap3m1	0.517	0.866	0.448	0.000	0.006	0.003	9.326	8.374	8.168	55946	Ap3m1	chr14	-	NM_0188	P9766	protein_cc	1200013D	adaptor-re	Ap3m1
5868	Adk	0.557	1.260	0.701	0.000	0.001	0.008	12.524	11.678	12.012	11534	Adk	chr14	+	NM_1340	P5900	protein_cc	2310026J	adenosine	Adk
5869	Kat6b	1.206	1.118	1.348	0.515	0.553	0.533	6.220	6.490	6.651	54169	Kat6b	chr14	+	NM_0012	P17156	protein_cc	AI507552|	K(lysine) a	Kat6b
5870	Dupd1	1.000	1.000	1.000	1.000	1.000	1.000	0.000	0.000	0.000	435391	Dupd1	chr14	-	NM_0010	P14091	protein_cc	EG435391	dual speci	Dupd1
5871	Dusp13	1.000	1.000	1.000	1.000	1.000	1.000	0.000	0.000	0.000	27389	Dusp13	chr14	-	NM_0010	P19051	protein_cc	DUSP13A|	dual speci	Dusp13
5872	Samd8	0.437	1.128	0.492	0.001	0.076	0.042	8.803	7.607	7.781	67630	Samd8	chr14	+	NM_0262	P24598	protein_cc	1110053F	sterile alp	Samd8
5873	Vdac2	0.670	1.149	0.770	0.000	0.024	0.073	10.152	9.574	9.775	22334	Vdac2	chr14	+	NM_0116	P20524	protein_cc	mVDAC2|	voltage-de	Vdac2
5874	Comtd1	0.647	1.711	1.107	0.310	0.093	0.800	6.157	5.529	6.303	69156	Comtd1	chr14	-	NM_0269	P6489	protein_cc	1810030N	catechol-C	Comtd1
5875	Zfp503	0.169	2.397	0.405	0.002	0.131	0.162	5.735	3.169	4.430	218820	Zfp503	chr14	-	NM_1454	P17914	protein_cc	AI181838|	zinc finger	Zfp503
5876	1700112E	0.738	0.612	0.452	0.647	0.454	0.502	1.577	1.140	0.432	76633	1700112E	chr14	+	NM_0282	P16065	protein_cc	2700009F	RIKEN cDN	1700112E
5877	4930405A	1.000	1.000	1.000	1.000	1.000	1.000	0.000	0.000	0.000	74896	4930405A	chr14	-	NR_04630	.	lncRNA	4930456G	RIKEN cDN	4930405A
5878	Gm10248	0.716	0.995	0.712	0.182	1.000	0.423	0.490	0.008	0.000	791307	Gm10248	chr14	-	NR_03355	.	lncRNA	ENSMUSG	predicted	Gm10248
5879	Kcnma1	0.419	1.535	0.644	0.047	0.472	0.425	2.431	1.176	1.795	16531	Kcnma1	chr14	-	NM_0012	P15064	protein_cc	5730414M	potassium	Kcnma1
5880	4930519K	1.000	1.000	1.000	1.000	1.000	1.000	0.000	0.000	0.000	78019	4930519K	chr14	+	NR_13110	.	lncRNA	.	RIKEN cDN	4930519K
5881	Dlg5	2.678	1.144	3.063	0.000	0.663	0.261	4.845	6.266	6.460	71228	Dlg5	chr14	-	NM_0277	P16797	protein_cc	4933429D	discs, larg	Dlg5
5882	E330034G	1.000	1.000	1.000	1.000	1.000	1.000	0.000	0.000	0.000	105418	E330034G	chr14	+	NM_0010	P4580	protein_cc	AA415275	RIKEN cDN	E330034G
5883	Polr3a	0.934	1.273	1.189	0.749	0.272	0.521	5.571	5.472	5.821	218832	Polr3a	chr14	-	NM_0010	P23382	protein_cc	9330175N	polymeras	Polr3a
5884	Rps24	1.299	1.574	2.046	0.187	0.016	0.001	11.405	11.782	12.437	20088	Rps24	chr14	+	NM_0112	P25231	protein_cc	.	ribosomal	Rps24
5885	4930572O	1.000	1.000	1.000	1.000	1.000	1.000	0.000	0.000	0.000	75862	4930572O	chr14	-	NR_04571	.	lncRNA	.	RIKEN cDN	4930572O
5886	Zmiz1	0.423	1.649	0.698	0.000	0.196	0.397	7.964	6.723	7.445	328365	Zmiz1	chr14	+	NM_1832	P23698	protein_cc	BC065120	zinc finger	Zmiz1
5887	4931406H	0.294	0.974	0.286	0.181	1.000	0.423	1.806	0.038	0.000	77592	4931406H	chr14	+	NR_03349	.	lncRNA	.	RIKEN cDN	4931406H
5888	Israa	0.397	0.746	0.296	0.108	0.675	0.167	4.020	2.688	2.265	10018891	Israa	chr14	+	NM_0013	P684	protein_cc	.	immune s	Israa
5889	Ppif	0.457	2.122	0.969	0.002	0.002	0.072	8.493	7.361	8.447	105675	Ppif	chr14	+	NM_1340	P3152	protein_cc	AW45719	peptidylpr	Ppif
5890	Ppifos	1.000	1.000	1.000	1.000	1.000	1.000	0.000	0.000	0.000	68173	Ppifos	chr14	-	NR_02802	.	lncRNA	4930520F	peptidylpr	Ppifos
5891	Zcchc24	0.563	1.116	0.628	0.004	0.413	0.016	10.112	9.282	9.440	71918	Zcchc24	chr14	-	NM_0011	P4151	protein_cc	2310047A	zinc finger	Zcchc24
5892	Anxa11	0.742	1.334	0.990	0.000	0.088	0.963	6.055	5.625	6.041	11744	Anxa11	chr14	+	NM_0134	P1729	protein_cc	A830099C	annexin A	Anxa11
5893	Plac9b	0.648	1.561	1.012	0.048	0.157	0.979	5.725	5.100	5.742	10003924	Plac9b	chr14	-	NM_0012	P14215	protein_cc	ENSMUSG	placenta s	Plac9b
5894	Plac9a	0.607	1.667	1.013	0.026	0.118	0.968	5.597	4.877	5.615	211623	Plac9a	chr14	-	NM_2072	P14215	protein_cc	Plac9	placenta s	Plac9a
5895	Tmem254	0.252	1.881	0.474	0.000	0.032	0.032	10.606	8.617	9.529	66039	Tmem254	chr14	-	NM_0266	P17126	protein_cc	0610008K	transmem	Tmem254
5896	Tmem254	0.251	1.895	0.475	0.000	0.033	0.034	10.594	8.599	9.521	10003925	Tmem254	chr14	-	NM_0012	P17126	protein_cc	ENSMUSG	transmem	Tmem254
5897	Tmem254	0.253	1.885	0.476	0.000	0.033	0.033	10.582	8.597	9.512	10003919	Tmem254	chr14	-	NM_0012	P3690	protein_cc	ENSMUSG	transmem	Tmem254
5898	Cphx2	1.000	1.000	1.000	1.000	1.000	1.000	0.000	0.000	0.000	10003921	Cphx2	chr14	+	NM_0012	P14373	protein_cc	10003921	cytoplasm	Cphx2
5899	Cphx1	0.466	1.084	0.505	0.040	0.887	0.249	3.133	2.033	2.148	105594	Cphx1	chr14	+	NM_1753	P14373	protein_cc	AU019881	cytoplasm	Cphx1
5900	Duxbl2	1.010	1.825	1.844	0.000	0.182	0.423	0.000	0.015	0.883	10003922	Duxbl2	chr14	+	NM_0011	P7343	protein_cc	ENSMUSG	double ho	Duxbl2
5901	Duxbl3	0.716	0.995	0.712	0.182	1.000	0.423	0.490	0.008	0.000	10003929	Duxbl3	chr14	+	NM_0011	P7343	protein_cc	ENSMUSG	double ho	Duxbl3
5902	Duxbl1	1.000	1.000	1.000	1.000	1.000	1.000	0.000	0.000	0.000	278672	Duxbl1	chr14	+	NM_1833	P7343	protein_cc	1110051B	double ho	Duxbl1

5903	4933413J(	1.000	1.000	1.000	1.000	1.000	1.000	0.000	0.000	0.000	71106	4933413J(	chr14	+	NR_03800	.	lncRNA	GCSE	RIKEN cDN	4933413J0
5904	Slmap	0.841	0.818	0.688	0.362	0.254	0.226	8.842	8.593	8.303	83997	Slmap	chr14	-	NM_0320	P4136	protein_cc	D330001L	sarcolemm	Slmap
5905	Dennd6a	0.940	0.932	0.876	0.608	0.529	0.303	7.026	6.937	6.835	211922	Dennd6a	chr14	+	NM_0012	P19746	protein_cc	Fam116a	DENN/MA	Dennd6a
5906	Arf4	3.123	0.609	1.902	0.000	0.013	0.178	9.814	11.457	10.742	11843	Arf4	chr14	+	NM_0074	P12327	protein_cc	AA407803	ADP-ribos	Arf4
5907	Pde12	5.322	0.746	3.969	0.000	0.020	0.008	5.591	8.003	7.580	211948	Pde12	chr14	-	NM_1786	P16424	protein_cc	2'-PDE| E4	phosphod	Pde12
5908	Asb14	0.716	0.995	0.712	0.182	1.000	0.423	0.490	0.008	0.000	142687	Asb14	chr14	+	NM_0808	P7836	protein_cc	Asb-14	ankyrin re	Asb14
5909	Appl1	1.337	0.944	1.262	0.000	0.014	0.008	8.054	8.473	8.389	72993	Appl1	chr14	-	NM_1452	P5147	protein_cc	2900057D	adaptor p	Appl1
5910	Hesx1	1.581	0.906	1.432	0.301	0.802	0.423	0.000	0.661	0.519	15209	Hesx1	chr14	+	NM_0104	P2942	protein_cc	HES-1| Rp	homeobox	Hesx1
5911	Il17rd	0.712	1.734	1.234	0.579	0.016	0.788	3.048	2.558	3.352	171463	Il17rd	chr14	+	NM_1344	P5061	protein_cc	2810004A	interleukin	Il17rd
5912	Arhgef3	0.913	1.209	1.104	0.136	0.010	0.304	7.673	7.541	7.815	71704	Arhgef3	chr14	+	NM_0012	P3684	protein_cc	1200004I2	Rho guani	Arhgef3
5913	Fam208a	1.138	0.765	0.871	0.138	0.169	0.649	8.767	8.953	8.567	218850	Fam208a	chr14	+	NM_0289	P13350	protein_cc	4732418E	family wit	Fam208a
5914	Ccdc66	2.825	0.882	2.491	0.037	0.591	0.051	5.606	7.104	6.923	320234	Ccdc66	chr14	-	NM_1771	P5611	protein_cc	E230015L	coiled-coil	Ccdc66
5915	Erc2	0.381	0.946	0.361	0.100	0.888	0.308	5.614	4.222	4.142	238988	Erc2	chr14	+	NM_1778	P21052	protein_cc	6430531D	ELKS/RAB	Erc2
5916	Wnt5a	0.723	2.566	1.854	0.444	0.010	0.141	3.028	2.559	3.918	22418	Wnt5a	chr14	+	NM_0095	P21393	protein_cc	8030457G	wingless-t	Wnt5a
5917	Cacna2d3	0.669	0.995	0.666	0.181	1.000	0.423	0.587	0.008	0.000	12294	Cacna2d3	chr14	-	NM_0097	P13483	protein_cc	alapha2de	calcium ch	Cacna2d3
5918	Lrtm1	0.019	7.646	0.149	0.000	0.036	0.006	9.045	3.363	6.297	319476	Lrtm1	chr14	+	NM_1769	P8996	protein_cc	A930016D	leucine-ric	Lrtm1
5919	Selk	2.104	0.746	1.569	0.000	0.029	0.157	10.400	11.473	11.050	80795	Selk	chr14	+	NM_0199	P16680	protein_cc	1110001C	selenopro	Selk
5920	Actr8	0.965	0.931	0.899	0.040	0.373	0.449	7.744	7.693	7.590	56249	Actr8	chr14	+	NM_0274	P6895	protein_cc	5730542K	ARP8 actin	Actr8
5921	Il17rb	1.246	1.472	1.834	0.065	0.396	0.498	5.077	5.394	5.952	50905	Il17rb	chr14	-	NM_0195	P18056	protein_cc	Evi27| IL-1	interleukin	Il17rb
5922	Chdh	0.226	1.487	0.336	0.000	0.280	0.043	9.501	7.356	7.928	218865	Chdh	chr14	+	NM_0011	P7190	protein_cc	AU041472	choline de	Chdh
5923	Cacna1d	0.168	1.893	0.317	0.000	0.136	0.028	9.253	6.676	7.597	12289	Cacna1d	chr14	-	NM_0013	P26123	protein_cc	8430418G	calcium ch	Cacna1d
5924	4831407H	1.000	1.000	1.000	1.000	1.000	1.000	0.000	0.000	0.000	77710	4831407H	chr14	+	NR_13109	.	lncRNA	.	RIKEN cDN	4831407H
5925	Dcp1a	2.430	0.579	1.406	0.000	0.005	0.273	4.110	5.391	4.602	75901	Dcp1a	chr14	+	NM_1337	P21612	protein_cc	1110066A	decapping	Dcp1a
5926	Tkt	0.698	1.613	1.126	0.003	0.001	0.027	8.525	8.007	8.697	21881	Tkt	chr14	+	NM_0093	P15343	protein_cc	p68	transketol	Tkt
5927	Prkcd	2.323	0.610	1.418	0.008	0.024	0.298	5.812	7.028	6.316	18753	Prkcd	chr14	-	NM_0111	P6012	protein_cc	AI385711|	protein kin	Prkcd
5928	Rft1	0.512	1.935	0.990	0.003	0.014	0.954	6.882	5.916	6.868	328370	Rft1	chr14	+	NM_1778	P20893	protein_cc	D930025H	RFT1 hom	Rft1
5929	Sfmbt1	0.798	1.095	0.874	0.112	0.587	0.453	7.879	7.553	7.685	54650	Sfmbt1	chr14	+	NM_0194	P18854	protein_cc	4930442N	Scm-like w	Sfmbt1
5930	Tmem110	2.478	1.340	3.321	0.006	0.128	0.054	6.660	7.969	8.392	69179	Tmem110	chr14	+	NM_0288	P10705	protein_cc	1810038N	transmem	Tmem110
5931	Mustn1	0.311	2.015	0.626	0.005	0.048	0.246	5.532	3.846	4.856	66175	Mustn1	chr14	+	NM_1813	P18608	protein_cc	1110028G	musculosk	Mustn1
5932	Itih4	2.947	1.135	3.345	0.001	0.144	0.001	12.796	14.355	14.538	16427	Itih4	chr14	+	NM_0011	P8852	protein_cc	ITI-HC4| It	inter alpha	Itih4
5933	Itih3	1.983	1.007	1.997	0.002	0.963	0.129	11.834	12.822	12.832	16426	Itih3	chr14	-	NM_0084	P14822	protein_cc	AW10809	inter-alph	Itih3
5934	Itih1	0.554	0.867	0.480	0.016	0.511	0.040	11.003	10.150	9.944	16424	Itih1	chr14	-	NM_0084	P20180	protein_cc	Intin1| ITI	inter-alph	Itih1
5935	Nek4	0.948	1.289	1.221	0.539	0.257	0.571	7.559	7.481	7.847	23955	Nek4	chr14	+	NM_0118	P5727	protein_cc	Stk2	NIMA (nev	Nek4
5936	Spcs1	1.345	1.021	1.373	0.071	0.836	0.142	9.555	9.982	10.012	69019	Spcs1	chr14	-	NM_0269	P14475	protein_cc	1810004F	signal pep	Spcs1
5937	Glt8d1	1.901	0.641	1.219	0.009	0.020	0.316	6.040	6.967	6.326	76485	Glt8d1	chr14	+	NM_0011	P16091	protein_cc	2410004H	glycosyltra	Glt8d1
5938	Gnl3	2.078	0.818	1.699	0.014	0.225	0.042	8.568	9.623	9.333	30877	Gnl3	chr14	-	NM_1535	P26165	protein_cc	Ns	guanine n	Gnl3
5939	Snord69	1.000	1.000	1.000	1.000	1.000	1.000	0.000	0.000	0.000	10021743	Snord69	chr14	-	NR_02853	.	snoRNA	MBII-210	small nucl	Snord69
5940	Snord19	1.000	1.000	1.000	1.000	1.000	1.000	0.000	0.000	0.000	10021742	Snord19	chr14	-	NR_02852	.	snoRNA	MBII-108	small nucl	Snord19
5941	Pbrm1	0.913	1.050	0.959	0.058	0.291	0.649	9.987	9.856	9.927	66923	Pbrm1	chr14	+	NM_0010	P52	protein_cc	2610016F	polybromo	Pbrm1
5942	Smim4	0.463	1.448	0.670	0.001	0.087	0.151	9.056	7.945	8.479	66487	Smim4	chr14	-	NM_0013	P13624	protein_cc	2010107H	small integ	Smim4
5943	Nt5dc2	0.573	1.560	0.895	0.001	0.316	0.841	7.455	6.653	7.295	70021	Nt5dc2	chr14	+	NM_0272	P6609	protein_cc	2510015F	5'-nucleot	Nt5dc2
5944	Stab1	0.680	1.460	0.993	0.006	0.385	0.991	7.373	6.817	7.363	192187	Stab1	chr14	-	NM_1386	P4703	protein_cc	FEEL-1| FE	stabilin 1	Stab1
5945	Nisch	1.219	0.806	0.983	0.019	0.024	0.817	8.085	8.370	8.060	64652	Nisch	chr14	-	NM_0226	P7757	protein_cc	1200007D	nischarin	Nisch
5946	Tnnc1	1.696	1.604	2.721	0.580	0.427	0.007	1.604	2.366	3.048	21924	Tnnc1	chr14	+	NM_0093	P20254	protein_cc	AI874626|	troponin C	Tnnc1
5947	Sema3g	1.000	1.000	1.000	1.000	1.000	1.000	0.000	0.000	0.000	218877	Sema3g	chr14	+	NM_0010	P11996	protein_cc	AK129018	sema dom	Sema3g
5948	Phf7	0.702	0.948	0.665	0.016	0.722	0.228	5.777	5.265	5.189	71838	Phf7	chr14	-	NM_0279	P15138	protein_cc	1700006H	PHD finge	Phf7
5949	Bap1	0.836	1.186	0.991	0.029	0.054	0.889	6.719	6.460	6.706	104416	Bap1	chr14	+	NM_0270	P18737	protein_cc	2300006C	Brca1 asso	Bap1
5950	Dnah1	3.368	0.944	3.181	0.069	0.915	0.423	0.001	1.753	1.670	110084	Dnah1	chr14	-	NM_0010	P18111	protein_cc	B230373P	dynein, ax	Dnah1
5951	Capn7	0.784	1.100	0.862	0.055	0.491	0.336	8.479	8.128	8.264	12339	Capn7	chr14	+	NM_0097	P11201	protein_cc	AU022319	calpain 7	Capn7
5952	Sh3bp5	0.367	1.352	0.496	0.000	0.311	0.120	10.577	9.129	9.565	24056	Sh3bp5	chr14	-	NM_0118	P14705	protein_cc	AI606498|	SH3-doma	Sh3bp5
5953	Mettl6	3.386	0.738	2.500	0.000	0.022	0.023	5.922	7.682	7.245	67011	Mettl6	chr14	-	NM_0259	P14233	protein_cc	1600013P	methyltra	Mettl6
5954	Eaf1	1.388	0.911	1.264	0.000	0.068	0.063	7.035	7.508	7.373	74427	Eaf1	chr14	+	NM_0289	P8276	protein_cc	4933403C	ELL associ	Eaf1
5955	Colq	0.451	0.986	0.444	0.182	1.000	0.423	1.170	0.021	0.000	382864	Colq	chr14	-	NM_0099	P10365	protein_cc	A130034K	collagen-li	Colq
5956	Hacl1	0.073	3.396	0.249	0.000	0.006	0.011	11.273	7.503	9.267	56794	Hacl1	chr14	-	NM_0199	P1189	protein_cc	1600020H	2-hydroxy	Hacl1
5957	Btd	0.486	1.496	0.726	0.009	0.015	0.213	8.888	7.846	8.427	26363	Btd	chr14	+	NM_0252	P6695	protein_cc	.	biotinidas	Btd
5958	Ankrd28	1.258	1.467	1.846	0.002	0.137	0.244	5.832	6.163	6.716	105522	Ankrd28	chr14	-	NM_0010	P1501	protein_cc	AI465466|	ankyrin re	Ankrd28
5959	Galnt15	1.000	1.000	1.000	1.000	1.000	1.000	0.000	0.000	0.000	78754	Galnt15	chr14	+	NM_0301	P9023	protein_cc	4631401E	UDP-N-ac	Galnt15
5960	Dph3	1.279	1.131	1.446	0.230	0.489	0.132	5.422	5.777	5.955	105638	Dph3	chr14	-	NM_0012	P21591	protein_cc	DELGIP1|	diphthami	Dph3
5961	Oxnad1	0.692	1.090	0.755	0.018	0.172	0.229	8.371	7.840	7.965	218885	Oxnad1	chr14	+	NM_1454	P14195	protein_cc	2410002F	oxidoredu	Oxnad1
5962	Msmb	1.000	1.000	1.000	1.000	1.000	1.000	0.000	0.000	0.000	17695	Msmb	chr14	+	NM_0205	P19532	protein_cc	beta-MSP	beta-micro	Msmb
5963	Ncoa4	0.991	0.964	0.955	0.914	0.690	0.266	8.521	8.508	8.455	27057	Ncoa4	chr14	+	NM_0010	P12421	protein_cc	ARA70| RI	nuclear re	Ncoa4
5964	Timm23	1.096	1.113	1.220	0.308	0.148	0.108	8.290	8.422	8.577	53600	Timm23	chr14	-	NM_0168	P15882	protein_cc	Tim23	transclocas	Timm23
5965	Parg	1.824	0.902	1.645	0.002	0.636	0.379	7.138	8.006	7.857	26430	Parg	chr14	+	NM_0119	P7900	protein_cc	AI413217	poly (ADP-	Parg
5966	Ogdhl	0.669	0.995	0.666	0.181	1.000	0.423	0.587	0.008	0.000	239017	Ogdhl	chr14	+	NM_0010	P14087	protein_cc	.	oxoglutara	Ogdhl
5967	1700024G	1.000	1.000	1.000	1.000	1.000	1.000	0.000	0.000	0.000	67085	1700024G	chr14	-	NM_0010	P21972	protein_cc	C10orf53	RIKEN cDN	1700024G
5968	Chat	0.750	2.739	2.055	0.200	0.182	0.516	0.448	0.033	1.487	12647	Chat	chr14	-	NM_0098	P12013	protein_cc	B230380D	choline ac	Chat
5969	Slc18a3	2.134	0.469	1.000	0.305	1.000	0.423	0.000	1.094	0.000	20508	Slc18a3	chr14	-	NM_0217	P19023	protein_cc	VAChT| VA	solute car	Slc18a3
5970	Ercc6	1.241	1.021	1.267	0.195	0.924	0.534	5.278	5.590	5.620	319955	Ercc6	chr14	+	NM_0010	P7326	protein_cc	4732403I(	excision re	Ercc6
5971	Prrxl1	1.000	1.000	1.000	1.000	1.000	1.000	0.000	0.000	0.000	107751	Prrxl1	chr14	+	NM_0010	P22195	protein_cc	Drg11| Dr	paired rela	Prrxl1
5972	3425401B	0.726	1.387	1.007	0.187	0.187	0.988	0.477	0.015	0.487	10050451	3425401B	chr14	+	NM_0011	P7673	protein_cc	.	RIKEN cDN	3425401B
5973	1810011H	0.335	1.335	0.447	0.182	0.204	0.504	1.614	0.038	0.454	69069	1810011H	chr14	+	NM_0011	P7260	protein_cc	4733401I(	RIKEN cDN	1810011H
5974	Fam170b	1.000	1.000	1.000	1.000	1.000	1.000	0.000	0.000	0.000	105511	Fam170b	chr14	+	NM_0011	P6137	protein_cc	AI847159	family wit	Fam170b
5975	Vstm4	0.126	3.567	0.450	0.001	0.129	0.221	5.412	2.426	4.261	320736	Vstm4	chr14	+	NM_1787	P12793	protein_cc	E130203B	V-set and	Vstm4
5976	Wdfy4	0.440	2.082	0.917	0.000	0.009	0.656	4.970	3.787	4.845	545030	Wdfy4	chr14	-	NM_0011	P10087	protein_cc	Gm18810	WD repea	Wdfy4
5977	Lrrc18	1.204	1.687	2.030	0.717	0.488	0.625	1.514	1.781	2.536	67580	Lrrc18	chr14	+	NM_0262	P9944	protein_cc	4930442L	leucine ric	Lrrc18
5978	Arhgap22	4.557	1.598	7.282	0.212	0.466	0.250	0.359	2.547	3.223	239027	Arhgap22	chr14	+	NM_1538	P26871	protein_cc	B230341L	Rho GTPas	Arhgap22
5979	Mapk8	1.302	1.500	1.952	0.208	0.102	0.233	7.428	7.808	8.393	26419	Mapk8	chr14	-	NM_0167	P15439	protein_cc	AI849689|	mitogen-a	Mapk8
5980	Ptpn20	1.000	1.000	1.000	1.000	1.000	1.000	0.000	0.000	0.000	19256	Ptpn20	chr14	+	NM_0089	P10026	protein_cc	typ	protein ty	Ptpn20
5981	4930503F	1.000	1.000	1.000	1.000	1.000	1.000	0.000	0.000	0.000	218902	4930503F	chr14	-	NR_13111	.	lncRNA	4930453C	RIKEN cDN	4930503F2
5982	Gdf10	0.014	34.718	0.502	0.000	0.081	0.287	6.315	0.203	5.320	14560	Gdf10	chr14	+	NM_1457	P20366	protein_cc	Bmp3b	growth dif	Gdf10
5983	Gdf2	0.056	7.852	0.440	0.000	0.044	0.124	7.431	3.275	6.248	12165	Gdf2	chr14	+	NM_0195	P18646	protein_cc	Bmp9	growth dif	Gdf2
5984	Rbp3	1.000	1.000	1.000	1.000	1.000	1.000	0.000	0.000	0.000	19661	Rbp3	chr14	+	NM_0157	P4908	protein_cc	Irbp| Rbp-	retinol bin	Rbp3
5985	Zfp488	1.000	1.000	1.000	1.000	1.000	1.000	0.000	0.000	0.000	382867	Zfp488	chr14	-	NM_0010	P23173	protein_cc	Gm1206|	zinc finger	Zfp488
5986	Gm5460	1.000	1.000	1.000	1.000	1.000	1.000	0.000	0.000	0.000	432838	Gm5460	chr14	+	NM_0010	P16748	protein_cc	EG432838	predicted	Gm5460
5987	A630023A	1.000	1.000	1.000	1.000	1.000	1.000	0.000	0.000	0.000	105518	A630023A	chr14	+	NM_0012	P26444	protein_cc	AI461879	RIKEN cDN	A630023A
5988	Antxrl	1.000	1.000	1.000	1.000	1.000	1.000	0.000	0.000	0.000	239029	Antxrl	chr14	+	NM_1728	P24665	protein_cc	1700112N	anthrax to	Antxrl
5989	Anxa8	1.295	2.124	2.750	0.668	0.070	0.058	0.774	1.146	2.233	11752	Anxa8	chr14	+	NM_0134	P9385	protein_cc	AI466995|	annexin A	Anxa8
5990	Npy4r	1.000	1.000	1.000	1.000	1.000	1.000	0.000	0.000	0.000	19065	Npy4r	chr14	-	NM_0089	P9958	protein_cc	NYYR-D| F	neuropept	Npy4r
5991	Gprin2	1.000	1.000	1.000	1.000	1.000	1.000	0.000	0.000	0.000	432839	Gprin2	chr14	-	NM_1832	P23024	protein_cc	C130040D	G protein	Gprin2
5992	Syt15	0.086	1.218	0.105	0.006	0.286	0.084	3.642	0.107	0.391	319508	Syt15	chr14	+	NM_1815	P9305	protein_cc	CHR10SYT	synaptota	Syt15
5993	Fam35a	0.374	2.267	0.847	0.002	0.094	0.706	8.210	6.790	7.970	75698	Fam35a	chr14	-	NM_0293	P11856	protein_cc	3110001K	family wit	Fam35a

5994	Glud1	0.189	1.383	0.261	0.000	0.007	0.009	13.560	11.153	11.621	14661	Glud1	chr14	+	NM_0081	P20518	protein_cc	AI118167|	glutamate	Glud1
5995	Gm3219	0.208	1.318	0.274	0.003	0.055	0.066	8.490	6.225	6.623	10004123	Gm3219	chr14	+	NR_02738	.	misc_RNA	10004123	predicted	Gm3219
5996	Fam25c	1.319	1.262	1.664	0.009	0.166	0.169	8.599	8.998	9.334	69134	Fam25c	chr14	-	NM_1832	P6689	protein_cc	2200001I1	family wit	Fam25c
5997	Sncg	0.568	0.452	0.257	0.335	1.000	0.265	1.960	1.145	0.000	20618	Sncg	chr14	-	NM_0114	P21347	protein_cc	C79089| p	synuclein,	Sncg
5998	Mmrn2	0.876	0.779	0.683	0.447	0.269	0.248	7.672	7.482	7.122	105450	Mmrn2	chr14	+	NM_1531	P4507	protein_cc	AA986839	multimeri	Mmrn2
5999	Bmpr1a	1.266	1.177	1.491	0.180	0.220	0.114	6.903	7.243	7.479	12166	Bmpr1a	chr14	-	NM_0097	P22558	protein_cc	1110037I2	bone mor	Bmpr1a
6000	9230112D	1.000	1.000	1.000	1.000	1.000	1.000	0.000	0.000	0.000	78243	9230112D	chr14	-	NM_0300	P2953	protein_cc	.	RIKEN cDN	9230112D
6001	Ldb3	0.757	0.664	0.502	0.729	0.626	0.608	2.170	1.768	1.177	24131	Ldb3	chr14	-	NM_0010	P15171	protein_cc	AW74227	LIM doma	Ldb3
6002	Opn4	1.010	1.885	1.904	0.000	0.182	0.423	0.000	0.015	0.929	30044	Opn4	chr14	-	NM_0011	P20740	protein_cc	1110007J(	opsin 4 (m	Opn4
6003	Wapal	1.014	1.141	1.158	0.694	0.358	0.547	9.348	9.369	9.559	.	Wapal	chr14	+	NM_0013	P8049	.	.	.	Wapal
6004	Grid1	0.257	1.314	0.338	0.009	0.213	0.132	2.009	0.048	0.442	14803	Grid1	chr14	+	NM_0081	P8346	protein_cc	GluD1| Gl	glutamate	Grid1
6005	4930596D	1.000	1.000	1.000	1.000	1.000	1.000	0.000	0.000	0.000	239036	4930596D	chr14	-	NM_0010	P14033	protein_cc	.	RIKEN cDN	4930596D
6006	4930474N	1.000	1.000	1.000	1.000	1.000	1.000	0.000	0.000	0.000	218921	4930474N	chr14	+	NM_1750	P4013	protein_cc	.	RIKEN cDN	4930474N
6007	Ccser2	1.101	0.829	0.912	0.281	0.197	0.745	8.062	8.201	7.930	72972	Ccser2	chr14	-	NM_0284	P24837	protein_cc	1700012P	coiled-coil	Ccser2
6008	Rgr	0.405	0.985	0.399	0.181	1.000	0.423	1.324	0.022	0.000	57811	Rgr	chr14	-	NM_0213	P15041	protein_cc	.	retinal G p	Rgr
6009	Lrit1	0.094	1.228	0.115	0.000	0.633	0.006	6.938	3.524	3.821	239037	Lrit1	chr14	+	NM_1462	P11024	protein_cc	BC032270	leucine-ric	Lrit1
6010	Lrit2	0.030	1.466	0.044	0.097	0.000	0.322	5.230	0.166	0.718	239038	Lrit2	chr14	-	NM_1734	P14337	protein_cc	A930010E	leucine-ric	Lrit2
6011	Cdhr1	1.000	1.000	1.000	1.000	1.000	1.000	0.000	0.000	0.000	170677	Cdhr1	chr14	-	NM_1308	P6049	protein_cc	mKIAA177	cadherin-r	Cdhr1
6012	2610528A	1.947	0.821	1.599	0.172	0.637	0.598	1.720	2.681	2.397	70045	2610528A	chr14	-	NM_0012	P22884	protein_cc	AW49483	RIKEN cDN	2610528A
6013	Ghitm	1.227	0.876	1.075	0.000	0.034	0.442	11.642	11.937	11.746	66092	Ghitm	chr14	-	NM_0784	P21455	protein_cc	1010001P	growth ho	Ghitm
6014	Nrg3	1.000	1.000	1.000	1.000	1.000	1.000	0.000	0.000	0.000	18183	Nrg3	chr14	-	NM_0011	P5266	protein_cc	ska	neureguli	Nrg3
6015	Nrg3os	1.000	1.000	1.000	1.000	1.000	1.000	0.000	0.000	0.000	75170	Nrg3os	chr14	+	NR_04571	.	lncRNA	4930529F	neureguli	Nrg3os
6016	LOC43284	1.000	1.000	1.000	1.000	1.000	1.000	0.000	0.000	0.000	432842	LOC43284	chr14	-	NR_13197	.	lncRNA	.	uncharact	LOC43284
6017	1700109I(	1.000	1.000	1.000	1.000	1.000	1.000	0.000	0.000	0.000	73591	1700109I(	chr14	+	NR_04593	.	lncRNA	.	RIKEN cDN	1700109I0
6018	Sh2d4b	3.701	0.208	0.772	0.010	0.009	0.775	2.051	3.939	1.677	328381	Sh2d4b	chr14	-	NM_1778	P8339	protein_cc	A430109N	SH2 doma	Sh2d4b
6019	Tspan14	1.486	0.465	0.691	0.062	0.015	0.111	8.625	9.197	8.092	52588	Tspan14	chr14	-	NM_1459	P22268	protein_cc	AA958793	tetraspani	Tspan14
6020	Fam213a	0.639	0.884	0.565	0.001	0.061	0.037	8.445	7.798	7.620	70564	Fam213a	chr14	-	NM_0274	P18355	protein_cc	5730469N	family wit	Fam213a
6021	Dydc2	1.000	1.000	1.000	1.000	1.000	1.000	0.000	0.000	0.000	71200	Dydc2	chr14	-	NM_0277	P10721	protein_cc	4933428D	DPY30 do	Dydc2
6022	Dydc1	1.010	1.825	1.844	0.000	0.182	0.423	0.000	0.015	0.883	69496	Dydc1	chr14	+	NM_0270	P8069	protein_cc	1700029N	DPY30 do	Dydc1
6023	Mat1a	0.647	0.832	0.538	0.000	0.005	0.007	15.835	15.207	14.942	11720	Mat1a	chr14	+	NM_1336	P22611	protein_cc	AdoMet|	methionin	Mat1a
6024	Sftpa1	2.307	2.542	5.865	0.162	0.239	0.363	2.866	4.072	5.418	20387	Sftpa1	chr14	+	NM_0231	P25778	protein_cc	Sftp-1| Sft	surfactant	Sftpa1
6025	Mbl1	2.357	0.783	1.846	0.000	0.000	0.002	10.709	11.946	11.593	17194	Mbl1	chr14	+	NM_0107	P842	protein_cc	MBL-A| M	mannose-	Mbl1
6026	Sftpd	1.678	1.500	2.517	0.134	0.124	0.087	4.134	4.881	5.466	20390	Sftpd	chr14	-	NM_0091	P18211	protein_cc	AI573415|	surfactant	Sftpd
6027	Gm10377	1.005	1.439	1.446	0.000	0.182	0.423	0.000	0.008	0.532	10004205	Gm10377	chr14	-	NM_0012	P19126	protein_cc	ENSMUSG	predicted	Gm10377
6028	1700091H	1.000	1.000	1.000	1.000	1.000	1.000	0.000	0.000	0.000	73553	1700091H	chr14	-	NR_07336	.	lncRNA	10004193	RIKEN cDN	1700091H
6029	Gm17026	1.000	1.000	1.000	1.000	1.000	1.000	0.000	0.000	0.000	10105613	Gm17026	chr14	-	NR_10505	.	lncRNA		predicted	Gm17026
6030	1700001F	1.005	1.439	1.446	0.000	0.182	0.423	0.000	0.008	0.532	71826	1700001F	chr14	-	NM_0279	P26888	protein_cc	Gm10377	RIKEN cDN	1700001F
6031	Gm10375	1.000	1.000	1.000	1.000	1.000	1.000	0.000	0.000	0.000	10004234	Gm10375	chr14	-	NM_0010	P18311	protein_cc	ENSMUSG	predicted	Gm10375
6032	Ear1	3.277	0.381	1.247	0.023	0.029	0.713	2.782	4.495	3.101	13586	Ear1	chr14	-	NM_0078	P2968	protein_cc	EAR-1| EC	eosinophi	Ear1
6033	Ear10	0.622	0.458	0.285	0.507	1.000	0.423	1.811	1.125	0.000	93725	Ear10	chr14	-	NM_0531	P21025	protein_cc	Raf2	eosinophi	Ear10
6034	Ang3	1.000	1.000	1.000	1.000	1.000	1.000	0.000	0.000	0.000	.	Ang3	chr14	+	NM_0011	P4963	.	.	.	Ang3
6035	Ang5	1.000	1.000	1.000	1.000	1.000	1.000	0.000	0.000	0.000	503844	Ang5	chr14	+	NM_0074	P4963	protein_cc	.	angiogeni	Ang5
6036	Ang6	1.000	1.000	1.000	1.000	1.000	1.000	0.000	0.000	0.000	630952	Ang6	chr14	-	NM_0010	P12844	protein_cc	EG630952	angiogeni	Ang6
6037	Ear2	2.200	0.413	0.909	0.134	0.114	0.298	3.814	4.952	3.676	13587	Ear2	chr14	+	NM_0078	P18715	protein_cc	EAR-13| E	eosinophi	Ear2
6038	Ear3	0.869	0.295	0.257	0.759	0.013	0.362	3.010	2.807	1.047	.	Ear3	chr14	+	NM_0173	P18715	.	.	.	Ear3
6039	Ear12	0.869	0.295	0.257	0.759	0.013	0.362	3.010	2.807	1.047	.	Ear12	chr14	+	NM_0010	P18715	.	.	.	Ear12
6040	4930503E	1.000	1.000	1.000	1.000	1.000	1.000	0.000	0.000	0.000	74954	4930503E	chr14	-	NM_0291	P15291	protein_cc	.	RIKEN cDN	4930503E
6041	1700047E	0.567	1.777	1.008	0.186	0.000	0.988	0.846	0.027	0.857	74328	1700047E	chr14	-	NR_07336	.	lncRNA	.	RIKEN cDN	1700047E
6042	BC061237	1.010	1.885	1.904	0.000	0.182	0.423	0.000	0.015	0.929	385138	BC061237	chr14	+	NM_1986	P18997	protein_cc	.	cDNA seq	BC061237
6043	Gm8267	1.000	1.000	1.000	1.000	1.000	1.000	0.000	0.000	0.000	666744	Gm8267	chr14	-	NM_0011	P64	protein_cc	EG666744	predicted	Gm8267
6044	Ptgdr	1.000	1.000	1.000	1.000	1.000	1.000	0.000	0.000	0.000	19214	Ptgdr	chr14	-	NM_0089	P16250	protein_cc	DP| PGD	prostaglan	Ptgdr
6045	4930431P	0.339	0.981	0.333	0.181	1.000	0.423	1.588	0.028	0.000	73895	4930431P	chr14	-	NR_04505	.	lncRNA	.	RIKEN cDN	4930431P
6046	Ptger2	0.645	0.627	0.405	0.225	0.415	0.134	3.672	3.041	2.368	19217	Ptger2	chr14	+	NM_0089	P20884	protein_cc	EP2| Ptge	prostaglan	Ptger2
6047	Txndc16	0.139	0.848	0.118	0.000	0.604	0.006	5.270	2.425	2.187	70561	Txndc16	chr14	-	NM_1725	P11884	protein_cc	5730420B	thioredox	Txndc16
6048	Gpr137c	0.433	0.614	0.266	0.083	1.000	0.159	1.910	0.703	0.000	70713	Gpr137c	chr14	+	NM_0275	P8706	protein_cc	6330416L	G protein-	Gpr137c
6049	Ero1l	3.307	0.516	1.706	0.001	0.007	0.300	6.768	8.493	7.538	50527	Ero1l	chr14	-	NM_0157	P1482	protein_cc	ERO1-L| E	ERO1-like	Ero1l
6050	Psmc6	1.467	0.811	1.190	0.005	0.039	0.088	9.595	10.147	9.846	67089	Psmc6	chr14	+	NM_0259	P2902	protein_cc	2300001E	proteason	Psmc6
6051	Styx	0.676	1.485	1.004	0.059	0.051	0.978	7.095	6.530	7.100	56291	Styx	chr14	+	NM_0196	P21609	protein_cc	0610039A	serine/thr	Styx
6052	Gnpnat1	0.893	1.119	0.999	0.436	0.034	0.998	9.374	9.210	9.373	54342	Gnpnat1	chr14	-	NM_0194	P25785	protein_cc	AU017428	glucosami	Gnpnat1
6053	Fermt2	1.471	0.976	1.436	0.005	0.744	0.044	9.832	10.389	10.354	218952	Fermt2	chr14	-	NM_1460	P15621	protein_cc	AA960555	fermitin fa	Fermt2
6054	4930527F	8.142	0.597	4.858	0.025	0.353	0.423	0.002	3.027	2.282	67651	4930527F	chr14	+	NR_04580	.	lncRNA	.	RIKEN cDN	4930527F
6055	Ddhd1	1.256	0.665	0.835	0.002	0.020	0.439	6.234	6.562	5.973	114874	Ddhd1	chr14	-	NM_0012	P17165	protein_cc	Mir5131|	DDHD dor	Ddhd1
6056	Gm1821	2.545	0.715	1.820	0.002	0.048	0.070	10.669	12.016	11.533	218963	Gm1821	chr14	+	NR_00287	.	misc_RNA	.	predicted	Gm1821
6057	Gm15217	0.522	0.285	0.149	0.013	0.037	0.020	4.358	3.421	1.611	10004172	Gm15217	chr14	-	NR_03798	.	lncRNA	OTTMUSG	predicted	Gm15217
6058	Bmp4	0.625	0.995	0.622	0.016	0.971	0.057	6.743	6.065	6.058	12159	Bmp4	chr14	-	NM_0075	P4434	protein_cc	Bmp-4| Br	bone mor	Bmp4
6059	Rubie	6.904	0.477	3.292	0.003	0.099	0.423	0.002	2.789	1.720	10087309	Rubie	chr14	-	NR_04645	.	lncRNA	.	RNA upstr	Rubie
6060	4933425B	5.673	0.321	1.821	0.072	0.125	0.423	0.001	2.505	0.866	74458	4933425B	chr14	-	NR_04646	.	lncRNA	.	RIKEN cDN	4933425B
6061	D330046F	1.000	1.000	1.000	1.000	1.000	1.000	0.000	0.000	0.000	328385	D330046F	chr14	-	NR_13190	.	lncRNA	Gm808	RIKEN cDN	D330046F
6062	Cdkn3	0.511	2.202	1.125	0.186	0.016	0.844	1.003	0.033	1.172	72391	Cdkn3	chr14	+	NM_0282	P6743	protein_cc	2410006H	cyclin-dep	Cdkn3
6063	Cnih1	0.758	1.305	0.990	0.154	0.213	0.970	9.383	8.983	9.367	12793	Cnih1	chr14	-	NM_0099	P20735	protein_cc	0610007J1	cornichon	Cnih1
6064	Gmfb	1.312	1.139	1.495	0.003	0.027	0.005	7.828	8.220	8.407	63985	Gmfb	chr14	-	NM_0220	P21211	protein_cc	3110001H	glia matur	Gmfb
6065	Cgrrf1	0.655	1.152	0.754	0.005	0.137	0.164	7.984	7.373	7.577	68755	Cgrrf1	chr14	+	NM_0268	P13839	protein_cc	1110038G	cell growt	Cgrrf1
6066	Samd4	1.902	0.518	0.984	0.001	0.009	0.963	5.799	6.726	5.776	74480	Samd4	chr14	+	NM_0010	P10720	protein_cc	1700024G	sterile alp	Samd4
6067	Gm10371	2.762	1.438	3.971	0.300	0.586	0.325	0.001	1.467	1.991	10003865	Gm10371	chr14	-	NR_12657	.	lncRNA	ENSMUSG	predicted	Gm10371
6068	Gch1	0.482	0.767	0.369	0.000	0.162	0.027	11.557	10.503	10.121	14528	Gch1	chr14	-	NM_0081	P3292	protein_cc	Gch| GTP-	GTP cyclo	Gch1
6069	Wdhd1	1.488	1.486	2.211	0.407	0.111	0.198	2.470	3.044	3.615	218973	Wdhd1	chr14	-	NM_1725	P17801	protein_cc	A630092E	WD repea	Wdhd1
6070	Socs4	3.103	0.779	2.417	0.011	0.382	0.404	2.942	4.576	4.216	67296	Socs4	chr14	+	NM_0808	P8288	protein_cc	3110032N	suppresso	Socs4
6071	Mapk1ip1	1.024	0.963	0.986	0.635	0.731	0.933	9.261	9.294	9.240	218975	Mapk1ip1	chr14	+	NM_1786	P15881	protein_cc	9130202B	mitogen-a	Mapk1ip1
6072	Lgals3	3.137	0.753	2.361	0.000	0.015	0.048	6.431	8.081	7.671	16854	Lgals3	chr14	+	NM_0107	P16966	protein_cc	gal3| GBP	lectin, gal	Lgals3
6073	Dlgap5	2.162	1.029	2.226	0.300	0.954	0.135	0.001	1.113	1.155	218977	Dlgap5	chr14	-	NM_1445	P8829	protein_cc	C77459| C	discs, larg	Dlgap5
6074	Fbxo34	1.304	0.597	0.778	0.063	0.029	0.453	7.683	8.066	7.321	78938	Fbxo34	chr14	+	NR_02741	.	protein_cc	2900057B	F-box prot	Fbxo34
6075	Atg14	0.765	1.287	0.985	0.393	0.021	0.973	7.284	6.898	7.262	10050466	Atg14	chr14	-	NM_1725	P8843	protein_cc	4832427N	autophagy	Atg14
6076	Ktn1	2.005	0.667	1.338	0.020	0.066	0.035	8.717	9.721	9.137	16709	Ktn1	chr14	+	NM_0012	P22126	protein_cc	.	kinectin 1	Ktn1
6077	4930447J1	1.000	1.000	1.000	1.000	1.000	1.000	0.000	0.000	0.000	73961	4930447J1	chr14	-	NR_04595	.	lncRNA	4930425N	RIKEN cDN	4930447J1
6078	Peli2	0.288	1.986	0.573	0.000	0.045	0.100	8.471	6.677	7.667	93834	Peli2	chr14	+	NM_0336	P5515	protein_cc	AW04758	pellino 2	Peli2
6079	Gm6498	0.698	1.324	0.924	0.083	0.001	0.762	2.311	1.792	2.197	624367	Gm6498	chr14	+	NR_00363	.	misc_RNA	EG624367	predicted	Gm6498
6080	Tmem260	0.749	0.482	0.361	0.018	0.020	0.075	6.605	6.187	5.133	218989	Tmem260	chr14	+	NM_1726	P19074	protein_cc	5930435P	transmem	Tmem260
6081	Otx2	1.000	1.000	1.000	1.000	1.000	1.000	0.000	0.000	0.000	18424	Otx2	chr14	-	NM_0012	P14227	protein_cc	E130306E	orthodent	Otx2
6082	Otx2os1	1.000	1.000	1.000	1.000	1.000	1.000	0.000	0.000	0.000	606497	Otx2os1	chr14	+	NR_02938	.	lncRNA	.	orthodent	Otx2os1
6083	4933429O	1.000	1.000	1.000	1.000	1.000	1.000	0.000	0.000	0.000	66770	4933429O	chr14	+	NR_03378	.	lncRNA	1700031B	RIKEN cDN	4933429O
6084	Exoc5	1.408	1.152	1.622	0.011	0.406	0.221	7.869	8.362	8.566	105504	Exoc5	chr14	-	NM_2072	P102	protein_cc	AI447711|	exocyst cc	Exoc5

6085	Ap5m1	0.652	1.791	1.168	0.167	0.030	0.551	6.110	5.492	6.334	74385	Ap5m1	chr14	+	NM_1445	P4934	protein_cc	4932432K	adaptor-re	Ap5m1
6086	Naa30	1.208	1.116	1.348	0.180	0.381	0.192	7.852	8.124	8.283	70646	Naa30	chr14	+	NM_0010	P15117	protein_cc	4930487N	N(alpha)-a	Naa30
6087	1700011H	0.216	1.773	0.384	0.027	0.196	0.276	2.269	0.060	0.887	67082	1700011H	chr14	-	NM_0259	P10421	protein_cc	.	RIKEN cDN	1700011H
6088	Slc35f4	1.000	1.000	1.000	1.000	1.000	1.000	0.000	0.000	0.000	75288	Slc35f4	chr14	-	NM_0292	P14243	protein_cc	4930550L	solute carr	Slc35f4
6089	3632451O	1.160	0.898	1.041	0.707	0.786	0.930	0.455	0.669	0.513	67419	3632451O	chr14	-	NM_0261	P5493	protein_cc	AU067705	RIKEN cDN	3632451O
6090	Olfr722	1.000	1.000	1.000	1.000	1.000	1.000	0.000	0.000	0.000	258487	Olfr722	chr14	-	NM_1464	P20310	protein_cc	MOR241-3	olfactory r	Olfr722
6091	Olfr723	1.000	1.000	1.000	1.000	1.000	1.000	0.000	0.000	0.000	259147	Olfr723	chr14	-	NM_0010	P523	protein_cc	MOR247-3	olfactory r	Olfr723
6092	Olfr724	1.000	1.000	1.000	1.000	1.000	1.000	0.000	0.000	0.000	258485	Olfr724	chr14	-	NM_1464	P981	protein_cc	MOR247-2	olfactory r	Olfr724
6093	Olfr725	1.000	1.000	1.000	1.000	1.000	1.000	0.000	0.000	0.000	258314	Olfr725	chr14	-	NM_1463	P18499	protein_cc	MOR246-3	olfactory r	Olfr725
6094	Olfr726	1.000	1.000	1.000	1.000	1.000	1.000	0.000	0.000	0.000	258313	Olfr726	chr14	-	NM_1463	P2664	protein_cc	MOR246-4	olfactory r	Olfr726
6095	Olfr727	1.000	1.000	1.000	1.000	1.000	1.000	0.000	0.000	0.000	258316	Olfr727	chr14	+	NM_1463	P21129	protein_cc	MOR246-2	olfactory r	Olfr727
6096	Olfr728	1.000	1.000	1.000	1.000	1.000	1.000	0.000	0.000	0.000	258039	Olfr728	chr14	-	NM_0010	P12449	protein_cc	MOR246-1	olfactory r	Olfr728
6097	Olfr729	1.000	1.000	1.000	1.000	1.000	1.000	0.000	0.000	0.000	258275	Olfr729	chr14	-	NM_1462	P3034	protein_cc	MOR246-6	olfactory r	Olfr729
6098	Olfr730	1.000	1.000	1.000	1.000	1.000	1.000	0.000	0.000	0.000	258486	Olfr730	chr14	-	NM_1464	P13768	protein_cc	MOR247-1	olfactory r	Olfr730
6099	Olfr731	1.000	1.000	1.000	1.000	1.000	1.000	0.000	0.000	0.000	258360	Olfr731	chr14	-	NM_1463	P7065	protein_cc	MOR246-5	olfactory r	Olfr731
6100	Olfr732	1.000	1.000	1.000	1.000	1.000	1.000	0.000	0.000	0.000	258659	Olfr732	chr14	-	NM_1466	P24921	protein_cc	MOR241-1	olfactory r	Olfr732
6101	Olfr733	1.000	1.000	1.000	1.000	1.000	1.000	0.000	0.000	0.000	258657	Olfr733	chr14	-	NM_1466	P6504	protein_cc	MOR241-2	olfactory r	Olfr733
6102	Olfr734	1.000	1.000	1.000	1.000	1.000	1.000	0.000	0.000	0.000	258658	Olfr734	chr14	-	NM_1466	P4115	protein_cc	MOR242-1	olfactory r	Olfr734
6103	Olfr735	1.000	1.000	1.000	1.000	1.000	1.000	0.000	0.000	0.000	257909	Olfr735	chr14	-	NM_0010	P20275	protein_cc	MOR243-1	olfactory r	Olfr735
6104	Tlr11	0.318	0.977	0.311	0.000	1.000	0.009	1.685	0.033	0.000	239081	Tlr11	chr14	+	NM_2058	P6767	protein_cc	Gm287	toll-like re	Tlr11
6105	Olfr736	1.000	1.000	1.000	1.000	1.000	1.000	0.000	0.000	0.000	258660	Olfr736	chr14	+	NM_1466	P16829	protein_cc	MOR106-1	olfactory r	Olfr736
6106	Olfr738	1.000	1.000	1.000	1.000	1.000	1.000	0.000	0.000	0.000	258662	Olfr738	chr14	+	NM_1464	P12004	protein_cc	MOR106-3	olfactory r	Olfr738
6107	Olfr739	1.000	1.000	1.000	1.000	1.000	1.000	0.000	0.000	0.000	258663	Olfr739	chr14	+	NM_1466	P18835	protein_cc	MOR106-2	olfactory r	Olfr739
6108	Olfr740	1.000	1.000	1.000	1.000	1.000	1.000	0.000	0.000	0.000	258661	Olfr740	chr14	+	NM_1466	P16027	protein_cc	MOR106-4	olfactory r	Olfr740
6109	Olfr741	1.000	1.000	1.000	1.000	1.000	1.000	0.000	0.000	0.000	258233	Olfr741	chr14	+	NM_2071	P2547	protein_cc	GA_x5J8B	olfactory r	Olfr741
6110	4930413M	1.000	1.000	1.000	1.000	1.000	1.000	0.000	0.000	0.000	73889	4930413M	chr14	+	NR_04575	.	lncRNA	.	RIKEN cDN	4930413M
6111	Olfr742	0.716	0.995	0.712	0.182	1.000	0.423	0.490	0.008	0.000	258422	Olfr742	chr14	+	NM_1464	P15513	protein_cc	MOR106-6	olfactory r	Olfr742
6112	Olfr743	1.000	1.000	1.000	1.000	1.000	1.000	0.000	0.000	0.000	219019	Olfr743	chr14	+	NM_0011	P1068	protein_cc	GA_x6K02	olfactory r	Olfr743
6113	Olfr744	1.000	1.000	1.000	1.000	1.000	1.000	0.000	0.000	0.000	257884	Olfr744	chr14	+	NM_0010	P3373	protein_cc	MOR106-1	olfactory r	Olfr744
6114	Olfr745	1.000	1.000	1.000	1.000	1.000	1.000	0.000	0.000	0.000	258296	Olfr745	chr14	+	NM_1462	P21029	protein_cc	MOR106-1	olfactory r	Olfr745
6115	Olfr746	1.000	1.000	1.000	1.000	1.000	1.000	0.000	0.000	0.000	258295	Olfr746	chr14	+	NM_1462	P6788	protein_cc	MOR106-1	olfactory r	Olfr746
6116	Olfr747	1.000	1.000	1.000	1.000	1.000	1.000	0.000	0.000	0.000	258264	Olfr747	chr14	-	NM_2071	P2233	protein_cc	MOR106-1	olfactory r	Olfr747
6117	Olfr748	1.000	1.000	1.000	1.000	1.000	1.000	0.000	0.000	0.000	258113	Olfr748	chr14	+	NM_0010	P13555	protein_cc	MOR106-9	olfactory r	Olfr748
6118	Olfr749	1.000	1.000	1.000	1.000	1.000	1.000	0.000	0.000	0.000	56858	Olfr749	chr14	-	NM_0202	P25406	protein_cc	MOR106-1	olfactory r	Olfr749
6119	Ttc5	0.631	2.814	1.774	0.000	0.000	0.023	4.319	3.653	5.146	219022	Ttc5	chr14	-	NM_0010	P8962	protein_cc	.	tetratricop	Ttc5
6120	Ccnb1ip1	0.716	0.995	0.712	0.182	1.000	0.423	0.490	0.008	0.000	239083	Ccnb1ip1	chr14	-	NM_0011	P22911	protein_cc	Gm288| H	cyclin B1 i	Ccnb1ip1
6121	Rpph1	1.985	0.738	1.466	0.081	0.585	0.749	5.212	6.201	5.763	85029	Rpph1	chr14	-	NR_00214	.	RNase_P_	Gm24821	ribonuclea	Rpph1
6122	Parp2	1.731	0.849	1.470	0.010	0.410	0.363	6.545	7.337	7.102	11546	Parp2	chr14	+	NM_0096	P9879	protein_cc	Adprt2| A	poly (ADP-	Parp2
6123	Tep1	0.463	1.421	0.659	0.000	0.000	0.024	5.867	4.758	5.265	21745	Tep1	chr14	-	NM_0093	P20005	protein_cc	Tp1	telomeras	Tep1
6124	Klhl33	0.716	0.995	0.712	0.182	1.000	0.423	0.490	0.008	0.000	546611	Klhl33	chr14	-	NM_0011	P10058	protein_cc	EG546611	kelch-like	Klhl33
6125	Osgep	1.259	0.800	1.008	0.075	0.102	0.965	7.034	7.366	7.045	66246	Osgep	chr14	-	NM_1336	P20866	protein_cc	1500019L	O-sialoglyc	Osgep
6126	Apex1	5.483	0.651	3.572	0.000	0.001	0.006	7.843	10.298	9.680	11792	Apex1	chr14	+	NM_0096	P11256	protein_cc	APE| Apex	apurinic/a	Apex1
6127	Tmem55b	3.334	0.776	2.588	0.000	0.006	0.007	8.737	10.475	10.109	219024	Tmem55b	chr14	-	NM_0010	P7955	protein_cc	BC039161	transmem	Tmem55b
6128	Pnp	7.246	0.552	4.001	0.000	0.006	0.058	9.849	12.706	11.850	18950	Pnp	chr14	+	NM_0136	P22669	protein_cc	AL024301	purine-nu	Pnp
6129	Pnp2	3.264	1.468	4.791	0.013	0.258	0.153	2.453	4.159	4.713	667034	Pnp2	chr14	+	NM_0011	P20932	protein_cc	.	purine-nu	Pnp2
6130	Rnase10	0.250	2.213	0.554	0.004	0.189	0.357	2.055	0.058	1.203	75019	Rnase10	chr14	+	NM_0011	P1427	protein_cc	4930474F	ribonuclea	Rnase10
6131	Rnase9	1.000	1.000	1.000	1.000	1.000	1.000	0.000	0.000	0.000	328401	Rnase9	chr14	-	NM_1830	P23188	protein_cc	ESRL| Rak	ribonuclea	Rnase9
6132	Rnase11	1.000	1.000	1.000	1.000	1.000	1.000	0.000	0.000	0.000	497113	Rnase11	chr14	-	NM_0010	P14787	protein_cc	Raj1	ribonuclea	Rnase11
6133	Rnase12	1.000	1.000	1.000	1.000	1.000	1.000	0.000	0.000	0.000	497106	Rnase12	chr14	-	NM_0010	P20995	protein_cc	Rai1	ribonuclea	Rnase12
6134	Olfr750	1.000	1.000	1.000	1.000	1.000	1.000	0.000	0.000	0.000	404319	Olfr750	chr14	-	NM_2075	P25881	protein_cc	GA_x5J8B	olfactory r	Olfr750
6135	Ang	0.592	0.599	0.354	0.001	0.005	0.012	12.512	11.756	11.015	11727	Ang	chr14	+	NM_0011	P4196	protein_cc	AI385586|	angiogenii	Ang
6136	Rnase4	0.220	0.938	0.207	0.000	0.279	0.004	14.837	12.653	12.561	58809	Rnase4	chr14	+	NM_0214	P2361	protein_cc	C730049F	ribonuclea	Rnase4
6137	Eddm3b	1.000	1.000	1.000	1.000	1.000	1.000	0.000	0.000	0.000	219026	Eddm3b	chr14	+	NM_2035	P24643	protein_cc	Fam12| G	epididyma	Eddm3b
6138	Rnase6	0.425	0.842	0.358	0.231	0.640	0.419	3.037	1.800	1.553	78416	Rnase6	chr14	+	NM_0300	P21002	protein_cc	9530043P	ribonuclea	Rnase6
6139	Rnase1	1.000	1.000	1.000	1.000	1.000	1.000	0.000	0.000	0.000	19752	Rnase1	chr14	-	NM_0112	P25069	protein_cc	AI574248|	ribonuclea	Rnase1
6140	Rnase2b	1.000	1.000	1.000	1.000	1.000	1.000	0.000	0.000	0.000	54159	Rnase2b	chr14	+	NM_0193	P12178	protein_cc	Ear5| mR	ribonuclea	Rnase2b
6141	Ang2	1.005	1.405	1.412	0.000	0.182	0.423	0.000	0.008	0.498	11731	Ang2	chr14	-	NM_0074	P23111	protein_cc	Angrp| Ra	angiogenii	Ang2
6142	Ear4	1.000	1.000	1.000	1.000	1.000	1.000	0.000	0.000	0.000	.	Ear4	chr14	+	NM_0010	P22971	.	.	.	Ear4
6143	Ear14	1.000	1.000	1.000	1.000	1.000	1.000	0.000	0.000	0.000	503847	Ear14	chr14	+	NM_0173	P5355	protein_cc	.	eosinophil	Ear14
6144	Rnase2a	1.010	1.885	1.904	0.000	0.182	0.423	0.000	0.015	0.929	93726	Rnase2a	chr14	-	NM_0531	P23990	protein_cc	Ear11	ribonuclea	Rnase2a
6145	4930597G	1.000	1.000	1.000	1.000	1.000	1.000	0.000	0.000	0.000	75377	4930597G	chr14	-	NR_04573	.	lncRNA	.	RIKEN cDN	4930597G
6146	Vmn2r88	1.000	1.000	1.000	1.000	1.000	1.000	0.000	0.000	0.000	669149	Vmn2r88	chr14	+	NM_0116	P9117	protein_cc	V2r13| V2	vomerona	Vmn2r88
6147	Vmn2r89	1.000	1.000	1.000	1.000	1.000	1.000	0.000	0.000	0.000	22301	Vmn2r89	chr14	+	NM_0094	P22323	protein_cc	V2r10| V2	vomerona	Vmn2r89
6148	Gm5622	1.000	1.000	1.000	1.000	1.000	1.000	0.000	0.000	0.000	434459	Gm5622	chr14	+	NM_0010	P18600	protein_cc	EG434459	predicted	Gm5622
6149	Gm5800	1.000	1.000	1.000	1.000	1.000	1.000	0.000	0.000	0.000	545047	Gm5800	chr14	-	NM_0010	P2248	protein_cc	EG545047	predicted	Gm5800
6150	Ang4	0.669	0.995	0.666	0.181	1.000	0.423	0.587	0.008	0.000	219033	Ang4	chr14	-	NM_1775	P24112	protein_cc	Raa4| Rna	angiogenii	Ang4
6151	AY358078	1.239	0.415	0.514	0.541	0.117	0.115	1.443	1.753	0.483	278676	AY358078	chr14	+	NM_1943	P18584	protein_cc	.	cDNA seq	AY358078
6152	Ear6	1.000	1.000	1.000	1.000	1.000	1.000	0.000	0.000	0.000	93719	Ear6	chr14	+	NM_0531	P19071	protein_cc	.	eosinophil	Ear6
6153	Ear7	1.000	1.000	1.000	1.000	1.000	1.000	0.000	0.000	0.000	.	Ear7	chr14	+	NM_0173	P19071	.	.	.	Ear7
6154	Mettl17	0.353	0.728	0.257	0.003	0.289	0.060	6.941	5.440	4.982	52535	Mettl17	chr14	+	NM_0010	P13690	protein_cc	2310032K	methyltra	Mettl17
6155	Slc39a2	0.914	0.294	0.269	0.814	1.000	0.172	1.894	1.764	0.000	214922	Slc39a2	chr14	+	NM_0010	P5286	protein_cc	F730005G	solute carr	Slc39a2
6156	Ndrg2	0.066	1.460	0.097	0.000	0.050	0.007	14.591	10.673	11.219	29811	Ndrg2	chr14	+	NM_0011	P2054	protein_cc	AI182517|	N-myc dov	Ndrg2
6157	Tppp2	1.000	1.000	1.000	1.000	1.000	1.000	0.000	0.000	0.000	219038	Tppp2	chr14	+	NM_0011	P21831	protein_cc	Gm1790|	tubulin po	Tppp2
6158	Rnase13	1.000	1.000	1.000	1.000	1.000	1.000	0.000	0.000	0.000	497071	Rnase13	chr14	-	NM_0010	P14345	protein_cc	.	ribonuclea	Rnase13
6159	Arhgef40	0.769	0.861	0.662	0.008	0.109	0.029	7.219	6.840	6.624	268739	Arhgef40	chr14	+	NM_1982	P1485	protein_cc	E130112L	Rho guani	Arhgef40
6160	Gm16617	0.838	0.737	0.618	0.306	0.390	0.473	2.033	1.777	1.338	10050276	Gm16617	chr14	-	NR_04572	.	lncRNA	.	predicted	Gm16617
6161	Zfp219	0.957	0.806	0.772	0.618	0.072	0.022	8.871	8.808	8.497	69890	Zfp219	chr14	-	NM_0012	P6464	protein_cc	2010302A	zinc finger	Zfp219
6162	Tmem253	0.669	0.995	0.666	0.181	1.000	0.423	0.587	0.008	0.000	619301	Tmem253	chr14	+	NM_0010	P1253	protein_cc	G630016D	transmem	Tmem253
6163	Olfr221	1.000	1.000	1.000	1.000	1.000	1.000	0.000	0.000	0.000	258420	Olfr221	chr14	-	NM_0010	P4822	protein_cc	MOR205-1	olfactory r	Olfr221
6164	Snord58b	2.144	0.641	1.374	0.303	0.482	0.423	0.000	1.101	0.458	10021745	Snord58b	chr14	+	NR_02855	.	snoRNA	MBII-199	small nucl	Snord58b
6165	Hnrnpc	1.220	0.646	0.788	0.035	0.009	0.215	10.202	10.488	9.857	15381	Hnrnpc	chr14	-	NM_0168	P9953	protein_cc	AL022939	heteroger	Hnrnpc
6166	Rpgrip1	1.544	0.906	1.398	0.005	0.211	0.055	6.857	7.483	7.341	77945	Rpgrip1	chr14	+	NM_0238	P21163	protein_cc	0610005A	retinitis pi	Rpgrip1
6167	Supt16	1.299	0.918	1.193	0.001	0.379	0.387	8.217	8.595	8.472	114741	Supt16	chr14	-	NM_0336	P16267	protein_cc	Cdc68| Fa	suppresso	Supt16
6168	Chd8	0.953	0.990	0.944	0.270	0.875	0.535	8.044	7.975	7.961	67772	Chd8	chr14	-	NM_2016	P7580	protein_cc	5830451P	chromodo	Chd8
6169	Snord8	1.159	0.870	1.008	0.708	0.723	0.985	0.455	0.668	0.467	10021744	Snord8	chr14	-	NR_02854	.	snoRNA	MBII-28	small nucl	Snord8
6170	Rab2b	0.958	0.610	0.585	0.663	0.030	0.104	5.231	5.170	4.458	76338	Rab2b	chr14	-	NM_1726	P6150	protein_cc	1500012D	RAB2B, m	Rab2b
6171	Tox4	2.287	0.971	2.220	0.000	0.862	0.178	7.431	8.624	8.581	268741	Tox4	chr14	+	NM_0234	P602	protein_cc	5730589K	TOX high r	Tox4
6172	Mettl3	1.119	0.859	0.961	0.041	0.126	0.772	6.151	6.313	6.094	56335	Mettl3	chr14	-	NM_0197	P6305	protein_cc	2310024F	methyltra	Mettl3
6173	Sall2	0.791	1.631	1.289	0.442	0.013	0.511	4.455	4.116	4.821	50524	Sall2	chr14	-	NM_0157	P1321	protein_cc	AI225809|	sal-like 2 (	Sall2
6174	Olfr1513	1.000	1.000	1.000	1.000	1.000	1.000	0.000	0.000	0.000	258008	Olfr1513	chr14	-	NM_0010	P8722	protein_cc	MOR223-1	olfactory r	Olfr1513
6175	Olfr1512	1.000	1.000	1.000	1.000	1.000	1.000	0.000	0.000	0.000	258424	Olfr1512	chr14	-	NM_1464	P8570	protein_cc	MOR223-5	olfactory r	Olfr1512

6176	Olfr1511	1.000	1.000	1.000	1.000	1.000	1.000	0.000	0.000	0.000	258268	Olfr1511	chr14	-	NM_1462	P15650	protein_cc	MOR223-!	olfactory r	Olfr1511
6177	Olfr1510	1.000	1.000	1.000	1.000	1.000	1.000	0.000	0.000	0.000	258423	Olfr1510	chr14	-	NM_1464	P20829	protein_cc	MOR223-(	olfactory r	Olfr1510
6178	Olfr1509	1.000	1.000	1.000	1.000	1.000	1.000	0.000	0.000	0.000	57271	Olfr1509	chr14	+	NM_0205	P9622	protein_cc	MOR244-:	olfactory r	Olfr1509
6179	Olfr1508	1.000	1.000	1.000	1.000	1.000	1.000	0.000	0.000	0.000	57270	Olfr1508	chr14	-	NM_0205	P16731	protein_cc	Mor10| M	olfactory r	Olfr1508
6180	Olfr1507	1.000	1.000	1.000	1.000	1.000	1.000	0.000	0.000	0.000	57269	Olfr1507	chr14	-	NM_0011	P1040	protein_cc	MOR244-1	olfactory r	Olfr1507
6181	Dad1	1.097	1.022	1.121	0.179	0.796	0.355	10.325	10.459	10.490	13135	Dad1	chr14	-	NM_0011	P17302	protein_cc	AI323713	defender a	Dad1
6182	Abhd4	1.136	1.141	1.296	0.032	0.102	0.106	8.652	8.836	9.026	105501	Abhd4	chr14	+	NM_0012	P17492	protein_cc	1110035H	abhydrola	Abhd4
6183	Olfr49	1.000	1.000	1.000	1.000	1.000	1.000	0.000	0.000	0.000	18348	Olfr49	chr14	-	NM_0109	P2818	protein_cc	IC6| MOR	olfactory r	Olfr49
6184	Oxa1l	0.525	1.297	0.680	0.002	0.184	0.191	8.762	7.832	8.207	69089	Oxa1l	chr14	+	NM_0269	P10213	protein_cc	1810020M	oxidase as	Oxa1l
6185	Slc7a7	1.604	1.107	1.776	0.021	0.769	0.463	5.090	5.772	5.919	20540	Slc7a7	chr14	-	NM_0114	P3448	protein_cc	AI790233|	solute car	Slc7a7
6186	Mrpl52	1.301	1.106	1.440	0.005	0.304	0.125	9.622	10.002	10.148	68836	Mrpl52	chr14	+	NM_0268	P6894	protein_cc	1110047B	mitochond	Mrpl52
6187	Mmp14	1.395	1.572	2.192	0.005	0.142	0.232	8.963	9.443	10.095	17387	Mmp14	chr14	+	NM_0086	P20894	protein_cc	AI325305|	matrix me	Mmp14
6188	Lrp10	1.927	0.885	1.705	0.002	0.013	0.082	7.740	8.686	8.509	65107	Lrp10	chr14	+	NM_0229	P4100	protein_cc	Lrp9	low-densi	Lrp10
6189	Rem2	1.010	1.885	1.904	0.000	0.182	0.423	0.000	0.015	0.929	140743	Rem2	chr14	+	NM_0807	P23827	protein_cc	AW41189	rad and ge	Rem2
6190	Prmt5	1.875	0.966	1.811	0.001	0.229	0.034	7.402	8.309	8.258	27374	Prmt5	chr14	-	NM_0137	P9889	protein_cc	Jbp1| Skb	protein ar	Prmt5
6191	Haus4	0.168	3.254	0.547	0.000	0.000	0.023	6.301	3.728	5.430	219072	Haus4	chr14	-	NM_1454	P13954	protein_cc	9430093H	HAUS aug	Haus4
6192	Ajuba	0.624	2.446	1.526	0.098	0.076	0.480	5.617	4.936	6.226	16475	Ajuba	chr14	-	NM_0105	P8015	protein_cc	Jub	ajuba LIM	Ajuba
6193	4931414P	0.466	0.756	0.352	0.060	0.398	0.227	5.791	4.689	4.285	74359	4931414P	chr14	-	NM_0288	P16635	protein_cc	.	RIKEN cDN	4931414P1
6194	Psmb5	1.319	1.270	1.674	0.121	0.136	0.061	9.931	10.330	10.675	19173	Psmb5	chr14	-	NM_0111	P4765	protein_cc	.	proteason	Psmb5
6195	Psmb11	0.544	2.093	1.139	0.361	0.058	0.863	1.589	0.711	1.776	73902	Psmb11	chr14	+	NM_1752	P16555	protein_cc	5830406J	proteason	Psmb11
6196	Cdh24	1.000	1.000	1.000	1.000	1.000	1.000	0.000	0.000	0.000	239096	Cdh24	chr14	-	NM_1994	P2396	protein_cc	1700040A	cadherin-l	Cdh24
6197	Acin1	1.457	0.631	0.919	0.044	0.025	0.342	9.521	10.064	9.398	56215	Acin1	chr14	-	NM_0195	P22757	protein_cc	2610036I1	apoptotic	Acin1
6198	4930579G	1.859	0.862	1.602	0.022	0.411	0.262	4.743	5.638	5.424	75913	4930579G	chr14	+	NR_03805	.	lncRNA		RIKEN cDN	4930579G
6199	1700123O	2.127	0.884	1.879	0.000	0.200	0.081	7.890	8.979	8.800	58248	1700123O	chr14	+	NM_0214	P8252	protein_cc	MNCb-29	RIKEN cDN	1700123O
6200	Cebpe	0.224	3.564	0.799	0.000	0.000	0.116	4.873	2.716	4.549	110794	Cebpe	chr14	-	NM_2071	P5312	protein_cc	C/EBPe| C	CCAAT/en	Cebpe
6201	Slc7a8	0.413	1.185	0.489	0.000	0.041	0.002	5.237	3.961	4.205	50934	Slc7a8	chr14	-	NM_0169	P10801	protein_cc	AA408822	solute car	Slc7a8
6202	Homez	1.358	0.805	1.093	0.399	0.527	0.517	4.826	5.268	4.955	239099	Homez	chr14	-	NM_0011	P6795	protein_cc	mKIAA144	homeodo	Homez
6203	Ppp1r3e	1.164	0.539	0.628	0.704	0.037	0.655	1.546	1.765	0.874	105651	Ppp1r3e	chr14	-	NM_0011	P1594	protein_cc	A630071A	protein ph	Ppp1r3e
6204	Bcl2l2	1.424	0.833	1.187	0.174	0.007	0.695	4.682	5.193	4.929	12050	Bcl2l2	chr14	+	NM_0075	P22836	protein_cc	AW04883	BCL2-like	Bcl2l2
6205	Pabpn1	1.236	1.093	1.350	0.181	0.465	0.021	9.884	10.189	10.317	54196	Pabpn1	chr14	+	NM_0194	P16478	protein_cc	mPABII| P	poly(A) bi	Pabpn1
6206	Slc22a17	0.271	1.670	0.453	0.004	0.234	0.021	3.060	1.177	1.917	59049	Slc22a17	chr14	-	NM_0215	P10843	protein_cc	1700094C	solute car	Slc22a17
6207	Efs	1.000	1.000	1.000	1.000	1.000	1.000	0.000	0.000	0.000	13644	Efs	chr14	-	NM_0101	P20456	protein_cc	.	embryona	Efs
6208	Il25	1.000	1.000	1.000	1.000	1.000	1.000	0.000	0.000	0.000	140806	Il25	chr14	+	NM_0807	P10386	protein_cc	IL-17e| Il1	interleuki	Il25
6209	Cmtm5	1.000	1.000	1.000	1.000	1.000	1.000	0.000	0.000	0.000	67272	Cmtm5	chr14	+	NM_0260	P24123	protein_cc	1500005P	CKLF-like	Cmtm5
6210	Myh6	1.000	1.000	1.000	1.000	1.000	1.000	0.000	0.000	0.000	17888	Myh6	chr14	-	NM_0108	P1681	protein_cc	A830009F	myosin, h	Myh6
6211	Mhrt	1.000	1.000	1.000	1.000	1.000	1.000	0.000	0.000	0.000	791403	Mhrt	chr14	+	NR_03349	.	lncRNA	D830015G	myosin he	Mhrt
6212	Myh7	0.332	0.978	0.324	0.181	1.000	0.423	1.625	0.032	0.000	140781	Myh7	chr14	-	NM_0807	P7038	protein_cc	B-MHC| b	myosin, h	Myh7
6213	Ngdn	3.187	0.794	2.530	0.037	0.406	0.042	8.480	10.152	9.819	68966	Ngdn	chr14	+	NM_0268	P21477	protein_cc	1500001L	neuroguid	Ngdn
6214	Gm10364	1.509	2.829	4.270	0.068	0.001	0.029	0.588	1.182	2.682	10015169	Gm10364	chr14	+	NR_07353	.	misc_RNA	EG545052	predicted	Gm10364
6215	Zfhx2	0.942	1.071	1.009	0.491	0.434	0.899	6.864	6.778	6.877	239102	Zfhx2	chr14	-	NM_0010	P21891	protein_cc	ZFH-5	zinc finger	Zfhx2
6216	Zfhx2os	1.000	1.000	1.000	1.000	1.000	1.000	0.000	0.000	0.000	432855	Zfhx2os	chr14	+	NR_00444	.	antisense	Zfhx2as	zinc finger	Zfhx2os
6217	Thtpa	0.981	0.941	0.923	0.893	0.671	0.541	7.030	7.002	6.914	105663	Thtpa	chr14	+	NM_1530	P21149	protein_cc	AW12296	thiamine t	Thtpa
6218	Ap1g2	0.981	1.148	1.127	0.925	0.491	0.558	6.728	6.701	6.900	11766	Ap1g2	chr14	-	NM_0013	P10680	protein_cc	Adtg2| G2	adaptor p	Ap1g2
6219	Jph4	1.000	1.000	1.000	1.000	1.000	1.000	0.000	0.000	0.000	319984	Jph4	chr14	-	NM_1770	P15993	protein_cc	9330157P	junctophil	Jph4
6220	Dhrs2	1.000	1.000	1.000	1.000	1.000	1.000	0.000	0.000	0.000	71412	Dhrs2	chr14	+	NM_0277	P16022	protein_cc	5430405K	dehydrog	Dhrs2
6221	Dhrs4	0.343	1.372	0.470	0.000	0.001	0.022	11.554	10.010	10.466	28200	Dhrs4	chr14	+	NM_0306	P4906	protein_cc	AI043103|	dehydrog	Dhrs4
6222	Lrrc16b	0.683	3.019	2.063	0.197	0.182	0.536	0.587	0.038	1.632	268747	Lrrc16b	chr14	+	NM_0010	P20711	protein_cc	CARMIL3|	leucine ric	Lrrc16b
6223	Cpne6	1.000	1.000	1.000	1.000	1.000	1.000	0.000	0.000	0.000	12891	Cpne6	chr14	+	NM_0011	P18513	protein_cc	AU067659	copine VI	Cpne6
6224	Nrl	1.000	1.000	1.000	1.000	1.000	1.000	0.000	0.000	0.000	18185	Nrl	chr14	-	NM_0012	P20483	protein_cc	D14H14S4	neural ret	Nrl
6225	Pck2	0.937	1.045	0.978	0.686	0.829	0.959	4.397	4.303	4.365	74551	Pck2	chr14	+	NM_0289	P5729	protein_cc	1810010O	phosphoe	Pck2
6226	Dcaf11	0.284	1.080	0.307	0.000	0.323	0.004	10.833	9.017	9.128	28199	Dcaf11	chr14	+	NR_03757	.	protein_cc	0710008A	DDB1 and	Dcaf11
6227	Fitm1	0.258	3.550	0.916	0.036	0.043	0.886	5.862	3.907	5.735	68680	Fitm1	chr14	+	NM_0268	P9372	protein_cc	1110028A	fat storag	Fitm1
6228	Psme1	2.169	0.946	2.052	0.000	0.027	0.001	9.393	10.510	10.430	19186	Psme1	chr14	+	NM_0111	P10347	protein_cc	AW41392	proteason	Psme1
6229	Emc9	1.167	1.239	1.445	0.027	0.002	0.030	8.397	8.619	8.928	85308	Emc9	chr14	-	NM_0331	P23950	protein_cc	1500005A	ER membr	Emc9
6230	Psme2	2.636	0.815	2.149	0.003	0.126	0.002	8.349	9.748	9.453	19188	Psme2	chr14	-	NM_0010	P10393	protein_cc	AA589371	proteason	Psme2
6231	Rnf31	1.724	0.371	0.639	0.176	0.068	0.095	5.929	6.714	5.283	268749	Rnf31	chr14	+	NM_1943	P2597	protein_cc	AL033293	ring finger	Rnf31
6232	Irf9	1.998	0.624	1.247	0.000	0.013	0.429	8.767	9.765	9.085	16391	Irf9	chr14	+	NM_0011	P26682	protein_cc	Irf-9| Isgf3	interferon	Irf9
6233	Rec8	0.290	4.066	1.179	0.000	0.002	0.505	1.858	0.072	2.095	56739	Rec8	chr14	+	NM_0200	P17167	protein_cc	mrec| Rec	REC8 meio	Rec8
6234	Ipo4	1.031	1.232	1.270	0.292	0.440	0.614	7.084	7.128	7.429	75751	Ipo4	chr14	-	NM_0242	P12041	protein_cc	8430408O	importin 4	Ipo4
6235	Tm9sf1	1.064	1.080	1.149	0.872	0.837	0.834	6.611	6.700	6.811	74140	Tm9sf1	chr14	-	NM_0287	P5950	protein_cc	1200014D	transmem	Tm9sf1
6236	Tssk4	1.026	3.449	3.539	0.000	0.181	0.423	0.001	0.038	1.824	71099	Tssk4	chr14	+	NM_0276	P25333	protein_cc	1700020B	testis-spe	Tssk4
6237	Mdp1	0.486	2.080	1.011	0.028	0.000	0.968	7.866	6.826	7.882	67881	Mdp1	chr14	-	NM_0233	P10132	protein_cc	1810034K	magnesiu	Mdp1
6238	Nedd8	0.949	1.393	1.322	0.044	0.000	0.003	11.046	10.970	11.449	18002	Nedd8	chr14	-	NM_0086	P25353	protein_cc	Rub1	neural pre	Nedd8
6239	Gmpr2	0.825	1.138	0.939	0.429	0.664	0.871	6.276	6.000	6.185	105446	Gmpr2	chr14	+	NM_1779	P12438	protein_cc	1810008P	guanosine	Gmpr2
6240	Tinf2	1.777	1.300	2.310	0.010	0.034	0.001	5.961	6.791	7.169	28113	Tinf2	chr14	-	NM_1457	P11900	protein_cc	AW55211	Terf1 (TRF	Tinf2
6241	Tgm1	5.409	0.830	4.488	0.004	0.324	0.027	4.129	6.564	6.295	21816	Tgm1	chr14	-	NM_0011	P16243	protein_cc	2310004J	transgluta	Tgm1
6242	Rabggta	0.474	1.487	0.705	0.004	0.064	0.132	7.321	6.244	6.816	56187	Rabggta	chr14	-	NM_0195	P5643	protein_cc	gm	Rab geran	Rabggta
6243	Dhrs1	0.295	1.559	0.460	0.000	0.000	0.012	11.538	9.776	10.416	52585	Dhrs1	chr14	-	NM_0268	P1853	protein_cc	1110029G	dehydrog	Dhrs1
6244	Nop9	0.204	1.595	0.325	0.000	0.002	0.001	12.326	10.030	10.704	67842	Nop9	chr14	+	NM_0264	P21373	protein_cc	2610027L	NOP9 nuc	Nop9
6245	Cideb	0.175	1.652	0.289	0.000	0.005	0.000	12.278	9.764	10.489	12684	Cideb	chr14	-	NM_0098	P20160	protein_cc	1110030C	cell death	Cideb
6246	Ltb4r2	0.213	3.286	0.700	0.008	0.024	0.314	3.763	1.533	3.249	57260	Ltb4r2	chr14	+	NM_0204	P12254	protein_cc	5830462O	leukotrien	Ltb4r2
6247	Ltb4r1	6.315	1.444	9.120	0.107	0.340	0.007	2.309	4.968	5.498	16995	Ltb4r1	chr14	+	NM_0085	P19537	protein_cc	BLT1| BLT	leukotrien	Ltb4r1
6248	Adcy4	0.883	0.890	0.786	0.642	0.742	0.707	4.993	4.814	4.646	104110	Adcy4	chr14	-	NM_0804	P3148	protein_cc	mKIAA400	adenylate	Adcy4
6249	Ripk3	2.232	1.539	3.434	0.046	0.047	0.003	3.383	4.542	5.163	56532	Ripk3	chr14	-	NM_0011	P3089	protein_cc	2610528K	receptor-i	Ripk3
6250	Nfatc4	2.099	3.072	6.450	0.011	0.119	0.270	0.733	1.803	3.422	73181	Nfatc4	chr14	+	NM_0011	P11963	protein_cc	3110041H	nuclear fa	Nfatc4
6251	Nynrin	0.212	0.605	0.129	0.000	0.169	0.009	4.483	2.248	1.523	277154	Nynrin	chr14	+	NM_0010	P23303	protein_cc	AA409373	NYN doma	Nynrin
6252	Cbln3	0.174	0.957	0.167	0.113	1.000	0.337	2.584	0.063	0.000	56410	Cbln3	chr14	-	NM_0198	P12649	protein_cc	.	cerebellin	Cbln3
6253	Khnyn	0.720	0.774	0.557	0.026	0.197	0.075	5.495	5.020	4.651	219094	Khnyn	chr14	+	NM_0271	P22419	protein_cc	6330555F	KH and NY	Khnyn
6254	Sdr39u1	1.233	1.680	2.072	0.487	0.004	0.189	5.748	6.050	6.799	654795	Sdr39u1	chr14	-	NM_0010	P5880	protein_cc	2310014G	short chai	Sdr39u1
6255	Cma1	1.005	1.439	1.446	0.000	0.182	0.423	0.000	0.008	0.532	17228	Cma1	chr14	-	NM_0107	P11379	protein_cc	Mcp-5| M	chymase 1	Cma1
6256	Mcpt-ps1	1.000	1.000	1.000	1.000	1.000	1.000	0.000	0.000	0.000	17223	Mcpt-ps1	chr14	-	NR_02828	.	misc_RNA	MMCP-(Y)	mast cell p	Mcpt-ps1
6257	Cma2	1.005	1.439	1.446	0.000	0.182	0.423	0.000	0.008	0.532	545055	Cma2	chr14	+	NM_0010	P7652	protein_cc	Mcp10	chymase 2	Cma2
6258	Gm5801	2.078	2.388	4.962	0.001	0.054	0.153	2.522	3.577	4.833	545056	Gm5801	chr14	-	NR_00288	.	misc_RNA	EG545056	predicted	Gm5801
6259	Mcpt1	1.010	1.885	1.904	0.000	0.182	0.423	0.000	0.015	0.929	17224	Mcpt1	chr14	+	NM_0085	P1371	protein_cc	AV080368	mast cell p	Mcpt1
6260	Mcpt9	1.000	1.000	1.000	1.000	1.000	1.000	0.000	0.000	0.000	17232	Mcpt9	chr14	-	NM_0107	P5033	protein_cc	MMCP-1|	mast cell p	Mcpt9
6261	Mcpt2	1.015	2.326	2.360	0.000	0.182	0.423	0.000	0.021	1.239	17225	Mcpt2	chr14	+	NM_0085	P5767	protein_cc	Mcp-2| M	mast cell p	Mcpt2
6262	Mcpt4	1.000	1.000	1.000	1.000	1.000	1.000	0.000	0.000	0.000	17227	Mcpt4	chr14	-	NM_0107	P4230	protein_cc	Mcp-4| M	mast cell p	Mcpt4
6263	Mcpt8	3.936	0.804	3.166	0.305	0.799	0.423	0.001	1.978	1.664	17231	Mcpt8	chr14	-	NM_0085	P3135	protein_cc	BB139338	mast cell p	Mcpt8
6264	Ctsg	1.015	2.253	2.285	0.000	0.015	0.125	0.000	0.021	1.193	13035	Ctsg	chr14	-	NM_0078	P9402	protein_cc	CatG| VSF	cathepsin	Ctsg
6265	Gzme	1.000	1.000	1.000	1.000	1.000	1.000	0.000	0.000	0.000	14942	Gzme	chr14	-	NM_0103	P18205	protein_cc	CCP3| Ctl	granzyme	Gzme
6266	Gzmd	1.000	1.000	1.000	1.000	1.000	1.000	0.000	0.000	0.000	14941	Gzmd	chr14	-	NM_0103	P11063	protein_cc	CCP5| Ctl	granzyme	Gzmd

6267	Gzmg	1.000	1.000	1.000	1.000	1.000	1.000	0.000	0.000	0.000	14944	Gzmg	chr14	-	NM_0103	P4689	protein_cc	Ctla-7| Ctl	granzyme	Gzmg
6268	Gzmn	1.000	1.000	1.000	1.000	1.000	1.000	0.000	0.000	0.000	245839	Gzmn	chr14	-	NM_0013	P26394	protein_cc	GrN	granzyme	Gzmn
6269	Gzmf	1.000	1.000	1.000	1.000	1.000	1.000	0.000	0.000	0.000	14943	Gzmf	chr14	-	NM_0103	P5867	protein_cc	CCP4| Ctla	granzyme	Gzmf
6270	Gzmc	6.275	0.159	0.999	0.025	1.000	0.423	0.001	2.651	0.000	14940	Gzmc	chr14	-	NM_0103	P3586	protein_cc	AI323531|	granzyme	Gzmc
6271	Gzmb	7.140	0.217	1.547	0.002	0.003	0.355	2.201	5.037	2.830	14939	Gzmb	chr14	-	NM_0135	P13672	protein_cc	AI553453|	granzyme	Gzmb
6272	Atp12a	1.000	1.000	1.000	1.000	1.000	1.000	0.000	0.000	0.000	192113	Atp12a	chr14	+	NM_1386	P5264	protein_cc	Atp1al1| c	ATPase, H	Atp12a
6273	Rnf17	1.000	1.000	1.000	1.000	1.000	1.000	0.000	0.000	0.000	30054	Rnf17	chr14	+	NM_0010	P3878	protein_cc	MMIP-2| I	ring finger	Rnf17
6274	Cenpj	2.256	0.590	1.331	0.003	0.087	0.691	5.416	6.590	5.829	219103	Cenpj	chr14	-	NM_0010	P12250	protein_cc	4932437H	centromer	Cenpj
6275	Parp4	1.203	1.036	1.247	0.118	0.657	0.090	6.258	6.525	6.576	328417	Parp4	chr14	+	NM_0011	P24402	protein_cc	Adprtl1| C	poly (ADP-	Parp4
6276	Mphosph8	1.359	0.773	1.050	0.004	0.073	0.845	9.553	9.996	9.624	75339	Mphosph8	chr14	+	NM_0237	P4867	protein_cc	1500035L	M-phase p	Mphosph8
6277	Gm16973	1.139	1.078	1.228	0.294	0.554	0.453	6.492	6.679	6.788	10050382	Gm16973	chr14	-	NR_04620	.	lncRNA	.	predicted	Gm16973
6278	Pspc1	4.648	1.033	4.802	0.000	0.907	0.199	5.664	7.881	7.928	66645	Pspc1	chr14	-	NM_0256	P16046	protein_cc	5730470C	paraspeck	Pspc1
6279	Zmym5	0.775	1.106	0.856	0.015	0.363	0.411	9.180	8.811	8.956	219105	Zmym5	chr14	-	NM_0012	P21853	protein_cc	9830124H	zinc finger	Zmym5
6280	Zmym2	1.135	0.865	0.982	0.429	0.341	0.912	7.464	7.647	7.439	76007	Zmym2	chr14	+	NM_0294	P1404	protein_cc	5830413P	zinc finger	Zmym2
6281	B020004C	1.000	1.000	1.000	1.000	1.000	1.000	0.000	0.000	0.000	432860	B020004C	chr14	+	NM_0012	P24745	protein_cc	.	RIKEN cDN	B020004C
6282	Gja3	1.000	1.000	1.000	1.000	1.000	1.000	0.000	0.000	0.000	14611	Gja3	chr14	-	NM_0169	P26393	protein_cc	Cnx46| Cx	gap juncti	Gja3
6283	Gjb2	1.675	0.992	1.661	0.000	0.759	0.000	10.876	11.620	11.608	14619	Gjb2	chr14	-	NM_0081	P7473	protein_cc	AI325222|	gap juncti	Gjb2
6284	Gjb6	1.000	1.000	1.000	1.000	1.000	1.000	0.000	0.000	0.000	14623	Gjb6	chr14	-	NM_0012	P1580	protein_cc	AA958971	gap juncti	Gjb6
6285	Cryl1	0.157	1.871	0.293	0.000	0.003	0.013	6.469	3.794	4.698	68631	Cryl1	chr14	-	NM_0300	P11416	protein_cc	1110025H	crystallin,	Cryl1
6286	Ift88	0.640	1.977	1.264	0.245	0.058	0.305	4.901	4.256	5.239	21821	Ift88	chr14	+	NM_0093	P26640	protein_cc	AW55202	intraflagel	Ift88
6287	Il17d	1.606	1.393	2.237	0.297	0.527	0.423	0.000	0.684	1.162	239114	Il17d	chr14	+	NM_1458	P24755	protein_cc	AI462269|	interleukin	Il17d
6288	N6amt2	1.571	1.128	1.773	0.155	0.697	0.351	6.408	7.060	7.234	.	N6amt2	chr14	-	NM_0265	P13752	.			N6amt2
6289	Xpo4	7.221	0.614	4.434	0.014	0.168	0.102	1.093	3.945	3.241	57258	Xpo4	chr14	-	NM_0205	P26264	protein_cc	B430309A	exportin 4	Xpo4
6290	Lats2	0.943	1.431	1.350	0.636	0.023	0.011	7.210	7.126	7.643	50523	Lats2	chr14	-	NM_0157	P7810	protein_cc	4932411G	large tum	Lats2
6291	Sap18	2.162	1.021	2.208	0.300	0.972	0.423	0.001	1.113	1.143	20220	Sap18	chr14	+	NM_0091	P18651	protein_cc	C530046K	Sin3-assoc	Sap18
6292	Gm10094	1.206	0.976	1.177	0.019	0.626	0.010	8.943	9.213	9.178	.	Gm10094	chr14	+	NM_0011	P18651	.			Gm10094
6293	Ska3	0.614	1.119	0.687	0.487	0.780	0.765	2.495	1.793	1.955	219114	Ska3	chr14	-	NM_1986	P17488	protein_cc	F630043A	spindle an	Ska3
6294	Mrpl57	1.194	1.536	1.834	0.013	0.000	0.004	8.983	9.239	9.858	67840	Mrpl57	chr14	+	NM_0264	P3290	protein_cc	2610025P	mitochond	Mrpl57
6295	Zdhhc20	1.694	0.869	1.472	0.001	0.168	0.157	7.635	8.395	8.193	75965	Zdhhc20	chr14	-	NM_0294	P9013	protein_cc	4930542A	zinc finger	Zdhhc20
6296	Micu2	0.836	1.499	1.254	0.023	0.002	0.075	8.067	7.809	8.393	68514	Micu2	chr14	-	NM_0286	P22888	protein_cc	1110008L	mitochond	Micu2
6297	Fgf9	0.841	0.357	0.300	0.667	1.000	0.299	1.738	1.488	0.000	14180	Fgf9	chr14	+	NM_0135	P11918	protein_cc	Eks	fibroblast	Fgf9
6298	1700129C	1.000	1.000	1.000	1.000	1.000	1.000	0.000	0.000	0.000	67932	1700129C	chr14	-	NM_0264	P5046	protein_cc	.	RIKEN cDN	1700129C
6299	Gm5142	1.000	1.000	1.000	1.000	1.000	1.000	0.000	0.000	0.000	380907	Gm5142	chr14	-	NM_0010	P17548	protein_cc	EG380907	predicted	Gm5142
6300	Rcbtb1	0.358	1.240	0.444	0.001	0.158	0.043	9.158	7.675	7.985	71330	Rcbtb1	chr14	+	NM_0277	P13445	protein_cc	5430409I1	regulator	Rcbtb1
6301	Gm6904	6.302	0.331	2.089	0.001	0.002	0.078	0.001	2.657	1.064	628693	Gm6904	chr14	-	NM_0011	P4285	protein_cc	EG628693	predicted	Gm6904
6302	Phf11a	19.552	0.358	6.992	0.000	0.004	0.220	2.791	7.080	5.597	219131	Phf11a	chr14	-	NM_1726	P26056	protein_cc	4933417L	PHD finge	Phf11a
6303	Phf11b	11.420	0.331	3.781	0.000	0.000	0.027	4.836	8.350	6.755	236451	Phf11b	chr14	-	NM_0011	P7099	protein_cc	EG236451	PHD finge	Phf11b
6304	Phf11d	24.582	0.338	8.314	0.000	0.005	0.186	4.752	9.371	7.807	219132	Phf11d	chr14	-	NM_1990	P14049	protein_cc	D14Ertd6	PHD finge	Phf11d
6305	Phf11c	2.423	0.461	1.117	0.000	0.001	0.614	5.159	6.436	5.319	628705	Phf11c	chr14	-	NM_0011	P26494	protein_cc	EG628705	PHD finge	Phf11c
6306	Setdb2	0.729	0.952	0.694	0.000	0.849	0.394	4.886	4.431	4.359	219122	Setdb2	chr14	-	NM_0010	P24051	protein_cc	Clld8| Gm	SET doma	Setdb2
6307	Cab39l	0.435	1.204	0.524	0.000	0.404	0.105	9.089	7.889	8.157	69008	Cab39l	chr14	+	NM_0269	P4629	protein_cc	1500031K	calcium bi	Cab39l
6308	Cdadc1	0.517	1.347	0.697	0.008	0.085	0.108	8.743	7.792	8.222	71891	Cdadc1	chr14	-	NM_0011	P11682	protein_cc	2310010M	cytidine a	Cdadc1
6309	Shisa2	1.000	1.000	1.000	1.000	1.000	1.000	0.000	0.000	0.000	219134	Shisa2	chr14	+	NM_1454	P20996	protein_cc	9430059P	shisa fami	Shisa2
6310	Atp8a2	0.716	0.995	0.712	0.182	1.000	0.423	0.490	0.008	0.000	50769	Atp8a2	chr14	-	NM_0158	P8681	protein_cc	agil| AI41	ATPase, a	Atp8a2
6311	Nupl1	1.500	0.650	0.975	0.003	0.000	0.852	5.266	5.851	5.230	71844	Nupl1	chr14	-	NM_1705	P1139	protein_cc	1700017N	nucleopor	Nupl1
6312	Mtmr6	1.188	1.013	1.204	0.035	0.902	0.363	7.663	7.912	7.930	219135	Mtmr6	chr14	+	NM_1448	P1640	protein_cc	4022440C	myotubula	Mtmr6
6313	Amer2	0.295	0.479	0.142	0.000	0.049	0.003	3.285	1.525	0.465	72125	Amer2	chr14	+	NM_0011	P17127	protein_cc	2600011E	APC mem	Amer2
6314	Spata13	1.311	0.684	0.896	0.011	0.029	0.600	8.239	8.630	8.081	219140	Spata13	chr14	+	NM_0010	P25419	protein_cc	C79050| E	spermatog	Spata13
6315	C1qtnf9	0.186	2.056	0.383	0.035	0.193	0.320	2.495	0.069	1.109	239126	C1qtnf9	chr14	+	NM_1831	P11549	protein_cc	9130217G	C1q and tu	C1qtnf9
6316	Mipep	0.585	0.920	0.539	0.002	0.638	0.121	7.129	6.356	6.237	70478	Mipep	chr14	+	NM_0274	P18024	protein_cc	5730405E	mitochond	Mipep
6317	Tnfrsf19	0.021	2.936	0.063	0.012	0.011	0.125	6.364	0.812	2.365	29820	Tnfrsf19	chr14	-	NM_0011	P15270	protein_cc	AL023044	tumor nec	Tnfrsf19
6318	Sacs	0.744	1.362	1.013	0.560	0.547	0.988	1.119	0.692	1.138	50720	Sacs	chr14	+	NM_1728	P22358	protein_cc	A230052M	sacsin	Sacs
6319	Sgcg	1.000	1.000	1.000	1.000	1.000	1.000	0.000	0.000	0.000	24053	Sgcg	chr14	-	NM_0118	P12281	protein_cc	35kDa| 54	sarcoglyca	Sgcg
6320	1700109G	1.000	1.000	1.000	1.000	1.000	1.000	0.000	0.000	0.000	67355	1700109G	chr14	-	NR_03378	.	lncRNA	1700126G	RIKEN cDN	1700109G
6321	Arl11	1.171	1.137	1.332	0.157	0.555	0.483	2.710	2.938	3.123	219144	Arl11	chr14	+	NM_1773	P22170	protein_cc	ARLTS1| C	ADP-ribos	Arl11
6322	Ebpl	0.549	1.086	0.596	0.001	0.510	0.034	11.011	10.145	10.263	68177	Ebpl	chr14	-	NM_0265	P25852	protein_cc	5730442K	emopamil	Ebpl
6323	Kpna3	1.220	0.915	1.116	0.010	0.369	0.516	7.834	8.122	7.993	16648	Kpna3	chr14	-	NM_0084	P21536	protein_cc	IPOA4	karyopher	Kpna3
6324	4930556J	1.000	1.000	1.000	1.000	1.000	1.000	0.000	0.000	0.000	75349	4930556J	chr14	+	NR_04571	.	lncRNA		RIKEN cDN	4930556J0
6325	Spryd7	1.701	1.181	2.009	0.009	0.433	0.208	6.685	7.451	7.691	66674	Spryd7	chr14	-	NM_0256	P2366	protein_cc	6330409N	SPRY dom	Spryd7
6326	Trim13	12.811	0.776	9.945	0.000	0.322	0.165	2.154	5.833	5.468	66597	Trim13	chr14	+	NM_0011	P9620	protein_cc	3110001L	tripartite	Trim13
6327	Dleu2	0.390	0.421	0.165	0.002	0.047	0.001	8.007	6.650	5.403	668253	Dleu2	chr14	-	NR_02826	.	lncRNA	1810047A	deleted in	Dleu2
6328	Kcnrg	0.598	0.501	0.300	0.444	0.410	0.347	2.217	1.475	0.478	328424	Kcnrg	chr14	+	NM_0010	P16924	protein_cc	Clld4| E03	potassium	Kcnrg
6329	Dleu7	1.000	1.000	1.000	1.000	1.000	1.000	0.000	0.000	0.000	239133	Dleu7	chr14	-	NM_1734	P18486	protein_cc	BC038059	deleted in	Dleu7
6330	Rnaseh2b	0.855	2.319	1.982	0.181	0.000	0.006	6.225	5.999	7.212	67153	Rnaseh2b	chr14	+	NM_0260	P7934	protein_cc	1110019N	ribonuclea	Rnaseh2b
6331	Gucy1b2	1.000	1.000	1.000	1.000	1.000	1.000	0.000	0.000	0.000	239134	Gucy1b2	chr14	-	NM_0012	P2883	protein_cc	B930025B	guanylate	Gucy1b2
6332	Fam124a	0.538	1.333	0.717	0.002	0.036	0.128	6.878	5.984	6.399	629059	Fam124a	chr14	+	NM_0012	P17142	protein_cc	EG629059	family wit	Fam124a
6333	Serpine3	0.474	2.171	1.028	0.187	0.106	0.970	3.649	2.571	3.689	319433	Serpine3	chr14	+	NM_0011	P1214	protein_cc	E130113E	serpin pep	Serpine3
6334	Ints6	1.522	0.650	0.989	0.320	0.338	0.972	6.490	7.096	6.474	18130	Ints6	chr14	-	NM_0087	P21791	protein_cc	2900075H	integrator	Ints6
6335	4931440J1	1.000	1.000	1.000	1.000	1.000	1.000	0.000	0.000	0.000	71000	4931440J1	chr14	+	NR_04550	.	lncRNA		RIKEN cDN	4931440J1
6336	Wdfy2	1.161	0.924	1.073	0.855	0.932	0.943	2.302	2.517	2.403	268752	Wdfy2	chr14	+	NM_1755	P18228	protein_cc	5830485M	WD repeat	Wdfy2
6337	Defb48	1.000	1.000	1.000	1.000	1.000	1.000	0.000	0.000	0.000	432867	Defb48	chr14	-	NM_0010	P5212	protein_cc	Defb42| E	defensin b	Defb48
6338	Defb47	1.000	1.000	1.000	1.000	1.000	1.000	0.000	0.000	0.000	654465	Defb47	chr14	-	NM_0010	P25409	protein_cc	Defb41| E	defensin b	Defb47
6339	Defb43	1.000	1.000	1.000	1.000	1.000	1.000	0.000	0.000	0.000	654458	Defb43	chr14	+	NM_0010	P19250	protein_cc	BD-43| EG	defensin b	Defb43
6340	Defb30	1.000	1.000	1.000	1.000	1.000	1.000	0.000	0.000	0.000	73670	Defb30	chr14	-	NM_0010	P23601	protein_cc	2410125J	defensin b	Defb30
6341	Defb42	1.000	1.000	1.000	1.000	1.000	1.000	0.000	0.000	0.000	619548	Defb42	chr14	+	NM_0010	P19191	protein_cc	BD-42| De	defensin b	Defb42
6342	2700070H	0.726	1.387	1.007	0.187	0.187	0.988	0.477	0.015	0.487	72609	2700070H	chr14	+	NR_04601	.	lncRNA	.	RIKEN cDN	2700070H
6343	Ctsb	0.760	0.723	0.549	0.137	0.131	0.057	11.975	11.580	11.111	13030	Ctsb	chr14	+	NM_0077	P15246	protein_cc	CB	cathepsin	Ctsb
6344	Fdft1	0.723	1.697	1.227	0.026	0.012	0.247	9.773	9.306	10.068	14137	Fdft1	chr14	-	NM_0101	P4208	protein_cc	SQS| SS	farnesyl d	Fdft1
6345	Neil2	0.338	0.737	0.249	0.291	0.280	0.485	3.092	1.528	1.087	382913	Neil2	chr14	-	NM_2016	P25716	protein_cc	Gm1212|	nei like 2 (	Neil2
6346	Gata4	0.720	0.651	0.468	0.018	0.007	0.027	6.549	6.074	5.455	14463	Gata4	chr14	-	NM_0080	P10822	protein_cc	Gata-4	GATA bind	Gata4
6347	Blk	0.699	0.626	0.438	0.260	1.000	0.044	1.192	0.675	0.000	12143	Blk	chr14	-	NM_0075	P2481	protein_cc	.	B lymphoi	Blk
6348	Fam167a	1.551	0.358	0.555	0.626	0.368	0.548	1.327	1.961	0.479	219148	Fam167a	chr14	+	NM_1776	P10983	protein_cc	A030013D	family wit	Fam167a
6349	Tdh	1.000	1.000	1.000	1.000	1.000	1.000	0.000	0.000	0.000	58865	Tdh	chr14	-	NM_0214	P18821	protein_cc	.	L-threonin	Tdh
6350	Mtmr9	1.890	1.011	1.910	0.112	0.959	0.174	4.718	5.637	5.652	210376	Mtmr9	chr14	-	NM_1775	P16350	protein_cc	9430075G	myotubula	Mtmr9
6351	Xkr6	0.732	0.617	0.452	0.636	0.455	0.502	1.578	1.127	0.432	219149	Xkr6	chr14	+	NM_1733	P7548	protein_cc	2700008G	X-linked K	Xkr6
6352	Pinx1	1.717	1.009	1.733	0.031	0.957	0.106	7.035	7.816	7.829	72400	Pinx1	chr14	+	NM_0282	P17187	protein_cc	2210403I1	PIN2/TERF	Pinx1
6353	Sox7	0.762	0.486	0.370	0.128	0.003	0.079	4.693	4.300	3.259	20680	Sox7	chr14	+	NM_0114	P10399	protein_cc	.	SRY (sex d	Sox7
6354	4930578I0	1.000	1.000	1.000	1.000	1.000	1.000	0.000	0.000	0.000	67750	4930578I0	chr14	-	NM_0263	P13061	protein_cc	AI429111	RIKEN cDN	4930578I0
6355	Rp1l1	1.000	1.000	1.000	1.000	1.000	1.000	0.000	0.000	0.000	271209	Rp1l1	chr14	+	NM_1462	P8740	protein_cc	BC031365	retinitis pi	Rp1l1
6356	Prss55	1.000	1.000	1.000	1.000	1.000	1.000	0.000	0.000	0.000	71037	Prss55	chr14	-	NM_0010	P935	protein_cc	4933401F	protease,	Prss55
6357	Prss51	1.000	1.000	1.000	1.000	1.000	1.000	0.000	0.000	0.000	10050416	Prss51	chr14	+	NM_0011	P10385	protein_cc	1700007N	protease,	Prss51

6358	4930471C	1.000	1.000	1.000	1.000	1.000	1.000	0.000	0.000	0.000	78176	4930471C	chr14	-	NR_04619	.	lncRNA	.	RIKEN cDN	4930471C
6359	Prss52	1.000	1.000	1.000	1.000	1.000	1.000	0.000	0.000	0.000	73382	Prss52	chr14	+	NM_0285	P11299	protein_cc	1700049K	protease,	Prss52
6360	Msra	0.391	1.560	0.611	0.000	0.066	0.114	10.474	9.121	9.762	110265	Msra	chr14	-	NM_0012	P26744	protein_cc	2310045J	methionin	Msra
6361	Kif13b	0.264	1.362	0.360	0.002	0.388	0.112	5.086	3.165	3.610	16554	Kif13b	chr14	+	NM_0010	P16782	protein_cc	5330429L	kinesin fa	Kif13b
6362	Hmbox1	3.684	1.308	4.820	0.122	0.525	0.166	1.946	3.827	4.215	219150	Hmbox1	chr14	-	NM_1773	P1859	protein_cc	AI451877 |	homeobox	Hmbox1
6363	Ints9	1.125	0.825	0.928	0.538	0.374	0.699	6.642	6.811	6.533	210925	Ints9	chr14	+	NM_1534	P20255	protein_cc	BC028953	integrator	Ints9
6364	Extl3	2.078	0.715	1.487	0.002	0.026	0.095	7.462	8.517	8.034	54616	Extl3	chr14	-	NM_0187	P5207	protein_cc	2900009G	exostoses	Extl3
6365	Fzd3	0.623	1.720	1.071	0.309	0.274	0.925	2.476	1.793	2.575	14365	Fzd3	chr14	-	NM_0214	P22169	protein_cc	AU020229	frizzled cla	Fzd3
6366	Fbxo16	1.000	1.000	1.000	1.000	1.000	1.000	0.000	0.000	0.000	50759	Fbxo16	chr14	+	NM_0157	P17638	protein_cc	4932435C	F-box prot	Fbxo16
6367	Zfp395	0.031	3.939	0.122	0.000	0.031	0.008	7.871	2.860	4.838	380912	Zfp395	chr14	+	NM_1990	P16175	protein_cc	BC053701	zinc finger	Zfp395
6368	Pnoc	0.345	3.002	1.036	0.184	0.074	0.969	1.588	0.054	1.640	18155	Pnoc	chr14	-	NM_0109	P5760	protein_cc	N/OFQ | N	preprono	Pnoc
6369	Elp3	1.059	1.183	1.252	0.576	0.073	0.247	7.985	8.067	8.310	74195	Elp3	chr14	-	NM_0288	P17079	protein_cc	2610507P	elongator	Elp3
6370	Nuggc	0.553	2.718	1.502	0.001	0.183	0.656	0.894	0.039	1.481	10050354	Nuggc	chr14	+	NM_0011	P17522	protein_cc	Gm600 | S	nuclear G	Nuggc
6371	Scara5	33.042	2.908	96.098	0.000	0.000	0.000	1.005	6.051	7.592	71145	Scara5	chr14	+	NM_0289	P21979	protein_cc	4932433F	scavenger	Scara5
6372	Pbk	4.439	0.413	1.832	0.305	0.430	0.423	0.001	2.151	0.875	52033	Pbk	chr14	+	NM_0232	P19662	protein_cc	2810434B	PDZ bindir	Pbk
6373	Esco2	0.511	2.101	1.074	0.108	0.143	0.852	2.132	1.163	2.235	71988	Esco2	chr14	-	NM_0280	P6328	protein_cc	2410004I1	establishn	Esco2
6374	Ccdc25	1.906	1.139	2.172	0.000	0.394	0.125	9.778	10.708	10.896	67179	Ccdc25	chr14	+	NM_1459	P17398	protein_cc	2610528H	coiled-coil	Ccdc25
6375	Scara3	0.109	2.257	0.245	0.005	0.135	0.093	4.745	1.544	2.719	219151	Scara3	chr14	-	NM_1726	P12723	protein_cc	APC7 | C1	scavenger	Scara3
6376	Clu	0.728	1.237	0.900	0.000	0.028	0.359	13.859	13.400	13.707	12759	Clu	chr14	+	NM_0134	P25478	protein_cc	AI893575 |	clusterin	Clu
6377	Gulo	0.159	0.983	0.156	0.000	0.924	0.013	12.957	10.301	10.276	268756	Gulo	chr14	-	NM_1787	P7351	protein_cc	5730581M	gulonolact	Gulo
6378	Adam2	1.000	1.000	1.000	1.000	1.000	1.000	0.000	0.000	0.000	11495	Adam2	chr14	-	NM_0096	P25656	protein_cc	AI323749 |	a disinteg	Adam2
6379	Ephx2	0.292	1.438	0.420	0.000	0.035	0.013	12.697	10.922	11.445	13850	Ephx2	chr14	-	NM_0012	P14392	protein_cc	CEH | Eph2	epoxide h	Ephx2
6380	Chrna2	0.192	6.353	1.218	0.078	0.019	0.785	3.166	0.784	3.451	110902	Chrna2	chr14	+	NM_1448	P24076	protein_cc	Acra-2 | Ac	cholinergi	Chrna2
6381	Ptk2b	0.346	1.295	0.449	0.000	0.006	0.016	8.331	6.801	7.174	19229	Ptk2b	chr14	-	NM_0011	P11958	protein_cc	CADTK | C	PTK2 prot	Ptk2b
6382	1700001G	0.552	0.990	0.547	0.182	1.000	0.423	0.871	0.015	0.000	69303	1700001G	chr14	-	NR_03807	.	lncRNA	.	RIKEN cDN	1700001G
6383	Trim35	1.711	0.803	1.374	0.010	0.116	0.384	6.102	6.877	6.561	66854	Trim35	chr14	+	NM_0299	P18365	protein_cc	0710005M	tripartite	Trim35
6384	Stmn4	1.572	0.636	1.000	0.304	1.000	0.423	0.000	0.653	0.000	56471	Stmn4	chr14	+	NM_0196	P14261	protein_cc	Lagl | R75	stathmin-	Stmn4
6385	Adra1a	0.059	4.847	0.287	0.007	0.018	0.156	5.323	1.246	3.523	11549	Adra1a	chr14	+	NM_0134	P21440	protein_cc	Adra1c	adrenergi	Adra1a
6386	Dpysl2	0.150	1.863	0.279	0.001	0.032	0.041	6.258	3.519	4.417	12934	Dpysl2	chr14	-	NM_0099	P5271	protein_cc	AI851130 |	dihydropy	Dpysl2
6387	Gm5464	1.010	1.825	1.844	0.000	0.182	0.423	0.000	0.015	0.883	432870	Gm5464	chr14	+	NM_0010	P26392	protein_cc	EG432870	predicted	Gm5464
6388	Pnma2	1.000	1.000	1.000	1.000	1.000	1.000	0.000	0.000	0.000	239157	Pnma2	chr14	+	NM_1754	P12165	protein_cc	A830049P	paraneopl	Pnma2
6389	Bnip3l	0.880	1.276	1.124	0.231	0.033	0.187	9.889	9.706	10.058	12177	Bnip3l	chr14	-	NM_0097	P1113	protein_cc	C86132 | D	BCL2/ade	Bnip3l
6390	Ppp2r2a	1.624	0.630	1.023	0.000	0.002	0.893	5.195	5.895	5.227	71978	Ppp2r2a	chr14	-	NM_0012	P20427	protein_cc	2410004D	protein ph	Ppp2r2a
6391	Ebf2	1.000	1.000	1.000	1.000	1.000	1.000	0.000	0.000	0.000	13592	Ebf2	chr14	+	NM_0012	P13855	protein_cc	D14Ggc1e	early B cel	Ebf2
6392	Gm6878	1.000	1.000	1.000	1.000	1.000	1.000	0.000	0.000	0.000	628416	Gm6878	chr14	-	NM_0010	P24475	protein_cc	EG628416	predicted	Gm6878
6393	4930438E	1.000	1.000	1.000	1.000	1.000	1.000	0.000	0.000	0.000	73977	4930438E	chr14	-	NR_04596	.	lncRNA	.	RIKEN cDN	4930438E
6394	Cdca2	1.305	1.103	1.440	0.717	0.844	0.623	2.005	2.389	2.531	108912	Cdca2	chr14	-	NM_1753	P20992	protein_cc	2610311M	cell divisio	Cdca2
6395	Kctd9	1.969	1.895	3.732	0.004	0.074	0.149	5.346	6.323	7.245	105440	Kctd9	chr14	+	NM_1340	P15915	protein_cc	.	potassium	Kctd9
6396	Gnrh1	1.338	1.797	2.404	0.689	0.483	0.554	1.063	1.483	2.329	14714	Gnrh1	chr14	+	NM_0081	P23462	protein_cc	Gnrh | Gnr	gonadotro	Gnrh1
6397	Dock5	1.094	1.060	1.160	0.013	0.657	0.549	6.278	6.408	6.493	68813	Dock5	chr14	-	NM_1777	P10845	protein_cc	1110060D	dedicator	Dock5
6398	Nefl	1.030	3.852	3.968	0.000	0.000	0.012	0.001	0.043	1.989	18039	Nefl	chr14	+	NM_0109	P15095	protein_cc	AI847934 |	neurofilan	Nefl
6399	Nefm	1.026	3.449	3.539	0.000	0.181	0.423	0.001	0.038	1.824	18040	Nefm	chr14	-	NM_0086	P4280	protein_cc	Nef3 | NF-	neurofilan	Nefm
6400	A230070E	1.000	1.000	1.000	1.000	1.000	1.000	0.000	0.000	0.000	10004307	A230070E	chr14	+	NR_04589	.	lncRNA	10004307	RIKEN cDN	A230070E
6401	Adam7	1.000	1.000	1.000	1.000	1.000	1.000	0.000	0.000	0.000	11500	Adam7	chr14	-	NM_0074	P17002	protein_cc	ADAM 7 |	a disinteg	Adam7
6402	Adamdec1	1.372	1.950	2.675	0.436	0.038	0.207	3.500	3.956	4.920	58860	Adamdec1	chr14	-	NM_0214	P23046	protein_cc	2210414L	ADAM-like	Adamdec1
6403	Adam28	2.022	1.462	2.956	0.178	0.414	0.310	0.757	1.772	2.321	13522	Adam28	chr14	-	NR_10239	.	protein_cc	C130072N	a disinteg	Adam28
6404	Stc1	18.038	0.334	6.022	0.003	0.016	0.234	4.116	8.289	6.706	20855	Stc1	chr14	+	NM_0092	P22489	protein_cc	Stc	stanniocal	Stc1
6405	1700092C	0.461	0.839	0.387	0.307	0.658	0.485	1.811	0.694	0.440	73554	1700092C	chr14	-	NR_04593	.	lncRNA	.	RIKEN cDN	1700092C
6406	Nkx2-6	0.669	0.995	0.666	0.181	1.000	0.423	0.587	0.008	0.000	18092	Nkx2-6	chr14	+	NM_0109	P1728	protein_cc	AV044928	NK2 home	Nkx2-6
6407	Nkx3-1	1.000	1.000	1.000	1.000	1.000	1.000	0.000	0.000	0.000	18095	Nkx3-1	chr14	+	NM_0109	P18628	protein_cc	bagpipe |	NK-3 trans	Nkx3-1
6408	Slc25a37	0.991	0.733	0.727	0.951	0.037	0.242	8.401	8.389	7.940	67712	Slc25a37	chr14	-	NM_0263	P12258	protein_cc	1700020E	solute car	Slc25a37
6409	Synb	1.000	1.000	1.000	1.000	1.000	1.000	0.000	0.000	0.000	239167	Synb	chr14	-	NM_1734	P20897	protein_cc	D930020E	syncytin b	Synb
6410	Gm16677	1.000	1.000	1.000	1.000	1.000	1.000	0.000	0.000	0.000	10050447	Gm16677	chr14	-	NR_04509	.	lncRNA	.	predicted	Gm16677
6411	Entpd4	0.805	1.213	0.976	0.035	0.082	0.893	7.397	7.084	7.363	67464	Entpd4	chr14	+	NM_0261	P23682	protein_cc	1200014F	ectonucle	Entpd4
6412	Loxl2	1.727	1.267	2.189	0.017	0.429	0.298	5.579	6.368	6.709	94352	Loxl2	chr14	+	NM_0333	P19801	protein_cc	1110004B	lysyl oxida	Loxl2
6413	R3hcc1	0.631	1.645	1.038	0.019	0.001	0.817	6.830	6.165	6.884	71843	R3hcc1	chr14	+	NM_0013	P17124	protein_cc	1700020M	R3H doma	R3hcc1
6414	Chmp7	1.073	0.807	0.866	0.865	0.627	0.599	7.437	7.539	7.229	105513	Chmp7	chr14	-	NM_1340	P3024	protein_cc	4930596K	charged m	Chmp7
6415	4930480K	0.130	1.185	0.154	0.013	0.753	0.134	4.504	1.562	1.808	75016	4930480K	chr14	+	NR_13016	.	lncRNA	.	RIKEN cDN	4930480K
6416	Tnfrsf10b	17.900	0.427	7.645	0.001	0.008	0.081	2.828	6.990	5.763	21933	Tnfrsf10b	chr14	+	NM_0202	P25763	protein_cc	DR5 | KILL	tumor nec	Tnfrsf10b
6417	Rhobtb2	0.454	1.370	0.622	0.001	0.066	0.018	6.163	5.024	5.478	246710	Rhobtb2	chr14	-	NM_1535	P14835	protein_cc	Dbc2 | E13	Rho-relate	Rhobtb2
6418	Pebp4	1.000	1.000	1.000	1.000	1.000	1.000	0.000	0.000	0.000	73523	Pebp4	chr14	+	NM_0285	P18892	protein_cc	PEBP-4	phosphati	Pebp4
6419	Egr3	1.605	0.629	1.010	0.224	0.097	0.990	1.316	1.998	1.330	13655	Egr3	chr14	+	NM_0187	P9971	protein_cc	Pilot	early grow	Egr3
6420	Bin3	1.457	1.350	1.967	0.000	0.006	0.026	6.468	7.011	7.443	57784	Bin3	chr14	+	NM_0213	P16974	protein_cc	1700015F	bridging in	Bin3
6421	Ccar2	0.977	0.837	0.818	0.952	0.677	0.748	4.467	4.433	4.176	219158	Ccar2	chr14	-	NM_1460	P12717	protein_cc	2610301G	cell cycle a	Ccar2
6422	9930012K	1.025	1.205	1.235	0.967	0.716	0.767	1.097	1.132	1.401	268759	9930012K	chr14	-	NM_0010	P10844	protein_cc	.	RIKEN cDN	9930012K
6423	Pdlim2	0.614	1.170	0.718	0.070	0.472	0.439	5.615	4.911	5.137	213019	Pdlim2	chr14	-	NM_0012	P26628	protein_cc	4732462F	PDZ and L	Pdlim2
6424	Sorbs3	0.067	3.694	0.231	0.000	0.002	0.028	6.431	2.431	4.316	20410	Sorbs3	chr14	-	NM_0012	P9213	protein_cc	SCAM-1 |	sorbin and	Sorbs3
6425	Ppp3cc	0.475	0.685	0.325	0.120	0.625	0.188	4.117	3.042	2.495	19057	Ppp3cc	chr14	-	NM_0013	P8423	protein_cc	Calnc | PP	protein ph	Ppp3cc
6426	Slc39a14	5.267	0.677	3.567	0.000	0.000	0.003	8.110	10.507	9.944	213053	Slc39a14	chr14	-	NM_0011	P2121	protein_cc	FAD-123 |	solute car	Slc39a14
6427	Piwil2	6.284	1.785	11.216	0.091	0.105	0.002	0.995	3.647	4.483	57746	Piwil2	chr14	-	NM_0213	P18945	protein_cc	mili | Piwil	piwi-like R	Piwil2
6428	Polr3d	4.524	0.556	2.514	0.001	0.005	0.015	6.619	8.797	7.949	67065	Polr3d	chr14	-	NM_0011	P26429	protein_cc	2810426M	polymeras	Polr3d
6429	Phyhip	1.000	1.000	1.000	1.000	1.000	1.000	0.000	0.000	0.000	105653	Phyhip	chr14	+	NM_1459	P24658	protein_cc	AW04987	phytanoyl	Phyhip
6430	Bmp1	0.230	1.711	0.393	0.001	0.056	0.061	8.738	6.616	7.391	12153	Bmp1	chr14	-	NR_03324	.	protein_cc	Pcp | Tld	bone mor	Bmp1
6431	Sftpc	1.010	1.885	1.904	0.000	0.182	0.423	0.000	0.015	0.929	20389	Sftpc	chr14	-	NM_0113	P13971	protein_cc	Bricd6 | pr	surfactant	Sftpc
6432	Lgi3	0.292	0.977	0.285	0.181	1.000	0.423	1.810	0.034	0.000	213469	Lgi3	chr14	+	NM_1452	P23702	protein_cc	AI841179 |	leucine-ric	Lgi3
6433	Reep4	0.350	2.646	0.926	0.001	0.000	0.478	6.992	5.477	6.881	72549	Reep4	chr14	+	NM_1805	P19620	protein_cc	2700029E	receptor a	Reep4
6434	Hr	0.326	2.240	0.729	0.000	0.187	0.583	1.667	0.048	1.212	15460	Hr	chr14	+	NM_0218	P20801	protein_cc	ALUNC | A	hairless	Hr
6435	Nudt18	1.152	1.148	1.322	0.089	0.276	0.294	6.499	6.703	6.902	213484	Nudt18	chr14	+	NM_1531	P2686	protein_cc	BC036718	nudix (nuc	Nudt18
6436	Fam160b2	0.914	0.853	0.780	0.052	0.055	0.138	6.930	6.801	6.572	239170	Fam160b2	chr14	-	NM_1943	P11290	protein_cc	G430067P	family wit	Fam160b2
6437	Dmtn	0.678	0.796	0.540	0.398	0.676	0.197	1.694	1.133	0.805	13829	Dmtn	chr14	-	NM_0012	P2518	protein_cc	AI325486 |	dematin a	Dmtn
6438	Fgf17	1.000	1.000	1.000	1.000	1.000	1.000	0.000	0.000	0.000	14171	Fgf17	chr14	-	NM_0080	P18167	protein_cc	.	fibroblast	Fgf17
6439	Npm2	1.445	0.630	0.910	0.554	0.473	0.848	0.587	1.119	0.451	328440	Npm2	chr14	-	NM_1813	P1553	protein_cc	.	nucleopho	Npm2
6440	Xpo7	0.605	1.217	0.736	0.000	0.112	0.124	8.877	8.151	8.435	65246	Xpo7	chr14	-	NM_0230	P868	protein_cc	4930506C	exportin 7	Xpo7
6441	Dok2	1.639	0.848	1.389	0.205	0.647	0.598	3.709	4.422	4.183	13449	Dok2	chr14	+	NM_0100	P21410	protein_cc	DokR | Frip	docking pr	Dok2
6442	2410012E	1.000	1.000	1.000	1.000	1.000	1.000	0.000	0.000	0.000	73648	2410012E	chr14	+	NR_04593	.	lncRNA	.	RIKEN cDN	2410012E
6443	Gfra2	0.269	1.437	0.386	0.002	0.516	0.196	3.096	1.201	1.724	14586	Gfra2	chr14	+	NM_0081	P25264	protein_cc	.	glial cell lin	Gfra2
6444	Gm4251	1.000	1.000	1.000	1.000	1.000	1.000	0.000	0.000	0.000	10004313	Gm4251	chr14	+	NR_04607	.	lncRNA	10004313	predicted	Gm4251
6445	4930434J	1.000	1.000	1.000	1.000	1.000	1.000	0.000	0.000	0.000	74667	4930434J	chr14	+	NR_04627	.	lncRNA	.	RIKEN cDN	4930434J
6446	Fndc3a	1.127	0.663	0.747	0.438	0.065	0.478	8.213	8.386	7.792	319448	Fndc3a	chr14	-	NM_2076	P16012	protein_cc	1700094E	fibronecti	Fndc3a
6447	Gm9199	0.480	1.247	0.598	0.012	0.197	0.194	3.464	2.405	2.723	668489	Gm9199	chr14	+	NR_02786	.	misc_RNA	EG668489	predicted	Gm9199
6448	Cysltr2	6.860	0.201	1.382	0.003	0.005	0.424	0.001	2.780	0.468	70086	Cysltr2	chr14	-	NM_1337	P26287	protein_cc	2300001H	cysteinyl	Cysltr2

6449	Rcbtb2	1.034	0.769	0.796	0.582	0.036	0.298	7.484	7.533	7.154	105670	Rcbtb2	chr14	+	NM_1340	P17707	protein_cc	2610028E	regulator	Rcbtb2
6450	Rb1	1.444	0.741	1.070	0.083	0.079	0.857	6.141	6.670	6.238	19645	Rb1	chr14	-	NM_0090	P25535	protein_cc	pRb| Rb|	retinoblas	Rb1
6451	Lpar6	2.187	0.448	0.979	0.001	0.000	0.888	8.197	9.326	8.166	67168	Lpar6	chr14	+	NM_1751	P6546	protein_cc	2610302I(	lysophosp	Lpar6
6452	Itm2b	0.435	1.133	0.493	0.001	0.175	0.041	13.209	12.010	12.190	16432	Itm2b	chr14	-	NM_0084	P25532	protein_cc	AI256040|	integral m	Itm2b
6453	Med4	1.251	1.166	1.459	0.420	0.537	0.217	7.381	7.705	7.926	67381	Med4	chr14	+	NM_0261	P7068	protein_cc	2410046H	mediator	Med4
6454	Nudt15	0.442	2.066	0.913	0.301	0.030	0.916	1.909	0.730	1.777	214254	Nudt15	chr14	-	NM_1725	P21639	protein_cc	65304030	nudix (nuc	Nudt15
6455	Sucla2	0.562	1.105	0.621	0.000	0.143	0.016	10.650	9.819	9.962	20916	Sucla2	chr14	+	NM_0115	P19583	protein_cc	4930547K	succinate-	Sucla2
6456	4933402J1	1.000	1.000	1.000	1.000	1.000	1.000	0.000	0.000	0.000	74425	4933402J1	chr14	+	NR_04605	.	lncRNA	.	RIKEN cDN	4933402J1
6457	Htr2a	1.000	1.000	1.000	1.000	1.000	1.000	0.000	0.000	0.000	15558	Htr2a	chr14	+	NM_1728	P23141	protein_cc	E030013E	5-hydroxy	Htr2a
6458	Esd	0.542	1.175	0.637	0.006	0.220	0.067	11.587	10.703	10.936	13885	Esd	chr14	+	NM_0169	P19759	protein_cc	Es-10| Es1	esterase D	Esd
6459	Lrch1	1.884	0.594	1.119	0.000	0.002	0.487	5.863	6.776	6.024	380916	Lrch1	chr14	-	NM_0010	P26115	protein_cc	4832412D	leucine-ric	Lrch1
6460	Gm4278	1.000	1.000	1.000	1.000	1.000	1.000	0.000	0.000	0.000	10004316	Gm4278	chr14	+	NR_04607	.	lncRNA	10004316	predicted	Gm4278
6461	5031414D	5.642	0.640	3.611	0.042	0.287	0.094	1.324	3.820	3.176	271221	5031414D	chr14	+	NM_1986	P25253	protein_cc	Gm744| G	RIKEN cDN	5031414D
6462	Lrrc63	9.284	0.226	2.100	0.176	0.228	0.158	0.002	3.216	1.072	70859	Lrrc63	chr14	-	NM_0275	P21550	protein_cc	4921509B	leucine ric	Lrrc63
6463	Lcp1	1.297	0.741	0.961	0.061	0.029	0.748	9.411	9.785	9.354	18826	Lcp1	chr14	+	NM_0012	P18203	protein_cc	AW53623	lymphocyt	Lcp1
6464	Cpb2	2.944	0.848	2.497	0.046	0.543	0.018	11.811	13.369	13.132	56373	Cpb2	chr14	+	NM_0197	P4701	protein_cc	1110032P	carboxype	Cpb2
6465	Zc3h13	2.264	0.583	1.320	0.004	0.026	0.442	8.441	9.620	8.842	67302	Zc3h13	chr14	+	NM_0260	P237	protein_cc	2600010B	zinc finger	Zc3h13
6466	Siah3	1.000	1.000	1.000	1.000	1.000	1.000	0.000	0.000	0.000	380918	Siah3	chr14	+	NM_0011	P9439	protein_cc	Gm912	seven in a	Siah3
6467	Spert	1.000	1.000	1.000	1.000	1.000	1.000	0.000	0.000	0.000	67926	Spert	chr14	-	NM_0011	P6802	protein_cc	1700086N	spermatid	Spert
6468	4930564B	1.000	1.000	1.000	1.000	1.000	1.000	0.000	0.000	0.000	.	4930564B	chr14	+	NM_0292	P18799	.	.	.	4930564B
6469	Cog3	1.563	1.519	2.375	0.378	0.523	0.496	5.287	5.932	6.535	338337	Cog3	chr14	-	NM_1773	P21238	protein_cc	E430004N	componer	Cog3
6470	Slc25a30	0.726	1.425	1.034	0.187	0.186	0.943	0.476	0.015	0.525	67554	Slc25a30	chr14	-	NM_0262	P12679	protein_cc	4933433D	solute car	Slc25a30
6471	Gm4285	0.499	2.063	1.030	0.002	0.001	0.648	7.029	6.027	7.071	10004318	Gm4285	chr14	-	NR_04529	.	lncRNA	10004318	predicted	Gm4285
6472	Tpt1	1.130	0.974	1.100	0.002	0.136	0.061	13.231	13.407	13.369	22070	Tpt1	chr14	+	NM_0094	P17514	protein_cc	p21| p23|	tumor pro	Tpt1
6473	Snora31	2.734	0.498	1.360	0.305	0.409	0.423	0.001	1.452	0.445	10030375	Snora31	chr14	+	NR_02848	.	snoRNA	MBI-161	small nucl	Snora31
6474	Gtf2f2	2.722	0.882	2.401	0.000	0.415	0.138	6.724	8.169	7.988	68705	Gtf2f2	chr14	-	NM_0268	P13150	protein_cc	1110031C	general tra	Gtf2f2
6475	Kctd4	1.024	0.444	0.454	0.955	0.208	0.188	2.997	3.031	1.859	67516	Kctd4	chr14	+	NM_0262	P11032	protein_cc	2210017A	potassium	Kctd4
6476	Gpalpp1	1.572	1.226	1.927	0.012	0.339	0.200	6.768	7.421	7.715	67467	Gpalpp1	chr14	-	NM_0261	P3503	protein_cc	1200011I1	GPALPP m	Gpalpp1
6477	Nufip1	1.691	1.267	2.143	0.002	0.466	0.344	6.338	7.096	7.438	27275	Nufip1	chr14	+	NM_0137	P9239	protein_cc	Nufip	nuclear fra	Nufip1
6478	Tsc22d1	0.751	0.647	0.486	0.116	0.104	0.024	8.588	8.175	7.545	21807	Tsc22d1	chr14	+	NM_2076	P20917	protein_cc	AA589566	TSC22 dor	Tsc22d1
6479	4930444M	1.000	1.000	1.000	1.000	1.000	1.000	0.000	0.000	0.000	75789	4930444M	chr14	-	NR_04566	.	lncRNA	.	RIKEN cDN	4930444M
6480	Serp2	0.341	1.390	0.473	0.182	0.198	0.521	1.588	0.034	0.508	72661	Serp2	chr14	-	NM_0011	P721	protein_cc	2810032E	stress-ass	Serp2
6481	1700108F	1.010	1.885	1.904	0.000	0.182	0.423	0.000	0.015	0.929	73272	1700108F	chr14	+	NR_01548	.	lncRNA	1700020I(	RIKEN cDN	1700108F1
6482	Lacc1	3.541	0.705	2.498	0.004	0.066	0.077	4.834	6.658	6.154	210808	Lacc1	chr14	-	NM_1724	P17819	protein_cc	9030625A	laccase (m	Lacc1
6483	Ccdc122	1.864	1.984	3.698	0.002	0.005	0.033	5.980	6.878	7.867	108811	Ccdc122	chr14	+	NM_1753	P11390	protein_cc	4933415L(	coiled-coil	Ccdc122
6484	Enox1	1.000	1.000	1.000	1.000	1.000	1.000	0.000	0.000	0.000	239188	Enox1	chr14	+	NM_1728	P18427	protein_cc	B230207J(	ecto-NOX	Enox1
6485	Gm6994	1.572	0.636	1.000	0.304	1.000	0.423	0.000	0.653	0.000	629678	Gm6994	chr14	-	NR_03314	.	lncRNA	EG629678	predicted	Gm6994
6486	Gm1587	1.000	1.000	1.000	1.000	1.000	1.000	0.000	0.000	0.000	380920	Gm1587	chr14	-	NM_0010	P22965	protein_cc	.	predicted	Gm1587
6487	Dnajc15	0.882	1.422	1.253	0.204	0.008	0.022	8.069	7.888	8.395	66148	Dnajc15	chr14	-	NM_0253	P26753	protein_cc	1110003P	DnaJ heat	Dnajc15
6488	Epsti1	1.762	0.405	0.714	0.003	0.001	0.147	5.988	6.806	5.503	108670	Epsti1	chr14	+	NM_0294	P13772	protein_cc	2310046K	epithelial	Epsti1
6489	Fam216b	1.000	1.000	1.000	1.000	1.000	1.000	0.000	0.000	0.000	219170	Fam216b	chr14	-	NM_1776	P560	protein_cc	9330158N	family wit	Fam216b
6490	Tnfsf11	1.000	1.000	1.000	1.000	1.000	1.000	0.000	0.000	0.000	21943	Tnfsf11	chr14	-	NM_0116	P10991	protein_cc	Ly109l| Ol	tumor nec	Tnfsf11
6491	Akap11	2.109	0.858	1.810	0.000	0.548	0.391	8.718	9.795	9.574	219181	Akap11	chr14	-	NM_0011	P3915	protein_cc	6330501D	A kinase (	Akap11
6492	Dgkh	2.346	1.028	2.413	0.259	0.960	0.376	2.326	3.557	3.597	380921	Dgkh	chr14	-	NM_0012	P9751	protein_cc	5930402B	diacylglyce	Dgkh
6493	Vwa8	0.269	1.594	0.429	0.000	0.077	0.019	9.048	7.156	7.829	219189	Vwa8	chr14	+	NM_0279	P26848	protein_cc	1300010F	von Willeb	Vwa8
6494	Zfp957	2.149	0.465	1.000	0.306	1.000	0.423	0.000	1.104	0.000	105590	Zfp957	chr14	-	NM_0010	P13420	protein_cc	AU017455	zinc finger	Zfp957
6495	Rgcc	13.595	1.031	14.010	0.000	0.877	0.083	0.002	3.767	3.811	66214	Rgcc	chr14	-	NM_0254	P23519	protein_cc	1190002H	regulator	Rgcc
6496	Naa16	1.075	0.886	0.952	0.665	0.493	0.387	7.039	7.143	6.969	66897	Naa16	chr14	-	NM_0258	P21557	protein_cc	1300019C	N(alpha)-a	Naa16
6497	Mtrf1	0.763	1.307	0.997	0.325	0.173	0.994	6.230	5.840	6.226	211253	Mtrf1	chr14	+	NM_1459	P13917	protein_cc	A830062K	mitochonc	Mtrf1
6498	Kbtbd7	1.004	0.652	0.655	0.974	0.041	0.056	6.617	6.624	6.007	211255	Kbtbd7	chr14	+	NM_0010	P18124	protein_cc	1110008P	kelch repe	Kbtbd7
6499	Kbtbd6	0.641	0.884	0.566	0.439	0.755	0.558	1.325	0.683	0.504	432879	Kbtbd6	chr14	+	NM_0010	P25851	protein_cc	EG432879	kelch repe	Kbtbd6
6500	Wbp4	1.284	0.816	1.047	0.001	0.001	0.317	8.250	8.610	8.316	22380	Wbp4	chr14	-	NM_0187	P11665	protein_cc	AW54503	WW doma	Wbp4
6501	Elf1	1.912	0.670	1.280	0.001	0.004	0.300	7.877	8.812	8.234	13709	Elf1	chr14	+	NM_0079	P22079	protein_cc	Elf-1| mEl	E74-like fa	Elf1
6502	Sugt1	1.826	0.802	1.465	0.002	0.031	0.013	8.820	9.689	9.372	67955	Sugt1	chr14	+	NM_0264	P13726	protein_cc	2410174K	SGT1, sup	Sugt1
6503	Lect1	0.045	1.480	0.068	0.000	0.573	0.005	5.683	1.229	1.795	16840	Lect1	chr14	-	NM_0107	P6999	protein_cc	Bricd3| Ch	leukocyte	Lect1
6504	4930452G	1.000	1.000	1.000	1.000	1.000	1.000	0.000	0.000	0.000	73989	4930452G	chr14	+	NR_04506	.	lncRNA	.	RIKEN cDN	4930452G
6505	Pcdh8	1.000	1.000	1.000	1.000	1.000	1.000	0.000	0.000	0.000	18530	Pcdh8	chr14	-	NM_0010	P14910	protein_cc	1700080P	protocadh	Pcdh8
6506	Gm10845	0.669	0.995	0.666	0.181	1.000	0.423	0.587	0.008	0.000	10003873	Gm10845	chr14	-	NR_03353	.	lncRNA	ENSMUSG	predicted	Gm10845
6507	Olfm4	1.000	1.000	1.000	1.000	1.000	1.000	0.000	0.000	0.000	380924	Olfm4	chr14	+	NM_0010	P6447	protein_cc	GC1| Gm2	olfactome	Olfm4
6508	Pcdh17	1.073	1.799	1.930	0.725	0.014	0.020	6.237	6.338	7.186	219228	Pcdh17	chr14	+	NM_0010	P25816	protein_cc	C030033F	protocadh	Pcdh17
6509	9630013A	3.938	0.442	1.742	0.004	0.032	0.423	0.001	1.978	0.802	319903	9630013A	chr14	-	NR_01553	.	lncRNA	.	RIKEN cDN	9630013A
6510	4930529K	1.000	1.000	1.000	1.000	1.000	1.000	0.000	0.000	0.000	75155	4930529K	chr14	+	NR_04045	.	lncRNA	4930507G	RIKEN cDN	4930529K
6511	Diap3	6.929	0.974	6.751	0.114	0.961	0.178	0.002	2.794	2.757	.	Diap3	chr14	-	NM_0196	P15021	.	.	.	Diap3
6512	Tdrd3	0.600	0.595	0.357	0.000	0.000	0.005	6.091	5.353	4.603	219249	Tdrd3	chr14	+	NM_0012	P2319	protein_cc	4732418C	tudor dom	Tdrd3
6513	Pcdh20	0.203	1.770	0.359	0.023	0.196	0.249	2.364	0.062	0.886	219257	Pcdh20	chr14	-	NM_1786	P4044	protein_cc	C630015B	protocadh	Pcdh20
6514	Gm5088	1.077	0.624	0.672	0.429	0.070	0.311	6.382	6.488	5.809	328451	Gm5088	chr14	+	NR_00286	.	misc_RNA	EG328451	predicted	Gm5088
6515	4930474H	1.005	1.405	1.412	0.000	0.182	0.423	0.000	0.008	0.498	74967	4930474H	chr14	+	NR_04571	.	lncRNA	.	RIKEN cDN	4930474H
6516	Pcdh9	1.589	0.448	0.712	0.585	0.384	0.753	2.005	2.674	1.514	211712	Pcdh9	chr14	-	NM_0012	P14449	protein_cc	.	protocadh	Pcdh9
6517	4921530L	1.000	1.000	1.000	1.000	1.000	1.000	0.000	0.000	0.000	66732	4921530L	chr14	+	NM_0257	P26368	protein_cc	.	RIKEN cDN	4921530L
6518	Klhl1	1.000	1.000	1.000	1.000	1.000	1.000	0.000	0.000	0.000	93688	Klhl1	chr14	-	NM_0531	P4730	protein_cc	mKIAA149	kelch-like	Klhl1
6519	Dach1	4.346	0.391	1.701	0.028	0.042	0.481	2.082	4.202	2.849	13134	Dach1	chr14	-	NM_0010	P3523	protein_cc	Dac| Dach	dachshund	Dach1
6520	Mzt1	0.799	1.085	0.867	0.063	0.202	0.344	6.679	6.355	6.472	76789	Mzt1	chr14	-	NM_1752	P26054	protein_cc	2410174K	mitotic sp	Mzt1
6521	Bora	0.898	2.467	2.215	0.862	0.041	0.205	1.326	1.171	2.473	77744	Bora	chr14	+	NM_1752	P12308	protein_cc	6720463N	bora, auro	Bora
6522	Dis3	1.905	1.393	2.655	0.001	0.092	0.102	6.300	7.230	7.709	72662	Dis3	chr14	-	NM_0283	P21685	protein_cc	2810028N	DIS3 hom	Dis3
6523	Pibf1	1.825	0.482	0.880	0.002	0.004	0.588	4.524	5.392	4.340	52023	Pibf1	chr14	+	NM_0293	P4569	protein_cc	.	progester	Pibf1
6524	Klf5	0.819	1.670	1.367	0.659	0.525	0.789	4.916	4.628	5.367	12224	Klf5	chr14	+	NM_0097	P25331	protein_cc	4930520J(	Kruppel-lil	Klf5
6525	Klf12	0.016	8.908	0.145	0.000	0.102	0.014	8.200	2.262	5.417	16597	Klf12	chr14	-	NM_0106	P21746	protein_cc	2700063E	Kruppel-lil	Klf12
6526	Prr30	1.000	1.000	1.000	1.000	1.000	1.000	0.000	0.000	0.000	76627	Prr30	chr14	-	NM_0296	P17028	protein_cc	1700110N	proline ric	Prr30
6527	Tbc1d4	0.469	1.652	0.775	0.023	0.161	0.297	5.167	4.075	4.799	210789	Tbc1d4	chr14	-	NM_0010	P19948	protein_cc	5930406J(	TBC1 dom	Tbc1d4
6528	Commd6	0.999	1.214	1.213	0.997	0.192	0.154	7.960	7.960	8.239	66200	Commd6	chr14	-	NM_0010	P16369	protein_cc	1110059J(	COMM do	Commd6
6529	Uchl3	1.157	1.090	1.262	0.444	0.601	0.052	7.967	8.178	8.302	50933	Uchl3	chr14	+	NM_0167	P26054	protein_cc	.	ubiquitin c	Uchl3
6530	Lmo7	0.451	1.154	0.521	0.006	0.007	0.107	7.853	6.705	6.912	380928	Lmo7	chr14	+	NM_2015	P25502	protein_cc	C78582| F	LIM doma	Lmo7
6531	Kctd12	0.930	1.025	0.954	0.244	0.843	0.835	6.649	6.545	6.580	239217	Kctd12	chr14	-	NM_1777	P8609	protein_cc	AU046135	potassium	Kctd12
6532	4933432I(	2.700	0.370	1.000	0.305	1.000	0.423	0.001	1.433	0.000	71264	4933432I(	chr14	-	NR_04565	.	lncRNA	.	RIKEN cDN	4933432I0
6533	Irg1	734.393	0.193	141.640	0.000	0.000	0.108	0.003	9.523	7.149	.	Irg1	chr14	+	NM_0083	P25464	.	.	.	Irg1
6534	Cln5	1.900	0.756	1.436	0.029	0.185	0.180	8.215	9.141	8.737	211286	Cln5	chr14	+	NM_0010	P20746	protein_cc	A730075N	ceroid-lipc	Cln5
6535	Fbxl3	0.835	1.017	0.849	0.124	0.908	0.522	7.466	7.205	7.229	50789	Fbxl3	chr14	-	NM_0158	P25913	protein_cc	AU041772	F-box and	Fbxl3
6536	Mycbp2	1.536	0.620	0.952	0.002	0.003	0.578	7.989	8.607	7.918	105689	Mycbp2	chr14	-	NM_2072	P24575	protein_cc	AU023734	MYC bindi	Mycbp2
6537	Scel	0.669	0.995	0.666	0.181	1.000	0.423	0.587	0.008	0.000	64929	Scel	chr14	+	NM_0228	P22559	protein_cc	9230114I(	sciellin	Scel
6538	Slain1	1.015	2.326	2.360	0.000	0.182	0.423	0.000	0.021	1.239	105439	Slain1	chr14	+	NM_1980	P20477	protein_cc	96300440	SLAIN mot	Slain1
6539	Slain1os	1.000	1.000	1.000	1.000	1.000	1.000	0.000	0.000	0.000	70898	Slain1os	chr14	-	NR_04514	.	lncRNA	4921525B	SLAIN mot	Slain1os

6540	Ednrb	1.233	1.855	2.287	0.588	0.060	0.061	6.161	6.463	7.355	13618	Ednrb	chr14	-	NM_0012	P21634	protein_cc	ET-B| ET-E	endothelin	Ednrb
6541	4930432J(	0.552	0.990	0.547	0.182	1.000	0.423	0.871	0.015	0.000	73877	4930432J(	chr14	-	NR_04595	.	lncRNA	.	RIKEN cDN	4930432J0
6542	Pou4f1	2.474	0.332	0.820	0.136	0.085	0.762	1.058	2.364	0.772	18996	Pou4f1	chr14	-	NM_0111	P17320	protein_cc	Brn-3| Brr	POU doma	Pou4f1
6543	Rnf219	1.648	0.728	1.200	0.341	0.456	0.715	4.558	5.279	4.821	72486	Rnf219	chr14	-	NM_0260	P21545	protein_cc	2610206B	ring finger	Rnf219
6544	D130009I	0.552	0.990	0.547	0.182	1.000	0.423	0.871	0.015	0.000	320249	D130009I	chr14	+	NR_01559	.	lncRNA	.	RIKEN cDN	D130009I1
6545	Gm17066	1.653	0.905	1.496	0.000	0.289	0.125	7.509	8.234	8.090	10050298	Gm17066	chr14	-	NR_04062	.	lncRNA	.	predicted	Gm17066
6546	Rbm26	1.753	0.795	1.395	0.003	0.037	0.021	8.037	8.847	8.517	74213	Rbm26	chr14	-	NM_1340	P19471	protein_cc	1700009P	RNA bindi	Rbm26
6547	Ndfip2	0.562	1.328	0.746	0.019	0.225	0.203	9.852	9.020	9.429	76273	Ndfip2	chr14	+	NM_0295	P20016	protein_cc	0710001O	Nedd4 far	Ndfip2
6548	5430440P	1.000	1.000	1.000	1.000	1.000	1.000	0.000	0.000	0.000	71362	5430440P	chr14	+	NR_04585	.	lncRNA	.	RIKEN cDN	5430440P1
6549	4930449E	1.000	1.000	1.000	1.000	1.000	1.000	0.000	0.000	0.000	74864	4930449E	chr14	+	NR_04592	.	lncRNA	.	RIKEN cDN	4930449E(
6550	9330188P	0.339	0.981	0.333	0.181	1.000	0.423	1.588	0.028	0.000	380930	9330188P	chr14	+	NR_10231	.	lncRNA	Gm1589|	RIKEN cDN	9330188P(
6551	Spry2	2.370	1.085	2.573	0.038	0.768	0.224	4.956	6.201	6.319	24064	Spry2	chr14	-	NM_0118	P19077	protein_cc	sproutyZ	sprouty hc	Spry2
6552	Trim52	1.000	1.000	1.000	1.000	1.000	1.000	0.000	0.000	0.000	212085	Trim52	chr14	+	NM_07320	.	protein_cc	4921513B	tripartite	Trim52
6553	1700128A	1.000	1.000	1.000	1.000	1.000	1.000	0.000	0.000	0.000	73631	1700128A	chr14	-	NR_04593	.	lncRNA	.	RIKEN cDN	1700128A(
6554	Slitrk1	1.000	1.000	1.000	1.000	1.000	1.000	0.000	0.000	0.000	76965	Slitrk1	chr14	-	NM_1990	P4075	protein_cc	3200001I(	SLIT and N	Slitrk1
6555	Slitrk6	0.552	0.990	0.547	0.182	1.000	0.423	0.871	0.015	0.000	239250	Slitrk6	chr14	-	NM_1754	P24208	protein_cc	4832410J2	SLIT and N	Slitrk6
6556	Slitrk5	4.466	0.224	0.999	0.307	0.159	0.423	0.001	2.160	0.000	75409	Slitrk5	chr14	+	NM_1988	P19923	protein_cc	2610019D	SLIT and N	Slitrk5
6557	4930524C	1.000	1.000	1.000	1.000	1.000	1.000	0.000	0.000	0.000	75822	4930524C	chr14	+	NR_04566	.	lncRNA	.	RIKEN cDN	4930524C1
6558	1700100I	1.603	1.658	2.658	0.292	0.404	0.423	0.001	0.681	1.411	74982	1700100I1	chr14	-	NR_12655	.	lncRNA	1700085D	RIKEN cDN	1700100I1
6559	Gpc5	1.000	1.000	1.000	1.000	1.000	1.000	0.000	0.000	0.000	103978	Gpc5	chr14	+	NM_1755	P21154	protein_cc	A230034F	glypican 5	Gpc5
6560	4930505G	1.000	1.000	1.000	1.000	1.000	1.000	0.000	0.000	0.000	75088	4930505G	chr14	-	NR_04576	.	lncRNA	.	RIKEN cDN	4930505G1
6561	Gpc6	0.487	0.678	0.331	0.164	0.572	0.124	4.197	3.160	2.599	23888	Gpc6	chr14	+	NM_0010	P11819	protein_cc	.	glypican 6	Gpc6
6562	Dct	0.505	0.555	0.280	0.002	0.023	0.030	6.926	5.939	5.091	13190	Dct	chr14	-	NM_0100	P15996	protein_cc	DT| slaty|	dopachror	Dct
6563	Tgds	1.122	0.562	0.631	0.765	0.258	0.264	6.345	6.512	5.682	76355	Tgds	chr14	-	NM_0295	P5670	protein_cc	2610017J1	TDP-gluco	Tgds
6564	Gpr180	1.940	0.607	1.178	0.010	0.011	0.571	7.365	8.322	7.602	58245	Gpr180	chr14	+	NM_0214	P2980	protein_cc	AB041545	G protein-	Gpr180
6565	Sox21	0.716	0.995	0.712	0.182	1.000	0.423	0.490	0.008	0.000	223227	Sox21	chr14	-	NM_1777	P1146	protein_cc	Sox25	SRY (sex d	Sox21
6566	LOC10524	1.000	1.000	1.000	1.000	1.000	1.000	0.000	0.000	0.000	10524586	LOC10524	chr14	+	NR_13197	.	lncRNA	.	uncharact	LOC10524
6567	Gm9376	1.000	1.000	1.000	1.000	1.000	1.000	0.000	0.000	0.000	668814	Gm9376	chr14	+	NM_0011	P16849	protein_cc	EG668814	predicted	Gm9376
6568	1700044C	1.000	1.000	1.000	1.000	1.000	1.000	0.000	0.000	0.000	73306	1700044C	chr14	+	NR_04562	.	lncRNA	.	RIKEN cDN	1700044C(
6569	Abcc4	0.732	0.441	0.323	0.389	0.221	0.021	5.332	4.882	3.702	239273	Abcc4	chr14	-	NM_0011	P11922	protein_cc	D630049P	ATP-bindi	Abcc4
6570	Cldn10	1.572	0.636	1.000	0.304	1.000	0.423	0.000	0.653	0.000	58187	Cldn10	chr14	+	NM_0011	P128	protein_cc	6720456I1	claudin 10	Cldn10
6571	Dzip1	0.653	1.195	0.780	0.428	0.805	0.758	2.102	1.486	1.744	66573	Dzip1	chr14	-	NM_0259	P20122	protein_cc	2510025K	DAZ intera	Dzip1
6572	Dnajc3	1.810	0.759	1.373	0.001	0.054	0.242	11.803	12.660	12.261	10003725	Dnajc3	chr14	+	NM_0089	P12392	protein_cc	AA408985	DnaJ heat	Dnajc3
6573	Uggt2	1.103	0.390	0.431	0.372	0.003	0.003	4.067	4.208	2.851	66435	Uggt2	chr14	-	NM_0010	P11593	protein_cc	1810064L	UDP-gluco	Uggt2
6574	Hs6st3	1.000	1.000	1.000	1.000	1.000	1.000	0.000	0.000	0.000	50787	Hs6st3	chr14	+	NM_0158	P13176	protein_cc	6OST3| H!	heparan s	Hs6st3
6575	1700006F	1.000	1.000	1.000	1.000	1.000	1.000	0.000	0.000	0.000	75432	1700006F	chr14	+	NR_04562	.	lncRNA	.	RIKEN cDN	1700006F(
6576	Oxgr1	1.005	1.439	1.446	0.000	0.182	0.423	0.000	0.008	0.532	239283	Oxgr1	chr14	-	NM_0010	P8410	protein_cc	Cysltr3| G	oxoglutara	Oxgr1
6577	Mbnl2	1.418	0.988	1.402	0.002	0.935	0.321	8.700	9.204	9.187	105559	Mbnl2	chr14	+	NM_1753	P10073	protein_cc	1110002M	musclebli	Mbnl2
6578	Rap2a	0.879	0.755	0.663	0.532	0.306	0.049	7.483	7.296	6.890	76108	Rap2a	chr14	+	NM_0295	P24113	protein_cc	5830461H	RAS relate	Rap2a
6579	Ipo5	1.007	1.243	1.252	0.852	0.034	0.171	7.618	7.629	7.942	70572	Ipo5	chr14	+	NM_0235	P9948	protein_cc	1110011C	importin 5	Ipo5
6580	Farp1	4.540	0.966	4.385	0.000	0.859	0.111	5.681	7.864	7.814	223254	Farp1	chr14	+	NM_1340	P9893	protein_cc	AW22884	FERM, Rhc	Farp1
6581	Stk24	1.436	0.913	1.312	0.008	0.685	0.568	7.485	8.008	7.877	223255	Stk24	chr14	-	NM_1454	P16119	protein_cc	1810013H	serine/thr	Stk24
6582	Slc15a1	1.053	0.632	0.666	0.897	1.000	0.423	0.587	0.661	0.000	56643	Slc15a1	chr14	-	NM_0530	P17602	protein_cc	D630032F	solute car	Slc15a1
6583	Dock9	0.297	1.966	0.584	0.009	0.008	0.211	6.020	4.270	5.245	105445	Dock9	chr14	-	NM_0011	P2758	protein_cc	AA959601	dedicator	Dock9
6584	1810041H	1.597	0.658	1.050	0.137	0.089	0.905	3.025	3.701	3.096	78312	1810041H	chr14	-	NR_13099	.	lncRNA	.	RIKEN cDN	1810041H1
6585	Ubac2	0.572	1.205	0.689	0.002	0.152	0.153	8.186	7.380	7.649	68889	Ubac2	chr14	+	NM_0268	P23666	protein_cc	1190008A	ubiquitin a	Ubac2
6586	Gpr18	23.630	0.367	8.666	0.019	0.080	0.202	0.002	4.565	3.118	110168	Gpr18	chr14	-	NM_1828	P14771	protein_cc	.	G protein-	Gpr18
6587	Gpr183	3.790	0.522	1.976	0.036	0.114	0.230	0.740	2.662	1.723	321019	Gpr183	chr14	-	NM_1830	P12336	protein_cc	Ebi2	G protein-	Gpr183
6588	Timm8a2	2.700	0.370	1.000	0.305	1.000	0.423	0.001	1.433	0.000	223262	Timm8a2	chr14	+	NM_0010	P8505	protein_cc	.	translocas	Timm8a2
6589	A330035P	2.097	1.424	2.987	0.077	0.127	0.086	1.480	2.548	3.058	319727	A330035P	chr14	-	NR_01558	.	lncRNA	.	RIKEN cDN	A330035P1
6590	Tm9sf2	1.118	1.158	1.295	0.279	0.184	0.140	10.702	10.863	11.075	68059	Tm9sf2	chr14	+	NM_0805	P5462	protein_cc	1500001N	transmem	Tm9sf2
6591	Clybl	0.414	1.752	0.726	0.000	0.003	0.062	9.213	7.941	8.751	69634	Clybl	chr14	+	NM_0295	P25880	protein_cc	0610033J(	citrate lya	Clybl
6592	1700108J(	1.000	1.000	1.000	1.000	1.000	1.000	0.000	0.000	0.000	68245	1700108J(	chr14	-	NR_01553	.	lncRNA	.	RIKEN cDN	1700108J0
6593	Gm5089	1.000	1.000	1.000	1.000	1.000	1.000	0.000	0.000	0.000	328479	Gm5089	chr14	-	NR_03332	.	lncRNA	6530402A	predicted	Gm5089
6594	Zic5	1.000	1.000	1.000	1.000	1.000	1.000	0.000	0.000	0.000	65100	Zic5	chr14	-	NM_0229	P25427	protein_cc	1700049L	zinc finger	Zic5
6595	2610035F	1.000	1.000	1.000	1.000	1.000	1.000	0.000	0.000	0.000	69248	2610035F	chr14	-	NR_04504	.	lncRNA	.	RIKEN cDN	2610035F2
6596	Zic2	1.000	1.000	1.000	1.000	1.000	1.000	0.000	0.000	0.000	22772	Zic2	chr14	+	NM_0095	P7712	protein_cc	HPE5| Ku	zinc finger	Zic2
6597	Pcca	0.395	1.057	0.417	0.002	0.661	0.065	9.113	7.771	7.851	110821	Pcca	chr14	+	NM_1448	P12108	protein_cc	C79630	propionyl-	Pcca
6598	Ggact	0.200	2.047	0.409	0.000	0.005	0.010	10.125	7.802	8.836	223267	Ggact	chr14	-	NM_1454	P698	protein_cc	A2ld1	gamma-gl	Ggact
6599	Tmtc4	0.433	0.882	0.382	0.157	0.766	0.387	5.754	4.546	4.365	70551	Tmtc4	chr14	-	NM_0286	P10839	protein_cc	4930403J	transmem	Tmtc4
6600	AA536875	1.000	1.000	1.000	1.000	1.000	1.000	0.000	0.000	0.000	380934	AA536875	chr14	+	NR_04514	.	lncRNA	Gm1591	expressed	AA536875
6601	Nalcn	0.552	0.990	0.547	0.182	1.000	0.423	0.871	0.015	0.000	338370	Nalcn	chr14	-	NM_1773	P453	protein_cc	A530023G	sodium lea	Nalcn
6602	Itgbl1	0.304	2.511	0.764	0.183	0.186	0.807	1.772	0.056	1.384	223272	Itgbl1	chr14	+	NM_1454	P12935	protein_cc	B930011D	integrin, b	Itgbl1
6603	Fgf14	1.000	1.000	1.000	1.000	1.000	1.000	0.000	0.000	0.000	14169	Fgf14	chr14	-	NM_2076	P9134	protein_cc	FHF-4| Fh	fibroblast	Fgf14
6604	1700024B	1.000	1.000	1.000	1.000	1.000	1.000	0.000	0.000	0.000	69393	1700024B	chr14	+	NR_04547	.	lncRNA	.	RIKEN cDN	1700024B1
6605	Sepp1	0.261	1.599	0.417	0.000	0.003	0.006	15.762	13.823	14.500	20363	Sepp1	chr15	+	NM_0010	P25837	protein_cc	AU018766	selenopro	Sepp1
6606	Ccdc152	0.111	3.860	0.430	0.000	0.000	0.008	7.690	4.522	6.470	10003913	Ccdc152	chr15	-	NM_0011	P21181	protein_cc	ENSMUSG	coiled-coil	Ccdc152
6607	Ghr	0.198	1.539	0.305	0.000	0.150	0.024	11.742	9.409	10.031	14600	Ghr	chr15	-	NM_0102	P8015	protein_cc	GHBP| GH	growth hc	Ghr
6608	Fbxo4	2.329	1.168	2.720	0.003	0.275	0.066	7.629	8.849	9.072	106052	Fbxo4	chr15	-	NM_1340	P23605	protein_cc	1700096C	F-box prot	Fbxo4
6609	AW54987	0.657	0.688	0.452	0.076	0.142	0.091	7.173	6.566	6.027	106064	AW54987	chr15	-	NM_1459	P15987	protein_cc	AI195826	expressed	AW54987
6610	A630020A	1.009	0.811	0.818	0.988	0.725	0.804	1.110	1.123	0.821	328489	A630020A	chr15	+	NR_04574	.	lncRNA	.	uncharact	A630020A(
6611	BC037032	0.716	0.995	0.712	0.182	1.000	0.423	0.490	0.008	0.000	414066	BC037032	chr15	-	NR_02826	.	lncRNA	.	cDNA Seq	BC037032
6612	Oxct1	1.196	0.783	0.936	0.480	0.383	0.783	6.320	6.577	6.224	67041	Oxct1	chr15	+	NM_0241	P18620	protein_cc	2610008O	3-oxoacid	Oxct1
6613	Plcxd3	0.291	2.514	0.732	0.002	0.000	0.235	1.837	0.057	1.387	239318	Plcxd3	chr15	+	NM_1773	P4365	protein_cc	B130016O	phosphati	Plcxd3
6614	C6	0.417	7.444	3.107	0.012	0.008	0.134	6.792	5.532	8.428	12274	C6	chr15	+	NM_0167	P4164	protein_cc	AW11162	compleme	C6
6615	Mroh2b	1.000	1.000	1.000	1.000	1.000	1.000	0.000	0.000	0.000	223825	Mroh2b	chr15	+	NM_0011	P1952	protein_cc	4930455B	maestro h	Mroh2b
6616	C7	1.019	2.651	2.701	0.000	0.000	0.002	0.000	0.027	1.434	109828	C7	chr15	-	NM_0012	P16104	protein_cc	A530026G	compleme	C7
6617	Card6	0.769	0.911	0.701	0.031	0.008	0.119	6.624	6.245	6.111	239319	Card6	chr15	-	NM_0011	P8292	protein_cc	D730008L	caspase re	Card6
6618	Rpl37	1.315	1.492	1.961	0.007	0.001	0.002	10.837	11.232	11.808	67281	Rpl37	chr15	+	NM_0260	P25200	protein_cc	3110005M	ribosomal	Rpl37
6619	Snord72	4.026	1.102	4.435	0.002	0.803	0.292	0.370	2.380	2.520	10030252	Snord72	chr15	+	NR_02809	.	snoRNA	MBII-240	small nucl	Snord72
6620	Prkaa1	2.490	0.698	1.738	0.023	0.172	0.149	6.209	7.525	7.006	105787	Prkaa1	chr15	+	NM_0010	P19130	protein_cc	AI194361	protein kir	Prkaa1
6621	Ttc33	1.295	1.333	1.727	0.087	0.051	0.058	8.144	8.517	8.932	67515	Ttc33	chr15	+	NM_0262	P26326	protein_cc	2410099N	tetratricop	Ttc33
6622	Ptger4	2.977	0.380	1.133	0.007	0.033	0.902	3.917	5.491	4.097	19219	Ptger4	chr15	-	NM_0089	P992	protein_cc	EP4| Ptge	prostaglar	Ptger4
6623	5430437J	0.750	2.739	2.055	0.200	0.182	0.516	0.448	0.033	1.487	71432	5430437J1	chr15	+	NR_04527	.	lncRNA	.	RIKEN cDN	5430437J1
6624	Dab2	1.308	0.864	1.129	0.086	0.396	0.684	7.518	7.905	7.694	13132	Dab2	chr15	+	NM_0010	P15835	protein_cc	5730435J1	disabled 2	Dab2
6625	C9	0.602	2.214	1.334	0.024	0.001	0.248	9.199	8.467	9.614	12279	C9	chr15	+	NM_0134	P5425	protein_cc	.	compleme	C9
6626	Fyb	2.019	0.644	1.299	0.011	0.100	0.548	6.437	7.451	6.815	23880	Fyb	chr15	+	NM_0012	P5495	protein_cc	ADAP| B6	FYN bindir	Fyb
6627	Rictor	1.048	0.851	0.892	0.840	0.532	0.463	7.427	7.495	7.261	78757	Rictor	chr15	+	NM_0301	P19559	protein_cc	4921505C	RPTOR ind	Rictor
6628	Osmr	11.634	0.634	7.381	0.000	0.014	0.013	4.583	8.123	7.467	18414	Osmr	chr15	-	NM_0110	P26053	protein_cc	OSMRB	oncostatir	Osmr
6629	Lifr	0.173	1.125	0.195	0.001	0.516	0.037	11.295	8.766	8.936	16880	Lifr	chr15	+	NM_0011	P12094	protein_cc	A230075M	leukemia i	Lifr
6630	Egflam	0.310	2.663	0.826	0.087	0.185	0.833	1.742	0.054	1.466	268780	Egflam	chr15	-	NM_0012	P25577	protein_cc	5930412K	EGF-like, f	Egflam

6631	Gdnf	1.000	1.000	1.000	1.000	1.000	1.000	0.000	0.000	0.000	14573	Gdnf	chr15	+	NM_0013(	P8002	protein_cc	AI385739	glial cell lir	Gdnf
6632	Wdr70	0.605	0.869	0.526	0.045	0.096	0.175	6.056	5.333	5.130	545085	Wdr70	chr15	-	NM_0010:	P16516	protein_cc	4833422F(	WD repea	Wdr70
6633	Nup155	1.272	0.610	0.777	0.031	0.008	0.166	6.522	6.870	6.158	170762	Nup155	chr15	+	NM_1332	P12769	protein_cc	D930027N	nucleopor	Nup155
6634	2410089E(	1.458	0.588	0.857	0.325	0.185	0.693	4.448	4.992	4.226	73692	2410089E(	chr15	-	NM_0011(	P26423	protein_cc	4732468D	RIKEN cDN	2410089E(
6635	Nipbl	1.300	0.611	0.795	0.005	0.000	0.090	9.726	10.105	9.395	71175	Nipbl	chr15	-	NM_0277(	P21469	protein_cc	Idn3	Nipped-B	Nipbl
6636	Slc1a3	0.499	1.459	0.728	0.286	0.057	0.717	2.525	1.522	2.067	20512	Slc1a3	chr15	-	NM_1489:	P14674	protein_cc	AI504299|	solute car	Slc1a3
6637	Ranbp3l	0.150	0.438	0.066	0.001	1.000	0.007	3.931	1.190	0.000	223332	Ranbp3l	chr15	+	NM_1980:	P25152	protein_cc	C130037N	RAN bindi	Ranbp3l
6638	Nadk2	0.254	1.181	0.300	0.000	0.286	0.014	9.391	7.417	7.657	68646	Nadk2	chr15	+	NM_0010:	P24562	protein_cc	MNADK| I	NAD kinas	Nadk2
6639	Skp2	1.494	0.495	0.739	0.394	0.235	0.463	3.193	3.772	2.756	27401	Skp2	chr15	-	NM_0012:	P96	protein_cc	4930500A	S-phase ki	Skp2
6640	Lmbrd2	0.398	1.143	0.455	0.000	0.492	0.050	9.293	7.965	8.157	320506	Lmbrd2	chr15	+	NM_1771	P26141	protein_cc	9930036E:	LMBR1 do	Lmbrd2
6641	Ugt3a1	0.605	2.134	1.291	0.030	0.066	0.590	8.284	7.559	8.652	105887	Ugt3a1	chr15	+	NM_2072	P16755	protein_cc	AI746432	UDP glyco	Ugt3a1
6642	Ugt3a2	0.253	0.846	0.214	0.002	0.218	0.042	10.956	8.973	8.731	223337	Ugt3a2	chr15	+	NM_1448	P22553	protein_cc	AI313915	UDP glyco	Ugt3a2
6643	Capsl	0.423	2.240	0.949	0.051	0.289	0.952	2.773	1.534	2.697	75568	Capsl	chr15	+	NM_0293	P8488	protein_cc	1700028N	calcyphos	Capsl
6644	Il7r	6.284	0.407	2.555	0.041	0.142	0.471	1.849	4.501	3.202	16197	Il7r	chr15	-	NM_0083	P6711	protein_cc	CD127| IL	interleukin	Il7r
6645	Spef2	16.088	0.385	6.191	0.008	0.069	0.353	0.698	4.706	3.329	320277	Spef2	chr15	-	NM_0013(	P10423	protein_cc	C230086A	sperm flag	Spef2
6646	Prlr	0.245	0.559	0.137	0.000	0.000	0.001	11.082	9.054	8.216	19116	Prlr	chr15	+	NM_0111(	P14979	protein_cc	AI987712|	prolactin r	Prlr
6647	Agxt2	0.682	0.494	0.337	0.167	0.000	0.161	6.639	6.087	5.068	268782	Agxt2	chr15	+	NM_0010:	P18522	protein_cc	AI303810|	alanine-gl	Agxt2
6648	Dnajc21	1.355	0.689	0.933	0.072	0.024	0.681	9.255	9.694	9.155	78244	Dnajc21	chr15	-	NM_0300	P14412	protein_cc	4930461P.	DnaJ heat	Dnajc21
6649	Brix1	2.443	0.872	2.131	0.000	0.146	0.064	8.101	9.390	9.192	67832	Brix1	chr15	-	NM_0263	P12989	protein_cc	1110064N	BRX1, biog	Brix1
6650	Rad1	2.106	0.486	1.024	0.000	0.001	0.924	5.873	6.947	5.907	19355	Rad1	chr15	+	NM_0112:	P4345	protein_cc	mRAD1	RAD1 chec	Rad1
6651	Ttc23l	1.000	1.000	1.000	1.000	1.000	1.000	0.000	0.000	0.000	75777	Ttc23l	chr15	-	NM_0294	P14859	protein_cc	4930401A	tetratricop	Ttc23l
6652	Rai14	1.522	0.559	0.850	0.171	0.110	0.463	7.038	7.644	6.804	75646	Rai14	chr15	-	NM_0306	P20170	protein_cc	1700008J1	retinoic ac	Rai14
6653	Gm10389	1.983	0.085	0.169	0.139	1.000	0.032	2.567	3.555	0.000	10003860	Gm10389	chr15	+	NR_03354	.	lncRNA	ENSMUSG	predicted	Gm10389
6654	4930556M	0.146	3.013	0.441	0.000	0.104	0.136	6.202	3.430	5.021	75259	4930556N	chr15	+	NR_04506	.	lncRNA	1700009D	RIKEN cDN	4930556M
6655	Gm19276	1.000	1.000	1.000	1.000	1.000	1.000	0.000	0.000	0.000	10050260	Gm19276	chr15	+	NR_04035	.	lncRNA	.	predicted	Gm19276
6656	C1qtnf3	1.030	3.842	3.956	0.000	0.181	0.423	0.001	0.043	1.985	81799	C1qtnf3	chr15	+	NM_0308	P14628	protein_cc	2310005P	C1q and tu	C1qtnf3
6657	Amacr	0.250	1.771	0.443	0.000	0.011	0.040	9.884	7.886	8.710	17117	Amacr	chr15	+	NM_0085	P22746	protein_cc	Macr1	alpha-met	Amacr
6658	Slc45a2	1.000	1.000	1.000	1.000	1.000	1.000	0.000	0.000	0.000	22293	Slc45a2	chr15	+	NM_0530	P2355	protein_cc	Aim-1| Aiı	solute car	Slc45a2
6659	Rxfp3	1.000	1.000	1.000	1.000	1.000	1.000	0.000	0.000	0.000	239336	Rxfp3	chr15	-	NM_1787	P23420	protein_cc	BC053073	relaxin far	Rxfp3
6660	Adamts12	1.000	1.000	1.000	1.000	1.000	1.000	0.000	0.000	0.000	239337	Adamts12	chr15	+	NM_1755(	P26462	protein_cc	ADAMTS-:	a disinteg	Adamts12
6661	Tars	1.537	0.638	0.980	0.060	0.056	0.714	10.551	11.171	10.523	110960	Tars	chr15	-	NM_0330	P7101	protein_cc	D15Wsu5!	threonyl-t	Tars
6662	Npr3	0.080	3.734	0.299	0.000	0.094	0.053	4.865	1.221	3.121	18162	Npr3	chr15	-	NM_0012:	P15473	protein_cc	ANP-C| AI	natriuretic	Npr3
6663	Gm5144	1.000	1.000	1.000	1.000	1.000	1.000	0.000	0.000	0.000	381002	Gm5144	chr15	+	NR_13105	.	lncRNA	381002	predicted	Gm5144
6664	1700047G	1.000	1.000	1.000	1.000	1.000	1.000	0.000	0.000	0.000	73339	1700047G	chr15	+	NR_04044	.	lncRNA	.	RIKEN cDN	1700047G(
6665	Sub1	1.772	0.840	1.488	0.000	0.320	0.358	10.041	10.867	10.614	20024	Sub1	chr15	-	NM_0112	P23467	protein_cc	AI842364|	SUB1 hom	Sub1
6666	Zfr	1.567	0.704	1.102	0.004	0.003	0.497	9.922	10.569	10.062	22763	Zfr	chr15	+	NM_0117	P8940	protein_cc	C920030H	zinc finger	Zfr
6667	Mtmr12	1.966	0.600	1.179	0.000	0.000	0.160	5.623	6.598	5.861	268783	Mtmr12	chr15	+	NM_1729	P20864	protein_cc	3Pap| 493	myotubula	Mtmr12
6668	Golph3	2.175	0.733	1.595	0.000	0.013	0.082	8.386	9.507	9.059	66629	Golph3	chr15	+	NM_0256	P22250	protein_cc	4733401N	golgi phos	Golph3
6669	Pdzd2	1.410	0.977	1.377	0.531	0.962	0.606	2.193	2.688	2.654	68070	Pdzd2	chr15	-	NM_0010:	P16920	protein_cc	4930537L(	PDZ doma	Pdzd2
6670	6030458C	1.530	0.978	1.495	0.116	0.895	0.399	6.649	7.262	7.229	77877	6030458C	chr15	-	NM_0299	P11190	protein_cc	.	RIKEN cDN	6030458C:
6671	Drosha	1.568	0.749	1.175	0.034	0.104	0.399	6.203	6.852	6.435	14000	Drosha	chr15	+	NM_0267	P10313	protein_cc	1110013A	drosha, rib	Drosha
6672	Cdh6	0.552	0.990	0.547	0.182	1.000	0.423	0.871	0.015	0.000	12563	Cdh6	chr15	-	NM_0076	P13598	protein_cc	cad6	cadherin 6	Cdh6
6673	Cdh9	1.000	1.000	1.000	1.000	1.000	1.000	0.000	0.000	0.000	12565	Cdh9	chr15	+	NM_0098	P18154	protein_cc	.	cadherin 9	Cdh9
6674	4921515E(	1.000	1.000	1.000	1.000	1.000	1.000	0.000	0.000	0.000	70886	4921515E(	chr15	-	NR_04571	.	lncRNA	.	.	4921515E(
6675	Cdh10	1.000	1.000	1.000	1.000	1.000	1.000	0.000	0.000	0.000	320873	Cdh10	chr15	+	NM_0098	P15936	protein_cc	A830016G	cadherin 1	Cdh10
6676	Acot10	2.652	1.373	3.641	0.033	0.132	0.058	2.485	3.893	4.350	64833	Acot10	chr15	-	NM_0228	P17751	protein_cc	Acate3| N	acyl-CoA t	Acot10
6677	Cdh12	1.005	1.405	1.412	0.000	0.182	0.423	0.000	0.008	0.498	215654	Cdh12	chr15	+	NM_0010	P14066	protein_cc	Cdhb	cadherin 1	Cdh12
6678	Gm5803	1.598	1.179	1.885	0.300	0.718	0.423	0.000	0.677	0.915	545091	Gm5803	chr15	-	NM_0011(	P16733	protein_cc	EG545091	predicted	Gm5803
6679	Cdh18	1.000	1.000	1.000	1.000	1.000	1.000	0.000	0.000	0.000	320865	Cdh18	chr15	+	NM_0010:	P6054	protein_cc	B230220E	cadherin 1	Cdh18
6680	Basp1	4.179	0.517	2.159	0.004	0.020	0.037	6.039	8.102	7.149	70350	Basp1	chr15	-	NM_0273	P21281	protein_cc	2610024P	brain abur	Basp1
6681	Gm5468	1.000	1.000	1.000	1.000	1.000	1.000	0.000	0.000	0.000	432939	Gm5468	chr15	+	NR_02737	.	lncRNA	10004058:	predicted	Gm5468
6682	Myo10	1.164	0.612	0.712	0.184	0.017	0.236	8.221	8.439	7.731	17909	Myo10	chr15	+	NM_0194	P10279	protein_cc	AW04872	myosin X	Myo10
6683	Fam134b	0.771	0.874	0.674	0.083	0.243	0.142	10.921	10.546	10.351	66270	Fam134b	chr15	+	NM_0010:	P23531	protein_cc	1810015C	family wit	Fam134b
6684	Zfp622	1.113	0.827	0.921	0.515	0.340	0.627	8.158	8.312	8.039	52521	Zfp622	chr15	+	NM_1445	P16581	protein_cc	1110033B	zinc finger	Zfp622
6685	March11	0.716	0.995	0.712	0.182	1.000	0.423	0.490	0.008	0.000	211147	March11	chr15	+	NM_1775	P7220	protein_cc	9630025C	membrane	March11
6686	Fbxl7	0.407	0.907	0.369	0.085	0.885	0.106	4.112	2.816	2.675	448987	Fbxl7	chr15	-	NM_1769	P146	protein_cc	AL023057	F-box and	Fbxl7
6687	Ank	0.193	1.909	0.368	0.000	0.007	0.007	8.214	5.840	6.773	11732	Ank	chr15	+	NM_0203	P10290	protein_cc	Ankh| D1!	progressiv	Ank
6688	Otulin	0.909	1.501	1.364	0.327	0.023	0.260	8.288	8.150	8.736	432940	Otulin	chr15	-	NM_0010	P7359	protein_cc	C79097| F	OTU deub	Otulin
6689	Fam105a	0.499	1.262	0.630	0.085	0.280	0.450	3.564	2.562	2.897	223433	Fam105a	chr15	-	NM_1983	P23907	protein_cc	9830126N	family wit	Fam105a
6690	Trio	1.172	0.835	0.979	0.734	0.685	0.944	6.100	6.330	6.070	223435	Trio	chr15	-	NM_0010:	P1076	protein_cc	6720464I(	triple func	Trio
6691	Dnah5	1.935	0.298	0.577	0.209	1.000	0.422	0.794	1.746	0.000	110082	Dnah5	chr15	+	NM_1333	P9323	protein_cc	AU022615	dynein, ax	Dnah5
6692	Ctnnd2	0.370	1.823	0.675	0.000	0.189	0.422	1.473	0.039	0.905	18163	Ctnnd2	chr15	+	NM_0087	P2989	protein_cc	Catnd2| n	catenin (c	Ctnnd2
6693	4930430F:	1.000	1.000	1.000	1.000	1.000	1.000	0.000	0.000	0.000	73784	4930430F:	chr15	+	NR_04518	.	lncRNA	.	RIKEN cDN	4930430F2
6694	Dap	1.636	0.968	1.584	0.000	0.479	0.020	11.714	12.424	12.378	223453	Dap	chr15	+	NM_1460	P7873	protein_cc	4921531N	death-ass	Dap
6695	Ankrd33b	0.173	1.436	0.248	0.000	0.040	0.001	7.127	4.594	5.116	67434	Ankrd33b	chr15	-	NM_0011(	P26593	protein_cc	0610012A	ankyrin re	Ankrd33b
6696	Ropn1l	0.394	0.883	0.348	0.000	0.228	0.008	7.485	6.141	5.961	252967	Ropn1l	chr15	-	NM_1458	P194	protein_cc	Akapasp|	ropporin 1	Ropn1l
6697	March6	0.591	1.082	0.640	0.003	0.516	0.120	9.748	8.990	9.103	223455	March6	chr15	-	NM_1726	P4920	protein_cc	3830408G	membrane	March6
6698	Cmbl	0.269	0.925	0.249	0.000	0.760	0.022	11.009	9.117	9.004	69574	Cmbl	chr15	+	NM_1815	P15761	protein_cc	2310016A	carboxym	Cmbl
6699	Cct5	1.048	1.216	1.274	0.630	0.080	0.291	9.440	9.508	9.790	12465	Cct5	chr15	-	NM_0076	P26281	protein_cc	Ccte| mKI	chaperoni	Cct5
6700	Fam173b	0.885	1.261	1.116	0.219	0.295	0.724	7.765	7.589	7.924	68073	Fam173b	chr15	+	NM_0265	P14356	protein_cc	A930016P	family wit	Fam173b
6701	Tas2r119	1.000	1.000	1.000	1.000	1.000	1.000	0.000	0.000	0.000	57254	Tas2r119	chr15	+	NM_0205	P12197	protein_cc	mGR19| n	taste rece	Tas2r119
6702	Snhg18	0.336	3.032	1.018	0.001	0.027	0.963	5.541	3.967	5.567	10061609	Snhg18	chr15	-	NR_03818	.	lncRNA	0610007N	small nucl	Snhg18
6703	Snord123	2.149	0.465	1.000	0.306	1.000	0.423	0.000	1.104	0.000	66835	Snord123	chr15	-	NR_02857	.	snoRNA	.	small nucl	Snord123
6704	Sema5a	0.388	1.202	0.467	0.005	0.307	0.092	4.426	3.062	3.327	20356	Sema5a	chr15	+	NM_0091	P17297	protein_cc	5930434A	sema dom	Sema5a
6705	Sdc2	0.166	1.262	0.209	0.000	0.123	0.003	10.992	8.397	8.732	15529	Sdc2	chr15	+	NM_0083	P7044	protein_cc	4833414L(	syndecan	Sdc2
6706	Cpq	0.537	0.913	0.491	0.001	0.450	0.025	9.645	8.750	8.619	54381	Cpq	chr15	+	NM_0187	P18718	protein_cc	1190003P	carboxype	Cpq
6707	1700084J1	2.778	0.578	1.605	0.046	0.223	0.556	2.779	4.253	3.461	73486	1700084J1	chr15	-	NR_03360	.	misc_RNA	.	RIKEN cDN	1700084J1
6708	4930592A	0.528	1.606	0.848	0.015	0.033	0.323	5.318	4.396	5.080	75880	4930592A	chr15	-	NR_04507	.	lncRNA	.	RIKEN cDN	4930592A(
6709	Tspyl5	10.068	2.428	24.444	0.003	0.001	0.000	0.002	3.334	4.614	239364	Tspyl5	chr15	-	NM_0010:	P17342	protein_cc	6330530B	testis-spe	Tspyl5
6710	Mtdh	1.232	0.984	1.213	0.004	0.715	0.108	10.970	11.272	11.249	67154	Mtdh	chr15	+	NM_0260	P18870	protein_cc	2610103J:	metadher	Mtdh
6711	Laptm4b	1.428	0.818	1.168	0.003	0.107	0.538	8.619	9.132	8.842	114128	Laptm4b	chr15	+	NM_0335	P1490	protein_cc	C330023F	lysosomal	Laptm4b
6712	Matn2	0.394	1.501	0.591	0.001	0.030	0.076	4.053	2.709	3.295	17181	Matn2	chr15	+	NM_0167	P258	protein_cc	Crtm2| m	matrilin 2	Matn2
6713	9430069I(	1.000	1.000	1.000	1.000	1.000	1.000	0.000	0.000	0.000	77358	9430069I(	chr15	-	NM_0012:	P9630	protein_cc	.	RIKEN cDN	9430069I0
6714	Gm12191	1.183	1.668	1.974	0.364	0.006	0.040	7.128	7.370	8.109	666899	Gm12191	chr15	-	NR_02810	.	misc_RNA	OTTMUSG	predicted	Gm12191
6715	Rpl30	1.305	1.044	1.363	0.080	0.644	0.081	9.159	9.543	9.606	19946	Rpl30	chr15	-	NM_0090:	P23266	protein_cc	.	ribosomal	Rpl30
6716	Erich5	1.572	0.636	1.000	0.304	1.000	0.423	0.000	0.653	0.000	239368	Erich5	chr15	+	NM_1734	P13427	protein_cc	.	glutamate	Erich5
6717	Hrsp12	0.404	0.975	0.394	0.001	0.913	0.033	13.447	12.140	12.105	.	Hrsp12	chr15	-	NM_0082	P25806	.	.	.	Hrsp12
6718	Pop1	1.396	0.671	0.937	0.003	0.020	0.804	6.196	6.677	6.102	67724	Pop1	chr15	+	NM_0263	P11085	protein_cc	4932434G	processing	Pop1
6719	Nipal2	0.758	0.619	0.469	0.624	0.559	0.062	2.167	1.767	1.074	223473	Nipal2	chr15	-	NM_1454	P16494	protein_cc	9330161F(	NIPA-like	Nipal2
6720	4930413F:	1.000	1.000	1.000	1.000	1.000	1.000	0.000	0.000	0.000	71637	4930413F:	chr15	+	NR_04588	.	lncRNA	.	RIKEN cDN	4930413F2
6721	Kcns2	1.000	1.000	1.000	1.000	1.000	1.000	0.000	0.000	0.000	16539	Kcns2	chr15	+	NM_1813	P8012	protein_cc	E130006J:	K+ voltage	Kcns2

6722	Stk3	1.730	0.637	1.103	0.094	0.129	0.420	6.685	7.475	6.826	56274	Stk3	chr15	-	NM_0196	P4140	protein_cc	0610042I0	serine/thr	Stk3
6723	Osr2	1.596	1.393	2.224	0.295	0.315	0.084	0.000	0.675	1.154	107587	Osr2	chr15	+	NM_0540	P3619	protein_cc	5430409I1	odd-skipp	Osr2
6724	BC048602	1.000	1.000	1.000	1.000	1.000	1.000	0.000	0.000	0.000	10012626	BC048602	chr15	-	NR_04528	.	lncRNA	.	cDNA seq	BC048602
6725	Vps13b	0.562	1.107	0.623	0.000	0.082	0.013	7.155	6.324	6.472	666173	Vps13b	chr15	+	NM_1771	P21702	protein_cc	1810042B	vacuolar p	Vps13b
6726	Cox6c	1.018	1.184	1.206	0.718	0.126	0.279	11.914	11.939	12.184	12864	Cox6c	chr15	-	NM_0530	P24590	protein_cc	.	cytochrom	Cox6c
6727	Rgs22	1.000	1.000	1.000	1.000	1.000	1.000	0.000	0.000	0.000	626596	Rgs22	chr15	-	NM_0011	P3443	protein_cc	.	regulator	Rgs22
6728	Fbxo43	1.000	1.000	1.000	1.000	1.000	1.000	0.000	0.000	0.000	78803	Fbxo43	chr15	-	NM_0010	P15986	protein_cc	4930533G	F-box prot	Fbxo43
6729	Polr2k	1.257	1.139	1.432	0.019	0.007	0.054	7.845	8.175	8.363	17749	Polr2k	chr15	+	NM_0231	P11039	protein_cc	ABC10-alp	polymeras	Polr2k
6730	Spag1	1.989	0.582	1.157	0.095	0.009	0.841	1.682	2.675	1.893	26942	Spag1	chr15	+	NM_0120	P8868	protein_cc	tpis	sperm ass	Spag1
6731	Rnf19a	1.603	0.742	1.190	0.142	0.230	0.670	7.855	8.536	8.106	30945	Rnf19a	chr15	-	NM_0139	P2935	protein_cc	AA032313	ring finger	Rnf19a
6732	Ankrd46	0.900	0.968	0.872	0.257	0.296	0.414	8.136	7.985	7.938	68839	Ankrd46	chr15	-	NM_1751	P182	protein_cc	1110054N	ankyrin re	Ankrd46
6733	Snx31	0.552	0.990	0.547	0.182	1.000	0.423	0.871	0.015	0.000	66696	Snx31	chr15	-	NM_0257	P8225	protein_cc	4631426E	sorting ne	Snx31
6734	Pabpc1	1.331	1.099	1.462	0.037	0.278	0.012	11.356	11.768	11.904	18458	Pabpc1	chr15	-	NM_0087	P3016	protein_cc	ePAB | PAI	poly(A) bi	Pabpc1
6735	Ywhaz	1.607	0.883	1.419	0.002	0.041	0.075	9.892	10.576	10.397	22631	Ywhaz	chr15	-	NM_0012	P13032	protein_cc	1110013I1	tyrosine 3	Ywhaz
6736	Zfp706	0.533	1.087	0.580	0.002	0.414	0.047	9.600	8.694	8.815	68036	Zfp706	chr15	-	NM_0265	P7416	protein_cc	3110006P	zinc finger	Zfp706
6737	Grhl2	0.252	1.365	0.344	0.000	0.209	0.024	2.035	0.048	0.498	252973	Grhl2	chr15	+	NM_0264	P20151	protein_cc	0610015A	grainyhea	Grhl2
6738	Ncald	0.116	0.929	0.107	0.000	0.672	0.000	8.379	5.268	5.161	52589	Ncald	chr15	-	NM_0011	P553	protein_cc	AI848120 |	neurocalci	Ncald
6739	Gm15941	1.000	1.000	1.000	1.000	1.000	1.000	0.000	0.000	0.000	10050353	Gm15941	chr15	-	NR_04528	.	lncRNA	OTTMUSG	predicted	Gm15941
6740	4930447A	1.000	1.000	1.000	1.000	1.000	1.000	0.000	0.000	0.000	74892	4930447A	chr15	+	NM_0291	P2192	protein_cc	.	RIKEN cDN	4930447A
6741	Rrm2b	1.574	0.846	1.331	0.011	0.128	0.043	4.251	4.906	4.664	382985	Rrm2b	chr15	-	NM_1994	P9641	protein_cc	p53R2	ribonucle	Rrm2b
6742	Ubr5	1.055	1.124	1.185	0.292	0.018	0.074	8.710	8.787	8.956	70790	Ubr5	chr15	-	NM_0010	P26844	protein_cc	Edd | Edd1	ubiquitin	Ubr5
6743	Odf1	1.000	1.000	1.000	1.000	1.000	1.000	0.000	0.000	0.000	18285	Odf1	chr15	+	NM_0087	P7770	protein_cc	.	outer den	Odf1
6744	Klf10	0.796	1.751	1.393	0.000	0.033	0.325	7.909	7.579	8.387	21847	Klf10	chr15	-	NM_0012	P23300	protein_cc	AI115143 |	Kruppel-li	Klf10
6745	Azin1	0.864	1.206	1.042	0.259	0.070	0.846	9.301	9.091	9.360	54375	Azin1	chr15	-	NM_0013	P15842	protein_cc	1700085L	antizyme i	Azin1
6746	2310043O	1.000	1.000	1.000	1.000	1.000	1.000	0.000	0.000	0.000	69679	2310043O	chr15	-	NR_04575	.	lncRNA	.	RIKEN cDN	2310043O
6747	Atp6v1c1	1.131	0.985	1.114	0.323	0.894	0.072	8.378	8.556	8.534	66335	Atp6v1c1	chr15	+	NM_0254	P14535	protein_cc	1700025B	ATPase, H	Atp6v1c1
6748	Borg	1.000	1.000	1.000	1.000	1.000	1.000	0.000	0.000	0.000	10546312	Borg	chr15	-	NR_13120	.	lncRNA	.	BMP/OP-r	Borg
6749	Baalc	2.716	0.783	2.128	0.002	0.565	0.551	0.754	2.195	1.843	118452	Baalc	chr15	+	NM_0806	P7070	protein_cc	2810457D	brain and	Baalc
6750	Fzd6	1.256	0.794	0.997	0.399	0.416	0.986	5.122	5.450	5.117	14368	Fzd6	chr15	+	NM_0011	P26265	protein_cc	Fz6	frizzled cla	Fzd6
6751	Cthrc1	1.000	1.000	1.000	1.000	1.000	1.000	0.000	0.000	0.000	68588	Cthrc1	chr15	+	NM_0267	P5125	protein_cc	1110014B	collagen t	Cthrc1
6752	Slc25a32	0.844	1.360	1.148	0.772	0.569	0.513	5.691	5.446	5.890	69906	Slc25a32	chr15	-	NM_1724	P14455	protein_cc	2610043O	solute car	Slc25a32
6753	Dcaf13	1.347	1.109	1.494	0.153	0.543	0.226	8.982	9.412	9.561	223499	Dcaf13	chr15	+	NM_1986	P8712	protein_cc	Gm83 | W	DDB1 and	Dcaf13
6754	Rims2	2.215	4.296	9.516	0.365	0.014	0.055	0.364	1.512	3.614	116838	Rims2	chr15	+	NM_0012	P12974	protein_cc	2810036I1	regulating	Rims2
6755	Dcstamp	0.552	0.990	0.547	0.182	1.000	0.423	0.871	0.015	0.000	75766	Dcstamp	chr15	+	NM_0012	P17368	protein_cc	4833414I0	dendrocyt	Dcstamp
6756	Dpys	0.109	0.365	0.040	0.002	0.008	0.038	11.331	8.131	6.676	64705	Dpys	chr15	-	NM_0227	P15680	protein_cc	1200017I1	dihydropy	Dpys
6757	Gm16291	0.102	1.233	0.125	0.004	0.270	0.070	3.393	0.096	0.399	10050307	Gm16291	chr15	+	NR_04578	.	lncRNA	OTTMUSG	predicted	Gm16291
6758	Lrp12	1.843	0.657	1.211	0.001	0.003	0.304	5.216	6.098	5.492	239393	Lrp12	chr15	-	NM_1728	P686	protein_cc	AI848829 |	low densit	Lrp12
6759	AU022793	1.005	1.439	1.446	0.000	0.182	0.423	0.000	0.008	0.532	105976	AU022793	chr15	+	NR_04571	.	lncRNA	.	expressed	AU022793
6760	9330182O	1.000	1.000	1.000	1.000	1.000	1.000	0.000	0.000	0.000	328531	9330182O	chr15	+	NM_0012	P7004	protein_cc	.	RIKEN cDN	9330182O
6761	Gm16294	1.000	1.000	1.000	1.000	1.000	1.000	0.000	0.000	0.000	432945	Gm16294	chr15	-	NR_04618	.	lncRNA	EG432945	predicted	Gm16294
6762	Zfpm2	0.370	2.194	0.811	0.000	0.054	0.558	3.251	1.815	2.948	22762	Zfpm2	chr15	+	NM_0117	P296	protein_cc	B330005D	zinc finger	Zfpm2
6763	Oxr1	0.351	2.249	0.789	0.004	0.010	0.463	8.192	6.680	7.849	170719	Oxr1	chr15	+	NM_0011	P9582	protein_cc	2210416C	oxidation	Oxr1
6764	Abra	1.005	1.439	1.446	0.000	0.182	0.423	0.000	0.008	0.532	223513	Abra	chr15	-	NM_1754	P20349	protein_cc	C130068O	actin-bind	Abra
6765	Angpt1	0.305	1.102	0.337	0.043	0.833	0.237	4.552	2.841	2.981	11600	Angpt1	chr15	-	NM_0096	P26273	protein_cc	1110046O	angiopoie	Angpt1
6766	Rspo2	0.716	0.995	0.712	0.182	1.000	0.423	0.490	0.008	0.000	239405	Rspo2	chr15	-	NM_1728	P26079	protein_cc	2610028F	R-spondin	Rspo2
6767	Eif3e	1.480	0.961	1.422	0.041	0.738	0.322	9.743	10.309	10.251	16341	Eif3e	chr15	-	NM_0083	P12313	protein_cc	48kDa | eI	eukaryotic	Eif3e
6768	Gm10373	1.000	1.000	1.000	1.000	1.000	1.000	0.000	0.000	0.000	10003872	Gm10373	chr15	-	NR_04606	.	lncRNA	ENSMUSG	predicted	Gm10373
6769	Emc2	1.217	1.247	1.517	0.334	0.069	0.201	8.239	8.522	8.840	66736	Emc2	chr15	+	NM_0257	P19450	protein_cc	4921531G	ER membr	Emc2
6770	Tmem74	1.000	1.000	1.000	1.000	1.000	1.000	0.000	0.000	0.000	239408	Tmem74	chr15	-	NM_1755	P9437	protein_cc	AA549547	transmem	Tmem74
6771	Trhr	1.000	1.000	1.000	1.000	1.000	1.000	0.000	0.000	0.000	22045	Trhr	chr15	+	NM_0136	P9999	protein_cc	TRH-R1	thyrotropi	Trhr
6772	Nudcd1	2.019	0.597	1.206	0.000	0.001	0.442	7.526	8.539	7.795	67429	Nudcd1	chr15	-	NM_0261	P13069	protein_cc	4921532K	NudC dom	Nudcd1
6773	Eny2	1.577	0.813	1.283	0.000	0.042	0.255	10.279	10.936	10.638	223527	Eny2	chr15	+	NM_1750	P1407	protein_cc	1810057B	enhancer	Eny2
6774	Pkhd1l1	0.197	0.962	0.190	0.038	1.000	0.199	2.397	0.056	0.000	192190	Pkhd1l1	chr15	+	NM_1386	P6665	protein_cc	AB055648	polycystic	Pkhd1l1
6775	Ebag9	0.647	1.323	0.856	0.005	0.067	0.481	8.023	7.394	7.798	55960	Ebag9	chr15	+	NM_0194	P15347	protein_cc	AI835379 |	estrogen	Ebag9
6776	Sybu	3.264	2.301	7.511	0.128	0.070	0.103	2.256	3.963	5.165	319613	Sybu	chr15	-	NM_0012	P5502	protein_cc	Golsyn	syntabulin	Sybu
6777	2310069G	0.485	1.143	0.555	0.205	0.765	0.510	1.742	0.699	0.891	69659	2310069G	chr15	+	NR_04030	.	lncRNA	.	RIKEN cDN	2310069G
6778	A930017N	1.000	1.000	1.000	1.000	1.000	1.000	0.000	0.000	0.000	239410	A930017N	chr15	+	NR_03360	.	misc_RNA	Gm305	RIKEN cDN	A930017N
6779	Kcnv1	1.000	1.000	1.000	1.000	1.000	1.000	0.000	0.000	0.000	67498	Kcnv1	chr15	-	NM_0262	P14911	protein_cc	2700023A	potassium	Kcnv1
6780	1700022A	1.000	1.000	1.000	1.000	1.000	1.000	0.000	0.000	0.000	71120	1700022A	chr15	+	NR_04550	.	lncRNA	4933413D	RIKEN cDN	1700022A
6781	4930523O	0.451	0.986	0.444	0.182	1.000	0.423	1.170	0.021	0.000	74726	4930523O	chr15	+	NR_04045	.	lncRNA	4921524H	RIKEN cDN	4930523O
6782	4930548G	1.000	1.000	1.000	1.000	1.000	1.000	0.000	0.000	0.000	75281	4930548G	chr15	+	NR_04581	.	lncRNA	.	RIKEN cDN	4930548G
6783	Csmd3	0.716	0.995	0.712	0.182	1.000	0.423	0.490	0.008	0.000	239420	Csmd3	chr15	-	NM_0010	P1452	protein_cc	4930500N	CUB and S	Csmd3
6784	Trps1	0.564	1.076	0.606	0.092	0.750	0.263	4.325	3.497	3.603	83925	Trps1	chr15	-	NM_0320	P22326	protein_cc	AI115454 |	trichorhin	Trps1
6785	Gm19303	1.000	1.000	1.000	1.000	1.000	1.000	0.000	0.000	0.000	10050264	Gm19303	chr15	-	NR_04509	.	lncRNA	.	predicted	Gm19303
6786	Eif3h	0.773	1.375	1.064	0.000	0.012	0.622	10.283	9.913	10.373	68135	Eif3h	chr15	-	NM_0806	P4804	protein_cc	1110008A	eukaryotic	Eif3h
6787	Utp23	3.345	0.820	2.742	0.003	0.209	0.066	6.281	8.023	7.736	78581	Utp23	chr15	+	NM_0301	P318	protein_cc	1700010I2	UTP23 sm	Utp23
6788	Rad21	1.023	1.030	1.054	0.816	0.704	0.744	9.322	9.355	9.397	19357	Rad21	chr15	-	NM_0090	P24686	protein_cc	mKIAA007	RAD21 co	Rad21
6789	Aard	0.113	0.435	0.049	0.026	1.000	0.146	4.351	1.202	0.000	239435	Aard	chr15	+	NM_1755	P16062	protein_cc	A5D3 | AV	alanine an	Aard
6790	Slc30a8	0.390	1.748	0.682	0.183	0.190	0.690	1.395	0.038	0.843	239436	Slc30a8	chr15	+	NM_1728	P9668	protein_cc	C820002P	solute car	Slc30a8
6791	Med30	2.329	0.506	1.179	0.160	0.228	0.770	4.994	6.214	5.232	69790	Med30	chr15	+	NM_0272	P16539	protein_cc	1810038N	mediator	Med30
6792	Ext1	1.094	0.936	1.024	0.517	0.695	0.915	8.987	9.117	9.021	14042	Ext1	chr15	-	NM_0101	P10111	protein_cc	AA409028	exostoses	Ext1
6793	Samd12	1.000	1.000	1.000	1.000	1.000	1.000	0.000	0.000	0.000	320679	Samd12	chr15	-	NM_1772	P1687	protein_cc	4930594I0	sterile alp	Samd12
6794	Tnfrsf11b	3.447	0.384	1.323	0.011	0.005	0.791	3.053	4.839	3.458	18383	Tnfrsf11b	chr15	-	NM_0087	P15839	protein_cc	OCIF | Opg	tumor nec	Tnfrsf11b
6795	Colec10	0.320	0.706	0.226	0.001	0.236	0.033	7.807	6.162	5.660	239447	Colec10	chr15	+	NM_1734	P14524	protein_cc	CL-L1	collectin s	Colec10
6796	Mal2	1.774	0.661	1.172	0.000	0.002	0.255	8.859	9.685	9.087	105853	Mal2	chr15	+	NM_1789	P4509	protein_cc	AI461653	mal, T cell	Mal2
6797	Nov	1.210	2.143	2.592	0.633	0.054	0.125	0.422	0.697	1.796	18133	Nov	chr15	+	NM_0109	P15225	protein_cc	C130088N	nephrobla	Nov
6798	Enpp2	0.280	5.295	1.484	0.000	0.001	0.183	9.367	7.532	9.936	18606	Enpp2	chr15	-	NM_0012	P10996	protein_cc	ATX | E-NP	ectonucle	Enpp2
6799	Taf2	1.901	1.311	2.491	0.081	0.324	0.130	5.870	6.797	7.187	319944	Taf2	chr15	-	NM_0010	P19325	protein_cc	150kDa | 4	TATA-box	Taf2
6800	Dscc1	3.947	0.544	2.149	0.118	0.335	0.423	0.001	1.982	1.105	72107	Dscc1	chr15	-	NM_1830	P25490	protein_cc	2010006I0	DNA replic	Dscc1
6801	Gm9920	0.394	0.496	0.196	0.080	0.441	0.050	3.110	1.768	0.756	10050379	Gm9920	chr15	-	NR_04509	.	lncRNA	ENSMUSG	predicted	Gm9920
6802	Deptor	0.118	1.812	0.213	0.000	0.046	0.013	12.004	8.919	9.776	97998	Deptor	chr15	+	NM_1454	P12457	protein_cc	Depdc6	DEP doma	Deptor
6803	Col14a1	0.640	0.575	0.368	0.000	0.008	0.018	8.190	7.546	6.747	12818	Col14a1	chr15	+	NM_1812	P21378	protein_cc	5730412L	collagen, t	Col14a1
6804	Mrpl13	0.728	1.086	0.790	0.004	0.332	0.097	8.551	8.093	8.212	68537	Mrpl13	chr15	-	NM_0267	P7570	protein_cc	1110002D	mitochond	Mrpl13
6805	Mtbp	4.092	0.420	1.720	0.034	0.091	0.412	2.580	4.612	3.362	105837	Mtbp	chr15	+	NM_0011	P13826	protein_cc	AI429604 |	Mdm2, tra	Mtbp
6806	Sntb1	2.312	0.437	1.011	0.024	0.009	0.982	0.774	1.983	0.789	20649	Sntb1	chr15	-	NM_0166	P17594	protein_cc	.	syntrophin	Sntb1
6807	Has2	1.000	1.000	1.000	1.000	1.000	1.000	0.000	0.000	0.000	15117	Has2	chr15	-	NM_0082	P3879	protein_cc	.	hyalurona	Has2
6808	Has2os	1.000	1.000	1.000	1.000	1.000	1.000	0.000	0.000	0.000	594843	Has2os	chr15	+	NR_00287	.	lncRNA	EG594843	hyalurona	Has2os
6809	Slc22a22	1.000	1.000	1.000	1.000	1.000	1.000	0.000	0.000	0.000	210463	Slc22a22	chr15	-	NM_1723	P21555	protein_cc	oat-pg	solute car	Slc22a22
6810	Zhx2	0.906	0.914	0.828	0.445	0.561	0.334	6.150	6.007	5.878	387609	Zhx2	chr15	+	NM_1994	P8070	protein_cc	Afr-1 | Afr	zinc finger	Zhx2
6811	Derl1	0.869	1.246	1.082	0.030	0.014	0.280	10.271	10.068	10.385	67819	Derl1	chr15	-	NM_0242	P13758	protein_cc	1110021N	Der1-like	Derl1
6812	Tbc1d31	0.304	0.454	0.138	0.128	0.331	0.253	4.129	2.412	1.273	210544	Tbc1d31	chr15	+	NM_0010	P16211	protein_cc	4B3 | D33C	TBC1 dom	Tbc1d31

6813	Fam83a	78.301	1.669	130.711	0.012	0.106	0.046	1.005	7.296	8.035	239463	Fam83a	chr15	+	NM_1738	P6575	protein_cc	.	family wit	Fam83a
6814	9130401M	2.266	1.372	3.108	0.002	0.015	0.009	7.454	8.634	9.090	75758	9130401M	chr15	-	NM_0294	P13541	protein_cc	AI849328	RIKEN cDN	9130401M
6815	Zhx1	1.162	0.752	0.874	0.016	0.005	0.202	8.469	8.685	8.274	22770	Zhx1	chr15	-	NM_0010	P20826	protein_cc	.	zinc finger	Zhx1
6816	Atad2	1.287	1.021	1.315	0.094	0.910	0.436	4.807	5.172	5.203	70472	Atad2	chr15	-	NM_0274	P5578	protein_cc	2610509G	ATPase fa	Atad2
6817	Wdyhv1	1.140	0.547	0.623	0.350	0.027	0.090	5.913	6.101	5.231	76773	Wdyhv1	chr15	+	NM_0297	P1706	protein_cc	2410187C	WDYHV m	Wdyhv1
6818	Fbxo32	0.285	2.000	0.571	0.002	0.033	0.173	6.288	4.479	5.480	67731	Fbxo32	chr15	-	NM_0263	P22694	protein_cc	4833442G	F-box prot	Fbxo32
6819	Klhl38	1.000	1.000	1.000	1.000	1.000	1.000	0.000	0.000	0.000	268807	Klhl38	chr15	-	NM_1777	P13849	protein_cc	8230402K	kelch-like	Klhl38
6820	Anxa13	0.115	3.089	0.355	0.005	0.252	0.236	3.893	0.771	2.398	69787	Anxa13	chr15	-	NM_0272	P17676	protein_cc	1810034H	annexin A	Anxa13
6821	D15Ertd62	0.843	0.884	0.745	0.604	0.663	0.487	8.883	8.637	8.459		D15Ertd62	chr15	+	NM_1459	P1545				D15Ertd62
6822	Tmem65	1.779	1.133	2.016	0.057	0.405	0.094	5.861	6.692	6.873	74868	Tmem65	chr15	-	NM_1752	P7974	protein_cc	2610029O	transmem	Tmem65
6823	Trmt12	3.228	0.644	2.081	0.002	0.035	0.122	4.806	6.497	5.863	68260	Trmt12	chr15	+	NM_0266	P25888	protein_cc	4632406N	tRNA met	Trmt12
6824	Rnf139	1.032	0.997	1.029	0.642	0.979	0.894	8.282	8.328	8.323	75841	Rnf139	chr15	+	NM_1752	P11790	protein_cc	4930555P	ring finger	Rnf139
6825	Tatdn1	0.846	0.891	0.754	0.493	0.226	0.529	5.026	4.784	4.618	69694	Tatdn1	chr15	-	NM_1751	P15139	protein_cc	2310079P	TatD DNa	Tatdn1
6826	Ndufb9	0.678	1.080	0.732	0.000	0.078	0.004	12.148	11.588	11.698	66218	Ndufb9	chr15	+	NM_0231	P16146	protein_cc	1190008J1	NADH deh	Ndufb9
6827	Mtss1	0.773	1.021	0.789	0.041	0.042	0.233	10.338	9.967	9.997	211401	Mtss1	chr15	-	NM_1448	P15707	protein_cc	2310003N	metastasis	Mtss1
6828	Zfp572	1.000	1.000	1.000	1.000	1.000	1.000	0.000	0.000	0.000	10041683	Zfp572	chr15	+	NR_04561	.	misc_RNA	4930472F	zinc finger	Zfp572
6829	Sqle	2.813	1.313	3.694	0.000	0.002	0.005	8.819	10.311	10.704	20775	Sqle	chr15	+	NM_0092	P21821	protein_cc	AI323792	squalene e	Sqle
6830	E430025E	1.239	0.777	0.963	0.356	0.307	0.851	7.417	7.726	7.362	223593	E430025E	chr15	-	NM_1535	P17857	protein_cc	AL022848	RIKEN cDN	E430025E
6831	Nsmce2	1.011	0.870	0.880	0.895	0.059	0.418	7.260	7.276	7.076	68501	Nsmce2	chr15	+	NM_0011	P12834	protein_cc	1110014D	NSE2/MM	Nsmce2
6832	Trib1	1.556	0.418	0.651	0.004	0.001	0.101	10.540	11.178	9.922	211770	Trib1	chr15	+	NM_1445	P20101	protein_cc	A530090C	tribbles ps	Trib1
6833	Gm19510	1.972	0.675	1.331	0.019	0.235	0.736	0.779	1.758	1.192	10050302	Gm19510	chr15	-	NR_04507	.	lncRNA	.	predicted	Gm19510
6834	4933412E	0.716	0.995	0.712	0.182	1.000	0.423	0.490	0.008	0.000	71088	4933412E	chr15	-	NM_0276	P2763	protein_cc	.	RIKEN cDN	4933412E
6835	Fam84b	0.625	0.834	0.522	0.065	0.095	0.198	6.864	6.187	5.925	399603	Fam84b	chr15	-	NM_0011	P2690	protein_cc	BF642811	family wit	Fam84b
6836	9930014A	1.169	0.328	0.383	0.748	0.007	0.517	2.151	2.376	0.766	320469	9930014A	chr15	+	NR_03069	.	lncRNA	.	RIKEN cDN	9930014A
6837	A1bg	0.571	0.120	0.069	0.000	0.000	0.004	6.397	5.589	2.535	117586	A1bg	chr15	-	NM_0010	P17517	protein_cc	C44	alpha-1-B	A1bg
6838	D030024E	1.000	1.000	1.000	1.000	1.000	1.000	0.000	0.000	0.000	432956	D030024E	chr15	-	NR_04035	.	lncRNA	.	RIKEN cDN	D030024E
6839	Myc	1.581	0.837	1.323	0.522	0.779	0.462	4.740	5.401	5.144	17869	Myc	chr15	+	NM_0011	P26346	protein_cc	AU016757	myelocyto	Myc
6840	Pvt1	8.510	0.452	3.849	0.024	0.116	0.251	1.240	4.329	3.185	19296	Pvt1	chr15	+	NR_00336	.	lncRNA	Ayu21-84	plasmacyt	Pvt1
6841	H2afy3	1.000	1.000	1.000	1.000	1.000	1.000	0.000	0.000	0.000	67552	H2afy3	chr15	-	NR_00352	.	misc_RNA	4933432H	H2A histo	H2afy3
6842	Gm20740	1.000	1.000	1.000	1.000	1.000	1.000	0.000	0.000	0.000	432958	Gm20740	chr15	-	NR_04527	.	lncRNA	.	predicted	Gm20740
6843	Gsdmc	1.000	1.000	1.000	1.000	1.000	1.000	0.000	0.000	0.000	83492	Gsdmc	chr15	-	NM_0313	P22206	protein_cc	Gsdmc1|	gasdermin	Gsdmc
6844	1700065I1	0.669	0.995	0.666	0.181	1.000	0.423	0.587	0.008	0.000	78462	1700065I1	chr15	+	NR_04031	.	misc_RNA	1700084L	RIKEN cDN	1700065I1
6845	Gsdmc2	1.000	1.000	1.000	1.000	1.000	1.000	0.000	0.000	0.000	331063	Gsdmc2	chr15	-	NM_1779	P6780	protein_cc	9130023H	gasdermin	Gsdmc2
6846	Gsdmcl1	1.000	1.000	1.000	1.000	1.000	1.000	0.000	0.000	0.000	74236	Gsdmcl1	chr15	+	NR_10805	.	misc_RNA	1700022L	gasdermin	Gsdmcl1
6847	Gsdmc3	1.000	1.000	1.000	1.000	1.000	1.000	0.000	0.000	0.000	270328	Gsdmc3	chr15	-	NM_1831	P22407	protein_cc	9930109F	gasdermin	Gsdmc3
6848	Gsdmcl2	1.000	1.000	1.000	1.000	1.000	1.000	0.000	0.000	0.000	665769	Gsdmcl2	chr15	+	NR_10805	.	misc_RNA	4921501E	gasdermin	Gsdmcl2
6849	Gsdmc4	1.821	0.366	0.665	0.460	1.000	0.423	0.588	1.452	0.000	74548	Gsdmc4	chr15	-	NM_0289	P13975	protein_cc	9030605I0	gasdermin	Gsdmc4
6850	Gsdmcl-ps	1.000	1.000	1.000	1.000	1.000	1.000	0.000	0.000	0.000	74460	Gsdmcl-ps	chr15	+	NR_02941	.	misc_RNA	4933426G	gasdermin	Gsdmcl-ps
6851	Fam49b	2.245	1.209	2.714	0.000	0.092	0.027	7.649	8.815	9.089	223601	Fam49b	chr15	-	NM_1448	P8697	protein_cc	0910001A	family wit	Fam49b
6852	Asap1	0.964	1.190	1.147	0.418	0.009	0.246	7.548	7.494	7.745	13196	Asap1	chr15	-	NM_0012	P16408	protein_cc	AV239055	ArfGAP wi	Asap1
6853	Adcy8	0.669	0.995	0.666	0.181	1.000	0.423	0.587	0.008	0.000	11514	Adcy8	chr15	-	NM_0012	P21499	protein_cc	AC8| AWC	adenylate	Adcy8
6854	Efr3a	0.876	0.883	0.773	0.431	0.336	0.336	9.492	9.300	9.121	76740	Efr3a	chr15	+	NM_1337	P1911	protein_cc	A130089N	EFR3 hom	Efr3a
6855	Oc90	1.000	1.000	1.000	1.000	1.000	1.000	0.000	0.000	0.000	18256	Oc90	chr15	-	NM_0109	P12175	protein_cc	oc95| Ocn	otoconin 9	Oc90
6856	Hhla1	1.000	1.000	1.000	1.000	1.000	1.000	0.000	0.000	0.000	654498	Hhla1	chr15	-	NM_0011	P11771	protein_cc	F930104E	HERV-H LT	Hhla1
6857	Kcnq3	1.005	1.405	1.412	0.000	0.182	0.423	0.000	0.008	0.498	110862	Kcnq3	chr15	-	NM_1529	P23615	protein_cc	.	potassium	Kcnq3
6858	Lrrc6	1.000	1.000	1.000	1.000	1.000	1.000	0.000	0.000	0.000	54562	Lrrc6	chr15	-	NM_0194	P16274	protein_cc	LRTP	leucine ric	Lrrc6
6859	Tmem71	6.694	1.764	11.810	0.000	0.000	0.001	2.713	5.456	6.275	213068	Tmem71	chr15	-	NM_1725	P8381	protein_cc	AI661017|	transmem	Tmem71
6860	Phf20l1	2.649	0.809	2.143	0.003	0.112	0.004	8.505	9.911	9.605	239510	Phf20l1	chr15	+	NM_0010	P25456	protein_cc	CGI-72| E	PHD finge	Phf20l1
6861	Tg	2.134	0.469	1.000	0.305	1.000	0.423	0.000	1.094	0.000	21819	Tg	chr15	+	NM_0093	P7364	protein_cc	cog| Tgn	thyroglob	Tg
6862	Sla	4.095	0.973	3.983	0.000	0.897	0.157	4.983	7.018	6.977	20491	Sla	chr15	-	NM_0010	P1378	protein_cc	Slap| Slap	src-like ad	Sla
6863	Wisp1	2.184	0.283	0.618	0.002	0.000	0.256	4.865	5.992	4.170	22402	Wisp1	chr15	+	NM_0188	P25677	protein_cc	AW14626	WNT1 ind	Wisp1
6864	Ndrg1	1.455	1.088	1.582	0.000	0.025	0.003	8.458	8.999	9.120	17988	Ndrg1	chr15	-	NM_0086	P9551	protein_cc	CAP43| C	N-myc do	Ndrg1
6865	St3gal1	1.321	0.685	0.905	0.074	0.026	0.476	9.476	9.877	9.332	20442	St3gal1	chr15	-	NM_0091	P20453	protein_cc	5330418N	ST3 beta-g	St3gal1
6866	1700012I1	1.010	1.825	1.844	0.000	0.182	0.423	0.000	0.015	0.883	69334	1700012I1	chr15	-	NR_04514	.	lncRNA	.	RIKEN cDN	1700012I1
6867	Zfat	1.332	0.415	0.553	0.564	0.117	0.548	1.338	1.752	0.483	380993	Zfat	chr15	-	NM_0011	P21774	protein_cc	Gm922| Z	zinc finger	Zfat
6868	Khdrbs3	0.534	1.300	0.694	0.018	0.269	0.030	7.663	6.758	7.137	13992	Khdrbs3	chr15	+	NM_0101	P11381	protein_cc	Etle| Salp	KH domai	Khdrbs3
6869	4930504C	1.000	1.000	1.000	1.000	1.000	1.000	0.000	0.000	0.000	75076	4930504C	chr15	+	NR_13098	.	lncRNA	.	RIKEN cDN	4930504C
6870	Gm19782	1.000	1.000	1.000	1.000	1.000	1.000	0.000	0.000	0.000	10050358	Gm19782	chr15	+	NR_04507	.	lncRNA	.	predicted	Gm19782
6871	Fam135b	1.000	1.000	1.000	1.000	1.000	1.000	0.000	0.000	0.000	70363	Fam135b	chr15	-	NM_1778	P22515	protein_cc	1700010C	family wit	Fam135b
6872	Col22a1	0.726	1.425	1.034	0.187	0.186	0.943	0.476	0.015	0.525	69700	Col22a1	chr15	-	NM_0271	P10950	protein_cc	2310067L	collagen, t	Col22a1
6873	Kcnk9	1.000	1.000	1.000	1.000	1.000	1.000	0.000	0.000	0.000	223604	Kcnk9	chr15	-	NM_0010	P17040	protein_cc	Task3	potassium	Kcnk9
6874	Trappc9	0.253	1.955	0.496	0.000	0.028	0.070	6.640	4.660	5.627	76510	Trappc9	chr15	-	NM_0296	P21298	protein_cc	1810044A	trafficking	Trappc9
6875	Peg13	0.339	3.339	1.131	0.072	0.154	0.892	4.734	3.172	4.911	353342	Peg13	chr15	-	NR_00286	.	lncRNA		paternally	Peg13
6876	Chrac1	1.409	1.414	1.992	0.003	0.000	0.009	7.405	7.900	8.400	93696	Chrac1	chr15	+	NM_0530	P2345	protein_cc	2410152E	chromatin	Chrac1
6877	Ago2	1.161	0.796	0.924	0.550	0.375	0.759	6.649	6.865	6.535	239528	Ago2	chr15	-	NM_1531	P19677	protein_cc	1110029L	argonaute	Ago2
6878	Ptk2	1.511	1.238	1.871	0.026	0.074	0.015	6.786	7.382	7.690	14083	Ptk2	chr15	-	NM_0079	P18431	protein_cc	Fadk| FAK	PTK2 prot	Ptk2
6879	Dennd3	0.964	1.114	1.074	0.945	0.561	0.929	4.668	4.615	4.771	105841	Dennd3	chr15	+	NM_0010	P12835	protein_cc	AI447457|	DENN/MA	Dennd3
6880	Slc45a4	1.315	0.786	1.034	0.222	0.438	0.941	6.237	6.632	6.284	106068	Slc45a4	chr15	-	NM_0011	P13601	protein_cc	9330175B	solute car	Slc45a4
6881	Gpr20	1.000	1.000	1.000	1.000	1.000	1.000	0.000	0.000	0.000	239530	Gpr20	chr15	-	NM_1733	P1267	protein_cc	A430106B	G protein-	Gpr20
6882	Ptp4a3	1.142	0.544	0.621	0.715	0.121	0.456	5.624	5.816	4.937	19245	Ptp4a3	chr15	+	NM_0011	P15684	protein_cc	AV088979	protein ty	Ptp4a3
6883	Mroh5	1.000	1.000	1.000	1.000	1.000	1.000	0.000	0.000	0.000	268816	Mroh5	chr15	-	NM_0010	P26458	protein_cc	Gm628	maestro h	Mroh5
6884	Adgrb1	1.572	0.636	1.000	0.304	1.000	0.423	0.000	0.653	0.000	107831	Adgrb1	chr15	+	NM_1749	P10300	protein_cc	B830018M	adhesion G	Adgrb1
6885	Mroh4	1.000	1.000	1.000	1.000	1.000	1.000	0.000	0.000	0.000	69439	Mroh4	chr15	-	NM_0011	P9804	protein_cc	1700016M	maestro h	Mroh4
6886	Arc	0.716	0.995	0.712	0.182	1.000	0.423	0.490	0.008	0.000	11838	Arc	chr15	-	NM_0187	P24442	protein_cc	Arc3.1| ar	activity re	Arc
6887	Jrk	2.811	1.354	3.806	0.002	0.257	0.136	2.851	4.342	4.780	16469	Jrk	chr15	-	NM_0084	P18246	protein_cc	.	jerky	Jrk
6888	4933427E	1.015	2.326	2.360	0.000	0.182	0.423	0.000	0.021	1.239	66769	4933427E	chr15	+	NR_03319	.	lncRNA	.	RIKEN cDN	4933427E1
6889	Psca	1.000	1.000	1.000	1.000	1.000	1.000	0.000	0.000	0.000	72373	Psca	chr15	+	NM_0282	P8109	protein_cc	2210408B	prostate s	Psca
6890	Them6	0.383	1.629	0.624	0.013	0.346	0.459	5.107	3.724	4.428	223626	Them6	chr15	+	NM_1986	P17836	protein_cc	4930572J	thioestera	Them6
6891	Slurp1	1.000	1.000	1.000	1.000	1.000	1.000	0.000	0.000	0.000	57277	Slurp1	chr15	-	NM_0205	P3908	protein_cc	1110021N	secreted L	Slurp1
6892	Lypd2	1.000	1.000	1.000	1.000	1.000	1.000	0.000	0.000	0.000	68311	Lypd2	chr15	-	NM_0266	P16492	protein_cc	0610005K	Ly6/Plaur	Lypd2
6893	2300005B	1.000	1.000	1.000	1.000	1.000	1.000	0.000	0.000	0.000		2300005B	chr15	-	NM_0010	P25292	.			2300005B
6894	Lynx1	2.776	0.589	1.636	0.004	0.081	0.430	2.950	4.423	3.661	23936	Lynx1	chr15	-	NM_0118	P11601	protein_cc	AI838844|	Ly6/neuro	Lynx1
6895	Ly6d	4.252	0.814	3.461	0.001	0.413	0.212	4.810	6.899	6.602	17068	Ly6d	chr15	-	NM_0107	P16543	protein_cc	Ly-61| Ly6	lymphocyt	Ly6d
6896	D730001G	3.915	0.545	2.134	0.305	0.514	0.080	0.001	1.970	1.095	78725	D730001G	chr15	-	NR_02783	.	lncRNA	.	RIKEN cDN	D730001G
6897	Ly6k	3.173	0.766	2.432	0.001	0.216	0.231	1.654	3.320	2.936	76486	Ly6k	chr15	-	NM_0296	P13241	protein_cc	2410015A	lymphocyt	Ly6k
6898	Gml	6.475	1.661	10.755	0.002	0.452	0.362	0.347	3.042	3.774	625599	Gml	chr15	-	NM_0011	P21705	protein_cc	EG625599	glycosylph	Gml
6899	Gml2	3.455	2.867	9.906	0.067	0.162	0.272	0.002	1.790	3.310	15202	Gml2	chr15	+	NM_0104	P24975	protein_cc	1700057K	glycosylph	Gml2
6900	Cyp11b1	1.000	1.000	1.000	1.000	1.000	1.000	0.000	0.000	0.000	110115	Cyp11b1	chr15	-	NM_0010	P2822	protein_cc	AA389061	cytochrom	Cyp11b1
6901	Cyp11b2	1.005	1.439	1.446	0.000	0.182	0.423	0.000	0.008	0.532	13072	Cyp11b2	chr15	-	NM_0099	P1567	protein_cc	Cpn2| Cyp	cytochrom	Cyp11b2
6902	2010109I0	1.572	0.636	1.000	0.304	1.000	0.423	0.000	0.653	0.000	67038	2010109I0	chr15	-	NM_0259	P17251	protein_cc	.	RIKEN cDN	2010109I0
6903	Ly6e	3.078	0.562	1.731	0.000	0.000	0.046	10.820	12.442	11.611	17069	Ly6e	chr15	+	NM_0011	P14057	protein_cc	Ly67| RIG	lymphocyt	Ly6e

6904	Ly6i	4.931	0.676	3.334	0.030	0.412	0.381	0.369	2.670	2.106	57248	Ly6i	chr15	-	NM_0204	P2446	protein_cc	AI789751|	lymphocy	Ly6i
6905	Ly6a	14.231	0.552	7.857	0.001	0.010	0.002	6.811	10.642	9.785	110454	Ly6a	chr15	-	NM_0012	P14256	protein_cc	Ly-6A.2| L	lymphocy	Ly6a
6906	Ly6c1	8.439	1.103	9.307	0.019	0.767	0.122	3.405	6.482	6.623	17067	Ly6c1	chr15	-	NM_0012	P41542	protein_cc	AA682074	lymphocy	Ly6c1
6907	Ly6c2	23.816	0.443	10.541	0.027	0.120	0.008	2.979	7.553	6.377	10004154	Ly6c2	chr15	-	NM_0010	P17452	protein_cc	Ly-6C.2| L	lymphocy	Ly6c2
6908	Ly6f	5.099	1.797	9.163	0.019	0.118	0.106	0.589	2.939	3.785	17071	Ly6f	chr15	+	NM_0085	P18611	protein_cc	.	lymphocy	Ly6f
6909	9030619P	0.083	0.297	0.025	0.000	0.034	0.000	10.128	6.544	4.792	105892	9030619P	chr15	-	NR_10804	.	misc_RNA	.	RIKEN cDN	9030619P
6910	Ly6h	1.000	1.000	1.000	1.000	1.000	1.000	0.000	0.000	0.000	23934	Ly6h	chr15	-	NM_0118	P18210	protein_cc	.	lymphocy	Ly6h
6911	Gpihbp1	0.227	1.990	0.451	0.000	0.001	0.004	8.334	6.192	7.184	68453	Gpihbp1	chr15	+	NM_0267	P20494	protein_cc	1110002J1	GPI-ancho	Gpihbp1
6912	Zfp41	1.125	0.855	0.962	0.230	0.289	0.866	4.216	4.385	4.160	22701	Zfp41	chr15	+	NM_0010	P14332	protein_cc	CTfin92| Z	zinc finger	Zfp41
6913	Top1mt	0.860	0.987	0.849	0.008	0.897	0.354	7.153	6.935	6.917	72960	Top1mt	chr15	-	NM_0284	P7108	protein_cc	2900052H	DNA topo	Top1mt
6914	Rhpn1	1.000	1.000	1.000	1.000	1.000	1.000	0.000	0.000	0.000	14787	Rhpn1	chr15	+	NM_0081	P3098	protein_cc	BB023497	rhophilin,	Rhpn1
6915	Mafa	1.000	1.000	1.000	1.000	1.000	1.000	0.000	0.000	0.000	378435	Mafa	chr15	-	NM_1943	P26592	protein_cc	RIPE3b1	v-maf mus	Mafa
6916	Zc3h3	0.943	1.187	1.119	0.820	0.357	0.801	4.098	4.013	4.261	223642	Zc3h3	chr15	-	NM_1721	P13713	protein_cc	BC049953	zinc finger	Zc3h3
6917	Gsdmd	4.195	0.734	3.080	0.001	0.048	0.037	7.046	9.115	8.669	69146	Gsdmd	chr15	+	NM_0269	P14060	protein_cc	1810036L(	gasdermin	Gsdmd
6918	Mroh6	0.473	0.869	0.411	0.314	0.726	0.498	1.777	0.697	0.495	223645	Mroh6	chr15	-	NM_0012	P26719	protein_cc	Gm19570	maestro h	Mroh6
6919	Naprt	0.252	1.352	0.341	0.000	0.191	0.027	9.002	7.013	7.448	223646	Naprt	chr15	-	NM_1726	P1450	protein_cc	9130210N	nicotinate	Naprt
6920	Eef1d	0.813	1.648	1.339	0.009	0.022	0.276	9.474	9.175	9.895	66656	Eef1d	chr15	-	NM_0012	P8020	protein_cc	EF-1-delta	eukaryotic	Eef1d
6921	Tigd5	0.685	1.251	0.857	0.182	0.365	0.672	4.854	4.309	4.631	105734	Tigd5	chr15	+	NM_1786	P17435	protein_cc	AA409802	tigger tran	Tigd5
6922	Pycrl	0.528	1.254	0.662	0.000	0.314	0.225	7.309	6.388	6.715	66194	Pycrl	chr15	-	NM_0254	P10303	protein_cc	1110058B	pyrroline-	Pycrl
6923	Tsta3	1.120	1.406	1.574	0.060	0.019	0.099	7.797	7.960	8.451	22122	Tsta3	chr15	-	NM_0312	P23366	protein_cc	AI256181|	tissue spe	Tsta3
6924	Zfp623	0.636	1.257	0.800	0.000	0.021	0.066	6.852	6.199	6.529	78834	Zfp623	chr15	+	NM_0301	P18097	protein_cc	2610029D	zinc finger	Zfp623
6925	Zfp707	0.722	0.824	0.595	0.023	0.167	0.014	7.787	7.316	7.037	69020	Zfp707	chr15	+	NM_0010	P16743	protein_cc	1500031N	zinc finger	Zfp707
6926	Ccdc166	0.322	2.209	0.712	0.096	0.158	0.540	3.852	2.219	3.363	223648	Ccdc166	chr15	-	NM_1460	P1051	protein_cc	2410075B	coiled-coil	Ccdc166
6927	Mapk15	0.048	2.921	0.141	0.000	0.006	0.013	4.521	0.146	1.692	332110	Mapk15	chr15	+	NM_1779	P18583	protein_cc	BC048082	mitogen-a	Mapk15
6928	Fam83h	1.222	0.761	0.930	0.579	0.494	0.794	5.557	5.845	5.451	105732	Fam83h	chr15	-	NM_0011	P14362	protein_cc	AA409316	family wit	Fam83h
6929	Scrib	0.726	1.253	0.909	0.222	0.393	0.200	6.311	5.848	6.173	105782	Scrib	chr15	-	NM_1340	P26356	protein_cc	AI118201|	scribbled	Scrib
6930	Puf60	1.144	1.082	1.238	0.108	0.259	0.012	9.776	9.969	10.083	67959	Puf60	chr15	-	NM_0011	P9868	protein_cc	2410104I1	poly-U bin	Puf60
6931	Nrbp2	0.566	1.353	0.765	0.064	0.333	0.481	8.125	7.303	7.739	223649	Nrbp2	chr15	-	NM_1448	P18532	protein_cc	BC011468	nuclear re	Nrbp2
6932	Eppk1	3.472	1.164	4.040	0.000	0.643	0.238	6.646	8.442	8.660	223650	Eppk1	chr15	-	NM_1448	P15372	protein_cc	6230424I1	epiplakin	Eppk1
6933	BC024139	0.091	2.054	0.188	0.002	0.133	0.054	5.020	1.569	2.607	271278	BC024139	chr15	-	NM_0011	P15543	protein_cc	6230424I1	cDNA seq	BC024139
6934	Plec	2.183	0.944	2.060	0.000	0.041	0.004	8.176	9.302	9.219	18810	Plec	chr15	-	NM_2013	P4139	protein_cc	AA591047	plectin	Plec
6935	Parp10	4.738	0.607	2.876	0.001	0.019	0.061	6.447	8.691	7.971	671535	Parp10	chr15	-	NM_0011	P3583	protein_cc	.	poly (ADP-	Parp10
6936	Grina	2.686	1.065	2.861	0.000	0.334	0.012	9.693	11.118	11.209	66168	Grina	chr15	+	NM_0231	P16345	protein_cc	1110025J1	glutamate	Grina
6937	Gm10872	35.718	0.693	24.750	0.000	0.001	0.001	3.359	8.517	7.988	.	Gm10872	chr15	-	NR_04574	.	.	.	.	Gm10872
6938	Spatc1	1.000	1.000	1.000	1.000	1.000	1.000	0.000	0.000	0.000	74281	Spatc1	chr15	+	NM_0288	P16917	protein_cc	1700084J2	spermatog	Spatc1
6939	Smpd5	0.804	1.190	0.957	0.415	0.489	0.879	9.122	8.807	9.058	10050391	Smpd5	chr15	+	NM_0011	P15147	protein_cc	ENSMUSG	sphingom	Smpd5
6940	Oplah	0.812	1.173	0.952	0.401	0.502	0.870	9.214	8.913	9.143	75475	Oplah	chr15	-	NM_1531	P19901	protein_cc	1700010G	5-oxoproli	Oplah
6941	Exosc4	1.961	1.248	2.448	0.002	0.026	0.000	7.059	8.030	8.350	109075	Exosc4	chr15	+	NM_1753	P8106	protein_cc	1110039I0	exosome c	Exosc4
6942	Gpaa1	0.875	1.082	0.947	0.023	0.606	0.823	8.578	8.385	8.500	14731	Gpaa1	chr15	+	NM_0103	P18514	protein_cc	C80044| n	GPI ancho	Gpaa1
6943	Cyc1	0.607	1.263	0.767	0.044	0.105	0.366	10.305	9.585	9.922	66445	Cyc1	chr15	+	NM_0255	P22430	protein_cc	2610002H	cytochrom	Cyc1
6944	Sharpin	1.034	0.712	0.737	0.646	0.010	0.227	7.622	7.671	7.181	106025	Sharpin	chr15	-	NM_0253	P24314	protein_cc	0610041B	SHANK-as	Sharpin
6945	Maf1	0.811	1.422	1.153	0.028	0.065	0.585	7.480	7.177	7.685	68877	Maf1	chr15	+	NM_0011	P12357	protein_cc	1110068E	MAF1 hon	Maf1
6946	Hgh1	1.925	0.950	1.829	0.006	0.648	0.075	4.966	5.911	5.837	59053	Hgh1	chr15	+	NM_0215	P5105	protein_cc	Brp16| D1	HGH1 hon	Hgh1
6947	Tssk5	1.000	1.000	1.000	1.000	1.000	1.000	0.000	0.000	0.000	73542	Tssk5	chr15	-	NM_1830	P26560	protein_cc	1700091F	testis-spe	Tssk5
6948	Mroh1	1.007	1.088	1.095	0.966	0.593	0.456	6.645	6.655	6.777	223658	Mroh1	chr15	+	NM_1754	P11523	protein_cc	D330001F	maestro h	Mroh1
6949	Bop1	1.356	0.963	1.305	0.056	0.637	0.332	7.122	7.561	7.506	12181	Bop1	chr15	-	NM_0134	P3321	protein_cc	AU020183	block of p	Bop1
6950	Scx	2.716	0.745	2.024	0.231	0.607	0.287	0.754	2.196	1.771	20289	Scx	chr15	+	NM_1988	P5238	protein_cc	BB114693	scleraxis	Scx
6951	Hsf1	1.024	0.879	0.900	0.749	0.473	0.750	6.165	6.199	6.013	15499	Hsf1	chr15	+	NM_0082	P22142	protein_cc	AA960185	heat shocl	Hsf1
6952	Dgat1	0.958	0.593	0.568	0.627	0.008	0.044	7.981	7.919	7.165	13350	Dgat1	chr15	-	NM_0100	P201	protein_cc	ARAT| C7!	diacylglyc	Dgat1
6953	Scrt1	1.000	1.000	1.000	1.000	1.000	1.000	0.000	0.000	0.000	170729	Scrt1	chr15	-	NM_1308	P7499	protein_cc	.	scratch fa	Scrt1
6954	Fbxl6	0.916	0.691	0.633	0.060	0.003	0.021	6.189	6.062	5.528	30840	Fbxl6	chr15	-	NM_0139	P2018	protein_cc	AU021795	F-box and	Fbxl6
6955	Slc52a2	1.405	1.164	1.636	0.211	0.551	0.230	5.327	5.818	6.037	52710	Slc52a2	chr15	+	NM_0296	P15320	protein_cc	2010003P	solute car	Slc52a2
6956	Adck5	0.287	1.612	0.462	0.001	0.013	0.051	7.699	5.897	6.585	268822	Adck5	chr15	+	NM_1729	P4681	protein_cc	A230108P	aarF doma	Adck5
6957	Cpsf1	0.566	1.177	0.666	0.000	0.144	0.056	8.670	7.849	8.085	94230	Cpsf1	chr15	-	NM_0011	P26556	protein_cc	.	cleavage a	Cpsf1
6958	Slc39a4	0.693	1.350	0.936	0.003	0.009	0.182	9.554	9.026	9.459	72027	Slc39a4	chr15	-	NM_0280	P22223	protein_cc	1600025H	solute car	Slc39a4
6959	Vps28	0.955	1.107	1.057	0.575	0.263	0.749	9.900	9.833	9.980	66914	Vps28	chr15	-	NM_0258	P20900	protein_cc	1110014J0	vacuolar p	Vps28
6960	Tonsl	0.517	1.293	0.668	0.015	0.428	0.431	2.752	1.799	2.169	72749	Tonsl	chr15	-	NM_1830	P12235	protein_cc	2810439M	tonsoku-li	Tonsl
6961	Cyhr1	0.420	1.335	0.560	0.000	0.046	0.013	9.020	7.769	8.185	54151	Cyhr1	chr15	-	NM_0193	P3848	protein_cc	1110031M	cysteine a	Cyhr1
6962	Kifc2	0.070	1.659	0.116	0.004	0.150	0.076	4.630	0.796	1.526	16581	Kifc2	chr15	+	NM_0106	P5282	protein_cc	.	kinesin far	Kifc2
6963	Foxh1	1.000	1.000	1.000	1.000	1.000	1.000	0.000	0.000	0.000	14106	Foxh1	chr15	-	NM_0079	P6189	protein_cc	fast-1| fas	forkhead l	Foxh1
6964	Ppp1r16a	0.469	1.371	0.643	0.004	0.017	0.107	6.755	5.663	6.118	73062	Ppp1r16a	chr15	+	NM_0333	P1059	protein_cc	2900084E	protein ph	Ppp1r16a
6965	Gpt	0.148	1.081	0.160	0.001	0.753	0.028	10.945	8.188	8.301	76282	Gpt	chr15	+	NM_1828	P5260	protein_cc	ALT| ALT1	glutamic p	Gpt
6966	Mfsd3	0.180	2.534	0.457	0.005	0.032	0.124	6.917	4.447	5.789	69572	Mfsd3	chr15	+	NM_0271	P21158	protein_cc	2310010G	major faci	Mfsd3
6967	Recql4	0.331	1.983	0.656	0.054	0.187	0.297	4.558	2.962	3.950	79456	Recql4	chr15	-	NM_0582	P26880	protein_cc	RecQ4	RecQ prot	Recql4
6968	Lrrc14	0.959	1.100	1.054	0.554	0.088	0.617	7.696	7.636	7.773	223664	Lrrc14	chr15	+	NM_1454	P16987	protein_cc	E130306I0	leucine ric	Lrrc14
6969	Lrrc24	1.428	0.466	0.666	0.562	1.000	0.423	0.587	1.101	0.000	378937	Lrrc24	chr15	-	NM_1981	P15321	protein_cc	6430402H	leucine ric	Lrrc24
6970	C030006K	0.360	1.880	0.676	0.027	0.022	0.364	7.890	6.416	7.326	223665	C030006K	chr15	-	NM_1454	P22603	protein_cc	1110033I1	RIKEN cDN	C030006K
6971	Arhgap39	2.151	0.407	0.876	0.020	0.037	0.823	3.503	4.608	3.313	223666	Arhgap39	chr15	-	NM_0011	P17714	protein_cc	9530053N	Rho GTPas	Arhgap39
6972	Zfp251	2.264	0.676	1.531	0.003	0.048	0.197	3.864	5.042	4.478	71591	Zfp251	chr15	-	NM_0010	P9567	protein_cc	9130001M	zinc finger	Zfp251
6973	Zfp7	0.668	1.199	0.801	0.128	0.503	0.273	4.152	3.571	3.832	223669	Zfp7	chr15	+	NM_1459	P12331	protein_cc	KRAB20| ł	zinc finger	Zfp7
6974	Commd5	0.838	1.439	1.206	0.087	0.007	0.050	7.812	7.557	8.082	66398	Commd5	chr15	+	NM_0255	P13180	protein_cc	2310065H	COMM do	Commd5
6975	Rpl8	1.109	1.325	1.469	0.029	0.008	0.070	11.371	11.520	11.926	26961	Rpl8	chr15	+	NM_0120	P1470	protein_cc	.	ribosomal	Rpl8
6976	Zfp647	0.325	1.340	0.436	0.283	0.594	0.586	2.788	1.168	1.590	239546	Zfp647	chr15	-	NM_0011	P6483	protein_cc	6030449J2	zinc finger	Zfp647
6977	1110038F	2.884	0.559	1.612	0.002	0.011	0.094	7.252	8.780	7.941	117171	1110038F	chr15	+	NM_0540	P18385	protein_cc	.	RIKEN cDN	1110038F1
6978	Mb	0.251	0.970	0.243	0.000	1.000	0.001	2.041	0.044	0.000	17189	Mb	chr15	-	NM_0011	P21822	protein_cc	AI325109	myoglobin	Mb
6979	Apol6	6.317	0.406	2.563	0.025	0.082	0.237	0.001	2.661	1.359	71939	Apol6	chr15	+	NM_0011	P733	protein_cc	2310076O	apolipopro	Apol6
6980	Rbfox2	0.589	1.052	0.619	0.021	0.748	0.124	7.241	6.476	6.549	93686	Rbfox2	chr15	-	NM_0012	P23940	protein_cc	Fbm2| Fxł	RNA bindi	Rbfox2
6981	1700109K	1.000	1.000	1.000	1.000	1.000	1.000	0.000	0.000	0.000	74303	1700109K	chr15	+	NR_10803	.	lncRNA	4933425G	RIKEN cDN	1700109K2
6982	Apol7a	0.032	1.380	0.044	0.000	0.452	0.002	11.047	6.067	6.532	75761	Apol7a	chr15	-	NM_0294	P17750	protein_cc	9130022K	apolipopro	Apol7a
6983	Apol9a	1.796	0.260	0.468	0.002	0.002	0.156	8.479	9.324	7.382	223672	Apol9a	chr15	-	NM_0011	P20639	protein_cc	.	apolipopro	Apol9a
6984	Apol7b	0.207	0.964	0.200	0.000	1.000	0.000	2.324	0.053	0.000	278679	Apol7b	chr15	-	NM_0010	P18686	protein_cc	.	apolipopro	Apol7b
6985	Apol10a	1.000	1.000	1.000	1.000	1.000	1.000	0.000	0.000	0.000	245282	Apol10a	chr15	+	NM_1777	P23455	protein_cc	9030421J0	apolipopro	Apol10a
6986	Apol11a	1.000	1.000	1.000	1.000	1.000	1.000	0.000	0.000	0.000	626615	Apol11a	chr15	+	NM_0011	P24205	protein_cc	EG626615	apolipopro	Apol11a
6987	Apol7c	0.428	1.322	0.566	0.075	0.523	0.208	2.381	1.158	1.560	108956	Apol7c	chr15	-	NM_1753	P25736	protein_cc	2210421G	apolipopro	Apol7c
6988	1700025B	3.354	1.555	5.214	0.301	0.515	0.268	0.001	1.747	2.384	76409	1700025B	chr15	-	NR_04031	.	lncRNA	.	RIKEN cDN	1700025B1
6989	Apol10b	33.254	0.156	5.193	0.000	0.000	0.138	1.231	6.286	3.607	328561	Apol10b	chr15	-	NM_1778	P26351	protein_cc	9130218O	apolipopro	Apol10b
6990	Gm8221	0.583	1.867	1.089	0.040	0.000	0.738	1.962	1.184	2.085	666661	Gm8221	chr15	+	NR_03357	.	pseudogei	666661	predicted	Gm8221
6991	Apol11b	1.627	0.569	0.926	0.001	0.004	0.777	1.831	2.533	1.719	328563	Apol11b	chr15	-	NM_0011	P15026	protein_cc	A330102K	apolipopro	Apol11b
6992	Apol7e	0.677	0.455	0.308	0.398	1.000	0.099	1.699	1.136	0.000	666348	Apol7e	chr15	+	NM_0011	P21199	protein_cc	ENSMUSG	apolipopro	Apol7e
6993	Apol9b	1.434	0.343	0.493	0.002	0.006	0.210	9.707	10.228	8.686	71898	Apol9b	chr15	+	NM_1737	P24945	protein_cc	2310016F	apolipopro	Apol9b
6994	Apol8	1.000	1.000	1.000	1.000	1.000	1.000	0.000	0.000	0.000	239552	Apol8	chr15	-	NM_0010	P23435	protein_cc	9830006J2	apolipopro	Apol8

6995	Myh9	1.802	0.670	1.208	0.005	0.001	0.402	9.912	10.762	10.185	17886	Myh9	chr15	-	NM_0224	P16781	protein_cc	Fltn| Myh	myosin, he	Myh9
6996	Txn2	0.595	0.930	0.553	0.000	0.380	0.029	10.946	10.197	10.092	56551	Txn2	chr15	-	NM_0199	P4392	protein_cc	2510006J1	thioredoxi	Txn2
6997	Foxred2	0.570	0.835	0.476	0.334	0.762	0.426	1.950	1.140	0.880	239554	Foxred2	chr15	-	NM_0011	P9843	protein_cc	A430097D	FAD-depe	Foxred2
6998	Eif3d	1.363	1.123	1.531	0.129	0.464	0.124	9.275	9.722	9.889	55944	Eif3d	chr15	-	NM_0187	P4702	protein_cc	66/67kDa	eukaryotic	Eif3d
6999	Cacng2	1.005	1.405	1.412	0.000	0.182	0.423	0.000	0.008	0.498	12300	Cacng2	chr15	-	NM_0075	P24329	protein_cc	AW06099	calcium ch	Cacng2
7000	Ift27	0.592	1.280	0.758	0.039	0.278	0.499	5.904	5.148	5.504	67042	Ift27	chr15	-	NM_0259	P11040	protein_cc	2600013G	intraflagel	Ift27
7001	Pvalb	0.552	0.990	0.547	0.182	1.000	0.423	0.871	0.015	0.000	19293	Pvalb	chr15	-	NM_0136	P14430	protein_cc	Parv| PV|	parvalbum	Pvalb
7002	Ncf4	5.107	0.916	4.679	0.001	0.675	0.117	3.465	5.818	5.691	17972	Ncf4	chr15	+	NM_0086	P19413	protein_cc	AI451400|	neutrophi	Ncf4
7003	Csf2rb2	3.762	0.860	3.235	0.103	0.722	0.094	3.431	5.343	5.125	12984	Csf2rb2	chr15	-	NM_0077	P8670	protein_cc	AIC2A| Be	colony stir	Csf2rb2
7004	Csf2rb	6.876	0.615	4.228	0.001	0.021	0.017	5.378	8.160	7.458	12983	Csf2rb	chr15	+	NM_0077	P5100	protein_cc	AI848964|	colony stir	Csf2rb
7005	Tex33	1.000	1.000	1.000	1.000	1.000	1.000	0.000	0.000	0.000	73376	Tex33	chr15	-	NM_0285	P17110	protein_cc	1700061J0	testis expr	Tex33
7006	Tst	0.423	0.952	0.402	0.007	0.413	0.084	8.677	7.435	7.364	22117	Tst	chr15	-	NM_0094	P11311	protein_cc	Rhodanese	thiosulfate	Tst
7007	Mpst	0.340	1.459	0.496	0.000	0.071	0.074	9.225	7.667	8.212	246221	Mpst	chr15	+	NM_0011	P20959	protein_cc	Mst	mercaptop	Mpst
7008	Kctd17	1.934	1.621	3.135	0.089	0.287	0.278	4.956	5.907	6.604	72844	Kctd17	chr15	+	NM_0012	P12276	protein_cc	2900008M	potassium	Kctd17
7009	Tmprss6	0.205	1.173	0.240	0.000	0.319	0.009	10.988	8.699	8.930	71753	Tmprss6	chr15	-	NM_0279	P19027	protein_cc	1300008A	transmem	Tmprss6
7010	Il2rb	1.952	0.401	0.783	0.067	0.014	0.692	4.227	5.192	3.875	16185	Il2rb	chr15	-	NM_0083	P22113	protein_cc	CD122| IL-	interleukin	Il2rb
7011	C1qtnf6	0.891	2.808	2.503	0.772	0.020	0.130	3.426	3.261	4.750	72709	C1qtnf6	chr15	-	NM_0012	P19119	protein_cc	2810036M	C1q and tu	C1qtnf6
7012	Sstr3	1.000	1.000	1.000	1.000	1.000	1.000	0.000	0.000	0.000	20607	Sstr3	chr15	-	NM_0092	P4037	protein_cc	Smstr-3| S	somatostat	Sstr3
7013	Rac2	4.943	0.556	2.750	0.000	0.002	0.053	6.309	8.614	7.768	19354	Rac2	chr15	-	NM_0090	P4061	protein_cc	AI323801|	RAS-relate	Rac2
7014	Cyth4	1.342	1.275	1.711	0.082	0.325	0.289	4.792	5.217	5.567	72318	Cyth4	chr15	+	NM_0281	P21879	protein_cc	2510004M	cytohesin	Cyth4
7015	Elfn2	1.000	1.000	1.000	1.000	1.000	1.000	0.000	0.000	0.000	207393	Elfn2	chr15	-	NM_1831	P18465	protein_cc	6330514E	leucine ric	Elfn2
7016	Mfng	0.193	7.135	1.380	0.001	0.023	0.545	3.583	1.213	4.048	17305	Mfng	chr15	-	NM_0085	P11407	protein_cc	AW54656	MFNG O-fu	Mfng
7017	Card10	0.418	0.827	0.346	0.006	0.441	0.069	6.885	5.628	5.354	105844	Card10	chr15	-	NM_1308	P13643	protein_cc	AI449026|	caspase re	Card10
7018	Cdc42ep1	1.167	1.364	1.592	0.232	0.000	0.097	6.774	6.997	7.445	104445	Cdc42ep1	chr15	+	NM_0272	P3199	protein_cc	1810058K	CDC42 eff	Cdc42ep1
7019	Lgals2	1.306	1.803	2.354	0.272	0.005	0.046	5.805	6.190	7.040	107753	Lgals2	chr15	-	NM_0256	P17757	protein_cc	2200008F	lectin, gala	Lgals2
7020	Gga1	1.348	0.905	1.219	0.071	0.414	0.103	7.037	7.468	7.323	106039	Gga1	chr15	+	NM_1459	P1218	protein_cc	4930406E	golgi asso	Gga1
7021	Sh3bp1	0.797	1.905	1.519	0.674	0.211	0.675	3.030	2.703	3.632	20401	Sh3bp1	chr15	+	NM_0091	P10681	protein_cc	3BP-1	SH3-doma	Sh3bp1
7022	Pdxp	1.328	0.115	0.153	0.396	0.000	0.265	3.073	3.482	0.367	57028	Pdxp	chr15	+	NM_0202	P8514	protein_cc	1600027H	pyridoxal	Pdxp
7023	Lgals1	0.715	1.823	1.303	0.009	0.010	0.252	6.610	6.126	6.992	16852	Lgals1	chr15	+	NM_0084	P23845	protein_cc	AA410090	lectin, gala	Lgals1
7024	Nol12	2.518	0.900	2.265	0.019	0.630	0.109	6.394	7.726	7.574	97961	Nol12	chr15	+	NM_1338	P6005	protein_cc	C78541| N	nucleolar	Nol12
7025	Triobp	1.043	1.169	1.219	0.725	0.172	0.109	7.057	7.117	7.342	110253	Triobp	chr15	+	NM_0010	P7456	protein_cc	AI428493|	TRIO and F	Triobp
7026	Gm10865	0.073	2.592	0.189	0.000	0.007	0.001	4.558	0.784	2.158	10003860	Gm10865	chr15	-	NR_04574	.	lncRNA	ENSMUSG	predicted	Gm10865
7027	H1f0	1.515	1.757	2.661	0.001	0.114	0.217	10.236	10.835	11.648	14958	H1f0	chr15	+	NM_0081	P23588	protein_cc	D130017D	H1 histone	H1f0
7028	Gcat	0.570	0.792	0.451	0.002	0.206	0.032	9.523	8.712	8.375	26912	Gcat	chr15	+	NM_0138	P7193	protein_cc	AI526977|	glycine C-a	Gcat
7029	Galr3	1.000	1.000	1.000	1.000	1.000	1.000	0.000	0.000	0.000	14429	Galr3	chr15	+	NM_0157	P21353	protein_cc	Galnr3	galanin re	Galr3
7030	Ankrd54	1.025	1.279	1.311	0.617	0.006	0.097	6.342	6.378	6.733	223690	Ankrd54	chr15	-	NM_1448	P11393	protein_cc	C730048E	ankyrin re	Ankrd54
7031	Eif3l	0.975	1.117	1.089	0.749	0.081	0.597	8.641	8.604	8.764	223691	Eif3l	chr15	+	NM_1451	P25689	protein_cc	0610011H	eukaryotic	Eif3l
7032	Micall1	1.351	0.535	0.723	0.124	0.022	0.100	6.740	7.174	6.271	27008	Micall1	chr15	+	NM_1774	P4330	protein_cc	AI448246|	microtubu	Micall1
7033	1700088E	0.911	0.368	0.335	0.739	0.000	0.242	2.338	2.204	0.762	27660	1700088E	chr15	-	NM_1385	P15336	protein_cc	ESTJ0827E	RIKEN cDN	1700088E
7034	Polr2f	0.878	1.166	1.023	0.123	0.064	0.830	8.382	8.194	8.415	69833	Polr2f	chr15	+	NM_0272	P25220	protein_cc	1810060D	polymeras	Polr2f
7035	Sox10	1.005	1.439	1.446	0.000	0.182	0.423	0.000	0.008	0.532	20665	Sox10	chr15	-	NM_0114	P22235	protein_cc	Dom| gt|	SRY (sex d	Sox10
7036	Gm10863	1.010	1.885	1.904	0.000	0.182	0.423	0.000	0.015	0.929	10004165	Gm10863	chr15	+	NR_02947	.	lncRNA	ENSMUSG	predicted	Gm10863
7037	Pick1	1.392	0.884	1.231	0.043	0.021	0.412	7.016	7.494	7.316	18693	Pick1	chr15	+	NM_0088	P24332	protein_cc	Prkcabp	protein int	Pick1
7038	Slc16a8	1.000	1.000	1.000	1.000	1.000	1.000	0.000	0.000	0.000	57274	Slc16a8	chr15	-	NM_0205	P20062	protein_cc	Mct3	solute carr	Slc16a8
7039	Baiap2l2	1.000	1.000	1.000	1.000	1.000	1.000	0.000	0.000	0.000	207495	Baiap2l2	chr15	-	NM_1775	P3751	protein_cc	BC048937	BAI1-asso	Baiap2l2
7040	Pla2g6	0.544	0.937	0.510	0.003	0.816	0.127	7.635	6.756	6.663	53357	Pla2g6	chr15	-	NM_0169	P21521	protein_cc	BB112799	phospholi	Pla2g6
7041	Maff	3.718	0.210	0.782	0.033	0.021	0.704	6.331	8.226	5.977	17133	Maff	chr15	+	NM_0010	P16646	protein_cc	.	v-maf mus	Maff
7042	Tmem184	1.211	0.816	0.988	0.248	0.147	0.970	7.072	7.347	7.054	223693	Tmem184	chr15	-	NM_0012	P26577	protein_cc	2610507A	transmem	Tmem184
7043	Csnk1e	1.758	0.871	1.531	0.007	0.210	0.215	5.644	6.458	6.259	27373	Csnk1e	chr15	-	NM_0137	P10545	protein_cc	AI426939|	casein kin	Csnk1e
7044	Kcnj4	1.581	0.879	1.390	0.301	0.744	0.423	0.000	0.661	0.476	16520	Kcnj4	chr15	-	NM_0084	P1788	protein_cc	IRK3| Kcn	potassium	Kcnj4
7045	Kdelr3	2.868	0.546	1.566	0.002	0.021	0.504	4.135	5.655	4.782	105785	Kdelr3	chr15	+	NM_1340	P24125	protein_cc	AI173274	KDEL (Lys-	Kdelr3
7046	Ddx17	0.834	0.475	0.396	0.016	0.021	0.096	9.292	9.030	7.955	67040	Ddx17	chr15	-	NM_1528	P13532	protein_cc	2610007K	DEAD (Asp	Ddx17
7047	Dmc1	1.015	2.326	2.360	0.000	0.182	0.423	0.000	0.021	1.239	13404	Dmc1	chr15	-	NM_0100	P23771	protein_cc	Dmc1h| Li	DNA meio	Dmc1
7048	Fam227a	0.644	0.925	0.596	0.029	0.887	0.514	3.455	2.821	2.708	75729	Fam227a	chr15	-	NM_0294	P19731	protein_cc	4933432B	family wit	Fam227a
7049	Cby1	0.543	0.866	0.470	0.009	0.537	0.026	5.982	5.102	4.894	73739	Cby1	chr15	+	NM_0286	P25155	protein_cc	1110014P	chibby hor	Cby1
7050	Tomm22	1.113	1.129	1.257	0.306	0.277	0.128	9.088	9.243	9.418	223696	Tomm22	chr15	+	NM_1726	P9227	protein_cc	2310047D	translocas	Tomm22
7051	Josd1	0.566	1.320	0.748	0.013	0.133	0.182	7.800	6.980	7.381	74158	Josd1	chr15	-	NM_0287	P6286	protein_cc	1300006C	Josephin d	Josd1
7052	Gtpbp1	0.885	1.029	0.911	0.140	0.619	0.499	8.479	8.304	8.345	14904	Gtpbp1	chr15	+	NM_0138	P21965	protein_cc	AL022987	GTP bindin	Gtpbp1
7053	Sun2	0.421	1.282	0.540	0.000	0.425	0.170	9.321	8.073	8.431	223697	Sun2	chr15	-	NM_1943	P10889	protein_cc	B230369L	Sad1 and U	Sun2
7054	Gm16576	0.506	0.621	0.314	0.037	1.000	0.007	1.671	0.688	0.000	10050419	Gm16576	chr15	+	NR_04506	.	lncRNA	.	predicted	Gm16576
7055	Dnal4	1.168	0.869	1.015	0.027	0.027	0.704	6.813	7.037	6.835	54152	Dnal4	chr15	-	NM_0174	P11944	protein_cc	D15Ertd42	dynein, ax	Dnal4
7056	Nptxr	3.720	0.668	2.484	0.004	0.160	0.263	1.334	3.230	2.647	73340	Nptxr	chr15	-	NM_0306	P14551	protein_cc	1200009K	neuronal p	Nptxr
7057	Npcd	3.720	0.668	2.484	0.004	0.160	0.263	1.334	3.230	2.647	504193	Npcd	chr15	-	NM_0010	P19731	protein_cc	Cbx6-Nptx	neuronal p	Npcd
7058	Cbx6	0.212	2.399	0.508	0.000	0.018	0.030	6.753	4.512	5.775	494448	Cbx6	chr15	-	NM_0287	P14807	protein_cc	.	chromobo	Cbx6
7059	D730005E	13.204	0.223	2.941	0.005	0.010	0.169	1.686	5.409	3.243	109361	D730005E	chr15	-	NR_03067	.	lncRNA	.	RIKEN cDN	D730005E
7060	Apobec3	5.720	0.380	2.176	0.000	0.001	0.139	4.751	7.267	5.873	80287	Apobec3	chr15	+	NM_0302	P5103	protein_cc	Apobec| A	apolipopro	Apobec3
7061	Cbx7	0.763	0.890	0.679	0.000	0.041	0.019	6.809	6.419	6.251	52609	Cbx7	chr15	-	NM_1448	P12141	protein_cc	1600014J0	chromobo	Cbx7
7062	Pdgfb	1.603	0.226	0.362	0.360	0.009	0.560	3.142	3.823	1.675	18591	Pdgfb	chr15	-	NM_0110	P10808	protein_cc	PDGF-B| S	platelet de	Pdgfb
7063	Rpl3	1.455	1.767	2.570	0.020	0.000	0.007	9.476	10.017	10.838	27367	Rpl3	chr15	-	NM_0137	P20020	protein_cc	F2| J1	ribosomal	Rpl3
7064	Snord83b	6.473	0.514	3.326	0.152	0.373	0.257	2.594	5.288	4.327	10030260	Snord83b	chr15	-	NR_02828	.	snoRNA	MBII-10	small nucl	Snord83b
7065	Snord43	2.134	0.469	1.000	0.305	1.000	0.423	0.000	1.094	0.000	10030260	Snord43	chr15	-	NR_02828	.	snoRNA	MBII-47	small nucl	Snord43
7066	Syngr1	0.486	4.112	1.998	0.191	0.099	0.515	1.101	0.060	2.099	20972	Syngr1	chr15	+	NM_0093	P7234	protein_cc	p29| R754	synaptogy	Syngr1
7067	Tab1	1.182	0.872	1.031	0.412	0.182	0.923	4.227	4.469	4.272	66513	Tab1	chr15	+	NM_0256	P5496	protein_cc	2310012M	TGF-beta a	Tab1
7068	Mgat3	0.299	1.720	0.515	0.182	0.193	0.582	1.787	0.047	0.830	17309	Mgat3	chr15	+	NM_0107	P17591	protein_cc	1110038J1	mannosid	Mgat3
7069	Mief1	0.948	1.050	0.995	0.091	0.105	0.712	7.290	7.213	7.283	239555	Mief1	chr15	+	NM_1787	P16876	protein_cc	A230016E	mitochonc	Mief1
7070	Atf4	2.291	1.158	2.652	0.000	0.288	0.075	10.565	11.761	11.972	11911	Atf4	chr15	+	NM_0012	P22789	protein_cc	Atf-4| C/A	activating	Atf4
7071	Rps19bp1	1.602	1.046	1.677	0.006	0.566	0.006	7.465	8.146	8.211	66538	Rps19bp1	chr15	-	NM_1751	P8641	protein_cc	2510038A	ribosomal	Rps19bp1
7072	Cacna1i	0.716	0.995	0.712	0.182	1.000	0.423	0.490	0.008	0.000	239556	Cacna1i	chr15	+	NM_0010	P8591	protein_cc	Ca(V)3.3	calcium ch	Cacna1i
7073	Enthd1	0.716	0.995	0.712	0.182	1.000	0.423	0.490	0.008	0.000	383075	Enthd1	chr15	-	NM_0011	P12287	protein_cc	Gm1242|	ENTH dom	Enthd1
7074	Grap2	1.544	0.107	0.165	0.552	1.000	0.000	2.601	3.228	0.000	17444	Grap2	chr15	+	NM_0012	P3765	protein_cc	Gads| GR/	GRB2-rela	Grap2
7075	Fam83f	0.296	5.282	1.566	0.159	0.027	0.584	2.511	0.757	3.158	213956	Fam83f	chr15	+	NM_1459	P24550	protein_cc	AW54498	family wit	Fam83f
7076	A430088P	0.699	2.146	1.500	0.343	0.078	0.434	1.223	0.707	1.808	106059	A430088P	chr15	-	NR_04531	.	lncRNA	.	RIKEN cDN	A430088P
7077	Tnrc6b	0.544	1.335	0.725	0.000	0.001	0.047	8.938	8.058	8.475	213988	Tnrc6b	chr15	+	NM_1448	P23956	protein_cc	2700090M	trinucleoti	Tnrc6b
7078	Adsl	1.662	1.237	2.055	0.002	0.002	0.012	6.667	7.400	7.707	11564	Adsl	chr15	+	NM_0096	P23695	protein_cc	Adl| Asl	adenylosu	Adsl
7079	Sgsm3	1.448	1.539	2.228	0.177	0.052	0.012	5.104	5.638	6.260	105835	Sgsm3	chr15	+	NM_1340	P20666	protein_cc	1810012I0	small G pr	Sgsm3
7080	Mkl1	3.198	0.986	3.153	0.000	0.668	0.000	4.828	6.505	6.485	223701	Mkl1	chr15	-	NM_1530	P24161	protein_cc	AMKL| Bs	MKL (meg	Mkl1
7081	4930483J1	1.000	1.000	1.000	1.000	1.000	1.000	0.000	0.000	0.000	67638	4930483J1	chr15	+	NR_01560	.	lncRNA	.	RIKEN cDN	4930483J1
7082	Mchr1	1.572	0.636	1.000	0.304	1.000	0.423	0.000	0.653	0.000	207911	Mchr1	chr15	+	NM_1451	P1289	protein_cc	AW04999	melanin-c	Mchr1
7083	8430426J0	3.514	0.813	2.858	0.179	0.733	0.345	0.382	2.196	1.898	78102	8430426J0	chr15	+	NR_07723	.	lncRNA	.	RIKEN cDN	8430426J0
7084	Slc25a17	0.729	1.247	0.909	0.118	0.082	0.717	8.439	7.983	8.302	20524	Slc25a17	chr15	-	NM_0113	P1387	protein_cc	34kDa| 47	solute carr	Slc25a17
7085	St13	0.717	0.786	0.563	0.001	0.077	0.064	10.541	10.061	9.713	70356	St13	chr15	-	NM_1337	P13766	protein_cc	1110007I0	suppressio	St13

7086	Xpnpep3	0.684	1.082	0.740	0.053	0.701	0.265	6.153	5.604	5.718	321003	Xpnpep3	chr15	+	NM_1773	P18181	protein_cc	APP3| E43	X-prolyl ar	Xpnpep3
7087	Dnajb7	1.000	1.000	1.000	1.000	1.000	1.000	0.000	0.000	0.000	57755	Dnajb7	chr15	-	NM_0213	P18341	protein_cc	4933424H	DnaJ heat	Dnajb7
7088	Rbx1	1.184	1.030	1.219	0.249	0.803	0.077	9.315	9.558	9.601	56438	Rbx1	chr15	+	NM_0197	P2724	protein_cc	1500002P	ring-box 1	Rbx1
7089	Ep300	1.636	0.751	1.228	0.001	0.015	0.230	7.868	8.578	8.164	328572	Ep300	chr15	+	NM_1778	P19141	protein_cc	A430090G	E1A bindir	Ep300
7090	L3mbtl2	1.120	1.203	1.347	0.284	0.238	0.355	7.152	7.315	7.582	214669	L3mbtl2	chr15	+	NR_11036	.	protein_cc	4732493N	l(3)mbt-lik	L3mbtl2
7091	Chadl	1.000	0.705	0.705	0.998	0.128	0.294	2.384	2.384	1.881	214685	Chadl	chr15	-	NM_0011	P40	protein_cc	AY100452	chondroa	Chadl
7092	Rangap1	1.219	1.140	1.390	0.012	0.262	0.227	9.028	9.314	9.503	19387	Rangap1	chr15	-	NM_0011	P9790	protein_cc	C79654| F	RAN GTPa	Rangap1
7093	Zc3h7b	0.744	0.962	0.716	0.093	0.830	0.098	7.400	6.973	6.918	20286	Zc3h7b	chr15	+	NM_0010	P9195	protein_cc	Gm627| n	zinc finger	Zc3h7b
7094	Tef	0.056	10.853	0.606	0.000	0.000	0.007	10.150	5.987	9.427	21685	Tef	chr15	+	NM_1534	P17695	protein_cc	2310028D	thyrotrop	Tef
7095	Tob2	0.579	1.262	0.730	0.000	0.015	0.076	9.669	8.880	9.216	57259	Tob2	chr15	-	NM_0205	P13891	protein_cc	2900090N	transduce	Tob2
7096	Phf5a	1.506	1.116	1.681	0.046	0.431	0.052	9.434	10.024	10.183	68479	Phf5a	chr15	-	NM_0267	P11348	protein_cc	1110007B	PHD finge	Phf5a
7097	Aco2	0.417	0.968	0.403	0.032	0.909	0.135	10.728	9.465	9.418	11429	Aco2	chr15	+	NM_0806	P20704	protein_cc	Aco-2| Ac	aconitase	Aco2
7098	Polr3h	0.567	1.028	0.583	0.090	0.902	0.268	10.202	9.383	9.423	78929	Polr3h	chr15	-	NM_0302	P5523	protein_cc	5031409G	polymeras	Polr3h
7099	Csdc2	0.504	0.990	0.499	0.181	1.000	0.423	1.002	0.015	0.000	105859	Csdc2	chr15	+	NM_1454	P8499	protein_cc	AI415250|	cold shock	Csdc2
7100	Pmm1	0.466	2.683	1.249	0.019	0.005	0.045	4.538	3.435	4.859	29858	Pmm1	chr15	-	NM_0138	P3425	protein_cc	C77612	phosphom	Pmm1
7101	1700029P	0.716	0.995	0.712	0.182	1.000	0.423	0.490	0.008	0.000	66346	1700029P	chr15	+	NM_0255	P24261	protein_cc	.	RIKEN cDN	1700029P
7102	Desi1	1.015	1.121	1.138	0.642	0.152	0.381	8.697	8.718	8.883	28075	Desi1	chr15	-	NM_1340	P3117	protein_cc	AI427858|	desumoyl	Desi1
7103	Xrcc6	0.338	1.949	0.659	0.000	0.171	0.396	6.324	4.758	5.721	14375	Xrcc6	chr15	+	NM_0102	P16285	protein_cc	70kDa| G	X-ray repa	Xrcc6
7104	Nhp2l1	1.780	0.850	1.513	0.011	0.245	0.107	8.037	8.869	8.634	20826	Nhp2l1	chr15	-	NM_0114	P18182	protein_cc	FA-1| Fta1	NHP2 non	Nhp2l1
7105	4930407I1	1.000	1.000	1.000	1.000	1.000	1.000	0.000	0.000	0.000	328573	4930407I1	chr15	+	NM_0011	P6891	protein_cc	Gm747	RIKEN cDN	4930407I1
7106	Mei1	1.000	1.000	1.000	1.000	1.000	1.000	0.000	0.000	0.000	74369	Mei1	chr15	+	NM_0288	P8383	protein_cc	4932408F	meiotic do	Mei1
7107	Ccdc134	2.783	0.805	2.240	0.004	0.016	0.132	4.544	6.021	5.708	76457	Ccdc134	chr15	+	NM_1724	P10780	protein_cc	2310042L	coiled-coil	Ccdc134
7108	Srebf2	1.874	0.999	1.871	0.040	0.993	0.001	6.918	7.824	7.822	20788	Srebf2	chr15	+	NM_0332	P22054	protein_cc	AI608257|	sterol regu	Srebf2
7109	Shisa8	0.439	0.985	0.432	0.002	1.000	0.051	1.210	0.021	0.000	435145	Shisa8	chr15	-	NM_0012	P9909	protein_cc	ckamp39|	shisa fami	Shisa8
7110	Tnfrsf13c	12.529	0.175	2.187	0.004	0.006	0.245	0.589	4.236	1.718	72049	Tnfrsf13c	chr15	-	NM_0280	P13632	protein_cc	2010006P	tumor nec	Tnfrsf13c
7111	Cenpm	0.828	1.092	0.903	0.217	0.556	0.615	5.804	5.531	5.658	66570	Cenpm	chr15	-	NM_0256	P21897	protein_cc	2610019I	centromer	Cenpm
7112	1500009C	1.005	1.439	1.446	0.000	0.182	0.423	0.000	0.008	0.532	76505	1500009C	chr15	+	NR_03769	.	lncRNA		RIKEN cDN	1500009C
7113	Sept3	0.220	0.965	0.212	0.181	1.000	0.423	2.238	0.051	0.000	24050	Sept3	chr15	+	NM_0118	P26372	protein_cc	3110018K	septin 3	Sept3
7114	Wbp2nl	1.000	1.000	1.000	1.000	1.000	1.000	0.000	0.000	0.000	74716	Wbp2nl	chr15	+	NM_0290	P25259	protein_cc	4930521I2	WBP2 N-te	Wbp2nl
7115	Naga	0.400	1.618	0.647	0.010	0.034	0.235	5.839	4.516	5.211	17939	Naga	chr15	-	NM_0086	P23546	protein_cc	.	N-acetyl g	Naga
7116	Fam109b	0.689	1.544	0.823	0.421	0.297	0.823	1.685	1.147	1.774	338368	Fam109b	chr15	+	NM_1773	P3629	protein_cc	C920005C	family wit	Fam109b
7117	Smdt1	0.730	1.119	0.816	0.001	0.058	0.122	9.997	9.543	9.705	69029	Smdt1	chr15	+	NM_0269	P18601	protein_cc	1500032L	single-pas	Smdt1
7118	Ndufa6	0.420	0.938	0.394	0.000	0.145	0.001	12.544	11.292	11.199	67130	Ndufa6	chr15	-	NM_0259	P145	protein_cc	14kDa| 27	NADH deh	Ndufa6
7119	Cyp2d22	0.223	1.286	0.286	0.000	0.107	0.010	10.397	8.230	8.593	56448	Cyp2d22	chr15	-	NM_0198	P5239	protein_cc	2D22	cytochrom	Cyp2d22
7120	Cyp2d11	0.319	1.076	0.343	0.000	0.633	0.019	7.862	6.214	6.320	545123	Cyp2d11	chr15	-	NM_0011	P10662	protein_cc	Cyp2d| P4	cytochrom	Cyp2d11
7121	Cyp2d10	0.303	0.914	0.277	0.001	0.584	0.020	12.502	10.780	10.650	13101	Cyp2d10	chr15	-	NM_0100	P21493	protein_cc	AI303445|	cytochrom	Cyp2d10
7122	Cyp2d9	0.314	6.268	1.966	0.001	0.008	0.227	10.777	9.104	11.752	13105	Cyp2d9	chr15	+	NM_0100	P21084	protein_cc	Cyp2d| P4	cytochrom	Cyp2d9
7123	Cyp2d12	0.114	8.012	0.912	0.000	0.012	0.808	4.711	1.577	4.579	380997	Cyp2d12	chr15	+	NM_2013	P21949	protein_cc	9030605E	cytochrom	Cyp2d12
7124	Cyp2d34	0.202	1.733	0.351	0.000	0.025	0.026	5.566	3.262	4.055	223706	Cyp2d34	chr15	-	NM_1454	P4520	protein_cc	.	cytochrom	Cyp2d34
7125	Cyp2d13	0.118	1.840	0.216	0.000	0.012	0.028	8.444	5.356	6.236	68444	Cyp2d13	chr15	-	NR_00355	.	misc_RNA	1300007K	cytochrom	Cyp2d13
7126	Cyp2d37-	0.103	5.208	0.535	0.000	0.045	0.201	7.410	4.128	6.508	627860	Cyp2d37-	chr15	-	NR_03351	.	misc_RNA	EG627860	cytochrom	Cyp2d37-p
7127	Cyp2d40	0.469	1.400	0.656	0.000	0.027	0.086	8.990	7.897	8.382	71754	Cyp2d40	chr15	-	NM_0236	P4865	protein_cc	1300013D	cytochrom	Cyp2d40
7128	Cyp2d26	0.242	0.922	0.223	0.000	0.419	0.006	12.736	10.691	10.574	76279	Cyp2d26	chr15	-	NM_0295	P7727	protein_cc	1300006E	cytochrom	Cyp2d26
7129	Tcf20	0.580	1.598	0.927	0.087	0.136	0.838	5.987	5.202	5.878	21411	Tcf20	chr15	-	NM_0138	P19975	protein_cc	2810438H	transcripti	Tcf20
7130	Tbrg3	0.893	0.757	0.676	0.804	0.581	0.699	3.490	3.326	2.925	21378	Tbrg3	chr15	-	NR_02779	.	lncRNA	TB-11	transform	Tbrg3
7131	Gm20324	0.243	2.464	0.599	0.014	0.185	0.455	2.783	0.742	2.043	10050463	Gm20324	chr15	+	NR_04506	.	lncRNA		predicted	Gm20324
7132	Nfam1	2.169	1.245	2.699	0.106	0.472	0.176	3.904	5.021	5.337	74039	Nfam1	chr15	-	NM_0012	P20461	protein_cc	4921501M	Nfat activa	Nfam1
7133	Serhl	0.461	1.309	0.604	0.003	0.049	0.115	8.584	7.467	7.856	68607	Serhl	chr15	+	NM_0234	P6110	protein_cc	0610008B	serine hyd	Serhl
7134	Rrp7a	0.531	1.249	0.663	0.005	0.106	0.107	8.633	7.720	8.040	74778	Rrp7a	chr15	-	NM_0291	P15032	protein_cc	1110014J	ribosomal	Rrp7a
7135	Poldip3	1.299	0.769	0.999	0.028	0.183	0.998	8.628	9.006	8.627	73826	Poldip3	chr15	-	NM_1786	P15607	protein_cc	1110008P	polymeras	Poldip3
7136	Rnu12	4.477	1.844	8.256	0.001	0.477	0.454	3.911	6.074	6.957	104307	Rnu12	chr15	+	NR_00443	.	snRNA	.	RNA U12,	Rnu12
7137	Cyb5r3	0.332	1.535	0.510	0.000	0.050	0.045	11.451	9.861	10.479	109754	Cyb5r3	chr15	-	NM_0297	P8942	protein_cc	0610016L	cytochrom	Cyb5r3
7138	A4galt	3.425	0.486	1.665	0.077	0.168	0.377	0.588	2.365	1.324	239559	A4galt	chr15	-	NM_0010	P20753	protein_cc	.	alpha 1,4-	A4galt
7139	Arfgap3	6.172	0.834	5.150	0.000	0.047	0.014	5.500	8.125	7.864	66251	Arfgap3	chr15	-	NM_0254	P5778	protein_cc	0610009H	ADP-ribos	Arfgap3
7140	1700001L	0.596	2.231	1.330	0.123	0.255	0.732	4.537	3.790	4.948	69291	1700001L	chr15	-	NR_02798	.	lncRNA		RIKEN cDN	1700001L
7141	Pacsin2	0.580	1.012	0.587	0.052	0.842	0.235	7.447	6.661	6.678	23970	Pacsin2	chr15	-	NM_0011	P16126	protein_cc	AI197433	protein ki	Pacsin2
7142	Ttll1	0.989	0.778	0.769	0.971	0.449	0.357	4.488	4.472	4.110	319953	Ttll1	chr15	-	NM_1788	P2631	protein_cc	6330444E	tubulin ty	Ttll1
7143	Bik	0.229	2.205	0.504	0.004	0.197	0.131	5.928	3.798	4.939	12124	Bik	chr15	+	NM_0075	P9925	protein_cc	Biklk| Blk|	BCL2-inter	Bik
7144	Mcat	0.731	1.403	1.026	0.052	0.105	0.923	7.609	7.157	7.645	223722	Mcat	chr15	-	NM_0010	P4223	protein_cc	AI225907|	malonyl C	Mcat
7145	Tspo	2.369	0.920	2.180	0.008	0.548	0.017	8.846	10.090	9.970	12257	Tspo	chr15	+	NM_0097	P25960	protein_cc	Bzrp| IBP|	translocat	Tspo
7146	Ttll12	1.075	0.923	0.993	0.679	0.673	0.981	6.322	6.426	6.311	223723	Ttll12	chr15	-	NM_1830	P1944	protein_cc	BC055368	tubulin ty	Ttll12
7147	Scube1	0.516	1.136	0.587	0.044	0.777	0.272	1.661	0.708	0.892	64706	Scube1	chr15	-	NM_0227	P11623	protein_cc	7330410C	signal pep	Scube1
7148	Mpped1	1.005	1.439	1.446	0.000	0.182	0.423	0.000	0.008	0.532	233575	Mpped1	chr15	+	NM_1726	P5280	protein_cc	6230403M	metalloph	Mpped1
7149	Efcab6	1.000	1.000	1.000	1.000	1.000	1.000	0.000	0.000	0.000	77627	Efcab6	chr15	-	NM_0299	P11314	protein_cc	4931407K	EF-hand c	Efcab6
7150	Sult4a1	0.363	1.320	0.479	0.263	0.582	0.590	2.174	0.712	1.112	29859	Sult4a1	chr15	-	NM_0138	P16537	protein_cc	2400007A	sulfotrans	Sult4a1
7151	Pnpla5	1.000	1.000	1.000	1.000	1.000	1.000	0.000	0.000	0.000	75772	Pnpla5	chr15	-	NM_0294	P15048	protein_cc	4833426H	patatin-lik	Pnpla5
7152	Pnpla3	2.266	0.230	0.521	0.082	0.019	0.462	2.220	3.400	1.278	116939	Pnpla3	chr15	+	NM_0540	P17275	protein_cc	Adpn	patatin-lik	Pnpla3
7153	Samm50	0.817	1.442	1.178	0.087	0.010	0.075	9.751	9.459	9.988	68653	Samm50	chr15	+	NM_1786	P6225	protein_cc	1110030L	SAMM50	Samm50
7154	Parvb	1.308	1.134	1.484	0.506	0.742	0.409	4.359	4.747	4.929	170736	Parvb	chr15	+	NM_1331	P23057	protein_cc	affixin| AI	parvin, be	Parvb
7155	Parvg	1.476	0.456	0.673	0.412	0.126	0.590	4.056	4.617	3.484	64099	Parvg	chr15	+	NM_0223	P19473	protein_cc	AI413459	parvin, ga	Parvg
7156	1810041L	1.000	1.000	1.000	1.000	1.000	1.000	0.000	0.000	0.000	72301	1810041L	chr15	-	NM_0011	P14389	protein_cc	Kiaa1644	RIKEN cDN	1810041L1
7157	Ldoc1l	0.767	2.134	1.637	0.598	0.081	0.463	1.098	0.716	1.809	223732	Ldoc1l	chr15	-	NM_1776	P7030	protein_cc	BC058638	leucine zip	Ldoc1l
7158	Prr5	0.422	2.376	1.003	0.006	0.155	0.995	5.025	3.782	5.030	109270	Prr5	chr15	+	NM_1460	P2996	protein_cc	Arhgap8|	proline ric	Prr5
7159	Gm20556	1.572	0.636	1.000	0.304	1.000	0.423	0.000	0.653	0.000	328576	Gm20556	chr15	-	NR_04034	.	lncRNA	A430075N	predicted	Gm20556
7160	Arhgap8	4.898	0.562	2.755	0.000	0.165	0.472	0.371	2.664	1.833	73167	Arhgap8	chr15	+	NM_0011	P11273	protein_cc	3110043J	Rho GTPa	Arhgap8
7161	Phf21b	4.172	0.689	2.874	0.002	0.062	0.180	1.258	3.319	2.782	271305	Phf21b	chr15	-	NR_03073	.	protein_cc	A730032D	PHD finge	Phf21b
7162	Nup50	1.834	0.986	1.808	0.001	0.879	0.082	8.454	9.328	9.308	18141	Nup50	chr15	+	NM_0167	P16476	protein_cc	1700030K	nucleopor	Nup50
7163	5031439G	0.400	1.964	0.786	0.000	0.000	0.046	7.634	6.313	7.287	223739	5031439G	chr15	-	NM_0010	P26379	protein_cc	AW55457	RIKEN cDN	5031439G
7164	Upk3a	1.000	1.000	1.000	1.000	1.000	1.000	0.000	0.000	0.000	22270	Upk3a	chr15	+	NM_0234	P489	protein_cc	1110017C	uroplakin	Upk3a
7165	Fam118a	2.826	1.290	3.645	0.014	0.323	0.116	4.485	5.984	6.351	73225	Fam118a	chr15	+	NM_1337	P17967	protein_cc	.	family wit	Fam118a
7166	Smc1b	1.310	0.626	0.820	0.483	0.200	0.804	1.096	1.486	0.809	140557	Smc1b	chr15	-	NM_0804	P23662	protein_cc	SMC-1B| S	structural	Smc1b
7167	Ribc2	1.023	3.106	3.177	0.000	0.069	0.267	0.000	0.033	1.668	67747	Ribc2	chr15	+	NM_0263	P26774	protein_cc	4930579A	RIB43A do	Ribc2
7168	Gm19277	1.000	1.000	1.000	1.000	1.000	1.000	0.000	0.000	0.000	10050260	Gm19277	chr15	-	NR_04036	.	lncRNA	.	predicted	Gm19277
7169	Fbln1	0.790	1.098	0.868	0.542	0.854	0.770	3.037	2.697	2.833	14114	Fbln1	chr15	+	NM_0101	P1963	protein_cc	.	fibulin 1	Fbln1
7170	Atxn10	0.728	1.256	0.914	0.027	0.256	0.723	8.951	8.493	8.822	54138	Atxn10	chr15	+	NM_0168	P26302	protein_cc	AI325283|	ataxin 10	Atxn10
7171	7530416G	1.000	1.000	1.000	1.000	1.000	1.000	0.000	0.000	0.000	328577	7530416G	chr15	+	NM_0012	P7257	protein_cc	.	RIKEN cDN	7530416G
7172	Wnt7b	0.259	2.095	0.542	0.182	0.007	0.606	2.004	0.054	1.121	22422	Wnt7b	chr15	-	NM_0095	P752	protein_cc	Wnt-7b	wingless-t	Wnt7b
7173	AU022754	1.000	1.000	1.000	1.000	1.000	1.000	0.000	0.000	0.000	105975	AU022754	chr15	-	NR_04043	.	lncRNA	.	expressed	AU022754
7174	Lincppara	0.787	1.091	0.858	0.701	0.883	0.757	3.771	3.425	3.551	.	Lincppara	chr15	+	NR_11048	.	.	.	.	Lincppara
7175	Ppara	0.045	6.634	0.299	0.000	0.019	0.050	11.819	7.347	10.077	19013	Ppara	chr15	+	NM_0011	P25356	protein_cc	4933429D	peroxisom	Ppara
7176	Cdpf1	0.831	1.734	1.440	0.276	0.309	0.652	5.687	5.420	6.214	72355	Cdpf1	chr15	-	NM_1979	P19523	protein_cc	2210021J	cysteine ri	Cdpf1

7177	Pkdrej	0.373	0.981	0.366	0.018	1.000	0.140	1.450	0.027	0.000	18766	Pkdrej	chr15	-	NM_0111(	P1790	protein_cc	.	polycystin	Pkdrej
7178	Ttc38	0.066	2.490	0.165	0.000	0.025	0.004	9.914	6.002	7.318	239570	Ttc38	chr15	+	NM_0010.	P627	protein_cc	9030019H	tetratricop	Ttc38
7179	Gtse1	13.716	0.254	3.477	0.059	0.103	0.044	2.722	6.500	4.520	29870	Gtse1	chr15	+	NM_0011	P1758	protein_cc	B99| Gtse	G two S pl	Gtse1
7180	Trmu	1.863	1.035	1.929	0.106	0.894	0.259	5.072	5.970	6.020	72026	Trmu	chr15	+	NM_0280(	P19067	protein_cc	1110005N	tRNA 5-m	Trmu
7181	Celsr1	0.033	8.373	0.279	0.000	0.024	0.037	6.494	1.586	4.652	12614	Celsr1	chr15	-	NM_0098	P24355	protein_cc	Adgrc1| cı	cadherin,	Celsr1
7182	Gramd4	0.781	0.930	0.727	0.594	0.906	0.523	4.576	4.219	4.115	223752	Gramd4	chr15	+	NM_1726	P4575	protein_cc	9930016O	GRAM dor	Gramd4
7183	Cerk	1.832	0.820	1.503	0.000	0.194	0.276	5.744	6.617	6.332	223753	Cerk	chr15	-	NM_1454	P12578	protein_cc	AI848610|	ceramide	Cerk
7184	Tbc1d22a	0.941	1.625	1.529	0.320	0.012	0.121	7.855	7.768	8.468	223754	Tbc1d22a	chr15	+	NM_1454	P16980	protein_cc	BC023106	TBC1 dom	Tbc1d22a
7185	Fam19a5	0.455	4.311	1.962	0.097	0.024	0.276	4.086	2.950	5.058	106014	Fam19a5	chr15	+	NM_0012	P2484	protein_cc	Tafa-5| TA	family wit	Fam19a5
7186	B230214G	1.000	1.000	1.000	1.000	1.000	1.000	0.000	0.000	0.000	328579	B230214G	chr15	+	NR_04528	.	lncRNA	B230214G	RIKEN cDN	B230214G
7187	Zdhhc25	1.000	1.000	1.000	1.000	1.000	1.000	0.000	0.000	0.000	70073	Zdhhc25	chr15	+	NM_0273(	P759	protein_cc	1700030J1	zinc finger	Zdhhc25
7188	Brd1	1.032	0.800	0.826	0.702	0.050	0.071	8.338	8.383	8.062	223770	Brd1	chr15	-	NM_0010.	P7663	protein_cc	1110059H	bromodor	Brd1
7189	Zbed4	1.871	0.857	1.604	0.037	0.425	0.072	6.407	7.311	7.089	223773	Zbed4	chr15	+	NM_1814	P12846	protein_cc	1700009F(	zinc finger	Zbed4
7190	Alg12	2.190	0.913	2.001	0.004	0.437	0.022	5.115	6.246	6.115	223774	Alg12	chr15	-	NM_0011.	P13907	protein_cc	ECM39	asparagine	Alg12
7191	Creld2	8.835	0.404	3.569	0.000	0.001	0.041	7.710	10.853	9.546	76737	Creld2	chr15	+	NM_0297.	P26561	protein_cc	5730592L:	cysteine-r	Creld2
7192	Pim3	0.726	1.041	0.756	0.115	0.836	0.331	12.005	11.544	11.601	223775	Pim3	chr15	+	NM_1454	P411	protein_cc	BC026639	proviral in	Pim3
7193	Ttll8	1.590	0.901	1.432	0.302	0.794	0.423	0.000	0.669	0.519	239591	Ttll8	chr15	-	NM_1728	P18100	protein_cc	1700019P(	tubulin ty	Ttll8
7194	Mlc1	1.030	3.946	4.065	0.000	0.101	0.320	0.001	0.044	2.024	170790	Mlc1	chr15	-	NM_1332.	P4991	protein_cc	AW04863(	megalenc	Mlc1
7195	Mov10l1	1.572	0.636	1.000	0.304	1.000	0.423	0.000	0.653	0.000	83456	Mov10l1	chr15	+	NM_0312(	P8781	protein_cc	CHAMP| (	Moloney	Mov10l1
7196	Panx2	1.000	1.000	1.000	1.000	1.000	1.000	0.000	0.000	0.000	406218	Panx2	chr15	+	NM_0010(	P5446	protein_cc	.	pannexin	Panx2
7197	1810021B	0.241	2.609	0.628	0.032	0.018	0.408	6.616	4.561	5.944	69120	1810021B	chr15	-	NR_04041	.	lncRNA		RIKEN cDN	1810021B
7198	Trabd	1.183	1.087	1.286	0.352	0.601	0.334	9.310	9.552	9.672	67976	Trabd	chr15	+	NM_0264	P11336	protein_cc	5730502D	TraB doma	Trabd
7199	Selo	0.388	1.340	0.520	0.008	0.119	0.124	8.633	7.268	7.690	223776	Selo	chr15	+	NM_0279(	P21099	protein_cc	1300018J1	selenopro	Selo
7200	Tubgcp6	0.405	1.375	0.558	0.006	0.056	0.118	8.700	7.398	7.858	328580	Tubgcp6	chr15	-	NM_0011(	P19122	protein_cc	AI843275|	tubulin, ga	Tubgcp6
7201	Hdac10	0.395	1.790	0.707	0.021	0.043	0.401	5.409	4.068	4.908	170787	Hdac10	chr15	-	NM_1991	P26881	protein_cc	AW54889	histone de	Hdac10
7202	Mapk12	0.847	0.800	0.678	0.743	0.721	0.136	4.392	4.153	3.831	29857	Mapk12	chr15	-	NM_0138	P10291	protein_cc	AW12370	mitogen-a	Mapk12
7203	Mapk11	1.689	0.709	1.198	0.012	0.235	0.770	2.732	3.489	2.993	19094	Mapk11	chr15	-	NM_0111(	P16039	protein_cc	p38-2| P3	mitogen-a	Mapk11
7204	Plxnb2	0.397	1.276	0.506	0.000	0.331	0.086	9.654	8.320	8.671	140570	Plxnb2	chr15	-	NM_0012	P6825	protein_cc	Debt| ple:	plexin B2	Plxnb2
7205	Dennd6b	0.357	0.523	0.187	0.083	0.061	0.213	4.831	3.344	2.409	69440	Dennd6b	chr15	-	NM_0270.	P12303	protein_cc	1700027J(	DENN/MA	Dennd6b
7206	Ppp6r2	0.421	1.177	0.496	0.114	0.761	0.439	4.083	2.836	3.071	71474	Ppp6r2	chr15	+	NM_0268	P21395	protein_cc	1110033O	protein ph	Ppp6r2
7207	Sbf1	1.004	0.950	0.954	0.966	0.516	0.651	6.909	6.914	6.841	77980	Sbf1	chr15	-	NM_0011	P20211	protein_cc	2610510A	SET bindin	Sbf1
7208	Adm2	1.000	1.000	1.000	1.000	1.000	1.000	0.000	0.000	0.000	223780	Adm2	chr15	+	NM_1829.	P13476	protein_cc	Am2| IMD	adrenome	Adm2
7209	Miox	1.698	2.457	4.172	0.542	0.071	0.029	1.004	1.768	3.065	56727	Miox	chr15	+	NM_0199	P3526	protein_cc	0610009I1	myo-inosit	Miox
7210	Lmf2	0.683	1.072	0.732	0.005	0.566	0.172	6.575	6.024	6.125	105847	Lmf2	chr15	-	NM_1789	P8257	protein_cc	AI451006|	lipase mat	Lmf2
7211	Ncaph2	0.404	1.206	0.488	0.000	0.049	0.003	10.181	8.874	9.145	52683	Ncaph2	chr15	+	NM_0012	P19046	protein_cc	.	non-SMC	Ncaph2
7212	Sco2	0.240	1.164	0.279	0.000	0.015	0.009	9.819	7.759	7.978	10012682	Sco2	chr15	-	NM_0011	P1238	protein_cc	.	SCO2 cyto	Sco2
7213	Tymp	0.269	0.901	0.242	0.000	0.332	0.020	6.290	4.395	4.245	72962	Tymp	chr15	-	NM_1383(	P22266	protein_cc	2900072D	thymidine	Tymp
7214	Odf3b	0.147	1.821	0.268	0.000	0.228	0.018	5.606	2.838	3.704	70113	Odf3b	chr15	-	NM_0010	P826	protein_cc	2010001J:	outer den:	Odf3b
7215	Klhdc7b	1.000	1.000	1.000	1.000	1.000	1.000	0.000	0.000	0.000	546648	Klhdc7b	chr15	+	NM_0011(	P12257	protein_cc	EG546648	kelch dom	Klhdc7b
7216	Syce3	0.534	0.990	0.528	0.000	1.000	0.015	0.921	0.015	0.000	75459	Syce3	chr15	-	NM_0011(	P13920	protein_cc	1700007E(	synaptone	Syce3
7217	Cpt1b	0.965	0.797	0.769	0.956	0.758	0.324	3.080	3.028	2.701	12895	Cpt1b	chr15	-	NM_0099.	P2986	protein_cc	Cpt1| Cpt	carnitine	Cpt1b
7218	ChkbCpt1l	0.632	1.238	0.783	0.040	0.213	0.491	6.788	6.127	6.435	.	ChkbCpt1l	chr15	-	NR_00484	.	.	.	.	ChkbCpt1b
7219	Chkb	0.696	1.124	0.782	0.008	0.485	0.374	8.309	7.785	7.954	12651	Chkb	chr15	-	NM_0076	P26051	protein_cc	Chetk| Ch	choline kir	Chkb
7220	Mapk8ip2	1.000	1.000	1.000	1.000	1.000	1.000	0.000	0.000	0.000	60597	Mapk8ip2	chr15	+	NM_0219.	P14251	protein_cc	3230402N	mitogen-a	Mapk8ip2
7221	Arsa	1.642	1.464	2.403	0.079	0.178	0.160	7.184	7.899	8.449	11883	Arsa	chr15	-	NM_0097	P25638	protein_cc	As-2| AS-/	arylsulfata	Arsa
7222	C230037L	4.250	1.408	5.984	0.001	0.080	0.062	2.213	4.301	4.794	10053273	C230037L	chr15	+	NR_07723	.	lncRNA		RIKEN cDN	C230037L
7223	Shank3	2.891	1.746	5.049	0.035	0.337	0.328	1.020	2.551	3.355	58234	Shank3	chr15	+	NM_0214	P14054	protein_cc	AI841104|	SH3/anky	Shank3
7224	Acr	1.812	0.913	1.654	0.126	0.857	0.707	1.681	2.538	2.407	11434	Acr	chr15	+	NM_0012	P6625	protein_cc	.	acrosin pr	Acr
7225	Rabl2	1.280	1.085	1.389	0.341	0.729	0.221	4.630	4.987	5.104	68708	Rabl2	chr15	-	NM_0268	P14884	protein_cc	1110031N	RAB, mem	Rabl2
7226	Syt10	1.000	1.000	1.000	1.000	1.000	1.000	0.000	0.000	0.000	54526	Syt10	chr15	-	NM_0188(	P21647	protein_cc	.	synaptota	Syt10
7227	Alg10b	3.507	1.129	3.959	0.007	0.672	0.196	3.427	5.238	5.413	380959	Alg10b	chr15	+	NM_0010.	P8099	protein_cc	AA469671	asparagine	Alg10b
7228	Cpne8	23.675	0.877	20.774	0.000	0.053	0.006	4.060	8.625	8.437	66871	Cpne8	chr15	-	NM_0258	P26827	protein_cc	1200003E:	copine VII	Cpne8
7229	Spn-ps	1.000	1.000	1.000	1.000	1.000	1.000	0.000	0.000	0.000	20738	Spn-ps	chr15	-	NR_03358	.	misc_RNA	Cd43-ps	sialophori	Spn-ps
7230	Kif21a	1.221	0.792	0.967	0.051	0.152	0.911	9.134	9.422	9.085	16564	Kif21a	chr15	-	NM_0011(	P6639	protein_cc	.	kinesin fai	Kif21a
7231	Abcd2	0.013	1.885	0.024	0.000	0.262	0.010	8.739	2.427	3.342	26874	Abcd2	chr15	-	NM_0119	P11181	protein_cc	ABC39| AI	ATP-bindi	Abcd2
7232	Slc2a13	0.357	0.443	0.158	0.230	1.000	0.375	2.663	1.176	0.000	239606	Slc2a13	chr15	-	NM_0010.	P21797	protein_cc	6530403A	solute car	Slc2a13
7233	Lrrk2	2.622	0.481	1.260	0.061	0.138	0.718	3.224	4.615	3.557	66725	Lrrk2	chr15	+	NM_0257.	P9453	protein_cc	49215130	leucine-ric	Lrrk2
7234	Muc19	1.152	0.629	0.725	0.722	1.000	0.422	0.465	0.669	0.000	239611	Muc19	chr15	+	NM_2072.	P23585	protein_cc	MUC-19|	mucin 19	Muc19
7235	Smgc	1.000	1.000	1.000	1.000	1.000	1.000	0.000	0.000	0.000	223809	Smgc	chr15	+	NM_1989.	P23864	protein_cc	2310010P.	submandi	Smgc
7236	Cntn1	1.010	1.825	1.844	0.000	0.182	0.423	0.000	0.015	0.883	12805	Cntn1	chr15	+	NM_0011	P24433	protein_cc	AW49509	contactin	Cntn1
7237	Pdzrn4	1.000	1.000	1.000	1.000	1.000	1.000	0.000	0.000	0.000	239618	Pdzrn4	chr15	+	NM_0011(	P614	protein_cc	1110017D	PDZ doma	Pdzrn4
7238	Gxylt1	1.412	0.975	1.377	0.036	0.902	0.490	7.366	7.864	7.828	223827	Gxylt1	chr15	-	NM_0010.	P17101	protein_cc	Glt8d3| G	glucoside	Gxylt1
7239	Yaf2	0.991	1.272	1.261	0.928	0.039	0.008	7.836	7.823	8.171	67057	Yaf2	chr15	-	NM_0241	P22391	protein_cc	2810021N	YY1 assoc	Yaf2
7240	Zcrb1	1.110	1.133	1.257	0.451	0.317	0.028	9.760	9.910	10.090	67197	Zcrb1	chr15	-	NM_0260.	P17792	protein_cc	2700088N	zinc finger	Zcrb1
7241	Pphln1	1.878	0.788	1.479	0.051	0.274	0.054	6.998	7.907	7.563	223828	Pphln1	chr15	+	NM_0012	P9755	protein_cc	CR| HSPC:	periphilin	Pphln1
7242	Prickle1	0.886	1.398	1.238	0.432	0.281	0.620	5.086	4.911	5.394	106042	Prickle1	chr15	-	NM_0010.	P9908	protein_cc	1110058P.	prickle pla	Prickle1
7243	9430014N	1.000	1.000	1.000	1.000	1.000	1.000	0.000	0.000	0.000	77248	9430014N	chr15	-	NR_04573	.	lncRNA		RIKEN cDN	9430014N
7244	D630010B	1.581	0.879	1.390	0.301	0.744	0.423	0.000	0.661	0.476	328594	D630010B	chr15	+	NR_04562	.	lncRNA	Gm811	RIKEN cDN	D630010B
7245	Adamts20	0.716	0.995	0.712	0.182	1.000	0.423	0.490	0.008	0.000	223838	Adamts20	chr15	-	NM_1774	P5436	protein_cc	Adamst20	a disinteg	Adamts20
7246	Pus7l	4.387	0.463	2.030	0.020	0.068	0.385	2.819	4.953	3.841	78895	Pus7l	chr15	-	NM_1724	P25272	protein_cc	3000003F(	pseudouri	Pus7l
7247	Irak4	2.420	0.588	1.423	0.000	0.000	0.118	6.779	8.054	7.288	266632	Irak4	chr15	+	NM_0299	P15431	protein_cc	8430405N	interleukir	Irak4
7248	Twf1	1.470	0.898	1.320	0.066	0.500	0.163	8.233	8.788	8.634	19230	Twf1	chr15	-	NM_0089	P25928	protein_cc	A6| Ptk9|	twinfilin	Twf1
7249	Tmem117	0.716	0.995	0.712	0.182	1.000	0.423	0.490	0.008	0.000	320709	Tmem117	chr15	+	NM_1787	P1836	protein_cc	B930062P	transmem	Tmem117
7250	LOC10004	1.000	1.000	1.000	1.000	1.000	1.000	0.000	0.000	0.000	10004331	LOC10004	chr15	-	NR_01548	.	lncRNA		uncharact	LOC10004
7251	Nell2	1.000	1.000	1.000	1.000	1.000	1.000	0.000	0.000	0.000	54003	Nell2	chr15	-	NM_0012	P15636	protein_cc	A330108N	NEL-like 2	Nell2
7252	Dbx2	1.000	1.000	1.000	1.000	1.000	1.000	0.000	0.000	0.000	223843	Dbx2	chr15	-	NM_2075	P983	protein_cc	9430056A	developin	Dbx2
7253	Gm6961	1.000	1.000	1.000	1.000	1.000	1.000	0.000	0.000	0.000	629300	Gm6961	chr15	-	NR_13114	.	lncRNA	EG629300	predicted	Gm6961
7254	Ano6	0.725	1.033	0.749	0.010	0.831	0.331	6.615	6.151	6.198	105722	Ano6	chr15	+	NM_0012	P7885	protein_cc	2900059G	anoctamir	Ano6
7255	D030018L	1.589	1.186	1.885	0.298	0.707	0.423	0.000	0.669	0.915	402773	D030018L	chr15	-	NR_12649	.	misc_RNA	.	RIKEN cDN	D030018L
7256	Gm4371	1.005	1.439	1.446	0.000	0.182	0.423	0.000	0.008	0.532	10004333	Gm4371	chr15	+	NR_02831	.	misc_RNA	10004333	predicted	Gm4371
7257	2610037D	1.000	1.000	1.000	1.000	1.000	1.000	0.000	0.000	0.000	70040	2610037D	chr15	-	NR_04042	.	lncRNA	AU024679	RIKEN cDN	2610037D
7258	4833422M	1.000	1.000	1.000	1.000	1.000	1.000	0.000	0.000	0.000	75768	4833422N	chr15	-	NR_13016	.	lncRNA	4933406G	RIKEN cDN	4833422M
7259	E330033B	1.467	0.299	0.438	0.000	0.000	0.006	3.869	4.422	2.678	319722	E330033B	chr15	-	NR_03069	.	lncRNA	.	RIKEN cDN	E330033B
7260	Arid2	0.848	0.808	0.685	0.330	0.229	0.335	7.681	7.444	7.135	77044	Arid2	chr15	+	NM_1752	P19972	protein_cc	1700124K	AT rich int	Arid2
7261	Scaf11	0.825	0.794	0.655	0.023	0.015	0.040	9.637	9.359	9.026	72193	Scaf11	chr15	-	NM_0281	P26382	protein_cc	1110061H	SR-related	Scaf11
7262	Slc38a1	1.248	0.794	0.991	0.108	0.427	0.986	4.851	5.171	4.838	105727	Slc38a1	chr15	-	NM_1340	P4201	protein_cc	AA408026	solute car	Slc38a1
7263	Slc38a2	1.262	0.662	0.835	0.018	0.024	0.460	11.316	11.651	11.056	67760	Slc38a2	chr15	-	NM_1751	P22112	protein_cc	5033402L:	solute car	Slc38a2
7264	Slc38a4	0.193	1.254	0.242	0.000	0.123	0.004	13.633	11.260	11.587	69354	Slc38a4	chr15	-	NM_0270	P1568	protein_cc	1110012E:	solute car	Slc38a4
7265	Amigo2	5.161	0.469	2.419	0.000	0.003	0.175	6.181	8.548	7.455	105827	Amigo2	chr15	-	NM_0011(	P26735	protein_cc	AI415330|	adhesion r	Amigo2
7266	Pced1b	0.725	1.010	0.733	0.556	0.983	0.642	4.925	4.462	4.476	239647	Pced1b	chr15	+	NM_1722	P17017	protein_cc	Fam113b	PC-esteras	Pced1b
7267	Rpap3	11.021	1.229	13.544	0.000	0.137	0.030	5.523	8.985	9.282	71919	Rpap3	chr15	-	NM_0280(	P14070	protein_cc	2310042P.	RNA polyr	Rpap3

7268	Endou	0.431	0.603	0.260	0.130	0.439	0.188	2.357	1.145	0.416	19011	Endou	chr15	-	NM_0011	P20241	protein_cc	Pp11r| Tcl	endonucle	Endou
7269	Rapgef3	0.743	1.263	0.939	0.106	0.554	0.913	4.331	3.903	4.239	223864	Rapgef3	chr15	-	NM_1448	P17172	protein_cc	2310016P	Rap guani	Rapgef3
7270	Slc48a1	0.422	1.536	0.648	0.001	0.012	0.085	9.867	8.621	9.241	67739	Slc48a1	chr15	+	NM_0263	P18314	protein_cc	4930570C	solute car	Slc48a1
7271	Hdac7	0.420	2.468	1.037	0.002	0.004	0.873	8.039	6.788	8.091	56233	Hdac7	chr15	-	NM_0012	P26751	protein_cc	5830434K	histone de	Hdac7
7272	Vdr	7.250	1.180	8.555	0.000	0.769	0.380	1.090	3.948	4.187	22337	Vdr	chr15	-	NM_0095	P4984	protein_cc	Nr1i1	vitamin D	Vdr
7273	Tmem106	0.432	1.371	0.593	0.013	0.117	0.247	6.359	5.150	5.604	380967	Tmem106	chr15	+	NM_0012	P7362	protein_cc	AI046681|	transmem	Tmem106
7274	Col2a1	1.000	1.000	1.000	1.000	1.000	1.000	0.000	0.000	0.000	12824	Col2a1	chr15	-	NM_0011	P23963	protein_cc	Col2| Col2	collagen, t	Col2a1
7275	Senp1	1.986	0.447	0.888	0.022	0.013	0.505	5.924	6.914	5.752	223870	Senp1	chr15	-	NM_1448	P24815	protein_cc	2310046A	SUMO1/se	Senp1
7276	Pfkm	0.179	2.942	0.526	0.000	0.099	0.235	6.142	3.658	5.215	18642	Pfkm	chr15	+	NM_0011	P20454	protein_cc	AI131669|	phosphofr	Pfkm
7277	Asb8	1.292	1.075	1.389	0.031	0.725	0.474	8.274	8.644	8.748	78541	Asb8	chr15	-	NM_0011	P9734	protein_cc	4930539L	ankyrin re	Asb8
7278	Ccdc184	1.014	2.240	2.272	0.000	0.182	0.423	0.000	0.021	1.185	239650	Ccdc184	chr15	+	NM_1777	P22401	protein_cc	5630400A	coiled-coil	Ccdc184
7279	Olfr288	1.000	1.000	1.000	1.000	1.000	1.000	0.000	0.000	0.000	545140	Olfr288	chr15	-	NM_0010	P17535	protein_cc	C85333| C	olfactory r	Olfr288
7280	Olfr287	1.000	1.000	1.000	1.000	1.000	1.000	0.000	0.000	0.000	634104	Olfr287	chr15	-	NM_0010	P4368	protein_cc	MOR286-1	olfactory r	Olfr287
7281	Olfr286	1.000	1.000	1.000	1.000	1.000	1.000	0.000	0.000	0.000	629524	Olfr286	chr15	-	NM_0010	P6704	protein_cc	EG629524	olfactory r	Olfr286
7282	H1fnt	1.000	1.000	1.000	1.000	1.000	1.000	0.000	0.000	0.000	70069	H1fnt	chr15	-	NM_0273	P6723	protein_cc	1700026P	H1 histone	H1fnt
7283	Zfp641	9.444	0.791	7.471	0.000	0.533	0.299	0.704	3.943	3.605	239652	Zfp641	chr15	-	NM_1737	P23555	protein_cc	9930016F	zinc finger	Zfp641
7284	Olfr285	1.000	1.000	1.000	1.000	1.000	1.000	0.000	0.000	0.000	257943	Olfr285	chr15	-	NM_0010	P5587	protein_cc	MOR160-3	olfactory r	Olfr285
7285	Olfr257	1.000	1.000	1.000	1.000	1.000	1.000	0.000	0.000	0.000	.	Olfr257	chr15	-	NM_2075	P5587	.	.	.	Olfr257
7286	Olfr284	1.000	1.000	1.000	1.000	1.000	1.000	0.000	0.000	0.000	258278	Olfr284	chr15	-	NM_1462	P3401	protein_cc	MOR160-4	olfactory r	Olfr284
7287	Olfr283	1.000	1.000	1.000	1.000	1.000	1.000	0.000	0.000	0.000	259038	Olfr283	chr15	-	NM_1470	P11141	protein_cc	MOR160-1	olfactory r	Olfr283
7288	1700031M	0.669	0.995	0.666	0.181	1.000	0.423	0.587	0.008	0.000	73302	1700031M	chr15	+	NR_01549	.	lncRNA	4930413L	RIKEN cDN	1700031M
7289	Olfr282	1.000	1.000	1.000	1.000	1.000	1.000	0.000	0.000	0.000	258449	Olfr282	chr15	-	NM_1464	P3149	protein_cc	MOR160-2	olfactory r	Olfr282
7290	Olfr281	1.000	1.000	1.000	1.000	1.000	1.000	0.000	0.000	0.000	258277	Olfr281	chr15	+	NM_1462	P2896	protein_cc	MOR160-5	olfactory r	Olfr281
7291	Lalba	0.364	0.981	0.357	0.000	1.000	0.013	1.485	0.027	0.000	16770	Lalba	chr15	-	NM_0106	P10070	protein_cc	AW20882	lactalbum	Lalba
7292	Olfr279	1.000	1.000	1.000	1.000	1.000	1.000	0.000	0.000	0.000	258502	Olfr279	chr15	+	NM_0010	P26822	protein_cc	MOR122-1	olfactory r	Olfr279
7293	Kansl2	1.061	0.981	1.040	0.668	0.874	0.889	7.103	7.188	7.160	69612	Kansl2	chr15	-	NM_1337	P1873	protein_cc	2310037I2	KAT8 regu	Kansl2
7294	Snora34	1.000	1.000	1.000	1.000	1.000	1.000	0.000	0.000	0.000	10021741	Snora34	chr15	-	NR_03405	.	snoRNA	MBI-89	small nucl	Snora34
7295	Snora2b	0.785	1.399	1.099	0.729	0.542	0.931	2.375	2.026	2.510	10021741	Snora2b	chr15	-	NR_03405	.	snoRNA	MBI-137	small nucl	Snora2b
7296	Ccnt1	1.738	0.378	0.657	0.001	0.000	0.124	4.892	5.689	4.285	12455	Ccnt1	chr15	-	NM_0098	P11752	protein_cc	2810478G	cyclin T1	Ccnt1
7297	9330020H	1.589	1.185	1.882	0.000	0.053	0.037	3.169	3.836	4.081	10004889	9330020H	chr15	+	NR_02844	.	lncRNA	ENSMUSG	RIKEN cDN	9330020H
7298	4930415O	1.000	1.000	1.000	1.000	1.000	1.000	0.000	0.000	0.000	73863	4930415O	chr15	+	NM_0012	P18060	protein_cc	.	RIKEN cDN	4930415O
7299	Adcy6	0.216	1.779	0.384	0.000	0.037	0.013	6.475	4.264	5.095	11512	Adcy6	chr15	-	NM_0074	P10793	protein_cc	AC6| mKIA	adenylate	Adcy6
7300	Cacnb3	1.257	1.634	2.054	0.595	0.057	0.324	1.688	2.019	2.727	12297	Cacnb3	chr15	+	NM_0010	P6372	protein_cc	Beta3| Ca	calcium ch	Cacnb3
7301	Ddx23	0.812	0.282	0.229	0.420	0.024	0.105	3.869	3.569	1.741	74351	Ddx23	chr15	-	NM_0010	P9265	protein_cc	3110082M	DEAD (Asp	Ddx23
7302	Rnd1	12.953	0.190	2.467	0.005	0.010	0.411	6.436	10.131	7.739	223881	Rnd1	chr15	-	NM_1726	P1033	protein_cc	A830014L	Rho family	Rnd1
7303	Ccdc65	1.000	1.000	1.000	1.000	1.000	1.000	0.000	0.000	0.000	105833	Ccdc65	chr15	+	NM_1535	P5391	protein_cc	4933417K	coiled-coil	Ccdc65
7304	Fkbp11	2.537	1.294	3.282	0.000	0.421	0.233	6.889	8.233	8.604	66120	Fkbp11	chr15	-	NM_0241	P893	protein_cc	1110002O	FK506 bin	Fkbp11
7305	Arf3	1.170	1.062	1.242	0.343	0.659	0.330	7.951	8.177	8.264	11842	Arf3	chr15	-	NM_0074	P12774	protein_cc	5430400P	ADP-ribos	Arf3
7306	Wnt10b	1.146	0.632	0.725	0.729	1.000	0.422	0.465	0.661	0.000	22410	Wnt10b	chr15	-	NM_0117	P7046	protein_cc	Wnt12	wingless-t	Wnt10b
7307	Wnt1	1.000	1.000	1.000	1.000	1.000	1.000	0.000	0.000	0.000	22408	Wnt1	chr15	+	NM_0212	P16393	protein_cc	Int-1| sw|	wingless-t	Wnt1
7308	Ddn	3.363	0.650	2.186	0.000	0.019	0.142	0.001	1.751	1.129	13199	Ddn	chr15	-	NM_0010	P15818	protein_cc	Gm748	dendrin	Ddn
7309	Prkag1	1.415	1.121	1.587	0.067	0.325	0.069	9.005	9.507	9.672	19082	Prkag1	chr15	-	NM_0167	P18003	protein_cc	AA571379	protein kin	Prkag1
7310	Kmt2d	1.256	0.706	0.887	0.045	0.000	0.521	6.722	7.051	6.549	381022	Kmt2d	chr15	-	NM_0010	P17963	protein_cc	ALR| BC03	lysine (K)-	Kmt2d
7311	Rhebl1	0.996	1.482	1.476	0.993	0.276	0.461	3.510	3.504	4.071	69159	Rhebl1	chr15	-	NM_0269	P24656	protein_cc	1810036J2	Ras homo	Rhebl1
7312	Dhh	0.747	1.304	0.974	0.281	0.670	0.978	2.445	2.025	2.407	13363	Dhh	chr15	-	NM_0078	P24700	protein_cc	C78960	desert hed	Dhh
7313	Lmbr1l	0.715	1.284	0.917	0.183	0.299	0.786	5.534	5.050	5.410	74775	Lmbr1l	chr15	-	NM_0290	P2672	protein_cc	1110013E	limb regio	Lmbr1l
7314	Tuba1b	1.808	0.921	1.666	0.025	0.717	0.317	9.512	10.366	10.248	22143	Tuba1b	chr15	-	NM_0116	P21289	protein_cc	Tuba2	tubulin, al	Tuba1b
7315	Tuba1a	0.924	1.432	1.323	0.557	0.073	0.400	7.657	7.543	8.061	22142	Tuba1a	chr15	-	NM_0116	P7531	protein_cc	Tuba-1| Tu	tubulin, al	Tuba1a
7316	4930578M	2.144	0.663	1.422	0.303	0.511	0.423	0.000	1.101	0.508	75951	4930578M	chr15	+	NR_04599	.	lncRNA	3010001K	RIKEN cDN	4930578M
7317	Tuba1c	1.337	1.489	1.991	0.000	0.000	0.003	11.334	11.754	12.328	22146	Tuba1c	chr15	+	NM_0094	P4921	protein_cc	M[a]6| Tu	tubulin, al	Tuba1c
7318	Prph	0.382	0.982	0.375	0.182	1.000	0.423	1.416	0.027	0.000	19132	Prph	chr15	+	NM_0136	P4302	protein_cc	Prph1	peripherin	Prph
7319	Troap	1.497	0.522	0.782	0.605	0.464	0.159	1.149	1.731	0.794	78733	Troap	chr15	+	NM_0011	P11695	protein_cc	AW47606	trophinin	Troap
7320	C1ql4	1.005	1.405	1.412	0.000	0.182	0.423	0.000	0.008	0.498	239659	C1ql4	chr15	-	NM_0010	P8379	protein_cc	C1qtnf11|	compleme	C1ql4
7321	Dnajc22	1.464	1.158	1.696	0.127	0.581	0.324	7.323	7.873	8.085	72778	Dnajc22	chr15	+	NM_1768	P25997	protein_cc	2810451A	DnaJ heat	Dnajc22
7322	Spats2	2.643	1.469	3.883	0.003	0.005	0.010	4.898	6.300	6.855	72572	Spats2	chr15	+	NM_1391	P1341	protein_cc	2700012F	spermatog	Spats2
7323	Kcnh3	1.590	0.901	1.432	0.302	0.794	0.423	0.000	0.669	0.519	16512	Kcnh3	chr15	+	NM_0106	P19695	protein_cc	AU019351	potassium	Kcnh3
7324	Mcrs1	1.712	1.051	1.800	0.029	0.826	0.343	4.639	5.415	5.487	51812	Mcrs1	chr15	-	NM_0167	P3873	protein_cc	C78274| I	microsphe	Mcrs1
7325	1700120C	2.041	0.646	1.319	0.399	0.564	0.660	0.420	1.449	0.819	73600	1700120C	chr15	+	NR_04562	.	lncRNA	.	RIKEN cDN	1700120C
7326	Fam186b	0.451	0.986	0.444	0.182	1.000	0.423	1.170	0.021	0.000	545136	Fam186b	chr15	-	NM_0010	P19297	protein_cc	EG545136	family wit	Fam186b
7327	Prpf40b	0.892	0.668	0.595	0.458	0.166	0.374	6.399	6.234	5.651	54614	Prpf40b	chr15	+	NM_0187	P1768	protein_cc	2610317D	pre-mRNA	Prpf40b
7328	Fmnl3	1.219	0.906	1.105	0.732	0.871	0.911	3.962	4.247	4.106	22379	Fmnl3	chr15	-	NM_0117	P7988	protein_cc	2700073B	formin-like	Fmnl3
7329	Tmbim6	0.440	0.984	0.433	0.001	0.783	0.029	13.173	11.988	11.965	110213	Tmbim6	chr15	+	NM_0011	P13897	protein_cc	5031406P	transmem	Tmbim6
7330	Nckap5l	1.083	2.437	2.639	0.809	0.000	0.091	1.067	1.181	2.467	380969	Nckap5l	chr15	-	NM_0010	P3312	protein_cc	C230021P	NCK-assoc	Nckap5l
7331	Bcdin3d	1.434	0.729	1.045	0.502	0.545	0.033	5.244	5.764	5.308	75284	Bcdin3d	chr15	-	NM_0292	P4476	protein_cc	4930556P	BCDIN3 do	Bcdin3d
7332	Faim2	0.355	0.981	0.348	0.056	1.000	0.240	1.524	0.028	0.000	72393	Faim2	chr15	-	NM_0010	P9930	protein_cc	2900002L	Fas apopt	Faim2
7333	Aqp2	1.000	1.000	1.000	1.000	1.000	1.000	0.000	0.000	0.000	11827	Aqp2	chr15	+	NM_0096	P14512	protein_cc	AQP-CD| (	aquaporin	Aqp2
7334	Aqp5	0.325	0.978	0.318	0.039	1.000	0.201	1.655	0.033	0.000	11830	Aqp5	chr15	+	NM_0097	P1645	protein_cc	.	aquaporin	Aqp5
7335	Aqp6	1.000	1.000	1.000	1.000	1.000	1.000	0.000	0.000	0.000	11831	Aqp6	chr15	+	NM_1750	P22657	protein_cc	AI790337	aquaporin	Aqp6
7336	Racgap1	1.511	0.575	0.868	0.177	0.164	0.835	2.540	3.135	2.336	26934	Racgap1	chr15	-	NM_0120	P17158	protein_cc	AI227039|	Rac GTPas	Racgap1
7337	Asic1	5.135	0.492	2.526	0.051	0.215	0.423	0.001	2.361	1.338	11419	Asic1	chr15	+	NM_0095	P19571	protein_cc	Accn2| AI	acid-sensi	Asic1
7338	Smarcd1	1.085	1.172	1.271	0.622	0.081	0.442	3.893	4.010	4.238	83797	Smarcd1	chr15	+	NM_0318	P3587	protein_cc	AA407987	SWI/SNF r	Smarcd1
7339	Gpd1	0.056	4.126	0.230	0.000	0.000	0.001	10.695	6.529	8.573	14555	Gpd1	chr15	+	NM_0102	P3621	protein_cc	AI747587|	glycerol-3	Gpd1
7340	Cox14	0.740	1.406	1.040	0.054	0.035	0.609	9.360	8.925	9.416	66379	Cox14	chr15	+	NM_1832	P8541	protein_cc	2310016M	cytochrom	Cox14
7341	Cers5	1.644	1.184	1.947	0.149	0.445	0.009	4.995	5.713	5.956	71949	Cers5	chr15	-	NM_0280	P11976	protein_cc	2310081H	ceramide	Cers5
7342	Lima1	0.201	1.438	0.289	0.006	0.336	0.118	10.471	8.157	8.682	65970	Lima1	chr15	-	NM_0011	P24170	protein_cc	Eplin	LIM doma	Lima1
7343	1700030F	1.000	1.000	1.000	1.000	1.000	1.000	0.000	0.000	0.000	72277	1700030F	chr15	-	NM_0281	P5369	protein_cc	FAM186A	RIKEN cDN	1700030F1
7344	Larp4	1.027	1.287	1.322	0.736	0.173	0.350	8.617	8.656	9.020	207214	Larp4	chr15	+	NM_0010	P17174	protein_cc	DXErtd79	La ribonuc	Larp4
7345	2310068J1	0.936	1.757	1.645	0.416	0.058	0.274	3.251	3.156	3.969	70281	2310068J1	chr15	-	NR_02812	.	lncRNA	.	RIKEN cDN	2310068J1
7346	Dip2b	0.824	1.375	1.133	0.039	0.011	0.230	5.376	5.097	5.556	239667	Dip2b	chr15	+	NM_0011	P5072	protein_cc	4932422C	disco inter	Dip2b
7347	Atf1	1.352	1.065	1.440	0.000	0.705	0.338	7.454	7.889	7.979	11908	Atf1	chr15	+	NM_0074	P2332	protein_cc	.	activating	Atf1
7348	Tmprss12	1.000	1.000	1.000	1.000	1.000	1.000	0.000	0.000	0.000	75002	Tmprss12	chr15	+	NM_1831	P11347	protein_cc	4930478A	transmem	Tmprss12
7349	Mettl7a1	0.078	1.317	0.103	0.000	0.009	0.000	11.519	7.837	8.234	70152	Mettl7a1	chr15	+	NM_0273	P18352	protein_cc	2210414H	methyltra	Mettl7a1
7350	Mettl7a3	0.443	1.401	0.621	0.002	0.194	0.235	1.201	0.027	0.514	668178	Mettl7a3	chr15	+	NM_0010	P344	protein_cc	ENSMUSG	methyltra	Mettl7a3
7351	Methig1	1.000	1.000	1.000	1.000	1.000	1.000	0.000	0.000	0.000	554292	Methig1	chr15	+	NM_0010	P8517	protein_cc	Mettl7a2-	methyltra	Methig1
7352	Mettl7a2	0.079	1.342	0.106	0.001	0.018	0.042	9.678	6.012	6.436	393082	Mettl7a2	chr15	+	NM_1994	P2669	protein_cc	Gm921| U	methyltra	Mettl7a2
7353	Higd1c	1.000	1.000	1.000	1.000	1.000	1.000	0.000	0.000	0.000	380975	Higd1c	chr15	+	NM_0010	P10489	protein_cc	Gm921| H	HIG1 dom	Higd1c
7354	Slc11a2	6.814	0.824	5.616	0.000	0.042	0.017	7.514	10.282	10.003	18174	Slc11a2	chr15	-	NM_0087	P17495	protein_cc	DCT1| DM	solute car	Slc11a2
7355	Gm5475	1.488	3.410	5.074	0.526	0.267	0.454	0.588	1.162	2.932	432982	Gm5475	chr15	+	NR_04035	.	lncRNA	EG432982	predicted	Gm5475
7356	Letmd1	0.508	1.282	0.651	0.022	0.384	0.104	7.932	6.954	7.313	68614	Letmd1	chr15	+	NM_1340	P17410	protein_cc	1110019O	LETM1 do	Letmd1
7357	Csrnp2	1.397	0.761	1.063	0.311	0.393	0.641	3.855	4.338	3.943	207785	Csrnp2	chr15	-	NM_1534	P7817	protein_cc	Csrnp2| C	cysteine-s	Csrnp2
7358	Tfcp2	1.096	1.033	1.132	0.621	0.627	0.674	4.713	4.846	4.893	21422	Tfcp2	chr15	-	NM_0334	P6004	protein_cc	CP-2| CP2	transcripti	Tfcp2

7359	Pou6f1	0.931	2.198	2.046	0.818	0.067	0.310	4.407	4.304	5.440	19009	Pou6f1	chr15	-	NM_0101	P125	protein_cc	2310038G	POU doma	Pou6f1
7360	C330013E	0.785	1.572	1.235	0.256	0.036	0.575	3.771	3.423	4.076	78445	C330013E	chr15	-	NR_04570	.	lncRNA	.	RIKEN cDN	C330013E
7361	Dazap2	0.782	1.475	1.153	0.001	0.113	0.656	10.630	10.275	10.836	23994	Dazap2	chr15	+	NM_0118	P16190	protein_cc	AI314727	DAZ assoc	Dazap2
7362	Smagp	0.976	0.683	0.667	0.701	0.006	0.007	9.433	9.398	8.848	207818	Smagp	chr15	-	NR_10433	.	protein_cc	.	small cell	Smagp
7363	Bin2	0.882	1.247	1.099	0.129	0.456	0.842	4.729	4.548	4.866	668218	Bin2	chr15	-	NM_0012	P6324	protein_cc	.	bridging ir	Bin2
7364	Cela1	0.250	2.194	0.548	0.000	0.053	0.118	8.376	6.376	7.510	109901	Cela1	chr15	-	NM_0336	P540	protein_cc	1810009A	chymotryp	Cela1
7365	Galnt6	0.937	1.308	1.226	0.693	0.256	0.648	3.871	3.777	4.165	207839	Galnt6	chr15	-	NM_1724	P11603	protein_cc	4632410F	UDP-N-ac	Galnt6
7366	I730030J2	0.504	0.990	0.499	0.181	1.000	0.423	1.002	0.015	0.000	619313	I730030J2	chr15	-	NR_04578	.	lncRNA	.	RIKEN cDN	I730030J2
7367	Slc4a8	4.054	0.512	2.077	0.010	0.053	0.153	1.107	3.126	2.161	59033	Slc4a8	chr15	+	NM_0215	P9343	protein_cc	AW49384	solute car	Slc4a8
7368	Scn8a	37.051	0.924	34.246	0.000	0.678	0.076	2.095	7.307	7.193	20273	Scn8a	chr15	+	NM_0010	P23628	protein_cc	AI853486	sodium ch	Scn8a
7369	Fignl2	0.785	0.956	0.751	0.145	0.827	0.274	6.888	6.538	6.474	668225	Fignl2	chr15	-	NM_0012	P18172	protein_cc	EG668225	fidgetin-lik	Fignl2
7370	Ankrd33	1.000	1.000	1.000	1.000	1.000	1.000	0.000	0.000	0.000	208258	Ankrd33	chr15	+	NM_1447	P777	protein_cc	A930021G	ankyrin re	Ankrd33
7371	Acvrl1	1.500	0.672	1.008	0.059	0.153	0.984	4.999	5.584	5.011	11482	Acvrl1	chr15	+	NM_0096	P19873	protein_cc	Acvrlk1 | A	activin A r	Acvrl1
7372	Acvr1b	5.648	0.999	5.642	0.061	0.998	0.129	1.397	3.894	3.893	11479	Acvr1b	chr15	+	NM_0073	P5592	protein_cc	6820432J	activin A r	Acvr1b
7373	A330009N	0.157	3.225	0.508	0.068	0.017	0.481	2.756	0.088	1.778	380977	A330009N	chr15	-	NR_04532	.	lncRNA	.	RIKEN cDN	A330009N
7374	AU021063	0.228	2.926	0.667	0.182	0.077	0.730	2.204	0.071	1.620	105968	AU021063	chr15	+	NR_04599	.	lncRNA	.	expressed	AU021063
7375	Grasp	0.517	5.735	2.968	0.001	0.020	0.209	3.375	2.425	4.945	56149	Grasp	chr15	+	NM_0195	P20983	protein_cc	tamalin	GRP1 (ger	Grasp
7376	Nr4a1	1.146	0.401	0.459	0.228	0.001	0.086	6.429	6.626	5.307	15370	Nr4a1	chr15	+	NM_0104	P25267	protein_cc	Gfrp | GFR	nuclear re	Nr4a1
7377	Atg101	0.430	1.123	0.483	0.000	0.314	0.016	9.438	8.221	8.389	68118	Atg101	chr15	+	NM_0265	P6422	protein_cc	94300231	autophagy	Atg101
7378	6030408B	1.000	1.000	1.000	1.000	1.000	1.000	0.000	0.000	0.000	77717	6030408B	chr15	+	NR_03380	.	lncRNA	AI225877	RIKEN cDN	6030408B
7379	Krt80	1.000	1.000	1.000	1.000	1.000	1.000	0.000	0.000	0.000	74127	Krt80	chr15	-	NM_0287	P987	protein_cc	1200016G	keratin 80	Krt80
7380	Krt7	0.218	0.567	0.124	0.012	0.566	0.045	4.415	2.218	1.400	110310	Krt7	chr15	+	NM_0330	P10420	protein_cc	D15Wsu7	keratin 7	Krt7
7381	Krt85	1.000	1.000	1.000	1.000	1.000	1.000	0.000	0.000	0.000	.	Krt85	chr15	-	NM_0168	P19548	.	.	.	Krt85
7382	Krt83	1.000	1.000	1.000	1.000	1.000	1.000	0.000	0.000	0.000	10012622	Krt83	chr15	-	NM_0010	P17411	protein_cc	5430421N	keratin 83	Krt83
7383	1700011A	1.000	1.000	1.000	1.000	1.000	1.000	0.000	0.000	0.000	.	1700011A	chr15	+	NM_0254	P25533	.	.	.	1700011A
7384	Krt81	1.000	1.000	1.000	1.000	1.000	1.000	0.000	0.000	0.000	64818	Krt81	chr15	-	NM_0011	P17687	protein_cc	AA589625	keratin 81	Krt81
7385	Krt86	1.000	1.000	1.000	1.000	1.000	1.000	0.000	0.000	0.000	16679	Krt86	chr15	+	NM_0106	P20685	protein_cc	5430421N	keratin 86	Krt86
7386	5430421N	1.000	1.000	1.000	1.000	1.000	1.000	0.000	0.000	0.000	.	5430421N	chr15	-	NM_0012	P17629	.	.	.	5430421N
7387	Gm6042	1.000	1.000	1.000	1.000	1.000	1.000	0.000	0.000	0.000	574415	Gm6042	chr15	+	NR_00287	.	misc_RNA	EG574415	predicted	Gm6042
7388	Krt84	1.000	1.000	1.000	1.000	1.000	1.000	0.000	0.000	0.000	16680	Krt84	chr15	-	NM_0084	P19184	protein_cc	AV235125	keratin 84	Krt84
7389	Krt82	1.000	1.000	1.000	1.000	1.000	1.000	0.000	0.000	0.000	114566	Krt82	chr15	-	NM_0532	P20257	protein_cc	Krt2-20	keratin 82	Krt82
7390	4732456N	1.000	1.000	1.000	1.000	1.000	1.000	0.000	0.000	0.000	.	4732456N	chr15	-	NM_1777	P24185	.	.	.	4732456N
7391	Krt75	1.000	1.000	1.000	1.000	1.000	1.000	0.000	0.000	0.000	109052	Krt75	chr15	-	NM_1333	P16383	protein_cc	4732468K	keratin 75	Krt75
7392	Gm5476	1.000	1.000	1.000	1.000	1.000	1.000	0.000	0.000	0.000	432985	Gm5476	chr15	-	NR_00286	.	misc_RNA	EG432985	predicted	Gm5476
7393	Gm5477	1.000	1.000	1.000	1.000	1.000	1.000	0.000	0.000	0.000	432986	Gm5477	chr15	+	NR_00286	.	misc_RNA	EG432986	predicted	Gm5477
7394	Gm5414	1.000	1.000	1.000	1.000	1.000	1.000	0.000	0.000	0.000	406223	Gm5414	chr15	-	NM_0010	P15802	protein_cc	EG406223	predicted	Gm5414
7395	Gm5478	1.000	1.000	1.000	1.000	1.000	1.000	0.000	0.000	0.000	432987	Gm5478	chr15	-	NR_00396	.	misc_RNA	EG432987	predicted	Gm5478
7396	Krt6b	1.000	1.000	1.000	1.000	1.000	1.000	0.000	0.000	0.000	16688	Krt6b	chr15	-	NM_0106	P10985	protein_cc	BB144589	keratin 6B	Krt6b
7397	Krt6a	1.000	1.000	1.000	1.000	1.000	1.000	0.000	0.000	0.000	16687	Krt6a	chr15	-	NM_0084	P9165	protein_cc	2310016L	keratin 6A	Krt6a
7398	Krt5	1.000	1.000	1.000	1.000	1.000	1.000	0.000	0.000	0.000	110308	Krt5	chr15	-	NM_0270	P19839	protein_cc	3300001P	keratin 5	Krt5
7399	Krt71	1.000	1.000	1.000	1.000	1.000	1.000	0.000	0.000	0.000	56735	Krt71	chr15	-	NM_0199	P20153	protein_cc	AA589543	keratin 71	Krt71
7400	Krt74	1.000	1.000	1.000	1.000	1.000	1.000	0.000	0.000	0.000	406222	Krt74	chr15	-	NR_03344	.	misc_RNA	K74 | Kb37	keratin 74	Krt74
7401	Krt72	1.000	1.000	1.000	1.000	1.000	1.000	0.000	0.000	0.000	105866	Krt72	chr15	-	NM_2137	P5106	protein_cc	AI507495	keratin 72	Krt72
7402	Krt73	1.000	1.000	1.000	1.000	1.000	1.000	0.000	0.000	0.000	223915	Krt73	chr15	-	NM_2124	P9795	protein_cc	BC067067	keratin 73	Krt73
7403	Krt2	1.000	1.000	1.000	1.000	1.000	1.000	0.000	0.000	0.000	16681	Krt2	chr15	-	NM_0106	P22265	protein_cc	BB005427	keratin 2	Krt2
7404	Krt1	1.000	1.000	1.000	1.000	1.000	1.000	0.000	0.000	0.000	16678	Krt1	chr15	-	NM_0084	P4734	protein_cc	Krt-2.1 | K	keratin 1	Krt1
7405	Krt77	1.000	1.000	1.000	1.000	1.000	1.000	0.000	0.000	0.000	406220	Krt77	chr15	-	NM_0010	P13673	protein_cc	4732484G	keratin 77	Krt77
7406	Krt76	0.716	0.995	0.712	0.182	1.000	0.423	0.490	0.008	0.000	77055	Krt76	chr15	-	NM_0010	P24799	protein_cc	2310001L	keratin 76	Krt76
7407	Krt4	1.000	1.000	1.000	1.000	1.000	1.000	0.000	0.000	0.000	16682	Krt4	chr15	-	NM_0084	P23254	protein_cc	AW10809	keratin 4	Krt4
7408	Krt79	1.000	1.000	1.000	1.000	1.000	1.000	0.000	0.000	0.000	223917	Krt79	chr15	-	NM_1460	P6125	protein_cc	BC031593	keratin 79	Krt79
7409	Krt78	0.716	0.995	0.712	0.182	1.000	0.423	0.490	0.008	0.000	332131	Krt78	chr15	-	NM_2124	P23626	protein_cc	2310030B	keratin 78	Krt78
7410	Krt8	1.422	1.158	1.647	0.042	0.147	0.037	9.821	10.328	10.540	16691	Krt8	chr15	-	NM_0311	P8926	protein_cc	AA960620	keratin 8	Krt8
7411	Krt18	3.996	0.662	2.646	0.000	0.001	0.011	10.097	12.096	11.501	16668	Krt18	chr15	+	NM_0106	P9280	protein_cc	CK18 | K18	keratin 18	Krt18
7412	Eif4b	0.640	0.850	0.544	0.003	0.101	0.022	10.085	9.441	9.207	75705	Eif4b	chr15	+	NM_1456	P24227	protein_cc	2310046H	eukaryotic	Eif4b
7413	Tns2	0.343	1.841	0.632	0.000	0.029	0.121	7.744	6.201	7.081	209039	Tns2	chr15	+	NM_1535	P15165	protein_cc	C1-ten | ne	tensin 2	Tns2
7414	Spryd3	2.257	0.933	2.106	0.000	0.357	0.026	7.530	8.704	8.604	223918	Spryd3	chr15	-	NM_0010	P14246	protein_cc	BC008150	SPRY dom	Spryd3
7415	Igfbp6	4.168	0.682	2.843	0.349	0.679	0.141	1.644	3.704	3.151	16012	Igfbp6	chr15	+	NM_0083	P24086	protein_cc	IGFBP-6	insulin-like	Igfbp6
7416	Soat2	0.979	1.202	1.177	0.869	0.468	0.720	7.271	7.241	7.506	223920	Soat2	chr15	+	NM_1460	P11104	protein_cc	ACAT2 | D	sterol O-ac	Soat2
7417	Csad	0.358	1.852	0.664	0.000	0.002	0.058	10.451	8.970	9.860	246277	Csad	chr15	-	NM_1449	P16087	protein_cc	Csd	cysteine s	Csad
7418	Zfp740	0.980	1.030	1.010	0.909	0.842	0.953	6.586	6.557	6.600	68744	Zfp740	chr15	+	NM_0012	P4657	protein_cc	1110034O	zinc finger	Zfp740
7419	Itgb7	1.152	0.956	1.101	0.675	0.854	0.830	3.033	3.237	3.173	16421	Itgb7	chr15	-	NM_0135	P2275	protein_cc	Ly69	integrin be	Itgb7
7420	Rarg	0.694	0.629	0.436	0.229	0.412	0.232	5.380	4.852	4.182	19411	Rarg	chr15	-	NM_0010	P7878	protein_cc	Nr1b3 | RA	retinoic ac	Rarg
7421	Mfsd5	1.320	1.228	1.621	0.236	0.261	0.203	8.007	8.408	8.704	106073	Mfsd5	chr15	+	NM_1341	P2730	protein_cc	AA409375	major faci	Mfsd5
7422	Espl1	2.987	0.466	1.391	0.006	0.038	0.622	0.401	1.980	0.878	105988	Espl1	chr15	+	NM_0010	P13198	protein_cc	AL024103	extra spin	Espl1
7423	Pfdn5	0.708	1.489	1.054	0.001	0.002	0.488	9.625	9.127	9.701	56612	Pfdn5	chr15	+	NM_0270	P111	protein_cc	1190001O	prefoldin	Pfdn5
7424	Myg1	0.772	1.469	1.134	0.331	0.118	0.643	7.178	6.805	7.360	60315	Myg1	chr15	+	NM_0217	P8771	protein_cc	0610023A	melanocyt	Myg1
7425	Aaas	3.003	0.689	2.070	0.000	0.000	0.000	6.394	7.980	7.444	223921	Aaas	chr15	-	NM_1534	P7726	protein_cc	AAA | ADR	achalasia,	Aaas
7426	Sp7	1.010	1.885	1.904	0.000	0.182	0.423	0.000	0.015	0.929	170574	Sp7	chr15	-	NM_1304	P4815	protein_cc	6430578P	Sp7 transc	Sp7
7427	Sp1	0.983	0.710	0.697	0.863	0.020	0.255	9.509	9.484	8.989	20683	Sp1	chr15	+	NM_0136	P5635	protein_cc	1110003E	trans-acti	Sp1
7428	Amhr2	0.272	0.609	0.165	0.029	1.000	0.126	2.595	0.715	0.000	110542	Amhr2	chr15	+	NM_1445	P7708	protein_cc	Misiir | Mi	anti-Mulle	Amhr2
7429	Prr13	1.097	0.979	1.074	0.192	0.711	0.189	9.340	9.474	9.443	66151	Prr13	chr15	+	NM_0011	P20021	protein_cc	1110020C	proline ric	Prr13
7430	Pcbp2	0.862	0.822	0.708	0.705	0.616	0.472	11.433	11.219	10.936	18521	Pcbp2	chr15	+	NM_0110	P650	protein_cc	alphaCP-2	poly(rC) bi	Pcbp2
7431	Map3k12	0.879	0.810	0.712	0.757	0.610	0.505	11.328	11.143	10.838	26404	Map3k12	chr15	-	NM_0095	P21418	protein_cc	DLK | MUK	mitogen-a	Map3k12
7432	Tarbp2	1.132	1.487	1.683	0.346	0.011	0.011	5.907	6.086	6.658	21357	Tarbp2	chr15	+	NM_0012	P15285	protein_cc	Prbp | TRB	TAR (HIV)	Tarbp2
7433	Npff	1.426	2.131	3.039	0.348	0.233	0.395	1.004	1.516	2.608	54615	Npff	chr15	-	NR_03340	.	protein_cc	.	neuropept	Npff
7434	Atf7	0.862	0.799	0.689	0.108	0.000	0.104	7.403	7.189	6.864	223922	Atf7	chr15	-	NM_0013	P24186	protein_cc	1110012F	activating	Atf7
7435	Atp5g2	0.668	1.350	0.902	0.001	0.015	0.282	9.323	8.742	9.175	67942	Atp5g2	chr15	-	NM_0264	P24109	protein_cc	1810041M	ATP synth	Atp5g2
7436	Calcoco1	0.098	2.958	0.291	0.018	0.057	0.210	10.016	6.671	8.235	67488	Calcoco1	chr15	-	NM_0261	P3785	protein_cc	1810009B	calcium bi	Calcoco1
7437	Gm38436	1.000	1.000	1.000	1.000	1.000	1.000	0.000	0.000	0.000	10221626	Gm38436	chr15	-	NR_10433	.	lncRNA	Cistr | Cist	predicted	Gm38436
7438	Hoxc13	1.005	1.405	1.412	0.000	0.182	0.423	0.000	0.008	0.498	15422	Hoxc13	chr15	+	NM_0104	P8255	protein_cc	.	homeobox	Hoxc13
7439	Hoxc12	1.000	1.000	1.000	1.000	1.000	1.000	0.000	0.000	0.000	15421	Hoxc12	chr15	+	NM_0104	P2975	protein_cc	Hox-3.8	homeobox	Hoxc12
7440	Hotair	1.000	1.000	1.000	1.000	1.000	1.000	0.000	0.000	0.000	10050387	Hotair	chr15	-	NR_04752	.	lncRNA	Gm16258	HOX trans	Hotair
7441	Hoxc11	1.000	1.000	1.000	1.000	1.000	1.000	0.000	0.000	0.000	109663	Hoxc11	chr15	+	NM_0010	P25233	protein_cc	Hox-3.7	homeobox	Hoxc11
7442	Hoxc10	1.019	2.651	2.701	0.000	0.000	0.002	0.000	0.027	1.434	209448	Hoxc10	chr15	+	NM_0104	P18191	protein_cc	AI506621	homeobox	Hoxc10
7443	Hoxc9	1.010	1.825	1.844	0.000	0.182	0.423	0.000	0.015	0.883	15427	Hoxc9	chr15	+	NM_0082	P9849	protein_cc	Hox-3.2	homeobox	Hoxc9
7444	Hoxc8	1.000	1.000	1.000	1.000	1.000	1.000	0.000	0.000	0.000	15426	Hoxc8	chr15	+	NM_0104	P16205	protein_cc	D130011F	homeobox	Hoxc8
7445	Hoxc6	0.509	1.780	0.906	0.184	0.186	0.897	1.002	0.028	0.859	15425	Hoxc6	chr15	+	NM_0104	P9976	protein_cc	Hox-3.3 | H	homeobox	Hoxc6
7446	Hoxc5	1.000	1.000	1.000	1.000	1.000	1.000	0.000	0.000	0.000	15424	Hoxc5	chr15	+	NM_1757	P2232	protein_cc	AI181837	homeobox	Hoxc5
7447	Hoxc4	1.000	1.000	1.000	1.000	1.000	1.000	0.000	0.000	0.000	15423	Hoxc4	chr15	+	NM_0135	P9202	protein_cc	Hox-3.5	homeobox	Hoxc4
7448	D930007P	1.000	1.000	1.000	1.000	1.000	1.000	0.000	0.000	0.000	432989	D930007P	chr15	-	NR_04574	.	lncRNA	.	Riken cDN	D930007P
7449	Smug1	2.080	0.960	1.998	0.144	0.893	0.051	6.112	7.169	7.111	71726	Smug1	chr15	-	NM_0278	P15570	protein_cc	1200013B	single-stra	Smug1

7450	Cbx5	0.606	1.348	0.817	0.000	0.079	0.335	8.672	7.950	8.380	12419	Cbx5	chr15	-	NM_0011	P12337	protein_cc	2610029O	chromobo	Cbx5
7451	Hnrnpa1	1.070	0.786	0.841	0.814	0.545	0.713	5.649	5.746	5.398	15382	Hnrnpa1	chr15	+	NR_10442	.	protein_cc	Hdp| HDP	heterogen	Hnrnpa1
7452	Gm10052	1.193	1.601	1.910	0.759	0.409	0.399	2.144	2.399	3.078	654467	Gm10052	chr15	+	NR_00288	.	misc_RNA	654467| E	predicted	Gm10052
7453	Gm5643	0.163	2.462	0.402	0.023	0.056	0.280	2.695	0.081	1.380	434858	Gm5643	chr15	+	NR_00288	.	misc_RNA	EG434858	predicted	Gm5643
7454	Nfe2	7.967	1.200	9.564	0.046	0.572	0.031	0.704	3.698	3.962	18022	Nfe2	chr15	-	NM_0013	P8662	protein_cc	NF-E2| NF	nuclear fa	Nfe2
7455	Copz1	0.856	1.096	0.938	0.092	0.210	0.393	10.490	10.266	10.398	56447	Copz1	chr15	+	NM_0198	P12800	protein_cc	5930435A	coatomer	Copz1
7456	Gpr84	36.954	0.452	16.688	0.000	0.038	0.283	0.003	5.210	4.063	80910	Gpr84	chr15	-	NM_0307	P11662	protein_cc	EX33	G protein-	Gpr84
7457	Zfp385a	0.882	0.794	0.700	0.493	0.001	0.359	5.822	5.641	5.308	29813	Zfp385a	chr15	-	NM_0138	P13540	protein_cc	Hzf| Zfp38	zinc finger	Zfp385a
7458	Itga5	2.576	0.571	1.472	0.046	0.088	0.381	3.590	4.955	4.147	16402	Itga5	chr15	-	NM_0105	P12109	protein_cc	Cd49e| Fn	integrin al	Itga5
7459	Gtsf1	1.000	1.000	1.000	1.000	1.000	1.000	0.000	0.000	0.000	74174	Gtsf1	chr15	-	NM_0287	P9194	protein_cc	1700006H	gametocy	Gtsf1
7460	BC048502	1.000	1.000	1.000	1.000	1.000	1.000	0.000	0.000	0.000	223927	BC048502	chr15	-	NM_1776	P1924	protein_cc	.	cDNA seq	BC048502
7461	Nckap1l	1.764	0.926	1.633	0.003	0.750	0.404	5.031	5.850	5.739	105855	Nckap1l	chr15	+	NM_1535	P26145	protein_cc	4930568P	NCK assoc	Nckap1l
7462	Pde1b	1.062	1.294	1.374	0.940	0.627	0.706	2.591	2.677	3.049	18574	Pde1b	chr15	+	NM_0088	P11648	protein_cc	Pde1b1	phosphod	Pde1b
7463	Ppp1r1a	1.000	1.000	1.000	1.000	1.000	1.000	0.000	0.000	0.000	58200	Ppp1r1a	chr15	-	NM_0213	P8563	protein_cc	0610038N	protein ph	Ppp1r1a
7464	Glycam1	1.000	1.000	1.000	1.000	1.000	1.000	0.000	0.000	0.000	14663	Glycam1	chr15	-	NM_0081	P335	protein_cc	glyCAM-1	glycosylat	Glycam1
7465	Mucl1	1.000	1.000	1.000	1.000	1.000	1.000	0.000	0.000	0.000	20771	Mucl1	chr15	-	NR_12608	.	misc_RNA	Spt-2| Spt	mucin-like	Mucl1
7466	Spt1	1.000	1.000	1.000	1.000	1.000	1.000	0.000	0.000	0.000	.	Spt1	chr15	-	NM_0092	P15869	.	.	.	Spt1
7467	Olfr161	1.000	1.000	1.000	1.000	1.000	1.000	0.000	0.000	0.000	258859	Olfr161	chr16	+	NM_1468	P23523	protein_cc	MOR131-1	olfactory r	Olfr161
7468	Mefv	90.632	0.278	25.202	0.001	0.007	0.213	0.003	6.505	4.658	54483	Mefv	chr16	-	NM_0194	P17947	protein_cc	FMF| pyri	Mediterra	Mefv
7469	Zfp263	1.226	0.456	0.559	0.156	0.009	0.040	6.480	6.774	5.640	74120	Zfp263	chr16	+	NM_1489	P18022	protein_cc	1200014J0	zinc finger	Zfp263
7470	Olfr15	1.000	1.000	1.000	1.000	1.000	1.000	0.000	0.000	0.000	18312	Olfr15	chr16	+	NM_0087	P10739	protein_cc	MOR256-1	olfactory r	Olfr15
7471	Zfp174	0.439	1.897	0.833	0.026	0.057	0.623	3.421	2.234	3.158	385674	Zfp174	chr16	+	NM_0010	P15143	protein_cc	Gm1754	zinc finger	Zfp174
7472	Zfp597	1.292	0.840	1.085	0.173	0.469	0.814	6.182	6.551	6.299	71063	Zfp597	chr16	-	NM_0010	P526	protein_cc	4933407K	zinc finger	Zfp597
7473	Naa60	0.298	1.400	0.418	0.000	0.280	0.073	9.511	7.766	8.251	74763	Naa60	chr16	+	NM_0012	P19389	protein_cc	1200013P	N(alpha)-a	Naa60
7474	1700037C	5.915	0.584	3.456	0.056	0.251	0.006	1.900	4.464	3.689	73261	1700037C	chr16	-	NM_0284	P24326	protein_cc	2810022L	RIKEN cDN	1700037C
7475	Cluap1	0.984	0.650	0.640	0.914	0.000	0.250	6.831	6.807	6.187	76779	Cluap1	chr16	+	NM_0297	P14257	protein_cc	2310030D	clusterin a	Cluap1
7476	Nlrc3	1.000	1.000	1.000	1.000	1.000	1.000	0.000	0.000	0.000	268857	Nlrc3	chr16	-	NM_0010	P2160	protein_cc	CLR16.2| I	NLR family	Nlrc3
7477	Slx4	2.207	1.198	2.644	0.001	0.041	0.002	3.243	4.385	4.645	52864	Slx4	chr16	-	NM_1774	P3247	protein_cc	AI256635|	SLX4 struc	Slx4
7478	Dnase1	0.704	1.203	0.847	0.003	0.003	0.207	8.403	7.897	8.164	13419	Dnase1	chr16	+	NM_0100	P936	protein_cc	AI788650|	deoxyribo	Dnase1
7479	Trap1	0.724	0.961	0.696	0.001	0.430	0.002	9.761	9.296	9.237	68015	Trap1	chr16	-	NM_0265	P11492	protein_cc	2410002K	TNF recep	Trap1
7480	Crebbp	1.085	0.614	0.666	0.385	0.006	0.066	9.229	9.347	8.643	12914	Crebbp	chr16	-	NM_0010	P15848	protein_cc	AW55829	CREB bind	Crebbp
7481	4930455F	1.000	1.000	1.000	1.000	1.000	1.000	0.000	0.000	0.000	76397	4930455F	chr16	-	NR_04057	.	lncRNA	1700013J1	RIKEN cDN	4930455F1
7482	Gm5766	0.725	1.234	0.894	0.416	0.603	0.501	3.622	3.158	3.461	436332	Gm5766	chr16	+	NR_00362	.	misc_RNA	EG436332	predicted	Gm5766
7483	Adcy9	0.324	0.806	0.261	0.000	0.187	0.007	8.692	7.066	6.754	11515	Adcy9	chr16	-	NM_0012	P7090	protein_cc	AC9| ACtp	adenylate	Adcy9
7484	Srl	0.082	3.055	0.250	0.004	0.000	0.092	3.732	0.119	1.730	106393	Srl	chr16	-	NM_1753	P14697	protein_cc	9830004M	sarcalume	Srl
7485	Tfap4	0.132	2.248	0.296	0.001	0.223	0.096	5.885	2.962	4.131	83383	Tfap4	chr16	-	NM_0311	P25229	protein_cc	AI642933|	transcripti	Tfap4
7486	Glis2	0.394	0.943	0.372	0.001	0.917	0.189	4.172	2.829	2.745	83396	Glis2	chr16	+	NM_0311	P17847	protein_cc	Gli5| Klf16	GLIS famil	Glis2
7487	Pam16	1.241	1.091	1.354	0.255	0.610	0.234	8.505	8.817	8.942	66449	Pam16	chr16	-	NM_0255	P13503	protein_cc	2010110I0	presequen	Pam16
7488	Coro7	0.393	1.223	0.481	0.010	0.527	0.020	6.427	5.080	5.371	78885	Coro7	chr16	-	NM_0302	P7420	protein_cc	0610011B	coronin 7	Coro7
7489	Vasn	0.435	1.595	0.694	0.000	0.188	0.372	6.222	5.021	5.695	246154	Vasn	chr16	+	NM_1393	P24116	protein_cc	2610528G	vasorin	Vasn
7490	Dnaja3	0.939	0.826	0.775	0.567	0.276	0.323	9.305	9.213	8.938	83945	Dnaja3	chr16	+	NM_0236	P19723	protein_cc	1200003J1	DnaJ heat	Dnaja3
7491	Nmral1	0.937	0.976	0.915	0.874	0.947	0.823	6.082	5.989	5.954	67824	Nmral1	chr16	-	NM_0012	P2702	protein_cc	1110025F	NmrA-like	Nmral1
7492	Hmox2	0.805	1.084	0.872	0.228	0.598	0.442	10.002	9.690	9.806	15369	Hmox2	chr16	+	NM_0011	P18759	protein_cc	HO-2| HO	heme oxy	Hmox2
7493	Cdip1	0.693	1.267	0.878	0.010	0.023	0.227	10.818	10.289	10.630	66626	Cdip1	chr16	-	NM_0256	P5366	protein_cc	2700048O	cell death	Cdip1
7494	4930562C	1.000	1.000	1.000	1.000	1.000	1.000	0.000	0.000	0.000	78809	4930562C	chr16	+	NM_0301	P18813	protein_cc	.	RIKEN cDN	4930562C
7495	Ubald1	1.749	0.449	0.786	0.003	0.001	0.012	8.176	8.983	7.829	207740	Ubald1	chr16	-	NM_1453	P9990	protein_cc	1500031H	UBA-like d	Ubald1
7496	Mgrn1	0.403	1.482	0.597	0.000	0.002	0.039	8.961	7.650	8.217	17237	Mgrn1	chr16	+	NM_0296	P17344	protein_cc	2610042J	mahoguni	Mgrn1
7497	Gm16861	0.730	1.089	0.796	0.636	0.892	0.792	1.934	1.480	1.604	10050370	Gm16861	chr16	-	NR_03766	.	lncRNA	.	predicted	Gm16861
7498	Nudt16l1	0.799	1.118	0.893	0.204	0.467	0.452	8.857	8.533	8.694	66911	Nudt16l1	chr16	+	NM_0258	P11909	protein_cc	1110001K	nudix (nuc	Nudt16l1
7499	Anks3	1.194	1.160	1.385	0.249	0.634	0.603	5.098	5.353	5.567	72615	Anks3	chr16	-	NM_0283	P19595	protein_cc	2700067D	ankyrin re	Anks3
7500	4930451G	0.175	1.282	0.225	0.002	0.231	0.053	2.578	0.065	0.424	74684	4930451G	chr16	+	NM_0012	P19987	misc_RNA	.	RIKEN cDN	4930451G
7501	Sept12	0.534	0.990	0.528	0.000	1.000	0.015	0.921	0.015	0.000	71089	Sept12	chr16	-	NM_0276	P12580	protein_cc	1700028G	septin 12	Sept12
7502	Smim22	0.376	1.162	0.437	0.004	0.587	0.003	8.262	6.850	7.066	432995	Smim22	chr16	+	NM_0012	P21225	protein_cc	EG432995	small integ	Smim22
7503	Rogdi	0.372	1.862	0.693	0.009	0.070	0.311	6.370	4.944	5.841	66049	Rogdi	chr16	-	NM_1331	P9224	protein_cc	0610011C	rogdi hom	Rogdi
7504	Glyr1	1.414	0.812	1.148	0.005	0.007	0.413	9.427	9.928	9.626	74022	Glyr1	chr16	-	NM_0010	P10928	protein_cc	2810419J	glyoxylate	Glyr1
7505	Ubn1	1.334	0.650	0.867	0.017	0.039	0.678	9.348	9.765	9.142	170644	Ubn1	chr16	+	NM_0266	P20249	protein_cc	1110029L	ubinuclein	Ubn1
7506	Ppl	0.228	1.518	0.346	0.001	0.240	0.080	6.487	4.353	4.955	19041	Ppl	chr16	-	NM_0089	P4227	protein_cc	AW55387	periplakin	Ppl
7507	Sec14l5	1.000	1.000	1.000	1.000	1.000	1.000	0.000	0.000	0.000	665119	Sec14l5	chr16	+	NM_0011	P17442	protein_cc	.	SEC14-like	Sec14l5
7508	Nagpa	0.921	0.920	0.847	0.444	0.612	0.446	8.489	8.370	8.250	27426	Nagpa	chr16	-	NM_0137	P21970	protein_cc	AI596180|	N-acetylgl	Nagpa
7509	AU021092	0.154	0.577	0.089	0.036	0.292	0.174	4.526	1.829	1.035	239691	AU021092	chr16	-	NM_0010	P10392	protein_cc	.	expressed	AU021092
7510	Alg1	1.082	0.996	1.078	0.542	0.975	0.368	8.219	8.334	8.328	208211	Alg1	chr16	+	NM_1453	P20237	protein_cc	HMAT1| H	asparagine	Alg1
7511	Eef2kmt	1.172	1.183	1.387	0.226	0.293	0.210	8.634	8.864	9.106	70511	Eef2kmt	chr16	-	NM_0274	P24659	protein_cc	5730409G	eukaryotic	Eef2kmt
7512	Rbfox1	1.015	2.229	2.261	0.000	0.007	0.086	0.000	0.021	1.177	268859	Rbfox1	chr16	+	NM_0214	P14402	protein_cc	A2bp| A2b	RNA bindi	Rbfox1
7513	1700123O	1.000	1.000	1.000	1.000	1.000	1.000	0.000	0.000	0.000	68238	1700123O	chr16	+	NR_04579	.	lncRNA	.	RIKEN cDN	1700123O
7514	Tmem114	1.000	1.000	1.000	1.000	1.000	1.000	0.000	0.000	0.000	74720	Tmem114	chr16	-	NM_0290	P5063	protein_cc	4930511J1	transmem	Tmem114
7515	Mettl22	0.500	0.983	0.492	0.036	0.937	0.145	5.996	4.997	4.973	239706	Mettl22	chr16	+	NM_1462	P13378	protein_cc	.	methyltra	Mettl22
7516	Abat	0.156	0.873	0.136	0.000	0.372	0.001	11.040	8.363	8.166	268860	Abat	chr16	+	NM_1729	P1191	protein_cc	9630038C	4-aminobu	Abat
7517	Tmem186	1.245	1.234	1.537	0.239	0.147	0.219	5.705	6.021	6.325	66690	Tmem186	chr16	-	NM_0257	P12491	protein_cc	2810440M	transmem	Tmem186
7518	Pmm2	1.341	1.394	1.870	0.170	0.026	0.066	7.369	7.792	8.272	54128	Pmm2	chr16	+	NM_0168	P9099	protein_cc	AI585868|	phosphom	Pmm2
7519	Carhsp1	1.555	1.148	1.786	0.014	0.329	0.096	9.153	9.790	9.989	52502	Carhsp1	chr16	-	NM_0258	P24489	protein_cc	1200011K	calcium re	Carhsp1
7520	Usp7	1.023	0.975	0.997	0.833	0.825	0.976	9.692	9.724	9.688	252870	Usp7	chr16	-	NM_0010	P755	protein_cc	2210010O	ubiquitin s	Usp7
7521	1810013L	0.957	1.024	0.980	0.668	0.731	0.878	8.520	8.457	8.492	69053	1810013L	chr16	+	NM_0010	P23223	protein_cc	1110017P	RIKEN cDN	1810013L2
7522	Grin2a	1.000	1.000	1.000	1.000	1.000	1.000	0.000	0.000	0.000	14811	Grin2a	chr16	-	NM_0081	P23730	protein_cc	GluN2A| N	glutamate	Grin2a
7523	Rpl39l	0.540	1.145	0.618	0.356	0.762	0.651	1.586	0.697	0.893	68172	Rpl39l	chr16	+	NM_0265	P9714	protein_cc	4930517K	ribosomal	Rpl39l
7524	Atf7ip2	1.019	2.648	2.698	0.000	0.182	0.423	0.000	0.027	1.432	75329	Atf7ip2	chr16	+	NM_0292	P21236	protein_cc	4930558K	activating	Atf7ip2
7525	Emp2	0.244	0.969	0.237	0.000	0.866	0.016	7.003	4.971	4.926	13731	Emp2	chr16	-	NM_0079	P1325	protein_cc	XMP	epithelial	Emp2
7526	Gm1600	0.118	0.942	0.111	0.031	1.000	0.183	3.172	0.086	0.000	381033	Gm1600	chr16	-	NR_12606	.	lncRNA	Gm1776	predicted	Gm1600
7527	Tekt5	1.000	1.000	1.000	1.000	1.000	1.000	0.000	0.000	0.000	70426	Tekt5	chr16	-	NM_0012	P26082	protein_cc	3300001K	tektin 5	Tekt5
7528	Nubp1	1.410	2.012	2.837	0.002	0.000	0.003	7.530	8.025	9.034	26425	Nubp1	chr16	+	NM_0119	P10596	protein_cc	.	nucleotide	Nubp1
7529	Tvp23a	1.369	1.356	1.855	0.372	0.264	0.416	3.610	4.062	4.501	383103	Tvp23a	chr16	-	NM_0010	P18728	protein_cc	Fam18a	trans-golg	Tvp23a
7530	Ciita	2.204	0.588	1.295	0.002	0.002	0.472	1.662	2.802	2.035	12265	Ciita	chr16	+	NM_0012	P2978	protein_cc	C2ta| EG6	class II tra	Ciita
7531	Dexi	0.546	0.793	0.433	0.005	0.007	0.049	9.103	8.230	7.896	58239	Dexi	chr16	-	NM_0214	P12822	protein_cc	1810029J1	dexameth	Dexi
7532	Clec16a	1.009	1.651	1.666	0.978	0.142	0.143	5.623	5.637	6.360	74374	Clec16a	chr16	+	NM_0012	P2744	protein_cc	4932416N	C-type lec	Clec16a
7533	Socs1	128.264	0.261	33.535	0.006	0.020	0.091	3.585	10.588	8.653	12703	Socs1	chr16	-	NM_0098	P8360	protein_cc	Cish1| Cis	suppresso	Socs1
7534	Tnp2	2.149	0.465	1.000	0.306	1.000	0.423	0.000	1.104	0.000	21959	Tnp2	chr16	-	NM_0136	P19431	protein_cc	Tp-2| TP2	transition	Tnp2
7535	Prm3	1.000	1.000	1.000	1.000	1.000	1.000	0.000	0.000	0.000	19120	Prm3	chr16	-	NM_0136	P13709	protein_cc	BC061127	protamine	Prm3
7536	Prm2	1.000	1.000	1.000	1.000	1.000	1.000	0.000	0.000	0.000	19119	Prm2	chr16	-	NM_0089	P5461	protein_cc	AI528784|	protamine	Prm2
7537	Prm1	3.438	0.145	0.499	0.015	1.000	0.423	1.004	2.786	0.000	19118	Prm1	chr16	-	NM_0136	P11082	protein_cc	Prm-1	protamine	Prm1
7538	Rmi2	8.234	0.237	1.952	0.002	0.008	0.570	1.243	4.284	2.208	223970	Rmi2	chr16	+	NM_0011	P3419	protein_cc	A630055G	RecQ med	Rmi2
7539	Gm21859	1.000	1.000	1.000	1.000	1.000	1.000	0.000	0.000	0.000	545180	Gm21859	chr16	-	NR_13113	.	lncRNA	.	predicted	Gm21859
7540	Litaf	7.468	0.683	5.099	0.000	0.034	0.051	9.118	12.019	11.469	56722	Litaf	chr16	-	NM_0199	P22609	protein_cc	3222402J1	LPS-induce	Litaf

7541	Gm4262	42.320	0.472	19.956	0.001	0.043	0.169	0.003	5.406	4.321	10004314	Gm4262	chr16	+	NR_04051	.	lncRNA	10004314	predicted	Gm4262
7542	Snn	1.315	0.987	1.298	0.133	0.901	0.226	6.509	6.904	6.885	20621	Snn	chr16	+	NM_0092	P20814	protein_cc	2810407J(	stannin	Snn
7543	Txndc11	1.351	0.967	1.307	0.079	0.750	0.134	7.129	7.563	7.515	106200	Txndc11	chr16	-	NM_1341	P1959	protein_cc	2810028E	thioredoxi	Txndc11
7544	Zc3h7a	2.620	0.689	1.805	0.001	0.009	0.000	6.696	8.086	7.548	106205	Zc3h7a	chr16	-	NR_02750	.	protein_cc	A430104C	zinc finger	Zc3h7a
7545	Rsl1d1	1.543	1.088	1.679	0.000	0.366	0.084	8.934	9.560	9.682	66409	Rsl1d1	chr16	-	NM_0255	P2911	protein_cc	2410005K	ribosomal	Rsl1d1
7546	2610020C	1.160	0.743	0.861	0.414	0.553	0.860	2.601	2.815	2.386	69918	2610020C	chr16	+	NR_03815	.	lncRNA	.	RIKEN cDN	2610020C
7547	Gspt1	1.004	0.887	0.890	0.985	0.559	0.344	9.493	9.499	9.326	14852	Gspt1	chr16	-	NM_1460	P16331	protein_cc	AI314175 |	G1 to S ph	Gspt1
7548	Tnfrsf17	1.000	1.000	1.000	1.000	1.000	1.000	0.000	0.000	0.000	21935	Tnfrsf17	chr16	+	NM_0116	P7513	protein_cc	BCM | BCN	tumor nec	Tnfrsf17
7549	Snx29	0.509	3.518	1.789	0.342	0.016	0.441	1.720	0.744	2.559	74478	Snx29	chr16	+	NM_0289	P21877	protein_cc	4933437K	sorting ne	Snx29
7550	4833415N	0.716	0.995	0.712	0.182	1.000	0.423	0.490	0.008	0.000	73761	4833415N	chr16	+	NR_12643	.	lncRNA	.	RIKEN cDN	4833415N
7551	Cpped1	0.427	1.293	0.552	0.017	0.416	0.055	9.075	7.847	8.217	223978	Cpped1	chr16	-	NM_1460	P11655	protein_cc	C530044N	calcineuri	Cpped1
7552	Gm9961	1.000	1.000	1.000	1.000	1.000	1.000	0.000	0.000	0.000	791359	Gm9961	chr16	+	NR_03350	.	lncRNA	ENSMUSG	predicted	Gm9961
7553	Shisa9	1.000	1.000	1.000	1.000	1.000	1.000	0.000	0.000	0.000	72555	Shisa9	chr16	+	NM_0282	P15825	protein_cc	2700045P	shisa fami	Shisa9
7554	1700003L	1.000	1.000	1.000	1.000	1.000	1.000	0.000	0.000	0.000	75493	1700003L	chr16	+	NR_04050	.	lncRNA	.	RIKEN cDN	1700003L1
7555	Ercc4	1.038	1.028	1.067	0.814	0.834	0.839	5.827	5.881	5.920	50505	Ercc4	chr16	+	NM_0157	P9085	protein_cc	AI606920 |	excision re	Ercc4
7556	Mkl2	0.805	1.201	0.966	0.121	0.050	0.826	7.007	6.694	6.958	239719	Mkl2	chr16	+	NM_0011	P21296	protein_cc	ENSMUSG	MKL/myo	Mkl2
7557	2310015D	1.000	1.000	1.000	1.000	1.000	1.000	0.000	0.000	0.000	70100	2310015D	chr16	+	NR_03799	.	lncRNA	.	RIKEN cDN	2310015D
7558	Parn	0.580	1.672	0.970	0.004	0.006	0.862	7.516	6.730	7.472	74108	Parn	chr16	-	NM_0287	P5655	protein_cc	1200003I1	poly(A)-sp	Parn
7559	Bfar	1.071	0.756	0.810	0.003	0.013	0.203	10.300	10.400	9.995	67118	Bfar	chr16	+	NM_0011	P11537	protein_cc	3010001A	bifunction	Bfar
7560	3110001I2	1.892	1.159	2.193	0.321	0.709	0.098	4.831	5.750	5.963	66598	3110001I2	chr16	+	NM_0256	P10161	protein_cc	.	RIKEN cDN	3110001I2
7561	Pla2g10	1.000	1.000	1.000	1.000	1.000	1.000	0.000	0.000	0.000	26565	Pla2g10	chr16	-	NM_0119	P4646	protein_cc	GX sPLA2 |	phospholi	Pla2g10
7562	Pla2g10os	1.005	1.439	1.446	0.000	0.182	0.423	0.000	0.008	0.532	76684	Pla2g10os	chr16	+	NR_04057	.	lncRNA	1810007I(	phospholi	Pla2g10os
7563	Rrn3	1.825	0.829	1.513	0.001	0.009	0.091	7.099	7.967	7.696	106298	Rrn3	chr16	+	NM_0010	P7061	protein_cc	AL023001	RRN3 RNA	Rrn3
7564	Ntan1	0.287	1.997	0.573	0.000	0.007	0.034	7.397	5.594	6.592	18203	Ntan1	chr16	+	NM_0109	P19838	protein_cc	.	N-termina	Ntan1
7565	Pdxdc1	0.986	0.893	0.880	0.823	0.151	0.027	8.567	8.546	8.383	94184	Pdxdc1	chr16	-	NM_0010	P16438	protein_cc	2210010A	pyridoxal-	Pdxdc1
7566	Mpv17l	0.215	2.021	0.434	0.000	0.011	0.020	6.813	4.593	5.608	93734	Mpv17l	chr16	+	NM_0012	P14969	protein_cc	M-LP	Mpv17 tra	Mpv17l
7567	Ifitm7	1.183	1.397	1.652	0.672	0.338	0.288	0.439	0.682	1.164	74482	Ifitm7	chr16	-	NM_0012	P11395	protein_cc	4933438K	interferon	Ifitm7
7568	2900011O	0.696	1.882	1.309	0.336	0.077	0.490	1.226	0.702	1.614	67254	2900011O	chr16	+	NM_1445	P6091	protein_cc	MINP	RIKEN cDN	2900011O
7569	Marf1	0.534	1.440	0.769	0.012	0.310	0.498	7.663	6.758	7.284	223989	Marf1	chr16	-	NM_0010	P23227	protein_cc	4921513D	meiosis ar	Marf1
7570	Nde1	0.810	1.673	1.354	0.131	0.004	0.038	8.693	8.388	9.130	67203	Nde1	chr16	+	NM_0011	P10872	protein_cc	2810027M	nudE neur	Nde1
7571	Myh11	0.082	11.487	0.945	0.008	0.088	0.945	4.850	1.247	4.769	17880	Myh11	chr16	-	NM_0136	P13728	protein_cc	AV071570	myosin, he	Myh11
7572	Fopnl	1.059	1.542	1.633	0.503	0.034	0.142	8.085	8.167	8.792	66086	Fopnl	chr16	-	NM_0253	P20210	protein_cc	0610037P	Fgfr1op N	Fopnl
7573	Abcc1	2.898	0.513	1.487	0.013	0.060	0.473	4.346	5.881	4.918	17250	Abcc1	chr16	+	NM_0085	P2652	protein_cc	Abcc1a | A	ATP-bindi	Abcc1
7574	A630010A	1.000	1.000	1.000	1.000	1.000	1.000	0.000	0.000	0.000	545280	A630010A	chr16	+	NR_03355	.	lncRNA	RP24-255I	RIKEN cDN	A630010A
7575	Snai2	0.121	1.894	0.228	0.000	0.019	0.007	6.904	3.852	4.773	20583	Snai2	chr16	+	NM_0114	P11973	protein_cc	Slug | Slug	snail famil	Snai2
7576	Efcab1	1.000	1.000	1.000	1.000	1.000	1.000	0.000	0.000	0.000	66793	Efcab1	chr16	+	NM_0257	P2532	protein_cc	4930449A	EF hand ca	Efcab1
7577	Ube2v2	1.890	0.920	1.739	0.014	0.526	0.007	7.621	8.540	8.420	70620	Ube2v2	chr16	-	NM_0235	P8766	protein_cc	1110021H	ubiquitin-c	Ube2v2
7578	Mcm4	4.557	0.571	2.600	0.003	0.014	0.241	4.554	6.742	5.932	17217	Mcm4	chr16	-	NM_0085	P17766	protein_cc	19G | AI32	minichrom	Mcm4
7579	Prkdc	0.392	1.431	0.561	0.048	0.466	0.378	5.128	3.778	4.295	19090	Prkdc	chr16	+	NM_0111	P21483	protein_cc	AI326420 |	protein kir	Prkdc
7580	Mzt2	0.992	1.131	1.122	0.959	0.562	0.791	7.801	7.790	7.968	72083	Mzt2	chr16	-	NM_0293	P8042	protein_cc	2410018G	mitotic sp	Mzt2
7581	Cebpd	9.543	0.629	5.999	0.001	0.037	0.018	7.788	11.043	10.373	12609	Cebpd	chr16	+	NM_0076	P15389	protein_cc	c/EBPdelta	CCAAT/en	Cebpd
7582	Spidr	3.747	0.801	3.000	0.000	0.289	0.170	5.005	6.911	6.590	224008	Spidr	chr16	-	NM_1460	P1732	protein_cc	2310008H	scaffolding	Spidr
7583	Pkp2	1.002	1.027	1.029	0.985	0.832	0.762	7.399	7.402	7.441	67451	Pkp2	chr16	+	NM_0261	P23749	protein_cc	1200008D	plakophili	Pkp2
7584	Yars2	0.885	1.189	1.052	0.490	0.351	0.810	7.482	7.306	7.556	70120	Yars2	chr16	+	NM_1982	P11540	protein_cc	2210023C	tyrosyl-tR	Yars2
7585	Dnm1l	0.763	1.200	0.915	0.036	0.138	0.687	7.806	7.415	7.677	74006	Dnm1l	chr16	-	NM_0012	P1696	protein_cc	6330417M	dynamin 1	Dnm1l
7586	Fgd4	0.363	0.768	0.278	0.009	0.580	0.027	6.986	5.522	5.141	224014	Fgd4	chr16	-	NM_1392	P22697	protein_cc	9030023J(	FYVE, Rho	Fgd4
7587	Olfr19	1.000	1.000	1.000	1.000	1.000	1.000	0.000	0.000	0.000	18316	Olfr19	chr16	-	NM_1463	P21172	protein_cc	M12 | MO	olfactory r	Olfr19
7588	4933404G	1.000	1.000	1.000	1.000	1.000	1.000	0.000	0.000	0.000	74066	4933404G	chr16	-	NR_04592	.	lncRNA	4933401J2	RIKEN cDN	4933404G
7589	Spag6	1.005	1.405	1.412	0.000	0.182	0.423	0.000	0.008	0.498	381350	Spag6	chr16	+	NM_0157	P16575	protein_cc	Spag6l	sperm ass	Spag6
7590	Rpl31-ps1	1.543	0.969	1.495	0.028	0.786	0.067	8.473	9.099	9.053	665562	Rpl31-ps1	chr16	-	NM_0012	P19506	protein_cc	EG665562	ribosomal	Rpl31-ps1
7591	Igll1	1.935	0.298	0.577	0.023	1.000	0.422	0.794	1.746	0.000	16136	Igll1	chr16	-	NM_0011	P13900	protein_cc	BB139905	immunogl	Igll1
7592	Vpreb1	0.654	0.376	0.246	0.566	0.294	0.407	3.747	3.135	1.724	22362	Vpreb1	chr16	-	NM_0169	P2390	protein_cc	CD179a | \	pre-B lym	Vpreb1
7593	Top3b	1.180	0.646	0.762	0.579	0.213	0.345	7.437	7.675	7.045	21976	Top3b	chr16	+	NM_0116	P7113	protein_cc	.	topoisome	Top3b
7594	Ppm1f	1.216	1.147	1.394	0.263	0.482	0.335	6.457	6.739	6.936	68606	Ppm1f	chr16	+	NM_1768	P23878	protein_cc	1110021B	protein ph	Ppm1f
7595	Mapk1	0.847	0.695	0.588	0.237	0.042	0.075	9.460	9.221	8.695	26413	Mapk1	chr16	+	NM_0010	P8760	protein_cc	9030612K	mitogen-a	Mapk1
7596	Ypel1	1.610	1.049	1.689	0.000	0.788	0.265	6.396	7.083	7.152	106369	Ypel1	chr16	+	NM_0012	P20148	protein_cc	0610009L(	yippee-lik	Ypel1
7597	Ppil2	1.307	1.044	1.364	0.007	0.420	0.091	7.477	7.863	7.925	66053	Ppil2	chr16	-	NM_1449	P7566	protein_cc	0610009L(	peptidylpr	Ppil2
7598	2610318N	2.153	0.831	1.790	0.302	0.755	0.423	0.001	1.107	0.840	70458	2610318N	chr16	-	NM_1832	P21354	protein_cc	AL033325	RIKEN cDN	2610318N
7599	Sdf2l1	3.847	0.905	3.481	0.001	0.529	0.079	8.743	10.686	10.542	64136	Sdf2l1	chr16	-	NM_0223	P11508	protein_cc	.	stromal ce	Sdf2l1
7600	Ccdc116	1.010	1.821	1.840	0.000	0.000	0.002	0.000	0.015	0.880	76872	Ccdc116	chr16	-	NM_0013	P6159	protein_cc	4930432J1	coiled-coil	Ccdc116
7601	Ydjc	3.453	2.764	9.546	0.067	0.030	0.079	0.002	1.789	3.256	69101	Ydjc	chr16	+	NM_0269	P13544	protein_cc	1810015A	YdjC homo	Ydjc
7602	Ube2l3	1.081	0.964	1.042	0.076	0.508	0.636	9.866	9.978	9.925	22195	Ube2l3	chr16	-	NM_0094	P10155	protein_cc	C79827 | U	ubiquitin-c	Ube2l3
7603	Rimbp3	1.000	1.000	1.000	1.000	1.000	1.000	0.000	0.000	0.000	239731	Rimbp3	chr16	+	NM_0010	P12	protein_cc	Gm1759 |	RIMS bind	Rimbp3
7604	Hic2	0.524	1.510	0.791	0.007	0.116	0.407	5.869	4.936	5.531	58180	Hic2	chr16	+	NM_1789	P16933	protein_cc	AA409108	hypermet	Hic2
7605	Tmem191	0.127	1.719	0.218	0.001	0.206	0.053	3.063	0.087	0.869	224019	Tmem191	chr16	+	NM_1774	P12278	protein_cc	4933405M	transmem	Tmem191
7606	Pi4ka	0.797	1.594	1.270	0.007	0.000	0.050	7.733	7.405	8.078	224020	Pi4ka	chr16	-	NM_0010	P22503	protein_cc	Pik4ca	phosphati	Pi4ka
7607	Serpind1	0.148	0.698	0.103	0.000	0.047	0.008	12.221	9.462	8.942	15160	Serpind1	chr16	+	NM_0082	P13337	protein_cc	AA985900	serine (or	Serpind1
7608	Snap29	1.008	0.759	0.765	0.945	0.012	0.255	7.768	7.779	7.381	67474	Snap29	chr16	+	NM_0233	P14860	protein_cc	1300018G	synaptoso	Snap29
7609	Crkl	1.411	0.732	1.033	0.000	0.000	0.656	7.751	8.248	7.797	12929	Crkl	chr16	+	NM_0012	P15028	protein_cc	1110025F(	v-crk avian	Crkl
7610	Aifm3	0.126	0.501	0.063	0.016	0.203	0.118	4.818	1.834	0.836	72168	Aifm3	chr16	+	NM_1751	P10897	protein_cc	2810401C	apoptosis-	Aifm3
7611	Lztr1	0.678	0.937	0.635	0.000	0.715	0.176	7.385	6.824	6.731	66863	Lztr1	chr16	+	NM_0258	P24620	protein_cc	1200003E	leucine-zip	Lztr1
7612	Thap7	1.049	1.076	1.129	0.686	0.564	0.478	6.813	6.882	6.988	69009	Thap7	chr16	-	NM_0269	P5973	protein_cc	1810004B	THAP dom	Thap7
7613	Lrrc74b	0.716	0.995	0.712	0.182	1.000	0.423	0.490	0.008	0.000	74685	Lrrc74b	chr16	-	NM_0290	P5826	protein_cc	4930451C	leucine ric	Lrrc74b
7614	P2rx6	1.000	1.000	1.000	1.000	1.000	1.000	0.000	0.000	0.000	18440	P2rx6	chr16	+	NM_0110	P21761	protein_cc	P2rxl1 | P2	purinergic	P2rx6
7615	Slc7a4	0.451	1.073	0.484	0.042	0.886	0.382	3.711	2.562	2.663	224022	Slc7a4	chr16	-	NM_1448	P2203	protein_cc	AI853530	solute car	Slc7a4
7616	Smpd4	1.368	1.175	1.607	0.262	0.228	0.273	5.239	5.690	5.923	77626	Smpd4	chr16	+	NM_0011	P19684	protein_cc	41224020	sphingom	Smpd4
7617	Ccdc74a	1.069	0.365	0.390	0.924	1.000	0.422	1.357	1.454	0.000	72315	Ccdc74a	chr16	+	NM_0011	P4624	protein_cc	2310015A	coiled-coil	Ccdc74a
7618	Med15	1.755	0.936	1.642	0.003	0.581	0.133	7.432	8.243	8.148	94112	Med15	chr16	-	NM_0010	P18615	protein_cc	A230074L	mediator	Med15
7619	Klhl22	0.652	0.789	0.515	0.000	0.030	0.021	6.241	5.624	5.282	224023	Klhl22	chr16	+	NM_1454	P13508	protein_cc	2610318I1	kelch-like	Klhl22
7620	Scarf2	0.204	2.750	0.562	0.032	0.117	0.415	3.823	1.531	2.991	224024	Scarf2	chr16	+	NM_1537	P21759	protein_cc	NSR1 | Sca	scavenger	Scarf2
7621	B830017H	1.000	1.000	1.000	1.000	1.000	1.000	0.000	0.000	0.000	433004	B830017H	chr16	-	NR_02795	.	lncRNA	.	RIKEN cDN	B830017H
7622	Car15	0.973	0.355	0.346	0.972	0.287	0.515	2.579	2.539	1.046	80733	Car15	chr16	-	NM_0305	P8816	protein_cc	AI315043 |	carbonic a	Car15
7623	Dgcr2	0.710	0.850	0.604	0.001	0.241	0.103	8.799	8.304	8.070	13356	Dgcr2	chr16	-	NM_0100	P16008	protein_cc	99300340	DiGeorge	Dgcr2
7624	Tssk1	0.288	0.974	0.281	0.056	1.000	0.242	1.833	0.038	0.000	22114	Tssk1	chr16	+	NM_0094	P17672	protein_cc	Stk22a | T	testis-spe	Tssk1
7625	Tssk2	1.010	1.825	1.844	0.000	0.182	0.423	0.000	0.015	0.883	22115	Tssk2	chr16	+	NM_0094	P7529	protein_cc	DGS-G | SF	testis-spe	Tssk2
7626	Dgcr14	1.587	0.818	1.299	0.031	0.110	0.442	4.847	5.513	5.224	27886	Dgcr14	chr16	-	NM_0224	P937	protein_cc	AI462402 |	DiGeorge	Dgcr14
7627	Gsc2	1.000	1.000	1.000	1.000	1.000	1.000	0.000	0.000	0.000	195333	Gsc2	chr16	-	NM_0294	P22309	protein_cc	4930568H	goosecoid	Gsc2
7628	Slc25a1	0.763	1.564	1.193	0.033	0.004	0.324	10.015	9.624	10.269	13358	Slc25a1	chr16	-	NM_1531	P24652	protein_cc	1300019P	solute car	Slc25a1
7629	Vpreb2	1.000	1.000	1.000	1.000	1.000	1.000	0.000	0.000	0.000	22363	Vpreb2	chr16	+	NM_0169	P20144	protein_cc	CD179b | \	pre-B lym	Vpreb2
7630	Dgcr6	0.208	1.716	0.357	0.000	0.038	0.013	8.572	6.306	7.085	13353	Dgcr6	chr16	+	NM_0100	P18951	protein_cc	.	DiGeorge	Dgcr6
7631	Prodh	0.061	1.495	0.092	0.000	0.141	0.021	10.077	6.049	6.629	19125	Prodh	chr16	-	NM_0111	P15258	protein_cc	Pro-1 | Prc	proline de	Prodh

7632	Rtn4r	0.669	0.995	0.666	0.181	1.000	0.423	0.587	0.008	0.000	65079	Rtn4r	chr16	+	NM_0229	P10846	protein_cc	NgR| NgR	reticulon 4	Rtn4r
7633	4933432I0	1.000	1.000	1.000	1.000	1.000	1.000	0.000	0.000	0.000	66779	4933432I0	chr16	-	NR_02784	.	lncRNA	.	RIKEN cDN	4933432I0
7634	Zdhhc8	0.977	0.753	0.736	0.940	0.425	0.438	5.746	5.713	5.304	27801	Zdhhc8	chr16	-	NM_1721	P16388	protein_cc	D16H22S1	zinc finger	Zdhhc8
7635	Ranbp1	2.226	0.735	1.636	0.000	0.002	0.039	8.265	9.419	8.975	19385	Ranbp1	chr16	-	NM_0112	P24833	protein_cc	Htf9a	RAN bindi	Ranbp1
7636	Trmt2a	2.828	0.725	2.050	0.001	0.048	0.099	6.469	7.969	7.505	15547	Trmt2a	chr16	+	NM_0010	P25609	protein_cc	Htf9c	TRM2 tRN	Trmt2a
7637	Dgcr8	1.983	0.892	1.769	0.003	0.346	0.088	5.592	6.579	6.415	94223	Dgcr8	chr16	-	NM_0333	P24784	protein_cc	D16H22S1	DiGeorge	Dgcr8
7638	Gm24572	1.000	1.000	1.000	1.000	1.000	1.000	0.000	0.000	0.000	10479566	Gm24572	chr16	-	NR_12856	.	miRNA	Mir3618|	predicted	Gm24572
7639	Tango2	0.282	1.375	0.387	0.000	0.126	0.004	8.989	7.162	7.622	27883	Tango2	chr16	-	NM_1385	P10412	protein_cc	D16H22S6	transport	Tango2
7640	Arvcf	0.254	1.552	0.394	0.000	0.139	0.025	9.496	7.518	8.153	11877	Arvcf	chr16	+	NM_0012	P21370	protein_cc	.	armadillo	Arvcf
7641	Comt	0.212	1.228	0.261	0.000	0.098	0.000	12.695	10.460	10.756	12846	Comt	chr16	-	NM_0011	P23788	protein_cc	Comt1| D	catechol-O	Comt
7642	Txnrd2	0.359	1.006	0.361	0.000	0.967	0.009	8.017	6.539	6.547	26462	Txnrd2	chr16	+	NM_0137	P8056	protein_cc	AA118373	thioredoxi	Txnrd2
7643	Gnb1l	0.554	3.809	2.111	0.127	0.027	0.241	3.557	2.706	4.635	13972	Gnb1l	chr16	+	NM_0010	P24548	protein_cc	ESTM55|	guanine n	Gnb1l
7644	Tbx1	0.552	0.990	0.547	0.182	1.000	0.423	0.871	0.015	0.000	21380	Tbx1	chr16	-	NM_0112	P23174	protein_cc	.	T-box 1	Tbx1
7645	Gp1bb	10.058	0.780	7.845	0.000	0.169	0.084	2.198	5.528	5.169	14724	Gp1bb	chr16	-	NM_0103	P19768	protein_cc	.	glycoprote	Gp1bb
7646	Sept5	7.959	0.919	7.311	0.000	0.695	0.129	2.198	5.191	5.068	18951	Sept5	chr16	-	NM_2136	P20885	protein_cc	Cdcrel-1|	septin 5	Sept5
7647	Cldn5	1.047	1.138	1.192	0.175	0.081	0.224	3.645	3.711	3.898	12741	Cldn5	chr16	+	NM_0138	P20104	protein_cc	AI854493|	claudin 5	Cldn5
7648	Cdc45	1.572	0.767	1.206	0.309	0.401	0.735	2.030	2.683	2.300	12544	Cdc45	chr16	-	NM_0098	P13989	protein_cc	Cdc45l	cell divisio	Cdc45
7649	Ufd1l	1.567	1.085	1.700	0.000	0.444	0.112	8.877	9.525	9.643	22230	Ufd1l	chr16	+	NM_0116	P11568	protein_cc	Ufd1	ubiquitin f	Ufd1l
7650	2510002D	0.426	1.405	0.599	0.000	0.000	0.044	8.650	7.421	7.912	72307	2510002D	chr16	+	NM_0010	P5541	protein_cc	2410043I0	RIKEN cDN	2510002D
7651	Mrpl40	0.792	1.301	1.030	0.112	0.062	0.720	8.363	8.027	8.406	18100	Mrpl40	chr16	-	NM_0109	P20475	protein_cc	Nlvcf	mitochond	Mrpl40
7652	Hira	1.737	0.436	0.758	0.012	0.007	0.271	5.084	5.881	4.684	15260	Hira	chr16	+	NM_0104	P4066	protein_cc	AA138857	histone ce	Hira
7653	Olfr164	2.479	0.302	0.748	0.375	1.000	0.421	0.419	1.728	0.000	258443	Olfr164	chr16	-	NM_1464	P3474	protein_cc	MOR279-2	olfactory r	Olfr164
7654	Olfr165	1.005	1.439	1.446	0.000	0.182	0.423	0.000	0.008	0.532	258458	Olfr165	chr16	-	NM_1464	P7649	protein_cc	MOR279-1	olfactory r	Olfr165
7655	Olfr166	0.716	0.995	0.712	0.182	1.000	0.423	0.490	0.008	0.000	259071	Olfr166	chr16	+	NM_1470	P6331	protein_cc	MOR270-1	olfactory r	Olfr166
7656	Olfr167	1.000	1.000	1.000	1.000	1.000	1.000	0.000	0.000	0.000	258937	Olfr167	chr16	-	NM_1469	P1228	protein_cc	MOR272-1	olfactory r	Olfr167
7657	Olfr168	1.000	1.000	1.000	1.000	1.000	1.000	0.000	0.000	0.000	258354	Olfr168	chr16	-	NM_1463	P15062	protein_cc	MOR271-1	olfactory r	Olfr168
7658	Olfr169	1.000	1.000	1.000	1.000	1.000	1.000	0.000	0.000	0.000	258158	Olfr169	chr16	-	NM_0010	P7715	protein_cc	MOR273-3	olfactory r	Olfr169
7659	Olfr170	1.000	1.000	1.000	1.000	1.000	1.000	0.000	0.000	0.000	258959	Olfr170	chr16	-	NM_1469	P9137	protein_cc	MOR273-2	olfactory r	Olfr170
7660	Olfr171	1.000	1.000	1.000	1.000	1.000	1.000	0.000	0.000	0.000	258960	Olfr171	chr16	-	NM_1469	P8177	protein_cc	MOR273-1	olfactory r	Olfr171
7661	Lamp3	1.840	0.359	0.661	0.524	0.369	0.605	1.078	1.958	0.481	239739	Lamp3	chr16	-	NM_1773	P14284	protein_cc	1200002D	lysosomal	Lamp3
7662	B3gnt5	1.615	2.150	3.472	0.287	0.175	0.273	0.001	0.692	1.796	108105	B3gnt5	chr16	+	NM_0540	P19867	protein_cc	beta3GnT	UDP-GlcN	B3gnt5
7663	A930003A	1.000	1.000	1.000	1.000	1.000	1.000	0.000	0.000	0.000	68162	A930003A	chr16	-	NR_02789	.	lncRNA	AI853191	RIKEN cDN	A930003A
7664	Klhl6	1.971	1.647	3.246	0.001	0.060	0.105	3.956	4.935	5.655	239743	Klhl6	chr16	-	NM_1833	P19737	protein_cc	.	kelch-like	Klhl6
7665	Klhl24	0.935	0.899	0.840	0.533	0.685	0.719	8.263	8.165	8.012	75785	Klhl24	chr16	+	NM_0294	P5478	protein_cc	1110046J1	kelch-like	Klhl24
7666	Yeats2	1.098	0.787	0.864	0.398	0.306	0.747	5.859	5.995	5.649	208146	Yeats2	chr16	+	NM_0011	P8861	protein_cc	BC042768	YEATS dor	Yeats2
7667	Map6d1	0.345	2.668	0.920	0.184	0.185	0.937	1.587	0.052	1.468	208158	Map6d1	chr16	-	NM_1985	P23812	protein_cc	A330066I1	MAP6 dor	Map6d1
7668	Parl	0.743	1.554	1.155	0.052	0.025	0.312	8.637	8.208	8.845	381038	Parl	chr16	-	NM_0010	P10411	protein_cc	D16Ertd60	presenilin	Parl
7669	Cyp2ab1	1.000	1.000	1.000	1.000	1.000	1.000	0.000	0.000	0.000	224044	Cyp2ab1	chr16	-	NM_1831	P20403	protein_cc	EG224044	cytochrom	Cyp2ab1
7670	Abcc5	2.893	0.813	2.352	0.008	0.248	0.017	4.417	5.949	5.651	27416	Abcc5	chr16	-	NM_0137	P14000	protein_cc	2900011L	ATP-bindi	Abcc5
7671	Eif2b5	0.837	1.329	1.112	0.083	0.029	0.578	8.479	8.222	8.632	224045	Eif2b5	chr16	+	NM_1722	P3459	protein_cc	C81315	eukaryotic	Eif2b5
7672	Dvl3	1.292	0.528	0.682	0.582	0.282	0.102	1.629	1.998	1.076	13544	Dvl3	chr16	-	NM_0078	P8913	protein_cc	.	dishevelle	Dvl3
7673	Ap2m1	0.730	1.221	0.892	0.009	0.269	0.627	9.089	8.636	8.924	11773	Ap2m1	chr16	+	NM_0013	P24743	protein_cc	.	adaptor-re	Ap2m1
7674	Gm15760	0.800	1.334	1.068	0.530	0.654	0.937	3.365	3.044	3.461	10004294	Gm15760	chr16	-	NR_03067	.	misc_RNA	ENSMUSG	predicted	Gm15760
7675	Abcf3	1.786	0.639	1.142	0.015	0.005	0.734	7.211	8.048	7.403	27406	Abcf3	chr16	+	NM_0138	P13625	protein_cc	AI326318|	ATP-bindi	Abcf3
7676	Vwa5b2	0.451	0.986	0.444	0.182	1.000	0.423	1.170	0.021	0.000	328643	Vwa5b2	chr16	+	NM_1826	P19341	protein_cc	BB182430	von Willeb	Vwa5b2
7677	Alg3	0.470	1.607	0.756	0.007	0.065	0.107	6.997	5.909	6.593	208624	Alg3	chr16	-	NM_1459	P12801	protein_cc	D16Ertd36	asparagine	Alg3
7678	Ece2	3.460	0.882	3.052	0.000	0.279	0.060	5.918	7.709	7.528	107522	Ece2	chr16	+	NM_0254	P1347	protein_cc	1810009K	endothelin	Ece2
7679	Camk2n2	1.166	1.353	1.578	0.561	0.325	0.503	4.081	4.302	4.739	73047	Camk2n2	chr16	-	NM_0284	P21223	protein_cc	2900075A	calcium/ca	Camk2n2
7680	Psmd2	0.985	1.050	1.034	0.643	0.382	0.655	10.591	10.570	10.640	21762	Psmd2	chr16	+	NM_1341	P23017	protein_cc	9430095H	proteason	Psmd2
7681	Eif4g1	0.842	0.935	0.788	0.120	0.561	0.174	11.833	11.585	11.489	208643	Eif4g1	chr16	+	NM_0013	P8820	protein_cc	E030015G	eukaryotic	Eif4g1
7682	Snord66	3.128	0.933	2.919	0.079	0.859	0.273	2.127	3.772	3.673	10021743	Snord66	chr16	+	NR_02853	.	snoRNA	MBII-142|	small nucl	Snord66
7683	Fam131a	0.128	1.888	0.242	0.004	0.365	0.088	6.325	3.363	4.280	78408	Fam131a	chr16	+	NM_1337	P21657	protein_cc	2900046G	family wit	Fam131a
7684	Clcn2	0.109	1.817	0.198	0.000	0.430	0.028	7.179	3.979	4.840	12724	Clcn2	chr16	-	NM_0099	P22402	protein_cc	AL118368	chloride c	Clcn2
7685	Polr2h	1.744	0.868	1.513	0.003	0.169	0.105	5.595	6.398	6.193	245841	Polr2h	chr16	+	NM_1456	P26035	protein_cc	.	polymeras	Polr2h
7686	Thpo	2.486	0.618	1.537	0.168	0.352	0.354	6.928	8.242	7.549	21832	Thpo	chr16	-	NM_0012	P18019	protein_cc	Mgdf| MI	thrombop	Thpo
7687	Chrd	0.603	0.651	0.393	0.317	0.361	0.444	4.505	3.776	3.158	12667	Chrd	chr16	+	NM_0098	P16433	protein_cc	Chd	chordin	Chrd
7688	Teddm3	1.000	1.000	1.000	1.000	1.000	1.000	0.000	0.000	0.000	66561	Teddm3	chr16	-	NM_0256	P18308	protein_cc	2310042E	transmem	Teddm3
7689	Ephb3	0.096	1.227	0.118	0.016	0.276	0.140	3.476	0.100	0.395	13845	Ephb3	chr16	+	NM_0101	P20652	protein_cc	AW45689	Eph recep	Ephb3
7690	Gm16863	1.000	1.000	1.000	1.000	1.000	1.000	0.000	0.000	0.000	328646	Gm16863	chr16	-	NR_04616	.	lncRNA	A830060N	predicted	Gm16863
7691	Magef1	1.158	1.317	1.526	0.451	0.295	0.440	3.359	3.571	3.968	76222	Magef1	chr16	-	NR_13198	.	misc_RNA	6430590I0	melanoma	Magef1
7692	Vps8	0.958	0.859	0.823	0.564	0.130	0.230	5.159	5.097	4.878	209018	Vps8	chr16	+	NM_0012	P19494	protein_cc	AI315068|	VPS8 COR	Vps8
7693	2510009E	0.973	0.783	0.762	0.885	0.355	0.281	6.494	6.455	6.103	72190	2510009E	chr16	-	NM_0010	P19930	protein_cc	AI194952	RIKEN cDN	2510009E
7694	Ehhadh	0.278	1.331	0.370	0.003	0.558	0.089	6.850	5.003	5.415	74147	Ehhadh	chr16	-	NM_0237	P15287	protein_cc	1300002P	enoyl-Coe	Ehhadh
7695	1300002E	1.084	1.355	1.469	0.524	0.032	0.105	7.095	7.211	7.649	10004348	1300002E	chr16	+	NR_03795	.	lncRNA	1500026P	RIKEN cDN	1300002E
7696	Map3k13	0.191	1.597	0.305	0.067	0.437	0.349	5.556	3.169	3.844	71751	Map3k13	chr16	+	NM_1728	P11088	protein_cc	C130026N	mitogen-a	Map3k13
7697	Tmem41a	1.708	1.778	3.036	0.010	0.001	0.002	7.097	7.869	8.699	66664	Tmem41a	chr16	-	NR_03777	.	protein_cc	2900010K	transmem	Tmem41a
7698	Liph	1.346	3.006	4.045	0.239	0.003	0.061	0.758	1.186	2.774	239759	Liph	chr16	-	NM_0012	P21186	protein_cc	C130037N	lipase, me	Liph
7699	Senp2	1.780	0.750	1.336	0.014	0.012	0.316	7.013	7.845	7.430	75826	Senp2	chr16	+	NR_02748	.	protein_cc	2310007L	SUMO/sen	Senp2
7700	Igf2bp2	3.976	0.624	2.480	0.019	0.240	0.365	4.755	6.747	6.065	319765	Igf2bp2	chr16	-	NM_1830	P3867	protein_cc	C330012H	insulin-lik	Igf2bp2
7701	Tra2b	1.764	0.779	1.375	0.001	0.036	0.140	7.860	8.679	8.320	20462	Tra2b	chr16	-	NM_0091	P4814	protein_cc	5730405G	transform	Tra2b
7702	Etv5	1.014	1.069	1.084	0.949	0.777	0.738	5.179	5.198	5.295	104156	Etv5	chr16	-	NM_0237	P22452	protein_cc	1110005E	ets variant	Etv5
7703	Dgkg	0.437	1.330	0.581	0.087	0.573	0.461	1.902	0.707	1.118	110197	Dgkg	chr16	-	NM_1386	P1240	protein_cc	2900055E	diacylglyc	Dgkg
7704	9230117E	1.000	1.000	1.000	1.000	1.000	1.000	0.000	0.000	0.000	78082	9230117E	chr16	+	NR_13098	.	lncRNA	.	RIKEN cDN	9230117E
7705	Crygs	0.716	0.995	0.712	0.182	1.000	0.423	0.490	0.008	0.000	12970	Crygs	chr16	-	NM_0099	P122	protein_cc	AI327013|	crystallin,	Crygs
7706	Tbccd1	0.450	1.344	0.604	0.012	0.146	0.181	7.432	6.278	6.704	70573	Tbccd1	chr16	-	NM_0010	P17931	protein_cc	5730478N	TBCC dom	Tbccd1
7707	Dnajb11	1.778	0.886	1.575	0.006	0.258	0.050	8.716	9.546	9.371	67838	Dnajb11	chr16	+	NM_0264	P19424	protein_cc	1810031F	DnaJ heat	Dnajb11
7708	Gm30505	0.522	1.895	0.989	0.000	0.002	0.932	4.443	3.506	4.428	10263243	Gm30505	chr16	-	NR_11050	.	lncRNA		predicted	Gm30505
7709	Ahsg	0.351	0.876	0.307	0.002	0.346	0.036	17.377	15.867	15.676	11625	Ahsg	chr16	+	NR_07509	.	protein_cc	.	alpha-2-H	Ahsg
7710	Fetub	0.703	0.965	0.679	0.016	0.749	0.009	11.374	10.867	10.815	59083	Fetub	chr16	+	NM_0010	P7152	protein_cc	2310011O	fetuin bet	Fetub
7711	Hrg	0.279	0.905	0.252	0.000	0.093	0.001	12.776	10.931	10.787	94175	Hrg	chr16	+	NM_0531	P4662	protein_cc	AI265597|	histidine-r	Hrg
7712	Kng2	0.663	0.957	0.635	0.000	0.316	0.012	12.016	11.424	11.361	385643	Kng2	chr16	-	NM_0011	P6443	protein_cc	.	kininogen	Kng2
7713	Kng1	0.609	0.846	0.515	0.000	0.010	0.005	13.946	13.231	12.989	16644	Kng1	chr16	+	NM_0011	P8757	protein_cc	Kng	kininogen	Kng1
7714	Eif4a2	0.523	0.740	0.387	0.000	0.006	0.006	10.226	9.292	8.858	13682	Eif4a2	chr16	+	NM_0011	P23567	protein_cc	4833432N	eukaryotic	Eif4a2
7715	Snord2	7.348	0.411	3.017	0.000	0.065	0.542	2.722	5.599	4.315	10021653	Snord2	chr16	+	NR_03070	.	snoRNA	.	small nucl	Snord2
7716	Snora81	1.971	0.916	1.805	0.088	0.819	0.472	8.056	9.035	8.908	10021742	Snora81	chr16	+	NR_03404	.	snoRNA	MBI-61	small nucl	Snora81
7717	Rfc4	0.577	1.711	0.986	0.046	0.147	0.976	4.642	3.848	4.622	106344	Rfc4	chr16	-	NM_1454	P3477	protein_cc	A1| AI894	replication	Rfc4
7718	Adipoq	0.180	11.159	2.012	0.116	0.106	0.589	2.597	0.126	3.606	11450	Adipoq	chr16	+	NM_0096	P23098	protein_cc	30kDa| Ac	adiponect	Adipoq
7719	St6gal1	2.733	0.679	1.854	0.000	0.017	0.132	9.573	11.024	10.464	20440	St6gal1	chr16	+	NM_1459	P21687	protein_cc	AW74232	beta galac	St6gal1
7720	Rtp1	1.000	1.000	1.000	1.000	1.000	1.000	0.000	0.000	0.000	239766	Rtp1	chr16	+	NM_0010	P2399	protein_cc	Gm1766|	receptor t	Rtp1
7721	Masp1	0.206	2.232	0.461	0.000	0.019	0.007	5.231	2.955	4.113	17174	Masp1	chr16	-	NM_0085	P14810	protein_cc	AW04806	mannan-b	Masp1
7722	Rtp4	9.698	0.297	2.876	0.000	0.001	0.248	8.108	11.386	9.632	67775	Rtp4	chr16	+	NM_0233	P22140	protein_cc	5830458K	receptor t	Rtp4

7723	Sst	1.019	2.648	2.698	0.000	0.182	0.423	0.000	0.027	1.432	20604	Sst	chr16	-	NM_0092	P9954	protein_cc	Smst| SOI	somatosta	Sst
7724	Rtp2	1.005	1.405	1.412	0.000	0.182	0.423	0.000	0.008	0.498	224055	Rtp2	chr16	-	NM_0010	P22831	protein_cc	Gm605	receptor t	Rtp2
7725	Bcl6	4.016	0.589	2.365	0.000	0.012	0.213	6.113	8.118	7.355	12053	Bcl6	chr16	-	NM_0097	P13865	protein_cc	Bcl5	B cell leuk	Bcl6
7726	Lppos	0.150	1.222	0.183	0.007	0.010	0.092	7.499	4.761	5.050	68841	Lppos	chr16	-	NR_03795	.	lncRNA	1110054M	LIM doma	Lppos
7727	Lpp	0.560	0.848	0.475	0.001	0.331	0.056	9.621	8.784	8.546	210126	Lpp	chr16	+	NM_1786	P4837	protein_cc	9430020K	LIM doma	Lpp
7728	Morf4l1-p	0.877	0.970	0.851	0.010	0.250	0.074	9.334	9.144	9.101	624582	Morf4l1-p	chr16	+	NR_03809	.	misc_RNA	A730098P	mortality	Morf4l1-ps
7729	A230028C	0.143	0.951	0.136	0.003	1.000	0.062	2.880	0.073	0.000	319487	A230028C	chr16	+	NR_04037	.	lncRNA	.	RIKEN cDN	A230028O
7730	Tprg	1.000	1.000	1.000	1.000	1.000	1.000	0.000	0.000	0.000	71338	Tprg	chr16	+	NM_1751	P13487	protein_cc	5430420C	transform	Tprg
7731	Trp63	16.223	2.942	47.730	0.000	0.164	0.261	0.331	4.351	5.908	22061	Trp63	chr16	+	NM_0011	P23763	protein_cc	AI462811|	transform	Trp63
7732	P3h2	1.777	0.225	0.399	0.562	1.000	0.423	1.326	2.155	0.000	210530	P3h2	chr16	-	NM_1733	P6841	protein_cc	4832416N	prolyl 3-hy	P3h2
7733	Cldn1	0.402	0.990	0.397	0.000	0.965	0.051	9.023	7.707	7.692	12737	Cldn1	chr16	-	NM_0166	P17448	protein_cc	AI596271	claudin 1	Cldn1
7734	Cldn16	1.000	1.000	1.000	1.000	1.000	1.000	0.000	0.000	0.000	114141	Cldn16	chr16	+	NM_0532	P14368	protein_cc	claudin-16	claudin 16	Cldn16
7735	Tmem207	1.000	1.000	1.000	1.000	1.000	1.000	0.000	0.000	0.000	10004305	Tmem207	chr16	-	NM_0011	P4267	protein_cc	10004305	transmem	Tmem207
7736	Il1rap	0.543	1.056	0.573	0.059	0.700	0.282	5.356	4.474	4.552	16180	Il1rap	chr16	+	NM_0011	P16506	protein_cc	6430709H	interleukir	Il1rap
7737	Gm20319	0.266	2.225	0.593	0.121	0.018	0.568	3.722	1.814	2.968	10050462	Gm20319	chr16	-	NR_10506	.	lncRNA	.	predicted	Gm20319
7738	Gmnc	1.000	1.000	1.000	1.000	1.000	1.000	0.000	0.000	0.000	239789	Gmnc	chr16	-	NM_0012	P24605	protein_cc	Gemc1| G	geminin c	Gmnc
7739	Ostn	1.000	1.000	1.000	1.000	1.000	1.000	0.000	0.000	0.000	239790	Ostn	chr16	+	NM_1981	P17892	protein_cc	Ostc	osteocrin	Ostn
7740	Uts2b	1.000	1.000	1.000	1.000	1.000	1.000	0.000	0.000	0.000	224065	Uts2b	chr16	-	NM_1981	P25839	protein_cc	Gm538| U	urotensin	Uts2b
7741	Ccdc50	0.879	1.078	0.947	0.000	0.403	0.703	8.917	8.730	8.839	67501	Ccdc50	chr16	+	NM_0010	P425	protein_cc	C3orf6	coiled-coil	Ccdc50
7742	Gm10823	1.000	1.000	1.000	1.000	1.000	1.000	0.000	0.000	0.000	10003862	Gm10823	chr16	+	NR_03347	.	lncRNA	ENSMUSG	predicted	Gm10823
7743	Fgf12	0.180	2.431	0.438	0.000	0.190	0.171	2.548	0.075	1.357	14167	Fgf12	chr16	-	NM_0012	P23684	protein_cc	AV114868	fibroblast	Fgf12
7744	Mb21d2	1.243	0.786	0.977	0.018	0.133	0.932	6.990	7.303	6.956	239796	Mb21d2	chr16	-	NM_1777	P22773	protein_cc	1600021P	Mab-21 d	Mb21d2
7745	Hrasls	4.542	0.559	2.540	0.149	0.414	0.423	0.001	2.185	1.346	27281	Hrasls	chr16	+	NM_0137	P15248	protein_cc	2810012B	HRAS-like	Hrasls
7746	Atp13a5	0.716	0.995	0.712	0.182	1.000	0.423	0.490	0.008	0.000	268878	Atp13a5	chr16	-	NM_0012	P4551	protein_cc	C630015F	ATPase ty	Atp13a5
7747	Atp13a4	1.000	1.000	1.000	1.000	1.000	1.000	0.000	0.000	0.000	224079	Atp13a4	chr16	-	NM_1726	P26875	protein_cc	4631413J1	ATPase ty	Atp13a4
7748	Opa1	0.510	1.208	0.617	0.000	0.014	0.027	8.962	7.991	8.264	74143	Opa1	chr16	+	NM_1337	P2349	protein_cc	1200011N	optic atro	Opa1
7749	Gm1968	1.000	1.000	1.000	1.000	1.000	1.000	0.000	0.000	0.000	328657	Gm1968	chr16	-	NR_03768	.	lncRNA		predicted	Gm1968
7750	4632428C	0.198	3.189	0.630	0.000	0.075	0.345	2.416	0.078	1.751	10004310	4632428C	chr16	+	NR_03363	.	lncRNA	10004310	RIKEN cDN	4632428C
7751	9030404E	1.000	1.000	1.000	1.000	1.000	1.000	0.000	0.000	0.000	71528	9030404E	chr16	+	NR_04587	.	lncRNA	.	RIKEN cDN	9030404E1
7752	Hes1	0.525	1.376	0.722	0.000	0.268	0.377	7.700	6.769	7.230	15205	Hes1	chr16	+	NM_0082	P5882	protein_cc	bHLHb39|	hairy and	Hes1
7753	Cpn2	0.144	1.977	0.285	0.000	0.001	0.009	10.824	8.030	9.013	71756	Cpn2	chr16	-	NM_0279	P8518	protein_cc	1300018K	carboxype	Cpn2
7754	Lrrc15	0.794	0.629	0.499	0.627	1.000	0.423	1.003	0.669	0.000	74488	Lrrc15	chr16	-	NM_0289	P15958	protein_cc	5430427N	leucine ric	Lrrc15
7755	Gp5	5.364	0.124	0.665	0.000	1.000	0.423	0.589	3.012	0.000	14729	Gp5	chr16	-	NM_0081	P13410	protein_cc	GPV	glycoprote	Gp5
7756	Atp13a3	0.922	0.804	0.741	0.311	0.062	0.194	9.436	9.318	9.004	224088	Atp13a3	chr16	-	NM_0011	P25715	protein_cc	AU022875	ATPase ty	Atp13a3
7757	Tmem44	0.365	1.538	0.562	0.001	0.352	0.265	4.619	3.166	3.787	224090	Tmem44	chr16	-	NM_1726	P17590	protein_cc	1700007N	transmem	Tmem44
7758	Lsg1	1.865	1.104	2.058	0.058	0.602	0.021	7.192	8.091	8.233	224092	Lsg1	chr16	-	NM_1780	P18364	protein_cc	5830465I2	large 60S	Lsg1
7759	Fam43a	1.484	1.319	1.958	0.002	0.111	0.120	6.379	6.948	7.348	224093	Fam43a	chr16	+	NM_1776	P26759	protein_cc	Tuf1	family wit	Fam43a
7760	Xxylt1	0.618	1.551	0.958	0.394	0.559	0.957	2.465	1.770	2.404	268880	Xxylt1	chr16	-	NM_1986	P7857	protein_cc	AI480653	xyloside x	Xxylt1
7761	Acap2	1.270	0.967	1.229	0.149	0.511	0.423	6.686	7.032	6.984	78618	Acap2	chr16	-	NM_0301	P7639	protein_cc	4832442G	ArfGAP wi	Acap2
7762	Ppp1r2	0.911	1.245	1.134	0.522	0.056	0.531	8.040	7.906	8.222	66849	Ppp1r2	chr16	-	NM_0258	P11113	protein_cc	0610025N	protein ph	Ppp1r2
7763	Apod	0.635	7.618	4.834	0.158	0.069	0.299	1.415	0.759	3.688	11815	Apod	chr16	-	NM_0013	P26513	protein_cc	.	apolipopro	Apod
7764	Bdh1	0.098	1.201	0.118	0.000	0.213	0.003	11.860	8.514	8.779	71911	Bdh1	chr16	+	NM_1751	P5739	protein_cc	2310032J2	3-hydroxy	Bdh1
7765	Dlg1	0.811	0.692	0.562	0.040	0.012	0.077	8.448	8.147	7.616	13383	Dlg1	chr16	+	NM_0012	P462	protein_cc	B130052P	discs, larg	Dlg1
7766	Mfi2	2.144	0.663	1.422	0.303	0.511	0.423	0.000	1.101	0.508	30060	Mfi2	chr16	+	NM_0139	P16672	protein_cc	CD228| M	antigen p9	Mfi2
7767	Pigz	0.726	1.387	1.007	0.187	0.187	0.988	0.477	0.015	0.487	239827	Pigz	chr16	+	NM_1728	P11056	protein_cc	F630022B	phosphati	Pigz
7768	0610012G	0.686	1.057	0.725	0.002	0.324	0.069	8.408	7.864	7.944	106264	0610012G	chr16	-	NR_02789	.	lncRNA	AI839730	RIKEN cDN	0610012G
7769	Ncbp2	1.117	0.552	0.616	0.164	0.003	0.029	7.848	8.007	7.149	68092	Ncbp2	chr16	+	NM_0265	P13818	protein_cc	20kDa| 5S	nuclear ca	Ncbp2
7770	Senp5	1.072	1.061	1.137	0.534	0.445	0.365	7.210	7.310	7.396	320213	Senp5	chr16	-	NM_1771	P26119	protein_cc	6230429P	SUMO/ser	Senp5
7771	Gm15694	1.329	0.851	1.131	0.246	0.385	0.647	6.201	6.611	6.378	10166976	Gm15694	chr16	-	NR_10351	.	misc_RNA	OTTMUSG	predicted	Gm15694
7772	Pak2	2.083	0.688	1.434	0.000	0.000	0.039	8.416	9.475	8.936	224105	Pak2	chr16	-	NM_1773	P25489	protein_cc	5330420P	p21 prote	Pak2
7773	Pigx	0.841	1.243	1.045	0.208	0.004	0.813	7.644	7.394	7.708	72084	Pigx	chr16	-	NM_0011	P7319	protein_cc	2010319C	phosphati	Pigx
7774	Cep19	0.411	2.118	0.870	0.000	0.025	0.596	6.073	4.789	5.872	66994	Cep19	chr16	+	NM_0258	P18567	protein_cc	1500031L(	centrosom	Cep19
7775	Nrros	0.986	1.370	1.351	0.521	0.003	0.066	6.243	6.223	6.677	224109	Nrros	chr16	-	NM_1460	P2090	protein_cc	E430025L(	negative r	Nrros
7776	Bex6	0.716	0.995	0.712	0.182	1.000	0.423	0.490	0.008	0.000	328660	Bex6	chr16	+	NM_0010	P6779	protein_cc	B020003O	brain expr	Bex6
7777	Fbxo45	1.462	0.764	1.117	0.001	0.007	0.427	6.138	6.687	6.298	268882	Fbxo45	chr16	-	NM_1734	P2782	protein_cc	2610017J(	F-box prot	Fbxo45
7778	Wdr53	0.781	1.192	0.931	0.043	0.295	0.768	7.008	6.652	6.906	68980	Wdr53	chr16	+	NM_0011	P5225	protein_cc	1500002B	WD repea	Wdr53
7779	Smco1	0.676	1.797	1.214	0.188	0.000	0.588	0.587	0.021	0.867	69576	Smco1	chr16	+	NM_1832	P23434	protein_cc	2310010M	single-pas	Smco1
7780	Rnf168	1.679	0.766	1.287	0.000	0.006	0.094	7.686	8.434	8.050	70238	Rnf168	chr16	+	NM_0273	P9312	protein_cc	3110001H	ring finger	Rnf168
7781	Ubxn7	0.669	1.091	0.730	0.029	0.653	0.177	7.801	7.221	7.347	224111	Ubxn7	chr16	+	NM_1776	P9852	protein_cc	A630090P	UBX doma	Ubxn7
7782	Tm4sf19	1.000	1.000	1.000	1.000	1.000	1.000	0.000	0.000	0.000	277203	Tm4sf19	chr16	+	NM_0011	P1311	protein_cc	EG277203	transmem	Tm4sf19
7783	Tctex1d2	2.056	1.276	2.623	0.261	0.527	0.027	4.402	5.441	5.793	66061	Tctex1d2	chr16	-	NM_0253	P10774	protein_cc	0610012D	Tctex1 do	Tctex1d2
7784	Pcyt1a	0.522	1.276	0.666	0.000	0.012	0.007	9.433	8.494	8.845	13026	Pcyt1a	chr16	+	NM_0099	P20865	protein_cc	Cctalpha|	phosphate	Pcyt1a
7785	Slc51a	1.000	1.000	1.000	1.000	1.000	1.000	0.000	0.000	0.000	106407	Slc51a	chr16	-	NM_1459	P15742	protein_cc	AV001382	solute car	Slc51a
7786	Zdhhc19	1.000	1.000	1.000	1.000	1.000	1.000	0.000	0.000	0.000	245308	Zdhhc19	chr16	+	NM_1993	P2753	protein_cc	Gm1744|	zinc finger	Zdhhc19
7787	Tfrc	1.178	0.879	1.036	0.012	0.021	0.720	7.902	8.138	7.953	22042	Tfrc	chr16	+	NM_0116	P20949	protein_cc	2610028K	transferrin	Tfrc
7788	Tnk2	0.355	2.057	0.731	0.015	0.045	0.455	6.846	5.353	6.393	51789	Tnk2	chr16	+	NM_0011	P9512	protein_cc	Ack| Ack-1	tyrosine k	Tnk2
7789	Tnk2os	1.572	0.636	1.000	0.304	1.000	0.423	0.000	0.653	0.000	675921	Tnk2os	chr16	-	NR_03349	.	lncRNA	ENSMUSG	tyrosine k	Tnk2os
7790	Muc4	1.097	3.846	4.217	0.820	0.229	0.462	0.588	0.721	2.664	140474	Muc4	chr16	+	NM_0804	P16373	protein_cc	4933405I1	mucin 4	Muc4
7791	Muc20	0.451	0.986	0.444	0.182	1.000	0.423	1.170	0.021	0.000	224116	Muc20	chr16	-	NM_0011	P17155	protein_cc	BC026367	mucin 20	Muc20
7792	Gm933	1.000	1.000	1.000	1.000	1.000	1.000	0.000	0.000	0.000	381043	Gm933	chr16	-	NM_0012	P26517	protein_cc	Gm1743	predicted	Gm933
7793	1700021K	0.560	0.756	0.423	0.044	0.129	0.166	6.610	5.775	5.370	.	1700021K	chr16	+	NM_1726	P8479	.	.	.	1700021K1
7794	Fyttd1	1.503	1.046	1.572	0.006	0.587	0.052	8.024	8.612	8.676	69823	Fyttd1	chr16	+	NM_0272	P6072	protein_cc	2010005M	forty-two-	Fyttd1
7795	Lrch3	2.333	0.411	0.959	0.040	0.011	0.950	5.555	6.777	5.495	70144	Lrch3	chr16	+	NM_0010	P8478	protein_cc	2210409B	leucine-ric	Lrch3
7796	Iqcg	1.378	0.545	0.752	0.000	0.000	0.037	5.723	6.186	5.311	69707	Iqcg	chr16	-	NM_1783	P17641	protein_cc	2400003L(	IQ motif c	Iqcg
7797	Rpl35a	0.990	1.348	1.334	0.695	0.001	0.041	9.039	9.024	9.455	57808	Rpl35a	chr16	+	NM_0011	P8352	protein_cc	2810431L1	ribosomal	Rpl35a
7798	Lmln	0.549	2.042	1.122	0.479	0.128	0.894	2.385	1.521	2.551	239833	Lmln	chr16	+	NM_1728	P1061	protein_cc	5330415H	leishmano	Lmln
7799	Osbpl11	1.057	1.219	1.288	0.540	0.213	0.424	6.790	6.870	7.155	106326	Osbpl11	chr16	+	NM_1768	P7168	protein_cc	9430097N	oxysterol	Osbpl11
7800	Snx4	1.124	1.298	1.459	0.052	0.025	0.113	8.218	8.387	8.763	69150	Snx4	chr16	+	NM_0805	P10194	protein_cc	1810036H	sorting ne	Snx4
7801	1700007L1	0.502	0.771	0.387	0.003	0.370	0.062	4.834	3.840	3.465	10050274	1700007L1	chr16	-	NR_04570	.	lncRNA	linc-Iqcg|	RIKEN cDN	1700007L1
7802	Zfp148	0.775	0.989	0.766	0.071	0.899	0.166	8.464	8.096	8.080	22661	Zfp148	chr16	+	NM_0117	P26320	protein_cc	2210405J(	zinc finger	Zfp148
7803	Slc12a8	0.980	1.277	1.251	0.965	0.609	0.625	2.237	2.208	2.560	171286	Slc12a8	chr16	+	NM_1342	P1394	protein_cc	CCC9| E33	solute car	Slc12a8
7804	Heg1	1.420	1.201	1.706	0.001	0.042	0.057	4.038	4.545	4.809	77446	Heg1	chr16	+	NM_1752	P21505	protein_cc	4632417D	heart deve	Heg1
7805	Muc13	1.282	1.753	2.246	0.680	0.177	0.109	0.782	1.140	1.949	17063	Muc13	chr16	+	NM_0107	P2028	protein_cc	114/A10|	mucin 13,	Muc13
7806	Itgb5	0.207	1.370	0.283	0.000	0.344	0.021	8.342	6.068	6.522	16419	Itgb5	chr16	+	NM_0105	P7911	protein_cc	AA475909	integrin be	Itgb5
7807	Umps	0.589	1.070	0.630	0.016	0.716	0.023	7.544	6.781	6.878	22247	Umps	chr16	-	NM_0094	P6048	protein_cc	1700095D	uridine mo	Umps
7808	Kalrn	0.401	2.024	0.811	0.037	0.367	0.776	5.048	3.729	4.745	545156	Kalrn	chr16	-	NM_1773	P23210	protein_cc	2210407G	kalirin, Rh	Kalrn
7809	Ropn1	1.000	1.000	1.000	1.000	1.000	1.000	0.000	0.000	0.000	76378	Ropn1	chr16	+	NM_0307	P13189	protein_cc	1700008N	ropporin,	Ropn1
7810	Ccdc14	1.257	1.725	2.169	0.463	0.167	0.253	3.002	3.332	4.118	239839	Ccdc14	chr16	+	NM_1728	P7277	protein_cc	G630039H	coiled-coil	Ccdc14
7811	Mylk	0.153	1.871	0.287	0.000	0.000	0.006	10.338	7.634	8.537	107589	Mylk	chr16	+	NM_1393	P17755	protein_cc	9530072E	myosin, lig	Mylk
7812	1700119H	0.775	1.402	1.086	0.348	0.018	0.822	2.767	2.398	2.885	76649	1700119H	chr16	-	NR_04053	.	lncRNA	.	RIKEN cDN	1700119H1
7813	E130310I0	3.048	0.411	1.253	0.092	0.141	0.775	0.756	2.364	1.082	278725	E130310I0	chr16	-	NR_04572	.	lncRNA	A930019C	RIKEN cDN	E130310I0

7814	Hacd2	0.679	0.889	0.604	0.242	0.783	0.158	10.067	9.509	9.340	70757	Hacd2	chr16	+	NM_0235	P13597	protein_cc	6330408J2	3-hydroxy	Hacd2
7815	Adcy5	0.144	1.322	0.190	0.037	0.233	0.217	2.873	0.077	0.480	224129	Adcy5	chr16	+	NM_0010	P5485	protein_cc	Ac5 | AW1	adenylate	Adcy5
7816	Sec22a	1.698	0.841	1.427	0.002	0.072	0.083	7.360	8.123	7.873	317717	Sec22a	chr16	-	NM_1337	P6265	protein_cc	1810005C	SEC22 hor	Sec22a
7817	Pdia5	1.335	0.686	0.916	0.021	0.016	0.664	8.818	9.234	8.692	72599	Pdia5	chr16	-	NM_0282	P12084	protein_cc	2700053F	protein dis	Pdia5
7818	1600019K	1.000	1.000	1.000	1.000	1.000	1.000	0.000	0.000	0.000	69768	1600019K	chr16	-	NR_04048	.	lncRNA	.	RIKEN cDN	1600019K
7819	Sema5b	0.125	1.615	0.201	0.055	0.000	0.272	3.090	0.087	0.778	20357	Sema5b	chr16	+	NM_0136	P18239	protein_cc	AI893641 |	sema dom	Sema5b
7820	Dirc2	0.411	1.433	0.589	0.000	0.012	0.045	8.295	7.013	7.532	224132	Dirc2	chr16	-	NM_1535	P4399	protein_cc	RCC4	disrupted	Dirc2
7821	Hspbap1	2.567	1.609	4.131	0.024	0.057	0.038	3.296	4.656	5.342	66667	Hspbap1	chr16	+	NM_1751	P13235	protein_cc	3830421G	Hspb asso	Hspbap1
7822	Parp14	6.607	0.213	1.409	0.000	0.000	0.001	7.688	10.412	8.182	547253	Parp14	chr16	-	NM_0010	P4404	protein_cc	1600029O	poly (ADP-	Parp14
7823	Dtx3l	2.612	0.350	0.915	0.003	0.002	0.686	8.832	10.217	8.704	209200	Dtx3l	chr16	-	NM_0010	P8413	protein_cc	AU042200	deltex 3-li	Dtx3l
7824	Parp9	3.778	0.447	1.689	0.000	0.000	0.176	8.687	10.605	9.444	80285	Parp9	chr16	+	NM_0302	P11696	protein_cc	ARTD9 | A'	poly (ADP-	Parp9
7825	Kpna1	1.493	0.959	1.432	0.071	0.511	0.351	7.661	8.239	8.179	16646	Kpna1	chr16	+	NM_0084	P24551	protein_cc	AW494490	karyopher	Kpna1
7826	Wdr5b	2.319	0.780	1.810	0.033	0.325	0.152	3.749	4.963	4.605	69544	Wdr5b	chr16	+	NM_0271	P6060	protein_cc	2310009C	WD repea	Wdr5b
7827	Fam162a	1.698	0.558	0.947	0.000	0.016	0.873	10.100	10.863	10.021	70186	Fam162a	chr16	-	NM_0273	P4370	protein_cc	2310056P	family wit	Fam162a
7828	Ccdc58	1.037	1.025	1.064	0.820	0.875	0.541	8.754	8.807	8.843	381045	Ccdc58	chr16	+	NM_1986	P21402	protein_cc	A930007B	coiled-coil	Ccdc58
7829	Csta1	1.152	0.629	0.725	0.722	1.000	0.422	0.465	0.669	0.000	209294	Csta1	chr16	-	NM_0010	P10023	protein_cc	Csta	cystatin A	Csta1
7830	Stfa2l1	20.794	0.954	19.844	0.000	0.370	0.006	0.002	4.381	4.313	268885	Stfa2l1	chr16	-	NM_1738	P20382	protein_cc	.	stefin A2 l	Stfa2l1
7831	Gm5483	69.068	0.572	39.472	0.019	0.183	0.026	0.330	6.440	5.633	433016	Gm5483	chr16	+	NM_0010	P4224	protein_cc	EG433016	predicted	Gm5483
7832	Gm6815	1.000	1.000	1.000	1.000	1.000	1.000	0.000	0.000	0.000	627962	Gm6815	chr16	-	NR_10268	.	misc_RNA	EG627962	predicted	Gm6815
7833	Gm5416	17.865	2.413	43.114	0.013	0.334	0.373	0.003	4.162	5.433	408196	Gm5416	chr16	+	NM_0010	P9360	protein_cc	EG408196	predicted	Gm5416
7834	2010005H	19.505	0.723	14.107	0.014	0.437	0.212	0.002	4.288	3.821	76770	2010005H	chr16	+	NM_0297	P16014	protein_cc	.	RIKEN cDN	2010005H
7835	Stfa1	3.966	2.025	8.031	0.301	0.205	0.050	0.001	1.989	3.007	20861	Stfa1	chr16	-	NM_0010	P23995	protein_cc	Stefin-3 | S	stefin A1	Stfa1
7836	BC117090	2.197	1.967	4.322	0.295	0.100	0.002	0.001	1.136	2.113	10003885	BC117090	chr16	-	NM_0010	P5261	protein_cc	10003885	cDNA seq	BC117090
7837	BC100530	5.739	0.591	3.394	0.190	0.487	0.136	0.001	2.522	1.764	10003468	BC100530	chr16	-	NM_0010	P10903	protein_cc	.	cDNA seq	BC100530
7838	Stfa2	1.627	2.267	3.687	0.290	0.298	0.423	0.001	0.703	1.883	20862	Stfa2	chr16	-	NM_0010	P21842	protein_cc	Stf2	stefin A2	Stfa2
7839	Stfa3	4.626	1.879	8.692	0.020	0.047	0.047	0.002	2.211	3.121	20863	Stfa3	chr16	-	NM_0252	P24324	protein_cc	Stf3	stefin A3	Stfa3
7840	Casr	1.000	1.000	1.000	1.000	1.000	1.000	0.000	0.000	0.000	12374	Casr	chr16	-	NM_0138	P26782	protein_cc	CaR | Gprc	calcium-se	Casr
7841	Cd86	3.550	0.777	2.757	0.047	0.483	0.175	4.085	5.913	5.548	12524	Cd86	chr16	-	NM_0193	P10472	protein_cc	B7 | B7-2 |	CD86 anti	Cd86
7842	Ildr1	0.642	2.099	1.348	0.444	0.058	0.657	1.348	0.709	1.779	106347	Ildr1	chr16	+	NM_0012	P18286	protein_cc	AU041483	immunogl	Ildr1
7843	Slc15a2	0.152	19.483	2.964	0.000	0.062	0.387	2.868	0.151	4.435	57738	Slc15a2	chr16	-	NM_0213	P15359	protein_cc	8430408C	solute car	Slc15a2
7844	Eaf2	0.287	1.589	0.456	0.154	0.333	0.469	3.315	1.514	2.182	106389	Eaf2	chr16	-	NM_1341	P2870	protein_cc	AW04886	ELL associ	Eaf2
7845	Iqcb1	1.621	0.708	1.147	0.079	0.160	0.334	4.469	5.165	4.667	320299	Iqcb1	chr16	+	NM_1771	P10422	protein_cc	6820449I0	IQ calmod	Iqcb1
7846	4930565N	1.005	1.439	1.446	0.000	0.182	0.423	0.000	0.008	0.532	68306	4930565N	chr16	-	NR_04047	.	lncRNA	4930428L0	RIKEN cDN	4930565N
7847	Golgb1	1.734	0.691	1.197	0.007	0.012	0.471	9.117	9.911	9.377	224139	Golgb1	chr16	+	NM_0300	P18876	protein_cc	4930428L0	golgi auto	Golgb1
7848	Hcls1	5.140	0.530	2.723	0.001	0.011	0.022	6.080	8.442	7.525	15163	Hcls1	chr16	+	NM_0082	P6822	protein_cc	AW21326	hematopo	Hcls1
7849	Fbxo40	1.000	1.000	1.000	1.000	1.000	1.000	0.000	0.000	0.000	207215	Fbxo40	chr16	-	NM_0010	P5965	protein_cc	9830003A	F-box prot	Fbxo40
7850	Polq	1.000	1.000	1.000	1.000	1.000	1.000	0.000	0.000	0.000	77782	Polq	chr16	+	NM_0011	P9363	protein_cc	A430110D	polymeras	Polq
7851	Stxbp5l	0.133	0.947	0.126	0.004	1.000	0.067	2.983	0.078	0.000	207227	Stxbp5l	chr16	-	NM_1724	P1930	protein_cc	9430094L0	syntaxin b	Stxbp5l
7852	Gtf2e1	1.673	0.775	1.296	0.006	0.175	0.485	6.008	6.751	6.383	74197	Gtf2e1	chr16	-	NM_0288	P5029	protein_cc	2610024P	general tra	Gtf2e1
7853	Rabl3	1.521	0.851	1.294	0.021	0.263	0.227	6.961	7.566	7.333	67657	Rabl3	chr16	+	NM_0010	P2565	protein_cc	4930553C	RAB, mem	Rabl3
7854	Hgd	0.102	0.817	0.084	0.000	0.509	0.004	12.407	9.120	8.828	15233	Hgd	chr16	-	NR_02777	.	protein_cc	aku | Hgo	homogent	Hgd
7855	Ndufb4	0.719	1.337	0.961	0.012	0.015	0.791	10.313	9.837	10.255	68194	Ndufb4	chr16	-	NM_0266	P2272	protein_cc	0610006N	NADH deh	Ndufb4
7856	Fstl1	0.875	1.852	1.621	0.109	0.000	0.054	6.709	6.517	7.406	14314	Fstl1	chr16	+	NM_0080	P9143	protein_cc	AI316791 |	follistatin-	Fstl1
7857	Lrrc58	1.826	1.064	1.942	0.000	0.387	0.029	10.681	11.550	11.639	320184	Lrrc58	chr16	+	NM_1770	P21692	protein_cc	1810010N	leucine ric	Lrrc58
7858	Gpr156	1.000	1.000	1.000	1.000	1.000	1.000	0.000	0.000	0.000	239845	Gpr156	chr16	+	NM_1533	P11	protein_cc	Gababl	G protein-	Gpr156
7859	4930542C	1.000	1.000	1.000	1.000	1.000	1.000	0.000	0.000	0.000	75175	4930542C	chr16	+	NR_04056	.	lncRNA	.	RIKEN cDN	4930542C
7860	BC031361	1.138	1.841	2.095	0.762	0.115	0.112	1.829	2.015	2.896	414072	BC031361	chr16	-	NR_03322	.	lncRNA	.	cDNA seq	BC031361
7861	Gsk3b	0.898	1.141	1.024	0.291	0.210	0.667	9.495	9.340	9.530	56637	Gsk3b	chr16	+	NM_0198	P1521	protein_cc	7330414F	glycogen s	Gsk3b
7862	Nr1i2	0.206	1.634	0.337	0.000	0.001	0.001	9.374	7.098	7.806	18171	Nr1i2	chr16	-	NM_0109	P14880	protein_cc	mPXR | PX	nuclear re	Nr1i2
7863	Maats1	2.149	0.465	1.000	0.306	1.000	0.423	0.000	1.104	0.000	320214	Maats1	chr16	-	NM_0010	P22731	protein_cc	4932425I2	MYCBP-as	Maats1
7864	Cox17	1.239	0.852	1.056	0.006	0.004	0.593	9.870	10.179	9.948	12856	Cox17	chr16	+	NM_0010	P23199	protein_cc	AI037035	cytochrom	Cox17
7865	Popdc2	0.703	0.495	0.348	0.568	0.065	0.480	2.004	1.496	0.482	64082	Popdc2	chr16	+	NM_0223	P13314	protein_cc	AV006127	popeye do	Popdc2
7866	Pla1a	1.203	0.874	1.052	0.032	0.338	0.837	9.318	9.584	9.390	85031	Pla1a	chr16	-	NM_1341	P13522	protein_cc	AA986889	phospholi	Pla1a
7867	Adprh	5.338	0.515	2.747	0.004	0.024	0.051	6.203	8.619	7.660	11544	Adprh	chr16	-	NM_0074	P12836	protein_cc	Arh1	ADP-ribos	Adprh
7868	Cd80	3.733	1.064	3.973	0.000	0.698	0.078	1.589	3.489	3.579	12519	Cd80	chr16	+	NM_0098	P5156	protein_cc	B71 | Cd28	CD80 anti	Cd80
7869	Timmdc1	0.370	1.479	0.548	0.000	0.327	0.227	9.066	7.633	8.198	76916	Timmdc1	chr16	-	NM_0242	P10588	protein_cc	2810021C	translocas	Timmdc1
7870	Poglut1	1.388	0.975	1.354	0.012	0.801	0.172	6.970	7.444	7.407	224143	Poglut1	chr16	-	NM_0013	P14757	protein_cc	9630046K	protein O-	Poglut1
7871	Tmem39a	2.858	0.784	2.240	0.019	0.264	0.023	5.215	6.730	6.378	67846	Tmem39a	chr16	+	NM_0012	P18551	protein_cc	2610033C	transmem	Tmem39a
7872	Arhgap31	1.534	0.887	1.361	0.179	0.615	0.294	6.171	6.789	6.616	12549	Arhgap31	chr16	-	NM_0202	P10456	protein_cc	5830477L0	Rho GTPas	Arhgap31
7873	B4galt4	0.519	0.457	0.237	0.025	0.165	0.041	4.284	3.339	2.208	56375	B4galt4	chr16	+	NM_0198	P5296	protein_cc	91304020	UDP-Gal:b	B4galt4
7874	Upk1b	0.184	0.959	0.176	0.047	1.000	0.221	2.503	0.060	0.000	22268	Upk1b	chr16	-	NM_1789	P19326	protein_cc	AI413235 |	uroplakin	Upk1b
7875	4930435E	1.000	1.000	1.000	1.000	1.000	1.000	0.000	0.000	0.000	74663	4930435E	chr16	-	NM_0290	P16886	protein_cc	TSCPA	RIKEN cDN	4930435E1
7876	Igsf11	0.464	1.682	0.781	0.043	0.153	0.091	6.791	5.685	6.436	207683	Igsf11	chr16	+	NM_1705	P5820	protein_cc	1700025L0	immunogl	Igsf11
7877	Lsamp	2.049	0.362	0.742	0.058	1.000	0.422	0.431	1.466	0.000	268890	Lsamp	chr16	+	NM_1755	P14748	protein_cc	5430428I1	limbic syst	Lsamp
7878	Gap43	1.010	1.825	1.844	0.000	0.182	0.423	0.000	0.015	0.883	14432	Gap43	chr16	-	NM_0080	P21823	protein_cc	B-50 | Bas	growth as	Gap43
7879	4932412D	1.000	1.000	1.000	1.000	1.000	1.000	0.000	0.000	0.000	75722	4932412D	chr16	-	NR_04052	.	lncRNA	ENSMUSG	RIKEN cDN	4932412D
7880	Gm19522	0.470	0.493	0.232	0.036	0.169	0.047	4.247	3.157	2.137	10050304	Gm19522	chr16	-	NR_04040	.	lncRNA	.	predicted	Gm19522
7881	Zbtb20	0.223	2.135	0.477	0.000	0.012	0.040	8.845	6.682	7.776	56490	Zbtb20	chr16	+	NM_0012	P13622	protein_cc	1300017A	zinc finger	Zbtb20
7882	BC002163	0.736	1.022	0.752	0.011	0.812	0.026	9.677	9.234	9.265	170658	BC002163	chr16	-	NR_00244	.	misc_RNA	.	cDNA seq	BC002163
7883	Gm15713	1.258	1.192	1.499	0.701	0.779	0.672	0.797	1.127	1.381	626693	Gm15713	chr16	-	NR_04602	.	lncRNA	OTTMUSG	predicted	Gm15713
7884	Tigit	1.000	1.000	1.000	1.000	1.000	1.000	0.000	0.000	0.000	10004331	Tigit	chr16	-	NM_0011	P14143	protein_cc	ENSMUSG	T cell imm	Tigit
7885	Drd3	1.000	1.000	1.000	1.000	1.000	1.000	0.000	0.000	0.000	13490	Drd3	chr16	+	NM_0078	P5122	protein_cc	D3R	dopamine	Drd3
7886	Qtrtd1	1.875	0.431	0.808	0.195	0.131	0.090	4.154	5.060	3.846	106248	Qtrtd1	chr16	-	NM_0291	P19337	protein_cc	3110012M	queuine tR	Qtrtd1
7887	2610015P	0.581	0.768	0.446	0.001	0.089	0.014	6.874	6.090	5.709	.	2610015P	chr16	+	NM_0278	P6733	.	.	.	2610015P
7888	Zdhhc23	2.700	0.370	1.000	0.305	1.000	0.423	0.001	1.433	0.000	332175	Zdhhc23	chr16	-	NM_0010	P13866	protein_cc	Gm1751 |	zinc finger	Zdhhc23
7889	Gramd1c	0.396	1.597	0.632	0.000	0.004	0.022	7.806	6.469	7.144	207798	Gramd1c	chr16	-	NM_0011	P7307	protein_cc	4921521N	GRAM dom	Gramd1c
7890	Atp6v1a	1.934	0.746	1.443	0.000	0.006	0.074	9.958	10.909	10.487	11964	Atp6v1a	chr16	-	NM_0075	P18286	protein_cc	AI647066 |	ATPase, H	Atp6v1a
7891	Naa50	0.961	1.167	1.121	0.444	0.026	0.193	9.464	9.406	9.629	72117	Naa50	chr16	+	NM_0281	P18231	protein_cc	2600005K	N(alpha)-a	Naa50
7892	Gm608	0.406	1.509	0.613	0.002	0.000	0.096	7.809	6.509	7.102	.	Gm608	chr16	+	NM_0010	P11022	.	.	.	Gm608
7893	Sidt1	2.189	0.566	1.240	0.100	0.151	0.761	1.059	2.189	1.369	320007	Sidt1	chr16	-	NM_1980	P281	protein_cc	AW04592	SID1 trans	Sidt1
7894	Spice1	1.053	0.913	0.961	0.932	0.784	0.971	3.931	4.005	3.874	212514	Spice1	chr16	+	NM_1445	P24420	protein_cc	Ccdc52 | D	spindle an	Spice1
7895	Cfap44	1.000	1.000	1.000	1.000	1.000	1.000	0.000	0.000	0.000	212517	Cfap44	chr16	+	NM_0010	P4441	protein_cc	6330444M	cilia and fl	Cfap44
7896	Boc	0.534	0.990	0.528	0.000	1.000	0.015	0.921	0.015	0.000	117606	Boc	chr16	-	NM_1725	P5084	protein_cc	4732455C	biregional	Boc
7897	BC027231	1.736	0.877	1.522	0.050	0.652	0.467	6.171	6.967	6.777	.	BC027231	chr16	+	NM_1459	P17740	.	.	.	BC027231
7898	Gtpbp8	2.280	0.620	1.413	0.126	0.258	0.175	5.600	6.789	6.099	66067	Gtpbp8	chr16	-	NM_0253	P6160	protein_cc	0610037H	GTP-bindi	Gtpbp8
7899	Cd200r1	0.897	1.175	1.055	0.855	0.576	0.955	2.371	2.215	2.448	57781	Cd200r1	chr16	+	NM_0213	P7491	protein_cc	CD200R | I	CD200 rec	Cd200r1
7900	Cd200r4	3.024	0.086	0.259	0.000	0.000	0.044	2.396	3.992	0.447	239849	Cd200r4	chr16	+	NM_2072	P10877	protein_cc	F630107N	CD200 rec	Cd200r4
7901	Cd200r2	1.000	1.000	1.000	1.000	1.000	1.000	0.000	0.000	0.000	271375	Cd200r2	chr16	+	NM_2065	P12207	protein_cc	AY230198	Cd200 rec	Cd200r2
7902	Cd200r3	0.321	1.382	0.443	0.000	0.201	0.061	1.678	0.038	0.505	74603	Cd200r3	chr16	+	NM_0290	P22457	protein_cc	4733401I1	CD200 rec	Cd200r3
7903	Ccdc80	0.812	0.669	0.544	0.177	0.026	0.081	8.035	7.736	7.156	67896	Ccdc80	chr16	+	NM_0264	P25943	protein_cc	2610001E	coiled-coil	Ccdc80
7904	Slc35a5	0.591	0.908	0.536	0.029	0.394	0.123	6.767	6.008	5.868	74102	Slc35a5	chr16	-	NM_0287	P15342	protein_cc	1010001J0	solute car	Slc35a5

7905	Atg3	0.574	0.688	0.395	0.049	0.090	0.118	9.608	8.808	8.268	67841	Atg3	chr16	+	NM_0264	P24110	protein_cc	2610016C	autophagy	Atg3
7906	Btla	2.707	0.081	0.220	0.010	1.000	0.146	2.182	3.619	0.000	208154	Btla	chr16	+	NM_1775	P23602	protein_cc	A630002H	B and T ly	Btla
7907	Cd200	27.491	0.427	11.733	0.033	0.129	0.005	1.784	6.565	5.337	17470	Cd200	chr16	-	NM_0108	P26668	protein_cc	Mox2| OX	CD200 ant	Cd200
7908	Gm609	1.000	1.000	1.000	1.000	1.000	1.000	0.000	0.000	0.000	208166	Gm609	chr16	-	NM_0010	P6401	protein_cc	Gm1749|	predicted	Gm609
7909	Slc9c1	1.000	1.000	1.000	1.000	1.000	1.000	0.000	0.000	0.000	208169	Slc9c1	chr16	+	NM_1981	P3928	protein_cc	Gm610| N	solute car	Slc9c1
7910	Gcsam	0.283	0.974	0.275	0.002	1.000	0.052	1.860	0.038	0.000	14525	Gcsam	chr16	+	NM_0080	P8805	protein_cc	Gcet| Gce	germinal c	Gcsam
7911	BC016579	9.463	0.053	0.500	0.111	1.000	0.421	1.000	4.242	0.000	212998	BC016579	chr16	-	NM_1453	P24032	protein_cc	.	cDNA seq	BC016579
7912	Tmprss7	4.256	0.408	1.736	0.281	0.407	0.675	1.468	3.557	2.264	208171	Tmprss7	chr16	-	NM_1724	P11959	protein_cc	B230219I2	transmem	Tmprss7
7913	Tagln3	0.291	2.508	0.730	0.002	0.186	0.628	1.837	0.056	1.383	56370	Tagln3	chr16	-	NM_0197	P24483	protein_cc	2700038H	transgelin	Tagln3
7914	Abhd10	1.876	0.640	1.201	0.068	0.046	0.694	5.150	6.058	5.414	213012	Abhd10	chr16	-	NM_0012	P15968	protein_cc	.	abhydrola	Abhd10
7915	Phldb2	0.751	1.592	1.195	0.064	0.114	0.662	8.703	8.289	8.959	208177	Phldb2	chr16	-	NM_1534	P2064	protein_cc	AV253284	pleckstrin	Phldb2
7916	Plcxd2	0.388	1.425	0.552	0.000	0.482	0.313	8.897	7.530	8.041	433022	Plcxd2	chr16	-	NM_0011	P25606	protein_cc	AW49463	phosphati	Plcxd2
7917	Cd96	0.459	0.619	0.284	0.145	1.000	0.238	1.816	0.692	0.000	84544	Cd96	chr16	-	NM_0324	P16508	protein_cc	1700109I1	CD96 anti	Cd96
7918	Gm4737	0.370	1.111	0.410	0.000	0.234	0.012	12.783	11.346	11.498	11615	Gm4737	chr16	-	NM_0013	P15373	protein_cc	AL024110	predicted	Gm4737
7919	Pvrl3	0.561	0.996	0.559	0.002	0.965	0.047	9.346	8.513	8.508	.	Pvrl3	chr16	-	NM_0214	P25289	.	.	.	Pvrl3
7920	Dppa4	1.000	1.000	1.000	1.000	1.000	1.000	0.000	0.000	0.000	73693	Dppa4	chr16	+	NM_0010	P15019	protein_cc	2410091M	developm	Dppa4
7921	Dppa2	1.005	1.439	1.446	0.000	0.182	0.423	0.000	0.008	0.532	73703	Dppa2	chr16	+	NM_0286	P18941	protein_cc	2410088E	developm	Dppa2
7922	Gm5485	1.000	1.000	1.000	1.000	1.000	1.000	0.000	0.000	0.000	433023	Gm5485	chr16	-	NR_01537	.	lncRNA	EG433023	predicted	Gm5485
7923	Morc1	1.030	3.946	4.065	0.000	0.101	0.320	0.001	0.044	2.024	17450	Morc1	chr16	+	NM_0108	P12233	protein_cc	Morc	microrchid	Morc1
7924	Trat1	1.580	0.633	1.000	0.305	1.000	0.423	0.000	0.660	0.000	77647	Trat1	chr16	-	NM_1982	P15074	protein_cc	C030046M	T cell rece	Trat1
7925	Retnlb	5.053	0.198	0.999	0.307	1.000	0.423	0.001	2.338	0.000	57263	Retnlb	chr16	+	NM_0238	P14581	protein_cc	9030012B	resistin lik	Retnlb
7926	Retnla	0.635	2.355	1.495	0.012	0.059	0.447	2.691	2.035	3.271	57262	Retnla	chr16	+	NM_0205	P19484	protein_cc	1810019L	resistin lik	Retnla
7927	Retnlg	8.465	0.722	6.115	0.002	0.101	0.003	4.319	7.401	6.932	245195	Retnlg	chr16	+	NM_1815	P17148	protein_cc	Fizz3| Reli	resistin lik	Retnlg
7928	Dzip3	2.771	1.027	2.846	0.001	0.939	0.350	4.453	5.923	5.962	224170	Dzip3	chr16	-	NM_0273	P5487	protein_cc	2310047C	DAZ intera	Dzip3
7929	C330027C	0.408	3.377	1.379	0.009	0.103	0.657	3.706	2.415	4.170	224171	C330027C	chr16	+	NM_1726	P15661	protein_cc	AA408511	RIKEN cDN	C330027C
7930	Myh15	0.716	0.995	0.712	0.182	1.000	0.423	0.490	0.008	0.000	667772	Myh15	chr16	+	NM_0011	P3692	protein_cc	EG667772	myosin, he	Myh15
7931	Ift57	1.970	1.050	2.068	0.037	0.769	0.231	3.869	4.847	4.917	73916	Ift57	chr16	+	NM_0286	P12905	protein_cc	4833420A	intraflagel	Ift57
7932	Cd47	1.586	0.955	1.516	0.009	0.772	0.244	9.749	10.415	10.349	16423	Cd47	chr16	+	NM_0105	P17623	protein_cc	9130415E	CD47 anti	Cd47
7933	Gm6936	1.000	1.000	1.000	1.000	1.000	1.000	0.000	0.000	0.000	628951	Gm6936	chr16	+	NR_04500	.	lncRNA	628951	predicted	Gm6936
7934	Gm4827	1.010	1.825	1.844	0.000	0.182	0.423	0.000	0.015	0.883	224180	Gm4827	chr16	-	NR_04593	.	lncRNA	B130050K	predicted	Gm4827
7935	Bbx	2.409	0.734	1.769	0.002	0.011	0.080	7.339	8.607	8.161	70508	Bbx	chr16	-	NM_0274	P25840	protein_cc	5530401J	bobby sox	Bbx
7936	G730013B	1.580	0.633	1.000	0.305	1.000	0.423	0.000	0.660	0.000	619312	G730013B	chr16	+	NR_04038	.	lncRNA	linc-Cblb|	RIKEN cDN	G730013B
7937	Ccdc54	2.700	0.370	1.000	0.305	1.000	0.423	0.001	1.433	0.000	69339	Ccdc54	chr16	-	NM_0270	P18735	protein_cc	1700007N	coiled-coil	Ccdc54
7938	4930542D	1.000	1.000	1.000	1.000	1.000	1.000	0.000	0.000	0.000	75211	4930542D	chr16	+	NR_04056	.	lncRNA	.	RIKEN cDN	4930542D
7939	5330426P	20.687	0.702	14.517	0.110	0.560	0.030	0.333	4.704	4.193	.	5330426P	chr16	-	NR_02830	.	.	.	.	5330426P
7940	1700116B	1.000	1.000	1.000	1.000	1.000	1.000	0.000	0.000	0.000	78639	1700116B	chr16	+	NR_13100	.	lncRNA	.	RIKEN cDN	1700116B
7941	Cblb	2.214	0.828	1.832	0.297	0.759	0.464	2.494	3.640	3.368	208650	Cblb	chr16	+	NM_0010	P22403	protein_cc	AI429560|	Casitas B-l	Cblb
7942	Alcam	0.528	1.028	0.543	0.001	0.784	0.068	9.673	8.753	8.793	11658	Alcam	chr16	-	NM_0096	P23714	protein_cc	AI853494|	activated	Alcam
7943	4930404A	1.000	1.000	1.000	1.000	1.000	1.000	0.000	0.000	0.000	73820	4930404A	chr16	-	NR_04049	.	lncRNA		RIKEN cDN	4930404A
7944	Zpld1	0.332	0.978	0.324	0.181	1.000	0.423	1.625	0.032	0.000	239852	Zpld1	chr16	-	NM_1787	P13986	protein_cc	9430016A	zona pellu	Zpld1
7945	Nfkbiz	35.989	0.567	20.418	0.000	0.005	0.050	4.882	10.051	9.233	80859	Nfkbiz	chr16	-	NM_0011	P23109	protein_cc	AA408868	nuclear fa	Nfkbiz
7946	Nxpe3	2.328	0.378	0.879	0.020	0.015	0.045	3.624	4.843	3.439	385658	Nxpe3	chr16	-	NM_0011	P21303	protein_cc	Fam55c| (	neurexoph	Nxpe3
7947	Cep97	0.728	2.272	1.655	0.034	0.003	0.126	4.970	4.512	5.697	74201	Cep97	chr16	-	NM_0288	P9020	protein_cc	2810403B	centrosom	Cep97
7948	Rpl24	1.230	1.593	1.960	0.010	0.000	0.009	9.659	9.958	10.630	68193	Rpl24	chr16	+	NM_0242	P25922	protein_cc	0610008L	ribosomal	Rpl24
7949	Zbtb11os1	0.326	2.603	0.849	0.019	0.026	0.458	5.781	4.165	5.545	66391	Zbtb11os1	chr16	-	NR_02796	.	lncRNA	2310061J	zinc finger	Zbtb11os1
7950	Zbtb11	1.064	0.529	0.563	0.628	0.014	0.012	7.352	7.442	6.524	271377	Zbtb11	chr16	+	NM_1730	P4456	protein_cc	9230110G	zinc finger	Zbtb11
7951	Pcnp	0.748	0.995	0.744	0.034	0.967	0.067	9.095	8.676	8.669	76302	Pcnp	chr16	-	NM_0010	P4938	protein_cc	1110018D	PEST prote	Pcnp
7952	Trmt10c	1.927	0.895	1.725	0.221	0.782	0.205	6.937	7.884	7.723	52575	Trmt10c	chr16	-	NM_0290	P3539	protein_cc	1300018J1	tRNA met	Trmt10c
7953	Senp7	0.629	0.879	0.553	0.010	0.775	0.389	6.771	6.103	5.916	66315	Senp7	chr16	+	NM_0010	P15410	protein_cc	2410152H	SUMO1/se	Senp7
7954	Impg2	0.113	7.507	0.848	0.001	0.134	0.834	4.969	1.822	4.731	224224	Impg2	chr16	+	NM_1748	P26301	protein_cc	IPM200| I	interphoto	Impg2
7955	Abi3bp	1.037	1.043	1.082	0.920	0.789	0.897	5.016	5.069	5.129	320712	Abi3bp	chr16	+	NM_0010	P22575	protein_cc	5033411B	ABI gene f	Abi3bp
7956	Tfg	2.710	0.489	1.326	0.016	0.033	0.170	9.313	10.751	9.720	21787	Tfg	chr16	-	NM_0196	P21629	protein_cc	AI173908	Trk-fused	Tfg
7957	Adgrg7	1.010	1.885	1.904	0.000	0.182	0.423	0.000	0.015	0.929	239853	Adgrg7	chr16	-	NM_1728	P22272	protein_cc	9130020O	adhesion	Adgrg7
7958	Tmem45a	0.472	2.631	1.241	0.216	0.052	0.588	2.870	1.786	3.182	56277	Tmem45a	chr16	-	NM_0196	P16955	protein_cc	C630002M	transmem	Tmem45a
7959	Tmem45a	1.000	1.000	1.000	1.000	1.000	1.000	0.000	0.000	0.000	69457	Tmem45a	chr16	-	NM_1832	P9695	protein_cc	2310005G	transmem	Tmem45a
7960	Tomm70a	0.634	1.661	1.053	0.001	0.000	0.211	10.404	9.745	10.477	28185	Tomm70a	chr16	+	NM_1385	P12372	protein_cc	2610044B	translocas	Tomm70a
7961	Nit2	0.188	0.867	0.163	0.001	0.149	0.025	10.443	8.034	7.828	52633	Nit2	chr16	-	NM_0231	P10642	protein_cc	1190017B	nitrilase fa	Nit2
7962	Tbc1d23	1.708	0.964	1.648	0.008	0.846	0.348	5.714	6.487	6.435	67581	Tbc1d23	chr16	-	NM_0262	P6240	protein_cc	4930541A	TBC1 dom	Tbc1d23
7963	Tmem30c	1.000	1.000	1.000	1.000	1.000	1.000	0.000	0.000	0.000	71027	Tmem30c	chr16	-	NM_0276	P17712	protein_cc	4933401B	transmem	Tmem30c
7964	Cmss1	0.944	1.356	1.280	0.843	0.013	0.597	5.075	4.992	5.431	66497	Cmss1	chr16	-	NM_0255	P17075	protein_cc	1110001A	cms small	Cmss1
7965	Filip1l	1.472	0.749	1.102	0.027	0.072	0.489	6.760	7.318	6.901	78749	Filip1l	chr16	+	NM_0010	P12964	protein_cc	4631422O	filamin A i	Filip1l
7966	Col8a1	0.375	3.619	1.359	0.003	0.064	0.635	4.132	2.718	4.574	12837	Col8a1	chr16	-	NM_0077	P18327	protein_cc	Col8a-1	collagen, t	Col8a1
7967	Dcbld2	1.515	1.185	1.796	0.003	0.343	0.193	5.093	5.693	5.938	73379	Dcbld2	chr16	+	NM_0285	P13542	protein_cc	1700055P	discoidin,	Dcbld2
7968	St3gal6	0.635	1.049	0.666	0.094	0.877	0.416	5.343	4.686	4.756	54613	St3gal6	chr16	-	NM_0187	P1019	protein_cc	1700023B	ST3 beta-g	St3gal6
7969	Gm813	0.716	0.995	0.712	0.182	1.000	0.423	0.490	0.008	0.000	328695	Gm813	chr16	-	NM_0010	P26477	protein_cc	Gm1781	predicted	Gm813
7970	E330017A	1.000	1.000	1.000	1.000	1.000	1.000	0.000	0.000	0.000	224247	E330017A	chr16	-	NM_1750	P6873	protein_cc	.	RIKEN cDN	E330017A
7971	Cpox	0.135	2.289	0.308	0.000	0.175	0.074	11.262	8.368	9.563	12892	Cpox	chr16	+	NM_0077	P2101	protein_cc	cac| Cpo|	coproporp	Cpox
7972	Gpr15	1.000	1.000	1.000	1.000	1.000	1.000	0.000	0.000	0.000	71223	Gpr15	chr16	-	NM_0011	P1339	protein_cc	4933really	G protein-	Gpr15
7973	Cldnd1	1.585	1.087	1.724	0.036	0.345	0.083	7.099	7.764	7.884	224250	Cldnd1	chr16	+	NR_04551	.	protein_cc	1110019C	claudin do	Cldnd1
7974	Olfr172	1.000	1.000	1.000	1.000	1.000	1.000	0.000	0.000	0.000	259003	Olfr172	chr16	-	NM_1470	P11017	protein_cc	MOR184-2	olfactory r	Olfr172
7975	Olfr173	1.000	1.000	1.000	1.000	1.000	1.000	0.000	0.000	0.000	259002	Olfr173	chr16	-	NM_1470	P6523	protein_cc	MOR184-3	olfactory r	Olfr173
7976	Olfr175-ps	1.000	1.000	1.000	1.000	1.000	1.000	0.000	0.000	0.000	259004	Olfr175-ps	chr16	-	NM_1470	P3768	protein_cc	MOR184-1	olfactory r	Olfr175-ps
7977	Olfr176	1.000	1.000	1.000	1.000	1.000	1.000	0.000	0.000	0.000	.	Olfr176	chr16	-	NM_1469	P24058	.	.	.	Olfr176
7978	Olfr177	1.000	1.000	1.000	1.000	1.000	1.000	0.000	0.000	0.000	258998	Olfr177	chr16	-	NM_1469	P13927	protein_cc	MOR184-7	olfactory r	Olfr177
7979	Olfr178	1.000	1.000	1.000	1.000	1.000	1.000	0.000	0.000	0.000	258999	Olfr178	chr16	-	NM_1469	P12417	protein_cc	MOR184-6	olfactory r	Olfr178
7980	Olfr180	1.000	1.000	1.000	1.000	1.000	1.000	0.000	0.000	0.000	258178	Olfr180	chr16	-	NM_0010	P10034	protein_cc	MOR184-1	olfactory r	Olfr180
7981	Olfr181	1.000	1.000	1.000	1.000	1.000	1.000	0.000	0.000	0.000	259001	Olfr181	chr16	-	NM_1469	P7674	protein_cc	MOR184-4	olfactory r	Olfr181
7982	Olfr183	1.000	1.000	1.000	1.000	1.000	1.000	0.000	0.000	0.000	258478	Olfr183	chr16	+	NM_1464	P12071	protein_cc	MOR183-2	olfactory r	Olfr183
7983	Olfr186	1.000	1.000	1.000	1.000	1.000	1.000	0.000	0.000	0.000	258318	Olfr186	chr16	-	NM_1463	P6901	protein_cc	MOR183-9	olfactory r	Olfr186
7984	Olfr187	1.000	1.000	1.000	1.000	1.000	1.000	0.000	0.000	0.000	258319	Olfr187	chr16	-	NM_1463	P8704	protein_cc	MOR183-8	olfactory r	Olfr187
7985	Olfr190	1.000	1.000	1.000	1.000	1.000	1.000	0.000	0.000	0.000	258592	Olfr190	chr16	-	NM_1463	P1002	protein_cc	MOR183-4	olfactory r	Olfr190
7986	Olfr191	1.000	1.000	1.000	1.000	1.000	1.000	0.000	0.000	0.000	258035	Olfr191	chr16	-	NM_0010	P23859	protein_cc	MOR183-5	olfactory r	Olfr191
7987	Olfr192	1.000	1.000	1.000	1.000	1.000	1.000	0.000	0.000	0.000	404309	Olfr192	chr16	-	NM_2075	P11075	protein_cc	.	olfactory r	Olfr192
7988	Olfr193	1.000	1.000	1.000	1.000	1.000	1.000	0.000	0.000	0.000	257972	Olfr193	chr16	-	NM_0010	P18576	protein_cc	MOR113-7	olfactory r	Olfr193
7989	Olfr194	1.000	1.000	1.000	1.000	1.000	1.000	0.000	0.000	0.000	433031	Olfr194	chr16	-	NM_0010	P11341	protein_cc	MOR182-1	olfactory r	Olfr194
7990	Olfr195	1.000	1.000	1.000	1.000	1.000	1.000	0.000	0.000	0.000	259000	Olfr195	chr16	+	NM_1469	P23495	protein_cc	MOR184-5	olfactory r	Olfr195
7991	Olfr196	1.000	1.000	1.000	1.000	1.000	1.000	0.000	0.000	0.000	258775	Olfr196	chr16	-	NM_1467	P18669	protein_cc	MOR183-1	olfactory r	Olfr196
7992	Olfr197	1.000	1.000	1.000	1.000	1.000	1.000	0.000	0.000	0.000	258477	Olfr197	chr16	-	NM_1464	P11991	protein_cc	MOR183-3	olfactory r	Olfr197
7993	Olfr198	1.000	1.000	1.000	1.000	1.000	1.000	0.000	0.000	0.000	258036	Olfr198	chr16	-	NM_0010	P6337	protein_cc	MOR182-8	olfactory r	Olfr198
7994	Olfr199	1.000	1.000	1.000	1.000	1.000	1.000	0.000	0.000	0.000	404310	Olfr199	chr16	-	NM_2075	P17624	protein_cc	GA_x5J8B	olfactory r	Olfr199
7995	Olfr201	1.000	1.000	1.000	1.000	1.000	1.000	0.000	0.000	0.000	258996	Olfr201	chr16	-	NM_1469	P15796	protein_cc	MOR182-2	olfactory r	Olfr201

7996	Olfr202	1.000	1.000	1.000	1.000	1.000	1.000	0.000	0.000	0.000	258997	Olfr202	chr16	-	NM_1469	P24802	protein_cc	MOR182-1	olfactory r	Olfr202
7997	Olfr203	1.000	1.000	1.000	1.000	1.000	1.000	0.000	0.000	0.000	258479	Olfr203	chr16	+	NM_1464	P11344	protein_cc	MOR182-5	olfactory r	Olfr203
7998	Olfr204	1.000	1.000	1.000	1.000	1.000	1.000	0.000	0.000	0.000	258994	Olfr204	chr16	-	NM_1469	P7185	protein_cc	MOR182-3	olfactory r	Olfr204
7999	Olfr205	1.000	1.000	1.000	1.000	1.000	1.000	0.000	0.000	0.000	257881	Olfr205	chr16	-	NM_0010	P26754	protein_cc	GA_x54KR	olfactory r	Olfr205
8000	Olfr206	1.000	1.000	1.000	1.000	1.000	1.000	0.000	0.000	0.000	258993	Olfr206	chr16	-	NM_1469	P20350	protein_cc	MOR182-4	olfactory r	Olfr206
8001	Olfr207	1.000	1.000	1.000	1.000	1.000	1.000	0.000	0.000	0.000	.	Olfr207	chr16	-	NM_0010	P13938	.	.	.	Olfr207
8002	Olfr208	1.000	1.000	1.000	1.000	1.000	1.000	0.000	0.000	0.000	.	Olfr208	chr16	-	NM_1463	P13938	.	.	.	Olfr208
8003	Olfr209	1.000	1.000	1.000	1.000	1.000	1.000	0.000	0.000	0.000	404311	Olfr209	chr16	-	NM_2075	P13938	protein_cc	MOR182-6	olfactory r	Olfr209
8004	Gabrr3	1.000	1.000	1.000	1.000	1.000	1.000	0.000	0.000	0.000	328699	Gabrr3	chr16	+	NM_0010	P21675	protein_cc	.	gamma-ar	Gabrr3
8005	1700022E	1.000	1.000	1.000	1.000	1.000	1.000	0.000	0.000	0.000	75636	1700022E	chr16	-	NR_04056	.	lncRNA	.	RIKEN cDN	1700022E
8006	Mina	1.527	0.717	1.095	0.011	0.061	0.697	7.169	7.780	7.300	67014	Mina	chr16	+	NM_0259	P26384	protein_cc	1810047J	myc induc	Mina
8007	Crybg3	1.372	0.808	1.108	0.008	0.063	0.539	7.618	8.074	7.766	224273	Crybg3	chr16	-	NM_1748	P12020	protein_cc	5031404N	beta-gamr	Crybg3
8008	Arl6	1.300	1.746	2.269	0.123	0.002	0.045	4.529	4.907	5.711	56297	Arl6	chr16	-	NM_0196	P1985	protein_cc	1110018H	ADP-ribos	Arl6
8009	4930547E	2.134	0.469	1.000	0.305	1.000	0.423	0.000	1.094	0.000	75145	4930547E	chr16	+	NR_04056	.	lncRNA	.	RIKEN cDN	4930547E
8010	Epha6	1.000	1.000	1.000	1.000	1.000	1.000	0.000	0.000	0.000	13840	Epha6	chr16	-	NM_0079	P25791	protein_cc	Ehk2| Hek	Eph recep	Epha6
8011	Nsun3	2.003	0.796	1.595	0.092	0.500	0.642	1.677	2.679	2.351	106338	Nsun3	chr16	-	NM_1789	P405	protein_cc	6720484A	NOL1/NO	Nsun3
8012	Arl13b	0.905	0.471	0.427	0.645	0.064	0.024	6.765	6.621	5.536	68146	Arl13b	chr16	-	NM_0265	P11325	protein_cc	A530097K	ADP-ribos	Arl13b
8013	Stx19	1.000	1.000	1.000	1.000	1.000	1.000	0.000	0.000	0.000	68159	Stx19	chr16	+	NM_0265	P22387	protein_cc	A030009B	syntaxin 1	Stx19
8014	Pros1	1.127	1.367	1.541	0.483	0.059	0.025	9.495	9.668	10.119	19128	Pros1	chr16	+	NM_0111	P1504	protein_cc	AW21436	protein S (	Pros1
8015	Epha3	0.552	0.990	0.547	0.182	1.000	0.423	0.871	0.015	0.000	13837	Epha3	chr16	-	NM_0101	P4762	protein_cc	AW49208	Eph recep	Epha3
8016	Csnka2ip	1.000	1.000	1.000	1.000	1.000	1.000	0.000	0.000	0.000	224291	Csnka2ip	chr16	-	NM_1738	P24814	protein_cc	Ckt2	casein kin	Csnka2ip
8017	4930453N	2.121	0.912	1.933	0.032	0.634	0.166	7.361	8.445	8.312	67609	4930453N	chr16	-	NM_0262	P17472	protein_cc	AI649104|	RIKEN cDN	4930453N
8018	Zfp654	1.314	0.682	0.896	0.157	0.002	0.769	6.033	6.427	5.875	72020	Zfp654	chr16	-	NM_0280	P4711	protein_cc	1600021C	zinc finger	Zfp654
8019	Cggbp1	1.303	0.806	1.051	0.128	0.152	0.715	9.333	9.715	9.405	106143	Cggbp1	chr16	+	NM_1786	P297	protein_cc	AA960172	CGG triple	Cggbp1
8020	Htr1f	1.000	1.000	1.000	1.000	1.000	1.000	0.000	0.000	0.000	15557	Htr1f	chr16	-	NM_0083	P1593	protein_cc	Htr1eb	5-hydroxy	Htr1f
8021	Pou1f1	1.000	1.000	1.000	1.000	1.000	1.000	0.000	0.000	0.000	18736	Pou1f1	chr16	+	NM_0088	P7222	protein_cc	dw| dwarf	POU doma	Pou1f1
8022	Chmp2b	1.707	1.243	2.122	0.006	0.186	0.099	8.793	9.565	9.878	68942	Chmp2b	chr16	-	NM_0268	P19367	protein_cc	1190006E	charged m	Chmp2b
8023	Vgll3	0.241	2.873	0.693	0.116	0.001	0.671	2.117	0.066	1.589	73569	Vgll3	chr16	+	NM_0285	P16323	protein_cc	1700110N	vestigial li	Vgll3
8024	Cadm2	0.342	2.132	0.729	0.183	0.188	0.761	1.596	0.047	1.140	239857	Cadm2	chr16	-	NM_0011	P4171	protein_cc	2900078E	cell adhes	Cadm2
8025	1700010K	1.000	1.000	1.000	1.000	1.000	1.000	0.000	0.000	0.000	75501	1700010K	chr16	+	NR_04051	.	lncRNA	.	RIKEN cDN	1700010K
8026	4930428D	1.000	1.000	1.000	1.000	1.000	1.000	0.000	0.000	0.000	78110	4930428D	chr16	-	NR_13099	.	lncRNA	.	RIKEN cDN	4930428D
8027	Speer2	1.000	1.000	1.000	1.000	1.000	1.000	0.000	0.000	0.000	224318	Speer2	chr16	-	NM_1730	P18502	protein_cc	SPEER-2	spermatog	Speer2
8028	Gbe1	0.288	1.771	0.510	0.000	0.027	0.045	10.115	8.318	9.143	74185	Gbe1	chr16	+	NM_0288	P21998	protein_cc	2310045H	glucan (1,4	Gbe1
8029	D16Ertd51	1.000	1.000	1.000	1.000	1.000	1.000	0.000	0.000	0.000	52645	D16Ertd51	chr16	+	NR_04047	.	lncRNA	.	DNA segm	D16Ertd51
8030	4931420L	1.000	1.000	1.000	1.000	1.000	1.000	0.000	0.000	0.000	70972	4931420L	chr16	+	NR_04056	.	lncRNA	.	RIKEN cDN	4931420L
8031	4930567J	1.000	1.000	1.000	1.000	1.000	1.000	0.000	0.000	0.000	78221	4930567J	chr16	+	NR_04057	.	lncRNA	.	RIKEN cDN	4930567J
8032	8030451O	1.000	1.000	1.000	1.000	1.000	1.000	0.000	0.000	0.000	319533	8030451O	chr16	-	NR_13111	.	lncRNA	.	RIKEN cDN	8030451O
8033	Robo1	0.161	2.100	0.338	0.004	0.169	0.075	5.361	2.727	3.797	19876	Robo1	chr16	+	NM_0194	P16562	protein_cc	AW49463	roundabo	Robo1
8034	Robo2	0.683	1.836	1.254	0.537	0.171	0.814	3.112	2.562	3.439	268902	Robo2	chr16	-	NM_1755	P9504	protein_cc	2600013A	roundabo	Robo2
8035	Lipi	1.000	1.000	1.000	1.000	1.000	1.000	0.000	0.000	0.000	320355	Lipi	chr16	-	NM_0012	P19244	protein_cc	D930038D	lipase, me	Lipi
8036	Rbm11	2.149	0.465	1.000	0.306	1.000	0.423	0.000	1.104	0.000	224344	Rbm11	chr16	+	NM_1983	P25674	protein_cc	A330018F	RNA bindi	Rbm11
8037	Hspa13	3.184	0.641	2.042	0.005	0.039	0.051	7.969	9.640	8.999	110920	Hspa13	chr16	-	NR_02749	.	protein_cc	1600002I	heat shock	Hspa13
8038	Samsn1	15.619	0.413	6.457	0.002	0.015	0.031	4.137	8.102	6.827	67742	Samsn1	chr16	-	NM_0233	P6538	protein_cc	4930571B	SAM doma	Samsn1
8039	4930578N	1.000	1.000	1.000	1.000	1.000	1.000	0.000	0.000	0.000	78215	4930578N	chr16	+	NR_04057	.	lncRNA	.	RIKEN cDN	4930578N
8040	Nrip1	2.150	0.375	0.806	0.022	0.007	0.566	5.445	6.550	5.134	268903	Nrip1	chr16	-	NM_1734	P5794	protein_cc	6030458L	nuclear re	Nrip1
8041	1700041M	1.000	1.000	1.000	1.000	1.000	1.000	0.000	0.000	0.000	76601	1700041M	chr16	-	NR_04057	.	lncRNA	.	RIKEN cDN	1700041M
8042	Usp25	0.995	0.971	0.967	0.791	0.496	0.618	7.935	7.928	7.887	30940	Usp25	chr16	+	NM_0139	P20841	protein_cc	.	ubiquitin s	Usp25
8043	E330011O	0.204	3.586	0.731	0.012	0.002	0.444	4.727	2.432	4.275	10050361	E330011O	chr16	-	NR_04569	.	lncRNA	.	RIKEN cDN	E330011O
8044	Cxadr	1.527	1.524	2.326	0.006	0.058	0.115	4.764	5.375	5.982	13052	Cxadr	chr16	+	NM_0010	P24703	protein_cc	2610206D	coxsackie	Cxadr
8045	Btg3	17.185	1.046	17.968	0.000	0.894	0.199	4.299	8.402	8.466	12228	Btg3	chr16	-	NM_0097	P6202	protein_cc	ANA| tob5	B cell tran	Btg3
8046	Gm7334	16.972	1.065	18.071	0.000	0.852	0.199	4.299	8.384	8.474	654432	Gm7334	chr16	-	NR_00270	.	misc_RNA	EG654432	predicted	Gm7334
8047	D16Ertd47	2.946	0.593	1.747	0.000	0.001	0.016	7.238	8.797	8.043	67102	D16Ertd47	chr16	-	NM_0259	P15590	protein_cc	1700010I	DNA segm	D16Ertd47
8048	4930478L	2.746	0.181	0.498	0.047	0.021	0.033	3.146	4.603	2.141	75818	4930478L	chr16	+	NR_04056	.	lncRNA	.	RIKEN cDN	4930478L
8049	Chodl	1.000	1.000	1.000	1.000	1.000	1.000	0.000	0.000	0.000	246048	Chodl	chr16	+	NM_1391	P51	protein_cc	3110074E	chondrole	Chodl
8050	Tmprss15	1.000	1.000	1.000	1.000	1.000	1.000	0.000	0.000	0.000	19146	Tmprss15	chr16	-	NM_0089	P181	protein_cc	Entk| Prss	transmem	Tmprss15
8051	Ncam2	0.192	2.044	0.393	0.000	0.007	0.037	2.448	0.069	1.100	17968	Ncam2	chr16	+	NM_0109	P17218	protein_cc	Ncam-2| (	neural cell	Ncam2
8052	4930553E	1.000	1.000	1.000	1.000	1.000	1.000	0.000	0.000	0.000	75247	4930553E	chr16	+	NR_04056	.	lncRNA	.	RIKEN cDN	4930553E
8053	4930529L	1.000	1.000	1.000	1.000	1.000	1.000	0.000	0.000	0.000	78023	4930529L	chr16	+	NR_04053	.	lncRNA	.	RIKEN cDN	4930529L
8054	Mrpl39	0.795	0.982	0.781	0.090	0.885	0.153	8.937	8.606	8.579	27393	Mrpl39	chr16	-	NM_0174	P18902	protein_cc	C21orf8| I	mitochond	Mrpl39
8055	Jam2	3.187	1.111	3.542	0.006	0.709	0.187	6.230	7.902	8.054	67374	Jam2	chr16	+	NM_0238	P20359	protein_cc	1110002N	junction a	Jam2
8056	Atp5j	0.737	0.829	0.611	0.001	0.012	0.004	11.073	10.632	10.363	11957	Atp5j	chr16	-	NM_0167	P375	protein_cc	CF6	ATP synth	Atp5j
8057	Gabpa	2.286	0.899	2.054	0.010	0.750	0.408	6.579	7.772	7.618	14390	Gabpa	chr16	+	NM_0080	P3578	protein_cc	GABPalph	GA repeat	Gabpa
8058	App	1.085	0.898	0.975	0.154	0.106	0.849	9.109	9.227	9.072	11820	App	chr16	-	NM_0074	P11820	protein_cc	Abeta| Ab	amyloid be	App
8059	Gm10791	5.752	0.675	3.881	0.010	0.338	0.330	0.001	2.525	1.958	10003861	Gm10791	chr16	+	NR_04588	.	lncRNA	ENSMUSG	predicted	Gm10791
8060	Cyyr1	1.848	1.177	2.175	0.006	0.632	0.379	3.369	4.255	4.490	224405	Cyyr1	chr16	-	NM_1448	P25384	protein_cc	.	cysteine a	Cyyr1
8061	Adamts1	5.511	1.593	8.781	0.000	0.001	0.007	6.801	9.263	9.935	11504	Adamts1	chr16	-	NM_0096	P18276	protein_cc	ADAM-TS	a disinteg	Adamts1
8062	4930556C	1.000	1.000	1.000	1.000	1.000	1.000	0.000	0.000	0.000	75310	4930556C	chr16	+	NR_04050	.	lncRNA	.	RIKEN cDN	4930556C
8063	Adamts5	0.629	5.649	3.553	0.424	0.161	0.456	4.645	3.976	6.474	23794	Adamts5	chr16	-	NM_0117	P16578	protein_cc	9530092O	a disinteg	Adamts5
8064	N6amt1	1.029	2.103	2.163	0.704	0.005	0.080	8.107	8.148	9.220	67768	N6amt1	chr16	+	NM_0011	P23617	protein_cc	5830445C	N-6 adenii	N6amt1
8065	Ltn1	0.847	1.280	1.084	0.073	0.140	0.751	7.781	7.542	7.898	78913	Ltn1	chr16	-	NM_0010	P2448	protein_cc	4930528H	listerin E3	Ltn1
8066	Rwdd2b	2.098	1.112	2.333	0.000	0.266	0.048	5.530	6.600	6.752	53858	Rwdd2b	chr16	-	NM_0169	P19748	protein_cc	ORF5	RWD dom	Rwdd2b
8067	Usp16	1.566	0.712	1.116	0.004	0.000	0.533	8.676	9.324	8.834	74112	Usp16	chr16	+	NM_0242	P20601	protein_cc	1200004E	ubiquitin s	Usp16
8068	Cct8	0.983	0.990	0.973	0.851	0.884	0.844	10.820	10.795	10.781	12469	Cct8	chr16	-	NM_0098	P9464	protein_cc	AI132397|	chaperoni	Cct8
8069	B130034C	0.221	3.257	0.720	0.026	0.000	0.527	2.251	0.074	1.778	414095	B130034C	chr16	+	NR_04037	.	lncRNA	.	RIKEN cDN	B130034C
8070	Map3k7cl	1.010	1.885	1.904	0.000	0.182	0.423	0.000	0.015	0.929	224419	Map3k7cl	chr16	+	NM_1448	P22147	protein_cc	C21orf7| (	Map3k7 C	Map3k7cl
8071	Bach1	2.229	0.649	1.448	0.001	0.047	0.383	7.658	8.815	8.192	12013	Bach1	chr16	+	NM_0075	P10479	protein_cc	6230421P	BTB and C	Bach1
8072	Grik1	1.000	1.000	1.000	1.000	1.000	1.000	0.000	0.000	0.000	14805	Grik1	chr16	-	NM_0103	P21559	protein_cc	A830007B	glutamate	Grik1
8073	Cldn17	1.000	1.000	1.000	1.000	1.000	1.000	0.000	0.000	0.000	239931	Cldn17	chr16	-	NM_1814	P22825	protein_cc	.	claudin 17	Cldn17
8074	Cldn8	0.339	1.733	0.587	0.183	0.191	0.624	1.605	0.043	0.836	54420	Cldn8	chr16	-	NM_0187	P25170	protein_cc	AI648025	claudin 8	Cldn8
8075	Krtap24-1	1.000	1.000	1.000	1.000	1.000	1.000	0.000	0.000	0.000	239932	Krtap24-1	chr16	-	NM_0011	P23146	protein_cc	Gm312	keratin as	Krtap24-1
8076	2310079G	1.000	1.000	1.000	1.000	1.000	1.000	0.000	0.000	0.000	69699	2310079G	chr16	-	NM_0271	P19504	protein_cc	.	RIKEN cDN	2310079G
8077	Krtap26-1	1.000	1.000	1.000	1.000	1.000	1.000	0.000	0.000	0.000	69533	Krtap26-1	chr16	-	NM_0271	P3385	protein_cc	2310002B	keratin as	Krtap26-1
8078	Krtap27-1	1.000	1.000	1.000	1.000	1.000	1.000	0.000	0.000	0.000	239933	Krtap27-1	chr16	-	NM_0011	P2840	protein_cc	EG239933	keratin as	Krtap27-1
8079	2310061N	1.000	1.000	1.000	1.000	1.000	1.000	0.000	0.000	0.000	69661	2310061N	chr16	-	NM_0271	P7633	protein_cc	.	RIKEN cDN	2310061N
8080	Krtap13-1	1.000	1.000	1.000	1.000	1.000	1.000	0.000	0.000	0.000	268905	Krtap13-1	chr16	+	NM_1831	P26431	protein_cc	AA589563	keratin as	Krtap13-1
8081	Krtap13	1.000	1.000	1.000	1.000	1.000	1.000	0.000	0.000	0.000	16699	Krtap13	chr16	-	NM_0106	P23874	protein_cc	4C32	keratin as	Krtap13
8082	2310034C	1.000	1.000	1.000	1.000	1.000	1.000	0.000	0.000	0.000	117172	2310034C	chr16	+	NM_0541	P3734	protein_cc	.	RIKEN cDN	2310034C
8083	2310057N	1.000	1.000	1.000	1.000	1.000	1.000	0.000	0.000	0.000	69696	2310057N	chr16	-	NM_0271	P3647	protein_cc	Krtap13-1	RIKEN cDN	2310057N
8084	Gm20741	1.000	1.000	1.000	1.000	1.000	1.000	0.000	0.000	0.000	433047	Gm20741	chr16	-	NR_04515	.	misc_RNA	.	predicted	Gm20741
8085	Krtap14	1.000	1.000	1.000	1.000	1.000	1.000	0.000	0.000	0.000	23927	Krtap14	chr16	-	NM_0137	P17592	protein_cc	AA589630	keratin as	Krtap14
8086	Krtap15	0.716	0.995	0.712	0.182	1.000	0.423	0.490	0.008	0.000	26560	Krtap15	chr16	+	NM_0137	P18804	protein_cc	Krtap15-1	keratin as	Krtap15

8087	Krtap19-1	1.000	1.000	1.000	1.000	1.000	1.000	0.000	0.000	0.000	170657	Krtap19-1	chr16	-	NM_1308	P5918	protein_cc	Krtap16-2	keratin as	Krtap19-1
8088	Krtap19-2	1.000	1.000	1.000	1.000	1.000	1.000	0.000	0.000	0.000	170651	Krtap19-2	chr16	-	NM_1308	P1178	protein_cc	Krtap16-1	keratin as	Krtap19-2
8089	Krtap19-3	1.000	1.000	1.000	1.000	1.000	1.000	0.000	0.000	0.000	77918	Krtap19-3	chr16	-	NM_1308	P14228	protein_cc	A030006N	keratin as	Krtap19-3
8090	Krtap19-4	1.000	1.000	1.000	1.000	1.000	1.000	0.000	0.000	0.000	170654	Krtap19-4	chr16	-	NM_1308	P22693	protein_cc	Krtap16-4	keratin as	Krtap19-4
8091	Krtap19-5	1.000	1.000	1.000	1.000	1.000	1.000	0.000	0.000	0.000	16704	Krtap19-5	chr16	-	NM_0106	P19946	protein_cc	Krtap8-2	keratin as	Krtap19-5
8092	Krtap19-9	1.000	1.000	1.000	1.000	1.000	1.000	0.000	0.000	0.000	170939	Krtap19-9	chr16	-	NM_1333	P19150	protein_cc	Krtap16-1	keratin as	Krtap19-9
8093	Krtap16-3	1.000	1.000	1.000	1.000	1.000	1.000	0.000	0.000	0.000	71369	Krtap16-3	chr16	-	NM_1832	P25235	protein_cc	5430404J	keratin as	Krtap16-3
8094	Krtap22-2	1.000	1.000	1.000	1.000	1.000	1.000	0.000	0.000	0.000	68740	Krtap22-2	chr16	-	NM_0011	P13769	protein_cc	1110032D	keratin as	Krtap22-2
8095	Gm10229	1.000	1.000	1.000	1.000	1.000	1.000	0.000	0.000	0.000	10004020	Gm10229	chr16	+	NM_0011	P19576	protein_cc	ENSMUSG	predicted	Gm10229
8096	Krtap6-1	1.000	1.000	1.000	1.000	1.000	1.000	0.000	0.000	0.000	16700	Krtap6-1	chr16	+	NM_0106	P18623	protein_cc	HGTpII.1	keratin as	Krtap6-1
8097	Gm10228	1.000	1.000	1.000	1.000	1.000	1.000	0.000	0.000	0.000	10004021	Gm10228	chr16	-	NM_0012	P18664	protein_cc	ENSMUSG	predicted	Gm10228
8098	Krtap6-5	1.000	1.000	1.000	1.000	1.000	1.000	0.000	0.000	0.000	68484	Krtap6-5	chr16	-	NM_1308	P16596	protein_cc	1110002D	keratin as	Krtap6-5
8099	1110025L	1.000	1.000	1.000	1.000	1.000	1.000	0.000	0.000	0.000	68637	1110025L	chr16	-	NM_0012	P25027	protein_cc	.	RIKEN cDN	1110025L1
8100	Krtap20-2	1.000	1.000	1.000	1.000	1.000	1.000	0.000	0.000	0.000	622935	Krtap20-2	chr16	+	NM_0011	P26608	protein_cc	1190004K	keratin as	Krtap20-2
8101	Krtap21-1	1.000	1.000	1.000	1.000	1.000	1.000	0.000	0.000	0.000	170656	Krtap21-1	chr16	-	NM_0286	P12838	protein_cc	1110017B	keratin as	Krtap21-1
8102	Krtap6-2	1.000	1.000	1.000	1.000	1.000	1.000	0.000	0.000	0.000	16701	Krtap6-2	chr16	-	NM_0106	P6768	protein_cc	AI646275	keratin as	Krtap6-2
8103	Krtap8-1	1.000	1.000	1.000	1.000	1.000	1.000	0.000	0.000	0.000	16703	Krtap8-1	chr16	-	NM_0106	P4323	protein_cc	AI326176	keratin as	Krtap8-1
8104	Krtap7-1	1.000	1.000	1.000	1.000	1.000	1.000	0.000	0.000	0.000	71363	Krtap7-1	chr16	-	NM_0277	P24095	protein_cc	5430433J	keratin as	Krtap7-1
8105	Krtap11-1	1.000	1.000	1.000	1.000	1.000	1.000	0.000	0.000	0.000	16693	Krtap11-1	chr16	-	NM_0011	P8357	protein_cc	AA162070	keratin as	Krtap11-1
8106	Tiam1	0.558	0.834	0.466	0.414	0.784	0.555	3.670	2.829	2.567	21844	Tiam1	chr16	-	NM_0093	P13106	protein_cc	AI847750	T cell lymp	Tiam1
8107	Gm10789	1.000	1.000	1.000	1.000	1.000	1.000	0.000	0.000	0.000	10003870	Gm10789	chr16	+	NR_03347	.	lncRNA	ENSMUSG	predicted	Gm10789
8108	Sod1	0.584	1.230	0.719	0.000	0.000	0.009	14.762	13.987	14.286	20655	Sod1	chr16	+	NM_0114	P25874	protein_cc	B430204E	superoxid	Sod1
8109	Scaf4	0.775	0.993	0.770	0.003	0.899	0.020	7.501	7.133	7.123	224432	Scaf4	chr16	-	NM_1789	P20775	protein_cc	AA517739	SR-related	Scaf4
8110	Hunk	0.615	1.601	0.985	0.157	0.022	0.970	3.759	3.059	3.738	26559	Hunk	chr16	+	NM_0157	P14463	protein_cc	Bstk1 | Ma	hormonall	Hunk
8111	Mis18a	0.998	1.310	1.306	0.989	0.381	0.558	5.558	5.555	5.944	66578	Mis18a	chr16	-	NM_0256	P21987	protein_cc	2610039C	MIS18 kin	Mis18a
8112	Mrap	7.246	0.676	4.901	0.000	0.132	0.187	9.066	11.923	11.359	77037	Mrap	chr16	+	NM_0298	P18622	protein_cc	1110006O	melanoco	Mrap
8113	Urb1	2.949	0.404	1.192	0.076	0.109	0.754	4.072	5.632	4.325	207932	Urb1	chr16	-	NM_0294	P22738	protein_cc	4921511H	URB1 ribo	Urb1
8114	Eva1c	4.643	0.770	3.577	0.003	0.437	0.315	1.013	3.228	2.851	70967	Eva1c	chr16	+	NM_0011	P14700	protein_cc	1700092M	eva-1 hom	Eva1c
8115	1110004E	0.975	1.105	1.077	0.817	0.335	0.744	8.736	8.700	8.844	68001	1110004E	chr16	-	NM_0265	P13628	protein_cc	.	RIKEN cDN	1110004E
8116	Synj1	1.683	0.978	1.647	0.000	0.207	0.014	8.326	9.077	9.045	104015	Synj1	chr16	-	NM_0011	P14849	protein_cc	A930006D	synaptoja	Synj1
8117	4930404I	1.000	1.000	1.000	1.000	1.000	1.000	0.000	0.000	0.000	67394	4930404I	chr16	+	NR_02836	.	lncRNA		RIKEN cDN	4930404I0
8118	Paxbp1	1.629	0.485	0.790	0.057	0.009	0.500	6.136	6.840	5.796	67367	Paxbp1	chr16	-	NM_0261	P8886	protein_cc	1810007M	PAX3 and	Paxbp1
8119	4932438H	0.716	0.995	0.712	0.182	1.000	0.423	0.490	0.008	0.000	74387	4932438H	chr16	-	NM_0011	P5337	protein_cc	4931406G	RIKEN cDN	4932438H
8120	Olig2	1.000	1.000	1.000	1.000	1.000	1.000	0.000	0.000	0.000	50913	Olig2	chr16	+	NM_0169	P491	protein_cc	AI604895	oligodend	Olig2
8121	Olig1	0.282	3.654	1.031	0.005	0.118	0.964	4.397	2.572	4.441	50914	Olig1	chr16	+	NM_0169	P9967	protein_cc	AI836478	oligodend	Olig1
8122	Ifnar2	0.820	0.862	0.707	0.006	0.136	0.118	10.187	9.900	9.687	15976	Ifnar2	chr16	+	NM_0011	P16016	protein_cc	AI747302	interferon	Ifnar2
8123	Il10rb	2.601	0.601	1.564	0.002	0.019	0.250	5.992	7.371	6.637	16155	Il10rb	chr16	+	NM_0083	P24823	protein_cc	6620401D	interleuki	Il10rb
8124	A930006K	1.689	0.305	0.515	0.145	0.038	0.119	1.433	2.189	0.475	10050312	A930006K	chr16	+	NR_07721	.	lncRNA	.	RIKEN cDN	A930006K
8125	Ifnar1	0.819	1.003	0.821	0.025	0.983	0.384	7.699	7.411	7.415	15975	Ifnar1	chr16	+	NM_0105	P6860	protein_cc	CD118 | If	interferon	Ifnar1
8126	Ifngr2	1.595	1.105	1.761	0.001	0.007	0.019	7.212	7.885	8.029	15980	Ifngr2	chr16	+	NM_0083	P16480	protein_cc	Ifgr2 | Ifgt	interferon	Ifngr2
8127	Tmem50b	1.195	0.620	0.741	0.379	0.066	0.190	5.794	6.050	5.361	77975	Tmem50b	chr16	-	NM_0300	P9106	protein_cc	AU015466	transmem	Tmem50b
8128	Dnajc28	0.482	1.095	0.528	0.000	0.549	0.058	7.441	6.389	6.519	246738	Dnajc28	chr16	-	NM_1386	P18958	protein_cc	BC020175	DnaJ heat	Dnajc28
8129	Gart	0.860	1.052	0.905	0.106	0.698	0.572	7.501	7.283	7.356	14450	Gart	chr16	-	NM_0102	P10160	protein_cc	Gaps | Prg	phosphori	Gart
8130	Son	1.427	0.806	1.150	0.012	0.172	0.566	10.982	11.495	11.184	20658	Son	chr16	+	NM_0199	P5552	protein_cc	2900011L	Son DNA b	Son
8131	Donson	4.364	0.661	2.885	0.000	0.022	0.111	4.592	6.718	6.121	60364	Donson	chr16	-	NM_0217	P12219	protein_cc	1110025J	downstrea	Donson
8132	Gm10785	2.114	2.192	4.634	0.100	0.009	0.059	1.473	2.553	3.685	10003839	Gm10785	chr16	+	NR_04038	.	lncRNA	ENSMUSG	predicted	Gm10785
8133	Cryzl1	1.422	0.997	1.418	0.034	0.980	0.262	7.078	7.586	7.582	66609	Cryzl1	chr16	-	NM_1336	P26331	protein_cc	2210407J	crystallin,	Cryzl1
8134	Itsn1	1.422	0.423	0.601	0.009	0.000	0.158	8.260	8.768	7.525	16443	Itsn1	chr16	+	NM_0105	P11901	protein_cc	AA517634	intersecti	Itsn1
8135	Atp5o	0.638	1.306	0.833	0.003	0.023	0.178	11.732	11.082	11.467	28080	Atp5o	chr16	-	NM_1385	P9691	protein_cc	ATPO | D1	ATP synth	Atp5o
8136	Slc5a3	0.543	0.785	0.426	0.054	0.377	0.218	5.874	4.992	4.643	53881	Slc5a3	chr16	+	NM_0173	P25006	protein_cc	AA623876	solute car	Slc5a3
8137	Mrps6	1.331	0.717	0.954	0.211	0.128	0.895	8.010	8.423	7.942	121022	Mrps6	chr16	+	NM_0804	P10635	protein_cc	AW04632	mitochon	Mrps6
8138	Kcne2	1.010	1.885	1.904	0.000	0.182	0.423	0.000	0.015	0.929	246133	Kcne2	chr16	+	NM_1341	P24989	protein_cc	2200002I1	potassium	Kcne2
8139	Smim11	1.126	0.953	1.073	0.308	0.333	0.736	8.371	8.543	8.473	68936	Smim11	chr16	+	NM_1387	P11731	protein_cc	1190017O	small inte	Smim11
8140	1700048M	1.000	1.000	1.000	1.000	1.000	1.000	0.000	0.000	0.000	10050316	1700048M	chr16	-	NR_04530	.	lncRNA	Gm15980	RIKEN cDN	1700048M
8141	4930563D	1.000	1.000	1.000	1.000	1.000	1.000	0.000	0.000	0.000	75328	4930563D	chr16	-	NM_0292	P4826	protein_cc	.	RIKEN cDN	4930563D
8142	Kcne1	1.000	1.000	1.000	1.000	1.000	1.000	0.000	0.000	0.000	16509	Kcne1	chr16	-	NM_0084	P14084	protein_cc	Isk | MinK	potassium	Kcne1
8143	Gm29805	1.000	1.000	1.000	1.000	1.000	1.000	0.000	0.000	0.000	10105623	Gm29805	chr16	-	NR_10505	.	lncRNA	.	predicted	Gm29805
8144	Rcan1	2.530	0.965	2.441	0.000	0.794	0.112	7.182	8.521	8.469	54720	Rcan1	chr16	-	NM_0194	P12348	protein_cc	Adapt78 |	regulator	Rcan1
8145	2410124H	0.567	1.851	1.050	0.186	0.185	0.947	0.845	0.027	0.916	76785	2410124H	chr16	+	NM_0297	P12051	protein_cc	.	RIKEN cDN	2410124H
8146	Clic6	0.313	3.504	1.096	0.089	0.184	0.928	1.739	0.062	1.871	209195	Clic6	chr16	+	NM_1724	P4053	protein_cc	5730466J1	chloride ir	Clic6
8147	Runx1	13.727	0.514	7.053	0.000	0.000	0.028	3.019	6.798	5.837	12394	Runx1	chr16	-	NM_0011	P2337	protein_cc	AML1 | CB	runt relate	Runx1
8148	1810053B	0.039	5.753	0.222	0.003	0.173	0.124	6.548	1.853	4.378	69857	1810053B	chr16	-	NR_04048	.	lncRNA	1810008L	RIKEN cDN	1810053B
8149	1700029J	1.000	1.000	1.000	1.000	1.000	1.000	0.000	0.000	0.000	72264	1700029J	chr16	-	NR_04049	.	lncRNA		RIKEN cDN	1700029J0
8150	Setd4	0.167	0.732	0.122	0.019	0.604	0.125	5.757	3.175	2.725	224440	Setd4	chr16	-	NM_1454	P20619	protein_cc	AY037804	SET domai	Setd4
8151	Cbr1	0.182	1.685	0.307	0.000	0.026	0.028	11.360	8.901	9.654	12408	Cbr1	chr16	+	NM_0076	P5430	protein_cc	AW26179	carbonyl r	Cbr1
8152	Cbr3	0.975	1.873	1.827	0.926	0.124	0.348	2.984	2.949	3.854	109857	Cbr3	chr16	+	NM_1730	P16064	protein_cc	1110001J	carbonyl r	Cbr3
8153	Dopey2	1.353	0.653	0.883	0.001	0.101	0.774	7.248	7.684	7.069	70028	Dopey2	chr16	+	NM_0272	P9933	protein_cc	0610038M	dopey fam	Dopey2
8154	Morc3	2.556	0.643	1.644	0.000	0.003	0.170	5.997	7.351	6.714	338467	Morc3	chr16	+	NM_0010	P23008	protein_cc	1110051N	microrchi	Morc3
8155	Chaf1b	1.763	1.779	3.137	0.215	0.156	0.201	2.954	3.773	4.604	110749	Chaf1b	chr16	+	NM_0280	P23072	protein_cc	2600017H	chromatin	Chaf1b
8156	Cldn14	16.166	0.337	5.454	0.000	0.000	0.046	7.649	11.664	10.096	56173	Cldn14	chr16	-	NM_0011	P6052	protein_cc	AI851731	claudin 14	Cldn14
8157	Sim2	0.552	0.990	0.547	0.182	1.000	0.423	0.871	0.015	0.000	20465	Sim2	chr16	+	NM_0113	P1256	protein_cc	bHLHe15	single-mir	Sim2
8158	Hlcs	0.305	2.049	0.626	0.007	0.138	0.382	6.145	4.434	5.469	110948	Hlcs	chr16	-	NM_1391	P23250	protein_cc	410I21.SP	holocarbo	Hlcs
8159	Ripply3	0.443	2.301	1.019	0.030	0.019	0.955	5.436	4.262	5.464	170765	Ripply3	chr16	+	NM_1332	P26289	protein_cc	Dscr6	ripply tran	Ripply3
8160	Pigp	0.472	1.708	0.805	0.032	0.041	0.566	6.980	5.896	6.668	56176	Pigp	chr16	-	NM_0011	P2194	protein_cc	Dcrc | Dscr	phosphati	Pigp
8161	Ttc3	0.561	1.139	0.639	0.000	0.601	0.246	8.803	7.969	8.157	22129	Ttc3	chr16	+	NM_0094	P1612	protein_cc	2610202A	tetratrico	Ttc3
8162	Dscr3	1.433	1.186	1.699	0.123	0.311	0.040	6.760	7.278	7.525	13185	Dscr3	chr16	-	NM_0078	P19209	protein_cc	AW53812	Down syn	Dscr3
8163	Dyrk1a	1.082	0.874	0.945	0.294	0.284	0.814	7.975	8.088	7.894	13548	Dyrk1a	chr16	+	NM_0078	P14577	protein_cc	2310043O	dual-speci	Dyrk1a
8164	Kcnj6	1.000	1.000	1.000	1.000	1.000	1.000	0.000	0.000	0.000	16522	Kcnj6	chr16	-	NM_0010	P5743	protein_cc	BIR1 | GIRI	potassium	Kcnj6
8165	Kcnj15	0.331	2.683	0.889	0.018	0.118	0.810	2.791	1.197	2.621	16516	Kcnj15	chr16	+	NM_0010	P10761	protein_cc	4930414N	potassium	Kcnj15
8166	Erg	0.783	1.169	0.916	0.551	0.768	0.885	5.309	4.957	5.183	13876	Erg	chr16	-	NM_0013	P8274	protein_cc	D030036I	avian eryt	Erg
8167	Ets2	7.514	0.668	5.022	0.000	0.161	0.202	8.182	11.092	10.510	23872	Ets2	chr16	+	NM_0118	P22447	protein_cc	AU022856	E26 avian	Ets2
8168	1600002D	1.005	1.439	1.446	0.000	0.182	0.423	0.000	0.008	0.532	69776	1600002D	chr16	-	NR_04048	.	lncRNA		RIKEN cDN	1600002D
8169	Psmg1	0.707	1.278	0.904	0.007	0.273	0.754	7.565	7.065	7.418	56088	Psmg1	chr16	-	NM_0195	P4167	protein_cc	AW55210	proteason	Psmg1
8170	Brwd1	1.176	0.620	0.729	0.061	0.003	0.000	8.451	8.685	7.996	93871	Brwd1	chr16	-	NM_1451	P18910	protein_cc	5330419I	bromodor	Brwd1
8171	Hmgn1	0.703	2.016	1.418	0.031	0.000	0.061	10.161	9.654	10.665	15312	Hmgn1	chr16	-	NM_0082	P1213	protein_cc	HMG-14 |	high mobi	Hmgn1
8172	Wrb	1.179	1.276	1.505	0.468	0.225	0.201	5.347	5.585	5.936	71446	Wrb	chr16	+	NM_2073	P19785	protein_cc	5530402J	tryptopha	Wrb
8173	Lca5l	0.722	0.626	0.452	0.528	1.000	0.422	1.145	0.675	0.000	385668	Lca5l	chr16	-	NM_0010	P512	protein_cc	4921526F	Leber con	Lca5l
8174	Sh3bgr	1.118	0.304	0.340	0.899	0.355	0.477	2.027	2.188	0.470	50795	Sh3bgr	chr16	+	NM_0158	P8398	protein_cc	5430437A	SH3-bindi	Sh3bgr
8175	B3galt5	0.462	1.764	0.815	0.184	0.188	0.805	1.147	0.033	0.851	93961	B3galt5	chr16	+	NM_0011	P22163	protein_cc	1190002B	UDP-Gal:b	B3galt5
8176	Igsf5	0.479	2.170	1.039	0.002	0.001	0.799	7.663	6.600	7.718	72058	Igsf5	chr16	+	NM_0011	P9538	protein_cc	2010003D	immunogl	Igsf5
8177	Itgb2l	2.515	0.762	1.917	0.207	0.628	0.423	1.347	2.678	2.286	16415	Itgb2l	chr16	-	NM_0084	P9574	protein_cc	5033406G	integrin b	Itgb2l

8178	Pcp4	1.036	4.591	4.757	0.000	0.108	0.329	0.001	0.052	2.251	18546	Pcp4	chr16	+	NM_0087!	P8085	protein_cc	P16Rimb1	Purkinje c	Pcp4
8179	Dscam	0.451	0.986	0.444	0.182	1.000	0.423	1.170	0.021	0.000	13508	Dscam	chr16	-	NM_0311	P13647	protein_cc	4932410A	Down syn	Dscam
8180	Bace2	0.841	0.755	0.635	0.167	0.104	0.016	6.349	6.098	5.694	56175	Bace2	chr16	+	NM_0195	P10432	protein_cc	1110059C	beta-site	Bace2
8181	Mx1	26.581	0.463	12.316	0.000	0.077	0.369	3.943	8.675	7.565	17857	Mx1	chr16	-	NR_00352	.	misc_RNA	AI893580 |	MX dynan	Mx1
8182	Fam3b	1.005	1.439	1.446	0.000	0.182	0.423	0.000	0.008	0.532	52793	Fam3b	chr16	-	NM_0206	P23930	protein_cc	2-21| 903	family wit	Fam3b
8183	Mx2	12.716	0.385	4.895	0.000	0.009	0.280	4.259	7.928	6.551	17858	Mx2	chr16	+	NR_00350	.	misc_RNA	AI528743 |	MX dynan	Mx2
8184	Tmprss2	1.269	0.759	0.964	0.027	0.140	0.894	5.795	6.138	5.741	50528	Tmprss2	chr16	-	NM_0157	P5139	protein_cc	D16Ertd61	transmem	Tmprss2
8185	Ripk4	2.574	1.122	2.888	0.014	0.746	0.329	4.427	5.791	5.957	72388	Ripk4	chr16	-	NM_0236	P18069	protein_cc	2310069J1	receptor-i	Ripk4
8186	Prdm15	3.515	0.493	1.732	0.003	0.034	0.474	3.447	5.261	4.239	114604	Prdm15	chr16	-	NM_1447	P9432	protein_cc	C21orf83 |	PR domai	Prdm15
8187	C2cd2	0.125	1.268	0.159	0.000	0.207	0.003	10.478	7.481	7.823	207781	C2cd2	chr16	-	NM_1748	P16020	protein_cc	5730563M	C2 calcium	C2cd2
8188	Zbtb21	3.146	0.523	1.646	0.002	0.006	0.086	5.541	7.195	6.260	114565	Zbtb21	chr16	-	NM_0010	P22192	protein_cc	5430437K	zinc finger	Zbtb21
8189	B230307C	2.471	0.404	0.998	0.004	0.001	0.996	4.166	5.471	4.163	245305	B230307C	chr16	+	NR_13096	.	lncRNA	EG245305	RIKEN cDN	B230307C
8190	A630089N	1.403	0.357	0.500	0.146	0.015	0.083	3.510	3.999	2.511	320586	A630089N	chr16	-	NR_01549	.	lncRNA		RIKEN cDN	A630089N
8191	Pisd-ps2	0.827	0.479	0.396	0.018	0.001	0.024	5.089	4.816	3.753	328734	Pisd-ps2	chr17	-	NR_00351	.	misc_RNA	D030013I	phosphati	Pisd-ps2
8192	Scaf8	1.632	0.842	1.374	0.016	0.309	0.316	6.572	7.279	7.031	106583	Scaf8	chr17	+	NM_1341	P6138	protein_cc	A630086M	SR-related	Scaf8
8193	Tiam2	2.009	0.809	1.626	0.003	0.077	0.032	5.856	6.862	6.557	24001	Tiam2	chr17	+	NM_0011	P1181	protein_cc	3000002F	T cell lymp	Tiam2
8194	Tfb1m	2.145	0.686	1.470	0.006	0.034	0.023	6.370	7.471	6.926	224481	Tfb1m	chr17	-	NM_1460	P8021	protein_cc	AI429207	transcripti	Tfb1m
8195	Cldn20	2.134	0.469	1.000	0.305	1.000	0.423	0.000	1.094	0.000	621628	Cldn20	chr17	+	NM_0011	P15645	protein_cc	EG621628	claudin 20	Cldn20
8196	1700102H	0.835	2.738	2.286	0.824	0.075	0.148	3.070	2.810	4.263	68230	1700102H	chr17	+	NR_04530	.	lncRNA	.	RIKEN cDN	1700102H
8197	Nox3	1.000	1.000	1.000	1.000	1.000	1.000	0.000	0.000	0.000	224480	Nox3	chr17	-	NM_1989	P17234	protein_cc	GP91-3| h	NADPH ox	Nox3
8198	4930470H	0.669	0.995	0.666	0.181	1.000	0.423	0.587	0.008	0.000	75815	4930470H	chr17	-	NR_04576	.	lncRNA		RIKEN cDN	4930470H
8199	4930548J	1.000	1.000	1.000	1.000	1.000	1.000	0.000	0.000	0.000	78043	4930548J	chr17	-	NR_04546	.	lncRNA	1700113K	RIKEN cDN	4930548J0
8200	4930517M	1.000	1.000	1.000	1.000	1.000	1.000	0.000	0.000	0.000	75117	4930517M	chr17	+	NR_13098	.	lncRNA		RIKEN cDN	4930517M
8201	Arid1b	0.950	0.984	0.935	0.626	0.847	0.633	8.620	8.546	8.522	239985	Arid1b	chr17	+	NM_0010	P23579	protein_cc	8030481M	AT rich int	Arid1b
8202	Tmem242	0.753	1.217	0.916	0.129	0.157	0.665	9.668	9.258	9.541	70544	Tmem242	chr17	-	NM_0274	P1754	protein_cc	1110008A	transmem	Tmem242
8203	Ldhal6b	1.146	0.632	0.725	0.729	1.000	0.422	0.465	0.661	0.000	106557	Ldhal6b	chr17	-	NM_1753	P4316	protein_cc	4933402O	lactate de	Ldhal6b
8204	Gm8363	1.000	1.000	1.000	1.000	1.000	1.000	0.000	0.000	0.000	666919	Gm8363	chr17	-	NR_07337	.	misc_RNA	EG666919	predicted	Gm8363
8205	Zdhhc14	0.300	1.967	0.590	0.012	0.002	0.255	5.171	3.434	4.409	224454	Zdhhc14	chr17	+	NM_1460	P5003	protein_cc	B530001K	zinc finger	Zdhhc14
8206	3300005D	0.726	1.425	1.034	0.187	0.186	0.943	0.476	0.015	0.525	78512	3300005D	chr17	+	NR_04508	.	lncRNA	.	RIKEN cDN	3300005D
8207	Snx9	0.589	0.992	0.584	0.246	0.973	0.459	4.774	4.009	3.997	66616	Snx9	chr17	+	NM_0256	P14017	protein_cc	2700073N	sorting ne	Snx9
8208	Synj2	2.079	0.876	1.821	0.004	0.704	0.504	3.593	4.648	4.457	20975	Synj2	chr17	+	NM_0011	P2933	protein_cc		synaptoja	Synj2
8209	Serac1	0.584	0.666	0.389	0.020	0.027	0.094	4.674	3.899	3.313	321007	Serac1	chr17	-	NM_1773	P5356	protein_cc	4930511N	serine acti	Serac1
8210	Gtf2h5	1.007	1.303	1.313	0.922	0.012	0.003	9.705	9.716	10.098	66467	Gtf2h5	chr17	+	NM_1813	P185	protein_cc	2700017P	general tr	Gtf2h5
8211	Tulp4	0.950	0.567	0.539	0.538	0.005	0.019	5.938	5.865	5.046	68842	Tulp4	chr17	+	NM_0011	P21039	protein_cc	1110057P	tubby like	Tulp4
8212	Tmem181	1.612	1.521	2.451	0.176	0.096	0.128	5.024	5.713	6.317	77106	Tmem181	chr17	+	NM_0010	P19589	protein_cc	5930418K	transmem	Tmem181
8213	Dynlt1a	1.513	1.358	2.055	0.003	0.005	0.011	6.987	7.584	8.026	10031087	Dynlt1a	chr17	-	NM_0011	P26463	protein_cc	.	dynein ligl	Dynlt1a
8214	Dynlt1b	1.546	1.459	2.255	0.000	0.011	0.038	6.735	7.363	7.908	21648	Dynlt1b	chr17	+	NM_0093	P22128	protein_cc	AGS2| Dy	dynein ligl	Dynlt1b
8215	Tmem181	4.153	1.140	4.734	0.016	0.471	0.016	3.043	5.097	5.286	547127	Tmem181	chr17	-	NR_03352	.	misc_RNA	547127| T	transmem	Tmem181
8216	Dynlt1f	1.889	1.304	2.462	0.000	0.001	0.004	6.185	7.103	7.485	10004053	Dynlt1f	chr17	+	NM_0011	P15874	protein_cc	10004053	dynein ligl	Dynlt1f
8217	Dynlt1c	1.832	1.302	2.384	0.000	0.002	0.012	7.295	8.168	8.549	10004056	Dynlt1c	chr17	+	NM_0011	P15874	protein_cc	10004056	dynein ligl	Dynlt1c
8218	Tmem181	1.105	1.400	1.547	0.760	0.217	0.183	3.194	3.339	3.824	10004052	Tmem181	chr17	-	NR_02830	.	misc_RNA	10004052	transmem	Tmem181
8219	Sytl3	2.785	0.988	2.751	0.019	0.979	0.423	0.001	1.478	1.461	83672	Sytl3	chr17	+	NM_0313	P9812	protein_cc	A430092N	synaptota	Sytl3
8220	Ezr	8.254	0.522	4.307	0.000	0.000	0.014	6.415	9.460	8.522	22350	Ezr	chr17	-	NM_0095	P857	protein_cc	AW14636	ezrin	Ezr
8221	Rsph3b	0.733	1.197	0.877	0.393	0.635	0.432	6.982	6.533	6.793	10003728	Rsph3b	chr17	-	NM_0010	P23721	protein_cc	Rshl2b	radial spo	Rsph3b
8222	Tagap1	1.321	0.758	1.001	0.023	0.160	0.998	5.984	6.386	5.985	380608	Tagap1	chr17	-	NM_1471	P2771	protein_cc	2610315E	T cell activ	Tagap1
8223	Rnaset2b	0.703	0.930	0.653	0.003	0.471	0.053	11.529	11.020	10.915	68195	Rnaset2b	chr17	+	NM_0266	P2247	protein_cc	0610007O	ribonuclea	Rnaset2b
8224	Gm1604b	1.580	0.633	1.000	0.305	1.000	0.423	0.000	0.660	0.000	381059	Gm1604b	chr17	+	NM_0010	P3177	protein_cc	ENSMUSG	predicted	Gm1604b
8225	Rps6ka2	1.571	1.267	1.990	0.028	0.126	0.100	3.854	4.506	4.847	20112	Rps6ka2	chr17	+	NM_0112	P7250	protein_cc	90kDa| D	ribosomal	Rps6ka2
8226	Tcp10a	5.705	0.582	3.319	0.306	0.586	0.079	0.001	2.514	1.732	21461	Tcp10a	chr17	+	NM_0115	P5403	protein_cc	D17Leh66	t-complex	Tcp10a
8227	Ttll2	0.382	0.982	0.375	0.182	1.000	0.423	1.416	0.027	0.000	10021647	Ttll2	chr17	-	NM_0010	P18529	protein_cc	EG625850	tubulin ty	Ttll2
8228	Gm9992	0.156	0.954	0.149	0.000	1.000	0.014	2.746	0.068	0.000	667055	Gm9992	chr17	-	NM_0011	P7174	protein_cc	ENSMUSG	predicted	Gm9992
8229	Fndc1	0.243	3.635	0.883	0.117	0.017	0.878	2.115	0.073	1.935	68655	Fndc1	chr17	-	NM_0010	P10570	protein_cc	1110027O	fibronecti	Fndc1
8230	Tagap	5.766	0.404	2.328	0.000	0.002	0.236	4.023	6.551	5.242	72536	Tagap	chr17	+	NM_1459	P20747	protein_cc	2610315E	T cell activ	Tagap
8231	Rsph3a	0.782	1.096	0.857	0.323	0.779	0.641	6.978	6.622	6.755	66832	Rsph3a	chr17	-	NM_0257	P26833	protein_cc	1700012G	radial spo	Rsph3a
8232	Rnaset2a	0.599	1.046	0.626	0.001	0.722	0.060	10.645	9.905	9.969	10003728	Rnaset2a	chr17	-	NM_0010	P14147	protein_cc	0610007O	ribonuclea	Rnaset2a
8233	Fgfr1op	0.904	1.685	1.523	0.261	0.001	0.069	6.169	6.023	6.775	75296	Fgfr1op	chr17	+	NM_0011	P21732	protein_cc	49305530	Fgfr1 onco	Fgfr1op
8234	Ccr6	1.000	1.000	1.000	1.000	1.000	1.000	0.000	0.000	0.000	12458	Ccr6	chr17	+	NM_0011	P4560	protein_cc	CC-CKR-6|	chemokin	Ccr6
8235	Mpc1	0.425	1.107	0.470	0.002	0.126	0.047	10.105	8.870	9.017	55951	Mpc1	chr17	+	NM_0188	P22572	protein_cc	0610006G	mitochono	Mpc1
8236	4930506C	0.598	0.961	0.575	0.051	0.911	0.191	3.441	2.700	2.642	75060	4930506C	chr17	-	NR_07337	.	lncRNA		RIKEN cDN	4930506C
8237	Sft2d1	0.656	0.847	0.555	0.310	0.744	0.069	7.198	6.590	6.350	106489	Sft2d1	chr17	+	NM_1341	P14312	protein_cc	5630401J1	SFT2 dom	Sft2d1
8238	Prr18	0.356	1.010	0.360	0.166	0.959	0.407	3.015	1.525	1.539	320111	Prr18	chr17	+	NR_02828	.	protein_cc	9630019K	proline ric	Prr18
8239	T2	1.000	1.000	1.000	1.000	1.000	1.000	0.000	0.000	0.000	21331	T2	chr17	+	NM_0011	P18315	protein_cc	brachyury	brachyury	T2
8240	Gm16702	2.747	1.094	3.005	0.002	0.037	0.042	0.746	2.204	2.333	10050460	Gm16702	chr17	-	NR_04579	.	lncRNA	AU01660	predicted	Gm16702
8241	T	1.000	1.000	1.000	1.000	1.000	1.000	0.000	0.000	0.000	20997	T	chr17	+	NM_0093	P3944	protein_cc	Bra| cou|	brachyury	T
8242	Pde10a	8.664	0.916	7.938	0.051	0.853	0.223	0.589	3.704	3.578	23984	Pde10a	chr17	+	NM_0012	P14093	protein_cc	.	phosphod	Pde10a
8243	1700010I1	2.566	0.442	1.135	0.291	0.295	0.882	2.328	3.687	2.510	66931	1700010I1	chr17	+	NM_0258	P2834	protein_cc	.	RIKEN cDN	1700010I1
8244	6530411M	1.000	1.000	1.000	1.000	1.000	1.000	0.000	0.000	0.000	67791	6530411M	chr17	-	NR_02788	.	lncRNA		RIKEN cDN	6530411M
8245	Pabpc6	1.000	1.000	1.000	1.000	1.000	1.000	0.000	0.000	0.000	67543	Pabpc6	chr17	-	NM_0011	P21012	protein_cc	4932702K	poly(A) bi	Pabpc6
8246	4930452A	1.000	1.000	1.000	1.000	1.000	1.000	0.000	0.000	0.000	71092	4930452A	chr17	-	NR_04543	.	lncRNA	4933424P	RIKEN cDN	4930452A
8247	Qk	0.824	1.201	0.990	0.003	0.000	0.816	9.363	9.084	9.348	19317	Qk	chr17	-	NM_0011	P8044	protein_cc	1110003F	quaking	Qk
8248	Cahm	2.202	0.641	1.412	0.019	0.001	0.524	1.056	2.195	1.553	10052679	Cahm	chr17	+	NR_03758	.	lncRNA	B930003M	colon ade	Cahm
8249	1700110C	0.669	0.995	0.666	0.181	1.000	0.423	0.587	0.008	0.000	76634	1700110C	chr17	-	NR_04546	.	lncRNA		RIKEN cDN	1700110C
8250	Pacrg	0.959	2.847	2.730	0.923	0.260	0.512	0.784	0.723	2.232	69310	Pacrg	chr17	-	NM_0270	P6800	protein_cc	1700008H	PARK2 co-	Pacrg
8251	A230009B	0.716	0.995	0.712	0.182	1.000	0.423	0.490	0.008	0.000	319750	A230009B	chr17	+	NR_07723	.	lncRNA	.	RIKEN cDN	A230009B
8252	Park2	0.323	1.761	0.569	0.048	0.151	0.418	3.867	2.238	3.054	50873	Park2	chr17	+	NM_0166	P20470	protein_cc	PRKN	Parkinson	Park2
8253	D17Ertd64	1.000	1.000	1.000	1.000	1.000	1.000	0.000	0.000	0.000	52027	D17Ertd64	chr17	-	NR_04580	.	lncRNA		DNA segm	D17Ertd64
8254	Agpat4	3.250	0.544	1.768	0.000	0.000	0.069	4.292	5.993	5.114	68262	Agpat4	chr17	+	NM_0266	P24792	protein_cc	1500003P	1-acylglyc	Agpat4
8255	Map3k4	0.689	0.687	0.473	0.035	0.016	0.062	7.174	6.637	6.094	26407	Map3k4	chr17	-	NM_0119	P20051	protein_cc	D17Rp17|	mitogen-a	Map3k4
8256	4732491K	1.832	1.841	3.373	0.036	0.056	0.107	2.459	3.333	4.213	224523	4732491K	chr17	+	NR_04529	.	lncRNA	.	RIKEN cDN	4732491K
8257	Plg	0.569	0.845	0.481	0.036	0.434	0.085	14.774	13.960	13.717	18815	Plg	chr17	+	NM_0088	P6064	protein_cc	AI649309 |	plasminog	Plg
8258	Slc22a3	1.390	0.597	0.830	0.017	0.021	0.558	5.920	6.396	5.651	20519	Slc22a3	chr17	-	NM_0113	P22209	protein_cc	EMT| Oct	solute car	Slc22a3
8259	C030013G	1.000	1.000	1.000	1.000	1.000	1.000	0.000	0.000	0.000	77414	C030013G	chr17	+	NR_07721	.	lncRNA		RIKEN cDN	C030013G
8260	Slc22a2	1.000	1.000	1.000	1.000	1.000	1.000	0.000	0.000	0.000	20518	Slc22a2	chr17	+	NM_0136	P10837	protein_cc	Oct2| Orc	solute car	Slc22a2
8261	Slc22a1	0.290	1.079	0.313	0.000	0.061	0.001	11.277	9.492	9.602	20517	Slc22a1	chr17	-	NM_0092	P17105	protein_cc	Lx1| Oct1	solute car	Slc22a1
8262	Igf2r	0.339	0.982	0.333	0.002	0.925	0.044	8.601	7.042	7.016	16004	Igf2r	chr17	-	NM_0105	P998	protein_cc	AI661837 |	insulin-like	Igf2r
8263	Airn	0.141	2.743	0.388	0.000	0.057	0.066	2.912	0.089	1.545	104103	Airn	chr17	+	NR_02778	.	lncRNA	2810051F	antisense	Airn
8264	Mas1	1.000	1.000	1.000	1.000	1.000	1.000	0.000	0.000	0.000	17171	Mas1	chr17	-	NM_0085	P12867	protein_cc	Mas-1| M	MAS1 onc	Mas1
8265	Mrgprh	1.000	1.000	1.000	1.000	1.000	1.000	0.000	0.000	0.000	80978	Mrgprh	chr17	+	NM_0307	P26671	protein_cc	Gpr90| M	MAS-relat	Mrgprh
8266	Pnldc1	0.642	0.622	0.399	0.442	1.000	0.423	1.325	0.685	0.000	240023	Pnldc1	chr17	-	NM_0010	P17259	protein_cc	Gm313	poly(A)-sp	Pnldc1
8267	Mrpl18	0.802	0.864	0.693	0.132	0.462	0.244	9.958	9.640	9.429	67681	Mrpl18	chr17	-	NM_0263	P8890	protein_cc	1010001C	mitochond	Mrpl18
8268	Tcp1	0.619	0.703	0.435	0.000	0.028	0.026	11.282	10.590	10.081	21454	Tcp1	chr17	+	NM_0136	P18333	protein_cc	AI528772 |	t-complex	Tcp1

8269	Snora20	2.473	1.725	4.265	0.001	0.494	0.489	3.162	4.468	5.255	10030374	Snora20	chr17	+	NR_02847	.	snoRNA	MBI-125	small nucl	Snora20
8270	Acat3	0.627	0.704	0.441	0.000	0.007	0.015	11.537	10.863	10.356	224530	Acat3	chr17	-	NM_1531	P5628	protein_cc	ACTL	acetyl-Coe	Acat3
8271	Acat2	0.678	1.340	0.909	0.000	0.040	0.542	10.303	9.743	10.166	110460	Acat2	chr17	-	NM_0093	P22456	protein_cc	AW74279	acetyl-Coe	Acat2
8272	Wtap	3.567	0.584	2.083	0.001	0.030	0.170	7.952	9.787	9.011	60532	Wtap	chr17	-	NM_0011	P6846	protein_cc	2810408K	Wilms tun	Wtap
8273	Sod2	4.557	0.895	4.079	0.000	0.208	0.028	11.471	13.659	13.499	20656	Sod2	chr17	+	NM_0136	P2239	protein_cc	MnSOD| S	superoxid	Sod2
8274	Gpr31b	1.000	1.000	1.000	1.000	1.000	1.000	0.000	0.000	0.000	436440	Gpr31b	chr17	-	NM_0010	P20094	protein_cc	12-HETER	G protein-	Gpr31b
8275	Tcp10b	3.837	0.724	2.779	0.233	0.627	0.038	0.589	2.528	2.063	21462	Tcp10b	chr17	+	NM_0093	P4598	protein_cc	D17Leh66	t-complex	Tcp10b
8276	Gm11166	1.000	1.000	1.000	1.000	1.000	1.000	0.000	0.000	0.000	625850	Gm11166	chr17	-	NR_02455	.	lncRNA	ENSMUSG	predicted	Gm11166
8277	Unc93a	0.082	4.539	0.396	0.000	0.000	0.046	3.642	0.122	2.304	381058	Unc93a	chr17	-	NM_1992	P15262	protein_cc	unc-93A|	unc-93 ho	Unc93a
8278	Gm10512	1.000	1.000	1.000	1.000	1.000	1.000	0.000	0.000	0.000	10003839	Gm10512	chr17	+	NR_03345	.	lncRNA	ENSMUSG	predicted	Gm10512
8279	Smok2a	1.000	1.000	1.000	1.000	1.000	1.000	0.000	0.000	0.000	27263	Smok2a	chr17	+	NM_0137	P19452	protein_cc	Smok2	sperm mo	Smok2a
8280	Smok2b	1.000	1.000	1.000	1.000	1.000	1.000	0.000	0.000	0.000	236574	Smok2b	chr17	+	NM_0011	P9413	protein_cc	Gm1847|	sperm mo	Smok2b
8281	Tcp10c	3.957	0.576	2.280	0.199	0.436	0.116	0.381	2.366	1.570	10004135	Tcp10c	chr17	+	NM_0011	P325	protein_cc	D17Leh66	t-complex	Tcp10c
8282	2700054A	1.905	0.503	0.958	0.440	0.391	0.955	3.617	4.546	3.555	72578	2700054A	chr17	-	NR_04543	.	lncRNA	.	RIKEN cDN	2700054A
8283	Smok4a	1.000	1.000	1.000	1.000	1.000	1.000	0.000	0.000	0.000	272667	Smok4a	chr17	+	NR_03076	.	misc_RNA	4930513D	sperm mo	Smok4a
8284	Tcte2	2.630	0.438	1.152	0.015	0.012	0.696	0.588	1.983	0.792	21646	Tcte2	chr17	-	NM_0223	P20474	protein_cc	AI429268|	t-complex	Tcte2
8285	Mllt4	1.298	1.146	1.488	0.013	0.003	0.043	9.034	9.410	9.607	17356	Mllt4	chr17	+	NM_0108	P13390	protein_cc	5033403D	myeloid/ly	Mllt4
8286	Gm7168	1.000	1.000	1.000	1.000	1.000	1.000	0.000	0.000	0.000	635895	Gm7168	chr17	+	NM_0011	P8664	protein_cc	EG635895	predicted	Gm7168
8287	Dact2	0.197	1.740	0.343	0.008	0.131	0.147	7.653	5.311	6.110	240025	Dact2	chr17	-	NM_1728	P1817	protein_cc	2900084N	dishevelle	Dact2
8288	Smoc2	0.442	1.609	0.711	0.038	0.309	0.463	6.023	4.845	5.531	64074	Smoc2	chr17	+	NM_0223	P17570	protein_cc	1700056C	SPARC rel	Smoc2
8289	4930474M	1.000	1.000	1.000	1.000	1.000	1.000	0.000	0.000	0.000	74917	4930474M	chr17	-	NR_02798	.	lncRNA		RIKEN cDN	4930474M
8290	Thbs2	0.362	1.743	0.630	0.326	0.275	0.717	2.995	1.528	2.330	21826	Thbs2	chr17	-	NM_0115	P5044	protein_cc	Thbs-2| TS	thrombos	Thbs2
8291	Wdr27	0.504	0.990	0.499	0.181	1.000	0.423	1.002	0.015	0.000	71682	Wdr27	chr17	-	NM_1751	P23534	protein_cc	0610012K	WD repeat	Wdr27
8292	1600012H	1.157	1.008	1.166	0.157	0.947	0.583	9.879	10.089	10.100	67912	1600012H	chr17	+	NM_0010	P12293	protein_cc	.	RIKEN cDN	1600012H
8293	Phf10	1.255	1.222	1.533	0.133	0.048	0.191	7.326	7.654	7.943	72057	Phf10	chr17	-	NM_0242	P26791	protein_cc	1810055P	PHD finge	Phf10
8294	LOC10674	5.757	0.623	3.585	0.001	0.098	0.227	1.471	3.997	3.314	106740	LOC10674	chr17	-	NR_02790	.	lncRNA		uncharact	LOC10674
8295	Gm3417	2.845	2.820	8.023	0.297	0.028	0.000	0.001	1.510	3.006	10004158	Gm3417	chr17	-	NM_0011	P23288	protein_cc	10004158	predicted	Gm3417
8296	Gm3448	1.246	3.672	4.576	0.813	0.026	0.079	2.095	2.413	4.289	10004163	Gm3448	chr17	-	NM_0011	P23288	protein_cc	10004163	predicted	Gm3448
8297	Tcte3	1.437	3.234	4.647	0.662	0.020	0.025	1.276	1.799	3.492	21647	Tcte3	chr17	-	NM_1981	P12166	protein_cc	D17Leh11	t-complex	Tcte3
8298	9030025P	0.289	0.949	0.274	0.003	0.870	0.028	5.917	4.127	4.051	10004157	9030025P	chr17	+	NM_0011	P6897	protein_cc	D17Zt9e	RIKEN cDN	9030025P
8299	Gm3435	0.150	1.980	0.297	0.000	0.056	0.010	6.083	3.347	4.332	10004162	Gm3435	chr17	+	NM_0011	P13077	protein_cc	10004162	predicted	Gm3435
8300	Ermard	0.416	1.245	0.518	0.008	0.446	0.025	6.236	4.972	5.289	381062	Ermard	chr17	+	NM_0010	P24803	protein_cc	2210404J	ER membr	Ermard
8301	Gm5091	0.716	0.995	0.712	0.182	1.000	0.423	0.490	0.008	0.000	328747	Gm5091	chr17	-	NR_04616	.	lncRNA	328747| C	predicted	Gm5091
8302	Gm10510	1.000	1.000	1.000	1.000	1.000	1.000	0.000	0.000	0.000	10003848	Gm10510	chr17	-	NR_03351	.	lncRNA	ENSMUSG	predicted	Gm10510
8303	Dll1	1.020	1.853	1.891	0.941	0.165	0.443	3.393	3.423	4.312	13388	Dll1	chr17	-	NM_0078	P26680	protein_cc	Delta1	delta-like	Dll1
8304	Fam120b	0.866	0.904	0.782	0.114	0.713	0.607	6.075	5.867	5.721	67544	Fam120b	chr17	+	NR_03358	.	protein_cc	4932442K	family wit	Fam120b
8305	Psmb1	0.957	1.196	1.145	0.609	0.065	0.166	11.258	11.195	11.453	19170	Psmb1	chr17	-	NM_0111	P17850	protein_cc	AA409053	proteason	Psmb1
8306	Tbp	1.525	0.840	1.281	0.153	0.454	0.339	5.826	6.434	6.184	21374	Tbp	chr17	+	NM_0136	P4574	protein_cc	Gtf2d| GT	TATA box	Tbp
8307	Pdcd2	0.740	1.512	1.118	0.162	0.097	0.670	7.118	6.682	7.278	18567	Pdcd2	chr17	-	NM_0087	P20947	protein_cc	AI528543|	programm	Pdcd2
8308	Prdm9	1.280	0.966	1.237	0.489	0.902	0.778	4.763	5.119	5.070	213389	Prdm9	chr17	-	NM_1448	P6970	protein_cc	BC012016	PR domair	Prdm9
8309	4933401D	0.451	0.986	0.444	0.182	1.000	0.423	1.170	0.021	0.000	71008	4933401D	chr17	+	NR_04543	.	lncRNA		RIKEN cDN	4933401D
8310	Chd1	1.774	0.507	0.899	0.001	0.000	0.554	8.588	9.415	8.434	12648	Chd1	chr17	+	NM_0076	P6274	protein_cc	4930525N	chromodo	Chd1
8311	Rgmb	1.304	1.558	2.032	0.495	0.315	0.421	3.460	3.843	4.483	68799	Rgmb	chr17	-	NM_1786	P22389	protein_cc	1110059F	repulsive	Rgmb
8312	Zfp960	0.937	1.068	1.001	0.623	0.698	0.998	5.989	5.895	5.990	449000	Zfp960	chr17	+	NM_0011	P4605	protein_cc	.	zinc finger	Zfp960
8313	Zfp97	1.251	0.934	1.168	0.417	0.260	0.739	5.802	6.124	6.026	22759	Zfp97	chr17	+	NM_0117	P25594	protein_cc	NGD16-4	zinc finger	Zfp97
8314	Riok2	1.025	1.093	1.121	0.891	0.646	0.500	7.114	7.149	7.278	67045	Riok2	chr17	+	NM_0259	P829	protein_cc	2010110K	RIO kinase	Riok2
8315	Lix1	0.981	0.913	0.895	0.976	0.879	0.918	2.836	2.808	2.676	66643	Lix1	chr17	+	NM_0256	P2365	protein_cc	5730466L	limb and C	Lix1
8316	Lnpep	2.295	0.759	1.741	0.028	0.237	0.242	3.515	4.714	4.315	240028	Lnpep	chr17	-	NM_1728	P18493	protein_cc	2010309L	leucyl/cys	Lnpep
8317	Vmn2r90	1.000	1.000	1.000	1.000	1.000	1.000	0.000	0.000	0.000	626942	Vmn2r90	chr17	+	NM_0011	P17130	protein_cc	EG626942	vomerona	Vmn2r90
8318	Spaca6	1.033	1.907	1.969	0.935	0.216	0.438	1.741	1.788	2.719	75202	Spaca6	chr17	+	NM_0011	P12355	protein_cc	4930546H	sperm acr	Spaca6
8319	Has1	12.340	0.652	8.047	0.000	0.294	0.343	0.002	3.627	3.011	15116	Has1	chr17	-	NM_0082	P19970	protein_cc	HAS	hyalurona	Has1
8320	Fpr1	7.561	0.723	5.465	0.000	0.092	0.069	5.229	8.148	7.679	14293	Fpr1	chr17	-	NM_0135	P3821	protein_cc	fMLF-R| F	formyl pe	Fpr1
8321	Fpr2	30.230	0.910	27.509	0.000	0.223	0.004	4.580	9.498	9.362	14289	Fpr2	chr17	+	NM_0080	P20787	protein_cc	E330010I	formyl pe	Fpr2
8322	Fpr3	1.000	1.000	1.000	1.000	1.000	1.000	0.000	0.000	0.000	14294	Fpr3	chr17	+	NM_0080	P22626	protein_cc	ALX| Fpr-r	formyl pe	Fpr3
8323	Fpr-rs4	1.000	1.000	1.000	1.000	1.000	1.000	0.000	0.000	0.000	14291	Fpr-rs4	chr17	+	NM_0080	P732	protein_cc	.	formyl pe	Fpr-rs4
8324	Vmn2r124	1.000	1.000	1.000	1.000	1.000	1.000	0.000	0.000	0.000	637021	Vmn2r124	chr17	+	NM_0012	P18347	protein_cc	EG637021	vomerona	Vmn2r124
8325	Vmn2r91	1.000	1.000	1.000	1.000	1.000	1.000	0.000	0.000	0.000	665210	Vmn2r91	chr17	+	NM_0011	P26185	protein_cc	EG665210	vomerona	Vmn2r91
8326	Vmn2r92	1.000	1.000	1.000	1.000	1.000	1.000	0.000	0.000	0.000	627111	Vmn2r92	chr17	+	NM_0011	P23402	protein_cc	EG627111	vomerona	Vmn2r92
8327	Vmn2r94	1.000	1.000	1.000	1.000	1.000	1.000	0.000	0.000	0.000	665227	Vmn2r94	chr17	+	NM_0011	P15419	protein_cc	EG665227	vomerona	Vmn2r94
8328	Vmn2r93	1.000	1.000	1.000	1.000	1.000	1.000	0.000	0.000	0.000	627132	Vmn2r93	chr17	+	NM_0011	P24315	protein_cc	EG627132	vomerona	Vmn2r93
8329	Vmn2r95	1.000	1.000	1.000	1.000	1.000	1.000	0.000	0.000	0.000	328759	Vmn2r95	chr17	+	NM_0011	P15383	protein_cc	.	vomerona	Vmn2r95
8330	Vmn2r96	1.000	1.000	1.000	1.000	1.000	1.000	0.000	0.000	0.000	433070	Vmn2r96	chr17	+	NM_0011	P5083	protein_cc	EG433070	vomerona	Vmn2r96
8331	Vmn2r97	1.580	0.633	1.000	0.305	1.000	0.423	0.000	0.660	0.000	627367	Vmn2r97	chr17	+	NM_0011	P17664	protein_cc	EG627367	vomerona	Vmn2r97
8332	Vmn2r98	1.000	1.000	1.000	1.000	1.000	1.000	0.000	0.000	0.000	224552	Vmn2r98	chr17	+	NM_0011	P14497	protein_cc	EG224552	vomerona	Vmn2r98
8333	Vmn2r99	1.000	1.000	1.000	1.000	1.000	1.000	0.000	0.000	0.000	665376	Vmn2r99	chr17	+	NM_0011	P14847	protein_cc	EG665376	vomerona	Vmn2r99
8334	Vmn2r100	1.000	1.000	1.000	1.000	1.000	1.000	0.000	0.000	0.000	627537	Vmn2r100	chr17	+	NM_0011	P22431	protein_cc	EG627537	vomerona	Vmn2r100
8335	Vmn2r101	1.000	1.000	1.000	1.000	1.000	1.000	0.000	0.000	0.000	627576	Vmn2r101	chr17	+	NM_0011	P5672	protein_cc	EG627576	vomerona	Vmn2r101
8336	Vmn2r102	1.000	1.000	1.000	1.000	1.000	1.000	0.000	0.000	0.000	224572	Vmn2r102	chr17	+	NM_0011	P19352	protein_cc	EG224572	vomerona	Vmn2r102
8337	Vmn2r103	1.000	1.000	1.000	1.000	1.000	1.000	0.000	0.000	0.000	627636	Vmn2r103	chr17	+	NM_0011	P12622	protein_cc	EG627636	vomerona	Vmn2r103
8338	Vmn2r104	1.000	1.000	1.000	1.000	1.000	1.000	0.000	0.000	0.000	22313	Vmn2r104	chr17	+	NM_0011	P1835	protein_cc	V2r7	vomerona	Vmn2r104
8339	Fpr-rs6	1.000	1.000	1.000	1.000	1.000	1.000	0.000	0.000	0.000	321020	Fpr-rs6	chr17	-	NM_1773	P24154	protein_cc	.	formyl pe	Fpr-rs6
8340	Vmn2r105	1.000	1.000	1.000	1.000	1.000	1.000	0.000	0.000	0.000	627743	Vmn2r105	chr17	-	NM_0011	P2601	protein_cc	EG627743	vomerona	Vmn2r105
8341	Vmn2r106	1.000	1.000	1.000	1.000	1.000	1.000	0.000	0.000	0.000	224576	Vmn2r106	chr17	-	NM_0011	P20806	protein_cc	EG224576	vomerona	Vmn2r106
8342	Vmn2r107	1.000	1.000	1.000	1.000	1.000	1.000	0.000	0.000	0.000	22312	Vmn2r107	chr17	+	NM_0011	P20690	protein_cc	V2r6| Vmi	vomerona	Vmn2r107
8343	Vmn1r224	1.000	1.000	1.000	1.000	1.000	1.000	0.000	0.000	0.000	665525	Vmn1r224	chr17	+	NM_0011	P129	protein_cc	EG665525	vomerona	Vmn1r224
8344	Vmn2r108	1.000	1.000	1.000	1.000	1.000	1.000	0.000	0.000	0.000	627805	Vmn2r108	chr17	-	NM_0011	P8643	protein_cc	EG627805	vomerona	Vmn2r108
8345	Vmn1r225	0.716	0.995	0.712	0.182	1.000	0.423	0.490	0.008	0.000	171228	Vmn1r225	chr17	+	NM_1341	P9180	protein_cc	V1re5	vomerona	Vmn1r225
8346	Vmn2r109	1.000	1.000	1.000	1.000	1.000	1.000	0.000	0.000	0.000	627814	Vmn2r109	chr17	-	NM_0011	P26474	protein_cc	EG627814	vomerona	Vmn2r109
8347	Vmn2r110	1.000	1.000	1.000	1.000	1.000	1.000	0.000	0.000	0.000	224582	Vmn2r110	chr17	-	NM_0011	P1169	protein_cc	EG224582	vomerona	Vmn2r110
8348	Fpr-rs3	1.000	1.000	1.000	1.000	1.000	1.000	0.000	0.000	0.000	14290	Fpr-rs3	chr17	-	NM_0080	P8988	protein_cc	.	formyl pe	Fpr-rs3
8349	Vmn1r226	1.000	1.000	1.000	1.000	1.000	1.000	0.000	0.000	0.000	171225	Vmn1r226	chr17	+	NM_1341	P5554	protein_cc	V1re2	vomerona	Vmn1r226
8350	Vmn1r227	1.000	1.000	1.000	1.000	1.000	1.000	0.000	0.000	0.000	171229	Vmn1r227	chr17	+	NM_1341	P10249	protein_cc	V1re6	vomerona	Vmn1r227
8351	Vmn1r228	1.000	1.000	1.000	1.000	1.000	1.000	0.000	0.000	0.000	171226	Vmn1r228	chr17	-	NM_1341	P7808	protein_cc	V1re3| Vl	vomerona	Vmn1r228
8352	Vmn1r229	1.000	1.000	1.000	1.000	1.000	1.000	0.000	0.000	0.000	171224	Vmn1r229	chr17	+	NM_1341	P2582	protein_cc	V1re1	vomerona	Vmn1r229
8353	Vmn1r230	1.000	1.000	1.000	1.000	1.000	1.000	0.000	0.000	0.000	171231	Vmn1r230	chr17	+	NM_1341	P17175	protein_cc	V1re8	vomerona	Vmn1r230
8354	Vmn1r231	1.000	1.000	1.000	1.000	1.000	1.000	0.000	0.000	0.000	171230	Vmn1r231	chr17	-	NM_1341	P25485	protein_cc	V1re5| V1	vomerona	Vmn1r231
8355	Vmn1r232	1.000	1.000	1.000	1.000	1.000	1.000	0.000	0.000	0.000	171227	Vmn1r232	chr17	-	NM_1341	P16601	protein_cc	V1re4	vomerona	Vmn1r232
8356	Ppp2r1a	1.377	1.097	1.511	0.013	0.322	0.080	9.404	9.866	9.999	51792	Ppp2r1a	chr17	+	NM_0168	P24518	protein_cc	6330556D	protein ph	Ppp2r1a
8357	Vmn1r233	1.000	1.000	1.000	1.000	1.000	1.000	0.000	0.000	0.000	171236	Vmn1r233	chr17	-	NM_1342	P11908	protein_cc	V1rf5	vomerona	Vmn1r233
8358	Zfp160	1.813	0.592	1.073	0.003	0.001	0.765	6.159	7.017	6.261	224585	Zfp160	chr17	+	NM_1454	P11835	protein_cc	6720480D	zinc finger	Zfp160
8359	Vmn1r234	1.000	1.000	1.000	1.000	1.000	1.000	0.000	0.000	0.000	171232	Vmn1r234	chr17	+	NM_1341	P19065	protein_cc	V1rf1	vomerona	Vmn1r234

8360	Vmn1r235	1.000	1.000	1.000	1.000	1.000	1.000	0.000	0.000	0.000	171233	Vmn1r235	chr17	+	NM_1341	P5040	protein_cc	V1rf2	vomerona	Vmn1r235
8361	Vmn1r236	1.000	1.000	1.000	1.000	1.000	1.000	0.000	0.000	0.000	171235	Vmn1r236	chr17	+	NM_1342	P2934	protein_cc	V1rf4	vomerona	Vmn1r236
8362	Vmn1r237	1.000	1.000	1.000	1.000	1.000	1.000	0.000	0.000	0.000	171234	Vmn1r237	chr17	+	NM_1342	P14115	protein_cc	V1rf3| Vfr	vomerona	Vmn1r237
8363	Zfp677	3.756	0.219	0.823	0.142	0.122	0.740	0.749	2.658	0.468	210503	Zfp677	chr17	+	NM_1724	P11804	protein_cc	A830058L	zinc finger	Zfp677
8364	Zfp54	2.157	1.510	3.257	0.154	0.193	0.057	3.354	4.463	5.057	22712	Zfp54	chr17	+	NM_0117	P23283	protein_cc	2810034D	zinc finger	Zfp54
8365	Zfp51	2.867	1.123	3.219	0.069	0.758	0.212	4.465	5.985	6.152	22709	Zfp51	chr17	+	NM_0013	P24701	protein_cc	zfec12| Zf	zinc finger	Zfp51
8366	Zfp53	0.964	1.121	1.081	0.685	0.516	0.780	4.117	4.065	4.230	24132	Zfp53	chr17	+	NM_0138	P20648	protein_cc	D030067C	zinc finger	Zfp53
8367	9330136K	1.610	0.640	1.030	0.482	0.421	0.972	1.993	2.680	2.036	319870	9330136K	chr17	-	NR_13112	.	lncRNA	.	RIKEN cDN	9330136K2
8368	Zfp52	6.844	0.718	4.911	0.013	0.287	0.142	2.069	4.844	4.365	22710	Zfp52	chr17	+	NM_1445	P26161	protein_cc	KRAB11| r	zinc finger	Zfp52
8369	Zfp948	1.555	0.808	1.256	0.139	0.000	0.654	4.238	4.874	4.566	381066	Zfp948	chr17	+	NM_0010	P18545	protein_cc	.	zinc finger	Zfp948
8370	3110052M	0.705	0.769	0.542	0.380	0.616	0.010	5.634	5.129	4.751	73229	3110052M	chr17	+	NM_0011	P3660	protein_cc	mszf57| m	RIKEN cDN	3110052M
8371	Gm10509	1.000	0.459	0.459	0.999	1.000	0.422	1.125	1.124	0.000	10031080	Gm10509	chr17	+	NR_04576	.	misc_RNA	ENSMUSG	predicted	Gm10509
8372	Zfp760	0.652	0.613	0.400	0.267	0.401	0.039	4.579	3.961	3.256	240034	Zfp760	chr17	+	NM_0010	P5815	protein_cc	9430018I0	zinc finger	Zfp760
8373	Zfp229	1.213	0.955	1.158	0.278	0.901	0.849	5.115	5.394	5.326	381067	Zfp229	chr17	+	NM_0011	P1415	protein_cc	.	zinc finger	Zfp229
8374	Zfp820	0.972	1.023	0.994	0.798	0.785	0.976	5.981	5.939	5.971	75424	Zfp820	chr17	-	NM_0292	P4898	protein_cc	2610036F0	zinc finger	Zfp820
8375	2210404O	1.290	0.795	1.025	0.519	0.611	0.956	4.379	4.745	4.415	70081	2210404O	chr17	-	NM_0012	P4180	protein_cc	.	RIKEN cDN	2210404O
8376	Zfp942	1.326	0.740	0.981	0.032	0.124	0.947	5.249	5.656	5.222	73233	Zfp942	chr17	-	NM_0011	P26884	protein_cc	3110048L1	zinc finger	Zfp942
8377	Zfp943	1.150	0.848	0.975	0.319	0.473	0.940	6.120	6.321	6.084	74670	Zfp943	chr17	+	NM_0010	P23018	protein_cc	4930432O	zinc finger	Zfp943
8378	Zfp947	2.594	0.680	1.765	0.068	0.310	0.293	4.293	5.668	5.112	210853	Zfp947	chr17	-	NM_1775	P25040	protein_cc	6720451E	zinc finger	Zfp947
8379	Gm4944	2.437	0.562	1.369	0.021	0.049	0.480	5.369	6.653	5.822	240038	Gm4944	chr17	-	NM_0012	P1234	protein_cc	EG240038	predicted	Gm4944
8380	Zfp944	0.991	1.018	1.009	0.940	0.873	0.956	5.882	5.869	5.895	319615	Zfp944	chr17	-	NM_1769	P6477	protein_cc	6330416L0	zinc finger	Zfp944
8381	Zfp758	2.355	0.625	1.472	0.037	0.091	0.468	3.634	4.870	4.192	224598	Zfp758	chr17	+	NM_1454	P3714	protein_cc	BC021442	zinc finger	Zfp758
8382	Zfp946	0.952	1.385	1.318	0.559	0.049	0.325	6.500	6.429	6.899	74149	Zfp946	chr17	+	NM_1980	P13452	protein_cc	1300003B	zinc finger	Zfp946
8383	Vmn2r111	1.000	1.000	1.000	1.000	1.000	1.000	0.000	0.000	0.000	210876	Vmn2r111	chr17	-	NM_0011	P18319	protein_cc	EG210876	vomerona	Vmn2r111
8384	Vmn2r112	1.000	1.000	1.000	1.000	1.000	1.000	0.000	0.000	0.000	628185	Vmn2r112	chr17	+	NM_0011	P25143	protein_cc	EG628185	vomerona	Vmn2r112
8385	Gm16386	3.510	0.456	1.600	0.138	0.246	0.442	0.845	2.657	1.523	10004267	Gm16386	chr17	.	NR_03070	.	misc_RNA	EG545191	predicted	Gm16386
8386	Zfp945	1.775	0.658	1.168	0.013	0.081	0.703	6.858	7.686	7.082	240041	Zfp945	chr17	-	NM_1773	P21372	protein_cc	A630033E	zinc finger	Zfp945
8387	Vmn2r113	1.000	1.000	1.000	1.000	1.000	1.000	0.000	0.000	0.000	434701	Vmn2r113	chr17	+	NM_0011	P21846	protein_cc	EG434701	vomerona	Vmn2r113
8388	Vmn2r-ps1	0.943	1.357	1.280	0.908	0.559	0.680	1.221	1.137	1.576	665976	Vmn2r-ps1	chr17	+	NR_03364	.	misc_RNA	.	vomerona	Vmn2r-ps1
8389	Zfp40	3.535	1.755	6.204	0.215	0.225	0.047	2.822	4.644	5.455	22700	Zfp40	chr17	-	NM_0095	P19490	protein_cc	CTfin12| N	zinc finger	Zfp40
8390	Vmn2r114	1.000	1.000	1.000	1.000	1.000	1.000	0.000	0.000	0.000	666002	Vmn2r114	chr17	-	NM_0011	P24421	protein_cc	EG666002	vomerona	Vmn2r114
8391	Vmn2r115	1.000	1.000	1.000	1.000	1.000	1.000	0.000	0.000	0.000	638102	Vmn2r115	chr17	+	NM_0011	P8045	protein_cc	EG638102	vomerona	Vmn2r115
8392	Vmn2r116	1.000	1.000	1.000	1.000	1.000	1.000	0.000	0.000	0.000	619697	Vmn2r116	chr17	+	NM_0011	P1217	protein_cc	EG619697	vomerona	Vmn2r116
8393	Vmn2r117	1.000	1.000	1.000	1.000	1.000	1.000	0.000	0.000	0.000	619788	Vmn2r117	chr17	-	NM_0011	P20876	protein_cc	EG619788	vomerona	Vmn2r117
8394	6330415G	1.052	0.970	1.020	0.918	0.910	0.984	2.140	2.213	2.169	320020	6330415G	chr17	+	NR_12648	.	lncRNA	.	RIKEN cDN	6330415G
8395	Zfp213	1.482	0.834	1.236	0.144	0.261	0.533	5.300	5.868	5.606	449521	Zfp213	chr17	-	NM_0010	P23725	protein_cc	BC038178	zinc finger	Zfp213
8396	Zfp13	1.082	1.370	1.482	0.874	0.296	0.515	3.309	3.423	3.877	22654	Zfp13	chr17	-	NM_0117	P21180	protein_cc	4933429B	zinc finger	Zfp13
8397	Zscan10	1.000	1.000	1.000	1.000	1.000	1.000	0.000	0.000	0.000	332221	Zscan10	chr17	+	NM_0010	P26408	protein_cc	BC068284	zinc finger	Zscan10
8398	Mmp25	5.051	0.655	3.310	0.305	0.652	0.272	0.001	2.338	1.728	240047	Mmp25	chr17	-	NM_0010	P20833	protein_cc	F730048C	matrix me	Mmp25
8399	Ccdc64b	1.885	2.279	4.296	0.162	0.002	0.048	2.799	3.713	4.901	212733	Ccdc64b	chr17	+	NM_1537	P16032	protein_cc	BICDR-2	coiled-coil	Ccdc64b
8400	Thoc6	1.668	0.925	1.542	0.065	0.683	0.291	6.440	7.179	7.066	386612	Thoc6	chr17	-	NM_0010	P18426	protein_cc	F830014G	THO comp	Thoc6
8401	Hcfc1r1	0.463	1.390	0.643	0.001	0.096	0.116	9.129	8.018	8.493	353502	Hcfc1r1	chr17	+	NM_1818	P4454	protein_cc	HPIP	host cell fa	Hcfc1r1
8402	Tnfrsf12a	10.305	1.766	18.196	0.005	0.051	0.047	4.026	7.392	8.212	27279	Tnfrsf12a	chr17	-	NM_0011	P26566	protein_cc	AI255180|	tumor nec	Tnfrsf12a
8403	Cldn6	0.185	1.288	0.238	0.034	0.227	0.214	2.498	0.062	0.427	54419	Cldn6	chr17	+	NM_0187	P2606	protein_cc	AL024037	claudin 6	Cldn6
8404	Cldn9	1.000	1.000	1.000	1.000	1.000	1.000	0.000	0.000	0.000	56863	Cldn9	chr17	-	NM_0202	P2188	protein_cc	nmf329	claudin 9	Cldn9
8405	Pkmyt1	0.596	1.008	0.600	0.146	0.961	0.390	4.525	3.778	3.789	268930	Pkmyt1	chr17	+	NM_0230	P14221	protein_cc	6230424P	protein kir	Pkmyt1
8406	Paqr4	0.574	0.991	0.569	0.112	0.938	0.327	5.149	4.347	4.334	76498	Paqr4	chr17	-	NM_0238	P8915	protein_cc	1500004C	progestin	Paqr4
8407	Kremen2	2.097	1.382	2.898	0.388	0.667	0.471	0.401	1.469	1.936	73016	Kremen2	chr17	-	NM_0284	P2517	protein_cc	2900054E0	kringle co	Kremen2
8408	9530082P	1.430	0.919	1.314	0.021	0.042	0.251	6.878	7.394	7.272	638247	9530082P	chr17	+	NR_01547	.	lncRNA	E030034P	RIKEN cDN	9530082P2
8409	Flywch1	2.054	1.084	2.226	0.001	0.676	0.174	6.855	7.893	8.009	224613	Flywch1	chr17	-	NM_1537	P26314	protein_cc	E030034P	FLYWCH-t	Flywch1
8410	Flywch2	0.820	2.134	1.749	0.588	0.114	0.331	3.929	3.643	4.736	76917	Flywch2	chr17	-	NM_0297	P1635	protein_cc	2810417J1	FLYWCH fa	Flywch2
8411	Srrm2	1.314	0.527	0.692	0.002	0.000	0.026	10.694	11.088	10.163	75956	Srrm2	chr17	+	NM_1752	P15781	protein_cc	5033413A	serine/arg	Srrm2
8412	Tceb2	0.972	1.248	1.213	0.766	0.009	0.304	10.007	9.966	10.286	67673	Tceb2	chr17	-	NM_0263	P7929	protein_cc	0610040H	transcripti	Tceb2
8413	Prss33	1.000	1.000	1.000	1.000	1.000	1.000	0.000	0.000	0.000	353130	Prss33	chr17	-	NM_0010	P3854	protein_cc	Eos| mT6|	protease,	Prss33
8414	Prss41	1.000	1.000	1.000	1.000	1.000	1.000	0.000	0.000	0.000	71003	Prss41	chr17	-	NM_0276	P5037	protein_cc	4931440B	protease,	Prss41
8415	Prss32	1.000	1.000	1.000	1.000	1.000	1.000	0.000	0.000	0.000	69814	Prss32	chr17	-	NM_0272	P17909	protein_cc	2010001P	protease,	Prss32
8416	Prss21	1.000	1.000	1.000	1.000	1.000	1.000	0.000	0.000	0.000	57256	Prss21	chr17	-	NM_0204	P5006	protein_cc	1700023E	protease,	Prss21
8417	Dcpp1	1.000	1.000	1.000	1.000	1.000	1.000	0.000	0.000	0.000	13184	Dcpp1	chr17	-	NM_0199	P5267	protein_cc	BC005655	demilune	Dcpp1
8418	Dcpp2	1.000	1.000	1.000	1.000	1.000	1.000	0.000	0.000	0.000	630537	Dcpp2	chr17	-	NM_0010	P2548	protein_cc	Dcpp-2| E	demilune	Dcpp2
8419	Dcpp3	1.000	1.000	1.000	1.000	1.000	1.000	0.000	0.000	0.000	620253	Dcpp3	chr17	+	NM_0010	P19816	protein_cc	Dcpp-3| E	demilune	Dcpp3
8420	Sbp	1.000	1.000	1.000	1.000	1.000	1.000	0.000	0.000	0.000	20234	Sbp	chr17	+	NM_0113	P24913	protein_cc	AV082137	spermine	Sbp
8421	Sbpl	1.000	1.000	1.000	1.000	1.000	1.000	0.000	0.000	0.000	638345	Sbpl	chr17	-	NM_0010	P23741	protein_cc	2310068J2	spermine	Sbpl
8422	Prss30	1.027	3.619	3.716	0.000	0.181	0.423	0.001	0.039	1.894	30943	Prss30	chr17	-	NM_0139	P25771	protein_cc	2010015P	protease,	Prss30
8423	Prss22	1.015	2.326	2.360	0.000	0.182	0.423	0.000	0.021	1.239	70835	Prss22	chr17	-	NM_1337	P2062	protein_cc	4733401N	protease,	Prss22
8424	Prss27	1.005	1.439	1.446	0.000	0.182	0.423	0.000	0.008	0.532	213171	Prss27	chr17	+	NM_1754	P18136	protein_cc	CAPH2| m	protease,	Prss27
8425	Kctd5	1.832	1.026	1.881	0.011	0.916	0.312	5.706	6.579	6.617	69259	Kctd5	chr17	-	NM_0270	P2002	protein_cc	2610030N	potassium	Kctd5
8426	Pdpk1	0.992	1.283	1.272	0.971	0.073	0.514	5.256	5.244	5.603	18607	Pdpk1	chr17	-	NM_0010	P15506	protein_cc	Pdk1	3-phospho	Pdpk1
8427	Amdhd2	0.308	1.523	0.470	0.001	0.057	0.059	7.680	5.982	6.581	245847	Amdhd2	chr17	-	NM_1729	P19320	protein_cc	5730457F	amidohyd	Amdhd2
8428	Atp6v0c-p	1.474	0.918	1.353	0.088	0.615	0.188	10.401	10.961	10.838	10003963	Atp6v0c-p	chr17	-	NR_03785	.	misc_RNA	Atp6l-ps2|	ATPase, H	Atp6v0c-p
8429	Atp6v0c	1.473	0.918	1.352	0.088	0.616	0.189	10.401	10.960	10.837	11984	Atp6v0c	chr17	-	NM_0097	P7138	protein_cc	Atp6c| At	ATPase, H	Atp6v0c
8430	Tbc1d24	1.859	0.950	1.766	0.010	0.593	0.043	7.175	8.070	7.996	224617	Tbc1d24	chr17	-	NM_0011	P22770	protein_cc	9630033P	TBC1 dom	Tbc1d24
8431	Ntn3	0.213	0.498	0.106	0.005	0.218	0.053	5.667	3.436	2.429	18209	Ntn3	chr17	-	NM_0109	P9945	protein_cc	Ntn2l	netrin 3	Ntn3
8432	1600002H	0.192	2.483	0.477	0.000	0.128	0.182	8.999	6.620	7.932	72016	1600002H	chr17	-	NM_0280	P10048	protein_cc	.	RIKEN cDN	1600002H
8433	Ccnf	0.406	2.037	0.827	0.000	0.045	0.543	6.523	5.222	6.249	12449	Ccnf	chr17	-	NM_0076	P14481	protein_cc	CycF| Fbx	cyclin F	Ccnf
8434	Abca17	0.716	0.995	0.712	0.182	1.000	0.423	0.490	0.008	0.000	381072	Abca17	chr17	-	NM_0010	P20130	protein_cc	.	ATP-bindi	Abca17
8435	Abca3	0.257	0.836	0.215	0.000	0.256	0.000	8.447	6.487	6.228	27410	Abca3	chr17	+	NM_0138	P11486	protein_cc	1810036E	ATP-bindi	Abca3
8436	D330041H	0.247	0.858	0.212	0.000	0.338	0.005	8.374	6.356	6.134	654822	D330041H	chr17	-	NR_03355	.	lncRNA	.	RIKEN cDN	D330041H
8437	Rnps1	2.010	0.875	1.758	0.003	0.288	0.079	6.456	7.463	7.270	19826	Rnps1	chr17	+	NM_0010	P8118	protein_cc	.	ribonuclei	Rnps1
8438	Eci1	0.416	1.443	0.601	0.000	0.001	0.044	10.889	9.625	10.154	13177	Eci1	chr17	+	NM_0100	P26840	protein_cc	Dci| eci	enoyl-Coe	Eci1
8439	Dnase1l2	0.823	0.864	0.711	0.681	0.684	0.643	2.064	1.783	1.571	66705	Dnase1l2	chr17	-	NM_0257	P6581	protein_cc	4733401H	deoxyribo	Dnase1l2
8440	E4f1	0.718	1.157	0.831	0.013	0.094	0.148	5.199	4.721	4.932	13560	E4f1	chr17	-	NR_12599	.	protein_cc	p120E4F|	E4F transc	E4f1
8441	Pgp	1.578	1.361	2.148	0.019	0.095	0.129	7.479	8.137	8.582	67078	Pgp	chr17	+	NM_0259	P26669	protein_cc	1700012G	phosphog	Pgp
8442	Mlst8	1.367	0.992	1.356	0.333	0.980	0.587	5.865	6.316	6.305	56716	Mlst8	chr17	-	NM_0199	P7467	protein_cc	0610033N	MTOR ass	Mlst8
8443	Bricd5	1.510	2.275	3.436	0.055	0.015	0.099	1.200	1.795	2.981	319259	Bricd5	chr17	-	NM_1756	P9094	protein_cc	9930021D	BRICHOS d	Bricd5
8444	Caskin1	2.423	0.695	1.683	0.008	0.042	0.121	5.544	6.821	6.295	268932	Caskin1	chr17	+	NM_0279	P8441	protein_cc	3300002N	CASK inter	Caskin1
8445	Traf7	2.669	0.705	1.881	0.001	0.013	0.071	6.135	7.551	7.047	224619	Traf7	chr17	-	NM_1537	P20425	protein_cc	RFWD1	TNF recep	Traf7
8446	Rab26os	2.839	1.043	2.960	0.041	0.846	0.029	5.425	6.931	6.991	75614	Rab26os	chr17	+	NR_04528	.	lncRNA	2610019E	RAB26, me	Rab26os
8447	Rab26	1.000	1.000	1.000	1.000	1.000	1.000	0.000	0.000	0.000	328778	Rab26	chr17	-	NM_1773	P3814	protein_cc	A830020N	RAB26, me	Rab26
8448	Pkd1	0.690	1.045	0.720	0.026	0.769	0.137	6.639	6.103	6.166	18763	Pkd1	chr17	+	NM_0136	P24835	protein_cc	mFLJ0028	polycystic	Pkd1
8449	Tsc2	0.583	1.170	0.682	0.000	0.284	0.128	7.558	6.778	7.005	22084	Tsc2	chr17	-	NM_0010	P14445	protein_cc	Nafld| Tcs	tuberous s	Tsc2
8450	Nthl1	0.271	1.098	0.298	0.008	0.853	0.055	4.128	2.245	2.380	18207	Nthl1	chr17	+	NM_0087	P17959	protein_cc	Nth1| Oct	nth (endo	Nthl1

8451	Slc9a3r2	0.189	3.198	0.603	0.000	0.040	0.222	7.557	5.151	6.828	65962	Slc9a3r2	chr17	-	NM_0234	P17779	protein_cc	0610011L(	solute car	Slc9a3r2
8452	Npw	1.000	1.000	1.000	1.000	1.000	1.000	0.000	0.000	0.000	381073	Npw	chr17	-	NM_0010	P11052	protein_cc	Gm935	neuropept	Npw
8453	Zfp598	1.805	0.613	1.107	0.081	0.112	0.439	8.302	9.154	8.449	213573	Zfp598	chr17	+	NM_1831	P24899	protein_cc	BC023040	zinc finger	Zfp598
8454	Syngr3	1.010	1.885	1.904	0.000	0.182	0.423	0.000	0.015	0.929	20974	Syngr3	chr17	-	NM_0115	P2598	protein_cc		synaptogy	Syngr3
8455	Gfer	1.450	1.012	1.468	0.001	0.777	0.012	9.623	10.159	10.177	11692	Gfer	chr17	-	NM_0230	P19535	protein_cc	Alr| ERV1	growth fa	Gfer
8456	Noxo1	0.696	1.343	0.935	0.051	0.420	0.903	3.576	3.054	3.479	71893	Noxo1	chr17	+	NM_0279	P25925	protein_cc	2310034C	NADPH ox	Noxo1
8457	Tbl3	0.870	1.675	1.457	0.437	0.013	0.208	5.737	5.536	6.280	213773	Tbl3	chr17	-	NM_1453	P8154	protein_cc	9430070M	transducir	Tbl3
8458	Rnf151	1.005	1.439	1.446	0.000	0.182	0.423	0.000	0.008	0.532	67504	Rnf151	chr17	-	NM_0262	P9506	protein_cc	1700010O	ring finger	Rnf151
8459	Snhg9	1.044	2.364	2.469	0.280	0.000	0.001	5.666	5.729	6.970	73474	Snhg9	chr17	-	NR_02790	.	lncRNA	1700072N	small nucl	Snhg9
8460	Snora78	7.632	0.439	3.351	0.056	0.230	0.502	1.001	3.933	2.745	10030695	Snora78	chr17	-	NR_02851	.	snoRNA	ACA64	small nucl	Snora78
8461	Rps2	1.073	0.965	1.035	0.696	0.833	0.775	11.078	11.179	11.128	16898	Rps2	chr17	+	NM_0085	P11180	protein_cc	Llrep3| S4	ribosomal	Rps2
8462	Snora64	1.639	1.223	2.004	0.424	0.734	0.552	1.299	2.011	2.302	104366	Snora64	chr17	+	NR_00289	.	snoRNA	MBI-29| R	small nucl	Snora64
8463	Ndufb10	0.356	1.490	0.530	0.000	0.000	0.002	10.742	9.251	9.826	68342	Ndufb10	chr17	-	NM_0266	P15300	protein_cc	0610011B	NADH deh	Ndufb10
8464	Rpl3l	3.369	1.854	6.246	0.300	0.330	0.195	0.001	1.753	2.644	66211	Rpl3l	chr17	+	NM_0011	P19775	protein_cc	1110057H	ribosomal	Rpl3l
8465	Msrb1	0.233	1.700	0.397	0.000	0.000	0.002	11.873	9.773	10.538	27361	Msrb1	chr17	+	NM_0137	P10842	protein_cc	D17Wsu8	methionin	Msrb1
8466	Hs3st6	0.349	2.774	0.968	0.111	0.208	0.968	3.332	1.814	3.286	328779	Hs3st6	chr17	+	NM_0010	P21352	protein_cc	3-OST-6	heparan s	Hs3st6
8467	Meiob	0.065	3.221	0.208	0.000	0.238	0.072	6.018	2.068	3.755	75178	Meiob	chr17	+	NM_0291	P2897	protein_cc	4930528F	meiosis sp	Meiob
8468	Fahd1	0.066	3.634	0.241	0.001	0.010	0.042	9.116	5.199	7.061	68636	Fahd1	chr17	-	NM_0234	P10506	protein_cc	1110025H	fumarylac	Fahd1
8469	Hagh	0.148	1.433	0.212	0.000	0.053	0.002	10.881	8.122	8.640	14651	Hagh	chr17	+	NM_0242	P13948	protein_cc	BC019817	hydroxyac	Hagh
8470	Igfals	0.028	2.365	0.066	0.000	0.004	0.004	9.988	4.827	6.069	16005	Igfals	chr17	+	NM_0083	P20583	protein_cc	Albs| ALS|	insulin-like	Igfals
8471	Nubp2	0.911	1.457	1.328	0.504	0.007	0.282	7.998	7.864	8.407	26426	Nubp2	chr17	-	NM_0119	P4738	protein_cc	D17Wsu1	nucleotide	Nubp2
8472	Spsb3	0.782	1.082	0.846	0.118	0.451	0.477	6.754	6.399	6.514	79043	Spsb3	chr17	+	NM_0011	P15018	protein_cc	2310012N	splA/ryan	Spsb3
8473	Eme2	0.339	2.595	0.880	0.013	0.148	0.846	6.635	5.074	6.450	193838	Eme2	chr17	-	NM_0011	P12375	protein_cc	2810013J1	essential r	Eme2
8474	Mrps34	0.778	1.521	1.184	0.008	0.023	0.394	8.855	8.494	9.098	79044	Mrps34	chr17	+	NM_0232	P25642	protein_cc	0610007F	mitochonc	Mrps34
8475	Nme3	0.274	1.258	0.345	0.000	0.520	0.058	8.678	6.810	7.142	79059	Nme3	chr17	+	NM_0197	P16807	protein_cc	1810009F	NME/NM2	Nme3
8476	Mapk8ip3	1.461	0.942	1.376	0.282	0.846	0.404	5.072	5.618	5.532	30957	Mapk8ip3	chr17	-	NM_0139	P3307	protein_cc	BB120594	mitogen-a	Mapk8ip3
8477	Hn1l	3.092	0.519	1.606	0.000	0.000	0.025	6.282	7.911	6.965	52009	Hn1l	chr17	-	NM_1989	P19388	protein_cc	2810430B	hematolog	Hn1l
8478	Cramp1l	0.838	0.974	0.817	0.005	0.608	0.078	6.799	6.545	6.507	57354	Cramp1l	chr17	-	NM_0206	P18077	protein_cc	5830477H	Crm, cram	Cramp1l
8479	Ift140	0.282	0.640	0.180	0.042	0.043	0.172	5.616	3.787	3.144	106633	Ift140	chr17	+	NM_1341	P20480	protein_cc	AI661311|	intraflagel	Ift140
8480	Tmem204	0.137	0.672	0.092	0.000	0.314	0.005	6.837	3.965	3.391	407831	Tmem204	chr17	-	NM_0010	P16926	protein_cc	.	transmem	Tmem204
8481	Telo2	0.787	1.260	0.992	0.269	0.329	0.980	4.465	4.119	4.453	71718	Telo2	chr17	-	NM_0011	P4007	protein_cc	1200003M	telomere	Telo2
8482	Ptx4	1.000	1.000	1.000	1.000	1.000	1.000	0.000	0.000	0.000	68509	Ptx4	chr17	+	NM_0011	P7847	protein_cc	1110018H	pentraxin	Ptx4
8483	Clcn7	0.997	0.983	0.979	0.964	0.875	0.891	7.683	7.678	7.653	26373	Clcn7	chr17	+	NM_0119	P15880	protein_cc	ClC-7| D1	chloride c	Clcn7
8484	Ccdc154	1.000	1.000	1.000	1.000	1.000	1.000	0.000	0.000	0.000	207209	Ccdc154	chr17	+	NM_0010	P25836	protein_cc	Ccdc157|	coiled-coil	Ccdc154
8485	BC003965	0.870	0.918	0.798	0.047	0.115	0.053	8.359	8.157	8.033	214489	BC003965	chr17	+	NM_1831	P18880	protein_cc	Ccsmst1	cDNA seq	BC003965
8486	Unkl	0.682	1.108	0.755	0.077	0.368	0.428	7.544	6.991	7.139	74154	Unkl	chr17	+	NM_0011	P25352	protein_cc	1300004G	unkempt	Unkl
8487	Gnptg	0.598	1.055	0.631	0.002	0.655	0.027	8.188	7.447	7.525	214505	Gnptg	chr17	-	NM_1725	P20018	protein_cc	6430527N	N-acetylgl	Gnptg
8488	Tsr3	1.259	1.506	1.895	0.290	0.033	0.046	8.183	8.515	9.105	68327	Tsr3	chr17	+	NM_0266	P10907	protein_cc	0610007P	TSR3 20S	Tsr3
8489	Baiap3	1.217	1.558	1.896	0.315	0.020	0.056	8.124	8.408	9.048	545192	Baiap3	chr17	-	NM_0011	P7189	protein_cc	Bap3| Gm	BAI1-asso	Baiap3
8490	Ube2i	1.199	1.007	1.207	0.040	0.938	0.250	8.270	8.532	8.542	22196	Ube2i	chr17	-	NM_0011	P23192	protein_cc	5830467E	ubiquitin-	Ube2i
8491	Gm17801	0.507	1.411	0.715	0.183	0.190	0.654	1.002	0.022	0.519	10027222	Gm17801	chr17	+	NR_02745	.	lncRNA	.	predicted	Gm17801
8492	Prss34	3.925	0.581	2.281	0.112	0.373	0.423	0.001	1.974	1.191	328780	Prss34	chr17	+	NM_1783	P7528	protein_cc	C130091P	protease,	Prss34
8493	Prss28	1.000	1.000	1.000	1.000	1.000	1.000	0.000	0.000	0.000	114661	Prss28	chr17	+	NM_0532	P7902	protein_cc	Isp1| mIsp	protease,	Prss28
8494	Prss29	1.000	1.000	1.000	1.000	1.000	1.000	0.000	0.000	0.000	114662	Prss29	chr17	+	NM_0532	P11055	protein_cc	D19363| I	protease,	Prss29
8495	Tpsab1	1.572	0.636	1.000	0.304	1.000	0.423	0.000	0.653	0.000	10050389	Tpsab1	chr17	-	NR_03352	.	misc_RNA	Mcp-7| M	tryptase a	Tpsab1
8496	Tpsb2	1.005	1.439	1.446	0.000	0.182	0.423	0.000	0.008	0.532	17229	Tpsb2	chr17	+	NM_0107	P17185	protein_cc	AV011504	tryptase b	Tpsb2
8497	Tpsg1	1.000	1.000	1.000	1.000	1.000	1.000	0.000	0.000	0.000	26945	Tpsg1	chr17	+	NM_0120	P3839	protein_cc	Prss31| Tr	tryptase g	Tpsg1
8498	Cacna1h	1.572	0.636	1.000	0.304	1.000	0.423	0.000	0.653	0.000	58226	Cacna1h	chr17	-	NM_0214	P4067	protein_cc	alpha13.2	calcium ch	Cacna1h
8499	Tekt4	1.010	1.885	1.904	0.000	0.182	0.423	0.000	0.015	0.929	71840	Tekt4	chr17	+	NM_0279	P2776	protein_cc	1700010L	tektin 4	Tekt4
8500	Sstr5	1.000	1.000	1.000	1.000	1.000	1.000	0.000	0.000	0.000	20609	Sstr5	chr17	-	NM_0114	P24588	protein_cc	Smstr5| s	somatosta	Sstr5
8501	Sox8	1.000	1.000	1.000	1.000	1.000	1.000	0.000	0.000	0.000	20681	Sox8	chr17	-	NM_0114	P5504	protein_cc	.	SRY (sex d	Sox8
8502	2810468N	1.202	1.894	2.276	0.645	0.056	0.071	0.427	0.692	1.614	72834	2810468N	chr17	+	NR_04517	.	lncRNA	.	RIKEN cDN	2810468N
8503	Lmf1	0.458	0.986	0.452	0.001	0.940	0.012	8.271	7.144	7.124	76483	Lmf1	chr17	-	NM_0296	P4496	protein_cc	2400010G	lipase mat	Lmf1
8504	Gng13	0.730	0.885	0.646	0.540	0.758	0.602	1.135	0.681	0.505	64337	Gng13	chr17	-	NM_0224	P20715	protein_cc	1500031D	guanine n	Gng13
8505	Chtf18	0.535	0.874	0.468	0.352	0.735	0.517	1.594	0.692	0.498	214901	Chtf18	chr17	-	NM_1454	P17324	protein_cc	6030457N	CTF18, ch	Chtf18
8506	Rpusd1	2.069	0.617	1.276	0.000	0.003	0.305	3.667	4.716	4.018	106707	Rpusd1	chr17	+	NM_0280	P22629	protein_cc	2310051D	RNA pseu	Rpusd1
8507	Mslnl	1.000	1.000	1.000	1.000	1.000	1.000	0.000	0.000	0.000	328783	Mslnl	chr17	+	NM_1778	P2298	protein_cc	4732467B	mesotheli	Mslnl
8508	Msln	0.539	2.004	1.081	0.363	0.076	0.923	2.690	1.799	2.802	56047	Msln	chr17	-	NM_0188	P24164	protein_cc	MPF	mesotheli	Msln
8509	Narfl	1.022	1.210	1.236	0.800	0.030	0.104	7.064	7.095	7.370	67563	Narfl	chr17	+	NM_0262	P7967	protein_cc	9030612I2	nuclear pr	Narfl
8510	Haghl	0.756	2.068	1.563	0.285	0.015	0.036	5.090	4.687	5.735	68977	Haghl	chr17	-	NM_0012	P8786	protein_cc	1500017E	hydroxyac	Haghl
8511	Ccdc78	0.630	2.540	1.600	0.056	0.001	0.067	4.390	3.723	5.068	381077	Ccdc78	chr17	+	NM_0011	P24876	protein_cc	Gm938| G	coiled-coil	Ccdc78
8512	Fam173a	0.556	1.211	0.673	0.018	0.279	0.143	8.491	7.644	7.920	214917	Fam173a	chr17	-	NM_0012	P2899	protein_cc	.	family wit	Fam173a
8513	Metrn	0.238	2.830	0.673	0.001	0.001	0.132	4.785	2.714	4.214	70083	Metrn	chr17	-	NM_1337	P14323	protein_cc	1810034B	meteorin,	Metrn
8514	Fbxl16	0.471	2.570	1.211	0.186	0.184	0.841	1.128	0.043	1.404	214931	Fbxl16	chr17	+	NM_0011	P6226	protein_cc	BC042620	F-box and	Fbxl16
8515	Wdr24	0.896	0.958	0.858	0.010	0.600	0.304	6.415	6.256	6.194	268933	Wdr24	chr17	+	NM_1737	P3485	protein_cc	BC037651	WD repea	Wdr24
8516	Jmjd8	0.530	1.512	0.801	0.001	0.060	0.401	9.610	8.694	9.291	72106	Jmjd8	chr17	+	NM_0281	P19769	protein_cc	2610003J(	jumonji dc	Jmjd8
8517	Stub1	0.591	1.566	0.925	0.005	0.069	0.759	9.227	8.468	9.114	56424	Stub1	chr17	-	NM_0197	P8694	protein_cc	0610033N	STIP1 hom	Stub1
8518	Rhbdl1	1.103	0.455	0.502	0.806	0.063	0.442	2.057	2.198	1.063	214951	Rhbdl1	chr17	-	NM_1448	P21130	protein_cc	Rhbdl	rhomboid	Rhbdl1
8519	Rhot2	0.503	1.117	0.562	0.008	0.688	0.256	7.359	6.368	6.528	214952	Rhot2	chr17	-	NM_1459	P14061	protein_cc	Arht2| Mi	ras homol	Rhot2
8520	Wdr90	0.247	1.573	0.389	0.042	0.521	0.207	4.835	2.818	3.472	106618	Wdr90	chr17	-	NM_0011	P23664	protein_cc	3230401M	WD repea	Wdr90
8521	Fam195a	0.176	2.060	0.362	0.000	0.241	0.102	8.135	5.626	6.668	68241	Fam195a	chr17	-	NM_0266	P4188	protein_cc	9530058B	family wit	Fam195a
8522	0610011F	0.468	1.381	0.647	0.006	0.044	0.169	10.067	8.972	9.438	68347	0610011F	chr17	+	NM_0266	P11463	protein_cc	.		0610011F
8523	Wfikkn1	1.363	0.464	0.633	0.037	0.069	0.511	2.479	2.925	1.818	215001	Wfikkn1	chr17	-	NM_0011	P24496	protein_cc	Gasp2| W	WAP, FS, I	Wfikkn1
8524	Rab40c	1.425	0.947	1.349	0.009	0.505	0.130	6.754	7.265	7.186	224624	Rab40c	chr17	-	NM_1391	P19856	protein_cc	RAR3	Rab40C, m	Rab40c
8525	Gm10012	0.820	1.064	0.872	0.240	0.685	0.273	11.556	11.270	11.359	10004316	Gm10012	chr17	-	NR_02804	.	misc_RNA	ENSMUSG	predicted	Gm10012
8526	Pigq	0.551	1.161	0.640	0.000	0.419	0.168	8.309	7.450	7.666	14755	Pigq	chr17	-	NM_0012	P24988	protein_cc	Gpi1| Gpi	phosphati	Pigq
8527	Nhlrc4	1.000	1.000	1.000	1.000	1.000	1.000	0.000	0.000	0.000	621239	Nhlrc4	chr17	-	NM_0010	P1527	protein_cc	F430201B	NHL repea	Nhlrc4
8528	Capn15	1.077	0.820	0.883	0.624	0.291	0.587	5.113	5.220	4.934	50817	Capn15	chr17	-	NM_0158	P2623	protein_cc	Solh	calpain 15	Capn15
8529	Rab11fip3	0.533	0.772	0.411	0.052	0.581	0.082	6.319	5.410	5.037	215445	Rab11fip3	chr17	-	NM_1531	P25045	protein_cc	Cart1| D0	RAB11 fan	Rab11fip3
8530	Decr2	0.144	1.384	0.199	0.000	0.149	0.009	12.256	9.462	9.931	26378	Decr2	chr17	-	NM_0119	P7104	protein_cc	.	2-4-dieno	Decr2
8531	Nme4	0.131	2.475	0.325	0.001	0.180	0.118	5.514	2.585	3.892	56520	Nme4	chr17	-	NM_0197	P14317	protein_cc	2610027N	NME/NM2	Nme4
8532	Tmem8	0.848	1.126	0.955	0.171	0.513	0.886	7.673	7.436	7.607	60455	Tmem8	chr17	+	NM_0217	P16291	protein_cc	AW49009	transmem	Tmem8
8533	Mrpl28	0.518	1.650	0.854	0.000	0.013	0.383	8.254	7.304	8.027	68611	Mrpl28	chr17	+	NM_0242	P16625	protein_cc	1110015G	mitochonc	Mrpl28
8534	Axin1	1.401	0.927	1.299	0.004	0.537	0.344	7.280	7.767	7.658	12005	Axin1	chr17	+	NM_0011	P232	protein_cc	AI316800|	axin 1	Axin1
8535	Pdia2	1.000	1.000	1.000	1.000	1.000	1.000	0.000	0.000	0.000	69191	Pdia2	chr17	-	NM_0010	P24783	protein_cc	1810041F	protein di	Pdia2
8536	Arhgdig	1.000	1.000	1.000	1.000	1.000	1.000	0.000	0.000	0.000	14570	Arhgdig	chr17	-	NM_0081	P6344	protein_cc	Gdi5| Rho	Rho GDP c	Arhgdig
8537	Rgs11	1.000	1.000	1.000	1.000	1.000	1.000	0.000	0.000	0.000	50782	Rgs11	chr17	+	NM_0010	P985	protein_cc	C78048	regulator	Rgs11
8538	Itfg3	0.244	1.395	0.341	0.000	0.116	0.027	8.355	6.323	6.803	.	Itfg3	chr17	-	NM_0012	P15866	.	.	.	Itfg3
8539	Luc7l	1.456	0.484	0.705	0.008	0.001	0.095	7.504	8.046	7.000	66978	Luc7l	chr17	+	NM_0258	P604	protein_cc	1810045C	Luc7-like	Luc7l
8540	Neurl1b	0.152	2.606	0.397	0.001	0.023	0.036	4.978	2.261	3.643	240055	Neurl1b	chr17	+	NM_0010	P19169	protein_cc	C230078M	neuralized	Neurl1b
8541	Dusp1	1.126	0.721	0.812	0.531	0.114	0.418	10.209	10.381	9.909	19252	Dusp1	chr17	-	NM_0136	P12140	protein_cc	3CH134| E	dual speci	Dusp1

8542	Ergic1	0.408	1.180	0.482	0.001	0.424	0.080	9.379	8.087	8.326	67458	Ergic1	chr17	+	NM_0261	P26103	protein_cc	1200007D	endoplasn	Ergic1
8543	Atp6v0e	2.831	0.845	2.393	0.000	0.001	0.002	9.393	10.894	10.651	11974	Atp6v0e	chr17	+	NM_0252	P16901	protein_cc	Atp6k | At	ATPase, H	Atp6v0e
8544	Crebrf	0.555	0.759	0.421	0.000	0.003	0.006	9.755	8.905	8.508	77128	Crebrf	chr17	+	NM_0298	P17674	protein_cc	A930001N	CREB3 reg	Crebrf
8545	Bnip1	0.822	1.455	1.195	0.006	0.012	0.308	6.484	6.200	6.741	224630	Bnip1	chr17	+	NM_1721	P2527	protein_cc	2010005N	BCL2/ader	Bnip1
8546	Nkx2-5	1.000	1.000	1.000	1.000	1.000	1.000	0.000	0.000	0.000	18091	Nkx2-5	chr17	-	NM_0087	P8850	protein_cc	Csx | Nkx-2	NK2 home	Nkx2-5
8547	Kifc5b	3.924	0.553	2.171	0.112	0.299	0.145	0.001	1.973	1.120	16580	Kifc5b	chr17	+	NM_0531	P15396	protein_cc	Hset | Kifc	kinesin far	Kifc5b
8548	Gm27740	1.000	1.000	1.000	1.000	1.000	1.000	0.000	0.000	0.000	10479613	Gm27740	chr17	+	NR_12856	.	miRNA	Mir6967-2	predicted	Gm27740
8549	Gm27775	1.000	1.000	1.000	1.000	1.000	1.000	0.000	0.000	0.000	10246621	Gm27775	chr17	+	NR_10593	.	miRNA	Mir6967 |	predicted	Gm27775
8550	Phf1	1.235	1.028	1.269	0.423	0.899	0.382	5.113	5.418	5.457	21652	Phf1	chr17	+	NR_12615	.	protein_cc	AW55721	PHD finge	Phf1
8551	Cuta	0.619	1.195	0.739	0.060	0.347	0.325	8.420	7.727	7.984	67675	Cuta	chr17	-	NM_0269	P15409	protein_cc	0610039D	cutA dival	Cuta
8552	Syngap1	1.005	1.405	1.412	0.000	0.182	0.423	0.000	0.008	0.498	240057	Syngap1	chr17	+	NM_0012	P8879	protein_cc	Gm1963 |	synaptic R	Syngap1
8553	Zbtb9	2.044	1.109	2.267	0.058	0.588	0.025	5.182	6.213	6.363	474156	Zbtb9	chr17	+	NM_0010	P556	protein_cc	3930402F	zinc finger	Zbtb9
8554	Ggnbp1	0.289	1.555	0.449	0.012	0.043	0.172	5.136	3.345	3.982	70772	Ggnbp1	chr17	+	NM_0012	P15737	protein_cc	0610031G	gametoge	Ggnbp1
8555	Bak1	1.092	1.052	1.149	0.194	0.650	0.550	7.721	7.848	7.921	12018	Bak1	chr17	-	NM_0075	P15094	protein_cc	Bak | N-Ba	BCL2-anta	Bak1
8556	Itpr3	0.238	1.629	0.388	0.030	0.016	0.252	3.279	1.207	1.911	16440	Itpr3	chr17	+	NM_0805	P10126	protein_cc	IP3R 3 | Ip	inositol 1,	Itpr3
8557	Uqcc2	1.071	1.024	1.097	0.478	0.721	0.502	8.224	8.322	8.357	67267	Uqcc2	chr17	-	NM_0260	P22226	protein_cc	2900010N	ubiquinol-	Uqcc2
8558	Ip6k3	1.000	1.000	1.000	1.000	1.000	1.000	0.000	0.000	0.000	271424	Ip6k3	chr17	-	NM_1730	P24188	protein_cc	D830007E	inositol he	Ip6k3
8559	Lemd2	1.233	1.146	1.413	0.217	0.268	0.085	7.036	7.338	7.534	224640	Lemd2	chr17	-	NM_1460	P16066	protein_cc	BC026588	LEM doma	Lemd2
8560	9630028IC	1.000	1.000	1.000	1.000	1.000	1.000	0.000	0.000	0.000	319720	9630028IC	chr17	-	NR_13111	.	lncRNA	.	RIKEN cDN	9630028I0
8561	Grm4	1.152	0.629	0.725	0.722	1.000	0.422	0.465	0.669	0.000	268934	Grm4	chr17	-	NM_0010	P8623	protein_cc	Gprc1d | n	glutamate	Grm4
8562	Hmga1	1.873	0.914	1.711	0.187	0.783	0.142	5.900	6.805	6.675	15361	Hmga1	chr17	+	NM_0011	P584	protein_cc	AL023995	high mobi	Hmga1
8563	Hmga1-rs1	1.852	0.927	1.717	0.184	0.812	0.137	5.900	6.789	6.680	111241	Hmga1-rs1	chr17	+	NM_0011	P12686	protein_cc	ENSMUSG	high mobi	Hmga1-rs1
8564	AI413582	1.170	0.788	0.923	0.244	0.016	0.754	6.642	6.869	6.526	106672	AI413582	chr17	-	NM_0010	P6288	protein_cc	AI854251	expressed	AI413582
8565	Nudt3	1.439	0.847	1.220	0.005	0.120	0.267	10.299	10.825	10.586	56409	Nudt3	chr17	-	NM_0012	P13505	protein_cc	1110011B	nudix (nuc	Nudt3
8566	Rps10	1.207	1.910	2.306	0.278	0.013	0.063	9.373	9.645	10.578	67097	Rps10	chr17	-	NM_0259	P13352	protein_cc	2210402A	ribosomal	Rps10
8567	Pacsin1	5.648	0.457	2.580	0.002	0.054	0.442	0.722	3.220	2.090	23969	Pacsin1	chr17	+	NM_0118	P5785	protein_cc	A830061D	protein kir	Pacsin1
8568	Spdef	1.005	1.439	1.446	0.000	0.182	0.423	0.000	0.008	0.532	30051	Spdef	chr17	-	NM_0138	P26084	protein_cc	PDEF | Pse	SAM point	Spdef
8569	D17Wsu9	0.530	1.202	0.637	0.004	0.078	0.081	11.629	10.714	10.979	224647	D17Wsu9	chr17	-	NM_0010	P18779	protein_cc	AU020189	DNA segm	D17Wsu92
8570	Snrpc	1.496	1.020	1.526	0.002	0.474	0.026	6.546	7.128	7.156	20630	Snrpc	chr17	+	NM_0114	P12919	protein_cc	Snrp1c | U	U1 small r	Snrpc
8571	Uhrf1bp1	1.292	1.293	1.671	0.048	0.022	0.015	6.047	6.417	6.788	224648	Uhrf1bp1	chr17	+	NM_0010	P24902	protein_cc	1110020K	UHRF1 (IC	Uhrf1bp1
8572	Taf11	1.167	1.221	1.424	0.036	0.070	0.157	7.008	7.230	7.518	68776	Taf11	chr17	-	NM_0268	P7092	protein_cc	1110038O	TATA-box	Taf11
8573	Anks1	1.123	0.845	0.949	0.159	0.082	0.779	6.648	6.815	6.573	224650	Anks1	chr17	+	NM_0012	P26565	protein_cc	Anks1a | n	ankyrin re	Anks1
8574	Tcp11	1.000	1.000	1.000	1.000	1.000	1.000	0.000	0.000	0.000	21463	Tcp11	chr17	-	NM_0136	P26411	protein_cc	D17Ken1 |	t-complex	Tcp11
8575	Scube3	1.000	1.000	1.000	1.000	1.000	1.000	0.000	0.000	0.000	268935	Scube3	chr17	+	NM_0010	P103	protein_cc	CEGF3 | D(	signal pep	Scube3
8576	Zfp523	0.563	1.805	1.017	0.029	0.020	0.929	7.067	6.239	7.091	224656	Zfp523	chr17	+	NM_1726	P23409	protein_cc	2810027J(	zinc finger	Zfp523
8577	Def6	0.904	2.282	2.063	0.854	0.222	0.555	2.175	2.030	3.220	23853	Def6	chr17	+	NM_0271	P8113	protein_cc	2410003F(	differentia	Def6
8578	Ppard	1.973	0.425	0.839	0.021	0.001	0.728	4.639	5.619	4.386	19015	Ppard	chr17	+	NM_0111	P6126	protein_cc	Nr1c2 | NU	peroxisom	Ppard
8579	1810013A	1.000	1.000	1.000	1.000	1.000	1.000	0.000	0.000	0.000	69050	1810013A	chr17	+	NR_04542	.	lncRNA	.	RIKEN cDN	1810013A
8580	Fance	0.491	1.599	0.786	0.000	0.000	0.031	6.908	5.883	6.560	72775	Fance	chr17	+	NM_0011	P5269	protein_cc	2810451D	Fanconi ar	Fance
8581	Rpl10a	1.106	1.154	1.276	0.148	0.034	0.078	10.643	10.788	10.994	19896	Rpl10a	chr17	+	NM_0112	P19601	protein_cc	CsA-19 | N	ribosomal	Rpl10a
8582	Tead3	1.206	0.741	0.893	0.617	0.195	0.889	5.595	5.865	5.432	21678	Tead3	chr17	-	NM_0012	P14823	protein_cc	DTEF-1 | E	TEA doma	Tead3
8583	Tulp1	1.000	1.000	1.000	1.000	1.000	1.000	0.000	0.000	0.000	22157	Tulp1	chr17	-	NM_0214	P24854	protein_cc	Tulp1l	tubby like	Tulp1
8584	Fkbp5	3.446	0.806	2.778	0.001	0.182	0.068	8.688	10.473	10.163	14229	Fkbp5	chr17	-	NM_0102	P15703	protein_cc	D17Ertd59	FK506 bin	Fkbp5
8585	Armc12	1.010	1.821	1.840	0.000	0.000	0.002	0.000	0.015	0.880	67645	Armc12	chr17	+	NM_0262	P14620	protein_cc	4930511I1	armadillo	Armc12
8586	Clpsl2	1.000	1.000	1.000	1.000	1.000	1.000	0.000	0.000	0.000	328788	Clpsl2	chr17	-	NM_0010	P17630	protein_cc	Gm749	colipase-li	Clpsl2
8587	Clps	1.000	1.000	1.000	1.000	1.000	1.000	0.000	0.000	0.000	109791	Clps	chr17	-	NM_0254	P22941	protein_cc	2200003J(	colipase, p	Clps
8588	Lhfpl5	0.701	6.678	4.681	0.218	0.059	0.281	0.588	0.075	2.815	328789	Lhfpl5	chr17	+	NM_0265	P3582	protein_cc	93301790	lipoma HN	Lhfpl5
8589	Srpk1	1.782	0.625	1.115	0.002	0.009	0.678	7.408	8.241	7.564	20815	Srpk1	chr17	-	NM_0167	P349	protein_cc	AU01796C	serine/arg	Srpk1
8590	Slc26a8	1.010	1.821	1.840	0.000	0.000	0.002	0.000	0.015	0.880	224661	Slc26a8	chr17	-	NM_0012	P12614	protein_cc	.	solute carr	Slc26a8
8591	Mapk14	0.808	1.172	0.946	0.007	0.027	0.466	9.072	8.764	8.992	26416	Mapk14	chr17	+	NM_0011	P17285	protein_cc	Crk1 | Csb	mitogen-a	Mapk14
8592	Mapk13	2.977	2.726	8.115	0.003	0.144	0.262	0.831	2.405	3.852	26415	Mapk13	chr17	+	NM_0119	P24346	protein_cc	SAPK4 | Se	mitogen-a	Mapk13
8593	Brpf3	1.392	1.234	1.718	0.007	0.003	0.030	7.084	7.561	7.864	268936	Brpf3	chr17	+	NM_0010	P2223	protein_cc	AI413466 |	bromodor	Brpf3
8594	Pnpla1	0.750	2.619	1.964	0.200	0.183	0.524	0.448	0.033	1.422	433091	Pnpla1	chr17	+	NM_0010	P132	protein_cc	.	patatin-lik	Pnpla1
8595	4930539E	0.526	4.045	2.127	0.108	0.001	0.053	1.676	0.748	2.764	207819	4930539E	chr17	+	NM_1724	P9733	protein_cc	9130014G	RIKEN cDN	4930539E0
8596	1700030A	1.000	1.000	1.000	1.000	1.000	1.000	0.000	0.000	0.000	74179	1700030A	chr17	-	NR_04545	.	lncRNA	.	RIKEN cDN	1700030A
8597	Pxt1	1.000	1.000	1.000	1.000	1.000	1.000	0.000	0.000	0.000	69307	Pxt1	chr17	-	NM_1533	P18787	protein_cc	1700001G	peroxisom	Pxt1
8598	Kctd20	1.550	0.989	1.533	0.044	0.950	0.231	6.506	7.138	7.121	66989	Kctd20	chr17	+	NM_0258	P9553	protein_cc	2410004N	potassium	Kctd20
8599	Stk38	0.864	0.977	0.844	0.019	0.861	0.464	8.948	8.736	8.703	106504	Stk38	chr17	-	NM_1341	P1686	protein_cc	5830476G	serine/thr	Stk38
8600	Srsf3	2.977	0.751	2.237	0.000	0.065	0.121	9.179	10.753	10.341	20383	Srsf3	chr17	+	NM_0136	P6132	protein_cc	AL024116	serine/arg	Srsf3
8601	Trp53cor1	5.284	1.958	10.345	0.021	0.107	0.117	1.091	3.493	4.462	10050426	Trp53cor1	chr17	-	NR_03646	.	lncRNA	Gm16197	tumor pro	Trp53cor1
8602	Cdkn1a	46.903	0.519	24.339	0.000	0.003	0.028	6.695	12.247	11.301	12575	Cdkn1a	chr17	+	NM_0011	P3467	protein_cc	CAP20 | CI	cyclin-dep	Cdkn1a
8603	Rab44	1.000	1.000	1.000	1.000	1.000	1.000	0.000	0.000	0.000	442827	Rab44	chr17	+	NM_0010	P26607	protein_cc	9830134C	RAB44, me	Rab44
8604	Cpne5	1.000	1.000	1.000	1.000	1.000	1.000	0.000	0.000	0.000	240058	Cpne5	chr17	-	NM_1531	P23933	protein_cc	A830083G	copine V	Cpne5
8605	Ppil1	0.875	1.025	0.896	0.011	0.873	0.660	7.339	7.146	7.181	68816	Ppil1	chr17	-	NM_0268	P19078	protein_cc	1110060O	peptidylpr	Ppil1
8606	BC004004	0.404	1.379	0.556	0.002	0.070	0.042	9.532	8.222	8.686	80748	BC004004	chr17	+	NM_0305	P9383	protein_cc	2400006G	cDNA seq	BC004004
8607	Pi16	0.452	5.243	2.367	0.038	0.121	0.488	1.218	0.071	2.461	74116	Pi16	chr17	+	NM_0237	P24404	protein_cc	1200009H	peptidase	Pi16
8608	Mtch1	0.433	1.126	0.488	0.005	0.146	0.099	9.793	8.587	8.758	56462	Mtch1	chr17	-	NM_0198	P23692	protein_cc	2310034O	mitochonc	Mtch1
8609	Fgd2	0.649	0.332	0.215	0.451	0.347	0.110	4.199	3.575	1.983	26382	Fgd2	chr17	+	NM_0137	P2436	protein_cc	Tcd-2 | Tcc	FYVE, Rho	Fgd2
8610	Pim1	4.433	0.516	2.287	0.000	0.001	0.064	8.220	10.369	9.414	18712	Pim1	chr17	+	NM_0088	P6961	protein_cc	Pim-1	proviral in	Pim1
8611	Tmem217	1.000	1.000	1.000	1.000	1.000	1.000	0.000	0.000	0.000	71138	Tmem217	chr17	-	NM_0011	P20232	protein_cc	4933413N	transmem	Tmem217
8612	Tbc1d22b	1.821	0.576	1.048	0.001	0.069	0.936	2.837	3.702	2.905	381085	Tbc1d22b	chr17	+	NM_1986	P5547	protein_cc	BC045600	TBC1 dom	Tbc1d22b
8613	Tbc1d22b	2.134	0.469	1.000	0.305	1.000	0.423	0.000	1.094	0.000	74092	Tbc1d22b	chr17	+	NR_04544	.	lncRNA	0610038L(	TBC1 dom	Tbc1d22bc
8614	Rnf8	1.009	1.122	1.131	0.905	0.471	0.634	7.444	7.456	7.622	58230	Rnf8	chr17	+	NM_0214	P13984	protein_cc	3830404E	ring finger	Rnf8
8615	Rnf8-cmtr	1.765	0.764	1.348	0.025	0.173	0.246	8.756	9.576	9.187	10601425	Rnf8-cmtr	chr17	+	NR_13231	.	lncRNA	.	Rnf8-cmtr	Rnf8-cmtr
8616	Cmtr1	2.029	0.366	0.742	0.405	1.000	0.422	0.431	1.451	0.000	74157	Cmtr1	chr17	+	NM_0287	P23059	protein_cc	1300018I(	cap methy	Cmtr1
8617	Ccdc167	1.990	0.996	1.982	0.018	0.980	0.058	7.440	8.433	8.427	68597	Ccdc167	chr17	-	NM_0011	P18782	protein_cc	1110021J(	coiled-coil	Ccdc167
8618	Mdga1	4.020	0.115	0.462	0.139	1.000	0.018	1.114	3.121	0.000	74762	Mdga1	chr17	-	NM_0010	P15635	protein_cc	1200011(	MAM dom	Mdga1
8619	Zfand3	0.913	0.962	0.878	0.708	0.880	0.401	9.442	9.310	9.254	21769	Zfand3	chr17	+	NM_1489	P20140	protein_cc	AW53921	zinc finger	Zfand3
8620	Btbd9	1.241	1.254	1.556	0.162	0.120	0.049	7.579	7.891	8.217	224671	Btbd9	chr17	-	NM_1726	P13803	protein_cc	1700023F	BTB (POZ)	Btbd9
8621	Gm6402	1.204	1.211	1.459	0.105	0.180	0.267	6.323	6.591	6.867	623169	Gm6402	chr17	-	NR_03068	.	misc_RNA	EG623169	predicted	Gm6402
8622	Glo1	0.288	1.346	0.388	0.000	0.036	0.004	11.578	9.783	10.211	109801	Glo1	chr17	-	NM_0011	P9230	protein_cc	0610009E	glyoxalase	Glo1
8623	1700097N	0.885	0.629	0.557	0.781	1.000	0.422	0.844	0.668	0.000	67522	1700097N	chr17	-	NR_04528	.	lncRNA	.	RIKEN cDN	1700097N
8624	Dnah8	0.294	0.974	0.286	0.181	1.000	0.423	1.806	0.038	0.000	13417	Dnah8	chr17	+	NM_0138	P15538	protein_cc	ATPase | D	dynein, ax	Dnah8
8625	Glp1r	1.000	1.000	1.000	1.000	1.000	1.000	0.000	0.000	0.000	14652	Glp1r	chr17	+	NM_0213	P18917	protein_cc	GLP-1R | G	glucagon-	Glp1r
8626	Umodl1	1.572	0.636	1.000	0.304	1.000	0.423	0.000	0.653	0.000	52020	Umodl1	chr17	+	NM_1774	P24498	protein_cc	D17Ertd48	uromodul	Umodl1
8627	Abcg1	0.853	0.503	0.429	0.358	0.068	0.204	6.111	5.883	4.892	11307	Abcg1	chr17	+	NM_0095	P8184	protein_cc	A830083G	AW ATP-bindi	Abcg1
8628	Tff3	0.202	9.277	1.878	0.003	0.004	0.210	3.084	0.779	3.993	21786	Tff3	chr17	-	NM_0115	P4258	protein_cc	ITF | mITF	trefoil fact	Tff3
8629	Tff2	0.125	3.347	0.417	0.001	0.187	0.233	3.102	0.098	1.841	21785	Tff2	chr17	-	NM_0093	P24891	protein_cc	mSP | SP	trefoil fact	Tff2
8630	LOC10263	0.038	2.262	0.086	0.000	0.306	0.015	5.520	0.801	1.979	10263175	LOC10263	chr17	+	NR_11050	.	lncRNA	.	uncharact	LOC10263
8631	Tff1	0.311	0.977	0.304	0.007	1.000	0.087	1.717	0.033	0.000	21784	Tff1	chr17	-	NM_0093	P5620	protein_cc	Bcei | PS2	trefoil fact	Tff1
8632	Tmprss3	1.000	1.000	1.000	1.000	1.000	1.000	0.000	0.000	0.000	140765	Tmprss3	chr17	-	NM_0011	P7424	protein_cc	.	transmem	Tmprss3

8633	Ubash3a	1.589	1.141	1.813	0.298	0.765	0.423	0.000	0.669	0.859	328795	Ubash3a	chr17	+	NM_1778	P19271	protein_cc	5830413C	ubiquitin a	Ubash3a
8634	Rsph1	1.612	1.438	2.317	0.223	0.160	0.254	1.326	2.015	2.539	22092	Rsph1	chr17	-	NM_0252	P26254	protein_cc	MCA| Tsg	radial spo	Rsph1
8635	Slc37a1	16.390	0.646	10.590	0.013	0.190	0.017	3.524	7.558	6.928	224674	Slc37a1	chr17	+	NM_1530	P3665	protein_cc	G3PP	solute car	Slc37a1
8636	Pde9a	0.046	4.180	0.193	0.000	0.009	0.025	8.291	3.858	5.922	18585	Pde9a	chr17	+	NM_0088	P20145	protein_cc	PDE9A1	phosphod	Pde9a
8637	Wdr4	1.771	1.195	2.117	0.006	0.011	0.032	5.551	6.376	6.633	57773	Wdr4	chr17	-	NM_0213	P9073	protein_cc	AI415180|	WD repea	Wdr4
8638	Ndufv3	0.486	1.051	0.511	0.003	0.745	0.015	11.666	10.625	10.697	78330	Ndufv3	chr17	+	NM_0300	P20291	protein_cc	1500032D	NADH deh	Ndufv3
8639	Pknox1	1.708	0.691	1.179	0.001	0.008	0.461	7.112	7.884	7.350	18771	Pknox1	chr17	+	NM_0166	P11328	protein_cc	D17Wsu7	Pbx/knott	Pknox1
8640	Cbs	0.356	0.730	0.260	0.000	0.007	0.001	12.317	10.826	10.372	12411	Cbs	chr17	-	NM_1448	P7890	protein_cc	AI047524|	cystathion	Cbs
8641	U2af1	1.332	0.870	1.159	0.096	0.393	0.640	8.712	9.126	8.925	108121	U2af1	chr17	-	NM_0241	P7607	protein_cc	2010107D	U2 small r	U2af1
8642	Cryaa	1.000	1.000	1.000	1.000	1.000	1.000	0.000	0.000	0.000	12954	Cryaa	chr17	+	NM_0012	P5043	protein_cc	Acry-1| Cr	crystallin,	Cryaa
8643	Sik1	0.633	0.723	0.458	0.050	0.052	0.148	6.939	6.280	5.812	17691	Sik1	chr17	-	NM_0108	P1126	protein_cc	Msk| Sik|	salt induci	Sik1
8644	Hsf2bp	2.783	0.934	2.599	0.019	0.874	0.423	0.001	1.478	1.379	74377	Hsf2bp	chr17	-	NM_0289	P5661	protein_cc	4932437G	heat shoc	Hsf2bp
8645	Rrp1b	1.752	0.920	1.612	0.007	0.558	0.189	6.604	7.413	7.293	72462	Rrp1b	chr17	+	NM_0282	P22859	protein_cc	2600005C	ribosomal	Rrp1b
8646	Pdxk-ps	0.560	1.412	0.791	0.184	0.190	0.721	0.857	0.021	0.519	435518	Pdxk-ps	chr17	-	NR_02731	.	misc_RNA	ENSMUSG	pyridoxal	Pdxk-ps
8647	Notch3	0.596	0.632	0.377	0.358	0.014	0.427	3.442	2.695	2.033	18131	Notch3	chr17	-	NM_0087	P18429	protein_cc	AW22901	notch 3	Notch3
8648	Ephx3	0.669	0.995	0.666	0.181	1.000	0.423	0.587	0.008	0.000	71932	Ephx3	chr17	-	NM_0010	P10013	protein_cc	2310063B	epoxide h	Ephx3
8649	Brd4	0.914	0.763	0.697	0.160	0.008	0.021	9.516	9.385	8.995	57261	Brd4	chr17	-	NM_0205	P92	protein_cc	Brd5| HUI	bromodor	Brd4
8650	Gm4432	0.629	0.662	0.417	0.079	0.303	0.230	2.686	2.018	1.424	10004343	Gm4432	chr17	+	NR_10232	.	misc_RNA	10004343	predicted	Gm4432
8651	Akap8	1.394	0.781	1.088	0.011	0.061	0.579	7.001	7.480	7.123	56399	Akap8	chr17	-	NM_0197	P25915	protein_cc	1200016A	A kinase (	Akap8
8652	Akap8l	1.259	0.655	0.824	0.067	0.014	0.265	6.405	6.737	6.127	54194	Akap8l	chr17	-	NM_0174	P9652	protein_cc	HAP95| N	A kinase (	Akap8l
8653	Wiz	1.182	0.906	1.071	0.308	0.546	0.602	6.866	7.106	6.964	22404	Wiz	chr17	-	NM_2124	P13715	protein_cc	.	widely-int	Wiz
8654	Rasal3	2.090	1.316	2.750	0.389	0.710	0.475	0.403	1.466	1.863	320484	Rasal3	chr17	-	NM_1787	P14590	protein_cc	A430107D	RAS prote	Rasal3
8655	A530088E	3.300	0.303	1.000	0.306	1.000	0.423	0.001	1.723	0.000	664702	A530088E	chr17	+	NR_02945	.	lncRNA	.	RIKEN cDN	A530088E
8656	Pglyrp2	0.468	1.017	0.476	0.001	0.880	0.024	8.996	7.902	7.926	57757	Pglyrp2	chr17	-	NM_0213	P14573	protein_cc	C730002N	peptidogl	Pglyrp2
8657	Cyp4f39	2.761	1.396	3.855	0.300	0.504	0.010	0.001	1.466	1.948	320997	Cyp4f39	chr17	+	NM_1773	P22417	protein_cc	4732474A	cytochron	Cyp4f39
8658	Cyp4f17	1.107	1.592	1.762	0.865	0.437	0.544	1.003	1.150	1.821	208285	Cyp4f17	chr17	+	NM_0011	P5753	protein_cc	EG208285	cytochron	Cyp4f17
8659	Cyp4f16	1.554	1.105	1.716	0.288	0.795	0.398	3.903	4.539	4.682	70101	Cyp4f16	chr17	+	NM_0244	P939	protein_cc	2310021J	cytochron	Cyp4f16
8660	Cyp4f37	0.491	2.621	1.288	0.159	0.024	0.463	2.195	1.170	2.560	677156	Cyp4f37	chr17	+	NM_0011	P1585	protein_cc	677156| G	cytochron	Cyp4f37
8661	Cyp4f40	0.439	0.985	0.432	0.002	1.000	0.051	1.210	0.021	0.000	631304	Cyp4f40	chr17	+	NM_0011	P22542	protein_cc	EG631304	cytochron	Cyp4f40
8662	Cyp4f15	0.047	0.493	0.023	0.000	0.018	0.004	10.458	6.055	5.035	106648	Cyp4f15	chr17	+	NM_1341	P7875	protein_cc	AI787289|	cytochron	Cyp4f15
8663	Zfp871	0.317	0.914	0.290	0.000	0.479	0.017	9.798	8.141	8.011	208292	Zfp871	chr17	-	NM_1724	P1750	protein_cc	4732483N	zinc finger	Zfp871
8664	Zfp811	17.815	0.893	15.915	0.006	0.631	0.035	0.002	4.157	3.995	240063	Zfp811	chr17	-	NM_0012	P26757	protein_cc	BC052046	zinc finger	Zfp811
8665	Zfp799	0.965	1.056	1.019	0.934	0.899	0.927	4.551	4.500	4.578	240064	Zfp799	chr17	-	NM_1773	P7098	protein_cc	60304901	zinc finger	Zfp799
8666	Zfp870	0.814	0.960	0.781	0.121	0.885	0.574	5.809	5.512	5.453	240066	Zfp870	chr17	-	NM_2072	P26885	protein_cc	AI173952	zinc finger	Zfp870
8667	Cyp4f14	0.324	0.667	0.216	0.000	0.186	0.019	11.095	9.467	8.882	64385	Cyp4f14	chr17	-	NM_0012	P10875	protein_cc	1300014O	cytochron	Cyp4f14
8668	Cyp4f13	0.432	1.312	0.567	0.004	0.228	0.026	8.548	7.338	7.731	170716	Cyp4f13	chr17	-	NM_1308	P10716	protein_cc	0610030I1	cytochron	Cyp4f13
8669	Cyp4f41-p	1.010	1.885	1.904	0.000	0.182	0.423	0.000	0.015	0.929	77875	Cyp4f41-p	chr17	-	NR_03358	.	misc_RNA	6030422N	cytochron	Cyp4f41-p
8670	Zfp472	0.900	0.930	0.837	0.583	0.726	0.041	5.954	5.803	5.697	224691	Zfp472	chr17	+	NM_1530	P19853	protein_cc	KRIM-1| K	zinc finger	Zfp472
8671	Zfp952	2.044	0.657	1.342	0.008	0.008	0.341	5.322	6.353	5.746	240067	Zfp952	chr17	+	NM_0010	P18279	protein_cc	BC057593	zinc finger	Zfp952
8672	Zfp763	0.427	1.074	0.459	0.051	0.883	0.059	5.179	3.952	4.055	73451	Zfp763	chr17	-	NM_0285	P2889	protein_cc	1700065O	zinc finger	Zfp763
8673	4921501E	1.000	1.000	1.000	1.000	1.000	1.000	0.000	0.000	0.000	74042	4921501E	chr17	-	NM_0010	P3346	protein_cc	PHF8	RIKEN cDN	4921501E
8674	Zfp563	0.392	1.698	0.665	0.018	0.202	0.192	5.065	3.713	4.477	240068	Zfp563	chr17	+	NM_0010	P19474	protein_cc	D10628| [	zinc finger	Zfp563
8675	Morc2b	2.134	0.469	1.000	0.305	1.000	0.423	0.000	1.094	0.000	240069	Morc2b	chr17	-	NM_1777	P15034	protein_cc	4932411A	microrchid	Morc2b
8676	Olfr55	1.000	1.000	1.000	1.000	1.000	1.000	0.000	0.000	0.000	10003885	Olfr55	chr17	+	NM_0109	P10446	protein_cc	IF6| MOR	olfactory	Olfr55
8677	Olfr239	1.000	1.000	1.000	1.000	1.000	1.000	0.000	0.000	0.000	10003886	Olfr239	chr17	+	NM_2071	P25150	protein_cc	ENSMUSG	olfactory	Olfr239
8678	Gm4461	1.000	1.000	1.000	1.000	1.000	1.000	0.000	0.000	0.000	10004347	Gm4461	chr17	-	NM_0011	P2045	protein_cc	10004347	predicted	Gm4461
8679	Zfp955a	0.591	1.009	0.597	0.057	0.977	0.148	6.560	5.803	5.815	77652	Zfp955a	chr17	-	NM_0299	P17804	protein_cc	AI842447|	zinc finger	Zfp955a
8680	Olfr63	1.000	1.000	1.000	1.000	1.000	1.000	0.000	0.000	0.000	258939	Olfr63	chr17	+	NM_1469	P1907	protein_cc	M4| MOR	olfactory	Olfr63
8681	Zfp955b	0.930	0.663	0.617	0.519	0.030	0.218	6.480	6.376	5.783	10004346	Zfp955b	chr17	-	NM_0011	P197	protein_cc	10004346	zinc finger	Zfp955b
8682	Zfp81	0.560	0.619	0.347	0.251	0.416	0.254	3.534	2.698	2.005	224694	Zfp81	chr17	-	NM_2075	P25856	protein_cc	AA409460	zinc finger	Zfp81
8683	Zfp101	0.311	1.123	0.349	0.008	0.746	0.104	5.263	3.578	3.746	22643	Zfp101	chr17	-	NM_0095	P13620	protein_cc	AI316498|	zinc finger	Zfp101
8684	Actl9	1.000	1.000	1.000	1.000	1.000	1.000	0.000	0.000	0.000	69481	Actl9	chr17	+	NM_1832	P15009	protein_cc	1700029I	actin-like	Actl9
8685	Adamts10	0.175	2.975	0.519	0.030	0.107	0.427	4.080	1.562	3.135	224697	Adamts10	chr17	+	NM_1726	P5437	protein_cc	94300060	a disinteg	Adamts10
8686	Myo1f	1.757	0.536	0.942	0.001	0.000	0.698	4.599	5.411	4.513	17916	Myo1f	chr17	+	NM_0532	P26743	protein_cc	C330006B	myosin IF	Myo1f
8687	Zfp414	0.560	2.055	1.150	0.018	0.008	0.383	4.746	3.909	4.948	328801	Zfp414	chr17	+	NR_03415	.	protein_cc	0610030H	zinc finger	Zfp414
8688	Pram1	8.357	0.822	6.868	0.080	0.665	0.028	0.995	4.059	3.775	378460	Pram1	chr17	+	NM_0010	P19773	protein_cc	AY665714	PML-RAR	Pram1
8689	Hnrnpm	1.514	0.764	1.157	0.076	0.162	0.250	10.032	10.630	10.242	76936	Hnrnpm	chr17	-	NM_0298	P7893	protein_cc	2610023N	heteroger	Hnrnpm
8690	March2	0.705	1.489	1.049	0.107	0.002	0.843	8.356	7.851	8.426	224703	March2	chr17	-	NR_04552	.	protein_cc	9530046H	membran	March2
8691	Rab11b	0.905	1.176	1.065	0.073	0.184	0.739	9.564	9.420	9.654	19326	Rab11b	chr17	-	NM_0089	P14764	protein_cc	A730055L	RAB11B, r	Rab11b
8692	Angptl4	1.273	2.029	2.582	0.000	0.021	0.112	9.216	9.563	10.584	57875	Angptl4	chr17	-	NM_0205	P3482	protein_cc	Arp4| Bk8	angiopoie	Angptl4
8693	Kank3	0.900	0.612	0.551	0.773	0.333	0.358	4.865	4.714	4.005	80880	Kank3	chr17	+	NM_0306	P9404	protein_cc	0610013D	KN motif a	Kank3
8694	Rps28	1.091	1.193	1.302	0.238	0.024	0.024	9.423	9.548	9.804	54127	Rps28	chr17	+	NM_0168	P23394	protein_cc	.	ribosomal	Rps28
8695	Ndufa7	0.742	1.162	0.862	0.015	0.088	0.136	10.953	10.523	10.739	66416	Ndufa7	chr17	+	NR_10351	.	protein_cc	14.5kDa|	NADH deh	Ndufa7
8696	Cd320	0.746	1.658	1.237	0.002	0.022	0.406	5.734	5.312	6.041	54219	Cd320	chr17	+	NM_0194	P24275	protein_cc	425018-1	CD320 ant	Cd320
8697	Kifc1	2.191	0.817	1.790	0.000	0.453	0.423	0.001	1.132	0.840	10050276	Kifc1	chr17	-	NM_0011	P4971	protein_cc	10004297	kinesin fa	Kifc1
8698	Platr17	1.814	0.306	0.555	0.092	0.000	0.548	1.326	2.185	0.476	474160	Platr17	chr17	+	NR_04047	.	lncRNA	.	pluripoter	Platr17
8699	BC051226	0.475	1.010	0.480	0.151	0.983	0.440	5.979	4.906	4.920	407803	BC051226	chr17	-	NR_04514	.	lncRNA	.	cDNA seq	BC051226
8700	Daxx	3.327	0.498	1.655	0.002	0.010	0.153	7.953	9.687	8.681	13163	Daxx	chr17	+	NM_0078	P4941	protein_cc	.	Fas death	Daxx
8701	Zbtb22	0.471	1.715	0.807	0.000	0.000	0.006	7.776	6.689	7.467	81630	Zbtb22	chr17	+	NM_0206	P9541	protein_cc	1110008J	zinc finger	Zbtb22
8702	Tapbp	2.991	0.589	1.761	0.011	0.051	0.019	10.388	11.969	11.204	21356	Tapbp	chr17	+	NM_0010	P12365	protein_cc	D17Wsu9	TAP bindir	Tapbp
8703	Rgl2	0.723	0.962	0.696	0.104	0.909	0.545	5.050	4.583	4.528	19732	Rgl2	chr17	+	NM_0090	P23792	protein_cc	KE1.5| Ral	ral guanin	Rgl2
8704	Pfdn6	0.746	1.596	1.191	0.010	0.030	0.450	8.599	8.177	8.851	14976	Pfdn6	chr17	+	NM_0103	P21082	protein_cc	H2-Ke2	prefoldin	Pfdn6
8705	Wdr46	0.902	1.187	1.071	0.191	0.258	0.737	6.684	6.535	6.782	57315	Wdr46	chr17	+	NM_0206	P23221	protein_cc	2310007I	WD repea	Wdr46
8706	B3galt4	1.023	3.033	3.101	0.000	0.054	0.236	0.000	0.033	1.633	54218	B3galt4	chr17	-	NM_0194	P20859	protein_cc	Gal-T2| G	UDP-Gal:b	B3galt4
8707	Rps18	1.102	1.362	1.500	0.104	0.006	0.047	10.840	10.980	11.426	20084	Rps18	chr17	-	NM_0112	P3275	protein_cc	H-2Ke3| H	ribosomal	Rps18
8708	Vps52	1.219	0.991	1.208	0.607	0.982	0.639	5.313	5.599	5.586	224705	Vps52	chr17	+	NM_1726	P12758	protein_cc	ARE1| D1	vacuolar p	Vps52
8709	H2-K2	1.223	0.947	1.158	0.244	0.560	0.649	5.606	5.897	5.818	630499	H2-K2	chr17	-	NR_00444	.	misc_RNA	EG630499	histocomp	H2-K2
8710	AA388235	0.157	1.272	0.200	0.000	0.238	0.019	2.743	0.072	0.418	433100	AA388235	chr17	+	NR_03330	.	lncRNA	.	expressed	AA388235
8711	H2-K1	1.588	0.810	1.286	0.011	0.115	0.216	13.646	14.314	14.009	14972	H2-K1	chr17	-	NM_0010	P23153	protein_cc	H-2K| H-2	histocomp	H2-K1
8712	Ring1	0.653	0.937	0.612	0.035	0.628	0.186	6.072	5.458	5.364	19763	Ring1	chr17	-	NM_0090	P15595	protein_cc	Ring1A	ring finger	Ring1
8713	H2-Ke6	0.554	1.206	0.668	0.000	0.004	0.032	9.473	8.620	8.890	14979	H2-Ke6	chr17	-	NM_0135	P17402	protein_cc	D17H6S11	H2-K regio	H2-Ke6
8714	Slc39a7	1.974	0.754	1.488	0.088	0.314	0.127	9.727	10.708	10.301	14977	Slc39a7	chr17	-	NM_0010	P21915	protein_cc	AA408174	solute car	Slc39a7
8715	Rxrb	0.736	1.174	0.864	0.142	0.284	0.564	7.754	7.311	7.543	20182	Rxrb	chr17	+	NM_0012	P7828	protein_cc	AL023085	retinoid X	Rxrb
8716	Col11a2	0.911	1.111	1.011	0.831	0.812	0.987	0.825	0.690	0.842	12815	Col11a2	chr17	+	NM_0099	P19403	protein_cc	.	collagen, t	Col11a2
8717	BC051537	0.552	0.990	0.547	0.182	1.000	0.423	0.871	0.015	0.000	414076	BC051537	chr17	-	NR_04618	.	lncRNA	.	cDNA seq	BC051537
8718	H2-Oa	0.675	0.626	0.423	0.291	1.000	0.167	1.242	0.676	0.000	15001	H2-Oa	chr17	+	NM_0082	P7315	protein_cc	H-2Oa	histocomp	H2-Oa
8719	Brd2	1.735	0.548	0.950	0.050	0.039	0.766	10.629	11.424	10.555	14312	Brd2	chr17	-	NM_0102	P25973	protein_cc	AW22894	bromodor	Brd2
8720	H2-DMa	0.753	0.907	0.683	0.156	0.660	0.073	5.284	4.875	4.734	14998	H2-DMa	chr17	+	NM_0103	P4147	protein_cc	H-2Ma| H	histocomp	H2-DMa
8721	H2-DMb2	1.376	0.510	0.702	0.665	0.422	0.637	1.003	1.464	0.493	15000	H2-DMb2	chr17	+	NM_0103	P11264	protein_cc	H-2Mb2|	histocomp	H2-DMb2
8722	H2-DMb1	1.257	0.634	0.797	0.694	0.451	0.766	5.557	5.887	5.231	14999	H2-DMb1	chr17	+	NM_0103	P23734	protein_cc	AI385589|	histocomp	H2-DMb1
8723	Psmb9	6.805	0.368	2.502	0.016	0.068	0.444	7.677	10.443	9.000	16912	Psmb9	chr17	-	NM_0135	P12549	protein_cc	Lmp-2| Ln	proteason	Psmb9

8724	Tap1	12.847	0.312	4.005	0.000	0.001	0.127	7.299	10.982	9.301	21354	Tap1	chr17	+	NM_0136	P4907	protein_cc	ABC17| Al transporte	Tap1
8725	Psmb8	6.573	0.728	4.789	0.000	0.014	0.001	8.244	10.961	10.504	16913	Psmb8	chr17	-	NM_0107	P8465	protein_cc	Lmp-7| Ln proteason	Psmb8
8726	Tap2	3.841	0.480	1.845	0.227	0.382	0.370	7.349	9.290	8.232	21355	Tap2	chr17	+	NM_0115	P23127	protein_cc	ABC18| Al transporte	Tap2
8727	H2-Ob	0.318	0.483	0.154	0.047	0.182	0.146	3.456	1.805	0.756	15002	H2-Ob	chr17	+	NM_0103	P7897	protein_cc	A-beta-2| histocomp	H2-Ob
8728	H2-Ab1	1.351	0.592	0.799	0.002	0.000	0.130	7.948	8.382	7.625	14961	H2-Ab1	chr17	+	NM_2071	P19411	protein_cc	Abeta| Al histocomp	H2-Ab1
8729	H2-Aa	0.861	0.548	0.471	0.235	0.017	0.058	8.785	8.569	7.701	14960	H2-Aa	chr17	-	NM_0103	P6848	protein_cc	Aalpha| H histocomp	H2-Aa
8730	H2-Eb1	0.913	0.510	0.465	0.282	0.005	0.081	7.793	7.662	6.689	14969	H2-Eb1	chr17	+	NM_0103	P8819	protein_cc	Eb| H-2Eb histocomp	H2-Eb1
8731	H2-Eb2	2.045	0.494	1.010	0.402	0.406	0.981	0.425	1.457	0.439	381091	H2-Eb2	chr17	+	NM_0010	P16546	protein_cc	A130038H histocomp	H2-Eb2
8732	H2-Ea-ps	1.000	1.000	1.000	1.000	1.000	1.000	0.000	0.000	0.000	10050440	H2-Ea-ps	chr17	-	NM_0103	P7182	protein_cc	. .	H2-Ea-ps
8733	Btnl2	1.000	1.000	1.000	1.000	1.000	1.000	0.000	0.000	0.000	547431	Btnl2	chr17	+	NM_0798	P20501	protein_cc	BTL-II| Btl butyrophi	Btnl2
8734	Btnl1	1.000	1.000	1.000	1.000	1.000	1.000	0.000	0.000	0.000	10003886	Btnl1	chr17	+	NM_0011	P11936	protein_cc	Btnl3| Gm butyrophi	Btnl1
8735	BC051142	0.513	2.627	1.347	0.187	0.047	0.667	1.003	0.039	1.432	407788	BC051142	chr17	+	NM_0011	P24724	protein_cc	NG8| Test cDNA seq	BC051142
8736	Btnl4	1.000	1.000	1.000	1.000	1.000	1.000	0.000	0.000	0.000	632126	Btnl4	chr17	+	NM_0307	P25388	protein_cc	Btn3a3| E butyrophi	Btnl4
8737	Btnl5-ps	1.000	1.000	1.000	1.000	1.000	1.000	0.000	0.000	0.000	81497	Btnl5-ps	chr17	-	NR_00405	.	misc_RNA	Btnl5| ENS butyrophi	Btnl5-ps
8738	Btnl6	1.000	1.000	1.000	1.000	1.000	1.000	0.000	0.000	0.000	624681	Btnl6	chr17	-	NM_0307	P3792	protein_cc	EG624681 butyrophi	Btnl6
8739	Notch4	2.000	2.078	4.157	0.179	0.115	0.189	3.615	4.615	5.671	18132	Notch4	chr17	+	NM_0109	P11162	protein_cc	Int-3| Int3 notch 4	Notch4
8740	Gpsm3	2.793	0.720	2.010	0.000	0.006	0.023	4.190	5.672	5.198	106512	Gpsm3	chr17	+	NM_1341	P10977	protein_cc	AA960287 G-protein	Gpsm3
8741	Pbx2	0.825	1.201	0.990	0.283	0.353	0.938	7.523	7.245	7.508	18515	Pbx2	chr17	+	NM_0174	P17090	protein_cc	AU043397 pre B cell	Pbx2
8742	Ager	1.011	0.729	0.736	0.933	0.095	0.070	1.990	2.005	1.548	11596	Ager	chr17	+	NR_07317	.	protein_cc	RAGE advanced	Ager
8743	Rnf5	0.315	1.711	0.538	0.000	0.122	0.117	8.775	7.107	7.882	54197	Rnf5	chr17	-	NM_0194	P5413	protein_cc	24101310 ring finger	Rnf5
8744	Agpat1	1.575	0.862	1.357	0.020	0.359	0.489	5.494	6.149	5.934	55979	Agpat1	chr17	+	NM_0011	P15771	protein_cc	1-AGP| 1- 1-acylglyc	Agpat1
8745	Egfl8	1.490	0.868	1.293	0.074	0.486	0.627	5.392	5.968	5.763	81701	Egfl8	chr17	-	NM_1529	P13109	protein_cc	Ng3 EGF-like d	Egfl8
8746	Ppt2	0.669	0.981	0.656	0.008	0.888	0.162	7.367	6.786	6.758	54397	Ppt2	chr17	-	NM_0194	P20979	protein_cc	0610007N palmitoyl-	Ppt2
8747	Prrt1	1.000	1.000	1.000	1.000	1.000	1.000	0.000	0.000	0.000	260297	Prrt1	chr17	+	NM_0308	P21932	protein_cc	Bat-4| Bat proline-ric	Prrt1
8748	Fkbpl	0.847	1.428	1.210	0.212	0.000	0.345	5.933	5.694	6.208	56299	Fkbpl	chr17	+	NM_0198	P19290	protein_cc	DIR1| NG7 FK506 bin	Fkbpl
8749	Atf6b	1.572	0.741	1.165	0.099	0.175	0.588	8.128	8.781	8.349	12915	Atf6b	chr17	+	NM_0174	P8034	protein_cc	AA617266 activating	Atf6b
8750	Tnxb	0.479	3.996	1.914	0.037	0.227	0.632	1.800	0.737	2.736	81877	Tnxb	chr17	+	NM_0311	P25740	protein_cc	Tn-mhc| T tenascin X	Tnxb
8751	C4b	0.745	0.943	0.703	0.002	0.570	0.117	11.977	11.552	11.468	12268	C4b	chr17	-	NM_0097	P16847	protein_cc	C4| Ss compleme	C4b
8752	Cyp21a1	1.000	1.000	1.000	1.000	1.000	1.000	0.000	0.000	0.000	13079	Cyp21a1	chr17	-	NM_0099	P15485	protein_cc	21-OH| 21 cytochrom	Cyp21a1
8753	C4a	0.934	0.858	0.801	0.530	0.370	0.382	9.129	9.029	8.809	625018	C4a	chr17	-	NM_0114	P22980	protein_cc	Slp| SlpA compleme	C4a
8754	Stk19	2.820	0.798	2.252	0.034	0.421	0.107	7.310	8.806	8.481	54402	Stk19	chr17	-	NM_0194	P19481	protein_cc	G11| RP1 serine/thr	Stk19
8755	Dxo	0.717	1.347	0.966	0.122	0.265	0.939	6.503	6.024	6.453	112403	Dxo	chr17	+	NM_0011	P1820	protein_cc	Dom3z| N decapping	Dxo
8756	Skiv2l	0.361	1.291	0.466	0.000	0.059	0.015	8.316	6.847	7.215	108077	Skiv2l	chr17	-	NM_0213	P25982	protein_cc	4930534J superkiller	Skiv2l
8757	Nelfe	0.745	1.258	0.937	0.074	0.396	0.882	7.707	7.282	7.613	27632	Nelfe	chr17	+	NM_1385	P20548	protein_cc	D17H6S45 negative e	Nelfe
8758	Cfb	1.505	0.785	1.182	0.000	0.001	0.125	13.213	13.803	13.454	14962	Cfb	chr17	-	NM_0081	P19419	protein_cc	AI195813| compleme	Cfb
8759	C2	0.853	0.864	0.738	0.005	0.229	0.201	9.127	8.899	8.688	12263	C2	chr17	-	NM_0134	P23520	protein_cc	. compleme	C2
8760	Zbtb12	0.971	0.782	0.759	0.533	0.564	0.705	5.328	5.286	4.930	193736	Zbtb12	chr17	+	NM_1988	P11827	protein_cc	Bat-9| Bat zinc finger	Zbtb12
8761	Ehmt2	0.693	1.479	1.025	0.014	0.144	0.940	7.422	6.893	7.458	110147	Ehmt2	chr17	+	NM_0012	P22725	protein_cc	Bat8| D17 euchroma	Ehmt2
8762	Slc44a4	0.262	6.865	1.798	0.185	0.036	0.545	2.028	0.096	2.875	70129	Slc44a4	chr17	+	NM_0235	P22617	protein_cc	2210409B solute car	Slc44a4
8763	Neu1	0.462	1.270	0.587	0.001	0.380	0.194	9.274	8.162	8.507	18010	Neu1	chr17	+	NM_0108	P20057	protein_cc	AA407268 neuramini	Neu1
8764	1110038B	2.439	1.332	3.249	0.001	0.184	0.103	7.289	8.576	8.989	68763	1110038B	chr17	-	NR_02794	.	lncRNA	0610009C RIKEN cDN	1110038B
8765	Snord52	1.606	1.393	2.237	0.297	0.527	0.423	0.000	0.684	1.162	10021742	Snord52	chr17	-	NR_02852	.	snoRNA	MBII-297 small nucl	Snord52
8766	Hspa1b	2.422	0.447	1.082	0.012	0.055	0.907	4.047	5.324	4.162	15511	Hspa1b	chr17	-	NM_0104	P5257	protein_cc	hsp68| Hs heat shoc	Hspa1b
8767	Hspa1a	1.381	0.767	1.060	0.266	0.594	0.954	3.026	3.492	3.110	193740	Hspa1a	chr17	-	NM_0104	P11034	protein_cc	hsp68| Hs heat shoc	Hspa1a
8768	Hspa1l	0.543	1.894	1.028	0.360	0.218	0.976	1.589	0.707	1.628	15482	Hspa1l	chr17	+	NM_0135	P18726	protein_cc	Hsc70t| M heat shoc	Hspa1l
8769	Lsm2	1.104	0.661	0.730	0.215	0.021	0.150	8.081	8.223	7.627	27756	Lsm2	chr17	+	NM_0012	P358	protein_cc	D17H6S56 LSM2 hom	Lsm2
8770	D17H6S56	41.306	0.590	24.356	0.000	0.009	0.003	6.107	11.476	10.714	110956	D17H6S56	chr17	-	NM_0330	P17835	protein_cc	G7e DNA segm	D17H6S56
8771	Vars	2.273	0.830	1.887	0.003	0.369	0.245	6.742	7.927	7.658	22321	Vars	chr17	+	NM_0116	P24411	protein_cc	Bat6| D17 valyl-tRNA	Vars
8772	Vwa7	0.943	0.830	0.783	0.915	0.810	0.765	2.632	2.548	2.278	27762	Vwa7	chr17	+	NM_1385	P22367	protein_cc	C6orf27| I von Willeb	Vwa7
8773	Sapcd1	1.932	1.037	2.003	0.306	0.955	0.596	1.055	2.005	2.057	78376	Sapcd1	chr17	-	NR_02825	.	protein_cc	2810436B suppresso	Sapcd1
8774	Msh5	1.244	0.634	0.789	0.759	0.593	0.676	1.439	1.754	1.097	17687	Msh5	chr17	-	NR_13064	.	protein_cc	Mut5 mutS hom	Msh5
8775	Clic1	3.388	1.096	3.713	0.000	0.602	0.094	5.924	7.684	7.816	114584	Clic1	chr17	+	NM_0334	P14766	protein_cc	Clcp| G6 chloride ir	Clic1
8776	Ddah2	0.888	0.792	0.704	0.489	0.447	0.555	5.435	5.263	4.928	51793	Ddah2	chr17	+	NM_0011	P4986	protein_cc	1110003M dimethyla	Ddah2
8777	G6b	1.099	0.642	0.705	0.763	0.036	0.641	1.003	1.139	0.500	106722	G6b	chr17	-	NM_0010	P1927	protein_cc	AU023871 immunore	G6b
8778	G6bos	1.000	1.000	1.000	1.000	1.000	1.000	0.000	0.000	0.000	114763	G6bos	chr17	-	NR_00146	.	lncRNA	G6b| NG3 G6b oppo	G6bos
8779	Ly6g6c	1.005	1.439	1.446	0.000	0.182	0.423	0.000	0.008	0.532	68468	Ly6g6c	chr17	+	NM_0234	P24394	protein_cc	1110003M lymphocy	Ly6g6c
8780	Ly6g6d	0.716	0.995	0.712	0.182	1.000	0.423	0.490	0.008	0.000	114654	Ly6g6d	chr17	-	NM_0334	P24737	protein_cc	A930024F lymphocy	Ly6g6d
8781	Ly6g6e	1.000	1.000	1.000	1.000	1.000	1.000	0.000	0.000	0.000	70274	Ly6g6e	chr17	+	NM_0273	P21359	protein_cc	2310011C lymphocy	Ly6g6e
8782	Ly6g6f	1.000	1.000	1.000	1.000	1.000	1.000	0.000	0.000	0.000	433099	Ly6g6f	chr17	-	NM_0011	P18819	protein_cc	CJ068215 lymphocy	Ly6g6f
8783	Abhd16a	1.117	0.863	0.964	0.132	0.109	0.835	8.821	8.980	8.768	193742	Abhd16a	chr17	+	NM_1785	P22114	protein_cc	AI326074| abhydrola	Abhd16a
8784	Ly6g5c	1.000	1.000	1.000	1.000	1.000	1.000	0.000	0.000	0.000	114652	Ly6g5c	chr17	+	NM_1489	P14102	protein_cc	G5c| NG3 lymphocy	Ly6g5c
8785	Ly6g5b	3.059	0.219	0.669	0.062	0.033	0.607	1.047	2.660	0.468	266614	Ly6g5b	chr17	-	NM_1489	P1987	protein_cc	. lymphocy	Ly6g5b
8786	Csnk2b	0.933	1.224	1.142	0.680	0.194	0.551	9.162	9.062	9.353	13001	Csnk2b	chr17	-	NM_0013	P13536	protein_cc	CK II beta casein kin	Csnk2b
8787	Gpank1	1.362	0.625	0.851	0.046	0.013	0.519	7.064	7.509	6.831	81845	Gpank1	chr17	+	NM_0011	P21961	protein_cc	Bat-4| Bat G patch do	Gpank1
8788	D17H6S53	1.561	0.919	1.435	0.005	0.295	0.023	5.445	6.088	5.967	114585	D17H6S53	chr17	-	NM_0334	P15040	protein_cc	G4| NG34 DNA segm	D17H6S53
8789	Apom	0.439	0.987	0.433	0.000	0.586	0.001	12.214	11.027	11.007	55938	Apom	chr17	-	NM_0188	P11915	protein_cc	1190010O apolipopro	Apom
8790	Bag6	0.950	0.778	0.739	0.465	0.000	0.117	8.901	8.827	8.464	224727	Bag6	chr17	+	NM_0571	P7696	protein_cc	2410045D BCL2-asso	Bag6
8791	Prrc2a	1.651	0.771	1.272	0.030	0.179	0.479	7.486	8.209	7.833	53761	Prrc2a	chr17	-	NM_0200	P9092	protein_cc	3110039B proline-ric	Prrc2a
8792	Aif1	0.132	1.651	0.218	0.000	0.001	0.026	7.190	4.270	4.993	11629	Aif1	chr17	-	NM_0194	P2866	protein_cc	AI607846| allograft in	Aif1
8793	Lst1	1.474	0.482	0.710	0.206	0.098	0.445	5.330	5.890	4.836	16988	Lst1	chr17	-	NM_0107	P4197	protein_cc	B144 leukocyte	Lst1
8794	Ltb	2.335	1.069	2.495	0.170	0.851	0.255	1.590	2.814	2.909	16994	Ltb	chr17	+	NM_0085	P17546	protein_cc	AI662801| lymphotox	Ltb
8795	Tnf	32.289	0.130	4.206	0.047	0.064	0.055	2.184	7.197	4.256	21926	Tnf	chr17	-	NM_0136	P5209	protein_cc	DIF| TNF-a tumor nec	Tnf
8796	Lta	2.134	0.469	1.000	0.305	1.000	0.423	0.000	1.094	0.000	16992	Lta	chr17	-	NM_0107	P2774	protein_cc	hlb382| L1 lymphotox	Lta
8797	Nfkbil1	2.082	1.159	2.414	0.006	0.128	0.083	4.396	5.454	5.667	18038	Nfkbil1	chr17	-	NM_0109	P16959	protein_cc	Def-7| IKB nuclear fa	Nfkbil1
8798	Atp6v1g2	0.931	0.565	0.526	0.913	0.545	0.338	3.748	3.645	2.821	66237	Atp6v1g2	chr17	+	NM_0231	P22032	protein_cc	1500002D ATPase, H	Atp6v1g2
8799	Ddx39b	2.587	0.808	2.090	0.000	0.013	0.016	8.568	9.940	9.632	53817	Ddx39b	chr17	+	NM_0196	P13165	protein_cc	0610030D DEAD (Asp	Ddx39b
8800	H2-D1	2.961	0.881	2.610	0.016	0.584	0.101	12.149	13.715	13.533	14964	H2-D1	chr17	+	NM_0103	P17700	protein_cc	H-2D| H2- histocomp	H2-D1
8801	H2-L	1.000	1.000	1.000	1.000	1.000	1.000	0.000	0.000	0.000	.	H2-L	chr17	+	NM_0012	P19639			H2-L
8802	H2-Q1	0.389	0.532	0.207	0.013	0.037	0.067	6.241	4.877	3.968	15006	H2-Q1	chr17	+	NM_0103	P9048	protein_cc	15006| H- histocomp	H2-Q1
8803	H2-Q2	1.005	1.405	1.412	0.000	0.182	0.423	0.000	0.008	0.498	15013	H2-Q2	chr17	+	NM_0103	P6134	protein_cc	BE136769 histocomp	H2-Q2
8804	H2-Q4	6.558	0.554	3.631	0.100	0.382	0.380	1.584	4.297	3.444	15015	H2-Q4	chr17	+	NM_0011	P26828	protein_cc	H-2Q4| H2 histocomp	H2-Q4
8805	H2-Q5	7.829	0.410	3.214	0.009	0.031	0.027	1.183	4.152	2.867	15016	H2-Q5	chr17	+	NR_05198	.	misc_RNA	H-2Q5| Q histocomp	H2-Q5
8806	H2-Q8	1.960	0.546	1.070	0.020	0.041	0.923	0.783	1.755	0.881	15019	H2-Q8	chr17	+	NM_0231	P12436	protein_cc	H-2Q8| M histocomp	H2-Q8
8807	H2-Q6	1.580	0.633	1.000	0.305	1.000	0.423	0.000	0.660	0.000	110557	H2-Q6	chr17	+	NM_2076	P12436	protein_cc	0610037N histocomp	H2-Q6
8808	H2-Q7	3.740	0.893	3.342	0.002	0.740	0.336	1.020	2.923	2.761	15018	H2-Q7	chr17	+	NM_0103	P21717	protein_cc	H-2Q7| Pe histocomp	H2-Q7
8809	H2-Q9	6.184	0.718	4.437	0.000	0.060	0.082	8.706	11.334	10.855	110558	H2-Q9	chr17	+	NM_0012	P21717	protein_cc	H-2Q9| Pe histocomp	H2-Q9
8810	H2-Q10	0.778	1.139	0.886	0.003	0.333	0.553	13.523	13.161	13.348	15007	H2-Q10	chr17	+	NM_0103	P706	protein_cc	H-2Q10| C histocomp	H2-Q10
8811	Pou5f1	2.149	0.465	1.000	0.306	1.000	0.423	0.000	1.104	0.000	18999	Pou5f1	chr17	+	NM_0136	P24264	protein_cc	NF-A3| Oc POU doma	Pou5f1
8812	Tcf19	0.460	1.314	0.605	0.021	0.160	0.183	4.702	3.582	3.977	106795	Tcf19	chr17	-	NM_0256	P11186	protein_cc	5730403J1 transcripti	Tcf19
8813	Cchcr1	1.879	0.816	1.533	0.251	0.687	0.593	2.647	3.557	3.264	240084	Cchcr1	chr17	+	NM_1462	P19216	protein_cc	Hcr coiled-coil	Cchcr1
8814	Psors1c2	3.300	0.303	1.000	0.306	1.000	0.423	0.001	1.723	0.000	57390	Psors1c2	chr17	+	NM_0205	P25511	protein_cc	AI448740| psoriasis s	Psors1c2

8815	Cdsn	1.000	1.000	1.000	1.000	1.000	1.000	0.000	0.000	0.000	386463	Cdsn	chr17	+	NM_0010	P8040	protein_cc	AI747712	corneodes	Cdsn
8816	2300002M	1.000	1.000	1.000	1.000	1.000	1.000	0.000	0.000	0.000	69542	2300002M	chr17	+	NM_1751	P11818	protein_cc	emprin| S	RIKEN cDN	2300002M
8817	Gm9573	1.000	1.000	1.000	1.000	1.000	1.000	0.000	0.000	0.000	672682	Gm9573	chr17	-	NM_0012	P1822	protein_cc	672682| N	predicted	Gm9573
8818	Dpcr1	0.669	0.995	0.666	0.181	1.000	0.423	0.587	0.008	0.000	268949	Dpcr1	chr17	-	NM_0010	P2339	protein_cc	Gm630	diffuse pa	Dpcr1
8819	Sfta2	1.000	1.000	1.000	1.000	1.000	1.000	0.000	0.000	0.000	433102	Sfta2	chr17	+	NM_0011	P20303	protein_cc	EG433102	surfactant	Sfta2
8820	Vars2	0.348	1.597	0.556	0.002	0.170	0.214	7.249	5.728	6.404	68915	Vars2	chr17	-	NM_1751	P12054	protein_cc	1190004I2	valyl-tRNA	Vars2
8821	Gtf2h4	0.759	1.199	0.910	0.022	0.119	0.593	6.190	5.792	6.054	14885	Gtf2h4	chr17	-	NM_0103	P24917	protein_cc	AW54563	general tr	Gtf2h4
8822	Ddr1	7.521	0.221	1.665	0.001	0.002	0.529	4.446	7.357	5.182	12305	Ddr1	chr17	-	NM_0075	P17446	protein_cc	6030432F	discoidin c	Ddr1
8823	4833427F	8.179	1.018	8.322	0.000	0.915	0.071	0.002	3.034	3.059	74601	4833427F	chr17	+	NR_04545	.	lncRNA	.	RIKEN cDN	4833427F1
8824	Ier3	146.284	0.746	109.164	0.000	0.368	0.195	4.618	11.811	11.389	15937	Ier3	chr17	+	NM_1336	P9075	protein_cc	AI663993|	immediate	Ier3
8825	Flot1	3.249	1.549	5.032	0.000	0.071	0.078	7.465	9.165	9.796	14251	Flot1	chr17	+	NM_0080	P16261	protein_cc	reggie-2	flotillin 1	Flot1
8826	Tubb5	1.110	1.259	1.398	0.484	0.414	0.503	9.989	10.140	10.472	22154	Tubb5	chr17	-	NM_0116	P6043	protein_cc	AA408537	tubulin, be	Tubb5
8827	Mdc1	1.338	0.907	1.213	0.027	0.402	0.457	5.640	6.060	5.919	240087	Mdc1	chr17	+	NM_0010	P7791	protein_cc	6820401C	mediator	Mdc1
8828	Nrm	1.174	0.412	0.484	0.498	0.059	0.123	3.179	3.410	2.131	106582	Nrm	chr17	+	NM_1341	P5344	protein_cc	2610307N	nurim (nu	Nrm
8829	Ppp1r18	4.481	0.968	4.339	0.000	0.764	0.044	5.314	7.478	7.432	76448	Ppp1r18	chr17	+	NM_0011	P10154	protein_cc	2310014H	protein ph	Ppp1r18
8830	Dhx16	1.093	1.073	1.172	0.237	0.322	0.082	7.851	7.979	8.081	69192	Dhx16	chr17	+	NM_0269	P22087	protein_cc	2410006N	DEAH (Asp	Dhx16
8831	2310061I0	0.308	1.246	0.383	0.000	0.341	0.031	8.482	6.781	7.099	69662	2310061I0	chr17	-	NM_0010	P12727	protein_cc	.	RIKEN cDN	2310061I0
8832	Atat1	0.336	0.459	0.154	0.015	0.105	0.082	6.883	5.308	4.186	73242	Atat1	chr17	-	NM_0011	P6070	protein_cc	0610011P	alpha tubu	Atat1
8833	Mrps18b	1.717	1.116	1.916	0.000	0.182	0.038	8.549	9.328	9.487	66973	Mrps18b	chr17	-	NM_0258	P7164	protein_cc	2400002C	mitochond	Mrps18b
8834	Ppp1r10	2.036	0.611	1.245	0.000	0.001	0.204	7.498	8.524	7.814	52040	Ppp1r10	chr17	+	NM_0011	P8824	protein_cc	2610025H	protein ph	Ppp1r10
8835	Gm8801	2.153	0.554	1.193	0.061	0.118	0.626	4.813	5.919	5.067	667766	Gm8801	chr17	+	NR_02827	.	misc_RNA	667766	predicted	Gm8801
8836	Abcf1	1.285	1.035	1.330	0.079	0.726	0.018	9.355	9.717	9.766	224742	Abcf1	chr17	-	NM_0138	P20664	protein_cc	Abc50| AU	ATP-bindi	Abcf1
8837	Prr3	0.737	1.776	1.309	0.324	0.173	0.539	5.767	5.327	6.156	75210	Prr3	chr17	-	NM_1454	P593	protein_cc	4930540G	proline-ric	Prr3
8838	Gnl1	1.783	1.056	1.882	0.010	0.751	0.152	7.369	8.203	8.282	14670	Gnl1	chr17	+	NM_0081	P18922	protein_cc	Gna-rs1| (	guanine n	Gnl1
8839	A930015D	3.528	0.150	0.531	0.093	0.059	0.310	1.657	3.476	0.743	77810	A930015D	chr17	+	NR_01561	.	lncRNA	.	RIKEN cDN	A930015D
8840	H2-T24	1.628	0.458	0.745	0.139	0.040	0.628	3.941	4.645	3.517	15042	H2-T24	chr17	-	NM_0082	P636	protein_cc	H-2T24	histocomp	H2-T24
8841	H2-T23	1.129	2.110	2.382	0.793	0.392	0.561	3.791	3.966	5.043	15040	H2-T23	chr17	-	NM_0103	P7078	protein_cc	37b| 37c|	histocomp	H2-T23
8842	H2-T22	1.569	0.892	1.400	0.149	0.627	0.082	8.957	9.607	9.442	15039	H2-T22	chr17	-	NM_0103	P25423	protein_cc	27b| H-2T	histocomp	H2-T22
8843	H2-T9	1.570	0.891	1.399	0.147	0.621	0.088	8.931	9.582	9.416	.	H2-T9	chr17	-	NM_0103	P25423	.	.	.	H2-T9
8844	Gm6034	1.229	0.654	0.804	0.729	0.500	0.728	0.816	1.113	0.500	547347	Gm6034	chr17	+	NM_0010	P20731	protein_cc	EG547347	predicted	Gm6034
8845	Gm11127	1.053	0.632	0.666	0.897	1.000	0.423	0.587	0.661	0.000	10052908	Gm11127	chr17	-	NM_0011	P10523	protein_cc	ENSMUSG	predicted	Gm11127
8846	H2-Bl	2.116	0.985	2.084	0.356	0.979	0.384	1.288	2.369	2.347	14963	H2-Bl	chr17	-	NM_0081	P1075	protein_cc	H2-B1| H2	histocomp	H2-Bl
8847	C920025E	1.520	0.265	0.403	0.283	0.056	0.057	3.344	3.948	2.033	.	C920025E	chr17	-	NM_0012	P14796	.	.	.	C920025E
8848	H2-T10	1.392	0.685	0.953	0.084	0.109	0.845	7.482	7.959	7.413	15024	H2-T10	chr17	-	NR_04628	.	misc_RNA	H-2T10	histocomp	H2-T10
8849	Gm7030	1.572	0.636	1.000	0.304	1.000	0.423	0.000	0.653	0.000	630294	Gm7030	chr17	-	NM_0011	P1025	protein_cc	EG630294	predicted	Gm7030
8850	2410017I1	1.000	1.000	1.000	1.000	1.000	1.000	0.000	0.000	0.000	675325	2410017I1	chr17	+	NR_03351	.	lncRNA	.	RIKEN cDN	2410017I1
8851	Gm8909	1.251	0.643	0.804	0.397	0.036	0.728	0.816	1.138	0.500	667977	Gm8909	chr17	-	NM_0010	P14772	protein_cc	EG667977	predicted	Gm8909
8852	Gm6623	3.058	0.900	2.752	0.001	0.689	0.251	3.822	5.434	5.282	625785	Gm6623	chr17	+	NR_03361	.	misc_RNA	EG625785	predicted	Gm6623
8853	H2-T3	1.000	1.000	1.000	1.000	1.000	1.000	0.000	0.000	0.000	15043	H2-T3	chr17	-	NM_0082	P12847	protein_cc	H-2T3| H2	histocomp	H2-T3
8854	Rpp21	0.425	1.318	0.560	0.012	0.006	0.170	7.982	6.747	7.145	67676	Rpp21	chr17	-	NM_0263	P26576	protein_cc	0610037N	ribonuclea	Rpp21
8855	Trim39	4.509	0.510	2.298	0.001	0.007	0.025	4.786	6.959	5.987	79263	Trim39	chr17	-	NM_1782	P15785	protein_cc	1100001D	tripartite	Trim39
8856	H2-M10.2	1.000	1.000	1.000	1.000	1.000	1.000	0.000	0.000	0.000	333715	H2-M10.2	chr17	-	NM_1779	P14149	protein_cc	4.7H	histocomp	H2-M10.2
8857	H2-M10.1	1.026	3.449	3.539	0.000	0.181	0.423	0.001	0.038	1.824	14985	H2-M10.1	chr17	-	NM_0135	P5714	protein_cc	9.5H	histocomp	H2-M10.1
8858	H2-M10.3	1.603	1.658	2.658	0.292	0.404	0.423	0.001	0.681	1.411	110696	H2-M10.3	chr17	-	NM_2016	P9749	protein_cc	5.3H	histocomp	H2-M10.3
8859	H2-M10.4	1.000	1.000	1.000	1.000	1.000	1.000	0.000	0.000	0.000	224753	H2-M10.4	chr17	-	NM_1776	P25911	protein_cc	15H	histocomp	H2-M10.4
8860	H2-M11	1.000	1.000	1.000	1.000	1.000	1.000	0.000	0.000	0.000	224754	H2-M11	chr17	+	NM_1776	P13155	protein_cc	.	histocomp	H2-M11
8861	H2-M9	1.000	1.000	1.000	1.000	1.000	1.000	0.000	0.000	0.000	14997	H2-M9	chr17	-	NM_0082	P14501	protein_cc	M9	histocomp	H2-M9
8862	H2-M1	1.000	1.000	1.000	1.000	1.000	1.000	0.000	0.000	0.000	224756	H2-M1	chr17	-	NM_1776	P21076	protein_cc	H-2M1| H	histocomp	H2-M1
8863	H2-M10.5	6.397	1.095	7.006	0.025	0.730	0.023	0.002	2.679	2.810	224761	H2-M10.5	chr17	+	NM_1776	P25610	protein_cc	6.9H	histocomp	H2-M10.5
8864	H2-M10.6	1.000	1.000	1.000	1.000	1.000	1.000	0.000	0.000	0.000	399549	H2-M10.6	chr17	+	NM_2016	P8808	protein_cc	.	histocomp	H2-M10.6
8865	Trim26	1.072	0.724	0.777	0.555	0.027	0.224	7.680	7.781	7.315	22670	Trim26	chr17	+	NM_0010	P10601	protein_cc	AI462198|	tripartite	Trim26
8866	Trim15	2.711	0.521	1.413	0.304	0.428	0.423	0.001	1.439	0.499	69097	Trim15	chr17	-	NM_0011	P8512	protein_cc	1810012B	tripartite	Trim15
8867	Trim10	2.610	0.255	0.665	0.012	1.000	0.423	0.588	1.972	0.000	19824	Trim10	chr17	+	NM_0112	P3239	protein_cc	AI324236|	tripartite	Trim10
8868	Trim40	1.000	1.000	1.000	1.000	1.000	1.000	0.000	0.000	0.000	195359	Trim40	chr17	-	NM_0010	P13269	protein_cc	Gm319| G	tripartite	Trim40
8869	Trim31	1.000	1.000	1.000	1.000	1.000	1.000	0.000	0.000	0.000	224762	Trim31	chr17	+	NM_1460	P18814	protein_cc	BC026666	tripartite	Trim31
8870	1700031A	1.000	1.000	1.000	1.000	1.000	1.000	0.000	0.000	0.000	73286	1700031A	chr17	+	NR_04543	.	lncRNA	.	RIKEN cDN	1700031A
8871	Rnf39	1.000	1.000	1.000	1.000	1.000	1.000	0.000	0.000	0.000	386454	Rnf39	chr17	+	NM_0010	P17934	protein_cc	BC038661	ring finger	Rnf39
8872	Ppp1r11	2.172	0.823	1.788	0.001	0.056	0.009	8.941	10.060	9.780	76497	Ppp1r11	chr17	-	NM_0296	P8978	protein_cc	1500041B	protein ph	Ppp1r11
8873	Zrnd1	1.861	0.571	1.063	0.028	0.024	0.784	7.065	7.961	7.153	66136	Zrnd1	chr17	-	NM_0231	P5864	protein_cc	1110014N	zinc ribbo	Zrnd1
8874	Zrnd1as	0.877	1.089	0.955	0.209	0.547	0.800	8.066	7.876	8.000	76416	Zrnd1as	chr17	+	NM_0296	P18986	protein_cc	1700022C	zinc ribbo	Zrnd1as
8875	2410137M	2.134	0.469	1.000	0.305	1.000	0.423	0.000	1.094	0.000	76797	2410137M	chr17	-	NM_0297	P14616	protein_cc	RP23-98F2	RIKEN cDN	2410137M
8876	H2-M5	1.000	1.000	1.000	1.000	1.000	1.000	0.000	0.000	0.000	240095	H2-M5	chr17	-	NM_0011	P5876	protein_cc	CRW2| D1	histocomp	H2-M5
8877	Zfp57	1.010	1.825	1.844	0.000	0.182	0.423	0.000	0.015	0.883	22715	Zfp57	chr17	+	NM_0011	P25067	protein_cc	G19| Zfp-5	zinc finger	Zfp57
8878	Mog	1.000	1.000	1.000	1.000	1.000	1.000	0.000	0.000	0.000	17441	Mog	chr17	-	NM_0108	P17039	protein_cc	B230317G	myelin olig	Mog
8879	Gabbr1	1.519	1.350	2.051	0.670	0.667	0.121	2.332	2.935	3.368	54393	Gabbr1	chr17	+	NM_0194	P1135	protein_cc	bM573K1.	gamma-am	Gabbr1
8880	Olfr90	1.000	1.000	1.000	1.000	1.000	1.000	0.000	0.000	0.000	258469	Olfr90	chr17	-	NM_1464	P4393	protein_cc	bM573K1.	olfactory r	Olfr90
8881	Olfr91	1.000	1.000	1.000	1.000	1.000	1.000	0.000	0.000	0.000	258470	Olfr91	chr17	-	NM_1827	P6667	protein_cc	bM573K1.	olfactory r	Olfr91
8882	Olfr92	1.000	1.000	1.000	1.000	1.000	1.000	0.000	0.000	0.000	258448	Olfr92	chr17	-	NM_1464	P14933	protein_cc	bM573K1.	olfactory r	Olfr92
8883	Olfr93	1.000	1.000	1.000	1.000	1.000	1.000	0.000	0.000	0.000	258051	Olfr93	chr17	-	NM_0010	P26296	protein_cc	MOR256-3	olfactory r	Olfr93
8884	Ubd	203.062	0.137	27.747	0.000	0.000	0.277	3.461	11.127	8.255	24108	Ubd	chr17	+	NM_0231	P14502	protein_cc	FAT10	ubiquitin D	Ubd
8885	Olfr94	1.000	1.000	1.000	1.000	1.000	1.000	0.000	0.000	0.000	258219	Olfr94	chr17	-	NM_0010	P9780	protein_cc	MOR256-3	olfactory r	Olfr94
8886	Olfr95	1.000	1.000	1.000	1.000	1.000	1.000	0.000	0.000	0.000	258506	Olfr95	chr17	-	NM_1465	P24788	protein_cc	MOR263-6	olfactory r	Olfr95
8887	Olfr96	1.000	1.000	1.000	1.000	1.000	1.000	0.000	0.000	0.000	258507	Olfr96	chr17	+	NM_1465	P7274	protein_cc	MOR121-1	olfactory r	Olfr96
8888	Olfr97	1.000	1.000	1.000	1.000	1.000	1.000	0.000	0.000	0.000	258505	Olfr97	chr17	-	NM_1465	P10404	protein_cc	MOR156-2	olfactory r	Olfr97
8889	Olfr98	1.000	1.000	1.000	1.000	1.000	1.000	0.000	0.000	0.000	258503	Olfr98	chr17	-	NM_1465	P21048	protein_cc	MOR156-4	olfactory r	Olfr98
8890	H2-M3	6.360	0.893	5.678	0.002	0.371	0.009	2.765	5.434	5.270	14991	H2-M3	chr17	+	NM_0138	P20309	protein_cc	H-2M3| H	histocomp	H2-M3
8891	Olfr99	1.000	1.000	1.000	1.000	1.000	1.000	0.000	0.000	0.000	258508	Olfr99	chr17	-	NM_1465	P8984	protein_cc	MOR156-1	olfactory r	Olfr99
8892	Olfr101	1.005	1.405	1.412	0.000	0.182	0.423	0.000	0.008	0.498	258831	Olfr101	chr17	-	NM_1468	P10753	protein_cc	MOR250-2	olfactory r	Olfr101
8893	Olfr102	1.000	1.000	1.000	1.000	1.000	1.000	0.000	0.000	0.000	258218	Olfr102	chr17	-	NM_0010	P8615	protein_cc	MOR250-4	olfactory r	Olfr102
8894	Olfr100	1.000	1.000	1.000	1.000	1.000	1.000	0.000	0.000	0.000	404307	Olfr100	chr17	-	NM_2076	P8615	protein_cc	GA_x5J8B	olfactory r	Olfr100
8895	Olfr103	1.005	1.405	1.412	0.000	0.182	0.423	0.000	0.008	0.498	258830	Olfr103	chr17	-	NM_1468	P11474	protein_cc	MOR250-3	olfactory r	Olfr103
8896	Olfr107	1.000	1.000	1.000	1.000	1.000	1.000	0.000	0.000	0.000	258504	Olfr107	chr17	+	NM_1465	P5985	protein_cc	MOR156-3	olfactory r	Olfr107
8897	Olfr108	1.000	1.000	1.000	1.000	1.000	1.000	0.000	0.000	0.000	258457	Olfr108	chr17	+	NM_1464	P21988	protein_cc	MOR156-5	olfactory r	Olfr108
8898	Olfr109	1.000	1.000	1.000	1.000	1.000	1.000	0.000	0.000	0.000	258832	Olfr109	chr17	-	NM_1468	P654	protein_cc	MOR250-1	olfactory r	Olfr109
8899	H2-M2	3.079	2.615	8.052	0.001	0.035	0.111	1.325	2.947	4.334	14990	H2-M2	chr17	-	NM_0082	P20408	protein_cc	H-2M2| Tl	histocomp	H2-M2
8900	Olfr110	1.000	1.000	1.000	1.000	1.000	1.000	0.000	0.000	0.000	258325	Olfr110	chr17	-	NM_1463	P14956	protein_cc	MOR249-2	olfactory r	Olfr110
8901	Olfr111	1.000	1.000	1.000	1.000	1.000	1.000	0.000	0.000	0.000	545205	Olfr111	chr17	+	NM_0010	P15790	protein_cc	.	olfactory r	Olfr111
8902	Olfr112	0.716	0.995	0.712	0.182	1.000	0.423	0.490	0.008	0.000	258096	Olfr112	chr17	-	NM_0010	P8477	protein_cc	4930580F	olfactory r	Olfr112
8903	Olfr113	1.000	1.000	1.000	1.000	1.000	1.000	0.000	0.000	0.000	258286	Olfr113	chr17	-	NM_1462	P24365	protein_cc	MOR218-9	olfactory r	Olfr113
8904	Olfr114	1.000	1.000	1.000	1.000	1.000	1.000	0.000	0.000	0.000	258284	Olfr114	chr17	-	NM_1462	P8821	protein_cc	MOR218-1	olfactory r	Olfr114
8905	Olfr115	1.000	1.000	1.000	1.000	1.000	1.000	0.000	0.000	0.000	257908	Olfr115	chr17	-	NM_0010	P1618	protein_cc	MOR218-1	olfactory r	Olfr115

8906	Olfr116	1.000	1.000	1.000	1.000	1.000	1.000	0.000	0.000	0.000	258625	Olfr116	chr17	-	NM_1466	P15208	protein_cc	MOR218-2	olfactory r	Olfr116
8907	Olfr117	1.000	1.000	1.000	1.000	1.000	1.000	0.000	0.000	0.000	258263	Olfr117	chr17	-	NM_2071	P8376	protein_cc	MOR256-3	olfactory r	Olfr117
8908	Olfr118	1.000	1.000	1.000	1.000	1.000	1.000	0.000	0.000	0.000	404308	Olfr118	chr17	+	NM_2137	P16004	protein_cc	MOR263-1	olfactory r	Olfr118
8909	Olfr119	1.000	1.000	1.000	1.000	1.000	1.000	0.000	0.000	0.000	258095	Olfr119	chr17	+	NM_0010	P22286	protein_cc	MOR263-7	olfactory r	Olfr119
8910	Olfr120	1.000	1.000	1.000	1.000	1.000	1.000	0.000	0.000	0.000	258624	Olfr120	chr17	+	NM_1466	P24327	protein_cc	MOR263-3	olfactory r	Olfr120
8911	Olfr121	1.000	1.000	1.000	1.000	1.000	1.000	0.000	0.000	0.000	258622	Olfr121	chr17	+	NM_1466	P5343	protein_cc	MOR263-4	olfactory r	Olfr121
8912	Olfr122	1.000	1.000	1.000	1.000	1.000	1.000	0.000	0.000	0.000	258285	Olfr122	chr17	+	NM_1462	P10148	protein_cc	MOR263-1	olfactory r	Olfr122
8913	Olfr123	1.000	1.000	1.000	1.000	1.000	1.000	0.000	0.000	0.000	258623	Olfr123	chr17	+	NM_1466	P15810	protein_cc	MOR256-9	olfactory r	Olfr123
8914	Olfr124	1.000	1.000	1.000	1.000	1.000	1.000	0.000	0.000	0.000	259064	Olfr124	chr17	+	NM_1470	P22420	protein_cc	A3| MOR2	olfactory r	Olfr124
8915	Olfr125	1.000	1.000	1.000	1.000	1.000	1.000	0.000	0.000	0.000	258287	Olfr125	chr17	+	NM_1462	P22105	protein_cc	MOR218-8	olfactory r	Olfr125
8916	Olfr126	1.000	1.000	1.000	1.000	1.000	1.000	0.000	0.000	0.000	258892	Olfr126	chr17	+	NM_1468	P21060	protein_cc	MOR218-1	olfactory r	Olfr126
8917	Olfr127	1.000	1.000	1.000	1.000	1.000	1.000	0.000	0.000	0.000	258374	Olfr127	chr17	+	NM_1463	P5119	protein_cc	MOR218-7	olfactory r	Olfr127
8918	Olfr128	0.716	0.995	0.712	0.182	1.000	0.423	0.490	0.008	0.000	383243	Olfr128	chr17	+	NM_2068	P13475	protein_cc	GA_x5J8B	olfactory r	Olfr128
8919	Olfr761	1.000	1.000	1.000	1.000	1.000	1.000	0.000	0.000	0.000	258094	Olfr761	chr17	-	NM_0010	P26749	protein_cc	MOR218-1	olfactory r	Olfr761
8920	Olfr129	1.000	1.000	1.000	1.000	1.000	1.000	0.000	0.000	0.000	258324	Olfr129	chr17	-	NM_1463	P18783	protein_cc	MOR263-9	olfactory r	Olfr129
8921	Olfr130	1.000	1.000	1.000	1.000	1.000	1.000	0.000	0.000	0.000	258480	Olfr130	chr17	+	NM_1464	P10965	protein_cc	MOR256-1	olfactory r	Olfr130
8922	Olfr131	1.000	1.000	1.000	1.000	1.000	1.000	0.000	0.000	0.000	258867	Olfr131	chr17	+	NM_1468	P26376	protein_cc	MOR256-4	olfactory r	Olfr131
8923	Olfr132	1.000	1.000	1.000	1.000	1.000	1.000	0.000	0.000	0.000	257889	Olfr132	chr17	-	NM_0010	P10558	protein_cc	MOR256-4	olfactory r	Olfr132
8924	Olfr133	1.000	1.000	1.000	1.000	1.000	1.000	0.000	0.000	0.000	258828	Olfr133	chr17	+	NM_1468	P5276	protein_cc	MOR256-6	olfactory r	Olfr133
8925	Olfr134	1.000	1.000	1.000	1.000	1.000	1.000	0.000	0.000	0.000	258829	Olfr134	chr17	+	NM_1468	P6194	protein_cc	MOR256-5	olfactory r	Olfr134
8926	Olfr135	1.000	1.000	1.000	1.000	1.000	1.000	0.000	0.000	0.000	258329	Olfr135	chr17	+	NM_1463	P6675	protein_cc	MOR256-4	olfactory r	Olfr135
8927	Olfr138	1.000	1.000	1.000	1.000	1.000	1.000	0.000	0.000	0.000	170648	Olfr138	chr17	+	NM_1308	P7238	protein_cc	MOR256-4	olfactory r	Olfr138
8928	Olfr137	1.000	1.000	1.000	1.000	1.000	1.000	0.000	0.000	0.000	258481	Olfr137	chr17	-	NM_1464	P9667	protein_cc	MOR256-1	olfactory r	Olfr137
8929	Olfr136	1.000	1.000	1.000	1.000	1.000	1.000	0.000	0.000	0.000	258803	Olfr136	chr17	+	NM_1468	P13435	protein_cc	MOR256-7	olfactory r	Olfr136
8930	Esp36	1.000	1.000	1.000	1.000	1.000	1.000	0.000	0.000	0.000	10012676	Esp36	chr17	-	NM_0011	P10736	protein_cc	Gm20408	exocrine g	Esp36
8931	Esp34	1.000	1.000	1.000	1.000	1.000	1.000	0.000	0.000	0.000	10012677	Esp34	chr17	+	NM_0011	P17842	protein_cc	Gm20410	exocrine g	Esp34
8932	Esp31	1.000	1.000	1.000	1.000	1.000	1.000	0.000	0.000	0.000	10012676	Esp31	chr17	+	NM_0011	P16670	protein_cc	Gm20516	exocrine g	Esp31
8933	Esp24	1.000	1.000	1.000	1.000	1.000	1.000	0.000	0.000	0.000	10012677	Esp24	chr17	+	NM_0012	P3666	protein_cc	Gm21947	exocrine g	Esp24
8934	Esp23	1.000	1.000	1.000	1.000	1.000	1.000	0.000	0.000	0.000	10012677	Esp23	chr17	-	NM_0011	P17210	protein_cc	Gm20581	exocrine g	Esp23
8935	Esp18	1.000	1.000	1.000	1.000	1.000	1.000	0.000	0.000	0.000	10012677	Esp18	chr17	+	NM_0012	P7593	protein_cc	Gm20579	exocrine g	Esp18
8936	Esp16	1.000	1.000	1.000	1.000	1.000	1.000	0.000	0.000	0.000	10004330	Esp16	chr17	+	NM_0012	P3219	protein_cc	10004330	exocrine g	Esp16
8937	Esp15	1.014	2.240	2.272	0.000	0.182	0.423	0.000	0.021	1.185	668200	Esp15	chr17	+	NM_0012	P22895	protein_cc	EG668200	exocrine g	Esp15
8938	Rn45s	1.082	0.876	0.948	0.046	0.490	0.875	19.672	19.786	19.595	.	Rn45s	chr17	+	NR_04623	.	.	.	.	Rn45s
8939	Esp38	1.000	1.000	1.000	1.000	1.000	1.000	0.000	0.000	0.000	10012677	Esp38	chr17	+	NM_0012	P15003	protein_cc	Gm21946	exocrine g	Esp38
8940	Pgk2	1.000	1.000	1.000	1.000	1.000	1.000	0.000	0.000	0.000	18663	Pgk2	chr17	-	NM_0311	P10297	protein_cc	Pgk-2| Tcp	phosphog	Pgk2
8941	Crisp3	1.000	1.000	1.000	1.000	1.000	1.000	0.000	0.000	0.000	11572	Crisp3	chr17	-	NM_0096	P25054	protein_cc	Aeg2| CRI	cysteine-r	Crisp3
8942	Crisp1	1.000	1.000	1.000	1.000	1.000	1.000	0.000	0.000	0.000	11571	Crisp1	chr17	-	NM_0096	P12937	protein_cc	Aeg1| AW	cysteine-r	Crisp1
8943	Esp8	1.000	1.000	1.000	1.000	1.000	1.000	0.000	0.000	0.000	10012677	Esp8	chr17	+	NM_0011	P15805	protein_cc	Gm17495	exocrine g	Esp8
8944	Esp6Esp5	1.000	1.000	1.000	1.000	1.000	1.000	0.000	0.000	0.000	.	Esp6Esp5	chr17	+	NM_0012	P21590	.	.	.	Esp6Esp5
8945	Esp6	1.000	1.000	1.000	1.000	1.000	1.000	0.000	0.000	0.000	433107	Esp6	chr17	+	NM_0011	P10097	protein_cc	EG433107	exocrine g	Esp6
8946	Esp5	1.000	1.000	1.000	1.000	1.000	1.000	0.000	0.000	0.000	10257742	Esp5	chr17	+	NM_0012	P15303	protein_cc	.	exocrine g	Esp5
8947	Esp4	1.000	1.000	1.000	1.000	1.000	1.000	0.000	0.000	0.000	10012677	Esp4	chr17	+	NM_0011	P7409	protein_cc	Gm20580	exocrine g	Esp4
8948	Esp3	1.000	1.000	1.000	1.000	1.000	1.000	0.000	0.000	0.000	10012676	Esp3	chr17	+	NM_0012	P8208	protein_cc	Gm21945	exocrine g	Esp3
8949	Esp1	1.000	1.000	1.000	1.000	1.000	1.000	0.000	0.000	0.000	619517	Esp1	chr17	+	NM_0010	P26503	protein_cc	EG619517	exocrine g	Esp1
8950	Crisp2	1.010	1.885	1.904	0.000	0.182	0.423	0.000	0.015	0.929	22024	Crisp2	chr17	-	NM_0010	P8362	protein_cc	CRISP-2| (	cysteine-r	Crisp2
8951	Rhag	2.700	0.370	1.000	0.305	1.000	0.423	0.001	1.433	0.000	19743	Rhag	chr17	+	NM_0112	P10431	protein_cc	CD241| Rh	Rhesus blo	Rhag
8952	9130008F	3.902	0.736	2.872	0.338	0.723	0.166	0.378	2.342	1.900	71583	9130008F	chr17	-	NM_0278	P17698	protein_cc	.	RIKEN cDN	9130008F2
8953	Glyatl3	1.000	1.000	1.000	1.000	1.000	1.000	0.000	0.000	0.000	435528	Glyatl3	chr17	-	NM_0011	P21041	protein_cc	EG435528	glycine-N-	Glyatl3
8954	Cenpq	0.735	1.101	0.809	0.019	0.424	0.394	4.704	4.260	4.399	83815	Cenpq	chr17	-	NM_0318	P13138	protein_cc	2610528M	centromei	Cenpq
8955	Mut	0.479	0.815	0.390	0.001	0.021	0.023	11.363	10.301	10.006	17850	Mut	chr17	+	NM_0086	P8607	protein_cc	D230010K	methylma	Mut
8956	Ptchd4	0.339	0.981	0.333	0.181	1.000	0.423	1.588	0.028	0.000	627626	Ptchd4	chr17	+	NM_0284	P11284	protein_cc	3110082D	patched d	Ptchd4
8957	Opn5	1.000	1.000	1.000	1.000	1.000	1.000	0.000	0.000	0.000	353344	Opn5	chr17	-	NM_1817	P15078	protein_cc	Gpr136| N	opsin 5	Opn5
8958	Adgrf4	0.726	1.425	1.034	0.187	0.186	0.943	0.476	0.015	0.525	78249	Adgrf4	chr17	-	NM_0012	P3014	protein_cc	4632435A	adhesion (	Adgrf4
8959	Adgrf2	1.000	1.000	1.000	1.000	1.000	1.000	0.000	0.000	0.000	73504	Adgrf2	chr17	-	NM_0010	P25991	protein_cc	Gpr111| P	adhesion (	Adgrf2
8960	Cd2ap	1.427	0.779	1.112	0.031	0.104	0.465	8.897	9.410	9.050	12488	Cd2ap	chr17	-	NM_0098	P754	protein_cc	AL024079	CD2-asso(	Cd2ap
8961	Tnfrsf21	3.100	0.749	2.323	0.061	0.389	0.021	6.275	7.908	7.491	94185	Tnfrsf21	chr17	+	NM_1785	P21113	protein_cc	AA959878	tumor nec	Tnfrsf21
8962	E130008D	1.000	1.000	1.000	1.000	1.000	1.000	0.000	0.000	0.000	545207	E130008D	chr17	-	NR_04515	.	lncRNA	.	RIKEN cDN	E130008D(
8963	Adgrf1	1.312	1.697	2.227	0.027	0.041	0.141	4.545	4.937	5.700	77596	Adgrf1	chr17	+	NM_1337	P11703	protein_cc	5031409J1	adhesion (	Adgrf1
8964	Adgrf5	1.878	0.724	1.360	0.005	0.133	0.453	8.531	9.440	8.974	224792	Adgrf5	chr17	+	NM_0010	P5600	protein_cc	8430401C	adhesion (	Adgrf5
8965	Mep1a	1.589	1.186	1.885	0.298	0.707	0.423	0.000	0.669	0.915	17287	Mep1a	chr17	-	NM_0085	P10633	protein_cc	AI098089|	meprin 1 a	Mep1a
8966	Ankrd66	10.506	0.317	3.329	0.080	0.157	0.140	0.002	3.395	1.737	10004333	Ankrd66	chr17	-	NM_0012	P13218	protein_cc	AU041848	ankyrin re	Ankrd66
8967	Pla2g7	5.872	1.153	6.768	0.004	0.452	0.039	4.962	7.515	7.720	27226	Pla2g7	chr17	+	NM_0137	P2211	protein_cc	PAF-AH| F	phospholi	Pla2g7
8968	1700071M	3.915	0.255	0.999	0.004	1.000	0.423	0.001	1.970	0.000	73504	1700071M	chr17	-	NR_04544	.	lncRNA	.	RIKEN cDN	1700071M
8969	Tdrd6	1.000	1.000	1.000	1.000	1.000	1.000	0.000	0.000	0.000	210510	Tdrd6	chr17	-	NM_0011	P3933	protein_cc	AB097085	tudor dom	Tdrd6
8970	Slc25a27	0.881	0.717	0.631	0.623	0.258	0.454	2.195	2.011	1.531	74011	Slc25a27	chr17	-	NM_0287	P18965	protein_cc	3632410G	solute car	Slc25a27
8971	Cyp39a1	0.312	0.835	0.260	0.000	0.478	0.019	7.785	6.104	5.844	56050	Cyp39a1	chr17	+	NM_0012	P13066	protein_cc	mCYP39A	cytochrom	Cyp39a1
8972	Rcan2	1.581	1.760	2.782	0.002	0.209	0.306	6.692	7.353	8.169	53901	Rcan2	chr17	+	NM_2076	P18088	protein_cc	Csp2| Dsc	regulator	Rcan2
8973	Enpp5	0.253	1.488	0.377	0.008	0.293	0.081	7.141	5.159	5.732	83965	Enpp5	chr17	+	NM_0320	P23800	protein_cc	AW06100	ectonucle	Enpp5
8974	Enpp4	5.110	0.376	1.921	0.000	0.001	0.006	7.291	9.645	8.233	224794	Enpp4	chr17	-	NM_1990	P20537	protein_cc	4933413N	ectonucle	Enpp4
8975	Clic5	3.786	0.472	1.787	0.133	0.291	0.576	3.651	5.571	4.488	224796	Clic5	chr17	+	NM_1726	P15657	protein_cc	5730531E	chloride ir	Clic5
8976	Runx2	0.404	3.355	1.357	0.186	0.000	0.619	1.362	0.056	1.802	12393	Runx2	chr17	-	NR_07342	.	protein_cc	AML3| Cb	runt relate	Runx2
8977	Supt3	0.289	0.869	0.252	0.002	0.647	0.033	6.373	4.585	4.382	109115	Supt3	chr17	+	NM_1786	P13379	protein_cc	2310066G	suppresso	Supt3
8978	4930564C	1.000	1.000	1.000	1.000	1.000	1.000	0.000	0.000	0.000	75341	4930564C	chr17	-	NM_0292	P21957	protein_cc	.	RIKEN cDN	4930564C(
8979	Cdc5l	1.391	0.700	0.974	0.004	0.002	0.831	8.283	8.759	8.246	71702	Cdc5l	chr17	-	NM_1528	P18072	protein_cc	1200002IC	cell divisic	Cdc5l
8980	B230354K	0.759	0.606	0.460	0.272	0.204	0.083	6.728	6.330	5.607	10524627	B230354K	chr17	+	NR_13096	.	lncRNA	.	RIKEN cDN	B230354K
8981	Spats1	1.000	1.000	1.000	1.000	1.000	1.000	0.000	0.000	0.000	71020	Spats1	chr17	-	NM_0276	P13898	protein_cc	1700011H	spermatog	Spats1
8982	Aars2	0.271	1.596	0.433	0.000	0.303	0.144	6.765	4.884	5.558	224805	Aars2	chr17	+	NM_1986	P26146	protein_cc	Aarsl| Alai	alanyl-tRN	Aars2
8983	Tcte1	1.000	1.000	1.000	1.000	1.000	1.000	0.000	0.000	0.000	21645	Tcte1	chr17	+	NM_0136	P11000	protein_cc	D17Sil1| T	t-complex	Tcte1
8984	Tmem151	0.405	0.985	0.399	0.181	1.000	0.423	1.324	0.022	0.000	210573	Tmem151	chr17	-	NM_0010	P25317	protein_cc	Gm323	transmem	Tmem151I
8985	Nfkbie	9.517	0.773	7.358	0.001	0.169	0.042	4.564	7.815	7.443	18037	Nfkbie	chr17	+	NM_0130	P22976	protein_cc	I-kappa-B-	nuclear fa	Nfkbie
8986	Slc35b2	0.549	1.574	0.864	0.005	0.020	0.545	8.123	7.259	7.913	73836	Slc35b2	chr17	+	NM_0286	P20393	protein_cc	1110003M	solute car	Slc35b2
8987	Hsp90ab1	0.631	0.972	0.614	0.001	0.870	0.151	14.080	13.417	13.376	15516	Hsp90ab1	chr17	-	NM_0083	P16401	protein_cc	90kDa| AL	heat shocl	Hsp90ab1
8988	Slc29a1	0.048	1.838	0.085	0.000	0.071	0.004	10.842	6.411	7.290	63959	Slc29a1	chr17	-	NM_0011	P15403	protein_cc	1200014D	solute car	Slc29a1
8989	Gm7325	1.000	1.000	1.000	1.000	1.000	1.000	0.000	0.000	0.000	653016	Gm7325	chr17	-	NM_0011	P16078	protein_cc	EG653016	predicted	Gm7325
8990	Capn11	0.328	2.665	0.874	0.000	0.185	0.842	1.662	0.053	1.467	268958	Capn11	chr17	-	NM_0010	P16047	protein_cc	.	calpain 11	Capn11
8991	Tmem63b	0.209	0.915	0.191	0.018	0.853	0.124	8.839	6.582	6.453	224807	Tmem63b	chr17	-	NM_1981	P8364	protein_cc	BC026370	transmem	Tmem63b
8992	Mrpl14	0.406	1.107	0.450	0.000	0.594	0.061	10.118	8.817	8.965	68463	Mrpl14	chr17	+	NM_0267	P5856	protein_cc	1110006I1	mitochonc	Mrpl14
8993	1600014C	1.023	3.051	3.120	0.000	0.182	0.423	0.000	0.033	1.642	72240	1600014C	chr17	-	NM_0281	P8348	protein_cc	.	RIKEN cDN	1600014C
8994	Vegfa	0.807	0.370	0.299	0.056	0.000	0.019	9.534	9.225	7.791	22339	Vegfa	chr17	-	NM_0010	P19978	protein_cc	Vegf| Vpf	vascular e	Vegfa
8995	Mrps18a	1.187	1.516	1.799	0.049	0.017	0.074	7.510	7.757	8.357	68565	Mrps18a	chr17	+	NM_0267	P23635	protein_cc	1110004O	mitochonc	Mrps18a
8996	Rsph9	0.487	1.764	0.859	0.077	0.160	0.786	2.569	1.531	2.350	75564	Rsph9	chr17	-	NM_0293	P19223	protein_cc	1700027N	radial spo	Rsph9

8997	Mad2l1bp	1.723	1.071	1.845	0.107	0.770	0.121	6.168	6.952	7.051	66591	Mad2l1bp	chr17	-	NM_0256	P9374	protein_cc	0610009D	MAD2L1 b	Mad2l1bp
8998	Gtpbp2	1.823	0.828	1.510	0.097	0.594	0.522	6.692	7.559	7.287	56055	Gtpbp2	chr17	+	NM_0011	P6211	protein_cc	nmf205	GTP bindi	Gtpbp2
8999	Polh	1.092	0.828	0.903	0.901	0.790	0.914	1.340	1.466	1.193	80905	Polh	chr17	-	NM_0307	P24637	protein_cc	RAD30A|	polymeras	Polh
9000	Xpo5	1.474	1.359	2.003	0.001	0.080	0.117	4.705	5.265	5.707	72322	Xpo5	chr17	+	NM_0281	P3691	protein_cc	2410004H	exportin 5	Xpo5
9001	Polr1c	1.882	0.824	1.551	0.001	0.043	0.118	8.000	8.912	8.634	20016	Polr1c	chr17	-	NM_0090	P331	protein_cc	40kDa| A	polymeras	Polr1c
9002	Yipf3	0.906	1.184	1.073	0.220	0.269	0.783	8.909	8.765	9.010	28064	Yipf3	chr17	+	NM_1453	P7692	protein_cc	C6orf109|	Yip1 doma	Yipf3
9003	Lrrc73	2.298	0.591	1.357	0.042	0.071	0.744	1.832	3.032	2.272	224813	Lrrc73	chr17	+	NM_0011	P17635	protein_cc	Gm88	leucine ric	Lrrc73
9004	Tjap1	2.582	0.797	2.057	0.014	0.173	0.088	5.425	6.794	6.466	74094	Tjap1	chr17	-	NM_0287	P19578	protein_cc	0610041D	tight junct	Tjap1
9005	Dlk2	0.391	1.871	0.732	0.000	0.091	0.390	3.393	2.039	2.943	106565	Dlk2	chr17	+	NM_0012	P17854	protein_cc	AI413481|	delta-like	Dlk2
9006	Abcc10	0.459	1.813	0.833	0.050	0.282	0.741	4.176	3.053	3.912	224814	Abcc10	chr17	-	NM_1451	P2301	protein_cc	mFLJ0000	ATP-bindi	Abcc10
9007	Zfp318	1.361	0.710	0.967	0.014	0.000	0.836	8.598	9.042	8.549	57908	Zfp318	chr17	+	NM_0213	P26563	protein_cc	2610034E	zinc finger	Zfp318
9008	Crip3	0.283	0.974	0.275	0.002	1.000	0.052	1.860	0.038	0.000	114570	Crip3	chr17	+	NM_1816	P13012	protein_cc	Tlmp| TLP	cysteine-r	Crip3
9009	Slc22a7	0.360	1.673	0.602	0.033	0.345	0.436	4.422	2.948	3.690	108114	Slc22a7	chr17	-	NM_1448	P1130	protein_cc	NLT| OAT	solute car	Slc22a7
9010	Ttbk1	1.010	1.885	1.904	0.000	0.182	0.423	0.000	0.015	0.929	106763	Ttbk1	chr17	-	NM_0011	P13755	protein_cc	AU017937	tau tubuli	Ttbk1
9011	Dnph1	0.253	1.881	0.476	0.007	0.065	0.199	6.205	4.222	5.134	381101	Dnph1	chr17	+	NM_2071	P18393	protein_cc	C76683| R	2'-deoxyn	Dnph1
9012	Cul9	0.724	3.781	2.736	0.162	0.039	0.260	1.669	1.202	3.121	78309	Cul9	chr17	-	NM_0010	P26120	protein_cc	1810035I	cullin 9	Cul9
9013	Srf	2.229	0.813	1.812	0.013	0.327	0.177	7.381	8.537	8.238	20807	Srf	chr17	-	NM_0204	P22317	protein_cc	AW04994	serum res	Srf
9014	Ptk7	0.231	3.668	0.846	0.183	0.001	0.863	2.194	0.077	1.952	71461	Ptk7	chr17	-	NM_1751	P8074	protein_cc	8430404F	PTK7 prot	Ptk7
9015	Klc4	0.495	1.180	0.584	0.000	0.001	0.008	8.975	7.961	8.200	74764	Klc4	chr17	-	NM_0290	P19215	protein_cc	1200014P	kinesin lig	Klc4
9016	Mrpl2	0.538	1.904	1.025	0.001	0.002	0.835	9.111	8.218	9.147	27398	Mrpl2	chr17	+	NM_0253	P20299	protein_cc	CGI-22| L	mitochond	Mrpl2
9017	Cul7	1.027	1.604	1.648	0.836	0.049	0.188	4.395	4.433	5.115	66515	Cul7	chr17	+	NM_0256	P121	protein_cc	2510004L	cullin 7	Cul7
9018	Rrp36	1.993	0.937	1.868	0.071	0.810	0.256	5.571	6.566	6.473	224823	Rrp36	chr17	-	NM_1448	P9554	protein_cc	.	ribosomal	Rrp36
9019	Klhdc3	0.800	1.234	0.987	0.230	0.201	0.924	7.828	7.506	7.809	71765	Klhdc3	chr17	-	NM_0279	P18051	protein_cc	1300011D	kelch dom	Klhdc3
9020	Mea1	0.954	1.011	0.964	0.686	0.945	0.879	9.040	8.972	8.987	17256	Mea1	chr17	+	NM_0012	P23667	protein_cc	Mea-1	male enha	Mea1
9021	Ppp2r5d	0.941	1.025	0.964	0.479	0.849	0.864	8.793	8.705	8.740	21770	Ppp2r5d	chr17	-	NM_0093	P23232	protein_cc	B'delta| T	protein ph	Ppp2r5d
9022	Pex6	0.455	0.699	0.318	0.001	0.314	0.050	7.757	6.621	6.104	224824	Pex6	chr17	+	NM_1454	P3820	protein_cc	AI132582|	peroxisom	Pex6
9023	Gnmt	0.405	1.009	0.408	0.000	0.951	0.024	15.221	13.916	13.928	14711	Gnmt	chr17	-	NM_0103	P25828	protein_cc	.	glycine N-	Gnmt
9024	Cnpy3	0.814	0.875	0.713	0.194	0.479	0.179	9.493	9.197	9.005	72029	Cnpy3	chr17	-	NM_0013	P11070	protein_cc	1600025D	canopy FG	Cnpy3
9025	Ptcra	0.516	3.377	1.742	0.190	0.006	0.361	1.003	0.048	1.804	19208	Ptcra	chr17	-	NM_0111	P17270	protein_cc	gp33| pT-	pre T cell a	Ptcra
9026	2310039H	0.799	1.401	1.119	0.087	0.083	0.625	9.280	8.956	9.442	67101	2310039H	chr17	+	NM_0259	P10685	protein_cc	.	RIKEN cDN	2310039H
9027	Rpl7l1	1.924	1.108	2.131	0.002	0.292	0.033	8.706	9.650	9.797	66229	Rpl7l1	chr17	-	NM_0254	P17543	protein_cc	1500016H	ribosomal	Rpl7l1
9028	Gltscr1l	0.375	1.724	0.646	0.015	0.037	0.232	7.197	5.781	6.567	210982	Gltscr1l	chr17	-	NM_0011	P3150	protein_cc	mKIAA024	GLTSCR1-l	Gltscr1l
9029	Tbcc	2.185	0.950	2.076	0.007	0.660	0.005	7.261	8.389	8.315	72726	Tbcc	chr17	+	NM_1783	P24769	protein_cc	2810055C	tubulin-sp	Tbcc
9030	Prph2	1.000	1.000	1.000	1.000	1.000	1.000	0.000	0.000	0.000	19133	Prph2	chr17	+	NM_0089	P20623	protein_cc	AOFMD|	peripherin	Prph2
9031	Ubr2	0.488	0.685	0.334	0.002	0.023	0.036	9.674	8.638	8.093	224826	Ubr2	chr17	-	NM_1460	P16823	protein_cc	9930021A	ubiquitin	Ubr2
9032	Trerf1	0.567	1.851	1.050	0.186	0.185	0.947	0.845	0.027	0.916	224829	Trerf1	chr17	+	NM_0010	P17184	protein_cc	9430096I1	transcripti	Trerf1
9033	Mrps10	0.781	1.196	0.934	0.429	0.415	0.847	7.784	7.426	7.684	64657	Mrps10	chr17	+	NM_0011	P18144	protein_cc	1110038B	mitochond	Mrps10
9034	Guca1b	1.146	0.632	0.725	0.729	1.000	0.422	0.465	0.661	0.000	107477	Guca1b	chr17	+	NM_1460	P21527	protein_cc	BC018258	guanylate	Guca1b
9035	Guca1a	1.577	1.035	1.632	0.151	0.819	0.329	3.727	4.384	4.433	14913	Guca1a	chr17	-	NM_0081	P25154	protein_cc	GC-A| Gc	guanylate	Guca1a
9036	1700001C	0.110	2.260	0.250	0.001	0.007	0.039	7.092	3.913	5.090	75462	1700001C	chr17	-	NM_0292	P12630	protein_cc	.	RIKEN cDN	1700001C
9037	AI661453	0.396	1.885	0.747	0.001	0.010	0.014	6.947	5.613	6.527	224833	AI661453	chr17	+	NM_1454	P4967	protein_cc	.	expressed	AI661453
9038	Taf8	1.472	0.685	1.009	0.250	0.201	0.979	5.363	5.920	5.375	63856	Taf8	chr17	-	NM_0220	P3987	protein_cc	AW26025	TATA-box	Taf8
9039	Ccnd3	0.946	0.986	0.934	0.700	0.943	0.858	8.898	8.819	8.799	12445	Ccnd3	chr17	+	NM_0010	P3606	protein_cc	9230106B	cyclin D3	Ccnd3
9040	Bysl	1.194	1.053	1.257	0.205	0.638	0.463	9.138	9.393	9.468	53414	Bysl	chr17	-	NM_0168	P15213	protein_cc	Bys| Enp1	bystin-like	Bysl
9041	Med20	1.897	0.991	1.879	0.016	0.952	0.093	6.648	7.571	7.557	56771	Med20	chr17	+	NM_0200	P5906	protein_cc	1110011O	mediator	Med20
9042	Usp49	2.266	0.708	1.604	0.030	0.333	0.534	4.061	5.241	4.743	224836	Usp49	chr17	+	NM_1984	P5631	protein_cc	C330046L	ubiquitin s	Usp49
9043	Tomm6	0.993	1.162	1.154	0.950	0.019	0.428	10.500	10.490	10.707	66119	Tomm6	chr17	-	NM_0253	P12663	protein_cc	1110002E	translocas	Tomm6
9044	Tomm6os	1.589	0.635	1.009	0.468	0.475	0.983	0.439	1.107	0.452	10041578	Tomm6os	chr17	+	NR_04594	.	lncRNA	Gm14872	translocas	Tomm6os
9045	Prickle4	0.504	0.990	0.499	0.181	1.000	0.423	1.002	0.015	0.000	381104	Prickle4	chr17	-	NM_0012	P20667	protein_cc	Gm939	prickle pla	Prickle4
9046	Frs3	0.669	0.995	0.666	0.181	1.000	0.423	0.587	0.008	0.000	107971	Frs3	chr17	+	NM_1449	P21292	protein_cc	4930417B	fibroblast	Frs3
9047	Frs3os	1.000	1.000	1.000	1.000	1.000	1.000	0.000	0.000	0.000	10012624	Frs3os	chr17	-	NR_04591	.	lncRNA	4930417B	fibroblast	Frs3os
9048	Pgc	0.549	2.286	1.254	0.001	0.184	0.759	0.900	0.033	1.226	109820	Pgc	chr17	+	NM_0259	P6369	protein_cc	2210410L	progastric	Pgc
9049	Tfeb	0.951	1.297	1.234	0.784	0.407	0.714	4.582	4.510	4.885	21425	Tfeb	chr17	+	NM_0011	P14828	protein_cc	bHLHe35|	transcripti	Tfeb
9050	Mdfi	3.008	2.209	6.645	0.223	0.160	0.161	1.753	3.342	4.485	17240	Mdfi	chr17	-	NM_0012	P11776	protein_cc	I-mf| I-mf	MyoD fam	Mdfi
9051	Foxp4	2.290	0.823	1.885	0.005	0.458	0.365	6.243	7.438	7.158	74123	Foxp4	chr17	-	NM_0011	P11955	protein_cc	1200010K	forkhead	Foxp4
9052	1700122O	0.716	0.995	0.712	0.182	1.000	0.423	0.490	0.008	0.000	76651	1700122O	chr17	-	NM_0296	P26170	protein_cc	.	RIKEN cDN	1700122O
9053	A730006G	1.010	1.825	1.844	0.000	0.182	0.423	0.000	0.015	0.883	320638	A730006G	chr17	-	NR_11048	.	lncRNA	.	RIKEN cDN	A730006G
9054	1700067P	1.000	1.000	1.000	1.000	1.000	1.000	0.000	0.000	0.000	68224	1700067P	chr17	+	NM_0266	P22221	protein_cc	.	RIKEN cDN	1700067P
9055	9830107B	1.609	1.912	3.077	0.290	0.343	0.423	0.001	0.687	1.622	328829	9830107B	chr17	-	NM_0011	P1548	protein_cc	Trem5	RIKEN cDN	9830107B
9056	A530064D	3.743	0.980	3.668	0.024	0.923	0.038	1.018	2.923	2.894	328830	A530064D	chr17	-	NM_1787	P8129	protein_cc	pdctrem|	RIKEN cDN	A530064D
9057	Trem1	79.215	0.462	36.614	0.001	0.006	0.003	0.003	6.311	5.197	58217	Trem1	chr17	+	NM_0214	P19380	protein_cc	.	triggering	Trem1
9058	Trem3	24.487	1.315	32.204	0.001	0.641	0.332	0.003	4.617	5.012	58218	Trem3	chr17	+	NM_0214	P14136	protein_cc	BB134760	triggering	Trem3
9059	Treml4	1.151	0.624	0.718	0.619	0.320	0.584	4.543	4.747	4.066	224840	Treml4	chr17	+	NM_1726	P13634	protein_cc	5031403H	triggering	Treml4
9060	Treml2	6.171	0.260	1.607	0.013	0.020	0.327	2.970	5.596	3.654	328833	Treml2	chr17	+	NM_0010	P17723	protein_cc	AW04930	triggering	Treml2
9061	B430306N	4.654	0.574	2.670	0.005	0.041	0.058	3.403	5.622	4.820	320148	B430306N	chr17	+	NM_1770	P9490	protein_cc	Treml6	RIKEN cDN	B430306N
9062	Trem2	0.121	0.944	0.114	0.005	1.000	0.078	3.128	0.083	0.000	83433	Trem2	chr17	+	NM_0012	P5704	protein_cc	TREM-2|	triggering	Trem2
9063	Treml1	21.343	0.416	8.886	0.003	0.019	0.004	0.002	4.418	3.154	71326	Treml1	chr17	+	NM_0012	P20070	protein_cc	5430401J1	triggering	Treml1
9064	Nfya	2.664	0.705	1.878	0.047	0.305	0.358	4.469	5.883	5.378	18044	Nfya	chr17	-	NM_0011	P24057	protein_cc	AA407810	nuclear tr	Nfya
9065	Oard1	0.850	0.949	0.807	0.453	0.819	0.173	7.201	6.967	6.891	106821	Oard1	chr17	+	NM_0012	P18038	protein_cc	AI314976|	O-acyl-AD	Oard1
9066	Apobec2	1.000	1.000	1.000	1.000	1.000	1.000	0.000	0.000	0.000	11811	Apobec2	chr17	-	NM_0096	P21716	protein_cc	Arp1	apolipopr	Apobec2
9067	Tspo2	3.420	0.222	0.758	0.183	1.000	0.421	0.400	2.174	0.000	70026	Tspo2	chr17	-	NM_0272	P22578	protein_cc	2510027D	translocat	Tspo2
9068	Unc5cl	2.188	1.203	2.631	0.301	0.681	0.003	0.001	1.130	1.396	76589	Unc5cl	chr17	+	NM_1528	P18594	protein_cc	2510009H	unc-5 fam	Unc5cl
9069	Lrfn2	1.000	1.000	1.000	1.000	1.000	1.000	0.000	0.000	0.000	70530	Lrfn2	chr17	+	NM_0274	P10081	protein_cc	5730420O	leucine ric	Lrfn2
9070	1700008K	1.000	1.000	1.000	1.000	1.000	1.000	0.000	0.000	0.000	69311	1700008K	chr17	-	NR_03814	.	lncRNA	.	RIKEN cDN	1700008K
9071	Gm17830	2.149	0.465	1.000	0.306	1.000	0.423	0.000	1.104	0.000	10041578	Gm17830	chr17	-	NR_03348	.	lncRNA	.	predicted	Gm17830
9072	Mocs1	1.430	1.173	1.677	0.003	0.527	0.359	9.446	9.961	10.191	56738	Mocs1	chr17	+	NM_0284	P20554	protein_cc	3110045D	molybden	Mocs1
9073	Daam2	0.585	1.866	1.092	0.002	0.118	0.846	4.491	3.718	4.618	76441	Daam2	chr17	-	NM_0010	P272	protein_cc	2310016D	dishevelle	Daam2
9074	Kif6	1.000	1.000	1.000	1.000	1.000	1.000	0.000	0.000	0.000	319991	Kif6	chr17	+	NM_1770	P6130	protein_cc	D130004B	kinesin far	Kif6
9075	Rftn1	2.292	0.905	2.074	0.021	0.658	0.155	4.656	5.853	5.708	76438	Rftn1	chr17	-	NM_1813	P25449	protein_cc	2310015N	raftlin lipi	Rftn1
9076	AY702103	1.000	1.000	1.000	1.000	1.000	1.000	0.000	0.000	0.000	446210	AY702103	chr17	+	NR_13197	.	lncRNA	STDP2	cDNA sequ	AY702103
9077	Dazl	0.451	0.986	0.444	0.182	1.000	0.423	1.170	0.021	0.000	13164	Dazl	chr17	-	NM_0012	P24476	protein_cc	Daz-like|	deleted in	Dazl
9078	Plcl2	0.897	0.948	0.851	0.576	0.794	0.209	6.591	6.435	6.358	224860	Plcl2	chr17	+	NM_0138	P16227	protein_cc	mKIAA10	phospholi	Plcl2
9079	Btg3	15.042	1.123	16.898	0.000	0.745	0.209	4.473	8.384	8.552	12228	Btg3	chr17	+	NM_0012	P1062	protein_cc	ANA| tob	B cell tran	Btg3
9080	Tbc1d5	0.501	0.946	0.474	0.037	0.868	0.103	6.213	5.216	5.136	72238	Tbc1d5	chr17	-	NM_0012	P23816	protein_cc	1600014N	TBC1 dom	Tbc1d5
9081	C330011F	1.000	1.000	1.000	1.000	1.000	1.000	0.000	0.000	0.000	328837	C330011F	chr17	-	NR_04616	.	lncRNA	C330011F	RIKEN cDN	C330011F
9082	Satb1	1.235	0.370	0.456	0.486	0.003	0.363	4.629	4.933	3.497	20230	Satb1	chr17	-	NM_0091	P20397	protein_cc	2610306G	special AT	Satb1
9083	Gm20098	1.000	1.000	1.000	1.000	1.000	1.000	0.000	0.000	0.000	10050416	Gm20098	chr17	+	NR_04509	.	lncRNA	.	predicted	Gm20098
9084	Kcnh8	1.000	1.000	1.000	1.000	1.000	1.000	0.000	0.000	0.000	211468	Kcnh8	chr17	+	NM_0010	P5195	protein_cc	C130090D	potassium	Kcnh8
9085	Efhb	1.000	1.000	1.000	1.000	1.000	1.000	0.000	0.000	0.000	211482	Efhb	chr17	-	NM_1724	P24167	protein_cc	4921525D	EF hand d	Efhb
9086	Rab5a	1.368	0.981	1.343	0.068	0.918	0.376	7.584	8.037	8.010	271457	Rab5a	chr17	+	NM_0258	P11445	protein_cc	2410015H	RAB5A, m	Rab5a
9087	Pp2d1	1.308	1.036	1.355	0.123	0.868	0.425	7.397	7.784	7.835	110332	Pp2d1	chr17	-	NM_1734	P21835	protein_cc	4921523A	protein ph	Pp2d1

9088	Kat2b	0.239	1.986	0.474	0.000	0.001	0.039	9.389	7.321	8.311	18519	Kat2b	chr17	+	NM_0200	P23926	protein_cc	A930006P	K(lysine) a	Kat2b
9089	Sgol1	1.731	0.199	0.344	0.229	0.005	0.477	2.002	2.794	0.461	72415	Sgol1	chr17	-	NM_0282	P4608	protein_cc	3300001M	shugoshin	Sgol1
9090	4932415M	1.000	1.000	1.000	1.000	1.000	1.000	0.000	0.000	0.000	211496	4932415M	chr17	+	NR_07320	.	lncRNA		RIKEN cDN	4932415M
9091	Sult1c2	0.042	8.113	0.340	0.005	0.196	0.258	5.809	1.235	4.255	69083	Sult1c2	chr17	-	NM_0269	P11239	protein_cc	1810008N	sulfotrans	Sult1c2
9092	Sult1c1	1.000	1.000	1.000	1.000	1.000	1.000	0.000	0.000	0.000	20888	Sult1c1	chr17	-	NM_0187	P11684	protein_cc	(PST)G| m	sulfotrans	Sult1c1
9093	Slc5a7	1.000	1.000	1.000	1.000	1.000	1.000	0.000	0.000	0.000	63993	Slc5a7	chr17	-	NM_0220	P25341	protein_cc	CHT1	solute car	Slc5a7
9094	1700025K	1.000	1.000	1.000	1.000	1.000	1.000	0.000	0.000	0.000	69418	1700025K	chr17	-	NR_04582	.	lncRNA	.	RIKEN cDN	1700025K2
9095	St6gal2	1.000	1.000	1.000	1.000	1.000	1.000	0.000	0.000	0.000	240119	St6gal2	chr17	+	NM_1728	P25087	protein_cc	C230064G	beta galac	St6gal2
9096	Vmn2r118	1.000	1.000	1.000	1.000	1.000	1.000	0.000	0.000	0.000	383258	Vmn2r118	chr17	-	NM_0011	P6291	protein_cc	EG383258	vomerona	Vmn2r118
9097	Pot1b	3.066	0.724	2.221	0.137	0.545	0.365	1.408	3.025	2.559	72836	Pot1b	chr17	-	NM_0283	P15886	protein_cc	2810458H	protection	Pot1b
9098	Adgre4	0.571	0.445	0.254	0.038	0.036	0.105	5.314	4.507	3.338	52614	Adgre4	chr17	+	NM_1391	P9036	protein_cc	D17Ertd47	adhesion	Adgre4
9099	Zfp119a	1.316	1.208	1.590	0.667	0.689	0.360	4.348	4.744	5.016	104349	Zfp119a	chr17	-	NM_1445	P17649	protein_cc	AL024077	zinc finger	Zfp119a
9100	Zfp959	1.440	1.002	1.443	0.038	0.994	0.448	5.576	6.102	6.105	224893	Zfp959	chr17	+	NM_1454	P20115	protein_cc	.	zinc finger	Zfp959
9101	Zfp119b	2.760	0.419	1.157	0.067	0.080	0.764	4.010	5.474	4.221	240120	Zfp119b	chr17	-	NM_1462	P25640	protein_cc	.	zinc finger	Zfp119b
9102	Ebi3	24.707	0.426	10.524	0.001	0.005	0.007	2.531	7.158	5.927	50498	Ebi3	chr17	+	NM_0157	P6713	protein_cc	EBI-3| IL-2	Epstein-Ba	Ebi3
9103	Gm16712	25.557	0.416	10.643	0.003	0.017	0.010	2.335	7.011	5.747	10050433	Gm16712	chr17	-	NR_10802	.	lncRNA	.	predicted	Gm16712
9104	Ccdc94	1.471	1.567	2.306	0.302	0.128	0.074	4.735	5.292	5.941	72886	Ccdc94	chr17	+	NM_0283	P26514	protein_cc	2900016D	coiled-coil	Ccdc94
9105	Shd	0.716	0.995	0.712	0.182	1.000	0.423	0.490	0.008	0.000	20420	Shd	chr17	+	NM_0011	P20030	protein_cc	AI413439	src homol	Shd
9106	Fsd1	2.134	0.469	1.000	0.305	1.000	0.423	0.000	1.094	0.000	240121	Fsd1	chr17	+	NM_1831	P4123	protein_cc	.	fibronecti	Fsd1
9107	Stap2	4.754	0.935	4.443	0.000	0.196	0.005	7.260	9.509	9.411	106766	Stap2	chr17	-	NM_1459	P18665	protein_cc	AW04976	signal trar	Stap2
9108	Mpnd	0.855	1.368	1.169	0.268	0.016	0.437	9.156	8.929	9.381	68047	Mpnd	chr17	+	NM_0265	P3363	protein_cc	E130307M	MPN dom	Mpnd
9109	Sh3gl1	1.154	1.203	1.388	0.767	0.631	0.354	7.442	7.648	7.914	20405	Sh3gl1	chr17	-	NM_0012	P13500	protein_cc	C77078| E	SH3-doma	Sh3gl1
9110	Chaf1a	0.273	3.229	0.883	0.014	0.072	0.829	4.125	2.254	3.945	27221	Chaf1a	chr17	+	NM_0137	P17112	protein_cc	AL023013	chromatin	Chaf1a
9111	Ubxn6	0.521	1.722	0.898	0.001	0.003	0.293	8.673	7.733	8.517	66530	Ubxn6	chr17	-	NM_0244	P11499	protein_cc	1200008L	UBX doma	Ubxn6
9112	Hdgfrp2	0.792	1.093	0.865	0.019	0.631	0.606	8.872	8.534	8.663	15193	Hdgfrp2	chr17	+	NM_0013	P14051	protein_cc	HRP-2	hepatoma	Hdgfrp2
9113	Plin4	0.957	1.444	1.383	0.801	0.178	0.375	7.050	6.987	7.518	57435	Plin4	chr17	-	NM_0205	P18425	protein_cc	mKIAA188	perilipin 4	Plin4
9114	Plin5	0.094	7.334	0.666	0.000	0.009	0.281	9.211	5.750	8.624	66968	Plin5	chr17	-	NM_0258	P13471	protein_cc	2310076L	perilipin 5	Plin5
9115	Lrg1	12.613	0.833	10.505	0.000	0.022	0.005	12.337	15.994	15.730	76905	Lrg1	chr17	-	NM_0297	P18541	protein_cc	1300008B	leucine-ric	Lrg1
9116	Sema6b	9.951	0.923	9.185	0.003	0.628	0.011	3.651	6.966	6.851	20359	Sema6b	chr17	-	NM_0136	P1670	protein_cc	Sema| Ser	sema dom	Sema6b
9117	Tnfaip8l1	0.161	1.492	0.240	0.002	0.523	0.070	8.106	5.468	6.046	66443	Tnfaip8l1	chr17	+	NM_0255	P26769	protein_cc	2600017J	tumor nec	Tnfaip8l1
9118	Mydgf	0.996	0.934	0.930	0.958	0.538	0.711	9.294	9.288	9.190	28106	Mydgf	chr17	-	NM_0808	P19777	protein_cc	D17Wsu1	myeloid d	Mydgf
9119	Dpp9	3.978	0.455	1.809	0.000	0.001	0.170	8.974	10.967	9.830	224897	Dpp9	chr17	-	NM_1726	P13228	protein_cc	6430584G	dipeptidyl	Dpp9
9120	Fem1a	1.124	1.112	1.250	0.228	0.217	0.039	8.199	8.368	8.521	14154	Fem1a	chr17	+	NM_0101	P24669	protein_cc	AW61139	feminizati	Fem1a
9121	Ticam1	1.639	0.820	1.344	0.001	0.285	0.472	6.222	6.935	6.648	106759	Ticam1	chr17	-	NM_1749	P17011	protein_cc	AW04601	toll-like re	Ticam1
9122	Plin3	0.805	1.188	0.956	0.068	0.346	0.842	9.341	9.028	9.276	66905	Plin3	chr17	-	NM_0258	P6480	protein_cc	1300012C	perilipin 3	Plin3
9123	Arrdc5	1.000	1.000	1.000	1.000	1.000	1.000	0.000	0.000	0.000	76920	Arrdc5	chr17	-	NM_0297	P7746	protein_cc	1700013E	arrestin d	Arrdc5
9124	Uhrf1	3.464	1.140	3.948	0.243	0.817	0.168	0.732	2.525	2.713	18140	Uhrf1	chr17	+	NM_0109	P15315	protein_cc	AL022808	ubiquitin-l	Uhrf1
9125	Kdm4b	1.287	0.810	1.043	0.419	0.492	0.853	5.714	6.078	5.774	193796	Kdm4b	chr17	+	NM_1721	P6457	protein_cc	4732474L	lysine (K)-	Kdm4b
9126	Ptprs	0.338	0.801	0.271	0.069	0.330	0.233	4.399	2.836	2.516	19280	Ptprs	chr17	-	NM_0112	P5053	protein_cc	AL022616	protein ty	Ptprs
9127	Znrf4	1.000	1.000	1.000	1.000	1.000	1.000	0.000	0.000	0.000	20834	Znrf4	chr17	-	NM_0114	P3759	protein_cc	1700094M	zinc and ri	Znrf4
9128	Gm20219	1.005	1.439	1.446	0.000	0.182	0.423	0.000	0.008	0.532	10050442	Gm20219	chr17	-	NR_13012	.	lncRNA	Tincr	predicted	Gm20219
9129	Safb2	1.296	0.570	0.738	0.000	0.000	0.012	8.341	8.716	7.903	224902	Safb2	chr17	-	NM_0010	P4828	protein_cc	AA389433	scaffold at	Safb2
9130	Safb	1.427	0.591	0.844	0.002	0.000	0.108	7.929	8.442	7.685	224903	Safb	chr17	+	NM_0011	P7450	protein_cc	3110021E	scaffold at	Safb
9131	2410015M	0.737	1.232	0.908	0.000	0.013	0.284	9.607	9.167	9.469	224904	2410015M	chr17	-	NM_1531	P19918	protein_cc	Mic13| QI	RIKEN cDN	2410015M
9132	Rpl36	0.962	1.448	1.393	0.694	0.011	0.032	10.000	9.944	10.478	54217	Rpl36	chr17	+	NM_0187	P9821	protein_cc	.	ribosomal	Rpl36
9133	Lonp1	0.719	0.840	0.604	0.001	0.018	0.005	9.990	9.515	9.264	74142	Lonp1	chr17	-	NM_0287	P22409	protein_cc	1200017E	lon peptid	Lonp1
9134	Catsperd	0.191	3.052	0.581	0.004	0.010	0.130	3.589	1.197	2.807	106757	Catsperd	chr17	+	NM_1753	P9136	protein_cc	4921529N	catsper ch	Catsperd
9135	Ranbp3	1.513	0.846	1.280	0.022	0.061	0.303	7.355	7.952	7.712	71810	Ranbp3	chr17	+	NM_0012	P6341	protein_cc	2610024N	RAN bindi	Ranbp3
9136	Vmac	0.351	1.960	0.688	0.005	0.069	0.396	6.662	5.150	6.121	106639	Vmac	chr17	-	NM_0011	P23326	protein_cc	AI662250	vimentin-t	Vmac
9137	Ndufa11	0.667	1.753	1.170	0.002	0.000	0.035	9.452	8.869	9.679	69875	Ndufa11	chr17	+	NM_0272	P7959	protein_cc	2010012C	NADH deh	Ndufa11
9138	Fut4-ps1	1.000	1.000	1.000	1.000	1.000	1.000	0.000	0.000	0.000	14346	Fut4-ps1	chr17	+	NR_03364	.	misc_RNA	.	fucosyltra	Fut4-ps1
9139	Nrtn	0.657	3.190	2.097	0.486	0.046	0.134	2.844	2.239	3.912	18188	Nrtn	chr17	-	NM_0087	P2642	protein_cc	NTN	neurturin	Nrtn
9140	Dus3l	0.834	1.037	0.865	0.562	0.909	0.683	5.475	5.214	5.266	224907	Dus3l	chr17	+	NM_1448	P3947	protein_cc	AI662135|	dihydrour	Dus3l
9141	Prr22	1.589	1.138	1.809	0.298	0.679	0.003	0.000	0.669	0.855	10050444	Prr22	chr17	+	NM_0011	P8603	protein_cc	Gm546	proline ric	Prr22
9142	Rfx2	0.817	1.237	1.010	0.006	0.033	0.936	2.513	2.221	2.528	19725	Rfx2	chr17	-	NM_0090	P528	protein_cc	5430432H	regulatory	Rfx2
9143	Acsbg2	1.000	1.000	1.000	1.000	1.000	1.000	0.000	0.000	0.000	328845	Acsbg2	chr17	-	NM_0010	P26043	protein_cc	Bgr	acyl-CoA s	Acsbg2
9144	1700061G	1.000	1.000	1.000	1.000	1.000	1.000	0.000	0.000	0.000	78625	1700061G	chr17	+	NM_0301	P18962	protein_cc	1700026H	RIKEN cDN	1700061G
9145	Mllt1	1.533	0.931	1.427	0.016	0.340	0.119	5.444	6.060	5.957	64144	Mllt1	chr17	-	NM_0223	P14623	protein_cc	AA407901	myeloid/l	Mllt1
9146	Acer1	0.504	0.990	0.499	0.181	1.000	0.423	1.002	0.015	0.000	171168	Acer1	chr17	-	NM_1757	P15660	protein_cc	2310024P	alkaline ce	Acer1
9147	Clpp	0.953	1.067	1.017	0.060	0.488	0.911	8.980	8.911	9.004	53895	Clpp	chr17	+	NM_0173	P4511	protein_cc	AU01982C	caseinolyt	Clpp
9148	Alkbh7	0.428	1.551	0.664	0.006	0.211	0.263	7.270	6.047	6.680	66400	Alkbh7	chr17	+	NM_0273	P6626	protein_cc	2310045B	alkB homo	Alkbh7
9149	Pspn	1.005	1.405	1.412	0.000	0.182	0.423	0.000	0.008	0.498	19197	Pspn	chr17	-	NM_0089	P17529	protein_cc	PSP	persephin	Pspn
9150	Gtf2f1	2.734	0.743	2.033	0.020	0.194	0.034	7.226	8.677	8.249	98053	Gtf2f1	chr17	-	NM_1338	P17069	protein_cc	2810405K	general tra	Gtf2f1
9151	Khsrp	1.289	0.760	0.980	0.010	0.070	0.923	8.175	8.541	8.145	16549	Khsrp	chr17	-	NM_0106	P8093	protein_cc	6330409F	KH-type sp	Khsrp
9152	Slc25a41	1.000	1.000	1.000	1.000	1.000	1.000	0.000	0.000	0.000	103775	Slc25a41	chr17	-	NM_1753	P5877	protein_cc	4933406J	solute car	Slc25a41
9153	Slc25a23	0.062	1.573	0.098	0.000	0.218	0.003	10.714	6.714	7.367	66972	Slc25a23	chr17	-	NM_0258	P23399	protein_cc	2310067G	solute car	Slc25a23
9154	Crb3	0.465	1.274	0.593	0.003	0.197	0.041	7.152	6.048	6.397	224912	Crb3	chr17	+	NM_1776	P18283	protein_cc	5730439B	crumbs fa	Crb3
9155	Dennd1c	2.915	0.421	1.229	0.022	0.037	0.805	2.751	4.294	3.048	70785	Dennd1c	chr17	-	NM_1535	P158	protein_cc	4432409M	DENN/MA	Dennd1c
9156	Tubb4a	1.015	2.253	2.285	0.000	0.015	0.125	0.000	0.021	1.193	22153	Tubb4a	chr17	-	NM_0094	P22806	protein_cc	AI325297|	tubulin, be	Tubb4a
9157	Gm11110	1.598	1.135	1.813	0.300	0.776	0.423	0.000	0.677	0.859	10016987	Gm11110	chr17	-	NR_03350	.	lncRNA	ENSMUSG	predicted	Gm11110
9158	Tnfsf9	1.000	1.000	1.000	1.000	1.000	1.000	0.000	0.000	0.000	21950	Tnfsf9	chr17	+	NM_0094	P21394	protein_cc	4-1BB-L| 4	tumor nec	Tnfsf9
9159	Cd70	1.000	1.000	1.000	1.000	1.000	1.000	0.000	0.000	0.000	21948	Cd70	chr17	-	NM_0116	P21134	protein_cc	Cd27l| CD	CD70 anti	Cd70
9160	Tnfsf14	3.660	0.669	2.450	0.158	0.504	0.378	1.264	3.136	2.557	50930	Tnfsf14	chr17	-	NM_0194	P1079	protein_cc	HVEM-L| I	tumor nec	Tnfsf14
9161	C3	1.197	0.791	0.947	0.046	0.046	0.688	15.389	15.649	15.311	12266	C3	chr17	-	NM_0097	P3784	protein_cc	AI255234|	compleme	C3
9162	Gpr108	0.492	1.421	0.699	0.001	0.208	0.289	7.950	6.927	7.433	78308	Gpr108	chr17	-	NM_0013	P116	protein_cc	1810015L	G protein-	Gpr108
9163	Trip10	1.714	1.395	2.391	0.027	0.410	0.351	5.247	6.024	6.504	106628	Trip10	chr17	+	NM_0012	P12154	protein_cc	AI646975|	thyroid ho	Trip10
9164	Vav1	3.456	0.458	1.583	0.002	0.006	0.010	4.885	6.674	5.547	22324	Vav1	chr17	+	NM_0011	P6700	protein_cc	Vav| vav-1	vav 1 onco	Vav1
9165	Adgre1	0.982	0.701	0.689	0.934	0.288	0.282	7.150	7.124	6.612	13733	Adgre1	chr17	+	NM_0101	P20544	protein_cc	DD7A5-7|	adhesion	Adgre1
9166	Vmn2r120	1.000	1.000	1.000	1.000	1.000	1.000	0.000	0.000	0.000	224916	Vmn2r120	chr17	-	NM_0011	P19582	protein_cc	EG224916	vomerona	Vmn2r120
9167	Cntnap5c	1.000	1.000	1.000	1.000	1.000	1.000	0.000	0.000	0.000	620292	Cntnap5c	chr17	+	NM_0010	P1794	protein_cc	Caspr5-3|	contactin	Cntnap5c
9168	Pdzph1	1.000	1.000	1.000	1.000	1.000	1.000	0.000	0.000	0.000	69239	Pdzph1	chr17	-	NM_0270	P420	protein_cc	2610034M	PDZ and p	Pdzph1
9169	Nudt12	0.930	0.935	0.870	0.866	0.873	0.813	3.873	3.769	3.672	67993	Nudt12	chr17	-	NM_0264	P11276	protein_cc	0610016O	nudix (nuc	Nudt12
9170	Efna5	1.316	1.199	1.579	0.346	0.289	0.431	6.443	6.840	7.102	13640	Efna5	chr17	-	NM_2076	P10876	protein_cc	AL-1| AV1	ephrin A5	Efna5
9171	Fbxl17	0.316	1.644	0.519	0.000	0.041	0.058	7.988	6.325	7.042	50758	Fbxl17	chr17	-	NM_0157	P24453	protein_cc	6330576B	F-box and	Fbxl17
9172	4930405O	1.000	1.000	1.000	1.000	1.000	1.000	0.000	0.000	0.000	414079	4930405O	chr17	-	NR_13017	.	lncRNA	.	RIKEN cDN	4930405O
9173	Fer	1.514	0.859	1.300	0.000	0.177	0.275	6.780	7.379	7.159	14158	Fer	chr17	+	NM_0010	P20502	protein_cc	AV082135	fer (fms/fp	Fer
9174	1110058D	0.570	0.457	0.260	0.376	1.000	0.322	1.942	1.129	0.000	68771	1110058D	chr17	-	NR_13098	.	lncRNA	.	RIKEN cDN	1110058D
9175	Pja2	1.270	0.818	1.039	0.131	0.047	0.877	8.353	8.698	8.408	224938	Pja2	chr17	-	NM_1448	P8193	protein_cc	AI447901|	praja 2, RI	Pja2
9176	AU016765	1.000	1.000	1.000	1.000	1.000	1.000	0.000	0.000	0.000	10004358	AU016765	chr17	-	NR_04589	.	lncRNA	.	expressed	AU016765
9177	Man2a1	0.852	0.981	0.836	0.292	0.881	0.303	10.129	9.897	9.870	17158	Man2a1	chr17	+	NM_0085	P11911	protein_cc	Mana-2| M	mannosida	Man2a1
9178	Dreh	0.599	16.113	9.649	0.466	0.000	0.028	2.311	1.571	5.581	10120268	Dreh	chr17	+	NR_10505	.	lncRNA	.	down-regu	Dreh

9179	Tmem232	1.000	1.000	1.000	1.000	1.000	1.000	0.000	0.000	0.000	381107	Tmem232	chr17	-	NM_0012	P23447	protein_cc	E130009J1	transmem	Tmem232
9180	Vapa	1.208	1.062	1.284	0.000	0.211	0.077	10.165	10.438	10.525	30960	Vapa	chr17	-	NM_0139	P10315	protein_cc	33kDa| VA	vesicle-ass	Vapa
9181	Txndc2	1.000	1.000	1.000	1.000	1.000	1.000	0.000	0.000	0.000	213272	Txndc2	chr17	-	NM_1535	P18049	protein_cc	AU021712	thioredoxi	Txndc2
9182	Rab31	3.241	1.414	4.583	0.028	0.214	0.073	5.249	6.945	7.445	106572	Rab31	chr17	-	NM_1336	P26471	protein_cc	1700093E	RAB31, m	Rab31
9183	Ppp4r1	2.114	1.296	2.740	0.003	0.237	0.125	6.486	7.566	7.940	70351	Ppp4r1	chr17	+	NM_1460	P15168	protein_cc	3110001J1	protein ph	Ppp4r1
9184	Ralbp1	1.089	0.905	0.985	0.281	0.417	0.933	9.599	9.721	9.577	19765	Ralbp1	chr17	-	NM_0011	P1601	protein_cc	Rip1| RLIP	ralA bindi	Ralbp1
9185	Twsg1	0.935	0.886	0.828	0.628	0.520	0.381	7.889	7.792	7.618	65960	Twsg1	chr17	-	NM_0230	P2950	protein_cc	1810013J1	twisted ga	Twsg1
9186	Ankrd12	1.329	0.714	0.949	0.109	0.069	0.718	8.097	8.507	8.021	106585	Ankrd12	chr17	-	NM_0010	P17924	protein_cc	2900001A	ankyrin re	Ankrd12
9187	Ndufv2	0.826	0.925	0.763	0.002	0.040	0.010	10.470	10.194	10.081	72900	Ndufv2	chr17	-	NM_0012	P1506	protein_cc	2900010C	NADH deh	Ndufv2
9188	Wash1	0.755	1.023	0.772	0.034	0.809	0.302	7.830	7.425	7.457	68767	Wash1	chr17	+	NM_0268	P23051	protein_cc	1110049F	WAS prote	Wash1
9189	Ddx11	0.834	0.926	0.773	0.262	0.879	0.739	4.219	3.958	3.847	320209	Ddx11	chr17	+	NM_0010	P7536	protein_cc	4732462I1	DEAD/H (A	Ddx11
9190	Mtcl1	0.321	1.382	0.443	0.000	0.201	0.061	1.678	0.038	0.505	68617	Mtcl1	chr17	-	NM_0011	P22820	protein_cc	1110012J1	microtubu	Mtcl1
9191	Rab12	1.546	0.748	1.156	0.002	0.002	0.312	8.860	9.489	9.069	19328	Rab12	chr17	-	NM_0244	P25133	protein_cc	2900054P	RAB12, m	Rab12
9192	Themis3	1.000	1.000	1.000	1.000	1.000	1.000	0.000	0.000	0.000	74556	Themis3	chr17	-	NM_0289	P23260	protein_cc	9130404H	thymocyte	Themis3
9193	Ptprm	1.104	1.171	1.292	0.176	0.043	0.174	5.754	5.896	6.123	19274	Ptprm	chr17	-	NM_0089	P26030	protein_cc	mKIAA404	protein ty	Ptprm
9194	Lrrc30	1.000	1.000	1.000	1.000	1.000	1.000	0.000	0.000	0.000	240131	Lrrc30	chr17	-	NM_0010	P26854	protein_cc	Gm326	leucine ric	Lrrc30
9195	Lama1	0.287	0.583	0.167	0.036	0.263	0.146	4.630	2.830	2.050	16772	Lama1	chr17	+	NM_0084	P21148	protein_cc	AA408497	laminin, al	Lama1
9196	Arhgap28	0.516	2.554	1.318	0.096	0.277	0.788	1.686	0.731	2.084	268970	Arhgap28	chr17	-	NM_1729	P1700	protein_cc	AU044757	Rho GTPas	Arhgap28
9197	L3mbtl4	0.229	0.969	0.222	0.181	1.000	0.423	2.173	0.045	0.000	320858	L3mbtl4	chr17	+	NM_1772	P3284	protein_cc	A730037L	l(3)mbt-lik	L3mbtl4
9198	Tmem200	1.000	1.000	1.000	1.000	1.000	1.000	0.000	0.000	0.000	622645	Tmem200	chr17	+	NM_0012	P6912	protein_cc	EG622645	transmem	Tmem200
9199	Epb4.1l3	0.201	3.130	0.628	0.060	0.126	0.588	3.530	1.214	2.860	.	Epb4.1l3	chr17	+	NM_0138	P226	.	.	.	Epb4.1l3
9200	2410021H	1.000	1.000	1.000	1.000	1.000	1.000	0.000	0.000	0.000	69750	2410021H	chr17	-	NR_04542	.	lncRNA	.	RIKEN cDN	2410021H
9201	Zbtb14	1.334	1.060	1.415	0.360	0.838	0.335	5.185	5.601	5.685	22666	Zbtb14	chr17	+	NM_0095	P25005	protein_cc	b2b1982C	zinc finger	Zbtb14
9202	C030034I2	1.893	0.638	1.208	0.003	0.024	0.634	4.449	5.370	4.722	77533	C030034I2	chr17	+	NR_02684	.	lncRNA	.	RIKEN cDN	C030034I2
9203	A330050F	1.000	1.000	1.000	1.000	1.000	1.000	0.000	0.000	0.000	.	A330050F	chr17	+	NM_0011	P20839	.	.	.	A330050F
9204	Dlgap1	0.358	1.625	0.582	0.024	0.284	0.311	4.042	2.562	3.262	224997	Dlgap1	chr17	+	NM_0277	P16714	protein_cc	4933422O	discs, larg	Dlgap1
9205	Tgif1	1.668	0.689	1.149	0.002	0.009	0.389	6.861	7.600	7.062	21815	Tgif1	chr17	-	NM_0011	P3180	protein_cc	AA959811	TGFB-indu	Tgif1
9206	Myl12b	0.613	1.297	0.795	0.003	0.048	0.105	8.717	8.011	8.386	67938	Myl12b	chr17	-	NM_0234	P19896	protein_cc	1500001M	myosin, lig	Myl12b
9207	Myl12a	1.852	0.920	1.704	0.000	0.000	0.028	10.135	11.025	10.904	67268	Myl12a	chr17	-	NM_0260	P24334	protein_cc	2900073G	myosin, lig	Myl12a
9208	Myom1	0.234	3.995	0.937	0.142	0.004	0.936	2.846	0.753	2.752	17929	Myom1	chr17	+	NM_0010	P22988	protein_cc	D430047A	myomesin	Myom1
9209	Lpin2	0.088	3.639	0.319	0.000	0.008	0.015	10.688	7.175	9.039	64898	Lpin2	chr17	+	NM_0228	P11584	protein_cc	2610511G	lipin 2	Lpin2
9210	Emilin2	2.193	0.772	1.694	0.035	0.349	0.348	4.108	5.242	4.869	246707	Emilin2	chr17	-	NM_1451	P8174	protein_cc	FOAP-10	elastin mi	Emilin2
9211	Gm4566	1.000	1.000	1.000	1.000	1.000	1.000	0.000	0.000	0.000	10004364	Gm4566	chr17	-	NR_02802	.	misc_RNA	10004364	predicted	Gm4566
9212	Smchd1	1.435	0.742	1.064	0.180	0.219	0.792	6.101	6.622	6.191	74355	Smchd1	chr17	-	NM_0288	P5593	protein_cc	4931400A	SMC hinge	Smchd1
9213	Ndc80	1.584	0.288	0.456	0.677	0.402	0.582	2.465	3.129	1.333	67052	Ndc80	chr17	-	NM_0232	P18805	protein_cc	2610020P	NDC80 kin	Ndc80
9214	Spdya	1.775	1.684	2.989	0.031	0.022	0.066	4.913	5.741	6.493	70891	Spdya	chr17	+	NM_0292	P6540	protein_cc	4921517J0	speedy/RI	Spdya
9215	Trmt61b	1.705	1.865	3.180	0.030	0.002	0.018	5.081	5.851	6.750	68789	Trmt61b	chr17	-	NR_02795	.	lncRNA	1110039F	tRNA met	Trmt61b
9216	Wdr43	2.533	0.781	1.979	0.000	0.005	0.029	8.573	9.914	9.558	72515	Wdr43	chr17	+	NM_1756	P18032	protein_cc	2610318G	WD repea	Wdr43
9217	Snord92	3.371	0.355	1.197	0.007	0.006	0.777	2.005	3.758	2.264	10021746	Snord92	chr17	+	NR_02855	.	snoRNA	MBII-316	small nucl	Snord92
9218	Snord53	2.134	0.469	1.000	0.305	1.000	0.423	0.000	1.094	0.000	10021745	Snord53	chr17	+	NR_02855	.	snoRNA	MBII-35	small nucl	Snord53
9219	Fam179a	1.005	1.405	1.412	0.000	0.182	0.423	0.000	0.008	0.498	320159	Fam179a	chr17	+	NM_1770	P4111	protein_cc	4632412N	family wit	Fam179a
9220	BC027072	1.000	1.000	1.000	1.000	1.000	1.000	0.000	0.000	0.000	225004	BC027072	chr17	-	NM_1460	P24000	protein_cc	.	cDNA seq	BC027072
9221	Clip4	0.552	0.990	0.547	0.182	1.000	0.423	0.871	0.015	0.000	78785	Clip4	chr17	+	NM_1753	P15730	protein_cc	1700024K	CAP-GLY d	Clip4
9222	Alk	1.000	1.000	1.000	1.000	1.000	1.000	0.000	0.000	0.000	11682	Alk	chr17	-	NM_0074	P6207	protein_cc	CD246| Tc	anaplastic	Alk
9223	Ypel5	1.007	1.307	1.316	0.896	0.334	0.558	8.672	8.681	9.068	383295	Ypel5	chr17	+	NM_0271	P25247	protein_cc	2610524G	yippee-lik	Ypel5
9224	Lbh	0.503	0.415	0.209	0.024	0.096	0.040	6.510	5.519	4.251	77889	Lbh	chr17	+	NM_0299	P7856	protein_cc	1810009F	limb-bud a	Lbh
9225	Lclat1	0.388	1.388	0.539	0.002	0.053	0.099	8.211	6.847	7.319	225010	Lclat1	chr17	+	NM_0011	P21061	protein_cc	Agpat8| A	lysocardio	Lclat1
9226	Capn13	1.000	1.000	1.000	1.000	1.000	1.000	0.000	0.000	0.000	381122	Capn13	chr17	-	NM_0010	P16346	protein_cc	CANP 13|	calpain 13	Capn13
9227	Galnt14	0.451	0.986	0.444	0.182	1.000	0.423	1.170	0.021	0.000	71685	Galnt14	chr17	-	NM_0278	P14479	protein_cc	0610033N	UDP-N-ac	Galnt14
9228	Ehd3	0.121	0.445	0.054	0.000	0.001	0.002	10.650	7.597	6.428	57440	Ehd3	chr17	+	NM_0205	P26538	protein_cc	Ehd2	EH-domai	Ehd3
9229	Xdh	4.302	0.739	3.178	0.000	0.058	0.079	8.863	10.968	10.531	22436	Xdh	chr17	-	NM_0117	P8799	protein_cc	XO| Xor| X	xanthine d	Xdh
9230	Srd5a2	0.457	1.358	0.620	0.183	0.195	0.578	1.158	0.027	0.468	94224	Srd5a2	chr17	-	NM_0531	P3015	protein_cc	5ART2| S5	steroid 5 a	Srd5a2
9231	Memo1	0.568	1.891	1.073	0.019	0.001	0.743	7.237	6.421	7.340	76890	Memo1	chr17	-	NM_1337	P7107	protein_cc	0610016J1	mediator	Memo1
9232	Dpy30	0.525	1.472	0.773	0.000	0.134	0.420	8.277	7.348	7.906	66310	Dpy30	chr17	-	NM_0244	P22882	protein_cc	2810410M	dpy-30, hi	Dpy30
9233	Spast	0.514	1.046	0.537	0.017	0.743	0.118	7.930	6.968	7.033	50850	Spast	chr17	+	NM_0169	P23678	protein_cc	mKIAA108	spastin	Spast
9234	Slc30a6	1.191	0.866	1.032	0.450	0.524	0.501	6.885	7.137	6.930	210148	Slc30a6	chr17	+	NM_0012	P23094	protein_cc	9530029F	solute car	Slc30a6
9235	Nlrc4	3.489	0.287	1.001	0.004	0.001	0.999	2.488	4.291	2.489	268973	Nlrc4	chr17	-	NM_0010	P15830	protein_cc	9530011P	NLR family	Nlrc4
9236	Yipf4	1.014	0.824	0.836	0.956	0.514	0.299	8.194	8.215	7.935	67864	Yipf4	chr17	+	NM_0264	P5829	protein_cc	2310034L	Yip1 doma	Yipf4
9237	Birc6	0.823	0.889	0.732	0.245	0.507	0.344	9.146	8.865	8.695	12211	Birc6	chr17	+	NM_0075	P12722	protein_cc	A430032G	baculovira	Birc6
9238	Ttc27	0.794	0.971	0.771	0.375	0.910	0.323	6.021	5.689	5.646	74196	Ttc27	chr17	+	NM_1528	P16079	protein_cc	2610511O	tetratrico	Ttc27
9239	Ltbp1	0.622	1.157	0.719	0.164	0.349	0.493	4.801	4.115	4.325	268977	Ltbp1	chr17	+	NM_0199	P25121	protein_cc	9430031G	latent tra	Ltbp1
9240	Rasgrp3	0.115	3.021	0.347	0.000	0.080	0.076	7.148	4.025	5.620	240168	Rasgrp3	chr17	+	NM_0011	P9728	protein_cc	BC066069	RAS, guan	Rasgrp3
9241	Fam98a	1.496	0.815	1.219	0.079	0.262	0.164	7.261	7.841	7.547	72722	Fam98a	chr17	-	NM_1337	P9832	protein_cc	2810405J0	family wit	Fam98a
9242	Gm4710	1.000	1.000	1.000	1.000	1.000	1.000	0.000	0.000	0.000	10004388	Gm4710	chr17	+	NR_03345	.	lncRNA	10004388	predicted	Gm4710
9243	Crim1	0.797	0.911	0.727	0.070	0.630	0.397	7.802	7.475	7.341	50766	Crim1	chr17	+	NM_0158	P26818	protein_cc	AU015004	cysteine ri	Crim1
9244	Fez2	1.687	1.291	2.178	0.003	0.001	0.027	7.488	8.242	8.610	225020	Fez2	chr17	-	NM_0012	P24382	protein_cc	9030616F	fasciculati	Fez2
9245	Vit	0.308	1.060	0.326	0.186	0.891	0.441	2.416	0.715	0.800	74199	Vit	chr17	+	NM_0288	P14029	protein_cc	1700052E	vitrin	Vit
9246	Strn	2.679	0.555	1.486	0.013	0.004	0.507	2.828	4.250	3.400	268980	Strn	chr17	-	NM_0115	P18743	protein_cc	AU022939	striatin, ca	Strn
9247	Heatr5b	1.084	0.873	0.947	0.844	0.708	0.904	7.426	7.542	7.347	320473	Heatr5b	chr17	-	NM_0010	P15617	protein_cc	2010013B	HEAT repe	Heatr5b
9248	Gpatch11	1.965	0.803	1.578	0.000	0.007	0.079	7.256	8.231	7.914	53951	Gpatch11	chr17	+	NM_1816	P2641	protein_cc	2310002B	G patch do	Gpatch11
9249	Gm6548	1.192	0.840	1.002	0.069	0.008	0.991	9.351	9.604	9.353	625054	Gm6548	chr17	-	NR_00336	.	misc_RNA	EG625054	predicted	Gm6548
9250	Eif2ak2	5.443	0.484	2.637	0.000	0.010	0.181	6.482	8.926	7.881	19106	Eif2ak2	chr17	-	NM_0111	P9278	protein_cc	2310047A	eukaryotic	Eif2ak2
9251	Sult6b1	1.580	0.633	1.000	0.305	1.000	0.423	0.000	0.660	0.000	73671	Sult6b1	chr17	-	NM_0011	P12746	protein_cc	2410078J0	sulfotrans	Sult6b1
9252	Cebpzos	0.930	0.952	0.886	0.285	0.507	0.208	8.643	8.539	8.468	68554	Cebpzos	chr17	+	NM_0011	P25276	protein_cc	1110001A	CCAAT/en	Cebpzos
9253	Cebpz	1.755	0.670	1.175	0.007	0.018	0.060	9.682	10.494	9.915	12607	Cebpz	chr17	-	NM_0010	P26588	protein_cc	AI848081|	CCAAT/en	Cebpz
9254	Ndufaf7	0.725	1.705	1.236	0.025	0.000	0.209	6.811	6.346	7.116	73694	Ndufaf7	chr17	+	NM_0286	P13193	protein_cc	2410091C	NADH deh	Ndufaf7
9255	Prkd3	0.179	1.443	0.258	0.000	0.105	0.025	9.458	6.975	7.505	75292	Prkd3	chr17	-	NM_0292	P11747	protein_cc	4930557O	protein kin	Prkd3
9256	Qpct	0.340	1.473	0.501	0.002	0.192	0.078	5.827	4.269	4.828	70536	Qpct	chr17	+	NM_0274	P16456	protein_cc	5730422A	glutaminy	Qpct
9257	Cdc42ep3	0.552	1.029	0.567	0.068	0.801	0.298	5.369	4.511	4.552	260409	Cdc42ep3	chr17	-	NM_0265	P17247	protein_cc	3200001F	CDC42 eff	Cdc42ep3
9258	4930429F	1.000	1.000	1.000	1.000	1.000	1.000	0.000	0.000	0.000	74622	4930429F	chr17	-	NR_03346	.	lncRNA	.	RIKEN cDN	4930429F1
9259	Gm10494	1.000	1.000	1.000	1.000	1.000	1.000	0.000	0.000	0.000	10003871	Gm10494	chr17	-	NR_03346	.	lncRNA	ENSMUSG	predicted	Gm10494
9260	Rmdn2	0.139	1.284	0.179	0.000	0.110	0.003	9.047	6.203	6.564	381110	Rmdn2	chr17	+	NM_2013	P5381	protein_cc	AW06129	regulator	Rmdn2
9261	Cyp1b1	10.351	1.426	14.758	0.001	0.020	0.011	1.441	4.813	5.325	13078	Cyp1b1	chr17	-	NM_0099	P16948	protein_cc	CP1B| P45	cytochrom	Cyp1b1
9262	Atl2	0.932	1.459	1.359	0.408	0.141	0.446	9.485	9.384	9.928	56298	Atl2	chr17	-	NM_1780	P20379	protein_cc	2010110I2	atlastin G	Atl2
9263	Hnrnpll	0.995	1.732	1.724	0.960	0.005	0.050	6.986	6.979	7.771	72692	Hnrnpll	chr17	-	NM_1448	P12495	protein_cc	2510028H	heterogen	Hnrnpll
9264	Galm	0.188	1.112	0.209	0.000	0.537	0.010	9.924	7.514	7.667	319625	Galm	chr17	+	NM_1769	P6618	protein_cc	4930557N	galactose	Galm
9265	Srsf7	3.162	0.819	2.590	0.000	0.174	0.094	7.790	9.450	9.163	225027	Srsf7	chr17	-	NM_0011	P9284	protein_cc	35kDa| 94	serine/arg	Srsf7
9266	Ttc39d	2.144	0.663	1.422	0.303	0.511	0.423	0.000	1.101	0.508	67737	Ttc39d	chr17	+	NM_0263	P13845	protein_cc	4930560E	tetratrico	Ttc39d
9267	Gemin6	1.621	1.720	2.789	0.028	0.002	0.016	5.230	5.928	6.710	67242	Gemin6	chr17	+	NM_0260	P24604	protein_cc	2610019B	gem (nucl	Gemin6
9268	Dhx57	1.185	0.538	0.637	0.655	0.180	0.363	5.485	5.729	4.835	106794	Dhx57	chr17	-	NM_0011	P15220	protein_cc	AW49491	DEAH (Asp	Dhx57
9269	Morn2	0.715	0.995	0.711	0.010	0.702	0.105	7.384	6.899	6.891	378462	Morn2	chr17	+	NM_1942	P4472	protein_cc	Mopt	MORN rep	Morn2

9270	Arhgef33	1.580	0.633	1.000	0.305	1.000	0.423	0.000	0.660	0.000	381112	Arhgef33	chr17	+	NM_0011	P5434	protein_cc	Gm941	Rho guani	Arhgef33
9271	Gm10190	3.091	1.344	4.156	0.150	0.509	0.166	0.379	2.008	2.435	791338	Gm10190	chr17	-	NR_02838	.	lncRNA	ENSMUSG	predicted	Gm10190
9272	Sos1	0.772	0.767	0.592	0.059	0.000	0.103	8.255	7.881	7.499	20662	Sos1	chr17	-	NM_0092	P12486	protein_cc	4430401P	son of sev	Sos1
9273	Cdkl4	0.264	0.971	0.256	0.181	1.000	0.423	1.965	0.043	0.000	381113	Cdkl4	chr17	-	NM_0010	P13323	protein_cc	AU067824	cyclin-dep	Cdkl4
9274	Map4k3	1.616	1.037	1.676	0.103	0.840	0.081	6.774	7.466	7.519	225028	Map4k3	chr17	-	NM_0012	P25695	protein_cc	4833416M	mitogen-a	Map4k3
9275	C230072F	1.000	1.000	1.000	1.000	1.000	1.000	0.000	0.000	0.000	320784	C230072F	chr17	+	NR_13112	.	lncRNA	.	RIKEN cDN	C230072F
9276	Tmem178	2.573	2.054	5.285	0.281	0.101	0.031	1.021	2.384	3.423	68027	Tmem178	chr17	+	NM_0265	P215	protein_cc	2810417M	transmem	Tmem178
9277	Thumpd2	1.762	0.592	1.042	0.087	0.096	0.762	4.173	4.990	4.233	72167	Thumpd2	chr17	-	NM_0281	P4449	protein_cc	2810025A	THUMP do	Thumpd2
9278	Slc8a1	0.401	1.481	0.594	0.072	0.465	0.152	4.137	2.820	3.386	20541	Slc8a1	chr17	-	NM_0012	P17387	protein_cc	AI852629 |	solute car	Slc8a1
9279	Gm19689	1.000	1.000	1.000	1.000	1.000	1.000	0.000	0.000	0.000	10050343	Gm19689	chr17	-	NR_04509	.	lncRNA	.	predicted	Gm19689
9280	Pkdcc	1.894	0.373	0.707	0.006	0.013	0.494	6.901	7.822	6.401	106522	Pkdcc	chr17	+	NM_1341	P23717	protein_cc	Adtk1| AI:	protein ki	Pkdcc
9281	Eml4	1.110	1.252	1.389	0.320	0.003	0.130	7.120	7.271	7.594	78798	Eml4	chr17	+	NM_0012	P3315	protein_cc	4930443C	echinoder	Eml4
9282	Cox7a2l	0.788	1.448	1.141	0.005	0.002	0.098	10.513	10.169	10.703	20463	Cox7a2l	chr17	-	NM_0011	P134	protein_cc	COX7AR|	cytochrom	Cox7a2l
9283	Kcng3	1.000	1.000	1.000	1.000	1.000	1.000	0.000	0.000	0.000	225030	Kcng3	chr17	-	NM_1535	P6115	protein_cc	Kv10.1a| I	potassium	Kcng3
9284	Mta3	0.824	1.766	1.455	0.079	0.268	0.624	5.005	4.725	5.546	116871	Mta3	chr17	+	NM_0011	P7444	protein_cc	1110002J2	metastasis	Mta3
9285	Haao	0.366	0.819	0.300	0.000	0.209	0.015	12.289	10.841	10.553	107766	Haao	chr17	-	NM_0253	P19093	protein_cc	0610007K	3-hydroxy	Haao
9286	8430430B	0.552	0.990	0.547	0.182	1.000	0.423	0.871	0.015	0.000	78148	8430430B	chr17	+	NR_13099	.	lncRNA	.	RIKEN cDN	8430430B
9287	4933433H	1.000	1.000	1.000	1.000	1.000	1.000	0.000	0.000	0.000	74473	4933433H	chr17	-	NR_04545	.	lncRNA	.	RIKEN cDN	4933433H
9288	Zfp36l2	0.317	1.070	0.340	0.007	0.275	0.090	8.680	7.025	7.123	12193	Zfp36l2	chr17	-	NM_0010	P26590	protein_cc	Brf2| ERF2	zinc finger	Zfp36l2
9289	Thada	1.068	1.100	1.175	0.770	0.610	0.587	5.805	5.899	6.037	240174	Thada	chr17	-	NM_1830	P2	protein_cc	6530405K	thyroid ad	Thada
9290	Plekhh2	0.159	5.535	0.881	0.214	0.140	0.926	5.826	3.174	5.643	213556	Plekhh2	chr17	+	NM_1776	P4025	protein_cc	AI256725|	pleckstrin	Plekhh2
9291	Dync2li1	34.578	1.135	39.234	0.003	0.622	0.079	2.473	7.585	7.767	213575	Dync2li1	chr17	+	NM_1722	P3279	protein_cc	4933404O	dynein cyt	Dync2li1
9292	Abcg5	0.095	1.918	0.182	0.000	0.004	0.018	9.271	5.874	6.813	27409	Abcg5	chr17	-	NM_0318	P3799	protein_cc	cmp| ster	ATP-bindi	Abcg5
9293	Abcg8	0.090	1.441	0.129	0.000	0.008	0.001	9.532	6.055	6.582	67470	Abcg8	chr17	+	NM_0012	P10704	protein_cc	1300003C	ATP-bindi	Abcg8
9294	Lrpprc	0.502	1.526	0.767	0.002	0.000	0.182	11.691	10.698	11.308	72416	Lrpprc	chr17	-	NM_0282	P21270	protein_cc	3110001K	leucine-ric	Lrpprc
9295	1110020A	0.141	0.485	0.069	0.000	0.020	0.004	5.404	2.580	1.537	68531	1110020A	chr17	-	NR_02792	.	lncRNA	2600015P	RIKEN cDN	1110020A
9296	Ppm1b	0.420	1.078	0.453	0.000	0.500	0.023	10.315	9.063	9.172	19043	Ppm1b	chr17	+	NM_0011	P17252	protein_cc	PP2CB	protein ph	Ppm1b
9297	Slc3a1	0.378	1.409	0.533	0.012	0.166	0.229	6.956	5.553	6.048	20532	Slc3a1	chr17	+	NM_0092	P14759	protein_cc	D2H| NBA	solute car	Slc3a1
9298	Prepl	0.439	1.405	0.617	0.001	0.145	0.178	7.753	6.566	7.057	213760	Prepl	chr17	-	NM_1459	P7446	protein_cc	2810457N	prolyl end	Prepl
9299	Camkmt	0.884	0.536	0.474	0.598	0.117	0.327	3.815	3.637	2.739	73582	Camkmt	chr17	+	NM_0285	P18797	protein_cc	1700106N	calmoduli	Camkmt
9300	Six3os1	1.000	1.000	1.000	1.000	1.000	1.000	0.000	0.000	0.000	10004390	Six3os1	chr17	-	NR_03808	.	lncRNA	D17Mgi26	SIX homeo	Six3os1
9301	Six3	1.000	1.000	1.000	1.000	1.000	1.000	0.000	0.000	0.000	20473	Six3	chr17	+	NM_0113	P9559	protein_cc	E130112M	sine oculis	Six3
9302	Six2	1.000	1.000	1.000	1.000	1.000	1.000	0.000	0.000	0.000	20472	Six2	chr17	-	NM_0113	P25823	protein_cc	.	sine oculis	Six2
9303	Srbd1	0.358	0.946	0.338	0.002	0.861	0.038	6.770	5.286	5.206	78586	Srbd1	chr17	-	NM_0301	P3466	protein_cc	AI461933|	S1 RNA bi	Srbd1
9304	Prkce	1.206	1.007	1.215	0.169	0.936	0.197	8.144	8.414	8.425	18754	Prkce	chr17	+	NM_0111	P18356	protein_cc	5830406C	protein ki	Prkce
9305	2010106C	1.000	1.000	1.000	1.000	1.000	1.000	0.000	0.000	0.000	72104	2010106C	chr17	-	NR_04543	.	lncRNA	.	RIKEN cDN	2010106C
9306	Epas1	1.122	1.202	1.349	0.147	0.070	0.141	9.815	9.981	10.247	13819	Epas1	chr17	+	NM_0101	P13209	protein_cc	bHLHe73|	endothelia	Epas1
9307	Tmem247	1.000	1.000	1.000	1.000	1.000	1.000	0.000	0.000	0.000	78469	Tmem247	chr17	+	NM_0301	P12490	protein_cc	1700090G	transmem	Tmem247
9308	Atp6v1e2	1.000	1.000	1.000	1.000	1.000	1.000	0.000	0.000	0.000	74915	Atp6v1e2	chr17	-	NM_0291	P11100	protein_cc	4930500C	ATPase, H	Atp6v1e2
9309	Rhoq	1.664	1.225	2.038	0.012	0.209	0.122	7.585	8.319	8.612	104215	Rhoq	chr17	+	NM_1454	P22297	protein_cc	Arhq| Tc1	ras homol	Rhoq
9310	Pigf	1.333	1.260	1.680	0.001	0.005	0.021	5.875	6.290	6.624	18701	Pigf	chr17	-	NM_0088	P23991	protein_cc	.	phosphati	Pigf
9311	Cript	1.344	1.159	1.558	0.006	0.031	0.040	8.323	8.750	8.962	56724	Cript	chr17	+	NM_0199	P591	protein_cc	1200020A	cysteine-r	Cript
9312	Socs5	1.716	0.811	1.392	0.010	0.395	0.551	5.419	6.199	5.897	56468	Socs5	chr17	+	NM_0196	P1180	protein_cc	1810018L	suppresso	Socs5
9313	C330024C	1.589	1.141	1.813	0.298	0.765	0.423	0.000	0.669	0.859	399626	C330024C	chr17	-	NR_04601	.	lncRNA	.	RIKEN cDN	C330024C
9314	Mcfd2	1.438	0.810	1.166	0.047	0.205	0.510	10.614	11.138	10.835	193813	Mcfd2	chr17	-	NM_1768	P15991	protein_cc	1810021C	multiple c	Mcfd2
9315	4833418N	0.408	1.408	0.574	0.126	0.142	0.478	4.243	2.948	3.442	74597	4833418N	chr17	-	NR_01550	.	lncRNA	.	RIKEN cDN	4833418N
9316	Ttc7	0.377	1.362	0.514	0.001	0.318	0.115	8.679	7.273	7.719	225049	Ttc7	chr17	+	NM_0286	P19342	protein_cc	1110035E	tetratricop	Ttc7
9317	1700011E	1.000	1.000	1.000	1.000	1.000	1.000	0.000	0.000	0.000	75467	1700011E	chr17	-	NM_0292	P4438	protein_cc	GSE	RIKEN cDN	1700011E
9318	Calm2	1.041	0.950	0.989	0.544	0.488	0.944	10.765	10.823	10.748	12314	Calm2	chr17	-	NM_0075	P13415	protein_cc	1500001E	calmoduli	Calm2
9319	Epcam	0.278	1.544	0.429	0.006	0.402	0.219	4.422	2.576	3.202	17075	Epcam	chr17	+	NM_0085	P3983	protein_cc	CD326| E(	epithelial	Epcam
9320	Msh2	4.310	0.631	2.718	0.008	0.143	0.228	7.132	9.239	8.574	17685	Msh2	chr17	+	NM_0086	P15326	protein_cc	AI788990	mutS hom	Msh2
9321	Kcnk12	1.000	1.000	1.000	1.000	1.000	1.000	0.000	0.000	0.000	210741	Kcnk12	chr17	-	NM_1992	P1701	protein_cc	mntk1	potassium	Kcnk12
9322	Msh6	1.488	0.672	0.999	0.034	0.076	0.998	6.951	7.524	6.950	17688	Msh6	chr17	+	NM_0108	P5938	protein_cc	AU044881	mutS hom	Msh6
9323	Fbxo11	1.246	0.737	0.919	0.043	0.085	0.733	7.612	7.930	7.489	225055	Fbxo11	chr17	-	NM_0010	P1586	protein_cc	C80048| J	F-box prot	Fbxo11
9324	Gm4832	1.879	2.270	4.264	0.017	0.005	0.031	1.114	2.023	3.206	225058	Gm4832	chr17	+	NM_0011	P1027	protein_cc	EG225058	predicted	Gm4832
9325	Foxn2	1.108	0.601	0.666	0.603	0.085	0.056	6.832	6.979	6.245	14236	Foxn2	chr17	+	NM_1809	P16302	protein_cc	3230402J	forkhead	Foxn2
9326	Ppp1r21	0.504	1.416	0.714	0.004	0.170	0.232	7.010	6.021	6.523	73825	Ppp1r21	chr17	+	NM_0286	P14422	protein_cc	1110018J1	protein ph	Ppp1r21
9327	Ston1	0.467	2.184	1.020	0.185	0.017	0.976	1.137	0.038	1.165	77057	Ston1	chr17	+	NM_0298	P24008	protein_cc	4921524J	stonin 1	Ston1
9328	Gtf2a1l	1.000	1.000	1.000	1.000	1.000	1.000	0.000	0.000	0.000	71828	Gtf2a1l	chr17	+	NM_0236	P7643	protein_cc	1700011N	general tr	Gtf2a1l
9329	Lhcgr	1.000	1.000	1.000	1.000	1.000	1.000	0.000	0.000	0.000	16867	Lhcgr	chr17	-	NM_0135	P20985	protein_cc	Gpcr19-rs	luteinizing	Lhcgr
9330	Fshr	1.000	1.000	1.000	1.000	1.000	1.000	0.000	0.000	0.000	14309	Fshr	chr17	-	NM_0135	P1611	protein_cc	.	follicle sti	Fshr
9331	4933400B	1.000	1.000	1.000	1.000	1.000	1.000	0.000	0.000	0.000	71052	4933400B	chr17	+	NR_04543	.	lncRNA	.	RIKEN cDN	4933400B
9332	Gm4719	1.000	1.000	1.000	1.000	1.000	1.000	0.000	0.000	0.000	10004390	Gm4719	chr17	-	NR_04595	.	lncRNA	10004390	predicted	Gm4719
9333	Nrxn1	0.408	0.475	0.194	0.016	0.002	0.077	6.936	5.642	4.569	18189	Nrxn1	chr17	-	NM_1772	P14666	protein_cc	1700062G	neurexin I	Nrxn1
9334	4930480K	0.373	0.981	0.366	0.018	1.000	0.140	1.450	0.027	0.000	78800	4930480K	chr17	-	NR_04546	.	lncRNA	.	RIKEN cDN	4930480K
9335	Adcyap1	1.000	1.000	1.000	1.000	1.000	1.000	0.000	0.000	0.000	11516	Adcyap1	chr17	+	NM_0096	P25710	protein_cc	PACAP	adenylate	Adcyap1
9336	Mettl4	2.986	0.433	1.293	0.006	0.015	0.394	4.025	5.604	4.396	76781	Mettl4	chr17	-	NM_1769	P5736	protein_cc	2410198H	methyltra	Mettl4
9337	2700099C	0.268	1.045	0.280	0.007	0.893	0.061	2.623	0.723	0.786	77022	2700099C	chr17	-	NR_02472	.	misc_RNA	9430041D	RIKEN cDN	2700099C
9338	Gm1976	0.364	4.586	1.668	0.212	0.035	0.520	3.008	1.549	3.746	10003874	Gm1976	chr17	-	NR_04596	.	lncRNA	10003886	predicted	Gm1976
9339	Gm20939	2.343	1.307	3.062	0.001	0.043	0.027	3.649	4.877	5.263	10004419	Gm20939	chr17	-	NM_0010	P5624	protein_cc	.	predicted	Gm20939
9340	Vmn1r238	1.000	1.000	1.000	1.000	1.000	1.000	0.000	0.000	0.000	10031247	Vmn1r238	chr18	-	NM_0011	P20577	protein_cc	.	vomerona	Vmn1r238
9341	Crem	5.085	0.316	1.604	0.000	0.000	0.360	7.448	9.794	8.130	12916	Crem	chr18	-	NM_0011	P2589	protein_cc	ICER| ICEF	cAMP resp	Crem
9342	Gm6225	1.136	0.708	0.805	0.754	0.445	0.510	2.350	2.534	2.036	633947	Gm6225	chr18	+	NR_03345	.	lncRNA	EG621440	predicted	Gm6225
9343	Cul2	0.971	0.990	0.961	0.867	0.944	0.826	8.008	7.965	7.950	71745	Cul2	chr18	+	NM_0294	P25479	protein_cc	1300003D	cullin 2	Cul2
9344	Bambi	0.706	1.760	1.242	0.313	0.004	0.627	4.572	4.070	4.885	68010	Bambi	chr18	+	NM_0265	P23311	protein_cc	2610003H	BMP and a	Bambi
9345	Lyzl1	1.000	1.000	1.000	1.000	1.000	1.000	0.000	0.000	0.000	67328	Lyzl1	chr18	-	NM_0260	P19698	protein_cc	1700038F	lysozyme-	Lyzl1
9346	Map3k8	32.586	0.822	26.769	0.000	0.495	0.175	1.804	6.830	6.547	26410	Map3k8	chr18	-	NM_0077	P5674	protein_cc	c-COT| Co	mitogen-a	Map3k8
9347	4833419F	16.625	1.402	23.316	0.015	0.424	0.169	0.333	4.388	4.876	73915	4833419F	chr18	+	NR_04032	.	lncRNA	.	RIKEN cDN	4833419F
9348	Mtpap	1.208	0.835	1.009	0.421	0.532	0.986	6.687	6.960	6.699	67440	Mtpap	chr18	+	NM_0261	P13696	protein_cc	0610027A	mitochond	Mtpap
9349	9430020K	2.036	0.566	1.153	0.000	0.001	0.409	6.949	7.974	7.154	240185	9430020K	chr18	+	NM_0010	P551	protein_cc	1110056P	RIKEN cDN	9430020K
9350	Gm10556	0.264	0.971	0.256	0.181	1.000	0.423	1.965	0.043	0.000	10003843	Gm10556	chr18	+	NR_04588	.	lncRNA	ENSMUSG	predicted	Gm10556
9351	Svil	0.578	0.655	0.378	0.000	0.002	0.008	7.823	7.032	6.421	225115	Svil	chr18	+	NM_1531	P16293	protein_cc	AU024053	supervillin	Svil
9352	Zfp438	1.288	0.921	1.186	0.499	0.842	0.840	3.271	3.636	3.518	240186	Zfp438	chr18	-	NM_1787	P16303	protein_cc	9430031G	zinc finger	Zfp438
9353	Gm10125	1.000	1.000	1.000	1.000	1.000	1.000	0.000	0.000	0.000	791318	Gm10125	chr18	-	NR_03355	.	lncRNA	ENSMUSG	predicted	Gm10125
9354	Zeb1	1.367	0.564	0.770	0.001	0.001	0.208	7.366	7.817	6.990	21417	Zeb1	chr18	+	NM_0115	P26028	protein_cc	3110032K	zinc finger	Zeb1
9355	Arhgap12	0.646	0.807	0.521	0.004	0.074	0.043	8.509	7.879	7.569	75415	Arhgap12	chr18	-	NM_0292	P23594	protein_cc	2810011M	Rho GTPas	Arhgap12
9356	Kif5b	2.332	0.805	1.876	0.000	0.035	0.037	10.201	11.423	11.109	16573	Kif5b	chr18	-	NM_0084	P4802	protein_cc	AL022807	kinesin fa	Kif5b
9357	Epc1	1.171	0.993	1.163	0.234	0.980	0.744	7.255	7.482	7.473	13831	Epc1	chr18	-	NM_0274	P10269	protein_cc	2400007E	enhancer	Epc1
9358	4921524L	1.000	1.000	1.000	1.000	1.000	1.000	0.000	0.000	0.000	70901	4921524L	chr18	+	NM_0275	P12854	protein_cc	.	RIKEN cDN	4921524L
9359	Rab18	1.819	0.897	1.632	0.000	0.406	0.191	9.707	10.570	10.413	19330	Rab18	chr18	+	NM_0012	P723	protein_cc	AA959686	RAB18, m	Rab18
9360	Mkx	0.906	0.222	0.201	0.899	1.000	0.312	2.315	2.172	0.000	210719	Mkx	chr18	-	NM_1775	P22728	protein_cc	9430023B	mohawk h	Mkx

9361	Armc4	1.000	1.000	1.000	1.000	1.000	1.000	0.000	0.000	0.000	74934	Armc4	chr18	-	NM_0010	P10439	protein_cc	4930463I2	armadillo	Armc4
9362	Mpp7	3.077	0.668	2.055	0.000	0.000	0.013	4.940	6.561	5.979	75739	Mpp7	chr18	-	NM_0010	P6671	protein_cc	1110068J(	membran	Mpp7
9363	Wac	1.119	0.931	1.041	0.312	0.428	0.758	8.979	9.142	9.038	225131	Wac	chr18	+	NM_0012	P514	protein_cc	1110067P	WW doma	Wac
9364	Fzd8	0.117	0.863	0.101	0.002	0.359	0.043	7.616	4.517	4.305	14370	Fzd8	chr18	+	NM_0080	P14720	protein_cc	Fz8	frizzled cla	Fzd8
9365	Gjd4	1.000	1.000	1.000	1.000	1.000	1.000	0.000	0.000	0.000	225152	Gjd4	chr18	-	NM_1530	P1112	protein_cc	9430022F	gap juncti	Gjd4
9366	Ccny	0.754	1.256	0.947	0.002	0.001	0.464	8.708	8.301	8.630	67974	Ccny	chr18	-	NM_0264	P20586	protein_cc	1700025H	cyclin Y	Ccny
9367	Cetn1	1.005	1.439	1.446	0.000	0.182	0.423	0.000	0.008	0.532	26369	Cetn1	chr18	-	NM_0075	P19979	protein_cc	caltractin	centrin 1	Cetn1
9368	Colec12	0.138	4.146	0.574	0.013	0.002	0.311	6.574	3.721	5.773	140792	Colec12	chr18	+	NM_1304	P17562	protein_cc	CL-P1 | Sca	collectin s	Colec12
9369	Thoc1	1.846	0.761	1.404	0.045	0.224	0.169	7.696	8.581	8.186	225160	Thoc1	chr18	+	NM_1535	P16384	protein_cc	3110002N	THO comp	Thoc1
9370	Usp14	1.236	1.055	1.304	0.367	0.784	0.208	8.792	9.098	9.175	59025	Usp14	chr18	-	NM_0215	P5589	protein_cc	2610005K	ubiquitin s	Usp14
9371	Rock1	1.231	0.729	0.898	0.000	0.002	0.341	10.606	10.905	10.450	19877	Rock1	chr18	-	NM_0090	P2464	protein_cc	1110055K	Rho-assoc	Rock1
9372	Greb1l	3.653	0.301	1.098	0.011	0.007	0.932	2.422	4.291	2.557	381157	Greb1l	chr18	+	NM_0010	P1420	protein_cc	mKIAA409	growth re	Greb1l
9373	Esco1	2.148	0.688	1.478	0.001	0.005	0.170	6.419	7.521	6.982	77805	Esco1	chr18	-	NM_0010	P5091	protein_cc	A930014I1	establishn	Esco1
9374	Snrpd1	2.395	0.737	1.766	0.000	0.011	0.060	8.683	9.943	9.503	20641	Snrpd1	chr18	+	NM_0092	P26555	protein_cc	AA407109	small nucl	Snrpd1
9375	Abhd3	0.370	1.091	0.404	0.001	0.593	0.025	9.113	7.679	7.804	106861	Abhd3	chr18	-	NM_1341	P18771	protein_cc	AA675331	abhydrola	Abhd3
9376	4930563E	0.534	0.623	0.333	0.350	1.000	0.423	1.588	0.683	0.000	75370	4930563E	chr18	+	NR_04537	.	lncRNA	.	RIKEN cDN	4930563E1
9377	Mib1	3.004	0.713	2.141	0.017	0.393	0.545	1.816	3.403	2.915	225164	Mib1	chr18	+	NM_1448	P4027	protein_cc	DIP-1 | E3	mindbom	Mib1
9378	1010001N	0.449	0.951	0.427	0.307	0.881	0.522	2.946	1.792	1.719	68434	1010001N	chr18	-	NR_10502	.	lncRNA	.	RIKEN cDN	1010001N
9379	Gata6	2.630	1.054	2.773	0.000	0.812	0.194	6.950	8.345	8.422	14465	Gata6	chr18	+	NM_0102	P9735	protein_cc	AA410133	GATA bind	Gata6
9380	Rbbp8	2.582	0.713	1.842	0.004	0.093	0.120	5.563	6.932	6.445	225182	Rbbp8	chr18	+	NM_0012	P13313	protein_cc	9930104E	retinoblas	Rbbp8
9381	Gm6277	1.000	1.000	1.000	1.000	1.000	1.000	0.000	0.000	0.000	621998	Gm6277	chr18	-	NR_04542	.	lncRNA	621998	predicted	Gm6277
9382	Cables1	0.115	0.507	0.058	0.004	0.225	0.056	6.088	2.970	1.991	63955	Cables1	chr18	+	NM_0220	P6276	protein_cc	Cables | ik	CDK5 and	Cables1
9383	Tmem241	0.413	2.018	0.833	0.002	0.085	0.595	5.538	4.261	5.274	338363	Tmem241	chr18	-	NR_11035	.	protein_cc	6030446N	transmem	Tmem241
9384	Riok3	1.735	1.151	1.996	0.002	0.370	0.129	8.866	9.660	9.863	66878	Riok3	chr18	+	NM_0241	P19703	protein_cc	1200013N	RIO kinase	Riok3
9385	3110002H	0.097	2.067	0.201	0.000	0.020	0.002	8.739	5.376	6.424	76482	3110002H	chr18	+	NM_0296	P11947	protein_cc	2400010D	RIKEN cDN	3110002H
9386	Npc1	0.076	2.717	0.207	0.000	0.005	0.003	10.427	6.713	8.155	18145	Npc1	chr18	-	NM_0087	P16055	protein_cc	A430089E	Niemann-	Npc1
9387	Ankrd29	2.110	0.639	1.349	0.365	0.553	0.784	1.296	2.374	1.728	225187	Ankrd29	chr18	-	NM_0011	P5360	protein_cc	G630054C	ankyrin re	Ankrd29
9388	Lama3	0.292	7.573	2.210	0.187	0.002	0.291	1.875	0.098	3.019	16774	Lama3	chr18	+	NM_0106	P18396	protein_cc	Lama3B |	laminin, al	Lama3
9389	Ttc39c	0.166	2.789	0.464	0.000	0.031	0.062	8.661	6.072	7.552	72747	Ttc39c	chr18	+	NM_0283	P20444	protein_cc	1700008N	tetratrico	Ttc39c
9390	Cabyr	0.036	2.116	0.077	0.005	0.232	0.084	6.346	1.563	2.644	71132	Cabyr	chr18	+	NM_0010	P796	protein_cc	1700016C	calcium-bi	Cabyr
9391	Osbpl1a	0.058	4.763	0.274	0.000	0.004	0.023	10.409	6.291	8.543	64291	Osbpl1a	chr18	-	NM_0012	P7916	protein_cc	G430090F	oxysterol	Osbpl1a
9392	Impact	1.976	0.859	1.697	0.139	0.629	0.098	5.439	6.422	6.202	16210	Impact	chr18	+	NM_0083	P5960	protein_cc	E430016J1	impact, R\	Impact
9393	Hrh4	1.000	1.000	1.000	1.000	1.000	1.000	0.000	0.000	0.000	225192	Hrh4	chr18	+	NM_1530	P19556	protein_cc	AXOR35 |	histamine	Hrh4
9394	Zfp521	0.126	3.843	0.483	0.181	0.102	0.632	3.092	0.100	2.042	225207	Zfp521	chr18	-	NM_1454	P3897	protein_cc	B930086A	zinc finger	Zfp521
9395	8430422H	1.000	1.000	1.000	1.000	1.000	1.000	0.000	0.000	0.000	74529	8430422H	chr18	+	NR_04537	.	lncRNA	.	RIKEN cDN	8430422H
9396	Ss18	1.377	0.746	1.027	0.001	0.003	0.699	8.603	9.064	8.641	268996	Ss18	chr18	-	NM_0011	P8307	protein_cc	D130059H	synovial s	Ss18
9397	Psma8	2.161	1.641	3.546	0.416	0.355	0.083	1.711	2.823	3.537	73677	Psma8	chr18	+	NM_0011	P15938	protein_cc	2410072D	proteason	Psma8
9398	Taf4b	4.101	0.677	2.777	0.000	0.235	0.372	4.786	6.823	6.260	72504	Taf4b	chr18	+	NM_0011	P21363	protein_cc	105kDa | 2	TATA-box	Taf4b
9399	Kctd1	3.591	1.097	3.938	0.047	0.719	0.011	3.352	5.197	5.330	106931	Kctd1	chr18	-	NM_0011	P9825	protein_cc	4933402K	potassium	Kctd1
9400	4933424G	0.729	0.456	0.333	0.634	1.000	0.423	1.588	1.132	0.000	74456	4933424G	chr18	+	NR_04537	.	lncRNA	.	RIKEN cDN	4933424G
9401	Aqp4	0.193	2.580	0.498	0.004	0.172	0.300	3.931	1.559	2.926	11829	Aqp4	chr18	-	NM_0097	P11525	protein_cc	WCH4	aquaporin	Aqp4
9402	Chst9	0.339	0.981	0.333	0.181	1.000	0.423	1.588	0.028	0.000	71367	Chst9	chr18	-	NM_1990	P20343	protein_cc	5430438D	carbohydr	Chst9
9403	Cdh2	0.154	1.854	0.286	0.000	0.006	0.004	9.863	7.167	8.057	12558	Cdh2	chr18	-	NM_0076	P15332	protein_cc	CDHN | N-	cadherin 2	Cdh2
9404	Gm15328	2.029	0.366	0.742	0.405	1.000	0.422	0.431	1.451	0.000	328906	Gm15328	chr18	+	NR_04539	.	lncRNA	D830029L	predicted	Gm15328
9405	1700001G	0.669	0.995	0.666	0.181	1.000	0.423	0.587	0.008	0.000	75433	1700001G	chr18	+	NR_13015	.	lncRNA	.	RIKEN cDN	1700001G
9406	4921533I2	1.315	0.767	1.008	0.468	0.276	0.989	1.090	1.486	1.103	664619	4921533I2	chr18	-	NR_03360	.	misc_RNA	.	Riken cDN	4921533I2
9407	4930545E	1.000	1.000	1.000	1.000	1.000	1.000	0.000	0.000	0.000	75162	4930545E	chr18	-	NR_04537	.	lncRNA	.	RIKEN cDN	4930545E
9408	Dsc3	1.000	1.000	1.000	1.000	1.000	1.000	0.000	0.000	0.000	13507	Dsc3	chr18	-	NM_0012	P4680	protein_cc	5430426I2	desmocoll	Dsc3
9409	Dsc2	0.080	4.747	0.378	0.001	0.000	0.071	7.316	3.666	5.913	13506	Dsc2	chr18	-	NM_0135	P6985	protein_cc	AW22816	desmocoll	Dsc2
9410	Dsc1	1.000	1.000	1.000	1.000	1.000	1.000	0.000	0.000	0.000	13505	Dsc1	chr18	-	NM_0012	P12227	protein_cc	1110020A	desmocoll	Dsc1
9411	Dsg1c	0.162	1.276	0.207	0.005	0.235	0.086	2.691	0.069	0.421	211924	Dsg1c	chr18	+	NM_1816	P22815	protein_cc	dsg1g | Ds	desmoglei	Dsg1c
9412	Dsg1a	0.534	0.990	0.528	0.000	1.000	0.015	0.921	0.015	0.000	13510	Dsg1a	chr18	+	NM_0100	P25863	protein_cc	DG1 | DGI	desmoglei	Dsg1a
9413	Dsg1b	0.255	0.970	0.247	0.009	1.000	0.099	2.015	0.043	0.000	225256	Dsg1b	chr18	+	NM_1816	P17145	protein_cc	dsg1-beta	desmoglei	Dsg1b
9414	Dsg4	1.000	1.000	1.000	1.000	1.000	1.000	0.000	0.000	0.000	16769	Dsg4	chr18	+	NM_1815	P19584	protein_cc	CDHF13 | I	desmoglei	Dsg4
9415	Dsg3	1.000	1.000	1.000	1.000	1.000	1.000	0.000	0.000	0.000	13512	Dsg3	chr18	+	NM_0305	P14031	protein_cc	bal	desmoglei	Dsg3
9416	Dsg2	0.278	0.788	0.219	0.000	0.066	0.014	11.016	9.168	8.825	13511	Dsg2	chr18	+	NM_0078	P21374	protein_cc	AA408168	desmoglei	Dsg2
9417	Ttr	0.363	0.995	0.361	0.000	0.956	0.009	17.027	15.565	15.557	22139	Ttr	chr18	+	NM_0136	P14160	protein_cc	AA408768	transthyre	Ttr
9418	B4galt6	0.480	3.220	1.547	0.014	0.001	0.145	5.076	4.018	5.705	56386	B4galt6	chr18	-	NM_0197	P22429	protein_cc	AA536803	UDP-Gal:b	B4galt6
9419	Trappc8	0.959	0.851	0.816	0.911	0.714	0.489	5.824	5.764	5.531	75964	Trappc8	chr18	-	NM_1770	P24193	protein_cc	5033403J1	trafficking	Trappc8
9420	Rnf125	0.583	0.776	0.452	0.001	0.495	0.203	10.884	10.107	9.740	67664	Rnf125	chr18	+	NM_0263	P13733	protein_cc	4930553F	ring finger	Rnf125
9421	Rnf138	0.814	1.033	0.842	0.074	0.896	0.660	6.930	6.634	6.682	56515	Rnf138	chr18	+	NM_0013	P9914	protein_cc	2410015A	ring finger	Rnf138
9422	Mep1b	1.000	1.000	1.000	1.000	1.000	1.000	0.000	0.000	0.000	17288	Mep1b	chr18	+	NM_0085	P16771	protein_cc	Mep-1b	meprin 1 b	Mep1b
9423	Garem	2.349	0.718	1.686	0.000	0.063	0.246	8.108	9.340	8.861	381126	Garem	chr18	-	NM_0010	P22736	protein_cc	C86169 | F	GRB2 asso	Garem
9424	Klhl14	1.000	1.000	1.000	1.000	1.000	1.000	0.000	0.000	0.000	225266	Klhl14	chr18	-	NM_0010	P25614	protein_cc	6330403N	kelch-like	Klhl14
9425	4930426D	1.572	0.636	1.000	0.304	1.000	0.423	0.000	0.653	0.000	74644	4930426D	chr18	-	NM_0012	P1395	protein_cc	.	RIKEN cDN	4930426D
9426	Ccdc178	1.000	1.000	1.000	1.000	1.000	1.000	0.000	0.000	0.000	70950	Ccdc178	chr18	-	NM_0276	P778	protein_cc	4921528I(	coiled coil	Ccdc178
9427	Asxl3	0.669	0.995	0.666	0.181	1.000	0.423	0.587	0.008	0.000	211961	Asxl3	chr18	+	NM_0011	P8594	protein_cc	C230079D	additional	Asxl3
9428	Nol4	0.716	0.995	0.712	0.182	1.000	0.423	0.490	0.008	0.000	319211	Nol4	chr18	-	NM_1990	P19053	protein_cc	1700013J1	nucleolar	Nol4
9429	Gm7788	0.589	3.404	2.005	0.193	0.000	0.147	0.811	0.048	1.815	665797	Gm7788	chr18	+	NR_11049	.	misc_RNA	EG665797	predicted	Gm7788
9430	Dtna	0.535	2.302	1.233	0.233	0.146	0.806	2.710	1.809	3.012	13527	Dtna	chr18	+	NM_0012	P19332	protein_cc	2210407P	dystrobre	Dtna
9431	Gm15972	1.023	3.051	3.120	0.000	0.182	0.423	0.000	0.033	1.642	10050283	Gm15972	chr18	-	NR_12606	.	lncRNA	OTTMUSG	predicted	Gm15972
9432	Mapre2	1.445	0.729	1.054	0.002	0.004	0.337	7.321	7.852	7.396	212307	Mapre2	chr18	+	NM_0011	P691	protein_cc	AI314113	microtubu	Mapre2
9433	Zfp397	1.446	0.706	1.021	0.132	0.119	0.907	5.577	6.109	5.607	69256	Zfp397	chr18	+	NM_0270	P6899	protein_cc	2810411K	zinc finger	Zfp397
9434	Zfp35	1.345	0.898	1.208	0.010	0.233	0.410	6.486	6.913	6.758	22694	Zfp35	chr18	+	NM_0117	P6350	protein_cc	mszf13 | m	zinc finger	Zfp35
9435	Zfp191	1.408	1.135	1.598	0.066	0.028	0.143	7.331	7.824	8.008	.	Zfp191	chr18	+	NM_0215	P17400	.	.	.	Zfp191
9436	Ino80c	1.428	1.444	2.062	0.178	0.173	0.185	8.336	8.850	9.380	225280	Ino80c	chr18	-	NM_1726	P2579	protein_cc	D030070L	INO80 cor	Ino80c
9437	Galnt1	0.747	1.242	0.928	0.020	0.132	0.635	9.268	8.848	9.161	14423	Galnt1	chr18	+	NM_0138	P5137	protein_cc	.	UDP-N-ac	Galnt1
9438	2700062C	1.119	1.158	1.296	0.210	0.320	0.369	6.011	6.174	6.385	68046	2700062C	chr18	+	NM_0265	P1591	protein_cc	AI195775	RIKEN cDN	2700062C
9439	Rprd1a	1.419	0.524	0.744	0.019	0.104	0.690	5.750	6.255	5.324	225283	Rprd1a	chr18	-	NM_1448	P12055	protein_cc	C77387 | n	regulation	Rprd1a
9440	Slc39a6	4.455	0.529	2.357	0.000	0.003	0.108	4.718	6.874	5.955	106957	Slc39a6	chr18	-	NM_1391	P4408	protein_cc	Ermelin	solute car	Slc39a6
9441	Elp2	0.853	0.951	0.811	0.056	0.524	0.199	8.293	8.064	7.991	58523	Elp2	chr18	+	NM_0214	P14526	protein_cc	AU023723	elongator	Elp2
9442	Mocos	1.134	0.839	0.951	0.047	0.035	0.537	9.164	9.345	9.091	68591	Mocos	chr18	+	NM_0267	P26405	protein_cc	1110018O	molybden	Mocos
9443	Fhod3	0.208	1.033	0.215	0.066	0.939	0.265	3.001	0.734	0.781	225288	Fhod3	chr18	+	NM_0012	P6136	protein_cc	A930009H	formin ho	Fhod3
9444	Tpgs2	0.427	1.106	0.473	0.041	0.822	0.093	5.991	4.764	4.909	66648	Tpgs2	chr18	-	NM_0011	P12400	protein_cc	5730437P	tubulin po	Tpgs2
9445	AW55491	0.754	0.989	0.746	0.172	0.963	0.240	4.950	4.543	4.527	225289	AW55491	chr18	+	NM_0010	P8320	protein_cc	4921538P	expressed	AW554918
9446	Celf4	0.757	2.586	1.957	0.388	0.102	0.406	3.741	3.340	4.710	108013	Celf4	chr18	-	NM_0011	P20389	protein_cc	A230070D	CUGBP, El	Celf4
9447	4930474G	1.000	1.000	1.000	1.000	1.000	1.000	0.000	0.000	0.000	320624	4930474G	chr18	+	NR_04539	.	lncRNA	.	RIKEN cDN	4930474G
9448	4930578E	1.000	1.000	1.000	1.000	1.000	1.000	0.000	0.000	0.000	78058	4930578E	chr18	-	NR_04539	.	lncRNA	.	RIKEN cDN	4930578E1
9449	4930527G	1.000	1.000	1.000	1.000	1.000	1.000	0.000	0.000	0.000	78199	4930527G	chr18	+	NR_04539	.	lncRNA	.	RIKEN cDN	4930527G
9450	Pik3c3	0.784	1.005	0.788	0.116	0.972	0.043	7.188	6.837	6.844	225326	Pik3c3	chr18	+	NM_1814	P13191	protein_cc	5330434F	phosphoir	Pik3c3
9451	Rit2	1.000	1.000	1.000	1.000	1.000	1.000	0.000	0.000	0.000	19762	Rit2	chr18	-	NM_0090	P22740	protein_cc	RIBA | Rin |	Ras-like w	Rit2

9452	Syt4	1.014	2.240	2.272	0.000	0.182	0.423	0.000	0.021	1.185	20983	Syt4	chr18	-	NM_0093	P23390	protein_cc	SytIV	synaptota	Syt4
9453	Slc25a46	0.764	0.964	0.737	0.044	0.721	0.048	9.238	8.851	8.797	67453	Slc25a46	chr18	-	NM_0261	P24104	protein_cc	1200007B	solute car	Slc25a46
9454	Sap130	0.668	1.391	0.930	0.013	0.001	0.613	5.892	5.310	5.786	269003	Sap130	chr18	+	NM_1729	P9711	protein_cc	2610304F	Sin3A asso	Sap130
9455	Ammecr1l	0.782	0.841	0.657	0.000	0.001	0.004	9.859	9.503	9.253	225339	Ammecr1l	chr18	+	NM_1535	P140	protein_cc	5430429D	AMME chr	Ammecr1l
9456	Polr2d	2.872	0.689	1.980	0.002	0.023	0.071	4.652	6.174	5.638	69241	Polr2d	chr18	+	NM_0270	P2357	protein_cc	2310002G	polymeras	Polr2d
9457	Wdr33	1.509	0.851	1.284	0.126	0.425	0.310	6.907	7.500	7.268	74320	Wdr33	chr18	+	NM_0288	P17659	protein_cc	1110001N	WD repea	Wdr33
9458	Sft2d3	1.086	1.617	1.757	0.729	0.324	0.494	7.237	7.356	8.049	67158	Sft2d3	chr18	-	NM_0260	P13185	protein_cc	2610206C	SFT2 dom	Sft2d3
9459	Lims2	0.040	6.448	0.257	0.000	0.044	0.057	8.923	4.276	6.965	225341	Lims2	chr18	+	NM_1448	P5702	protein_cc	PINCH2	LIM and s	Lims2
9460	Gpr17	1.000	1.000	1.000	1.000	1.000	1.000	0.000	0.000	0.000	574402	Gpr17	chr18	-	NM_0010	P12458	protein_cc	AI853548	G protein-	Gpr17
9461	Myo7b	1.240	2.865	3.551	0.600	0.259	0.460	0.410	0.719	2.238	17922	Myo7b	chr18	-	NM_0323	P24386	protein_cc	.	myosin VI	Myo7b
9462	Iws1	1.059	0.846	0.896	0.339	0.012	0.259	8.133	8.215	7.975	73473	Iws1	chr18	+	NM_1734	P17334	protein_cc	1700069O	IWS1 hom	Iws1
9463	Proc	0.322	0.868	0.279	0.001	0.262	0.020	12.069	10.434	10.229	19123	Proc	chr18	-	NM_0010	P18157	protein_cc	PC	protein C	Proc
9464	Map3k2	1.006	0.837	0.842	0.945	0.119	0.050	7.534	7.543	7.286	26405	Map3k2	chr18	+	NM_0119	P12699	protein_cc	9630061B	mitogen-a	Map3k2
9465	Ercc3	1.270	1.398	1.775	0.001	0.013	0.053	7.551	7.896	8.379	13872	Ercc3	chr18	+	NM_1336	P26761	protein_cc	BTF2 p89		excision re	Ercc3
9466	A830052D	0.073	1.203	0.088	0.084	0.304	0.302	3.888	0.117	0.384	402767	A830052D	chr18	-	NR_04540	.	lncRNA	.	RIKEN cDN	A830052D
9467	Bin1	1.091	1.674	1.826	0.284	0.000	0.003	6.176	6.301	7.044	30948	Bin1	chr18	+	NM_0010	P3343	protein_cc	ALP-1 | Am	bridging in	Bin1
9468	Gypc	0.447	1.749	0.782	0.008	0.008	0.284	5.857	4.695	5.502	71683	Gypc	chr18	-	NM_0010	P26447	protein_cc	0610037F	glycophor	Gypc
9469	4930455D	1.000	1.000	1.000	1.000	1.000	1.000	0.000	0.000	0.000	75795	4930455D	chr18	-	NR_04538	.	lncRNA	.	RIKEN cDN	4930455D
9470	Tslp	0.268	2.707	0.726	0.091	0.269	0.756	2.653	0.753	2.190	53603	Tslp	chr18	+	NM_0213	P7912	protein_cc	.	thymic str	Tslp
9471	Wdr36	0.975	0.805	0.785	0.936	0.580	0.371	6.987	6.950	6.637	225348	Wdr36	chr18	+	NM_1448	P20553	protein_cc	5730444A	WD repea	Wdr36
9472	Camk4	0.613	0.347	0.213	0.010	0.007	0.051	3.523	2.818	1.290	12326	Camk4	chr18	+	NM_0097	P12130	protein_cc	A430110E	calcium/ca	Camk4
9473	Stard4	0.505	1.956	0.988	0.000	0.000	0.896	8.349	7.363	8.331	170459	Stard4	chr18	-	NM_1337	P12925	protein_cc	4632419C	StAR-relat	Stard4
9474	Nrep	0.063	1.356	0.086	0.000	0.343	0.005	7.713	3.729	4.168	27528	Nrep	chr18	-	NM_0530	P817	protein_cc	AI325076 |	neuronal r	Nrep
9475	Gm10549	1.000	1.000	1.000	1.000	1.000	1.000	0.000	0.000	0.000	433171	Gm10549	chr18	+	NR_04541	.	lncRNA	ENSMUSG	predicted	Gm10549
9476	Epb4.1l4a	0.436	1.650	0.720	0.000	0.202	0.452	8.652	7.455	8.178	.	Epb4.1l4a	chr18	+	NR_03815	.	.	.	.	Epb4.1l4a
9477	Epb4.1l4a	2.336	1.184	2.765	0.421	0.788	0.085	0.753	1.977	2.220	.	Epb4.1l4a	chr18	-	NM_0135	P24811	.	.	.	Epb4.1l4a
9478	Gm10548	0.560	1.371	0.768	0.184	0.191	0.687	0.857	0.021	0.477	633395	Gm10548	chr18	-	NR_04053	.	misc_RNA	ENSMUSG	predicted	Gm10548
9479	Apc	1.158	0.881	1.020	0.017	0.165	0.903	8.253	8.465	8.282	11789	Apc	chr18	+	NM_0074	P23348	protein_cc	AI047805 |	adenomat	Apc
9480	Srp19	3.929	0.818	3.215	0.000	0.127	0.063	8.620	10.594	10.305	66384	Srp19	chr18	+	NM_0255	P9628	protein_cc	2310020D	signal reco	Srp19
9481	Reep5	1.405	1.648	2.315	0.006	0.010	0.059	7.027	7.518	8.238	13476	Reep5	chr18	-	NM_0078	P26074	protein_cc	AI324241 |	receptor a	Reep5
9482	Pkd2l2	2.434	1.165	2.836	0.170	0.697	0.196	4.223	5.506	5.727	53871	Pkd2l2	chr18	+	NM_0011	P24878	protein_cc	TRPP5	polycystic	Pkd2l2
9483	Fam13b	0.974	0.806	0.785	0.869	0.276	0.135	7.086	7.048	6.737	225358	Fam13b	chr18	-	NM_1460	P24507	protein_cc	2610024E	family wit	Fam13b
9484	Wnt8a	1.000	1.000	1.000	1.000	1.000	1.000	0.000	0.000	0.000	20890	Wnt8a	chr18	+	NM_0092	P12569	protein_cc	Stra11 | W	wingless-t	Wnt8a
9485	Nme5	0.462	1.764	0.815	0.184	0.188	0.805	1.147	0.033	0.851	75533	Nme5	chr18	-	NM_0806	P21444	protein_cc	1700019D	NME/NM2	Nme5
9486	4933408B	0.817	0.363	0.297	0.780	1.000	0.422	1.754	1.462	0.000	271508	4933408B	chr18	+	NM_1777	P10102	protein_cc	.	RIKEN cDN	4933408B
9487	Brd8	1.964	0.724	1.422	0.013	0.073	0.088	7.592	8.565	8.100	78656	Brd8	chr18	-	NM_0301	P11050	protein_cc	2610007E	bromodor	Brd8
9488	Kif20a	1.328	0.411	0.545	0.001	0.000	0.062	5.944	6.354	5.070	19348	Kif20a	chr18	+	NM_0011	P14809	protein_cc	AA415432	kinesin fan	Kif20a
9489	Cdc23	1.469	0.407	0.598	0.000	0.000	0.090	5.778	6.333	5.035	52563	Cdc23	chr18	-	NM_1783	P26496	protein_cc	6030435O	CDC23 cel	Cdc23
9490	Gfra3	1.000	1.000	1.000	1.000	1.000	1.000	0.000	0.000	0.000	14587	Gfra3	chr18	-	NM_0102	P12831	protein_cc	GFRalpha3	glial cell lin	Gfra3
9491	Cdc25c	1.580	0.633	1.000	0.305	1.000	0.423	0.000	0.660	0.000	12532	Cdc25c	chr18	-	NM_0098	P12890	protein_cc	Cdc25	cell divisio	Cdc25c
9492	Fam53c	2.068	0.676	1.398	0.034	0.172	0.422	7.395	8.443	7.878	66306	Fam53c	chr18	+	NM_1751	P21539	protein_cc	2810012G	family wit	Fam53c
9493	Kdm3b	2.244	0.582	1.306	0.049	0.110	0.432	5.202	6.368	5.587	277250	Kdm3b	chr18	+	NM_0010	P22151	protein_cc	5830462I2	KDM3B lys	Kdm3b
9494	Reep2	4.539	0.644	2.924	0.020	0.243	0.235	0.001	2.184	1.549	225362	Reep2	chr18	+	NM_1448	P26428	protein_cc	.	receptor a	Reep2
9495	Egr1	4.215	0.256	1.077	0.000	0.000	0.806	7.946	10.021	8.053	13653	Egr1	chr18	+	NM_0079	P3328	protein_cc	A530045N	early grow	Egr1
9496	Etf1	2.331	0.924	2.155	0.002	0.659	0.167	9.116	10.337	10.223	225363	Etf1	chr18	-	NM_1448	P792	protein_cc	AI463371 |	eukaryotic	Etf1
9497	Hspa9	0.824	0.999	0.823	0.015	0.975	0.112	11.900	11.620	11.618	15526	Hspa9	chr18	-	NM_0104	P13824	protein_cc	74kDa | Cs	heat shocl	Hspa9
9498	Ctnna1	1.282	0.941	1.206	0.021	0.287	0.324	9.451	9.809	9.721	12385	Ctnna1	chr18	+	NM_0098	P21691	protein_cc	2010010M	catenin (ca	Ctnna1
9499	Lrrtm2	0.895	1.317	1.178	0.741	0.672	0.868	1.949	1.789	2.186	107065	Lrrtm2	chr18	-	NM_1780	P23624	protein_cc	AI851755 |	leucine ric	Lrrtm2
9500	Sil1	0.594	1.025	0.609	0.001	0.865	0.077	9.341	8.589	8.625	81500	Sil1	chr18	-	NM_0307	P25354	protein_cc	1810057E	endoplasm	Sil1
9501	Snhg4	1.118	0.850	0.950	0.794	0.621	0.959	4.655	4.817	4.582	10050338	Snhg4	chr18	+	NR_03807	.	lncRNA	Gm17457	small nucl	Snhg4
9502	Snora74a	3.926	0.357	1.400	0.118	0.167	0.423	0.001	1.974	0.487	436583	Snora74a	chr18	+	NR_00290	.	snoRNA	EG436583	small nucl	Snora74a
9503	Matr3	1.383	1.030	1.425	0.034	0.844	0.301	8.756	9.224	9.267	17184	Matr3	chr18	+	NM_0107	P12863	protein_cc	1110061A	matrin 3	Matr3
9504	Paip2	1.149	1.008	1.158	0.482	0.975	0.694	10.005	10.205	10.217	67869	Paip2	chr18	+	NM_0264	P2020	protein_cc	2310050K	polyadeny	Paip2
9505	Slc23a1	0.185	2.718	0.502	0.000	0.000	0.007	9.493	7.057	8.499	20522	Slc23a1	chr18	-	NM_0113	P16528	protein_cc	D18Ucla2	solute car	Slc23a1
9506	Mzb1	0.293	0.940	0.275	0.061	0.850	0.228	3.585	1.812	1.722	69816	Mzb1	chr18	-	NM_0272	P26877	protein_cc	2010001M	marginal z	Mzb1
9507	Prob1	0.566	2.039	1.154	0.101	0.137	0.770	5.178	4.357	5.385	381148	Prob1	chr18	-	NM_0012	P21088	protein_cc	Gm1614	proline ric	Prob1
9508	Spata24	0.435	13.219	5.745	0.101	0.001	0.040	2.778	1.576	5.300	71242	Spata24	chr18	-	NM_0294	P15820	protein_cc	2700012K	spermatog	Spata24
9509	Dnajc18	1.078	1.430	1.541	0.775	0.138	0.088	5.856	5.964	6.480	76594	Dnajc18	chr18	-	NM_0296	P7230	protein_cc	2700075B	DnaJ heat	Dnajc18
9510	Ecscr	5.480	1.008	5.526	0.000	0.960	0.081	4.691	7.145	7.157	68545	Ecscr	chr18	-	NM_0010	P8920	protein_cc	1110006O	endothelia	Ecscr
9511	1700066B	1.603	1.658	2.658	0.292	0.404	0.423	0.001	0.681	1.411	73449	1700066B	chr18	+	NM_0010	P2515	protein_cc	.	RIKEN cDN	1700066B
9512	Tmem173	9.291	0.469	4.360	0.000	0.000	0.006	4.508	7.724	6.632	72512	Tmem173	chr18	-	NM_0282	P16309	protein_cc	2610307O	transmem	Tmem173
9513	Ube2d2a	1.264	0.995	1.257	0.111	0.968	0.436	9.638	9.976	9.968	56550	Ube2d2a	chr18	+	NM_0199	P10364	protein_cc	1500034D	ubiquitin-	Ube2d2a
9514	Cxxc5	0.120	1.931	0.231	0.000	0.041	0.003	9.208	6.146	7.095	67393	Cxxc5	chr18	+	NM_1336	P9660	protein_cc	4930415K	CXXC finge	Cxxc5
9515	Psd2	1.000	1.000	1.000	1.000	1.000	1.000	0.000	0.000	0.000	74002	Psd2	chr18	+	NM_0287	P12114	protein_cc	6330404E	pleckstrin	Psd2
9516	Nrg2	1.000	1.000	1.000	1.000	1.000	1.000	0.000	0.000	0.000	10004215	Nrg2	chr18	-	NM_0011	P4458	protein_cc	Don1 | NT	neureguli	Nrg2
9517	4930471G	1.000	1.000	1.000	1.000	1.000	1.000	0.000	0.000	0.000	74966	4930471G	chr18	+	NR_01557	.	lncRNA	1700007N	RIKEN cDN	4930471G
9518	Pura	0.658	1.653	1.087	0.015	0.016	0.719	10.096	9.491	10.217	19290	Pura	chr18	+	NM_0089	P4725	protein_cc	CAGER-1 |	purine rich	Pura
9519	Igip	0.263	0.885	0.233	0.000	0.831	0.069	6.920	4.994	4.819	109169	Igip	chr18	+	NM_0012	P18568	protein_cc	6330403M	IgA induci	Igip
9520	Cystm1	0.909	1.804	1.640	0.819	0.288	0.522	3.196	3.059	3.910	66060	Cystm1	chr18	+	NM_0010	P11649	protein_cc	0610010O	cysteine-r	Cystm1
9521	Pfdn1	0.785	1.050	0.824	0.000	0.354	0.103	9.300	8.950	9.021	67199	Pfdn1	chr18	-	NM_0260	P13940	protein_cc	2700086I2	prefoldin	Pfdn1
9522	Hbegf	3.603	0.219	0.789	0.000	0.000	0.542	4.799	6.649	4.458	15200	Hbegf	chr18	-	NM_0104	P26459	protein_cc	AW04731	heparin-bi	Hbegf
9523	Slc4a9	0.552	0.990	0.547	0.182	1.000	0.423	0.871	0.015	0.000	240215	Slc4a9	chr18	+	NM_1728	P22491	protein_cc	AE4 | D63	solute car	Slc4a9
9524	Ankhd1	0.750	0.788	0.591	0.004	0.143	0.120	9.490	9.075	8.730	108857	Ankhd1	chr18	+	NM_1753	P729	protein_cc	1110004O	ankyrin re	Ankhd1
9525	Eif4ebp3	2.202	1.852	4.077	0.000	0.032	0.084	8.867	10.005	10.894	108112	Eif4ebp3	chr18	+	NM_2012	P22762	protein_cc	4e-bp3	eukaryotic	Eif4ebp3
9526	Sra1	1.528	0.967	1.477	0.155	0.873	0.166	8.995	9.607	9.558	24068	Sra1	chr18	-	NR_02835	.	protein_cc	AA959952	steroid re	Sra1
9527	Apbb3	5.408	0.347	1.877	0.000	0.002	0.254	6.100	8.536	7.009	225372	Apbb3	chr18	-	NM_1460	P16910	protein_cc	Fe65l2 | Ri	amyloid b	Apbb3
9528	Slc35a4	1.536	1.090	1.674	0.110	0.654	0.076	6.409	7.028	7.153	67843	Slc35a4	chr18	+	NM_0010	P5625	protein_cc	2610030J1	solute car	Slc35a4
9529	E230025N	2.149	0.465	1.000	0.306	1.000	0.423	0.000	1.104	0.000	240216	E230025N	chr18	-	NM_1728	P24046	protein_cc	EG240216	Riken cDN	E230025N
9530	Cd14	278.173	0.855	237.814	0.000	0.205	0.006	4.876	12.996	12.769	12475	Cd14	chr18	-	NM_0098	P18689	protein_cc	.	CD14 anti	Cd14
9531	Tmco6	1.063	1.006	1.069	0.425	0.940	0.496	6.350	6.437	6.446	71983	Tmco6	chr18	+	NM_0280	P13538	protein_cc	2410015B	transmem	Tmco6
9532	Ndufa2	0.934	1.429	1.335	0.322	0.002	0.003	11.672	11.574	12.089	17991	Ndufa2	chr18	-	NM_0108	P9455	protein_cc	AV000592	NADH deh	Ndufa2
9533	Ik	1.740	0.672	1.169	0.000	0.001	0.126	9.536	10.336	9.762	24010	Ik	chr18	+	NM_0118	P3917	protein_cc	MuRED	IK cytokin	Ik
9534	Wdr55	1.237	1.105	1.367	0.177	0.514	0.262	6.860	7.166	7.311	67936	Wdr55	chr18	+	NM_0264	P19969	protein_cc	2410080P	WD repea	Wdr55
9535	Dnd1	1.254	1.509	1.891	0.164	0.034	0.161	5.800	6.126	6.720	213236	Dnd1	chr18	-	NM_1733	P22000	protein_cc	BC034897	DND micro	Dnd1
9536	Hars	1.267	1.321	1.674	0.000	0.000	0.003	7.806	8.147	8.549	15115	Hars	chr18	-	NM_0082	P25028	protein_cc	MMHRS	histidyl-tR	Hars
9537	Hars2	0.660	0.832	0.549	0.000	0.005	0.011	7.876	7.276	7.010	70791	Hars2	chr18	+	NM_0806	P23415	protein_cc	4631412B	histidyl-tR	Hars2
9538	Zmat2	0.957	1.421	1.360	0.438	0.000	0.054	9.894	9.831	10.338	66492	Zmat2	chr18	+	NM_0255	P22493	protein_cc	2610510D	zinc finger	Zmat2
9539	Vaultrc5	4.896	0.920	4.503	0.013	0.890	0.485	5.348	7.639	7.519	378472	Vaultrc5	chr18	+	NR_02788	.	lncRNA	Gm22653	vault RNA	Vaultrc5
9540	Gm6756	1.000	1.000	1.000	1.000	1.000	1.000	0.000	0.000	0.000	627427	Gm6756	chr18	+	NR_07639	.	misc_RNA	EG627427	predicted	Gm6756
9541	Pcdha1	1.000	1.000	1.000	1.000	1.000	1.000	0.000	0.000	0.000	116731	Pcdha1	chr18	+	NM_0540	P18050	protein_cc	CNR	protocadh	Pcdha1
9542	Pcdha2	1.000	1.000	1.000	1.000	1.000	1.000	0.000	0.000	0.000	353234	Pcdha2	chr18	+	NM_1981	P17088	protein_cc	.	protocadh	Pcdha2

9543	Pcdha3	1.000	1.000	1.000	1.000	1.000	1.000	0.000	0.000	0.000	192163	Pcdha3	chr18	+	NM_1386	P1137	protein_cc	mKIAA034	protocadh	Pcdha3
9544	Gm37013	1.000	1.000	1.000	1.000	1.000	1.000	0.000	0.000	0.000	10038486	Gm37013	chr18	+	NM_0011	P17538	protein_cc	Pcdha4-g	predicted	Gm37013
9545	Pcdha4	1.000	1.000	1.000	1.000	1.000	1.000	0.000	0.000	0.000	12936	Pcdha4	chr18	+	NM_0077	P13284	protein_cc	Cnr1| Crn	protocadh	Pcdha4
9546	Pcdha5	1.000	1.000	1.000	1.000	1.000	1.000	0.000	0.000	0.000	12941	Pcdha5	chr18	+	NM_0099	P23681	protein_cc	Cnr6| Crn	protocadh	Pcdha5
9547	Gm38666	0.716	0.995	0.712	0.182	1.000	0.423	0.490	0.008	0.000	10361115	Gm38666	chr18	+	NM_0013	P13459	protein_cc	Pcdha6-g	predicted	Gm38666
9548	Pcdha6	0.716	0.995	0.712	0.182	1.000	0.423	0.490	0.008	0.000	12937	Pcdha6	chr18	+	NM_0077	P15253	protein_cc	CNR| Cnr2	protocadh	Pcdha6
9549	Pcdha7-g	1.000	1.000	1.000	1.000	1.000	1.000	0.000	0.000	0.000	.	Pcdha7-g	chr18	+	NM_0013	P19887	.	.	.	Pcdha7-g
9550	Pcdha7	1.000	1.000	1.000	1.000	1.000	1.000	0.000	0.000	0.000	12939	Pcdha7	chr18	+	NM_0099	P1973	protein_cc	Cnr4| Crn	protocadh	Pcdha7
9551	Pcdha8	1.000	1.000	1.000	1.000	1.000	1.000	0.000	0.000	0.000	353235	Pcdha8	chr18	+	NM_2012	P5660	protein_cc	.	protocadh	Pcdha8
9552	Pcdha9	1.000	1.000	1.000	1.000	1.000	1.000	0.000	0.000	0.000	192161	Pcdha9	chr18	+	NM_1386	P2644	protein_cc	.	protocadh	Pcdha9
9553	Pcdha10	1.000	1.000	1.000	1.000	1.000	1.000	0.000	0.000	0.000	12943	Pcdha10	chr18	+	NM_0099	P5155	protein_cc	Cnr3| Cnr	protocadh	Pcdha10
9554	Pcdha11	1.000	1.000	1.000	1.000	1.000	1.000	0.000	0.000	0.000	12942	Pcdha11	chr18	+	NM_0099	P23360	protein_cc	A830022B	protocadh	Pcdha11
9555	Pcdha12	1.000	1.000	1.000	1.000	1.000	1.000	0.000	0.000	0.000	192164	Pcdha12	chr18	+	NM_1386	P26857	protein_cc	Cnr5| Crn	protocadh	Pcdha12
9556	Pcdhac1	0.242	1.360	0.329	0.182	0.211	0.477	2.100	0.052	0.496	353236	Pcdhac1	chr18	+	NM_0010	P7758	protein_cc	CNRc1	protocadh	Pcdhac1
9557	Pcdhac2	1.000	1.000	1.000	1.000	1.000	1.000	0.000	0.000	0.000	353237	Pcdhac2	chr18	+	NM_0010	P21358	protein_cc	CNRc2	protocadh	Pcdhac2
9558	Pcdhb1	1.000	1.000	1.000	1.000	1.000	1.000	0.000	0.000	0.000	93872	Pcdhb1	chr18	+	NM_0531	P1519	protein_cc	PcdhbA	protocadh	Pcdhb1
9559	Pcdhb2	1.000	1.000	1.000	1.000	1.000	1.000	0.000	0.000	0.000	93873	Pcdhb2	chr18	+	NM_0531	P8807	protein_cc	PcdhbB	protocadh	Pcdhb2
9560	Pcdhb3	1.000	1.000	1.000	1.000	1.000	1.000	0.000	0.000	0.000	93874	Pcdhb3	chr18	+	NM_0531	P329	protein_cc	PcdhbC	protocadh	Pcdhb3
9561	Pcdhb4	0.593	3.778	2.241	0.195	0.182	0.546	0.806	0.052	1.969	93875	Pcdhb4	chr18	+	NM_0531	P22824	protein_cc	Pcdhb5A|	protocadh	Pcdhb4
9562	Pcdhb5	1.014	2.240	2.272	0.000	0.182	0.423	0.000	0.021	1.185	93876	Pcdhb5	chr18	+	NM_0531	P24228	protein_cc	Pcdhb4A|	protocadh	Pcdhb5
9563	Pcdhb6	1.000	1.000	1.000	1.000	1.000	1.000	0.000	0.000	0.000	93877	Pcdhb6	chr18	+	NM_0531	P9789	protein_cc	Pcdhb5B|	protocadh	Pcdhb6
9564	Pcdhb7	2.699	2.028	5.472	0.178	0.214	0.227	0.589	2.021	3.041	93878	Pcdhb7	chr18	+	NM_0531	P12095	protein_cc	Pcdhb4B|	protocadh	Pcdhb7
9565	Pcdhb8	1.000	1.000	1.000	1.000	1.000	1.000	0.000	0.000	0.000	93879	Pcdhb8	chr18	+	NM_0531	P17287	protein_cc	Pcdhb5C|	protocadh	Pcdhb8
9566	Pcdhb9	0.560	1.371	0.768	0.184	0.191	0.687	0.857	0.021	0.477	93880	Pcdhb9	chr18	+	NM_0531	P25159	protein_cc	Pcdhb4C|	protocadh	Pcdhb9
9567	Pcdhb10	1.000	1.000	1.000	1.000	1.000	1.000	0.000	0.000	0.000	93881	Pcdhb10	chr18	+	NM_0531	P5298	protein_cc	Pcdhb5D|	protocadh	Pcdhb10
9568	Pcdhb11	1.000	1.000	1.000	1.000	1.000	1.000	0.000	0.000	0.000	93882	Pcdhb11	chr18	+	NM_0531	P10945	protein_cc	Pcdhb5E|	protocadh	Pcdhb11
9569	Pcdhb12	0.457	1.401	0.640	0.183	0.194	0.600	1.158	0.027	0.514	93883	Pcdhb12	chr18	+	NM_0531	P25931	protein_cc	Pcdh3| Pc	protocadh	Pcdhb12
9570	Pcdhb13	1.000	1.000	1.000	1.000	1.000	1.000	0.000	0.000	0.000	93884	Pcdhb13	chr18	+	NM_0531	P15234	protein_cc	Pcdbh6| P	protocadh	Pcdhb13
9571	Pcdhb14	1.932	0.935	1.807	0.561	0.945	0.680	1.834	2.784	2.688	93885	Pcdhb14	chr18	+	NM_0531	P10346	protein_cc	2210006M	protocadh	Pcdhb14
9572	Pcdhb15	1.610	2.012	3.239	0.289	0.327	0.423	0.001	0.688	1.696	93886	Pcdhb15	chr18	+	NM_0531	P8372	protein_cc	Pcdhb7| P	protocadh	Pcdhb15
9573	Pcdhb16	0.668	1.020	0.681	0.482	0.958	0.728	1.749	1.166	1.194	93887	Pcdhb16	chr18	+	NM_0531	P25062	protein_cc	Pcdhb8| P	protocadh	Pcdhb16
9574	Pcdhb17	4.572	0.821	3.754	0.024	0.539	0.132	0.001	2.194	1.910	93888	Pcdhb17	chr18	+	NM_0531	P9357	protein_cc	mKIAA162	protocadh	Pcdhb17
9575	Pcdhb18	1.983	0.879	1.742	0.187	0.697	0.242	0.772	1.759	1.572	93889	Pcdhb18	chr18	+	NM_0531	P13389	protein_cc	Pcdhb9| P	protocadh	Pcdhb18
9576	Pcdhb19	0.310	0.817	0.253	0.017	0.613	0.096	2.402	0.713	0.421	93890	Pcdhb19	chr18	+	NM_0531	P12653	protein_cc	Pcdhb11|	protocadh	Pcdhb19
9577	Pcdhb20	0.398	2.684	1.069	0.185	0.184	0.947	1.376	0.048	1.472	93891	Pcdhb20	chr18	+	NM_0531	P23022	protein_cc	Pcdhb14|	protocadh	Pcdhb20
9578	Pcdhb21	2.747	1.124	3.088	0.301	0.851	0.275	0.001	1.459	1.628	93892	Pcdhb21	chr18	+	NM_0531	P15194	protein_cc	Pcdhb18|	protocadh	Pcdhb21
9579	Pcdhb22	2.195	1.569	3.446	0.509	0.517	0.222	1.658	2.793	3.443	93893	Pcdhb22	chr18	+	NM_0531	P25056	protein_cc	Pcdhb15|	protocadh	Pcdhb22
9580	Slc25a2	0.537	0.619	0.333	0.355	1.000	0.423	1.588	0.692	0.000	83885	Slc25a2	chr18	-	NM_0011	P6158	protein_cc	Ornt2	solute car	Slc25a2
9581	Taf7	2.634	0.278	0.731	0.002	0.000	0.429	6.479	7.876	6.027	24074	Taf7	chr18	-	NM_1757	P2793	protein_cc	55kDa| AI	TATA-box	Taf7
9582	Pcdhga1	2.237	3.648	8.162	0.288	0.032	0.102	0.001	1.163	3.030	93709	Pcdhga1	chr18	+	NM_0335	P14781	protein_cc	.	protocadh	Pcdhga1
9583	Pcdhga2	0.579	2.593	1.501	0.190	0.183	0.676	0.827	0.038	1.413	93710	Pcdhga2	chr18	+	NM_0335	P24707	protein_cc	mKIAA058	protocadh	Pcdhga2
9584	Pcdhga3	1.729	2.299	3.974	0.077	0.000	0.027	1.004	1.794	2.995	93711	Pcdhga3	chr18	+	NM_0335	P4231	protein_cc	.	protocadh	Pcdhga3
9585	Pcdhgb1	0.716	0.995	0.712	0.182	1.000	0.423	0.490	0.008	0.000	93699	Pcdhgb1	chr18	+	NM_0335	P18434	protein_cc	.	protocadh	Pcdhgb1
9586	Pcdhga4	3.881	0.258	0.999	0.307	1.000	0.423	0.001	1.957	0.000	93712	Pcdhga4	chr18	+	NM_0335	P14040	protein_cc	.	protocadh	Pcdhga4
9587	Pcdhgb2	1.005	1.439	1.446	0.000	0.182	0.423	0.000	0.008	0.532	93700	Pcdhgb2	chr18	+	NM_0335	P21102	protein_cc	.	protocadh	Pcdhgb2
9588	Pcdhga5	1.000	1.000	1.000	1.000	1.000	1.000	0.000	0.000	0.000	93713	Pcdhga5	chr18	+	NM_0335	P11937	protein_cc	.	protocadh	Pcdhga5
9589	Pcdhga6	1.000	1.000	1.000	1.000	1.000	1.000	0.000	0.000	0.000	93714	Pcdhga6	chr18	+	NM_0335	P23597	protein_cc	.	protocadh	Pcdhga6
9590	Pcdhga7	1.572	0.636	1.000	0.304	1.000	0.423	0.000	0.653	0.000	93715	Pcdhga7	chr18	+	NM_0335	P17282	protein_cc	.	protocadh	Pcdhga7
9591	Pcdhgb4	1.000	1.000	1.000	1.000	1.000	1.000	0.000	0.000	0.000	93701	Pcdhgb4	chr18	+	NM_0335	P6412	protein_cc	.	protocadh	Pcdhgb4
9592	Pcdhga8	1.000	1.000	1.000	1.000	1.000	1.000	0.000	0.000	0.000	93716	Pcdhga8	chr18	+	NM_0335	P20356	protein_cc	mKIAA405	protocadh	Pcdhga8
9593	Pcdhgb5	1.580	0.633	1.000	0.305	1.000	0.423	0.000	0.660	0.000	93702	Pcdhgb5	chr18	+	NM_0335	P26001	protein_cc	.	protocadh	Pcdhgb5
9594	Pcdhga9	1.499	0.993	1.489	0.104	0.980	0.408	7.626	8.210	8.201	93717	Pcdhga9	chr18	+	NM_0335	P17516	protein_cc	.	protocadh	Pcdhga9
9595	Pcdhgb6	1.005	1.439	1.446	0.000	0.182	0.423	0.000	0.008	0.532	93703	Pcdhgb6	chr18	+	NM_0335	P3300	protein_cc	.	protocadh	Pcdhgb6
9596	Pcdhga10	1.000	1.000	1.000	1.000	1.000	1.000	0.000	0.000	0.000	93722	Pcdhga10	chr18	+	NM_0335	P14342	protein_cc	.	protocadh	Pcdhga10
9597	Pcdhgb7	0.716	0.995	0.712	0.182	1.000	0.423	0.490	0.008	0.000	93704	Pcdhgb7	chr18	+	NM_0335	P16356	protein_cc	.	protocadh	Pcdhgb7
9598	Pcdhga11	1.023	3.051	3.120	0.000	0.182	0.423	0.000	0.033	1.642	93723	Pcdhga11	chr18	+	NM_0335	P3989	protein_cc	.	protocadh	Pcdhga11
9599	Pcdhgb8	0.716	0.995	0.712	0.182	1.000	0.423	0.490	0.008	0.000	93705	Pcdhgb8	chr18	+	NM_0335	P1896	protein_cc	.	protocadh	Pcdhgb8
9600	Pcdhga12	1.005	1.439	1.446	0.000	0.182	0.423	0.000	0.008	0.532	93724	Pcdhga12	chr18	+	NM_0335	P20812	protein_cc	pc2c| Pcd	protocadh	Pcdhga12
9601	Pcdhgc3	0.716	0.995	0.712	0.182	1.000	0.423	0.490	0.008	0.000	93706	Pcdhgc3	chr18	+	NM_0335	P17131	protein_cc	PC43| Pcd	protocadh	Pcdhgc3
9602	Pcdhgc4	1.000	1.000	1.000	1.000	1.000	1.000	0.000	0.000	0.000	93707	Pcdhgc4	chr18	+	NM_0335	P13078	protein_cc	.	protocadh	Pcdhgc4
9603	Pcdhgc5	1.000	1.000	1.000	1.000	1.000	1.000	0.000	0.000	0.000	93708	Pcdhgc5	chr18	+	NM_0335	P5989	protein_cc	.	protocadh	Pcdhgc5
9604	Diap1	0.663	0.893	0.592	0.000	0.392	0.077	9.241	8.648	8.484	.	Diap1	chr18	-	NM_0078	P8107	.	.	.	Diap1
9605	Hdac3	0.652	1.532	0.999	0.040	0.029	0.988	6.556	5.938	6.554	15183	Hdac3	chr18	-	NM_0104	P7512	protein_cc	AW53736	histone de	Hdac3
9606	Rell2	8.805	1.134	9.987	0.008	0.649	0.094	0.002	3.140	3.322	225392	Rell2	chr18	+	NM_1537	P10323	protein_cc	4631403P	RELT-like 2	Rell2
9607	Fchsd1	3.810	1.428	5.442	0.005	0.172	0.112	2.496	4.426	4.940	319262	Fchsd1	chr18	-	NM_1756	P25385	protein_cc	A030002D	FCH and d	Fchsd1
9608	Arap3	1.492	0.856	1.276	0.003	0.515	0.625	5.610	6.187	5.962	106952	Arap3	chr18	-	NM_0012	P5124	protein_cc	Centd3| c	ArfGAP wi	Arap3
9609	Pcdh1	0.958	0.638	0.611	0.516	0.007	0.047	8.126	8.064	7.415	75599	Pcdh1	chr18	-	NM_0293	P25359	protein_cc	2010005A	protocadh	Pcdh1
9610	1700086O	0.983	2.980	2.928	0.962	0.009	0.150	4.720	4.695	6.270	73516	1700086O	chr18	-	NR_02790	.	lncRNA		RIKEN cDN	1700086O
9611	0610009O	0.818	1.158	0.947	0.074	0.004	0.704	7.715	7.425	7.636	66839	0610009O	chr18	+	NM_0241	P7625	protein_cc	2700004E	RIKEN cDN	0610009O
9612	Pcdh12	0.058	5.282	0.305	0.005	0.021	0.139	4.910	0.795	3.196	53601	Pcdh12	chr18	-	NM_0173	P11441	protein_cc	Pcdh14| V	protocadh	Pcdh12
9613	Rnf14	0.792	1.115	0.882	0.000	0.000	0.002	9.460	9.123	9.280	56736	Rnf14	chr18	+	NM_0200	P25566	protein_cc	2310075C	ring finger	Rnf14
9614	Gnpda1	0.168	0.971	0.163	0.000	0.671	0.022	7.845	5.271	5.229	26384	Gnpda1	chr18	-	NM_0119	P17970	protein_cc	Gnp1| GN	glucosami	Gnpda1
9615	Ndfip1	0.524	1.458	0.764	0.002	0.003	0.178	7.467	6.536	7.080	65113	Ndfip1	chr18	+	NM_0229	P23923	protein_cc	0610010M	Nedd4 fan	Ndfip1
9616	Spry4	0.362	0.840	0.304	0.021	0.517	0.110	6.634	5.170	4.919	24066	Spry4	chr18	-	NM_0118	P19395	protein_cc	A030006C	sprouty ho	Spry4
9617	Fgf1	0.118	1.789	0.211	0.000	0.170	0.013	9.442	6.360	7.199	14164	Fgf1	chr18	-	NM_0101	P21638	protein_cc	Dffrx| Fan	fibroblast	Fgf1
9618	Gm5820	0.716	0.995	0.712	0.182	1.000	0.423	0.490	0.008	0.000	545253	Gm5820	chr18	+	NM_0010	P13368	protein_cc	EG545253	predicted	Gm5820
9619	Arhgap26	0.430	1.586	0.681	0.000	0.009	0.023	6.292	5.073	5.738	71302	Arhgap26	chr18	+	NM_1751	P13409	protein_cc	1810044B	Rho GTPas	Arhgap26
9620	Nr3c1	0.437	1.041	0.455	0.009	0.810	0.077	9.671	8.478	8.536	14815	Nr3c1	chr18	-	NM_0081	P22801	protein_cc	GR| Grl-1|	nuclear re	Nr3c1
9621	Pabpc2	1.000	1.000	1.000	1.000	1.000	1.000	0.000	0.000	0.000	18459	Pabpc2	chr18	+	NM_0110	P2761	protein_cc	Pabp| Pab	poly(A) bin	Pabpc2
9622	Yipf5	2.432	0.758	1.843	0.000	0.000	0.000	9.552	10.834	10.434	67180	Yipf5	chr18	-	NM_0233	P17327	protein_cc	2610311I1	Yip1 doma	Yipf5
9623	2900055J2	1.000	1.000	1.000	1.000	1.000	1.000	0.000	0.000	0.000	73001	2900055J2	chr18	+	NR_04517	.	lncRNA		RIKEN cDN	2900055J2
9624	Kctd16	1.000	1.000	1.000	1.000	1.000	1.000	0.000	0.000	0.000	383348	Kctd16	chr18	+	NM_0261	P7062	protein_cc	4930434H	potassium	Kctd16
9625	Prelid2	2.040	0.945	1.927	0.164	0.905	0.479	5.221	6.250	6.167	77619	Prelid2	chr18	-	NM_0299	P18878	protein_cc	1700003A	PRELI dom	Prelid2
9626	Grxcr2	1.000	1.000	1.000	1.000	1.000	1.000	0.000	0.000	0.000	332309	Grxcr2	chr18	+	NM_0010	P3931	protein_cc	Gm851	glutaredox	Grxcr2
9627	Sh3rf2	1.318	0.303	0.399	0.731	1.000	0.423	1.326	1.724	0.000	269016	Sh3rf2	chr18	+	NM_0011	P19113	protein_cc	2310046K	SH3 doma	Sh3rf2
9628	Plac8l1	1.000	1.000	1.000	1.000	1.000	1.000	0.000	0.000	0.000	69401	Plac8l1	chr18	-	NM_0270	P25256	protein_cc	1700009N	PLAC8-like	Plac8l1
9629	Lars	2.359	0.845	1.993	0.000	0.027	0.019	8.158	9.396	9.153	107045	Lars	chr18	-	NM_1341	P19384	protein_cc	2310045K	leucyl-tRN	Lars
9630	Gm4013	0.388	2.251	0.873	0.000	0.127	0.788	4.623	3.257	4.428	10004275	Gm4013	chr18	-	NR_03345	.	lncRNA	10004275	predicted	Gm4013
9631	Rbm27	1.767	0.624	1.102	0.001	0.002	0.488	8.120	8.941	8.260	225432	Rbm27	chr18	+	NM_1726	P9390	protein_cc	A730010I	RNA bindi	Rbm27
9632	Pou4f3	1.000	1.000	1.000	1.000	1.000	1.000	0.000	0.000	0.000	18998	Pou4f3	chr18	+	NM_1389	P7634	protein_cc	Brn3.1| Br	POU doma	Pou4f3
9633	Tcerg1	2.761	0.666	1.839	0.001	0.017	0.077	8.159	9.624	9.038	56070	Tcerg1	chr18	+	NM_0010	P11233	protein_cc	2410022J	transcripti	Tcerg1

9634	Gpr151	1.000	1.000	1.000	1.000	1.000	1.000	0.000	0.000	0.000	240239	Gpr151	chr18	-	NM_1815	P15722	protein_cc	C130082O	G protein-	Gpr151
9635	Ppp2r2b	0.348	3.091	1.075	0.185	0.184	0.947	1.580	0.056	1.685	72930	Ppp2r2b	chr18	-	NR_07358	.	protein_cc	2900026H	protein ph	Ppp2r2b
9636	Stk32a	1.000	1.000	1.000	1.000	1.000	1.000	0.000	0.000	0.000	269019	Stk32a	chr18	+	NM_1787	P18065	protein_cc	A930015B	serine/thr	Stk32a
9637	Dpysl3	2.409	0.622	1.498	0.042	0.120	0.308	5.898	7.167	6.481	22240	Dpysl3	chr18	-	NM_0012	P3608	protein_cc	CRMP-4| I	dihydropy	Dpysl3
9638	Jakmip2	1.089	3.063	3.335	0.833	0.250	0.477	0.588	0.711	2.326	76217	Jakmip2	chr18	-	NM_0011	P7609	protein_cc	6430702L	janus kina	Jakmip2
9639	Spink1	2.353	6.331	14.898	0.003	0.072	0.240	2.423	3.658	6.320	20730	Spink1	chr18	-	NM_0092	P17917	protein_cc	p12| Spinl	serine pep	Spink1
9640	Scgb3a2	1.000	1.000	1.000	1.000	1.000	1.000	0.000	0.000	0.000	117158	Scgb3a2	chr18	+	NM_0012	P6192	protein_cc	LuLeu1| P	secretoglo	Scgb3a2
9641	Gm94	1.000	1.000	1.000	1.000	1.000	1.000	0.000	0.000	0.000	225443	Gm94	chr18	-	NM_0010	P12886	protein_cc	.	predicted	Gm94
9642	Spink5	2.490	1.894	4.716	0.007	0.001	0.004	3.674	4.990	5.912	72432	Spink5	chr18	+	NM_0010	P21627	protein_cc	2310065D	serine pep	Spink5
9643	Spink14	1.000	1.000	1.000	1.000	1.000	1.000	0.000	0.000	0.000	433178	Spink14	chr18	+	NM_0010	P17399	protein_cc	EG433178	serine pep	Spink14
9644	Spink6	0.504	0.990	0.499	0.181	1.000	0.423	1.002	0.015	0.000	433180	Spink6	chr18	+	NM_0010	P1518	protein_cc	EG433180	serine pep	Spink6
9645	Spink12	1.000	1.000	1.000	1.000	1.000	1.000	0.000	0.000	0.000	78242	Spink12	chr18	+	NM_0300	P13151	protein_cc	9230117E	serine pep	Spink12
9646	Gm10267	1.000	1.000	1.000	1.000	1.000	1.000	0.000	0.000	0.000	10004285	Gm10267	chr18	-	NM_0012	P12756	protein_cc	ENSMUSG	predicted	Gm10267
9647	Spinkl	1.000	1.000	1.000	1.000	1.000	1.000	0.000	0.000	0.000	77424	Spinkl	chr18	-	NM_1831	P8145	protein_cc	9530002K	serine pro	Spinkl
9648	Spink11	1.000	1.000	1.000	1.000	1.000	1.000	0.000	0.000	0.000	433181	Spink11	chr18	-	NM_0010	P1989	protein_cc	9230112K	serine pep	Spink11
9649	Npy6r	1.000	1.000	1.000	1.000	1.000	1.000	0.000	0.000	0.000	18169	Npy6r	chr18	+	NM_0109	P24950	protein_cc	.	neuropept	Npy6r
9650	Myot	0.457	1.358	0.620	0.183	0.195	0.578	1.158	0.027	0.468	58916	Myot	chr18	+	NM_0010	P3500	protein_cc	5530402I0	myotilin	Myot
9651	Dcp2	2.009	0.603	1.212	0.009	0.041	0.519	6.958	7.965	7.235	70640	Dcp2	chr18	+	NM_0274	P5562	protein_cc	2410015D	decapping	Dcp2
9652	Mcc	0.084	1.870	0.157	0.008	0.223	0.113	7.241	3.668	4.571	328949	Mcc	chr18	-	NM_0010	P5131	protein_cc	D18Ertd45	mutated in	Mcc
9653	A930012L	1.053	0.632	0.666	0.897	1.000	0.423	0.587	0.661	0.000	626275	A930012L	chr18	+	NR_02685	.	lncRNA	.	RIKEN cDN	A930012L
9654	Ythdc2	1.889	1.470	2.777	0.000	0.006	0.022	4.920	5.837	6.393	240255	Ythdc2	chr18	+	NM_0011	P24292	protein_cc	3010002F	YTH doma	Ythdc2
9655	Kcnn2	0.113	1.212	0.137	0.000	0.159	0.010	6.661	3.517	3.795	140492	Kcnn2	chr18	+	NM_0804	P6358	protein_cc	bc| fri| KC	potassium	Kcnn2
9656	A330093E	0.254	1.790	0.454	0.000	0.193	0.122	2.030	0.053	0.893	107003	A330093E	chr18	-	NR_04034	.	lncRNA	AU043608	RIKEN cDN	A330093E
9657	Trim36	2.059	0.293	0.602	0.072	0.030	0.148	1.139	2.181	0.408	28105	Trim36	chr18	-	NM_0011	P22648	protein_cc	D18Wsu1(	tripartite	Trim36
9658	Pggt1b	1.556	0.544	0.847	0.069	0.003	0.700	4.236	4.874	3.997	225467	Pggt1b	chr18	-	NM_1726	P3962	protein_cc	2010207C	protein ge	Pggt1b
9659	Ccdc112	2.208	0.457	1.010	0.041	0.054	0.976	2.256	3.399	2.271	240261	Ccdc112	chr18	-	NM_0011	P19436	protein_cc	8430438N	coiled-coil	Ccdc112
9660	Mospd4	2.171	1.275	2.769	0.299	0.704	0.423	0.001	1.119	1.470	72076	Mospd4	chr18	-	NR_04543	.	misc_RNA	2010013H	motile spe	Mospd4
9661	Fem1c	1.533	0.891	1.366	0.159	0.545	0.584	4.036	4.652	4.486	240263	Fem1c	chr18	-	NM_1734	P25869	protein_cc	2610312A	fem-1 hon	Fem1c
9662	Ticam2	3.603	0.947	3.413	0.002	0.683	0.087	1.475	3.324	3.246	225471	Ticam2	chr18	-	NM_1733	P7958	protein_cc	B430113A	toll-like re	Ticam2
9663	Tmed7	1.576	1.047	1.651	0.025	0.840	0.408	8.640	9.296	9.363	66676	Tmed7	chr18	-	NM_0256	P15158	protein_cc	3930401E	transmem	Tmed7
9664	Eif1a	7.303	0.570	4.165	0.000	0.017	0.085	8.463	11.332	10.521	13664	Eif1a	chr18	+	NM_0101	P26796	protein_cc	C76390| E	eukaryotic	Eif1a
9665	Cdo1	0.859	0.916	0.787	0.066	0.726	0.557	14.950	14.730	14.603	12583	Cdo1	chr18	-	NM_0330	P18977	protein_cc	1300002L	cysteine d	Cdo1
9666	Atg12	1.417	1.237	1.752	0.029	0.299	0.240	8.553	9.055	9.362	67526	Atg12	chr18	-	NM_0262	P23700	protein_cc	4931423H	autophagy	Atg12
9667	Ap3s1	1.774	0.792	1.405	0.095	0.326	0.218	6.477	7.305	6.968	11777	Ap3s1	chr18	+	NM_0096	P586	protein_cc	.	adaptor-re	Ap3s1
9668	Lvrn	1.000	1.000	1.000	1.000	1.000	1.000	0.000	0.000	0.000	74574	Lvrn	chr18	+	NM_0290	P9068	protein_cc	4833403I1	laeverin	Lvrn
9669	Arl14epl	1.000	1.000	1.000	1.000	1.000	1.000	0.000	0.000	0.000	381142	Arl14epl	chr18	+	NM_0010	P18904	protein_cc	Gm949	ADP-ribos	Arl14epl
9670	Commd10	1.047	2.060	2.157	0.747	0.000	0.024	5.876	5.942	6.985	69456	Commd10	chr18	+	NM_1783	P240	protein_cc	2310003A	COMM do	Commd10
9671	Sema6a	0.133	1.883	0.250	0.000	0.073	0.019	7.396	4.482	5.395	20358	Sema6a	chr18	-	NM_0187	P15933	protein_cc	A730020P	sema dom	Sema6a
9672	9130209A	1.000	1.000	1.000	1.000	1.000	1.000	0.000	0.000	0.000	77668	9130209A	chr18	+	NR_03345	.	lncRNA	.	RIKEN cDN	9130209A
9673	Gm5095	1.000	1.000	1.000	1.000	1.000	1.000	0.000	0.000	0.000	328953	Gm5095	chr18	+	NR_03345	.	lncRNA	328953| A	predicted	Gm5095
9674	G630055G	1.000	1.000	1.000	1.000	1.000	1.000	0.000	0.000	0.000	414127	G630055G	chr18	+	NR_04540	.	lncRNA	.	RIKEN cDN	G630055G
9675	Eno1b	0.784	0.725	0.569	0.235	0.166	0.105	8.892	8.542	8.078	433182	Eno1b	chr18	+	NM_0010	P6329	protein_cc	EG433182	enolase 1	Eno1b
9676	1700044K	1.000	1.000	1.000	1.000	1.000	1.000	0.000	0.000	0.000	67321	1700044K	chr18	+	NR_03378	.	lncRNA	.	RIKEN cDN	1700044K
9677	Dtwd2	0.727	0.902	0.655	0.520	0.773	0.593	4.896	4.435	4.285	68857	Dtwd2	chr18	-	NM_0268	P20904	protein_cc	1190002H	DTW dom	Dtwd2
9678	1700065O	1.000	1.000	1.000	1.000	1.000	1.000	0.000	0.000	0.000	76609	1700065O	chr18	-	NR_04538	.	lncRNA	.	RIKEN cDN	1700065O
9679	Dmxl1	1.371	1.010	1.384	0.246	0.957	0.257	6.260	6.715	6.729	240283	Dmxl1	chr18	+	NM_0010	P25860	protein_cc	C630007L	Dmx-like 1	Dmxl1
9680	Tnfaip8	1.497	1.486	2.226	0.057	0.076	0.092	4.591	5.174	5.745	106869	Tnfaip8	chr18	+	NM_0011	P9301	protein_cc	AA987150	tumor nec	Tnfaip8
9681	Hsd17b4	0.282	1.359	0.384	0.000	0.041	0.002	10.959	9.135	9.578	15488	Hsd17b4	chr18	+	NM_0082	P18992	protein_cc	17-beta-H	hydroxyst	Hsd17b4
9682	Fam170a	1.000	1.000	1.000	1.000	1.000	1.000	0.000	0.000	0.000	225497	Fam170a	chr18	+	NM_0010	P9500	protein_cc	Gm93| Zn	family wit	Fam170a
9683	Hdhd1a	1.000	1.000	1.000	1.000	1.000	1.000	0.000	0.000	0.000	67365	Hdhd1a	chr18	-	NM_0261	P25572	protein_cc	1700121L	haloacid d	Hdhd1a
9684	Prr16	0.916	0.999	0.915	0.753	0.998	0.794	5.906	5.779	5.778	71373	Prr16	chr18	+	NM_0010	P9216	protein_cc	5430406N	proline ric	Prr16
9685	Ftmt	1.000	1.000	1.000	1.000	1.000	1.000	0.000	0.000	0.000	67634	Ftmt	chr18	+	NM_0262	P9656	protein_cc	4930447C	ferritin mi	Ftmt
9686	Srfbp1	1.173	0.849	0.996	0.487	0.597	0.991	5.221	5.451	5.214	67222	Srfbp1	chr18	+	NM_0260	P9231	protein_cc	2810036K	serum res	Srfbp1
9687	Lox	5.844	1.264	7.390	0.011	0.546	0.214	2.999	5.547	5.885	16948	Lox	chr18	-	NM_0012	P4983	protein_cc	AI893619|	lysyl oxida	Lox
9688	Zfp474	1.000	1.000	1.000	1.000	1.000	1.000	0.000	0.000	0.000	66758	Zfp474	chr18	+	NM_0257	P3738	protein_cc	4933409D	zinc finger	Zfp474
9689	1700034E	1.000	1.000	1.000	1.000	1.000	1.000	0.000	0.000	0.000	78414	1700034E	chr18	+	NM_0300	P6947	protein_cc	.	RIKEN cDN	1700034E
9690	Gykl1	1.000	1.000	1.000	1.000	1.000	1.000	0.000	0.000	0.000	14625	Gykl1	chr18	+	NM_0102	P1575	protein_cc	AI449806|	glycerol ki	Gykl1
9691	Sncaip	0.744	0.764	0.569	0.692	0.685	0.618	1.901	1.475	1.086	67847	Sncaip	chr18	+	NM_0011	P8616	protein_cc	2810407O	synuclein,	Sncaip
9692	Snx2	0.797	1.131	0.901	0.223	0.493	0.284	7.845	7.517	7.695	67804	Snx2	chr18	+	NM_0263	P24677	protein_cc	0610030A	sorting ne	Snx2
9693	Snx24	0.854	1.017	0.868	0.003	0.835	0.339	7.435	7.207	7.231	69226	Snx24	chr18	+	NM_0293	P11458	protein_cc	2810011K	sorting ne	Snx24
9694	Ppic	3.772	0.699	2.635	0.009	0.123	0.008	5.501	7.416	6.899	19038	Ppic	chr18	-	NM_0089	P21023	protein_cc	CyP-20c	peptidylpr	Ppic
9695	Prdm6	0.296	2.102	0.621	0.055	0.007	0.480	1.811	0.052	1.124	225518	Prdm6	chr18	+	NM_0010	P3846	protein_cc	Gm92| PR	PR domair	Prdm6
9696	Gm19466	1.000	1.000	1.000	1.000	1.000	1.000	0.000	0.000	0.000	10050294	Gm19466	chr18	+	NR_04034	.	lncRNA	.	predicted	Gm19466
9697	Cep120	1.027	1.067	1.096	0.341	0.689	0.749	6.488	6.526	6.620	225523	Cep120	chr18	-	NM_1786	P14931	protein_cc	A230075C	centrosom	Cep120
9698	Csnk1g3	0.951	0.995	0.946	0.622	0.953	0.606	7.330	7.257	7.250	70425	Csnk1g3	chr18	+	NM_1528	P6339	protein_cc	3300002K	casein kin	Csnk1g3
9699	Redrum	1.000	1.000	1.000	1.000	1.000	1.000	0.000	0.000	0.000	77433	Redrum	chr18	+	NR_04033	.	lncRNA	9430076G	Redrum, e	Redrum
9700	9330117O	1.000	1.000	1.000	1.000	1.000	1.000	0.000	0.000	0.000	328957	9330117O	chr18	+	NR_04540	.	lncRNA	9330117O	RIKEN cDN	9330117O
9701	Zfp608	1.459	1.741	2.540	0.369	0.025	0.119	4.206	4.751	5.551	269023	Zfp608	chr18	-	NM_1757	P22354	protein_cc	4932417D	zinc finger	Zfp608
9702	Gramd3	0.787	1.105	0.870	0.052	0.275	0.210	8.805	8.459	8.603	107022	Gramd3	chr18	+	NM_0262	P6020	protein_cc	9030613F	GRAM dor	Gramd3
9703	Aldh7a1	0.426	0.824	0.351	0.001	0.122	0.026	11.635	10.403	10.124	110695	Aldh7a1	chr18	-	NM_0011	P18888	protein_cc	Atq1| D18	aldehyde	Aldh7a1
9704	Phax	1.587	0.757	1.202	0.000	0.010	0.246	8.284	8.951	8.549	56698	Phax	chr18	+	NM_0011	P22784	protein_cc	2810055C	phosphory	Phax
9705	Tex43	1.000	1.000	1.000	1.000	1.000	1.000	0.000	0.000	0.000	67343	Tex43	chr18	+	NM_0260	P2914	protein_cc	1700065I1	testis expr	Tex43
9706	Lmnb1	0.984	0.567	0.558	0.960	0.151	0.298	6.705	6.682	5.864	16906	Lmnb1	chr18	+	NM_0107	P14500	protein_cc	.	lamin B1	Lmnb1
9707	March3	3.842	0.260	0.999	0.306	1.000	0.423	0.001	1.943	0.000	320253	March3	chr18	-	NM_1771	P21589	protein_cc	6330411I1	membran	March3
9708	Gm10536	0.382	0.982	0.375	0.182	1.000	0.423	1.416	0.027	0.000	10003841	Gm10536	chr18	+	NR_03345	.	lncRNA	ENSMUSG	predicted	Gm10536
9709	C330018D	1.649	0.479	0.791	0.001	0.005	0.519	6.717	7.439	6.379	77422	C330018D	chr18	+	NM_0299	P11801	protein_cc	AU018666	RIKEN cDN	C330018D
9710	Megf10	0.735	1.798	1.321	0.191	0.000	0.366	0.466	0.021	0.867	70417	Megf10	chr18	+	NM_0010	P6552	protein_cc	3000002B	multiple E	Megf10
9711	Prrc1	1.980	0.826	1.637	0.006	0.305	0.256	7.881	8.867	8.592	73137	Prrc1	chr18	+	NM_0284	P1793	protein_cc	1190002C	proline-ric	Prrc1
9712	Ctxn3	1.000	1.000	1.000	1.000	1.000	1.000	0.000	0.000	0.000	629147	Ctxn3	chr18	+	NM_0011	P6190	protein_cc	ENSMUSG	cortexin 3	Ctxn3
9713	1700066O	1.000	1.000	1.000	1.000	1.000	1.000	0.000	0.000	0.000	74916	1700066O	chr18	-	NR_01554	.	lncRNA	4930500F	RIKEN cDN	1700066O
9714	1700011I0	1.000	1.000	1.000	1.000	1.000	1.000	0.000	0.000	0.000	75444	1700011I0	chr18	+	NM_0292	P6128	protein_cc	4930583I0	RIKEN cDN	1700011I0
9715	4930511M	1.000	1.000	1.000	1.000	1.000	1.000	0.000	0.000	0.000	75084	4930511M	chr18	+	NR_02794	.	lncRNA	.	RIKEN cDN	4930511M
9716	2210409D	1.000	1.000	1.000	1.000	1.000	1.000	0.000	0.000	0.000	70157	2210409D	chr18	-	NR_04536	.	lncRNA	.	RIKEN cDN	2210409D
9717	Slc12a2	0.787	1.031	0.811	0.239	0.895	0.278	6.553	6.207	6.251	20496	Slc12a2	chr18	+	NM_0091	P7355	protein_cc	9330166H	solute car	Slc12a2
9718	Fbn2	0.236	5.107	1.205	0.000	0.122	0.826	2.170	0.086	2.439	14119	Fbn2	chr18	-	NM_0101	P10192	protein_cc	BC063774	fibrillin 2	Fbn2
9719	Slc27a6	1.010	1.885	1.904	0.000	0.182	0.423	0.000	0.015	0.929	225579	Slc27a6	chr18	+	NM_0010	P1023	protein_cc	4930583I0	solute car	Slc27a6
9720	Isoc1	0.367	0.942	0.346	0.000	0.350	0.000	9.735	8.289	8.203	66307	Isoc1	chr18	+	NM_0254	P5594	protein_cc	2610034N	isochorism	Isoc1
9721	Adamts19	1.000	1.000	1.000	1.000	1.000	1.000	0.000	0.000	0.000	240322	Adamts19	chr18	+	NM_1755	P19800	protein_cc	4831407I2	a disinteg	Adamts19
9722	A730017C	1.000	1.000	1.000	1.000	1.000	1.000	0.000	0.000	0.000	225583	A730017C	chr18	+	NR_03376	.	protein_cc	.	RIKEN cDN	A730017C
9723	Chsy3	1.397	1.441	2.013	0.258	0.301	0.357	4.996	5.478	6.005	78923	Chsy3	chr18	+	NM_0010	P1047	protein_cc	4833446K	chondroiti	Chsy3
9724	Gm4951	2.726	0.360	0.983	0.000	0.001	0.962	10.450	11.897	10.424	240327	Gm4951	chr18	+	NM_0010	P7761	protein_cc	EG240327	predicted	Gm4951

9725	Gm4841	235.703	0.064	14.986	0.000	0.000	0.392	4.291	12.172	8.197	225594	Gm4841	chr18	-	NM_0010	P22508	protein_cc	EG225594	predicted	Gm4841
9726	F830016B	9.204	0.058	0.538	0.000	0.000	0.298	7.472	10.674	6.577	240328	F830016B	chr18	+	NM_0011	P24153	protein_cc	Ifgga4	RIKEN cDN	F830016B
9727	Iigp1	9.185	0.375	3.442	0.000	0.006	0.279	12.822	16.021	14.605	60440	Iigp1	chr18	+	NM_0011	P18575	protein_cc	2900074L	interferon	Iigp1
9728	Smim3	11.074	0.848	9.388	0.008	0.486	0.028	3.365	6.834	6.596	106878	Smim3	chr18	-	NM_1341	P24826	protein_cc	2010002N	small integ	Smim3
9729	Dctn4	1.189	0.860	1.022	0.497	0.450	0.955	7.384	7.634	7.416	67665	Dctn4	chr18	+	NM_0263	P19997	protein_cc	1110001K	dynactin 4	Dctn4
9730	Rbm22	1.521	1.091	1.659	0.003	0.610	0.232	6.888	7.493	7.618	66810	Rbm22	chr18	+	NM_0257	P3302	protein_cc	8430430L	RNA bindi	Rbm22
9731	Myoz3	0.716	0.995	0.712	0.182	1.000	0.423	0.490	0.008	0.000	170947	Myoz3	chr18	-	NM_1333	P14058	protein_cc	4833419K	myozenin	Myoz3
9732	Synpo	18.986	0.990	18.789	0.000	0.637	0.000	4.754	9.001	8.986	104027	Synpo	chr18	-	NM_1773	P22133	protein_cc	9030217H	synaptopo	Synpo
9733	G630071F	2.134	0.469	1.000	0.305	1.000	0.423	0.000	1.094	0.000	328963	G630071F	chr18	+	NR_04540	.	lncRNA	G630071F	RIKEN cDN	G630071F
9734	Ndst1	0.477	0.969	0.462	0.003	0.799	0.047	10.840	9.772	9.727	15531	Ndst1	chr18	-	NM_0083	P9764	protein_cc	1200015G	N-deacety	Ndst1
9735	Rps14	1.135	1.452	1.648	0.763	0.250	0.212	12.687	12.869	13.407	20044	Rps14	chr18	+	NM_0206	P18699	protein_cc	2600014J	ribosomal	Rps14
9736	Cd74	1.369	0.742	1.016	0.173	0.220	0.966	7.932	8.384	7.954	16149	Cd74	chr18	+	NM_0010	P4109	protein_cc	CLIP| DHL	CD74 anti	Cd74
9737	Tcof1	2.140	0.547	1.169	0.000	0.000	0.329	7.456	8.554	7.682	21453	Tcof1	chr18	-	NM_0115	P11291	protein_cc	AA408847	treacle rib	Tcof1
9738	Arsi	1.000	1.000	1.000	1.000	1.000	1.000	0.000	0.000	0.000	545260	Arsi	chr18	+	NM_0010	P22415	protein_cc	BC020108	arylsulfata	Arsi
9739	Camk2a	0.756	1.866	1.410	0.577	0.354	0.742	1.105	0.701	1.601	12322	Camk2a	chr18	+	NM_1774	P5581	protein_cc	CaMKII| m	calcium/ca	Camk2a
9740	Slc6a7	0.552	0.990	0.547	0.182	1.000	0.423	0.871	0.015	0.000	240332	Slc6a7	chr18	-	NM_2013	P25255	protein_cc	AW45593	solute car	Slc6a7
9741	Cdx1	1.000	1.000	1.000	1.000	1.000	1.000	0.000	0.000	0.000	12590	Cdx1	chr18	-	NM_0098	P24505	protein_cc	Cdx| Cdx-	caudal typ	Cdx1
9742	Pdgfrb	0.205	1.963	0.403	0.000	0.147	0.103	5.717	3.433	4.407	18596	Pdgfrb	chr18	+	NM_0088	P357	protein_cc	AI528809|	platelet de	Pdgfrb
9743	Csf1r	0.202	1.686	0.341	0.000	0.005	0.011	9.384	7.079	7.833	12978	Csf1r	chr18	+	NM_0010	P5510	protein_cc	AI323359|	colony stir	Csf1r
9744	Hmgxb3	1.195	0.933	1.115	0.485	0.742	0.660	6.491	6.748	6.648	106894	Hmgxb3	chr18	-	NM_1782	P26670	protein_cc	2510002C	HMG box	Hmgxb3
9745	Slc26a2	2.919	0.590	1.723	0.007	0.070	0.344	4.503	6.048	5.288	13521	Slc26a2	chr18	-	NM_0078	P17956	protein_cc	Dtd| ST-O	solute car	Slc26a2
9746	Pde6a	0.451	0.986	0.444	0.182	1.000	0.423	1.170	0.021	0.000	225600	Pde6a	chr18	+	NM_1460	P13835	protein_cc	nmf282| F	phosphod	Pde6a
9747	Ppargc1b	0.062	1.200	0.074	0.000	0.116	0.001	8.422	4.400	4.663	170826	Ppargc1b	chr18	-	NM_1332	P13757	protein_cc	4631412G	peroxisom	Ppargc1b
9748	A530050N	1.000	1.000	1.000	1.000	1.000	1.000	0.000	0.000	0.000	442846	A530050N	chr18	-	NR_04541	.	lncRNA	.	RIKEN cDN	A530050N
9749	Arhgef37	0.179	0.600	0.107	0.031	1.000	0.157	3.218	0.736	0.000	328967	Arhgef37	chr18	-	NM_1778	P14428	protein_cc	4933429F	Rho guani	Arhgef37
9750	Csnk1a1	1.620	0.728	1.179	0.010	0.032	0.337	9.555	10.251	9.792	93687	Csnk1a1	chr18	+	NM_1460	P3986	protein_cc	2610208K	casein kin	Csnk1a1
9751	Bvht	0.605	2.427	1.469	0.072	0.001	0.215	3.784	3.059	4.338	545261	Bvht	chr18	-	NR_04542	.	lncRNA	Bravehear	bravehear	Bvht
9752	Il17b	1.000	1.000	1.000	1.000	1.000	1.000	0.000	0.000	0.000	56069	Il17b	chr18	+	NM_0195	P9594	protein_cc	1110006O	interleukin	Il17b
9753	Pcyox1l	0.632	1.510	0.954	0.015	0.409	0.942	4.779	4.116	4.711	240334	Pcyox1l	chr18	-	NM_1728	P1540	protein_cc	BC060677	prenylcyst	Pcyox1l
9754	Grpel2	0.349	1.650	0.576	0.000	0.027	0.023	8.354	6.836	7.558	17714	Grpel2	chr18	-	NM_0212	P15517	protein_cc	mt-GrpE#	GrpE-like	Grpel2
9755	1500015A	2.967	1.609	4.775	0.259	0.290	0.045	1.465	3.034	3.721	68982	1500015A	chr18	+	NR_02943	.	lncRNA	.	RIKEN cDN	1500015A
9756	Afap1l1	1.985	1.253	2.487	0.000	0.346	0.193	3.887	4.876	5.201	106877	Afap1l1	chr18	-	NM_1789	P14558	protein_cc	AI173486	actin filam	Afap1l1
9757	Ablim3	1.008	0.686	0.692	0.964	0.003	0.402	7.417	7.429	6.885	319713	Ablim3	chr18	-	NM_1986	P15917	protein_cc	AW99071	actin bind	Ablim3
9758	Sh3tc2	0.519	1.171	0.608	0.219	0.829	0.522	2.454	1.509	1.736	225608	Sh3tc2	chr18	+	NM_1726	P24819	protein_cc	D430044G	SH3 doma	Sh3tc2
9759	Adrb2	11.143	0.968	10.782	0.000	0.878	0.104	2.308	5.786	5.739	11555	Adrb2	chr18	-	NM_0074	P22296	protein_cc	Adrb-2| B	adrenergi	Adrb2
9760	Htr4	1.000	1.000	1.000	1.000	1.000	1.000	0.000	0.000	0.000	15562	Htr4	chr18	+	NM_0083	P26248	protein_cc	5-HT-4| 5-	5 hydroxy	Htr4
9761	Fbxo38	1.463	0.827	1.211	0.062	0.394	0.612	7.166	7.715	7.442	107035	Fbxo38	chr18	-	NM_1341	P8182	protein_cc	6030410I2	F-box prot	Fbxo38
9762	Spink10	1.617	1.449	2.343	0.107	0.237	0.250	4.126	4.819	5.354	328971	Spink10	chr18	+	NM_1778	P23403	protein_cc	A230091H	serine pep	Spink10
9763	Spink7	1.000	1.000	1.000	1.000	1.000	1.000	0.000	0.000	0.000	408198	Spink7	chr18	-	NM_0010	P20189	protein_cc	Ecg2| EG4	serine pep	Spink7
9764	Spink13	1.000	1.000	1.000	1.000	1.000	1.000	0.000	0.000	0.000	10003841	Spink13	chr18	-	NM_0011	P18610	protein_cc	ENSMUSG	serine pep	Spink13
9765	2700046A	1.000	1.000	1.000	1.000	1.000	1.000	0.000	0.000	0.000	78449	2700046A	chr18	-	NR_03769	.	lncRNA	A330042I	RIKEN cDN	2700046A
9766	Apcdd1	1.000	1.000	1.000	1.000	1.000	1.000	0.000	0.000	0.000	494504	Apcdd1	chr18	+	NM_1332	P20495	protein_cc	AB023957	adenomat	Apcdd1
9767	Napg	0.973	0.868	0.844	0.755	0.288	0.381	7.542	7.502	7.298	108123	Napg	chr18	+	NM_0280	P12816	protein_cc	2400003O	N-ethylma	Napg
9768	Piezo2	0.168	0.598	0.100	0.008	0.536	0.058	4.375	1.798	1.056	667742	Piezo2	chr18	-	NM_0010	P15490	protein_cc	5930434P	piezo-type	Piezo2
9769	Txnl1	1.700	0.953	1.621	0.018	0.757	0.170	9.835	10.601	10.532	53382	Txnl1	chr18	-	NM_0167	P12732	protein_cc	32kDa| TF	thioredoxi	Txnl1
9770	Wdr7	0.817	1.180	0.964	0.134	0.242	0.670	6.768	6.476	6.715	104082	Wdr7	chr18	+	NM_0010	P23451	protein_cc	AI462727|	WD repea	Wdr7
9771	St8sia3os	1.000	1.000	1.000	1.000	1.000	1.000	0.000	0.000	0.000	73588	St8sia3os	chr18	-	NR_04536	.	lncRNA	1700091E	ST8 alpha-	St8sia3os
9772	St8sia3	1.755	1.342	2.355	0.009	0.008	0.052	2.431	3.242	3.666	20451	St8sia3	chr18	+	NM_0091	P11121	protein_cc	AI847333|	ST8 alpha-	St8sia3
9773	Onecut2	0.318	0.955	0.304	0.000	0.871	0.048	11.951	10.299	10.232	225631	Onecut2	chr18	+	NM_1942	P17692	protein_cc	C730009D	one cut do	Onecut2
9774	Fech	0.282	1.432	0.404	0.000	0.043	0.022	10.192	8.367	8.885	14151	Fech	chr18	-	NM_0079	P6353	protein_cc	AI894116|	ferrochela	Fech
9775	Nars	2.870	0.706	2.026	0.002	0.025	0.003	9.798	11.319	10.817	70223	Nars	chr18	-	NM_0273	P2976	protein_cc	3010001M	asparagin	Nars
9776	Atp8b1	0.710	0.992	0.704	0.001	0.956	0.192	8.956	8.462	8.450	54670	Atp8b1	chr18	-	NM_0010	P10760	protein_cc	AI451886|	ATPase, cl	Atp8b1
9777	Nedd4l	0.990	0.589	0.583	0.892	0.018	0.177	8.865	8.850	8.087	83814	Nedd4l	chr18	+	NM_0011	P549	protein_cc	1300012C	neural pre	Nedd4l
9778	Alpk2	1.572	0.636	1.000	0.304	1.000	0.423	0.000	0.653	0.000	225638	Alpk2	chr18	-	NM_0010	P5071	protein_cc	Gm549| H	alpha-kina	Alpk2
9779	Malt1	2.079	0.510	1.060	0.021	0.023	0.695	4.295	5.351	4.379	240354	Malt1	chr18	+	NM_1728	P750	protein_cc	A630046N	MALT1 pa	Malt1
9780	Zfp532	0.974	0.535	0.521	0.888	0.030	0.262	4.505	4.468	3.564	328977	Zfp532	chr18	+	NM_2072	P15387	protein_cc	C530030I1	zinc finger	Zfp532
9781	Oacyl	2.134	0.469	1.000	0.305	1.000	0.423	0.000	1.094	0.000	319888	Oacyl	chr18	+	NM_1770	P9431	protein_cc	5330437I	O-acyltran	Oacyl
9782	Sec11c	1.269	1.259	1.597	0.060	0.065	0.072	8.549	8.893	9.225	66286	Sec11c	chr18	+	NM_0254	P12588	protein_cc	1810029G	SEC11 hor	Sec11c
9783	Grp	1.000	1.000	1.000	1.000	1.000	1.000	0.000	0.000	0.000	225642	Grp	chr18	+	NM_1750	P1652	protein_cc	BLP	gastrin rel	Grp
9784	Rax	1.000	1.000	1.000	1.000	1.000	1.000	0.000	0.000	0.000	19434	Rax	chr18	-	NM_0138	P6355	protein_cc	7530406A	retina and	Rax
9785	Cplx4	1.000	1.000	1.000	1.000	1.000	1.000	0.000	0.000	0.000	225644	Cplx4	chr18	-	NM_1454	P14892	protein_cc	A930004D	complexin	Cplx4
9786	Lman1	0.580	0.825	0.479	0.000	0.006	0.003	12.008	11.223	10.945	70361	Lman1	chr18	-	NM_0274	P19206	protein_cc	2610020P	lectin, ma	Lman1
9787	Ccbe1	0.594	1.116	0.662	0.070	0.746	0.425	4.922	4.170	4.328	320924	Ccbe1	chr18	-	NM_1787	P6308	protein_cc	4933426F	collagen a	Ccbe1
9788	Pmaip1	14.792	0.363	5.369	0.000	0.004	0.230	2.497	6.384	4.922	58801	Pmaip1	chr18	+	NM_0214	P5740	protein_cc	Noxa	phorbol-1	Pmaip1
9789	Gm9926	0.716	0.995	0.712	0.182	1.000	0.423	0.490	0.008	0.000	10050356	Gm9926	chr18	+	NR_04052	.	lncRNA	ENSMUSG	predicted	Gm9926
9790	Mc4r	1.000	1.000	1.000	1.000	1.000	1.000	0.000	0.000	0.000	17202	Mc4r	chr18	-	NM_0169	P6845	protein_cc	Mc4-r	melanoco	Mc4r
9791	Gm19784	1.000	1.000	1.000	1.000	1.000	1.000	0.000	0.000	0.000	10050358	Gm19784	chr18	-	NR_04046	.	lncRNA	.	predicted	Gm19784
9792	Gnal	1.916	0.921	1.765	0.001	0.037	0.061	6.933	7.870	7.752	14680	Gnal	chr18	+	NM_1771	P8845	protein_cc	2610011C	guanine n	Gnal
9793	Chmp1b	1.392	0.808	1.124	0.000	0.015	0.369	8.302	8.779	8.471	67064	Chmp1b	chr18	+	NM_0241	P20980	protein_cc	2810405I1	charged m	Chmp1b
9794	Mppe1	4.148	1.019	4.226	0.010	0.916	0.007	3.785	5.838	5.865	225651	Mppe1	chr18	-	NM_1726	P13581	protein_cc	A530095G	metalloph	Mppe1
9795	Impa2	0.074	1.719	0.127	0.003	0.469	0.065	5.307	1.552	2.333	114663	Impa2	chr18	+	NM_0532	P9391	protein_cc	2210415D	inositol (m	Impa2
9796	B430212C	0.735	1.798	1.321	0.191	0.000	0.366	0.466	0.021	0.867	338360	B430212C	chr18	-	NR_03321	.	lncRNA	.	RIKEN cDN	B430212C
9797	Cidea	0.708	1.381	0.977	0.548	0.607	0.980	2.712	2.214	2.679	12683	Cidea	chr18	+	NM_0077	P19882	protein_cc	AW21274	cell death-	Cidea
9798	Tubb6	19.587	0.431	8.443	0.000	0.004	0.135	5.823	10.115	8.901	67951	Tubb6	chr18	+	NM_0264	P12080	protein_cc	2310057H	tubulin, be	Tubb6
9799	Afg3l2	0.816	1.050	0.857	0.190	0.674	0.364	8.764	8.471	8.541	69597	Afg3l2	chr18	-	NM_0271	P24930	protein_cc	2310036I	AFG3-like	Afg3l2
9800	Slmo1	1.000	1.000	1.000	1.000	1.000	1.000	0.000	0.000	0.000	225655	Slmo1	chr18	+	NM_1448	P15103	protein_cc	A230074A	slowmo ho	Slmo1
9801	Spire1	3.697	0.486	1.796	0.172	0.328	0.463	2.207	4.094	3.052	68166	Spire1	chr18	-	NM_1943	P17295	protein_cc	6030430B	spire hom	Spire1
9802	Cep76	2.460	0.848	2.086	0.003	0.110	0.060	4.801	6.100	5.862	225659	Cep76	chr18	-	NM_0010	P12802	protein_cc	6230425F	centrosom	Cep76
9803	Psmg2	1.009	1.300	1.312	0.948	0.092	0.160	7.493	7.506	7.885	107047	Psmg2	chr18	+	NM_1341	P16201	protein_cc	1700017I1	proteason	Psmg2
9804	Ptpn2	5.268	0.624	3.285	0.000	0.082	0.237	7.081	9.479	8.797	19255	Ptpn2	chr18	-	NM_0089	P5618	protein_cc	AI325124|	protein ty	Ptpn2
9805	Seh1l	2.161	0.730	1.577	0.001	0.013	0.009	7.788	8.899	8.445	72124	Seh1l	chr18	+	NM_0010	P14253	protein_cc	2610007A	SEH1-like	Seh1l
9806	4930549G	3.733	0.221	0.823	0.368	0.340	0.740	0.749	2.649	0.468	75286	4930549G	chr18	-	NR_04537	.	lncRNA	.	RIKEN cDN	4930549G
9807	Cep192	2.203	0.624	1.376	0.084	0.177	0.560	4.147	5.287	4.608	70799	Cep192	chr18	+	NM_0275	P1149	protein_cc	4631422C	centrosom	Cep192
9808	Ldlrad4	0.119	2.798	0.334	0.000	0.099	0.073	4.897	1.830	3.315	52662	Ldlrad4	chr18	+	NM_1726	P3780	protein_cc	8230401C	low densit	Ldlrad4
9809	Fam210a	0.202	1.427	0.288	0.000	0.007	0.002	9.264	6.957	7.469	108654	Fam210a	chr18	-	NM_1537	P3031	protein_cc	4933403F	family wit	Fam210a
9810	Rnmt	2.143	0.939	2.012	0.002	0.753	0.237	6.954	8.054	7.963	67897	Rnmt	chr18	+	NM_0264	P15627	protein_cc	2610002P	RNA (guar	Rnmt
9811	Mc5r	1.000	1.000	1.000	1.000	1.000	1.000	0.000	0.000	0.000	17203	Mc5r	chr18	+	NM_0135	P13486	protein_cc	.	melanoco	Mc5r
9812	Mc2r	1.000	1.000	1.000	1.000	1.000	1.000	0.000	0.000	0.000	17200	Mc2r	chr18	-	NM_0085	P22834	protein_cc	ACTH-R| A	melanoco	Mc2r
9813	4930546C	1.000	1.000	1.000	1.000	1.000	1.000	0.000	0.000	0.000	78931	4930546C	chr18	+	NR_03805	.	lncRNA	.	RIKEN cDN	4930546C
9814	Tcf4	1.280	1.179	1.510	0.050	0.031	0.085	8.930	9.286	9.524	21413	Tcf4	chr18	+	NM_0136	P23735	protein_cc	5730422P	transcripti	Tcf4
9815	Ccdc68	0.301	1.412	0.425	0.021	0.624	0.160	4.672	2.940	3.438	381175	Ccdc68	chr18	+	NM_2013	P3313	protein_cc	BC046343	coiled-coil	Ccdc68

9816	Rab27b	0.710	0.636	0.452	0.234	0.008	0.237	3.182	2.689	2.037	80718	Rab27b	chr18	-	NM_0305	P3706	protein_cc	2310021G	RAB27B, r	Rab27b
9817	Dynap	1.000	1.000	1.000	1.000	1.000	1.000	0.000	0.000	0.000	75577	Dynap	chr18	-	NM_0293	P16732	protein_cc	2310002L:	dynactin a	Dynap
9818	4930503L1	1.837	0.764	1.403	0.143	0.489	0.552	3.551	4.429	4.040	269033	4930503L:	chr18	-	NM_1729	P24886	protein_cc	9930107F:	RIKEN cDN	4930503L1
9819	Stard6	7.294	0.450	3.285	0.018	0.095	0.258	0.356	3.223	2.072	170461	Stard6	chr18	+	NM_0290	P19762	protein_cc	1700011K	StAR-relat	Stard6
9820	Poli	1.003	0.466	0.468	0.989	0.024	0.149	5.829	5.833	4.732	26447	Poli	chr18	-	NM_0012	P25261	protein_cc	Rad30b	polymeras	Poli
9821	Mbd2	1.929	0.883	1.704	0.020	0.497	0.208	7.844	8.792	8.613	17191	Mbd2	chr18	+	NM_0107	P20409	protein_cc	MBD2a	methyl-Cp	Mbd2
9822	Dcc	0.708	0.459	0.325	0.490	1.000	0.192	1.622	1.123	0.000	13176	Dcc	chr18	-	NM_0078	P4690	protein_cc	C030036D	deleted in	Dcc
9823	Mex3c	1.916	0.753	1.442	0.036	0.209	0.155	7.073	8.011	7.601	240396	Mex3c	chr18	+	NM_0010	P9616	protein_cc	A130001D	mex3 RNA	Mex3c
9824	Smad4	1.307	0.695	0.908	0.073	0.080	0.674	8.235	8.621	8.096	17128	Smad4	chr18	-	NM_0085	P3614	protein_cc	AW74385	SMAD fam	Smad4
9825	Elac1	0.625	1.284	0.802	0.121	0.247	0.489	5.406	4.727	5.087	114615	Elac1	chr18	-	NM_0532	P5045	protein_cc	26100180	elaC ribon	Elac1
9826	Me2	1.540	1.224	1.885	0.000	0.305	0.208	3.087	3.710	4.001	107029	Me2	chr18	-	NM_1454	P738	protein_cc	AW12056	malic enzy	Me2
9827	Mro	1.000	1.000	1.000	1.000	1.000	1.000	0.000	0.000	0.000	71263	Mro	chr18	+	NM_0013	P12433	protein_cc	4930507C	maestro	Mro
9828	Mapk4	0.325	3.573	1.163	0.010	0.126	0.830	2.807	1.188	3.025	225724	Mapk4	chr18	-	NM_1726	P25556	protein_cc	A330097D	mitogen-a	Mapk4
9829	D730045A	0.716	0.995	0.712	0.182	1.000	0.423	0.490	0.008	0.000	77855	D730045A	chr18	+	NR_04539	.	lncRNA	.	RIKEN cDN	D730045A
9830	Ska1	1.686	1.712	2.887	0.010	0.269	0.347	0.408	1.162	1.938	66468	Ska1	chr18	-	NM_0011	P9988	protein_cc	2810433K	spindle an	Ska1
9831	Cxxc1	2.064	0.825	1.702	0.001	0.033	0.002	7.673	8.719	8.440	74322	Cxxc1	chr18	+	NM_0288	P11489	protein_cc	2410002I1	CXXC finge	Cxxc1
9832	Mbd1	6.529	0.409	2.670	0.000	0.000	0.001	7.335	10.042	8.752	17190	Mbd1	chr18	+	NM_0135	P24135	protein_cc	PCM1	methyl-Cp	Mbd1
9833	Cfap53	1.581	0.879	1.390	0.301	0.744	0.423	0.000	0.661	0.476	74453	Cfap53	chr18	+	NR_02942	.	protein_cc	4933415I(	cilia and fl	Cfap53
9834	Myo5b	0.629	1.505	0.946	0.000	0.065	0.816	7.178	6.509	7.098	17919	Myo5b	chr18	+	NM_2016	P7311	protein_cc	AI661750	myosin VE	Myo5b
9835	1700120E	4.488	0.223	0.999	0.021	1.000	0.423	0.001	2.167	0.000	73606	1700120E	chr18	-	NR_04536	.	lncRNA	.	RIKEN cDN	1700120E1
9836	Scarna17	1.092	0.709	0.774	0.436	0.295	0.633	5.563	5.690	5.194	10021746	Scarna17	chr18	-	NR_02856	.	guide_RN/	MBII-119	small Caja	Scarna17
9837	Acaa2	0.304	1.515	0.461	0.000	0.020	0.031	13.080	11.363	11.963	52538	Acaa2	chr18	+	NM_1774	P12915	protein_cc	0610011L(	acetyl-Coe	Acaa2
9838	Lipg	0.892	0.347	0.310	0.549	0.008	0.093	6.018	5.853	4.326	16891	Lipg	chr18	-	NM_0107	P17778	protein_cc	3110013K	lipase, end	Lipg
9839	9030625G	2.124	1.237	2.628	0.044	0.436	0.175	0.403	1.490	1.797	71545	9030625G	chr18	-	NR_11044	.	lncRNA	.	RIKEN cDN	9030625G
9840	Rpl17	1.038	1.564	1.624	0.584	0.022	0.150	9.320	9.374	10.019	319195	Rpl17	chr18	+	NM_0010	P10202	protein_cc	.	ribosomal	Rpl17
9841	Snord58b	2.783	0.934	2.599	0.019	0.874	0.423	0.001	1.478	1.379	10021745	Snord58b	chr18	+	NR_02855	.	snoRNA	MBII-199	small nucl	Snord58b
9842	BC031181	0.955	0.974	0.930	0.130	0.241	0.137	9.872	9.805	9.767	407819	BC031181	chr18	+	NM_0010	P7800	protein_cc	.	cDNA seq	BC031181
9843	Dym	0.373	1.179	0.440	0.001	0.482	0.024	7.596	6.173	6.410	69190	Dym	chr18	+	NM_0277	P24731	protein_cc	1810041N	dymeclin	Dym
9844	2010010A	1.005	1.439	1.446	0.000	0.182	0.423	0.000	0.008	0.532	10050311	2010010A	chr18	-	NR_04539	.	lncRNA	5033420E:	RIKEN cDN	2010010A
9845	Smad7	0.317	1.982	0.628	0.009	0.179	0.440	6.172	4.515	5.501	17131	Smad7	chr18	+	NM_0010	P5151	protein_cc	Madh7	SMAD fam	Smad7
9846	Ctif	0.449	1.392	0.625	0.006	0.160	0.018	7.451	6.296	6.773	269037	Ctif	chr18	-	NM_2013	P23744	protein_cc	Gm672	CBP80/20	Ctif
9847	Gm10532	0.552	0.990	0.547	0.182	1.000	0.423	0.871	0.015	0.000	10003835	Gm10532	chr18	+	NR_04587	.	lncRNA	ENSMUSG	predicted	Gm10532
9848	Zbtb7c	19.727	1.374	27.111	0.000	0.164	0.063	2.030	6.332	6.791	207259	Zbtb7c	chr18	+	NM_1453	P22514	protein_cc	B230208J	zinc finger	Zbtb7c
9849	2900057B	1.000	1.000	1.000	1.000	1.000	1.000	0.000	0.000	0.000	73009	2900057B	chr18	-	NR_04536	.	lncRNA	.	RIKEN cDN	2900057B
9850	Smad2	1.178	1.209	1.424	0.071	0.248	0.293	6.959	7.195	7.469	17126	Smad2	chr18	+	NM_0107	P3949	protein_cc	7120426N	SMAD fam	Smad2
9851	Skor2	1.000	1.000	1.000	1.000	1.000	1.000	0.000	0.000	0.000	664805	Skor2	chr18	+	NM_0011	P11016	protein_cc	Corl2 | EG	SKI family	Skor2
9852	Ier3ip1	1.318	1.085	1.430	0.005	0.342	0.096	9.659	10.057	10.175	66191	Ier3ip1	chr18	+	NM_0254	P127	protein_cc	1110057H	immediate	Ier3ip1
9853	Hdhd2	0.692	1.198	0.829	0.086	0.038	0.534	7.714	7.183	7.444	76987	Hdhd2	chr18	+	NM_0010	P25381	protein_cc	0610039H	haloacid d	Hdhd2
9854	Katnal2	1.000	1.000	1.000	1.000	1.000	1.000	0.000	0.000	0.000	71206	Katnal2	chr18	-	NM_0277	P12621	protein_cc	3110023G	katanin p6	Katnal2
9855	Pias2	0.929	1.045	0.971	0.203	0.675	0.865	8.299	8.192	8.256	17344	Pias2	chr18	+	NM_0011	P5533	protein_cc	6330408K	protein in	Pias2
9856	St8sia5	1.000	1.000	1.000	1.000	1.000	1.000	0.000	0.000	0.000	225742	St8sia5	chr18	+	NM_0136	P23445	protein_cc	Siat8e | ST	ST8 alpha-	St8sia5
9857	Loxhd1	1.000	1.000	1.000	1.000	1.000	1.000	0.000	0.000	0.000	240411	Loxhd1	chr18	+	NM_1728	P12721	protein_cc	1700096C	lipoxygena	Loxhd1
9858	Rnf165	0.476	1.017	0.484	0.185	0.978	0.307	2.580	1.510	1.534	225743	Rnf165	chr18	-	NM_0011	P23965	protein_cc	2900024N	ring finger	Rnf165
9859	8030462N	4.468	0.476	2.128	0.084	0.198	0.021	2.802	4.961	3.891	212163	8030462N	chr18	-	NM_1786	P18666	protein_cc	Akd2 | Ark	RIKEN cDN	8030462N
9860	4930465K	1.000	1.000	1.000	1.000	1.000	1.000	0.000	0.000	0.000	67640	4930465K	chr18	+	NR_02797	.	lncRNA	.	RIKEN cDN	4930465K
9861	Haus1	0.489	2.212	1.082	0.051	0.000	0.812	5.050	4.018	5.163	225745	Haus1	chr18	-	NM_1460	P21858	protein_cc	BC024400	HAUS aug	Haus1
9862	Atp5a1	0.576	1.256	0.723	0.000	0.041	0.083	12.783	11.986	12.315	11946	Atp5a1	chr18	+	NM_0075	P9710	protein_cc	AI035633	ATP synth	Atp5a1
9863	Pstpip2	0.799	1.375	1.098	0.244	0.040	0.636	5.499	5.175	5.634	19201	Pstpip2	chr18	+	NM_0138	P1502	protein_cc	cmo | MA\	proline-se	Pstpip2
9864	Epg5	0.709	0.831	0.590	0.011	0.359	0.190	6.584	6.089	5.822	10050284	Epg5	chr18	+	NM_0011	P20756	protein_cc	4732475F	ectopic P-	Epg5
9865	Siglec15	1.000	1.000	1.000	1.000	1.000	1.000	0.000	0.000	0.000	620235	Siglec15	chr18	-	NM_0011	P3847	protein_cc	Cd33l3 | E	sialic acid	Siglec15
9866	Slc14a1	0.735	0.879	0.646	0.549	0.750	0.602	1.135	0.690	0.505	108052	Slc14a1	chr18	-	NM_0281	P6801	protein_cc	2610507K	solute car	Slc14a1
9867	Slc14a2	1.000	1.000	1.000	1.000	1.000	1.000	0.000	0.000	0.000	27411	Slc14a2	chr18	-	NM_2076	P12520	protein_cc	UT-A1 | U1	solute car	Slc14a2
9868	Setbp1	0.380	1.003	0.382	0.023	0.993	0.161	4.449	3.054	3.059	240427	Setbp1	chr18	-	NM_0530	P14107	protein_cc	C130092E	SET bindin	Setbp1
9869	Pard6g	0.646	0.883	0.570	0.376	0.751	0.451	4.593	3.962	3.783	93737	Pard6g	chr18	-	NM_0531	P11778	protein_cc	2419049N	par-6 fami	Pard6g
9870	Adnp2	2.708	0.655	1.774	0.046	0.265	0.381	3.060	4.497	3.887	240442	Adnp2	chr18	-	NM_1750	P3712	protein_cc	8430420L(	ADNP hon	Adnp2
9871	LOC10524	0.405	1.873	0.759	0.000	0.001	0.083	7.203	5.899	6.805	10524650	LOC10524	chr18	+	NR_13103	.	lncRNA	.	uncharact	LOC10524
9872	Rbfa	0.423	2.021	0.855	0.000	0.004	0.381	7.292	6.051	7.067	68731	Rbfa	chr18	-	NM_1991	P15142	protein_cc	1110032A	ribosome	Rbfa
9873	Txnl4a	1.031	1.047	1.079	0.480	0.432	0.403	8.910	8.953	9.020	27366	Txnl4a	chr18	+	NM_0252	P10151	protein_cc	D18Wsu98	thioredoxi	Txnl4a
9874	Hsbp1l1	0.122	3.838	0.468	0.000	0.015	0.049	5.093	2.056	3.996	66255	Hsbp1l1	chr18	-	NM_0011	P943	protein_cc	1810005K	heat shocl	Hsbp1l1
9875	Pqlc1	0.282	1.535	0.432	0.000	0.013	0.023	9.758	7.929	8.548	66943	Pqlc1	chr18	+	NM_0011	P8936	protein_cc	2310009N	PQ loop re	Pqlc1
9876	Kcng2	0.518	0.621	0.322	0.149	1.000	0.196	1.637	0.687	0.000	240444	Kcng2	chr18	-	NM_0011	P400	protein_cc	AA591524	potassium	Kcng2
9877	Ctdp1	1.902	0.844	1.604	0.005	0.313	0.210	5.361	6.288	6.042	67655	Ctdp1	chr18	-	NM_0262	P22968	protein_cc	4930563P	CTD (carb	Ctdp1
9878	Gm2176	1.000	1.000	1.000	1.000	1.000	1.000	0.000	0.000	0.000	10003934	Gm2176	chr18	-	NR_02842	.	misc_RNA	10003934	predicted	Gm2176
9879	Nfatc1	1.680	0.690	1.159	0.000	0.129	0.752	6.515	7.263	6.728	18018	Nfatc1	chr18	-	NM_1984	P18245	protein_cc	2210017P	nuclear fa	Nfatc1
9880	Atp9b	0.962	0.703	0.676	0.809	0.000	0.311	7.753	7.697	7.188	50771	Atp9b	chr18	-	NM_0012	P17828	protein_cc	AA934181	ATPase, cl	Atp9b
9881	Sall3	1.152	0.629	0.725	0.722	1.000	0.422	0.465	0.669	0.000	20689	Sall3	chr18	-	NM_1782	P441	protein_cc	B130022O	sal-like 3 (	Sall3
9882	Galr1	1.000	1.000	1.000	1.000	1.000	1.000	0.000	0.000	0.000	14427	Galr1	chr18	-	NM_0080	P1517	protein_cc	Galnr1	galanin re	Galr1
9883	Mbp	0.326	7.391	2.409	0.188	0.101	0.505	1.712	0.095	2.981	17196	Mbp	chr18	+	NM_0107	P13785	protein_cc	C76307 | g	myelin bas	Mbp
9884	Zfp236	2.807	0.574	1.612	0.004	0.002	0.273	5.744	7.233	6.433	329002	Zfp236	chr18	-	NM_1778	P12419	protein_cc	AI447957	zinc finger	Zfp236
9885	2210420H	1.000	1.000	1.000	1.000	1.000	1.000	0.000	0.000	0.000	76961	2210420H	chr18	-	NR_04538	.	lncRNA	.	RIKEN cDN	2210420H
9886	4930445N	1.000	1.000	1.000	1.000	1.000	1.000	0.000	0.000	0.000	73985	4930445N	chr18	+	NR_12572	.	lncRNA	3010027C	RIKEN cDN	4930445N
9887	Zfp516	1.109	0.901	0.999	0.617	0.519	0.997	5.307	5.457	5.306	329003	Zfp516	chr18	+	NM_1830	P3603	protein_cc	C330029B	zinc finger	Zfp516
9888	4930592I(	2.734	0.517	1.413	0.305	0.427	0.423	0.001	1.452	0.499	75853	4930592I(	chr18	-	NR_03330	.	lncRNA	.	RIKEN cDN	4930592I0
9889	4921531P	1.000	1.000	1.000	1.000	1.000	1.000	0.000	0.000	0.000	70960	4921531P	chr18	-	NR_04536	.	lncRNA	1700108N	RIKEN cDN	4921531P
9890	Tshz1	0.295	2.267	0.668	0.000	0.001	0.069	8.784	7.021	8.201	110796	Tshz1	chr18	-	NM_0010	P21234	protein_cc	5730407I(	teashirt zi	Tshz1
9891	Zadh2	0.219	1.826	0.399	0.000	0.030	0.007	9.524	7.330	8.199	225791	Zadh2	chr18	+	NM_1460	P5339	protein_cc	C530046K	zinc bindir	Zadh2
9892	Zfp407	2.323	0.528	1.226	0.026	0.004	0.766	5.444	6.660	5.738	240476	Zfp407	chr18	-	NM_0010	P10694	protein_cc	6430585N	zinc finger	Zfp407
9893	Cndp1	0.251	2.488	0.626	0.004	0.187	0.482	2.053	0.061	1.376	338403	Cndp1	chr18	-	NM_1774	P18068	protein_cc	AI746433	carnosine	Cndp1
9894	Cndp2	1.400	0.717	1.003	0.003	0.001	0.975	6.346	6.831	6.351	66054	Cndp2	chr18	-	NM_0012	P24818	protein_cc	0610010E	CNDP dipe	Cndp2
9895	Fam69c	1.010	1.825	1.844	0.000	0.182	0.423	0.000	0.015	0.883	240479	Fam69c	chr18	+	NM_1737	P785	protein_cc	B230399E	family wit	Fam69c
9896	Cyb5a	0.301	0.827	0.249	0.006	0.436	0.068	14.335	12.602	12.328	109672	Cyb5a	chr18	+	NM_0257	P7414	protein_cc	0610009N	cytochron	Cyb5a
9897	Fbxo15	0.426	0.985	0.420	0.023	1.000	0.157	1.253	0.021	0.000	50764	Fbxo15	chr18	+	NM_0157	P75	protein_cc	AU019763	F-box prot	Fbxo15
9898	Timm21	0.675	0.846	0.571	0.007	0.186	0.096	6.913	6.346	6.105	67105	Timm21	chr18	-	NM_0259	P3202	protein_cc	1700034H	tranlocase	Timm21
9899	Neto1	0.457	1.358	0.620	0.183	0.195	0.578	1.158	0.027	0.468	246317	Neto1	chr18	+	NM_1449	P25910	protein_cc	AI851453	neuropilin	Neto1
9900	Cbln2	1.000	1.000	1.000	1.000	1.000	1.000	0.000	0.000	0.000	12405	Cbln2	chr18	+	NM_0013	P17972	protein_cc	6330593N	cerebellin	Cbln2
9901	Socs6	2.282	0.894	2.040	0.006	0.644	0.278	7.127	8.317	8.155	54607	Socs6	chr18	-	NM_0188	P19604	protein_cc	1500012N	suppresso	Socs6
9902	Rttn	1.404	1.188	1.668	0.338	0.506	0.391	2.058	2.548	2.796	246102	Rttn	chr18	+	NM_1755	P14241	protein_cc	4921538A	rotatin	Rttn
9903	Cd226	1.368	0.350	0.479	0.449	0.010	0.518	1.540	1.992	0.477	225825	Cd226	chr18	+	NM_0010	P13846	protein_cc	BC051526	CD226 an	Cd226
9904	Dok6	1.000	1.000	1.000	1.000	1.000	1.000	0.000	0.000	0.000	623279	Dok6	chr18	-	NM_0010	P1558	protein_cc	Dok-6	docking pi	Dok6
9905	Tmx3	1.632	0.641	1.047	0.002	0.014	0.844	7.801	8.508	7.867	67988	Tmx3	chr18	+	NM_1982	P17359	protein_cc	6430411B	thioredoxi	Tmx3
9906	1700030N	1.000	1.000	1.000	1.000	1.000	1.000	0.000	0.000	0.000	70011	1700030N	chr19	-	NR_04530	.	lncRNA	1700087C	RIKEN cDN	1700030N

9907	Ighmbp2	1.447	0.493	0.713	0.257	0.009	0.655	5.208	5.741	4.720	20589	Ighmbp2	chr19	-	NM_0092	P22868	protein_cc	AEP| Catf:	immunogl	Ighmbp2
9908	Mrpl21	1.387	0.596	0.826	0.065	0.072	0.642	4.858	5.330	4.583	353242	Mrpl21	chr19	+	NM_1722	P10509	protein_cc	BC028768	mitochond	Mrpl21
9909	Cpt1a	0.325	1.971	0.640	0.000	0.001	0.052	10.631	9.007	9.986	12894	Cpt1a	chr19	+	NM_0134	P17255	protein_cc	C730027G	carnitine	Cpt1a
9910	Mtl5	1.503	2.999	4.509	0.333	0.041	0.188	1.819	2.407	3.992	17771	Mtl5	chr19	+	NM_0010	P16609	protein_cc	tesmin	metallothi	Mtl5
9911	Gal	1.000	1.000	1.000	1.000	1.000	1.000	0.000	0.000	0.000	14419	Gal	chr19	-	NM_0102	P26558	protein_cc	Galn	galanin	Gal
9912	Ppp6r3	1.054	1.041	1.097	0.442	0.622	0.592	9.921	9.997	10.055	52036	Ppp6r3	chr19	-	NM_0011	P16101	protein_cc	4930528G	protein ph	Ppp6r3
9913	Lrp5	0.387	1.076	0.416	0.001	0.702	0.015	6.946	5.575	5.681	16973	Lrp5	chr19	-	NM_0085	P15295	protein_cc	BMND1| I	low densit	Lrp5
9914	1810055G	1.677	1.050	1.761	0.003	0.715	0.134	7.916	8.662	8.732	72056	1810055G	chr19	+	NM_0280	P3469	protein_cc	AI256204	RIKEN cDN	1810055G
9915	Suv420h1	1.530	0.838	1.282	0.012	0.391	0.572	7.396	8.009	7.754	225888	Suv420h1	chr19	+	NM_0011	P19883	protein_cc	AA117471	suppresso	Suv420h1
9916	Chka	2.078	0.324	0.673	0.001	0.000	0.029	6.981	8.036	6.408	12660	Chka	chr19	+	NM_0012	P6045	protein_cc	Chetk-alpł	choline kir	Chka
9917	Tcirg1	2.547	0.583	1.484	0.000	0.000	0.007	7.136	8.484	7.706	27060	Tcirg1	chr19	-	NM_0011	P17061	protein_cc	ATP6a3| A	T cell, imn	Tcirg1
9918	Ndufs8	0.829	0.932	0.773	0.146	0.441	0.204	10.006	9.736	9.634	225887	Ndufs8	chr19	-	NM_1448	P23900	protein_cc	BC021616	NADH deh	Ndufs8
9919	Aldh3b1	1.725	1.387	2.392	0.096	0.522	0.440	4.027	4.813	5.285	67689	Aldh3b1	chr19	-	NM_0263	P9392	protein_cc	1700001N	aldehyde	Aldh3b1
9920	Unc93b1	1.544	0.772	1.192	0.021	0.072	0.110	8.251	8.878	8.505	54445	Unc93b1	chr19	+	NM_0194	P21672	protein_cc	Unc-93B1	unc-93 ho	Unc93b1
9921	Aldh3b3	1.000	1.000	1.000	1.000	1.000	1.000	0.000	0.000	0.000	73458	Aldh3b3	chr19	+	NM_0285	P8692	protein_cc	1700055N	aldehyde	Aldh3b3
9922	Aldh3b2	1.000	1.000	1.000	1.000	1.000	1.000	0.000	0.000	0.000	621603	Aldh3b2	chr19	+	NM_0011	P25515	protein_cc	AI848594|	aldehyde	Aldh3b2
9923	Acy3	0.085	1.733	0.147	0.000	0.003	0.001	10.433	6.872	7.665	71670	Acy3	chr19	+	NM_0013	P20228	protein_cc	0610006H	aspartoac	Acy3
9924	Tbx10	0.716	0.995	0.712	0.182	1.000	0.423	0.490	0.008	0.000	109575	Tbx10	chr19	+	NM_0115	P20828	protein_cc	Dc| Tbx13	T-box 10	Tbx10
9925	Nudt8	0.508	1.497	0.760	0.005	0.090	0.416	7.213	6.235	6.817	66387	Nudt8	chr19	+	NM_0255	P13346	protein_cc	2310039H	nudix (nuc	Nudt8
9926	Doc2g	0.489	0.943	0.461	0.051	0.567	0.226	2.207	1.174	1.089	60425	Doc2g	chr19	+	NM_0217	P1904	protein_cc	D830013C	double C2	Doc2g
9927	Ndufv1	0.731	1.244	0.910	0.000	0.000	0.146	10.739	10.288	10.603	17995	Ndufv1	chr19	-	NM_1336	P9459	protein_cc	CI-51kD	NADH deh	Ndufv1
9928	Gstp1	0.588	6.236	3.666	0.000	0.001	0.050	11.041	10.274	12.915	14870	Gstp1	chr19	-	NM_0135	P22118	protein_cc	GstpiB	glutathion	Gstp1
9929	Gstp2	0.562	6.471	3.637	0.001	0.002	0.056	11.049	10.217	12.912	14869	Gstp2	chr19	-	NM_1817	P19382	protein_cc	Gst-3| Gst	glutathion	Gstp2
9930	BC021614	1.025	0.893	0.916	0.950	0.777	0.823	5.266	5.303	5.140	225884	BC021614	chr19	-	NM_1448	P17070	protein_cc	.	cDNA seq	BC021614
9931	Cabp2	1.000	1.000	1.000	1.000	1.000	1.000	0.000	0.000	0.000	29866	Cabp2	chr19	+	NM_0011	P1825	protein_cc	.	calcium bi	Cabp2
9932	Cdk2ap2	2.376	0.595	1.415	0.016	0.045	0.241	9.349	10.598	9.850	52004	Cdk2ap2	chr19	+	NM_0263	P7957	protein_cc	5830466O	CDK2-asso	Cdk2ap2
9933	Pitpnm1	2.082	1.149	2.392	0.009	0.520	0.219	2.950	4.008	4.208	18739	Pitpnm1	chr19	+	NR_07507	.	protein_cc	DRES9| M	phosphati	Pitpnm1
9934	Aip	1.024	1.088	1.114	0.736	0.135	0.482	7.580	7.614	7.735	11632	Aip	chr19	-	NM_0166	P19466	protein_cc	AA408703	aryl-hydro	Aip
9935	Tmem134	0.339	1.802	0.610	0.000	0.002	0.011	10.251	8.689	9.538	66990	Tmem134	chr19	+	NM_0258	P12284	protein_cc	2410001H	transmem	Tmem134
9936	Cabp4	1.058	0.898	0.950	0.887	0.785	0.920	0.587	0.669	0.513	73660	Cabp4	chr19	-	NM_1445	P4811	protein_cc	2410038D	calcium bi	Cabp4
9937	Gpr152	1.000	1.000	1.000	1.000	1.000	1.000	0.000	0.000	0.000	269053	Gpr152	chr19	+	NM_2069	P26166	protein_cc	A930009H	G protein-	Gpr152
9938	Coro1b	0.805	0.823	0.663	0.007	0.071	0.068	9.518	9.206	8.925	23789	Coro1b	chr19	+	NM_0117	P18539	protein_cc	.	coronin, a	Coro1b
9939	Ptprcap	3.313	0.335	1.109	0.037	0.030	0.887	3.306	5.034	3.455	19265	Ptprcap	chr19	+	NM_0169	P20324	protein_cc	AI605264|	protein ty	Ptprcap
9940	Rps6kb2	0.435	0.716	0.312	0.034	0.017	0.146	5.161	3.960	3.478	58988	Rps6kb2	chr19	-	NM_0214	P2267	protein_cc	70kDa| S6	ribosomal	Rps6kb2
9941	Carns1	1.582	0.844	1.335	0.201	0.476	0.501	3.285	3.947	3.702	107239	Carns1	chr19	-	NM_1341	P14755	protein_cc	AI790298|	carnosine	Carns1
9942	Tbc1d10c	1.492	1.284	1.916	0.371	0.122	0.380	2.113	2.690	3.051	108995	Tbc1d10c	chr19	-	NM_1786	P14671	protein_cc	18100620	TBC1 dom	Tbc1d10c
9943	Ppp1ca	0.626	1.156	0.723	0.002	0.251	0.085	10.171	9.495	9.704	19045	Ppp1ca	chr19	+	NM_0318	P12697	protein_cc	dism2| Pp	protein ph	Ppp1ca
9944	Rad9a	0.643	1.176	0.757	0.012	0.296	0.126	9.895	9.258	9.492	19367	Rad9a	chr19	-	NM_0112	P19551	protein_cc	Rad9	RAD9 chec	Rad9a
9945	Clcf1	1.337	1.460	1.952	0.583	0.453	0.517	2.735	3.154	3.699	56708	Clcf1	chr19	+	NM_0199	P24794	protein_cc	BSF-3| Bsf	cardiotrop	Clcf1
9946	Clcf1-pold	1.099	1.527	1.678	0.525	0.034	0.113	6.207	6.344	6.954	10594858	Clcf1-pold	chr19	+	NR_13230	.	lncRNA	.	Clcf1-pold	Clcf1-pold
9947	Pold4	1.000	1.000	1.000	1.000	1.000	1.000	0.000	0.000	0.000	69745	Pold4	chr19	+	NM_0271	P16308	protein_cc	2410012M	polymeras	Pold4
9948	Ssh3	1.479	1.121	1.658	0.068	0.296	0.209	5.726	6.291	6.456	245857	Ssh3	chr19	-	NM_1981	P16939	protein_cc	BC028922	slingshot	Ssh3
9949	Ankrd13d	0.637	0.608	0.387	0.404	0.450	0.293	1.796	1.145	0.427	68423	Ankrd13d	chr19	-	NM_0267	P4261	protein_cc	0710001P	ankyrin re	Ankrd13d
9950	Adrbk1	2.172	0.845	1.836	0.000	0.066	0.033	6.987	8.105	7.863	110355	Adrbk1	chr19	-	NM_0129	P25400	protein_cc	Adrbk-1| I	adrenergi	Adrbk1
9951	Kdm2a	1.442	0.577	1.051	0.009	0.003	0.036	8.607	9.135	8.342	225876	Kdm2a	chr19	-	NM_0010	P17998	protein_cc	10004362	lysine (K)-	Kdm2a
9952	Rhod	0.628	1.944	1.221	0.010	0.005	0.256	7.770	7.099	8.058	11854	Rhod	chr19	-	NM_0074	P3197	protein_cc	AI326383|	ras homol	Rhod
9953	A930001C	18.863	0.667	12.574	0.000	0.024	0.048	3.482	7.720	7.135	319314	A930001C	chr19	+	NR_04599	.	lncRNA	.	RIKEN cDN	A930001C
9954	Syt12	139.773	0.622	86.977	0.000	0.042	0.050	1.005	8.132	7.448	171180	Syt12	chr19	-	NM_1341	P6166	protein_cc	.	synaptota	Syt12
9955	2010003K	5.261	0.790	4.158	0.002	0.557	0.356	5.415	7.811	7.471	69861	2010003K	chr19	-	NM_0272	P25334	protein_cc	.	RIKEN cDN	2010003K
9956	Pcx	1.054	1.166	1.228	0.741	0.480	0.535	9.895	9.970	10.191	18563	Pcx	chr19	+	NM_0011	P15438	protein_cc	Pc| Pcb	pyruvate c	Pcx
9957	Lrfn4	4.332	0.115	0.498	0.218	1.000	0.423	1.004	3.119	0.000	225875	Lrfn4	chr19	-	NM_1533	P4618	protein_cc	BC023156	leucine ric	Lrfn4
9958	Rce1	0.668	0.957	0.640	0.002	0.356	0.023	6.549	5.968	5.905	19671	Rce1	chr19	-	NM_0231	P23976	protein_cc	D19Ertd28	Ras conve	Rce1
9959	Gm960	1.000	1.000	1.000	1.000	1.000	1.000	0.000	0.000	0.000	381196	Gm960	chr19	-	NM_0010	P10516	protein_cc	Top6bl| T(	predicted	Gm960
9960	Sptbn2	0.109	1.909	0.207	0.000	0.002	0.003	8.304	5.102	6.035	20743	Sptbn2	chr19	+	NM_0212	P1993	protein_cc	mKIAA030	spectrin b	Sptbn2
9961	Rbm4b	0.986	0.742	0.732	0.951	0.327	0.267	6.667	6.647	6.217	66704	Rbm4b	chr19	-	NM_0257	P7032	protein_cc	49215061	RNA bindi	Rbm4b
9962	Gm21992	1.778	0.719	1.278	0.055	0.173	0.255	7.482	8.312	7.836	10290267	Gm21992	chr19	-	NM_0012	P9655	protein_cc	Rbm14-rb	predicted	Gm21992
9963	Rbm4	1.781	0.724	1.290	0.053	0.176	0.246	7.482	8.315	7.849	19653	Rbm4	chr19	-	NM_0090	P13828	protein_cc	49215061	RNA bindi	Rbm4
9964	Rbm14	2.872	0.463	1.329	0.000	0.020	0.676	6.117	7.639	6.527	56275	Rbm14	chr19	-	NM_0198	P12709	protein_cc	1300007E	RNA bindi	Rbm14
9965	Ccs	0.305	1.062	0.324	0.000	0.538	0.005	10.612	8.898	8.985	12460	Ccs	chr19	-	NM_0168	P21841	protein_cc	Ccsd	copper ch	Ccs
9966	Ccdc87	1.010	1.825	1.844	0.000	0.182	0.423	0.000	0.015	0.883	399599	Ccdc87	chr19	+	NM_2072	P21696	protein_cc	4931419P	coiled-coil	Ccdc87
9967	Ctsf	0.350	1.260	0.441	0.003	0.057	0.077	7.529	6.013	6.346	56464	Ctsf	chr19	+	NM_0198	P386	protein_cc	AI481912	cathepsin	Ctsf
9968	Actn3	1.000	1.000	1.000	1.000	1.000	1.000	0.000	0.000	0.000	11474	Actn3	chr19	-	NM_0134	P13396	protein_cc	.	actinin alp	Actn3
9969	Zdhhc24	1.330	0.775	1.030	0.004	0.179	0.932	4.307	4.719	4.350	70605	Zdhhc24	chr19	+	NM_0011	P8322	protein_cc	5730496N	zinc finger	Zdhhc24
9970	Bbs1	1.334	1.894	2.527	0.183	0.000	0.050	3.598	4.014	4.936	52028	Bbs1	chr19	-	NM_0010	P10692	protein_cc	AI451249|	Bardet-Bie	Bbs1
9971	Dpp3	0.657	1.486	0.976	0.093	0.096	0.801	9.006	8.399	8.970	75221	Dpp3	chr19	-	NM_1338	P11133	protein_cc	49305330	dipeptidyl	Dpp3
9972	Peli3	0.469	4.796	2.251	0.001	0.182	0.560	1.156	0.065	2.327	240518	Peli3	chr19	-	NM_1728	P8634	protein_cc	6030441F	pellino 3	Peli3
9973	Mrpl11	0.942	1.137	1.071	0.574	0.292	0.467	8.737	8.651	8.836	66419	Mrpl11	chr19	+	NM_0255	P22585	protein_cc	2410001P	mitochond	Mrpl11
9974	Npas4	1.572	0.636	1.000	0.304	1.000	0.423	0.000	0.653	0.000	225872	Npas4	chr19	-	NM_1535	P5278	protein_cc	LE-PAS| N	neuronal P	Npas4
9975	Slc29a2	0.192	2.125	0.407	0.003	0.103	0.047	3.913	1.529	2.617	13340	Slc29a2	chr19	+	NM_0078	P10504	protein_cc	Der12| En	solute car	Slc29a2
9976	B4gat1	0.153	2.056	0.315	0.000	0.059	0.027	7.810	5.103	6.143	108902	B4gat1	chr19	+	NM_1753	P10966	protein_cc	1500032N	beta-1,4-g	B4gat1
9977	Brms1	1.727	1.060	1.831	0.053	0.694	0.152	5.400	6.188	6.273	107392	Brms1	chr19	+	NM_1341	P6267	protein_cc	AV003220	breast car	Brms1
9978	Rin1	2.137	1.869	3.994	0.282	0.186	0.127	1.113	2.208	3.111	225870	Rin1	chr19	+	NM_1454	P10729	protein_cc	.	Ras and R	Rin1
9979	Cd248	0.082	3.019	0.246	0.000	0.191	0.053	3.734	0.119	1.713	70445	Cd248	chr19	+	NM_0540	P5433	protein_cc	2610111G	CD248 ant	Cd248
9980	Tmem151	0.726	1.425	1.034	0.187	0.186	0.943	0.476	0.015	0.525	381199	Tmem151	chr19	-	NM_0010	P18815	protein_cc	AW12568	transmem	Tmem151
9981	Yif1a	0.981	1.129	1.108	0.728	0.021	0.328	9.457	9.429	9.605	68090	Yif1a	chr19	+	NM_0265	P10112	protein_cc	5330422J	Yip1 intera	Yif1a
9982	Cnih2	6.018	0.324	1.952	0.034	0.070	0.433	0.722	3.311	1.687	12794	Cnih2	chr19	-	NM_0013	P24232	protein_cc	CNIH-2| C	cornichon	Cnih2
9983	Rab1b	0.569	1.550	0.882	0.000	0.000	0.090	9.132	8.318	8.950	76308	Rab1b	chr19	-	NM_0295	P26546	protein_cc	1110011F	RAB1B, m	Rab1b
9984	Klc2	1.284	1.058	1.359	0.003	0.767	0.444	5.074	5.435	5.517	16594	Klc2	chr19	-	NM_0084	P13258	protein_cc	8030455F	kinesin lig	Klc2
9985	Pacs1	1.015	2.098	2.130	0.921	0.042	0.225	3.628	3.650	4.719	107975	Pacs1	chr19	-	NM_1531	P4651	protein_cc	AI325977	phosphofu	Pacs1
9986	Sf3b2	0.936	0.897	0.840	0.283	0.231	0.337	10.804	10.709	10.552	319322	Sf3b2	chr19	-	NM_0301	P15468	protein_cc	145kDa| 2	splicing fa	Sf3b2
9987	Gal3st3	0.743	2.201	1.635	0.196	0.007	0.246	0.456	0.027	1.165	545276	Gal3st3	chr19	+	NM_0010	P15604	protein_cc	.	galactose-	Gal3st3
9988	Catsper1	1.000	1.000	1.000	1.000	1.000	1.000	0.000	0.000	0.000	225865	Catsper1	chr19	+	NM_1393	P14277	protein_cc	Catsper| I	cation cha	Catsper1
9989	Cst6	1.000	1.000	1.000	1.000	1.000	1.000	0.000	0.000	0.000	73720	Cst6	chr19	-	NM_0286	P15050	protein_cc	1110017E	cystatin E/	Cst6
9990	Banf1	0.900	1.130	1.017	0.043	0.030	0.835	9.710	9.559	9.735	23825	Banf1	chr19	-	NR_10448	.	protein_cc	Baf| Bcrp1	barrier to	Banf1
9991	Eif1ad	1.339	1.220	1.633	0.019	0.031	0.067	7.920	8.340	8.627	69860	Eif1ad	chr19	+	NM_0272	P20084	protein_cc	2010003J(	eukaryoti	Eif1ad
9992	Sart1	1.026	1.140	1.169	0.851	0.307	0.432	7.212	7.249	7.438	20227	Sart1	chr19	-	NM_0168	P7564	protein_cc	U5-110K	squamous	Sart1
9993	D330050I	0.638	1.678	1.071	0.384	0.361	0.884	3.335	2.688	3.434	414115	D330050I	chr19	+	NR_03322	.	lncRNA	.	RIKEN cDN	D330050I1
9994	4930481A	0.665	1.372	0.913	0.001	0.044	0.581	9.143	8.555	9.012	74931	4930481A	chr19	+	NR_02793	.	lncRNA	.	RIKEN cDN	4930481A
9995	Drap1	0.569	1.648	0.938	0.000	0.005	0.590	9.841	9.029	9.749	66556	Drap1	chr19	-	NM_0241	P21157	protein_cc	2310074H	Dr1 associ	Drap1
9996	AI837181	1.312	0.907	1.190	0.269	0.680	0.624	9.158	9.550	9.410	107242	AI837181	chr19	+	NM_1341	P6558	protein_cc	Bles03| N	expressed	AI837181
9997	Fosl1	41.299	0.430	17.761	0.025	0.118	0.065	0.330	5.698	4.481	14283	Fosl1	chr19	+	NM_0102	P14839	protein_cc	AW53819	fos-like ar	Fosl1

9998	Ccdc85b	3.925	0.530	2.082	0.017	0.072	0.053	4.056	6.029	5.114	240514	Ccdc85b	chr19	-	NM_0012	P25861	protein_cc	AI842788|	coiled-coil	Ccdc85b
9999	Fibp	0.939	1.642	1.542	0.017	0.000	0.003	7.531	7.440	8.156	58249	Fibp	chr19	+	NM_0012	P5771	protein_cc	2010004G	fibroblast	Fibp
10000	Ctsw	0.543	0.420	0.228	0.003	0.021	0.019	2.895	2.015	0.764	13041	Ctsw	chr19	-	NM_0099	P69	protein_cc	lymphopa	cathepsin	Ctsw
10001	Efemp2	0.291	2.244	0.653	0.128	0.129	0.648	4.195	2.414	3.580	58859	Efemp2	chr19	+	NM_0214	P4283	protein_cc	0610011K	epidermal	Efemp2
10002	Mus81	1.965	0.575	1.129	0.028	0.002	0.795	3.637	4.612	3.812	71711	Mus81	chr19	-	NM_0278	P20171	protein_cc	1200008A	MUS81 st	Mus81
10003	Cfl1	1.358	0.992	1.347	0.108	0.953	0.126	10.314	10.756	10.743	12631	Cfl1	chr19	+	NM_0076	P22104	protein_cc	AA959946	cofilin 1, n	Cfl1
10004	Snx32	0.163	3.835	0.626	0.007	0.068	0.437	3.378	0.763	2.702	225861	Snx32	chr19	-	NM_0010	P20721	protein_cc	B930037P	sorting ne	Snx32
10005	1700020D	1.010	1.885	1.904	0.000	0.182	0.423	0.000	0.015	0.929	75555	1700020D	chr19	-	NM_0237	P10124	protein_cc	mage-g2	RIKEN cDN	1700020D
10006	Ovol1	1.792	1.109	1.986	0.389	0.806	0.364	1.835	2.676	2.825	18426	Ovol1	chr19	-	NM_0199	P13502	protein_cc	BB147136	ovo like zi	Ovol1
10007	Ap5b1	4.759	0.325	1.544	0.003	0.012	0.680	2.962	5.213	3.589	381201	Ap5b1	chr19	+	NM_0010	P12754	protein_cc	Gm962	adaptor-re	Ap5b1
10008	Rnaseh2c	0.614	1.299	0.798	0.004	0.020	0.229	8.792	8.089	8.467	68209	Rnaseh2c	chr19	+	NM_0266	P24753	protein_cc	1500026D	ribonuclea	Rnaseh2c
10009	Kat5	1.578	0.997	1.574	0.020	0.985	0.176	5.850	6.508	6.504	81601	Kat5	chr19	-	NR_03760	.	protein_cc	AI839539|	K(lysine) a	Kat5
10010	Rela	3.025	0.734	2.220	0.002	0.180	0.220	8.374	9.971	9.524	19697	Rela	chr19	+	NM_0090	P25365	protein_cc	p65	v-rel retic	Rela
10011	Sipa1	1.034	0.775	0.802	0.694	0.242	0.519	6.447	6.496	6.128	20469	Sipa1	chr19	-	NM_0113	P17860	protein_cc	Spa1	signal-indu	Sipa1
10012	Pcnxl3	0.668	1.047	0.700	0.111	0.818	0.457	8.028	7.446	7.513	.	Pcnxl3	chr19	-	NM_1448	P21978	.	.	.	Pcnxl3
10013	Map3k11	1.217	1.146	1.395	0.203	0.459	0.340	6.639	6.922	7.119	26403	Map3k11	chr19	+	NM_0220	P19793	protein_cc	2610017K	mitogen-a	Map3k11
10014	Kcnk7	1.000	1.000	1.000	1.000	1.000	1.000	0.000	0.000	0.000	16530	Kcnk7	chr19	+	NM_0106	P7177	protein_cc	2310014G	potassium	Kcnk7
10015	Ehbp1l1	1.162	1.163	1.351	0.589	0.531	0.105	5.690	5.907	6.124	114601	Ehbp1l1	chr19	-	NM_0532	P22377	protein_cc	G430002G	EH domain	Ehbp1l1
10016	Fam89b	0.580	1.478	0.858	0.091	0.027	0.689	7.182	6.397	6.960	17826	Fam89b	chr19	-	NM_0231	P13537	protein_cc	1110021A	family wit	Fam89b
10017	Sssca1	0.907	1.441	1.306	0.342	0.024	0.225	7.289	7.148	7.675	56390	Sssca1	chr19	-	NM_0204	P16233	protein_cc	1500016H	Sjogren's s	Sssca1
10018	Ltbp3	1.533	1.061	1.626	0.011	0.657	0.131	8.045	8.662	8.747	16998	Ltbp3	chr19	+	NM_0085	P20656	protein_cc	Ltbp2| mF	latent tran	Ltbp3
10019	Scyl1	1.478	1.024	1.513	0.004	0.814	0.126	8.391	8.955	8.989	78891	Scyl1	chr19	-	NM_0239	P15710	protein_cc	2810011O	SCY1-like 1	Scyl1
10020	Malat1	1.563	0.327	0.512	0.014	0.001	0.019	13.339	13.984	12.373	72289	Malat1	chr19	-	NR_00284	.	lncRNA	2210401K	metastasis	Malat1
10021	Neat1	1.147	0.782	0.897	0.047	0.035	0.479	9.756	9.954	9.599	66961	Neat1	chr19	-	NR_13121	.	lncRNA	2310043N	nuclear pa	Neat1
10022	Frmd8	0.678	2.302	1.560	0.015	0.000	0.054	7.511	6.950	8.153	67457	Frmd8	chr19	-	NM_0261	P19965	protein_cc	1200004M	FERM dom	Frmd8
10023	Slc25a45	0.248	1.512	0.375	0.000	0.028	0.033	8.441	6.429	7.026	107375	Slc25a45	chr19	+	NM_1341	P23154	protein_cc	AW49144	solute car	Slc25a45
10024	Tigd3	0.346	0.610	0.211	0.105	1.000	0.242	2.246	0.713	0.000	332359	Tigd3	chr19	-	NM_1986	P22099	protein_cc	.	tigger tran	Tigd3
10025	Dpf2	1.590	0.728	1.157	0.000	0.076	0.659	7.298	7.967	7.509	19708	Dpf2	chr19	-	NM_0112	P5252	protein_cc	2210010M	D4, zinc ar	Dpf2
10026	Cdc42ep2	3.850	0.473	1.820	0.000	0.000	0.029	6.177	8.122	7.041	104252	Cdc42ep2	chr19	-	NM_0267	P5443	protein_cc	1110008C	CDC42 eff	Cdc42ep2
10027	Pola2	1.010	0.865	0.874	0.929	0.453	0.733	5.677	5.692	5.484	18969	Pola2	chr19	-	NM_0088	P17696	protein_cc	AI573378	polymeras	Pola2
10028	Slc22a20	1.000	1.000	1.000	1.000	1.000	1.000	0.000	0.000	0.000	381203	Slc22a20	chr19	-	NM_1986	P18588	protein_cc	Gm963| n	solute car	Slc22a20
10029	Capn1	0.700	2.350	1.645	0.067	0.024	0.262	5.970	5.456	6.688	12333	Capn1	chr19	-	NM_0011	P7150	protein_cc	Capa-1| C	calpain 1	Capn1
10030	Gm10814	1.000	1.000	1.000	1.000	1.000	1.000	0.000	0.000	0.000	10050298	Gm10814	chr19	+	NR_04578	.	lncRNA	ENSMUSG	predicted	Gm10814
10031	Syvn1	4.249	0.401	1.706	0.003	0.011	0.247	9.010	11.097	9.781	74126	Syvn1	chr19	+	NM_0287	P751	protein_cc	1200010C	synovial a	Syvn1
10032	Mrpl49	2.553	0.467	1.193	0.001	0.002	0.252	8.964	10.316	9.219	18120	Mrpl49	chr19	-	NM_0262	P21916	protein_cc	4833419D	mitochond	Mrpl49
10033	Fau	0.829	1.386	1.148	0.124	0.040	0.424	10.805	10.534	11.004	14109	Fau	chr19	+	NM_0011	P16116	protein_cc	MNSFbeta	Finkel-Bisk	Fau
10034	Znhit2	1.140	1.050	1.197	0.651	0.817	0.558	7.325	7.514	7.585	29805	Znhit2	chr19	+	NM_0138	P14014	protein_cc	C11orf5| I	zinc finger	Znhit2
10035	Tm7sf2	0.479	0.966	0.462	0.002	0.864	0.050	8.685	7.622	7.572	73166	Tm7sf2	chr19	-	NM_0284	P465	protein_cc	3110041O	transmem	Tm7sf2
10036	Vps51	0.713	1.325	0.945	0.045	0.088	0.636	7.906	7.417	7.823	68505	Vps51	chr19	-	NM_0010	P1653	protein_cc	1110014N	vacuolar p	Vps51
10037	BC048609	1.542	1.641	2.531	0.504	0.235	0.065	3.280	3.905	4.620	433215	BC048609	chr19	+	NM_0011	P3828	protein_cc	.	cDNA seq	BC048609
10038	Zfpl1	1.067	1.127	1.203	0.597	0.503	0.550	6.739	6.833	7.006	81909	Zfpl1	chr19	-	NM_0242	P3352	protein_cc	1500015B	zinc finger	Zfpl1
10039	Cdca5	0.726	0.459	0.333	0.629	1.000	0.422	1.585	1.123	0.000	67849	Cdca5	chr19	+	NM_0264	P12963	protein_cc	2610036L	cell divisio	Cdca5
10040	Naaladl1	1.000	1.000	1.000	1.000	1.000	1.000	0.000	0.000	0.000	381204	Naaladl1	chr19	+	NM_0010	P24682	protein_cc	Gm964| N	N-acetylat	Naaladl1
10041	Sac3d1	0.986	1.149	1.133	0.918	0.097	0.624	6.807	6.786	6.986	66406	Sac3d1	chr19	-	NM_1336	P5585	protein_cc	2410004C	SAC3 dom	Sac3d1
10042	Snx15	0.888	0.980	0.871	0.393	0.918	0.596	6.099	5.928	5.899	69024	Snx15	chr19	-	NM_0269	P19107	protein_cc	1500032B	sorting ne	Snx15
10043	Arl2	0.845	1.230	1.040	0.044	0.000	0.692	5.531	5.288	5.587	56327	Arl2	chr19	-	NM_0197	P13123	protein_cc	2610009M	ADP-ribos	Arl2
10044	Batf2	100.090	0.173	17.346	0.004	0.008	0.147	1.234	7.879	5.350	74481	Batf2	chr19	+	NM_0289	P14482	protein_cc	4933430F	basic leuc	Batf2
10045	1700123I0	3.945	0.872	3.440	0.305	0.854	0.133	0.001	1.981	1.784	.	1700123I0	chr19	+	NM_0011	P18016	.	.	.	1700123I0
10046	Gpha2	4.991	0.810	4.040	0.000	0.096	0.055	4.305	6.624	6.319	170458	Gpha2	chr19	-	NM_1304	P10331	protein_cc	GPA2| zsi	glycoprote	Gpha2
10047	Ppp2r5b	3.781	0.859	3.247	0.001	0.319	0.125	5.239	7.157	6.938	225849	Ppp2r5b	chr19	-	NM_1981	P1667	protein_cc	B'beta| B(	protein ph	Ppp2r5b
10048	Atg2a	0.689	1.708	1.178	0.001	0.000	0.122	7.966	7.430	8.202	329015	Atg2a	chr19	+	NM_1943	P11650	protein_cc	1810013C	autophagy	Atg2a
10049	Ehd1	2.221	0.605	1.343	0.000	0.003	0.323	8.979	10.131	9.405	13660	Ehd1	chr19	+	NM_0101	P20557	protein_cc	AA409636	EH-domain	Ehd1
10050	Cdc42bpg	0.291	1.630	0.475	0.000	0.002	0.005	6.294	4.514	5.219	240505	Cdc42bpg	chr19	+	NM_0010	P1483	protein_cc	BC046418	CDC42 bin	Cdc42bpg
10051	Men1	1.152	0.685	0.789	0.503	0.167	0.457	7.107	7.311	6.766	17283	Men1	chr19	+	NM_0011	P19874	protein_cc	AW04561	multiple e	Men1
10052	Map4k2	0.711	1.021	0.726	0.213	0.968	0.622	5.185	4.693	4.723	26412	Map4k2	chr19	+	NM_0090	P21502	protein_cc	AI385662|	mitogen-a	Map4k2
10053	Sf1	1.141	0.725	0.827	0.074	0.087	0.541	10.920	11.111	10.647	22668	Sf1	chr19	+	NM_0117	P18411	protein_cc	BBP| MZF	splicing fa	Sf1
10054	Pygm	0.567	0.462	0.262	0.288	0.407	0.070	3.642	2.824	1.709	19309	Pygm	chr19	+	NM_0112	P18021	protein_cc	AI115133|	muscle gly	Pygm
10055	Rasgrp2	0.404	1.182	0.478	0.001	0.630	0.125	7.293	5.987	6.228	19395	Rasgrp2	chr19	+	NM_0112	P17426	protein_cc	Caldaggef	RAS, guan	Rasgrp2
10056	Nrxn2	0.112	1.265	0.142	0.000	0.569	0.005	6.740	3.582	3.921	18190	Nrxn2	chr19	+	NM_0202	P7214	protein_cc	6430591O	neurexin I	Nrxn2
10057	Slc22a12	1.010	1.825	1.844	0.000	0.182	0.423	0.000	0.015	0.883	20521	Slc22a12	chr19	-	NM_0092	P3600	protein_cc	AI987855|	solute car	Slc22a12
10058	Rps6ka4	1.909	1.667	3.181	0.039	0.057	0.073	4.724	5.656	6.393	56613	Rps6ka4	chr19	-	NM_0199	P12730	protein_cc	1110069D	ribosomal	Rps6ka4
10059	Ccdc88b	4.031	0.244	0.982	0.000	0.000	0.974	2.857	4.868	2.831	78317	Ccdc88b	chr19	-	NM_0010	P25096	protein_cc	2610041P	coiled-coil	Ccdc88b
10060	Prdx5	0.975	0.961	0.937	0.718	0.279	0.638	10.615	10.578	10.522	54683	Prdx5	chr19	-	NM_0120	P13848	protein_cc	AOEB166|	peroxired	Prdx5
10061	Trmt112	1.059	1.117	1.183	0.825	0.632	0.395	9.252	9.335	9.494	67674	Trmt112	chr19	+	NM_0263	P19430	protein_cc	0610038D	tRNA met	Trmt112
10062	Esrra	0.933	1.234	1.151	0.787	0.380	0.431	8.961	8.860	9.164	26379	Esrra	chr19	-	NM_0079	P19041	protein_cc	Err1| ERRa	estrogen r	Esrra
10063	Tex40	1.000	1.000	1.000	1.000	1.000	1.000	0.000	0.000	0.000	67077	Tex40	chr19	-	NM_0010	P5126	protein_cc	1700019N	testis expr	Tex40
10064	Kcnk4	1.000	1.000	1.000	1.000	1.000	1.000	0.000	0.000	0.000	16528	Kcnk4	chr19	-	NM_0084	P10946	protein_cc	MLZ-622|	potassium	Kcnk4
10065	Gpr137	0.512	0.831	0.426	0.000	0.140	0.017	7.054	6.089	5.822	107173	Gpr137	chr19	-	NM_0011	P7294	protein_cc	AI428855	G protein-	Gpr137
10066	Bad	1.056	1.366	1.443	0.700	0.198	0.352	8.268	8.347	8.797	12015	Bad	chr19	+	NM_0075	P25539	protein_cc	AI325008|	BCL2-asso	Bad
10067	Plcb3	0.485	1.782	0.865	0.011	0.183	0.772	5.112	4.068	4.902	18797	Plcb3	chr19	-	NM_0088	P26688	protein_cc	mKIAA40S	phospholi	Plcb3
10068	Ppp1r14b	1.998	1.335	2.667	0.027	0.095	0.026	6.777	7.776	8.192	18938	Ppp1r14b	chr19	+	NM_0088	P120	protein_cc	AOM172|	protein ph	Ppp1r14b
10069	Fkbp2	0.678	1.436	0.974	0.004	0.030	0.893	8.683	8.123	8.645	14227	Fkbp2	chr19	-	NM_0011	P11346	protein_cc	FKBP13| FK	FK506 bin	Fkbp2
10070	Vegfb	0.396	1.612	0.639	0.002	0.188	0.237	7.217	5.880	6.570	22340	Vegfb	chr19	-	NM_0113	P20128	protein_cc	VEGF-B| V	vascular e	Vegfb
10071	Dnajc4	0.572	1.471	0.842	0.005	0.009	0.191	6.790	5.985	6.542	57431	Dnajc4	chr19	-	NM_0205	P6289	protein_cc	2010301J2	DnaJ heat	Dnajc4
10072	Nudt22	0.828	1.130	0.936	0.420	0.570	0.687	6.813	6.541	6.718	68323	Nudt22	chr19	-	NM_0266	P23328	protein_cc	0610006K	nudix (nuc	Nudt22
10073	Trpt1	1.164	1.037	1.207	0.069	0.882	0.681	5.854	6.074	6.126	107328	Trpt1	chr19	+	NM_1535	P20891	protein_cc	AU079016	tRNA phos	Trpt1
10074	Fermt3	1.297	1.069	1.387	0.004	0.756	0.470	5.937	6.312	6.408	108101	Fermt3	chr19	-	NM_1537	P3173	protein_cc	C79673| K	fermitin fa	Fermt3
10075	Stip1	1.493	0.602	0.898	0.041	0.029	0.469	8.528	9.106	8.373	20867	Stip1	chr19	-	NM_0167	P24258	protein_cc	Hop| p60|	stress-ind	Stip1
10076	Macrod1	0.310	0.925	0.286	0.000	0.678	0.024	8.718	7.027	6.914	107227	Macrod1	chr19	+	NM_1341	P13311	protein_cc	AI604841|	MACRO do	Macrod1
10077	Flrt1	0.041	8.755	0.360	0.000	0.154	0.180	4.767	0.163	3.294	396184	Flrt1	chr19	-	NM_2014	P8091	protein_cc	AW74216	fibronecti	Flrt1
10078	Otub1	0.755	1.758	1.327	0.017	0.001	0.022	7.609	7.203	8.017	107260	Otub1	chr19	-	NM_1341	P1507	protein_cc	AI850305	OTU doma	Otub1
10079	Cox8a	0.737	0.856	0.631	0.154	0.430	0.126	12.165	11.725	11.501	12868	Cox8a	chr19	-	NM_0077	P23955	protein_cc	COX8L	cytochrom	Cox8a
10080	Naa40	0.856	0.735	0.629	0.492	0.134	0.273	6.635	6.410	5.965	70999	Naa40	chr19	-	NM_0276	P3806	protein_cc	4931433E	N(alpha)-a	Naa40
10081	Rcor2	3.316	0.301	1.000	0.070	1.000	0.423	0.001	1.730	0.000	104383	Rcor2	chr19	+	NM_0540	P21966	protein_cc	1A13| AW	REST core	Rcor2
10082	Mark2	2.028	0.789	1.599	0.000	0.011	0.048	6.102	7.121	6.779	13728	Mark2	chr19	-	NM_0010	P5757	protein_cc	Emk| EMK	MAP/micr	Mark2
10083	AI846148	2.341	0.757	1.772	0.027	0.395	0.443	3.419	4.646	4.244	68229	AI846148	chr19	-	NM_0010	P24413	protein_cc	1700105P	expressed	AI846148
10084	1700105P	1.010	1.885	1.904	0.000	0.182	0.423	0.000	0.015	0.929	654802	1700105P	chr19	+	NR_04570	.	lncRNA		RIKEN cDN	1700105P
10085	2700081O	1.051	0.945	0.994	0.642	0.834	0.990	5.258	5.330	5.248	108899	2700081O	chr19	+	NM_1753	P7460	protein_cc	AI255177|	RIKEN cDN	2700081O
10086	Rtn3	0.803	1.170	0.940	0.001	0.013	0.449	10.675	10.358	10.585	20168	Rtn3	chr19	-	NM_0530	P11188	protein_cc	RTN3-A1	reticulon 3	Rtn3
10087	Snord118	1.572	0.636	1.000	0.304	1.000	0.423	0.000	0.653	0.000	10021653	Snord118	chr19	+	NR_02856	.	snoRNA	MBI-34	small nucl	Snord118
10088	Atl3	0.350	1.864	0.653	0.005	0.000	0.213	7.983	6.470	7.368	109168	Atl3	chr19	+	NM_0011	P20761	protein_cc	4633402C	atlastin GT	Atl3

10089	Pla2g16	2.125	0.626	1.330	0.017	0.056	0.435	8.338	9.426	8.750	225845	Pla2g16	chr19	+	NM_1392	P14708	protein_cc	C78643| H	phospholi	Pla2g16
10090	Lgals12	1.000	1.000	1.000	1.000	1.000	1.000	0.000	0.000	0.000	56072	Lgals12	chr19	-	NM_0195	P1132	protein_cc	AW98743	lectin, gal	Lgals12
10091	Hrasls5	1.000	1.000	1.000	1.000	1.000	1.000	0.000	0.000	0.000	66727	Hrasls5	chr19	+	NM_0257	P12067	protein_cc	1700055A	HRAS-like	Hrasls5
10092	Slc22a19	1.000	1.000	1.000	1.000	1.000	1.000	0.000	0.000	0.000	207151	Slc22a19	chr19	-	NM_1447	P14687	protein_cc	D630043A	solute car	Slc22a19
10093	Slc22a26	0.483	0.039	0.019	0.008	0.005	0.013	7.602	6.551	1.882	236149	Slc22a26	chr19	-	NM_1462	P6273	protein_cc	.	solute car	Slc22a26
10094	Slc22a27	0.220	0.076	0.017	0.000	0.000	0.005	7.993	5.808	2.092	171405	Slc22a27	chr19	-	NM_1342	P26192	protein_cc	mOAT6-L|	solute car	Slc22a27
10095	Slc22a28	0.291	1.464	0.426	0.074	0.530	0.337	4.356	2.576	3.126	434674	Slc22a28	chr19	-	NM_0010	P22945	protein_cc	EG434674	solute car	Slc22a28
10096	Slc22a29	0.069	0.324	0.023	0.000	0.006	0.007	5.915	2.066	0.441	236293	Slc22a29	chr19	-	NM_1727	P2047	protein_cc	D630002G	solute car	Slc22a29
10097	Slc22a30	0.201	0.483	0.097	0.000	0.074	0.006	7.910	5.595	4.546	319800	Slc22a30	chr19	-	NM_1770	P6349	protein_cc	AA986766	solute car	Slc22a30
10098	Slc22a8	1.000	1.000	1.000	1.000	1.000	1.000	0.000	0.000	0.000	19879	Slc22a8	chr19	+	NM_0011	P21669	protein_cc	Oat3| Roc	solute car	Slc22a8
10099	Slc22a6	1.000	1.000	1.000	1.000	1.000	1.000	0.000	0.000	0.000	18399	Slc22a6	chr19	+	NM_0087	P25983	protein_cc	mOat1| N	solute car	Slc22a6
10100	9830166K	1.005	1.439	1.446	0.000	0.182	0.423	0.000	0.008	0.532	402757	9830166K	chr19	-	NR_04531	.	lncRNA	.	RIKEN cDN	9830166K
10101	Chrm1	0.294	0.974	0.286	0.181	1.000	0.423	1.806	0.038	0.000	12669	Chrm1	chr19	+	NM_0076	P11178	protein_cc	Chrm-1| N	cholinergi	Chrm1
10102	Slc3a2	2.092	0.695	1.455	0.001	0.075	0.418	9.687	10.752	10.228	17254	Slc3a2	chr19	-	NM_0085	P4529	protein_cc	4F2| 4F2H	solute car	Slc3a2
10103	Snhg1	2.558	0.890	2.277	0.016	0.704	0.348	7.180	8.535	8.367	83673	Snhg1	chr19	+	NR_00289	.	lncRNA	.	small nucl	Snhg1
10104	Snord22	2.105	0.922	1.940	0.222	0.794	0.362	2.331	3.404	3.287	10012711	Snord22	chr19	+	NR_00444	.	snoRNA	Rnu22| U2	small nucl	Snord22
10105	Wdr74	1.581	0.983	1.554	0.005	0.815	0.036	7.071	7.733	7.707	107071	Wdr74	chr19	+	NM_1341	P17026	protein_cc	5730436H	WD repea	Wdr74
10106	1700092M	1.581	0.879	1.390	0.301	0.744	0.423	0.000	0.661	0.476	74307	1700092N	chr19	+	NM_0011	P8471	protein_cc	.	RIKEN cDN	1700092M
10107	Stx5a	2.005	0.674	1.352	0.005	0.022	0.005	7.690	8.693	8.124	56389	Stx5a	chr19	+	NM_0011	P26569	protein_cc	0610031F	syntaxin 5	Stx5a
10108	Nxf1	1.305	0.689	0.899	0.099	0.041	0.424	7.581	7.966	7.427	53319	Nxf1	chr19	+	NM_0168	P2495	protein_cc	Mex67| N	nuclear RN	Nxf1
10109	Tmem223	0.434	1.445	0.627	0.006	0.058	0.168	7.957	6.752	7.283	66836	Tmem223	chr19	+	NM_0257	P21376	protein_cc	0610006IC	transmem	Tmem223
10110	Tmem179	0.968	0.858	0.830	0.813	0.477	0.494	8.824	8.777	8.556	67706	Tmem179	chr19	-	NR_02841	.	protein_cc	0610031L	transmem	Tmem179l
10111	Taf6l	0.747	1.473	1.101	0.480	0.545	0.910	3.835	3.415	3.974	225895	Taf6l	chr19	-	NM_0011	P22368	protein_cc	2810417N	TATA-box	Taf6l
10112	Gm2518	0.809	1.393	1.127	0.636	0.600	0.882	3.872	3.566	4.044	10003995	Gm2518	chr19	+	NR_01553	.	misc_RNA	10003995	predicted	Gm2518
10113	Polr2g	1.153	0.987	1.138	0.210	0.489	0.502	7.557	7.762	7.744	67710	Polr2g	chr19	-	NM_0263	P7604	protein_cc	2410046K	polymeras	Polr2g
10114	Zbtb3	2.021	0.322	0.651	0.000	0.003	0.456	1.781	2.797	1.162	75291	Zbtb3	chr19	+	NM_1337	P18332	protein_cc	4930563N	zinc finger	Zbtb3
10115	Ttc9c	1.801	0.528	0.951	0.000	0.000	0.606	7.779	8.628	7.706	70387	Ttc9c	chr19	-	NM_0274	P25730	protein_cc	2210019E	tetratricop	Ttc9c
10116	Hnrnpul2	0.874	0.769	0.673	0.072	0.002	0.060	8.647	8.454	8.076	68693	Hnrnpul2	chr19	+	NM_0010	P24478	protein_cc	1110031N	heterogen	Hnrnpul2
10117	Gng3	3.300	0.303	1.000	0.306	1.000	0.423	0.001	1.723	0.000	14704	Gng3	chr19	-	NM_0103	P22512	protein_cc	.	guanine n	Gng3
10118	Bscl2	0.493	1.487	0.733	0.002	0.021	0.028	8.574	7.554	8.126	14705	Bscl2	chr19	+	NM_0011	P23207	protein_cc	2900097C	Berardine	Bscl2
10119	Lrrn4cl	0.756	1.547	1.170	0.669	0.294	0.820	1.549	1.146	1.776	68852	Lrrn4cl	chr19	+	NM_0010	P340	protein_cc	1110067I	LRRN4 C-t	Lrrn4cl
10120	Ubxn1	0.566	1.541	0.872	0.005	0.008	0.288	10.983	10.162	10.786	225896	Ubxn1	chr19	+	NM_1460	P20558	protein_cc	2B28| 493	UBX doma	Ubxn1
10121	Uqcc3	0.333	1.605	0.534	0.000	0.028	0.045	8.697	7.110	7.792	107197	Uqcc3	chr19	-	NM_0011	P13652	protein_cc	AI462493|	ubiquinol-	Uqcc3
10122	Lbhd1	1.507	1.284	1.934	0.031	0.336	0.254	7.466	8.057	8.417	10230857	Lbhd1	chr19	+	NM_0012	P22473	protein_cc	Gm21743	LBH doma	Lbhd1
10123	5730408K	1.585	1.076	1.705	0.001	0.657	0.222	7.371	8.035	8.140	67531	5730408K	chr19	-	NR_02786	.	lncRNA	2610018C	RIKEN cDN	5730408K
10124	1810009A	1.075	1.443	1.550	0.860	0.498	0.620	0.587	0.691	1.220	66276	1810009A	chr19	+	NM_0254	P2296	protein_cc	.	RIKEN cDN	1810009A
10125	Ints5	2.781	0.648	1.801	0.000	0.002	0.072	6.898	8.373	7.747	109077	Ints5	chr19	+	NM_1768	P17825	protein_cc	1110055N	integrator	Ints5
10126	Ganab	0.673	0.865	0.582	0.006	0.204	0.049	10.648	10.077	9.868	14376	Ganab	chr19	+	NM_0080	P7405	protein_cc	AU042638	alpha gluc	Ganab
10127	B3gat3	0.767	0.722	0.554	0.038	0.067	0.081	7.683	7.299	6.830	72727	B3gat3	chr19	+	NM_0242	P15192	protein_cc	2810405N	beta-1,3-g	B3gat3
10128	Rom1	0.321	2.300	0.738	0.001	0.023	0.335	5.761	4.122	5.323	19881	Rom1	chr19	-	NM_0090	P18938	protein_cc	M101156	rod outer	Rom1
10129	Eml3	0.522	1.600	0.836	0.018	0.276	0.691	7.255	6.318	6.997	225898	Eml3	chr19	+	NM_1448	P3766	protein_cc	BC022146	echinoder	Eml3
10130	Mta2	1.639	0.769	1.260	0.043	0.139	0.527	7.951	8.664	8.285	23942	Mta2	chr19	+	NM_0118	P13941	protein_cc	AW55079	metastasi	Mta2
10131	Tut1	3.760	0.825	3.101	0.001	0.105	0.030	5.376	7.287	7.009	70044	Tut1	chr19	+	NM_1979	P22203	protein_cc	2700038E	terminal u	Tut1
10132	Eef1g	1.035	1.106	1.145	0.203	0.642	0.725	9.194	9.244	9.389	67160	Eef1g	chr19	+	NM_0260	P15102	protein_cc	2610301D	eukaryotic	Eef1g
10133	Ahnak	0.341	3.045	1.038	0.002	0.010	0.880	7.600	6.048	7.654	66395	Ahnak	chr19	+	NM_0096	P13251	protein_cc	DY6	AHNAK nu	Ahnak
10134	Scgb1a1	1.000	1.000	1.000	1.000	1.000	1.000	0.000	0.000	0.000	22287	Scgb1a1	chr19	-	NM_0116	P541	protein_cc	CC10| CC1	secretoglo	Scgb1a1
10135	Asrgl1	0.366	1.701	0.622	0.016	0.171	0.314	6.479	5.028	5.794	66514	Asrgl1	chr19	-	NM_0256	P11645	protein_cc	2410004D	asparagin	Asrgl1
10136	Stxbp3-ps	4.178	0.797	3.328	0.012	0.348	0.052	3.239	5.302	4.974	619371	Stxbp3-ps	chr19	-	NR_07355	.	misc_RNA	Munc18c(	syntaxin-b	Stxbp3-ps
10137	Incenp	1.900	1.395	2.651	0.000	0.172	0.155	3.761	4.687	5.167	16319	Incenp	chr19	-	NM_0166	P22953	protein_cc	2700067E	inner cent	Incenp
10138	Fth1	1.387	0.798	1.107	0.235	0.378	0.744	14.121	14.593	14.267	14319	Fth1	chr19	+	NR_07318	.	protein_cc	FHC| Fth|	ferritin he	Fth1
10139	Best1	2.225	3.013	6.702	0.290	0.170	0.294	0.001	1.155	2.746	24115	Best1	chr19	-	NM_0119	P26266	protein_cc	Bmd| mBe	bestrophi	Best1
10140	Rab3il1	0.094	2.280	0.215	0.012	0.100	0.143	4.640	1.232	2.420	74760	Rab3il1	chr19	+	NM_1445	P5972	protein_cc	1200014K	RAB3A int	Rab3il1
10141	Fads3	0.605	1.625	0.983	0.439	0.004	0.984	4.306	3.580	4.281	60527	Fads3	chr19	+	NM_0218	P8585	protein_cc	AI464531	fatty acid	Fads3
10142	Fads2	0.254	1.878	0.477	0.007	0.004	0.163	6.494	4.518	5.427	56473	Fads2	chr19	-	NM_0196	P10450	protein_cc	2900042N	fatty acid	Fads2
10143	Fads1	0.214	1.161	0.248	0.000	0.026	0.001	11.421	9.193	9.409	76267	Fads1	chr19	+	NM_1460	P9096	protein_cc	0710001O	fatty acid	Fads1
10144	Fen1	2.303	1.482	3.412	0.008	0.014	0.003	5.227	6.430	6.997	14156	Fen1	chr19	-	NM_0012	P1669	protein_cc	AW53843	flap struct	Fen1
10145	Tmem258	2.166	0.925	2.003	0.006	0.491	0.010	9.485	10.600	10.487	69038	Tmem258	chr19	+	NM_0269	P25693	protein_cc	1810006K	transmem	Tmem258
10146	Myrf	0.527	1.324	0.698	0.001	0.093	0.125	7.514	6.590	6.995	225908	Myrf	chr19	-	NM_0010	P18988	protein_cc	6030439E	myelin reg	Myrf
10147	Dagla	1.720	0.131	0.226	0.592	0.293	0.047	2.983	3.765	0.836	269060	Dagla	chr19	-	NM_1981	P8422	protein_cc	Nsddr	diacylglyc	Dagla
10148	Syt7	0.778	0.489	0.381	0.063	0.002	0.029	4.980	4.618	3.587	54525	Syt7	chr19	+	NM_1730	P26142	protein_cc	AI851541|	synaptota	Syt7
10149	Lrrc10b	1.005	1.405	1.412	0.000	0.182	0.423	0.000	0.008	0.498	278795	Lrrc10b	chr19	-	NM_0011	P26810	protein_cc	Gm705| S	leucine ric	Lrrc10b
10150	Ppp1r32	2.722	0.367	1.000	0.306	1.000	0.423	0.001	1.445	0.000	67752	Ppp1r32	chr19	-	NM_1336	P18716	protein_cc	4930579JC	protein ph	Ppp1r32
10151	Sdhaf2	1.135	0.871	0.989	0.000	0.001	0.649	8.213	8.395	8.196	66072	Sdhaf2	chr19	-	NM_0253	P113	protein_cc	0610038F	succinate	Sdhaf2
10152	Cpsf7	1.532	0.878	1.345	0.000	0.497	0.485	6.897	7.513	7.325	269061	Cpsf7	chr19	+	NM_0011	P22696	protein_cc	5730453I1	cleavage a	Cpsf7
10153	Tmem216	0.615	1.020	0.627	0.008	0.952	0.324	5.848	5.146	5.175	68642	Tmem216	chr19	-	NM_0012	P9750	protein_cc	1110017C	transmem	Tmem216
10154	Tmem138	2.013	0.807	1.624	0.008	0.079	0.226	3.641	4.650	4.340	72982	Tmem138	chr19	-	NM_0284	P3886	protein_cc	1700113I0	transmem	Tmem138
10155	Cyb561a3	0.833	0.731	0.609	0.537	0.217	0.347	8.319	8.055	7.604	225912	Cyb561a3	chr19	+	NM_0012	P8919	protein_cc	2310004G	cytochrom	Cyb561a3
10156	Tkfc	0.272	3.412	0.929	0.000	0.026	0.842	10.493	8.617	10.387	225913	Tkfc	chr19	-	NM_1454	P11185	protein_cc	BC021917	triokinase	Tkfc
10157	Ddb1	0.867	1.165	1.011	0.441	0.385	0.953	10.735	10.529	10.750	13194	Ddb1	chr19	+	NM_0157	P22088	protein_cc	127kDa| A	damage sp	Ddb1
10158	Vwce	0.051	4.972	0.252	0.001	0.027	0.053	6.373	2.069	4.383	71768	Vwce	chr19	+	NM_0279	P10745	protein_cc	1300015B	von Willet	Vwce
10159	4930524O	1.000	1.000	1.000	1.000	1.000	1.000	0.000	0.000	0.000	75096	4930524O	chr19	-	NR_04531	.	lncRNA	.	RIKEN cDN	4930524O
10160	Pga5	1.000	1.000	1.000	1.000	1.000	1.000	0.000	0.000	0.000	58803	Pga5	chr19	-	NM_0214	P11411	protein_cc	1110035E	pepsinoge	Pga5
10161	2210404E	1.000	1.000	1.000	1.000	1.000	1.000	0.000	0.000	0.000	72305	2210404E	chr19	+	NR_13116	.	lncRNA	.	RIKEN cDN	2210404E1
10162	Vps37c	1.714	0.844	1.447	0.010	0.175	0.069	4.825	5.603	5.358	107305	Vps37c	chr19	+	NM_1814	P1106	protein_cc	5730409F	vacuolar p	Vps37c
10163	Cd5	0.467	2.271	1.061	0.185	0.185	0.947	1.136	0.038	1.221	12507	Cd5	chr19	-	NM_0076	P1831	protein_cc	Ly-1| Ly-1	CD5 antig	Cd5
10164	A430093F	0.382	0.982	0.375	0.182	1.000	0.423	1.416	0.027	0.000	403202	A430093F	chr19	+	NR_02780	.	lncRNA	Gm674	RIKEN cDN	A430093F1
10165	Cd6	1.851	1.708	3.161	0.448	0.322	0.173	0.588	1.476	2.248	12511	Cd6	chr19	-	NM_0010	P12072	protein_cc	.	CD6 antig	Cd6
10166	Slc15a3	9.457	0.412	3.897	0.000	0.003	0.036	5.671	8.912	7.633	65221	Slc15a3	chr19	+	NM_0230	P26194	protein_cc	cI-1| Ci1	solute car	Slc15a3
10167	Tmem132	8.602	0.415	3.573	0.001	0.007	0.053	5.403	8.508	7.241	98170	Tmem132	chr19	-	NM_1338	P846	protein_cc	6720481D	transmem	Tmem132a
10168	Tmem109	0.578	1.216	0.703	0.001	0.414	0.300	8.533	7.744	8.026	68539	Tmem109	chr19	-	NM_1341	P21644	protein_cc	1110006I1	transmem	Tmem109
10169	Prpf19	1.656	0.922	1.526	0.000	0.384	0.137	8.643	9.371	9.253	28000	Prpf19	chr19	+	NM_1341	P15324	protein_cc	AA617263	pre-mRNA	Prpf19
10170	Zp1	1.000	1.000	1.000	1.000	1.000	1.000	0.000	0.000	0.000	22786	Zp1	chr19	-	NM_0095	P2422	protein_cc	.	zona pellu	Zp1
10171	Ptgdr2	2.836	1.352	3.833	0.001	0.310	0.177	4.434	5.938	6.373	14764	Ptgdr2	chr19	+	NM_0099	P4917	protein_cc	Crth2| Gp	prostaglan	Ptgdr2
10172	Ccdc86	3.741	1.016	3.800	0.003	0.951	0.173	6.732	8.635	8.658	108673	Ccdc86	chr19	-	NM_0237	P12698	protein_cc	4933411H	coiled-coil	Ccdc86
10173	Ms4a10	1.000	1.000	1.000	1.000	1.000	1.000	0.000	0.000	0.000	69826	Ms4a10	chr19	-	NM_0235	P15665	protein_cc	2010001N	membran	Ms4a10
10174	Ms4a15	0.716	0.995	0.712	0.182	1.000	0.423	0.490	0.008	0.000	545279	Ms4a15	chr19	-	NM_0010	P19592	protein_cc	E530011F	membran	Ms4a15
10175	Ms4a18	1.000	1.000	1.000	1.000	1.000	1.000	0.000	0.000	0.000	76002	Ms4a18	chr19	-	NM_0012	P25323	protein_cc	5033428B	membran	Ms4a18
10176	AW11201(	4.029	0.924	3.722	0.008	0.692	0.053	10.052	12.062	11.948	107350	AW11201(	chr19	-	NR_10236	.	lncRNA	.	expressed	AW11201(
10177	Ms4a8a	4.911	1.995	9.798	0.050	0.350	0.347	1.710	4.006	5.002	64381	Ms4a8a	chr19	-	NM_0224	P24661	protein_cc	2010004L	membran	Ms4a8a
10178	1700017D	1.010	1.885	1.904	0.000	0.182	0.423	0.000	0.015	0.929	69369	1700017D	chr19	-	NM_0270	P16117	protein_cc	.	RIKEN cDN	1700017D
10179	1700025F	1.000	1.000	1.000	1.000	1.000	1.000	0.000	0.000	0.000	69416	1700025F	chr19	-	NM_0270	P8366	protein_cc	.	RIKEN cDN	1700025F2

10180	Ms4a13	1.000	1.000	1.000	1.000	1.000	1.000	0.000	0.000	0.000	73466	Ms4a13	chr19	-	NM_1982	P14609	protein_cc	1700060E	membran	Ms4a13
10181	4930526L	1.000	1.000	1.000	1.000	1.000	1.000	0.000	0.000	0.000	75138	4930526L	chr19	+	NR_04531	.	lncRNA	.	RIKEN cDN	4930526L
10182	Ms4a1	1.779	0.117	0.208	0.293	0.059	0.181	2.716	3.547	0.450	12482	Ms4a1	chr19	-	NM_0076	P7498	protein_cc	AA960661	membran	Ms4a1
10183	Ms4a5	1.000	1.000	1.000	1.000	1.000	1.000	0.000	0.000	0.000	269063	Ms4a5	chr19	-	NM_1831	P14669	protein_cc	.	membran	Ms4a5
10184	Ms4a7	2.525	0.260	0.656	0.015	0.010	0.600	2.953	4.289	2.344	109225	Ms4a7	chr19	-	NM_0278	P17935	protein_cc	9130422I	membran	Ms4a7
10185	Ms4a4c	46.810	0.418	19.563	0.000	0.003	0.034	2.185	7.734	6.475	64380	Ms4a4c	chr19	+	NM_0294	P11697	protein_cc	5830413L	membran	Ms4a4c
10186	Ms4a4b	4.060	0.316	1.284	0.055	0.073	0.457	3.432	5.454	3.793	60361	Ms4a4b	chr19	+	NM_0217	P24714	protein_cc	AI463180	membran	Ms4a4b
10187	Ms4a6c	8.834	0.702	6.200	0.001	0.132	0.106	6.258	9.401	8.890	73656	Ms4a6c	chr19	+	NM_0285	P6850	protein_cc	2200009H	membran	Ms4a6c
10188	Gm8369	3.924	0.553	2.171	0.112	0.299	0.145	0.001	1.973	1.120	666926	Gm8369	chr19	+	NM_0011	P21279	protein_cc	EG666926	predicted	Gm8369
10189	Ms4a6b	6.591	0.691	4.552	0.000	0.190	0.236	4.811	7.531	6.997	69774	Ms4a6b	chr19	+	NM_0272	P17697	protein_cc	1810027D	membran	Ms4a6b
10190	Ms4a4d	2.714	0.520	1.412	0.000	0.000	0.031	5.350	6.791	5.848	66607	Ms4a4d	chr19	+	NM_0256	P23960	protein_cc	2210417J	membran	Ms4a4d
10191	Ms4a6d	30.140	0.431	12.982	0.000	0.000	0.014	4.748	9.662	8.446	68774	Ms4a6d	chr19	-	NM_0268	P22849	protein_cc	1110058E	membran	Ms4a6d
10192	Ms4a2	1.000	1.000	1.000	1.000	1.000	1.000	0.000	0.000	0.000	14126	Ms4a2	chr19	-	NM_0012	P18148	protein_cc	Fce1b | Fc	membran	Ms4a2
10193	Ms4a3	3.352	0.524	1.755	0.000	0.026	0.423	0.001	1.746	0.813	170813	Ms4a3	chr19	-	NM_1332	P9444	protein_cc	HTm4	membran	Ms4a3
10194	Oosp2	2.170	0.461	1.000	0.000	1.000	0.423	0.000	1.118	0.000	225922	Oosp2	chr19	-	NM_0010	P6176	protein_cc	Gm99 | Pl	oocyte se	Oosp2
10195	Oosp1	1.000	1.000	1.000	1.000	1.000	1.000	0.000	0.000	0.000	170834	Oosp1	chr19	-	NM_1333	P8725	protein_cc	AA536827	oocyte se	Oosp1
10196	Oosp3	1.000	1.000	1.000	1.000	1.000	1.000	0.000	0.000	0.000	225923	Oosp3	chr19	+	NM_0010	P23925	protein_cc	Gm97	oocyte se	Oosp3
10197	Gif	1.000	1.000	1.000	1.000	1.000	1.000	0.000	0.000	0.000	14603	Gif	chr19	+	NM_0081	P4488	protein_cc	AV073125	gastric int	Gif
10198	Mrpl16	1.092	1.274	1.390	0.433	0.073	0.067	9.198	9.325	9.674	94063	Mrpl16	chr19	+	NM_0256	P19996	protein_cc	2310039D	mitochon	Mrpl16
10199	Stx3	1.422	0.957	1.361	0.114	0.810	0.201	7.068	7.576	7.512	20908	Stx3	chr19	-	NM_0115	P1194	protein_cc	Syn-3	syntaxin 3	Stx3
10200	Olfr1417	1.000	1.000	1.000	1.000	1.000	1.000	0.000	0.000	0.000	258938	Olfr1417	chr19	-	NM_1469	P1147	protein_cc	MOR266-2	olfactory	Olfr1417
10201	Olfr1418	1.000	1.000	1.000	1.000	1.000	1.000	0.000	0.000	0.000	258227	Olfr1418	chr19	-	NM_0010	P18209	protein_cc	MOR266-5	olfactory	Olfr1418
10202	Olfr1419	1.000	1.000	1.000	1.000	1.000	1.000	0.000	0.000	0.000	257938	Olfr1419	chr19	-	NM_0010	P25965	protein_cc	MOR266-1	olfactory	Olfr1419
10203	Olfr1420	1.000	1.000	1.000	1.000	1.000	1.000	0.000	0.000	0.000	258405	Olfr1420	chr19	+	NM_1464	P17563	protein_cc	MOR266-4	olfactory	Olfr1420
10204	Patl1	1.510	0.771	1.164	0.000	0.064	0.528	7.601	8.196	7.821	225929	Patl1	chr19	+	NM_1726	P21880	protein_cc	AV312086	protein as	Patl1
10205	Osbp	1.601	0.854	1.367	0.224	0.643	0.472	7.063	7.742	7.514	76303	Osbp	chr19	+	NM_0010	P19118	protein_cc	1110018F	oxysterol	Osbp
10206	Olfr1423	1.000	1.000	1.000	1.000	1.000	1.000	0.000	0.000	0.000	258675	Olfr1423	chr19	-	NM_1466	P2069	protein_cc	MOR239-3	olfactory	Olfr1423
10207	Olfr1424	1.000	1.000	1.000	1.000	1.000	1.000	0.000	0.000	0.000	258676	Olfr1424	chr19	-	NM_1466	P2204	protein_cc	MOR239-2	olfactory	Olfr1424
10208	Olfr1425	1.000	1.000	1.000	1.000	1.000	1.000	0.000	0.000	0.000	258155	Olfr1425	chr19	-	NM_0010	P12353	protein_cc	MOR239-7	olfactory	Olfr1425
10209	Olfr1426	1.000	1.000	1.000	1.000	1.000	1.000	0.000	0.000	0.000	258805	Olfr1426	chr19	-	NM_1468	P10351	protein_cc	MOR239-1	olfactory	Olfr1426
10210	Olfr1427	1.000	1.000	1.000	1.000	1.000	1.000	0.000	0.000	0.000	258674	Olfr1427	chr19	-	NM_1466	P11941	protein_cc	MOR239-4	olfactory	Olfr1427
10211	Olfr1428	1.000	1.000	1.000	1.000	1.000	1.000	0.000	0.000	0.000	258673	Olfr1428	chr19	-	NM_1466	P12532	protein_cc	MOR239-5	olfactory	Olfr1428
10212	Olfr76	1.000	1.000	1.000	1.000	1.000	1.000	0.000	0.000	0.000	258677	Olfr76	chr19	-	NM_1466	P22314	protein_cc	MOR215-3	olfactory	Olfr76
10213	Olfr1431	0.716	0.995	0.712	0.182	1.000	0.423	0.490	0.008	0.000	258409	Olfr1431	chr19	+	NM_1464	P10344	protein_cc	MOR214-5	olfactory	Olfr1431
10214	Olfr1432	1.000	1.000	1.000	1.000	1.000	1.000	0.000	0.000	0.000	257961	Olfr1432	chr19	-	NM_0010	P16794	protein_cc	GA_x5J8B	olfactory	Olfr1432
10215	Olfr262	1.000	1.000	1.000	1.000	1.000	1.000	0.000	0.000	0.000	258683	Olfr262	chr19	-	NM_1466	P8455	protein_cc	MOR214-1	olfactory	Olfr262
10216	Olfr235	1.000	1.000	1.000	1.000	1.000	1.000	0.000	0.000	0.000	258681	Olfr235	chr19	+	NM_1466	P15947	protein_cc	MOR214-3	olfactory	Olfr235
10217	Olfr1434	1.000	1.000	1.000	1.000	1.000	1.000	0.000	0.000	0.000	258680	Olfr1434	chr19	+	NM_1466	P22495	protein_cc	MOR214-4	olfactory	Olfr1434
10218	Olfr1436	1.000	1.000	1.000	1.000	1.000	1.000	0.000	0.000	0.000	258682	Olfr1436	chr19	-	NM_1466	P11899	protein_cc	MOR214-2	olfactory	Olfr1436
10219	Olfr1437	1.000	1.000	1.000	1.000	1.000	1.000	0.000	0.000	0.000	258117	Olfr1437	chr19	-	NM_0010	P2387	protein_cc	MOR214-6	olfactory	Olfr1437
10220	Olfr1440	1.000	1.000	1.000	1.000	1.000	1.000	0.000	0.000	0.000	258679	Olfr1440	chr19	+	NM_1466	P5573	protein_cc	MOR215-1	olfactory	Olfr1440
10221	Olfr1441	1.000	1.000	1.000	1.000	1.000	1.000	0.000	0.000	0.000	258678	Olfr1441	chr19	+	NM_1466	P21851	protein_cc	MOR215-2	olfactory	Olfr1441
10222	Pfpl	1.005	1.405	1.412	0.000	0.182	0.423	0.000	0.008	0.498	56093	Pfpl	chr19	+	NM_0195	P7149	protein_cc	Epcs5 | Ep	pore form	Pfpl
10223	Mpeg1	5.378	0.565	3.038	0.000	0.007	0.082	7.289	9.716	8.892	17476	Mpeg1	chr19	+	NM_0108	P17406	protein_cc	Mpg-1 | M	macropha	Mpeg1
10224	Dtx4	0.526	0.913	0.481	0.050	0.416	0.215	7.735	6.809	6.679	207521	Dtx4	chr19	-	NM_1724	P10515	protein_cc	AI449438	deltex 4, E	Dtx4
10225	Fam111a	2.241	0.612	1.372	0.001	0.106	0.623	6.291	7.455	6.748	107373	Fam111a	chr19	+	NM_0266	P7329	protein_cc	4632417K	family wit	Fam111a
10226	A330040F	0.246	3.275	0.805	0.081	0.000	0.749	2.096	0.071	1.782	74333	A330040F	chr19	-	NR_01550	.	lncRNA	.	RIKEN cDN	A330040F
10227	Gm4952	0.024	0.753	0.018	0.002	0.494	0.042	10.357	4.967	4.558	240549	Gm4952	chr19	+	NM_0011	P15630	protein_cc	EG240549	predicted	Gm4952
10228	Glyat	0.166	3.021	0.502	0.000	0.010	0.054	11.285	8.694	10.289	107146	Glyat	chr19	+	NM_1459	P13593	protein_cc	A330009E	glycine-N-	Glyat
10229	Olfr1442	1.000	1.000	1.000	1.000	1.000	1.000	0.000	0.000	0.000	258692	Olfr1442	chr19	+	NM_1466	P11712	protein_cc	MOR202-9	olfactory	Olfr1442
10230	Olfr1443	0.504	0.990	0.499	0.181	1.000	0.423	1.002	0.015	0.000	258693	Olfr1443	chr19	+	NM_1466	P15089	protein_cc	MOR202-8	olfactory	Olfr1443
10231	Keg1	0.023	3.068	0.070	0.000	0.122	0.011	9.105	3.658	5.275	64697	Keg1	chr19	+	NM_0295	P6869	protein_cc	0610008P	kidney exp	Keg1
10232	Zfp91Cntf	0.690	1.416	0.976	0.007	0.056	0.914	9.784	9.248	9.749	.	Zfp91Cntf	chr19	-	NR_02409	.	.	.	.	Zfp91Cntf
10233	Cntf	0.723	1.214	0.878	0.044	0.087	0.435	3.887	3.419	3.700	12803	Cntf	chr19	-	NM_1707	P13853	protein_cc	AI429687	ciliary neu	Cntf
10234	Zfp91	0.771	1.312	1.011	0.099	0.084	0.769	9.642	9.267	9.658	109910	Zfp91	chr19	-	NM_0530	P23431	protein_cc	9130014I0	zinc finger	Zfp91
10235	Lpxn	1.428	1.174	1.677	0.481	0.631	0.291	3.781	4.295	4.527	107321	Lpxn	chr19	+	NM_1341	P15840	protein_cc	4933402K	leupaxin	Lpxn
10236	Olfr1444	1.000	1.000	1.000	1.000	1.000	1.000	0.000	0.000	0.000	258697	Olfr1444	chr19	+	NM_1467	P1043	protein_cc	MOR202-4	olfactory	Olfr1444
10237	Olfr1445	1.000	1.000	1.000	1.000	1.000	1.000	0.000	0.000	0.000	258694	Olfr1445	chr19	+	NM_1466	P5136	protein_cc	MOR202-7	olfactory	Olfr1445
10238	Olfr1446	1.000	1.000	1.000	1.000	1.000	1.000	0.000	0.000	0.000	258699	Olfr1446	chr19	+	NM_1467	P13808	protein_cc	MOR202-2	olfactory	Olfr1446
10239	Olfr1447	1.000	1.000	1.000	1.000	1.000	1.000	0.000	0.000	0.000	258698	Olfr1447	chr19	-	NM_1467	P25597	protein_cc	MOR202-3	olfactory	Olfr1447
10240	Gm5512	0.299	0.940	0.281	0.000	0.795	0.022	6.434	4.693	4.604	433224	Gm5512	chr19	+	NR_00289	.	misc_RNA	EG433224	predicted	Gm5512
10241	Olfr1448	1.000	1.000	1.000	1.000	1.000	1.000	0.000	0.000	0.000	258696	Olfr1448	chr19	-	NM_1467	P9415	protein_cc	MOR202-5	olfactory	Olfr1448
10242	Olfr1449	1.000	1.000	1.000	1.000	1.000	1.000	0.000	0.000	0.000	258300	Olfr1449	chr19	+	NM_1463	P21534	protein_cc	MOR202-3	olfactory	Olfr1449
10243	Olfr1450	1.000	1.000	1.000	1.000	1.000	1.000	0.000	0.000	0.000	258368	Olfr1450	chr19	+	NM_1463	P25420	protein_cc	MOR202-3	olfactory	Olfr1450
10244	Olfr1451	1.000	1.000	1.000	1.000	1.000	1.000	0.000	0.000	0.000	258700	Olfr1451	chr19	+	NM_1467	P17151	protein_cc	MOR202-1	olfactory	Olfr1451
10245	Olfr1453	1.000	1.000	1.000	1.000	1.000	1.000	0.000	0.000	0.000	258695	Olfr1453	chr19	-	NM_1467	P11161	protein_cc	MOR202-6	olfactory	Olfr1453
10246	Olfr1454	1.000	1.000	1.000	1.000	1.000	1.000	0.000	0.000	0.000	258687	Olfr1454	chr19	+	NM_1466	P22955	protein_cc	MOR202-1	olfactory	Olfr1454
10247	Olfr1457	1.000	1.000	1.000	1.000	1.000	1.000	0.000	0.000	0.000	258568	Olfr1457	chr19	-	NM_1465	P7534	protein_cc	MOR202-2	olfactory	Olfr1457
10248	Olfr1459	1.000	1.000	1.000	1.000	1.000	1.000	0.000	0.000	0.000	258684	Olfr1459	chr19	-	NM_1466	P25110	protein_cc	MOR202-1	olfactory	Olfr1459
10249	Olfr1461	1.000	1.000	1.000	1.000	1.000	1.000	0.000	0.000	0.000	258299	Olfr1461	chr19	+	NM_1463	P10254	protein_cc	MOR202-3	olfactory	Olfr1461
10250	Olfr1462	1.000	1.000	1.000	1.000	1.000	1.000	0.000	0.000	0.000	258688	Olfr1462	chr19	+	NM_1466	P15845	protein_cc	MOR202-1	olfactory	Olfr1462
10251	Olfr1463	1.000	1.000	1.000	1.000	1.000	1.000	0.000	0.000	0.000	258120	Olfr1463	chr19	-	NM_0010	P19144	protein_cc	MOR202-2	olfactory	Olfr1463
10252	Olfr1465	1.000	1.000	1.000	1.000	1.000	1.000	0.000	0.000	0.000	258121	Olfr1465	chr19	-	NM_0010	P5772	protein_cc	MOR202-2	olfactory	Olfr1465
10253	Olfr1466	1.000	1.000	1.000	1.000	1.000	1.000	0.000	0.000	0.000	258689	Olfr1466	chr19	+	NM_1466	P12136	protein_cc	MOR202-1	olfactory	Olfr1466
10254	Olfr1467	1.000	1.000	1.000	1.000	1.000	1.000	0.000	0.000	0.000	258686	Olfr1467	chr19	+	NM_1466	P25897	protein_cc	MOR202-1	olfactory	Olfr1467
10255	Olfr1469	1.000	1.000	1.000	1.000	1.000	1.000	0.000	0.000	0.000	258690	Olfr1469	chr19	+	NM_1466	P23826	protein_cc	MOR202-1	olfactory	Olfr1469
10256	Olfr1471	1.000	1.000	1.000	1.000	1.000	1.000	0.000	0.000	0.000	258231	Olfr1471	chr19	+	NM_2071	P18515	protein_cc	GA_x5J8B	olfactory	Olfr1471
10257	Olfr1472	1.000	1.000	1.000	1.000	1.000	1.000	0.000	0.000	0.000	258685	Olfr1472	chr19	-	NM_1466	P5568	protein_cc	MOR202-1	olfactory	Olfr1472
10258	Olfr1474	1.000	1.000	1.000	1.000	1.000	1.000	0.000	0.000	0.000	258123	Olfr1474	chr19	+	NM_0010	P6406	protein_cc	MOR202-2	olfactory	Olfr1474
10259	Olfr1475	1.000	1.000	1.000	1.000	1.000	1.000	0.000	0.000	0.000	258298	Olfr1475	chr19	-	NM_1463	P1824	protein_cc	MOR202-3	olfactory	Olfr1475
10260	Olfr1477	1.000	1.000	1.000	1.000	1.000	1.000	0.000	0.000	0.000	258691	Olfr1477	chr19	+	NM_1466	P6407	protein_cc	MOR202-1	olfactory	Olfr1477
10261	Olfr1480	1.000	1.000	1.000	1.000	1.000	1.000	0.000	0.000	0.000	404339	Olfr1480	chr19	+	NM_2075	P21720	protein_cc	MOR202-4	olfactory	Olfr1480
10262	Olfr1484	1.000	1.000	1.000	1.000	1.000	1.000	0.000	0.000	0.000	258288	Olfr1484	chr19	+	NM_1462	P66	protein_cc	MOR202-3	olfactory	Olfr1484
10263	Olfr1487	1.000	1.000	1.000	1.000	1.000	1.000	0.000	0.000	0.000	258629	Olfr1487	chr19	+	NM_1466	P19542	protein_cc	MOR202-1	olfactory	Olfr1487
10264	Olfr1489	1.000	1.000	1.000	1.000	1.000	1.000	0.000	0.000	0.000	258628	Olfr1489	chr19	+	NM_1466	P16606	protein_cc	MOR202-1	olfactory	Olfr1489
10265	Olfr1490	1.000	1.000	1.000	1.000	1.000	1.000	0.000	0.000	0.000	258098	Olfr1490	chr19	+	NM_0010	P19498	protein_cc	MOR266-6	olfactory	Olfr1490
10266	Olfr1491	1.000	1.000	1.000	1.000	1.000	1.000	0.000	0.000	0.000	258342	Olfr1491	chr19	+	NM_1463	P16083	protein_cc	MOR266-8	olfactory	Olfr1491
10267	Olfr1494	1.000	1.000	1.000	1.000	1.000	1.000	0.000	0.000	0.000	258992	Olfr1494	chr19	+	NM_1469	P15749	protein_cc	MOR266-8	olfactory	Olfr1494
10268	Olfr1495	1.000	1.000	1.000	1.000	1.000	1.000	0.000	0.000	0.000	258341	Olfr1495	chr19	+	NM_1463	P2704	protein_cc	MOR266-9	olfactory	Olfr1495
10269	Olfr1496	1.000	1.000	1.000	1.000	1.000	1.000	0.000	0.000	0.000	258991	Olfr1496	chr19	+	NM_1469	P24381	protein_cc	MOR127-1	olfactory	Olfr1496
10270	Olfr1497	1.000	1.000	1.000	1.000	1.000	1.000	0.000	0.000	0.000	258736	Olfr1497	chr19	-	NM_1467	P3109	protein_cc	MOR212-1	olfactory	Olfr1497

10271	Olfr1499	1.000	1.000	1.000	1.000	1.000	1.000	0.000	0.000	0.000	258792	Olfr1499	chr19	-	NM_1467	P16028	protein_cc	MOR211-2	olfactory r	Olfr1499
10272	Olfr1500	1.000	1.000	1.000	1.000	1.000	1.000	0.000	0.000	0.000	258097	Olfr1500	chr19	-	NM_0010	P23086	protein_cc	MOR212-4	olfactory r	Olfr1500
10273	Olfr1501	1.000	1.000	1.000	1.000	1.000	1.000	0.000	0.000	0.000	258626	Olfr1501	chr19	-	NM_1466	P21679	protein_cc	MOR212-3	olfactory r	Olfr1501
10274	Olfr1502	1.000	1.000	1.000	1.000	1.000	1.000	0.000	0.000	0.000	258793	Olfr1502	chr19	+	NM_1467	P10180	protein_cc	MOR211-1	olfactory r	Olfr1502
10275	Olfr1504	1.000	1.000	1.000	1.000	1.000	1.000	0.000	0.000	0.000	258627	Olfr1504	chr19	-	NM_1466	P11886	protein_cc	MOR212-2	olfactory r	Olfr1504
10276	Olfr1505	1.000	1.000	1.000	1.000	1.000	1.000	0.000	0.000	0.000	258151	Olfr1505	chr19	+	NM_0010	P14272	protein_cc	MOR211-4	olfactory r	Olfr1505
10277	Rnu6	1.000	1.000	1.000	1.000	1.000	1.000	0.000	0.000	0.000	19862	Rnu6	chr19	-	NR_00302	.	snRNA	.	U6 small r	Rnu6
10278	Tle4	1.362	0.976	1.329	0.037	0.875	0.330	6.924	7.369	7.334	21888	Tle4	chr19	-	NM_0013	P8509	protein_cc	5730411N	transducir	Tle4
10279	Psat1	9.370	1.693	15.860	0.001	0.002	0.005	3.306	6.534	7.293	107272	Psat1	chr19	-	NM_1774	P4692	protein_cc	D8Ertd814	phosphos	Psat1
10280	Cep78	2.141	0.803	1.718	0.033	0.440	0.515	3.367	4.466	4.148	208518	Cep78	chr19	-	NM_1980	P7972	protein_cc	5730599I0	centrosom	Cep78
10281	C130060C	1.000	1.000	1.000	1.000	1.000	1.000	0.000	0.000	0.000	10050265	C130060C	chr19	+	NR_04535	.	lncRNA	.	RIKEN cDN	C130060C
10282	Gnaq	0.816	1.020	0.832	0.070	0.594	0.280	8.612	8.319	8.347	14682	Gnaq	chr19	+	NM_0081	P18446	protein_cc	1110005L	guanine n	Gnaq
10283	E030024N	1.035	1.031	1.068	0.474	0.611	0.403	8.854	8.903	8.948	595139	E030024N	chr19	-	NR_03322	.	misc_RNA	9030417F	RIKEN cDN	E030024N
10284	Gna14	0.342	1.620	0.554	0.000	0.148	0.131	4.612	3.065	3.761	14675	Gna14	chr19	+	NM_0081	P68	protein_cc	AU023208	guanine n	Gna14
10285	Vps13a	0.799	0.903	0.721	0.003	0.527	0.259	6.743	6.419	6.272	271564	Vps13a	chr19	-	NM_1730	P21206	protein_cc	4930425F	vacuolar p	Vps13a
10286	Foxb2	1.000	1.000	1.000	1.000	1.000	1.000	0.000	0.000	0.000	14240	Foxb2	chr19	-	NM_0080	P13104	protein_cc	Fkh4	forkhead b	Foxb2
10287	Prune2	0.562	0.448	0.252	0.042	1.000	0.091	1.990	1.158	0.000	353211	Prune2	chr19	+	NM_1813	P10318	protein_cc	6330414G	prune hon	Prune2
10288	Gcnt1	0.719	1.469	1.055	0.251	0.518	0.952	3.533	3.056	3.611	14537	Gcnt1	chr19	-	NM_1734	P12878	protein_cc	5630400D	glucosami	Gcnt1
10289	Rfk	0.919	1.155	1.061	0.219	0.028	0.410	9.752	9.630	9.838	54391	Rfk	chr19	+	NM_0194	P71	protein_cc	0610038L	riboflavin	Rfk
10290	Pcsk5	2.183	0.874	1.908	0.005	0.343	0.060	7.240	8.367	8.173	18552	Pcsk5	chr19	-	NM_0011	P12866	protein_cc	b2b1549C	proprotei	Pcsk5
10291	Ostf1	1.422	1.044	1.484	0.017	0.630	0.045	8.599	9.107	9.169	20409	Ostf1	chr19	-	NM_0173	P16407	protein_cc	C78236 | S	osteoclast	Ostf1
10292	Nmrk1	1.730	1.695	2.932	0.000	0.040	0.098	6.273	7.064	7.825	225994	Nmrk1	chr19	+	NM_1454	P12366	protein_cc	AA408380	nicotinam	Nmrk1
10293	2410127L	0.689	1.136	0.782	0.000	0.034	0.037	7.235	6.696	6.880	.	2410127L	chr19	+	NM_0261	P334	.	.	.	2410127L
10294	D030056L	1.058	0.603	0.638	0.711	0.036	0.059	6.432	6.514	5.784	225995	D030056L	chr19	+	NM_1776	P17159	protein_cc	.	RIKEN cDN	D030056L
10295	Trpm6	0.693	1.111	0.770	0.164	0.834	0.726	2.035	1.507	1.658	225997	Trpm6	chr19	+	NM_1534	P5211	protein_cc	CHAK2 | TI	transient r	Trpm6
10296	Rorb	1.005	1.405	1.412	0.000	0.182	0.423	0.000	0.008	0.498	225998	Rorb	chr19	-	NM_0012	P12570	protein_cc	hstp| Nr1	RAR-relate	Rorb
10297	Anxa1	1.149	1.677	1.926	0.747	0.022	0.299	4.616	4.816	5.561	16952	Anxa1	chr19	-	NM_0107	P19988	protein_cc	Anx-1 | An	annexin A	Anxa1
10298	1500015L	1.000	1.000	1.000	1.000	1.000	1.000	0.000	0.000	0.000	68994	1500015L	chr19	+	NR_04581	.	lncRNA	.	RIKEN cDN	1500015L
10299	E030003E	0.457	1.358	0.620	0.183	0.195	0.578	1.158	0.027	0.468	320092	E030003E	chr19	+	NR_01550	.	lncRNA	1700127G	RIKEN cDN	E030003E
10300	C730002L	0.213	0.964	0.206	0.028	1.000	0.172	2.281	0.052	0.000	320155	C730002L	chr19	+	NR_04577	.	lncRNA	.	RIKEN cDN	C730002L
10301	Aldh1a1	0.362	0.823	0.298	0.005	0.365	0.048	13.000	11.533	11.251	11668	Aldh1a1	chr19	+	NM_0134	P3729	protein_cc	Ahd-2 | Ah	aldehyde c	Aldh1a1
10302	Aldh1a7	0.625	1.171	0.732	0.016	0.116	0.185	10.490	9.812	10.040	26358	Aldh1a7	chr19	-	NM_0119	P8822	protein_cc	Ahd2-like |	aldehyde c	Aldh1a7
10303	Tmc1	0.292	0.977	0.285	0.181	1.000	0.423	1.810	0.034	0.000	13409	Tmc1	chr19	-	NM_0289	P6294	protein_cc	4933416G	transmem	Tmc1
10304	4930554I0	1.000	1.000	1.000	1.000	1.000	1.000	0.000	0.000	0.000	75354	4930554I0	chr19	+	NR_12645	.	lncRNA	.	RIKEN cDN	4930554I0
10305	Zfand5	1.049	0.762	0.799	0.732	0.160	0.529	9.273	9.341	8.949	22682	Zfand5	chr19	+	NM_0095	P20906	protein_cc	2310057A	zinc finger	Zfand5
10306	Gda	0.970	1.466	1.422	0.736	0.080	0.278	9.054	9.011	9.563	14544	Gda	chr19	-	NM_0102	P7550	protein_cc	AU015411	guanine d	Gda
10307	1110059E	1.752	1.024	1.794	0.003	0.923	0.338	8.250	9.059	9.093	66206	1110059E	chr19	-	NM_0254	P21754	protein_cc	.	RIKEN cDN	1110059E
10308	Abhd17b	1.407	1.224	1.722	0.004	0.221	0.184	8.809	9.301	9.593	226016	Abhd17b	chr19	+	NM_1460	P8500	protein_cc	5730446C	abhydrola	Abhd17b
10309	Tmem2	3.013	0.940	2.831	0.004	0.673	0.036	9.041	10.632	10.542	83921	Tmem2	chr19	+	NM_0010	P22066	protein_cc	.	transmem	Tmem2
10310	Trpm3	1.000	1.000	1.000	1.000	1.000	1.000	0.000	0.000	0.000	226025	Trpm3	chr19	+	NM_0010	P3823	protein_cc	6330504F	transient r	Trpm3
10311	Klf9	0.300	1.346	0.404	0.001	0.001	0.035	12.878	11.141	11.569	16601	Klf9	chr19	+	NM_0106	P8849	protein_cc	2310051E	Kruppel-lil	Klf9
10312	Smc5	1.905	0.662	1.262	0.000	0.018	0.455	6.836	7.766	7.171	226026	Smc5	chr19	+	NM_1538	P3845	protein_cc	mKIAA059	structural	Smc5
10313	Mamdc2	0.315	0.516	0.163	0.003	0.215	0.017	3.472	1.805	0.851	71738	Mamdc2	chr19	-	NM_1748	P12594	protein_cc	1200015L	MAM dom	Mamdc2
10314	1700028P	1.572	0.636	1.000	0.304	1.000	0.423	0.000	0.653	0.000	67483	1700028P	chr19	-	NM_0261	P17471	protein_cc	AV209940	RIKEN cDN	1700028P
10315	Ptar1	1.867	1.441	2.690	0.329	0.432	0.227	3.745	4.645	5.172	72351	Ptar1	chr19	+	NM_0282	P20967	protein_cc	1700084D	protein pr	Ptar1
10316	Apba1	0.356	3.863	1.377	0.089	0.140	0.722	3.034	1.545	3.495	319924	Apba1	chr19	+	NM_1770	P10757	protein_cc	6430513E	amyloid be	Apba1
10317	Fam189a2	0.114	1.600	0.182	0.036	0.000	0.220	3.227	0.093	0.771	381217	Fam189a2	chr19	-	NM_0011	P20298	protein_cc	AI414343 |	family wit	Fam189a2
10318	Tjp2	1.619	1.055	1.707	0.000	0.005	0.002	7.935	8.630	8.707	21873	Tjp2	chr19	-	NM_0011	P6638	protein_cc	ZO-2	tight junct	Tjp2
10319	Fxn	0.481	1.971	0.947	0.024	0.007	0.821	7.218	6.161	7.140	14297	Fxn	chr19	-	NM_0080	P12229	protein_cc	FA| FARR|	frataxin	Fxn
10320	Pip5k1b	1.015	2.326	2.360	0.000	0.182	0.423	0.000	0.021	1.239	18719	Pip5k1b	chr19	-	NM_0088	P23484	protein_cc	PI4P5K-I[b	phosphati	Pip5k1b
10321	Fam122a	1.760	0.786	1.383	0.009	0.037	0.192	7.345	8.161	7.813	68034	Fam122a	chr19	-	NM_0265	P7419	protein_cc	2410124L	family wit	Fam122a
10322	Tmem252	15.690	0.780	12.241	0.009	0.458	0.137	3.931	7.903	7.545	226040	Tmem252	chr19	+	NM_1831	P16632	protein_cc	E030010A	transmem	Tmem252
10323	Pgm5	0.951	1.376	1.309	0.845	0.002	0.506	4.692	4.620	5.081	226041	Pgm5	chr19	-	NM_1750	P3345	protein_cc	4833423B	phosphog	Pgm5
10324	Foxd4	0.797	0.890	0.710	0.634	0.769	0.647	1.003	0.676	0.508	14237	Foxd4	chr19	-	NM_0080	P13047	protein_cc	Fkh2 | FRE	forkhead b	Foxd4
10325	Cbwd1	1.475	1.105	1.630	0.172	0.681	0.206	5.339	5.899	6.043	226043	Cbwd1	chr19	-	NR_03374	.	protein_cc	AV349248	COBW dor	Cbwd1
10326	Snora19	0.689	1.873	1.291	0.001	0.000	0.046	3.100	2.562	3.468	10021743	Snora19	chr19	-	NR_03404	.	snoRNA	MBI-51	small nucl	Snora19
10327	Gm38437	0.863	1.530	1.320	0.398	0.016	0.169	2.239	2.026	2.639	10246511	Gm38437	chr19	-	NR_10574	.	miRNA	Mir3084-2	predicted	Gm38437
10328	Dock8	0.166	0.706	0.117	0.000	0.211	0.012	6.669	4.078	3.577	76088	Dock8	chr19	+	NM_0287	P14920	protein_cc	1200017A	dedicator	Dock8
10329	Kank1	0.255	1.597	0.407	0.000	0.044	0.013	9.256	7.285	7.961	107351	Kank1	chr19	+	NM_1814	P19611	protein_cc	A930031B	KN motif a	Kank1
10330	Dmrt1	0.339	0.613	0.208	0.059	1.000	0.169	2.268	0.706	0.000	50796	Dmrt1	chr19	+	NM_0158	P25156	protein_cc	.	doublesex	Dmrt1
10331	Dmrt3	1.000	1.000	1.000	1.000	1.000	1.000	0.000	0.000	0.000	240590	Dmrt3	chr19	+	NM_1773	P9523	protein_cc	.	doublesex	Dmrt3
10332	2610016A	0.549	0.837	0.459	0.437	0.765	0.560	2.004	1.138	0.881	10050307	2610016A	chr19	+	NR_04534	.	lncRNA	2610317C	RIKEN cDN	2610016A
10333	Dmrt2	5.705	0.319	1.821	0.010	0.027	0.423	0.001	2.513	0.866	226049	Dmrt2	chr19	+	NM_1458	P1286	protein_cc	Terra	doublesex	Dmrt2
10334	Smarca2	0.161	1.323	0.213	0.000	0.453	0.018	10.924	8.288	8.691	67155	Smarca2	chr19	+	NM_0114	P5039	protein_cc	2610209L	SWI/SNF r	Smarca2
10335	4931403E	0.598	0.495	0.296	0.294	0.393	0.065	3.293	2.552	1.537	70942	4931403E	chr19	-	NR_04530	.	lncRNA	.	RIKEN cDN	4931403E
10336	Gm815	1.000	1.000	1.000	1.000	1.000	1.000	0.000	0.000	0.000	329047	Gm815	chr19	+	NM_0010	P2439	protein_cc	.	predicted	Gm815
10337	Vldlr	0.057	1.127	0.064	0.000	0.635	0.020	7.934	3.796	3.968	22359	Vldlr	chr19	+	NM_0011	P21118	protein_cc	AA408956	very low d	Vldlr
10338	Kcnv2	1.000	1.000	1.000	1.000	1.000	1.000	0.000	0.000	0.000	240595	Kcnv2	chr19	+	NM_1831	P17736	protein_cc	KV11.1	potassium	Kcnv2
10339	D19Bwg13	1.046	0.786	0.822	0.553	0.012	0.286	8.750	8.815	8.468	.	D19Bwg13	chr19	-	NR_13194	.	.	.	.	D19Bwg13
10340	C030016D	3.341	0.421	1.406	0.000	0.001	0.423	0.001	1.741	0.492	107372	C030016D	chr19	+	NR_02798	.	lncRNA	AW41334	RIKEN cDN	C030016D
10341	Rfx3	0.532	1.994	1.061	0.040	0.001	0.820	4.561	3.650	4.646	19726	Rfx3	chr19	-	NM_0011	P24391	protein_cc	C230093O	regulatory	Rfx3
10342	Glis3	7.840	0.346	2.711	0.065	0.136	0.293	1.450	4.421	2.889	226075	Glis3	chr19	-	NM_1754	P13431	protein_cc	4833409N	GLIS famil	Glis3
10343	D930032P	1.000	1.000	1.000	1.000	1.000	1.000	0.000	0.000	0.000	320176	D930032P	chr19	+	NR_04533	.	lncRNA	.	RIKEN cDN	D930032P
10344	Slc1a1	8.146	0.755	6.149	0.000	0.561	0.423	0.002	3.028	2.622	20510	Slc1a1	chr19	+	NM_0091	P22949	protein_cc	D130048G	solute car	Slc1a1
10345	4430402I1	0.048	1.623	0.077	0.000	0.229	0.022	4.537	0.143	0.841	381218	4430402I1	chr19	-	NM_1986	P19940	protein_cc	AW12261	RIKEN cDN	4430402I1
10346	Ppapdc2	0.638	1.527	0.975	0.030	0.039	0.922	7.370	6.723	7.333	.	Ppapdc2	chr19	+	NM_0289	P7768	.	.	.	Ppapdc2
10347	Cdc37l1	0.858	0.930	0.798	0.328	0.669	0.057	9.879	9.658	9.553	67072	Cdc37l1	chr19	+	NM_0013	P19771	protein_cc	2700033A	cell divisio	Cdc37l1
10348	Ak3	0.404	1.365	0.551	0.001	0.030	0.025	11.601	10.293	10.742	56248	Ak3	chr19	-	NM_0212	P22930	protein_cc	AA407498	adenylate	Ak3
10349	1700018L	1.023	1.333	1.364	0.956	0.501	0.687	4.841	4.873	5.288	67329	1700018L	chr19	+	NR_02836	.	lncRNA	.	RIKEN cDN	1700018L
10350	Gm9895	1.327	1.695	2.248	0.453	0.270	0.334	4.028	4.436	5.197	10050333	Gm9895	chr19	+	NR_04568	.	lncRNA	ENSMUSG	predicted	Gm9895
10351	Rcl1	0.773	3.772	2.914	0.302	0.117	0.391	12.114	11.742	13.657	59028	Rcl1	chr19	+	NM_0215	P21338	protein_cc	2310040A	RNA termi	Rcl1
10352	Jak2	2.780	0.569	1.582	0.001	0.001	0.254	6.443	7.918	7.104	16452	Jak2	chr19	+	NM_0010	P21942	protein_cc	Fd17	Janus kina	Jak2
10353	Insl6	0.728	0.988	0.719	0.105	0.939	0.221	3.278	2.820	2.802	27356	Insl6	chr19	-	NM_0137	P13223	protein_cc	RIF1	insulin-like	Insl6
10354	Rln1	1.572	0.636	1.000	0.304	1.000	0.423	0.000	0.653	0.000	19773	Rln1	chr19	-	NM_0112	P9354	protein_cc	Rln | rlx	relaxin 1	Rln1
10355	Plgrkt	2.382	0.614	1.461	0.006	0.010	0.475	4.699	5.951	5.246	67759	Plgrkt	chr19	-	NM_0263	P26348	protein_cc	1110007H	plasminog	Plgrkt
10356	Cd274	32.013	0.441	14.130	0.000	0.012	0.165	4.526	9.527	8.347	60533	Cd274	chr19	+	NM_0218	P19758	protein_cc	A530045L	CD274 ant	Cd274
10357	Pdcd1lg2	1.589	1.186	1.885	0.298	0.707	0.423	0.000	0.669	0.915	58205	Pdcd1lg2	chr19	+	NM_0213	P1730	protein_cc	B7-DC | Bt	programm	Pdcd1lg2
10358	A930007I	3.908	0.256	0.999	0.003	1.000	0.423	0.001	1.967	0.000	77779	A930007I	chr19	-	NR_01556	.	lncRNA	.	RIKEN cDN	A930007I
10359	Ric1	1.333	0.722	0.962	0.007	0.026	0.870	5.908	6.323	5.853	226089	Ric1	chr19	+	NM_0010	P22160	protein_cc	C030046E	RAB6A GE	Ric1
10360	Ermp1	0.631	1.662	1.049	0.090	0.116	0.919	6.863	6.199	6.933	226090	Ermp1	chr19	-	NM_0010	P9144	protein_cc	D19Ertd41	endoplasn	Ermp1
10361	Mlana	1.010	1.885	1.904	0.000	0.182	0.423	0.000	0.015	0.929	77836	Mlana	chr19	+	NM_0299	P26817	protein_cc	A930034P	melan-A	Mlana

10362	9930021J(	0.862	0.932	0.804	0.011	0.114	0.009	8.254	8.039	7.939	240613	9930021J(	chr19	-	NM_1728	P11146	protein_cc	ENSMUSG	RIKEN cDN	9930021J0
10363	Ranbp6	1.194	1.125	1.343	0.233	0.445	0.368	5.859	6.115	6.284	240614	Ranbp6	chr19	-	NM_1777	P18578	protein_cc	C630001B	RAN bindi	Ranbp6
10364	Il33	78.661	1.274	100.221	0.001	0.507	0.167	2.765	9.062	9.412	77125	Il33	chr19	+	NM_1337	P25593	protein_cc	9230117N	interleuki	Il33
10365	Trpd52l3	1.000	1.000	1.000	1.000	1.000	1.000	0.000	0.000	0.000	66745	Trpd52l3	chr19	+	NM_0257	P20935	protein_cc	4931412G	tumor pro	Trpd52l3
10366	Uhrf2	1.520	0.493	0.749	0.002	0.000	0.115	7.587	8.192	7.170	109113	Uhrf2	chr19	+	NM_1448	P22987	protein_cc	2310065A	ubiquitin-	Uhrf2
10367	Gldc	0.146	1.061	0.155	0.000	0.699	0.006	9.240	6.463	6.548	104174	Gldc	chr19	-	NM_1385	P26179	protein_cc	D030049L	glycine de	Gldc
10368	Mbl2	0.551	0.646	0.355	0.000	0.012	0.003	13.196	12.335	11.704	17195	Mbl2	chr19	+	NM_0107	P20322	protein_cc	L-MBP| M	mannose-	Mbl2
10369	Ppp1r2-ps	1.023	1.007	1.030	0.878	0.980	0.945	3.011	3.044	3.054	546723	Ppp1r2-ps	chr19	-	NR_00365	.	misc_RNA	EG546723	protein ph	Ppp1r2-ps
10370	Dkk1	1.000	1.000	1.000	1.000	1.000	1.000	0.000	0.000	0.000	13380	Dkk1	chr19	-	NM_0100	P4376	protein_cc	mdkk-1	dickkopf	Dkk1
10371	Prkg1	0.220	0.767	0.169	0.013	0.742	0.074	3.973	1.790	1.408	19091	Prkg1	chr19	-	NM_0111	P25601	protein_cc	AW12541(	protein ki	Prkg1
10372	Gm6642	1.019	2.633	2.682	0.000	0.030	0.179	0.000	0.027	1.424	625995	Gm6642	chr19	-	NR_03364	.	misc_RNA	EG625995	predicted	Gm6642
10373	Cstf2t	1.224	0.779	0.953	0.191	0.186	0.829	7.752	8.043	7.683	83410	Cstf2t	chr19	+	NM_0312	P7691	protein_cc	64kDa| C7	cleavage s	Cstf2t
10374	8430431K	1.005	1.439	1.446	0.000	0.182	0.423	0.000	0.008	0.532	78103	8430431K	chr19	-	NR_00284	.	lncRNA	.	RIKEN cDN	8430431K
10375	A1cf	0.074	0.697	0.051	0.000	0.195	0.003	8.655	4.883	4.363	69865	A1cf	chr19	+	NM_0010	P1654	protein_cc	1810073H	APOBEC1	A1cf
10376	Asah2	0.470	0.649	0.305	0.016	0.145	0.058	7.170	6.079	5.455	54447	Asah2	chr19	-	NM_0188	P12058	protein_cc	AI585898	N-acylsph	Asah2
10377	Sgms1	3.449	0.602	2.075	0.000	0.035	0.219	7.864	9.651	8.917	208449	Sgms1	chr19	-	NM_0011	P6594	protein_cc	95300580	sphingom	Sgms1
10378	2700046G	0.430	1.891	0.813	0.008	0.000	0.437	5.005	3.786	4.706	67188	2700046G	chr19	+	NR_03319	.	lncRNA	.	RIKEN cDN	2700046G
10379	Minpp1	0.673	1.113	0.749	0.105	0.415	0.409	6.793	6.221	6.376	17330	Minpp1	chr19	+	NM_0107	P8295	protein_cc	AA408516	multiple in	Minpp1
10380	Papss2	0.138	0.528	0.073	0.000	0.008	0.002	10.889	8.031	7.111	23972	Papss2	chr19	+	NM_0012	P26370	protein_cc	1810018P	3'-phosph	Papss2
10381	Atad1	1.263	0.887	1.120	0.357	0.684	0.760	9.675	10.012	9.839	67979	Atad1	chr19	-	NM_0264	P10713	protein_cc	4921525H	ATPase fa	Atad1
10382	Pten	0.632	0.984	0.622	0.001	0.379	0.033	10.189	9.528	9.505	19211	Pten	chr19	+	NM_0089	P26432	protein_cc	23100350	phosphata	Pten
10383	Rnls	1.307	0.500	0.653	0.576	0.036	0.701	3.103	3.490	2.489	67795	Rnls	chr19	-	NM_0011	P17204	protein_cc	6530404N	renalase,	Rnls
10384	Lipo1	1.216	0.454	0.552	0.665	0.196	0.270	3.542	3.824	2.686	329055	Lipo1	chr19	-	NM_0010	P10488	protein_cc	EG329055	lipase, me	Lipo1
10385	Lipf	1.000	1.000	1.000	1.000	1.000	1.000	0.000	0.000	0.000	67717	Lipf	chr19	+	NM_0263	P12381	protein_cc	2310051B	lipase, gas	Lipf
10386	Lipk	1.000	1.000	1.000	1.000	1.000	1.000	0.000	0.000	0.000	240633	Lipk	chr19	+	NM_1728	P3350	protein_cc	9930115F	lipase, fan	Lipk
10387	Lipn	1.000	1.000	1.000	1.000	1.000	1.000	0.000	0.000	0.000	70166	Lipn	chr19	+	NM_0273	P24595	protein_cc	2210418G	lipase, fan	Lipn
10388	Lipm	1.000	1.000	1.000	1.000	1.000	1.000	0.000	0.000	0.000	78753	Lipm	chr19	+	NM_0239	P7266	protein_cc	4632427C	lipase, fan	Lipm
10389	Ankrd22	12.296	0.228	2.809	0.114	0.168	0.235	0.002	3.622	1.492	52024	Ankrd22	chr19	-	NM_0242	P875	protein_cc	5430429D	ankyrin re	Ankrd22
10390	Stambpl1	0.455	0.883	0.401	0.153	0.680	0.338	3.840	2.704	2.524	76630	Stambpl1	chr19	+	NM_0296	P124	protein_cc	1700095N	STAM bind	Stambpl1
10391	Acta2	0.182	5.835	1.062	0.001	0.023	0.897	6.051	3.593	6.138	11475	Acta2	chr19	-	NM_0073	P4612	protein_cc	0610041G	actin, alph	Acta2
10392	Fas	5.904	0.486	2.869	0.002	0.011	0.016	7.212	9.774	8.733	14102	Fas	chr19	+	NM_0079	P7087	protein_cc	AI196731|	Fas (TNF r	Fas
10393	Ch25h	177.536	0.227	40.243	0.005	0.013	0.024	1.627	9.099	6.957	12642	Ch25h	chr19	-	NM_0098	P21072	protein_cc	AI462618|	cholestero	Ch25h
10394	Lipa	0.116	1.543	0.178	0.000	0.069	0.004	11.103	7.991	8.617	16889	Lipa	chr19	-	NM_0011	P17298	protein_cc	AA960673	lysosomal	Lipa
10395	Ifit2	17.057	0.239	4.078	0.000	0.003	0.350	4.369	8.461	6.397	15958	Ifit2	chr19	+	NM_0083	P13767	protein_cc	AV302338	interferon	Ifit2
10396	Ifit3	9.048	0.376	3.407	0.000	0.005	0.270	8.174	11.352	9.942	15959	Ifit3	chr19	+	NM_0105	P3976	protein_cc	Ifi49| P49	interferon	Ifit3
10397	Ifit1bl1	1.145	1.378	1.578	0.745	0.511	0.662	2.623	2.819	3.281	667373	Ifit1bl1	chr19	-	NM_0011	P11852	protein_cc	Gm14446	interferon	Ifit1bl1
10398	Ifit3b	8.711	0.353	3.077	0.000	0.007	0.347	6.416	9.539	8.038	667370	Ifit3b	chr19	+	NM_0010	P18404	protein_cc	I830012O	interferon	Ifit3b
10399	Ifit1bl2	15.409	0.252	3.880	0.006	0.016	0.216	1.841	5.787	3.797	112419	Ifit1bl2	chr19	-	NM_0532	P5089	protein_cc	2010002N	interferon	Ifit1bl2
10400	Ifit1	9.221	0.291	2.685	0.000	0.001	0.320	8.462	11.667	9.887	15957	Ifit1	chr19	+	NM_0083	P5664	protein_cc	Ifi56| ISG5	interferon	Ifit1
10401	Slc16a12	0.016	5.253	0.086	0.001	0.004	0.043	8.181	2.251	4.644	240638	Slc16a12	chr19	-	NM_1728	P21105	protein_cc	3230401A	solute car	Slc16a12
10402	Pank1	0.043	4.407	0.188	0.000	0.016	0.007	12.357	7.803	9.942	75735	Pank1	chr19	-	NM_0237	P20856	protein_cc	Pank| Pan	pantother	Pank1
10403	Kif20b	1.348	0.608	0.819	0.490	0.144	0.817	3.464	3.895	3.176	240641	Kif20b	chr19	+	NM_1830	P17769	protein_cc	33cex| B1	kinesin fa	Kif20b
10404	Htr7	2.754	0.513	1.413	0.020	0.065	0.423	0.001	1.462	0.499	15566	Htr7	chr19	-	NM_0083	P5253	protein_cc	5-HT7	5-hydroxy	Htr7
10405	Rpp30	1.734	1.209	2.096	0.025	0.103	0.036	5.662	6.456	6.730	54364	Rpp30	chr19	+	NM_0194	P2035	protein_cc	Rnasep2|	ribonuclea	Rpp30
10406	Ankrd1	1.018	1.816	1.849	0.961	0.068	0.062	2.912	2.938	3.799	107765	Ankrd1	chr19	-	NM_0134	P2130	protein_cc	Alrp| CAR	ankyrin re	Ankrd1
10407	Pcgf5	3.227	0.824	2.660	0.133	0.725	0.404	5.271	6.961	6.683	76073	Pcgf5	chr19	+	NM_0295	P19396	protein_cc	0610009F	polycomb	Pcgf5
10408	Hectd2	0.169	0.283	0.046	0.001	0.005	0.027	9.264	6.696	4.875	226098	Hectd2	chr19	+	NM_0011	P10706	protein_cc	4921524L(	HECT dom	Hectd2
10409	1500017E	0.171	0.274	0.047	0.001	0.003	0.027	9.270	6.722	4.854	668215	1500017E	chr19	-	NR_03351	.	lncRNA	668215	RIKEN cDN	1500017E
10410	Ppp1r3c	0.579	0.302	0.175	0.003	0.001	0.008	8.533	7.745	6.017	53412	Ppp1r3c	chr19	-	NM_0168	P13355	protein_cc	Ppp1r5| P	protein ph	Ppp1r3c
10411	Tnks2	1.363	0.940	1.282	0.004	0.430	0.137	8.992	9.439	9.351	74493	Tnks2	chr19	+	NM_0011	P4430	protein_cc	5430432P	tankyrase,	Tnks2
10412	Fgfbp3	1.092	0.781	0.853	0.904	0.736	0.898	2.260	2.387	2.030	72514	Fgfbp3	chr19	-	NM_0282	P16753	protein_cc	2610306H	fibroblast	Fgfbp3
10413	Btaf1	1.706	0.943	1.609	0.001	0.000	0.051	7.440	8.210	8.126	107182	Btaf1	chr19	+	NM_0010	P1658	protein_cc	AI414500|	B-TFIID TA	Btaf1
10414	Cpeb3	0.520	1.218	0.634	0.000	0.083	0.054	7.664	6.721	7.005	208922	Cpeb3	chr19	-	NM_1983	P3288	protein_cc	48314440	cytoplasm	Cpeb3
10415	A330032B	2.252	0.461	1.037	0.136	0.094	0.961	1.500	2.672	1.553	319931	A330032B	chr19	-	NR_04532	.	lncRNA	.	RIKEN cDN	A330032B
10416	March5	1.607	0.991	1.593	0.000	0.956	0.266	9.775	10.459	10.447	69104	March5	chr19	+	NM_0273	P19981	protein_cc	1810015H	membran	March5
10417	4931408D	0.017	7.262	0.121	0.000	0.047	0.014	7.489	1.585	4.445	77059	4931408D	chr19	-	NR_04029	.	lncRNA	.	RIKEN cDN	4931408D
10418	Ide	0.522	1.012	0.528	0.006	0.950	0.150	8.897	7.958	7.976	15925	Ide	chr19	-	NM_0311	P26031	protein_cc	1300012G	insulin de	Ide
10419	Kif11	2.046	0.838	1.716	0.022	0.499	0.343	1.773	2.806	2.551	16551	Kif11	chr19	+	NM_0106	P12647	protein_cc	Eg5| Kif8|	kinesin fa	Kif11
10420	Hhex	0.141	2.335	0.328	0.000	0.119	0.049	9.985	7.156	8.379	15242	Hhex	chr19	+	NM_0082	P11478	protein_cc	Hex| Hex1	hematopo	Hhex
10421	1700122C	1.000	1.000	1.000	1.000	1.000	1.000	0.000	0.000	0.000	78640	1700122C	chr19	+	NR_13100	.	lncRNA	.	RIKEN cDN	1700122C
10422	Exoc6	1.535	0.991	1.522	0.453	0.987	0.582	5.668	6.286	6.273	107371	Exoc6	chr19	+	NM_1753	P1385	protein_cc	4833405E	exocyst cc	Exoc6
10423	Cyp26c1	0.716	0.995	0.712	0.182	1.000	0.423	0.490	0.008	0.000	546726	Cyp26c1	chr19	+	NM_0011	P12253	protein_cc	EG546726	cytochron	Cyp26c1
10424	Cyp26a1	0.020	0.392	0.008	0.000	0.015	0.009	7.709	2.071	0.721	13082	Cyp26a1	chr19	+	NM_0078	P17427	protein_cc	Cyp26| P4	cytochron	Cyp26a1
10425	Myof	1.351	1.232	1.665	0.026	0.176	0.154	4.876	5.310	5.611	226101	Myof	chr19	-	NM_0013	P20075	protein_cc	2310004N	myoferlin	Myof
10426	Cep55	3.149	0.087	0.275	0.458	0.326	0.480	2.591	4.246	0.729	74107	Cep55	chr19	+	NM_0282	P10090	protein_cc	12000080	centrosom	Cep55
10427	Ffar4	3.152	0.269	0.847	0.021	0.004	0.863	1.966	3.622	1.727	107221	Ffar4	chr19	+	NM_1817	P23701	protein_cc	AI552415|	free fatty	Ffar4
10428	Rbp4	0.204	0.933	0.191	0.000	0.601	0.012	15.409	13.118	13.018	19662	Rbp4	chr19	-	NM_0112	P4349	protein_cc	Rbp-4	retinol bin	Rbp4
10429	Pde6c	0.076	0.798	0.060	0.001	0.576	0.025	4.504	0.778	0.452	110855	Pde6c	chr19	+	NM_0011	P11	protein_cc	cpfl1	phosphod	Pde6c
10430	Fra10ac1	0.753	0.819	0.616	0.022	0.015	0.059	7.879	7.469	7.181	70567	Fra10ac1	chr19	-	NM_0010	P14137	protein_cc	57304550	FRA10AC1	Fra10ac1
10431	Lgi1	1.005	1.405	1.412	0.000	0.182	0.423	0.000	0.008	0.498	56839	Lgi1	chr19	+	NM_0202	P2405	protein_cc	BB130740	leucine-ric	Lgi1
10432	Slc35g1	0.288	1.930	0.556	0.001	0.022	0.111	8.335	6.540	7.488	240660	Slc35g1	chr19	+	NM_1755	P1711	protein_cc	AA590464	solute car	Slc35g1
10433	Plce1	0.632	1.489	0.940	0.002	0.000	0.473	6.305	5.642	6.216	74055	Plce1	chr19	+	NM_0195	P21152	protein_cc	3230403A	phospholi	Plce1
10434	Noc3l	1.436	0.766	1.100	0.152	0.230	0.684	5.948	6.470	6.085	57753	Noc3l	chr19	-	NM_0213	P25429	protein_cc	AF233884	NOC3 like	Noc3l
10435	Tbc1d12	0.795	0.787	0.626	0.326	0.295	0.222	6.573	6.242	5.897	209478	Tbc1d12	chr19	+	NM_1459	P3952	protein_cc	BC033574	TBC1D12:	Tbc1d12
10436	Hells	2.481	0.994	2.467	0.156	0.989	0.276	2.947	4.258	4.250	15201	Hells	chr19	+	NM_0082	P17898	protein_cc	AI323785|	helicase, l	Hells
10437	Cyp2c55	0.190	1.106	0.210	0.001	0.861	0.052	4.813	2.416	2.562	72082	Cyp2c55	chr19	+	NM_0280	P10101	protein_cc	2010318C	cytochron	Cyp2c55
10438	Cyp2c65	1.015	2.326	2.360	0.000	0.182	0.423	0.000	0.021	1.239	72303	Cyp2c65	chr19	+	NM_0281	P23532	protein_cc	2210009K	cytochron	Cyp2c65
10439	Cyp2c66	0.534	0.990	0.528	0.000	1.000	0.015	0.921	0.015	0.000	69888	Cyp2c66	chr19	+	NM_0010	P26686	protein_cc	2010301N	cytochron	Cyp2c66
10440	Cyp2c53-p	0.422	0.618	0.261	0.000	0.024	0.016	2.417	1.174	0.480	638988	Cyp2c53-p	chr19	+	NR_03361	.	misc_RNA	Cyp2c53|	cytochron	Cyp2c53-p
10441	Cyp2c29	0.315	0.720	0.227	0.000	0.228	0.016	13.737	12.072	11.598	13095	Cyp2c29	chr19	+	NM_0078	P25043	protein_cc	Ah-2| Ahh	cytochron	Cyp2c29
10442	Cyp2c38	0.252	1.000	0.252	0.000	0.998	0.012	8.732	6.746	6.746	13097	Cyp2c38	chr19	-	NM_0100	P23742	protein_cc	.	cytochron	Cyp2c38
10443	Cyp2c39	0.114	0.672	0.077	0.000	0.495	0.001	5.374	2.246	1.673	13098	Cyp2c39	chr19	+	NM_0100	P7361	protein_cc	CYPIIC39	cytochron	Cyp2c39
10444	Cyp2c67	0.461	0.609	0.281	0.000	0.007	0.008	11.909	10.792	10.076	545288	Cyp2c67	chr19	-	NM_0010	P703	protein_cc	C730004C	cytochron	Cyp2c67
10445	Cyp2c68	0.325	0.676	0.219	0.000	0.039	0.005	12.362	10.740	10.174	433247	Cyp2c68	chr19	-	NM_0010	P25095	protein_cc	9030012A	cytochron	Cyp2c68
10446	Cyp2c40	0.543	0.417	0.226	0.000	0.000	0.007	12.679	11.798	10.536	13099	Cyp2c40	chr19	-	NM_0100	P15367	protein_cc	AI265721|	cytochron	Cyp2c40
10447	Cyp2c69	0.456	0.332	0.151	0.000	0.000	0.003	11.847	10.715	9.124	10004310	Cyp2c69	chr19	-	NM_0011	P11080	protein_cc	AI098658	cytochron	Cyp2c69
10448	Cyp2c37	0.195	0.403	0.079	0.001	0.006	0.023	12.406	10.048	8.736	13096	Cyp2c37	chr19	+	NM_0100	P20576	protein_cc	.	cytochron	Cyp2c37
10449	Cyp2c54	0.291	0.522	0.152	0.000	0.008	0.010	12.056	10.275	9.337	404195	Cyp2c54	chr19	-	NM_2065	P5519	protein_cc	EG404195	cytochron	Cyp2c54
10450	Cyp2c50	0.262	0.785	0.205	0.000	0.299	0.016	13.318	11.384	11.034	107141	Cyp2c50	chr19	+	NM_1341	P16908	protein_cc	AI266900	cytochron	Cyp2c50
10451	Cyp2c70	0.634	1.102	0.699	0.042	0.709	0.373	12.360	11.702	11.842	226105	Cyp2c70	chr19	-	NM_1454	P7595	protein_cc	.	cytochron	Cyp2c70
10452	Pdlim1	0.834	1.199	1.001	0.129	0.136	0.994	9.066	8.805	9.067	54132	Pdlim1	chr19	-	NM_0168	P25068	protein_cc	Clim1| CLI	PDZ and L	Pdlim1

10453	Sorbs1	0.538	1.081	0.582	0.001	0.675	0.123	9.527	8.632	8.745	20411	Sorbs1	chr19	-	NM_1783	P83	protein_cc	2310065E	sorbin and	Sorbs1
10454	Aldh18a1	22.485	0.518	11.649	0.000	0.026	0.148	0.696	5.187	4.238	56454	Aldh18a1	chr19	-	NM_1535	P10917	protein_cc	2810433K	aldehyde	Aldh18a1
10455	Tctn3	1.039	3.367	3.498	0.951	0.013	0.134	1.976	2.031	3.783	67590	Tctn3	chr19	-	NM_0262	P4096	protein_cc	4930521E	tectonic fa	Tctn3
10456	Entpd1	2.685	1.513	4.061	0.002	0.192	0.169	4.585	6.010	6.607	12495	Entpd1	chr19	+	NM_0013	P17107	protein_cc	2610206B	ectonucle	Entpd1
10457	Ccnj	3.278	0.446	1.462	0.004	0.025	0.694	3.427	5.140	3.975	240665	Ccnj	chr19	+	NM_1728	P16183	protein_cc	D430039C	cyclin J	Ccnj
10458	E030044B	1.000	1.000	1.000	1.000	1.000	1.000	0.000	0.000	0.000	381223	E030044B	chr19	+	NR_04534	.	lncRNA	E030007J	RIKEN cDN	E030044B
10459	Zfp518a	2.913	0.500	1.456	0.000	0.023	0.562	6.699	8.241	7.241	72672	Zfp518a	chr19	+	NM_0283	P8444	protein_cc	2810401C	zinc finger	Zfp518a
10460	Blnk	0.682	1.829	1.248	0.408	0.459	0.849	3.106	2.554	3.425	17060	Blnk	chr19	-	NM_0085	P8289	protein_cc	BASH| Bca	B cell linke	Blnk
10461	Dntt	0.910	1.504	1.368	0.824	0.023	0.631	3.558	3.421	4.009	21673	Dntt	chr19	+	NM_0093	P10458	protein_cc	Tdt	deoxynucl	Dntt
10462	Opalin	1.000	1.000	1.000	1.000	1.000	1.000	0.000	0.000	0.000	226115	Opalin	chr19	-	NM_1535	P18571	protein_cc	Tmem10|	oligodend	Opalin
10463	Tll2	1.000	1.000	1.000	1.000	1.000	1.000	0.000	0.000	0.000	24087	Tll2	chr19	-	NM_0119	P11268	protein_cc	.	tolloid-like	Tll2
10464	Tm9sf3	1.001	0.796	0.797	0.986	0.023	0.130	11.041	11.042	10.713	107358	Tm9sf3	chr19	-	NM_1333	P1576	protein_cc	1810073N	transmem	Tm9sf3
10465	Pik3ap1	3.088	0.256	0.790	0.000	0.000	0.530	5.771	7.398	5.431	83490	Pik3ap1	chr19	-	NM_0313	P1818	protein_cc	1810044J	phosphoir	Pik3ap1
10466	Lcor	0.780	0.828	0.646	0.066	0.239	0.057	6.506	6.149	5.876	212391	Lcor	chr19	+	NM_1721	P2370	protein_cc	3110023F	ligand dep	Lcor
10467	Slit1	1.005	1.439	1.446	0.000	0.182	0.423	0.000	0.008	0.532	20562	Slit1	chr19	-	NM_0157	P16746	protein_cc	mKIAA081	slit homol	Slit1
10468	Gm19424	1.000	1.000	1.000	1.000	1.000	1.000	0.000	0.000	0.000	10050287	Gm19424	chr19	+	NR_04032	.	lncRNA	.	predicted	Gm19424
10469	Arhgap19	0.462	0.637	0.294	0.003	0.224	0.035	2.632	1.516	0.865	71085	Arhgap19	chr19	-	NM_0276	P2314	protein_cc	4933411B	Rho GTPas	Arhgap19
10470	Frat1	0.355	1.666	0.591	0.001	0.059	0.044	6.079	4.585	5.321	14296	Frat1	chr19	+	NM_0080	P24192	protein_cc	AW06038	frequently	Frat1
10471	Frat2	0.823	0.625	0.515	0.509	0.026	0.322	5.000	4.718	4.041	212398	Frat2	chr19	-	NM_1776	P26025	protein_cc	.	frequently	Frat2
10472	Rrp12	3.601	0.966	3.480	0.000	0.759	0.052	4.276	6.125	6.075	107094	Rrp12	chr19	-	NM_1994	P25343	protein_cc	AA408556	ribosomal	Rrp12
10473	Pgam1	1.053	0.939	0.989	0.229	0.136	0.875	9.692	9.767	9.676	18648	Pgam1	chr19	+	NM_0234	P4577	protein_cc	2310050F	phosphog	Pgam1
10474	Exosc1	3.022	0.757	2.287	0.001	0.019	0.078	6.648	8.244	7.842	66583	Exosc1	chr19	-	NM_0256	P23706	protein_cc	2610035C	exosome	Exosc1
10475	Zdhhc16	1.667	1.133	1.889	0.023	0.066	0.082	5.462	6.199	6.379	74168	Zdhhc16	chr19	+	NM_0237	P20224	protein_cc	1500015N	zinc finger	Zdhhc16
10476	Mms19	1.142	0.985	1.125	0.346	0.686	0.609	7.388	7.579	7.557	72199	Mms19	chr19	-	NM_0281	P3246	protein_cc	2410001K	MMS19 (N	Mms19
10477	Ubtd1	3.618	0.901	3.261	0.009	0.701	0.190	4.614	6.469	6.319	226122	Ubtd1	chr19	+	NM_1455	P13153	protein_cc	BC016129	ubiquitin d	Ubtd1
10478	Ankrd2	33.076	0.883	29.207	0.000	0.227	0.018	2.811	7.859	7.680	56642	Ankrd2	chr19	+	NM_0200	P17476	protein_cc	Arpp| mA	ankyrin re	Ankrd2
10479	Hoga1	0.446	1.879	0.838	0.000	0.097	0.647	11.451	10.285	11.196	67432	Hoga1	chr19	+	NM_0261	P17078	protein_cc	0610010D	4-hydroxy	Hoga1
10480	4933411K	2.455	0.305	0.748	0.375	1.000	0.421	0.419	1.714	0.000	66765	4933411K	chr19	+	NM_0257	P4671	protein_cc	.	RIKEN cDN	4933411K
10481	Morn4	1.232	2.943	3.626	0.603	0.038	0.149	0.409	0.710	2.267	226123	Morn4	chr19	-	NM_1981	P21779	protein_cc	.	MORN rep	Morn4
10482	Pi4k2a	1.347	0.743	1.001	0.160	0.167	0.994	8.976	9.405	8.977	84095	Pi4k2a	chr19	+	NM_1455	P16787	protein_cc	Pi4k2	phosphati	Pi4k2a
10483	Avpi1	1.012	1.565	1.584	0.790	0.117	0.341	8.765	8.783	9.429	69534	Avpi1	chr19	-	NM_0271	P2354	protein_cc	2310008N	arginine v	Avpi1
10484	Marveld1	0.264	2.115	0.558	0.003	0.093	0.264	7.822	5.900	6.981	277010	Marveld1	chr19	+	NM_1831	P10470	protein_cc	AI504298|	MARVEL (	Marveld1
10485	Zfyve27	0.780	0.755	0.589	0.189	0.237	0.036	6.048	5.690	5.284	319740	Zfyve27	chr19	+	NM_0011	P23208	protein_cc	2210011N	zinc finger	Zfyve27
10486	Sfrp5	0.118	1.619	0.191	0.181	0.210	0.460	3.174	0.088	0.783	54612	Sfrp5	chr19	-	NM_0187	P11364	protein_cc	AI605071|	secreted f	Sfrp5
10487	Golga7b	0.716	0.995	0.712	0.182	1.000	0.423	0.490	0.008	0.000	71146	Golga7b	chr19	+	NM_0011	P11267	protein_cc	4933417O	golgi auto	Golga7b
10488	Crtac1	1.005	1.405	1.412	0.000	0.182	0.423	0.000	0.008	0.498	72832	Crtac1	chr19	-	NM_1451	P1242	protein_cc	2810454P	cartilage a	Crtac1
10489	R3hcc1l	2.382	0.798	1.902	0.000	0.034	0.029	6.458	7.710	7.385	52013	R3hcc1l	chr19	+	NM_1774	P10809	protein_cc	1700036B	R3H doma	R3hcc1l
10490	Loxl4	1.332	0.250	0.332	0.120	0.002	0.077	4.197	4.610	2.608	67573	Loxl4	chr19	-	NM_0011	P26013	protein_cc	4833426I2	lysyl oxida	Loxl4
10491	Pyroxd2	1.076	1.155	1.244	0.844	0.194	0.726	5.090	5.196	5.405	74580	Pyroxd2	chr19	-	NM_0290	P6379	protein_cc	3830409H	pyridine n	Pyroxd2
10492	Hps1	0.481	2.152	1.035	0.065	0.035	0.943	5.877	4.821	5.927	192236	Hps1	chr19	-	NM_0194	P20571	protein_cc	6030422N	Hermansk	Hps1
10493	Hpse2	1.000	1.000	1.000	1.000	1.000	1.000	0.000	0.000	0.000	545291	Hpse2	chr19	-	NM_0010	P12933	protein_cc	Gm968| H	heparanas	Hpse2
10494	Cnnm1	1.146	0.632	0.725	0.729	1.000	0.422	0.465	0.661	0.000	83674	Cnnm1	chr19	+	NM_0313	P6833	protein_cc	Acdp1| AI	cyclin M1	Cnnm1
10495	Got1	0.439	0.456	0.200	0.001	0.003	0.016	11.849	10.663	9.531	14718	Got1	chr19	-	NM_0103	P25838	protein_cc	AI789014|	glutamic-o	Got1
10496	Nkx2-3	1.010	1.825	1.844	0.000	0.182	0.423	0.000	0.015	0.883	18089	Nkx2-3	chr19	+	NM_0086	P10978	protein_cc	Nkx-2.3| N	NK2 home	Nkx2-3
10497	Slc25a28	1.211	1.151	1.395	0.180	0.001	0.208	7.416	7.693	7.896	246696	Slc25a28	chr19	-	NM_1451	P24318	protein_cc	2210403D	solute car	Slc25a28
10498	BC037704	1.631	2.397	3.909	0.289	0.287	0.423	0.001	0.707	1.968	10050298	BC037704	chr19	+	NR_04564	.	lncRNA		cDNA seq	BC037704
10499	Entpd7	5.426	0.863	4.683	0.005	0.380	0.011	4.750	7.190	6.978	93685	Entpd7	chr19	+	NM_0531	P9485	protein_cc	1810012B	ectonucle	Entpd7
10500	Cox15	0.730	1.853	1.353	0.022	0.012	0.215	9.074	8.620	9.510	226139	Cox15	chr19	-	NM_1448	P25501	protein_cc	2900026G	cytochrom	Cox15
10501	Cutc	0.308	1.378	0.425	0.000	0.008	0.006	8.335	6.637	7.100	66388	Cutc	chr19	+	NM_0011	P4387	protein_cc	2310039I1	cutC copp	Cutc
10502	Abcc2	0.039	2.141	0.083	0.000	0.029	0.003	11.590	6.900	7.998	12780	Abcc2	chr19	+	NM_0138	P22370	protein_cc	Abc30| AI	ATP-bindi	Abcc2
10503	Gm10768	0.003	14.035	0.043	0.000	0.076	0.010	9.915	1.568	5.379	10003862	Gm10768	chr19	+	NR_03347	.	lncRNA	ENSMUSG	predicted	Gm10768
10504	Dnmbp	0.227	1.286	0.291	0.003	0.507	0.042	8.174	6.032	6.394	71972	Dnmbp	chr19	-	NM_0280	P7003	protein_cc	2410003L	dynamin b	Dnmbp
10505	Cpn1	0.640	0.923	0.591	0.033	0.649	0.104	11.593	10.949	10.833	93721	Cpn1	chr19	-	NM_0307	P16051	protein_cc	0610011F	carboxype	Cpn1
10506	Cyp2c44	0.160	0.845	0.135	0.000	0.478	0.005	11.958	9.313	9.070	226143	Cyp2c44	chr19	-	NM_0010	P10590	protein_cc	AW10771	cytochrom	Cyp2c44
10507	Erlin1	0.718	1.482	1.064	0.016	0.000	0.644	7.191	6.713	7.281	226144	Erlin1	chr19	-	NM_0011	P23206	protein_cc	2810439N	ER lipid ra	Erlin1
10508	Chuk	0.668	1.453	0.971	0.024	0.079	0.920	9.373	8.791	9.330	12675	Chuk	chr19	-	NM_0077	P9924	protein_cc	AI256658|	conserved	Chuk
10509	Cwf19l1	0.338	1.187	0.401	0.000	0.058	0.007	6.833	5.268	5.515	72502	Cwf19l1	chr19	-	NM_0010	P26904	protein_cc	2610528C	CWF19-lik	Cwf19l1
10510	Bloc1s2	2.924	1.099	3.213	0.006	0.757	0.285	4.708	6.256	6.392	73689	Bloc1s2	chr19	-	NM_0286	P13604	protein_cc	2410089B	biogenesis	Bloc1s2
10511	Pkd2l1	1.000	1.000	1.000	1.000	1.000	1.000	0.000	0.000	0.000	329064	Pkd2l1	chr19	-	NM_1814	P14288	protein_cc	B830002B	polycystic	Pkd2l1
10512	Scd3	0.283	4.466	1.266	0.029	0.000	0.533	1.895	0.075	2.234	30049	Scd3	chr19	+	NM_0244	P19334	protein_cc	4930513N	stearoyl-c	Scd3
10513	Scd2	0.490	3.493	1.711	0.213	0.035	0.152	4.669	3.639	5.444	20250	Scd2	chr19	+	NM_0091	P8516	protein_cc	mir-5114|	stearoyl-C	Scd2
10514	Scd4	1.000	1.000	1.000	1.000	1.000	1.000	0.000	0.000	0.000	329065	Scd4	chr19	+	NM_1832	P9938	protein_cc	ACOD4	stearoyl-c	Scd4
10515	Scd1	1.411	0.743	1.049	0.162	0.433	0.941	12.949	13.446	13.018	20249	Scd1	chr19	-	NM_0091	P6565	protein_cc	AA589638	stearoyl-C	Scd1
10516	Wnt8b	1.000	1.000	1.000	1.000	1.000	1.000	0.000	0.000	0.000	22423	Wnt8b	chr19	+	NM_0117	P10305	protein_cc	.	wingless-t	Wnt8b
10517	Sec31b	1.572	0.636	1.000	0.304	1.000	0.423	0.000	0.653	0.000	240667	Sec31b	chr19	-	NM_0010	P11128	protein_cc	Gm341| S	Sec31 hon	Sec31b
10518	Ndufb8	0.586	1.189	0.697	0.000	0.034	0.005	11.627	10.856	11.105	67264	Ndufb8	chr19	-	NM_0260	P11797	protein_cc	2900010I	NADH deh	Ndufb8
10519	Hif1an	1.143	0.857	0.980	0.286	0.044	0.926	7.849	8.043	7.820	319594	Hif1an	chr19	+	NM_1769	P25762	protein_cc	2310046N	hypoxia-in	Hif1an
10520	Pax2	1.000	1.000	1.000	1.000	1.000	1.000	0.000	0.000	0.000	18504	Pax2	chr19	+	NM_0110	P6050	protein_cc	Opdc| Pax	paired box	Pax2
10521	1700039E	1.000	1.000	1.000	1.000	1.000	1.000	0.000	0.000	0.000	73322	1700039E	chr19	-	NR_04531	.	lncRNA		RIKEN cDN	1700039E
10522	Fam178a	1.194	0.880	1.051	0.236	0.319	0.697	7.027	7.283	7.098	226151	Fam178a	chr19	+	NM_0010	P13614	protein_cc	3632432H	family wit	Fam178a
10523	4930414N	1.000	1.000	1.000	1.000	1.000	1.000	0.000	0.000	0.000	73894	4930414N	chr19	-	NR_04029	.	lncRNA	.	RIKEN cDN	4930414N
10524	Sema4g	0.062	1.371	0.086	0.000	0.406	0.002	9.655	5.652	6.107	26456	Sema4g	chr19	+	NM_0119	P2221	protein_cc	AI507908|	sema dom	Sema4g
10525	Mrpl43	0.778	1.644	1.280	0.000	0.000	0.005	9.552	9.190	9.908	94067	Mrpl43	chr19	-	NM_0531	P5027	protein_cc	4930442D	mitochon	Mrpl43
10526	Peo1	0.628	1.257	0.790	0.058	0.360	0.489	6.257	5.586	5.917	226153	Peo1	chr19	+	NM_1537	P2472	protein_cc	D19Ertd62	progressiv	Peo1
10527	Lzts2	0.554	1.518	0.841	0.102	0.016	0.696	5.507	4.656	5.258	226154	Lzts2	chr19	+	NM_1455	P16930	protein_cc	BC014695	leucine zip	Lzts2
10528	Pdzd7	0.593	1.221	0.724	0.124	0.149	0.502	5.444	4.689	4.977	10050304	Pdzd7	chr19	-	NM_0011	P6716	protein_cc	9130207N	PDZ doma	Pdzd7
10529	Sfxn3	0.596	1.869	1.113	0.111	0.070	0.787	4.872	4.124	5.026	94280	Sfxn3	chr19	+	NM_0011	P25465	protein_cc	.	sideroflex	Sfxn3
10530	Kazald1	0.441	0.894	0.394	0.121	0.874	0.226	2.674	1.492	1.330	107250	Kazald1	chr19	+	NM_1789	P20319	protein_cc	AI842353|	Kazal-type	Kazald1
10531	Tlx1	0.426	0.985	0.420	0.023	1.000	0.157	1.253	0.021	0.000	21908	Tlx1	chr19	+	NM_0219	P5905	protein_cc	Hox-11| H	T cell leuk	Tlx1
10532	Lbx1	1.000	1.000	1.000	1.000	1.000	1.000	0.000	0.000	0.000	16814	Lbx1	chr19	-	NM_0106	P21778	protein_cc	Lbx1h	ladybird h	Lbx1
10533	Btrc	1.095	0.840	0.920	0.488	0.214	0.719	6.739	6.870	6.619	12234	Btrc	chr19	+	NM_0097	P15794	protein_cc	b-TrCP| be	beta-trans	Btrc
10534	Poll	0.899	0.718	0.645	0.736	0.424	0.185	6.112	5.959	5.480	56626	Poll	chr19	-	NM_0200	P2781	protein_cc	1110003P	polymeras	Poll
10535	Dpcd	1.167	1.370	1.599	0.618	0.228	0.020	7.277	7.499	7.954	226162	Dpcd	chr19	+	NM_1726	P19646	protein_cc	5330431N	deleted in	Dpcd
10536	Fbxw4	0.683	1.133	0.775	0.128	0.683	0.491	6.213	5.664	5.844	30838	Fbxw4	chr19	-	NM_0139	P2419	protein_cc	Dac| dacty	F-box and	Fbxw4
10537	4933429K	1.000	1.000	1.000	1.000	1.000	1.000	0.000	0.000	0.000	71232	4933429K	chr19	-	NR_04530	.	lncRNA		RIKEN cDN	4933429K
10538	Fgf8	1.000	1.000	1.000	1.000	1.000	1.000	0.000	0.000	0.000	14179	Fgf8	chr19	-	NM_0102	P16169	protein_cc	Aigf| Fgf-8	fibroblast	Fgf8
10539	Npm3	1.881	0.824	1.550	0.052	0.387	0.126	7.347	8.259	7.979	18150	Npm3	chr19	-	NM_0087	P24139	protein_cc	Nub1	nucleopla	Npm3
10540	Mgea5	2.120	0.580	1.229	0.000	0.006	0.479	8.951	10.035	9.249	76055	Mgea5	chr19	-	NM_0237	P19369	protein_cc	2810009A	meningior	Mgea5
10541	Kcnip2	0.267	1.321	0.353	0.099	0.210	0.373	1.948	0.044	0.446	80906	Kcnip2	chr19	-	NM_0307	P20378	protein_cc	KChIP2	Kv channe	Kcnip2
10542	9130011E	3.130	0.464	1.454	0.007	0.012	0.484	2.775	4.422	3.315	71617	9130011E	chr19	-	NM_1982	P22063	protein_cc	AI431055|	RIKEN cDN	9130011E
10543	Hps6	1.495	0.935	1.398	0.083	0.740	0.336	5.045	5.625	5.529	20170	Hps6	chr19	+	NM_1767	P23623	protein_cc	5330434N	Hermansk	Hps6

10544	Ldb1	0.834	1.508	1.258	0.527	0.293	0.634	6.368	6.106	6.699	16825	Ldb1	chr19	-	NM_0106	P26225	protein_cc	CLIM2| NI	LIM domai	Ldb1
10545	Pprc1	3.999	0.695	2.780	0.043	0.297	0.013	4.787	6.786	6.262	226169	Pprc1	chr19	+	NM_0010	P7342	protein_cc	A930018N	peroxisom	Pprc1
10546	Nolc1	2.429	0.722	1.755	0.001	0.024	0.018	7.762	9.043	8.573	70769	Nolc1	chr19	-	NM_0010	P830	protein_cc	NOPP130|	nucleolar	Nolc1
10547	Elovl3	0.167	4.686	0.783	0.004	0.025	0.527	5.438	2.857	5.086	12686	Elovl3	chr19	+	NM_0077	P9737	protein_cc	Cig30| CIN	elongation	Elovl3
10548	Pitx3	1.010	1.885	1.904	0.000	0.182	0.423	0.000	0.015	0.929	18742	Pitx3	chr19	-	NM_0088	P4895	protein_cc	ak| Ptx3	paired-like	Pitx3
10549	Gbf1	1.355	0.747	1.012	0.034	0.083	0.952	8.445	8.883	8.462	107338	Gbf1	chr19	+	NM_1789	P5088	protein_cc	1700083E	golgi-spec	Gbf1
10550	Nfkb2	13.741	0.311	4.274	0.003	0.008	0.001	6.216	9.996	8.312	18034	Nfkb2	chr19	+	NM_0011	P18194	protein_cc	lyt| NF-ka	nuclear fa	Nfkb2
10551	Psd	8.491	0.151	1.286	0.005	0.006	0.491	3.202	6.288	3.565	73728	Psd	chr19	-	NM_0286	P6155	protein_cc	1110007H	pleckstrin	Psd
10552	Fbxl15	0.955	0.786	0.751	0.769	0.215	0.278	6.408	6.343	5.995	68431	Fbxl15	chr19	+	NM_1336	P4425	protein_cc	0710008C	F-box and	Fbxl15
10553	Cuedc2	0.810	0.951	0.770	0.006	0.463	0.055	7.510	7.206	7.133	67116	Cuedc2	chr19	-	NM_0011	P23047	protein_cc	3010002G	CUE doma	Cuedc2
10554	2310034G	0.204	1.508	0.307	0.006	0.470	0.141	3.034	0.739	1.332	75579	2310034G	chr19	-	NR_04041	.	lncRNA	.	RIKEN cDN	2310034G
10555	Tmem180	0.432	1.518	0.656	0.026	0.524	0.549	4.166	2.956	3.559	75146	Tmem180	chr19	+	NM_0291	P20925	protein_cc	4930449A	transmem	Tmem180
10556	Actr1a	1.152	0.914	1.053	0.263	0.001	0.806	7.841	8.045	7.916	54130	Actr1a	chr19	-	NM_0168	P21765	protein_cc	alpha-Arp:	ARP1 actin	Actr1a
10557	Sufu	1.340	1.337	1.791	0.311	0.040	0.269	3.923	4.345	4.764	24069	Sufu	chr19	+	NM_0010	P21319	protein_cc	Su(fu)	suppresso	Sufu
10558	Trim8	1.750	0.881	1.542	0.022	0.606	0.400	7.226	8.034	7.851	93679	Trim8	chr19	+	NM_0531	P18889	protein_cc	AA408830	tripartite	Trim8
10559	Arl3	0.739	0.963	0.711	0.004	0.847	0.323	8.099	7.662	7.608	56350	Arl3	chr19	-	NM_0197	P9176	protein_cc	.	ADP-ribos	Arl3
10560	Sfxn2	0.274	0.840	0.230	0.000	0.154	0.017	9.857	7.991	7.740	94279	Sfxn2	chr19	+	NM_0531	P18101	protein_cc	C79732| F	sideroflexi	Sfxn2
10561	Wbp1l	0.504	1.314	0.663	0.004	0.105	0.084	11.164	10.177	10.572	226178	Wbp1l	chr19	+	NM_1460	P2058	protein_cc	D19Wsu1	WW doma	Wbp1l
10562	Cyp17a1	0.152	0.548	0.084	0.000	0.130	0.004	11.777	9.062	8.196	13074	Cyp17a1	chr19	-	NM_0078	P6512	protein_cc	Cyp17| p4	cytochrom	Cyp17a1
10563	2010012O	0.859	1.543	1.325	0.226	0.003	0.151	7.870	7.651	8.277	.	2010012O	chr19	+	NM_0255	P9978	.	.	.	2010012O
10564	As3mt	0.303	2.568	0.778	0.000	0.062	0.540	8.290	6.568	7.929	57344	As3mt	chr19	+	NM_0205	P3808	protein_cc	2310045H	arsenic (+	As3mt
10565	Cnnm2	2.131	0.790	1.684	0.000	0.018	0.036	4.649	5.741	5.401	94219	Cnnm2	chr19	+	NM_0011	P19521	protein_cc	Acdp2| AI	cyclin M2	Cnnm2
10566	Nt5c2	0.857	2.220	1.903	0.558	0.001	0.102	5.755	5.532	6.683	76952	Nt5c2	chr19	-	NM_0011	P319	protein_cc	2010002I2	5'-nucleot	Nt5c2
10567	Ina	1.000	1.000	1.000	1.000	1.000	1.000	0.000	0.000	0.000	226180	Ina	chr19	+	NM_1461	P14118	protein_cc	AV028420	internexin	Ina
10568	Pcgf6	3.251	0.891	2.897	0.016	0.529	0.036	4.569	6.269	6.103	71041	Pcgf6	chr19	-	NM_0276	P16716	protein_cc	4933407A	polycomb	Pcgf6
10569	Taf5	1.552	1.008	1.563	0.002	0.918	0.107	8.191	8.825	8.836	226182	Taf5	chr19	+	NM_1773	P18860	protein_cc	6330528C	TATA-box	Taf5
10570	Usmg5	1.203	1.018	1.225	0.002	0.759	0.150	10.736	11.003	11.029	66477	Usmg5	chr19	-	NM_0232	P14682	protein_cc	2010301L	upregulate	Usmg5
10571	Pdcd11	2.137	0.743	1.589	0.025	0.178	0.074	6.673	7.768	7.341	18572	Pdcd11	chr19	+	NM_0110	P6454	protein_cc	1110021I2	programm	Pdcd11
10572	Calhm2	0.558	1.735	0.969	0.339	0.347	0.975	3.794	2.953	3.748	72691	Calhm2	chr19	-	NM_1337	P19457	protein_cc	2810048G	calcium hc	Calhm2
10573	Calhm1	1.000	1.000	1.000	1.000	1.000	1.000	0.000	0.000	0.000	546729	Calhm1	chr19	-	NM_0010	P13658	protein_cc	EG546729	calcium hc	Calhm1
10574	Neurl1a	1.120	1.289	1.444	0.874	0.732	0.748	1.326	1.489	1.856	18011	Neurl1a	chr19	+	NM_0213	P11156	protein_cc	2410129E	neuralized	Neurl1a
10575	Sh3pxd2a	0.322	0.912	0.294	0.001	0.519	0.042	8.054	6.420	6.287	14218	Sh3pxd2a	chr19	-	NM_0080	P1350	protein_cc	2310014D	SH3 and P	Sh3pxd2a
10576	Obfc1	1.472	0.666	0.980	0.250	0.282	0.962	3.830	4.387	3.801	108689	Obfc1	chr19	-	NM_1753	P18530	protein_cc	0610009H	oligonucle	Obfc1
10577	Gm19557	1.010	1.825	1.844	0.000	0.182	0.423	0.000	0.015	0.883	10050310	Gm19557	chr19	+	NR_04532	.	lncRNA		predicted	Gm19557
10578	Slk	1.029	0.845	0.869	0.521	0.168	0.481	9.096	9.138	8.894	20874	Slk	chr19	+	NM_0011	P1475	protein_cc	9A2| AW	STE20-like	Slk
10579	Col17a1	1.000	1.000	1.000	1.000	1.000	1.000	0.000	0.000	0.000	12821	Col17a1	chr19	-	NM_0077	P18506	protein_cc	BP180| Bp	collagen, t	Col17a1
10580	Sfr1	1.519	1.044	1.587	0.011	0.702	0.089	10.127	10.731	10.793	67788	Sfr1	chr19	+	NM_0263	P780	protein_cc	6330577E	SWI5 depe	Sfr1
10581	Cfap43	0.583	1.869	1.090	0.232	0.237	0.898	4.555	3.777	4.679	10004853	Cfap43	chr19	-	NM_0275	P15916	protein_cc	4632415N	cilia and fl	Cfap43
10582	Gsto1	0.379	1.077	0.409	0.000	0.019	0.003	10.769	9.370	9.478	14873	Gsto1	chr19	+	NM_0103	P19878	protein_cc	AA407097	glutathion	Gsto1
10583	Gsto2	1.282	0.334	0.428	0.590	0.154	0.035	1.634	1.992	0.409	68214	Gsto2	chr19	+	NM_0300	P11283	protein_cc	1700020F	glutathion	Gsto2
10584	Itprip	6.499	1.379	8.965	0.009	0.188	0.051	3.358	6.058	6.522	414801	Itprip	chr19	-	NM_0010	P5825	protein_cc	4833420	inositol 1,	Itprip
10585	Cfap58	1.000	1.000	1.000	1.000	1.000	1.000	0.000	0.000	0.000	381229	Cfap58	chr19	+	NM_0011	P13867	protein_cc	Ccdc147|	cilia and fl	Cfap58
10586	Sorcs3	0.202	1.350	0.273	0.023	0.216	0.188	2.367	0.058	0.491	66673	Sorcs3	chr19	+	NM_0256	P9292	protein_cc	6330404A	sortilin-re	Sorcs3
10587	Sorcs1	1.000	1.000	1.000	1.000	1.000	1.000	0.000	0.000	0.000	58178	Sorcs1	chr19	-	NM_0012	P23052	protein_cc	mSorCS| S	sortilin-re	Sorcs1
10588	Ins1	1.019	2.681	2.732	0.000	0.044	0.215	0.000	0.027	1.450	16333	Ins1	chr19	+	NM_0083	P6534	protein_cc	Ins-1| Ins	insulin I	Ins1
10589	AA387883	1.000	1.000	1.000	1.000	1.000	1.000	0.000	0.000	0.000	10004345	AA387883	chr19	+	NR_03067	.	lncRNA	10004345	expressed	AA387883
10590	Xpnpep1	1.414	1.019	1.441	0.118	0.923	0.217	7.283	7.783	7.810	170750	Xpnpep1	chr19	-	NM_1332	P10830	protein_cc	D230045I	X-prolyl ar	Xpnpep1
10591	1700054A	1.000	1.000	1.000	1.000	1.000	1.000	0.000	0.000	0.000	78457	1700054A	chr19	+	NR_04532	.	lncRNA	.	RIKEN cDN	1700054A
10592	Add3	0.250	2.495	0.622	0.000	0.007	0.083	7.646	5.643	6.962	27360	Add3	chr19	+	NM_0011	P17188	protein_cc	AI463285|	adducin 3	Add3
10593	1700001K	1.000	1.000	1.000	1.000	1.000	1.000	0.000	0.000	0.000	69319	1700001K	chr19	+	NR_03659	.	lncRNA	.	RIKEN cDN	1700001K
10594	Mxi1	0.253	2.074	0.524	0.001	0.017	0.065	7.526	5.541	6.593	17859	Mxi1	chr19	+	NM_0010	P12277	protein_cc	bHLHc11|	MAX inter	Mxi1
10595	Smndc1	1.338	0.793	1.061	0.014	0.120	0.833	8.336	8.756	8.422	76479	Smndc1	chr19	-	NM_1724	P23120	protein_cc	2410004J	survival m	Smndc1
10596	5830416P	2.275	0.580	1.319	0.281	0.398	0.710	2.365	3.551	2.764	.	5830416P	chr19	-	NR_02842	.	.	.	.	5830416P
10597	Dusp5	1.000	1.000	1.000	1.000	1.000	1.000	0.000	0.000	0.000	240672	Dusp5	chr19	+	NM_0010	P17573	protein_cc	Gm337	dual speci	Dusp5
10598	Smc3	0.904	0.727	0.657	0.019	0.030	0.109	8.897	8.750	8.291	13006	Smc3	chr19	+	NM_0077	P22028	protein_cc	Bamacan|	structural	Smc3
10599	Rbm20	1.579	0.827	1.305	0.006	0.247	0.405	4.484	5.143	4.869	73713	Rbm20	chr19	+	NM_0011	P23373	protein_cc	1110018J	RNA bindi	Rbm20
10600	Pdcd4	0.689	1.678	1.157	0.014	0.055	0.655	10.380	9.843	10.590	18569	Pdcd4	chr19	+	NM_0110	P14800	protein_cc	D19Ucla1|	programm	Pdcd4
10601	Bbip1	0.693	2.024	1.402	0.005	0.021	0.328	10.843	10.313	11.331	10050357	Bbip1	chr19	-	NM_0011	P21837	protein_cc	2310002J	BBSome in	Bbip1
10602	Shoc2	1.141	0.848	0.968	0.013	0.016	0.652	8.396	8.586	8.349	56392	Shoc2	chr19	+	NM_0011	P25548	protein_cc	AU017287	soc-2 (sup	Shoc2
10603	Adra2a	0.509	1.780	0.906	0.184	0.186	0.897	1.002	0.028	0.859	11551	Adra2a	chr19	+	NM_0074	P26300	protein_cc	Adra-2| A	adrenergic	Adra2a
10604	Gpam	0.101	8.204	0.829	0.000	0.000	0.152	5.151	1.844	4.881	14732	Gpam	chr19	-	NM_0081	P3494	protein_cc	GPAT| GP	glycerol-3	Gpam
10605	Tectb	9.333	0.579	5.407	0.001	0.050	0.099	0.002	3.224	2.437	21684	Tectb	chr19	+	NM_0093	P17572	protein_cc	Tctnb	tectorin b	Tectb
10606	Gucy2g	3.300	0.303	1.000	0.306	1.000	0.423	0.001	1.723	0.000	73707	Gucy2g	chr19	-	NM_0010	P25668	protein_cc	2410077I	guanylate	Gucy2g
10607	Acsl5	0.549	0.597	0.328	0.007	0.048	0.011	11.139	10.274	9.531	433256	Acsl5	chr19	+	NM_0279	P18694	protein_cc	1700030F	acyl-CoA s	Acsl5
10608	Zdhhc6	0.650	1.053	0.684	0.015	0.586	0.183	8.585	7.964	8.037	66980	Zdhhc6	chr19	-	NM_0258	P17785	protein_cc	2400007G	zinc finger	Zdhhc6
10609	Vti1a	0.614	1.587	0.975	0.059	0.010	0.915	6.888	6.186	6.852	53611	Vti1a	chr19	+	NM_0168	P17403	protein_cc	1110014F	vesicle tra	Vti1a
10610	4930552P	0.552	0.990	0.547	0.182	1.000	0.423	0.871	0.015	0.000	77594	4930552P	chr19	+	NR_04531	.	lncRNA	.	RIKEN cDN	4930552P
10611	Tcf7l2	0.406	1.096	0.445	0.000	0.084	0.013	9.453	8.154	8.286	21416	Tcf7l2	chr19	+	NM_0011	P16139	protein_cc	mTcf-4B|	transcripti	Tcf7l2
10612	Habp2	0.438	0.774	0.339	0.000	0.001	0.008	9.492	8.302	7.932	226243	Habp2	chr19	+	NM_1461	P20852	protein_cc	AI035669|	hyaluronic	Habp2
10613	Nrap	0.550	0.788	0.434	0.000	0.010	0.001	8.143	7.282	6.939	18175	Nrap	chr19	-	NM_0012	P11015	protein_cc	.	nebulin-re	Nrap
10614	Casp7	2.481	0.772	1.915	0.000	0.131	0.205	6.430	7.741	7.367	12369	Casp7	chr19	+	NM_0076	P13741	protein_cc	AI314680|	caspase 7	Casp7
10615	Plekhs1	0.999	0.430	0.430	0.998	0.033	0.568	2.383	2.381	1.165	226245	Plekhs1	chr19	+	NM_1726	P24282	protein_cc	9930023K	pleckstrin	Plekhs1
10616	Dclre1a	0.377	1.196	0.451	0.000	0.089	0.009	7.199	5.791	6.049	55947	Dclre1a	chr19	-	NM_0188	P13716	protein_cc	2810043H	DNA cross	Dclre1a
10617	Nhlrc2	0.973	1.062	1.033	0.854	0.690	0.927	7.360	7.320	7.407	66866	Nhlrc2	chr19	+	NM_0258	P26407	protein_cc	1200003G	NHL repea	Nhlrc2
10618	Adrb1	0.069	0.581	0.038	0.005	1.000	0.069	4.709	0.782	0.000	11554	Adrb1	chr19	+	NM_0074	P2775	protein_cc	Adrb-1| b	adrenergic	Adrb1
10619	Ccdc186	2.820	0.457	1.288	0.011	0.015	0.436	5.820	7.316	6.186	213993	Ccdc186	chr19	-	NM_1707	P2142	protein_cc	1810028B	coiled-coil	Ccdc186
10620	Tdrd1	0.716	0.995	0.712	0.182	1.000	0.423	0.490	0.008	0.000	83561	Tdrd1	chr19	+	NM_0010	P5455	protein_cc	MTR-1	tudor dom	Tdrd1
10621	Vwa2	2.305	10.121	23.332	0.275	0.082	0.269	0.002	1.207	4.546	240675	Vwa2	chr19	+	NM_1728	P20401	protein_cc	Amaco	von Willeb	Vwa2
10622	Afap1l2	1.449	0.480	0.696	0.310	0.079	0.739	2.388	2.923	1.865	226250	Afap1l2	chr19	-	NM_1461	P24431	protein_cc	AU041783	actin filam	Afap1l2
10623	Ablim1	0.390	1.346	0.525	0.000	0.261	0.109	7.828	6.469	6.898	226251	Ablim1	chr19	-	NM_0012	P7913	protein_cc	2210411C	actin-bind	Ablim1
10624	4930449E	2.722	0.367	1.000	0.306	1.000	0.423	0.001	1.445	0.000	78120	4930449E	chr19	+	NR_04531	.	lncRNA	.	RIKEN cDN	4930449E
10625	B230217O	0.912	0.627	0.571	0.728	0.212	0.509	2.342	2.209	1.534	320879	B230217O	chr19	-	NR_04031	.	lncRNA	.	RIKEN cDN	B230217O
10626	Fam160b1	0.436	1.742	0.760	0.002	0.006	0.200	8.542	7.344	8.145	226252	Fam160b1	chr19	+	NM_1455	P10948	protein_cc	AI450540|	family wit	Fam160b1
10627	Trub1	0.363	1.570	0.571	0.000	0.000	0.034	7.588	6.127	6.779	72133	Trub1	chr19	+	NM_0298	P8391	protein_cc	2610009I	TruB pseu	Trub1
10628	6720468P	1.000	1.000	1.000	1.000	1.000	1.000	0.000	0.000	0.000	78073	6720468P	chr19	+	NR_04030	.	lncRNA	.	RIKEN cDN	6720468P
10629	Atrnl1	0.652	1.057	0.689	0.001	0.767	0.260	6.886	6.268	6.349	226255	Atrnl1	chr19	+	NM_1814	P10403	protein_cc	AI504415|	attractin li	Atrnl1
10630	Gfra1	1.271	1.040	1.322	0.037	0.723	0.195	11.516	11.862	11.919	14585	Gfra1	chr19	-	NR_10434	.	protein_cc	AU042498	glial cell lin	Gfra1
10631	Gm10007	1.000	1.000	1.000	1.000	1.000	1.000	0.000	0.000	0.000	791347	Gm10007	chr19	+	NR_04044	.	lncRNA	ENSMUSG	predicted	Gm10007
10632	Ccdc172	1.000	1.000	1.000	1.000	1.000	1.000	0.000	0.000	0.000	75645	Ccdc172	chr19	+	NM_0293	P7348	protein_cc	1700011F	coiled-coil	Ccdc172
10633	1810007D	7.479	0.242	1.808	0.224	0.279	0.423	0.001	2.904	0.856	69055	1810007D	chr19	+	NR_03813	.	lncRNA	.	RIKEN cDN	1810007D
10634	Pnlip	0.504	0.990	0.499	0.181	1.000	0.423	1.002	0.015	0.000	69060	Pnlip	chr19	+	NM_0269	P9226	protein_cc	1810007A	pancreatic	Pnlip

10635	1810018F	1.000	1.000	1.000	1.000	1.000	1.000	0.000	0.000	0.000	69166	1810018F	chr19	+	NR_03814	.	lncRNA	1810031L(	RIKEN cDN	1810018F1
10636	Pnliprp1	1.000	1.000	1.000	1.000	1.000	1.000	0.000	0.000	0.000	18946	Pnliprp1	chr19	+	NM_0188	P22957	protein_cc	Plrp1	pancreatic	Pnliprp1
10637	Pnliprp2	1.000	1.000	1.000	1.000	1.000	1.000	0.000	0.000	0.000	18947	Pnliprp2	chr19	+	NM_0111	P16053	protein_cc	PLRP2	pancreatic	Pnliprp2
10638	1700019N	1.000	1.000	1.000	1.000	1.000	1.000	0.000	0.000	0.000	67507	1700019N	chr19	-	NM_0262	P3427	protein_cc	.	RIKEN cDN	1700019N
10639	Hspa12a	0.491	1.012	0.497	0.007	0.983	0.356	3.588	2.562	2.580	73442	Hspa12a	chr19	-	NM_1751	P14490	protein_cc	1700063D	heat shocl	Hspa12a
10640	Eno4	1.000	1.000	1.000	1.000	1.000	1.000	0.000	0.000	0.000	226265	Eno4	chr19	+	NM_1786	P7054	protein_cc	6430537H	enolase 4	Eno4
10641	Shtn1	0.697	0.973	0.678	0.025	0.843	0.011	7.489	6.968	6.929	71653	Shtn1	chr19	-	NM_0011	P9161	protein_cc	4930506M	shootin 1	Shtn1
10642	Vax1	6.908	0.388	2.682	0.005	0.044	0.423	0.001	2.790	1.425	22326	Vax1	chr19	-	NM_0095	P19786	protein_cc	.	ventral an	Vax1
10643	Kcnk18	1.000	1.000	1.000	1.000	1.000	1.000	0.000	0.000	0.000	332396	Kcnk18	chr19	+	NM_2072	P1074	protein_cc	Gm781| T	potassium	Kcnk18
10644	Slc18a2	0.861	1.149	0.989	0.120	0.530	0.973	4.724	4.508	4.708	214084	Slc18a2	chr19	+	NM_1725	P17533	protein_cc	1110037L	solute car	Slc18a2
10645	Pdzd8	1.428	0.928	1.325	0.000	0.416	0.222	7.908	8.423	8.315	107368	Pdzd8	chr19	-	NM_0010	P7582	protein_cc	A630041P	PDZ doma	Pdzd8
10646	Emx2os	1.005	1.439	1.446	0.000	0.182	0.423	0.000	0.008	0.532	329078	Emx2os	chr19	-	NR_00286	.	lncRNA	.	Emx2 opp	Emx2os
10647	Emx2	1.000	1.000	1.000	1.000	1.000	1.000	0.000	0.000	0.000	13797	Emx2	chr19	+	NM_0101	P15070	protein_cc	Pdo	empty spi	Emx2
10648	2700089I2	1.000	1.000	1.000	1.000	1.000	1.000	0.000	0.000	0.000	72647	2700089I2	chr19	-	NR_04530	.	lncRNA	.	RIKEN cDN	2700089I2
10649	Rab11fip2	0.982	0.785	0.771	0.709	0.010	0.131	6.424	6.398	6.049	74998	Rab11fip2	chr19	-	NM_0010	P17614	protein_cc	4930470G	RAB11 fan	Rab11fip2
10650	E330013P	1.000	1.000	1.000	1.000	1.000	1.000	0.000	0.000	0.000	107376	E330013P	chr19	+	NR_02694	.	lncRNA	9930018I1	RIKEN cDN	E330013P
10651	Fam204a	2.116	1.061	2.245	0.029	0.821	0.209	6.230	7.311	7.397	76539	Fam204a	chr19	-	NM_0296	P10914	protein_cc	2310065H	family wit	Fam204a
10652	Prlhr	1.000	1.000	1.000	1.000	1.000	1.000	0.000	0.000	0.000	226278	Prlhr	chr19	-	NM_2016	P12075	protein_cc	Gm339| G	prolactin r	Prlhr
10653	Cacul1	0.989	1.165	1.152	0.896	0.373	0.578	9.230	9.214	9.435	78832	Cacul1	chr19	-	NM_0301	P3004	protein_cc	2700078E	CDK2 assc	Cacul1
10654	Nanos1	0.229	0.969	0.222	0.181	1.000	0.423	2.173	0.045	0.000	332397	Nanos1	chr19	+	NM_1784	P2930	protein_cc	NOS-1	nanos hor	Nanos1
10655	Eif3a	1.047	0.654	0.684	0.348	0.012	0.158	11.741	11.807	11.194	13669	Eif3a	chr19	-	NM_0101	P16552	protein_cc	A830012B	eukaryotic	Eif3a
10656	Fam45a	0.946	1.333	1.262	0.650	0.029	0.105	6.699	6.620	7.035	67894	Fam45a	chr19	+	NM_0011	P14485	protein_cc	1810055E	family wit	Fam45a
10657	Sfxn4	1.111	0.714	0.793	0.748	0.467	0.674	2.986	3.138	2.651	94281	Sfxn4	chr19	-	NM_0531	P24364	protein_cc	.	sideroflexi	Sfxn4
10658	Prdx3	0.912	1.078	0.983	0.137	0.055	0.861	9.426	9.292	9.401	11757	Prdx3	chr19	-	NM_0074	P813	protein_cc	Aop1| AW	peroxired	Prdx3
10659	Grk5	2.054	0.804	1.651	0.135	0.548	0.349	4.379	5.417	5.102	14773	Grk5	chr19	+	NM_0188	P10452	protein_cc	Gprk5	G protein-	Grk5
10660	Zfp950	1.125	0.554	0.623	0.417	0.022	0.024	7.502	7.673	6.820	414758	Zfp950	chr19	-	NR_13195	.	protein_cc	5830428H	zinc finger	Zfp950
10661	Gm7102	0.388	3.319	1.289	0.049	0.004	0.479	2.100	0.736	2.467	633057	Gm7102	chr19	-	NM_0011	P22698	protein_cc	EG633057	predicted	Gm7102
10662	Csf2ra	2.090	1.649	3.446	0.051	0.054	0.045	5.910	6.974	7.695	12982	Csf2ra	chr19	-	NM_0099	P16247	protein_cc	CD116| Cs	colony stir	Csf2ra
10663	LOC10004	1.000	1.000	1.000	1.000	1.000	1.000	0.000	0.000	0.000	10004103	LOC10004	chr1_GL45	-	NR_13198	.	misc_RNA	.	Sp110 nuc	LOC10004
10664	LOC66478	1.000	1.000	1.000	1.000	1.000	1.000	0.000	0.000	0.000	664787	LOC66478	chr1_GL45	-	NR_13198	.	misc_RNA	.	Sp110 nuc	LOC66478
10665	Csprs	1.000	1.000	1.000	1.000	1.000	1.000	0.000	0.000	0.000	114564	Csprs	chr1_GL45	-	NM_0336	P24083	protein_cc	D1Lub1| F	componer	Csprs
10666	Fam171a1	0.581	1.229	0.714	0.019	0.594	0.480	6.001	5.217	5.515	269233	Fam171a1	chr2	+	NM_0010	P23001	protein_cc	9630050M	family wit	Fam171a1
10667	Nmt2	0.683	1.303	0.890	0.129	0.204	0.611	7.496	6.946	7.327	18108	Nmt2	chr2	+	NM_0012	P17417	protein_cc	A930001K	N-myristo	Nmt2
10668	Rpp38	3.129	0.790	2.472	0.000	0.158	0.141	6.293	7.938	7.598	227522	Rpp38	chr2	-	NM_0010	P12230	protein_cc	C330006A	ribonuclea	Rpp38
10669	Acbd7	1.581	0.906	1.432	0.301	0.802	0.423	0.000	0.661	0.519	78245	Acbd7	chr2	+	NM_0300	P19916	protein_cc	9230116B	acyl-Coen	Acbd7
10670	Olah	2.817	1.451	4.086	0.023	0.552	0.423	0.001	1.495	2.032	99035	Olah	chr2	-	NM_1459	P690	protein_cc	AI607300|	oleoyl-ACI	Olah
10671	Meig1	2.855	3.163	9.029	0.297	0.110	0.197	0.001	1.515	3.176	104362	Meig1	chr2	-	NM_0085	P16503	protein_cc	Meg1| MI	meiosis ex	Meig1
10672	Dclre1c	0.993	0.973	0.967	0.958	0.878	0.920	5.965	5.955	5.916	227525	Dclre1c	chr2	+	NM_1756	P26642	protein_cc	99301211(	DNA cross	Dclre1c
10673	Suv39h2	2.429	1.056	2.565	0.005	0.922	0.536	2.724	4.004	4.082	64707	Suv39h2	chr2	-	NR_02750	.	protein_cc	4930507K	suppresso	Suv39h2
10674	Hspa14	2.025	0.933	1.889	0.083	0.809	0.209	7.298	8.316	8.216	50497	Hspa14	chr2	-	NM_0157	P11421	protein_cc	70kDa| Hs	heat shocl	Hspa14
10675	Cdnf	1.395	2.703	3.771	0.384	0.161	0.348	1.046	1.526	2.961	227526	Cdnf	chr2	+	NM_1776	P2581	protein_cc	9330140G	cerebral d	Cdnf
10676	Fam107b	0.936	0.365	0.342	0.748	0.003	0.130	8.668	8.573	7.118	66540	Fam107b	chr2	+	NM_0256	P11604	protein_cc	3110001A	family wit	Fam107b
10677	1700080N	1.000	1.000	1.000	1.000	1.000	1.000	0.000	0.000	0.000	73489	1700080N	chr2	-	NR_04050	.	lncRNA	4933408D	RIKEN cDN	1700080N
10678	Frmd4a	0.425	2.145	0.911	0.008	0.060	0.758	5.354	4.118	5.219	209630	Frmd4a	chr2	+	NM_1724	P15670	protein_cc	2700017I(	FERM don	Frmd4a
10679	Prpf18	1.103	0.671	0.740	0.610	0.162	0.478	7.962	8.103	7.527	67229	Prpf18	chr2	-	NM_0260	P17483	protein_cc	2810441A	pre-mRNA	Prpf18
10680	Bend7	0.701	0.874	0.613	0.042	0.406	0.063	7.109	6.598	6.404	209645	Bend7	chr2	+	NM_0011	P767	protein_cc	1110017O	BEN doma	Bend7
10681	Sephs1	1.228	1.112	1.365	0.212	0.530	0.237	8.080	8.376	8.529	109079	Sephs1	chr2	+	NM_1754	P17518	protein_cc	1110046B	selenopho	Sephs1
10682	Phyh	0.552	1.094	0.604	0.000	0.003	0.006	13.968	13.111	13.240	16922	Phyh	chr2	+	NM_0107	P2791	protein_cc	AI256161|	phytanoyl	Phyh
10683	Ucma	1.000	1.000	1.000	1.000	1.000	1.000	0.000	0.000	0.000	68527	Ucma	chr2	+	NM_0011	P25664	protein_cc	1110017I1	upper zon	Ucma
10684	Mcm10	0.088	4.975	0.436	0.000	0.044	0.110	8.456	4.944	7.259	70024	Mcm10	chr2	-	NM_0272	P14476	protein_cc	2410041F	minichron	Mcm10
10685	Optn	0.867	0.932	0.808	0.038	0.584	0.369	8.648	8.441	8.340	71648	Optn	chr2	-	NM_1818	P10850	protein_cc	4930441O	optineurin	Optn
10686	Ccdc3	0.040	2.169	0.087	0.000	0.036	0.001	4.801	0.153	1.270	74186	Ccdc3	chr2	+	NM_0288	P1056	protein_cc	2310045O	coiled-coil	Ccdc3
10687	Camk1d	0.040	2.098	0.084	0.000	0.009	0.001	10.275	5.629	6.698	227541	Camk1d	chr2	-	NM_0012	P7735	protein_cc	A630059D	calcium/ca	Camk1d
10688	Cdc123	1.318	0.896	1.181	0.000	0.151	0.298	8.784	9.183	9.024	98828	Cdc123	chr2	-	NM_1338	P20466	protein_cc	AA959893	cell divisio	Cdc123
10689	Nudt5	2.850	0.871	2.483	0.000	0.438	0.158	5.513	7.024	6.825	53893	Nudt5	chr2	+	NM_0169	P377	protein_cc	.	nudix (nuc	Nudt5
10690	Sec61a2	1.286	1.608	2.068	0.322	0.112	0.159	4.451	4.814	5.500	57743	Sec61a2	chr2	-	NM_0213	P1775	protein_cc	.	Sec61, alp	Sec61a2
10691	Dhtkd1	0.155	1.738	0.269	0.069	0.202	0.329	2.771	0.077	0.875	209692	Dhtkd1	chr2	-	NM_0010	P652	protein_cc	C330018I(	dehydrog	Dhtkd1
10692	Upf2	2.684	0.658	1.767	0.000	0.002	0.099	7.975	9.399	8.796	326622	Upf2	chr2	+	NM_0010	P19306	protein_cc	.	UPF2 regu	Upf2
10693	Proser2	0.153	3.231	0.495	0.004	0.066	0.250	6.555	3.850	5.542	227545	Proser2	chr2	-	NM_0011	P16682	protein_cc	5430407P	proline an	Proser2
10694	Gm10857	1.000	1.000	1.000	1.000	1.000	1.000	0.000	0.000	0.000	10003835	Gm10857	chr2	+	NR_03347	.	lncRNA	AU01585C	predicted	Gm10857
10695	Echdc3	0.229	0.695	0.159	0.000	0.196	0.006	9.769	7.640	7.115	67856	Echdc3	chr2	-	NM_0242	P3484	protein_cc	2310005D	enoyl Coe	Echdc3
10696	A230108P	1.000	1.000	1.000	1.000	1.000	1.000	0.000	0.000	0.000	320132	A230108P	chr2	+	NR_04033	.	lncRNA	.	RIKEN cDN	A230108P
10697	Usp6nl	1.337	0.719	0.962	0.439	0.400	0.909	5.039	5.458	4.983	98910	Usp6nl	chr2	+	NM_0010	P4361	protein_cc	AI316785|	USP6 N-te	Usp6nl
10698	Celf2	0.442	1.704	0.752	0.002	0.005	0.260	8.533	7.353	8.123	14007	Celf2	chr2	-	NM_0011	P5454	protein_cc	B230218O	CUGBP, El	Celf2
10699	5031426D	0.311	1.773	0.551	0.246	0.381	0.664	2.425	0.738	1.564	68144	5031426D	chr2	-	NR_02789	.	lncRNA	.	RIKEN cDN	5031426D
10700	1700061F	1.000	1.000	1.000	1.000	1.000	1.000	0.000	0.000	0.000	73438	1700061F	chr2	+	NR_03818	.	lncRNA	.	RIKEN cDN	1700061F1
10701	Gata3	0.494	1.419	0.701	0.000	0.180	0.297	3.249	2.232	2.737	14462	Gata3	chr2	-	NM_0080	P8393	protein_cc	Gata-3| ja	GATA bind	Gata3
10702	4930412O	1.572	0.636	1.000	0.304	1.000	0.423	0.000	0.653	0.000	381347	4930412O	chr2	-	NR_02425	.	lncRNA	ENSMUSG	RIKEN cDN	4930412O
10703	9230102O	1.000	1.000	1.000	1.000	1.000	1.000	0.000	0.000	0.000	77682	9230102O	chr2	-	NR_04051	.	lncRNA	.	RIKEN cDN	9230102O
10704	Taf3	1.309	0.707	0.926	0.011	0.005	0.136	8.211	8.600	8.100	209361	Taf3	chr2	-	NM_0277	P23848	protein_cc	140kDa| 4	TATA-box	Taf3
10705	Atp5c1	0.571	1.002	0.572	0.000	0.985	0.019	10.980	10.171	10.173	11949	Atp5c1	chr2	-	NM_0206	P4769	protein_cc	1700094F(	ATP synth	Atp5c1
10706	Kin	0.856	0.925	0.792	0.014	0.030	0.054	7.346	7.122	7.009	16588	Kin	chr2	+	NM_0252	P14011	protein_cc	Kin17	Kin17 DNA	Kin
10707	Itih2	0.795	0.637	0.507	0.050	0.001	0.056	12.107	11.777	11.126	16425	Itih2	chr2	-	NM_0105	P8218	protein_cc	AI747202|	inter-alph	Itih2
10708	Itih5	0.355	1.475	0.523	0.000	0.045	0.042	7.452	5.956	6.517	209378	Itih5	chr2	+	NM_1724	P15185	protein_cc	4631408O	inter-alph	Itih5
10709	Sfmbt2	0.865	0.290	0.251	0.787	0.251	0.077	2.749	2.539	0.756	353282	Sfmbt2	chr2	+	NM_0011	P16967	protein_cc	D2Wsu23e	Scm-like w	Sfmbt2
10710	Gm25285	1.000	1.000	1.000	1.000	1.000	1.000	0.000	0.000	0.000	10479737	Gm25285	chr2	+	NR_13034	.	miRNA	Mir466c-3	predicted	Gm25285
10711	Prkcq	2.779	1.292	3.591	0.133	0.474	0.080	2.642	4.117	4.487	18761	Prkcq	chr2	+	NM_0088	P10441	protein_cc	A130035A	protein kir	Prkcq
10712	8030442B	1.795	7.909	14.194	0.386	0.002	0.038	0.355	1.198	4.182	77543	8030442B	chr2	-	NR_04061	.	lncRNA	.	RIKEN cDN	8030442B
10713	Gm13293	47.276	0.378	17.855	0.005	0.024	0.024	0.003	5.566	4.161	10050314	Gm13293	chr2	+	NR_04036	.	lncRNA	OTTMUSG	predicted	Gm13293
10714	Pfkfb3	34.797	0.507	17.648	0.003	0.051	0.106	6.052	11.173	10.194	170768	Pfkfb3	chr2	-	NM_0011	P672	protein_cc	E330010H	6-phospho	Pfkfb3
10715	Rbm17	1.176	0.835	0.982	0.526	0.523	0.938	9.415	9.649	9.388	76938	Rbm17	chr2	-	NM_1528	P2489	protein_cc	2700027J(	RNA bindi	Rbm17
10716	Il2ra	11.528	0.288	3.319	0.039	0.091	0.383	0.345	3.872	2.076	16184	Il2ra	chr2	+	NM_0083	P7975	protein_cc	CD25| Il2r	interleukir	Il2ra
10717	Il15ra	1.314	0.679	0.891	0.048	0.041	0.735	7.814	8.208	7.648	16169	Il15ra	chr2	+	NM_0012	P14727	protein_cc	AA690181	interleukir	Il15ra
10718	Fbxo18	0.831	0.791	0.658	0.328	0.153	0.268	6.610	6.343	6.006	50755	Fbxo18	chr2	-	NM_0157	P4344	protein_cc	AU01575E	F-box prot	Fbxo18
10719	Ankrd16	0.908	1.110	1.008	0.777	0.678	0.983	5.452	5.314	5.464	320816	Ankrd16	chr2	+	NM_1772	P20871	protein_cc	2810455F(	ankyrin re	Ankrd16
10720	Itga8	0.160	0.398	0.064	0.001	0.102	0.024	5.482	2.839	1.511	241226	Itga8	chr2	-	NM_0010	P762	protein_cc	AI447669	integrin al	Itga8
10721	E030013I1	0.373	0.981	0.366	0.018	1.000	0.140	1.450	0.027	0.000	338535	E030013I1	chr2	+	NR_04035	.	lncRNA	.	RIKEN cDN	E030013I1
10722	Fam188a	1.374	0.914	1.257	0.131	0.225	0.474	7.390	7.849	7.720	66960	Fam188a	chr2	-	NM_0241	P9212	protein_cc	1810041E	family wit	Fam188a
10723	Pter	0.104	2.965	0.307	0.000	0.058	0.062	9.034	5.765	7.333	19212	Pter	chr2	+	NM_0013	P13055	protein_cc	AI790318|	phosphotr	Pter
10724	C1ql3	0.167	1.473	0.245	0.000	0.467	0.055	6.442	3.856	4.416	227580	C1ql3	chr2	-	NM_1531	P23599	protein_cc	1110065A	C1q-like 3	C1ql3
10725	Rsu1	0.684	0.981	0.671	0.110	0.779	0.319	7.470	6.922	6.894	20163	Rsu1	chr2	-	NM_0091	P9761	protein_cc	rsp-1| Rsu	Ras suppre	Rsu1

10726	Cubn	0.239	2.625	0.626	0.017	0.187	0.537	2.132	0.065	1.457	65969	Cubn	chr2	-	NM_0010	P9055	protein_cc	AA408369	cubilin (int	Cubn
10727	Trdmt1	1.156	0.696	0.804	0.416	0.072	0.348	3.953	4.162	3.639	13434	Trdmt1	chr2	-	NM_0100	P19293	protein_cc	Dnmt2| R	tRNA aspa	Trdmt1
10728	Vim	1.924	0.947	1.822	0.034	0.759	0.049	8.411	9.355	9.276	22352	Vim	chr2	+	NM_0117	P20697	protein_cc	.	vimentin	Vim
10729	St8sia6	1.603	1.647	2.641	0.292	0.212	0.178	0.001	0.681	1.402	241230	St8sia6	chr2	-	NM_1458	P26587	protein_cc	1700007J	ST8 alpha-	St8sia6
10730	Hacd1	0.430	1.693	0.728	0.004	0.160	0.468	7.685	6.468	7.228	30963	Hacd1	chr2	-	NM_0139	P3996	protein_cc	Ptpla	3-hydroxy	Hacd1
10731	Stamos	0.632	1.253	0.792	0.084	0.242	0.385	3.222	2.560	2.885	70957	Stamos	chr2	-	NR_03816	.	lncRNA	4921530L	signal trar	Stamos
10732	Stam	1.640	0.721	1.182	0.068	0.143	0.188	6.920	7.633	7.161	20844	Stam	chr2	+	NM_0114	P3052	protein_cc	STAM1	signal trar	Stam
10733	Tmem236	1.572	0.636	1.000	0.304	1.000	0.423	0.000	0.653	0.000	625286	Tmem236	chr2	+	NM_0010	P6238	protein_cc	2010003H	transmem	Tmem236
10734	Mrc1	0.154	0.716	0.110	0.000	0.001	0.001	8.916	6.214	5.731	17533	Mrc1	chr2	+	NM_0086	P19926	protein_cc	AW25968	mannose	Mrc1
10735	Slc39a12	1.000	1.000	1.000	1.000	1.000	1.000	0.000	0.000	0.000	277468	Slc39a12	chr2	+	NM_0010	P14138	protein_cc	AW04693	solute car	Slc39a12
10736	Cacnb2	0.984	0.254	0.250	0.982	0.339	0.009	2.392	2.368	0.390	12296	Cacnb2	chr2	+	NM_0231	P11669	protein_cc	AW06038	calcium ch	Cacnb2
10737	Gm13315	1.589	1.186	1.885	0.298	0.707	0.423	0.000	0.669	0.915	625342	Gm13315	chr2	+	NR_02849	.	misc_RNA	OTTMUSG	predicted	Gm13315
10738	Nsun6	1.949	0.611	1.190	0.007	0.013	0.609	3.969	4.932	4.221	74455	Nsun6	chr2	-	NM_0289	P18226	protein_cc	4933403D	NOL1/NOI	Nsun6
10739	Arl5b	0.859	0.631	0.542	0.188	0.041	0.107	6.573	6.354	5.690	75869	Arl5b	chr2	+	NM_0294	P5281	protein_cc	4930587A	ADP-ribos	Arl5b
10740	Plxdc2	1.546	1.485	2.296	0.053	0.149	0.179	2.618	3.246	3.817	67448	Plxdc2	chr2	+	NM_0261	P20650	protein_cc	1200007L	plexin dor	Plxdc2
10741	4930515L	1.000	1.000	1.000	1.000	1.000	1.000	0.000	0.000	0.000	75069	4930515L	chr2	-	NR_04063	.	lncRNA	.	RIKEN cDN	4930515L
10742	Nebl	1.000	1.000	1.000	1.000	1.000	1.000	0.000	0.000	0.000	74103	Nebl	chr2	-	NM_0287	P6321	protein_cc	1200007O	nebulette	Nebl
10743	H2afb1	1.000	1.000	1.000	1.000	1.000	1.000	0.000	0.000	0.000	68231	H2afb1	chr2	-	NM_0266	P17032	protein_cc	1700113O	H2A histo	H2afb1
10744	A930004D	0.109	6.257	0.683	0.007	0.003	0.411	3.978	0.782	3.428	77940	A930004D	chr2	-	NR_02837	.	lncRNA	AU022051	RIKEN cDN	A930004D
10745	Gm17762	0.375	2.681	1.006	0.000	0.185	0.994	1.463	0.049	1.471	10003473	Gm17762	chr2	+	NR_02837	.	lncRNA	.	predicted	Gm17762
10746	Skida1	0.204	0.603	0.123	0.001	1.000	0.015	3.024	0.730	0.000	72668	Skida1	chr2	-	NM_0283	P17073	protein_cc	2810030E	SKI/DACH	Skida1
10747	Mllt10	0.595	0.716	0.426	0.003	0.056	0.018	8.560	7.811	7.329	17354	Mllt10	chr2	+	NM_0108	P5273	protein_cc	Af10| B13	myeloid/l	Mllt10
10748	Dnajc1	1.204	0.816	0.983	0.286	0.184	0.928	9.547	9.815	9.522	13418	Dnajc1	chr2	-	NM_0071	P3550	protein_cc	4733401K	DnaJ heat	Dnajc1
10749	Commd3	1.068	1.071	1.144	0.003	0.001	0.009	9.903	9.998	10.097	12238	Commd3	chr2	+	NM_1477	P17276	protein_cc	AW55081	COMM do	Commd3
10750	Bmi1	1.161	1.046	1.214	0.502	0.819	0.030	7.677	7.892	7.957	12151	Bmi1	chr2	+	NM_0075	P4939	protein_cc	AW54669	Bmi1 poly	Bmi1
10751	Spag6l	1.000	1.000	1.000	1.000	1.000	1.000	0.000	0.000	0.000	50525	Spag6l	chr2	-	NM_0010	P24580	protein_cc	PF16| Spa	sperm ass	Spag6l
10752	Gm3363	1.000	1.000	1.000	1.000	1.000	1.000	0.000	0.000	0.000	10004147	Gm3363	chr2	-	NR_12607	.	lncRNA	10004147	predicted	Gm3363
10753	Carlr	1.000	1.000	1.000	1.000	1.000	1.000	0.000	0.000	0.000	66774	Carlr	chr2	-	NR_13125	.	lncRNA	4933439G	cardiac an	Carlr
10754	Pip4k2a	1.023	0.934	0.956	0.853	0.775	0.908	5.420	5.453	5.354	18718	Pip4k2a	chr2	-	NM_0088	P12728	protein_cc	AW74291	phosphati	Pip4k2a
10755	4930426L	1.015	2.326	2.360	0.000	0.182	0.423	0.000	0.021	1.239	74629	4930426L	chr2	+	NR_02432	.	lncRNA	.	RIKEN cDN	4930426L
10756	Armc3	0.793	0.646	0.513	0.601	0.490	0.110	1.458	1.124	0.494	70882	Armc3	chr2	+	NM_0010	P26762	protein_cc	4921513G	armadillo	Armc3
10757	4930447M	1.000	1.000	1.000	1.000	1.000	1.000	0.000	0.000	0.000	108871	4930447M	chr2	+	NR_12656	.	lncRNA	.	RIKEN cDN	4930447M
10758	Msrb2	0.692	1.647	1.140	0.164	0.014	0.671	7.673	7.143	7.863	76467	Msrb2	chr2	+	NM_0296	P24321	protein_cc	2310050L	methionin	Msrb2
10759	Ptf1aos	1.000	1.000	1.000	1.000	1.000	1.000	0.000	0.000	0.000	10263710	Ptf1aos	chr2	-	NR_13192	.	lncRNA	Gm13336	pancreas s	Ptf1aos
10760	Ptf1a	1.000	1.000	1.000	1.000	1.000	1.000	0.000	0.000	0.000	19213	Ptf1a	chr2	+	NM_0188	P18862	protein_cc	bHLHa29	pancreas s	Ptf1a
10761	4921504E	1.580	0.633	1.000	0.305	1.000	0.423	0.000	0.660	0.000	70909	4921504E	chr2	+	NM_0276	P3610	protein_cc	4921522E	RIKEN cDN	4921504E
10762	Gm3230	0.070	4.421	0.310	0.067	0.186	0.402	3.965	0.130	2.275	10004124	Gm3230	chr2	-	NR_03364	.	lncRNA	10004124	predicted	Gm3230
10763	Otud1	0.375	1.389	0.522	0.054	0.539	0.086	5.247	3.834	4.308	71198	Otud1	chr2	+	NM_0277	P17626	protein_cc	4933428L	OTU doma	Otud1
10764	Etl4	0.216	2.166	0.468	0.002	0.097	0.044	6.344	4.133	5.248	208618	Etl4	chr2	+	NM_0010	P10459	protein_cc	6620402G	enhancer	Etl4
10765	Arhgap21	0.774	1.365	1.057	0.053	0.046	0.806	7.582	7.212	7.661	71435	Arhgap21	chr2	-	NM_0010	P16469	protein_cc	5530401C	Rho GTPas	Arhgap21
10766	Gm13375	0.624	3.159	1.972	0.395	0.028	0.376	2.925	2.245	3.904	433408	Gm13375	chr2	+	NR_03322	.	lncRNA	OTTMUSG	predicted	Gm13375
10767	Enkur	1.344	1.343	1.805	0.431	0.314	0.396	1.073	1.500	1.925	71233	Enkur	chr2	-	NM_0277	P12666	protein_cc	4933434I	enkurin, T	Enkur
10768	Thnsl1	0.960	0.927	0.890	0.759	0.673	0.570	6.544	6.485	6.377	208967	Thnsl1	chr2	+	NM_0010	P18350	protein_cc	AW41363	threonine	Thnsl1
10769	Gpr158	1.000	1.000	1.000	1.000	1.000	1.000	0.000	0.000	0.000	241263	Gpr158	chr2	+	NM_0010	P1018	protein_cc	5330427M	G protein-	Gpr158
10770	Myo3a	1.000	1.000	1.000	1.000	1.000	1.000	0.000	0.000	0.000	667663	Myo3a	chr2	+	NM_1484	P17744	protein_cc	9030416P	myosin III	Myo3a
10771	Gad2	1.284	6.647	8.533	0.714	0.048	0.206	1.189	1.550	4.282	14417	Gad2	chr2	+	NM_0080	P23440	protein_cc	6330404F	glutamic a	Gad2
10772	Apbb1ip	1.287	0.928	1.194	0.082	0.609	0.515	5.547	5.912	5.804	54519	Apbb1ip	chr2	+	NM_0194	P18795	protein_cc	9930118P	amyloid b	Apbb1ip
10773	Pdss1	1.357	0.926	1.257	0.014	0.308	0.226	5.720	6.161	6.050	56075	Pdss1	chr2	+	NM_0195	P21208	protein_cc	2610203G	prenyl (so	Pdss1
10774	Abi1	1.574	0.849	1.336	0.019	0.324	0.384	7.995	8.649	8.413	11308	Abi1	chr2	-	NM_0073	P13107	protein_cc	abi-1| E3B	abl-intera	Abi1
10775	Acbd5	0.150	1.559	0.234	0.000	0.110	0.026	10.788	8.055	8.696	74159	Acbd5	chr2	+	NM_0011	P13161	protein_cc	1300014E	acyl-Coen	Acbd5
10776	Mastl	0.939	0.504	0.473	0.938	0.521	0.417	2.625	2.535	1.546	67121	Mastl	chr2	-	NM_0259	P4649	protein_cc	2700091H	microtubu	Mastl
10777	Yme1l1	1.547	0.946	1.462	0.000	0.423	0.092	9.312	9.941	9.861	27377	Yme1l1	chr2	+	NM_0137	P481	protein_cc	Ftsh| FtsH	YME1-like	Yme1l1
10778	4931423N	1.000	1.000	1.000	1.000	1.000	1.000	0.000	0.000	0.000	70981	4931423N	chr2	+	NM_0276	P15560	protein_cc	RP23-54E	RIKEN cDN	4931423N
10779	Nxph2	1.000	1.000	1.000	1.000	1.000	1.000	0.000	0.000	0.000	18232	Nxph2	chr2	+	NM_0087	P18416	protein_cc	.	neurexopl	Nxph2
10780	Spopl	1.265	1.092	1.381	0.413	0.705	0.058	5.665	6.004	6.131	76857	Spopl	chr2	-	NM_0297	P11554	protein_cc	4921517N	speckle-ty	Spopl
10781	Hnmt	0.185	1.284	0.237	0.000	0.410	0.011	8.325	5.888	6.249	140483	Hnmt	chr2	-	NM_0804	P18531	protein_cc	1500031F	histamine	Hnmt
10782	Tbpl2	1.000	1.000	1.000	1.000	1.000	1.000	0.000	0.000	0.000	227606	Tbpl2	chr2	-	NR_11035	.	protein_cc	Gm348| T	TATA box	Tbpl2
10783	Il1f8	1.000	1.000	1.000	1.000	1.000	1.000	0.000	0.000	0.000	69677	Il1f8	chr2	+	NM_0271	P14202	protein_cc	2310043N	interleuki	Il1f8
10784	Il1f9	52.117	0.285	14.855	0.004	0.013	0.028	0.815	6.519	4.708	215257	Il1f9	chr2	+	NM_1535	P21608	protein_cc	Il36g	interleuki	Il1f9
10785	Il1f6	6.931	0.532	3.691	0.005	0.058	0.130	0.002	2.795	1.885	54448	Il1f6	chr2	+	NM_0194	P13405	protein_cc	Fil1| IL-1H	interleuki	Il1f6
10786	Il1f5	1.000	1.000	1.000	1.000	1.000	1.000	0.000	0.000	0.000	54450	Il1f5	chr2	+	NM_0011	P17025	protein_cc	AI413231|	interleuki	Il1f5
10787	Il1f10	1.000	1.000	1.000	1.000	1.000	1.000	0.000	0.000	0.000	215274	Il1f10	chr2	+	NM_1530	P12210	protein_cc	.	interleuki	Il1f10
10788	Il1rn	82.308	0.591	48.643	0.000	0.010	0.048	6.705	13.068	12.309	16181	Il1rn	chr2	+	NM_0311	P25887	protein_cc	F630041P	interleuki	Il1rn
10789	Psd4	1.489	1.238	1.843	0.369	0.425	0.317	4.328	4.902	5.210	215632	Psd4	chr2	+	NM_1776	P3029	protein_cc	BC046518	pleckstrin	Psd4
10790	Pax8	1.000	1.000	1.000	1.000	1.000	1.000	0.000	0.000	0.000	18510	Pax8	chr2	-	NM_0110	P14486	protein_cc	Pax-8	paired box	Pax8
10791	Cacna1b	1.603	1.658	2.658	0.292	0.404	0.423	0.001	0.681	1.411	12287	Cacna1b	chr2	-	NM_0010	P24204	protein_cc	alpha(1B)|	calcium ch	Cacna1b
10792	Ehmt1	0.680	0.965	0.656	0.002	0.583	0.069	8.354	7.798	7.746	77683	Ehmt1	chr2	-	NM_0010	P19300	protein_cc	9230102N	euchroma	Ehmt1
10793	Arrdc1	1.512	1.178	1.780	0.093	0.542	0.401	4.551	5.147	5.383	215705	Arrdc1	chr2	-	NM_0011	P9160	protein_cc	AI957342|	arrestin do	Arrdc1
10794	Zmynd19	1.668	0.827	1.379	0.017	0.436	0.513	3.373	4.111	3.837	67187	Zmynd19	chr2	+	NM_0260	P5531	protein_cc	2700064H	zinc finger	Zmynd19
10795	Dph7	2.016	0.853	1.720	0.021	0.205	0.210	3.374	4.385	4.156	67228	Dph7	chr2	+	NM_0260	P26657	protein_cc	2810443J	diphthami	Dph7
10796	Mrpl41	1.083	1.668	1.807	0.299	0.000	0.012	8.269	8.384	9.122	107733	Mrpl41	chr2	-	NM_0010	P3537	protein_cc	MRP-L27|	mitochond	Mrpl41
10797	Pnpla7	0.602	1.756	1.057	0.013	0.021	0.777	9.487	8.755	9.567	241274	Pnpla7	chr2	+	NM_1462	P26602	protein_cc	BC027342	patatin-lik	Pnpla7
10798	Nsmf	0.271	1.913	0.518	0.001	0.002	0.059	8.710	6.824	7.760	56876	Nsmf	chr2	-	NM_0010	P10129	protein_cc	Jacob| Ne	NMDA rec	Nsmf
10799	Entpd8	0.105	1.993	0.210	0.001	0.043	0.054	8.297	5.051	6.046	72090	Entpd8	chr2	+	NM_0280	P4443	protein_cc	.	ectonucle	Entpd8
10800	Noxa1	0.082	2.508	0.206	0.001	0.119	0.051	7.627	4.018	5.344	241275	Noxa1	chr2	-	NM_0011	P8775	protein_cc	NY-CO-31	NADPH ox	Noxa1
10801	Nrarp	0.596	2.442	1.455	0.011	0.033	0.397	3.810	3.064	4.352	67122	Nrarp	chr2	+	NM_0259	P7178	protein_cc	2700054M	Notch-reg	Nrarp
10802	Tor4a	1.154	0.647	0.746	0.776	0.490	0.487	4.226	4.433	3.804	227612	Tor4a	chr2	-	NM_1461	P17906	protein_cc	A830007P	torsin fam	Tor4a
10803	Nelfb	1.113	1.133	1.261	0.295	0.162	0.205	6.758	6.912	7.092	58202	Nelfb	chr2	-	NM_0013	P3209	protein_cc	A730008L	negative e	Nelfb
10804	4933433C	1.000	1.000	1.000	1.000	1.000	1.000	0.000	0.000	0.000	.	4933433C	chr2	-	NM_0289	P26231	.	.	.	4933433C
10805	Fam166a	2.471	0.815	2.014	0.000	0.026	0.057	9.185	10.490	10.196	68222	Fam166a	chr2	+	NM_0266	P21269	protein_cc	4931415M	family wit	Fam166a
10806	Tubb4b	2.433	0.808	1.965	0.000	0.028	0.052	9.455	10.737	10.429	227613	Tubb4b	chr2	-	NM_1461	P8622	protein_cc	4930542G	tubulin, be	Tubb4b
10807	Slc34a3	1.000	1.000	1.000	1.000	1.000	1.000	0.000	0.000	0.000	142681	Slc34a3	chr2	-	NM_0808	P13659	protein_cc	AI649385|	solute car	Slc34a3
10808	Rnf224	1.000	1.000	1.000	1.000	1.000	1.000	0.000	0.000	0.000	329360	Rnf224	chr2	-	NM_0010	P3496	protein_cc	Gm757	ring finger	Rnf224
10809	Cysrt1	0.716	0.995	0.712	0.182	1.000	0.423	0.490	0.008	0.000	67859	Cysrt1	chr2	-	NM_0264	P22122	protein_cc	2310002J	cysteine ri	Cysrt1
10810	Rnf208	0.896	0.861	0.771	0.800	0.706	0.685	0.834	0.675	0.460	68846	Rnf208	chr2	+	NM_1768	P1840	protein_cc	1110061N	ring finger	Rnf208
10811	Ndor1	1.720	1.238	2.130	0.000	0.007	0.013	3.762	4.544	4.853	78797	Ndor1	chr2	-	NM_0012	P14020	protein_cc	4930447P	NADPH de	Ndor1
10812	Tmem203	0.643	1.586	1.020	0.019	0.013	0.928	7.265	6.628	7.293	227615	Tmem203	chr2	+	NM_1773	P16361	protein_cc	C730025P	transmem	Tmem203
10813	Tprn	1.862	0.794	1.478	0.271	0.631	0.470	4.124	5.021	4.687	97031	Tprn	chr2	+	NM_1752	P2624	protein_cc	C430004E	taperin	Tprn
10814	Ssna1	0.883	1.555	1.372	0.302	0.028	0.164	6.365	6.186	6.822	68475	Ssna1	chr2	-	NM_0234	P1666	protein_cc	1110003H	Sjogren's s	Ssna1
10815	Anapc2	0.517	1.469	0.760	0.009	0.099	0.347	7.773	6.822	7.377	99152	Anapc2	chr2	+	NM_1753	P15754	protein_cc	9230107K	anaphase	Anapc2
10816	Tmem210	1.000	1.000	1.000	1.000	1.000	1.000	0.000	0.000	0.000	78217	Tmem210	chr2	+	NM_0300	P11921	protein_cc	4930571C	transmem	Tmem210

10817	Lrrc26	0.567	1.777	1.008	0.186	0.000	0.988	0.846	0.027	0.857	227618	Lrrc26	chr2	+	NM_1461	P4412	protein_cc	.	leucine ric	Lrrc26
10818	Grin1	1.019	2.633	2.682	0.000	0.030	0.179	0.000	0.027	1.424	14810	Grin1	chr2	-	NM_0011	P13817	protein_cc	GluN1| Gl	glutamate	Grin1
10819	Grin1os	1.010	1.825	1.844	0.000	0.182	0.423	0.000	0.015	0.883	320354	Grin1os	chr2	+	NM_0012	P6010	protein_cc	E130003G	glutamate	Grin1os
10820	AA543186	0.899	1.872	1.684	0.798	0.375	0.678	1.328	1.175	2.080	10027221	AA543186	chr2	-	NR_02744	.	lncRNA		expressed	AA543186
10821	Man1b1	0.796	0.834	0.664	0.000	0.118	0.092	7.838	7.509	7.247	227619	Man1b1	chr2	+	NM_0010	P2479	protein_cc	E430019H	mannosid	Man1b1
10822	Dpp7	0.660	1.303	0.861	0.001	0.015	0.285	6.648	6.050	6.432	83768	Dpp7	chr2	-	NM_0318	P10535	protein_cc	Dpp2| DPI	dipeptidyl	Dpp7
10823	Uap1l1	1.538	0.720	1.107	0.095	0.114	0.822	4.597	5.218	4.744	227620	Uap1l1	chr2	-	NM_0010	P20361	protein_cc	5730445F	UDP-N-act	Uap1l1
10824	Sapcd2	1.005	1.405	1.412	0.000	0.182	0.423	0.000	0.008	0.498	72080	Sapcd2	chr2	+	NM_0010	P25992	protein_cc	2010317E	suppresso	Sapcd2
10825	Entpd2	0.560	0.989	0.554	0.159	0.980	0.096	4.894	4.058	4.041	12496	Entpd2	chr2	+	NM_0098	P2881	protein_cc	Cd39l1| N	ectonucle	Entpd2
10826	Npdc1	4.503	0.621	2.797	0.002	0.028	0.120	4.181	6.352	5.665	18146	Npdc1	chr2	+	NM_0087	P22630	protein_cc	AI314472|	neural pro	Npdc1
10827	Fut7	2.109	1.224	2.581	0.390	0.782	0.481	0.404	1.480	1.772	14347	Fut7	chr2	+	NM_0012	P15594	protein_cc	AI853193|	fucosyltra	Fut7
10828	Abca2	0.138	1.326	0.184	0.002	0.114	0.049	8.960	6.107	6.514	11305	Abca2	chr2	+	NM_0073	P22727	protein_cc	Abc2| AI4	ATP-bindi	Abca2
10829	Clic3	0.273	0.447	0.122	0.017	1.000	0.059	3.036	1.162	0.000	69454	Clic3	chr2	+	NM_0270	P22345	protein_cc	2300003G	chloride ir	Clic3
10830	BC029214	0.122	2.730	0.334	0.000	0.005	0.008	7.107	4.075	5.524	227622	BC029214	chr2	-	NM_1535	P21036	protein_cc	D930050G	cDNA seq	BC029214
10831	Ptgds	0.397	0.713	0.283	0.002	0.069	0.044	6.326	4.994	4.506	19215	Ptgds	chr2	+	NM_0089	P19287	protein_cc	21kDa| L-I	prostaglar	Ptgds
10832	Lcn12	1.472	0.219	0.323	0.505	1.000	0.002	1.630	2.188	0.000	77701	Lcn12	chr2	-	NM_0299	P23616	protein_cc	9230102N	lipocalin 1	Lcn12
10833	C8g	0.598	1.081	0.647	0.000	0.514	0.092	11.940	11.199	11.311	69379	C8g	chr2	-	NM_0012	P11018	protein_cc	.	compleme	C8g
10834	Fbxw5	0.710	1.412	1.003	0.208	0.181	0.989	7.267	6.773	7.271	30839	Fbxw5	chr2	+	NM_0139	P26755	protein_cc	AI159739|	F-box and	Fbxw5
10835	Traf2	3.334	0.696	2.321	0.001	0.066	0.125	4.708	6.445	5.923	22030	Traf2	chr2	-	NM_0012	P18293	protein_cc	AI325259	TNF recep	Traf2
10836	Edf1	0.938	1.527	1.433	0.562	0.005	0.027	10.895	10.803	11.413	59022	Edf1	chr2	+	NM_0215	P1801	protein_cc	0610008L	endothelia	Edf1
10837	Mamdc4	0.626	1.742	1.090	0.241	0.397	0.931	2.476	1.800	2.600	381352	Mamdc4	chr2	-	NM_0010	P16719	protein_cc	Gm995	MAM dom	Mamdc4
10838	Phpt1	0.473	1.670	0.789	0.001	0.005	0.030	8.936	7.854	8.594	75454	Phpt1	chr2	-	NM_0292	P3542	protein_cc	1700008C	phosphoh	Phpt1
10839	Gm996	1.603	0.824	1.320	0.462	0.744	0.664	0.432	1.113	0.833	381353	Gm996	chr2	-	NM_0010	P25107	protein_cc	.	predicted	Gm996
10840	Rabl6	1.356	0.904	1.225	0.002	0.025	0.075	7.354	7.793	7.647	227624	Rabl6	chr2	-	NM_0010	P2706	protein_cc	B230208H	RAB, mem	Rabl6
10841	Ccdc183	1.005	1.405	1.412	0.000	0.182	0.423	0.000	0.008	0.498	77058	Ccdc183	chr2	-	NM_0298	P13173	protein_cc	4921530D	coiled-coil	Ccdc183
10842	Tmem141	0.225	2.300	0.517	0.000	0.000	0.022	8.066	5.913	7.115	51875	Tmem141	chr2	-	NM_0010	P3462	protein_cc	1110065P	transmem	Tmem141
10843	Fcna	0.712	0.838	0.597	0.001	0.238	0.110	9.106	8.615	8.361	14133	Fcna	chr2	-	NM_0079	P19030	protein_cc	Fcn1	ficolin A	Fcna
10844	Lcn8	0.382	0.982	0.375	0.182	1.000	0.423	1.416	0.027	0.000	78076	Lcn8	chr2	+	NM_0331	P6828	protein_cc	9230106L	lipocalin 8	Lcn8
10845	Lcn5	2.722	0.367	1.000	0.306	1.000	0.423	0.001	1.445	0.000	13863	Lcn5	chr2	+	NM_0012	P4751	protein_cc	E-RABP| E	lipocalin 5	Lcn5
10846	Lcn6	1.000	1.000	1.000	1.000	1.000	1.000	0.000	0.000	0.000	620709	Lcn6	chr2	+	NM_1778	P14888	protein_cc	.	lipocalin 6	Lcn6
10847	Lcn10	1.000	1.000	1.000	1.000	1.000	1.000	0.000	0.000	0.000	332578	Lcn10	chr2	+	NM_1780	P1148	protein_cc	9230112J	lipocalin 1	Lcn10
10848	Obp2a	1.713	13.592	23.288	0.244	0.000	0.015	0.002	0.779	4.543	227627	Obp2a	chr2	+	NM_1535	P14252	protein_cc	BC027556	odorant b	Obp2a
10849	Bmyc	0.368	3.013	1.109	0.004	0.148	0.890	5.710	4.268	5.860	107771	Bmyc	chr2	+	NM_0233	P18563	protein_cc	2900002K	brain expr	Bmyc
10850	Obp2b	1.000	1.000	1.000	1.000	1.000	1.000	0.000	0.000	0.000	383678	Obp2b	chr2	+	NM_0010	P6424	protein_cc	Lcn14| OT	odorant b	Obp2b
10851	Lcn3	1.000	1.000	1.000	1.000	1.000	1.000	0.000	0.000	0.000	16820	Lcn3	chr2	+	NM_0106	P14942	protein_cc	Vnsp1	lipocalin 3	Lcn3
10852	Lcn11	1.000	1.000	1.000	1.000	1.000	1.000	0.000	0.000	0.000	227630	Lcn11	chr2	+	NM_0011	P9679	protein_cc	Gm109	lipocalin 1	Lcn11
10853	Gm13539	1.000	1.000	1.000	1.000	1.000	1.000	0.000	0.000	0.000	620858	Gm13539	chr2	+	NR_04534	.	lncRNA	OTTMUSG	predicted	Gm13539
10854	Glt6d1	1.000	1.000	1.000	1.000	1.000	1.000	0.000	0.000	0.000	71103	Glt6d1	chr2	-	NM_0010	P21383	protein_cc	4933411C	glycosyltra	Glt6d1
10855	Lcn9	1.000	1.000	1.000	1.000	1.000	1.000	0.000	0.000	0.000	77704	Lcn9	chr2	+	NM_0299	P19263	protein_cc	9230102I1	lipocalin 9	Lcn9
10856	Sohlh1	1.000	1.000	1.000	1.000	1.000	1.000	0.000	0.000	0.000	227631	Sohlh1	chr2	-	NM_0010	P26504	protein_cc	Gm110| N	spermatog	Sohlh1
10857	Kcnt1	1.000	1.000	1.000	1.000	1.000	1.000	0.000	0.000	0.000	227632	Kcnt1	chr2	+	NM_0011	P24866	protein_cc	C030030G	potassium	Kcnt1
10858	Camsap1	2.337	0.644	1.504	0.000	0.001	0.106	6.894	8.119	7.483	227634	Camsap1	chr2	-	NM_0012	P58	protein_cc	9530003A	calmoduli	Camsap1
10859	Ubac1	0.921	0.927	0.854	0.262	0.407	0.444	8.058	7.939	7.830	98766	Ubac1	chr2	-	NM_1338	P13010	protein_cc	1110033G	ubiquitin a	Ubac1
10860	Nacc2	0.463	0.958	0.443	0.020	0.823	0.101	8.413	7.301	7.239	67991	Nacc2	chr2	-	NM_0264	P6656	protein_cc	0610020I	nucleus ac	Nacc2
10861	C330006A	0.383	1.346	0.516	0.005	0.033	0.122	10.584	9.200	9.629	109299	C330006A	chr2	-	NM_0012	P8934	protein_cc	AI551216	RIKEN cDN	C330006A
10862	Lhx3	42.353	0.165	6.968	0.000	0.001	0.201	0.003	5.407	2.803	16871	Lhx3	chr2	-	NM_0010	P2143	protein_cc	Lim3| mLi	LIM home	Lhx3
10863	Qsox2	4.967	0.552	2.743	0.002	0.021	0.034	3.999	6.312	5.455	227638	Qsox2	chr2	-	NM_1535	P8804	protein_cc	BC030934	quiescin Q	Qsox2
10864	4932418E	1.000	1.000	1.000	1.000	1.000	1.000	0.000	0.000	0.000	.	4932418E	chr2	-	NM_1778	P13527	.	.	.	4932418E2
10865	Gpsm1	1.096	0.926	1.015	0.493	0.613	0.917	6.509	6.642	6.531	67839	Gpsm1	chr2	+	NM_0011	P17704	protein_cc	1810037C	G-protein	Gpsm1
10866	Dnlz	0.805	1.189	0.957	0.003	0.001	0.403	8.106	7.794	8.043	52838	Dnlz	chr2	-	NM_0011	P21335	protein_cc	1110034G	DNL-type	Dnlz
10867	Card9	1.000	1.000	1.000	1.000	1.000	1.000	0.000	0.000	0.000	332579	Card9	chr2	-	NM_0010	P10436	protein_cc	Gm782	caspase re	Card9
10868	Snapc4	1.762	0.790	1.391	0.041	0.209	0.540	4.617	5.434	5.093	227644	Snapc4	chr2	-	NM_0012	P5384	protein_cc	5730436L	small nucl	Snapc4
10869	Sdccag3	1.228	0.906	1.112	0.134	0.379	0.207	7.641	7.937	7.795	68112	Sdccag3	chr2	-	NM_0010	P7352	protein_cc	C330016H	serologica	Sdccag3
10870	Pmpca	0.844	1.288	1.087	0.320	0.239	0.678	8.764	8.520	8.885	66865	Pmpca	chr2	+	NM_1731	P20994	protein_cc	1200002L	peptidase	Pmpca
10871	Inpp5e	0.954	1.201	1.146	0.847	0.515	0.621	7.875	7.806	8.071	64436	Inpp5e	chr2	-	NM_0012	P25172	protein_cc	1200002L	inositol po	Inpp5e
10872	Sec16a	1.365	0.876	1.196	0.001	0.020	0.118	8.195	8.644	8.453	227648	Sec16a	chr2	-	NM_1531	P19374	protein_cc	AU024582	SEC16 hor	Sec16a
10873	Notch1	0.925	0.719	0.664	0.448	0.104	0.185	7.401	7.288	6.811	18128	Notch1	chr2	-	NM_0087	P18091	protein_cc	9930111A	notch 1	Notch1
10874	Egfl7	0.635	1.983	1.259	0.009	0.024	0.467	8.151	7.496	8.484	353156	Egfl7	chr2	+	NM_1784	P14207	protein_cc	VE-statin|	EGF-like d	Egfl7
10875	Agpat2	0.509	0.937	0.477	0.000	0.202	0.010	10.992	10.019	9.925	67512	Agpat2	chr2	-	NM_0262	P977	protein_cc	2510002J	1-acylglyc	Agpat2
10876	Fam69b	0.211	3.540	0.745	0.108	0.132	0.770	3.014	0.766	2.590	56279	Fam69b	chr2	+	NM_0198	P23035	protein_cc	AB030186	family wit	Fam69b
10877	Snhg7	1.897	0.611	1.159	0.130	0.196	0.568	4.387	5.310	4.599	72091	Snhg7	chr2	-	NR_02406	.	lncRNA	2610002F	small nucl	Snhg7
10878	Snora43	1.000	1.000	1.000	1.000	1.000	1.000	0.000	0.000	0.000	10030695	Snora43	chr2	-	NR_02857	.	snoRNA	ACA43	small nucl	Snora43
10879	Snora17	0.597	4.155	2.482	0.197	0.029	0.283	0.799	0.056	2.111	10030695	Snora17	chr2	-	NR_02857	.	snoRNA	ACA17	small nucl	Snora17
10880	Lcn4	1.572	0.636	1.000	0.304	1.000	0.423	0.000	0.653	0.000	16821	Lcn4	chr2	-	NM_0106	P23368	protein_cc	A630045N	lipocalin 4	Lcn4
10881	Abo	1.000	1.000	1.000	1.000	1.000	1.000	0.000	0.000	0.000	80908	Abo	chr2	-	NM_0307	P1310	protein_cc	NAGAT	ABO blood	Abo
10882	Surf6	2.099	0.707	1.484	0.001	0.007	0.032	6.158	7.228	6.728	20935	Surf6	chr2	-	NM_0092	P8285	protein_cc	D2Wsu12	surfeit ger	Surf6
10883	Med22	0.866	1.397	1.211	0.017	0.008	0.208	6.060	5.853	6.336	20933	Med22	chr2	-	NM_0010	P24501	protein_cc	AW21265	mediator	Med22
10884	Rpl7a	1.023	1.435	1.469	0.676	0.021	0.139	9.637	9.670	10.191	27176	Rpl7a	chr2	+	NM_0137	P1623	protein_cc	Surf3	ribosomal	Rpl7a
10885	Surf1	0.633	1.033	0.654	0.002	0.873	0.233	8.406	7.747	7.794	20930	Surf1	chr2	-	NM_0012	P21766	protein_cc	0610010F	surfeit ger	Surf1
10886	Surf2	2.147	0.701	1.505	0.000	0.003	0.047	10.258	11.361	10.848	20931	Surf2	chr2	+	NM_0136	P17665	protein_cc	Surf-2	surfeit ger	Surf2
10887	Surf4	1.746	0.794	1.386	0.000	0.000	0.013	11.189	11.993	11.660	20932	Surf4	chr2	-	NM_0115	P13876	protein_cc	AL033340	surfeit ger	Surf4
10888	Stkld1	1.005	1.405	1.412	0.000	0.182	0.423	0.000	0.008	0.498	279029	Stkld1	chr2	+	NM_1986	P23310	protein_cc	Gm771| S	serine/thr	Stkld1
10889	Rexo4	1.432	0.822	1.177	0.077	0.375	0.634	6.944	7.462	7.178	227656	Rexo4	chr2	-	NM_2072	P938	protein_cc	Gm111| R	REX4, 3'-5	Rexo4
10890	Adamts13	0.540	0.640	0.346	0.073	0.351	0.041	4.661	3.772	3.129	279028	Adamts13	chr2	+	NM_0010	P7903	protein_cc	ADAM-TS	a disinteg	Adamts13
10891	Cacfd1	1.644	0.851	1.399	0.003	0.152	0.144	8.128	8.846	8.613	381356	Cacfd1	chr2	+	NM_0298	P8900	protein_cc	5930434B	calcium ch	Cacfd1
10892	Slc2a6	51.041	0.372	18.999	0.019	0.100	0.261	1.232	6.906	5.480	227659	Slc2a6	chr2	-	NM_1726	P13074	protein_cc	A330096C	solute car	Slc2a6
10893	Tmem8c	1.000	1.000	1.000	1.000	1.000	1.000	0.000	0.000	0.000	66139	Tmem8c	chr2	-	NM_0011	P5012	protein_cc	1110019	transmem	Tmem8c
10894	Adamtsl2	1.000	1.000	1.000	1.000	1.000	1.000	0.000	0.000	0.000	77794	Adamtsl2	chr2	+	NM_0299	P7771	protein_cc	A930008K	ADAMTS-l	Adamtsl2
10895	Fam163b	1.010	1.821	1.840	0.000	0.000	0.002	0.000	0.015	0.880	109349	Fam163b	chr2	-	NM_1754	P14483	protein_cc	C630035N	family wit	Fam163b
10896	Dbhos	1.000	1.000	1.000	1.000	1.000	1.000	0.000	0.000	0.000	329371	Dbhos	chr2	-	NR_04052	.	lncRNA	6430548G	dopamine	Dbhos
10897	Dbh	1.000	1.000	1.000	1.000	1.000	1.000	0.000	0.000	0.000	13166	Dbh	chr2	+	NM_1389	P2610	protein_cc	.	dopamine	Dbh
10898	Sardh	0.209	0.726	0.151	0.000	0.091	0.002	12.575	10.314	9.852	192166	Sardh	chr2	-	NM_1386	P26298	protein_cc	.	sarcosine	Sardh
10899	Vav2	0.451	0.986	0.444	0.182	1.000	0.423	1.170	0.021	0.000	22325	Vav2	chr2	-	NM_0095	P19552	protein_cc	2810040F	vav 2 onco	Vav2
10900	Brd3	0.475	1.056	0.502	0.000	0.563	0.020	9.654	8.580	8.659	67382	Brd3	chr2	-	NM_0233	P2207	protein_cc	2410084F	bromodor	Brd3
10901	Wdr5	1.050	0.969	1.018	0.497	0.858	0.950	9.013	9.084	9.039	140858	Wdr5	chr2	+	NM_0808	P23638	protein_cc	24100080	WD repea	Wdr5
10902	Rxra	0.381	1.470	0.559	0.000	0.000	0.010	11.183	9.790	10.345	20181	Rxra	chr2	+	NM_0113	P26736	protein_cc	9530071D	retinoid X	Rxra
10903	Col5a1	0.282	1.445	0.407	0.007	0.436	0.106	6.424	4.596	5.127	12831	Col5a1	chr2	+	NM_0157	P6393	protein_cc	AI413331	collagen, t	Col5a1
10904	Fcnb	1.000	1.000	1.000	1.000	1.000	1.000	0.000	0.000	0.000	14134	Fcnb	chr2	-	NM_0101	P5553	protein_cc	Fcn2	ficolin B	Fcnb
10905	Olfm1	0.524	1.143	0.599	0.004	0.145	0.080	5.955	5.022	5.215	56177	Olfm1	chr2	+	NM_0010	P21095	protein_cc	AMY| AW	olfactome	Olfm1
10906	Ppp1r26	0.423	0.613	0.259	0.290	1.000	0.423	1.946	0.706	0.000	241289	Ppp1r26	chr2	+	NM_0010	P14196	protein_cc	Gm347| K	protein ph	Ppp1r26
10907	1700007K	7.333	0.216	1.586	0.342	0.384	0.574	1.005	3.879	1.670	69327	1700007K	chr2	-	NM_0270	P18564	protein_cc	Rbest47	RIKEN cDN	1700007K1

10908	Mrps2	1.266	1.091	1.382	0.026	0.405	0.150	8.809	9.150	9.276	118451	Mrps2	chr2	+	NM_0804	P11230	protein_cc	1500019N	mitochond	Mrps2
10909	Gbgt1	1.000	1.000	1.000	1.000	1.000	1.000	0.000	0.000	0.000	227671	Gbgt1	chr2	+	NM_1391	P4000	protein_cc	Fgs| Fs	globoside	Gbgt1
10910	Ralgds	26.068	0.177	4.609	0.011	0.019	0.141	4.196	8.900	6.400	19730	Ralgds	chr2	+	NM_0090	P20657	protein_cc	Gnds| mK	ral guanin	Ralgds
10911	Cel	1.000	1.000	1.000	1.000	1.000	1.000	0.000	0.000	0.000	12613	Cel	chr2	-	NM_0098	P2061	protein_cc	1810036E	carboxyl e	Cel
10912	Gtf3c5	2.760	0.507	1.399	0.053	0.126	0.476	4.078	5.543	4.563	70239	Gtf3c5	chr2	-	NM_0012	P1864	protein_cc	2700084A	general tra	Gtf3c5
10913	Gfi1b	1.014	2.240	2.272	0.000	0.182	0.423	0.000	0.021	1.185	14582	Gfi1b	chr2	-	NM_0081	P18973	protein_cc	Gfi-1B	growth fa	Gfi1b
10914	Tsc1	0.389	1.108	0.431	0.002	0.237	0.050	7.927	6.563	6.712	64930	Tsc1	chr2	+	NM_0228	P372	protein_cc	.	tuberous s	Tsc1
10915	1700026L	1.572	0.636	1.000	0.304	1.000	0.423	0.000	0.653	0.000	.	1700026L	chr2	-	NM_0272	P5019	.	.	.	1700026L
10916	Ak8	0.552	0.990	0.547	0.182	1.000	0.423	0.871	0.015	0.000	68870	Ak8	chr2	+	NM_0010	P383	protein_cc	1190002A	adenylate	Ak8
[truncated: 2,890,206 more chars]
